# A taxonomic monograph of the assassin bug genus *Zelus* Fabricius (Hemiptera: Reduviidae): 71 species based on 10,000 specimens

**DOI:** 10.3897/BDJ.4.e8150

**Published:** 2016-07-08

**Authors:** Guanyang Zhang, Elwood R Hart, Christiane Weirauch

**Affiliations:** ‡ Department of Entomology, University of California, Riverside, Riverside, United States of America; § School of Life Sciences, Arizona State University, Tempe, United States of America; | Department of Entomology, Iowa State University, Ames, United States of America; ¶ Department of Entomology, University of California, Riverside, CA 92521, United States of America

**Keywords:** Harpactorinae, Heteroptera, natural enemy, Nearctic, Neotropical, new species, Reduviidae, synonymy, systematics, taxonomic revision, *
Zelus
*

## Abstract

The New World assassin bug genus *Zelus* Fabricius, 1803 (Insecta: Hemiptera: Heteroptera: Reduviidae: Harpactorinae: Harpactorini) is revised based on more than 10,000 specimens. Seventy-one species are recognized and twenty-four described as new: *Zelus aithaleos* **sp. n.**, *Zelus amblycephalus* **sp. n.**, *Zelus antiguensis* **sp. n.**, *Zelus auralanus* **sp. n.**, *Zelus bahiaensis* **sp. n.**, *Zelus banksi* **sp. n.**, *Zelus casii* **sp. n.**, *Zelus championi* **sp. n.**, *Zelus cordazulus* **sp. n.**, *Zelus fuliginatus* **sp. n.**, *Zelus gilboventris* **sp. n.**, *Zelus gracilipes* **sp. n.**, *Zelus grandoculus* **sp. n.**, *Zelus kartaboides* **sp. n.**, *Zelus lewisi* **sp. n.**, *Zelus panamensis* **sp. n.**, *Zelus paracephalus* **sp. n.**, *Zelus rosulentus* **sp. n.**, *Zelus russulumus* **sp. n.**, *Zelus spatulosus* **sp. n.**, *Zelus truxali* **sp. n.**, *Zelus umbraculoides* **sp. n.**, *Zelus umbraculus* **sp. n.**, and *Zelus xouthos* **sp. n.** Five species, *Zelus araneiformis* Haviland, 1931, *Zelus gradarius* Bergroth, 1905, *Zelus modestus* (Stål, 1862), *Zelus subfasciatus* Stål, 1860 and *Zelus vittaticeps* Stål, 1866, are removed from *Zelus* and placed *incertae sedis* within Harpactorini. Nine new synonyms are recognized (senior synonym in parentheses): *Zelus atripes* Champion, 1898 **syn. nov.** (=*Zelus conjungens* [Stål, 1860]), *Zelus dispar* Fabricius, 1803 **syn. nov.** (=*Zelus pedestris* Fabricius, 1803), *Zelus formosus* Haviland, 1931 **syn. nov.** (=*Zelus laticornis* Herrich-Schaeffer, 1853), *Zelus obscuridorsis* (Stål, 1860) **syn. nov.** (=*Zelus pedestris*), *Zelus pallidinervus* Haviland, 1931 **syn. nov.** (=*Zelus kartabensis* Haviland, 1931), *Zelus personatus* Berg, 1879 **syn. nov.** (=*Zelus versicolor* Herrich-Schaeffer, 1848), *Zelus trimaculatus* Champion, 1898 **syn. nov.** (=*Zelus means* Fabricius, 1803), *Zelus trimaculicollis* (Stål, 1855) **syn. nov.** (=*Zelus means*), and *Zelus tristis* Haviland, 1931 **syn. nov.** (=*Zelus laticornis*). *Zelus conjungens* (Stål, 1860) **stat. rev.** Is resurrected from junior synonymy with *zealous armillatus* (Lepeletier & Seville, 1825). *Zelus ambulans* Stål, 1862 **stat. rev.** and *Zelus cognatus* (Costa, 1862) **stat. rev.** are resurrected from synonymy with *Zelus exsanguis* Stål, 1862. *Iquitozelus* Bérenger **syn. nov.** is synonymized with *Zelus* and its only species transferred to *Zelus*, hence resulting in a new combination, *Zelus couturieri* (Bérenger, 2003) **comb. nov.** Lectotypes, paralectotypes or neotypes are designated for a number of species. Habitus images, illustrations of male genitalia, distribution maps and measurements are provided for nearly all species. The three previously recognized subgenera of *Zelus* are found to be based upon superficial characters and these divisions do not reflect natural groupings. Using sets of characters, especially those of the male genitalia, eleven species groups are proposed. It is also hypothesized that *Zelus* is closely related to three other New World genera: *Atopozelus* Elkins, *Ischnoclopius* Stål and an undescribed genus "Hartzelus" [manuscript name]. *Zelus* is endemic to the New World, occurring naturally in the Caribbean and all but one of the continental countries, with introductions to Pacific islands, Europe and Chile.

## Introduction

*Zelus* Fabricius, 1803 is one of the largest reduviid genera (Maldonado 1990) and the largest New World genus in the tribe Harpactorini (Reduviidae: Harpactorinae). *Zelus* is endemic to and widely distributed throughout the New World, ranging from southern Canada through central Argentina. One species, *Zelus renardii* Kolenati, 1856, has been introduced to Hawaii (Kirkaldy 1902, Zimmerman 1948), and was recently found in Chile (Curkovik et al. 2004, Elgueta and Carpintero 2004), Greece (Davranoglou 2011, van der Heyden 2015 and Spain (Vivas 2012) (reviewed in Weirauch et al. 2012). Species of *Zelus*, among several other genera (e.g., *Arilus* Hahn, *Sinea* Amyot & Serville, and *Montina* Amyot & Serville), have been explored and studied as natural enemies in the Americas (Cohen and Tang 1997, Cogni et al. 2002). Species of *Zelus* prey on a wide range of insects in cotton, corn, soybean, alfalfa crops and fruit trees in California and elsewhere (Ali and Watson 1978, McPherson et al. 1982, Cisneros and Rosenheim 1998, Virla et al. 2015), may reach population densities of up to 50,000 to 75,000/ha, and prevent outbreaks of lepidopteran larvae (Ables 1978). Hart (1972) conducted a taxonomic revision of *Zelus* with descriptions of twenty-five new species and twenty-six new synonyms, most of which remained unpublished (see Hart 1986, Hart 1987 for treatments of twenty Canadian, US, northern Mexican and Caribbean species). The current state of taxonomy of *Zelus* remains unsatisfactory and impedes further research on the evolution and ecology of this group. Species identification is difficult in many instances, and misidentifications may arise. For example, *Z. renardii* was misidentified as *Zelus cervicalis* Stål, 1872 when it was reported as having been introduced to Chile (Curkovik et al. 2004). This project was thus undertaken to provide a taxonomic monograph of the genus *Zelus*.

In the current study seventy-one species are treated and twenty-four described as new. Five species are removed from *Zelus* and placed *incertae sedis* within Harpactorini. Nine new synonyms are recognized. Three species are resurrected. *Iquitozelus* Bérenger is synonymized with *Zelus*. Habitus images, illustrations of male genitalia, distribution maps, and identification keys are provided. This work evaluates and maintains most of the manuscript new species names proposed in Hart (1972)'s systematic revision of *Zelus*. Four additional new species are discovered and described herein, some based on specimens more recently collected, but some arising from different views of species boundaries. The vast areas of the Amazon and many mountainous regions of Central America and South America remain poorly sampled and should represent an immediate frontier for new species discoveries in this genus.

### Review of taxonomic history

The taxonomic history of *Zelus* is complex and the generic limit of *Zelus* has undergone constant fluctuations. The first species of *Zelus*, *Z. longipes* (Linnaeus), was described by Linnaeus in the 12th edition of *Systema Naturae* (Linnaeus 1767) under *Cimex*, a genus in which he also included various other Heteroptera that are now classified within a number of families. Fabricius (1775) transferred *Z. longipes* from *Cimex* to *Reduvius*, a genus that was established to accommodate most of the Reduviidae known at the time. It was again Fabricius who later in the first comprehensive treatment of Hemiptera (Fabricius 1803) erected the genus *Zelus*. In this work for each genus Fabricius selected one species for which he repeated the short generic description, expanded the species description and italicized the terms referring to the morphological structures described. *Zelus longipes* was treated by Fabricius this way for the genus *Zelus*. Therefore, many workers assumed that Fabricius had indicated *Z. longipes* as the type species of the genus (Kirkaldy 1900a).

Lepeletier and Serville (1825) expanded the limit of *Reduvius* to include nearly all then described Reduviidae and described many new species; several of them would now be considered members of *Zelus*. An erroneous designation of *Zelus festinans* as the type species of *Zelus* was made by Laporte (1832). He erected new genera and removed some species from *Zelus*, somewhat changing the generic limit of this genus. Perty (1834) treated *Reduvius* similarly to Lepeletier and Serville and described several new species of *Zelus*, placing them in *Reduvius*.

Burmeister (1835) modified the classification of Reduviidae and divided members of *Zelus* into two genera: *Euagoras* Burmeister, 1835 and *Arilus* Hahn, 1831. The limits of *Zelus* were expanded by Brullé (1836) to include part of *Reduvius* as defined by Fabricius, *Harpactor* and *Prionotus* as defined by Laporte and *Myocoris*, *Evagoras* (for Euagoras), *Notocyrtus* and *Arilus* as used by Burmeister. Blanchard (1840) again changed the limit of *Zelus*. It was to include some species of *Cimex* of Linnaeus and Stoll (1788), *Reduvius* of Fabricius and Wolff and *Prionotus* of Laporte. Amyot and Serville (1843) erected a new genus *Diplodus* to accommodate both described and new species of some *Zelus*. Herrich-Schaeffer (1848), Herrich-Schaeffer (1853) described several new species of what would be considered as members of *Zelus* by subsequent workers. Signoret (1862) described a new species of *Diplodus* which would eventually be transferred to *Zelus*.

A series of works by Stål greatly changed the generic limits of *Zelus* (Stål 1855, Stål 1859, Stål 1860, Stål 1861, Stål 1862, Stål 1866). Among those, Stål (1862) redefined *Zelus* as containing three subgenera: (1) *Zelus* of Fabricius, which also contained *Euagoras* Burmeister, characterized by the posterior pronotal lobe unarmed and the humeral angles rounded. One previously described species was listed, four species being therein synonymized as part of a total of nine varieties listed for that species. (2) *Diplodus* of Amyot and Serville, characterized by the disc of posterior pronotal lobe unarmed and the humeral angles armed with a tooth or spine. Ten new species were described therein, one of these with three varieties, one with two varieties and another with four varieties. (3) *Pindus* Stål, recognized by the posterior pronotal lobe armed with two spines on the disc and a spine on each humeral angle. One new species was described.

However, the subgeneric groups were raised to the generic rank by Stål (1866) in a key to the genera of the New World reduviids. Later Stål (1872) again recognized *Zelus*, *Diplodus* and *Pindus* as subgenera of the genus *Zelus*, with fifteen, thirty and two species respectively. A Fabrician species was not assigned to a subgenus. He also provided a key to the genera, subgenera and species and a list of synonymy. The geographical distribution of *Zelus*, according to this study, is restricted to the New World.

Uhler listed a species of *Diplodus* from one of the U. S. Geological Survey expeditions (Uhler 1872a), described a new species of *Pindus* (Uhler 1872b) and compiled a checklist of North American Hemiptera (Uhler 1886). This checklist recognized six species of *Zelus*, sixteen species of *Diplodus* and one species, which in reality should have been assigned to *Zelus*, had been incorrectly described as a *Darbanus* by Provancher (1872). Based on the framework established by Stål (1872), Berg (1879) described two new species of *Zelus* in his faunal study of the Hemiptera of Argentina.

Lethierry and Severin (1896), in their catalogue of the Hemiptera-Heteroptera of the world, adopted Stål (1872)'s definition of *Zelus*. Fifty-two species, nine synonymized names and eleven varieties were recognized. Champion (1898) recorded eighteen species within Mexico and Central America, eight of which were described as new species. A short discussion of the genus, a key to the eighteen species, drawings, descriptions, redescriptions and species discussions were included.

Kirkaldy (1900a), Kirkaldy (1900b), Kirkaldy (1901), Kirkaldy (1903) reviewed the nomenclatural validity and the history of the types, genera and subgenera of the new species, discussed the possible synonymy of some others and synonymized several species. As *Diplodus* Amyot and Serville was proven to be preoccupied, Kirkaldy (1900b) proposed *Diplacodus* as a new name for this subgenus. However, this name was also preoccupied and most following taxonomists used the name *Diplocodus*.

The generic and subgeneric definitions of Stål were also used by Fracker (1913). In North America, including Mexico, twenty species were included in a key. In a list of the geographic distribution, three additional species for which no specimens were available were discussed. One of these is noted as being a probable synonymy and another as probably belonging to *Castolus* Stål.

Van Duzee (1916)'s checklist of Hemiptera of the United States included *Zelus* as a genus of the tribe Zelini, subfamily Harpactorinae. Stål’s three subgenera were recognized. Kirkaldy's *Diplacodus* was accepted for *Diplodus*, but was here and after spelled as *Diplocodus*, no explanation being given for the change. There were nine species recognized in the genus and five names were listed in synonymy. The known references for the genus, subgenera and these nine species were compiled in Van Duzee (1917)'s catalogue of North American Heteroptera.

In his study of the Heteroptera of eastern North America, Blatchley (1926) used the subfamily name Zelinae to include *Zelus* and related genera. *Zelus* was redescribed and a short discussion of the distribution and habits of assassin bugs in the genus was included. Each of the six species of this area was keyed, redescribed, and short notes on its biology and distribution made. Readio (1927) made studies of the biology of the ten species which he recognized in North America north of Mexico. These species were keyed and the descriptions and distributions of each were included. A major addition to the number of species of *Zelus* was made in by Haviland (1931). A total of nine species, including six new species, were reported from British Guiana. Wygodzinsky (1949) published a checklist of the Reduviidae of the Americas. He included sixty-six species, most of which are Neotropical in distribution, as valid members of the genus. Zayas (1960) described a new species from Cuba. Elkins (1954) removed two species.

In his Ph.D. dissertation Hart (1972) provided a systematic revision of *Zelus*, in which he described new species, proposed new synonyms, created a species group classification, illustrated male genitalia of nearly all species, and provided an identification key. Most of this work remains unpublished, except for two regional treatments of species occurring in Canada, US, northern Mexico and the Caribbean (Hart 1986, Hart 1987), in which he dealt with twenty species, described four new species, proposed fifteen new synonyms and provided identification keys. Maldonado (1990) in the "Systematic Catalogue of the Reduviidae of the World" listed sixty valid species names and did not use subgeneric divisions within the genus *Zelus*.

Recent taxonomic activities on *Zelus* spp. are scant. Jadin et al. (2002) described a new species, *Zelus josephpaulusi* Jadin et al., which was later found to be a synonym of *Zelus araneiformis* Haviland, 1931 by Baena (2010), a species we do not consider as belonging to *Zelus*. Bérenger (2003) described a new genus, *Iquitozelus*, based on a single species, *Iquitozelus couturieri* Bérenger, 2003. This genus is synonymized with *Zelus* in the current study. Gil-Santana (2008) synonymized *Zelus nigrispinus* (Herrich-Schaeffer, 1848) with *Zelus versicolor* (Herrich-Schaeffer, 1848). Gil-Santana and Forero (2015) placed *Zelus iopterus* (Perty, 1832) in synonymy with *Aristathlus imperatorius* Bergroth 1913 and thus created a new combination *Aristathlus iopterus* (Perty, 1832), removing that species from *Zelus*.

## Materials and methods

### Specimens, databasing and georeferencing

During the course of this study, 10,626 specimens were examined and databased. Among those, 4,833 are males, 5,626 are females and the remainders are immatures or with sex undetermined (usually because of missing abdomen). Specimen loans were kindly provided by museums or collections listed in Table 1. Each specimen was affixed with a Unique Specimen Identifier (USI) label (see Schuh 2012). This label has a catalogue number that has a prefix indicating the institution that performed the databasing and almost all specimens examined in this study have the prefix "UCR_ENT" on their USI labels, which means that they were databased at the University of California, Riverside (UCR) or by people associated with that institution. The prefix "UCR_ENT" is followed by an eight digit number. The acronym of the owner or specimen-depositing museum or collection is also indicated on the USI label, e.g., USNM. Label information, including locality and collecting event, was entered into the PBI (Plant Bug Planetary Biodiversity Inventory) instance of the Arthropod Easy Capture database (Schuh 2012) (https://research.amnh.org/pbi/databases/locality_database.html) hosted at the American Museum of Natural History. Specimen records are publicly accessible at the 'Heteroptera Species Page'. When coordinates were not provided on specimen labels, which is the situation for the great majority of the specimens, the locality was geo-referenced using gazetteers. These included the USGS Geographic Names Information System (mainly for US localities), Google Earth, Global Gazetteer and Fuzzy Gazetteer. For localities outside the USA, higher level administration divisions included only country and state (province or department), and the county-level administrative division was not used. Localities consisting of only a US county, but not more detailed information, were given the coordinates of the county seat. Specimen information was then exported from the PBI database as Excel files. Data description terms used in PBI were manually changed to equivalent Darwin Core Terms as specified in the "Darwin Core Terms: A quick reference guide" maintained by the Biodiversity Information Standards - TDWG (2013). Specimen records representing holotype, allotype, lectotype, neotype, paratype and paralectotype of both valid species and synonyms are provided in the main text, and all specimen records including both type and non-type specimens in Suppl. material 1.

### Type specimens

Information of type specimens of described, valid species, when available, were reported in the 'Materials' section of each species, as holotype, lectotype or neotype. Hart (1972) examined nearly all primary type specimens of valid names and synonyms. Some of these were also examined in the present work and associated with USI labels, but some were not examined nor attached with USI labels. Holotypes of newly described species were designated and reported. Holotypes and lectotypes of junior synonyms, either previously established or newly designated in the current study, were also reported, but included in the 'Other materials' part of the 'Materials' section under the Darwin Core term 'occurrenceRemarks'. This is because no Darwin Core term specifically denotes the type status of a junior synonym. The primary type specimen stays as a type of a species name even if the name becomes a junior synonym and should be indicated as such. However, it would be confusing and incorrect to include the type specimen of a junior synonym as part of the primary type material of the valid name. It was thus decided to report the type specimens of junior synonyms with the aforementioned method. Dikow and Leon (2014) has used a similar method. Lectotypes, paralectotypes and sometimes allolectotypes of some described species (valid or synonym) were newly designated in this study and indicated with the term 'New Designation' in the materials section for the respective species.

Hart (1972) designated numerous type specimens including holotypes, allotypes and paratypes of his manuscript new species names and lectotypes, allolectotypes, and paralectotypes of some previous names. Most of these type designations were adopted in the present study, but the status "allotype" was not used, and the specimens were instead designated as paratypes. These pertained exclusively to new species. Hart's type designation labels remain attached to specimens, and new designation labels are also affixed to indicate the type status of the specimen designated in the current study.

### Distribution and mapping

Distributions were based on specimen records captured in the current study. We have gathered the largest samples ever known of all species, which represent the best available knowledge of the distributions of the species of *Zelus*. Maps were created using the Simple Mapper tool through the PBI website (http://research.amnh.org/pbi/maps) based on the geo-referenced locality data. Because accuracy and error of geo-referencing are highly variable, distribution records shown on the maps are at best indicative. Besides, ambiguous localities and other localities provided only at the country or state level were not geo-referenced and thus not reflected on the distribution maps. It is, therefore, advisable to look up actual specimen data for locality information (Suppl. material 1). Interactive specimen mapping is available using the Global Mapper module of "DiscoverLife" (http://www.discoverlife.org/mp/20m?act=make_map).

### Morphological methods – dissection, observation, imaging and measurement

Dissection. Male genitalia, including the eighth abdominal segment and pygophore with phallus enclosed, were removed, cleared in heated 10% potassium hydroxide (KOH) solution for 5–10 minutes, washed in distilled water, and stored in glycerol. To remove the genitalia, a specimen was softened by soaking the abdomen in water. This was achieved by stationing a pinned specimen on play clay with its abdomen pointing down and immersed in water, while the rest of the specimen was not submerged. This method avoided soaking the whole specimen or removing the entire abdomen. A 'genitalia hook' was made by melting the tip of a glass Pasteur pipette with a minuten insect pin inserted and fixed into it. The pygophore was carefully removed by holding the softened abdomen with forceps on one hand, and inserting the genitalia hook into the membranous connection between the seventh and eighth segments, breaking that membrane and pulling off the pygophore, a series of actions performed by the other hand.

Observation. Observations were made using a Nikon stereo dissecting microscope SMZ1500, illuminated by a Nikon NI-150 High Intensity Illuminator. Initial observations of morphological characters were made based on typically a small number of specimens (one to five) and intraspecific variations were subsequently examined based on a larger selection of geographical representation. Genitalia were observed in glycerol. Structures in this medium may look different from their dry state, especially for soft cuticles. For example, the apices of the parameres of *Z. cervicalis*, *Zelus luridus* Stål, 1862 and many other species usually appear shriveled in dry specimens, but are fully expanded in glycerol or 70% ethanol. The orientation of the dissected structures shown in illustrations does not necessarily reflect their natural condition.

Imaging and illustration. Dorsal and lateral habitus images of specimens were taken with a Microptics-USA system (now Visionary Digital, http://www.duninc.com/index.html) with a K2 lens and CF-2 or CF-4 objectives connected to a Canon EOS 1D digital SRL. Images were edited in Photoshop (CS3/CS4) to adjust levels and sharpness. Image background was removed and replaced using CorelDRAW or Photoshop. For most species illustrations of male genitalia were adapted from Hart (1972) except for several new species not included in that work.

Measurement. Measurements were made on a dissecting scope equipped with a two-axes movable stage (Mitutoyo Corp.), with the aid of two digital micrometers (Boeckeler®), which were connected to a Microcode II RS-232 digital readout (Boeckeler®). Most measurements were done in dorsal view, but various orientations were necessary for measuring appendages. Typically, five to ten specimens were measured for each species, but the number may be fewer for species without enough properly preserved specimens. All measurement values reported here are in mm, unless otherwise stated. In Suppl. material 2 measurement values are divided into length values and width values. Suppl. material 2 reports not only average values for each species but also values of all individual specimens. A total of twenty-nine measurements were captured as listed in the following.

Length measurements

1. Total length: length of body from clypeus to apex of hemelytron
2. Clyp-Abd: Clypeus-abdomen (length from clypeus to apex of abdomen)
3. Head (length of head from clypeus to collar of anterior pronotal lobe)
4. AntOc: Anteocular (length of anteocular region of head, from clypeus to anterior margins of eyes)
5. PostOc: Postocular (length of postocular region of head, from posterior margins of eyes to collar of anterior pronotal lobe)
6. AntPron: Anterior pronotal lobe (length from collar to transverse sulcus of pronotum)
7. PostPron: Posterior pronotal lobe (length from transverse sulcus of pronotum to posterior margin of posterior pronotal lobe)
8. Scut: Scutellum (only exposed part measured, from posterior margin of pronotum to apex of scutellum)
9. Scap: Scape
10. Ped: Pedicel
11. Antn3: Antennal segment 3/Basiflagellomere (the basiflagellomere tends to be curled and in that case two or several consecutive measurements were taken and their sum was used)
12. Antn4: Antennal segment 4/Distiflagellomere
13. Profem: Profemur
14. Protib: Protibia
15. Mesofem: Mesofemur
16. Mesotib: Mesotibia
17. Metafem: Metafemur
18. Metatib: Metatibia
19. Lb1: 1st visible labial segment (this is actually homologous to the second labial segment in other heteropteran insects, and Lb2 and Lb3 are homologous to the third and fourth segments. See Weirauch 2008b)
20. Lb2: 2nd visible labial segment
21. Lb3: 3rd visible labial segment

Width measurements

1. Head (width from outer margin of one eye to that of the other)
2. InterOcDi: Interocular distance (width from inner margin of one eye to that of the other)
3. AntPron: Anterior pronotal lobe (width across the widest part)
4. PosPron: Posterior pronotal lobe (width between humeral angles, not including processes)
5. Abd: Abdomen (measured at the widest part of the abdomen)
6. Profem: Profemur (measured at median point)
7. Mesofem: Mesofemur (measured at median point)
8. Metafem: Metafemur (measured at median point)

### Descriptive taxonomy

Description. Observations were recorded with the software DEscriptive Language for TAxonomy (DELTA) (Dallwitz 1980, Dallwitz et al. 1999). Natural descriptive language was exported and edited. Observations and descriptions were done primarily based on male specimens, descriptions of females were restricted to non-genitalic characters different from males. Four major character systems were described: coloration, vestiture, structure (non-genitalic), and male genitalia. Description of abdominal vestiture was restricted to the ventral surface. Description of a body part usually started with the overall appearance of that body part such as length, width, and general shape. Structural components of that body part were then described in the order from anterior to posterior, medial to lateral, dorsal to ventral, and proximal to distal. Ratios were determined by comparing mean values across specimens measured. Ratio between the segments of labium or antenna was in reference to the respective first segment. Unless otherwise stated, all measurement values reported in the text are mean values. For closely related or very similar species, a full description was provided for one representative and only variable characters were described for other species. The singular form was usually used for paired structures except when referring to spatial relationships between these structures, e.g., "struts (of phallus) separate, sub-parallel", or when referring to the different pairs of legs at the same time, e.g., "femora and tibiae with alternating yellow and dark brown bands". Descriptions of coloration were mainly based on observations of dried specimens, but notes were made if images or observations of live specimens were available. We note that many dried specimens show browning or fading of colors. This was particularly evident in lightly or brightly colored species. We observed in a number of species that the ventral outline of abdomen is curved, a condition apparently resulting from the folding of segments three to six (sometimes two as well). It was not clear if this was a preservation artifact or a natural condition, but it has been consistently seen in several species. Thus, this character was described as observed.

Description of intraspecific variations. Intraspecific variations were described and indicated by terms or phrases as the following: sometimes, occasionally and in some specimens. When variations in coloration can be roughly delimited to several patterns, they were described and the frequency of the patterns sometimes mentioned as well. Intraspecific variations in male genitalic structures were usually not described or documented unless they are important for species diagnosis and identification, which is usually the case only for closely related species.

Association of males and females. For the majority of species, males and females show limited sexual dimorphism in size and coloration, and could be readily associated based on external morphology, corroborated by collecting data. However, sexual dimorphism is pronounced in a number of species. Males and females differ drastically in size, body configuration and coloration. Association of sexes for these species was based mainly on locality data and series of specimens of both sexes. Observations of mating reported in the literature were also consulted and used as corroborative evidence whenever available.

**Figure 1.**
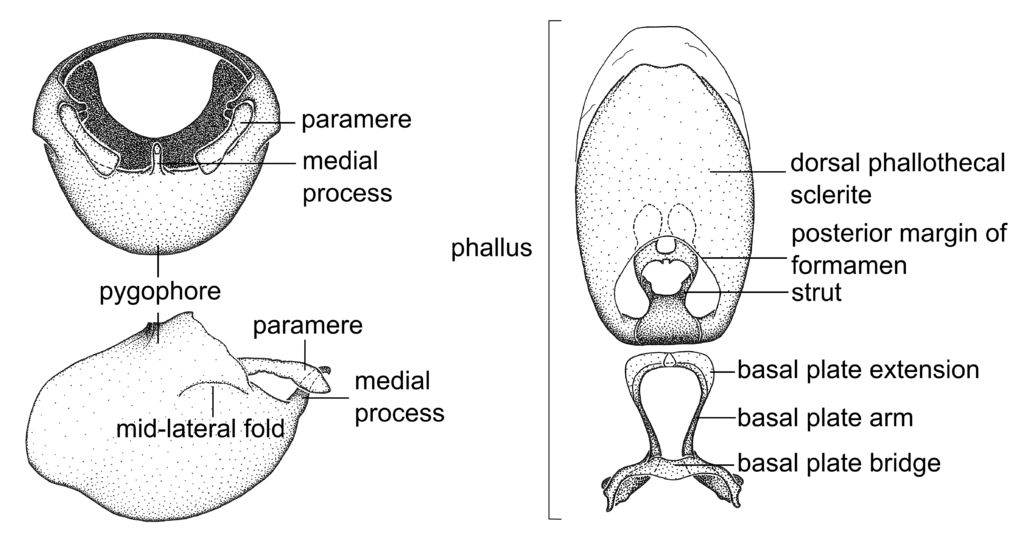
Male genitalic structure terms (*Zelus errans* Fabricius, 1803 is shown in the illustrations)

Terminology and abbreviation. External morphology and genitalic terminology followed Forero and Weirauch (2012), Davis (1966), Weirauch (2008a), Weirauch (2008b). The term ‘posterior margin of foramen’ was used to refer to the posterior margin of the foramen of the dorsal phallothecal sclerite. The foramen is located at the anterior part of the dorsal phallothecal sclerite and is an area that lacks sclerotization and surrounds the struts. The following abbreviations are used: BL = body length; C.A. = Central America; cat. = catalogue; comb. nov. = c*ombinatio nova*; Cu = Cubitus; descr. = description; desig. = designation; f = female; fig. = figure; L = length; m = male; M = median (vein), orig. = original; Pcu = Postcubitus; preocc. = preoccupied; R = radius, S.A. = South America; SD = standard deviation; sp. n. = *species nova*; syn. = synonym; syn. nov. = *synonymum novum*; stat. rev. = *status revisus*; W = width. Terms used to describe male genitalia are illustrated in Fig. 1.

Nomenclature (Annotated synonymic list). The synonymic list comprises abbreviated synonymies and included those names that previously appeared in the taxonomic literature or have affected the taxonomy of the species. Citation to ecological, agricultural or other non-taxonomic literature was presented when appropriate, but not meant to be exhaustive. Historical taxonomic publications were briefly annotated to indicate kind of taxonomic information or nomenclatural acts such as .orig descr., checklist, cat., note, fig. and key. When a species name is followed by the original author and year, there is no colon (:) separating the name and the author. A species name followed by a colon indicates that the author of the work is not the author of the name.

### Electronic publication

This publication is registered in ZooBank. In accordance with the 2012 Amendment to the International Code of Zoological Nomenclature regarding electronically published works (Krell and Pape 2015), all new species names have been registered in ZooBank and their lsid values provided.

## Taxon treatments

### Zelus

Fabricius, 1803

Zelus Fabricius, 1803, p. 281, orig. descr.; Latreille, 1804, p. 260, list; Latreille, 1807, p. 129, list; Latreille, 1810, p. 433, type desig.; Lepeletier and Serville, 1825, p.815, list and descr.; Laporte, 1832, p. 9, type desig.; Burmeister, 1835, p. 225, descr.; Brulle, 1836, p. 316-317, descr.; Blanchard, 1840, p. 100, descr. and note; Blanchard, 1845, p. 433, 438, list and note; Herrich-Schaeffer, 1848, p. 88, descr. and note; Kolenati, 1857, p. 458-459, descr.; Stål, 1861, p. 148, descr.; Stål, 1862, p. 449-454, key and subgeneric descr. (with subgenus *Zelus*); Carpenter and Westwood, 1863, p. 565, note; Mayr, 1866, p. 138, list; Stål, 1866, p. 296, list; Stål, 1868, p. 107, restriction of definition; Stål, 1872, p. 69, 88, key and cat. (with subgenus *Zelus*); Walker, 1873, VII., p. 49, key, VIII., p. 131-136, cat.; Berg, 1879, p. 150, list (with subgenus *Zelus*); Uhler, 1886, p. 24, checklist; Provancher,1887, p. 179, note; Lethierry and Severin, 1896, p. 151, cat.; Champion, 1898, p. 251, cat. and note; Kirkaldy, 1900a, p. 263, type verification; Kirkaldy, 1900b, p. 242, syn.; Kirkaldy, 1902, p. 149, note; Fracker, 1913, p. 223, 238-240, key and note (with subgenus *Zelus*); Van Duzee, 1916, p. 30, checklist (with subgenus *Zelus*); Van Duzee, 1917, p. 258-259, cat. (with subgenus *Zelus*); Blatchley, 1926, p. 567-568, key, descr. and note (with subgenus *Zelus*); Readio, 1927, p. 167, 168-169, key, descr. and note; Zimmerman, 1948, p. 137, note; Wygodzinsky, 1949a, p. 48, checklist; Fracker and Usinger, 1949, p. 277, key to nymphs; Alayo, 1967, p. 5, 35, list, key and note; Hart, 1986, key to North American species; Hart, 1987, key to Caribbean species; Maldonado, 1990, p. 325-332, cat.
Reduvius Fabricius, 1775 (type by subsequent desig., *Cimex personatus* Linnaeus, 1758), Lepeletier and Serville, 1825 (in part), p. 272, descr.; Perty, 1834 (in part), p. 173, list of species.
Arilus Hahn, 1831 (type by subsequent designation, *Cimex carinatus* Forster, 1771); Burmeister, 1835 (in part), p. 227-228, descr.; Herrich-Schaeffer, 1848 (in part), p. 33-35, descr.
Euagoras Burmeister, 1835 (type by subsequent designation, *E. stollii* Burmeister, 1835) (in part), p. 226, descr.; Amyot and Serville, 1843 (in part), p. 368, descr. (as *Evagoras*); Herrich-Schaeffer, 1848 (in part), p. 43-44, descr.; Stål, 1855 (in part), p. 189, list (as *Eccagoras*); Stål, 1861, p. 148, (in part) junior syn. of *Zelus* Fabr.; Mayr, 1866, p. 139, list; Walker, 1873, p. 49, 117, key and cat.; Provancher, 1887, p. 182, descr. (as *Evagoras*); Kirkaldy, 1900b, p. 242, junior syn. of *Zelus* Fabr.; Kirkaldy, 1903, p. 215-216, note.
Diplodus Amyot and Serville, 1843, p. 370, descr.; Burmeister, 1853, p. 91, list (included in *Euagoras* Burm.); Stål, 1860, p. 74, list; Stål, 1862, p. 450, descr. (as subgenus of *Zelus*); Stål, 1866, p. 296, key; Stål, 1872, p. 90, list (as subgenus of *Zelus*); Walker, 1873, VII., p. 49, VIII., p. 123, key and cat. (as *Diploda*); Berg, 1879, p. 151, list (as subgenus of *Zelus*); Uhler, 1886, p. 24, checklist; Provancher, 1887, p. 179, key and descr.; Kirkaldy, 1903, p. 232, note; Fracker, 1913, p. 239, 240, key and list (as subgenus of *Zelus*).
Darbanus Amyot and Serville, 1843 (type by monotypy, *D. nigrolineatus*); Provancher, 1872, p. 106, species descr.; Uhler, 1886, p. 24, checklist; Provancher, 1887, p. 179, 181, key and note; Van Duzee, 1912, p. 324; Fracker, 1913, p. 241, note.
Pindus Stål, 1862, p. 454, orig. descr. (as subgenus of *Zelus*); Stål, 1866, p. 296, key (as genus); Stål, 1872, p. 92, list and cat., as subgenus of *Zelus*); Walker, 1873, VII., p. 66, list and cat. (as genus); Berg, 1879, p. 150, list (as subgenus of *Zelus*) ; Thierry and Severin, 1896, p. 151, cat.; Fracker, 1913, p. 223, 240, key and list; Van Duzee, 1916, p. 30, checklist; Van Duzee, 1917, p. 261, cat.; Blatchley, 1926, p. 569, key.
Diplacodus Kirkaldy, 1900b, p. 242, new name for *Diplodus* A. and S. (preocc.).
Diplocodus Van Duzee, 1916, p. 30, checklist (new name for *Diplacodus* Kirkaldy, preocc.); Van Duzee, 1917, p. 260, cat.; Blatchley, 1926, p. 569, key.
Iquitozelus Bérenger, 2003, p. 23, orig. descr., **syn. nov.** (current study).
Zelus Cimex longipes Linnaeus, 1767

#### Description

***Male***: Small to large, total length 8-25 mm (Suppl. material 2), with most of moderate sizes (11-18 mm); usually slender (length/width = 4.0-5.0), some species relatively robust (<3.5) to rather slender (>6.0). **COLORATION**: Colors and patterns of preserved specimens variably yellowish-brown, reddish-brown, orange-brown, and brownish-black to black, with most species uniformly colored. **VESTITURE**: Most species with moderately dense or dense, fine, short, recumbent and short, long, erect setae; some species with short, spine-like setae on head and pronotum; few species nearly glabrous. Setation on legs sparse in most species; profemur and tibia with dense sundew setae in some species. **STRUCTURE**: **Head**: Length much greater than width across eye. Postocular lobe usually longer than anteocular, tube-like posteriorly in most species. Ocellus raised, directed somewhat laterally. Eye variably sized, not protruding above or below dorsal or ventral surfaces of head, with one exception (*Zelus grandoculus* sp. n.). *Antenna*: Scape and basiflagellomere long and subequal in length, usually longer than head and pronotum combined; pedicel and distiflagellomere short and about 1/3 length of scape. Scape thickest; basiflagellomere usually thicker than pedicel, subequal in some species. *Labium*: Segment II longest, 1.3-2.2x length of segment I; segment III shortest, usually 0.5x length of segment I; variably curved between segments I and II. **Thorax**: Anterior pronotal lobe about 1/2 to 3/4 length of posterior lobe; anterolateral angles of pronotal collar rounded, with or without tuberculate protrusion; medial dorsal longitudinal sulcus usually shallow at collar, deepening through posterior 1/2; sometimes with subtuberculate elevation near posterior margin laterad to medial sulcus. Posterior pronotal lobe rugulose (not conspicuous in species with dense setation); slightly or greatly wider than anterior lobe; disc of most species elevated above humeral angle and posterior margin of lobe; humeral angle with tuberculate to long spinous lateral process, rounded and unarmed in small number of species. Scutellum in most species with angulate apex, slightly produced and projected upward in some species. *Legs*: long, slender in most species; femoral diameters generally subequal; pro- and metafemoral lengths subequal, greater than mesofemoral length. *Hemelytron*: Attaining or surpassing apex of abdomen, by large proportion in some species. Quadrate cell small to large; median vein conspicuous in some species and not visible in many. Cu and M of cubital cell subparallel in most species, converging in some. **Abdomen**: Lateral margins subparallel; ventral outline usually straight, in some species somewhat concave and abdomen appearing arched (see "Material and methods" for discussion of this character). **Genitalia**: Segment eight usually short, less than 1/2 length of pygophore; posterior margin generally slightly concave, straight in some species, never convex. *Pygophore*: ovoid to elongated; slightly to greatly expanded laterally close to base of paramere; dorsal bridge short to long. Medial process single, not bifurcating, of variable length and shape; triangular or cylindrical as most common configuration; apex blunt or with hooklike process. Paramere generally cylindrical, often swollen and bending apically, length variable. *Phallus*: Dorsal phallothecal sclerite generally semi-cylindrical, broad and shield-like in many species, elongated in some; dorsal surface lacking armature in most species, with projection, process or elevation in some species; lateral margins usually straight or convex, constricted or recurved in some species; apical part keeled in middle and/or curved dorsad; apex usually rounded or truncate, with or without medial emargination. Struts attached to dorsal phallothecal sclerite in majority of species; apical part recurved dorsad and often semi-circular; bridge connecting two sides in many species. Basal plate arm slender to heavy, separate or fused; basal plate bridge present, variable in width and degree of sclerotization; basal plate extension short, often extended onto basal plate arm.

***Female***: Larger than male. Coloration usually similar to that of male and more variable in some species, but may differ between sexes dramatically in certain species. Eye and ocellus smaller than in male in some species. Basiflagellomere not swollen and about equal diameter as or smaller than pedicel. Lateral process on humeral angle, if present, usually more produced and longer than in male. Mesofemur slightly swollen in many species. Lateral margins of abdomen expanded in some species.

#### Diagnosis

This genus is distinguished from other genera of the New World Harpactorini by the cylindrical head, the length of the head being at least 1.9X its width; the unarmed antenniferous tubercles; the second labial segment being at least 1.3x the length of the first segment; the long scape and basiflagellomere that are subequal in length and the short pedicel and distiflagellomere; the generally unarmed (i.e. no tubercles or spines) disc of the posterior pronotal lobe (except in *Zelus tetracanthus* Stål, 1862, *Zelus lewisi* sp. n. and *Zelus minutus* Hart, 1987); the humeral angle with or without process, and if present, usually not prominently projected; the legs with sundew setae and sticky glands (Zhang and Weirauch 2013); the profemur subequal in length and diameter to the metafemur; and the medial process of pygophore single, not bifurcating. *Zelus* is apparently closely related to three other genera, *Atopozelus* Elkins, *Ischnoclopius* Stål and "Hartzelus" [manuscript name], that share many of the aforementioned characters. It is separated from *Atopozelus* by the presence of paramere (lacking in *Atopozelus*). *Ischnoclopius* is distinguished from *Zelus* by its rather slender body form (length:width ratio greater than seven), the very long profemur, at least 0.6x of body length, and the very short paramere. An undescribed genus, "Hartzelus" (Gil-Santana and Berenger, pers. comm.), which would be in part based on species removed by us from *Zelus*, differs from *Zelus* in having a bifurcating medial process of the pygophore (single in *Zelus*) and generally more slender legs. No Old World species of Harpactorini are similar or appear to be closely related to *Zelus*. Confusion may potentially arise with members of genera that show a similar slender body form and slender legs (e.g., *Euagoras* and *Vestula* Stål), but these are distinguished from *Zelus* based on the characters listed above.

#### Distribution

Native to (except for Chile) and throughout the New World, including the Caribbean, with highest diversity in the Neotropics. One species (*Z. renardii*) has been introduced to Hawaii, the Polynesian islands, Jamaica, Philippines, Spain, Greece and Chile.

#### Biology

We provide a non-exhaustive account of the biology of various species of this genus. As with other harpactorines, species of *Zelus* generally do not show associations with or preferences of host plants, probably due to their generalist habits. However, two recent studies have found two species of *Zelus* that have both nymphs and adults occurring in the same plant species in relatively large quantities. In Gil-Santana and Alves (2011), based on a multi-year study the authors observed forty-seven females, twenty-seven males and fifteen nymphs of *Z. versicolor* from *Bidens rubifolia* Kunth (Asterales, Asteraceae) in a single site in the city of Nova Friburgo, Brazil. Interestingly, they did not see individuals of the same species in other plants in the same site, which can be seen as evidence for host plant preference. In French Guiana Revel et al. (2010) counted as many as 405 individuals of *Zelus annulosus* (Stål, 1866) and its egg masses from several pubescent plant species, including (but not limited to) *Hirtella physophora* Mart. & Zucc. (Chrysobalanaceae), *Cordia nodosa* Lam. (Boraginaceae) and *Tococa guianensis* Aubl. (Melastomataceae); all three are myrmecophytes. They hypothesized an intriguing tri-party mutualistic relationship between the assassin bug, an ant (*Allomerus decemarticulatus* Mayr) and the plants.

Several species of *Zelus* are possibly mimics of various other insects. *Zelus errans* Fabricius, 1803, *Zelus vespiformis* Hart, 1987 and to some extent *Zelus vagans* Fabricius, 1803 and *Zelus gracilipes* sp. n. have wing and body color patterns similar to many braconid wasps, an intriguing form of mimicry seen also in a number of other Neotropical harpactorine genera. *Zelus vagans* shows areas of black and orange colors, however, the posterior pronotal lobe is medially dark and laterally orange. *Zelus gracilipes* also shows a uniformly orange posterior pronotal lobe, but the hemelytron is uniformly dark and lacks the banding pattern typical to a wasp mimic. *Zelus nigromaculatus* Champion, 1899 has an appearance similar to that of a typical vespid, the only species in this genus with that kind of color pattern. *Zelus laticornis* (Herrich-Schaeffer, 1853), *Zelus grassans* Stål, 1862 and *Zelus ruficeps* Stål, 1862 have red and dark markings on abdomens and orange or reddish dorsal surfaces, a pattern found in many species of pyrrhocorids (e.g., *Dysdercus* spp.) and coreids (e.g. *Hypselonotus* spp.). Interestingly, in *Z. laticornis*, it is only the females showing this coloration. Certain color forms of *Z. longipes* are possibly mimics of the milkweed bug, *Oncopeltus fasciatus* (Dallas).

Weirauch et al. (2012) studied predatory and mating behaviors of *Z. renardii* and *Z. tetracanthus* and discussed a possible link between biological attributes and invasion potential. Law and Sediqi (2010) experimentally demonstrated that sticky substance derived from egg mass coating improves predation success and substrate adhesion ability of *Z. renardii* first instar.

#### Taxon discussion

The generic limit of *Zelus* is now relatively well defined and the genus can be separated from all other but one genera of New World Harpactorini based on characters discussed in the diagnosis. Based on a molecular phylogeny, Zhang and Weirauch (2014) recovered the monophyly of *Zelus, Atopozelus* and "Hartzelus" (which includes *Z. araneiformis*, a species we remove from *Zelus*). In that analysis, *Ischnoclopius* was represented by a single species and placed as sister to *Atopozelus*. The genera *Atopozelus*, "Hartzelus", *Ischnoclopius* and *Zelus* together constitute a monophyletic group in the same study, and we here refer to this group the "*Zelus* clade". Without a cladistic analysis, questions remain if the characters used to diagnose *Zelus* are synapomorphies of that genus. It is almost certain that the unbifurcating medial process represents a symplesiomorphic state as that character can be seen in *Atopozelus*, *Ischnoclopius* and many other Neotropical harpactorines. The unarmed antenniferous tubercles are also plesiomorphic to *Zelus*, since all other genera of the *Zelus* clade exhibit that condition, but may be synapomorphic to the *Zelus* clade. We agree with Forero (2012) that *Zelus* is defined mainly by the absence of apomorphies seen in other genera. Future research should illuminate this issue by studying the distribution and the polarity of characters with a formal cladistic framework.

The genus that we are uncertain about its relationship with *Zelus* is *Pronozelus* Forero, erected by Forero (2012) to accommodate a new species, *Pronozelus schuhi* Forero, 2012. This species appears to possess all the characters diagnostic of *Zelus*, but also shows some peculiar characters. The principal characters separating *Pronozelus* from *Zelus* include the laterally expanded posterior pronotal lobe, the prominent, greatly expanded posterolateral rim of pygophore lateral to paramere socket, and the posterior pronotal lobe greater than 2.2x length of the anterior lobe. The conspicuous lateral expansion of the posterior pronotal lobe is not observed in any species of *Zelus*, but this character appears to be autapomorphic in the *Zelus* clade and does not support *P. schuhi* being phylogenetically separated from *Zelus*. We have not done an extensive survey of the condition of the posterolateral rim of the pygophore and cannot determine the distribution or polarity of the lateral prominence as exhibited in *P. schuhi*. In *Zelus rosulentus* sp. n., the posterolateral part of the pygophore also appears to be expanded, although not as prominent as that seen in *P. schuhi*. Finally, according to measurements done in this study, in *Zelus* spp. the posterior pronotal lobe frequently exceeds 2.2x length of the anterior lobe, thereby negating the use of that character as a basis for placing *P. schuhi* outside *Zelus*. Despite the foregoing discussion, we have opted to not transfer *P. schuhi* to *Zelus* or synonymize *Pronozelus* with *Zelus*. The polarity of the characters diagnostic to either genus has not been clearly defined. There remains a possibility, although we think a small one, that *P. schuhi* represents a lineage sister to *Zelus*.

Bérenger (2003)'s new species, *Iquitozelus couturieri*, exhibits all the characters diagnostic of *Zelus*, except for those of the male genitalia as the known specimens are all females. The main character that Bérenger used as the basis for erecting a new genus, i.e., the "foliaceous expansion of the VI connexivum segment", appears to be autapomorphic within the *Zelus* clade. Synonymy of *Iquitozelus* with *Zelus* is warranted and established here. We further postulate that *I. couturieri* is most closely related to *Zelus amblycephalus* sp. n., *Zelus umbraculus* sp. n. or *Zelus umbraculoides* sp. n. Further discussions regarding the status of *Iquitozelus* and the specific membership of *Zelus couturieri* syn. nov. (Bérenger, 2003) are presented in the treatment of that species.

Maldonado (1990) considered two unpublished, manuscript names invalid, and they are "Diplodus armiger" and "Diplodus melanophthalmus". They appeared in Dohrn (1860). We follow this treatment.

Except for several pairs or complexes of closely related species, identification of males can be almost always unambiguously performed based on exposed genitalic structures such as paramere and medial process, further corroborated with phallic structures, external morphology and coloration. Identification of females of many species, where females appear to be as distinct as males, is straightforward based on coloration and external morphology. However, identification can be difficult for closely related species, where females are indistinguishable based on external morphology. In these cases, association of males and females and identification of females were primarily based on collecting event information. Sexual dimorphism presents another special challenge. While most species show limited sexual dimorphism that does not go beyond minor size and coloration differences, some species exhibit pronounced differences between the sexes (see Material and Methods for discussion of association of male and female specimens). Based on the observation that species in closely related genera do not exhibit strong sexual dimorphism, we here hypothesize that pronounced sexual dimorphism is a derived condition within *Zelus*.

#### Species groups

We find here that previous subgeneric groups are based on superficial resemblance and these are not adopted. Instead, we recognize eleven species groups in the current study, based primarily on characters of the male genitalia, but also on non-genitalic external morphology if those characters can be applied to both sexes. Several species for which only females are known are therefore not assigned to a species group. Although the groupings proposed here are not based on a cladistic analysis, they show a degree of congruence with the relationships recovered in the phylogenetic analysis based on molecular data in Zhang and Weirauch (2014) and many of the characters are putative synapomorphies of the groups. A brief discussion of the species groups is presented below.

1. *Zelus tetracanthus* species group.

*Zelus minutus* Hart, 1987, *Zelus prolixus* Stål, 1860, *Zelus rosulentus* sp. n. and *Zelus tetracanthus* Stål, 1862.

**Figure 2.**
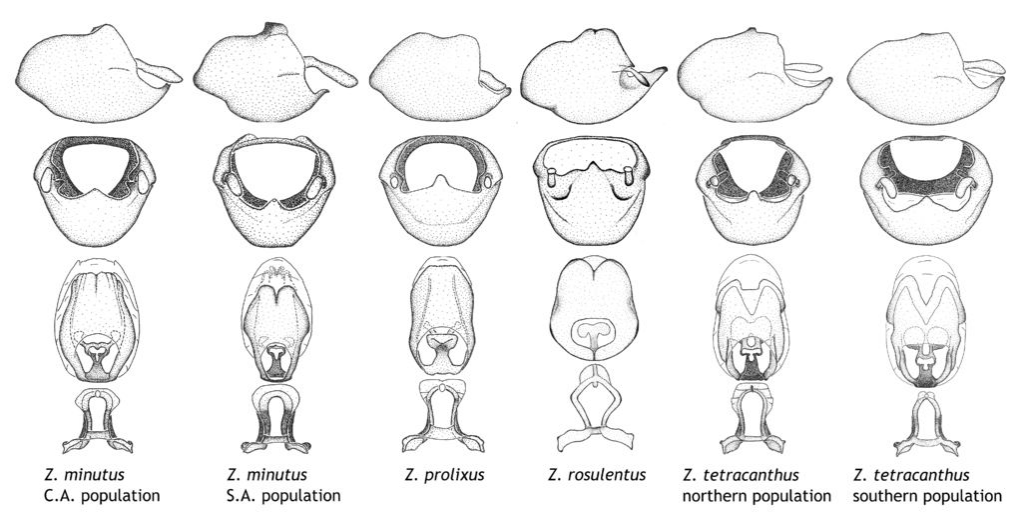
*Zelus tetracanthus* species group, male gentitalic structures

Members of this group have a rather broad, indistinct medial process, the base of which is nearly continuous with or inseparable from the ventral rim of the pygophore. We speculate that this character represents a plesiomorphic condition as it is seen in several other genera of the New World Harpactorini and thus the condition of the medial process does not necessarily support the monophyly of this group. *Zelus tetracanthus* and *Z. minutus* also both have tubercles on the disc of the posterior pronotal lobe, which are more pronounced in the former. Comparative views of male genitalia are shown in Fig. 2.

2. *Zelus luridus* species group.

*Zelus ambulans* Stål, 1862, *Zelus antiguensis* sp. n., *Zelus exsanguis* Stål, 1862, *Zelus grandoculus* sp. n., *Zelus luridus* Stål, 1862 and *Zelus spatulosus* sp. n.

**Figure 3.**
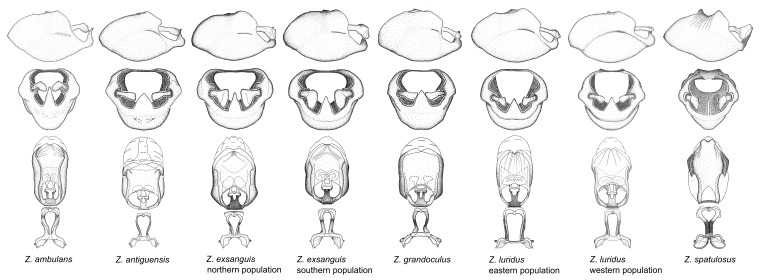
*Zelus luridus* species group, male genitalic characters

This is a group of species with primarily a North American distribution, with some species extending to northern Central America. The males show an apically expanded paramere and a triangular medial process that has a protrusion at the base but lacks any apical modifications. Notably, *Z. spatulosus* has a slender medial process, deviating greatly from the remainders of the group. It is placed in this group mainly because of the apically expanded paramere and the uniform coloration. *Zelus ambulans* and *Z. exsanguis* have the humeral angle elevated to about same level of and nearly continuous with the disc of the posterior pronotal lobe, a condition rarely seen in the genus. The coloration is quite homogenous among members of this genus, most of which have a uniform greenish (in live specimens) or dull brownish (in preserved specimens) habitus, with only *Z. ambulans* showing variable patterns or banding on the pronotum or legs. Comparative views of male genitalia are shown in Fig. 3.

3. *Zelus mimus* species group

*Zelus inconstans* Champion, 1898 and *Zelus mimus* Stål 1862.

**Figure 4.**
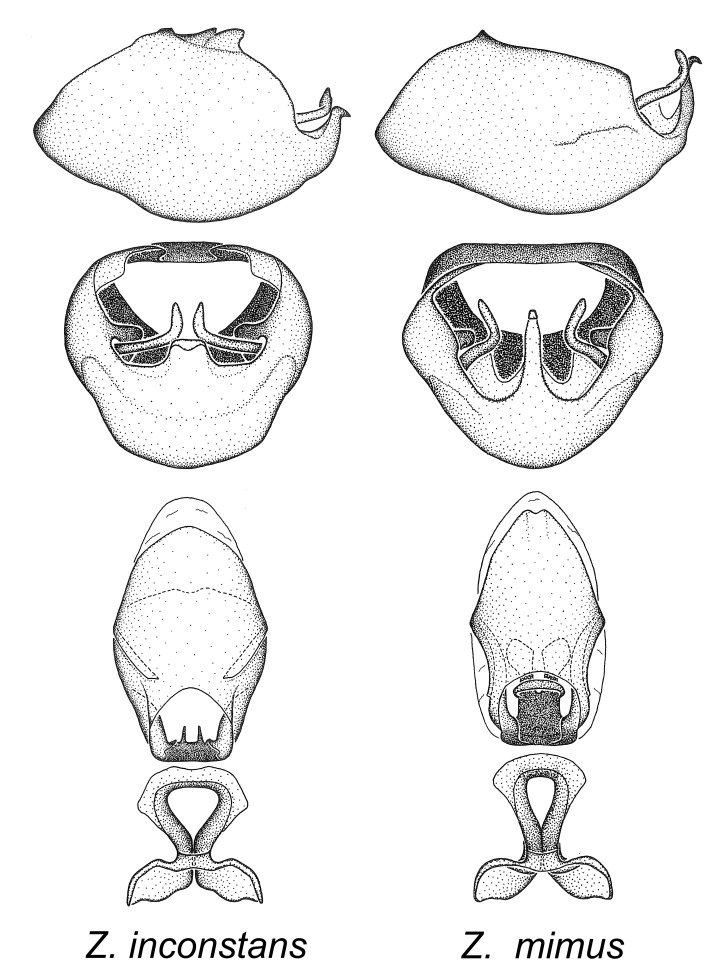
*Zelus mimus* species group, male genitalic structures

Members of this group, consisting of only two species, exhibit a highly unique paramere and a medial process of the pygophore. The paramere is slender and apically curved dorsad at an angle of nearly ninety degrees. The medial process, as is especially evident in *Z. inconstans*, possesses a simple posterior liplike fold at the apex; its lateral margins are subparallel and not broadened significantly at the base. Both have a semi-cylindrical dorsal phallothecal sclerite which is modified by a fold running obliquely toward the base from the middle of the lateral margins. Both species, being quite small, exhibit the usual reduction of setal tracts common to nearly all small species. Comparative views of male genitalia are shown in Fig. 4.

4. *Zelus nugax* species group.

*Zelus grassans* Stål, 1862, *Zelus illotus* Berg, 1879, *Zelus impar* Kuhlgatz, 1902, *Zelus nugax* Stål, 1862 and *Zelus pedestris* Fabricius, 1803.

**Figure 5.**
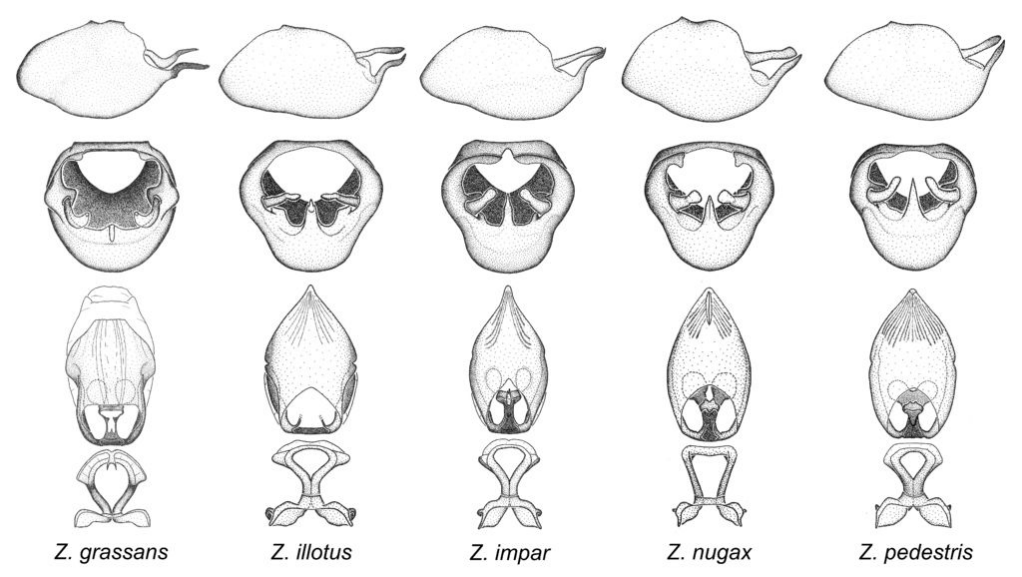
*Zelus nugax* species group, male genitalic structures

This is a group of smallish species with quite variable distributional ranges. The defining characters include a slender, laterally compressed medial process that is curved or recurved, and an acute apex of the dorsal phallothecal sclerite (except in *Z. grassans*). *Zelus nugax* has one of the widest distribution ranges in this genus, ranging from much of Mexico to northern South America. *Zelus grassans* is found primarily in Central America and the remaining two species mainly in northern South America. Comparative views of male genitalia are shown in Fig. 5.

5. *Zelus puertoricensis* species group.

*Zelus bruneri* De Zayas, 1960, *Zelus puertoricensis* Hart, 1987, *Zelus subimpressus* Stål, 1872 and *Zelus zayasi* Bruner and Barber, 1937.

**Figure 6.**
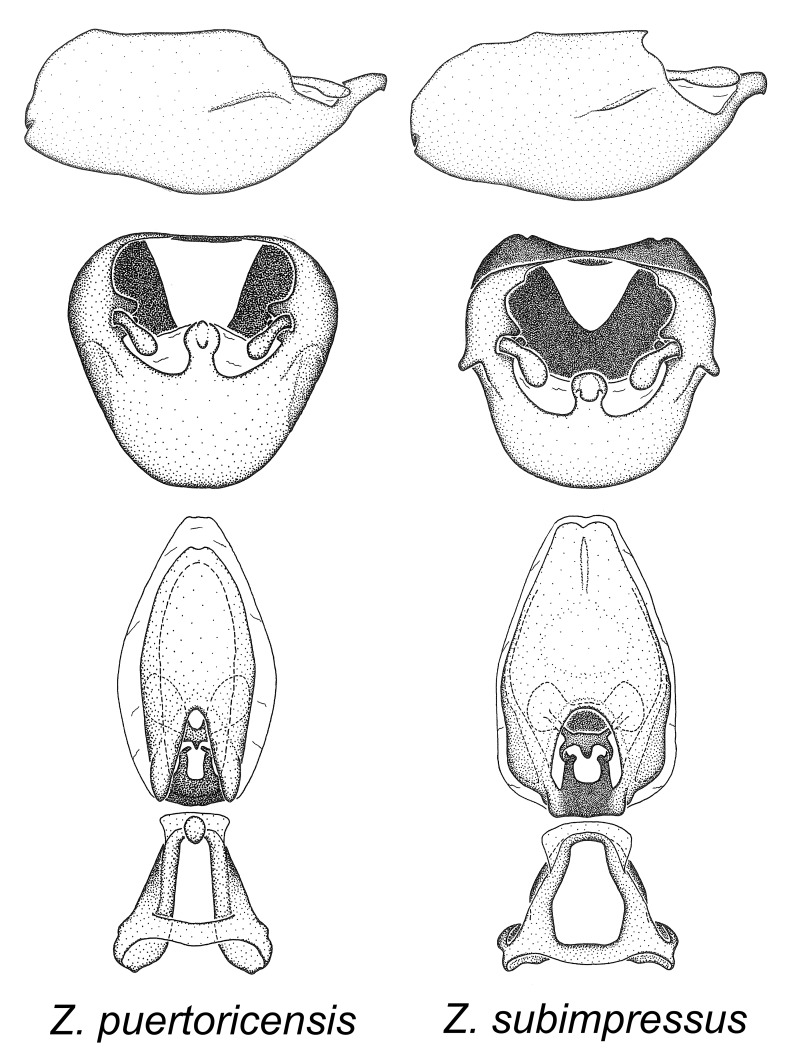
*Zelus puertoricensis* species group, male genitalic structures

Members of this group are restricted to the Caribbean. They can be easily recognized by the rather slender body form. The posteriorly directed, robust medial process with a somewhat blunt apical protrusion is also distinctive of this group. The basal plate arms are widely separate and diverging and these features are rare in other species in the genus. They show resemblance to species of the *Zelus renardii* species group, especially to *Z. cervicalis*. *Zelus bruneri* was not physically examined, but the rather slender body form as seen in the original illustration places it within this group. Comparative views of male genitalia are shown in Fig. 6.

6. *Zelus renardii* species group.

*Zelus cervicalis* Stål, 1872 and *Zelus renardii* Kolenati, 1856.

**Figure 7.**
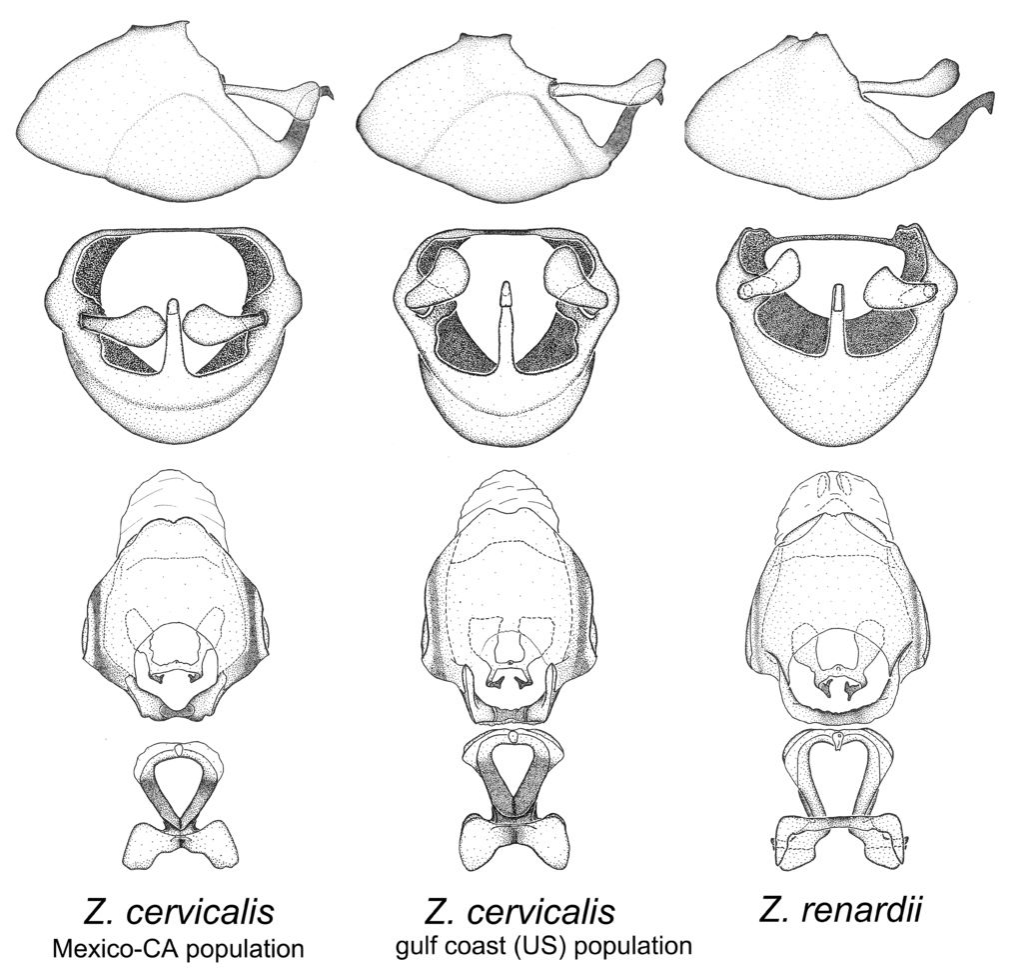
*Zelus renardii* species group, male genitalic structures

The two members of this group are very likely sister species since they share a number of unique characters: the apex of the medial process is greatly bent ventrad and hooklike, the lateral margin of the dorsal phallothecal sclerite is recurved dorsad and the basal part of the strut is absent. Both species are mainly distributed in North and Central America, but *Z. cervicalis* extends to northern South America. Comparative views of male genitalia are shown in Fig. 7 .

7. *Zelus armillatus* species group.

*Zelus amblycephalus* sp. n., *Zelus annulosus* (Stål, 1866), *Zelus armillatus* (Lepeletier & Serville, 1825), *Zelus conjungens* (Stål, 1860), *Zelus janus* Stål, 1862, *Zelus leucogrammus* (Perty, 1833), *Zelus lewisi* sp. n., *Zelus litigiosus* Stål, 1862, *Zelus ruficeps* Stål, 1862, *Zelus sulcicollis* Champion, 1899, *Zelus umbraculoides* sp. n. and *Zelus umbraculus* sp. n.

**Figure 8.**
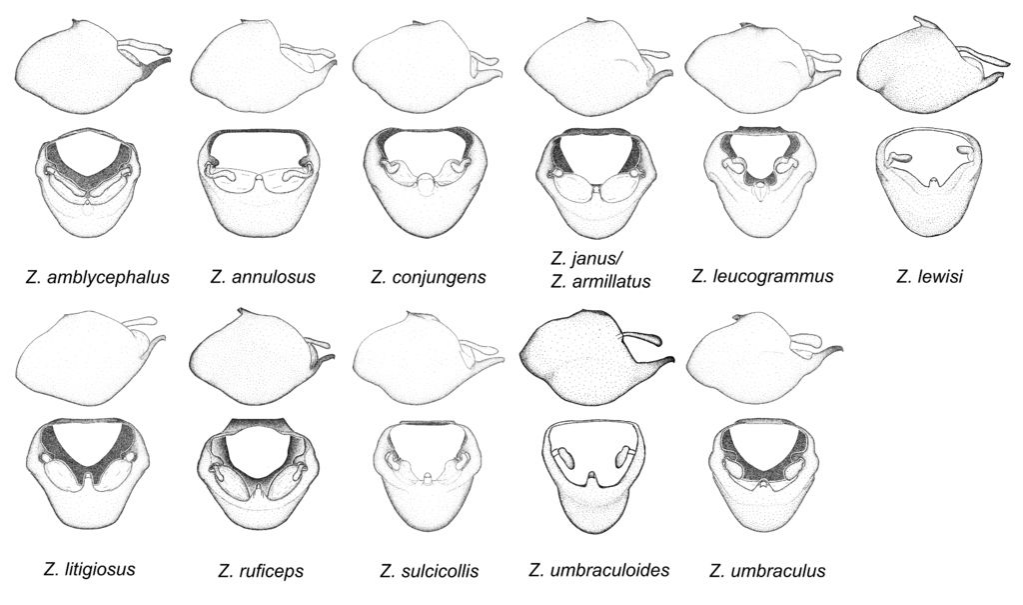
*Zelus armillatus* species group, male genitalic structures - pygophore

**Figure 9.**
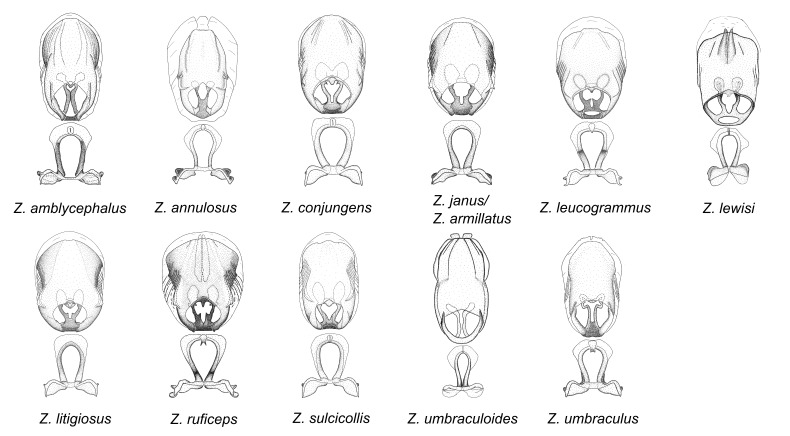
*Zelus armillatus* species group, male genitalic structures - phallus

This is one of the two largest groups in the genus (the other being the *Zelus panamensis* species group). Species in this group are generally robust and large-sized (15-25 mm), and some are among the largest in the genus. The most distinctive character is that of the medial process, which has the apex slightly projected into two minute small lateral prongs or processes. This condition is different from that in several species groups listed below, where the apex of the medial process is hook-like and more strongly projected. The lateral spine of the humeral angle tends to be pronounced and somewhat broadened into a dentate effect. The pygophore is large, rounded, and somewhat shortened relative to the total length of the individual. The dorsal phallothecal sclerite having dorsolateral expansions or projections close to the basal arm is also unique to some species of this group. This condition, however, is not seen in *Z. amblycephalus*, *Z. umbraculus*, or *Z. umbraculoides*, which appear to be divergent from the remainders of the group, but the features of the medial process unambiguously place them in this species group. Comparative views of male genitalia are shown in Figs 8, 9.

8. *Zelus longipes* species group.

*Zelus bahiaensis* sp. n., *Zelus errans* Fabricius, 1803, *Zelus longipes* (Linnaeus, 1767), 1803 and *Zelus vespiformis* Hart, 1987.

**Figure 10.**
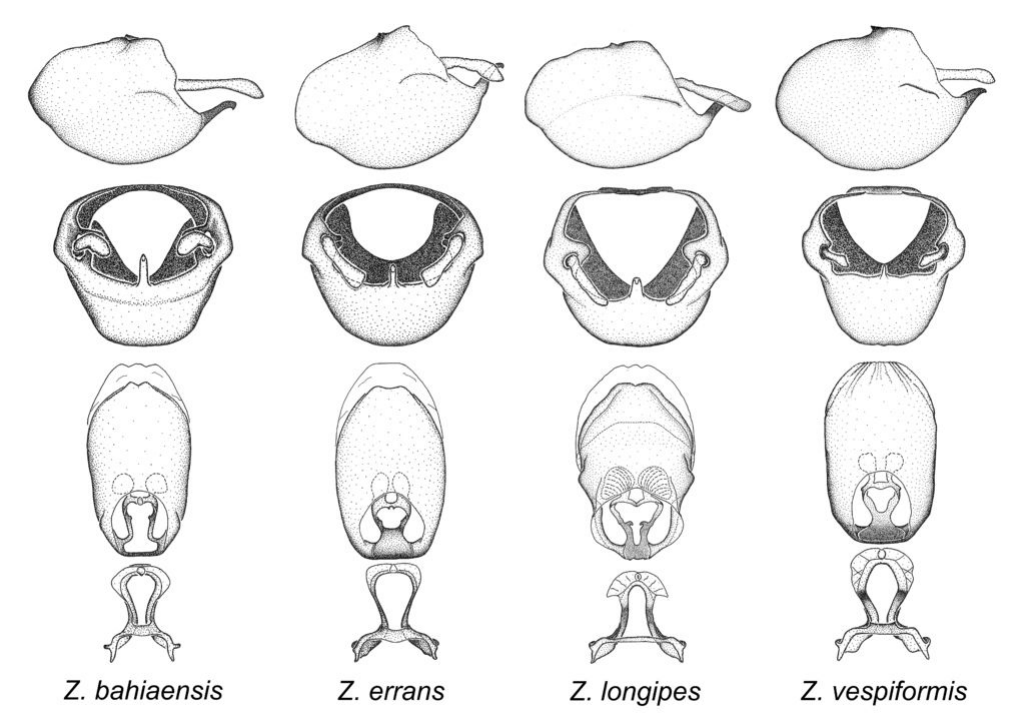
*Zelus longipes* species group, male genitalic structures

This and the next species group (*Zelus vagans* species group) possess dense, spine-like setae on the head and pronotum, and a rounded, unarmed humeral angle, both characters rather unique in *Zelus* and probably synapomorphies uniting the two groups. The former character is possibly homoplastic as it is also seen in two species in the *Zelus armillatus* species group. The medial process is slender and cylindrical and this condition is among the most extreme in the genus. It is semi-erect and posteriorly directed. The paramere exceeds the apex of the medial process. The dorsal phallothecal sclerite has subparallel margins and lacks obvious modifications or ornamentations (except for small lateral folds in *Z. longipes*). Some individuals of *Z. errans* and *Z. vespiformis* appear to be wasp mimics. Comparative views of male genitalia are shown in Fig. 10.

*9. Zelus vagans* species group.

*Zelus aithaleos, Zelus championi* sp. n., *Zelus fuliginatus* sp. n., *Zelus gracilipes* sp. n. and *Zelus vagans* Fabricius, 1803.

**Figure 11.**
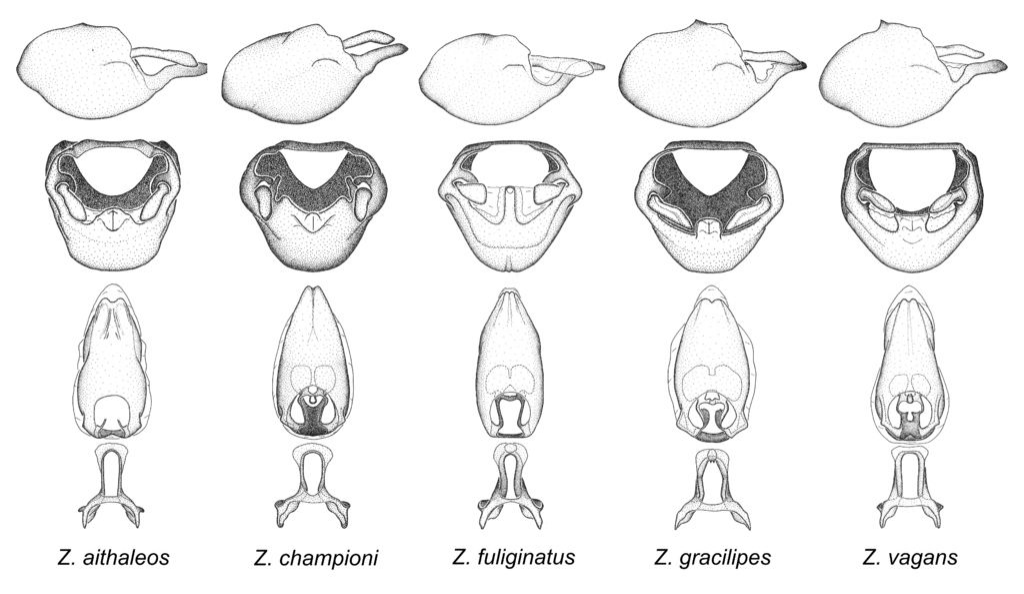
*Zelus vagans* species group, male genitalic structures

Species of the *Zelus vagans* group share two characters also present in the preceding group (*Zelus longipes* species group): spinelike setae and rounded humeral angle. However, they differ in the structure of the male genitalia in significant ways. The medial process shapes like a somewhat laterally flattened cone. It is relatively broad at base, narrowing toward the apex, and is laterally compressed. The medial process is posteriorly directly, nearly horizontal. The paramere is removed from or barely reaching apex of the medial process. Furthermore, the phallus is elongated and slightly constricted toward the apex (not conspicuous in *Z. gracilipes*). *Zelus vagans* and *Z. gracilipes* also resemble wasps to some extent, but both not as perfectly as seen in *Z. errans* and *Z. vespiformis*. Comparative views of male genitalia are shown in Fig. 11.

10. *Zelus panamensis* species group.

*Zelus banksi* sp. n., *Zelus cordazulus* sp. n., *Zelus filicauda* Bergroth, 1893, *Zelus gilboventris* sp. n., *Zelus korystos* Hart, 1986, *Zelus nigromaculatus* Champion, 1899, *Zelus panamensis* sp. n., *Zelus truxali* sp. n., *Zelus varius* (Herrich-Schaeffer, 1853) and *Zelus xouthos* sp. n.

**Figure 12.**
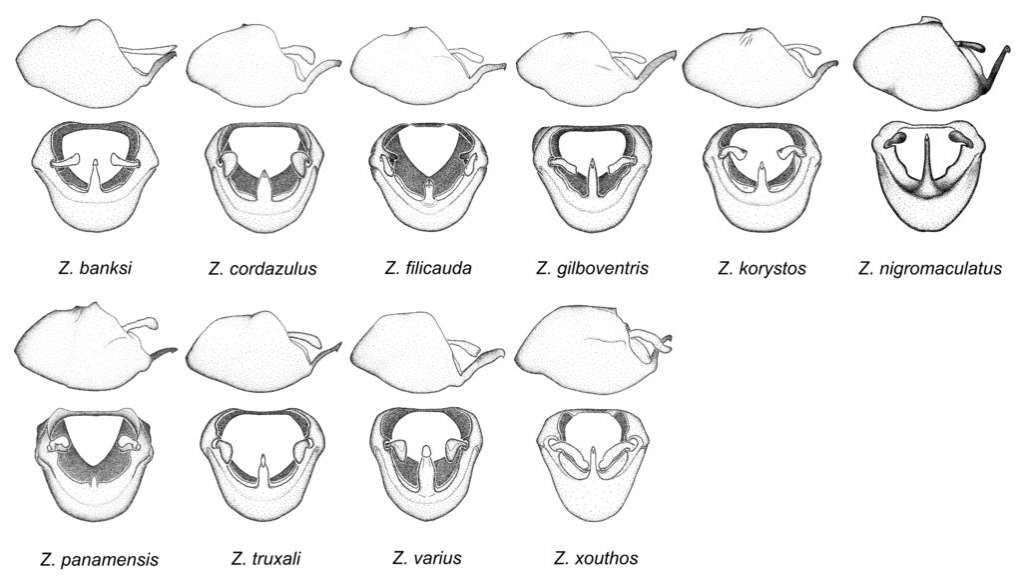
*Zelus panamensis* species group, male genitalic structures - pygophore

**Figure 13.**
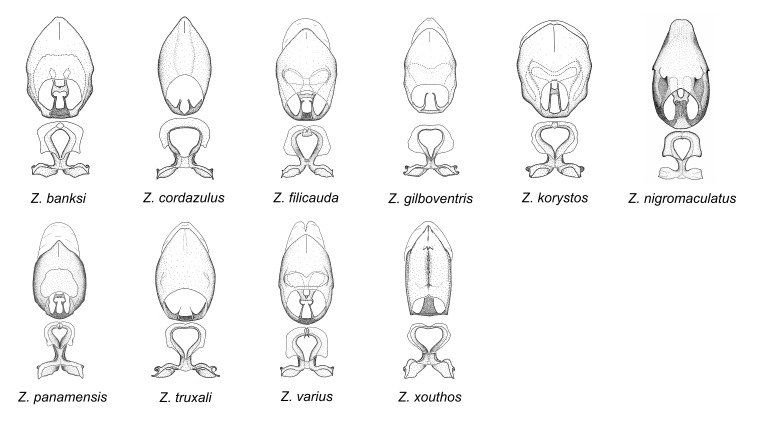
*Zelus panamensis* species group, male genitalic structures - phallus

This is another large group with ten species. Interestingly, most (seven) are new species. It is characterized by having an acute apical modification usually in the shape of a hook on the medial process and the conspicuous medial carination of the apical part of the dorsal phallothecal sclerite. The condition of the apical modification of the medial process differs from that in the *Zelus armillatus* species group in that it is much more prominent, usually acute and sometimes extending further ventrally. Rugulosity of the posterior pronotal lobe is highly pronounced relative to the other groups. Sexual dimorphism is pronounced in some species in this group (e.g., *Z. gilboventris* and *Z. truxali*). Most species in this group are concentrated in southern Central America and northern South America. Comparative views of male genitalia and habitus images are in Figs 12, 13.

11. *Zelus erythrocephalus* species group.

*Zelus auralanus* sp. n., *Zelus casii* sp. n., *Zelus chamaeleon* Stål, 1872, ​*Zelus erythrocephalus* Fabricius, 1803, *Zelus kartabenoides* sp. n., *Zelus kartabensis* Haviland, 1931, *Zelus laticornis* (Herrich-Schaeffer, 1853), *Zelus mattogrossensis* Wygodzinsky, 1947, *Zelus paracephalus* sp. n., *Zelus russulumus* sp. n. and *Zelus versicolor* (Herrich-Schaeffer, 1848).

**Figure 14.**
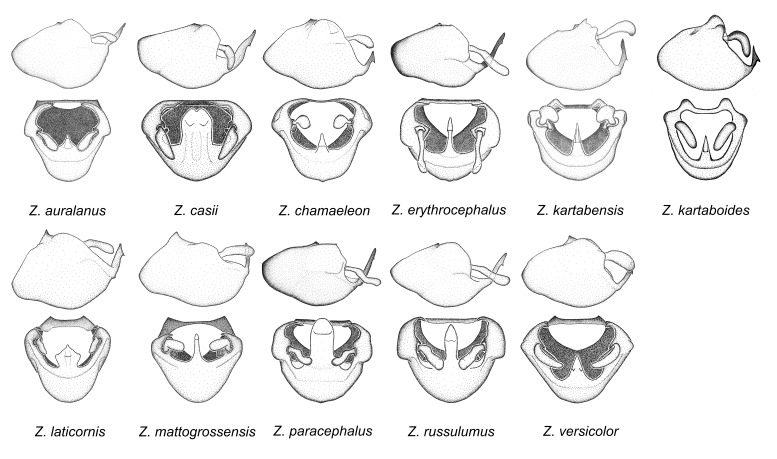
*Zelus erythrocephalus* species group, male genitalic structures - pygophore

**Figure 15.**
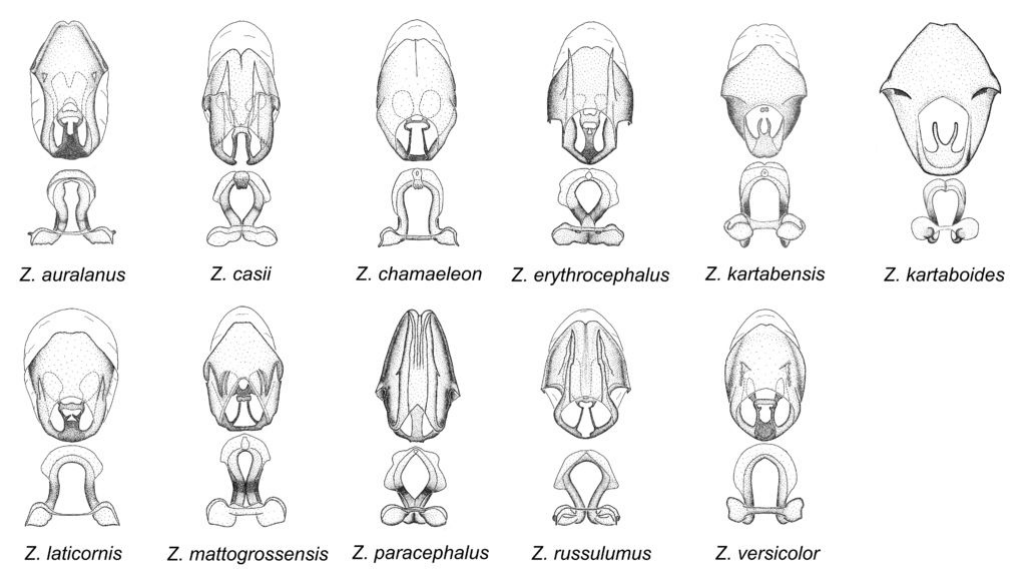
*Zelus erythrocephalus* species group, male genitalic structures - phallus

Two diagnostic characters identify members of this group. The medial process possesses a broad ridge-like projection or carina that initiates from the apex and extends ventrally or is removed from apex. The second feature is the apically oriented lateral sharp processes or projections on the dorsal phallothecal sclerite. These are not to be confused with the lateral expansion seen in the *Zelus armillatus* species group, where the direction of the expansion is laterad. In *Z. auralanus* and *Z. versicolor*, this process is short and somewhat dorsally directed, rather than apically directed. Three species, *Z. kartabenoides*, *Z. kartabensis* and *Z. chamaeleon* lack this structure. Their placement in this group is primarily based on the configuration of the medial process and the absence of characters of other groups. Also, the longitudinal ridge-like elevation or hook on the medial process is similar to the condition in another species, *Z. laticornis*, although the latter has a short modification. In this species group the parameres are usually somewhat bulbous and curved medially with moderate to long erect setae on the apical 1/2. The medial process is broadened at base, and usually anteroposteriorly compressed. Furthermore, the basal plate of the phallus is strongly curved in some members of this species group. Pronounced sexual dimorphism is seen in some species of this group. Notably, three species, *Z. erythrocephalus*, *Z. paracephalus* and *Z. russulumus* have purple, blue or greenish iridescence on the membrane of the hemelytron. Species of this group show a predominant southern South American distribution, with a few found only from the Amazons. Comparative views of male genitalia and habitus images are in Figs 14, 15.

Because of the heavy emphasis on male genitalic characters for grouping species, four species described only from females are not placed in any of the species groups defined in the above. These are: *Zelus fasciatus* Champion, 1899, *Zelus plagiatus* (Signoret, 1852), *Zelus sphegeus* Fabricius, 1803 and *Zelus means* Fabricius, 1803. *Zelus fasciatus* is similar to the females of some of the species in the *Zelus panamensis* species group and also occurs in an overlapping geographical region (southern Central America). *Zelus plagiatus and Z. sphegeus* show resemblance to the females of *Z. versicolor*, which is in the *Zelus erythrocephalus* species group. *Zelus means*, by possessing a rounded humeral angle and spinelike setae, aligns most closely with the *Zelus vagans* species group and the *Zelus longipes* species group. A future cladistic analysis, including morphological and molecular data, is needed to test the monophyly of these species groups and may also have the potential to place these female-based species.

#### Species removed from Zelus

Five species are removed from *Zelus*: *Z. araneiformis*, *Zelus gradarius* Bergroth, 1905, *Z. modestus* (Stål, 1862), *Zelus subfasciatus* Stål, 1860 and *Zelus vittaticeps* Stål, 1866. These species represent an undescribed genus "Hartzelus" and will be treated in a separate study. They will be listed as Harpactorini *incertae sedis* until their generic placement is formally clarified.

### Zelus aithaleos

Zhang & Hart, 2016 sp. n.

urn:lsid:zoobank.org:act:FD071442-BEAC-46E0-80E3-4A7CCCBCE0F3

#### Materials

- **Type status:** Holotype. **Occurrence:** catalogNumber: UCR_ENT 00047314; recordedBy: J. C. Pallister; sex: Adult Male; **Taxon:** scientificName: Zelus aithaleos; family: Reduviidae; genus: Zelus; scientificNameAuthorship: Zhang & Hart, 2016; **Location:** country: PERU; stateProvince: Huanuco; locality: Aerro Puerto, Tingo Maria; verbatimElevation: 671 m; decimalLatitude: -9.3; decimalLongitude: -76.01666; georeferenceSources: Gazetteer; **Identification:** identifiedBy: G. Zhang; dateIdentified: 2013; **Event:** eventDate: 1946-10-22; **Record Level:** institutionCode: AMNH
- **Type status:** Paratype. **Occurrence:** catalogNumber: UCR_ENT 00009327; recordedBy: L. Pena; sex: Adult Female; **Taxon:** scientificName: Zelus aithaleos; family: Reduviidae; genus: Zelus; scientificNameAuthorship: Zhang & Hart, 2016; **Location:** country: BOLIVIA; stateProvince: La Paz; locality: Guanay; decimalLatitude: -15.4833; decimalLongitude: -67.8833; georeferenceSources: Gazetteer; **Identification:** identifiedBy: G. Zhang; dateIdentified: 2013; **Event:** eventDate: 1993-10-01 to 1993-11-01; **Record Level:** institutionCode: USNM
- **Type status:** Paratype. **Occurrence:** catalogNumber: UCR_ENT 00009328; recordedBy: L. Pena; sex: Adult Female; **Taxon:** scientificName: Zelus aithaleos; family: Reduviidae; genus: Zelus; scientificNameAuthorship: Zhang & Hart, 2016; **Location:** country: BOLIVIA; stateProvince: La Paz; locality: Guanay; decimalLatitude: -15.4833; decimalLongitude: -67.8833; georeferenceSources: Gazetteer; **Identification:** identifiedBy: G. Zhang; dateIdentified: 2013; **Event:** eventDate: 1993-10-01 to 1993-11-01; **Record Level:** institutionCode: USNM
- **Type status:** Paratype. **Occurrence:** catalogNumber: UCR_ENT 00071251; recordedBy: Unknown; sex: Adult Female; **Taxon:** scientificName: Zelus aithaleos; family: Reduviidae; genus: Zelus; scientificNameAuthorship: Zhang & Hart, 2016; **Location:** country: BRAZIL; stateProvince: Goias; locality: Annapolis; decimalLatitude: -16.3333; decimalLongitude: -48.9667; georeferenceSources: Gazetteer; **Identification:** identifiedBy: G. Zhang; dateIdentified: 2013; **Event:** eventDate: 1936-02-07; **Record Level:** institutionCode: TAMU
- **Type status:** Paratype. **Occurrence:** catalogNumber: UCR_ENT 00071252; recordedBy: Foerster; sex: Adult Female; **Taxon:** scientificName: Zelus aithaleos; family: Reduviidae; genus: Zelus; scientificNameAuthorship: Zhang & Hart, 2016; **Location:** country: PARAGUAY; stateProvince: Guaira; locality: Paso-Yobai; verbatimElevation: 280 m; decimalLatitude: -25.72344; decimalLongitude: -55.9969; georeferenceSources: Google Earth; **Identification:** identifiedBy: G. Zhang; dateIdentified: 2013; **Event:** eventDate: 1951-09-28; **Record Level:** institutionCode: TAMU

#### Description

Figs 16, 17, 18

***Male***: (Fig. 16) Medium-sized, total length 13.47 mm (n=1, Suppl. material 2); slender. **COLORATION**: Entirely dark, nearly black; inconspicuous, light-colored, thin, medial longitudinal stripe on postocular lobe. Membrane of hemelytron semi-translucent. **VESTITURE**: Densely setose. Dorsum of anteocular lobe with moderately dense, short, recumbent and sparse, short , erect, spine-like setae. Dorsum of postocular lobe nearly glabrous; spine-like setae anteriorly between eyes; stripe of longitudinal whitish recumbent setae laterally. Ventral surface of head with moderately dense, recumbent setae, intermixed with erect setae. Scape nearly glabrous. Pronotum with dense, short, erect, stout, spine-like setae, also on lateral surfaces and pleura; scutellum with dense, apically curved, stout setae. Legs with sparse setation. Sundew setae on profemur sparse. Abdomen with moderately dense, short, semi-erect, fine setae. Ventral surface of pypophore with sparse, long, erect setae; posteroventral rim with long, erect setae; Paramere apically with dense, short to long, erect setae. **STRUCTURE: Head**: Cylindrical, L/W = 2.30. Postocular lobe short; in dorsal view anteriorly gradually narrowing, posterior portion constant, slightly narrower. Eye moderately sized; dorsal margin attaining postocular transverse groove, ventral margin removed from ventral surface of head. *Labium*: I: II: III = 1: 1.7: 0.5 . Basiflagellomere diameter slightly larger than that of pedicel. **Thorax**: Anterolateral angle rounded, without projection; medial longitudinal sulcus evident throughout, deepening posteriorly. Posterior pronotal lobe with rugulose surface; disc distinctly elevated above humeral angle; humeral angle rounded, without projection. Scutellum long; apex angulate, slightly projected upward. *Legs*: Very slender. *Hemelytron*: Greatly surpassing apex of abdomen by about 3x length of abdominal segment seven; quadrate cell large and broad; Cu and M of cubital cell subparallel. **GENITALIA**: (Fig. 17) *Pygophore*: Elongate ovoid; mid-lateral fold adjacent to paramere insertion; slightly expanded laterally near base of paramere in dorsal view. Medial process somewhat laterally compressed, cone-shaped; anterior and posterior surfaces angulate medially; long, nearly as long as paramere; posteriorly directed, nearly horizontal; basally slightly curved; apex in posterior view blunt, without modification. *Paramere*: Cylindrical; moderately long, not reaching apex of medial process; directed posteriad, slightly curved towards medial process; nearly straight; apical part slightly enlarged, depression along inner side. *Phallus*: Dorsal phallothecal sclerite elongated; slightly constricted near middle; apical 1/3 of phallothecal sclerite tapering to apex, dorsal surface strongly convex; apex medially notched; posterior margin of foramen deeply concave. Struts attached to dorsal phallothecal sclerite; apically separate, connected by bridge; basally separate. Basal plate arm extremely slender; separate; subparallel; bridge short; extension of basal plate well developed, only slightly expanded laterally.

***Female***: Similar to male, except for the following. Larger than male, total length 13.87–17.61 mm (mean 16.27 mm, Suppl. material 2). Abdomen expanded beyond margins of wings. Metafemoral diameter smallest, mesofemoral diameter significantly larger than that of profemur. Occasional specimens with orange posterior pronotal lobe and mesopleuron. Setae on some specimens golden.

#### Diagnosis

The nearly colorless cells of the membrane of the hemelytron contrast markedly with the dark veins, making *Z. aithaleos* an easily recognizable species in this genus. Also recognized by the following combination of characters: the postocular lobe short, 1.7x of the length of anteocular lobe in males and 1.2x in females; the anterior pronotal lobe short, abbreviated; the pronotum strongly convex; the humeral angle of pronotum rounded, unarmed; the cranium, the pronotum, the pleura and the scutellum with spinelike, short, stout setae (the last two characters also seen in the *Zelus longipes* species group and the *Zelus vagans* species group).

Males can also be recognized by the medial process laterally compressed, posteriorly directly and almost horizontal (also seen in the *Zelus vagans* species group). Within the *Zelus vagans* species group (Fig. 11), the medial process of *Z. aithaleos* is comparatively long, exceeding 1/2 length of the main body of the pygophore, whereas all other species in this group have the medial process less than 1/2 length of the pygophore. The basal plate arm is remarkably more slender than those in the same species group.

A unicolourous near-black dorsum, including the head, the pronotum and the corium, separates *Z. aithaleos* from both sexes of *Z. gracilipes*, *Z. vagans*, and *Z. means* (known from females only), all of which have some orange, yellow or reddish colors. The dark dorsal profile is shared with *Z. championi* (only the male is known) and *Z. fuliginatus*. A longitudinal lateral patch of whitish recumbent setae on the postocular lobe serves to separate this species from *Z. fuliginatus*. It is distinguished by a dark abdomen from *Z. championi*, which has a brightly red abdomen.

#### Etymology

From Greek *aithales*.

#### Distribution

**Figure 18.**
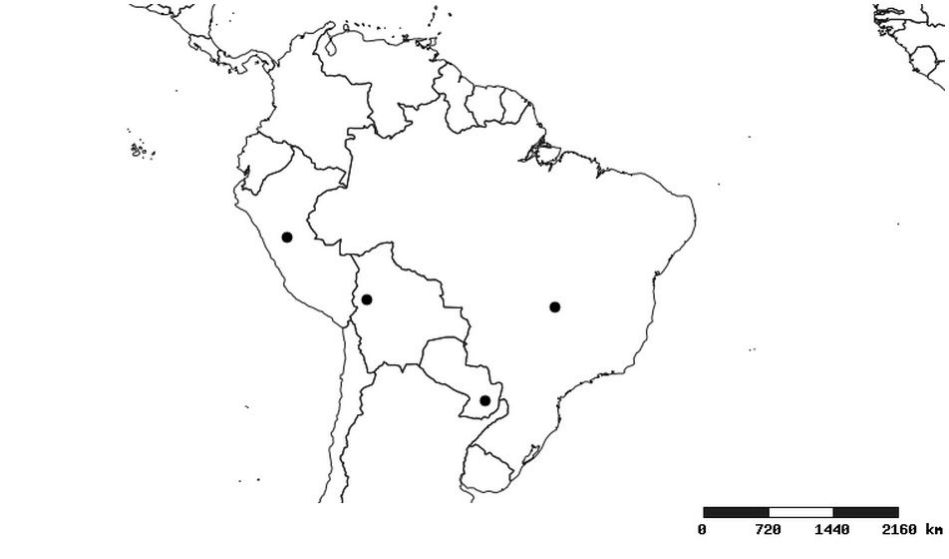
*Zelus aithaleos* Zhang & Hart, sp. n., specimen record map

South America (Fig. 18). Countries with specimen records: Bolivia, Brazil, Paraguay and Peru.

### Zelus amblycephalus

Zhang & Hart, 2016 sp. n.

urn:lsid:zoobank.org:act:EB0C6CBF-8CF9-4F9A-A095-B4E24EF8EE51

#### Materials

- **Type status:** Holotype. **Occurrence:** catalogNumber: UCR_ENT 00022669; occurrenceRemarks: Holotype of Zelus amblycephalus Zhang and Hart, 2016; recordedBy: A. S. Menke; sex: Adult Male; otherCatalogNumbers: LACM ENT 160232; **Taxon:** scientificName: Zelus amblycephalus; family: Reduviidae; genus: Zelus; scientificNameAuthorship: Zhang and Hart, 2016; **Location:** country: COSTA RICA; stateProvince: Puntarenas; locality: Golfito; decimalLatitude: 8.6407; decimalLongitude: -83.1686; georeferenceSources: Google Earth; **Identification:** identifiedBy: G. Zhang; dateIdentified: 2013; **Event:** eventDate: 1957-07-13; **Record Level:** institutionCode: LACM
- **Type status:** Paratype. **Occurrence:** catalogNumber: UCR_ENT 00009315; occurrenceRemarks: Paratype of Zelus amblycephalus Zhang and Hart, 2016; recordedBy: Unknown; sex: Adult Male; **Taxon:** scientificName: Zelus amblycephalus; family: Reduviidae; genus: Zelus; scientificNameAuthorship: Zhang and Hart, 2016; **Location:** country: BRAZIL; stateProvince: Amazonas; locality: Rio Janauaca, 40 km SW Manaus; decimalLatitude: -3.33333; decimalLongitude: -60.28333; georeferenceSources: Label; **Identification:** identifiedBy: G. Zhang; dateIdentified: 2013; **Event:** eventDate: 1979-03-10; **Record Level:** institutionCode: USNM
- **Type status:** Paratype. **Occurrence:** catalogNumber: UCR_ENT 00009316; occurrenceRemarks: Paratype of Zelus amblycephalus Zhang and Hart, 2016; recordedBy: Unknown; sex: Adult Male; **Taxon:** scientificName: Zelus amblycephalus; family: Reduviidae; genus: Zelus; scientificNameAuthorship: Zhang and Hart, 2016; **Location:** country: BRAZIL; stateProvince: Amazonas; locality: Teffe; decimalLatitude: -3.3667; decimalLongitude: -64.7; **Identification:** identifiedBy: G. Zhang; dateIdentified: 2013; **Event:** eventDate: 1918-12-06; **Record Level:** institutionCode: USNM
- **Type status:** Paratype. **Occurrence:** catalogNumber: UCR_ENT 00006070; occurrenceRemarks: Paratype of Zelus amblycephalus Zhang and Hart, 2016; recordedBy: B. Malkin; sex: Adult Male; **Taxon:** scientificName: Zelus amblycephalus; family: Reduviidae; genus: Zelus; scientificNameAuthorship: Zhang and Hart, 2016; **Location:** country: BRAZIL; stateProvince: Mato Grosso; locality: Barra do Tapirape; decimalLatitude: -10.46666; decimalLongitude: -50.51667; georeferenceSources: Gazetteer; **Identification:** identifiedBy: G. Zhang; dateIdentified: 2013; **Event:** eventDate: 1962-12-30; **Record Level:** institutionCode: CAS
- **Type status:** Paratype. **Occurrence:** catalogNumber: UCR_ENT 00029368; occurrenceRemarks: Paratype of Zelus amblycephalus Zhang and Hart, 2016. Drake Collection; recordedBy: J. E. Eger; sex: Adult Male; **Taxon:** scientificName: Zelus amblycephalus; family: Reduviidae; genus: Zelus; scientificNameAuthorship: Zhang and Hart, 2016; **Location:** country: BRAZIL; stateProvince: Rondonia; locality: 62 km SW of Ariquemes, near Fzda. Rancho Grande; decimalLatitude: -10.32921; decimalLongitude: -63.46881; **Identification:** identifiedBy: G. Zhang; dateIdentified: 2013; **Event:** eventDate: 1996-12-03 to 1996-12-15; **Record Level:** institutionCode: USNM
- **Type status:** Paratype. **Occurrence:** catalogNumber: UCR_ENT 00025328; occurrenceRemarks: Paratype of Zelus amblycephalus Zhang and Hart, 2016; recordedBy: C. Ardila, A. Montano, A. Pachon; sex: Adult Male; **Taxon:** scientificName: Zelus amblycephalus; family: Reduviidae; genus: Zelus; scientificNameAuthorship: Zhang and Hart, 2016; **Location:** country: COLOMBIA; stateProvince: Cundinamarca; locality: Villeta; verbatimElevation: 799 m; decimalLatitude: 5.01444; decimalLongitude: -74.47305; georeferenceSources: Label; **Identification:** identifiedBy: G. Zhang; dateIdentified: 2013; **Event:** eventDate: 2003-05-10; **Record Level:** institutionCode: UNAB
- **Type status:** Paratype. **Occurrence:** catalogNumber: UCR_ENT 00022670; occurrenceRemarks: Paratype of Zelus amblycephalus Zhang and Hart, 2016. Previously designated as 'allotype' of his manuscript name Zelus amblycephalus by Hart, a type status not used in the formal publication of this name (Zhang, Hart & Weirauch, 2016).; recordedBy: A. S. Menke; sex: Adult Female; otherCatalogNumbers: LACM ENT 160233; **Taxon:** scientificName: Zelus amblycephalus; family: Reduviidae; genus: Zelus; scientificNameAuthorship: Zhang and Hart, 2016; **Location:** country: COSTA RICA; stateProvince: Puntarenas; locality: Golfito; decimalLatitude: 8.6407; decimalLongitude: -83.1686; georeferenceSources: Google Earth; **Identification:** identifiedBy: G. Zhang; dateIdentified: 2013; **Event:** eventDate: 1957-07-13; **Record Level:** institutionCode: LACM
- **Type status:** Paratype. **Occurrence:** catalogNumber: UCR_ENT 00009473; occurrenceRemarks: Paratype of Zelus amblycephalus Zhang and Hart, 2016; recordedBy: T. L. Erwin et al.; sex: Adult Male; **Taxon:** scientificName: Zelus amblycephalus; family: Reduviidae; genus: Zelus; scientificNameAuthorship: Zhang and Hart, 2016; **Location:** country: ECUADOR; stateProvince: Orellana; locality: Reserva Etnica Waorani, 1 km S. Onkone Gare Camp, Transect Ent.; verbatimElevation: 216 m; decimalLatitude: -0.65714; decimalLongitude: -76.453; georeferenceSources: Label; **Identification:** identifiedBy: G. Zhang; dateIdentified: 2013; **Event:** samplingProtocol: Fogging; eventDate: 1994-10-09; **Record Level:** institutionCode: USNM
- **Type status:** Paratype. **Occurrence:** catalogNumber: UCR_ENT 00010840; occurrenceRemarks: Paratype of Zelus amblycephalus Zhang and Hart, 2016; recordedBy: A. Lewis; sex: Adult Male; **Taxon:** scientificName: Zelus amblycephalus; family: Reduviidae; genus: Zelus; scientificNameAuthorship: Zhang and Hart, 2016; **Location:** country: MEXICO; stateProvince: Chiapas; locality: 10 m N of Mexico 190 Tuztla Gutierrez; decimalLatitude: 16.90574; decimalLongitude: -93.16486; georeferenceSources: Google Earth; **Identification:** identifiedBy: G. Zhang; dateIdentified: 2013; **Event:** eventDate: 1956-08-24 to 1956-08-28; **Record Level:** institutionCode: LACM
- **Type status:** Paratype. **Occurrence:** catalogNumber: UCR_ENT 00034277; occurrenceRemarks: Paratype of Zelus amblycephalus Zhang and Hart, 2016; recordedBy: G. Ortega, E. Barrera, A. Casasola; sex: Adult Female; **Taxon:** scientificName: Zelus amblycephalus; family: Reduviidae; genus: Zelus; scientificNameAuthorship: Zhang and Hart, 2016; **Location:** country: MEXICO; stateProvince: Chiapas; locality: Reserva El Ocote; decimalLatitude: 16.99502; decimalLongitude: -93.64056; georeferenceSources: Google Earth; **Identification:** identifiedBy: G. Zhang; dateIdentified: 2013; **Event:** eventDate: 1993-12-02 to 1993-12-10; **Record Level:** institutionCode: IBUNAM
- **Type status:** Paratype. **Occurrence:** catalogNumber: UCR_ENT 00009493; occurrenceRemarks: Paratype of Zelus amblycephalus Zhang and Hart, 2016. Drake Collection; recordedBy: D.H. Jansen; sex: Adult Male; **Taxon:** scientificName: Zelus amblycephalus; family: Reduviidae; genus: Zelus; scientificNameAuthorship: Zhang and Hart, 2016; **Location:** country: MEXICO; stateProvince: Oaxaca; locality: Temascal; decimalLatitude: 18.23882; decimalLongitude: -96.40034; **Identification:** identifiedBy: G. Zhang; dateIdentified: 2013; **Event:** eventDate: 1963-10-31; **Record Level:** institutionCode: USNM
- **Type status:** Paratype. **Occurrence:** catalogNumber: UCR_ENT 00009494; occurrenceRemarks: Paratype of Zelus amblycephalus Zhang and Hart, 2016. Drake Collection; recordedBy: D.H. Jansen; sex: Adult Male; **Taxon:** scientificName: Zelus amblycephalus; family: Reduviidae; genus: Zelus; scientificNameAuthorship: Zhang and Hart, 2016; **Location:** country: MEXICO; stateProvince: Oaxaca; locality: Temascal; decimalLatitude: 18.23882; decimalLongitude: -96.40034; **Identification:** identifiedBy: G. Zhang; dateIdentified: 2013; **Event:** eventDate: 1963-10-31; **Record Level:** institutionCode: USNM
- **Type status:** Paratype. **Occurrence:** catalogNumber: UCR_ENT 00009495; occurrenceRemarks: Paratype of Zelus amblycephalus Zhang and Hart, 2016. Drake Collection; recordedBy: D.H. Jansen; sex: Adult Male; **Taxon:** scientificName: Zelus amblycephalus; family: Reduviidae; genus: Zelus; scientificNameAuthorship: Zhang and Hart, 2016; **Location:** country: MEXICO; stateProvince: Oaxaca; locality: Temascal; decimalLatitude: 18.23882; decimalLongitude: -96.40034; **Identification:** identifiedBy: G. Zhang; dateIdentified: 2013; **Event:** eventDate: 1963-10-31; **Record Level:** institutionCode: USNM
- **Type status:** Paratype. **Occurrence:** catalogNumber: UCR_ENT 00009496; occurrenceRemarks: Paratype of Zelus amblycephalus Zhang and Hart, 2016. Drake Collection; recordedBy: D.H. Jansen; sex: Adult Male; **Taxon:** scientificName: Zelus amblycephalus; family: Reduviidae; genus: Zelus; scientificNameAuthorship: Zhang and Hart, 2016; **Location:** country: MEXICO; stateProvince: Oaxaca; locality: Temascal; decimalLatitude: 18.23882; decimalLongitude: -96.40034; **Identification:** identifiedBy: G. Zhang; dateIdentified: 2013; **Event:** eventDate: 1963-10-31; **Record Level:** institutionCode: USNM
- **Type status:** Paratype. **Occurrence:** catalogNumber: UCR_ENT 00009497; occurrenceRemarks: Paratype of Zelus amblycephalus Zhang and Hart, 2016. Drake Collection; recordedBy: D.H. Jansen; sex: Adult Male; **Taxon:** scientificName: Zelus amblycephalus; family: Reduviidae; genus: Zelus; scientificNameAuthorship: Zhang and Hart, 2016; **Location:** country: MEXICO; stateProvince: Oaxaca; locality: Temascal; decimalLatitude: 18.23882; decimalLongitude: -96.40034; **Identification:** identifiedBy: G. Zhang; dateIdentified: 2013; **Event:** eventDate: 1963-10-31; **Record Level:** institutionCode: USNM
- **Type status:** Paratype. **Occurrence:** catalogNumber: UCR_ENT 00009270; occurrenceRemarks: Paratype of Zelus amblycephalus Zhang and Hart, 2016. Additional label: Collected at Night.; recordedBy: J. Zetek; sex: Adult Male; otherCatalogNumbers: 41-7231; **Taxon:** scientificName: Zelus amblycephalus; family: Reduviidae; genus: Zelus; scientificNameAuthorship: Zhang and Hart, 2016; **Location:** country: PANAMA; stateProvince: Canal Zone; locality: Barro Colorado; decimalLatitude: 9.16666; decimalLongitude: -79.83333; georeferenceSources: Google Earth; **Identification:** identifiedBy: G. Zhang; dateIdentified: 2013; **Event:** eventDate: 1941-04-01; **Record Level:** institutionCode: USNM
- **Type status:** Paratype. **Occurrence:** catalogNumber: UCR_ENT 00023698; occurrenceRemarks: Paratype of Zelus amblycephalus Zhang and Hart, 2016; recordedBy: Geyskes; sex: Adult sex unknown; **Taxon:** scientificName: Zelus amblycephalus; family: Reduviidae; genus: Zelus; scientificNameAuthorship: Zhang and Hart, 2016; **Location:** country: SURINAME; stateProvince: Unknown; locality: unknown; decimalLatitude: 5.804157; decimalLongitude: -55.149886; **Identification:** identifiedBy: G. Zhang; dateIdentified: 2013; **Event:** eventDate: 1965-12-12; **Record Level:** institutionCode: RMNH
- **Type status:** Paratype. **Occurrence:** catalogNumber: UCR_ENT 00017182; occurrenceRemarks: Paratype of Zelus amblycephalus Zhang and Hart, 2016; recordedBy: P. & C. Vaurie; sex: Adult Female; **Taxon:** scientificName: Zelus amblycephalus; family: Reduviidae; genus: Zelus; scientificNameAuthorship: Zhang and Hart, 2016; **Location:** country: MEXICO; stateProvince: Chiapas; locality: Tuxtla Gutierrez; verbatimElevation: 549 m; decimalLatitude: 16.75469; decimalLongitude: -93.11485; **Identification:** identifiedBy: G. Zhang; dateIdentified: 2013; **Event:** eventDate: 1955-07-06 to 1955-07-10; **Record Level:** institutionCode: AMNH

#### Description

Figs 19, 20, 21

***Male***: (Fig. 19a, b) Large, total length 15.0–17.5 mm (mean 15.7 mm, Suppl. material 2); slender, body length/width = 4.5. **COLORATION**: Yellowish or greenish-brown. Head yellowish-brown to brown. Antennae and femoral apices reddish. Anterior pronotal lobe uniformly yellowish-brown or brown. Posterior pronotal lobe yellowish-brown or brown with lateral processes and surrounding area darker, brown to dark brown. Clavus and corium brown, veins yellowish-brown, membrane brown. Abdomen pale brown. Pygophore yellowish-brown. **VESTITURE**: Sparsely setose; sparse, short, erect setae over most of integument. Short spinelike setae on dorsal surface of head, with some short recumbent setae dorsally on posterior lobe, sparse short recument and erect setae on lateral and ventral surfaces. Anterior pronotal lobe with short, spine-like setae dorsally and short, erect and recumbent setae laterally; posterior lobe with short, recumbent and erect setae. Scutellum with short erect and recumbent setae. Corium and clavus with short, recumbent setae. Microtrichia throughout posterior margin of membrane of hemelytron. Abdominal venter with short, erect setae, interspersed with long setae. Sparse, moderately long setae on apical 1/2 of paramere. **STRUCTURE: Head**: Cylindrical. In dorsal view anteriorly gradually narrowing, posterior portion constant, slightly narrower; dorsal outline in lateral view gradually sloping. Eye prominent; dorsal and ventral margins removed from outlines of head. *Labium*: I: II: III = 1: 1.4: 0.3. Segment I surpassing anterior margin of eye. *Antenna*: Basiflagellomere diameter larger than that of pedicel. **Thorax**: Anterior pronotal lobe with indistinct collar, anterolateral angle rounded, without projection; medial longitudinal sulcus evident throughout, deepening posteriorly; slightly raised inconspicuous protuberance laterad to longitudinal medial sulcus anterior to transverse pronotal sulcus, more apparent in lateral view. Posterior pronotal lobe with smooth surface; disc distinctly elevated above humeral angle, slightly convex; humeral angle slightly expanded and wider than abdomen, armed with spinous process; margin indistinct, convex. Scutellar apex angulate, not projected. *Legs*: Moderately robust. *Hemelytron*: Surpassing apex of abdomen by about length of abdominal segment seven; costal margin somewhat concave; Sc surpassing level of apex of cubital cell; quadrate cell small and slender; 1A and Pcu intersecting; Cu and M of cubital cell subparallel. **Abdomen**: Segments of sub-equal sizes; segment seven much shorter than preceding segments, posterior margin in lateral view slightly concave. **GENITALIA**: (Fig. 20) Segment eight with nearly straight posterior margin, slightly concave in middle. *Pygophore*: Ovoid; posterolateral rim in lateral view straight above paramere, concave below paramere. Slender. Medial process cylindrical, slender; long; posteriorly directed, in less than forty-five degree with body axis; nearly straight; apex rounded. *Paramere*: Cylindrical; long, surpassing medial process; directed posteriad; not distinctly curved; apical part very slightly enlarged. *Phallus*: Surface flat; laterally indistinctly angulate; apex truncate; posterior margin of foramen broadly concave; basal arm short. Struts attached to dorsal phallothecal sclerite; basally separate. Basal plate arm robust; separate; basally converging; in lateral view nearly straight, very slightly curved; bridge moderately long, slender; extension expanded and extended onto arm.

***Female***: (Fig. 19c, d) Similar to male, except for the following. Pleura and abdominal segments with patches of whitish exudation. Basiflagellomere diameter smaller or subequal to that of pedicel. Hemelytron barely surpassing apex of abdomen.

#### Diagnosis

Can be recognized by the uniform pale coloration, the unpatterned legs (Fig. 19), and the relatively large size (>15mm, Suppl. material 2). Males can also be recognized by the apex of medial process with two minute prongs; the long, somewhat recurved paramere, which, viewed laterally, is at least 1.5x length of the medial process; and the dorsal phallothecal sclerite without lateral expansion close to the basal arm.

Among species of the *Zelus armillatus* group (Fig. 8), only *Z. annulosus* also possesses a paramere much longer than the medial process, but the two can be easily separated by the general aspects of coloration. Both in general appearance and in the appearance of certain characters of the male genitalia, this species appears to be most closely related to *Z. umbraculus* and *Z. umbraculoides*, which have short parameres and are known only from male specimens.

#### Distribution

**Figure 21.**
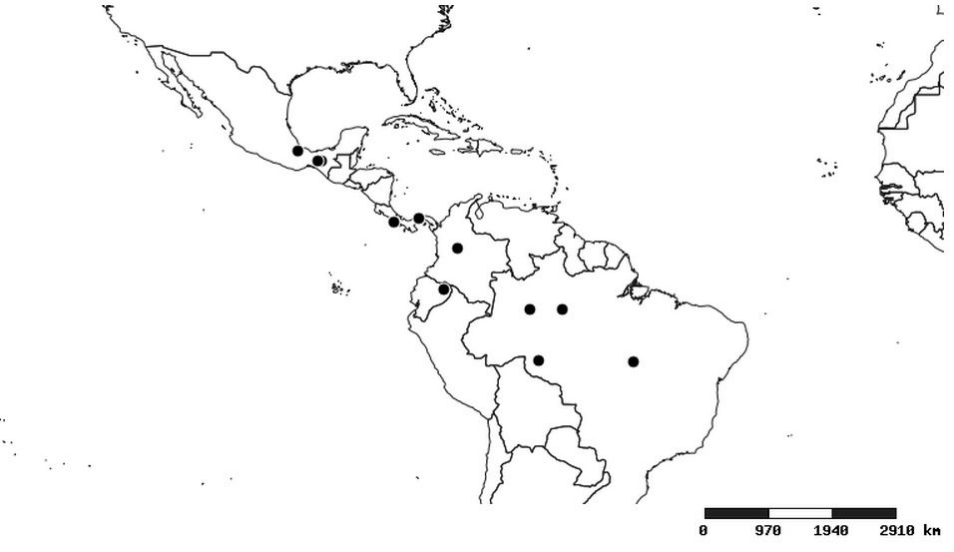
*Zelus amblycephalus* Zhang & Hart, sp. n., specimen record map

Southern Mexico to northern South America and part of Brazil (Fig. 21). Countries with records: Brazil, Colombia, Costa Rica, Ecuador, Mexico, Panama and Suriname.

### Zelus ambulans

Stål, 1862

Zelus ambulans Stål, 1862, p. 451, orig. descr.; Stål, 1872, p. 91, cat. (subgenus *Diplodus*); Lethierry and Severin, 1896, p. 151, cat.; Champion, 1898, p. 259–260, Tab. XV. fig. 23, 23a, junior syn. of *Z. exsanguis*; Maldonado, 1990, p. 327. cat. and junior syn. of *Z. exsanguis*. **stat. rev.** (current study).
Diplodus ambulans : Uhler, 1886, p. 24, checklist; Walker, 1873, cat.

#### Materials

- **Type status:** Lectotype. **Occurrence:** catalogNumber: UCR_ENT 00040998; occurrenceRemarks: Lectotype of *Zelus ambulans* Stål, 1862 (**New Designation** by Zhang, Hart & Weirauch, 2016). Verbatim label info: Mexico / Salle / ambulans Stal. / Lectotype Zelus ambulans Stal / designated by E. R. Hart / Typus / NHRS-GULI 000000318; recordedBy: Salle; sex: Adult Male; otherCatalogNumbers: NHRS-GULI 000000318; **Taxon:** scientificName: Zelus ambulans; family: Reduviidae; genus: Zelus; scientificNameAuthorship: Stål, 1862; **Location:** country: MEXICO; stateProvince: unknown; **Identification:** identifiedBy: G. Zhang; dateIdentified: 2012; **Event:** eventDate: No date provided; **Record Level:** institutionCode: NHRS
- **Type status:** Paralectotype. **Occurrence:** occurrenceRemarks: Paralectotype of *Zelus ambulans* Stål, 1862 (**New Designation** by Zhang, Hart & Weirauch, 2016). Verbatim label info: Mexico / Salle / Allotypus / *Zelus ambulans* Stal; recordedBy: Salle; sex: Adult Male; **Taxon:** scientificName: Zelus ambulans; family: Reduviidae; genus: Zelus; scientificNameAuthorship: Stål, 1862; **Location:** country: MEXICO; stateProvince: unknown; **Event:** eventDate: No date provided; **Record Level:** institutionCode: NHRS
- **Type status:** Paralectotype. **Occurrence:** occurrenceRemarks: Paralectotype of *Zelus ambulans* Stål, 1862. (**New Designation** by Zhang, Hart & Weirauch, 2016). Verbatim label info: Mexico Coll. Signoret / det. Stal; recordedBy: Signoret; sex: Adult Male; **Taxon:** scientificName: Zelus ambulans; family: Reduviidae; genus: Zelus; scientificNameAuthorship: Stål, 1862; **Location:** country: MEXICO; stateProvince: unknown; **Event:** eventDate: No date provided; **Record Level:** institutionCode: NHMW

#### Description

Figs 22, 23, 24

***Male***: (Fig. 22a, b) Medium-sized, total length 12.89–15.19 mm (mean 14.27 mm, Suppl. material 2); slender. **COLORATION**: Dorsal surface generally brown. Anteocular lobe yellowish-brown to light reddish with darker brown areas on lateral surfaces between compound eyes and antennal insertions, some specimens with dark brown areas on posterodorsal surface. Dorsal surface of postocular lobe dark brown with wide yellowish-brown mid-dorsal and circumocellar areas, remainder of surface yellowish-brown. Rostrum yellowish-brown to reddish-brown, some specimens with segment I and apex of segment II darker reddish-brown. Antennal segments I and II with varying dark brown areas at base and apex, remainder of I and II yellowish-brown to dark reddish-brown, III and IV dark reddish-brown. Anterior lobe yellowish-brown with varying dark brown areas on dorsolateral margins, anterolateral angles of collar, medial sulcus, and small patches at posterodorsal margin. Posterior lobe yellowish-brown with posterior 1/2, except for posterior margin, darkening brown in some specimens. Scutellum yellowish-brown to dark brown. Legs yellowish-brown, apical 1/5 of femora with brown to brownish-black band and apex of tibiae darkening to dark reddish-brown. Hemelytron brown with yellowish-brown costal margins and veins of clavus and corium yellowish-brown except for veins bounding discal cells. Abdominal venter yellowish-brown. **VESTITURE**: Moderately setose. Short recumbent setae predominating dorsally; short to moderate erect setae over entire body. Recumbent and erect setae over entire sure of head, recumbent setae predominating dorsally. Postocular lobe with recumbent and scattered erect setae over entire surface, recumbent setae more dense dorsally, erect setae more dense lateroventrally. Anterior pronotal lobe with erect and recumbent setae confined to setal tracts dorsally, erect setae laterally. Posterior lobe vestiture consisting of recumbent and scattered erect setae over entire surface. Scutellum with moderate to long, silky setae. Clavus and corium with short recumbent setae, longer near base of clavus. Abdomen with short, stiff, erect setae dorsally, remainder of surface with recumbent and scattered erect setae. **STRUCTURE: Head**: Cylindrical, L/W = 2.30. Postocular lobe moderately long, posterior 1/2 width constant, lateral margins subparallel. Eey moderately prominent; dorsal margin attaining postocular transverse groove, ventral margin removed from ventral surface of head. Ocellus only slightly elevated. *Labium*: I: II: III = 1: 1.8: 0.4. **Thorax**: Anterolateral angle with inconspicuous subtuberculate projection; medial longitudinal sulcus evident only on posterior 1/2, deepening to transverse sulcus of pronotum. Posterior pronotal lobe with finely rugulose surface; disc about same level of, and continuous with, humeral angle; humeral angle armed, with spinous process. Scutellum short; apex blunt to subtuberculate. *Legs*: Slender. Femoral diameters subequal. *Hemelytron*: Surpassing apex of abdomen by about length of abdominal segment seven; quadrate cell small; Cu and M of cubital cell subparallel. **GENITALIA**: (Fig. 23) *Pygophore*: Elongate ovoid; not expanded laterally in dorsal view. Medial process triangular, relatively broad, moderately long, semi-erect, nearly straight, curved slightly posteriad apically; apex in posterior view blunt, without modification. *Paramere*: Cylindrical; moderately long, nearly reaching apex of medial process; slightly curved ventrad; apical part enlarged. *Phallus*: Dorsal phallothecal sclerite elongated, somewhat flattened; medially slightly constricted; apical portion of phallothecal sclerite not distinctly tapered, slightly convex; apex truncate, medially emarginate; posterior margin of foramen broadly concave. Struts attached to dorsal phallothecal sclerite; apically separate, connected by bridge; basally separate. Basal plate arm moderately robust, separate, converging, in lateral view nearly straight, very slightly curved; bridge short; extension of basal plate small and confined to apex of basal plate arm.

***Female***: (Fig. 22c) Similar to male, except for the following. Larger than male, total length 15.43–18.59 mm (mean 16.55 mm, Suppl. material 2). Generall coloration slightly lighter; legs more or less uniformly colored, apices somewhat reddish, without dark bands.

#### Diagnosis

Among the species of *Zelus luridus* group, *Z. ambulans* has the humeral angle elevated to level of, and continuous with, disc of the posterior pronotal lobe, a condition that is also present in *Z. exsanguis*, but it can be separated from that species by the yellowish veins on corium, contrasting to the brown corium, whereas the entire corium is more or less uniformly colored in *Z. exsanguis*.

Among species of the *Zelus luridus* species group (Fig. 3) males of *Z. ambulans* can be recognized by the relatively slender medial process (Fig. 23a) and the paramere barely reaching the medial process. The apical enlargement of the paramere is smaller than that in *Z. spatulosus* and *Z. exsanguis*, but larger than that in *Z. grandoculus*, *Z. luridus* and *Z. antiguensis*.

#### Distribution

**Figure 24.**
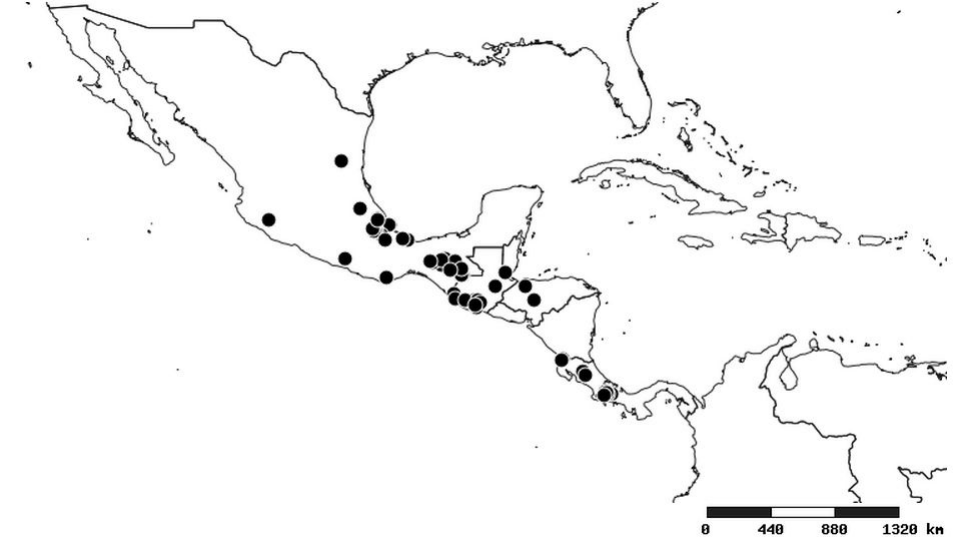
*Zelus ambulans* Stål, 1862, specimen record map

North and Central America (Fig. 24). Countries with records: Belize, Costa Rica, El Salvador, Guatemala, Honduras, Mexico, Nicaragua, Panama.

#### Taxon discussion

Champion (1898) synonymized *Z. ambulans*, *Z. luridus* and *Z. cognatus* under *Z. exsanguis*. Hart (1986) recognized all three as valid species, but did not formally reinstate *Z. ambulans*, probably because it is outside the geographic focus of that particular study. *Zelus ambulans* remained a synonym of *Z. exsanguis* in Maldonado (1990)'s catalogue or Reduviidae. We here resurrect *Z. ambulans* from synonymy. Champion (1898)'s figures of *Z. exsanguis* actually depict *Z. ambulans*.

Although this species shows very little morphological variations, color patterns within an area do vary considerably. The dark area at the posterior margin of the longitudinal medial sulcus of the anterior lobe, which serves to easily distinguish *Z. ambulans* from *Z. exsanguis*, is relatively constant. Other colors, specifically that of the posterior pronotal lobe and the femoral apices vary from quite light to very dark brown in any given locality. There is also an occasional specimen with somewhat darker hemelytron, but this does not show the wide range of variations of the aforementioned characters.

Most specimens examined have been collected from moderate to high altitudes.

### Zelus annulosus

(Stål, 1866)

Diplodus annulosus Stål, 1866, p. 299, orig. descr.; Walker, 1873, p. 126, cat.
Zelus annulosus : Stål, 1872, p. 92, cat. (subgenus *Diplodus*); Lethierry and Severin, 1896, p. 151, cat.; Fracker and Bruner, 1924, p. 170, note; Wygodzinsky, 1949a, p. 48, checklist; Maldonado, 1990, p. 326, cat.

#### Materials

- **Type status:** Holotype. **Occurrence:** catalogNumber: UCR_ENT 00040999; occurrenceRemarks: Verbatim label info: Amazon / Stevens. / annulosus Stal. / Typus / NHRS-GULI 000000319; recordedBy: Stevens; sex: Adult Female; otherCatalogNumbers: NHRS-GULI 000000319; **Taxon:** scientificName: Zelus annulosus; family: Reduviidae; genus: Zelus; scientificNameAuthorship: (Stål, 1866); **Location:** country: unknown; stateProvince: unknown; locality: Amazon; **Identification:** identifiedBy: G. Zhang; dateIdentified: 2012; **Event:** eventDate: No date provided; **Record Level:** institutionCode: NHRS

#### Description

Figs 25, 26, 27

***Male***: (Fig. 25) Large, total length 14.57 mm (n=1, Suppl. material 2); very slender. **COLORATION**: Yellowish with dark brown patches; green on posterior pronotal lobe and corium. Most surface of head yellowish, dark stripe between eye and antennal insertion, on postocular lobe behind ocellus, and on lateral surface. Scape dark brown with three yellowish bands. Labium yellowish, dark band on first and second segments. Anterior pronotal lobe yellowish, anterior medial brown patch, anterolateral angle dark brown, connected to dark brown patch on lateral surface. Posterior pronotal lobe, anterior part of corium green; rest of hemelytron brown to dark brown. Pleura yellowish with dark brown patch. Femora and tibiae with alternating yellow and dark brown bands, six of each on femora, four of each on tibiae, yellow band smaller, more so on tibiae. **VESTITURE**: Moderately setose. Entire dorsal surface, including corium and clavus, with dark, dense, short to moderately long, erect, spine-like setae. Ventral surface of head, pleura with short, semi-erect to recumbent setae. Abdomen with moderately dense, short, semi-erect to recumbent setae, intermixed with long, erect setae. Sundew setae on profemur sparse. **STRUCTURE: Head**: Elongated, L/W = 2.13. Postocular lobe very long; in dorsal view distinctly narrowing through anterior 1/2, posterior 1/2 constant, tube-like. Eye prominent; lateral margin much wider than postocular lobe; dorsal margin attaining postocular transverse groove, ventral margin removed from ventral surface of head. *Labium*: I: II: III = 1: 1.5: 0.4. Basiflagellomere diameter subequal to that of pedicel. **Thorax**: Anterolateral angle bearing small protuberance; medial longitudinal sulcus shallow near collar, deepening posteriorly. Posterior pronotal lobe with finely rugulose surface; disc distinctly elevated above humeral angle; humeral angle armed, with short tuberculate processes. Scutellum moderately long; apex angulate. *Legs*: Slender. *Hemelytron*: Slightly surpassing apex of abdomen, not more than length of abdominal segment seven; quadrate cell small and slender; Cu and M of cubital cell subparallel. **GENITALIA**: (Fig. 26) *Pygophore*: Ovoid; not expanded laterally in dorsal view; broad lightly sclerotized expansion between paramere and medial process. Medial process expanded laterally; short; semi-erect; basally slightly protruding; apex in posterior view truncate, with small sharp lateral projections. *Paramere*: Cylindrical; long, nearly reaching apex of medial process; directed toward medial process; apically recurved. *Phallus*: Dorsal phallothecal sclerite shield-shaped; sharp, dorsad projection arising close to base; apical portion of phallothecal sclerite not distinctly tapered, flat, laterally distinctly angulate, ridge-like; apex truncate, not emarginate; posterior margin of foramen broadly inversely v-shaped. Struts attached to dorsal phallothecal sclerite; apically separate, not connected by bridge; basally fused. Basal plate arm moderately robust; separate; converging; in lateral view slightly curved; bridge moderately long; extension of basal plate expanded onto arm.

***Female***: Similar to male, except for the following. Larger than male, total length 21.19–22.72 mm (mean 21.91 mm, Suppl. material 2). Some dry-preserved specimens have posterior pronotal lobe and corium not green but brown, probably a result of preservation artifact.

#### Diagnosis

Recognized by the following combination of characters: the posterior pronotal and corium dark green; the legs with four to five alternative yellow and black bands; the head, pronotum, scutellum and corium with moderately dense, black, erect, spine-like setae; the rather long and slender legs, the profemur 1/2 of body length; the rather long postocular lobe, enlarged at posterior 3/4; and the quadrate cell on corium rather slender, length more than 2x width.

Males can also be recognized by the long paramere, reaching apex of medial process; the apex of paramere recurved; the medial process apically with two lateral sharp projections; the membranous sclerite between paramere and medial process, not distinctly protruding posteriorly; and the dorsal phallothecal sclerite with lateral expansion close to basal arm, sharp, dorsad.

#### Distribution

**Figure 27.**
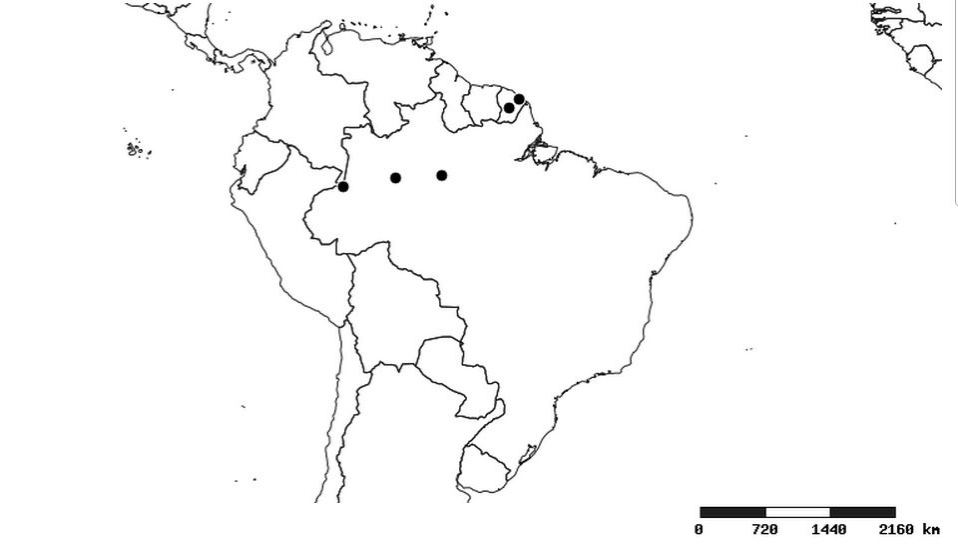
*Zelus annulosus* (Stål, 1866), specimen record map

South America (Fig. 27). The Colombian and Brazilian Amazonia and Frech Guiana. Countries with records: Brazil, Colombia, French Guiana.

### Zelus antiguensis

Zhang & Hart sp. n.

urn:lsid:zoobank.org:act:4E29C27A-E2EB-49B4-81B6-6F7AC2081788

#### Materials

- **Type status:** Holotype. **Occurrence:** catalogNumber: UCR_ENT 00007995; recordedBy: B. Lott; sex: Adult Male; **Taxon:** scientificName: Zelus antiguensis; family: Reduviidae; genus: Zelus; scientificNameAuthorship: Zhang & Hart, 2016; **Location:** country: GUATEMALA; stateProvince: Sacatepequez; locality: Antigua; verbatimElevation: 1583 m; decimalLatitude: 14.5611; decimalLongitude: -90.7344; georeferenceSources: Gazetteer; **Identification:** identifiedBy: G. Zhang; dateIdentified: 2013; **Event:** eventDate: no date provided; **Record Level:** institutionCode: USNM
- **Type status:** Paratype. **Occurrence:** catalogNumber: UCR_ENT 00007955; occurrenceRemarks: Genitallia dissected; recordedBy: B. Lott; sex: Adult Male; **Taxon:** scientificName: Zelus antiguensis; family: Reduviidae; genus: Zelus; scientificNameAuthorship: Zhang & Hart, 2016; **Location:** country: GUATEMALA; stateProvince: Sacatepequez; locality: Antigua; verbatimElevation: 1583 m; decimalLatitude: 14.5611; decimalLongitude: -90.7344; georeferenceSources: Gazetteer; **Identification:** identifiedBy: G. Zhang; dateIdentified: 2013; **Event:** eventDate: no date provided; **Record Level:** institutionCode: USNM
- **Type status:** Paratype. **Occurrence:** catalogNumber: UCR_ENT 00009305; recordedBy: N. L. H. Krauss; sex: Adult Female; **Taxon:** scientificName: Zelus antiguensis; family: Reduviidae; genus: Zelus; scientificNameAuthorship: Zhang & Hart, 2016; **Location:** country: GUATEMALA; stateProvince: Sacatepequez; locality: Antigua; decimalLatitude: 14.56667; decimalLongitude: -90.73333; georeferenceSources: Gazetteer; **Identification:** identifiedBy: G. Zhang; dateIdentified: 2013; **Event:** eventDate: 1965-10-01; **Record Level:** institutionCode: USNM
- **Type status:** Paratype. **Occurrence:** catalogNumber: UCR_ENT 00015069; recordedBy: D. M. Bates; sex: Adult Female; **Taxon:** scientificName: Zelus antiguensis; family: Reduviidae; genus: Zelus; scientificNameAuthorship: Zhang & Hart, 2016; **Location:** country: GUATEMALA; stateProvince: Sacatepequez; locality: Antigua; verbatimElevation: 1583 m; decimalLatitude: 14.5611; decimalLongitude: -90.7344; georeferenceSources: Gazetteer; **Identification:** identifiedBy: G. Zhang; dateIdentified: 2013; **Event:** eventDate: 1930-07-01; **Record Level:** institutionCode: AMNH
- **Type status:** Paratype. **Occurrence:** catalogNumber: UCR_ENT 00029478; recordedBy: R. H. Painter; sex: Adult Female; **Taxon:** scientificName: Zelus antiguensis; family: Reduviidae; genus: Zelus; scientificNameAuthorship: Zhang & Hart, 2016; **Location:** country: GUATEMALA; stateProvince: Sacatepequez; locality: Antigua; verbatimElevation: 1583 m; decimalLatitude: 14.5611; decimalLongitude: -90.7344; georeferenceSources: Gazetteer; **Identification:** identifiedBy: G. Zhang; dateIdentified: 2013; **Event:** eventDate: 1951-09-12; **Record Level:** institutionCode: USNM
- **Type status:** Paratype. **Occurrence:** catalogNumber: UCR_ENT 00017184; recordedBy: P. & C. Vaurie; sex: Adult Female; **Taxon:** scientificName: Zelus antiguensis; family: Reduviidae; genus: Zelus; scientificNameAuthorship: Zhang & Hart, 2016; **Location:** country: MEXICO; stateProvince: Chiapas; locality: Tuxtla Gutierrez; verbatimElevation: 549 m; decimalLatitude: 16.75469; decimalLongitude: -93.11485; **Identification:** identifiedBy: G. Zhang; dateIdentified: 2013; **Event:** eventDate: 1955-07-06 to 1955-07-10; **Record Level:** institutionCode: AMNH
- **Type status:** Paratype. **Occurrence:** catalogNumber: UCR_ENT 00006071; recordedBy: H. B. Leech; sex: Adult Female; **Taxon:** scientificName: Zelus antiguensis; family: Reduviidae; genus: Zelus; scientificNameAuthorship: Zhang & Hart, 2016; **Location:** country: MEXICO; stateProvince: Jalisco; locality: Pine Forst 87 miles S of Manzamitla; decimalLatitude: 19.17323; decimalLongitude: -103.66112; georeferenceSources: Google Earth; **Identification:** identifiedBy: G. Zhang; dateIdentified: 2013; **Event:** eventDate: 1948-12-01; **Record Level:** institutionCode: CAS
- **Type status:** Paratype. **Occurrence:** catalogNumber: UCR_ENT 00019699; recordedBy: E. S. Ross; sex: Adult Female; **Taxon:** scientificName: Zelus antiguensis; family: Reduviidae; genus: Zelus; scientificNameAuthorship: Zhang & Hart, 2016; **Location:** country: MEXICO; stateProvince: Jalisco; locality: Pine Forst 87 miles S of Manzamitla; decimalLatitude: 19.17323; decimalLongitude: -103.66112; georeferenceSources: Google Earth; **Identification:** identifiedBy: G. Zhang; dateIdentified: 2013; **Event:** eventDate: 1948-12-01; **Record Level:** institutionCode: CAS
- **Type status:** Paratype. **Occurrence:** catalogNumber: UCR_ENT 00038423; recordedBy: J. Doyen; sex: Adult Female; **Taxon:** scientificName: Zelus antiguensis; family: Reduviidae; genus: Zelus; scientificNameAuthorship: Zhang & Hart, 2016; **Location:** country: MEXICO; stateProvince: Jalisco; locality: 6 mi W of Chapala; decimalLatitude: 20.29709; decimalLongitude: -103.28149; georeferenceSources: Google Earth; **Identification:** identifiedBy: G. Zhang; dateIdentified: 2013; **Event:** eventDate: 1963-06-30; **Record Level:** institutionCode: UCB
- **Type status:** Paratype. **Occurrence:** catalogNumber: UCR_ENT 00023699; recordedBy: Unknown; sex: Adult Female; **Taxon:** scientificName: Zelus antiguensis; family: Reduviidae; genus: Zelus; scientificNameAuthorship: Zhang & Hart, 2016; **Location:** country: MEXICO; stateProvince: Veracruz; locality: Jalapa; decimalLatitude: 19.54381; decimalLongitude: -96.90993; georeferenceSources: Google Earth; **Identification:** identifiedBy: G. Zhang; dateIdentified: 2013; **Event:** eventDate: no date provided; **Record Level:** institutionCode: RMNH

#### Description

Figs 28, 29, 30

***Male***: (Fig. 28a, b) Medium-sized, total length 13.69–16.28 mm (mean 14.98 mm, Suppl. material 1); slender. **COLORATION**: Dorsal surface of anteocular lobe reddish-brown, yellowish-brown ventrally. Dorsum of postocular lobe dark brown with yellowish-brown mid-dorsal line and circumocellar areas, ventral surface yellowish-brown. Rostrum light reddish-brown. Scape and pedicel light reddish-brown with dark brown areas near base and apex. Anterior pronotal lobe reddish-brown with yellowish-brown anteroventral area. Dorsal surface of posterior lobe reddish-brown with yellowish-brown lateral and posterior margins, humeral angle dark brown and lateral surfaces yellowish-brown. Scutellum yellowish-brown to reddish-brown. Legs yellowish-brown to reddish-brown, femoral and tibial apices darker reddish-brown. Hemelytron brown, veins of clavus and corium slightly lighter in color than surrounding areas. Abdomen with dorsal surface reddish-brown, ventral surface yellowish-brown. **VESTITURE**: Moderately setose. Anteocular lobe with recumbent setae over dorsal surface, some erect setae ventrally. Postocular lobe with recumbent setae predominating dorsally, long silky erect setae posterodorsally and over lateral surface. Anterior pronotal lobe with recumbent and short erect setae on faint setal tracts dorsally and scattered on lateral surfaces, long silky erect setae on anterior margins. Posterior pronotal lobe with short recumbent and erect setae over entire surface, some long erect setae mid-dorsally on anterior half. Scutellum with moderate to long setae. Corium and clavus of hemelytron with recumbent setae. Abdomen with sparse, short, erect setae over entire surface, some short recumbent and longer erect setae on lateral and ventral surfaces, moderate to long erect setae posteroventrally on segment seven. Pygophore with short to moderate setae over exposed surface. **STRUCTURE: Head**: Cylindrical, L/W = 2.29. Postocular lobe relatively short; in dorsal view anteriorly gradually narrowing, posterior portion constant, slightly narrower. Eye moderately sized; dorsal margin attaining postocular transverse groove, ventral margin removed from ventral surface of head. Ocellus greatly elevated. *Labium*: I: II: III = 1: 1.8: 0.6. Basiflagellomere diameter larger than that of pedicel. **Thorax**: Anterolateral angle rounded, without projection; medial longitudinal sulcus shallow near collar, deepening slightly in posterior half. Posterior pronotal lobe with finely rugulose surface; disc slightly elevated above humeral angle; humeral angle armed, with short tuberculate processes. Scutellum moderately long; lateral depressions deep; apex slightly produced. *Legs*: Slender. Pro- and mesofemoral diameters subequal, metafemoral diameter slightly smaller. *Hemelytron*: Surpassing apex of abdomen by about length of abdominal segment seven; quadrate cell small, elongate; Cu and M of cubital cell subparallel. **GENITALIA**: (Fig. 29) *Pygophore*: Elongate ovoid; lateral margin above paramere insertion slightly expanded laterally in dorsal view. Medial process triangular, broad, short, erect; nearly straight; apex in posterior view blunt, without modification. *Paramere*: Cylindrical over basal half, slightly compressed and enlarged dorsoventrally over apical half; moderately long, nearly reaching medial process; not distinctly curved; apical part very slightly enlarged. *Phallus*: Dorsal phallothecal sclerite somewhat squarish; apex blunt, medially very slightly emarginate, not distinctly tapered; surface flat; posterior margin of foramen broadly concave. Struts attached to dorsal phallothecal sclerite; apically separate, connected by bridge; basally mostly separate, moderately fused. Basal plate arm slender; separate; converging; in lateral view nearly straight, very slightly curved; bridge moderately long; extension of basal plate small and confined to apex of basal plate arm.

***Female***: (Fig. 28c, d) Similar to male, except for the following. Larger than male, total length 15.77–18.15 mm (mean 16.91 mm, Suppl. material 2). Coloration very similar to that in male, slightly lighter overall. Anteocular lobe varying from reddish-brown to brown dorsally, legs of some specimens unicolorous. Ocellar elevation not pronounced; middle of mesothoracic femora slightly swollen.

#### Diagnosis

As with several members of the *Zelus luridus* species group, the coloration is greenish-brown, rather uniform. The medial process is triangular, its base distinct from the rest of pygophore ventral rim and apex without modification. Can be distinguished from males of other species of the *Zelus luridus* species group (Fig. 3) by the base of the medial process extended, the apex of the paramere not greatly enlarged, and the phallothecal sclerite rather short.

*Zelus antiguensis* is similar in appearance to *Z. luridus* and might easily be confused with that species. The comparatively broader medial process and posterior protrusion of the base of the medial process are readily evident in *Z. antiguensis* (Fig. 3). This species also shows an internal folding of the dorsolateral apical areas of the dorsal phallothecal sclerite, such folding being absent in *Z. luridus*. Generally, in both sexes the head is more pubescent and the pronotum more flattened dorsally than is normally found in *Z. luridus*. These two species do not overlap in distribution.

#### Etymology

Named after the type locality, Antigua, in Guatemala.

#### Distribution

**Figure 30.**
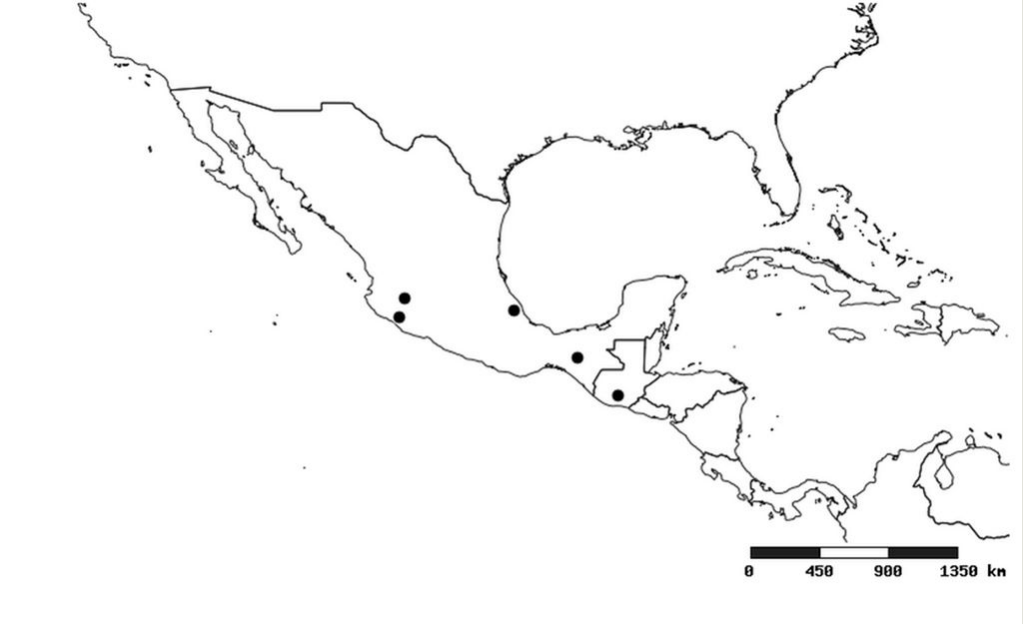
*Zelus antiguensis* Zhang & Hart, sp. n., specimen record map

Southern Mexico and Guatemala (Fig. 30).

### Zelus armillatus

(Lepeletier & Serville, 1825)

Reduvius armillatus Lepeletier and Serville, 1825, p. 278, orig. descr.
Diplodus armillatus : Amyot and Serville, 1843, p. 370, descr.; Stål, 1860, p. 75, list; Walker, 1873, p. 123, cat.
Euagoras armillatus : Herrich-Schaeffer, 1853, p. 91, list.
Zelus armillatus : Stål, 1872, p. 90, cat. (subgenus *Diplodus*); Lethierry and Severin, 1896, p. 151, cat.; Wygodzinsky, 1949a, p. 49, checklist; Mayr, 1866, p. 138-139, senior syn. of *Z. brasiliensis*, *Z. aurantiacus*, Z. guttifer and *Z. conjungens*; Berg, 1879, p. 151-152, list and nymphs (subgenus *Diplodus*); Costa Lima, 1940, 218, list (subgenus *Diplocodus*); Wygodzinsky, 1957, p. 268, note; Wygodzinsky, 1960; p. 307, list; Maldonado, 1990, p. 326, cat.; Van der Heyden, p. 85-90, new record (misidentification, should be *Zelus atripes*).
Reduvius brasiliensis Lepeletier and Serville, 1825, p. 278, orig. descr.
Diplodus brasiliensis : Amyot and Serville, 1843, p. 370, descr.; Stål, 1860, p. 75, note; Mayr, 1866, p. 138- 139, junior syn. of *Z. armillatus*; Walker, 1873, p. 123, cat.
Euagoras brasiliensis : Herrich-Schaeffer, 1853, p. 91, list.
Zelus brasiliensis : Stål, 1872, p. 90, cat. (subgenus *Diplodus*); Lethierry and Severin, 1896, p. 151, junior syn. of *Z. armillatus*.
Arilus aurantiacus Herrich-Schaeffer, 1848, p. 35-36 Tab. CCLXI. fig. 809, orig. descr. and fig.; Mayr, 1866, p. 138-139, junior syn. of *Z. armillatus*; Stål, 1872, p. 90, junior syn. of *Z. armillatus*.
Euagoras aurantiacus : Herrich-Schaeffer, 1853, p. 91, list (*aurantius* (sic)).
Ploeogaster aurantiacus : Herrich-Schaeffer, 1853, p. 168, list.
Arilus guttifer Herrich-Schaeffer, 1848, p. 36, Tab. CCLXI, fig. 810, orig. descr. and fig.; Mayr, 1866, p. 138-139, junior syn. of *Z. armillatus*; Stål, 1872, p. 90, junior syn. of *Z. brasiliensis*.
Euagoras guttifer : Herrich-Schaeffer, 1853, p. 92, list.
Ploeogaster guttifer : Herrich-Schaeffer, 1853, p. 168, list.
Diplodus guttifer : Stål , 1860, p. 74, descr.; Walker, 1873, p. 126, cat.
Zelus guttifer : Stål 1862, p. 453, note.
Arilus guttifer , Mayr, 1866, p. 138-139, junior syn. of *Z. armillatus* .

#### Description

**Figure 35.**
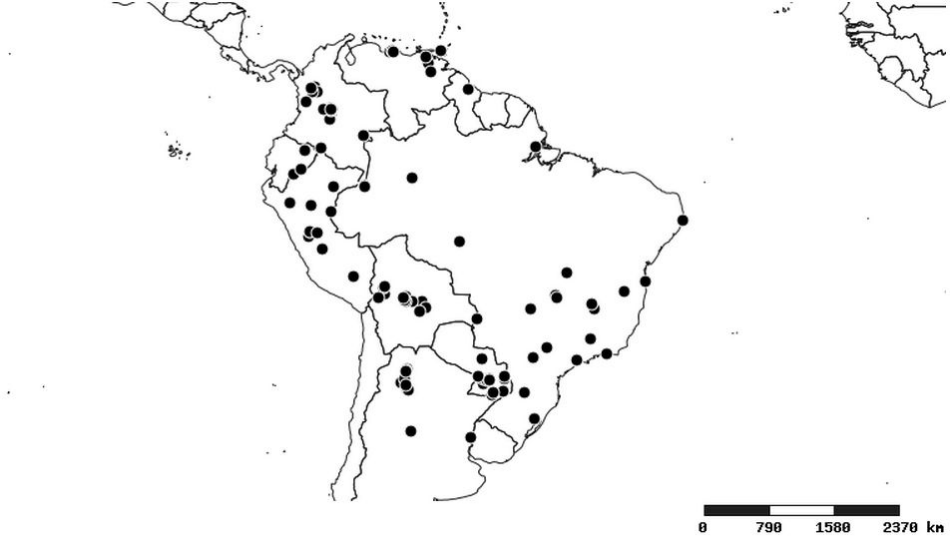
*Zelus armillatus* (Lepeletier & Serville, 1825), specimen record map

Figs 31, 32, 33, 34, 35

***Male***: (Fig. 31) Large, total length 17.15–19.02 mm (mean 17.87 mm, Suppl. material 2); robust. **COLORATION**: Highly variable, with varying combinations and amounts of yellow, yellowish-brown and brownish-black; margins of posterior pronotal lobe and corium yellowish, rest brownish-black as most common pattern, amount of black varies, sometimes almost entirely black; legs uniformly black or apically reddish, yellow-black banded in some specimens. Abdominal dorsal surface dark brown, segments with yellowish-brown posterior and lateral margins; lateral and ventral surfaces dark brown to brownish-black with lighter mid-ventral line or yellowish-brown with variable darker areas. Pygophore yellowish-brown to brownish-black, pattern variable. **VESTITURE**: Densely setose. Short recumbent and short to long erect setae over entire surface of head. Anterior pronotal lobe with short recumbent and short to long erect setae on lateral surface, short to long erect setae confined to tracts dorsally; posterior lobe with recumbent to erect setae on lateral surface, erect setae on dorsal surface; scutellum with dense moderate to long semi-erect to erect setae, denser on apex. Corium and clavus with short, recumbent or erect setae. Scattered short erect setae on abdominal dorsum, lateral and ventral surfaces with short recumbent setae interspersed with erect setae of varying lengths. Recumbent and erect setae on exposed surface of pygophore; apical 1/3 of parameres with erect setae on dorsal surface. **STRUCTURE: Head**: Cylindrical, L/W = 2.40. Postocular lobe relatively short; in dorsal view anteriorly gradually narrowing, posterior portion constant, slightly narrower. Eye moderately sized; dorsal and ventral margins removed from outlines of head. *Labium*: I: II: III = 1: 1.5: 0.4. Basiflagellomere diameter subequal to that of pedicel. **Thorax**: Anterolateral angle rounded, without projection; medial longitudinal sulcus evident throughout, deepening posteriorly. Posterior pronotal lobe with finely rugulose surface; disc slightly elevated above humeral angle; humeral angle armed, with spinous processes. Scutellum short; apex rounded, not projected. *Legs*: Robust. *Hemelytron*: Surpassing apex of abdomen by about length of abdominal segment seven; quadrate cell large and broad; Cu and M of cubital cell converging towards R. **GENITALIA**: (Fig. 34) *Pygophore*: Ovoid; posteriorly expanded sac-like sclerite between parameres and medial process. Medial process cylindrical; slender; moderately long, almost as long as exposed part of parameres; posteriorly directed, in less than forty-five degree with body axis; nearly straight; basally without protrusion; apex in posterior view rounded, with minute projection. *Paramere*: Cylindrical; long, not reaching apex of medial process; directed posteriad; not distinctly curved; apical part not enlarged to very slightly enlarged. *Phallus*: Sharp laterally oriented process close to posterior margin of foramen and basal arms; apical portion of phallothecal sclerite not distinctly tapered, flat, laterally angulate; apex truncate; posterior margin of foramen broadly concave. Struts attached to dorsal phallothecal sclerite; apically separate, not connected by bridge; basally mostly separate, moderately fused. Basal plate arm robust; separate; converging; in lateral view nearly straight, very slightly curved; bridge short; extension of basal plate expanded onto arm.

***Female***: (Figs 32, 33) Similar to male, except for the following. Larger than male, total length 19.0–24.7 mm (mean 21.94 mm, Suppl. material 2). Coloration variations more extensive than in male.

#### Diagnosis

The large and robust body, the dorsal coloration usually bright, yellow or red with black, the medial process short and relatively slender are characteristic to *Z. armillatus*. Male genitalic structures of *Z. armillatus* and *Z. janus* are nearly identical, but these two species do not overlap in range and are sufficiently different in non-genitalic morphological characters, which allow them to be easily separated.

The only species with which *Z. armillatus* is sympatric which may cause some identification problems is *Z. conjungens*. It may be distinguished from that species by the characters discussed under *Z. conjungens*.

#### Distribution

South America (Fig. 35). Countries with specimen records: Argentina, Bolivia, Brazil, Colombia, Ecuador, Guyana, and Paraguay.

#### Taxon discussion

*Zelus armillatus* is a very common, widespread, variable species in South America. It is known to occur in nearly all areas of the continent from central Argentina and northward, at altitudes from sea level to several thousand feet, and dry temperate to moist tropical areas. The coloration and markings of *Z. armillatus* are highly variable throughout the range and appear to be as variable in any given area (e.g., Fig. 33d) as they are between areas. This fact is responsible for the several descriptions based upon color forms of this species. The drawings of Herrich-Schaeffer (1848) illustrate two of the common variations encountered, although the dorsal coloration, as well as that of the legs, may vary from almost entirely yellowish-brown through various combinations of that color and brownish-black to almost entirely brownish-black.

### Zelus auralanus

Zhang & Hart sp. n.

urn:lsid:zoobank.org:act:CAC4A476-3BAE-4988-9D67-010CB3072C13

#### Materials

- **Type status:** Holotype. **Occurrence:** catalogNumber: UCR_ENT 00069892; recordedBy: J. R. de la Torre-Bueno; sex: Adult Male; **Taxon:** scientificName: Zelus auralanus; family: Reduviidae; genus: Zelus; scientificNameAuthorship: Zhang and Hart, 2016; **Location:** country: BRAZIL; stateProvince: Amazonas; locality: Vista Alegre [in formerly-named 'Rio Branco' territory]; decimalLatitude: 0.4578; decimalLongitude: -66.2489; georeferenceSources: Gazetteer; **Identification:** identifiedBy: G. Zhang; dateIdentified: 2013; **Event:** eventDate: 1924-09-06; **Record Level:** institutionCode: KU
- **Type status:** Paratype. **Occurrence:** catalogNumber: UCR_ENT 00009502; occurrenceRemarks: Drake Collection; recordedBy: L. E. Peńa; sex: Adult Female; **Taxon:** scientificName: Zelus auralanus; family: Reduviidae; genus: Zelus; scientificNameAuthorship: Zhang and Hart, 2016; **Location:** country: BOLIVIA; stateProvince: Cochabamba; locality: Sajta, Chapare; decimalLatitude: -17.00861; decimalLongitude: -64.78663; georeferenceSources: Google Earth; **Identification:** identifiedBy: G. Zhang; dateIdentified: 2013; **Event:** eventDate: 1992-03-01; **Record Level:** institutionCode: USNM
- **Type status:** Paratype. **Occurrence:** catalogNumber: UCR_ENT 00069893; occurrenceRemarks: Previously designated as 'allotype' of his manuscript name Zelus auralanus by Hart. This type status is not used in the formal publication of this name (Zhang et al.) and this specimen is instead designated as a paratype.; recordedBy: J. R. de la Torre-Bueno; sex: Adult Female; **Taxon:** scientificName: Zelus auralanus; family: Reduviidae; genus: Zelus; scientificNameAuthorship: Zhang and Hart, 2016; **Location:** country: BRAZIL; stateProvince: Amazonas; locality: Vista Alegre [in formerly-named 'Rio Branco' territory]; decimalLatitude: 0.4578; decimalLongitude: -66.2489; georeferenceSources: Gazetteer; **Identification:** identifiedBy: G. Zhang; dateIdentified: 2013; **Event:** eventDate: 1924-09-06; **Record Level:** institutionCode: KU
- **Type status:** Paratype. **Occurrence:** catalogNumber: UCR_ENT 00069894; recordedBy: J. R. de la Torre-Bueno; sex: Adult Male; **Taxon:** scientificName: Zelus auralanus; family: Reduviidae; genus: Zelus; scientificNameAuthorship: Zhang and Hart, 2016; **Location:** country: BRAZIL; stateProvince: Amazonas; locality: Vista Alegre [in formerly-named 'Rio Branco' territory]; decimalLatitude: 0.4578; decimalLongitude: -66.2489; georeferenceSources: Gazetteer; **Identification:** identifiedBy: G. Zhang; dateIdentified: 2013; **Event:** eventDate: 1924-09-06; **Record Level:** institutionCode: KU
- **Type status:** Paratype. **Occurrence:** catalogNumber: UCR_ENT 00006073; recordedBy: B. Malkin; sex: Adult Female; **Taxon:** scientificName: Zelus auralanus; family: Reduviidae; genus: Zelus; scientificNameAuthorship: Zhang and Hart, 2016; **Location:** country: BRAZIL; stateProvince: Mato Grosso; locality: Barra do Tapirape; decimalLatitude: -10.46666; decimalLongitude: -50.51667; georeferenceSources: Gazetteer; **Identification:** identifiedBy: G. Zhang; dateIdentified: 2013; **Event:** eventDate: 1962-12-26; **Record Level:** institutionCode: CAS
- **Type status:** Paratype. **Occurrence:** catalogNumber: UCR_ENT 00019695; recordedBy: B. Malkin; sex: Adult Male; **Taxon:** scientificName: Zelus auralanus; family: Reduviidae; genus: Zelus; scientificNameAuthorship: Zhang and Hart, 2016; **Location:** country: BRAZIL; stateProvince: Mato Grosso; locality: Barra do Tapirape; decimalLatitude: -10.46666; decimalLongitude: -50.51667; georeferenceSources: Gazetteer; **Identification:** identifiedBy: G. Zhang; dateIdentified: 2013; **Event:** eventDate: 1962-12-26; **Record Level:** institutionCode: CAS
- **Type status:** Paratype. **Occurrence:** catalogNumber: UCR_ENT 00019696; recordedBy: B. Malkin; sex: Adult Female; **Taxon:** scientificName: Zelus auralanus; family: Reduviidae; genus: Zelus; scientificNameAuthorship: Zhang and Hart, 2016; **Location:** country: BRAZIL; stateProvince: Mato Grosso; locality: Barra do Tapirape; decimalLatitude: -10.46666; decimalLongitude: -50.51667; georeferenceSources: Gazetteer; **Identification:** identifiedBy: G. Zhang; dateIdentified: 2013; **Event:** eventDate: 1962-12-26; **Record Level:** institutionCode: CAS
- **Type status:** Paratype. **Occurrence:** catalogNumber: UCR_ENT 00019697; recordedBy: B. Malkin; sex: Adult Female; **Taxon:** scientificName: Zelus auralanus; family: Reduviidae; genus: Zelus; scientificNameAuthorship: Zhang and Hart, 2016; **Location:** country: BRAZIL; stateProvince: Mato Grosso; locality: Barra do Tapirape; decimalLatitude: -10.46666; decimalLongitude: -50.51667; georeferenceSources: Gazetteer; **Identification:** identifiedBy: G. Zhang; dateIdentified: 2013; **Event:** eventDate: 1962-12-26; **Record Level:** institutionCode: CAS
- **Type status:** Paratype. **Occurrence:** catalogNumber: UCR_ENT 00019698; recordedBy: B. Malkin; sex: Adult Female; **Taxon:** scientificName: Zelus auralanus; family: Reduviidae; genus: Zelus; scientificNameAuthorship: Zhang and Hart, 2016; **Location:** country: BRAZIL; stateProvince: Mato Grosso; locality: Barra do Tapirape; decimalLatitude: -10.46666; decimalLongitude: -50.51667; georeferenceSources: Gazetteer; **Identification:** identifiedBy: G. Zhang; dateIdentified: 2013; **Event:** eventDate: 1962-12-26; **Record Level:** institutionCode: CAS
- **Type status:** Paratype. **Occurrence:** catalogNumber: UCR_ENT 00046999; recordedBy: D. Engleman; sex: Adult Female; **Taxon:** scientificName: Zelus auralanus; family: Reduviidae; genus: Zelus; scientificNameAuthorship: Zhang and Hart, 2016; **Location:** country: BRAZIL; stateProvince: Mato Grosso; locality: Mato Gr.; decimalLatitude: -10.41666; decimalLongitude: -59.46667; georeferenceSources: Label; **Identification:** identifiedBy: G. Zhang; dateIdentified: 2013; **Event:** eventDate: 1977-03-17 to 1977-03-22; **Record Level:** institutionCode: AMNH
- **Type status:** Paratype. **Occurrence:** catalogNumber: UCR_ENT 00047080; recordedBy: M. Alvarenga; sex: Adult Female; **Taxon:** scientificName: Zelus auralanus; family: Reduviidae; genus: Zelus; scientificNameAuthorship: Zhang and Hart, 2016; **Location:** country: BRAZIL; stateProvince: Para; locality: Tucurui; decimalLatitude: -3.7; decimalLongitude: -49.7; georeferenceSources: Gazetteer; **Identification:** identifiedBy: G. Zhang; dateIdentified: 2013; **Event:** eventDate: 1979-01-01; **Record Level:** institutionCode: AMNH
- **Type status:** Paratype. **Occurrence:** catalogNumber: UCR_ENT 00009459; recordedBy: B. C. Ratcliffe; sex: Adult Female; **Taxon:** scientificName: Zelus auralanus; family: Reduviidae; genus: Zelus; scientificNameAuthorship: Zhang and Hart, 2016; **Location:** country: BRAZIL; stateProvince: Rondonia; locality: 62 km S Ariquemes, Fazenda Rancho Grande; verbatimElevation: 300 m; decimalLatitude: -10.3; decimalLongitude: -62.86666; **Identification:** identifiedBy: G. Zhang; dateIdentified: 2013; **Event:** eventDate: 1991-11-11 to 1991-11-22; **Record Level:** institutionCode: USNM
- **Type status:** Paratype. **Occurrence:** catalogNumber: UCR_ENT 00072667; recordedBy: Unknown; sex: Adult Male; **Taxon:** scientificName: Zelus auralanus; family: Reduviidae; genus: Zelus; scientificNameAuthorship: Zhang and Hart, 2016; **Location:** country: ECUADOR; stateProvince: Napo; locality: 30 km E of Pto Napo; verbatimElevation: 410 m; decimalLatitude: -1.04256; decimalLongitude: -77.60111; georeferenceSources: Label; **Identification:** identifiedBy: G. Zhang; dateIdentified: 2013; **Event:** eventDate: 2005-03-04; **Record Level:** institutionCode: UCR
- **Type status:** Paratype. **Occurrence:** catalogNumber: UCR_ENT 00072668; recordedBy: Unknown; sex: Adult Female; **Taxon:** scientificName: Zelus auralanus; family: Reduviidae; genus: Zelus; scientificNameAuthorship: Zhang and Hart, 2016; **Location:** country: ECUADOR; stateProvince: Napo; locality: 30 km E of Pto Napo; verbatimElevation: 410 m; decimalLatitude: -1.04256; decimalLongitude: -77.60111; georeferenceSources: Label; **Identification:** identifiedBy: G. Zhang; dateIdentified: 2013; **Event:** eventDate: 2005-03-06; **Record Level:** institutionCode: UCR
- **Type status:** Paratype. **Occurrence:** catalogNumber: UCR_ENT 00009474; occurrenceRemarks: Lot#992 - Collection code moved to this field to prevent duplication; Drake Collection; recordedBy: T. L. Erwin et al.; sex: Adult Male; **Taxon:** scientificName: Zelus auralanus; family: Reduviidae; genus: Zelus; scientificNameAuthorship: Zhang and Hart, 2016; **Location:** country: ECUADOR; stateProvince: Orellana; locality: Reserva Etnica Waorani, 1 km S. Onkone Gare Camp, Transect Ent.; verbatimElevation: 216 m; decimalLatitude: -0.65714; decimalLongitude: -76.453; georeferenceSources: Label; **Identification:** identifiedBy: G. Zhang; dateIdentified: 2013; **Event:** samplingProtocol: Fogging; eventDate: 1995-02-10; **Record Level:** institutionCode: USNM
- **Type status:** Paratype. **Occurrence:** catalogNumber: UCR_ENT 00006072; recordedBy: E. I. Schlinger & E. S. Ross; sex: Adult Male; **Taxon:** scientificName: Zelus auralanus; family: Reduviidae; genus: Zelus; scientificNameAuthorship: Zhang and Hart, 2016; **Location:** country: PERU; stateProvince: Huanuco; locality: Monzon valley, Tingo Maria; decimalLatitude: -9.27816; decimalLongitude: -76.05562; georeferenceSources: Google Earth; **Identification:** identifiedBy: G. Zhang; dateIdentified: 2013; **Event:** eventDate: 1954-12-11; **Record Level:** institutionCode: CAS
- **Type status:** Paratype. **Occurrence:** catalogNumber: UCR_ENT 00047094; recordedBy: B. Malkin; sex: Adult Female; **Taxon:** scientificName: Zelus auralanus; family: Reduviidae; genus: Zelus; scientificNameAuthorship: Zhang and Hart, 2016; **Location:** country: ECUADOR; stateProvince: Pastaza; locality: Cuisimi, on Rio Cuisimi, 150km SE of Puyo; verbatimElevation: 350 m; decimalLatitude: -2.43129; decimalLongitude: -77.03292; **Identification:** identifiedBy: G. Zhang; dateIdentified: 2013; **Event:** eventDate: 1971-06-01 to 1971-06-05; **Record Level:** institutionCode: AMNH

#### Description

Figs 36, 37, 38

***Male***: (Fig. 36a, b, c, d) Medium-sized, total length 12.64–14.37 mm (mean 13.61 mm, Suppl. material 2); slender. **COLORATION**: Nearly entire surface medium dark brown; apices of femora dark colored; abdomen light brown to yellowish-brown. **VESTITURE**: Densely setose. Body surface covered with short, adpressed to recumbent setae, dorsal setae on head and pronotum golden, shining; ventral surface of head and abdomen with longer, erect setae; sometimes setae covered with white waxy exudation. Dense, long, erect setae on paramere; bush of short, erect, spine-like setae on posterior surface of medial process. Corium and clavus with short, recumbent setae. **STRUCTURE: Head**: Cylindrical, L/W = 2.63. Postocular lobe long; in dorsal view anteriorly gradually narrowing, posterior portion constant, slightly narrower. Eye prominent; much wider than postocular lobe; dorsal and ventral margins removed from surfaces of head. *Labium*: I: II: III = 1: 2.0: 0.4. Basiflagellomere diameter slightly larger than that of pedicel. **Thorax**: Anterolateral angle bearing small projection; medial longitudinal sulcus shallow near collar, deepening posteriorly. Posterior pronotal lobe with rugulose surface; disc about same level as humeral angle; humeral angle armed, with dentate or spinous process. Scutellum long; apex blunt, not projected. *Legs*: Slender. *Hemelytron*: Slightly surpassing apex of abdomen, not more than length of abdominal segment seven; quadrate cell small, relatively broad; Cu and M of cubital cell converging towards R. **GENITALIA**: (Fig. 37) *Pygophore*: Ovoid; slightly expanded laterally near base of paramere in dorsal view. Medial process triangular; slender; long; anteroposteriorly compressed; semi-erect; curved at middle; apex in posterior view acute, with small hooklike projection. *Paramere*: Cylindrical; moderately long, slightly exceeding medial process; directed posteriad, slightly curved towards medial process; basally narrower; curved ventrad; apical part very slightly enlarged. *Phallus*: Dorsal phallothecal sclerite somewhat pandurate, medially strongly constricted; laterally with dorsally directed small sharp projection at mid-portion; apical portion of phallothecal sclerite gradually tapering, lateral margin narrowly angulate, angulation ending anteriorly in sharp, dorsad projection; apex rounded, medially emarginate; posterior margin of foramen inversely V-shaped. Struts attached to dorsal phallothecal sclerite; apically separate, connected by bridge; basally almost completely fused. Basal plate arm moderately robust; separate; converging; in lateral view severely curved, nearly semi-circular; bridge short; extension of basal plate expanded onto arm.

***Female***: (Fig. 36e, f) Similar to male, except for the following. Larger than male, total length 17.21–18.32 mm (mean 17.85 mm, Suppl. material 2). Eye moderate size, smaller than in male.

#### Diagnosis

Can be readily recognized by the uniformly brown dorsal coloration; the darkened tibial apex; the humeral angle elevated to level of disc; the dorsal setae on head and pronotum appearing somewhat golden, shining when viewed under magnification. Males can also be recognized by the gradually enlarged paramere; the triangular medial process, curved slightly posteriad in the middle, apex with a hooklike projection; and the dorsal phallothecal sclerite with short, dorsad projections sub-laterally.

#### Etymology

The species epithet indicates the somewhat reddish tone of the coloration.

#### Distribution

**Figure 38.**
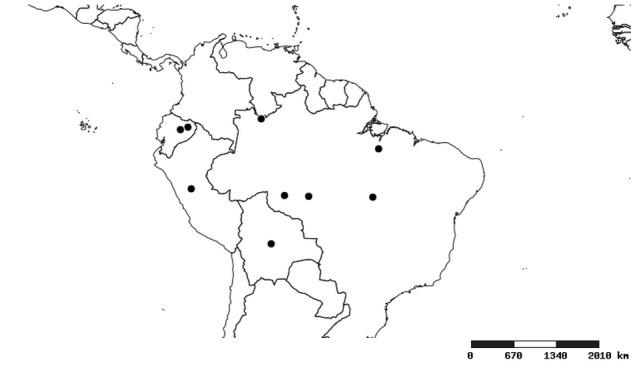
*Zelus auralanus* Zhang & Hart, **sp. n.**, specimen record map

South America (Fig. 38). Countries with specimen records: Bolivia, Brazil, Ecuador and Peru.

### Zelus bahiaensis

Zhang & Hart sp. n.

urn:lsid:zoobank.org:act:9E10BFBB-A5C0-4C49-95B4-BE01C0B7A995

#### Materials

- **Type status:** Holotype. **Occurrence:** catalogNumber: UCR_ENT 00071255; occurrenceRemarks: Name from type locality 'Bahia'. Wrongly spelled in Hart 1972, and changed to Zelusbahiaensis.; recordedBy: P. Silva; sex: Adult Male; **Taxon:** scientificName: Zelus bahiaensis; family: Reduviidae; genus: Zelus; scientificNameAuthorship: Zhang & Hart, 2016; **Location:** country: BRAZIL; stateProvince: Bahia; locality: Agua Preta; decimalLatitude: -14.58333; decimalLongitude: -39.26666; **Identification:** identifiedBy: G. Zhang; dateIdentified: 2013; **Event:** samplingProtocol: Unknown; eventDate: No date provided; **Record Level:** institutionCode: TAMU

#### Description

Figs 39, 40, 41

***Male***: (Fig. 39) Medium-sized, total length 12.35 mm (n=1); slender. **COLORATION**: Much of body surface including head, anterior pronotal lobe, membrane, legs dark brown; very slender lighter colored medial longitudinal stripe on postocular lobe. Posterior pronotal lobe and corium orange. Pleura, abdomen reddish-brown. **VESTITURE**: Moderately setose. Dorsum of anteocular and anterior part of postocular with moderately dense, short, erect, spine-like setae, posterior part of postocular nearly glabrous; ventral surface of head with sparse, short, erect or recumbent setae. Pronotum with dense, short, erect, spine-like setae on dorsum and lateral surfaces, anterior lobe also intermixed with sparse, long, fine setae. Pleura with spine-like setae, sparse on metapleuron; intermixed with short to long, erect, fine and short, recumbent setae; scutellum with spine-like setae and erect, fine setae. Legs with very sparse setation. Corium and clavus with short, recumbent setae. Abdomen with moderately dense, short, semi-erect, fine setae, intermixed with sparse, long setae. Bush of moderately long, erect setae flanking medial process on posteroventral rim of pygophore; paramere apically with sparse, short, erect setae. **STRUCTURE: Head**: Cylindrical, L/W = 2.27. Postocular lobe long; in dorsal view distinctly narrowing through anterior 2/3, posterior 1/3 constant, tube-like. Eye moderately sized; lateral margin much wider than postocular lobe; dorsal margin removed from postocular transverse groove, ventral margin attaining ventral surface of head. *Labium*: I: II: III = 1: 1.8: 0.6. Basiflagellomere diameter slightly larger than that of pedicel. **Thorax**: Anterolateral angle rounded, without projection; medial longitudinal sulcus evident throughout, deepening posteriorly. Posterior pronotal lobe with rugulose surface; disc distinctly elevated above humeral angle; humeral angle rounded, without projection. Scutellum moderately long; apex slightly pointed, not projected. *Legs*: Very slender. *Hemelytron*: Greatly surpassing apex of abdomen by about 3x length of abdominal segment seven; quadrate cell large and broad; Cu and M of cubital cell subparallel. **GENITALIA**: (Fig. 40) *Pygophore*: Ovoid; mid-lateral fold adjacent to paramere insertion; slightly expanded laterally near base of paramere in dorsal view. Medial process cylindrical; very slender; moderately long, nearly half length of paramere; posteriorly directed; basal 2/3 straight, apically curved; apex in posterior view blunt, folded posteriad, marginally narrower. *Paramere*: Cylindrical; long, surpassing medial process; directed posteriad; slightly curved ventrad; apical part very slightly enlarged. *Phallus*: Dorsal phallothecal sclerite elongated; apical portion of phallothecal sclerite not distinctly tapered, convex, laterally angulate; apex truncate, medially emarginate; posterior margin of foramen deeply concave. Struts attached to dorsal phallothecal sclerite; apically separate, connected by bridge; basally mostly separate, moderately fused. Basal plate arm slender; separate; converging; in lateral view apically curved; bridge short; extension of basal plate expanded onto arm.

***Female***: Unknown.

#### Diagnosis

Recognized by the following combination of characters: the anterior pronotal lobe dark brown and the posterior pronotal lobe orange; the 1A an Pcu not intersecting, short crossvein between them; the long and slender, cylindrical medial process; the medial process apically folded posteriad; and the rather long paramere.

#### Etymology

Named after the Brazilian state Bahia, where the holotype was collected.

#### Distribution

**Figure 41.**
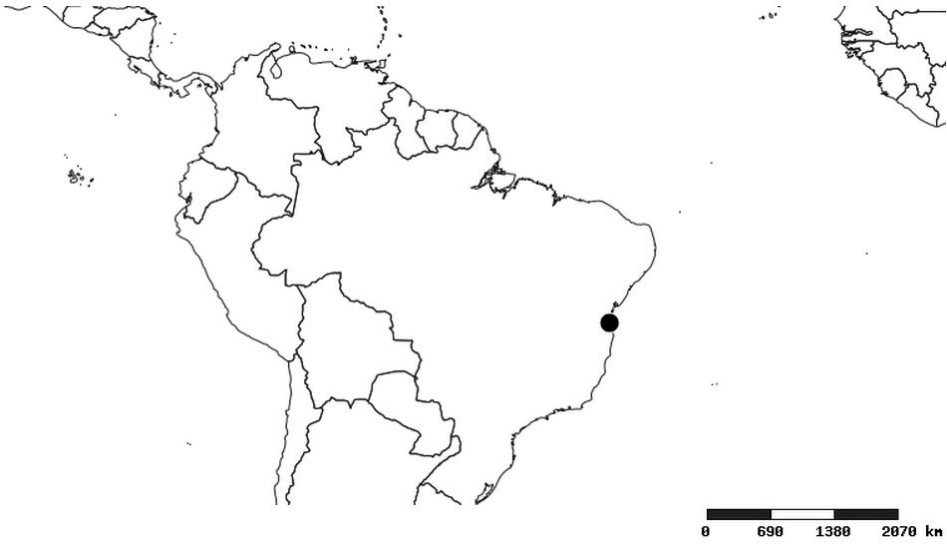
*Zelus bahiaensis* Zhang & Hart, sp. n., specimen record map

South America (Fig. 41). Known from the type locality in Brazil.

### Zelus banksi

Zhang & Hart sp. n.

urn:lsid:zoobank.org:act:900125F1-F738-43BB-BFEB-B4C6CAA40D80

#### Materials

- **Type status:** Holotype. **Occurrence:** catalogNumber: UCR_ENT 00057804; recordedBy: N. Banks; sex: Adult Male; **Taxon:** scientificName: Zelus banksi; family: Reduviidae; genus: Zelus; scientificNameAuthorship: Zhang & Hart, 2016; **Location:** country: PANAMA; stateProvince: Canal Zone; county: none; locality: Barro Colorado; decimalLatitude: 9.16666; decimalLongitude: -79.83333; georeferenceSources: Google Earth; **Identification:** identifiedBy: G. Zhang; dateIdentified: 2013; **Event:** eventDate: 1924-06-25; **Record Level:** institutionCode: AMNH
- **Type status:** Paratype. **Occurrence:** catalogNumber: UCR_ENT 00071253; recordedBy: Richter; sex: Adult Male; **Taxon:** scientificName: Zelus banksi; family: Reduviidae; genus: Zelus; scientificNameAuthorship: Zhang & Hart, 2016; **Location:** country: COLOMBIA; stateProvince: Meta; county: none; locality: Rio Guayuriba; verbatimElevation: 400 m; decimalLatitude: 4.01978; decimalLongitude: -73.60807; georeferenceSources: Google Earth; **Identification:** identifiedBy: G. Zhang; dateIdentified: 2013; **Event:** eventDate: 1947-09-06; **Record Level:** institutionCode: TAMU
- **Type status:** Paratype. **Occurrence:** catalogNumber: UCR_ENT 00009559; occurrenceRemarks: Drake Collection; recordedBy: F. J. Otoya; sex: Adult Male; **Taxon:** scientificName: Zelus banksi; family: Reduviidae; genus: Zelus; scientificNameAuthorship: Zhang & Hart, 2016; **Location:** country: COLOMBIA; stateProvince: Valle del Cauca; county: none; locality: Palmira; decimalLatitude: 3.5364; decimalLongitude: -76.3036; georeferenceSources: Gazetteer; **Identification:** identifiedBy: G. Zhang; dateIdentified: 2013; **Event:** eventDate: 1939-08-25; **Record Level:** institutionCode: USNM
- **Type status:** Paratype. **Occurrence:** catalogNumber: UCR_ENT 00029366; occurrenceRemarks: Genitalia dissected.; recordedBy: Pablo Schild; sex: Adult Male; **Taxon:** scientificName: Zelus banksi; family: Reduviidae; genus: Zelus; scientificNameAuthorship: Zhang & Hart, 2016; **Location:** country: COSTA RICA; stateProvince: Alajuela; county: none; locality: Higuito, San Mateo; verbatimElevation: 254 m; decimalLatitude: 9.95; decimalLongitude: -84.55; georeferenceSources: Gazetteer; **Identification:** identifiedBy: G. Zhang; dateIdentified: 2013; **Event:** eventDate: no date provided; **Record Level:** institutionCode: USNM
- **Type status:** Paratype. **Occurrence:** catalogNumber: UCR_ENT 00014431; recordedBy: F. Quesada; sex: Adult Male; **Taxon:** scientificName: Zelus banksi; family: Reduviidae; genus: Zelus; scientificNameAuthorship: Zhang & Hart, 2016; **Location:** country: COSTA RICA; stateProvince: Puntarenas; county: none; locality: Rancho Quemado, Peninsula de Osa; verbatimElevation: 200 m; decimalLatitude: 8.67776; decimalLongitude: -83.56478; georeferenceSources: Label; **Identification:** identifiedBy: G. Zhang; dateIdentified: 2013; **Event:** eventDate: 1991-11-01; **Record Level:** institutionCode: INBIO
- **Type status:** Paratype. **Occurrence:** catalogNumber: UCR_ENT 00015110; recordedBy: N. Banks; sex: Adult Male; **Taxon:** scientificName: Zelus banksi; family: Reduviidae; genus: Zelus; scientificNameAuthorship: Zhang & Hart, 2016; **Location:** country: PANAMA; stateProvince: Canal Zone; county: none; locality: Barro Colorado Island; decimalLatitude: 9.15562; decimalLongitude: -79.84895; georeferenceSources: Google Earth; **Identification:** identifiedBy: G. Zhang; dateIdentified: 2013; **Event:** eventDate: 1924-06-26; **Record Level:** institutionCode: AMNH

#### Description

Figs 42, 43, 44

***Male***: (Fig. 42) Medium-sized, total length 10.81–12.62 mm (mean 12.00 mm, Suppl. material 2); slender. **COLORATION**: Head uniformly brown; postocular lobe with very faint longitudinal medial stripe. Anterior pronotal lobe and hemelytron brown; posterior lobe yellowish-brown. Remainder of body surface mostly yellowish-brown, parts of pleura darker. Femora with two or three yellowish bands; tibiae with single band. **VESTITURE**: Sparsely setose. Short, recumbent setae on entire surface; very short, erect, spine-like setae on dorsum, denser on anterior lobe; few moderately long, erect, fine setae on ventral surface. Pronotum with sparse, recumbent setae and short, erect setae over dorsal surface; denser, long recumbent setae on lateral surface and pleura, intermixed with semierect or erect setae; scutellum with sparse, semi-erect and recumbent setae. Legs with sparse setation on femora and moderately dense setation on tibiae. Corium and clavus with mix of sparse, short, recumbent and erect setae. Abdomen with moderately dense, short recumbent setae, intermixed with sparse, short to long, erect setae. Apical half of dorsal surface of paramere with moderately dense, medium-length, semi-erect setae. **STRUCTURE: Head**: Cylindrical, L/W = 2.25. Postocular lobe long; in dorsal view distinctly narrowing through anterior 2/3, posterior 1/3 constant, tube-like. Eye prominent; lateral margin much wider than postocular lobe; dorsal margin attaining postocular transverse groove, ventral margin removed from ventral surface of head. *Labium*: I: II: III = 1: 2.1: 0.5. Basiflagellomere diameter larger than that of pedicel. **Thorax**: Anterolateral angle bearing small projection; medial longitudinal sulcus evident throughout, deepening posteriorly. Posterior pronotal lobe with rugulose surface; disc distinctly elevated above humeral angle; humeral angle armed, with dentate or spinous process. Scutellum short; apex angulate, slightly projected upward in some specimens. *Legs*: Slender. *Hemelytron*: Slightly surpassing apex of abdomen, not more than length of abdominal segment seven; quadrate cell small; Cu and M of cubital cell subparallel. **GENITALIA**: (Fig. 43) *Pygophore*: Elongate ovoid; not expanded laterally in dorsal view. Medial process cylindrical; slender; moderately long, nearly as long as paramere; laterally compressed towards apex; anterior surface towards apex ridged; minute spicules on posterior surface; semi-erect; very slightly curved at middle; apex in posterior view acute, with small hooklike projection. *Paramere*: Cylindrical; long, achieving apex of medial process; directed posteriad, slightly curved towards medial process; basally slightly narrower; nearly straight; apical part slightly enlarged, obliquely truncate. *Phallus*: Dorsal phallothecal sclerite somewhat ovoid; sclerotization reduced (yet not absent) on dorsal surface close to posterior margin of foramen; apical portion of phallothecal sclerite gradually tapering, distinctly keeled medially, laterally indistinctly angulate; apex acute; posterior margin of foramen broadly concave. Struts attached to dorsal phallothecal sclerite; apically separate, connected by bridge; basally separate throughout. Basal plate arm robust; basally fused; in lateral view mid-portion curved; bridge extremely short; extension of basal plate expanded laterally onto arm, covering more than 1/2 of arm, curved.

***Female***: Unknown.

#### Diagnosis

Recognized by the following combination of characters: the posterior pronotal lobe usually orangish-brown; the rather long paramere, apex obliquely truncate; and the medial process nearly straight, curvature small. Among the males of the *Zelus panamensis* species group (Fig. 12), *Z. banksi* has the longest paramere, which is longer than the medial process.

#### Etymology

Named after N. Banks, the collector of the type specimen.

#### Distribution

**Figure 44.**
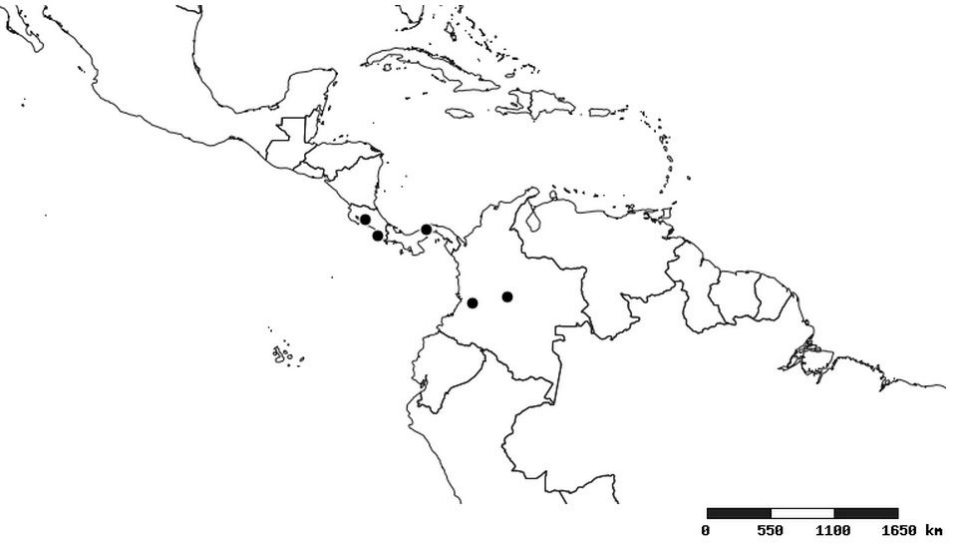
*Zelus banksi* Zhang & Hart, sp. n., specimen record map

Southern Central America and northern South America (Fig. 44). Countries with specimen records: Colombia, Costa Rica and Panama

### Zelus bruneri

De Zayas, 1960

Zelus bruneri De Zayas, 1960, p. 125–127, orig. descr. and fig; Alayo, 1967, p. 5, 36, 37, list, key and note; Hart, 1987, p. 296-297, note and key; Maldonado, 1990, p. 326, cat.

#### Materials

- **Type status:** Holotype. **Occurrence:** occurrenceRemarks: Deposited m the collection of Prof. F. de Zayas in Havana, Cuba. Not available for examination in current study.; sex: Adult male; **Taxon:** scientificName: Zelus bruneri; family: Reduviidae; genus: Zelus; scientificNameAuthorship: de Zayas, 1960; **Location:** country: Cuba; locality: Piloto, Moa, Oriente; decimalLatitude: 20.55; decimalLongitude: -75.783333; georeferenceSources: Gazetteer; **Event:** eventDate: 1954-06
- **Type status:** Paratype. **Occurrence:** occurrenceRemarks: Deposited m the collection of Prof. F. de Zayas in Havana, Cuba. Not available for examination in current study.; sex: Adult male; **Taxon:** scientificName: Zelus bruneri; family: Reduviidae; genus: Zelus; scientificNameAuthorship: de Zayas, 1960; **Location:** country: Cuba; locality: Piloto, Moa, Oriente; decimalLatitude: 20.55; decimalLongitude: -75.783333; georeferenceSources: Gazetteer; **Event:** eventDate: 1954-06

#### Distribution

Known only from Cuba.

#### Taxon discussion

Two male specimens are known from Cuba, which were not physically examined, but the original description and illustration provide a strong basis for placing this species in the *Zelus puertoricensis* species group. This is confirmed by the narrow, elongated body form; the flat and rectangular pronotum; the general genitalic shape indicated in the figure. The much smaller size and flat postocular lobe negates it being a male of *Z. subimpressus*. It is more likely to be the male of *Z. zayasi*.

### Zelus casii

Zhang & Hart sp. n.

urn:lsid:zoobank.org:act:4FCB0CDC-A7B4-45C6-B05A-4B609A12B293

#### Materials

- **Type status:** Holotype. **Occurrence:** catalogNumber: UCR_ENT 00048228; occurrenceRemarks: CAS Type No. 12716; recordedBy: C. E. & E. S. Ross; sex: Adult Male; otherCatalogNumbers: CAS Type No. 12716; **Taxon:** scientificName: Zelus casii; family: Reduviidae; genus: Zelus; scientificNameAuthorship: Zhang & Hart, 2016; **Location:** country: BRAZIL; stateProvince: Amapa; locality: Villa Amazonas; decimalLatitude: 0.03333; decimalLongitude: -51.05; **Identification:** identifiedBy: G. Zhang; dateIdentified: 2013; **Event:** eventDate: 1964-05-29; **Record Level:** institutionCode: CAS

#### Description

Figs 45, 46, 47

***Male***: (Fig. 45) Medium-sized; slender. **COLORATION**: Entirely brown, somewhat reddish; apices of femora slightly darkened; ventral surface of head, parts of pleura, and abdomen pale brown. **VESTITURE**: Sparsely setose. Dorsal surface of head with dark, short, erect, spine-like setae, denser on anterior lobe, and moderately dense, short, recumbent setae; ventral surface with sparse, short, recumbent setae and few long, erect setae. Anterior pronotal lobe nearly glabrous, few short, spine-like setae; posterior lobe with short, erect, spine-like setae, some apically curved; pleura with short to moderately long, recumbent and semi-erect setae, some covered with white waxy exudation; scutellum with recumbent setae. Legs with sparse setation. Corium and clavus with short, recumbent setae. Abdomen with sparse, short, recumbent setae, intermixed with few longer setae. Dorsal, outer surface of enlarged part of paramere with dense, long, erect setae. **STRUCTURE: Head**: Elongated. Postocular lobe very long; in dorsal view anteriorly gradually narrowing, posterior portion constant, slightly narrower. Eye prominent; lateral margin much wider than postocular lobe; dorsal margin attaining postocular transverse groove, ventral margin removed from ventral surface of head. **Thorax**: Anterolateral angle with inconspicuous subtuberculate projection; medial longitudinal sulcus evident throughout, deepening posteriorly. Posterior pronotal lobe with rugulose surface; disc distinctly elevated above humeral angle; humeral angle armed, with dentate projection. Scutellum moderately long; apex angulate, slightly projected upward. *Legs*: Slender. *Hemelytron*: Slightly surpassing apex of abdomen, not more than length of abdominal segment seven; quadrate cell moderately sized; Cu and M of cubital cell subparallel. **GENITALIA**: (Fig. 46) *Pygophore*: Ovoid; expanded laterally near base of paramere in dorsal view. Medial process expanded laterally; rather broad; moderately long; anteroposteriorly compressed; erect; curved at middle; apex emarginate, with pair of subapical, lateral, hooklike processes; lateral elevations running from below base of medial process through middle. *Paramere*: Short, not reaching apex of medial process; base slightly constricted; strongly curved ventrad. *Phallus*: Dorsal phallothecal sclerite somewhat squarish; lateral longitudinal blade-like heavy sclerotization pressed against phallothecal sclerite; apical portion of phallothecal sclerite gradually tapering, slightly convex, laterally angulate, angulation ending anteriorly in sharp, dorsad projection; apex rounded, medially emarginate; posterior margin of foramen concave. Struts attached to dorsal phallothecal sclerite; apically separate, connected by bridge; basally separate. Basal plate arm moderately robust; basally fused; in lateral view strongly curved at midpoint; bridge extremely short; extension of basal plate expanded onto arm.

***Female***: Unknown.

#### Diagnosis

Recognized by the uniform dark brown coloration; the extremely long postocular lobe; and the rather broad medial process, apex emarginate in the middle and bearing a pair of ventrally directed projections.

#### Etymology

Named after Casi.

#### Distribution

**Figure 47.**
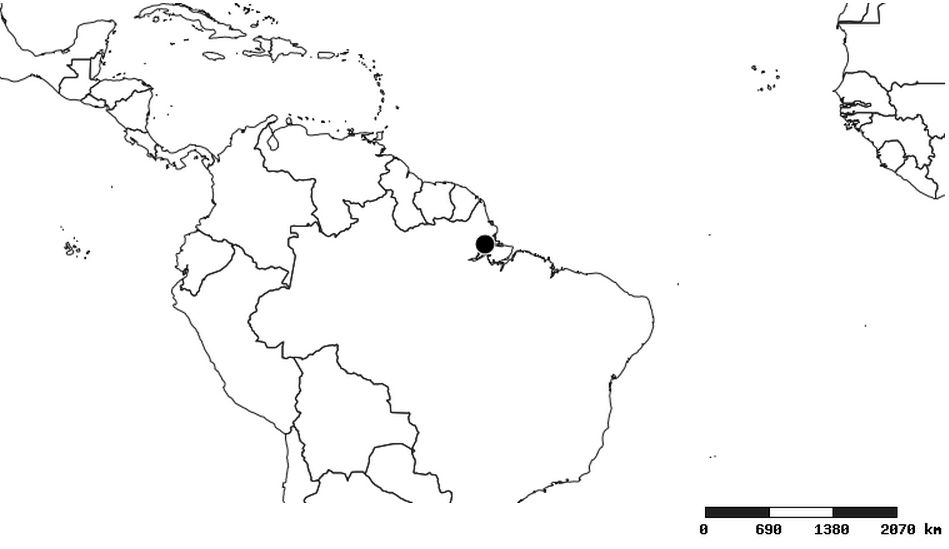
*Zelus casii* Zhang & Hart, **sp. n.**, specimen record map

South America (Fig. 47). A single specimen is known from the State of Amazonas, Brazil.

#### Taxon discussion

Several characters of *Z. casii* are highly unique among all species of *Zelus*. It has an extraordinarily long postocular lobe. The medial process is very broad, has lateral ridge-like elevations, and the apex is emarginate.

### Zelus cervicalis

Stål, 1872

Zelus cervicalis Stål, 1872, p. 90, orig. descr. (subgenus *Zelus*); Uhler, 1876, p. 61, list (reprint); Uhler, 1886, p. 24, checklist; Lethierry and Severin, 1896, p. 151, cat.; Champion, 1898, p. 255, cat.; Banks, 1910, p. 16, cat.; Torre-Bueno and Engelhardt, 1910, p. 150, note; Van Duzee, 1912, p. 324, senior syn. of *Z. marginata* (Provancher); Fracker, 1913, p. 239, 240, key and list (subgenus *Zelus*); Torre-Bueno, 1913, p. 60, list; Barber, 1914, p. 506, list; Van Duzee, 1916, p. 30, checklist (subgenus *Zelus*) ; Van Duzee, 1917, p. 260, cat. (subgenus *Zelus*); Dozier, 192,0 p. 357, list; Blatchley, 1926, p. 569, key and note (subgenus *Zelus*); Readio, 1927, p. 169, 170, key and descr.; Wygodzinsky, 1949a, p. 48, checklist; Elkins, 1951, p. 410, list; Sibley, 1951, p. 92, list; Kelton, 1968, p. 1071, note; Snow, 1906, p. 180, list; Van Duzee, 1909, p. 177, list; Osborne and Drake, 1915, p. 531, note; Brimley, 1938, p. 73, list; Elliott, 1938, p. 39, list; Tenhet and Howe, 1939, p. 24, note; Drew and Schaeffer, 1962, p. 106, list; Oliver, 1964, p. 316, note; Whitcomb and Bell , 1964, p. 22, List and note; Hart, 1986, p. 542-543, lectotype desig., redescription, note, fig. and key; Maldonado, 1990, p. 326, cat.
Evagoras marginata Provancher, 1887, p. 182–183, orig. descr.; Van Duzee, 1912, p. 324, junior syn. of *Z. cervicalis*; Kelton, 1968, p. 1071, note.
Zelus marginatus : Lethierry and Severin, 1896, p. 152, cat.; Banks, 1910, p. 16, cat.
Zelus pictipes Champion, 1898, p. 255, Tab. XV, fig. 14, orig. descr, and fig.; Fracker, 1913, p. 239, 240, key and list; Van Duzee, 1916, p. 30, checklist (subgenus *Zelus*) ; Van Duzee, 1917, p. 259, cat. (subgenus *Zelus*); Readio, 1927, p. 169, 170, key and descr.; Wygodzinsky, 1949a, p. 50, checklist; Snow, 1906, p. 180, list; Elkins, 1951, p. 410, list; Sibley, 1951, p. 92, list; Drew and Schaeffer, 1962, p. 106, list; Hart, 1986, p. 542, lectotype desig. and junior syn. of *Z. cervicalis*.

#### Description

Figs 48, 49, 50

***Male***: (Fig. 48a, b, c) Medium-sized, total length 9.81–13.08 mm (mean 11.78 mm, Suppl. material 2), very slender, body length/width=6.2. **COLORATION**: Yellowish-brown to dark brown, some specimens with dark spots or bands on legs. Anteocular lobe yellowish-brown to reddish-brown, dark brown between eye and antennal insertion, some specimens with dark brown mid-dorsal areas. Dorsum of postocular lobe dark brown, variably shaped medial longitudinal line and area between ocelli and eye yellowish-brown, ventral surface yellowish-brown. Labial segments I & II yellowish-brown; segment III reddish to dark brown. Antennal segments brown, sometimes scape darker on dorsal surface or pedicel darker apically. Anterior pronotal lobe yellowish-brown to brown, collar and setal tracts darker, some specimens with dark brown spot on pro-episternum. Posterior pronotal lobe yellowish-brown to brown. Pleura yellowish-brown. Sternites yellowish-brown; meso-sternum with dark brown area anterior to meso-coxa. Scutellum yellowish-brown to brown, apex lighter. Legs yellowish-brown, many specimens with dark brown raised spots or bands on femora and tibiae (see "Taxon Discussion" below). Corium and clavus reddish-brown, veins yellowish-brown; membrane yellowish-brown. Dorsum of abdomen yellowish, reddish, or dark brown; connexival margins and ventral surface yellowish-brown. Pygophore yellowish-brown; some specimens with medial process apically reddish-brown or brown. **VESTITURE**: Moderately setose. Pubescence of short recumbent and short to long erect setae. Anteocular lobe with short recumbent and erect setae over entire surface, more dense dorsally; postocular lobe with short to moderate recumbent and moderate to long erect setae, erect setae more dense posteriorly. With short to moderate recumbent setae over entire surface, confined to setal tracts on dorsum of anterior pronotal lobe, longer erect setae on lateral surface; scutellum with short recumbent and short to moderate semi-erect and erect setae over surface. Legs with short to long semi-erect to erect setae. Corium and clavus with short, recumbent setae. Abdomen with short recumbent and some short to moderate erect setae over ventral and lateral surfaces. Exposed surface of pygophore with short recumbent and short to long erect setae; short to moderately stiff erect setae on apical half of parameres. **STRUCTURE: Head**: Cylindrical, L/W = 2.83. Postocular lobe moderately long; in dorsal view anteriorly gradually narrowing, posterior portion constant, slightly narrower. Eye moderately sized; lateral margin only slightly wider than postocular lobe; dorsal and ventral margins removed from surfaces of head. *Labium*: I: II: III = 1.0: 2.0: 0.5. Basiflagellomere diameter larger than that of pedicel. **Thorax**: Anterolateral angle bearing small projection; medial longitudinal sulcus evident only on posterior 1/2, deepening anterior to transverse sulcus of pronotum. Posterior pronotal lobe with finely rugulose surface; disc slightly elevated above humeral angle; humeral angle armed, with dentate projection. Scutellum long; apex angulate, not projected. *Legs*: Slender. *Hemelytron*: Slightly surpassing apex of abdomen, not more than length of abdominal segment seven; quadrate cell small, elongate; Cu and M of cubital cell subparallel. **GENITALIA**: (Fig. 49) *Pygophore*: Ovoid. Medial process cylindrical; slender; long; laterally somewhat compressed; erect; nearly straight; basally without protrusion; apex in posterior view modified, hooklike. *Paramere*: Cylindrical; moderately long, achieving apex of medial process; directed toward medial process; basally narrower; curved dorsad; apical part enlarged. *Phallus*: Dorsal phallothecal sclerite shield-shaped; lateral margin recurved dorsad; apical portion of phallothecal sclerite gradually tapering, flat, lateral margin recurved; apex rounded, medially emarginate; posterior margin of foramen broadly concave. Struts attached to dorsal phallothecal sclerite; apically missing. Basal plate arm moderately robust; basally fused; in lateral view basally strongly curved; bridge short; extension of basal plate small, marginally expanded onto arm.

***Female***: (Fig. 48d, e, f) Similar to male, except for the following. Larger than male, total length 12.89–15.26 mm (mean 14.25 mm, Suppl. material 2). Basiflagellomere subequal in diameter to pedicel. Central 1/3 of mesofemur slightly swollen, pro- and meso-femoral diameters subequal, about 1.3–1.4x diameter of metafemur.

#### Diagnosis

The rather slender body form makes this species easy to separate from other species that occur in the same geographic region. Males can also be recognized by the paramere apically greatly enlarged; the medial process apically curved ventrad, hooklike; the lateral margin of the dorsal phallothecal sclerite recurved. *Zelus cervicalis* is most similar to *Z. renardii* and the two share a number putatively synapomorphic characters of structures of male genitalia. The more slender body separates both sexes of *Z. cervicalis* from *Z. renardii*. Males of *Z. cervicalis* also have the apex of medial process not bent as strongly as that in *Z. renardii*.

#### Distribution

**Figure 50.**
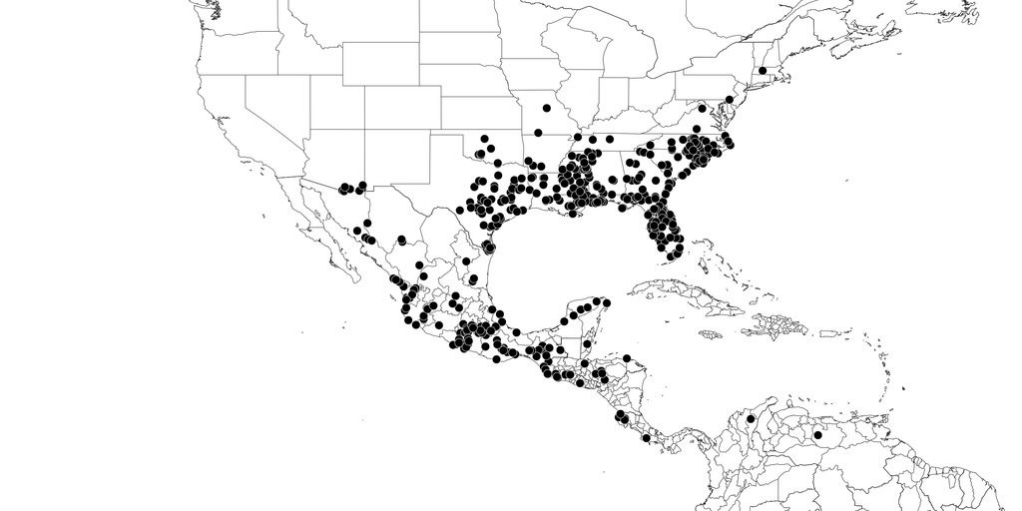
*Zelus cervicalis* Stål, 1872, specimen record map

South Atlantic and Gulf Coast states of the United States, southeastern Arizona, most of Mexico, Central America and Northern Colombia (Fig. 50). Countries with records: Belize, Colombia, Costa Rica, El Salvador, Guatemala, Honduras, Mexico, USA.

#### Taxon discussion

Hart (1986) stated that, based on male genitalic characters and pilosity, *Z. cervicalis* and *Z. renardii* are closely related species, and we agree with that view. We also corroborate, using a larger specimen sample, the western and eastern parapatric distribution pattern for *Z. renardii* and *Z. cervicalis* found by Hart. Based mainly on the coloration of the legs, Hart (1986) delimited two populations of *Z. cervicalis*, i.e., a South Atlantic and Gulf Coast population and a Mexico-Central America population, the latter also extending to southeastern Arizona and northern Colombia. Most individuals of the South Atlantic and Gulf Coast population have unicolorous legs, or, at most, only a few brownish to reddish spots. Specimens of the Mexico-Central America population have heavily spotted or banded legs. This pattern is also recovered in the current study. However, contrary to Hart's claim that "occasional specimens from either population may occur that do not conform to the normal pattern for that population", we found that all specimens of the Mexico-Central America population have spotted or banded legs. This condition also appears in a small number of specimens in other populations (e.g., UCR_ENT 00016129, UCR_ENT 00039079, UCR_ENT 00042740, UCR_ENT 00042741, UCR_ENT 39522, UCR_ENT 00039519, UCR_ENT 00039531, UCR_ENT 00039525, UCR_ENT 00039561, UCR_ENT 00039560, UCR_ENT 00039559, UCR_ENT 00039557, and more specimens from Texas). We also observed that compared to populations in other US states, specimens from southern Texas tend to have spotted legs, but the density of spots is lower than that in the Mexico-Central America population. By examining previously unstudied Mexican specimens from southern Sonora and northern Sinaloa, we also support Hart's second theory that the Arizona specimens are in continuity with the remainder of the population. The male genitalia are also variable in a number of respects between the two populations (Fig. 49), mainly in the shape of the paramere, the elevation of the lateral margins of the dorsal phallothecal sclerite near the base, and the relative massiveness of the basal plate arms. Hart remarked that the Mexico-Central American specimens show more similarities to the Gulf Coast specimens as one proceeds southward through Central America.

The images of the lectotype of *Z. cervicalis* are available on the 'Types of Heteroptera' website of the Swedish Museum of Natural History.

### Zelus chamaeleon

Stål, 1872

Zelus chamaeleon Stål, 1872, p. 90–91, orig. descr. and cat. (subgenus *Diplodus*); Lethierry and Severin, 1896, p. 151, cat.; Champion, 1898, p. 260, note; Wygodzinsky, 1949a, p. 48–49, checklist; Maldonado, 1990, p. 326, cat.

#### Materials

- **Type status:** Lectotype. **Occurrence:** catalogNumber: UCR_ENT 00041001; occurrenceRemarks: Lectotype of *Zelus chamaeleon* Stål, 1872. (**New designation** by Zhang, Hart & Weirauch, 2016). Verbatim label info: Bogota. / Lindig / v. niger Stal / designated by E.R.Hart / Lectotype Zelus chamaeleon Stal / NHRS-GULI 000000322; recordedBy: Lindig; sex: Adult Male; otherCatalogNumbers: NHRS-GULI 000000322; **Taxon:** scientificName: Zelus chamaeleon; family: Reduviidae; genus: Zelus; scientificNameAuthorship: Stål, 1872; **Location:** country: COLOMBIA; stateProvince: Cundinamarca; locality: Bogota; decimalLongitude: -75.16833; georeferenceSources: Gazetteer; **Identification:** dateIdentified: 2012; **Event:** eventDate: Date not provided; **Record Level:** institutionCode: NHRS
- **Type status:** Allolectotype. **Occurrence:** catalogNumber: None; occurrenceRemarks: Allolectotype of *Zelus chamaeleon* Stål, 1872. (**New designation** by Zhang, Hart & Weirauch, 2016). Labels: Bogota / Lindig / Typus / v. fasciativentris Stal.; recordedBy: Lindig; sex: Adult Female; **Taxon:** scientificName: Zelus chamaeleon; family: Reduviidae; genus: Zelus; scientificNameAuthorship: Stål, 1872; **Location:** country: COLOMBIA; stateProvince: Cundinamarca; locality: Bogota; decimalLongitude: -75.16833; georeferenceSources: Gazetteer; **Event:** eventDate: Date not provided; **Record Level:** institutionCode: NHRS
- **Type status:** Paralectotype. **Occurrence:** catalogNumber: UCR_ENT 00041002; occurrenceRemarks: Paralectotype of *Zelus chamaeleon* Stål, 1872 (**New designation** by Zhang, Hart & Weirauch, 2016). Verbatim label info: Lindig / Bogota. / Paralectotype Zelus chamaeleon Stal / designated by E.R.Hart / NHRS-GULI 000000323; recordedBy: Lindig; sex: Adult sex unknown; otherCatalogNumbers: NHRS-GULI 000000323; **Taxon:** scientificName: Zelus chamaeleon; family: Reduviidae; genus: Zelus; scientificNameAuthorship: Stål, 1872; **Location:** country: COLOMBIA; stateProvince: Cundinamarca; locality: Bogota; decimalLongitude: -75.16833; georeferenceSources: Gazetteer; **Identification:** dateIdentified: 2012; **Event:** eventDate: Date not provided; **Record Level:** institutionCode: NHRS
- **Type status:** Paralectotype. **Occurrence:** catalogNumber: UCR_ENT 00041003; occurrenceRemarks: Paralectotype of *Zelus chamaeleon* Stål, 1872 (**New designation** by Zhang, Hart & Weirauch, 2016). Verbatim label info: Lindig / Bogota. / v. mainticollis[?] Stal / Paralectotype *Zelus chamaeleon* Stal / designated by E.R.Hart / Typus / NHRS-GULI 000000327; recordedBy: Lindig; sex: Adult sex unknown; otherCatalogNumbers: NHRS-GULI 000000327; **Taxon:** scientificName: Zelus chamaeleon; family: Reduviidae; genus: Zelus; scientificNameAuthorship: Stål, 1872; **Location:** country: COLOMBIA; stateProvince: Cundinamarca; locality: Bogota; decimalLongitude: -75.16833; georeferenceSources: Gazetteer; **Identification:** dateIdentified: 2012; **Event:** eventDate: Date not provided; **Record Level:** institutionCode: NHRS
- **Type status:** Paralectotype. **Occurrence:** catalogNumber: UCR_ENT 00041013; occurrenceRemarks: Paralectotype of *Zelus chamaeleon* Stål, 1872 (**New designation** by Zhang, Hart & Weirauch, 2016). Verbatim label info: Bogota / Lindig / Paratypus / Lectotype *Zelus chamaeleon* Stal / designated by E.R.Hart / NHRS-GULI 0000003958; recordedBy: Lindig; sex: Adult sex unknown; otherCatalogNumbers: NHRS-GULI 0000003958; **Taxon:** scientificName: Zelus chamaeleon; family: Reduviidae; genus: Zelus; scientificNameAuthorship: Stål, 1872; **Location:** country: COLOMBIA; stateProvince: Cundinamarca; locality: Bogota; decimalLongitude: -75.16833; georeferenceSources: Gazetteer; **Identification:** dateIdentified: 2012; **Event:** eventDate: Date not provided; **Record Level:** institutionCode: NHRS

#### Description

Figs 51, 52, 53

***Male***: (Fig. 51a, b) Medium-sized, total length 12.50–14.06 mm (mean 13.45 mm, Suppl. material 2); robust. **COLORATION**: Usually entire surface of body black, some specimen with white posterior margin of posterior pronotal lobe, some with posterior pronotal lobe uniformly orangish-brown. Scape with or without band. Profemur with single pale brown band, protibia without band; meso- and metafemora with two or three bands, tibiae with single band. **VESTITURE**: Sparsely setose. Dorsum of head with dense, short, stout, recumbent, black setae and sparse, long, fine, erect setae, as long as width of eye in dorsal view; ventral surface with moderately dense, short, recumbent, fine setae and spare, moderately long, erect setae. Moderately dense, short, stout, recumbent setae on pronotum; anterior lobe also with sparse, long, erect setae; scutelum with dense, short to long, semi-erect setae; pleura with short to long, semi-erect or erect setae; sternites with dense, long, erect setae. Corium and clavus with short, recumbent setae. Abdomen with moderately dense, short, recumbent to sub-adpressed setae, intermixed with long, erect setae. With dense, short, adpressed setae, mixed with sparse, very long, moderately stout, semi-erect setae; apical half with dense, moderately long, stout, semi-erect setae. **STRUCTURE: Head**: Somewhat globular, L/W = 1.93. Postocular lobe short; in dorsal view narrowing till abrupt posterior constriction, very short behind constriction. Eye moderately sized; lateral margin much wider than postocular lobe; dorsal and ventral margins removed from surfaces of head. *Labium*: I: II: III = 1: 1.6: 0.4. Basiflagellomere diameter slightly larger than that of pedicel. **Thorax**: Anterolateral angle with inconspicuous subtuberculate projection; medial longitudinal sulcus evident only on posterior 1/2, deepening anterior to transverse sulcus of pronotum. Posterior pronotal lobe with rugulose surface; disc distinctly elevated above humeral angle; humeral angle armed, with spinous processes. Scutellum moderately long; apex angulate, slightly projected upward in some specimens. *Legs*: Slender. *Hemelytron*: Surpassing apex of abdomen by about twice length of abdominal segment seven; quadrate cell moderately large; Cu and M of cubital cell converging towards R. **GENITALIA**: (Fig. 52) *Pygophore*: Ovoid; mid-lateral fold adjacent to paramere insertion; expanded laterally near base of paramere in dorsal view. Medial process triangular; short, shorter than paramere; erect; straight; apex in posterior view acute, with hooklike projection extending towards downward, ending as transverse bridge. *Paramere*: Cylindrical; short, not reaching apex of medial process; directed posteriad, slightly curved towards medial process; basally narrower; slightly curved ventrad; apical part enlarged. *Phallus*: Dorsal phallothecal sclerite shield-shaped; lateral ridge-like dorsad expansion continuous with basal arm; apical portion of phallothecal sclerite gradually tapering, convex, medially keeled; apex truncate; posterior margin of foramen deeply concave. Struts attached to dorsal phallothecal sclerite; apically separate, connected by bridge; basally separate. Basal plate arm robust; separate; converging; in lateral view slightly curved; bridge short; extension of basal plate expanded onto arm.

***Female***: (Fig. 51c, d) Similar to male, except for the following. Larger than male, total length 13.45–15.02 mm (mean 14.39 mm, Suppl. material 2). More variable than in male. Dorsum of postocular lobe always black; pale, slender, medial longitudinal stripe; anteocular lobe usually black, if with part of surface red, clypeus always black. Dorsum of anterior pronotal lobe almost always black, collar sometimes red, lateral surface black or mixed with red. Dorsal surface of posterior pronotal lobe of four major color patterns: entirely black, entirely red or orange, anterior portion red and posteriorly black, mostly black with medial red circular patch; last pattern most common (eight out of fourteen); lateral surface always red or orange (when dorsal surface is orange). Variable amounts of black and red on pleura. Corium and clavus brownish-black or yellowish-brown; membrane always dark brown. Abdomen segment usually red, anterior black stripe; entirely black in some specimens. Hemelytron slightly surpassing apex of abdomen.

#### Diagnosis

Can be recognized by the following combination of characters: the long, erect, fine setae on head, anterior pronotal lobe, pleura and sternites; the stout and short head and the nearly hemispherical postocular lobe; the short paramere, not exceeding medial process; the medial process short and triangular, apex as hooklike process, extending ventrally as transverse ridge; and the apical surface of dorsal phallothecal sclerite medially with keel-like elevation.

#### Distribution

**Figure 53.**
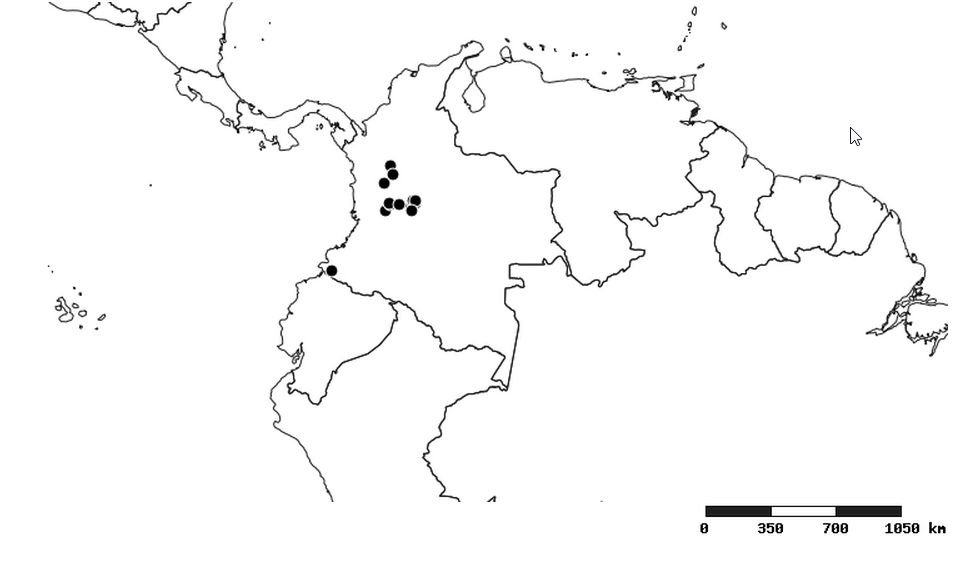
*Zelus chamaeleon* Stål, 1872, specimen record map

South America (Fig. 53). Known only from Colombia.

### Zelus championi

Zhang & Hart sp. n.

urn:lsid:zoobank.org:act:1A05766B-0C74-43CE-A5F9-A643EF07CA89

#### Materials

- **Type status:** Holotype. **Occurrence:** catalogNumber: UCR_ENT 00048759; occurrenceRemarks: Verbatim label info: B.C.A.Rhyn.II. Zelus inconstans Ch. / Bugaba, 800-1,500 ft. Champion. / Holotype *Zelus championi* Hart / [genitalia vial]; recordedBy: G.C. Champion; sex: Adult Male; **Taxon:** scientificName: Zelus championi; family: Reduviidae; genus: Zelus; scientificNameAuthorship: Zhang & Hart, 2016; **Location:** country: PANAMA; stateProvince: Chiriqui; locality: Bugaba; verbatimElevation: 457 m; decimalLatitude: 8.4833; decimalLongitude: -82.6167; georeferenceSources: Gazetteer; **Identification:** identifiedBy: G. Zhang; dateIdentified: 2013; **Event:** eventDate: No date provided; **Record Level:** institutionCode: BMNH
- **Type status:** Paratype. **Occurrence:** catalogNumber: UCR_ENT 00004770; occurrenceRemarks: Drake Collection; recordedBy: Unknown; sex: Adult Male; **Taxon:** scientificName: Zelus championi; family: Reduviidae; genus: Zelus; scientificNameAuthorship: Zhang & Hart, 2016; **Location:** country: ECUADOR; stateProvince: Napo; locality: 10 km W Cosanga; verbatimElevation: 2114 m; decimalLatitude: 0.59094; decimalLongitude: -77.88086; **Identification:** identifiedBy: G. Zhang; dateIdentified: 2013; **Event:** eventDate: 2005-03-10; **Record Level:** institutionCode: UCR
- **Type status:** Paratype. **Occurrence:** catalogNumber: UCR_ENT 00014406; occurrenceRemarks: Additional information on label: L N 217200_570300; recordedBy: G. Fonseca; sex: Adult Male; otherCatalogNumbers: INBIO CRI002 040338; **Taxon:** scientificName: Zelus championi; family: Reduviidae; genus: Zelus; scientificNameAuthorship: Zhang & Hart, 2016; **Location:** country: Costa Rica; stateProvince: Cartago; locality: Monumento Nacional Guayabo, Turrialba; verbatimElevation: 1100 m; decimalLatitude: 9.97159; decimalLongitude: -83.69072; georeferenceSources: Gazetteer; **Identification:** identifiedBy: G. Zhang; dateIdentified: 2013; **Event:** eventDate: 1903-01-04; **Record Level:** institutionCode: INBio

#### Description

Figs 54, 55, 56

***Male***: (Fig. 54) Medium-sized, total length 10.85–12.29 mm (mean 11.79 mm, Suppl. material 2); slender. **COLORATION**: Head, pronotum and hemelytron black; postocular lobe with light yellowish-brown mid-dorsal line; abdomen brightly red; pygophore black. **VESTITURE**: Densely setose. Dorsal surface of head with short, spine-like setae, pubescence of remainder of surface consisting of erect and recumbent setae. Pronotum with short, spine-like setae dorsally and laterally. Abdomen with scattered, erect setae of varying lengths. **STRUCTURE: Head**: Cylindrical, L/W = 2.00. Postocular lobe short; in dorsal view narrowing till abrupt posterior constriction, very short behind constriction. Eye smallish; lateral margin only slightly wider than postocular lobe; dorsal and ventral margins removed from surfaces of head. *Labium*: I: II: III=1: 1.4: 0.5. Basiflagellomere diameter larger than that of pedicel. **Thorax**: Anterolateral angle of collar rounded, without projection; medial longitudinal sulcus of anterior lobe shallow at collar, deepening posteriorly. Posterior pronotal lobe with rugulose surface; disc distinctly elevated above humeral angle; humeral angle rounded, without projection. Scutellum short; apex blunt, not projected. *Legs*: Moderately robust. Femoral diameters subequal. *Hemelytron*: Greatly surpassing apex of abdomen by about 3x length of abdominal segment seven; quadrate cell large and broad; Cu and M of cubital cell subparallel. **GENITALIA**: (Fig. 55) *Pygophore*: Ovoid; slightly expanded laterally near base of paramere in dorsal view; mid-lateral fold adjacent to paramere insertion. Medial process robust; tapering to apex; moderately long; laterally compressed towards apex; posteriorly directed; straight; apex in posterior view pointed, without modification or ornamentation. *Paramere*: Cylindrical; moderately long, nearly reaching apex of medial process; directed posteriad; narrower basally; slightly curved ventrad towards; apical portion not enlarged. *Phallus*: Dorsal phallothecal sclerite elongated; apical portion of phallothecal sclerite gradually tapering, slightly convex, laterally rounded, not forming angle; apex rounded, medially emarginate; angular processes arising near base, posterior margin of foramen deeply concave. Struts attached to dorsal phallothecal sclerite; apically separate, connected by bridge. Basal plate arm slender; separate; somewhat converging; in lateral view very slightly curved; bridge moderately long; extension of basal plate small, laterally not greatly expanded onto arm.

***Female***: Unknown.

#### Diagnosis

The strongly contrasting black dorsal surface and red abdomen is distinctive of this species. The features of the genitalia are rather similar to those of other species in the *Zelus vagans* species group (Fig. 11), but the apex of the medial process is more strongly bent ventrad. Other diagnostic characters shared with members of the *Zelus vagans* species group and the *Zelus longipes* species group include the unarmed rounded humeral angle and the spine-like setae on pronotum.

#### Etymology

This species epithet is a patronym, in honor of entomologist George C. Champion (1851-1927), who authored several volumes on Rhyncophora (Heteroptera) in the *Biologia Centrali Americana* series.

#### Distribution

**Figure 56.**
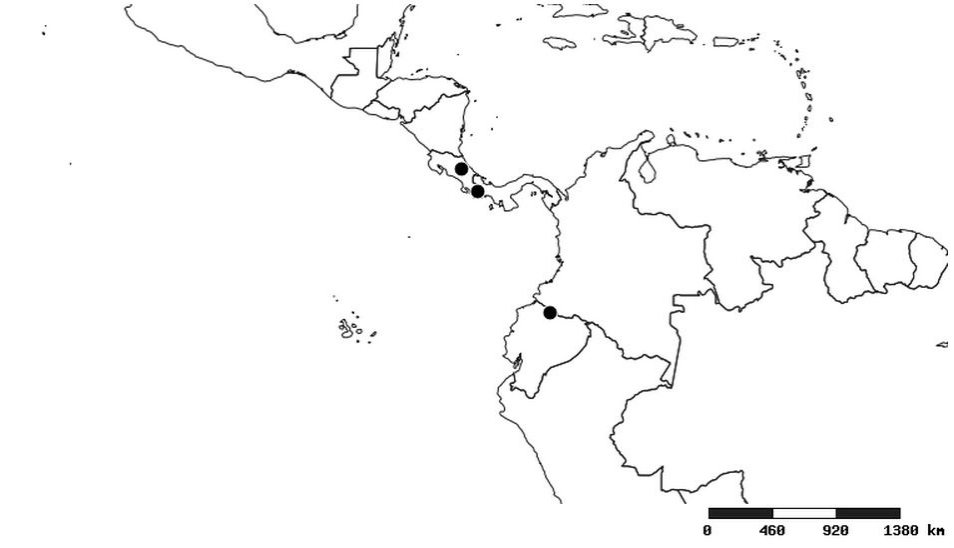
*Zelus championi*, Zhang & Hart, sp. n., specimen record map

Central and South America (Fig. 56). Countries with records: Costa Rica, Ecuador and Panama

#### Ecology

No natural history or ecological knowledge is known, but we hypothesize that the strikingly contrasting black and red coloration is at the same time cryptic and aposematic, and may also be mimetic. Based on observations of other species, we know that low vegetation is a common habitat of members of this genus. In a dense forest, predators from above may confuse the black dorsum of *Z. championi* with dark forest background, while the strong contrast formed by black and red colors is highly visible to predators (e.g., lizards) at the same level or approaching from below. Like many assassin bugs, species of *Zelus* may inflict a painful bite when attacked. Besides, harpactorines, including *Zelus* spp., emit a foul odor when handled. We do not know if vertebrate predators are deterred by this odor, but it is strong enough to be detected by a human even meters away. Hence *Z. championi* may be well defended against predators and the contrasting coloration serves as a signal for unpalatability. Of course, many other species of *Zelus* are dull colored, but expected to have the same kind of physical or chemical defense. There may be other ecological factors that determine the coloration of *Z. championi*. We suspect that mimicry is one. Many other unpalatable insects show similar contrasting bright red and black color patterns. *Zelus championi* may participate in Müllerian mimicry with those species.

#### Taxon discussion

The type specimen of this species was originally described as the male of *Z. inconstans*, a species very similar in general form to *Z. championi*. On the basis of pubescence, pronotal armature and whitish exudation, Champion himself questioned the conspecificity of this male with the three females of the original type series. As more material was available for the present work, his doubts have been substantiated, the male of *Z. inconstans* identified and this particular specimen found to be a male of a new species. The two species belong to different species groups, as verified by pubescence and genitalic characters.

### Zelus cognatus

(Costa, 1864)

Diplodus cognatus Costa, 1864, p. 81, orig. descr.; Uhler, 1886, p. 24, checklist; Walker, 1873, p. 125, cat.
Zelus cognatus : Stål, 1872, p. 91, cat. (subgenus *Diplodus*); Lethierry and Severin, 1896, p. 151, cat.; Champion, 1898, p. 259–260, junior syn. of *Z. exsanguis* Stål. **stat. rev.** (current study).

#### Description

The following is a translation of the original description:

"Closely related to the preceding species [*Z. ambulans*]; differing in that the spines of the humeral angle of the pronotum are conspicuously directed obliquely upward; dorsal surface of the head black, longitudinal line and transverse sulcus yellowish; first and second antennal segments testaceous, apex black. -- length 15 mm."

#### Taxon discussion

This species was originally described from a single specimen from Mexico. The original description did not indicate its sex. Champion’s synonymy was apparently based on the description and not upon examination of the specimen. Attempts to locate the holotype were unsuccessful. From the above original description it is impossible to determine whether this species may be synonymous with *Z. exsanguis* or is a separate species. It is reasonably certain, however, that it belongs to the *Zelus luridus* species group, as the comparison with *Z. ambulans* precludes any similarity to other reduviid genera or even groups in this genus within Mexico.

### Zelus conjungens

(Stål, 1860)

Diplodus conjungens Stål, 1860, p. 75, orig. descr.; Mayr, 1866, p. 138–139, junior syn. of *Z. armillatus*; Walker, 1873, p. 125, cat.
Zelus conjungens : Stål, 1872, p. 90, cat. (subgenus *Diplodus*); Lethierry and Severin, 1896, p. 151, junior syn. of *Z. armillatus*; Wygodzinsky, 1949a, p. 49, checklist. **stat. rev.** (current study).
Zelus atripes Champion, 1898, p. 259, Tab. XV. fig. 22, orig. descr. and fig.; Wygodzinsky, 1949a, p. 48, checklist; Maldonado, 1990, p. 326, cat. **syn. nov.** (current study).

#### Materials

- **Type status:** Lectotype. **Occurrence:** catalogNumber: UCR_ENT 00041000; occurrenceRemarks: Lectotype of *Zelus conjungens* (Stål, 1860). (**New Designation** by Zhang, Hart & Weirauch, 2016). Verbatim label info: Rio Jan / Stal / Lectotype *Zelus conjungens* (Stal) / designated by E.R.Hart / NHRS-GULI 000000320; recordedBy: Stal; sex: Adult Female; **Taxon:** scientificName: Zelus conjungens; family: Reduviidae; genus: Zelus; scientificNameAuthorship: (Stål, 1860); **Location:** country: BRAZIL; stateProvince: Rio de Janeiro; locality: unknown; verbatimElevation: NHRS-GULI 000000320; **Identification:** identifiedBy: G. Zhang; dateIdentified: 2013; **Event:** eventDate: No date provided; **Record Level:** institutionCode: NHRS
- **Type status:** Paralectotype. **Occurrence:** catalogNumber: UCR_ENT 00041000; occurrenceRemarks: Paralectotype of *Zelus conjungens* (Stål, 1860). (**New Designation** by Zhang, Hart & Weirauch, 2016). Verbatim label info: Rio Jan / Stal / Lectotype *Zelus conjungens* (Stal) / designated by E.R.Hart / NHRS-GULI 000000320; recordedBy: Stal; sex: Adult Female; **Taxon:** scientificName: Zelus conjungens; family: Reduviidae; genus: Zelus; scientificNameAuthorship: (Stål, 1860); **Location:** country: BRAZIL; stateProvince: Rio de Janeiro; locality: unknown; **Event:** eventDate: No date provided; **Record Level:** institutionCode: NHRS
- **Type status:** Other material. **Occurrence:** occurrenceRemarks: **Holotype** of *Zelus atripes* Champion, 1898 (junior synonym of *Zelus conjungens*). Bears the following labels: Type / Panama (Bouchard) / *Zelus atripes* Ch. / B.C.A. Sp. figured; recordedBy: Boucard; sex: Adult Female; **Taxon:** scientificName: Zelus conjungens; family: Reduviidae; genus: Zelus; scientificNameAuthorship: (Stål, 1860); **Location:** country: PANAMA; **Record Level:** institutionCode: BMNH

#### Description

Figs 57, 58, 59

***Male***: (Fig. 57a, b, c) Large, total length 16.64–19.80 mm (mean=18.34 mm, Suppl. material 2); robust. **COLORATION**: Variably yellowish-brown to brownish-black. Dorsal surface of head brown, mixed brownish-black; ventral surface brown. Anterior pronotal lobe variably brown to brownish-black, nearly entirely brownish-black in some specimens, never entirely yellowish-brown, with at least brownish-black spots. Posterior pronotal lobe usually brownish-black in center, margins yellowish-brown, entirely yellowish-brown in occasional specimens. Corium and clavus with proximal portion brownish-black, distal portion yellowish-brown, entire surface yellowish-brown in some specimens. Legs with or without bands. Lateral and ventral surfaces varying from most yellowish-brown with brownish-black spots to nearly entirely brownish-black. **VESTITURE**: Moderately setose. Very similar to that in *Z. armillatus*; adpressed setae more sparse. **STRUCTURE: Head**: Cylindrical, L/W = 2.29. Postocular lobe long; in dorsal view anteriorly gradually narrowing, posterior portion constant, slightly narrower. Eye smallish; lateral margin only slightly wider than postocular lobe; dorsal and ventral margins removed from surfaces of head. *Labium*: I: II: III = 1: 1.4: 0.4. Basiflagellomere diameter smaller than that of pedicel. **Thorax**: Anterolateral angle bearing small projection; medial longitudinal sulcus shallow near collar, deepening posteriorly. Posterior pronotal lobe with finely rugulose surface; disc slightly elevated above humeral angle; humeral angle armed, with short tuberculate processes. Scutellum moderately long; apex blunt, very slightly projected upward. *Legs*: Robust. *Hemelytron*: Slightly surpassing apex of abdomen, not more than length of abdominal segment seven; quadrate cell large and broad; Cu and M of cubital cell converging towards R. **GENITALIA**: (Fig. 58) *Pygophore*: Ovoid; slightly expanded laterally near base of paramere in dorsal view; posteriorly expanded sac-like sclerite between paramere and medial process. Medial process robust; broad; short; posteriorly directed; basally slightly protruding; apex in posterior view truncate, with very inconspicuous lateral prongs. *Paramere*: Cylindrical; moderately long, not reaching apex of medial process; directed posteriad; slightly curved dorsad; apical part not enlarged. *Phallus*: Dorsal phallothecal sclerite shield-shaped; lateral expansion arising close to base; apical portion of phallothecal sclerite not distinctly tapered, flat, laterally angulate; apex rounded; posterior margin of foramen broadly concave. Struts attached to dorsal phallothecal sclerite; apically separate, connected by bridge; basally separate. Basal plate arm moderately robust; separate; converging; in lateral view slightly curved; bridge moderately long; extension of basal plate expanded onto arm.

***Female***: (Fig. 57d, e, f) Similar to male, except for the following. Larger than male, total length 20.74–22.64 mm (mean 21.68 mm, Suppl. material 2). Mesofemur swollen.

#### Diagnosis

Among species of the *Zelus armillatus* species group, the medial process in *Z. conjungens* is the broadest, being more than 2x the diameter of paramere and more than 1.5x the diameter of ocellus. Other characters helpful for identification may include the lateral processes on apex of medial process minute, inconspicuous. Most similar to *Z. armillatus*, but can be separated by characters aforementioned, and also the lateral expansion on dorsal phallothecal sclerite close to basal arm not as sharp process. In females the mesofemur is swollen nearly throughout, much thicker than profemur, which will serve as a basis for separation from the females of *Z. armillatus*.

#### Distribution

**Figure 59.**
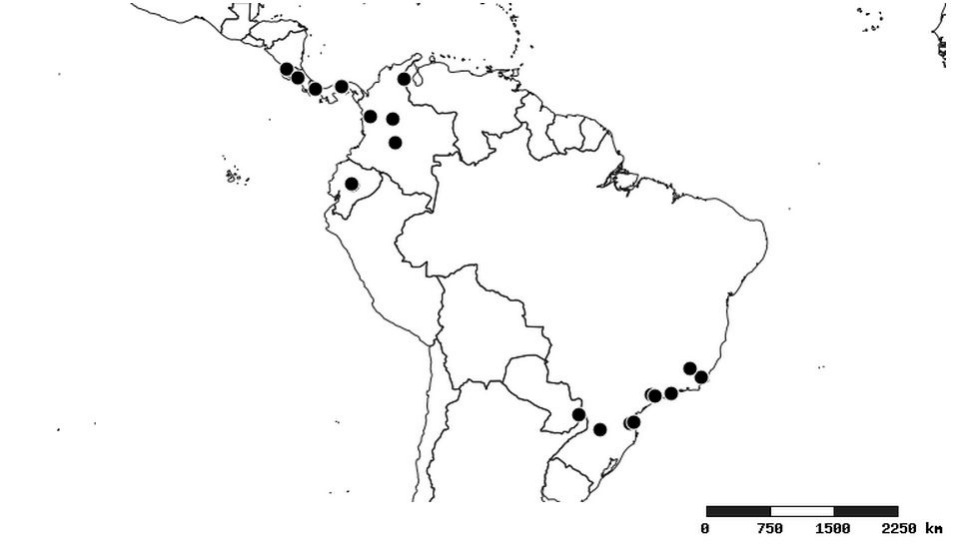
*Zelus conjungens* (Stål, 1860), specimen record map

Southern Central America, northern South America and Southern Brazil (Fig. 59). Countries with specimen records: Costa Rica, Colombia, Ecuador, Panama, Venezuela and Brazil.

#### Taxon discussion

Mayr (1866) synonymized *Z. conjungens* with *Z. armillatus*, the former as the junior synonym. We here resurrect this species on the basis of the characters described in the diagnosis. Two disjunct populations are recognized for this species, one in Southern Brazil and another in Central America and Northern South America. The latter represents a species formerly described by Champion, *Z. atripes*, which is here considered conspecific with *Z. conjungens*, as in both species the females show a swollen femur and the medial processes of males are broad. However, male genitalia of the two populations are somewhat different and may warrant a further closer examination. In any area in which the species occurs there is apparently a great range of color and pattern variations, but there does seem to be a general trend toward the lighter colorations in Colombia, Costa Rica and Panama.

Van der Heyden et al. (2014) reported this species as *Z. armillatus* when it was first discovered from Costa Rica. That identification is incorrect and should have been *Z. atripes*, which is now a synonym of *Z. conjungens*. *Zelus armillatus* does not occur in Central America.

### Zelus cordazulus

Zhang & Hart sp. n.

urn:lsid:zoobank.org:act:F74F78E1-D6F0-4A5D-820B-B21770431BAC

#### Materials

- **Type status:** Holotype. **Occurrence:** catalogNumber: UCR_ENT 00046973; recordedBy: Wygodzinsky; sex: Adult Male; **Taxon:** scientificName: Zelus cordazulus; family: Reduviidae; genus: Zelus; scientificNameAuthorship: Zhang & Hart, 2016; **Location:** country: PERU; stateProvince: Huanuco; locality: Divisoria, Cordillera Azul; verbatimElevation: 1300 m; decimalLatitude: -9.215; decimalLongitude: -75.846; georeferenceSources: Google Earth; **Identification:** identifiedBy: G. Zhang; dateIdentified: 2013; **Event:** eventDate: 1947-05-01; **Record Level:** institutionCode: AMNH
- **Type status:** Paratype. **Occurrence:** catalogNumber: UCR_ENT 00009529; recordedBy: Ranile; sex: Adult Male; **Taxon:** scientificName: Zelus cordazulus; family: Reduviidae; genus: Zelus; scientificNameAuthorship: Zhang & Hart, 2016; **Location:** country: PERU; stateProvince: Huanuco; locality: Divisoria, Cordillera Azul; verbatimElevation: 1300 m; decimalLatitude: -9.215; decimalLongitude: -75.846; georeferenceSources: Google Earth; **Identification:** identifiedBy: G. Zhang; dateIdentified: 2013; **Event:** eventDate: 1947-05-01; **Record Level:** institutionCode: USNM
- **Type status:** Paratype. **Occurrence:** catalogNumber: UCR_ENT 00023701; recordedBy: Unknown; sex: Adult Male; **Taxon:** scientificName: Zelus cordazulus; family: Reduviidae; genus: Zelus; scientificNameAuthorship: Zhang & Hart, 2016; **Location:** country: PERU; stateProvince: Junin; locality: Chanchamayo; decimalLatitude: -11.125; decimalLongitude: -75.357; georeferenceSources: Google Earth; **Identification:** identifiedBy: G. Zhang; dateIdentified: 2013; **Event:** eventDate: no date provided; **Record Level:** institutionCode: RMNH
- **Type status:** Paratype. **Occurrence:** catalogNumber: UCR_ENT 00026168; recordedBy: Ranile; sex: Adult Female; **Taxon:** scientificName: Zelus cordazulus; family: Reduviidae; genus: Zelus; scientificNameAuthorship: Zhang & Hart, 2016; **Location:** country: PERU; stateProvince: Huanuco; locality: Divisoria, Cordillera Azul; verbatimElevation: 1300 m; decimalLatitude: -9.215; decimalLongitude: -75.846; georeferenceSources: Google Earth; **Identification:** identifiedBy: G. Zhang; dateIdentified: 2013; **Event:** eventDate: 1947-05-01; **Record Level:** institutionCode: USNM
- **Type status:** Paratype. **Occurrence:** catalogNumber: UCR_ENT 00029354; recordedBy: F. Woytkowski; sex: Adult Female; **Taxon:** scientificName: Zelus cordazulus; family: Reduviidae; genus: Zelus; scientificNameAuthorship: Zhang & Hart, 2016; **Location:** country: PERU; stateProvince: Cusco; locality: S. Amer, Santa Isabel, Valley of River Ccosnipata; decimalLatitude: -13; decimalLongitude: -71.3; georeferenceSources: Gazetteer; **Identification:** identifiedBy: G. Zhang; dateIdentified: 2013; **Event:** eventDate: 1952-01-02; **Record Level:** institutionCode: USNM
- **Type status:** Paratype. **Occurrence:** catalogNumber: UCR_ENT 00029355; recordedBy: F. Woytkowski; sex: Adult Female; **Taxon:** scientificName: Zelus cordazulus; family: Reduviidae; genus: Zelus; scientificNameAuthorship: Zhang & Hart, 2016; **Location:** country: PERU; stateProvince: San Martin; locality: 15 kms SE of Moyobamba; verbatimElevation: 890 m; decimalLatitude: -6.09796; decimalLongitude: -76.8605; georeferenceSources: Google Earth; **Identification:** identifiedBy: G. Zhang; dateIdentified: 2013; **Event:** eventDate: 1947-08-10; **Record Level:** institutionCode: USNM
- **Type status:** Paratype. **Occurrence:** catalogNumber: UCR_ENT 00071256; recordedBy: Weyrauch; sex: Adult Female; **Taxon:** scientificName: Zelus cordazulus; family: Reduviidae; genus: Zelus; scientificNameAuthorship: Zhang & Hart, 2016; **Location:** country: PERU; stateProvince: Huanuco; locality: Divisoria (Cordillera Azul); verbatimElevation: 1500 m; decimalLatitude: -9.219; decimalLongitude: -75.829; georeferenceSources: Google Earth; **Identification:** identifiedBy: G. Zhang; dateIdentified: 2013; **Event:** eventDate: 1905-04-25; **Record Level:** institutionCode: TAMU

#### Description

Figs 60, 61, 62

***Male***: (Fig. 60a, b) Medium-sized, total length 14.80–15.07 mm (mean 14.94 mm, Suppl. material 2); slender. **COLORATION**: Head dark brown; yellowish patch between eye and ocellus; yellow, medial, longitudinal stripe on postocular lobe; ventral surface pale brown, lighter than dorsum. Pronotum and scutellum dark brown. Inconspicuous bands on legs. **VESTITURE**: Sparsely setose. Dorsum with moderately dense, short, recumbent setae and sparse, short, erect, somewhat spine-like setae; ventral surface with moderately dense, short, recumbent setae and few moderately long, erect, fine setae. Pronotum with very sparse, short, erect setae over dorsal surface, some setae curved apically, appearing recumbent; moderately dense, short to moderately long, recumbent setae on lateral surface and pleura, intermixed with semi-erect or erect setae; scutellum with sparse, semi-erect and recumbent setae. Legs with sparse setation on femora and moderately dense setation on tibiae. Corium and clavus with short, recumbent setae. Abdomen with moderately dense, short recumbent setae, intermixed with sparse, short to long, erect setae. **STRUCTURE: Head**: Cylindrical, L/W = 2.23. Postocular lobe long; in dorsal view distinctly narrowing through anterior 2/3, posterior 1/3 constant, tube-like. Eye prominent; lateral margin much wider than postocular lobe; dorsal margin attaining postocular transverse groove, ventral margin removed from ventral surface of head. *Labium*: I: II: III = 1: 1.8: 0.3. Basiflagellomere diameter larger than that of pedicel. **Thorax**: Anterolateral angle with inconspicuous subtuberculate projection; medial longitudinal sulcus evident throughout, deepening posteriorly. Posterior pronotal lobe with rugulose surface; disc distinctly elevated above humeral angle; humeral angle armed, with spinous processes. Scutellum moderately long; apex angulate, not projected. *Legs*: Slender. *Hemelytron*: Surpassing apex of abdomen by about length of abdominal segment seven; quadrate cell small and slender; Cu and M of cubital cell subparallel. **GENITALIA**: (Fig. 61) *Pygophore*: Elongate ovoid; lightly sclerotized expansion below paramere; not expanded laterally in dorsal view. Medial process cylindrical; slender; long, much longer than paramere; laterally compressed towards apex; anterior surface towards apex ridged; minute spicules on posterior surface; semi-erect; curved at middle; apex in posterior view acute, with small hooklike projection. *Paramere*: Cylindrical; short, not reaching medial process; directed posteriad; basally narrower; slightly curved ventrad; apical part very slightly enlarged. *Phallus*: Dorsal phallothecal sclerite shield-shaped; sclerotization reduced (yet not absent) on dorsal surface close to posterior margin of foramen; expansion of lateral margin at about mid-portion pronounced, covering lateral side of endosoma; apical portion of phallothecal sclerite gradually tapering, distinctly keeled medially, laterally flat, not forming angle; apex truncate; posterior margin of foramen broadly concave. Struts attached to dorsal phallothecal sclerite; apically separate, connected by bridge; basally separate. Basal plate arm moderately robust; separate; converging; in lateral view slightly curved; bridge short; extension of basal plate expanded laterally onto arm, covering more than 1/2 of arm, curved.

***Female***: (Fig. 60c, d) Similar to male, except for the following. Larger than male, total length 15.56–18.20 mm (mean 16.82 mm, Suppl. material 2). Dorsum nearly uniformly brown, lateral and ventral surfaces and legs yellowish; single dark spot on each abdominal segment.

#### Diagnosis

The nearly uniform brown dorsal coloration; the dorsum with short, erect, somewhat spine-like setae; and the posterior margin of the pronotum smoothly convex. The paramere short, broad; the apical part of medial process laterally compressed and ridged on the anterior surface; the posterolateral rim of pygophore with lightly sclerotized expansion below paramere; and the basal plate arm separate, not fused. In females the dorsum is nearly uniformly brown, the lateral and ventral surfaces yellowish, the legs apically reddish-brown, not conspicuously banded and the abdominal segment with single dark spot.

#### Etymology

Named after the type locality "Cordillera" in Peru.

#### Distribution

**Figure 62.**
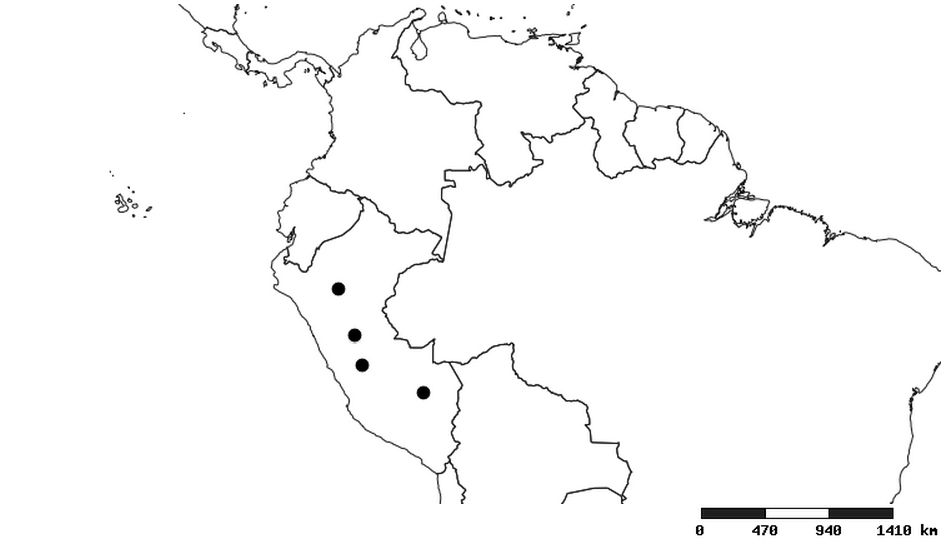
*Zelus cordazulus* Zhang & Hart, sp. n., specimen record map

South America (Fig. 62). Known only from Peru.

### Zelus couturieri

Iquitozelus couturieri Bérenger, 2003, p. 23, orig. descr. and fig.
Zelus couturieri (Bérenger, 2003), **comb. nov.** (current study).

#### Materials

- **Type status:** Holotype. **Occurrence:** occurrenceRemarks: Host plant: Psidium guajava; recordedBy: Couturier, G; sex: Adult Female; **Taxon:** scientificName: Zelus couturieri; family: Reduviidae; genus: Zelus; scientificNameAuthorship: (Berenger, 2003); **Location:** country: Peru; stateProvince: Loreto; locality: Iquitos; **Event:** eventDate: 1992-07-20; **Record Level:** institutionCode: Muséum national d'Histoire naturelle
- **Type status:** Paratype. **Occurrence:** occurrenceRemarks: Host plant: Psidium guajava; recordedBy: Couturier, G; sex: Adult Female; **Taxon:** scientificName: Zelus couturieri; family: Reduviidae; genus: Zelus; scientificNameAuthorship: (Berenger, 2003); **Location:** country: Peru; stateProvince: Loreto; locality: Iquitos; **Event:** eventDate: 1992-07-20; **Record Level:** institutionCode: Museo de Entomologia, Universidad Agraria La Molina, Lima
- **Type status:** Paratype. **Occurrence:** occurrenceRemarks: Host plant: Psidium guajava; recordedBy: Couturier, G; sex: Adult Female; **Taxon:** scientificName: Zelus couturieri; family: Reduviidae; genus: Zelus; scientificNameAuthorship: (Berenger, 2003); **Location:** country: Peru; stateProvince: Loreto; locality: Iquitos; **Event:** eventDate: 1992-07-20; **Record Level:** institutionCode: USNM
- **Type status:** Paratype. **Occurrence:** occurrenceRemarks: Host plant: Psidium guajava; recordedBy: Couturier, G; sex: Adult Female; **Taxon:** scientificName: Zelus couturieri; family: Reduviidae; genus: Zelus; scientificNameAuthorship: (Berenger, 2003); **Location:** country: Peru; stateProvince: Loreto; locality: Iquitos; **Event:** eventDate: 1992-07-20; **Record Level:** institutionCode: Jean-Michel Berenger Collection

#### Description

Figs 63, 64

#### Diagnosis

The connexivum segment VI with foliaceous expansion is unique among all females of *Zelus* spp.

#### Distribution

**Figure 64.**
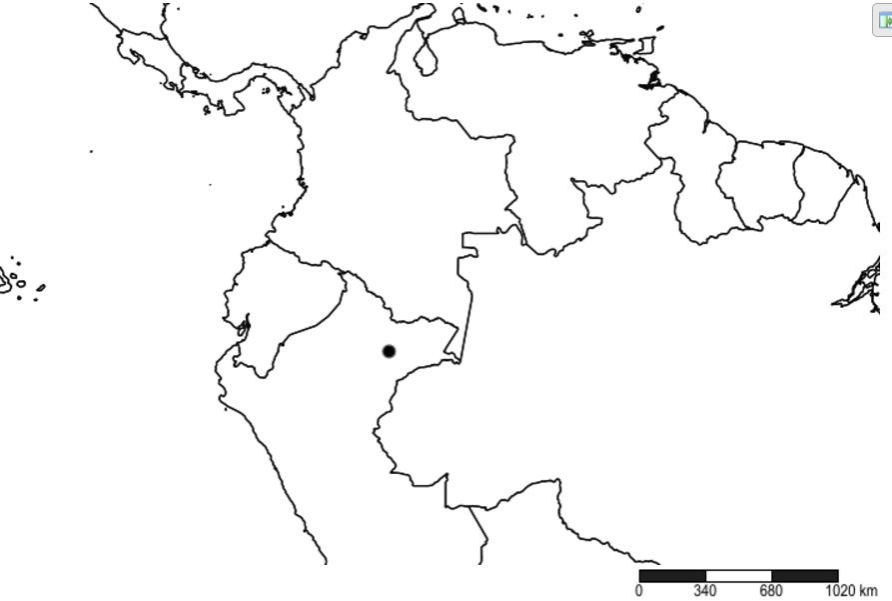
*Zelus couturieri* (Berenger, 2003), specimen record map.

South America (Fig. 64). Known only from Peru.

#### Taxon discussion

Bérenger (2003) described a new species of Harpactorini from Iquitos, Peru, and erected a new genus, *Iquitozelus* to accommodate this species. We here transfer *I. couturieri* to *Zelus* for reasons aforementioned. This species shows a great deal of resemblance to three other species of *Zelus*, *Z. amblycephalus*, *Z. umbraculus* and *Z. umbraculoides*, all newly described in the current study. In these species, the head, legs and pronotum are more or less uniformly greenish-brown. There are a number of differences. The posterior pronotal lobe disc in *Z. couturieri* appears to be only slightly above and nearly continuous with humeral angle. In the other three species, the discs are clearly above humeral angle. The posterior margin of the posterior pronotal lobe in *Z. couturieri* is not developed, whereas it is well defined in other species. Furthermore, the lateral process of the humeral angle is conspicuously darkened, also unique to *Z. couturieri*. Based the foregoing observations, it is likely that *Z. couturieri* represents a distinct a species, and not the female of *Z. umbraculoides* or *Z. umbraculus*. The specimens were not physically examined as Bérenger (2003) has provided a detailed description of this species.

### Zelus errans

Fabricius, 1803

Zelus errans Fabricius, 1803, p. 282, orig. descr.; Stål, 1868, p.108, descr., note and senior syn. of *Z. cursitans*; Stål, 1872, p.88, cat.; Walker, 1873, p. 135, cat.; Wygodzinsky, 1949a, p. 49, checklist; Maldonado, 1990, p. 326, cat.
Zelus cursitans Fabricius, 1803, p. 284, orig. descr.; Blanchard, 1840, p. 101, descr.; Stål, 1868,p. 108, junior syn. of *Z. errans*.

#### Materials

- **Type status:** Lectotype. **Occurrence:** occurrenceRemarks: Lectotype of *Zelus errans* Fabricius, 1803 (**New designation** by Zhang, Hart & Weirauch, 2016). Labels: *Zelus errans* in Am. mer. Schmidt / Type; recordedBy: Dom. Smidt; sex: Adult Male; **Taxon:** scientificName: Zelus errans; family: Reduviidae; genus: Zelus; scientificNameAuthorship: Fabricius, 1803; **Location:** country: unknown; stateProvince: unknown; locality: Habitat in America meridionali; **Event:** eventDate: No date provided; **Record Level:** institutionCode: ZMUC
- **Type status:** Paralectotype. **Occurrence:** occurrenceRemarks: Paralectotype of *Zelus errans* Fabricius, 1803 (**New designation** by Zhang, Hart & Weirauch, 2016). Labels: *Zelus errans* in Am. mer. Schmidt / Type; recordedBy: Dom. Smidt; sex: Adult Male; **Taxon:** scientificName: Zelus errans; family: Reduviidae; genus: Zelus; scientificNameAuthorship: Fabricius, 1803; **Location:** country: unknown; stateProvince: unknown; locality: Habitat in America meridionali; **Event:** eventDate: No date provided; **Record Level:** institutionCode: ZMUC
- **Type status:** Paralectotype. **Occurrence:** occurrenceRemarks: Paralectotype of *Zelus errans* Fabricius, 1803 (**New designation** by Zhang, Hart & Weirauch, 2016). Label: Zeluserrans; **Taxon:** scientificName: Zelus errans; family: Reduviidae; genus: Zelus; scientificNameAuthorship: Fabricius, 1803; **Record Level:** institutionCode: ZMUC

#### Description

Figs 65, 66, 67

***Male***: (Fig. 65a, b) Medium-sized, total length 13.62–17.91 mm (mean 14.10 mm, Suppl. material 2); slender. **COLORATION**: Many specimens with wasp-like habitus, with alternating black and yellow areas; anterior pronotal lobe usually dark brown, posterior lobe and proximal portion of corium yellowish; some specimens with nearly entire dorsal surface dark. Legs vary from nearly uniformly yellow or blackish-brown to yellow-brown banded. **VESTITURE**: Densely setose. Head with dark, erect, spine-like setae dorsally and light, recumbent setae ventrally. Anterior pronotal lobe with short, dark, spine-like setae, confined to setal tracts; posterior pronotal lobe with short, spine-like setae dorsally, fine, recumbent and erect setae on lateral surfaces. Abdomen with sparse, short, semi-erect setae. **STRUCTURE: Head**: Cylindrical, L/W = 2.28. Postocular lobe long; in dorsal view distinctly narrowing through anterior 2/3, posterior 1/3 constant, tube-like. Eye prominent; lateral margin much wider than postocular lobe; dorsal margin removed from postocular transverse groove, ventral margin attaining ventral surface of head. *Labium*: I: II: III = 1: 1.6: 0.4. Basiflagellomere diameter larger than that of pedicel. **Thorax**: Anterolateral angle rounded, without projection; medial longitudinal sulcus evident only on posterior 1/2, deepening anterior to transverse sulcus of pronotum. Posterior pronotal lobe with smooth surface; disc distinctly elevated above humeral angle; humeral angle rounded, without projection. Scutellum long; apex angulate, not projected. *Legs*: Very slender. *Hemelytron*: Greatly surpassing apex of abdomen by about 3x length of abdominal segment seven; quadrate cell large and broad; Cu and M of cubital cell subparallel. **GENITALIA**: (Fig. 66) *Pygophore*: Ovoid; mid-lateral fold adjacent to paramere insertion; not expanded laterally in dorsal view. Medial process cylindrical; extremely slender; moderately long; semi-erect; apex in posterior view blunt, slightly folded posteriad. *Paramere*: Cylindrical; long, surpassing medial process; directed posteriad, slightly curved towards medial process; slightly curved ventrad; apical part very slightly enlarged. *Phallus*: Dorsal phallothecal sclerite elongated; apical portion of phallothecal sclerite not distinctly tapered, convex, laterally indistinctly angulate; apex truncate, medially emarginate; posterior margin of foramen deeply concave. Struts attached to dorsal phallothecal sclerite; apically separate, connected by bridge; basally almost completely fused. Basal plate arm slender; separate; converging; in lateral view very slightly curved; bridge moderately long; extension of basal plate small, marginally expanded onto arm.

***Female***: (Fig. 65c, d, e) Similar to male, except for the following. Larger than male, total length 18.30–20.18 mm (mean 18.97 mm, Suppl. material 2). Coloration pattern more variable than in male.

#### Diagnosis

May be confused with *Z. vespiformis* and *Z. gracilipes*, species that have similar appearances. Distinguished from *Z. vespiformis* by the more elongated body; the longer medial process (Fig. 10), the Cu and Pcu of quadrate cell subparallel, and the Cu-Pcu2 (posterior cross vein) less than 1/2x length of Cu. Males of *Z. errans* can be separate from *Z. gracilipes* by features of the genitalia, the latter belonging to a different species group. Females typically have the anterior membrane of the hemelytron semi-translucent, whereas the entire membrane is colored or opaque in *Z. gracilipes*.

#### Distribution

**Figure 67.**
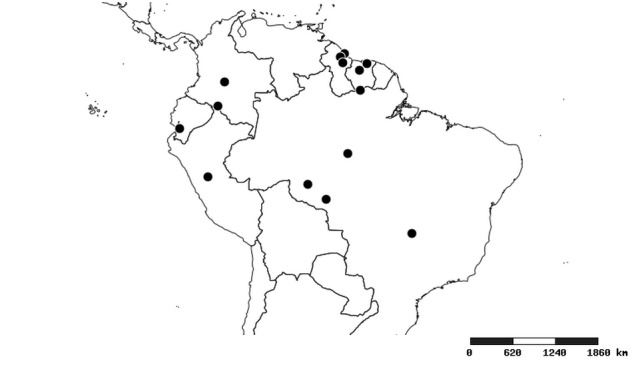
*Zelus errans* Fabricius 1803, specimen record map

South America (Fig. 67). Countries with specimen records: Bolivia, Brazil, Colombia, French Guiana, Paraguay, Peru and Venezuela.

#### Taxon discussion

*Zelus errans* is quite closely related to *Z. vespiformis*, and they are possibly sister species. These two species appear to be allopatric, with their boundaries roughly lying across central Colombia and southern Venezuela.

### Zelus erythrocephalus

Fabricius, 1803

Zelus erythrocephalus Fabricius, 1803, p. 283, orig. descr.; Blanchard, 1840, p. 101, cat. (erytrocephalus sic.); Stål, 1872, p. 92, cat. (subgenus *Diplodus*); Bergroth, 1893, p. 63, note; Lethierry and Severin, 1896, p. 152, cat.; Champion, 1898, p. 257, note; Brindley, 1931, p. 137, 151, list and note; Wygodzinsky, 1949a, p. 49, checklist; Zimsen, 1964, p. 338, list; Maldonado, 1990, p. 327, cat.
Euagoras erythrocephalus : Burmeister, 1835, p. 227, list.
Diplodus erythrocephalus : Stål, 1868, p. 283, descr.; Walker, 1873, p. 125, cat.; Van Duzee, 1901, p. 351, note.

#### Materials

- **Type status:** Lectotype. **Occurrence:** catalogNumber: UCR_ENT 00075109; occurrenceRemarks: Lectotype of *Zelus erythrocephalus* Fabricius, 1803 (**New Designation** by Zhang, Hart & Weirauch, 2016). Bears labels: Type /*Zelus erythrocephalus* in Am. mer. Schmidt; recordedBy: Dom. Smidt; sex: Adult Male; **Taxon:** scientificName: Zelus erythrocephalus; family: Reduviidae; genus: Zelus; scientificNameAuthorship: Fabricius, 1803; **Location:** country: unknown; stateProvince: unknown; locality: Habitat in America meridionali; **Identification:** identifiedBy: G. Zhang; dateIdentified: 2012; **Event:** eventDate: No date provided; **Record Level:** institutionCode: ZMUC

#### Description

Figs 68, 69, 70

***Male***: (Fig. 68a, b) Medium-sized, total length 12.07–12.77 mm (mean 12.49 mm, Suppl. material 2); slender. **COLORATION**: Head reddish-brown, anterior to antennal insertion and posterior third of postocular lobe lighter. Rest of surface of body nearly uniformly blackish-brown; area around humeral angle lighter, somewhat reddish. Membrane with blue, purple iridescence. **VESTITURE**: Moderately setose. Dark, moderately dense, short, erect, spine-like setae on dorsum of head, curved on postocular lobe; ventral surface with sparse, short, erect and recumbent setae, few long setae. Pronotal dorsum nearly glabrous, very sparse, short, erect, spine-like setae; lateral surface with sparse, erect to recumbent, spine-like setae; setal tracts on anterior lobe very reduced. Pleura with very sparse, spine-like setae and recumbent setae. Corium and clavus with sparse, short, recumbent setae. Abdomen with sparse, short, erect setae, intermixed with few long setae. Pygophore with sparse, short to long, semi-erect setae. Paramere apical 1/2 with dense, long setae, nearly as long as medial process. **STRUCTURE: Head**: Cylindrical, L/W = 2.29. Postocular lobe long; in dorsal view distinctly narrowing through anterior 2/3, posterior 1/3 constant, tube-like. Eye prominent; lateral margin much wider than postocular lobe; dorsal margin removed from postocular transverse groove, ventral margin attaining ventral surface of head. *Labium*: I: II: III = 1: 2.0: 0.4. Basiflagellomere diameter larger than that of pedicel. **Thorax**: Anterolateral angle bearing small projection; medial longitudinal sulcus shallow near collar, deepening posteriorly. Posterior pronotal lobe with rugulose surface; disc distinctly elevated above humeral angle; humeral angle armed, with dentate projection. Scutellum moderately long; apex angulate, very slightly projected upward. *Legs*: Very slender. *Hemelytron*: Surpassing apex of abdomen by about length of abdominal segment seven; quadrate cell small, relatively broad; Cu and M of cubital cell subparallel. **GENITALIA**: (Fig. 69) *Pygophore*: Ovoid; mid-lateral fold adjacent to paramere insertion inconspicuous; not expanded laterally in dorsal view. Medial process cylindrical; very slender; long; erect; straight; apex in posterior view acute, with subapical hooklike lateral processes. *Paramere*: Cylindrical; long, surpassing medial process; curved ventrad at mid-point, apex recurved. *Phallus*: Dorsal phallothecal sclerite shield-shaped, sclerite absent laterad to basal arms; lateral longitudinal blade-like heavy sclerotization, elevated, surpassing apical margins; apical portion of phallothecal sclerite not distinctly tapered, flat, lateral margin narrowly angulate, angulation ending anteriorly in sharp, dorsad projection; apex with small medial emargination; posterior margin of foramen broadly concave. Struts attached to dorsal phallothecal sclerite; apically separate, connected by bridge; basally fused. Basal plate arm robust; basally fused; in lateral view strongly curved at midpoint; bridge extremely short; extension of basal plate expanded onto arm.

***Female***: (Fig. 68c, d) Similar to male, except for the following. Larger than male, total length 16.85–19.06 mm (mean 17.92 mm, Suppl. material 2). Spinous process on humeral angle long.

#### Diagnosis

Recognized by the following combination of characters: the dorsal coloration nearly uniformly dark brown, the head reddish-brown, and the membrane with indistinct iridescence. Most similar to *Z. paracephalus* and *Z. russulumus*; can be distinguished from both by the rather slender medial process. Females of *Z. erythrocephalus*, *Z. paracephalus* and *Z. russulumus* are difficult to separate.

#### Distribution

**Figure 70.**
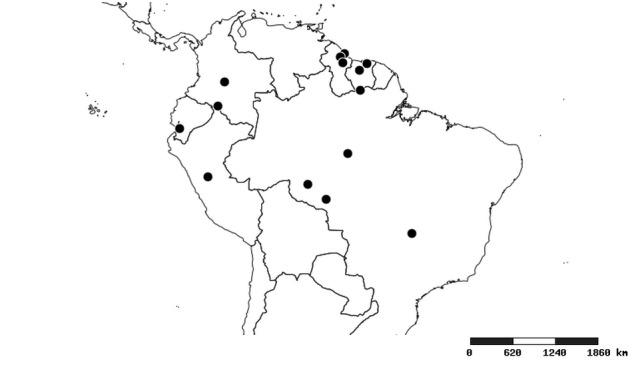
*Zelus erythrocephalus* Fabricius, 1803, specimen record map

South America (Fig. 70). Countries with records: Brazil, Colombia, Ecuador, Guyana, Peru and Suriname.

#### Taxon discussion

*Zelus erythrocephalus* and two other species in the same species group, *Z. paracephalus* and *Z. russulumus* superficially resemble *Z. panamensis*, a species in a different group. All have a orange, reddish head and a uniformly dark dorsum. These can be separated from *Z. panamensis* based on male genitalia and iridescence on membrane.

### Zelus exsanguis

Stål, 1862

Zelus exsanguis Stål, 1862, p. 452, orig. descr.; Stål, 1872, p. 91, cat. (subgenus *Diplodus*); Walker, 1873, p. 124, cat.; Uhler, 1894, p. 283, list; Lethierry and Severin, 1896, p. 152, cat.; Champion, 1898, p. 259–260, senior syn. of *Z. luridus* Stål, *Z. ambulans* Stål and *Z. cognatus* Costa; Banks, 1910, p. 16, cat.; Fracker, 1913, p- 239, 240, 241, key, list and note (subgenus *Diplodus*); Van Duzee, 1916, p, 30, checklist (subgenus *Diplodus*); Van Duzee, 1917, p. 260, cat. (subgenus *Diplodus*); Wygodzinsky, 1949a, p. 49, checklist; Hart, 1986, p. 539, redescription, lectotype desig., note, fig. and key; Maldonado, 1990, p. 327, cat.
Diplodus exsanguis : Uhler, 1886, p. 24, checklist.

#### Materials

- **Type status:** Lectotype. **Occurrence:** catalogNumber: UCR_ENT 00075071; occurrenceRemarks: Lectotype of *Zelus exsanguis* Stål, 1862, designated by Hart (1986). Verbatim label info: Mexico coll. Signoret / exsanguis det. Stal / B.C.A. Rhyn.II. *Zelus exsanguis* St. / Lectotype *Zelus exsanguis* Stal / designated by E. R. Hart / Lectotypus *Zelus exsanguis* STAL, 1862 etik. Hecher 1996 REDV. 470/1; recordedBy: Signoret; sex: Adult Female; **Taxon:** scientificName: Zelus exsanguis; family: Reduviidae; genus: Zelus; scientificNameAuthorship: Stål, 1862; **Location:** country: MEXICO; stateProvince: unknown; locality: unknown; **Identification:** identifiedBy: G. Zhang; dateIdentified: 2012; **Event:** eventDate: No date provided; **Record Level:** institutionCode: NHMW

#### Description

Figs 71, 72, 73

***Male***: (Fig. 71a, b) Medium-sized, total length 13.79–16.41 mm (mean 14.98 mm, Suppl. material 2); slender. **COLORATION**: Anteocular lobe reddish-brown, most specimens with central dorsal area darker reddish-brown and with lighter mid-dorsal line, dark areas between compound eyes and antennal insertions. Postocular lobe yellowish-brown to reddish-brown, dorsal surface dark brown to brownish-black with mid-dorsal and circumocellar areas yellowish-brown to reddish-brown. Rostrum yellowish-brown to reddish-brown. Antennae reddish-brown, flagellomeres darker, base and apex of scape and pedicel dark brown. Anterior pronotal lobe yellowish-brown to reddish-brown, some specimens with dark brown areas on longitudinal medial sulcus and anterolateral margins. Posterior lobe reddish-brown dorsally with yellowish-brown lateral and posterior margins, lateral surfaces yellowish-brown, dark brown areas at anterior or dorsolateral margins and humeral angle. Scutellum yellowish-brown to reddish-brown. Legs yellowish-brown, most specimens with wide reddish-brown to brownish-black band at apices of femora and small dark area at apices of tibiae. Hernelytron brown with costal margin and veins of corium yellowish-brown. Abdomen venter reddish-brown. **VESTITURE**: Moderately setose. Anteocular lobe with short recumbent setae dorsally and laterally, short to moderately long erect setae on ventral surface. Postocular lobe with short, recumbent setae on dorsal and lateral surfaces, moderately erect setae scattered over surface, long, fine setae laterally. Anterior pronotal lobe with short, recumbent setae confined to setal tracts, long silky erect setae laterally. Posterior pronotal lobe with recumbent and sparse, erect setae. Meso- and metapleural surfaces with long silky erect setae. Scutellum with silky, erect setae. Clavus and corium of hemelytron with recumbent setae. Dorsum of abdomen with short erect setae, remainder of surface with short recumbent and short to moderately long erect setae, margin of connexivum fringed with erect setae. Exposed surface of pygophore with short to long erect setae. Apex of paramere with long erect setae. **STRUCTURE: Head**: Cylindrical, L/W = 2.31. Postocular lobe moderately long; in dorsal view anteriorly gradually narrowing, posterior portion constant, slightly narrower. Eye smallish; lateral margin only slightly wider than postocular lobe; in lateral view margins removed from dorsal and ventral surfaces of head. Ocellus noticeably elevated. *Labium*: I: II: III = 1: 1.8: 0.5. Basiflagellomere diameter about 1.4x as large as that of pedicel. **Thorax**: Anterolateral angle with inconspicuous, rounded projection; medial longitudinal sulcus shallow near collar, deepening posteriorly. Posterior pronotal lobe with finely rugulose surface; humeral angle armed, with spinous process, raised to level of and nearly continuous with disc. Scutellum moderately long; apex slightly produced into short fingerlike process. *Legs*: Slender. Femoral diameters subequal. *Hemelytron*: Surpassing apex of abdomen by about length of abdominal segment seven; quadrate cell small; Cu and M of cubital cell subparallel. **GENITALIA**: (Fig. 72) *Pygophore*: Elongate ovoid. Medial process triangular, broad, moderately long, arising from extended posterior margin, erect, straight, apex slightly curving, blunt, without modification. *Paramere*: Cylindrical; moderately long, slightly exceeding medial process; slightly curved ventrad; apical part enlarged, compressed. *Phallus*: Dorsal phallothecal sclerite somewhat squarish; medially slightly constricted; Dorsal phallothecal sclerite flat; apex truncate, medially emarginate; apical portion with transverse furrows; posterior margin of foramen broadly inversely V-shaped. Struts attached to dorsal phallothecal sclerite; apically separate, connected by bridge; fused basally. Basal plate arm slender to moderate; separate; converging; in lateral view nearly straight, only very slightly curved; bridge short; extension of basal plate small and confined to apex of basal plate arm.

***Female***: (Fig. 71c, d) Similar to male, except for the following. Larger than male, total length 15.12–19.36 mm (mean 17.00 mm, Suppl. material 2). Coloration rather similar to that in male; more uniform on head. Lateral process on humeral angle spinous, usually longer. Pro- and mesofemoral diameters larger than that of metafemur. Basiflagellomere not swollen basally. Hemelytron slightly surpassing apex of abdomen.

#### Diagnosis

As with some species of the *Zelus luridus* species group, *Z. exsanguis* has a rather uniform greenish-brown coloration. Can be distinguished from most other species of the same species group by the humeral angle elevated to same level of and nearly continuous with disc. This is also seen in *Z. ambulans*, but the two species can be easily separated on the basis of coloration (Figs 22, 71). Males can be distinguished from species of the *Zelus luridus* species group by the greatly enlarged apical part of the paramere (Fig. 72a, b), the medial process moderately broad, and the apex of the medial process somewhat narrowed.

#### Distribution

**Figure 73.**
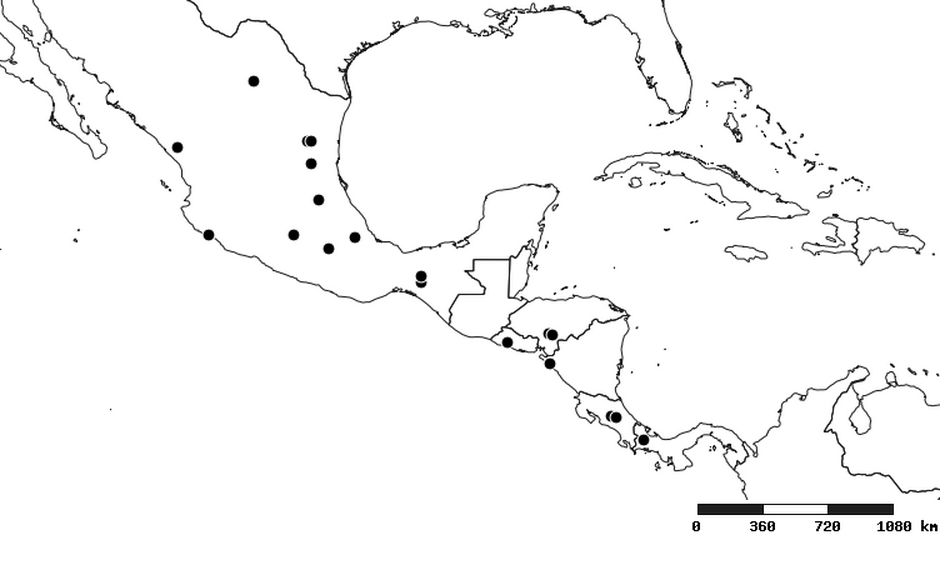
*Zelus exsanguis* Stål 1862, specimen record map

Mexico to Panama (Fig. 73). Countries with records: Costa Rica, El Salvador, Honduras, Mexico, Nicaragua and Panama.

#### Taxon discussion

Hart (1986) discussed the confusion over the use of the name *Z. exsanguis*. As this species appears highly similar to several other species, incorrect identification is common in museum specimens. Almost without exceptions specimens from the US identified as *Z. exsanguis* are actually *Z. luridus*. Champion (1898) incorrectly synonymized *Z. luridus* and *Z. ambulans* with *Z. exsanguis*. *Zelus exsanguis* also appears to prefer mountainous areas, all observed specimens being from moderate to high altitudes.

### Zelus fasciatus

Champion, 1899

Zelus fasciatus Champion, 1899, p. 257, Tab. XV. fig. 18, orig. descr. and fig.; Kuhlgatz, 1902, p. 266, note; Wygodzinsky, 1949a, p. 49, checklist; Maldonado, 1990, p. 327, cat.

#### Materials

- **Type status:** Holotype. **Occurrence:** catalogNumber: UCR_ENT 00048758; occurrenceRemarks: Verbatim label info: Type / B.C.A.Rhyn.II. *Zelus fasciatus* Ch. / Sp. figured. / Bugaba, 800-1,500 ft. Champion. / Holotype Sel. E.R. Hart letter II.iii.76; recordedBy: G.C. Champion; sex: Adult Female; **Taxon:** scientificName: Zelus fasciatus; family: Reduviidae; genus: Zelus; scientificNameAuthorship: Champion, 1899; **Location:** country: PANAMA; stateProvince: Chiriqui; locality: Bugaba; verbatimElevation: 457 m; decimalLatitude: 8.4833; decimalLongitude: -82.6167; georeferenceSources: Gazetteer; **Event:** eventDate: No date provided; **Record Level:** institutionCode: BMNH

#### Description

Figs 74, 75

***Male***: unknown.

***Female***: (Fig. 74) Medium-sized, total length 14.92–15.56 mm (mean 15.25 mm, Suppl. material 2); slender. **COLORATION**: Yellow and black. Yellow areas usually on posterior pronotal lobe, corium, lateral surfaces and abdomen. **VESTITURE**: Sparsely setose. Anteocular lobe with short, erect and recumbent setae; postocular lobe with short, erect and recumbent setae. Anterior pronotal lobe with short, inconspicuous, recumbent setae dorsally, some short to moderate, erect setae laterally; posterior pronotal lobe with short, inconspicuous, erect and recumbent setae. Abdomen with short to moderately long, erect setae and inconspicuous, short, recumbent setae. **STRUCTURE: Head**: Cylindrical, L/W = 2.46. Postocular lobe very long; in dorsal view distinctly narrowing through anterior 1/2, posterior 1/2 constant, tube-like. Eye moderately sized; lateral margin much wider than postocular lobe; dorsal and ventral margins removed from surfaces of head. *Labium*: I: II: III = 1: 2.0: 0.3. **Thorax**: Anterolateral angle rounded, without projection; medial longitudinal sulcus distinct throughout. Posterior pronotal lobe with finely rugulose surface; disc distinctly elevated above humeral angle; humeral angle armed, with spinous processes. Scutellum long; apex angulate. *Legs*: Slender. *Hemelytron*: Slightly surpassing apex of abdomen, not more than length of abdominal segment seven; quadrate cell moderately sized; Cu and M of cubital cell converging towards R.

#### Diagnosis

The rather unique dorsal color pattern easily distinguishes this species from all other species in the genus.

#### Distribution

**Figure 75.**
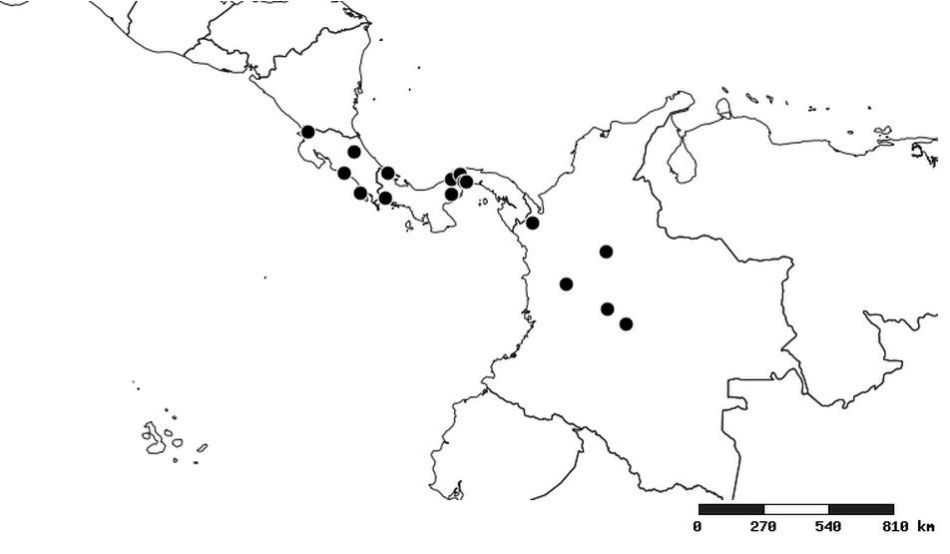
*Zelus fasciatus* Champion, 1899, specimen records

Southern Central America and northern South America (Fig. 75). Countries with specimen records: Colombia, Costa Rica and Panama.

### Zelus filicauda

Bergroth, 1893

Zelus filicauda Bergroth, 1893, p. 63, orig. descr.; Lethierry and Severin, 1896, p. 152, cat.; Wygodzinsky, 1949a, p. 49, checklist; Maldonado, 1990, p. 327, cat.

#### Materials

- **Type status:** Holotype. **Occurrence:** recordedBy: G. Fallou; sex: Adult Male; otherCatalogNumbers: 259-95; **Taxon:** scientificName: Zelus filicauda; family: Reduviidae; genus: Zelus; scientificNameAuthorship: Bergroth, 1893; **Location:** country: ECUADOR; stateProvince: Loja; locality: Loja; decimalLatitude: -4.003057; decimalLongitude: -79.207349; georeferenceSources: Google Earth; **Identification:** identifiedBy: ER Hart; dateIdentified: 1972; **Record Level:** institutionCode: Muséum national d'histoire naturelle
- **Type status:** Other material. **Occurrence:** catalogNumber: UCR_ENT 00019043; recordedBy: E. I. Schlinger & E. S. Ross; sex: Adult Male; **Taxon:** scientificName: Zelus filicauda; family: Reduviidae; genus: Zelus; scientificNameAuthorship: Bergroth, 1893; **Location:** country: ECUADOR; stateProvince: Tungurahua; locality: 13 mi W. Mera, Napo-Pastaza; decimalLatitude: -1.4628; decimalLongitude: -78.28538; georeferenceSources: Google Earth; **Identification:** identifiedBy: G. Zhang; dateIdentified: 2012; **Event:** eventDate: 1955-02-12; **Record Level:** institutionCode: CAS

#### Description

Figs 76, 77, 78

*Male*: (Fig. 76) Medium-sized, total length 12.71 mm (n=1, Suppl. material 2); slender. **COLORATION**: Nearly uniformly dark brown. Head dark brown; yellowish patch between eye and ocellus; inconspicuous, slender, medial yellow area on postocular lobe; ventral surface very slightly lighter than dorsal. Anterior pronotal lobe dark brown; posterior lobe slightly lighter than anterior lobe, somewhat reddish-brown; remainder of body surface and legs dark reddish-brown. **VESTITURE**: Sparsely setose. Head with moderately dense, short, recumbent on entire surface; dorsum also with short, spine-like setae, denser on anteocular lobe; ventral surface also with sparse, long, erect setae. Pronotum dorsal and lateral surfaces, pleura and sternites with short, recumbent setae and short to long, erect setae; recumbent setae dense on lateral surface of pronotum and pleuron; scutellum with sparse setation. Legs with sparse setation on femora and moderately dense setation on tibiae. Corium and clavus with sparse, short, recumbent setae. Abdomen with moderately dense, short, recumbent setae, intermixed with sparse, short to long, erect setae. Apical half of dorsal surface of paramere with moderately dense, medium-length, semi-erect setae. **STRUCTURE: Head**: Cylindrical, L/W = 2.03. Postocular lobe long; in dorsal view distinctly narrowing through anterior 2/3, posterior 1/3 constant, tube-like. Eye prominent; lateral margin much wider than postocular lobe; dorsal margin attaining postocular transverse groove, ventral margin removed from ventral surface of head. *Labium*: I: II: III = 1: 1.7: 0.3. **Thorax**: Anterolateral angle bearing small projection; medial longitudinal sulcus evident throughout, deepening posteriorly. Posterior pronotal lobe with rugulose surface; disc distinctly elevated above humeral angle; humeral angle armed, with spinous processes. Scutellum moderately long; apex angulate, slightly projected upward. *Legs*: Slender. *Hemelytron*: Slightly surpassing apex of abdomen, not more than length of abdominal segment seven; quadrate cell small and slender; Cu and M of cubital cell subparallel. **GENITALIA**: (Fig. 77) *Pygophore*: Elongate ovoid; lightly sclerotized expansion below paramere; not expanded laterally in dorsal view. Medial process cylindrical; slender; long, longer than paramere; laterally compressed towards apex; anterior surface towards apex ridged; minute spicules on posterior surface; posteriorly directed; curved at middle; apex in posterior view acute, with small hooklike projection. *Paramere*: Cylindrical; moderately long, not reaching medial process; directed posteriad; basally slightly constricted; curved ventrad; apical part very slightly enlarged, apex rounded, somewhat truncate. *Phallus*: Dorsal phallothecal sclerite shield-shaped; expansion of lateral margin at about mid-portion pronounced; apical portion of phallothecal sclerite gradually tapering, distinctly keeled medially, laterally flat, not forming angle; apex acute; posterior margin of foramen concave. Struts attached to dorsal phallothecal sclerite; apically separate, connected by bridge; basally separate throughout. Basal plate arm moderately robust; separate; converging; in lateral view slightly curved; bridge extremely short; extension of basal plate expanded laterally onto arm, covering more than 1/2 of arm, curved.

***Female***: Unknown.

#### Diagnosis

Recognized by the entire body dark brown, the posterior pronotal lobe slightly lighter and somewhat reddish, the legs without bands; the humeral angle projected into spinous process. Distinguished among species of the *Zelus panamensis* species group by the curved medial process (Fig. 12).

#### Distribution

**Figure 78.**
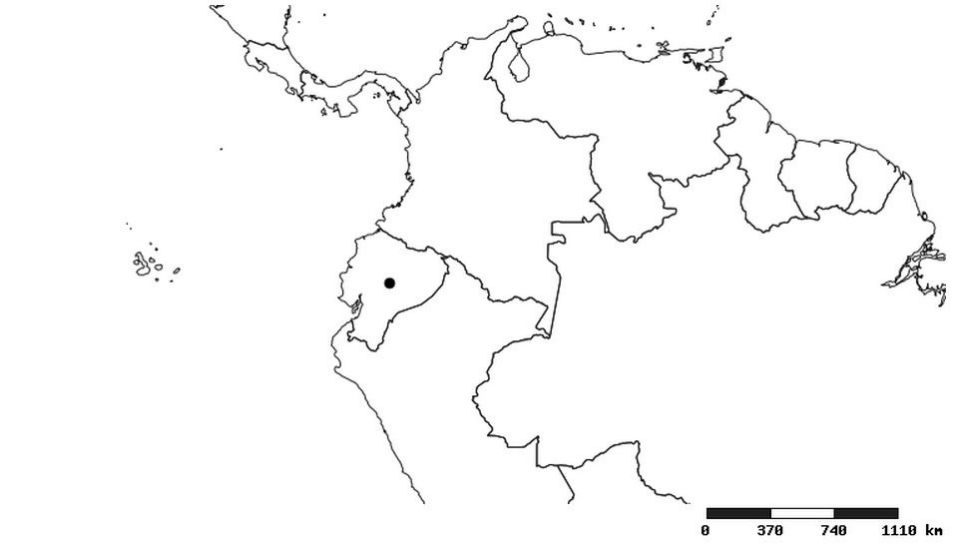
*Zelus filicauda* Bergroth, 1893, specimen record map

South America (Fig. 78). Known only from Ecuador.

### Zelus fuliginatus

Zhang & Hart sp. n.

urn:lsid:zoobank.org:act:69B9CAA6-14B8-4C04-A2A2-679A8FFF6895

#### Materials

- **Type status:** Holotype. **Occurrence:** catalogNumber: UCR_ENT 00007997; recordedBy: Richter; sex: Adult Male; **Taxon:** scientificName: Zelus fuliginatus; family: Reduviidae; genus: Zelus; scientificNameAuthorship: Zhang & Hart, 2016; **Location:** country: COLOMBIA; stateProvince: Quindio; locality: Salento; verbatimElevation: 1895 m; decimalLatitude: 4.6375; decimalLongitude: -75.57028; georeferenceSources: Gazetteer; **Identification:** identifiedBy: G. Zhang; dateIdentified: 2013; **Event:** eventDate: 1939-07-14; **Record Level:** institutionCode: USNM
- **Type status:** Paratype. **Occurrence:** catalogNumber: UCR_ENT 00009462; occurrenceRemarks: Previously designated as 'allotype' of his manuscript name *Zelus fuliginatus* by Hart, a type status not used in the formal publication of this name (Zhang et al.); recordedBy: Richter; sex: Adult Female; **Taxon:** scientificName: Zelus fuliginatus; family: Reduviidae; genus: Zelus; scientificNameAuthorship: Zhang & Hart, 2016; **Location:** country: COLOMBIA; stateProvince: Santander; locality: Rio Suarez; decimalLatitude: 6.76667; decimalLongitude: -73.26667; georeferenceSources: GeoLocate Software; **Identification:** identifiedBy: G. Zhang; dateIdentified: 2013; **Event:** eventDate: 1946-01-16; **Record Level:** institutionCode: USNM
- **Type status:** Paratype. **Occurrence:** catalogNumber: AMNH_PBI 00218883; recordedBy: J.S. Miller & E. Tapia; sex: Adult Male; **Taxon:** scientificName: Zelus fuliginatus; family: Reduviidae; genus: Zelus; scientificNameAuthorship: Zhang & Hart, 2016; **Location:** country: Ecuador; stateProvince: Napo; locality: W bank of Rio Quijos; verbatimElevation: 1750 m; decimalLatitude: 0.43333; decimalLongitude: -77.88333; georeferenceSources: Label; **Identification:** identifiedBy: G. Zhang; dateIdentified: 2013; **Event:** eventDate: 2006-03-03; **Record Level:** institutionCode: UCR

#### Description

Figs 79, 80, 81

***Male***: (Fig. 79a, b) Medium-sized, total length 12.08–13.76 mm (mean 12.92 mm, Suppl. material 2); slender. **COLORATION**: Entire surface, including antenna, labium and legs, black, except for yellowish abdominal segments 2–7 and very slender, medial longitudinal stripe on postocular lobe. **VESTITURE**: Densely setose. Anteocular with dense, short, spine-like setae, intermixed with short, recumbent, fine setae; dorsum of postocular with moderately dense, short to long, spine-like setae; ventral surface of head with sparse, long setae, varying from fine to spine-like, also with recumbent setae. Pronotum with dense, short, spine-like setae over entire surface; lateral surface of anterior pronotal lobe and pleura with both short and long, spine-like setae; scutellum with dense, short to long, spine-like setae. Legs with sparse setation. Corium and clavus with dense, recumbent, stout setae. Abdomen with moderately dense, short to long, semi-erect setae. **STRUCTURE: Head**: Cylindrical, L/W = 1.96. Postocular lobe short; in dorsal view anteriorly gradually narrowing, posterior portion constant, slightly narrower. Eye moderately sized; lateral margin only slightly wider than postocular lobe; dorsal and ventral margins removed from surfaces of head. *Labium*: I: II: III = 1: 1.5: 0.4. Basiflagellomere diameter larger than that of pedicel. **Thorax**: Anterolateral angle rounded, without projection; medial longitudinal sulcus evident throughout, deepening posteriorly. Disc distinctly elevated above humeral angle; humeral angle rounded, without projection. Scutellum long; apex angulate, not projected. *Legs*: Moderately robust. *Hemelytron*: Greatly surpassing apex of abdomen by about 3x length of abdominal segment seven; quadrate cell large and broad; Cu and M of cubital cell subparallel. **GENITALIA**: (Fig. 80) *Pygophore*: Elongate ovoid; mid-lateral fold adjacent to paramere insertion; slightly expanded laterally near base of paramere in dorsal view. Medial process somewhat cone-shaped; tapering to apex; long; posteriorly directed; basally slightly curved; apex in posterior view blunt. *Paramere*: Cylindrical, apically compressed; moderately long, nearly reaching apex of medial process; directed posteriad; basally constricted; not distinctly curved; apical part enlarged. *Phallus*: Dorsal phallothecal sclerite elongated; apical 1/3 of phallothecal sclerite tapering to apex, strong convex, laterally rounded, not forming angle; apex with small medial emargination; posterior margin of foramen broadly concave. Struts attached to dorsal phallothecal sclerite; apically separate, connected by bridge. Basal plate arm moderately robust; separate; subparallel; in lateral view very slightly curved; bridge short; extension of basal plate expanded onto arm.

***Female***: (Fig. 79c, d) Similar to male, except for the following. Larger than male, total length 17.68–18.13 mm (mean 17.91 mm, Suppl. material 2).

#### Diagnosis

Recognized by the strongly contrasting black dorsum and yellow abdomen, the rather short postocular lobe, and the Sc not reaching apex of cubital cell. Other diagnostic characters shared with members of the *Zelus vagans* species group and the *Zelus longipes* species group include the unarmed rounded humeral angle and the spine-like setae on pronotum. Males can also be separated from other species of the *Zelus vagans* species group by the medial process apically tapered, somewhat pointed (Fig. 11).

#### Etymology

The species epithet means 'soot' or painted black, referring to the black dorsal coloration of this species.

#### Distribution

**Figure 81.**
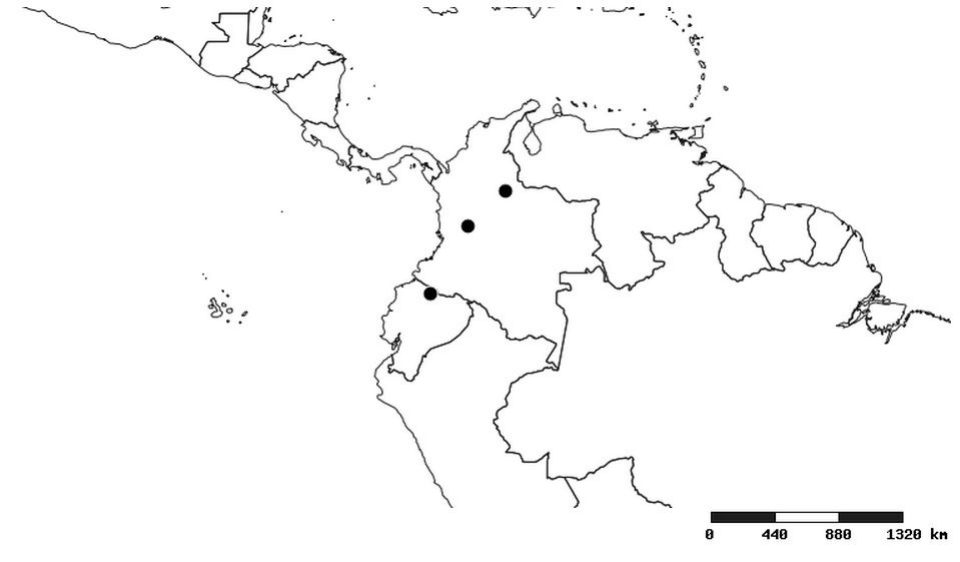
*Zelus fuliginatus* Zhang & Hart, sp. n., specimen record map

Northern South America (Fig. 81). Countries with records: Colombia and Ecuador.

### Zelus gilboventris

Zhang & Hart sp. n.

urn:lsid:zoobank.org:act:09ABAB1C-710B-441D-A9D8-B78E3B5FB3FE

#### Materials

- **Type status:** Holotype. **Occurrence:** catalogNumber: UCR_ENT 00016769; recordedBy: Wygodzinsky and Monros; sex: Adult Male; **Taxon:** scientificName: Zelus gilboventris; family: Reduviidae; genus: Zelus; scientificNameAuthorship: Zhang & Hart, 2016; **Location:** country: BOLIVIA; stateProvince: Cochabamba; locality: Villa Tunari, Chapare; verbatimElevation: 500 m; decimalLatitude: -16.91666; decimalLongitude: -65.36667; **Identification:** identifiedBy: G. Zhang; dateIdentified: 2013; **Event:** eventDate: 1958-01-09; **Record Level:** institutionCode: AMNH
- **Type status:** Paratype. **Occurrence:** catalogNumber: UCR_ENT 00038447; recordedBy: E.A. Bergey and K.R. Hobson; sex: Adult Male; **Taxon:** scientificName: Zelus gilboventris; family: Reduviidae; genus: Zelus; scientificNameAuthorship: Zhang & Hart, 2016; **Location:** country: ECUADOR; stateProvince: Napo; locality: Jatun Sacha Biological Station, 20 km E of Puerto Napo; verbatimElevation: 450 m; decimalLatitude: -1.0644; decimalLongitude: -77.613; georeferenceSources: Google Earth; **Identification:** identifiedBy: G. Zhang; dateIdentified: 2013; **Event:** eventDate: 1989-01-14; **Record Level:** institutionCode: UCB
- **Type status:** Paratype. **Occurrence:** catalogNumber: UCR_ENT 00006074; recordedBy: E. I. Schlinger & E. S. Ross; sex: Adult Female; **Taxon:** scientificName: Zelus gilboventris; family: Reduviidae; genus: Zelus; scientificNameAuthorship: Zhang & Hart, 2016; **Location:** country: PERU; stateProvince: Huanuco; county: Leoncio Prado; locality: Monzon Valley, Tingo Maria; verbatimElevation: 648 m; decimalLatitude: -9.29528; decimalLongitude: -75.99754; georeferenceSources: Google Earth; **Identification:** identifiedBy: G. Zhang; dateIdentified: 2013; **Event:** eventDate: 1954-10-15; **Record Level:** institutionCode: CAS
- **Type status:** Paratype. **Occurrence:** catalogNumber: UCR_ENT 00016997; recordedBy: J. C. Pallister; sex: Adult Male; **Taxon:** scientificName: Zelus gilboventris; family: Reduviidae; genus: Zelus; scientificNameAuthorship: Zhang & Hart, 2016; **Location:** country: PERU; stateProvince: Huanuco; county: Leoncio Prado; locality: Tingo Maria; verbatimElevation: 671 m; decimalLatitude: -9.3; decimalLongitude: -75.9833; **Identification:** identifiedBy: G. Zhang; dateIdentified: 2013; **Event:** eventDate: 1946-11-02; **Record Level:** institutionCode: AMNH
- **Type status:** Paratype. **Occurrence:** catalogNumber: UCR_ENT 00016998; recordedBy: H. Bassler; sex: Adult Female; **Taxon:** scientificName: Zelus gilboventris; family: Reduviidae; genus: Zelus; scientificNameAuthorship: Zhang & Hart, 2016; **Location:** country: PERU; stateProvince: Huanuco; locality: Upper Rio Huallaga; decimalLatitude: -9.2975; decimalLongitude: -76.005; georeferenceSources: Google Earth; **Identification:** identifiedBy: G. Zhang; dateIdentified: 2013; **Event:** eventDate: 1928-01-02; **Record Level:** institutionCode: AMNH
- **Type status:** Paratype. **Occurrence:** catalogNumber: UCR_ENT 00016999; recordedBy: J. C. Pallister; sex: Adult Female; **Taxon:** scientificName: Zelus gilboventris; family: Reduviidae; genus: Zelus; scientificNameAuthorship: Zhang & Hart, 2016; **Location:** country: PERU; stateProvince: Huanuco; county: Leoncio Prado; locality: Tingo Maria; verbatimElevation: 671 m; decimalLatitude: -9.3; decimalLongitude: -75.9833; **Identification:** identifiedBy: G. Zhang; dateIdentified: 2013; **Event:** eventDate: 1905-04-29; **Record Level:** institutionCode: AMNH
- **Type status:** Paratype. **Occurrence:** catalogNumber: UCR_ENT 00019700; recordedBy: E. I. Schlinger & E. S. Ross; sex: Adult Female; **Taxon:** scientificName: Zelus gilboventris; family: Reduviidae; genus: Zelus; scientificNameAuthorship: Zhang & Hart, 2016; **Location:** country: PERU; stateProvince: Huanuco; locality: Monzon valley, Tingo Maria; decimalLatitude: -9.27816; decimalLongitude: -76.05562; georeferenceSources: Google Earth; **Identification:** identifiedBy: G. Zhang; dateIdentified: 2013; **Event:** eventDate: 1954-09-23; **Record Level:** institutionCode: CAS
- **Type status:** Paratype. **Occurrence:** catalogNumber: UCR_ENT 00019701; recordedBy: E. I. Schlinger & E. S. Ross; sex: Adult Female; **Taxon:** scientificName: Zelus gilboventris; family: Reduviidae; genus: Zelus; scientificNameAuthorship: Zhang & Hart, 2016; **Location:** country: PERU; stateProvince: Huanuco; locality: Monzon valley, Tingo Maria; decimalLatitude: -9.27816; decimalLongitude: -76.05562; georeferenceSources: Google Earth; **Identification:** identifiedBy: G. Zhang; dateIdentified: 2013; **Event:** eventDate: 1954-11-02; **Record Level:** institutionCode: CAS
- **Type status:** Paratype. **Occurrence:** catalogNumber: UCR_ENT 00019702; recordedBy: E. I. Schlinger & E. S. Ross; sex: Adult Female; **Taxon:** scientificName: Zelus gilboventris; family: Reduviidae; genus: Zelus; scientificNameAuthorship: Zhang & Hart, 2016; **Location:** country: PERU; stateProvince: Huanuco; locality: Monzon valley, Tingo Maria; decimalLatitude: -9.27816; decimalLongitude: -76.05562; georeferenceSources: Google Earth; **Identification:** identifiedBy: G. Zhang; dateIdentified: 2013; **Event:** eventDate: 1954-11-21; **Record Level:** institutionCode: CAS
- **Type status:** Paratype. **Occurrence:** catalogNumber: UCR_ENT 00029356; occurrenceRemarks: Genitalia dissected. Paratype designated by Hart, unpublished; recordedBy: D. A. Young; sex: Adult Male; **Taxon:** scientificName: Zelus gilboventris; family: Reduviidae; genus: Zelus; scientificNameAuthorship: Zhang & Hart, 2016; **Location:** country: PERU; stateProvince: Huanuco; locality: Tingo Maria (Town of); verbatimElevation: 671 m; decimalLatitude: -9.3; decimalLongitude: -75.9833; georeferenceSources: GeoLocate Software; **Identification:** identifiedBy: G. Zhang; dateIdentified: 2013; **Event:** eventDate: 1960-08-10; **Record Level:** institutionCode: USNM
- **Type status:** Paratype. **Occurrence:** catalogNumber: UCR_ENT 00057801; occurrenceRemarks: Previously designated as 'allotype', a concept not used in the formal publication of this name (Zhang et al.); recordedBy: J. C. Pallister; sex: Adult Female; **Taxon:** scientificName: Zelus gilboventris; family: Reduviidae; genus: Zelus; scientificNameAuthorship: Zhang & Hart, 2016; **Location:** country: PERU; stateProvince: Huanuco; county: Leoncio Prado; locality: Tingo Maria; verbatimElevation: 671 m; decimalLatitude: -9.3; decimalLongitude: -75.9833; **Identification:** identifiedBy: G. Zhang; dateIdentified: 2013; **Event:** eventDate: 1940-11-23; **Record Level:** institutionCode: AMNH
- **Type status:** Paratype. **Occurrence:** catalogNumber: UCR_ENT 00029357; occurrenceRemarks: Drake collection; recordedBy: P. Papraychi; sex: Adult Male; **Taxon:** scientificName: Zelus gilboventris; family: Reduviidae; genus: Zelus; scientificNameAuthorship: Zhang & Hart, 2016; **Location:** country: PERU; stateProvince: Junin; locality: Satipo; decimalLatitude: -11.2667; decimalLongitude: -74.6833; georeferenceSources: Gazetteer; **Identification:** identifiedBy: G. Zhang; dateIdentified: 2013; **Event:** eventDate: 1940-07-01; **Record Level:** institutionCode: USNM
- **Type status:** Paratype. **Occurrence:** catalogNumber: UCR_ENT 00023700; recordedBy: Unknown; sex: Adult Male; **Taxon:** scientificName: Zelus gilboventris; family: Reduviidae; genus: Zelus; scientificNameAuthorship: Zhang & Hart, 2016; **Location:** country: PERU; stateProvince: unknown; locality: Pachitea; decimalLatitude: -9.41667; decimalLongitude: -75.16667; georeferenceSources: Gazetteer; **Identification:** identifiedBy: G. Zhang; dateIdentified: 2013; **Event:** eventDate: no date provided; **Record Level:** institutionCode: RMNH
- **Type status:** Paratype. **Occurrence:** catalogNumber: UCR_ENT 00023702; recordedBy: Unknown; sex: Adult Male; **Taxon:** scientificName: Zelus gilboventris; family: Reduviidae; genus: Zelus; scientificNameAuthorship: Zhang & Hart, 2016; **Location:** country: PERU; stateProvince: unknown; locality: Pachitea; decimalLatitude: -9.41667; decimalLongitude: -75.16667; georeferenceSources: Gazetteer; **Identification:** identifiedBy: G. Zhang; dateIdentified: 2013; **Event:** eventDate: no date provided; **Record Level:** institutionCode: RMNH

#### Description

Figs 82, 83, 84

***Male***: (Fig. 82a, b) Medium-sized, total length 12.07–13.69 mm (mean 13.19 mm, Suppl. material 2), slender. **COLORATION**: Brown. Head brown to dark brown; pale brown patch between eye and ocellus; medial stripe on postocular lobe. Pronotum either entirely dark brown or posterior pronotal lobe and scutellum orangish. Legs dark brown, with inconspicuous lighter-colored bands. Abdomen yellowish. **VESTITURE**: Sparsely setose. Head short, recumbent setae on entire surface; very short, erect, spine-like setae on dorsum, denser on anterior lobe; few moderately long, erect, fine setae on ventral surface. Pronotum with sparse, recumbent setae and short, erect setae over dorsal surface; denser, recumbent setae on lateral surface and pleura, intermixed with short, erect setae; scutellum with erect and recumbent setae. Legs with sparse setation on femora and moderately dense setation on tibiae. Corium and clavus with short, recumbent or erect setae. Abdomen with moderately dense, short recumbent setae, intermixed with sparse, short to long, erect setae. **STRUCTURE: Head**: Cylindrical, L/W = 2.48. Postocular lobe long; in dorsal view distinctly narrowing through anterior 2/3, posterior 1/3 constant, tube-like. Eye moderately sized; lateral margin much wider than postocular lobe; dorsal margin attaining postocular transverse groove, ventral margin removed from ventral surface of head. *Labium*: I: II: III = 1: 1.6: 0.4. Basiflagellomere diameter larger than that of pedicel. **Thorax**: Anterolateral angle bearing small, somewhat acute projection; medial longitudinal sulcus evident throughout, deepening posteriorly. Posterior pronotal lobe with rugulose surface; disc distinctly elevated above humeral angle; humeral angle armed, with dentate projection. Scutellum moderately long; apex angulate. *Legs*: Slender. *Hemelytron*: Slightly surpassing apex of abdomen, not more than length of abdominal segment seven; quadrate cell small, elongate; Cu and M of cubital cell subparallel. **GENITALIA**: (Fig. 83) *Pygophore*: Elongate ovoid; lightly sclerotized expansion below paramere; not expanded laterally in dorsal view. Medial process cylindrical; slender; long, much longer than paramere; laterally compressed towards apex; semi-erect; nearly straight, curvature slight; apex in posterior view acute, with small hooklike projection. *Paramere*: Cylindrical; moderately long, reaching about mid-point of medial process; directed posteriad; not distinctly curved; apical part not enlarged. *Phallus*: Dorsal phallothecal sclerite shield-shaped; sclerotization reduced (yet not absent) on dorsal surface close to posterior margin of foramen; apical portion of phallothecal sclerite gradually tapering, distinctly keeled medially, laterally flat, not forming angle; apex rounded; posterior margin of foramen nearly straight. Struts not attached to dorsal phallothecal sclerite; apically not evident or missing; basally separate throughout. Basal plate arm robust; basally fused; in lateral view slightly curved; bridge extremely short; extension of basal plate well expanded laterally onto arm, covering more than 1/2 of arm, curved.

***Female***: (Fig. 82c, d) Different from male as outlined below. Larger than male, total length 14.52–17.41 mm (mean 16.23 mm, Suppl. material 2). Dorsal surface, including hemelytron, lighter colored, nearly uniformly pale brown; quadrate cell and proximal margin of postcubital cell yellowish; lateral and ventral surfaces yellowish; legs without or with inconspicuous bands. Basiflagellomere subequal in diameter to pedicel. Process on humeral angle spinous, long.

#### Diagnosis

The posterior pronotal lobe usually orangish-brown; the medial process rather long, much longer than paramere; and the anterior side of medial process keeled medially at apex. In females the dorsal surface is nearly uniformly brown, the lateral and ventral surfaces yellowish, and the quadrate cell and proximal margin of postcubital cell yellowish.

#### Distribution

**Figure 84.**
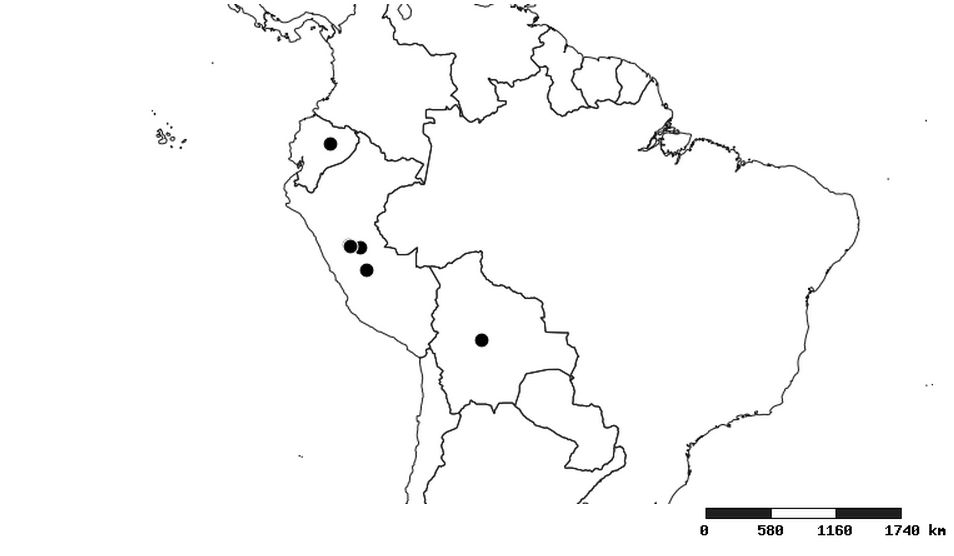
*Zelus gilboventris* Zhang & Hart, sp. n., specimen record map

South America (Fig. 84). Countries with records: Bolivia, Ecuador and Peru

### Zelus gracilipes

Zhang & Hart sp. n.

urn:lsid:zoobank.org:act:82638B0D-5B20-4B74-AFBD-49A55863EB63

#### Materials

- **Type status:** Holotype. **Occurrence:** catalogNumber: UCR_ENT 00007998; occurrenceRemarks: Genitalia dissected.; recordedBy: Mulford Bio. Expl; sex: Adult Male; **Taxon:** scientificName: Zelus gracilipes; family: Reduviidae; genus: Zelus; scientificNameAuthorship: Zhang & Hart, 2016; **Location:** country: BOLIVIA; stateProvince: La Paz; locality: Tumupasa; verbatimElevation: 457 m; decimalLatitude: -14.1487; decimalLongitude: -67.8876; georeferenceSources: Google Earth; **Identification:** identifiedBy: G. Zhang; dateIdentified: 2013; **Event:** eventDate: Dec 1921-22; **Record Level:** institutionCode: USNM
- **Type status:** Paratype. **Occurrence:** catalogNumber: UCR_ENT 00009463; recordedBy: Zischka; sex: Adult Female; **Taxon:** scientificName: Zelus gracilipes; family: Reduviidae; genus: Zelus; scientificNameAuthorship: Zhang & Hart, 2016; **Location:** country: BOLIVIA; stateProvince: Cochabamba; locality: Chapare; decimalLatitude: -16.711; decimalLongitude: -65.663; georeferenceSources: Google Earth; **Identification:** identifiedBy: G. Zhang; dateIdentified: 2013; **Event:** eventDate: no date provided; **Record Level:** institutionCode: USNM
- **Type status:** Paratype. **Occurrence:** catalogNumber: UCR_ENT 00071194; recordedBy: Zischka; sex: Adult Female; **Taxon:** scientificName: Zelus gracilipes; family: Reduviidae; genus: Zelus; scientificNameAuthorship: Zhang & Hart, 2016; **Location:** country: BOLIVIA; stateProvince: Cochabamba; locality: Chapare; decimalLatitude: -16.711; decimalLongitude: -65.663; georeferenceSources: Google Earth; **Identification:** identifiedBy: G. Zhang; dateIdentified: 2013; **Event:** eventDate: no date provided; **Record Level:** institutionCode: TAMU
- **Type status:** Paratype. **Occurrence:** catalogNumber: UCR_ENT 00009296; occurrenceRemarks: Additional Labels Mulford BioExpl 1921-1922; recordedBy: W. M. Mann; sex: Adult Male; **Taxon:** scientificName: Zelus gracilipes; family: Reduviidae; genus: Zelus; scientificNameAuthorship: Zhang & Hart, 2016; **Location:** country: BOLIVIA; stateProvince: none; locality: Rio Negro; **Identification:** identifiedBy: G. Zhang; dateIdentified: 2013; **Event:** eventDate: Jan; **Record Level:** institutionCode: USNM
- **Type status:** Paratype. **Occurrence:** catalogNumber: UCR_ENT 00009297; recordedBy: T. Borgmeier; sex: Adult Female; **Taxon:** scientificName: Zelus gracilipes; family: Reduviidae; genus: Zelus; scientificNameAuthorship: Zhang & Hart, 2016; **Location:** country: BRAZIL; stateProvince: Goias; locality: Campinas; decimalLatitude: -16.66785; decimalLongitude: -49.29149; georeferenceSources: Google Earth; **Identification:** identifiedBy: G. Zhang; dateIdentified: 2013; **Event:** eventDate: 1905-04-17; **Record Level:** institutionCode: USNM
- **Type status:** Paratype. **Occurrence:** catalogNumber: UCR_ENT 00009450; recordedBy: J. E. Eger; sex: Adult Female; **Taxon:** scientificName: Zelus gracilipes; family: Reduviidae; genus: Zelus; scientificNameAuthorship: Zhang & Hart, 2016; **Location:** country: BRAZIL; stateProvince: Rondonia; locality: 62 km S Ariquemes, Fazenda Rancho Grande; verbatimElevation: 300 m; decimalLatitude: -10.3; decimalLongitude: -62.86666; **Identification:** identifiedBy: G. Zhang; dateIdentified: 2013; **Event:** eventDate: 1996-07-03 to 1996-07-15; **Record Level:** institutionCode: USNM
- **Type status:** Paratype. **Occurrence:** catalogNumber: UCR_ENT 00009499; occurrenceRemarks: Drake Collection; recordedBy: J. E. Eger; sex: Adult Male; **Taxon:** scientificName: Zelus gracilipes; family: Reduviidae; genus: Zelus; scientificNameAuthorship: Zhang & Hart, 2016; **Location:** country: BRAZIL; stateProvince: Rondonia; locality: 62 km S Ariquemes, Fazenda Rancho Grande; verbatimElevation: 300 m; decimalLatitude: -10.3; decimalLongitude: -62.86666; **Identification:** identifiedBy: G. Zhang; dateIdentified: 2013; **Event:** eventDate: 1997-11-04 to 1997-11-16; **Record Level:** institutionCode: USNM
- **Type status:** Paratype. **Occurrence:** catalogNumber: UCR_ENT 00026166; occurrenceRemarks: Drake Collection; recordedBy: J. E. Eger; sex: Adult Male; **Taxon:** scientificName: Zelus gracilipes; family: Reduviidae; genus: Zelus; scientificNameAuthorship: Zhang & Hart, 2016; **Location:** country: BRAZIL; stateProvince: Rondonia; locality: 62 km SW of Ariquemes, near Fzda. Rancho Grande; decimalLatitude: -10.32921; decimalLongitude: -63.46881; **Identification:** identifiedBy: G. Zhang; dateIdentified: 2013; **Event:** eventDate: 1997-11-04 to 1997-11-16; **Record Level:** institutionCode: USNM
- **Type status:** Paratype. **Occurrence:** catalogNumber: UCR_ENT 00026167; occurrenceRemarks: Drake Collection; recordedBy: J. E. Eger; sex: Adult Male; **Taxon:** scientificName: Zelus gracilipes; family: Reduviidae; genus: Zelus; scientificNameAuthorship: Zhang & Hart, 2016; **Location:** country: BRAZIL; stateProvince: Rondonia; locality: 62 km SW of Ariquemes, near Fzda. Rancho Grande; decimalLatitude: -10.32921; decimalLongitude: -63.46881; **Identification:** identifiedBy: G. Zhang; dateIdentified: 2013; **Event:** eventDate: 1997-11-04 to 1997-11-16; **Record Level:** institutionCode: USNM
- **Type status:** Paratype. **Occurrence:** catalogNumber: UCR_ENT 00009449; recordedBy: J. E. Eger; sex: Adult Male; **Taxon:** scientificName: Zelus gracilipes; family: Reduviidae; genus: Zelus; scientificNameAuthorship: Zhang & Hart, 2016; **Location:** country: ECUADOR; stateProvince: Napo; locality: Puerto Misahuali; verbatimElevation: 541 m; decimalLatitude: -1.0345; decimalLongitude: -77.66366; georeferenceSources: Label; **Identification:** identifiedBy: G. Zhang; dateIdentified: 2013; **Event:** eventDate: 1998-09-06 to 1998-09-19; **Record Level:** institutionCode: USNM
- **Type status:** Paratype. **Occurrence:** catalogNumber: UCR_ENT 00009492; occurrenceRemarks: Drake Collection; recordedBy: J. E. Eger; sex: Adult Male; **Taxon:** scientificName: Zelus gracilipes; family: Reduviidae; genus: Zelus; scientificNameAuthorship: Zhang & Hart, 2016; **Location:** country: ECUADOR; stateProvince: Napo; locality: Puerto Misahuali; verbatimElevation: 541 m; decimalLatitude: -1.0345; decimalLongitude: -77.66366; georeferenceSources: Label; **Identification:** identifiedBy: G. Zhang; dateIdentified: 2013; **Event:** eventDate: 1998-09-06 to 1998-09-19; **Record Level:** institutionCode: USNM
- **Type status:** Paratype. **Occurrence:** catalogNumber: UCR_ENT 00047971; recordedBy: E. S. Ross; sex: Adult Male; **Taxon:** scientificName: Zelus gracilipes; family: Reduviidae; genus: Zelus; scientificNameAuthorship: Zhang & Hart, 2016; **Location:** country: ECUADOR; stateProvince: Napo; locality: Alinahui, 20 km E of Puerto Napo; verbatimElevation: 450 m; decimalLatitude: -1; decimalLongitude: -77.41666; **Identification:** identifiedBy: G. Zhang; dateIdentified: 2013; **Event:** eventDate: 1992-12-01; **Record Level:** institutionCode: CAS
- **Type status:** Paratype. **Occurrence:** catalogNumber: UCR_ENT 00006075; recordedBy: E. I. Schlinger & E. S. Ross; sex: Adult Female; **Taxon:** scientificName: Zelus gracilipes; family: Reduviidae; genus: Zelus; scientificNameAuthorship: Zhang & Hart, 2016; **Location:** country: PERU; stateProvince: Huanuco; locality: Monzon valley, Tingo Maria; decimalLatitude: -9.27816; decimalLongitude: -76.05562; georeferenceSources: Google Earth; **Identification:** identifiedBy: G. Zhang; dateIdentified: 2013; **Event:** eventDate: 1954-09-23; **Record Level:** institutionCode: CAS
- **Type status:** Paratype. **Occurrence:** catalogNumber: UCR_ENT 00044265; occurrenceRemarks: Primary DNA voucher RCW_2474; recordedBy: C. Weirauch; sex: Adult Female; **Taxon:** scientificName: Zelus gracilipes; family: Reduviidae; genus: Zelus; scientificNameAuthorship: Zhang & Hart, 2016; **Location:** country: PERU; stateProvince: Madre de Dios; locality: Los Amigos Biol.Sta. trail 14; verbatimElevation: 231 m; decimalLatitude: -12.57141; decimalLongitude: -70.09538; georeferenceSources: GPS; **Identification:** identifiedBy: G. Zhang; dateIdentified: 2013; **Event:** samplingProtocol: General Collecting; eventDate: 2010-12-20; **Record Level:** institutionCode: UCR

#### Description

Figs 85, 86, 87

***Male***: (Fig. 85a, b) Large, total length 15.43–16.74 mm (mean 16.09 mm, Suppl. material 2); slender. **COLORATION**: Hed, anterior pronotal lobe, hemelytron, and legs dark brown to brownish-black; very inconspicuous, light-colored, rather thin, medial longitudinal stripe on postocular lobe. Ventral surface of head yellowish in some specimens. Ventral surface of abdomen in some specimens reddish-brown. Posterior pronotal lobe and mesopleuron orange or reddish-brown. Setae on corium golden. Abdominal segments 2–5 reddish-brown in some specimens. Variations minimal between specimens. **VESTITURE**: Densely setose. Dorsum of anteocular and anterior part of postocular with moderately dense, short, erect, spine-like setae; rest with sparse, short, erect or recumbent setae; posterior part of postocular nearly glabrous. Pronotum with dense, short, erect, spine-like setae on dorsal and lateral surfaces. Pleura with mixed spine-like and fine setae; scutellum with dense, short to long, semi-erect to recument setae. Legs with sparse setae; sundew setae on profemur sparse and randomly arranged. Corium and clavus with dense, recumbent, stout setae. Abdomen with moderately dense, short, semi-erect, fine setae. Apically with moderately long, erect setae. **STRUCTURE: Head**: Cylindrical, L/W = 2.28. Postocular lobe in dorsal view anteriorly gradually narrowing, posterior portion constant, slightly narrower. Eye prominent; lateral margin much wider than postocular lobe; dorsal margin removed from postocular transverse groove, ventral margin attaining ventral surface of head. *Labium*: I: II: III = 1: 1.5: 0.4. Basiflagellomere diameter slightly larger than that of pedicel. **Thorax**: Anterolateral angle rounded, without projection; medial longitudinal sulcus evident throughout, deepening posteriorly. Disc distinctly elevated above humeral angle; humeral angle rounded, without projection. Scutellum moderately long; apex angulate, not projected. *Legs*: Very slender. *Hemelytron*: Greatly surpassing apex of abdomen by about 3x length of abdominal segment seven; quadrate cell large and broad; Cu and M of cubital cell subparallel. **GENITALIA**: (Fig. 86) *Pygophore*: Ovoid; mid-lateral fold adjacent to paramere insertion; slightly expanded laterally near base of paramere in dorsal view. Medial process somewhat cone-shaped; moderately long; posteriorly directed, in less than forty-five degree with body axis; nearly straight; apex in posterior view blunt. *Paramere*: Cylindrical; long, nearly reaching apex of medial process; directed toward medial process; nearly straight; apex oblique; apical part not enlarged. *Phallus*: Dorsal phallothecal sclerite elongated; apical 1/3 of phallothecal sclerite tapering to apex, strongly convex, laterally rounded, not forming angle; apex with small medial emargination; basal part expanded laterally; posterior margin of foramen broadly concave. Struts attached to dorsal phallothecal sclerite; apically separate, connected by bridge; basally fused in part. Basal plate arm slender; separate; subparallel; in lateral view very slightly curved; bridge moderately long; extension of basal plate small and confined to apex of basal plate arm.

***Female***: (Fig. 85c, d) Similar to male, except for the following. Larger than male, total length 19.62–20.74 mm (mean 20.06 mm, Suppl. material 2). Dorsal coloration variable, ranging from nearly entirely orange to predominantly dark brown.

#### Diagnosis

Recognized by the posterior pronotal lobe orangish-brown; the legs uniformly blackish-brown, without bands; the humeral angle rounded; and the head and pronotum with spine-like setae (last two characters shared with species of the *Zelus vagans* species group and the *Zelus longipes* species group). Males can also be recognized by the medial process posteriorly directed and apical portion laterally compressed (diagnostic of *Zelus vagans* species group). The medial process is comparatively shorter than those in other species of the *Zelus vagans* species group (Fig. 11). *Zelus gracilipes* may be confused with *Z. vespiformis* and *Z. errans*, which also tend to show a combination of black and orange colors. The males of *Z. gracilipes* can be separated on the basis of the male genitalia. The females of *Z. gracilipes* have the entire membrane colored or opaque, whereas the known females of *Z. errans* have the anterior 1/2 clear or semi-translucent. Some females of *Z. gracilipes* exhibit entirely orange-brown hemelytron, a character also found in some specimens of *Z. vespiformis*. As the ranges the these two do not appear to overlap, locality data may help in most cases separate the species. Additionally, *Z. gracilipes* is generally more slender than *Z. vespiformis*.

#### Etymology

From Latin *gracilis*, meaning slender.

#### Distribution

**Figure 87.**
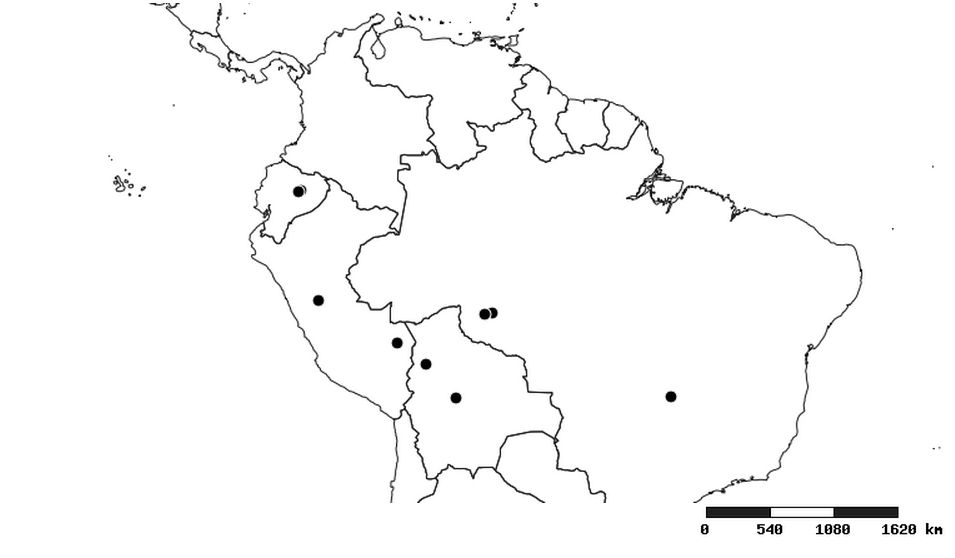
*Zelus gracilipes* Zhang & Hart, sp. n., specimen record map

South America (Fig. 87). Countries with records: Bolivia, Brazil, Ecuador and Peru.

### Zelus grandoculus

Zhang & Hart sp. n.

urn:lsid:zoobank.org:act:D48257E1-BF91-4DD1-A900-9A2A929FE91A

#### Materials

- **Type status:** Holotype. **Occurrence:** catalogNumber: UCR_ENT 00007999; occurrenceRemarks: Genitalia dissected; recordedBy: B. Lott; sex: Adult Male; **Taxon:** scientificName: Zelus grandoculus; family: Reduviidae; genus: Zelus; scientificNameAuthorship: Zhang & Hart, 2016; **Location:** country: GUATEMALA; stateProvince: Sacatepequez; locality: Antigua; verbatimElevation: 1583 m; decimalLatitude: 14.5611; decimalLongitude: -90.7344; georeferenceSources: Gazetteer; **Identification:** identifiedBy: G. Zhang; dateIdentified: 2013; **Event:** eventDate: no date provided; **Record Level:** institutionCode: USNM

#### Description

Figs 88, 89, 90

***Male***: (Fig. 88) Medium-sized, total length 14.63 mm (n=1, Suppl. material 2); slender. **COLORATION**: Yellowish-brown and reddish-brown with some dark brown areas. Dorsal surface of head reddish-brown, postocular lobe with mid-dorsal line and circumocellar areas yellowish-brown, ventral surface of both lobes yellowish-brown. Rostrum yellowish-brown. Scape and pedicel yellowish-brown with small dark brown areas at base and apex. Flagellomeres dark reddish-brown. Anterior pronotal lobe reddish-brown dorsally; lateral surface yellowish-brown. Posterior lobe reddish-brown dorsally with margins and lateral surface yellowish-brown, humeral angle dark brown, lateral surfaces yellowish-brown. Scutellum yellowish-brown. Legs yellowish-brown with dark reddish-brown areas near apices of femora and tibiae. Hemelytron yellowish-brown to reddish-brown, costal margin of discal cell and adjacent area of corium dark brown. Abdominal dorsal surface yellowish-brown to brown, remainder of surface yellowish-brown. **VESTITURE**: Moderately setose. Head with recumbent setae on entire surface, more dense dorsally, long erect setae ventrally and ventrolaterally. Anterior pronotal lobe with scattered patches of recumbent and semi-erect setae dorsally, semi-erect and erect setae longer and more dense on lateral surface. Posterior lobe with recumbent setae over entire surface, some erect setae lateroventrally. Scutellum with erect and semi-erect setae. Corium and clavus with recumbent setae. Abdomen with short erect setae over entire surface, longer erect setae on margins of connexivum and ventrally on terminal segments. Exposed area of pygophore with erect setae. Apical half of paramere with short erect setae. **STRUCTURE: Head**: Cylindrical, L/W = 1.75. Postocular lobe moderately long; in dorsal view anteriorly gradually narrowing, posterior portion constant, slightly narrower. Eye unusually large; lateral margins much wider than postocular lobe; margins beyond dorsal and ventral outlines of head in lateral view. Ocellus elevated, large, diameter over 1.3x ocular-ocellar distance. *Labium*: I: II: III = 1: 1.9: 0.6. Basiflagellomere diameter larger than that of pedicel. **Thorax**: Anterolateral angle with inconspicuous subtuberculate projection; medial longitudinal sulcus appearing after anterior third. Posterior pronotal lobe with finely rugulose surface; disc slightly elevated above humeral angle; humeral angle armed with short spinous process. Scutellum moderately long; apex slightly produced, blunt. *Legs*: Slender. Pro- and metafemora of equal diameter, mesofemur slightly larger. *Hemelytron*: Surpassing apex of abdomen by about length of abdominal segment seven; quadrate cell small, elongate; Cu and M of cubital cell subparallel. **GENITALIA**: (Fig. 89) *Pygophore*: Elongate ovoid; not expanded laterally in dorsal view. Medial process triangular; broad; moderately long; erect; nearly straight; apex in posterior view blunt, without modification. *Paramere*: Cylindrical; moderately long, slightly exceeding medial process; slightly curved ventrad; apical part feebly enlarged. *Phallus*: Dorsal phallothecal sclerite somewhat squarish; medially slightly constricted; apical portion of phallothecal sclerite not distinctly tapered, slightly convex; apex truncate, medially emarginate; posterior margin of foramen broadly concave. Struts attached to dorsal phallothecal sclerite; apically separate, connected by bridge; basally moderately fused. Basal plate arm slender; separate; converging; in lateral view nearly straight, very slightly curved; bridge moderately long; extension of basal plate small and confined to apex of basal plate arm.

***Female***: Unknown.

#### Diagnosis

This is the only species in the genus with the margins of the eye exceeding outlines of the head both dorsally and ventrally. Compared to other species of the *Zelus luridus* species group (Fig. 3), the paramere is short, more slender and its apex very slightly expanded.

#### Etymology

The species epithet combines *grandis*, meaning large, with *oculus*, meaning eye, to indicate the prominently large-sized compound eye.

#### Distribution

**Figure 90.**
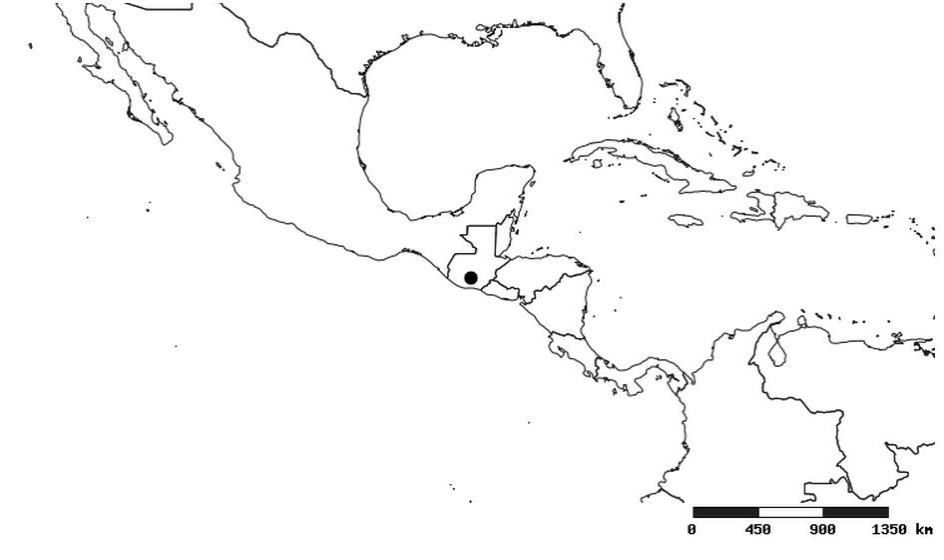
*Zelus grandoculus* Zhang & Hart, sp. n., specimen record map

Known only from type locality in Guatemala (Fig. 90).

### Zelus grassans

Stål, 1862

Zelus grassans Stål, 1862, p, 450, orig. descr.; Stål, 1872, p. 91, cat.; Lethierry and Severin, 1896, p. 152, cat.; Champion, 1898, p. 256–257, Tab. *XV*, fig. 16, 17, note and fig.; Kuhlgatz, 1902, p. 266, note; Fracker, 1913, p. 239, 240, key and list (subgenus *Diplodus*); Wygodzinsky, 1949a, p. 49, checklist; Hart, 1986, p. 546-547, redescription, note, fig. and key; Maldonado, 1990, p. 327, cat.
Diplodus grassans : Walker, 1872, p. 124, cat.; Uhler, 1886, p. 24, checklist.

#### Materials

- **Type status:** Holotype. **Occurrence:** catalogNumber: UCR_ENT 00075073; occurrenceRemarks: Verbatim label info: Mexico coll. Signoret / grassans det. Stal / B.C.A. Rhyn.II. *Zelus grassans* St. / *Zelus grassans* Stal / Holotype / Lectotypus *Zelus grassans* STAL, 1862 etik. Hecher 1996 REDV. 471/1; recordedBy: Signoret; sex: Adult Female; **Taxon:** scientificName: Zelus grassans; family: Reduviidae; genus: Zelus; scientificNameAuthorship: Stål, 1862; **Location:** country: MEXICO; **Identification:** identifiedBy: G. Zhang; dateIdentified: 2013; **Record Level:** institutionCode: NHMW

#### Description

**Figs 91, 92, 93**

***Male***: (Fig. 91a, b, c) Medium-sized, total length 12.42–15.02 mm (mean 13.76 mm, Suppl. material 2); slender. **COLORATION**: Orangish to reddish, with variable amount of black areas. Legs reddish-brown, pro-femur with broad dark bands; meso and meta-femora reddish-brown, apex dark, two narrow indistinct dark bands medially. Black, red or whitish markings on abdominal venter. **VESTITURE**: Sparsely setose. Head with short, spine-like setae dorsally, finer, short to long semi-erect and erect setae over entire surface. Anterior pronotal lobe with sparse, erect setae on entire surface; posterior lobe with short, heavy, erect setae on dorsal surface, longer laterally. Abdomen with short to moderately long erect setae on entire surface. **STRUCTURE: Head**: Cylindrical, L/W = 2.22. Postocular lobe short; in dorsal view distinctly narrowing through anterior 1/2, posterior 1/2 constant, tube-like. Eye prominent; lateral margin much wider than postocular lobe; dorsal margin attaining postocular transverse groove, ventral margin removed from ventral surface of head. *Labium*: I: II: III = 1: 1.5: 0.4. Basiflagellomere diameter larger than that of pedicel. **Thorax**: Anterolateral angle rounded; medial longitudinal sulcus evident throughout, deepening posteriorly. Posterior pronotal lobe with rugulose surface; disc distinctly elevated above humeral angle; humeral angle rounded or with minute projection. Scutellum short; apex angulate. *Legs*: Relatively robust, mesofemoral diameter greatest. *Hemelytron*: Surpassing apex of abdomen by more than length of abdominal segment seven; quadrate cell moderately sized; Cu and M of cubital cell converging. **GENITALIA**: (Fig. 92) *Pygophore*: Ovoid; not expanded laterally in dorsal view. Medial process cylindrical; slender; long, only slightly shorter than paramere; laterally somewhat compressed; posteriorly directed; curved at middle; apex in posterior view acute, without modification; base humped in lateral view. *Paramere*: Sickle-shaped; long, not reaching apex of medial process; directed toward medial process; strongly curved ventrad at mid-point, apex recurved dorsally; apical portion tapered. *Phallus*: Dorsal phallothecal sclerite somewhat pandurate, fiddle-shaped, medially constricted; apical portion of phallothecal sclerite gradually tapering, flat, laterally rounded, not forming angle; apex abruptly truncate, not emarginate; posterior margin of foramen nearly straight. Struts attached to dorsal phallothecal sclerite; apically separate, connected by bridge; basally separate. Basal plate arm moderately robust; basally slightly touching, not clearly fused; in lateral view very slightly curved; bridge extremely short; extension of basal plate expanded onto arm.

***Female***: (Fig. 91d) Similar to male, except for the following. Larger than male, total length 14.06–18.13 mm (mean 16.91 mm, Suppl. material 2). Reduced amount of dark areas on body, dorsal surface usually yellowish.

#### Diagnosis

Can be distinguished by the following combination of characters: Humeral angle without or with minute processes; head and legs predominantly reddish; abdominal segments usually banded; pronotum with erect, nearly spine-like setae. Males can also be recognized by the paramere greatly curved at middle and distinctly tapered apically; the medial process curved and directed posteriad; and the dorsal phallothecal sclerite constricted and the apex truncate, without emargination. The only species within the range of *Z. grassans* with which the females may be confused is *Z. ruficeps*. The pronotal armature readily separate them, that of *Z. ruficeps* consisting of broad dentate lateral processes while those of *Z. grassans* are as given above.

#### Distribution

**Figure 93.**
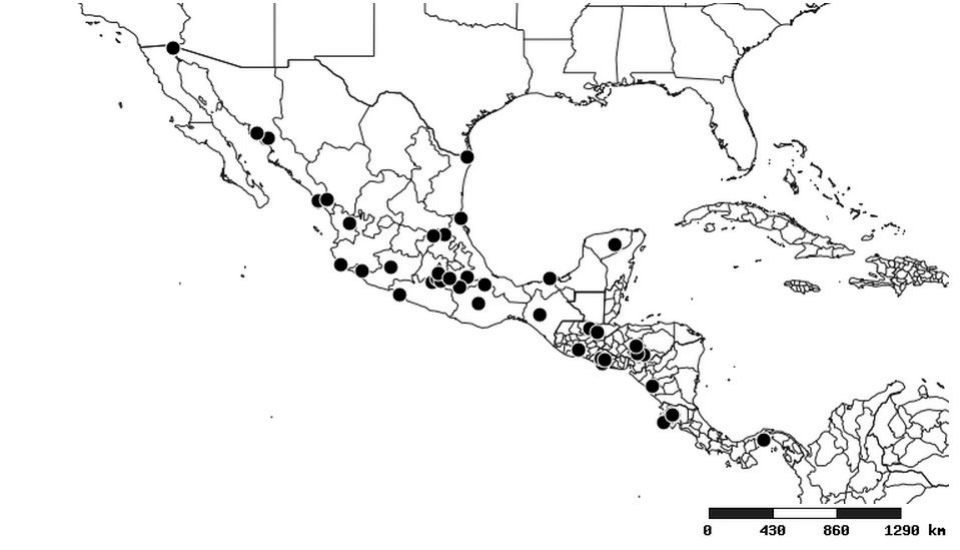
*Zelus grassans* Stål, 1862, specimen record map

From Mexico to Panama (Fig. 93). Countries with specimen records: Costa Rica, El Salvador, Guatemala, Honduras, Mexico, Nicaragua and Panama.

#### Taxon discussion

There is a great amount of size and color variations in *Z. grassans*. The dorsal coloration varies from almost entirely yellowish with only a narrow transverse dark band near the posterior margins of the pronotal lobes, to almost entirely dark brown. The legs show a similar range of coloration, from almost entirely light to entirely dark. The contrasting black and orange or red colors and the banded abdomen in many specimens of *Z. grassans* suggest that they may be mimics of *Dysdercus*, whose members have similarly strongly contrasting red and black colors. They have been observed to co-occur on the same plant (Zhang, unpublished), indicating that this may be a case of aggressive mimicry.

### Zelus illotus

Berg, 1879

Zelus illotus Berg, 1879, p. 153–154, orig. descr. (subgenus *Diplodus*); Lethierry and Severin, 1896, p. 152, cat.; Wygodzinsky, 1949a, p. 49, checklist; Wygodzinsky, 1949b, p. 336, note; Wygodzinsky, 1957, p. 264, 268, list and junior syn. of *Z. obscuridorsis*; Hart, 1987, p. 297, redescription, note, fig, key, lectotype desig. and stat. rev.; Maldonado, 1990, p. 330, cat.
Zelus carvalhoi Wygodzinsky, 1947, p. 428–431, orig. descr. and fig.; Zikan and Wygodzinsky, 1948, p. 17, list; Wygodzinsky, 1949a, p. 48, checklist; Wygodzinsky, 1949b, p. 336, note; wygodzinsky, 1957, p. 264, 268, list and junior syn. of *Z. obscuridorsis*; Hart, 1987, p. 297, junior syn. of *Z. illotus* Berg.

#### Materials

- **Type status:** Lectotype. **Occurrence:** occurrenceRemarks: Designated as lectotype by Hart (1987). Bears the following labels: Typus / Corrientes / 1554 / Lectotype, designated by E.R. Hart; recordedBy: Unknown; sex: Adult Male; **Taxon:** scientificName: Zelus illotus; family: Reduviidae; genus: Zelus; scientificNameAuthorship: Berg, 1879; **Location:** country: ARGENTINA; stateProvince: Corrientes; locality: Corrientes; decimalLatitude: -27.484102; decimalLongitude: -58.809555; georeferenceSources: Google map; **Identification:** identifiedBy: E.R. Hart 1972; **Event:** eventDate: no date provided; **Record Level:** institutionCode: Unversidad Nacional de La Plata
- **Type status:** Allolectotype. **Occurrence:** occurrenceRemarks: Designated as allolectotype by Hart (1987). Bears the following labels: Typus / Corrientes / *Zelus illotus* Bert / 168(9) / 1554 / Allolectotype, designated by E.R. Hart; recordedBy: Unknown; sex: Adult Female; **Taxon:** scientificName: Zelus illotus; family: Reduviidae; genus: Zelus; scientificNameAuthorship: Berg, 1879; **Location:** country: ARGENTINA; stateProvince: Corrientes; locality: Corrientes; decimalLatitude: -27.484102; decimalLongitude: -58.809555; georeferenceSources: Google map; **Identification:** identifiedBy: E.R. Hart 1972; **Event:** eventDate: no date provided; **Record Level:** institutionCode: Unversidad Nacional de La Plata
- **Type status:** Other material. **Occurrence:** occurrenceRemarks: **Holotype** of *Zelus carvalhoi* Wygodzinsky, 1947, junior synonym of *Zelus illotus* Berg, 1879 (Hart, 1987); recordedBy: J.C.M Carvalho; sex: Adult Male; **Taxon:** scientificName: Zelus illotus; family: Reduviidae; genus: Zelus; scientificNameAuthorship: Berg, 1879; **Location:** country: BRAZIL; stateProvince: Matto Grosso; locality: Chavantina, Rio des Mortes; decimalLatitude: -14.66667; decimalLongitude: -52.35; georeferenceSources: Gazetteer; **Identification:** identifiedBy: E.R. Hart 1972; **Event:** eventDate: 1947-06; **Record Level:** institutionCode: Museu Nacionaldo Rio de Janeiro
- **Type status:** Other material. **Occurrence:** occurrenceRemarks: **Paratype** of *Zelus carvalhoi* Wygodzinsky, 1947, junior synonym of *Zelus illotus* Berg, 1879 (Hart, 1987); recordedBy: J.C.M Carvalho; sex: Adult Male; **Taxon:** scientificName: Zelus illotus; family: Reduviidae; genus: Zelus; scientificNameAuthorship: Berg, 1879; **Location:** country: BRAZIL; stateProvince: Matto Grosso; locality: Chavantina, Rio des Mortes; decimalLatitude: -14.66667; decimalLongitude: -52.35; georeferenceSources: Gazetteer; **Identification:** identifiedBy: E.R. Hart 1972; **Event:** eventDate: 1947-06; **Record Level:** institutionCode: Instituto de Ecologia e Experimentacao Agricola, Rio de Janeiro
- **Type status:** Other material. **Occurrence:** occurrenceRemarks: **Paratype** of *Zelus carvalhoi* Wygodzinsky, 1947, junior synonym of *Zelus illotus* Berg, 1879 (Hart, 1987). Present location of specimen not known.; recordedBy: H. Sick; sex: Adult Male; **Taxon:** scientificName: Zelus illotus; family: Reduviidae; genus: Zelus; scientificNameAuthorship: Berg, 1879; **Location:** country: BRAZIL; stateProvince: Matto Grosso; locality: Chavantina; decimalLatitude: -14.66667; decimalLongitude: -52.35; georeferenceSources: Gazetteer; **Event:** eventDate: 1946-10; **Record Level:** institutionCode: None

#### Description

Figs 94, 95, 96

***Male***: (Fig. 94a, b) Small, total length 9.96–11.91 mm (mean 11.03 mm, Suppl. material 2); slender. **COLORATION**: Dorsal 1/2 brown to dark brown, ventral surface yellowish-brown. Rostrum yellowish-brown. Antennae reddish-brown to dark brown, bases and apices darker than shaft on scape and pedicel. Anterior pronotal lobe light to dark brown, yellowish-brown lateroventrally. Posterior lobe light to medium reddish-brown; humeral angle usually darkened, brownish-black; lateral surface lighter ventrally. Legs yellowish-brown, femora with reddish-brown to brownish-black bands at apices and basad to apical swelling, tibiae with at least two such dark bands, tibiae darkened toward apex. Hemelytron brown to dark brown. Dorsum of abdomen reddish-brown to dark brown, connexival margins and remainder of surface yellowish-brown. **VESTITURE**: Entire surface of head with short recumbent setae, short to moderate semi-erect and erect setae on lateroventral and ventral surfaces. Anterior pronotal lobe entire surface with short recumbent setae, confined to setal tracts dorsally, some longer erect setae laterally. Posterior lobe entire surface with short recumbent setae, some erect setae laterally. Recumbent setae over clavus and corium. Abdomen with short erect setae dorsally, lateral and ventral surfaces with short recumbent and scattered erect setae. Exposed surface of pygophore with short recumbent and short to long erect setae. **STRUCTURE: Head**: Elongated, L/W = 2.79. Postocular lobe long; in dorsal view anteriorly gradually narrowing, posterior portion constant, slightly narrower. Eye smallish; lateral margin only slightly wider than postocular lobe; dorsal and ventral margins removed from surfaces of head. *Labium*: I: II: III = 1: 2.0: 0.4. Basiflagellomere diameter larger than that of pedicel. **Thorax**: Anterolateral angle subtuberculate to tuberculate; medial longitudinal sulcus shallow near collar, deepening posteriorly. Posterior pronotal lobe with rugulose surface; disc distinctly elevated above humeral angle; humeral angle armed, with dentate or short spinous projection. Scutellum short; apex angulate. *Legs*: Very slender. *Hemelytron*: Slightly surpassing apex of abdomen, not more than length of abdominal segment seven; quadrate cell small, elongate; Cu and M of cubital cell subparallel. **GENITALIA**: (Fig. 95) *Pygophore*: Ovoid; not expanded laterally in dorsal view. Medial process slender; long, slightly shorter than paramere; laterally somewhat compressed; semi-erect; apically recurved; apex in posterior view acute, without modification. *Paramere*: Cylindrical; long, achieving apex of medial process; directed posteriad, basal half sharply curved towards medial process; apically slightly recurved; apical part not enlarged. *Phallus*: Dorsal phallothecal sclerite shield-shaped; small indentation of lateral margin at about mid-point; apical portion of phallothecal sclerite distinctly tapered, dorsal surface folded in middle, laterally rounded, not forming angle; posterior margin of foramen inversely V-shaped. Struts apical portion missing or not evident; basally separate. Basal plate arm moderately robust; basally fused; in lateral view very slightly curved; bridge extremely short; extension of basal plate heavily sclerotized, laterally expanded onto arm.

***Female***: (Fig. 94c) Similar to male, except for the following. Larger than male, total length 12.35–14.28 mm (mean 13.68 mm, Suppl. material 2). Coloration lighter than in male; pronotal coloration not as variable, usually concolorous yellowish-brown to dark brown.

#### Diagnosis

Among species of the *Zelus nugax* species group (Fig. 5), males of *Z. illotus* can be recognized by the paramere slender and long, curved in middle and recurved apically and the medial process also strongly recurved. The males of *Z. pedestris* and *Z. nugax* have straight blade-like medial processes. The females of this species cannot be consistently separated based on any character or combination of characters yet discovered from females of *Z. pedestris* and *Z. nugax*. Most females of *Z. impar* have almost no erect setae on the dorsal surface of the posterior pronotal lobe while most females of the other two species have readily noticeable erect setae on this area.

#### Distribution

**Figure 96.**
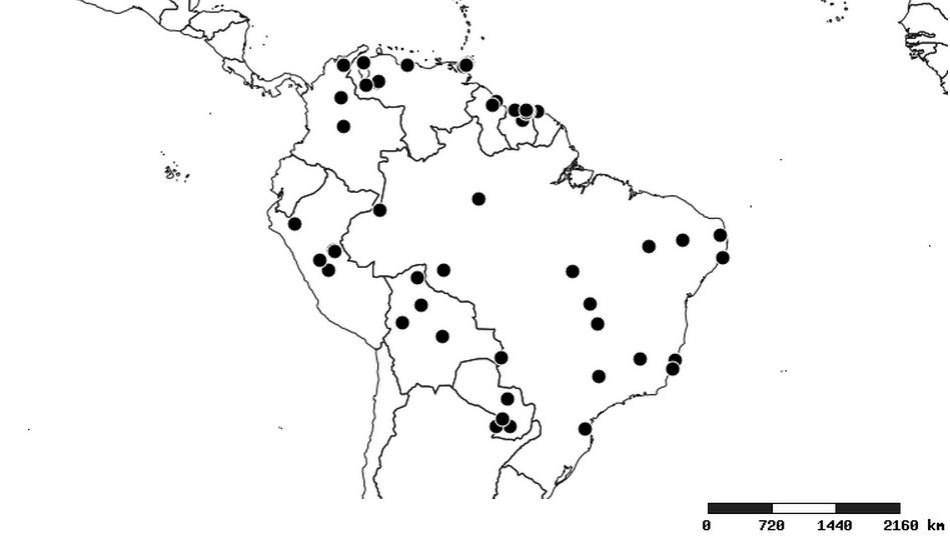
*Zelus illotus* Berg, 1879, specimen record map

South America and adjacent islands of the Caribbean (Fig. 96). Countries with records: Argentina, Bolivia, Brazil, Colombia, Guyana, Paraguay, Peru, Suriname, Trinidad and Tobago, and Venezuela.

### Zelus impar

Kuhlgatz & Melichar, 1902

Zelus impar Kuhlgatz, 1902, p. 264–266, Tab. IV, fig. 6, 6a, 6b, orig. descr. and fig.; Wygodzinsky, 1949a, p. 49, checklist; Hart, 1987, p. 297, redescription, note, fig., key and neotype desig.; Maldonado, 1990, p. 327, cat.

#### Materials

- **Type status:** Neotype. **Occurrence:** catalogNumber: UCR_ENT 00071190; occurrenceRemarks: Neotype designated by Hart (1987). Original type was destroyed; recordedBy: F. W. Walker; sex: Adult Male; **Taxon:** scientificName: Zelus impar; family: Reduviidae; genus: Zelus; scientificNameAuthorship: Kuhlgatz & Melichar, 1902; **Location:** country: COLOMBIA; stateProvince: Magdalena; locality: Santa Marta Mountains, Mount San Lorenzo; verbatimElevation: 1524 m; decimalLatitude: 11.12343; decimalLongitude: -74.0372; georeferenceSources: Gazetteer; **Identification:** identifiedBy: G. Zhang; dateIdentified: 2012; **Event:** eventDate: 1926-02-02; **Record Level:** institutionCode: TAMU

#### Description

Figs 97, 98, 99

***Male***: (Fig. 97) Small, total length 9.47–12.05 mm (mean 11.23 mm, Suppl. material 2); slender. **COLORATION**: Variable; at least two forms exist. In one form, most of surface dark brown, except posterior pronotal lobe, which is orangish-brown (Fig. 97). Femora brown with dark apical bands. In second form (Fig. 97), integument nearly uniformly blackish-brown, connexival margins lighter, femora with subapical inconspicuous lighter bands. **VESTITURE**: Sparsely setose. Head with short, recumbent and moderate to long erect setae, erect setae sparse dorsally. Anterior pronotal lobe with short, recumbent and moderately long, erect setae, confined to setal tracts; posterior pronotal lobe with short, recumbent and moderately long, erect setae. Abdomen with short, recumbent and short to moderately long erect setae laterally and ventrally. **STRUCTURE: Head**: Cylindrical, L/W = 2.24. Postocular lobe long; sloping to posterior constriction. Eye smallish; lateral margin only slightly wider than postocular lobe; dorsal and ventral margins removed from surfaces of head. *Labium*: I: II: III = 1: 1.8: 0.5. Basiflagellomere diameter larger than that of pedicel. **Thorax**: Anterolateral angle bearing small protuberance; medial longitudinal sulcus evident only on posterior 1/2, deepening anterior to transverse sulcus of pronotum. Posterior pronotal lobe with rugulose surface; disc distinctly elevated above humeral angle; humeral angle armed, with minute or dentate process. Scutellum long; apex slightly projected dorsad. *Legs*: Slender. Femoral diameters subequal. *Hemelytron*: Slightly surpassing apex of abdomen, not more than length of abdominal segment seven; quadrate cell small; Cu and M of cubital cell subparallel. **GENITALIA**: (Fig. 98) *Pygophore*: Elongate ovoid; not expanded laterally in dorsal view. Medial process cylindrical; very slender; long, only slightly shorter than paramere; somewhat laterally compressed; semi-erect; apically recurved; apex in posterior view acute, without modification. *Paramere*: Cylindrical; long, achieving apex of medial process; directed posteriad, slightly curved towards medial process; nearly straight; apical part not enlarged. *Phallus*: Dorsal phallothecal sclerite shield-shaped; small indentation of lateral margin at about mid-point; apical portion of phallothecal sclerite distinctly tapered, slightly folded in middle, laterally rounded, not forming angle; posterior margin of foramen inversely V-shaped. Struts attached to dorsal phallothecal sclerite; basally fused. Basal plate arm moderately robust; basally fused; in lateral view very slightly curved; bridge extremely short; extension of basal plate heavily sclerotized, laterally expanded onto arm.

***Female***: Unknown.

#### Diagnosis

Recognized by the slender, curved, laterally compressed, and apically tapered medial process (shared with members of the *Zelus nugax* species group; Fig. 5). Distinguished from *Z. nugax* and *Z. pedestris* by the recurved medial process. Similar to *Z. illotus* in having a recurved medial process, but is differentiated by the straight paramere.

#### Distribution

**Figure 99.**
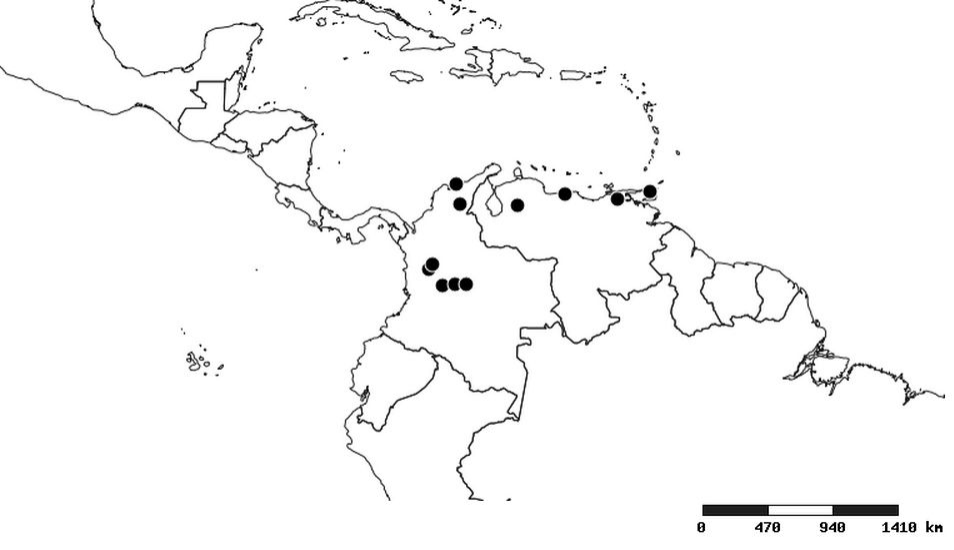
*Zelus impar* Kuhlgatz & Melichar, 1902, specimen record map

Panama and Northern South America and adjacent islands of the Caribbean (Fig. 99). Countries with records: Colombia, Panama (record not mapped), Trinidad and Tobago and Venezuela.

#### Taxon discussion

Hart (1987) designated a neotype for *Z. impar* because the original type material of that species was destroyed during World War II. This neotype specimen was at that time deposited in the private collection of J. C. Elkins, Houston,Texas. This specimen was eventually transferred to and deposited at TAMU, instead of AMNH as the author had indicated.

### Zelus inconstans

Champion, 1899

Zelus inconstans Champion, 1898, p. 254–255, Tab. XV., fig. 13, orig. descr. and fig.; Wygodzinsky, 1947, p. 43, note; Wygodzinsky, 1949a, p. 49, checklist; Maldonado, 1990, p. 328, cat.

#### Materials

- **Type status:** Lectotype. **Occurrence:** catalogNumber: UCR_ENT 00048756; occurrenceRemarks: Lectotype of *Zelus inconstans* Champion, 1898 (**New Designation** by Zhang, Hart & Weirauch, 2016). Verbatim label info: Type / B.C.A.Rhyn.II. *Zelus inconstans* Ch. / Sp. figured. / Bugaba, Panama. Champion. / Lectotype *Zelus inconstans* Champion des. by E.R. Hart/Lectotype *Zelus inconstans* Champion, 1898 designated and published by Zhang, Hart & Weirauch (2016); recordedBy: G.C. Champion; sex: Adult Female; **Taxon:** scientificName: Zelus inconstans; family: Reduviidae; genus: Zelus; scientificNameAuthorship: Champion, 1899; **Location:** country: PANAMA; stateProvince: Chiriqui; locality: Bugaba; decimalLatitude: 8.4833; decimalLongitude: -82.6167; **Identification:** identifiedBy: G. Zhang; dateIdentified: 2012; **Event:** eventDate: No date provided; **Record Level:** institutionCode: BMNH
- **Type status:** Paralectotype. **Occurrence:** occurrenceRemarks: Paralectotype of *Zelus inconstans* Champion, 1898 (**New Designation** by Zhang, Hart & Weirauch, 2016). Verbatim label info: Type / B.C.A.Rhyn.II. *Zelus inconstans* Ch. / Bugaba, Panama. Champion. / Paralectotype *Zelus inconstans* Champion des. by E.R. Hart/Lectotype Zelus inconstans Champion, 1898 designated and published by Zhang, Hart & Weirauch (2016); recordedBy: G.C. Champion; sex: Adult Female; **Taxon:** scientificName: Zelus inconstans; family: Reduviidae; genus: Zelus; scientificNameAuthorship: Champion, 1899; **Location:** country: PANAMA; stateProvince: Chiriqui; locality: Bugaba; decimalLatitude: 8.4833; decimalLongitude: -82.6167; **Identification:** identifiedBy: Hart; dateIdentified: 1972; **Event:** eventDate: No date provided; **Record Level:** institutionCode: BMNH
- **Type status:** Paralectotype. **Occurrence:** occurrenceRemarks: Paralectotype of *Zelus inconstans* Champion, 1898 (**New Designation** by Zhang, Hart & Weirauch, 2016). Verbatim label info: Type / B.C.A.Rhyn.II. *Zelus inconstans* Ch. / Bugaba, Panama. Champion. / Paralectotype *Zelus inconstans* Champion des. by E.R. Hart / Lectotype *Zelus inconstans* Champion, 1898 designated and published by Zhang, Hart & Weirauch (2016); recordedBy: G.C. Champion; sex: Adult Female; **Taxon:** scientificName: Zelus inconstans; family: Reduviidae; genus: Zelus; scientificNameAuthorship: Champion, 1899; **Location:** country: PANAMA; stateProvince: Chiriqui; locality: Bugaba; decimalLatitude: 8.4833; decimalLongitude: -82.6167; **Identification:** identifiedBy: Hart; dateIdentified: 1972; **Event:** eventDate: No date provided; **Record Level:** institutionCode: BMNH

#### Description

Figs 100, 101, 102

***Male***: (Fig. 100a, b​) Small, total length 10.05–12.70 mm (mean 11.42 mm, Suppl. material 2); slender. **COLORATION**: Head dark brown to brownish-black with yellowish-brown ventral surface, yellowish-brown areas lateroventrally behind compound eyes and mid-dorsally between ocellus. Anterior pronotal lobe yellowish-brown to brownish-black with posterior lateroventral surface light brown. Posterior lobe reddish-brown to dark brown dorsally but lighter than anterior lobe, light brown laterally. Meso- and metathorax light brown. Scutellum light brown, apex yellowish-brown. Coxae and trochanters dark brown to brownish-black, femora yellowish-brown to light brown with two-four darker brown bands, apex brownish-black, tibiae dark brown. Hemelytron dark brown. Dorsum of abdomen light brown anteriorly to dark brown at apex, lateral and ventral surfaces light brown. Pygophore brown, darker posteriorly. **VESTITURE**: Sparsely setose. Short recumbent and erect setae over surface of head. Anterior pronotal lobe with sparse short erect setae dorsally confined to vestigial setal tracts, dense recumbent and erect setae laterally, some anterolateral setae quite long. Posterior lobe with short erect and recumbent setae over surface. Scutellum with semi-erect to erect setae. Short recumbent setae over clavus and corium, some erect setae on veins. Short erect setae dorsally on abdomen, short recumbent and short to moderate erect setae over remainder of surface. Exposed surface of pygophore with short to long erect setae. **STRUCTURE: Head**: Cylindrical, L/W = 2.04. Postocular lobe long; somewhat abruptly constricted in posterior half. Eye moderately sized. Ocellus prominent. *Labium*: I: II: III = 1: 1.9: 0.5. Basiflagellomere diameter larger than that of pedicel. **Thorax**: Anterolateral angle of collar rounded to subtuberculate; medial longitudinal sulcus shallow near collar, deepening posteriorly. Posterior pronotal lobe with rugulose surface; disc distinctly elevated above humeral angle; humeral angle rounded. Scutellum moderately long; apex angulate. *Legs*: Slender. Pro- and metafemoral diameters subequal, mesofemora slightly larger. *Hemelytron*: Slightly surpassing apex of abdomen, not more than length of abdominal segment seven; quadrate cell small; Cu and M of cubital cell subparallel. **GENITALIA**: (Fig. 101) *Pygophore*: Elongate ovoid. Medial process broad; short; semi-erect; apex strongly folded posteriorly and ventrally. *Paramere*: Cylindrical; not reaching apex of medial process; directed posteriad, slightly curved towards medial process; apex strongly curved dorsad, nearly vertical; apical part not enlarged. *Phallus*: Dorsal phallothecal sclerite semi-cylindrical, sharp fold running obliquely from middle of margin to about 3/4 distance to foramen, apical portion of phallothecal sclerite gradually tapering, apex convex, laterally rounded, not forming angle; posterior margin of foramen broadly concave. Struts basally attached to dorsal phallothecal sclerite; poorly sclerotized and not readily evident beyond base. Basal plate arm robust; basally briefly fused; bridge short; extension of basal plate heavily sclerotized, extending well onto lateral margins of arms.

***Female***: (Fig. 100c, d) Similar to male, except for the following. Larger than male, total length 10.38–12.12 mm (mean 11.36 mm, Suppl. material 2). Posterior pronotal lobe orangish-brown.

#### Diagnosis

The humeral angle rounded and the pronotum lacking conspicuous spine-like setae are diagnostic of this species. Most other species with unarmed humeral angle also have spine-like setae on pronotum and are in the *Zelus longipes* species group. Males can also be recognized by the paramere apically greatly projected dorsad (Fig. 101a), nearly 90 degree; and the apex of the medial process folded posteriorly and ventrally. Very similar to *Z. mimus*, but the medial process is much shorter and broader.

#### Distribution

**Figure 102.**
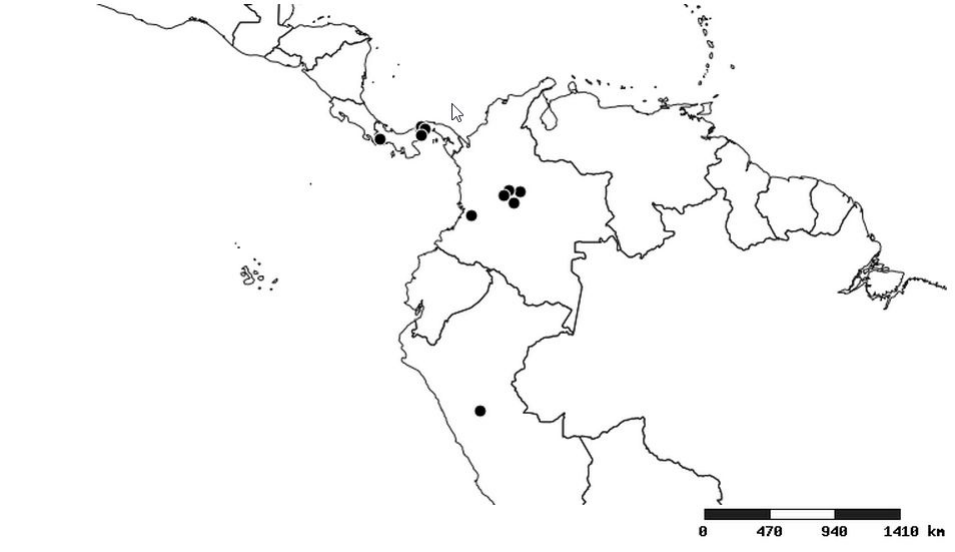
*Zelus inconstans* Champion, 1899, specimen record map

Southern Central America and northern South America (Fig. 102). Countries with records: Colombia, Panama and Peru.

#### Taxon discussion

Coloration variations are mainly seen on the pronotum. The two males from Panama show similar coloration, the central 1/3 of the pronotum being lighter than the margins. The Colombian male, however, has a nearly unicolorous pronotum. The females from Panama have pronotal coloration ranging from reddish-brown to brown, the lighter color apparently being more common. The single female from Colombia has the lighter pronotum while that of the Peruvian specimen is dark brown. The lectotype and two paralectotypes exhibit the lighter coloration. The banding patterns of the legs appear to be somewhat variable in Panama, the only area from which several specimens are available for comparison.

### Zelus janus

Stål, 1862

Zelus janus Stål, 1862, p. 452, orig. descr.; Stål, 1872, p. 90, cat. (subgenus *Diplodus*); Lethierry and Severin, 1896, p. 152, cat.; Champion, 1898, (in part), p. 257–258, Tab. XV. fig. 19, note, fig. and senior syn. of *Z. litigiosus*; Fracker, 1913, p. 239, 240, key and list (subgenus *Diplodus*); Fracker and Bruner, 1924, p. 170–171, note; Wygodzinsky, 1949a, p. 49, checklist; Hart, 1986, p. 543, redescription, lectotype desig., note, key and fig.; Maldonado, 1990, p. 328, cat.
Diplodus janus : Uhler, 1886, p. 24, checklist; Walker, 1873, p. 124, cat.

#### Materials

- **Type status:** Lectotype. **Occurrence:** catalogNumber: UCR_ENT 00041005; occurrenceRemarks: Lectotype designated by Hart (1986). Verbatim label info: Mexico / Salle / janus Stal / Lectotype *Zelus janus* Stal / designated by E.R.Hart / Typus / NHRS-GULI 000000331; recordedBy: Salle; sex: Adult Male; otherCatalogNumbers: NHRS-GULI 000000331; **Taxon:** scientificName: Zelus janus; family: Reduviidae; genus: Zelus; scientificNameAuthorship: Stål, 1862; **Location:** country: MEXICO; **Record Level:** institutionCode: NHRS
- **Type status:** Allolectotype. **Occurrence:** occurrenceRemarks: Allolectotype designated by Hart (1986).; recordedBy: Salle; sex: Adult Female; **Taxon:** scientificName: Zelus janus; family: Reduviidae; genus: Zelus; scientificNameAuthorship: Stål, 1862; **Location:** country: MEXICO; **Record Level:** institutionCode: NHRS
- **Type status:** Paralectotype. **Occurrence:** occurrenceRemarks: Paralectotype designated by Hart (1986).; recordedBy: Salle; sex: Adult Male; **Taxon:** scientificName: Zelus janus; family: Reduviidae; genus: Zelus; scientificNameAuthorship: Stål, 1862; **Location:** country: MEXICO; **Record Level:** institutionCode: NHRS
- **Type status:** Paralectotype. **Occurrence:** occurrenceRemarks: Paralectotype designated by Hart (1986).; recordedBy: Salle; sex: Adult Female; **Taxon:** scientificName: Zelus janus; family: Reduviidae; genus: Zelus; scientificNameAuthorship: Stål, 1862; **Location:** country: MEXICO; **Record Level:** institutionCode: NHRS
- **Type status:** Paralectotype. **Occurrence:** occurrenceRemarks: Paralectotype designated by Hart (1986). Bears labels: Mexico / CoIl. Signoret / Janus, det. Stal.; recordedBy: Signoret; sex: Adult Male; **Taxon:** scientificName: Zelus janus; family: Reduviidae; genus: Zelus; scientificNameAuthorship: Stål, 1862; **Location:** country: MEXICO; **Record Level:** institutionCode: NHMW
- **Type status:** Paralectotype. **Occurrence:** occurrenceRemarks: Paralectotypes designated by Hart (1986). Bear labels: Mexico / CoIl. Signoret / Janus, det. Stal.; recordedBy: Signoret; individualCount: 3; sex: Adult Female; **Taxon:** scientificName: Zelus janus; family: Reduviidae; genus: Zelus; scientificNameAuthorship: Stål, 1862; **Location:** country: MEXICO; **Record Level:** institutionCode: NHMW

#### Description

Figs 103, 104, 105

***Male***: (Fig. 103a, b, e) Large, total length 15.44–19.55 mm (mean 17.63 mm, Suppl. material 2); robust. **COLORATION**: Dorsal surface nearly uniformly brown; distal part of corium sometimes yellowish or reddish. Lateral and ventral surfaces yellowish-brown; dark brown stripe along posterior margin of abdominal segment, single dark spot anterolaterally on each segment. Legs with indistinct banding or completely dark brown. **VESTITURE**: Sparsely setose. Similar to that in *Z. armillatus*; adpressed setae denser; long, erect setae on head and pronotum relatively shorter. **STRUCTURE: Head**: Cylindrical, L/W = 2.28. Postocular lobe long; in dorsal view anteriorly gradually narrowing, posterior portion constant, slightly narrower. Eye smallish; lateral margin much wider than postocular lobe; dorsal and ventral margins removed from surfaces of head. *Labium*: I: II: III = 1: 1.3: 0.3. Basiflagellomere diameter smaller than that of pedicel. **Thorax**: Anterolateral angle bearing small projection; medial longitudinal sulcus shallow near collar, deepening posteriorly. Posterior pronotal lobe with finely rugulose surface; disc about same level as humeral angle; humeral angle armed, with short tuberculate processes. Scutellum short; apex angulate. *Legs*: Robust, metafemoral diameter smallest. *Hemelytron*: Slightly surpassing apex of abdomen, not more than length of abdominal segment seven; quadrate cell large and broad; Cu and M of cubital cell converging towards R. **GENITALIA**: (Fig. 104) *Pygophore*: Ovoid; slightly expanded laterally near base of paramere in dorsal view; posteriorly expanded sac-like sclerite between paramere and medial process. Medial process cylindrical; slender; short; semi-erect; slightly curved; apex in posterior view truncate, with small sharp lateral projections. *Paramere*: Cylindrical; short, not reaching apex of medial process; directed posteriad; not distinctly curved; apical part not enlarged. *Phallus*: Dorsal phallothecal sclerite shield-shaped; lateral expansion arising close to base; apical portion of phallothecal sclerite not distinctly tapered, flat, laterally angulate; apex truncate, not emarginate; posterior margin of foramen broadly concave, medially deeper. Struts attached to dorsal phallothecal sclerite; apically separate, connected by bridge; basally separate. Basal plate arm moderately robust; separate; converging; in lateral view slightly curved; bridge short; extension of basal plate expanded onto arm.

***Female***: (Fig. 103c, d) Similar to male, except for the following. Larger than male, total length 19.01–21.96 mm (mean 20.53 mm, Suppl. material 2). Whitish area on distal part of corium smaller, almost non-existent in some specimens; posterior pronotal lobe, corium and clavus entirely yellowish-brown in some specimens.

#### Diagnosis

Among closely related species in the *Zelus armillatus* species group with overlapping distribution ranges, *Z. janus* is the only species with the humeral angle elevated to about same level as the disc of the posterior pronotal lobe. This species is much larger than and the coloration different from two other species sharing this feature, *Z. exsanguis* and *Z. ambulans*, both from a different species group. Other characters useful for diagnosis include the dorsal surface usually mostly brown and the lateral and ventral surfaces yellowish-brown; the abdominal segment each with single dark spot anteriorly; and in males the medial process narrower, relatively short.

#### Distribution

**Figure 105.**
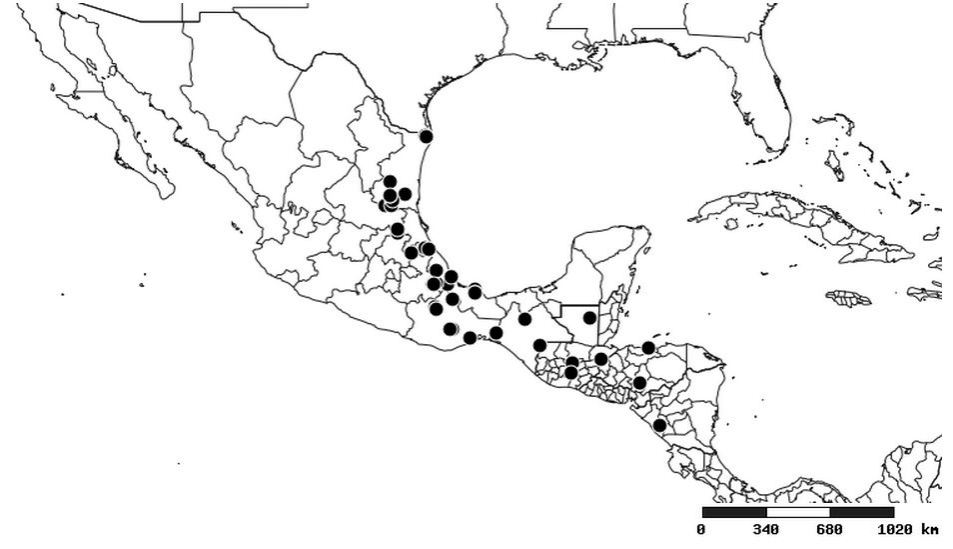
*Zelus janus* Stål, 1862, specimen record map

Southern Texas to Central America (Fig. 105). Countries with records: USA (Texas), Belize, Guatemala, Honduras, Mexico and Nicaragua.

#### Taxon discussion

It is rather difficult to distinguish *Z. janus* from *Z. armillatus* on the basis of the male genitalia alone, the only two species in the genus where such distinction cannot be made. As the humeral angle of the posterior pronotal lobe are raised to the level of and are nearly continuous with the disc, however, it is quite easy to separate specimens of these species. There is further divergence in coloration and pattern between the two which may be seen in the descriptions of these species. A sympatric and closely related species, *Z. litigiosus*, also has the disc elevated above the humeral angle and is easily distinguished. *Zelus janus* has a somewhat more uniform brown dorsal coloration, whereas the color pattern in *Z. litigiosus* is more variable.

### Zelus kartabensis

Haviland, 1931

Zelus kartabensis Haviland, 1931: 137, 148, 152 (checklist, fig. and orig. descr. [subgenus *Diplodus*]); Wygodzinsky, 1949a: 49 (checklist); Maldonado, 1990, p. 328, cat.
Zelus pallidinervus Haviland, 1931: 137, 148, 153 (checklist, fig and orig. descr. [subgenus *Diplodus*]); Wygodzinsky, 1949a: 50 (checklist); Maldonado, 1990, p. 330, cat. **syn. nov.** (current study).

#### Materials

- **Type status:** Lectotype. **Occurrence:** catalogNumber: UCR_ENT 00048761; occurrenceRemarks: Lectotype of *Zelus kartabensis* Haviland, 1931 (**New Designation** by Zhang, Hart & Weirauch, 2016). Verbatim label info: Type / [blue label, no content] / Kartabo, Brit. Guiana June 1922 e coll.M.D. Haviland d.d.Collegium Newnhamense / Pres. by Mrs Brindley. B.M.1928-172. / *Zelus kartabensis* Haviland / Lectotype *Zelus kartabensis* Haviland des. by E.R. Hart; recordedBy: M.D. Haviland; sex: Adult Male; **Taxon:** scientificName: Zelus kartabensis; family: Reduviidae; genus: Zelus; scientificNameAuthorship: Haviland, 1931; **Location:** country: GUYANA; stateProvince: Cuyuni-Mazaruni Region; locality: Kartabo, British Guiana; decimalLatitude: 6.384; decimalLongitude: -58.695; **Event:** eventDate: 1922-06; **Record Level:** institutionCode: BMNH
- **Type status:** Paralectotype. **Occurrence:** catalogNumber: UCR_ENT 00048761; occurrenceRemarks: Paralectotype of *Zelus kartabensis* Haviland, 1931 (**New Designation** by Zhang, Hart & Weirauch, 2016). Verbatim label info: Kartabo, Brit. Guiana July 1922 e coll.M.D. Haviland d.d.Collegium Newnhamense / Pres. by Mrs Brindley. B.M.1928-172. / *Zelus Kartabensis* Haviland / Paralectotype *Zelus kartabensis* Haviland des. by E.R. Hart; recordedBy: M.D. Haviland; sex: Adult Male; **Taxon:** scientificName: Zelus kartabensis; family: Reduviidae; genus: Zelus; scientificNameAuthorship: Haviland, 1931; **Location:** country: GUYANA; stateProvince: Cuyuni-Mazaruni Region; locality: Kartabo, British Guiana; decimalLatitude: 6.384; decimalLongitude: -58.695; **Event:** eventDate: 1922-07; **Record Level:** institutionCode: BMNH
- **Type status:** Other material. **Occurrence:** occurrenceRemarks: Lectotype of *Zelus pallidinervus* Haviland, 1931 (**New Designation** by Zhang, Hart & Weirauch, 2016), junior synonym of *Zelus kartabensis* Haviland, 1931. Bears labels: Type / Kartabo, Brit. Guiana, August 1922, e. coll. M. D. Haviland, d. d. collegium Newnhamense / Pres. by Mrs. Brindley, B. M. 1928-127 / *Zelus pallidinervis* Haviland; recordedBy: M.D. Haviland; sex: Adult Female; **Taxon:** scientificName: Zelus kartabensis; family: Reduviidae; genus: Zelus; scientificNameAuthorship: Haviland, 1931; **Location:** country: GUYANA; stateProvince: Cuyuni-Mazaruni Region; locality: Kartabo, British Guiana; decimalLatitude: 6.384; decimalLongitude: -58.695; **Event:** eventDate: 1922-08; **Record Level:** institutionCode: BMNH
- **Type status:** Other material. **Occurrence:** occurrenceRemarks: **Paralectotype** of *Zelus pallidinervus* Haviland, 1931 (**New Designation** by Zhang, Hart & Weirauch, 2016), junior synonym of *Zelus kartabensis* Haviland, 1931. Bears labels: Kartabo, Brit. Guiana, September 1922, e. coll. M. D. Haviland, d. d. collegium Newnhamense / Pres. by Mrs. Brindley, B. M. 1928-127 / *Zelus pallidinervis* Haviland; recordedBy: M.D. Haviland; sex: Adult Female; **Taxon:** scientificName: Zelus kartabensis; family: Reduviidae; genus: Zelus; scientificNameAuthorship: Haviland, 1931; **Location:** country: GUYANA; stateProvince: Cuyuni-Mazaruni Region; locality: Kartabo, British Guiana; decimalLatitude: 6.384; decimalLongitude: -58.695; **Event:** eventDate: 1922-08; **Record Level:** institutionCode: BMNH

#### Description

Figs 106, 107, 108

***Male***: (Fig. 106a, b) Medium-sized; slender; total length 10.97–12.67 mm (mean 11.94 mm, Suppl. material 2). **COLORATION**: Dorsum of pronotum and hemelytron dark brown. Lateral surface of posterior pronotal lobe, parts of pleura, sometimes medial regions of abdominal venter yellowish. Head dorsal surface mostly yellowish to pale, sometimes brown stripes or areas more dominant; brown medial longitudinal stripes on anteocular lobe dorsal surface; brown areas on dorsal surface of postocular lobe, anteriorly broad, medially separated by yellowish stripe, narrowing and converging posteriorly; brown stripe between eye and antennal insertion; ventral surface entirely yellowish. Legs brown; meso or metafemora sub-basally, medially, or sub-apically with yellowish band(s); meso and metatibiae occasionally with inconspicuous medial yellow band; fore leg never banded. **VESTITURE**: Sparsely setose. Dorsum primarily consisting of moderately dense, short, erect or recumbent setae; short spine-like setae also on dorsum of head, more concentrated on anteocular lobe, and on pronotum. Sundew setae on profemur sparsely and randomly distributed. Microtrichia throughout posterior margin of membrane of hemelytron. Abdominal venter with short, recumbent setae, intermixed with sparse, moderately long, erect setae. Setae on pypophore short to long, recumbent to erect; paramere apical 1/2 with dense, very long, almost as long as paramere, erect, apically curved setae, directed mediad. **STRUCTURE: Head**: Cylindrical, L/W = 2.5. Eye moderately sized; dorsal and ventral margins removed from surfaces of head. *Labium*: I: II: III = 1: 2.0: 0.4. Basiflagellomere diameter larger than that of pedicel. **Thorax**: Anterolateral angle rounded, sometimes bearing protuberance, never prominently acute; medial longitudinal sulcus shallow near collar, deepening posteriorly. Posterior pronotal lobe with rugulose surface; disc distinctly elevated above humeral angle; humeral angle armed, with short tuberculate processes. Scutellum long; apex angulate, slightly projected upward. *Legs*: Slender. *Hemelytron*: Surpassing apex of abdomen by about length of abdominal segment seven; quadrate cell small and slender; Cu and M of cubital cell subparallel. **GENITALIA**: (Fig. 107) *Pygophore*: Ovoid; expanded near base of paramere in dorsal view, expansion oriented dorsad in lateral view. Medial process triangular; long; erect; nearly straight; apex in posterior view acute. *Paramere*: Somewhat sickle-shaped; moderately long, slightly exceeding medial process; curved ventrad; apical part enlarged. *Phallus*: Dorsal phallothecal sclerite shield-shaped; sharp lateral transverse ridge-like expansions; convex; apex truncate or slightly rounded; posterior margin of foramen deeply concave. Struts weakly sclerotized to non-visible through apical 1/2; apically separate, not connected by bridge; basally almost completely fused. Basal plate arm moderately robust; separate; subparallel; bridge moderately long; extension of basal plate expanded onto arm.

***Female***: (Fig. 106c, d) Different from male as outlined below. Larger than male, total length 15.49–17.45 mm (mean 16.53 mm, Suppl. material 2). Mostly yellowish to greenish. No dark dorsal markings of anteocular lobe; remainder of dark cranial markings less pronounced than in male. Posterior margin of posterior pronotal lobe and corium dark. Femora unbanded. Spine-like setae more conspicuous than in male. Basiflagellomere not swollen basally. Humeral angle nearly elevated to level of pronotal disc; lateral processes spinous.

#### Diagnosis

The dorsal surface of posterior pronotal lobe uniformly dark brown. The paramere gradually enlarged, somewhat club-shaped; the medial process with ridge-like medial elevation through apical 1/2.

*Zelus kartabensis* is most similar to *Z. kartaboides*, and the differences between the two species are presented in the diagnosis of the latter species.

#### Distribution

**Figure 108.**
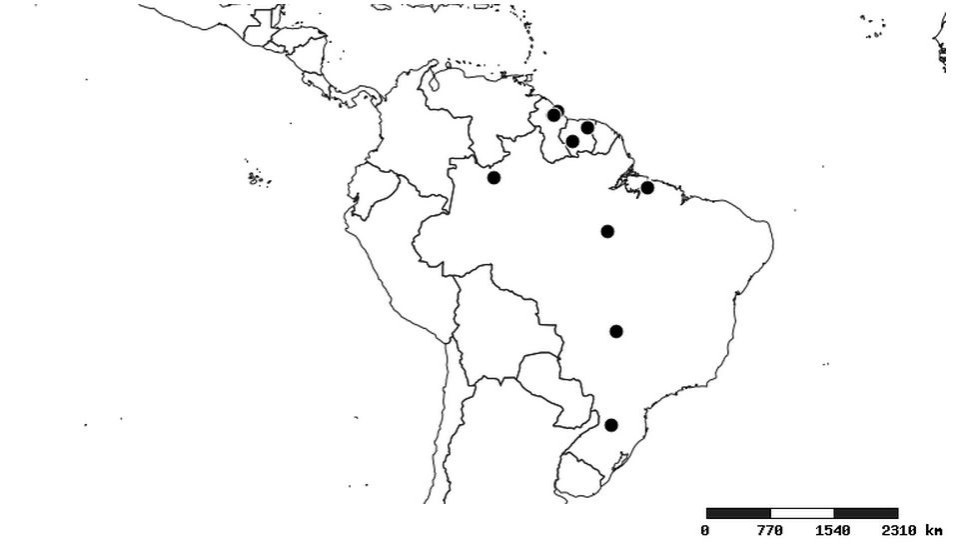
*Zelus kartabensis* Haviland, 1931, specimen record map

South America (Fig. 108). Countries with records: Brazil, Guyana and Suriname.

### Zelus kartaboides

Zhang & Hart sp. n.

urn:lsid:zoobank.org:act:897C32EA-F2DF-4478-9A61-EEC11F44E06C

#### Materials

- **Type status:** Holotype. **Occurrence:** catalogNumber: UCR_ENT 00071182; occurrenceRemarks: Holotype of *Zelus kartaboides* Zhang & Hart, 2016; recordedBy: Richter; sex: Adult Male; **Taxon:** scientificName: Zelus kartaboides; family: Reduviidae; genus: Zelus; scientificNameAuthorship: Zhang and Hart, 2016; **Location:** country: COLOMBIA; stateProvince: Meta; locality: Rio Guayuriba; verbatimElevation: 400 m; decimalLatitude: 4.01978; decimalLongitude: -73.60807; georeferenceSources: Google Earth; **Identification:** identifiedBy: G. Zhang; dateIdentified: 2013; **Event:** eventDate: 1947-09-06; **Record Level:** institutionCode: TAMU
- **Type status:** Paratype. **Occurrence:** catalogNumber: UCR_ENT 00042153; occurrenceRemarks: Paratype of *Zelus kartaboides* Zhang & Hart, 2016; recordedBy: C. Lindemann; sex: Adult Male; **Taxon:** scientificName: Zelus kartaboides; family: Reduviidae; genus: Zelus; scientificNameAuthorship: Zhang and Hart, 2016; **Location:** country: BRAZIL; stateProvince: Amazonas; locality: Tapurucuara Rio Negro; decimalLatitude: -0.4; decimalLongitude: -65.0333; georeferenceSources: Gazetteer; **Identification:** identifiedBy: G. Zhang; dateIdentified: 2013; **Event:** eventDate: 1963-02-22; **Record Level:** institutionCode: ZSM
- **Type status:** Paratype. **Occurrence:** catalogNumber: UCR_ENT 00017890; occurrenceRemarks: Paratype of *Zelus kartaboides* Zhang & Hart, 2016; recordedBy: D. Engleman; sex: Adult Male; **Taxon:** scientificName: Zelus kartaboides; family: Reduviidae; genus: Zelus; scientificNameAuthorship: Zhang and Hart, 2016; **Location:** country: BRAZIL; stateProvince: Mato Grosso; locality: Mato Gr.; decimalLatitude: -10.41666; decimalLongitude: -59.46667; georeferenceSources: Label; **Identification:** identifiedBy: G. Zhang; dateIdentified: 2013; **Event:** eventDate: 1977-03-17; **Record Level:** institutionCode: AMNH
- **Type status:** Paratype. **Occurrence:** catalogNumber: UCR_ENT 00046978; occurrenceRemarks: Paratype of *Zelus kartaboides* Zhang & Hart, 2016; recordedBy: D. Engleman; sex: Adult Male; **Taxon:** scientificName: Zelus kartaboides; family: Reduviidae; genus: Zelus; scientificNameAuthorship: Zhang and Hart, 2016; **Location:** country: BRAZIL; stateProvince: Mato Grosso; locality: Mato Gr.; decimalLatitude: -10.41666; decimalLongitude: -59.46667; georeferenceSources: Label; **Identification:** identifiedBy: G. Zhang; dateIdentified: 2013; **Event:** eventDate: 1977-03-17 to 1977-03-22; **Record Level:** institutionCode: AMNH
- **Type status:** Paratype. **Occurrence:** catalogNumber: UCR_ENT 00046982; occurrenceRemarks: Paratype of *Zelus kartaboides* Zhang & Hart, 2016; recordedBy: D. Engleman; sex: Adult Male; **Taxon:** scientificName: Zelus kartaboides; family: Reduviidae; genus: Zelus; scientificNameAuthorship: Zhang and Hart, 2016; **Location:** country: BRAZIL; stateProvince: Mato Grosso; locality: Mato Gr.; decimalLatitude: -10.41666; decimalLongitude: -59.46667; georeferenceSources: Label; **Identification:** identifiedBy: G. Zhang; dateIdentified: 2013; **Event:** eventDate: 1977-03-17 to 1977-03-22; **Record Level:** institutionCode: AMNH
- **Type status:** Paratype. **Occurrence:** catalogNumber: UCR_ENT 00047047; occurrenceRemarks: Paratype of *Zelus kartaboides* Zhang & Hart, 2016; recordedBy: M. Alvarenga; sex: Adult Male; **Taxon:** scientificName: Zelus kartaboides; family: Reduviidae; genus: Zelus; scientificNameAuthorship: Zhang and Hart, 2016; **Location:** country: BRAZIL; stateProvince: Para; locality: Tucurui; decimalLatitude: -3.7; decimalLongitude: -49.7; georeferenceSources: Gazetteer; **Identification:** identifiedBy: G. Zhang; dateIdentified: 2013; **Event:** eventDate: 1979-01-01; **Record Level:** institutionCode: AMNH
- **Type status:** Paratype. **Occurrence:** catalogNumber: UCR_ENT 00017862; occurrenceRemarks: Paratype of *Zelus kartaboides* Zhang & Hart, 2016; recordedBy: Richter; sex: Adult Male; **Taxon:** scientificName: Zelus kartaboides; family: Reduviidae; genus: Zelus; scientificNameAuthorship: Zhang and Hart, 2016; **Location:** country: COLOMBIA; stateProvince: Meta; locality: Rio Guayeriba, a triburary of Rio Meta; **Identification:** identifiedBy: G. Zhang; dateIdentified: 2013; **Event:** eventDate: 1948-05-17; **Record Level:** institutionCode: AMNH
- **Type status:** Paratype. **Occurrence:** catalogNumber: UCR_ENT 00071183; occurrenceRemarks: Paratype of *Zelus kartaboides* Zhang & Hart, 2016; recordedBy: Richter; sex: Adult Male; **Taxon:** scientificName: Zelus kartaboides; family: Reduviidae; genus: Zelus; scientificNameAuthorship: Zhang and Hart, 2016; **Location:** country: COLOMBIA; stateProvince: Meta; locality: Cano Grande; verbatimElevation: 490 m; **Identification:** identifiedBy: G. Zhang; dateIdentified: 2013; **Event:** eventDate: 1948-01-20; **Record Level:** institutionCode: TAMU
- **Type status:** Paratype. **Occurrence:** catalogNumber: UCR_ENT 00071184; occurrenceRemarks: Paratype of *Zelus kartaboides* Zhang & Hart, 2016; recordedBy: Richter; sex: Adult Male; **Taxon:** scientificName: Zelus kartaboides; family: Reduviidae; genus: Zelus; scientificNameAuthorship: Zhang and Hart, 2016; **Location:** country: COLOMBIA; stateProvince: unknown; locality: Buena Vista; verbatimElevation: 1300 m; **Identification:** identifiedBy: G. Zhang; dateIdentified: 2013; **Event:** eventDate: 1944-02-06; **Record Level:** institutionCode: TAMU
- **Type status:** Paratype. **Occurrence:** catalogNumber: UCR_ENT 00009521; occurrenceRemarks: Paratype of *Zelus kartaboides* Zhang & Hart, 2016. Drake Collection; recordedBy: L. Pena; sex: Adult Male; **Taxon:** scientificName: Zelus kartaboides; family: Reduviidae; genus: Zelus; scientificNameAuthorship: Zhang and Hart, 2016; **Location:** country: ECUADOR; stateProvince: Sucumbios; locality: Dureno; verbatimElevation: 150 m; decimalLatitude: 0.0444; decimalLongitude: -76.6972; georeferenceSources: Gazetteer; **Identification:** identifiedBy: G. Zhang; dateIdentified: 2013; **Event:** eventDate: 1977-23-09 to 1977-09-30; **Record Level:** institutionCode: USNM
- **Type status:** Paratype. **Occurrence:** catalogNumber: UCR_ENT 00019083; occurrenceRemarks: Paratype of *Zelus kartaboides* Zhang & Hart, 2016; recordedBy: E. I. Schlinger & E. S. Ross; sex: Adult Male; **Taxon:** scientificName: Zelus kartaboides; family: Reduviidae; genus: Zelus; scientificNameAuthorship: Zhang and Hart, 2016; **Location:** country: PERU; stateProvince: Huanuco; locality: Monzon valley, Tingo Maria; decimalLatitude: -9.27816; decimalLongitude: -76.05562; georeferenceSources: Google Earth; **Identification:** identifiedBy: G. Zhang; dateIdentified: 2013; **Event:** eventDate: 1954-10-15; **Record Level:** institutionCode: CAS

#### Description

Figs 109, 110, 111

***Male***: (Fig. 109) Medium-sized, total length 11.22-11.61 mm (mean 11.6 mm, Suppl. material 2); Coloration, vestiture and structure including genitalia very similar to *Z. kartabensis* except for the following. **COLORATION**: Lateral surface and posterior margin of posterior pronotal lobe, and scutellum yellowish, lighter than remainder of body surface. **GENITALIA**: (Fig. 110) *Pygophore*: Posterolateral rim of pygophore in smaller angle with body long-axis in lateral view, nearly horizontal; lateral protrusion on posterodorsal rim of pygophore more pronounced, proximal side of arch extending down as process. Medial process short. *Paramere*: Paramere more strongly curved, banana-like; diameter uniform throughout, apex not enlarged, base slightly constricted. *Phallus*: Struts apically diverging, V-shaped.

***Female***: Unknown.

#### Diagnosis

The posterior margin of the pronotum and the scutellum yellowish, strongly contrasting to the remaining dark brown dorsal surface, makes this species easily recognizable among all species of *Zelus*, including the very similarly looking *Z. kartabensis*. It can also be recognized by the medial process with ridge-like medial elevation through apical 1/2 (also in *Z. kartabensis* and *Z. chamaeleon*). It is separated from *Z. kartabensis* by the paramere uniquely shaped like a banana and its diameter uniform throughout. Some specimens of *Z. armillatus*, *Z. conjungens, Z. longipes* and *Z. ruficeps* also exhibit a predominantly dark brown pronotum with posterior and/or dorsolateral margins yellow or orange, but they are much more larger and robust than *Z. kartaboides* and the male genitalic structures are very different.

#### Etymology

The specific epithet indicates that this species is rather similar to *Z. kartabensis*.

#### Distribution

**Figure 111.**
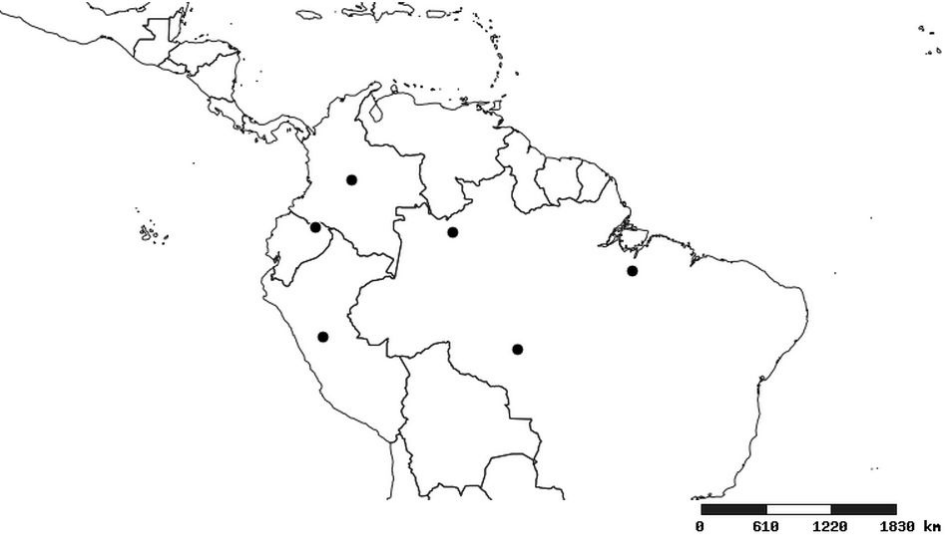
*Zelus kartaboides* Zhang & Hart, sp. n., specimen record map

South America (Fig. 111). Countries with records: Brazil, Colombia, Ecuador and Peru.

### Zelus korystos

Hart, 1986

Zelus korystos Hart, 1986, p. 303-304, figs. 25–27, orig. descri., note, fig. and key; Maldonado, 1990, p. 328, cat.

#### Materials

- **Type status:** Holotype. **Occurrence:** catalogNumber: UCR_ENT 00008000; occurrenceRemarks: Genitalia dissected; recordedBy: Aug Busck; sex: Adult Male; **Taxon:** scientificName: Zelus korystos; family: Reduviidae; genus: Zelus; scientificNameAuthorship: Hart, 1987; **Location:** country: TRINIDAD AND TOBAGO; stateProvince: Caroni; locality: Montserrado; verbatimElevation: 101 m; decimalLatitude: 10.41666; decimalLongitude: -61.35; **Identification:** identifiedBy: G. Zhang; dateIdentified: 2012; **Event:** eventDate: 1929-06-01; **Record Level:** institutionCode: USNM
- **Type status:** Paratype. **Occurrence:** catalogNumber: UCR_ENT 00017230; sex: Adult Male; **Taxon:** scientificName: Zelus korystos; family: Reduviidae; genus: Zelus; scientificNameAuthorship: Hart, 1987; **Location:** country: GUYANA; stateProvince: Cuyuni-Mazaruni Region; locality: Bartica , or 'Kartabo-Bartica'; verbatimElevation: 1 m; decimalLatitude: 6.4; decimalLongitude: -58.6166; georeferenceSources: GeoLocate Software; **Identification:** identifiedBy: G. Zhang; dateIdentified: 2012; **Event:** eventDate: 1922-04-08; **Record Level:** institutionCode: AMNH

#### Description

Figs 112, 113, 114

***Male***: (Fig. 112) Medium-sized, total length 10.42–11.55 mm (mean 10.98 mm, Suppl. material 2); slender. **COLORATION**: Head dark brown; inconspicuous yellowish patch between eye and ocellus; medial, yellow stripe on postocular lobe; ventral surface yellowish-brown, lighter than dorsum. Pronotum and scutellum dark brown. Abdomen yellowish-brown. **VESTITURE**: Sparsely setose. Dorsum of head with moderately dense, short, recumbent setae and sparse, short, erect, somewhat spine-like setae; ventral surface with sparse, short, recumbent setae and few moderately long, erect, fine setae. Pronotum with very sparse, short, erect setae over dorsal surface, some setae curved apically, appearing recumbent; moderately dense, short to moderately long, recumbent setae on lateral surface and pleura, intermixed with semi-erect or erect setae. Scutellum with sparse, semi-erect and recumbent setae. Legs with sparse setation on femora and moderately dense setation on tibiae. Corium and clavus with mix of sparse, short, recumbent and erect setae. Abdomen with moderately dense, short, erect setae, intermixed with sparse, long, erect setae. Apical half of dorsal surface with moderately dense, medium-length, semi-erect setae. **STRUCTURE: Head**: Cylindrical, L/W = 2.27. Postocular lobe long; in dorsal view distinctly narrowing through anterior 2/3, posterior 1/3 constant, tube-like. Eye prominent; lateral margin much wider than postocular lobe; dorsal and ventral margins removed from surfaces of head. *Labium*: I: II: III = 1: 2.0: 0.4. Basiflagellomere diameter larger than that of pedicel. **Thorax**: Anterolateral angle bearing small, somewhat acute projection; medial longitudinal sulcus evident throughout, deepening posteriorly. Posterior pronotal lobe with rugulose surface; disc distinctly elevated above humeral angle; humeral angle armed, with spinous processes. Scutellum moderately long; apex angulate, very slightly projected upward. *Legs*: Slender. *Hemelytron*: Slightly surpassing apex of abdomen, not more than length of abdominal segment seven; quadrate cell small, elongate; Cu and M of cubital cell subparallel. **GENITALIA**: (Fig. 113) *Pygophore*: Elongate ovoid; lightly sclerotized expansion below paramere; not expanded laterally in dorsal view. Medial process cylindrical; slender; moderately long; laterally compressed towards apex; anterior surface towards apex ridged; minute spicules on posterior surface; posteriorly directed; curved at middle; apex in posterior view acute, with small hooklike projection. *Paramere*: Cylindrical; moderately long, not reaching medial process; directed posteriad; basally slightly narrower; slightly curved ventrad; apical part not enlarged. *Phallus*: Dorsal phallothecal sclerite somewhat ovoid; sclerotization reduced (yet not absent) on dorsal surface close to posterior margin of foramen; expansion of lateral margin at about mid-portion small; apical portion of phallothecal sclerite gradually tapering, distinctly keeled medially; apex acute; posterior margin of foramen concave. Struts attached to dorsal phallothecal sclerite; apically separate, connected by bridge; basally separate. Basal plate arm robust; basally fused; in lateral view nearly straight, very slightly curved; bridge extremely short; extension of basal plate expanded laterally onto arm, covering more than 1/2 of arm, curved.

***Female***: Unknown.

#### Diagnosis

Recognized by the nearly uniformly dark brown dorsum; the abdomen light-colored, pale yellowish-brown; the posterolateral rim with lightly sclerotized expansion between paramere and medial process; the medial process curved at middle; the anterior surface of the medial process carinate; the apex of the medial process hooklike, the curvature of paramere small; the dorsal phallothecal sclerite with strong carination at apical part, the lateral expansion close to basal arm. Most similar to *Z. filicauda*, but the medial process is shorter and not as strongly curved and the paramere curvature is weaker.

#### Distribution

**Figure 114.**
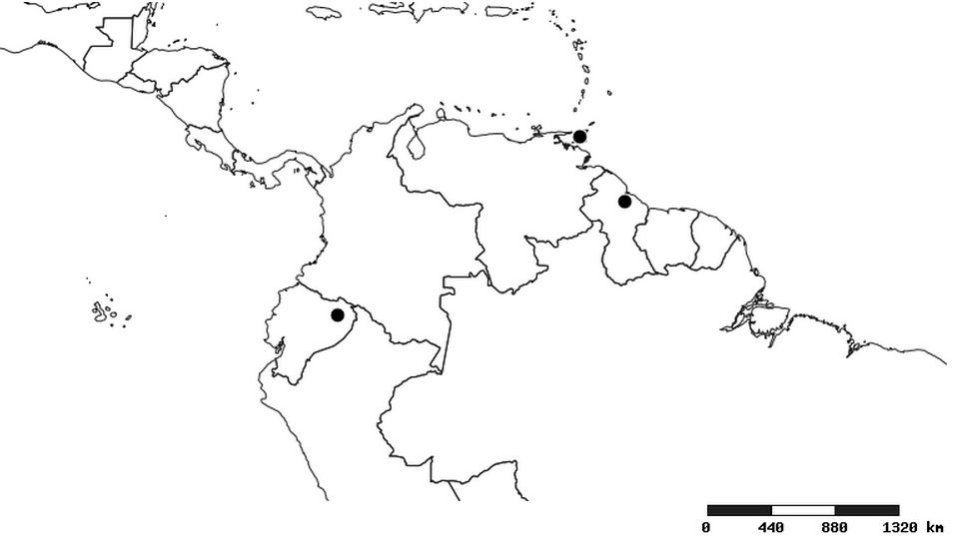
*Zelus korystos* Hart, 1986, specimen records

South America and adjacent islands of the Caribbean (Fig. 114). Countries with records: Ecuador, Guyana and Trinidad and Tobago.

### Zelus laticornis

(Herrich-Schaeffer, 1853)

Euagoras laticornis Herrich-Schaeffer, 1853, p. 123, Tab. CCCIX. Fig. C, orig. descr. and fig.
Zelus laticornis : Stål, 1872, p. 92, cat.; Lethierry and Severin, 1896, p. 152, cat.; Wygodzinsky, l949a, p. 49, checklist; Maldonado, 1990, p. 328, cat.
Darbanus laticornis : Walker, 1873, p. 127, cat.
Zelus formosus Haviland, 1931, p. 137, 151–152, list and orig. descr. (subgenus *Diplodus*); Wygodzinsky, 1949a, p. 49, checklist; Maldonado, 1990, p. 327, cat. **syn. nov.** (current study).
Zelus tristis Haviland, 1931, p. 137, 154, list and orig. descr. (subgenus *Diplodus*); Wygodzinsky, 1949a, p. 50, checklist; Maldonado, 1990, p. 331, cat. **syn. nov.** (current study).

#### Materials

- **Type status:** Other material. **Occurrence:** catalogNumber: UCR_ENT 00048764; occurrenceRemarks: **Holotype** of *Zelus formosus* Haviland, 1931, junior syonym of *Zelus laticornis* (Herrich-Schaeffer, 1853) Verbatim label info: Type / [blue label, no content] / Kartabo, Brit. Guiana August 1922 e coll.M.D. Haviland d.d.Collegium Newnhamense / Pres. by Mrs Brindley. B.M.1928-172. / *Zelus formosus* Haviland / Holotype / *Zelus laticornis* (Herrich-Schaeffer) det. E.R.Hart 1972; recordedBy: M.D. Haviland; sex: Adult Female; **Taxon:** scientificName: Zelus laticornis; family: Reduviidae; genus: Zelus; scientificNameAuthorship: (Herrich-Schaeffer, 1853); **Location:** country: GUYANA; stateProvince: Cuyuni-Mazaruni Region; locality: Kartabo, British Guiana; decimalLatitude: 6.384; decimalLongitude: -58.695; **Identification:** identifiedBy: G. Zhang; dateIdentified: 2012; **Event:** eventDate: 1922-08-01; **Record Level:** institutionCode: BMNH

#### Description

Figs 115, 116, 117

***Male***: (Fig. 115a, b, c, d) Small, total length 9.94–11.44 mm (mean 10.82 mm, Suppl. material 2); slender. **COLORATION**: Head mostly yellowish; some specimens with sub-medial stripes on anteocular lobe; variable brown areas on dorsal surface of postocular lobe, anteriorly broad, narrowing and fusing posteriorly, medially separated by yellowish stripe. Anterior pronotal lobe dark brown; posterior lobe variable, dark brown, orange, or medially and laterally yellowish-brown; pleura brown, mixed with yellow parts. Proportion of dark brown and yellow on posterior pronotal lobe variable, some specimens entirely dark and some entirely yellowish or orange. Scutellum broadly medially yellowish, lateral parts dark brown, some specimens nearly entirely dark or yellowish. Hemelytron uniformly dark brown, corium in some specimens yellowish. Profemur and protibia dark brown, sometimes with single inconspicuous yellowish band; meso and metafemora dark brown, with two or three yellow bands, sometimes basal band rather broad; meso- and meta-tibiae usually dark brown with single yellow band. **VESTITURE**: Moderately setose. Body surface with mostly short, recumbent setae, erect setae sparse. **STRUCTURE: Head**: Cylindrical, L/W = 2.24. Postocular lobe long; in dorsal view anteriorly gradually narrowing, posterior portion constant, slightly narrower. Eye moderately sized; lateral margin much wider than postocular lobe; dorsal and ventral margins removed from surfaces of head. *Labium*: I: II: III = 1: 1.8: 0.5. Basiflagellomere diameter slightly larger than that of pedicel. **Thorax**: Anterolateral angle bearing small projection; medial longitudinal sulcus evident only on posterior 1/2, deepening anterior to transverse sulcus of pronotum. Posterior pronotal lobe with rugulose surface; disc distinctly elevated above humeral angle; humeral angle armed, with dentate projection. Scutellum moderately long; apex angulate, slightly projected upward in some specimens. *Legs*: Moderately robust. *Hemelytron*: Slightly surpassing apex of abdomen, not more than length of abdominal segment seven; quadrate cell large and broad; Cu and M of cubital cell converging towards R. **GENITALIA**: (Fig. 116) *Pygophore*: Ovoid; slightly expanded laterally near base of paramere in dorsal view. Medial process pentagonal; moderately long; anteroposteriorly compressed; erect; straight; apex in posterior view angulate, subapical transverse hooklike bridge. *Paramere*: Cylindrical; short, not reaching apex of medial process; directed posteriad; basally slightly narrower; nearly straight; apical part not enlarged. *Phallus*: Dorsal phallothecal sclerite somewhat squarish; lateral small blade-like heavy sclerotization continuous from basal arm; apical portion of phallothecal sclerite not distinctly tapered, flat; apex truncate; posterior margin of foramen deeply concave. Struts attached to dorsal phallothecal sclerite; apically separate, connected by bridge; basally almost completely fused. Basal plate arm robust; separate; diverging; in lateral view severely curved, nearly semi-circular; bridge long; extension of basal plate expanded onto arm.

***Female***: (Fig. 115e, f) Different from male as outlined below. Larger than male, total length 12.38–14.14 mm (mean 13.67 mm, Suppl. material 2). Head, dorsum of pronotum and corium reddish orange, entirety or portion of posterior pronotal lobe dark brown in some specimens; membrane dark brown; lateral surface of pronotum, pleura and abdomen yellowish, with dark stripes; legs reddish, with dark bands. Hemelytron attaining apex of abdomen.

#### Diagnosis

The strongly convex pronotum distinguishes this species from most other species of the genus. The males can be distinguished by the relatively small size (mean 10.82 mm); the dorsum of the posterior pronotal lobe usually with lighter colored, pale brown, with medial stripe; the broad, pentagonal, apically angulate medial process; the short, blade-like process on dorsal phallothecal sclerite; and the ridge mesad to the blade-like process. In females the head, pronotum and corium are usually orangish-brown to reddish.

#### Distribution

**Figure 117.**
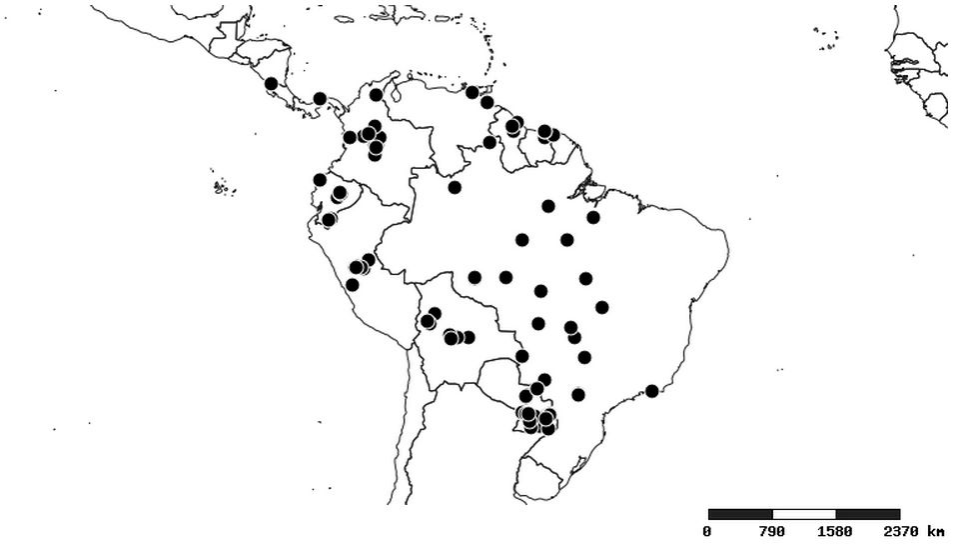
*Zelus laticornis* (Herrich-Schaeffer, 1853), specimen record map

Southern Central America (Panama) and South America (Fig. 117). Countries with records: Argentina, Bolivia, Brazil, Colombia, Ecuador, Guyana, Panama, Paraguay, Peru, Suriname and Venezuela.

#### Taxon discussion

The type material of *Z. laticornis* (under the name *Euagoras laticornis*) was destroyed during World War II. The female holotype of *Zelus formosus* Haviland, 1931 is deposited in the Natural History Museum, London.

### Zelus leucogrammus

(Perty, 1833)

Reduvius leucogrammus Perty, 1834, p. 174, pl. 34, fig. 14, orig. descr. and fig.
Zelus leucogrammus : Stål, 1872, p. 90, cat. (subgenus *Diplodus*); Berg, 1879, p. 152–153, cat., descr. and nymph (subgenus *Diplodus*); Lethierry and Severin, 1896, p. 152, cat.; Costa Lima, 1940, p. 7, 218, 224 illus., biol. notes (subgenus *Diplocodus*); Wygodzinsky, 1949a, p. 49, checklist; Wygodzinsky, 1957, p. 264, list (*Z. leucogrammus* (sic.)); Wygodzinsky, 1960, p. 307, locality; Maldonado, 1990, p. 328, cat.

#### Description

Figs 118, 119, 120

***Male***: (Fig. 118a, b, c, d) Large, total length 15.44–19.55 mm (mean=19.05 mm, Suppl. material 2); robust. **COLORATION**: Reddish and brownish-black. Surface of head primarily reddish, except for around ocellus and lateral stripe on postocular lobe brownish-black, occasional specimens with most of dorsal surface of head brownish-black. Scape and pedicel dark brown; flagellomeres reddish-brown. Dorsal surface of pronotum mostly brownish-black, margins usually reddish, sometimes also with reddish patch at center; lateral surface with mixed red and black. Scutellum red to brownish-black. Hemelytron nearly entirely brownish-black, extreme distal end somewhat reddish. Legs uniformly brownish-black, without bands. Abdomen nearly entirely reddish, sometimes with dark brown patch on connexivum or brownish stripe on posterior margin of segment. Often with whitish wax-like exudation. **VESTITURE**: Sparsely setose. Similar to that in *Z. armillatus*; lacking adpressed setae; some erect setae on dorsum of head and pronotum spine-like. **STRUCTURE: Head**: Cylindrical, L/W = 2.45. Postocular lobe long; in dorsal view anteriorly gradually narrowing, posterior portion constant, slightly narrower. Eye smallish; lateral margin much wider than postocular lobe; dorsal and ventral margins removed from surfaces of head. *Labium*: I: II: III = 1: 1.5: 0.4. Basiflagellomere diameter slightly larger than that of pedicel. **Thorax**: Anterolateral angle bearing small projection; medial longitudinal sulcus shallow near collar, deepening posteriorly. Posterior pronotal lobe with smooth surface; disc distinctly elevated above humeral angle; humeral angle armed, with spinous processes. Scutellum short; apex angulate. *Legs*: Robust. *Hemelytron*: Slightly surpassing apex of abdomen, not more than length of abdominal segment seven; quadrate cell moderately large; Cu and M of cubital cell converging towards R. **GENITALIA**: (Fig. 119) *Pygophore*: Ovoid; slightly expanded laterally near base of paramere in dorsal view; posteriorly expanded sac-like sclerite between parameres and medial process. Medial process cylindrical; slender; moderately long; posteriorly directed; basally slightly protruding; apex in posterior view truncate, with very inconspicuous lateral prongs. *Paramere*: Cylindrical; moderately long, not reaching apex of medial process; directed posteriad; slightly curved dorsad; apical part enlarged. *Phallus*: Dorsal phallothecal sclerite shield-shaped; lateral expansion arising close to base; apical portion of phallothecal sclerite not distinctly tapered, flat, laterally angulate; apex truncate, not emarginate; posterior margin of foramen broadly inversely V-shaped. Struts attached to dorsal phallothecal sclerite; apically separate, connected by bridge; basally separate. Basal plate arm moderately robust; separate; converging; in lateral view slightly curved; bridge short; extension of basal plate expanded onto arm.

***Female***: (Fig. 118e, f) Similar to male, except for the following. Larger than male, total length 21.67–24.62 mm (mean 21.67 mm, Suppl. material 2).

#### Diagnosis

The black dorsal and red ventral coloration is distinctive of this species. Other diagnostic characters include the legs uniformly black and the posterior pronotal lobe with medial depression.

#### Distribution

**Figure 120.**
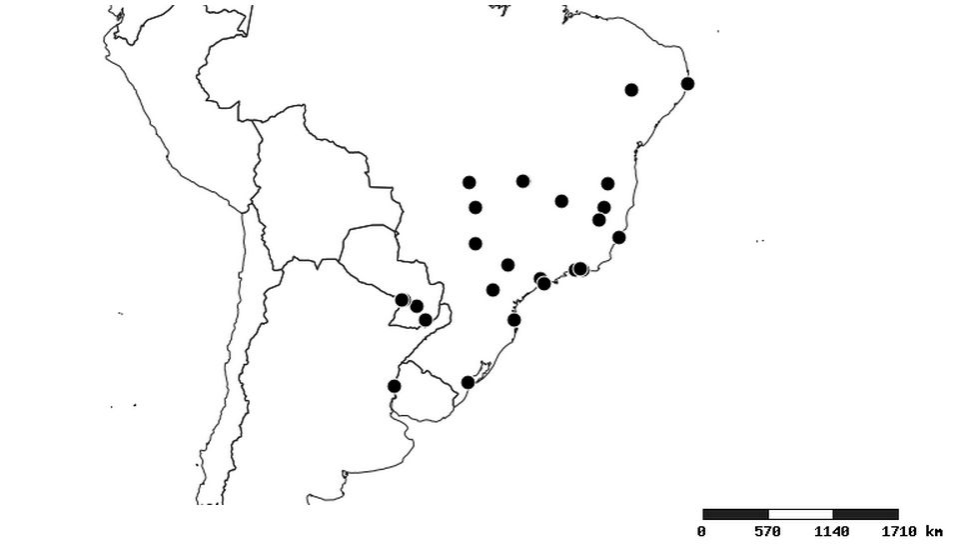
*Zelus leucogrammus* (Perty, 1833), specimen record map

South America (Fig. 120). Countries with records: Argentina, Brazil, Colombia and Paraguay.

#### Taxon discussion

*Zelus leucogrammus* is one of the most distinctive species among *Zelus*. It can be easily recognized the red and black coloration, the medial depression on the posterior pronotal lobe. Variations in coloration are minimal and is usually seen in the size the dark area on the posterior pronotal lobe.

According to Dr. Heinz Wundt at ZSM (pers. comm.), the type material for this species was destroyed during World War II. The original description lists this species from the Amazon River. As this is such a distinctive species, it is not felt that neotype is needed.

### Zelus lewisi

Zhang & Hart sp. n.

urn:lsid:zoobank.org:act:AB969B68-F47C-41D0-BCD5-8CF4F49F7887

#### Materials

- **Type status:** Holotype. **Occurrence:** catalogNumber: UCR_ENT 00014251; occurrenceRemarks: Verbatim coordinates info LN L N 318100 381900. Site number 5367; recordedBy: F. Quesada; sex: Adult Male; otherCatalogNumbers: INBIO CRI002211269; **Taxon:** scientificName: Zelus lewisi; family: Reduviidae; genus: Zelus; scientificNameAuthorship: Zhang and Hart, 2016; **Location:** country: COSTA RICA; stateProvince: Alajuela; locality: Sector San Ramon de Dos Rios; decimalLatitude: 10.16667; decimalLongitude: -84.08333; georeferenceSources: Google Earth; **Identification:** identifiedBy: G. Zhang; dateIdentified: 2013; **Event:** eventDate: 1995-06-26; **Record Level:** institutionCode: INBIO
- **Type status:** Paratype. **Occurrence:** catalogNumber: UCR_ENT 00014250; recordedBy: F. Quesada; sex: Adult Male; **Taxon:** scientificName: Zelus lewisi; family: Reduviidae; genus: Zelus; scientificNameAuthorship: Zhang and Hart, 2016; **Location:** country: COSTA RICA; stateProvince: Alajuela; locality: San Cristobal; decimalLatitude: 10.49557; decimalLongitude: -84.55206; georeferenceSources: Gazetteer; **Identification:** identifiedBy: G. Zhang; dateIdentified: 2013; **Event:** eventDate: 1997-10-01 to 1997-11-01; **Record Level:** institutionCode: INBIO
- **Type status:** Paratype. **Occurrence:** catalogNumber: UCR_ENT 00014261; recordedBy: C. Moraga; sex: Adult Male; **Taxon:** scientificName: Zelus lewisi; family: Reduviidae; genus: Zelus; scientificNameAuthorship: Zhang and Hart, 2016; **Location:** country: COSTA RICA; stateProvince: Guanacaste; locality: Parque Nacional Guanacaste, Finca Aguirrez, Lado N. Volcan Orosi; decimalLatitude: 10.99992; decimalLongitude: -85.46858; georeferenceSources: Google Earth; **Identification:** identifiedBy: G. Zhang; dateIdentified: 2013; **Event:** eventDate: 1994-03-01; **Record Level:** institutionCode: INBIO
- **Type status:** Paratype. **Occurrence:** catalogNumber: UCR_ENT 00014262; recordedBy: C. Moraga; sex: Adult Female; **Taxon:** scientificName: Zelus lewisi; family: Reduviidae; genus: Zelus; scientificNameAuthorship: Zhang and Hart, 2016; **Location:** country: COSTA RICA; stateProvince: Guanacaste; locality: Parque Nacional Guanacaste, Finca Aguirrez, Lado N. Volcan Orosi; decimalLatitude: 10.99992; decimalLongitude: -85.46858; georeferenceSources: Google Earth; **Identification:** identifiedBy: G. Zhang; dateIdentified: 2013; **Event:** eventDate: 1994-03-01; **Record Level:** institutionCode: INBIO
- **Type status:** Paratype. **Occurrence:** catalogNumber: UCR_ENT 00014263; recordedBy: C. Moraga; sex: Adult Female; **Taxon:** scientificName: Zelus lewisi; family: Reduviidae; genus: Zelus; scientificNameAuthorship: Zhang and Hart, 2016; **Location:** country: COSTA RICA; stateProvince: Guanacaste; locality: Parque Nacional Guanacaste, Finca Aguirrez, Lado N. Volcan Orosi; decimalLatitude: 10.99992; decimalLongitude: -85.46858; georeferenceSources: Google Earth; **Identification:** identifiedBy: G. Zhang; dateIdentified: 2013; **Event:** eventDate: 1994-03-01; **Record Level:** institutionCode: INBIO
- **Type status:** Paratype. **Occurrence:** catalogNumber: UCR_ENT 00014264; recordedBy: A. Azofeifa; sex: Adult Female; **Taxon:** scientificName: Zelus lewisi; family: Reduviidae; genus: Zelus; scientificNameAuthorship: Zhang and Hart, 2016; **Location:** country: COSTA RICA; stateProvince: Alajuela; locality: Parque Nacional Volcan Tenorio. Estacion El Pilon; decimalLatitude: 10.6607; decimalLongitude: -84.96272; georeferenceSources: Google Earth; **Identification:** identifiedBy: G. Zhang; dateIdentified: 2013; **Event:** eventDate: 2005-10-20; **Record Level:** institutionCode: INBIO
- **Type status:** Paratype. **Occurrence:** catalogNumber: UCR_ENT 00014265; recordedBy: J. F. Corrales; sex: Adult Female; **Taxon:** scientificName: Zelus lewisi; family: Reduviidae; genus: Zelus; scientificNameAuthorship: Zhang and Hart, 2016; **Location:** country: COSTA RICA; stateProvince: Cartago; locality: Monumento Nacional Guayabo, Turrialba; decimalLatitude: 9.97159; decimalLongitude: -83.69072; georeferenceSources: Google Earth; **Identification:** identifiedBy: G. Zhang; dateIdentified: 2013; **Event:** eventDate: 1994-06-21; **Record Level:** institutionCode: INBIO
- **Type status:** Paratype. **Occurrence:** catalogNumber: UCR_ENT 00014409; recordedBy: C. Moraga; sex: Adult Female; otherCatalogNumbers: INBIO CR1OO2 029708; **Taxon:** scientificName: Zelus lewisi; family: Reduviidae; genus: Zelus; scientificNameAuthorship: Zhang and Hart, 2016; **Location:** country: COSTA RICA; stateProvince: Guanacaste; locality: Est. Pitilla, 9 km S. Santa Cecilia, P.N. Guanacaste, A.C. Guanacaste; decimalLatitude: 10.99261; decimalLongitude: -85.42948; georeferenceSources: Label; **Identification:** identifiedBy: G. Zhang; dateIdentified: 2013; **Event:** eventDate: 1994-08-01; **Record Level:** institutionCode: INBIO
- **Type status:** Paratype. **Occurrence:** catalogNumber: UCR_ENT 00017828; recordedBy: H. A. Hespenheide; sex: Adult Male; **Taxon:** scientificName: Zelus lewisi; family: Reduviidae; genus: Zelus; scientificNameAuthorship: Zhang and Hart, 2016; **Location:** country: PANAMA; stateProvince: Panama; locality: Cerro Campana; decimalLatitude: 8.66666; decimalLongitude: -79.93333; georeferenceSources: Label; **Identification:** identifiedBy: G. Zhang; dateIdentified: 2013; **Event:** eventDate: 1977-07-01; **Record Level:** institutionCode: AMNH
- **Type status:** Paratype. **Occurrence:** catalogNumber: UCR_ENT 00038446; recordedBy: P. A. Opler; sex: Adult Male; **Taxon:** scientificName: Zelus lewisi; family: Reduviidae; genus: Zelus; scientificNameAuthorship: Zhang and Hart, 2016; **Location:** country: COSTA RICA; stateProvince: Heredia; locality: La Selva, 3 km S Puerto Viejo; decimalLatitude: 10.43333; decimalLongitude: -84.01666; georeferenceSources: Label; **Identification:** identifiedBy: G. Zhang; dateIdentified: 2013; **Event:** eventDate: 1973-10-15; **Record Level:** institutionCode: UCB

#### Description

Figs 121, 122, 123

***Male***: (Fig. 121a, b) Large, slender, total length 18.71–20.15 mm (mean 19.20 mm, Suppl. material 2); slender. **COLORATION**: Entire surface blackish-brown. Pleura, abdomen venter and sometimes scutellum with whitish markings. Antenna brown; scape with three dark annulations. Profemur brown, with dark medial and subapical rings; meso- and metafemora yellowish-brown, dark spot on base outer/anterior surface, and dark brown rings medially and subapically. **VESTITURE**: Sparsely setose. Similar to that in *Z. armillatus*, less dense. **STRUCTURE: Head**: Cylindrical, L/W = 2.36. Postocular lobe very long; in dorsal view distinctly narrowing through anterior 2/3, posterior 1/3 constant, tube-like. Eye moderately sized; lateral margin much wider than postocular lobe; dorsal and ventral margins removed from surfaces of head. *Labium*: I: II: III = 1: 1.1: 0.3. Basiflagellomere diameter very slightly larger than that of pedicel. **Thorax**: Anterolateral angle bears small protuberance; medial longitudinal sulcus distinct throughout, deepened posteriorly. Posterior pronotal lobe finely rugulose; disc distinctly elevated above humeral angle, bears two small tubercles; humeral angle armed, with spinous processes. Scutellum moderately long; apex angulate. *Legs*: Very slender. *Hemelytron*: Surpassing apex of abdomen by about length of abdominal segment seven; quadrate cell small, relatively broad; Cu and M of cubital cell subparallel. **GENITALIA**: (Fig. 122) *Pygophore*: Ovoid; not expanded laterally in dorsal view; broad, weakly sclerotized expansion between paramere and medial process. Medial process short; semi-erect; apex folded ventrad, with small sharp lateral projections. *Paramere*: Cylindrical; very long, exceeding apex of medial process; apical part not expanded. *Phallus*: Dorsal phallothecal sclerite rectangular; apical portion of phallothecal sclerite not distinctly tapered, surface flat, apex rounded, not emarginate; phallothecal sclerite laterally with wrinkles; posterior margin of foramen broadly inversely V-shaped. Struts attached to dorsal phallothecal sclerite; apically separate, not connected by bridge; basally separate. Basal plate arm moderately robust; separate; converging; in lateral view slightly curved; bridge short; extension of basal plate expanded onto arm.

***Female***: (Fig. 121c, d, e, f) Larger than male, total length 22.52–24.06 mm (mean 23.29 mm, Suppl. material 2). Coloration variable; yellowish or reddish with dark spots or markings.

#### Diagnosis

Recognized by the large and slender body and the posterior pronotal lobe bearing a pair of tubercles. Males can be easily recognized by the black coloration with white markings on scutellum and abdomen and females yellowish or reddish with black spots and markings. Among males of the *Zelus armillatus* group (Fig. 8), the paramere of *Z. annulosus* is more than 2x longer than the medial process. *Zelus amblycephalus* and *Z. annulosus* also have long parameres, but these are apically curved, whereas it is straight in *Z. annulosus*.

#### Etymology

The specific epithet is a patronym, named after Dr. James Lewis, in honor of his contribution to the curation of Heteroptera of Costa Rica at INBio. Without his and his fellow scientists' work the discovery of this species would not have been possible.

#### Distribution

**Figure 123.**
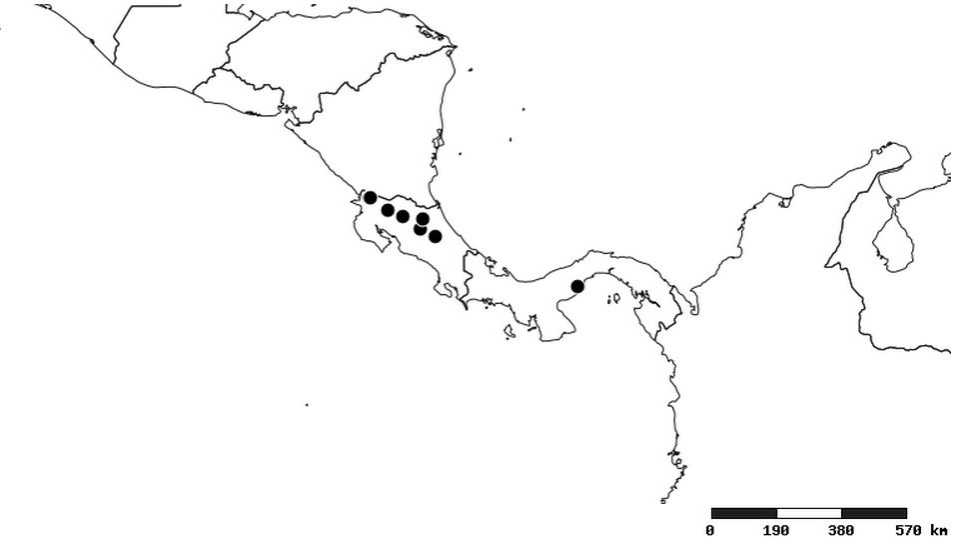
*Zelus lewisi* Zhang & Hart, sp. n., specimen record map

Central America (Fig. 123). Countries with records: Costa Rica and Panama.

### Zelus litigiosus

Stål, 1862

Zelus litigiosus Stål, 1862, p. 453, orig. descr.; Stål, 1872, p. 90, cat. (subgenus *Diplodus*); Lethierry and Severin, 1896, p. 152, cat.; Champion, 1898, p. 257, Tab. XV. fig. 20, 20a, fig. and junior syn. of *Z. janus*; Maldonado, 1990, p. 328, cat.
Diplodus litigiosus : Walker, 1873, p. 124, cat; Uhler, 1886, p. 24, checklist.

#### Materials

- **Type status:** Lectotype. **Occurrence:** catalogNumber: UCR_ENT 00041006; occurrenceRemarks: Lectotype of *Zelus litigiosus* Stål, 1862 (**New Designation** by Zhang, Hart & Weirauch, 2016) Verbatim label info: Mexico / Salle / litigiosus Stal / Lectotype Zelus litigiosus Stal / designated by E.R.Hart / Typus / NHRS-GULI 000000332; recordedBy: salle; sex: Adult Female; **Taxon:** scientificName: Zelus litigiosus; family: Reduviidae; genus: Zelus; scientificNameAuthorship: Stål, 1862; **Location:** country: MEXICO; **Identification:** identifiedBy: G. Zhang; dateIdentified: 2012; **Record Level:** institutionCode: NHRS
- **Type status:** Paralectotype. **Occurrence:** occurrenceRemarks: Paralectotype of *Zelus litigiosus* Stål, 1862 (**New Designation** by Zhang, Hart & Weirauch, 2016) Verbatim label info: Mexico / Salle / litigiosus Stal / Paralectotype *Zelus litigiosus* Stal / designated by E.R.Hart; recordedBy: salle; sex: Adult Female; **Taxon:** scientificName: Zelus litigiosus; family: Reduviidae; genus: Zelus; scientificNameAuthorship: Stål, 1862; **Location:** country: MEXICO; **Identification:** identifiedBy: G. Zhang; **Record Level:** institutionCode: NHRS

#### Description

Figs 124, 125, 126

***Male***: (Fig. 124a, b, c, d) Large, total length 17.10–18.80 mm (mean 18.15 mm, Suppl. material 2); robust. **COLORATION**: Brown, brownish-black, sometimes with orange or red. Dorsal and lateral surfaces of head usually brownish-black, ventral surface yellowish-brown; variable amount of yellowish-brown on anteocular lobe; yellowish-brown patch usually between eye and ocellus and medially on postocular lobe. Scape and pedicel with yellow and black bands. Areas of setal tracts on anterior pronotal lobe lighter than glabrous surface, difference subtle in some specimens. Posterior lobe usually uniformly brown, orange or red; lateral surfaces lighter in some specimens. Scutellum dark brown. Corium and clavus usually uniform, orange, brown or dark brown; some specimens with distal part lighter; membrane dark brown. Legs usually yellowish-brown with black bands, usually one on tibiae and two or three on femora; completely black in some dark specimens. **VESTITURE**: Densely setose. Head with both recumbent and erect setae dorsally, and predominantly short, recumbent setae ventrally. Anterior pronotal lobe with long erect setae, mainly occupying setal tracts; posterior pronotal lobe with fine, erect setae. Abdomen with short, recumbent setae, interspersed with long, erect setae. **STRUCTURE: Head**: Cylindrical, L/W = 2.25. Postocular lobe long; in dorsal view anteriorly gradually narrowing, posterior portion constant, slightly narrower. Eye smallish; lateral margin only slightly wider than postocular lobe; dorsal margin removed from postocular transverse groove, ventral margin attaining ventral surface of head. *Labium*: I: II: III = 1: 1.4: 0.4. Basiflagellomere diameter subequal to that of pedicel. **Thorax**: Anterolateral angle bearing small protuberance; medial longitudinal sulcus evident throughout, deepening posteriorly. Posterior pronotal lobe with rugulose surface; disc distinctly elevated above humeral angle; humeral angle armed, with short tuberculate process. Scutellum moderately long; apex angulate. *Legs*: Slender. *Hemelytron*: Slightly surpassing apex of abdomen, not more than length of abdominal segment seven; quadrate cell large and broad; Cu and M of cubital cell converging towards R. **GENITALIA**: (Fig. 125) *Pygophore*: Rounded; slightly expanded laterally near base of paramere in dorsal view; posteriorly expanded sac-like sclerite between paramere and medial process. Medial process cylindrical; slender; moderately long; posteriorly directed; straight; apex in posterior view rounded, with very inconspicuous lateral prongs. *Paramere*: Cylindrical; moderately long, not reaching apex of medial process; directed posteriad; nearly straight; apical part not enlarged. *Phallus*: Dorsal phallothecal sclerite somewhat squarish; sharp laterally oriented process close to posterior margin of foramen and basal arms; apical portion of phallothecal sclerite not distinctly tapered, flat, lateral margin narrowly angulate; apex rounded; posterior margin of foramen broadly concave, medially deeper. Struts attached to dorsal phallothecal sclerite; apically fused; basally mostly separate, moderately fused. Basal plate arm moderately robust; separate; diverging; in lateral view very slightly curved; bridge short; extension of basal plate small and confined to apex of basal plate arm.

***Female***: (Fig. 124e, f) Similar to male, except for the following. Larger than male, total length 19.75–21.95mm (mean 20.88 mm, Suppl. material 2). Dorsal surface never entirely brownish-black.

#### Diagnosis

Among species in the *Zelus armillatus* species group occurring in overlapping geographical regions, *Zelus litigiosus* can be easily distinguished from *Z. janus* by the elevated disc of the posterior pronotal lobe. It can be separated from *Z. sulcicollis* by the flat or slightly convex disc of the posterior pronotal lobe, and that being depressed in *Z. sulcicollis*.

#### Distribution

**Figure 126.**
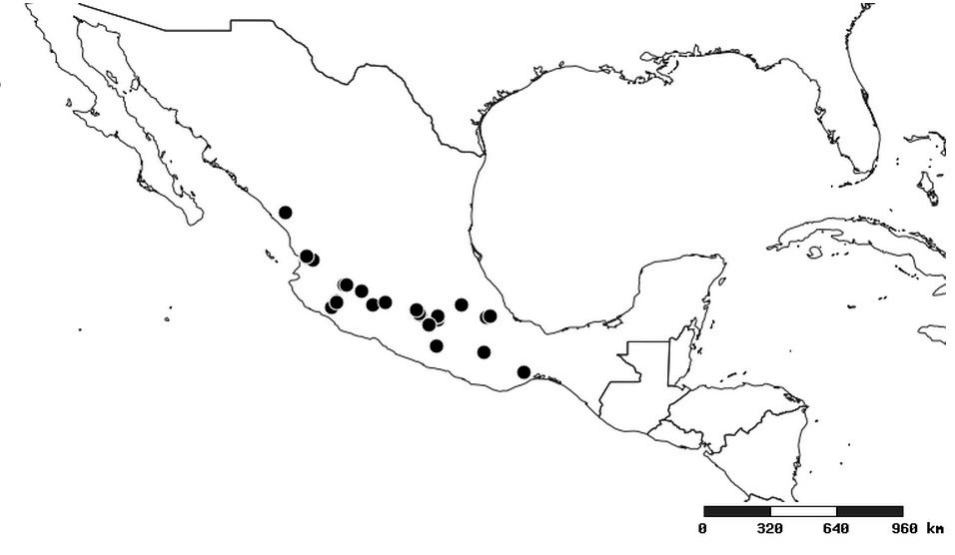
*Zelus litigiosus* Stål, 1872, specimen record map

Southwestern Mexico (Fig. 126).

### Zelus longipes

(L., 1767)

Cimex longipes Linnaeus, 1767, p. 724, orig. descr.; Gmelin, 1788, p. 2197, list (*Reduvius*); Turton, 1806, p. 690, descr.
Reduvius longipes : Fabricius, 1775, p. 730, descr.; Fabricius, 1781, p. 378, descr.; Fabricius, 1787, p. 309, list; Fabricius, 1794, p. 196, descr.
Zelus longipes : Fabricius, 1803, p. 283, descr.; Stål, 1872, p. 88-89, cat. (subgenus *Zelus*); Blanchard, 1840, p. 101, descr.; Stål, 1862, p. 449-450, descr.; Uhler, 1878, p. 427, list; Uhler, 1886, p, 24, checklist; Lethierry and Severin, 1896, p. 152, cat.; Champion, 1898, p. 253, note; Kirkaldy, 1900a, p. 263, note; Fracker, 1913, p. 239, 240, key and list (subgenus *Zelus*); Cotton, 1917, p. 170-173, note; Barber, 1923, p. 27-28, note and syn.; Wygodzinsky, 1949a, p. 49, checklist; Wolcott, 1950 (1948), p. 212, list and note; Elkins, 1951, p. 410, list; Guagliumi, 1953, p. 16, note; Barber, 1954, p. 13-14, list; Elkins, 1954, p. 44, 45, note and fig.; Simmonds, 1956, p. 232, note; Alayo, 1967, p. 5, 36-37, list and note; Hart, 1986, p. 543-546, redescription, note, fig. and key; Hart, 1987, p. 304, note and key; Maldonado, 1990, p. 328, cat.
Euagoras longipes : Walker, 1873, p. 117-118, cat.
Reduvius rubidus Lepeletier and Serville, 1825, p. 278, orig. descr,; Guerin-Meneville, 1857, p. 411-412, descr. and list (subgenus *Evagoras*).
Evagoras rubidus : Amyot and Serville, 1843, p. 368-369, descr. and senior syn. of *Evagoras speciosus* Burmeister. Stål, 1862, p. 449, junior syn. (in part) of *Z. longipes*. Walker, 1873, p. 117, junior syn. of *Euagoras longipes*.
Euagoras rubidus : Walker, 1873, p. 118, cat.
Zelus rubidus : Stål, 1872, p. 89, cat. and descr. (subgenus *Zelus*); Uhler, 1886, p. 24, checklist; Lethierry and Severin, 1896, p. 153, cat.; Champion, 1898, p. 252-253, cat. and note; Fracker, 1913, p. 238, 240, key and list (subgenus *Zelus*); Ballou, 1913, p. 65, note; Jones, 1914, p. 462, note; Osborne and Drake, 1915, p. 531, list; Cotton, 1917, p. 173, note; Ritchie, 1917, p. 94, note; Gibson, 1919, p. 276, list; Dash, 1920, p. 31, note; Barber, 1923, p. 27, junior syn. of *Z. longipes*; Bruner, 1926, p. 78, descr.; Gowdey, 1927, p. 16-17, note; Martorell, 1939, p. 189, list; Wygodzinsky, 1949a, p. 49, checklist and junior syn. of *Z. longipes*; Alayo, 1967, p. 36-37, note.
Reduvius phalangium Fabricius, 1794, p. 1966, orig. descr.; Zirnsen, 1964, p. 338, list; Hart, 1986, p. 543, junior syn. of *Z. longipes*.
Zelus phalangium : Fabricius, 1803, p. 283, descr.; Stål, 1872, p. 92, cat.; Lethierry and Severin, 1896, p. 153, cat.; Fracker, 1913, p. 240, descr. and list; Wygodzinsky, 1949a, p. 50, checklist.
Diplodus phalangium : Uhler, 1886, p. 24, checklist.
Zelus bilobus Say, 1832, p. 12, orig. descr.; LeConte, 1859, p. 306, descr.; Stål, 1862, p. 449, list (as variety of *Z. longipes*); Stål, 1872, p. 88, cat. (subgenus *Zelus*); Uhler,1876, p. 61, list; Uhler, 1886, p. 24, checklist; Lethierry and Severin, 1896, p. 151, cat.; Champion, 1898, p. 253, note; Van Duzee, 1909, p. 176, list; Torre-Bueno and Engelhardt, 1910, p. 150, list; Fracker, 1913, p. 239, 240, key and list (subgenus *Zelus*); Barber, 1914, p. 505, list; Van Duzee, 1916, p. 30, checklist (s .g. *Zelus*); Dozier, 1917, p. 542, note; Van Duzee, 1917, p. 259, cat. (subgenus *Zelus*); Dozier, 1920, p. 357, note; Blatchley, 1926, p. 568, 569, key and descr. (subgenus *Zelus*); Readio, 1927, p. 169-170, key, descr. and note; Miller, 1929, p. 462, note; Creighton 1936a p. 94, note; Creighton, 1936b, p. 382, note; Elliott , 1938, p. 39, key and list; Wygodzinsky, 1949a, p. 48, checklist; Elkins, 1951, p. 410, list; Sibley, 1951., p. 92, list ; Oliver, 1964, p. 316, note; Whitcomb and Bell , 1964, p. 22, list; Davis, 1969, p. 81, fig. and note (sic. *Zellus bilobatus*); Hart, 1986, p. 543, junior syn. of *Z. longipes*.
Euagoras speciosus Burmeister, 1835, p. 227, orig. descr.; Herrich-Schaeffer, 1848, p. 45, Tab. CCLXIV. fig. 817, descr. and fig; Hart, 1986, p. 543, junior syn. of *Z. longipes*.
Evagoras speciosus : Amyot and Serville, 1843, p. 368, junior syn. of *Evagoras rubidus* Le P. and Serv.
Zelus speciosus : Stål, 1862, p. 449, syn. (as variety of *Z. longipes*) Stål, 1872, p. 89, cat. (subgenus *Zelus)*; Berg, 1879, p. 151, note; Uhler, 1886, p. 24, checklist; Lethierry and Severin, 1896, p. 153, cat.;, Kirkaldy, 1909, p. 32, list and syn.; Wygodzinsky, 1949a, p. 50, checklist.
Zelus speciosus var. stolli Lethierry and Severin, 1896, p. 152, *nomen nudum*; Champion, 1898, p. 253, syn. (=*Zelus rubidus*). *Euagoras tricolor* Herrich-Schaeffer, 1848, p. 45-46, Tab. CCLXIV, fig. 818, orig. descr. and fig.; Stål, 1862, p. 450, syn. (as variety of *Zelus longipes*); Stål, 1872, p. 89, syn. (as variety *of Zelus speciosus*); Champion, 1898, p. 253, junior syn. of *Zelus rubidus*; Fracker and Bruner, 1924, p. 170, list; Bruner, 1926, p. 79, descr; Wygodzinsky, 1949a, p. 50, junior syn. of *Zelus speciosus*.
Zelus mactans Stål, 1861, p. 148, orig. descr.; Stål, 1872, p. 88, cat. (subgenus *Zelus*); Uhler, 1886, p. 24, checklist; Lethierry and Severin, 1896, p. 152, cat.; Fracker, 1913, p. 239, 240, key and list (subgenus *Zelus*); Barber, 1923, p. 28, note; Wygodzinsky, 1949a, p. 49, checklist; Alayo, 1967, p. 36, key and note; Hart, 1986, p. 544, lectotype desig. and junior syn. of *Z. longipes*.
Diplodus mactans : Walker, 1873, p. 125, cat.
Velia agavis Blasguez, 1870, p. 289, 290, fig. 14, orig. descr.; Champion, 1898, p. 253, junior syn. of *Z. rubidus*; Kirkaldy, 1909, p. 32, junior syn. (as variety of *Z. speciosus*); Fracker, 1913, p. 240, junior syn. of *Z. rubidus*.

#### Materials

- **Type status:** Holotype. **Occurrence:** occurrenceRemarks: Bears the following labels: longipes / 65; sex: Adult Female; **Taxon:** scientificName: Zelus longipes; family: Reduviidae; genus: Zelus; scientificNameAuthorship: (L., 1767); **Location:** country: St. Thomas; **Record Level:** institutionCode: Linnaean Society, London
- **Type status:** Other material. **Occurrence:** occurrenceRemarks: **Lectotype** of *Zelus mactans* Stal, 1861 (designated by Hart, 1986), junior synonym of *Zelus longipes* (Linnaeus, 1767). Bears the following labels: Cuba / Stal / mactans Stal / Typus.; sex: Adult Male; **Taxon:** scientificName: Zelus longipes; family: Reduviidae; genus: Zelus; scientificNameAuthorship: (L., 1767); **Location:** country: CUBA; **Record Level:** institutionCode: NHRS
- **Type status:** Other material. **Occurrence:** occurrenceRemarks: **Allolectotype** of *Zelus mactans* Fabricius, 1803 (designated by Hart, 1986), junior synonym of *Zelus mactans* Stal, 1861, junior synonym of *Zelus longipes* (Linnaeus, 1767). Bears the following labels: Cuba / Stal / Stal / Paratypus.; sex: Adult Female; **Taxon:** scientificName: Zelus longipes; family: Reduviidae; genus: Zelus; scientificNameAuthorship: (L., 1767); **Location:** country: CUBA; **Record Level:** institutionCode: NHRS

#### Description

Figs 127, 128, 129

***Male***: (Fig. 127a, b, c, d) Medium-sized, total length 13.62–17.91 mm (15.77 mm, Suppl. material 2); slender. **COLORATION**: Orangish red and brownish-black; pattern variable; most of dorsal surface brownish-black; orangish red usually on head, part or entire anterior pronotal lobe, lateral margins of posterior pronotal lobe, proximal and distal parts of corium; occasional specimens with nearly completely dark dorsum. Ventral and lateral surfaces usually orangish red; black stripe along anterior margin of abdominal segment, lacking in some specimens, sometimes also whitish exudation next to black stripe and on pleura and lateral surface of pronotum. Scape, pedicel and legs with or without bands. **VESTITURE**: Densely setose. Anteocular lobe with short to moderate, erect setae; postocular lobe with short to long, erect setae. Anterior pronotal lobe with short to long, erect setae, confined to setal tracts dorsally. Abdomen with short to moderately long, erect setae. **STRUCTURE: Head**: Cylindrical, L/W = 2.52. Postocular lobe moderately long; in dorsal view anteriorly gradually narrowing, posterior portion constant, slightly narrower. Eye smallish; lateral margin only slightly wider than postocular lobe; dorsal and ventral margins removed from surfaces of head. *Labium*: I: II: III = 1: 1.5: 0.3. Basiflagellomere diameter larger than that of pedicel. **Thorax**: Anterolateral angle rounded, without projection; medial longitudinal sulcus evident throughout, deepening posteriorly. Posterior pronotal lobe with finely rugulose surface; disc distinctly elevated above humeral angle; humeral angle rounded, without projection. Scutellum moderately long; apex blunt, not projected. *Legs*: Slender. *Hemelytron*: Surpassing apex of abdomen by about twice length of abdominal segment seven; quadrate cell large and broad; Cu and M of cubital cell subparallel. **GENITALIA**: (Fig. 128) *Pygophore*: Rounded; mid-lateral fold adjacent to paramere insertion; not expanded laterally in dorsal view. Medial process cylindrical; slender; moderately long; semi-erect; nearly straight; apex in posterior view rounded, slightly folded posteriad. *Paramere*: Cylindrical; long, surpassing medial process; directed posteriad; nearly straight; apical part not enlarged. *Phallus*: Dorsal phallothecal sclerite somewhat squarish; lateral expansion arising close to base; apical portion of phallothecal sclerite not distinctly tapered, slightly convex, laterally angulate; apex truncate, medially emarginate; posterior margin of foramen broadly concave. Struts attached to dorsal phallothecal sclerite; apically separate, not connected by bridge; basally separate throughout. Basal plate arm moderately robust; separate; diverging; in lateral view very slightly curved; bridge long; extension of basal plate small, laterally expanded onto arm.

***Female***: (Fig. 127e, f) Similar to male, except for the following. Larger than male, total length 15.19–18.36 mm (mean 17.39 mm, Suppl. material 2).

#### Diagnosis

Although highly variable, the black and red coloration is distinctive of *Z. longipes*. The combination of size, coloration, rounded humeral angle, and raised anterior pronotal lobe serves to separate this species from any others that may cause confusions. Males can also be recognized by the long and slender medial process, the apex slightly folded posteriad, and the long paramere, exceeding apex of medial process. Among the *Zelus longipes* species group (Fig. 10), *Z. bahiaensis* also has a long paramere clear exceedingly medial process, but the two species can be readily separated on the basis of coloration.

#### Distribution

**Figure 129.**
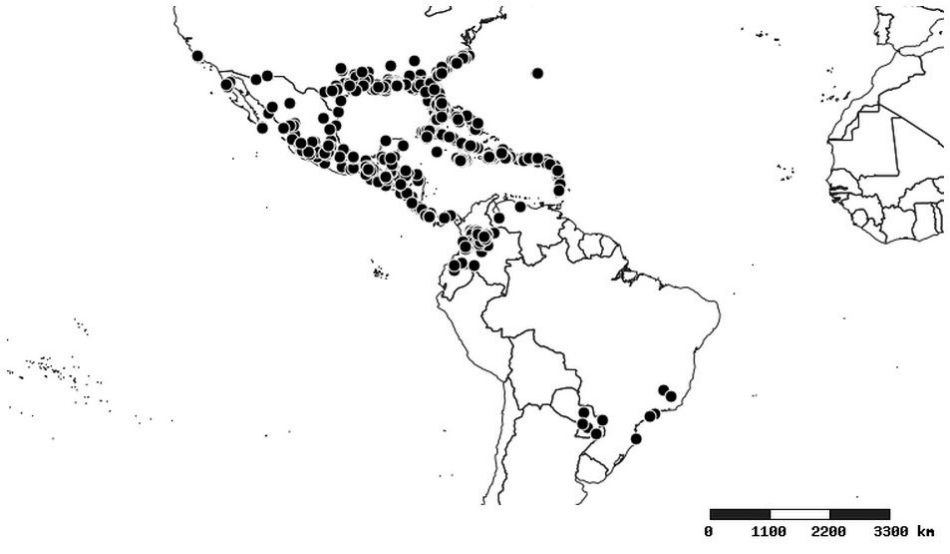
*Zelus longipes* (L., 1767), specimen record map

Southern parts of US, Mexico, Central America, the Caribbean, Northern South America, Paraguay and Southern Brazil (Fig. 129). Countries with records: Antigua and Barbuda, Bahamas, Belize, Cayman Islands, Colombia, Cuba, Dominica, Dominican Republic, El Salvador, Guadeloupe, Guatemala, Haiti, Honduras, Jamaica, Mexico, Nicaragua, Panama, Paraguay, Puerto Rico, Saba, Saint Kitts and Nevis, Trinidad and Tobago, USA, Venezuela, Virgin Islands (British) and Virgin Islands (US).

#### Taxon discussion

*Zelus longipes* is a highly variable species. In any given area there is a wide range of color and color pattern variations. The dorsal coloration can vary from nearly entirely orange brown, through various patterns of orange-brown and brownish-black, to almost completely black. The habitus images provided here only represent a subset of the range of variations. The most typical form is one with alternating orange and black areas on the dorsum, abdominal venter orange or reddish-brown, each segment black anteriorly, and legs black with two yellow rings medially. Hart (1986) discussed intraspecific variations and the history and uses of several synonyms.

### Zelus luridus

Stål, 1862

Zelus luridus Stål, 1862, p. 452, orig. descr.; Stål, 1872, p. 91, cat. (subgenus *Diplodus*) Lethierry and Severin, 1896, p. 152, cat.; Champion, 1898, p. 259–260, junior syn. of *Z. exsanguis*; Uhler, 1904, p. 364, list; Wirtner, 1904, p. 206, list; Torre-Bueno and Brimley, 1907, p. 437, list; Torre-Bueno, 1910, p. 32, note; Torre-Bueno and Engelhardt, 1910, p. 150, note; Torre-Bueno, 1913, p. 60, note (subgenus *Diplodus*); Barger, 1914, p. 506, list; Van Duzee, 1916, p. 30, junior syn. of *Z. exsanguis*; Hart, 1986, p. 537, redescription, lectotype desig, note, key, fig. and stat. rev.; Maldonado, 1990, p. 329, cat.
Diplodus luridus : Uhler, 1872a, p. 471, checklist; Uhler, 187213, p. 420, note; Walker, 1873, p. 124, cat.; Uhler, 1876, p. 61, note; Uhler, 1877, p. 429, note; Uhler, 1878, p. 427, note; Uhler, 1886, p. 24, checklist; Provancher, 1887, p. 181, note; Van Duzee, 1894, p. 183, list; Gillette and Baker, 1895, p. 60, list.
Diplocodus luridus : Van Duzee, 1912, p. 324, note.
Darbanus georgiae Provancher, 1872, p. 106, orig. descr.; Uhler, 1886, p. 24, checklist; Provancher, 1887, p. 181, note; Kelton, 1968, p. 1070, note; Hart, 1986, p. 53, junior syn. of *Z. luridus*.
Zelus georgiae : Lethierry and Severin, 1896, p. 152, cat.; Banks, 1910, p. 16, cat.; Van Duzee, 1916, p. 30, junior syn. of *Z. exsanguis*.
Zelus acanthogonius Say & Uhler, 1878, p. 427, manuscript name.
Evagoras viridis Uhler, 1878, p. 427, manuscript name
Darbanus palliatus Provancher, 1887, p. 182, orig. descr.; Kelton, 1968, 1070, note; Hart, 1986, p. 53, junior syn. of *Z. luridus*.
Zelus palliatus : Lethierry and Severin, 1896, p. 153, cat.; Banks, 1910, p. 16, cat.; Van Duzee, 1916, p. 30, junior syn. of *Z. exsanguis*.
Reduvius sp. Emmons, 1854, p. 168, P1. 7, fig. 3, note and fig.
Zelus exsanguis [misapplication of name due to Champion's synonymy of *Z. luridus* under *Z. exsanguis*]: Wirtner, 1904, p. 206, list; Barber, 1906, p. 285, list; Snow, 1907, p. 159, list; Parshley, 1914, p. 144, list; Hussey, 1922, p. 24, list; Britton, 1923, p. 687, list; Blatchley, 1926, p. 570–571, descr. and note (*Diplodus*); Readio, 1926, 167, P1. X, fig. 5, 6 and 7, note and fig.; Readio, 1927, p. 169, 171–177, P1. XIII, descr., notes and fig.; Leonard, 1928, p. 105, list; Brimley, 1938, p. 73, list; Harris, 1943, p. 151, list; Procter, 1946, p. 319, list; Elkins, 1951, p. 410, list; Sibley, 1951, p. 92, list; Hall, Downe, MacLellan and West, 1953; p. 199–204, note; Dome and West, 1954, p. 181–184, behavior; West and De Long, 1955, p. 97–101, biology; Davis, 1961, p. 351, note; Drew and Schaeffer, 1962, p. 106, list; Whitcomb and Bell, 1964, p. 22, list; Kelton, 1968, p. 1070– 1071, note; Yonke and Medler, 1970, p. 441–443, biology.

#### Materials

- **Type status:** Lectotype. **Occurrence:** catalogNumber: UCR_ENT 00041007; occurrenceRemarks: Lectotype of *Zelus luridus* Stål, 1862 (designated by Hart, 1986). Verbatim label info: Germar / Carolina / luridus Stal / Lectotype *Zelus luridus* Stal / designated by E.R.Hart / Typus / NHRS-GULI 000000333; recordedBy: Stal; sex: Adult Female; otherCatalogNumbers: NHRS-GULI 000000333; **Taxon:** scientificName: Zelus luridus; family: Reduviidae; genus: Zelus; scientificNameAuthorship: Stål, 1862; **Location:** country: USA; stateProvince: North Carolina; county: unknown; locality: unknown; **Identification:** identifiedBy: G. Zhang; dateIdentified: 2012; **Event:** eventDate: No date provided; **Record Level:** institutionCode: NHRS
- **Type status:** Paralectotype. **Occurrence:** catalogNumber: UCR_ENT 00041007; occurrenceRemarks: Paralectotype of *Zelus luridus* Stål, 1862 (Designated by Hart, 1986); recordedBy: Stal; sex: Adult Female; **Taxon:** scientificName: Zelus luridus; family: Reduviidae; genus: Zelus; scientificNameAuthorship: Stål, 1862; **Location:** country: USA; stateProvince: North Carolina; county: unknown; locality: unknown; **Identification:** identifiedBy: G. Zhang; dateIdentified: 2012; **Event:** eventDate: No date provided; **Record Level:** institutionCode: NHRS
- **Type status:** Other material. **Occurrence:** occurrenceRemarks: **Holotype** of *Darbanus georgiae* Provancher, 1872, junior synonym of *Zelus luridus* Stål, 1862. Bears the following label: No. 114 *Darbanus georgiae* Prov. *Zelus exsanguis* (Stal).; sex: Adult Male; **Taxon:** scientificName: Zelus luridus; family: Reduviidae; genus: Zelus; scientificNameAuthorship: Stål, 1862; **Location:** country: USA; stateProvince: Georgia; locality: unknown; **Event:** eventDate: Macon
- **Type status:** Other material. **Occurrence:** occurrenceRemarks: **Holotype** of *Darbanus palliatus* Provancher, junior synonym of *Zelus luridus* Stål, 1862. Bears the following labels: No. 247, *Darbanus palliatus* Prov. *Zelus exsanguis* (Stal); sex: Adult Male; **Taxon:** scientificName: Zelus luridus; family: Reduviidae; genus: Zelus; scientificNameAuthorship: Stål, 1862; **Location:** country: CANADA; stateProvince: Ontario; **Event:** eventDate: Ottawa

#### Description

Figs 130, 131, 132

***Male***: (Fig. 130a, b) Medium-sized, total length 13.21–15.27 mm (mean 13.89 mm, Suppl. material 2), slender. **COLORATION**: Yellowish-brown through reddish-brown to brownish-black, colors more vibrant in live individuals. Anteocular lobe uniformly yellowish-brown to reddish-brown or with dorsum variably reddish-brown to brownish-black with lighter lateral and ventral surfaces. Postocular lobe reddish-brown to brownish-black dorsally and laterally with mid-dorsal and occasionally circumocellar areas yellowish-brown, ventral surface yellowish-brown. Rostrum uniformly yellowish-brown to light reddish-brown. Antennae light reddish-brown, base and apex of scape and pedicel sometimes slightly darker than shaft. Anterior pronotal lobe yellowish-brown to brownish-black, light specimens usually with darker coloration on anterior portion of medial longitudinal sulcus and/or with setal tracts darker, many darker specimens with lateral margins and/or collar yellowish-brown, remainder of surface yellowish-brown. Dorsum of posterior lobe reddish-brown to brownish-black, lateral margins and usually posterior margins yellowish-brown, lateral processes of lighter specimens usually brownish-black, remainder of surface yellowish-brown. Scutellum reddish-brown to brownish-black with apex and sometimes posterior mid-dorsal surface yellowish-brown. Femora yellowish-brown to light reddish-brown, usually with dark bands or dorsal markings near apices, tibiae yellowish-brown to dark reddish-brown, usually slightly darker than femora. Hemelytron yellowish-brown to reddish-brown, some darker specimens with lighter veins in clavus and corium and/or lighter area along costal margin. Dorsum of abdomen reddish-brown, lateral and ventral surfaces yellowish-brown to light reddish-brown. **VESTITURE**: Moderately setose. Anteocular lobe with short, recumbent and erect setae dorsally, erect setae predominating on vertex, sparse short erect and semi-erect setae on ventral half. Postocular lobe with mostly recumbent, some erect setae dorsally; lateroventral and ventral surfaces with moderate to long erect and scattered recumbent setae. Anterior pronotal lobe with short erect and recumbent setae over surface, confined to setal tracts dorsally, long erect setae laterally. Dorsum of posterior lobe with short recumbent setae, some short recumbent setae laterally, long erect setae lateroventrally. Lateral surface of scutellum with moderate to long erect setae, elevated dorsal surface nearly bare. Clavus and corium with inconspicuous short erect and recumbent setae. Abdomen dorsally with short, sparse, erect setae, remainder of surface with short erect and recumbent setae and some scattered longer erect setae. Exposed area of pygophore with short recument and short to long erect setae. **STRUCTURE: Head**: Cylindrical, L/W = 2.44. Postocular lobe moderately long; in dorsal view anteriorly gradually narrowing, posterior portion somewhat constricted. Eye moderately sized; lateral margin only slightly wider than postocular lobe; in lateral view removed from both dorsal and ventral surfaces of head. *Labium*: I: II: III = 1: 1.8: 0.4. **Thorax**: Anterolateral angle bearing small protuberance; medial longitudinal sulcus shallow near collar, deepening anterior to transverse sulcus of pronotum. Posterior pronotal lobe with rugulose surface; disc elevated above humeral angle; humeral angle armed, with tuberculate to long spinous lateral processes. Scutellum moderately long; apex angulate, slightly projected upward. *Legs*: Slender. Profemoral diameter slightly larger than mesofemoral diameter, metafemoral diameter slightly less than that of mesofemur. *Hemelytron*: Surpassing apex of abdomen by about length of abdominal segment seven; quadrate cell small, elongate; Cu and M of cubital cell converging towards R. **GENITALIA**: (Fig. 131) *Pygophore*: Elongate ovoid; not expanded laterally in dorsal view. Medial process triangular; broad; short; erect; nearly straight; apex in posterior view blunt, without modification. *Paramere*: Cylindrical; moderately long, slightly exceeding medial process; nearly straight; apical part slightly enlarged. *Phallus*: Dorsal phallothecal sclerite somewhat squarish; apical portion of phallothecal sclerite not distinctly tapered, surface flat; apex truncate, medially emarginate; posterior margin of foramen broadly inversely V-shaped. Struts attached to dorsal phallothecal sclerite; apically separate, connected by bridge; basally fused. Basal plate arm slender; separate; subparallel; in lateral view nearly straight, very slightly curved; bridge moderately long; extension of basal plate small and confined to apex of basal plate arm.

***Female***: (Fig. 130c, d) Similar to male, except for the following. Larger than male, total length 13.26–18.48 mm (mean 15.48 mm, Suppl. material 2). Coloration rather similar to that in male; lateral process on humeral angle dark brown, often longer; apices of femora reddish. Hemelytron slightly surpassing apex of abdomen.

#### Diagnosis

Recognized by the following combination of characters: Yellow-green to green-black; apices of femora with reddish or brown bands; disc elevated above humeral angle. As with other members of the *Zelus luridus* species group, the medial process is triangular, its base distinct from rest of the ventral rim of pygophore and apex without modification (Fig. 131a, b). *Zelus luridus* has the pronotal disc noticeably elevated above and not continuous with the humeral angle, thus distinguishing it from both *Z. exsanguis* and *Z. ambulans*. Males of the two differ in significant ways in paramere and medial process (Fig. 3). Among males of the *Zelus luridus* species group, *Z. luridus* is similar to *Z. antiguensis*, and *Z. grandoculus* in having the paramere apex not greatly expanded. The medial process is narrower than that in *Z. antiguensis*. The eyes are not as prominent as those of *Z. grandoculus*. Among species with overlapping distributions, *Z. luridus* may be confused with *Z. renardii*. However, *Z. renardii* usually has the corium reddish, whereas it is greenish or dark brown in *Z. luridus*.

#### Distribution

**Figure 132.**
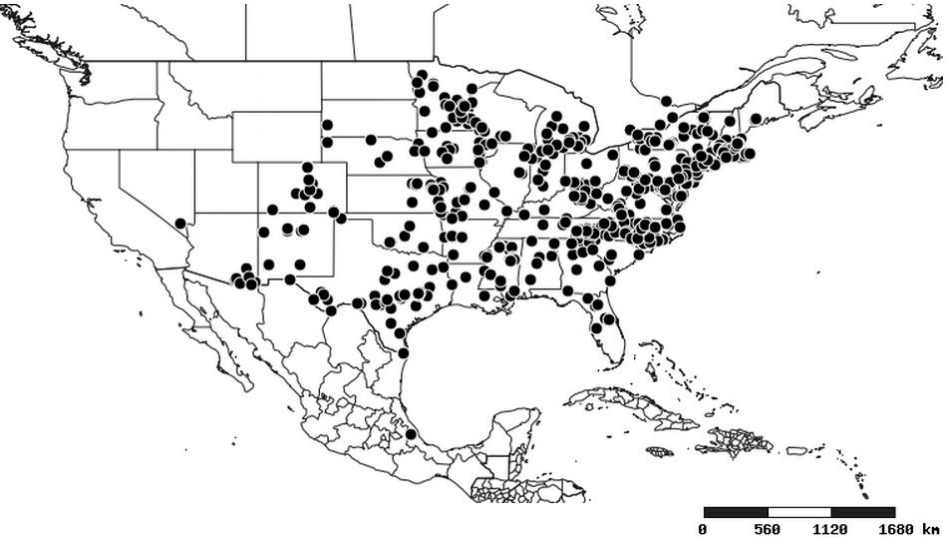
*Zelus luridus* Stål, 1862, specimen record map.

North America (Fig. 132). Countries with records: Canada, Mexico and USA.

#### Taxon discussion

This is one of the most commonly collected species in this genus. Hart (1986) discussed intraspecific variations and species identity confusions, which we briefly summarize here. Individuals in the western population (i.e., Southeastern Arizona) are larger than those from the eastern population. Eastern males have parameres somewhat more enlarged apically (Fig. 131a), and the apical half of the dorsal phallothecal sclerite is less expanded laterally (Fig. 131c). Since Champion (1898) synonymized *Z. luridus* and *Z. ambulans* under *Z. exsanguis*, almost all specimens collected from the US were labeled as *Z. exsanguis*. *Zelus luridus* has the disc clearly elevated above, and not continuous with, the humeral angle which distinguishes it from *Z. exsanguis*.

### Zelus mattogrossensis

Wygodzinsky, 1947

Zelus mattogrossensis Wygodzinsky, 1947, p. 431–434, orig. descr. and fig.; Wygodzinsky, 1948, p. 17, cat.; Wygodzinsky, 1949a, p. 49, checklist; Wygodzinsky, 1960, p. 308, note; Maldonado, 1990, p. 329, cat.

#### Materials

- **Type status:** Holotype. **Occurrence:** recordedBy: J.C.M. Carvalho; **Taxon:** scientificName: Zelus mattogrossensis; family: Reduviidae; genus: Zelus; scientificNameAuthorship: Wygodzinsky, 1947; **Location:** country: BRAZIL; stateProvince: Mato Grosso; locality: Chavantina; decimalLatitude: -14.66667; decimalLongitude: -52.35; **Event:** eventDate: 1947-06; **Record Level:** institutionCode: Instituto de Ecologia e Experimentacao Agricola, Rio de Janeiro
- **Type status:** Paratype. **Occurrence:** recordedBy: P. Wygodzinsky; **Taxon:** scientificName: Zelus mattogrossensis; family: Reduviidae; genus: Zelus; scientificNameAuthorship: Wygodzinsky, 1947; **Location:** country: BRAZIL; stateProvince: Goias; locality: Leopoldo Bulhoes; **Event:** eventDate: 1937-11; **Record Level:** institutionCode: collection of P. Wygodzinsky
- **Type status:** Paratype. **Occurrence:** catalogNumber: UCR_ENT 00046752; recordedBy: J. C. M. Carvalho; sex: Adult Male; **Taxon:** scientificName: Zelus mattogrossensis; family: Reduviidae; genus: Zelus; scientificNameAuthorship: Wygodzinsky, 1947; **Location:** country: BRAZIL; stateProvince: Minas Gerais; locality: Carmo do Rio Claro; verbatimElevation: 859 m; decimalLatitude: -20.9667; decimalLongitude: -46.1167; georeferenceSources: Gazetteer; **Identification:** identifiedBy: G. Zhang; dateIdentified: 2012; **Event:** eventDate: 1944-12-13; **Record Level:** institutionCode: AMNH

#### Description

Figs 133, 134, 135

***Male***: (Fig. 133a, b) Small, total length 9.18–10.49 mm (mean 9.85 mm, Suppl. material 2); slender. **COLORATION**: Brown. Medial longitudinal stripe on postocular lobe, patch between eye and ocellus, and ventral surface of head light-colored. Two yellow bands on scape and one on pedicel. Anterior pronotal lobe darker than posterior lobe. Lateral surface of pronotum and parts of pleura lighter colored. Femora and tibiae banded. **VESTITURE**: Moderately setose. Body surface with short, recumbent and erect setae; some longer setae on ventral surface of head and abdomen. Paramere with dense, moderately long, erect setae on dorsal surface. Corium and clavus with short, recumbent setae. **STRUCTURE: Head**: Cylindrical, L/W = 2.61. Postocular lobe long; in dorsal view anteriorly gradually narrowing, posterior portion constant, slightly narrower. Eye moderately sized; lateral margin only slightly wider than postocular lobe; dorsal margin attaining postocular transverse groove, ventral margin removed from ventral surface of head. *Labium*: I: II: III = 1: 1.8: 0.4. Basiflagellomere diameter larger than that of pedicel. **Thorax**: Anterolateral angle bearing small projection; medial longitudinal sulcus shallow near collar, deepening posteriorly. Posterior pronotal lobe with rugulose surface; disc distinctly elevated above humeral angle; humeral angle rounded, without projection. Scutellum moderately long; apex angulate, not projected. *Legs*: Moderately robust. *Hemelytron*: Slightly surpassing apex of abdomen, not more than length of abdominal segment seven; quadrate cell small, relatively broad; Cu and M of cubital cell converging towards R. **GENITALIA**: (Fig. 134) *Pygophore*: Elongate ovoid; slightly expanded laterally near base of paramere in dorsal view. Medial process cylindrical; slender; moderately long; erect; straight; apex in posterior view angulate, subapical ridge-like medial elevation, not across. *Paramere*: Cylindrical; moderately long, slightly exceeding medial process; directed posteriad, slightly curved towards medial process; basally slightly constricted; nearly straight. *Phallus*: Dorsal phallothecal sclerite somewhat pentagonal in dorsal view; sharp blade-like heavy sclerotization originating from basal arms, directed apically, elevated above dorsal surface, extending to about mid-point of phallothecal sclerite; ridge mesad to process; apical portion of phallothecal sclerite gradually tapering, convex, laterally angulate; apex truncate; posterior margin of foramen inversely V-shaped. Struts attached to dorsal phallothecal sclerite; apically fused; basally separate. Basal plate arm robust; medially fused; in lateral view strongly curved at midpoint; bridge short; extension of basal plate expanded onto arm.

***Female***: (Fig. 133c, d) Similar to male, except for the following. Larger than male, total length 10.30–11.72 mm (mean 10.98 mm, Suppl. material 2). Pale brown, anterior and posterior pronotal lobes same color, legs without bands or with very inconspicuous bands. Hemelytron attaining apex of abdomen.

#### Diagnosis

Distinguished by the small size; the robust form, the humeral angle rounded, without projection; the profemur much longer than the metafemur (1.20x); the profemoral length being less than 20.0x the profemoral width (16.94x). The paramere base not distinctly constricted; the medial process slender, apex angulate and bearing subapical medial protrusion; the presence of blade-like process on dorsal phallotheca and the process not extending beyond mid-point.

#### Distribution

**Figure 135.**
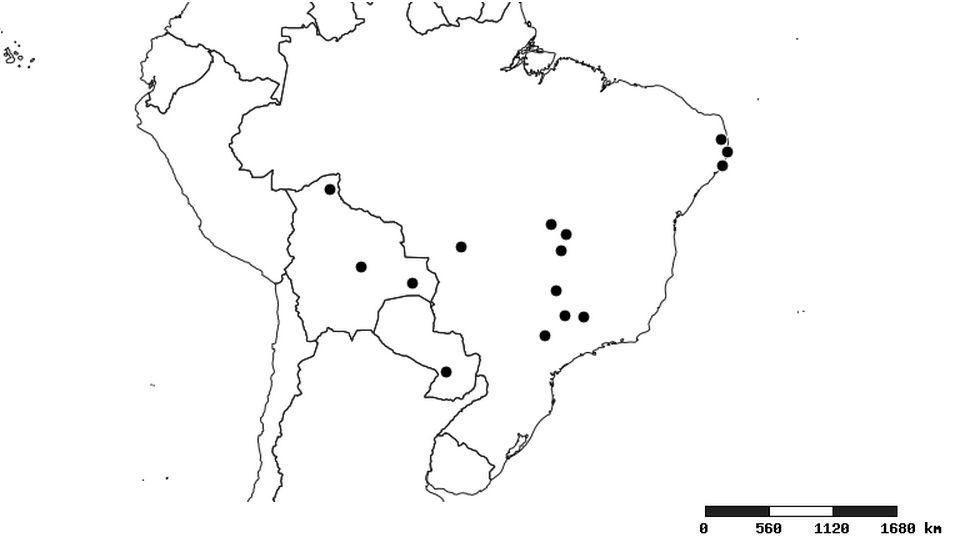
*Zelus mattogrossensis* Wygodzinsky, 1947, specimen record map

South America (Fig. 135). Countries with records: Bolivia, Brazil and Paraguay.

### Zelus means

Fabricius, 1803

Zelus means Fabricius, 1803, p. 282. orig. descr.; Stål, 1868, p. 107, descr.; Stål, 1872, p. 89, cat.; Walker, 1873, p. 134, cat; Lethierry and Severin, 1896, p.l62., cat.; Champion, 1898, p. 254, note; Wygodzinsky, 1949a, p. 49, checklist; Maldonado, 1990, p. 329, cat.
Zelus means *Eccagoras trimaculicollis* Stål 1855, p. 189, orig. descr.
Zelus trimaculicollis Stål, 1866, p. 298, descr.; Stål, 1872, p. 89, cat.; Lethierry and Severin, 1896, p. 153, cat.; Champion, 1898, p. 254, note; Wygodzinsky, 1949a, p. 50, checklist; Maldonado, 1990, p. 331, cat. **syn. nov.** (current study).
Zelus trimaculatus Champion, 1898, p. 254, orig. descr.; Wygodzinsky, 1949a, p. 50, checklist; Maldonado, 1990, p. 331, cat. **syn. nov.** (current study).

#### Materials

- **Type status:** Lectotype. **Occurrence:** occurrenceRemarks: Lectotype of *Zelus means* Fabricius, 1803 (**New Designation** by Zhang, Har & Weirauch, 2016). Bears the following labels: Type / Zelus means in Am. Mer. Schmidt; recordedBy: Schmidt; **Taxon:** scientificName: Zelus means; family: Reduviidae; genus: Zelus; scientificNameAuthorship: Fabricius, 1803; **Location:** locality: South America (American Meridonali); **Record Level:** institutionCode: ZMUC
- **Type status:** Other material. **Occurrence:** catalogNumber: UCR_ENT 00041011; occurrenceRemarks: **Lectotype** of *Zelus trimaculicollis* Stal 1866 (**New Designation** by Zhang, Hart & Weirauch, 2016), now synonym of Zelus means Fabricius 1803. Verbatim label info: Brasil / Typus / Zelustrimaculicollis Stal / Lectotype *Zelus trimaculicollis* Stal / designated by E.R.Hart / *Zelus means* Fabricius det. E.R. Hart 1972 / NHRS-GULI 000000350; sex: Adult Female; otherCatalogNumbers: NHRS-GULI 000000350; **Taxon:** scientificName: Zelus means; family: Reduviidae; genus: Zelus; scientificNameAuthorship: Fabricius, 1803; **Location:** country: BRAZIL; stateProvince: unknown; locality: Unknown; **Identification:** identifiedBy: G. Zhang; dateIdentified: 2012; **Event:** eventDate: No date provided; **Record Level:** institutionCode: NHRS
- **Type status:** Other material. **Occurrence:** occurrenceRemarks: **Paralectotype** of *Zelus trimaculicollis* Stal 1866 (**New Designation** by Zhang, Hart & Weirauch 2016), junior synonym of *Zelus means* Fabricius 1803. Verbatim label info: Brasil / Stal / Paratypus; sex: Adult Female; **Taxon:** scientificName: Zelus means; family: Reduviidae; genus: Zelus; scientificNameAuthorship: Fabricius, 1803; **Location:** country: BRAZIL; stateProvince: unknown; locality: Unknown; **Event:** eventDate: No date provided; **Record Level:** institutionCode: NHRS
- **Type status:** Other material. **Occurrence:** occurrenceRemarks: **Paralectotype** of *Zelus trimaculicollis* Stal 1866 (**New Designation** by Zhang, Hart & Weirauch, 2016), junior synonym of *Zelus means* Fabricius 1803. Verbatim label info: Brasil / Paratypus; sex: Adult Female; **Taxon:** scientificName: Zelus means; family: Reduviidae; genus: Zelus; scientificNameAuthorship: Fabricius, 1803; **Location:** country: BRAZIL; stateProvince: unknown; locality: Unknown; **Event:** eventDate: No date provided; **Record Level:** institutionCode: NHRS
- **Type status:** Other material. **Occurrence:** occurrenceRemarks: **Holotype** of *Zelus trimaculatus* Champion 1898, junior synonym of *Zelus means* Fabricius 1803. Bears the following labels: Type/B.C.A. Rhyn. II. *Zelus trimaculatus* Ch / Sp. figured / V. de Chiriqui, 25-4000 ft., Champion; sex: Adult Female; **Taxon:** scientificName: Zelus means; family: Reduviidae; genus: Zelus; scientificNameAuthorship: Fabricius, 1803; **Location:** country: PANAMA; stateProvince: Chiriqui; **Event:** eventDate: V. de Chiriqui; **Record Level:** institutionCode: BMNH

#### Description

Figs 136, 137

***Male***: Unknown.

***Female***: (Fig. 136) Large, total length 16.94–19.31 mm (mean 18.29 mm, Suppl. material 2); slender. **COLORATION**: Reddish, orangish, mixed with black; pattern variable. **VESTITURE**: Densely setose. Head with short recumbent setae and some scattered longer setae. Anterior pronotal lobe with erect spine-like setae, short to moderate in length; posterior pronotal lobe with short, erect, spine-like setae. Abdomen with short, recumbent and short to moderately long, erect setae. **STRUCTURE: Head**: Stout. Postocular lobe long; in dorsal view anteriorly gradually narrowing, posterior portion constant, slightly narrower. Eye smallish; lateral margin only slightly wider than postocular lobe; dorsal and ventral margins removed from surfaces of head. *Labium*: I: II: III = 1: 1.5: 0.4. Basiflagellomere diameter larger than that of pedicel. **Thorax**: Anterolateral angle rounded, without projection; medial longitudinal sulcus evident throughout, deepening posteriorly. Posterior pronotal lobe with rugulose surface; disc distinctly elevated above humeral angle; humeral angle rounded, without projection. Scutellum moderately long; apex blunt, not projected. *Legs*: Moderately robust. *Hemelytron*: Surpassing apex of abdomen by about twice length of abdominal segment seven; quadrate cell large and broad; Cu and M of cubital cell subparallel.

#### Diagnosis

The humeral angle rounded; the pronotum with spine-like setae; and the colors usually consisting of yellow, orange, red and black. The anterior pronotal lobe is rather small, margins not laterally expanded, nearly continuous with lateral margins of posterior lobe, dorsally nearly flat, not bulging. The small anterior lobe together with the regularly sized posterior lobe gives the pronotum a triangular appearance. Most similar to *Z. fuliginatus*, but the postocular lobe is shorter.

#### Distribution

**Figure 137.**
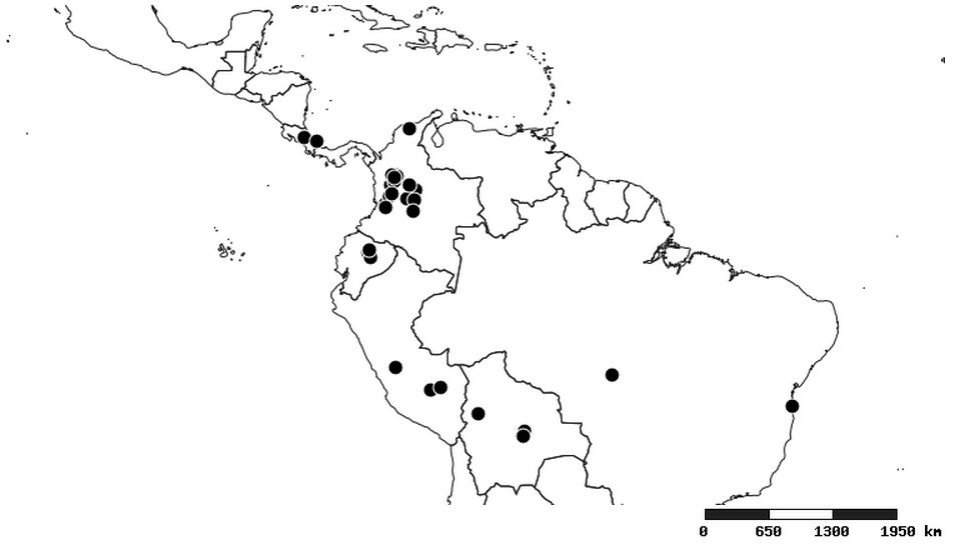
*Zelus means* Fabricius, 1803, specimen records

Southern Central America and South America (Fig. 137). Countries with records: Bolivia, Brazil, Costa Rica, Colombia, Ecuador and Peru.

#### Taxon discussion

On the basis of the spine-like setae on the head and pronotum and the rounded humeral angle, *Zelus means* would be placed in either the *Zelus longipes* species group or the *Zelus vagans* species group. Due to its close resemblance to *Z. fuliginatus*, this species is most likely part of the *Zelus vagans* species group.

This species appears to have a highly variable color pattern in all areas of its distribution. The clavus and corium range from entirely brownish-black to almost entirely light yellowish-brown. The dorsum of the pronotal lobe may be entirely brownish-black, but is usually variably patterned brownish-black and reddish-brown.

### Zelus mimus

Stål, 1862

Zelus mimus Stål, 1862, p. 451, orig. descr. (subgenus *Diplodus*); Stål, 1872, p. 91, cat. (subgenus *Diplodus*); Lethierry and Severin, 1896, p. 152, cat.; Champion, 1898, p. 257, 261, note, list and senior syn. of *Z. umbratilis*; Kuhlgatz, 1902, p. 266, note; Fracker, 1913, p. 239, 240, list (subgenus *Diplodus*); Williams, 1918, p. 163–173; Wygodzinsky, 1949a, p. 49, checklist; Maldonado, 1990, p. 329, cat.
Diplodus mimus : Walker, 1873, p. 124, cat.; Uhler, 1886, p. 24, checklist.
Zelus umbratilis Stål, 1862, p. 451, orig. descr. (subgenus *Diplodus*); Stål, 1872, p. 91, cat. (subgenus *Diplodus*); Lethierry and Severin, 1896, p. 153, cat.; Champion, 1898, p. 261, junior syn. of *Z. mimus*.
Diplodus umbratilis : Walker, 1873, p. 124, cat.; Uhler, 1886, p. 24, checklist.

#### Materials

- **Type status:** Lectotype. **Occurrence:** catalogNumber: UCR_ENT 00075074; occurrenceRemarks: Lectotype of *Zelus mimus* Stål, 1862 (**New Designation** by Zhang, Hart & Weirauch, 2016). Verbatim label info: Mexico coll. Signoret / mimus det. Stal / B.C.A. Rhyn.II. *Zelus mimus* St. / Lectotype *Zelus mimus* Stal / designated by E. R. Hart / Lectotypus *Zelus mimus* STAL, 1862 etik. Hecher 1996 REDV. 480/1; recordedBy: Signoret; sex: Adult Female; **Taxon:** scientificName: Zelus mimus; family: Reduviidae; genus: Zelus; scientificNameAuthorship: Stål, 1862; **Location:** country: MEXICO; stateProvince: None or Unknown; locality: unknown; **Identification:** identifiedBy: G. Zhang; dateIdentified: 2012; **Event:** eventDate: No date provided; **Record Level:** institutionCode: NHMW
- **Type status:** Other material. **Occurrence:** catalogNumber: UCR_ENT 00041012; occurrenceRemarks: **Lectotype** of *Zelus umbratilis* Stål, 1862 (**New Designation** by Zhang, Hart & Weirauch, 2016), junior synonym of *Zelus mimus* Stål, 1862. Verbatim label info: Mexico / Salle / umbratilis Stal / Lectotype *Zelus umbratilis* Stal / designated by E.R.Hart / *Zelus mimus* Stal det. E.R. Hart 1972 / Typus / NHRS-GULI 000000351; recordedBy: salle; sex: Adult Female; otherCatalogNumbers: NHRS-GULI 000000351; **Taxon:** scientificName: Zelus mimus; family: Reduviidae; genus: Zelus; scientificNameAuthorship: Stål, 1862; **Location:** country: MEXICO; stateProvince: None or Unknown; locality: unknown; **Identification:** identifiedBy: G. Zhang; dateIdentified: 2012; **Event:** eventDate: No date provided; **Record Level:** institutionCode: NHRS

#### Description

Figs 138, 139, 140

***Male***: (Fig. 138a, b) Small, total length 9.69–11.32 mm (mean 10.64 mm, Suppl. material 2); slender. **COLORATION**: Dorsum brown to dark brown; posterior pronotal lobe sometimes slightly lighter. Pale yellow or light brown on ventral surface of head, maxillary plate, lateral surface of posterior pronotal lobe, parts of pleura and medial surface of abdominal venter. Legs with yellow and brown bands. **VESTITURE**: Sparsely setose. Dorsum of head and anterior pronotal lobe with very sparse, short, erect or recumbent setae, nearly glabrous; ventral surface of head with sparse, long, erect and short, recumbent setae. Posterior pronotal lobe with short, erect or recument setae. Hemelytron primarily with short, recumbent setae. Pleura and abdominal venter with short, erect or recumbent setae, as well as wax-like setae. Very sparse, short setae on apical half of paramere. **STRUCTURE: Head**: Cylindrical, L/W = 2.17. Postocular lobe relatively short; in dorsal view distinctly narrowing through anterior 2/3, posterior 1/3 constant, tube-like. Eye prominent; lateral margin much wider than postocular lobe; dorsal margin attaining postocular transverse groove, ventral margin removed from ventral surface of head. *Labium*: I: II: III = 1: 1.8: 0.4. Basiflagellomere diameter larger than that of pedicel. **Thorax**: Anterolateral angle bearing small projection; medial longitudinal sulcus shallow near collar, deepening posteriorly. Posterior pronotal lobe with rugulose surface; disc distinctly elevated above humeral angle; humeral angle armed, with dentate projection. Scutellum long; apex angulate, slightly projected upward. *Legs*: Slender. *Hemelytron*: Surpassing apex of abdomen by about length of abdominal segment seven; quadrate cell small and slender; Cu and M of cubital cell subparallel. **GENITALIA**: (Fig. 139) *Pygophore*: Ovoid. Medial process cylindrical; slender; long; erect; apex in posterior view modified, folded posteriad. *Paramere*: Cylindrical; moderately long, achieving apex of medial process; directed posteriad; strongly curved dorsad, nearly vertical; apical part not enlarged. *Phallus*: Dorsal phallothecal sclerite shield-shaped; apical portion of phallothecal sclerite tapered, convex, laterally rounded, not forming angle; apex rounded; posterior margin of foramen broadly concave. Struts attached to dorsal phallothecal sclerite; apically separate, connected by bridge; basally almost completely fused. Basal plate arm moderately robust; basally fused; in lateral view nearly straight, very slightly curved; bridge short; extension of basal plate expanded onto arm.

***Female***: (Fig. 138c, d, e, f) Similar to male, except for the following. Larger than male, total length 11.81–14.06 mm (mean 13.14 mm, Suppl. material 2). Posterior pronotal lobe sometimes brownish orange or with orange longitudinal stripes; occasional specimens with entire pronotum reddish-brown. Legs banded or unicolorous.

#### Diagnosis

Dark brown coloration predominating dorsally in most specimens, posterior pronotal lobe laterally yellowish. Among species that have overlapping distributions (Southern Mexico and Central America), the coloration of *Z. mimus* is unique. Males can also be recognized by the paramere apically greatly projected dorsad (Fig. 139a); and the apex of the medial process folded posteriorly. Similar to *Z. inconstans*, but the medial process is much longer and more slender.

#### Distribution

**Figure 140.**
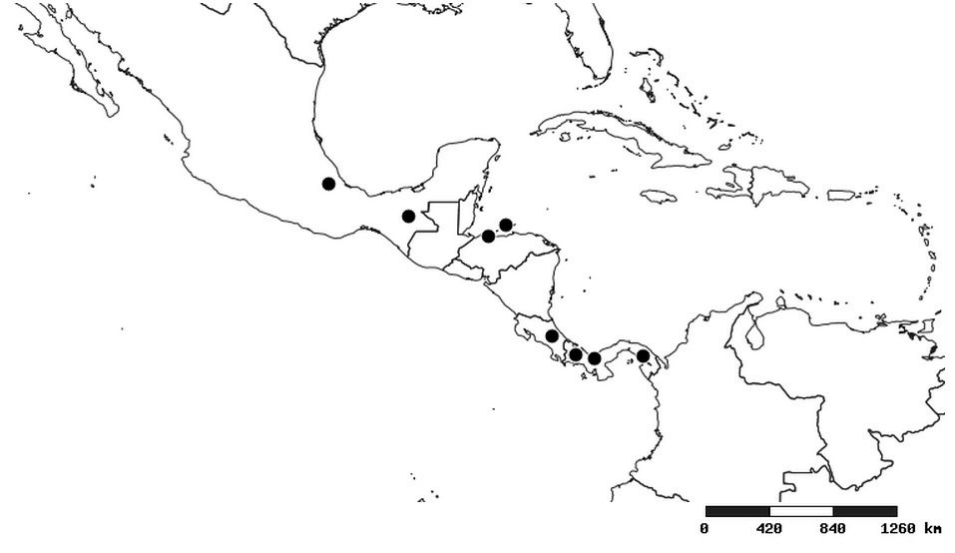
*Zelus mimus* Stål, 1862, specimen records

Southern Mexico and Central America (Fig. 140). Countries with records: Costa Rica, Honduras, Mexico and Panama.

### Zelus minutus

Hart, 1987

Zelus minutus Hart, 1987, p. 299-301, figs. 16-21, orig. descr., note, fig. and key; Maldonado, 1990, p. 330, cat.

#### Materials

- **Type status:** Holotype. **Occurrence:** catalogNumber: UCR_ENT 00023689; recordedBy: P.H. van Doesburg, Jr; sex: Adult Male; **Taxon:** scientificName: Zelus minutus; family: Reduviidae; genus: Zelus; scientificNameAuthorship: Hart, 1987; **Location:** country: SURINAME; stateProvince: Commewijne; locality: Pl. Leliendaal; decimalLatitude: 5.86666; decimalLongitude: -55.03333; georeferenceSources: Gazetteer; **Identification:** identifiedBy: G. Zhang; dateIdentified: 2012; **Event:** eventDate: 1963-03-17; **Record Level:** institutionCode: RMNH
- **Type status:** Allotype. **Occurrence:** recordedBy: P.H. van Doesburg, Jr; sex: Adult Female; **Taxon:** scientificName: Zelus minutus; family: Reduviidae; genus: Zelus; scientificNameAuthorship: Hart, 1987; **Location:** country: SURINAME; stateProvince: Para; locality: Republiek; decimalLatitude: 5.5; decimalLongitude: -55.2; georeferenceSources: Gazetteer; **Event:** eventDate: 1962-01-04; **Record Level:** institutionCode: RMNH
- **Type status:** Paratype. **Occurrence:** catalogNumber: UCR_ENT 00071201; recordedBy: F. W. Walker; sex: Adult Male; **Taxon:** scientificName: Zelus minutus; family: Reduviidae; genus: Zelus; scientificNameAuthorship: Hart, 1987; **Location:** country: COLOMBIA; stateProvince: Magdalena; locality: Sevilla; decimalLatitude: 10.76343; decimalLongitude: -74.13916; georeferenceSources: Gazetteer; **Identification:** identifiedBy: G. Zhang; dateIdentified: 2012; **Event:** eventDate: 1926-07-24; **Record Level:** institutionCode: TAMU
- **Type status:** Paratype. **Occurrence:** catalogNumber: UCR_ENT 00009273; recordedBy: J. Zetek; sex: Adult Male; otherCatalogNumbers: 40-14769; **Taxon:** scientificName: Zelus minutus; family: Reduviidae; genus: Zelus; scientificNameAuthorship: Hart, 1987; **Location:** country: PANAMA; stateProvince: Canal Zone; locality: Barro Colorado Island; decimalLatitude: 9.16666; decimalLongitude: -79.83333; **Identification:** identifiedBy: G. Zhang; dateIdentified: 2012; **Event:** eventDate: 1940-05-24; **Record Level:** institutionCode: USNM
- **Type status:** Paratype. **Occurrence:** catalogNumber: UCR_ENT 00017868; recordedBy: M. Bates; sex: Adult Male; **Taxon:** scientificName: Zelus minutus; family: Reduviidae; genus: Zelus; scientificNameAuthorship: Hart, 1987; **Location:** country: PANAMA; stateProvince: Colon; locality: Barro Colorado Island, Canal Zone; decimalLatitude: 9.15472; decimalLongitude: -79.84806; georeferenceSources: GeoLocate Software; **Identification:** identifiedBy: G. Zhang; dateIdentified: 2012; **Event:** eventDate: October; **Record Level:** institutionCode: AMNH
- **Type status:** Paratype. **Occurrence:** catalogNumber: UCR_ENT 00030177; occurrenceRemarks: 'Planett 7144' and WW Wirth #1619 44-12740 on label; recordedBy: Unknown; sex: Adult Male; **Taxon:** scientificName: Zelus minutus; family: Reduviidae; genus: Zelus; scientificNameAuthorship: Hart, 1987; **Location:** country: PANAMA; stateProvince: Colon; locality: Coco Solo; decimalLatitude: 9.37; decimalLongitude: -79.8817; georeferenceSources: Gazetteer; **Identification:** identifiedBy: G. Zhang; dateIdentified: 2012; **Event:** eventDate: 1944-04-27; **Record Level:** institutionCode: USNM
- **Type status:** Paratype. **Occurrence:** catalogNumber: UCR_ENT 00009272; recordedBy: Harold Morrison; sex: Adult Male; **Taxon:** scientificName: Zelus minutus; family: Reduviidae; genus: Zelus; scientificNameAuthorship: Hart, 1987; **Location:** country: TRINIDAD AND TOBAGO; stateProvince: Port-of-Spain; locality: Port-of-Spain, Department Agricultre Grounds; decimalLatitude: 10.66615; decimalLongitude: -61.51613; georeferenceSources: Google Earth; **Identification:** identifiedBy: G. Zhang; dateIdentified: 2012; **Event:** eventDate: 1918-11-23; **Record Level:** institutionCode: USNM
- **Type status:** Paratype. **Occurrence:** catalogNumber: UCR_ENT 00019690; recordedBy: Borys Malkin; sex: Adult Male; **Taxon:** scientificName: Zelus minutus; family: Reduviidae; genus: Zelus; scientificNameAuthorship: Hart, 1987; **Location:** country: VENEZUELA; stateProvince: Portuguesa; locality: Guanare; verbatimElevation: 173 m; decimalLatitude: 9.05; decimalLongitude: -65.75; georeferenceSources: Gazetteer; **Identification:** identifiedBy: G. Zhang; dateIdentified: 2012; **Event:** eventDate: 1957-09-10 to 1957-09-13; **Record Level:** institutionCode: CAS

#### Description

Figs 141, 142, 143

***Male***: (Fig. 141) Small, total length 7.80–10.13 mm (mean 9.00 mm, Suppl. material 2); slender. **COLORATION**: Yellowish-brown to brownish-black, shining. Antennae yellowish-brown to brown, apex of scape and pedicel and base of pedicel darker than remainder of segments. Anterior pronotal lobe yellowish-brown to brownish-black, shining. Posterior pronotal lobe yellowish-brown to brownish-black, pattern variable, medial and lateral areas lightest in dark specimens. Scutellum yellowish-brown, apex lighter than surrounding area. Legs yellowish-brown with two to four brown rings on apical 1/2 of femora, variable brown areas on tibiae. Clavus and corium yellowish-brown to brown, darkest along costal margin, membrane nearly clear, veins dark brown. Dorsum of abdomen yellowish-brown to dark brown, pattern variable, lateral surfaces yellowish-brown. Pygophore yellowish-brown to brown, parameres dark brown to brownish-black. **VESTITURE**: Moderately setose. Scattered erect and recumbent setae over entire surface of head, longer ventrally. Anterior pronotal lobe with very sparse short recumbent setae on reduced setal tracts dorsally, more dense laterally; posterior pronotal lobe with short recumbent setae over entire surface, some erect setae laterally; scutellum with long recumbent setae. Abdominal venter with sparse short to long erect setae. Parameres with erect setae. **STRUCTURE: Head**: Cylindrical, L/W = 2.13. Postocular lobe long; in dorsal view distinctly narrowing through anterior 2/3, posterior 1/3 constant, tube-like. Eye prominent; lateral margin much wider than postocular lobe; dorsal margin removed from postocular transverse groove, ventral margin attaining ventral surface of head. *Labium*: I: II: III = 1: 2.0: 0.5. Basiflagellomere diameter larger than that of pedicel. **Thorax**: Anterolateral angle with inconspicuous subtuberculate projection; medial longitudinal sulcus shallow near collar, deepening posteriorly. Posterior pronotal lobe with rugulose surface; disc slightly elevated above humeral angle; humeral angle rounded, without projection. Scutellum moderately long; apex angulate, very slightly projected upward. *Legs*: Slender. *Hemelytron*: Surpassing apex of abdomen by about length of abdominal segment seven; quadrate cell small; Cu and M of cubital cell subparallel. **GENITALIA**: (Fig. 142) *Pygophore*: Elongate ovoid; not expanded laterally in dorsal view. Medial process broadly triangular; short; posteriorly directed, in less than forty-five degree with body axis; straight; apex in posterior view angulate, without modification. *Paramere*: Cylindrical; long, surpassing medial process; directed posteriad; basally slightly narrower; nearly straight; apical part very slightly enlarged. *Phallus*: Dorsal phallothecal sclerite somewhat squarish; somewhat enlarged at base; apical portion of phallothecal sclerite not distinctly tapered, flat; apex truncate, medially slightly to greatly emarginate; posterior margin of foramen broadly concave. Struts attached to dorsal phallothecal sclerite; apically separate, connected by bridge; basally fused. Basal plate arm robust; separate; subparallel; in lateral view nearly straight, very slightly curved; bridge moderately long; extension of basal plate small, marginally expanded onto arm.

***Female***: Similar to male, except for the following. Larger than male, total length 10.70 mm (Suppl. material 2).

#### Diagnosis

This species can be readily recognized by the small size (<10.2 mm) and the disc of the posterior lobe with spinous tubercles. The medial process is broadly triangular, apex without modification (shared with the *Zelus tetracanthus* species group). Can be recognized among the *Zelus tetracanthus* species group by the relatively long paramere, exceeding the medial process.

#### Distribution

**Figure 143.**
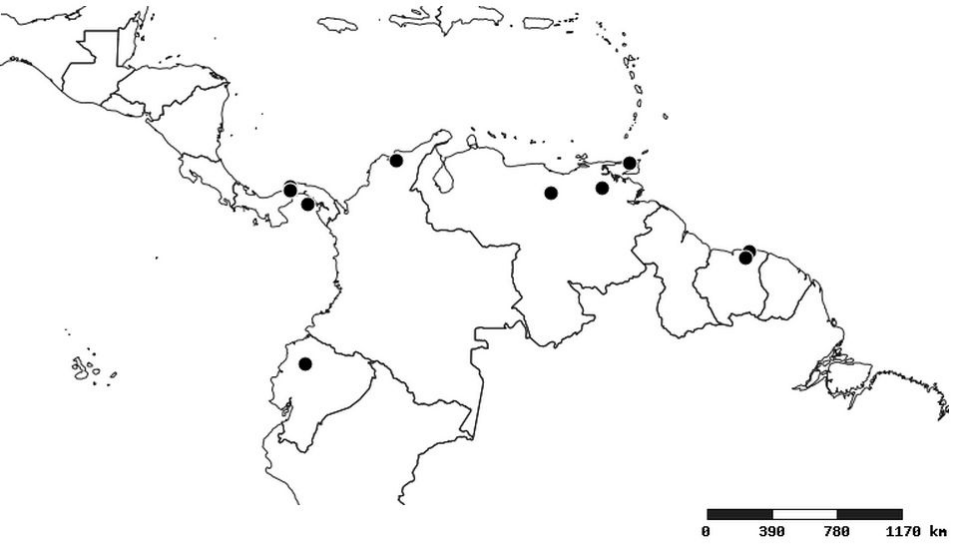
*Zelus minutus* Hart, 1987, specimen record map

Southern Central America, South America and adjacent islands of the Caribbean (Fig. 143). Countries with records: Brazil, Colombia, Ecuador, Panama, Suriname, Trinidad & Tobago and Venezuela.

#### Taxon discussion

*Zelus minutus* shows some variations through its range. The Panamanian specimens have noticeably shorter paramere and the apex of the dorsal phallothecal sclerite is somewhat less emarginate.

### Zelus nigromaculatus

Champion, 1899

Zelus nigromaculatus Champion, 1898, p. 261–262, Tab. XV, Fig. 26, orig. descr. and fig.; Wygodzinsky, 1949a, p. 49, checklist; Maldonado, 1990, p. 330, cat.

#### Materials

- **Type status:** Lectotype. **Occurrence:** catalogNumber: UCR_ENT 00048757; occurrenceRemarks: Lectotype of *Zelus nigromaculatus* Champion, 1898 (**New Designation** by Zhang, Hart & Weirauch, 2016) Verbatim label info: Type / B.C.A.Rhyn.II. *Zelus nigromaculatus* Ch. / Sp. figured. / Bugaba, 800-1,500 ft. Champion. / Lectotype *Zelus nigromaculatus* Champion des. by E.R. Hart; recordedBy: G.C. Champion; sex: Adult Male; **Taxon:** scientificName: Zelus nigromaculatus; family: Reduviidae; genus: Zelus; scientificNameAuthorship: Champion, 1899; **Location:** country: PANAMA; stateProvince: Chiriqui; locality: Bugaba; verbatimElevation: 457 m; decimalLatitude: 8.4833; decimalLongitude: -82.6167; georeferenceSources: Gazetteer; **Identification:** identifiedBy: E. R. Hart; dateIdentified: 1972; **Event:** eventDate: No date provided; **Record Level:** institutionCode: BMNH
- **Type status:** Allolectotype. **Occurrence:** occurrenceRemarks: Allolectotype of *Zelus nigromaculatus* Champion, 1898 (**New Designation** by Zhang, Hart & Weirauch, 2016) Verbatim label info: B.C.A.Rhyn.II. Zelus nigromaculatus Ch. / Bugaba, 800-1,500 ft. Champion. / Allolectotype Zelus nigromaculatus Champion des. by E.R. Hart; recordedBy: G.C. Champion; sex: Adult Female; **Taxon:** scientificName: Zelus nigromaculatus; family: Reduviidae; genus: Zelus; scientificNameAuthorship: Champion, 1899; **Location:** country: PANAMA; stateProvince: Chiriqui; locality: Bugaba; verbatimElevation: 457 m; decimalLatitude: 8.4833; decimalLongitude: -82.6167; georeferenceSources: Gazetteer; **Identification:** identifiedBy: E. R. Hart; dateIdentified: 1972; **Event:** eventDate: No date provided; **Record Level:** institutionCode: BMNH

#### Description

Figs 144, 145, 146

***Male***: (Fig. 144a, b) Medium-sized; slender. **COLORATION**: Mixed yellow and dark brown; ventral surfaces mostly yellow, dorsal pattern variable; some specimens wasp-like. **VESTITURE**: Sparsely setose. Dorsum of head with moderately dense, short, recumbent setae and sparse, short, erect, somewhat spine-like setae; ventral surface with moderately dense, short, recumbent setae and few moderately long, erect, fine setae. Pronotum with sparse, short, erect setae on dorsal surface, some setae curved apically, appearing recumbent; moderately dense, short to moderately long, recumbent setae on lateral surface and pleura, intermixed with semi-erect or erect setae. Legs with sparse setation on femora and moderately dense setation on tibiae. Corium and clavus with short, recumbent setae. **STRUCTURE: Head**: Cylindrical. Postocular lobe long; in dorsal view distinctly narrowing through anterior 2/3, posterior 1/3 constant, tube-like. Eye prominent; lateral margin much wider than postocular lobe; dorsal margin attaining postocular transverse groove, ventral margin removed from ventral surface of head in lateral view. *Labium*: I: II: III = 1: 1.7: 0.4. Basiflagellomere diameter larger than that of pedicel. **Thorax**: Anterolateral angle bearing small projection; medial longitudinal sulcus evident throughout, deepening posteriorly. Posterior pronotal lobe with rugulose surface; disc distinctly elevated above humeral angle; humeral angle armed, with spinous processes. Scutellum moderately long; apex angulate, very slightly projected upward. *Legs*: Slender. *Hemelytron*: Surpassing apex of abdomen by about length of abdominal segment seven; quadrate cell small and slender; Cu and M of cubital cell subparallel. **GENITALIA**: (Fig. 145) *Pygophore*: ovoid, not laterally expanded. Medial process cylindrical, slender; very long, much longer than paramere; laterally slightly compressed towards apex; semi-erect; straight; apex in posterior view acute, with small hooklike projection. *Paramere*: Cylindrical; relatively long, not reaching medial process; directed posteriad; slight bending at base; apical part very slightly enlarged. *Phallus*: Dorsal phallothecal sclerite somewhat shield-shaped; laterally with anteriorly directed small process; apical portion of phallothecal sclerite gradually tapering, apex rounded, medially slightly emarginate; posterior margin of foramen broadly V-shaped. Struts attached to dorsal phallothecal sclerite; apically separate, basally fused. Basal plate arms robust, fused; bridge extremely short; extension of basal plate well developed, greatly expanded laterally onto arm, covering more than 1/2 of arm.

***Female***: (Fig. 144c, d) Similar to male. Total length 16.46 mm (n=1, Suppl. material 2).

#### Diagnosis

Recognized by the conspicuous black and yellow color pattern, resembling vespid wasp; and the meso- and metafemora with at least three dark bands three yellow bands. Among males of the *Zelus panamensis* group (Fig. 12), *Z. nigromaculatus* has the shortest paramere and the longest medial process and the medial process is the most erect in this species group.

#### Distribution

**Figure 146.**
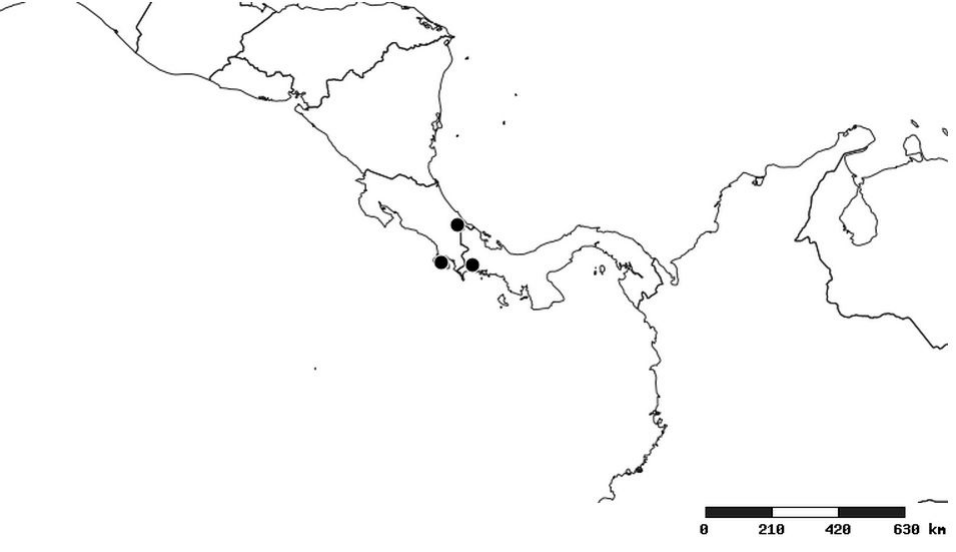
*Zelus nigromaculatus* Champion, 1899, specimen record map

Central America (Fig. 146). Countries with records: Costa Rica and Panama.

### Zelus nugax

Stål, 1862

Zelus nugax Stål, 1862, p. 450–451, orig. descr. (subgenus *Diplodus*); Stål, 1872, p. 91, cat. (subgenus *Diplodus*); Lethierry and Severin, 1896, p. 152, cat.; Champion, 1898, p. 257, 2, note and list; Kuhlgatz, 1902,, p. 266, note; Fracker, 1913, p. 239, 240, key and list (subgenus *Diplodus*); Fracker and Bruner, 1924, p. 171, list; Haviland, 1931, p. 137, 152, list and note; Leonard and Mills, 1931, p. 309–323, note; Wygodzinsky, 1947, p. 431, note; Wygodzinsky, 1949a, p. 49, checklist; Guagliumi, 1953, p. 16, note; Wygodzinsky, 1957, p. 268, junior syn. of *Zelus obscuridorsis*; Sucre, et. al., 1966, p. 31, note; Hart, 1986, p. 540, redescription, note, fig., key and lectotype desig.; Maldonado, 1990, p. 330, cat.
Diplodus nugax : Walker, 1873, p. 124, cat; Uhler, 1886, p. 24, checklist; Hart, 1986, p. 540, lectotype desig.
Zelus rufigeniculatus Haviland, 1931, p. 137, 148, 153–154, list, fig. and orig. descr. (subgenus *Diplodus*); Wygodzinsky, 1949a, p. 50, checklist; Hart, 1986, p. 540, junior syn. of *Z. nugax*.

#### Materials

- **Type status:** Lectotype. **Occurrence:** catalogNumber: UCR_ENT 00041008; occurrenceRemarks: Lectotype of *Zelus nugax* Stål, 1862, designated by Hart (1986). Verbatim label info: Mexico / Salle / nugax Stal / Lectotype *Zelus nugax* Stal / designated by E.R.Hart / Typus / NHRS-GULI 000000336; recordedBy: Salle; sex: Adult Female; otherCatalogNumbers: NHRS-GULI 000000336; **Taxon:** scientificName: Zelus nugax; family: Reduviidae; genus: Zelus; scientificNameAuthorship: Stål, 1862; **Location:** country: MEXICO; locality: unknown; **Identification:** identifiedBy: G. Zhang; dateIdentified: 2012; **Event:** eventDate: No date provided; **Record Level:** institutionCode: NHRS
- **Type status:** Other material. **Occurrence:** occurrenceRemarks: **Lectotype** of *Zelus rufigeniculatus* Haviland, 1931, designated by Hart (1986). Bears following labels: Type / Berbice Brit. Guiana-October 1922-e. coll. M. D. Haviland-d.d. Collegium Newnhamense / Pres. By Mrs. Brindley-B.M. 1928-172 / *Zelus rufigeniculatus* Haviland; recordedBy: Haviland, M.D.; sex: Adult Female; **Taxon:** scientificName: Zelus nugax; family: Reduviidae; genus: Zelus; scientificNameAuthorship: Stål, 1862; **Location:** country: Guyana; locality: Berbice; **Event:** eventDate: 1922-10; **Record Level:** institutionCode: BMNH
- **Type status:** Other material. **Occurrence:** occurrenceRemarks: **Paralectotype** of *Zelus rufigeniculatus* Haviland, 1931, designated by Hart (1986). Bears following labels: Type / Berbice Brit. Guiana-October 1922-e. coll. M. D. Haviland-d.d. Collegium Newnhamense / Pres. By Mrs. Brindley-B.M. 1928-172 / *Zelus rufigeniculatus* Haviland; recordedBy: Haviland, M.D.; sex: Adult Female; **Taxon:** scientificName: Zelus nugax; family: Reduviidae; genus: Zelus; scientificNameAuthorship: Stål, 1862; **Location:** country: Guyana; locality: Berbice; **Event:** eventDate: 1922-10; **Record Level:** institutionCode: BMNH

#### Description

Figs 147, 148, 149

***Male***: (Fig. 147a, b) Small, total length 9.54–10.78 mm (mean 10.23 mm, Suppl. material 2); slender. **COLORATION**: Dorsal surface brown, sometimes disc of posterior pronotal lobe and scutellum yellowish-brown. Lateral and ventral surfaces generally yellowish-brown. Femora with at least apices and often entire enlarged apical area dark reddish-brown,tibiae with variable dark reddish-brown bands. **VESTITURE**: Sparsely setose. Head with moderate to long erect setae and recumbent setae. Anterior pronotal lobe with short, recumbent setae dorsally confined to setal tracts, laterally with longer, recumbent to erect setae; posterior pronotal lobe with recumbent setae. Abdomen with short, recumbent and short to moderately long, erect setae. **STRUCTURE: Head**: Elongated, L/W = 2.48. Postocular lobe long; in dorsal view anteriorly gradually narrowing, posterior portion constant, slightly narrower. Eye smallish; lateral margin only slightly wider than postocular lobe; dorsal and ventral margins removed from surfaces of head. *Labium*: I: II: III = 1: 2.0: 0.4. Basiflagellomere diameter larger than that of pedicel. **Thorax**: Anterolateral angle with inconspicuous subtuberculate projection; medial longitudinal sulcus shallow near collar, deepening posteriorly. Posterior pronotal lobe with rugulose surface; disc distinctly elevated above humeral angle; humeral angle armed, with dentate projection. Scutellum long; apex slightly pointed dorsally. *Legs*: Very slender, femoral diameters subequal. *Hemelytron*: Slightly surpassing apex of abdomen, not more than length of abdominal segment seven; quadrate cell small; Cu and M of cubital cell subparallel, slightly converging. **GENITALIA**: (Fig. 148) *Pygophore*: Ovoid; not expanded laterally in dorsal view. Medial process cylindrical; very slender; long, only slightly shorter than paramere; laterally compressed; semi-erect; nearly straight; apex in posterior view acute, without modification. *Paramere*: Cylindrical; moderately long, achieving apex of medial process; directed posteriad; nearly straight; apical part not enlarged, apex somewhat truncate. *Phallus*: Dorsal phallothecal sclerite shield-shaped; apical portion of phallothecal sclerite distinctly tapered, slightly convex, laterally rounded, not forming angle; posterior margin of foramen deeply concave. Struts attached to dorsal phallothecal sclerite; massively fused. Basal plate arm moderately robust; widely separate; diverging; in lateral view very slightly curved; bridge moderately long; extension of basal plate not distinctly visible.

***Female***: (Fig. 147c, d) Similar to male, except for the following. Larger than male, total length 12.25–14.14 mm (mean 12.99 mm, Suppl. material 2).

#### Diagnosis

The slender, cylindrical paramere and the laterally compressed medial process can separate males of this species from most other species of the genus. Difficult to distinguish from *Z. pedestris*, but the paramere apex is generally more truncate, the dorsal phallothecal sclerite usually without lateral indentation, and the basal plate arms are separate in most specimens (see taxon discussion).

#### Distribution

**Figure 149.**
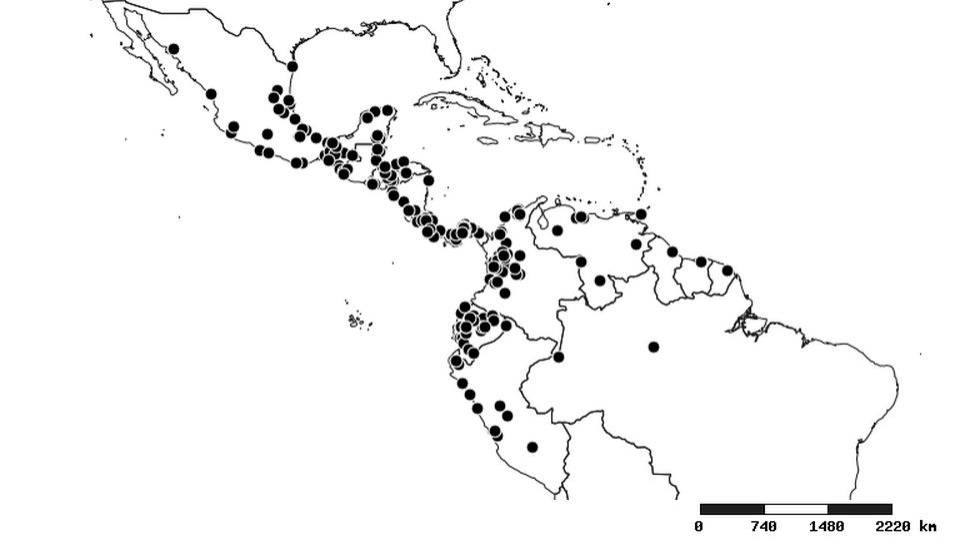
*Zelus nugax* Champion, 1899, specimen records

Mexico, Central America and South America (Fig. 149). Countries with records: Argentina, Belize, Brazil, Colombia, Costa Rica, Ecuador, El Salvador, French Guiana, Guatemala, Guyana, Honduras, Mexico, Nicaragua, Panama, Peru, Suriname, Trinidad and Tobago, USA, and Venezuela.

#### Taxon discussion

This is by far the most widespread species of the *Zelus nugax* species group, and one of the most widespread members of the genus. In Ecuador, Mexico and Central America, *Z. nugax* is apparently among the most common species of reduviids, occurring in second growth foliage and tall grasses throughout its range. Other than a variation in color from yellowish-brown to dark brown in any given area and a more noticeably produced scutellar apex in South America, there are little readily apparent external variations in the species. In the internal male genitalia, however, we find some noticeable geographic variations. In South America the basal plate arms are apparently always separate, while there seems to be tendency toward fusion of these arms as one progresses northward to Mexico.

The following discussion on some of the diagnostic characters may be useful for separating species when confusions arise, but as we have not clearly defined the boundaries between *Z. nugax* and *Z. pedestris*, reliable identification may not be achieved at all times. We will discuss this in the next paragraph. The straight bladelike medial process of *Z. nugax* distinguishes it from *Z. impar* and *Z. illotus*, both of which have similar appearances to *Z. nugax*, but both have recurved medial processes. The less rounded pygophore and more blunted paramere separate this species externally from most male specimens of *Z. pedestris*. Although it is difficult to separate the females of *Z. nugax* from *Z. illotus* and *Z. pedestris*, there appears to be some differences among these species. The normal lack of erect setae on the dorsal surface of the posterior pronotal lobe of *Z. illotus* and lower posterior margin of the anterior pronotal lobe of *Z. pedestris* usually serve as diagnostic characters for someone with large series and a familiarization with all three species.

It remains unresolved if *Z. nugax* and *Z. pedestris* are distinct species. They are currently delimited based on several characters of the male genitalia, which, however, do not appear to be fixed. With regards to four characters (paramere apex shape, fusion of basal plate arms, lateral indentation on dorsal phallothecal sclerite, and basal plate arm extension) used in delimitation, the following combinations have been observed: (1) paramere apex acute, basal plate arms fused, phallothecal indentation present and basal plate arm extension present and expanded laterally (observations based on specimens from Santa Catarina, Brazil); (2) paramere apex truncate, slightly enlarged, somewhat diamond-shaped, basal plate arms fused, indentation absent and extension present, laterally expanded (La Molina, Peru); and (3) paramere apex truncate, slightly enlarged, basal plate arms separate, phallothecal indentation absent and extension absent or inconspicuous (El Valle, Panama). Following the phylogenetic species concept *sensu* Wheeler and Platnick (Wheeler and Platnick 2000), we would call each population a different species. We restrained from doing this as we have not thoroughly examined the ranges of variations.

Both the types of *Z. nugax* and *Z. pedestris* are females, further complicating the application of the names as we have not been able to distinguish females of the two species. The type of *Z. nugax* is from Mexico and that of *Z. pedestris* from South America (country not recorded). *Zelus nugax* and *Z. pedestris* overlap broadly in northern South America, but *Z. nugax* appears to be absent South of Peru and in most of Brazil and *Z. pedestris* not recorded from North and Central Americas. This geographic pattern currently serves as an additional means to apply the names.

### Zelus panamensis

Zhang & Hart sp. n.

urn:lsid:zoobank.org:act:D4200CA7-DCDB-4D10-B0E3-9CD1CCB31AB6

#### Materials

- **Type status:** Holotype. **Occurrence:** catalogNumber: UCR_ENT 00008001; recordedBy: A. Busck; sex: Adult Male; **Taxon:** scientificName: Zelus panamensis; family: Reduviidae; genus: Zelus; scientificNameAuthorship: Zhang and Hart, 2016; **Location:** country: PANAMA; stateProvince: Canal Zone; locality: Alhajuelo; **Identification:** identifiedBy: G. Zhang; dateIdentified: 2013; **Event:** eventDate: 1911-04-05; **Record Level:** institutionCode: USNM
- **Type status:** Paratype. **Occurrence:** catalogNumber: UCR_ENT 00004645; occurrenceRemarks: Primary DNA voucher RCW_2806; recordedBy: J. Cryan, J. Urban, G. Svenson; sex: Adult Male; **Taxon:** scientificName: Zelus panamensis; family: Reduviidae; genus: Zelus; scientificNameAuthorship: Zhang and Hart, 2016; **Location:** country: NICARAGUA; stateProvince: Rio San Juan; locality: Refugio Bartola, nr. Indio Maíz Biological Reserve, ~6Km E of El Castillo; verbatimElevation: 50; decimalLatitude: 10.97252; decimalLongitude: -84.33916; georeferenceSources: Label; **Identification:** identifiedBy: G. Zhang; dateIdentified: 2013; **Event:** eventDate: 2010-11-01 to 2010-11-06; **Record Level:** institutionCode: UCR
- **Type status:** Paratype. **Occurrence:** catalogNumber: UCR_ENT 00007996; occurrenceRemarks: Designated as holotype for his new species *Zelus cestartus* [Mansucript name] by E. R. Hart (1972). *Zelus panamensis* and *Zelus cestartus* were considered to be synonymic by G. Zhang. This specimen hence loses its holotype status (which is not official since the name was never published). The red holotype label affixed to the specimen by Hart, however, remains on the specimen for the purpose of recording this (informal) taxonomic history.; recordedBy: Richter; sex: Adult Male; **Taxon:** scientificName: Zelus panamensis; family: Reduviidae; genus: Zelus; scientificNameAuthorship: Zhang and Hart, 2016; **Location:** country: COLOMBIA; stateProvince: Meta; locality: Cano Grande; decimalLatitude: 3.11171; decimalLongitude: -73.83689; georeferenceSources: Gazetteer; **Identification:** identifiedBy: G. Zhang; dateIdentified: 2013; **Event:** eventDate: 1948-01-20; **Record Level:** institutionCode: USNM
- **Type status:** Paratype. **Occurrence:** catalogNumber: UCR_ENT 00022969; recordedBy: Morales, Gilberto; sex: Adult Male; **Taxon:** scientificName: Zelus panamensis; family: Reduviidae; genus: Zelus; scientificNameAuthorship: Zhang and Hart, 2016; **Location:** country: COLOMBIA; stateProvince: Antioquia; locality: Carepa; decimalLatitude: 7.7664; decimalLongitude: -76.6611; georeferenceSources: Gazetteer; **Identification:** identifiedBy: G. Zhang; dateIdentified: 2013; **Event:** eventDate: 2001-09-13; **Record Level:** institutionCode: MEFLG
- **Type status:** Paratype. **Occurrence:** catalogNumber: UCR_ENT 00022970; recordedBy: R. Velez; sex: Adult Male; **Taxon:** scientificName: Zelus panamensis; family: Reduviidae; genus: Zelus; scientificNameAuthorship: Zhang and Hart, 2016; **Location:** country: COLOMBIA; stateProvince: Antioquia; locality: San Luis, En Bosque; decimalLatitude: 6.13562; decimalLongitude: -75.32785; georeferenceSources: Google Earth; **Identification:** identifiedBy: G. Zhang; dateIdentified: 2013; **Event:** eventDate: 1986-01-01; **Record Level:** institutionCode: MEFLG
- **Type status:** Paratype. **Occurrence:** catalogNumber: UCR_ENT 00022971; recordedBy: FL Gallego M; sex: Adult Male; **Taxon:** scientificName: Zelus panamensis; family: Reduviidae; genus: Zelus; scientificNameAuthorship: Zhang and Hart, 2016; **Location:** country: COLOMBIA; stateProvince: Antioquia; locality: Mutata, Villa Arteaga; decimalLatitude: 7.25498; decimalLongitude: -76.43018; georeferenceSources: Google Earth; **Identification:** identifiedBy: G. Zhang; dateIdentified: 2013; **Event:** eventDate: 1947-11-01; **Record Level:** institutionCode: MEFLG
- **Type status:** Paratype. **Occurrence:** catalogNumber: UCR_ENT 00022972; recordedBy: R. Velez; sex: Adult Male; **Taxon:** scientificName: Zelus panamensis; family: Reduviidae; genus: Zelus; scientificNameAuthorship: Zhang and Hart, 2016; **Location:** country: COLOMBIA; stateProvince: Antioquia; locality: San Luis, Rio Claro; decimalLatitude: 5.95906; decimalLongitude: -74.9418; georeferenceSources: Google Earth; **Identification:** identifiedBy: G. Zhang; dateIdentified: 2013; **Event:** eventDate: 1988-09-01; **Record Level:** institutionCode: MEFLG
- **Type status:** Paratype. **Occurrence:** catalogNumber: UCR_ENT 00017792; recordedBy: M. Madison; sex: Adult Female; **Taxon:** scientificName: Zelus panamensis; family: Reduviidae; genus: Zelus; scientificNameAuthorship: Zhang and Hart, 2016; **Location:** country: COLOMBIA; stateProvince: Choco; locality: Choco; verbatimElevation: 500; decimalLatitude: 5.75; decimalLongitude: -76.41667; georeferenceSources: Label; **Identification:** identifiedBy: G. Zhang; dateIdentified: 2013; **Event:** eventDate: 1973-04-01; **Record Level:** institutionCode: AMNH
- **Type status:** Paratype. **Occurrence:** catalogNumber: UCR_ENT 00022978; recordedBy: R. Velez; sex: Adult Female; **Taxon:** scientificName: Zelus panamensis; family: Reduviidae; genus: Zelus; scientificNameAuthorship: Zhang and Hart, 2016; **Location:** country: COLOMBIA; stateProvince: Choco; locality: Tutunendo; decimalLatitude: 5.75; decimalLongitude: -76.53333; georeferenceSources: Gazetteer; **Identification:** identifiedBy: G. Zhang; dateIdentified: 2013; **Event:** eventDate: 1983-11-01; **Record Level:** institutionCode: MEFLG
- **Type status:** Paratype. **Occurrence:** catalogNumber: UCR_ENT 00009336; occurrenceRemarks: Drake Collection; recordedBy: A. Diaz; sex: Adult Male; **Taxon:** scientificName: Zelus panamensis; family: Reduviidae; genus: Zelus; scientificNameAuthorship: Zhang and Hart, 2016; **Location:** country: COLOMBIA; stateProvince: Cordoba; locality: Monteria; decimalLatitude: 8.7575; decimalLongitude: -75.89; georeferenceSources: Gazetteer; **Identification:** identifiedBy: G. Zhang; dateIdentified: 2013; **Event:** eventDate: 1985-01-01; **Record Level:** institutionCode: USNM
- **Type status:** Paratype. **Occurrence:** catalogNumber: UCR_ENT 00029351; occurrenceRemarks: Drake Collection. Previously determined to be *Zelus cestartus* [Manuscript name] in Hart (1972)'s dissertation. *Zelus panamensis* and *Zelus cestartus* were considered to be synonymic by G. Zhang.; recordedBy: A. Dizz; sex: Adult Female; **Taxon:** scientificName: Zelus panamensis; family: Reduviidae; genus: Zelus; scientificNameAuthorship: Zhang and Hart, 2016; **Location:** country: COLOMBIA; stateProvince: Cordoba; locality: Monteria; verbatimElevation: 25; decimalLatitude: 8.7575; decimalLongitude: -75.89; georeferenceSources: Google Earth; **Identification:** identifiedBy: G. Zhang; dateIdentified: 2013; **Event:** eventDate: 1983-05-01; **Record Level:** institutionCode: USNM
- **Type status:** Paratype. **Occurrence:** catalogNumber: UCR_ENT 00017032; recordedBy: Unknown; sex: Adult Male; **Taxon:** scientificName: Zelus panamensis; family: Reduviidae; genus: Zelus; scientificNameAuthorship: Zhang and Hart, 2016; **Location:** country: COLOMBIA; stateProvince: unknown; locality: Laguayalana, W Colombia; **Identification:** identifiedBy: G. Zhang; dateIdentified: 2013; **Event:** eventDate: 1956-08-08; **Record Level:** institutionCode: AMNH
- **Type status:** Paratype. **Occurrence:** catalogNumber: UCR_ENT 00014395; recordedBy: F. Quesada; sex: Adult Female; otherCatalogNumbers: CRI001776475; **Taxon:** scientificName: Zelus panamensis; family: Reduviidae; genus: Zelus; scientificNameAuthorship: Zhang and Hart, 2016; **Location:** country: COSTA RICA; stateProvince: Alajuela; locality: Est. San Ramon Oeste; verbatimElevation: 620; decimalLatitude: 10.88327; decimalLongitude: -85.41354; georeferenceSources: Label; **Identification:** identifiedBy: G. Zhang; dateIdentified: 2013; **Event:** eventDate: 1994-04-03 to 1994-04-19; **Record Level:** institutionCode: INBIO
- **Type status:** Paratype. **Occurrence:** catalogNumber: UCR_ENT 00029358; occurrenceRemarks: Designated as allotype by Hart, unpublished. Changed to paratype in Zhang, Hart & Weirauch (2016); recordedBy: Unknown; sex: Adult Female; **Taxon:** scientificName: Zelus panamensis; family: Reduviidae; genus: Zelus; scientificNameAuthorship: Zhang and Hart, 2016; **Location:** country: COSTA RICA; stateProvince: Alajuela; locality: San Carlos; decimalLatitude: 10.324; decimalLongitude: -84.427; georeferenceSources: Google Earth; **Identification:** identifiedBy: G. Zhang; dateIdentified: 2013; **Event:** eventDate: no date provided; **Record Level:** institutionCode: USNM
- **Type status:** Paratype. **Occurrence:** catalogNumber: UCR_ENT 00029359; recordedBy: Unknown; sex: Adult Female; **Taxon:** scientificName: Zelus panamensis; family: Reduviidae; genus: Zelus; scientificNameAuthorship: Zhang and Hart, 2016; **Location:** country: COSTA RICA; stateProvince: Alajuela; locality: San Carlos; decimalLatitude: 10.324; decimalLongitude: -84.427; georeferenceSources: Google Earth; **Identification:** identifiedBy: G. Zhang; dateIdentified: 2013; **Event:** eventDate: no date provided; **Record Level:** institutionCode: USNM
- **Type status:** Paratype. **Occurrence:** catalogNumber: UCR_ENT 00029360; recordedBy: Unknown; sex: Adult Female; **Taxon:** scientificName: Zelus panamensis; family: Reduviidae; genus: Zelus; scientificNameAuthorship: Zhang and Hart, 2016; **Location:** country: COSTA RICA; stateProvince: Alajuela; locality: San Carlos; decimalLatitude: 10.324; decimalLongitude: -84.427; georeferenceSources: Google Earth; **Identification:** identifiedBy: G. Zhang; dateIdentified: 2013; **Event:** eventDate: no date provided; **Record Level:** institutionCode: USNM
- **Type status:** Paratype. **Occurrence:** catalogNumber: UCR_ENT 00029361; recordedBy: Unknown; sex: Adult Female; **Taxon:** scientificName: Zelus panamensis; family: Reduviidae; genus: Zelus; scientificNameAuthorship: Zhang and Hart, 2016; **Location:** country: COSTA RICA; stateProvince: Alajuela; locality: San Carlos; decimalLatitude: 10.324; decimalLongitude: -84.427; georeferenceSources: Google Earth; **Identification:** identifiedBy: G. Zhang; dateIdentified: 2013; **Event:** eventDate: no date provided; **Record Level:** institutionCode: USNM
- **Type status:** Paratype. **Occurrence:** catalogNumber: UCR_ENT 00029362; recordedBy: Unknown; sex: Adult Female; **Taxon:** scientificName: Zelus panamensis; family: Reduviidae; genus: Zelus; scientificNameAuthorship: Zhang and Hart, 2016; **Location:** country: COSTA RICA; stateProvince: Alajuela; locality: San Carlos; decimalLatitude: 10.324; decimalLongitude: -84.427; georeferenceSources: Google Earth; **Identification:** identifiedBy: G. Zhang; dateIdentified: 2013; **Event:** eventDate: no date provided; **Record Level:** institutionCode: USNM
- **Type status:** Paratype. **Occurrence:** catalogNumber: UCR_ENT 00014248; recordedBy: C. Moraga; sex: Adult Female; **Taxon:** scientificName: Zelus panamensis; family: Reduviidae; genus: Zelus; scientificNameAuthorship: Zhang and Hart, 2016; **Location:** country: COSTA RICA; stateProvince: Guanacaste; locality: Estacion Pitilla, 9 Km S Santa Cecilla; verbatimElevation: 700; decimalLatitude: 10.98888; decimalLongitude: -85.42524; georeferenceSources: Label; **Identification:** identifiedBy: G. Zhang; dateIdentified: 2013; **Event:** eventDate: 1994-04-01; **Record Level:** institutionCode: INBIO
- **Type status:** Paratype. **Occurrence:** catalogNumber: UCR_ENT 00014386; recordedBy: Unknown; sex: Adult Male; otherCatalogNumbers: CRI000305674; **Taxon:** scientificName: Zelus panamensis; family: Reduviidae; genus: Zelus; scientificNameAuthorship: Zhang and Hart, 2016; **Location:** country: COSTA RICA; stateProvince: Guanacaste; locality: Estacion Pitilla, 9 Km S Santa Cecilla; verbatimElevation: 700; decimalLatitude: 10.98888; decimalLongitude: -85.42524; georeferenceSources: Label; **Identification:** identifiedBy: G. Zhang; dateIdentified: 2013; **Event:** eventDate: 1905-06-13; **Record Level:** institutionCode: INBIO
- **Type status:** Paratype. **Occurrence:** catalogNumber: UCR_ENT 00014401; recordedBy: P.Rios; sex: Adult Male; otherCatalogNumbers: CRI000336629; **Taxon:** scientificName: Zelus panamensis; family: Reduviidae; genus: Zelus; scientificNameAuthorship: Zhang and Hart, 2016; **Location:** country: COSTA RICA; stateProvince: Guanacaste; locality: Est. Pitilla, 9 km S. Santa Cecilia, P.N. Guanacaste, A.C. Guanacaste; verbatimElevation: 700; decimalLatitude: 10.99261; decimalLongitude: -85.42948; georeferenceSources: Label; **Identification:** identifiedBy: G. Zhang; dateIdentified: 2013; **Event:** eventDate: 1991-07-01; **Record Level:** institutionCode: INBIO
- **Type status:** Paratype. **Occurrence:** catalogNumber: UCR_ENT 00014258; recordedBy: G. Carballo; sex: Adult Male; **Taxon:** scientificName: Zelus panamensis; family: Reduviidae; genus: Zelus; scientificNameAuthorship: Zhang and Hart, 2016; **Location:** country: COSTA RICA; stateProvince: Heredia; locality: Estacion Magsasay Parque Nacional Braullio Carrillo; verbatimElevation: 200; decimalLatitude: 10.39981; decimalLongitude: -84.04828; georeferenceSources: Label; **Identification:** identifiedBy: G. Zhang; dateIdentified: 2013; **Event:** eventDate: 1990-07-01; **Record Level:** institutionCode: INBIO
- **Type status:** Paratype. **Occurrence:** catalogNumber: UCR_ENT 00029350; recordedBy: D. C. Rentz; sex: Adult Female; **Taxon:** scientificName: Zelus panamensis; family: Reduviidae; genus: Zelus; scientificNameAuthorship: Zhang and Hart, 2016; **Location:** country: COSTA RICA; stateProvince: Heredia; locality: Finca La Selva, near Puerto Viejo; verbatimElevation: 48; decimalLatitude: 10.43114; decimalLongitude: -84.00321; georeferenceSources: Google Earth; **Identification:** identifiedBy: G. Zhang; dateIdentified: 2013; **Event:** eventDate: 1973-03-17 to 1973-03-19; **Record Level:** institutionCode: USNM
- **Type status:** Paratype. **Occurrence:** catalogNumber: UCR_ENT 00014239; recordedBy: G. Gallardo; sex: Adult Male; **Taxon:** scientificName: Zelus panamensis; family: Reduviidae; genus: Zelus; scientificNameAuthorship: Zhang and Hart, 2016; **Location:** country: COSTA RICA; stateProvince: Limon; locality: Amubri, Talamanca; verbatimElevation: 70; decimalLatitude: 9.51483; decimalLongitude: -82.95537; georeferenceSources: Label; **Identification:** identifiedBy: G. Zhang; dateIdentified: 2013; **Event:** eventDate: 1992-10-12 to 1992-10-30; **Record Level:** institutionCode: INBIO
- **Type status:** Paratype. **Occurrence:** catalogNumber: UCR_ENT 00014245; recordedBy: G. Gallardo; sex: Adult Female; **Taxon:** scientificName: Zelus panamensis; family: Reduviidae; genus: Zelus; scientificNameAuthorship: Zhang and Hart, 2016; **Location:** country: COSTA RICA; stateProvince: Limon; locality: Amubri, Talamanca; verbatimElevation: 70; decimalLatitude: 9.51483; decimalLongitude: -82.95537; georeferenceSources: Label; **Identification:** identifiedBy: G. Zhang; dateIdentified: 2013; **Event:** eventDate: 1992-10-12 to 1992-10-30; **Record Level:** institutionCode: INBIO
- **Type status:** Paratype. **Occurrence:** catalogNumber: UCR_ENT 00014257; recordedBy: F. Araya; sex: Adult Male; **Taxon:** scientificName: Zelus panamensis; family: Reduviidae; genus: Zelus; scientificNameAuthorship: Zhang and Hart, 2016; **Location:** country: COSTA RICA; stateProvince: Limon; locality: Rio Sardinas, R.N.S.F. Barra del Colorado; verbatimElevation: 10; decimalLatitude: 10.64405; decimalLongitude: -83.74201; georeferenceSources: Label; **Identification:** identifiedBy: G. Zhang; dateIdentified: 2013; **Event:** eventDate: 1994-03-16 to 1994-03-20; **Record Level:** institutionCode: INBIO
- **Type status:** Paratype. **Occurrence:** catalogNumber: UCR_ENT 00014387; recordedBy: G. Carballo; sex: Adult Female; otherCatalogNumbers: CRI001734765; **Taxon:** scientificName: Zelus panamensis; family: Reduviidae; genus: Zelus; scientificNameAuthorship: Zhang and Hart, 2016; **Location:** country: COSTA RICA; stateProvince: Limon; locality: R. B. Hitoy Cerere, Valle La Estrella; verbatimElevation: 150; decimalLatitude: 9.67177; decimalLongitude: -83.0277; georeferenceSources: Label; **Identification:** identifiedBy: G. Zhang; dateIdentified: 2013; **Event:** eventDate: 1994-02-21 to 1994-03-08; **Record Level:** institutionCode: INBIO
- **Type status:** Paratype. **Occurrence:** catalogNumber: UCR_ENT 00014391; recordedBy: E. Araya; sex: Adult Female; otherCatalogNumbers: CRI001833288; **Taxon:** scientificName: Zelus panamensis; family: Reduviidae; genus: Zelus; scientificNameAuthorship: Zhang and Hart, 2016; **Location:** country: COSTA RICA; stateProvince: Limon; locality: Rio Sardinas, R.N.S.F. Barra del Colorado; verbatimElevation: 10; decimalLatitude: 10.64405; decimalLongitude: -83.74201; georeferenceSources: Label; **Identification:** identifiedBy: G. Zhang; dateIdentified: 2013; **Event:** eventDate: 1994-02-01 to 1994-02-14; **Record Level:** institutionCode: INBIO
- **Type status:** Paratype. **Occurrence:** catalogNumber: UCR_ENT 00014393; recordedBy: M. Epstein; sex: Adult Female; otherCatalogNumbers: INB0003801591; **Taxon:** scientificName: Zelus panamensis; family: Reduviidae; genus: Zelus; scientificNameAuthorship: Zhang and Hart, 2016; **Location:** country: COSTA RICA; stateProvince: Limon; locality: Sector Cerro Cocori, Finca de E. Rojas; verbatimElevation: 150; decimalLatitude: 10.59281; decimalLongitude: -83.71456; georeferenceSources: Label; **Identification:** identifiedBy: G. Zhang; dateIdentified: 2013; **Event:** eventDate: 1994-02-24; **Record Level:** institutionCode: INBIO
- **Type status:** Paratype. **Occurrence:** catalogNumber: UCR_ENT 00014398; recordedBy: G. Carballo; sex: Adult Female; otherCatalogNumbers: CRI000454403; **Taxon:** scientificName: Zelus panamensis; family: Reduviidae; genus: Zelus; scientificNameAuthorship: Zhang and Hart, 2016; **Location:** country: COSTA RICA; stateProvince: Limon; locality: Estacion Hitoy Cerere, R. Cerere, Res. Biol. Hitoy Cerere; verbatimElevation: 100; decimalLatitude: 9.67177; decimalLongitude: -83.0277; georeferenceSources: Label; **Identification:** identifiedBy: G. Zhang; dateIdentified: 2013; **Event:** eventDate: 1992-03-05 to 1992-03-19; **Record Level:** institutionCode: INBIO
- **Type status:** Paratype. **Occurrence:** catalogNumber: UCR_ENT 00014399; recordedBy: G. Carballo; sex: Adult Female; otherCatalogNumbers: CRI000374162; **Taxon:** scientificName: Zelus panamensis; family: Reduviidae; genus: Zelus; scientificNameAuthorship: Zhang and Hart, 2016; **Location:** country: COSTA RICA; stateProvince: Limon; locality: Estacion Hitoy Cerere, R. Cerere, Res. Biol. Hitoy Cerere; verbatimElevation: 100; decimalLatitude: 9.67177; decimalLongitude: -83.0277; georeferenceSources: Label; **Identification:** identifiedBy: G. Zhang; dateIdentified: 2013; **Event:** eventDate: 1992-03-27 to 1992-04-13; **Record Level:** institutionCode: INBIO
- **Type status:** Paratype. **Occurrence:** catalogNumber: UCR_ENT 00014400; recordedBy: G. Carballo; sex: Adult Female; otherCatalogNumbers: CRI000374161; **Taxon:** scientificName: Zelus panamensis; family: Reduviidae; genus: Zelus; scientificNameAuthorship: Zhang and Hart, 2016; **Location:** country: COSTA RICA; stateProvince: Limon; locality: Estacion Hitoy Cerere, R. Cerere, Res. Biol. Hitoy Cerere; verbatimElevation: 100; decimalLatitude: 9.67177; decimalLongitude: -83.0277; georeferenceSources: Label; **Identification:** identifiedBy: G. Zhang; dateIdentified: 2013; **Event:** eventDate: 1992-03-27 to 1992-04-13; **Record Level:** institutionCode: INBIO
- **Type status:** Paratype. **Occurrence:** catalogNumber: UCR_ENT 00014402; occurrenceRemarks: Information on label: colecta libre; recordedBy: R. Villalobos; sex: Adult Male; otherCatalogNumbers: INB000416412; **Taxon:** scientificName: Zelus panamensis; family: Reduviidae; genus: Zelus; scientificNameAuthorship: Zhang and Hart, 2016; **Location:** country: COSTA RICA; stateProvince: Limon; locality: Veragua Rainforest, Rio Victoria (Returu Station); verbatimElevation: 250; **Identification:** identifiedBy: G. Zhang; dateIdentified: 2013; **Event:** eventDate: 2008-08-01; **Record Level:** institutionCode: INBIO
- **Type status:** Paratype. **Occurrence:** catalogNumber: UCR_ENT 00014240; recordedBy: A. Gutierrez; sex: Adult Male; **Taxon:** scientificName: Zelus panamensis; family: Reduviidae; genus: Zelus; scientificNameAuthorship: Zhang and Hart, 2016; **Location:** country: COSTA RICA; stateProvince: Puntarenas; locality: Rancho Quemado, Pen. de Osa, A. C. Osa; verbatimElevation: 200; decimalLatitude: 8.6791; decimalLongitude: -83.56671; **Identification:** identifiedBy: G. Zhang; dateIdentified: 2013; **Event:** eventDate: 1993-07-04 to 1993-07-28; **Record Level:** institutionCode: INBIO
- **Type status:** Paratype. **Occurrence:** catalogNumber: UCR_ENT 00014241; recordedBy: G. Varela; sex: Adult Male; **Taxon:** scientificName: Zelus panamensis; family: Reduviidae; genus: Zelus; scientificNameAuthorship: Zhang and Hart, 2016; **Location:** country: COSTA RICA; stateProvince: Puntarenas; locality: Parque Nacional Manuel Antonio, Quepos; verbatimElevation: 80; decimalLatitude: 9.39361; decimalLongitude: -84.12917; **Identification:** identifiedBy: G. Zhang; dateIdentified: 2013; **Event:** eventDate: 1992-04-01; **Record Level:** institutionCode: INBIO
- **Type status:** Paratype. **Occurrence:** catalogNumber: UCR_ENT 00014242; recordedBy: G. Varela; sex: Adult Male; **Taxon:** scientificName: Zelus panamensis; family: Reduviidae; genus: Zelus; scientificNameAuthorship: Zhang and Hart, 2016; **Location:** country: COSTA RICA; stateProvince: Puntarenas; locality: Parque Nacional Manuel Antonio, Quepos; verbatimElevation: 80; decimalLatitude: 9.39361; decimalLongitude: -84.12917; **Identification:** identifiedBy: G. Zhang; dateIdentified: 2013; **Event:** eventDate: 1992-10-01; **Record Level:** institutionCode: INBIO
- **Type status:** Paratype. **Occurrence:** catalogNumber: UCR_ENT 00014244; recordedBy: G. Rodriguez; sex: Adult Female; **Taxon:** scientificName: Zelus panamensis; family: Reduviidae; genus: Zelus; scientificNameAuthorship: Zhang and Hart, 2016; **Location:** country: COSTA RICA; stateProvince: Puntarenas; locality: Estacion Sirena, Parque Nacional Corcovado; verbatimElevation: 50; decimalLatitude: 8.48005; decimalLongitude: -83.58935; georeferenceSources: Google Earth; **Identification:** identifiedBy: G. Zhang; dateIdentified: 2013; **Event:** eventDate: 1992-04-01; **Record Level:** institutionCode: INBIO
- **Type status:** Paratype. **Occurrence:** catalogNumber: UCR_ENT 00014246; recordedBy: F. Quesada; sex: Adult Female; **Taxon:** scientificName: Zelus panamensis; family: Reduviidae; genus: Zelus; scientificNameAuthorship: Zhang and Hart, 2016; **Location:** country: COSTA RICA; stateProvince: Puntarenas; locality: Rancho Quemado, Peninsula de Osa; verbatimElevation: 200; decimalLatitude: 8.67776; decimalLongitude: -83.56478; georeferenceSources: Label; **Identification:** identifiedBy: G. Zhang; dateIdentified: 2013; **Event:** eventDate: 1992-09-01; **Record Level:** institutionCode: INBIO
- **Type status:** Paratype. **Occurrence:** catalogNumber: UCR_ENT 00014247; recordedBy: M. Segura; sex: Adult Female; **Taxon:** scientificName: Zelus panamensis; family: Reduviidae; genus: Zelus; scientificNameAuthorship: Zhang and Hart, 2016; **Location:** country: COSTA RICA; stateProvince: Puntarenas; locality: Rancho Quemado, Peninsula de Osa; verbatimElevation: 200; decimalLatitude: 8.67776; decimalLongitude: -83.56478; georeferenceSources: Label; **Identification:** identifiedBy: G. Zhang; dateIdentified: 2013; **Event:** eventDate: 1992-08-01; **Record Level:** institutionCode: INBIO
- **Type status:** Paratype. **Occurrence:** catalogNumber: UCR_ENT 00014359; recordedBy: F. Quesada; sex: Adult Male; otherCatalogNumbers: INBIO CRI000 905864; **Taxon:** scientificName: Zelus panamensis; family: Reduviidae; genus: Zelus; scientificNameAuthorship: Zhang and Hart, 2016; **Location:** country: COSTA RICA; stateProvince: Puntarenas; locality: Rancho Quemado, Peninsula de Osa; verbatimElevation: 200; decimalLatitude: 8.67776; decimalLongitude: -83.56478; georeferenceSources: Label; **Identification:** identifiedBy: G. Zhang; dateIdentified: 2013; **Event:** eventDate: 1992-12-01; **Record Level:** institutionCode: INBIO
- **Type status:** Paratype. **Occurrence:** catalogNumber: UCR_ENT 00014382; recordedBy: G. Varela; sex: Adult Male; otherCatalogNumbers: CRI000823105; **Taxon:** scientificName: Zelus panamensis; family: Reduviidae; genus: Zelus; scientificNameAuthorship: Zhang and Hart, 2016; **Location:** country: COSTA RICA; stateProvince: Puntarenas; locality: Parque Nacional Manuel Antonio, Quepos; verbatimElevation: 80; decimalLatitude: 9.39361; decimalLongitude: -84.12917; **Identification:** identifiedBy: G. Zhang; dateIdentified: 2013; **Event:** eventDate: 1992-11-01; **Record Level:** institutionCode: INBIO
- **Type status:** Paratype. **Occurrence:** catalogNumber: UCR_ENT 00014383; recordedBy: F. Quesada; sex: Adult Male; otherCatalogNumbers: CRI000552719; **Taxon:** scientificName: Zelus panamensis; family: Reduviidae; genus: Zelus; scientificNameAuthorship: Zhang and Hart, 2016; **Location:** country: COSTA RICA; stateProvince: Puntarenas; locality: Rancho Quemado, Peninsula de Osa; verbatimElevation: 200; decimalLatitude: 8.67776; decimalLongitude: -83.56478; georeferenceSources: Label; **Identification:** identifiedBy: G. Zhang; dateIdentified: 2013; **Event:** eventDate: 1991-11-01; **Record Level:** institutionCode: INBIO
- **Type status:** Paratype. **Occurrence:** catalogNumber: UCR_ENT 00014384; recordedBy: G. Fonseca; sex: Adult Male; otherCatalogNumbers: CRI001834042; **Taxon:** scientificName: Zelus panamensis; family: Reduviidae; genus: Zelus; scientificNameAuthorship: Zhang and Hart, 2016; **Location:** country: COSTA RICA; stateProvince: Puntarenas; locality: Estacion Sirena, Parque Nacional Corcovado; verbatimElevation: 50; decimalLatitude: 8.48005; decimalLongitude: -83.58935; georeferenceSources: Google Earth; **Identification:** identifiedBy: G. Zhang; dateIdentified: 2013; **Event:** eventDate: 1993-06-01; **Record Level:** institutionCode: INBIO
- **Type status:** Paratype. **Occurrence:** catalogNumber: UCR_ENT 00014385; recordedBy: A. Marin; sex: Adult Male; otherCatalogNumbers: CRI001690200; **Taxon:** scientificName: Zelus panamensis; family: Reduviidae; genus: Zelus; scientificNameAuthorship: Zhang and Hart, 2016; **Location:** country: COSTA RICA; stateProvince: Puntarenas; locality: Estero de Guerra, Peninsula de Osa; verbatimElevation: 50; **Identification:** identifiedBy: G. Zhang; dateIdentified: 2013; **Event:** eventDate: 1993-01-01 to 1993-01-14; **Record Level:** institutionCode: INBIO
- **Type status:** Paratype. **Occurrence:** catalogNumber: UCR_ENT 00014388; recordedBy: D. H. Janzen; sex: Adult Female; otherCatalogNumbers: CRI001686899; **Taxon:** scientificName: Zelus panamensis; family: Reduviidae; genus: Zelus; scientificNameAuthorship: Zhang and Hart, 2016; **Location:** country: COSTA RICA; stateProvince: Puntarenas; locality: Corcovado National Park Osa Peninsula; decimalLatitude: 8.55516; decimalLongitude: -83.51755; georeferenceSources: Google Earth; **Identification:** identifiedBy: G. Zhang; dateIdentified: 2013; **Event:** eventDate: 1997-08-02 to 1997-08-04; **Record Level:** institutionCode: INBIO
- **Type status:** Paratype. **Occurrence:** catalogNumber: UCR_ENT 00014389; recordedBy: B. Gamboa; sex: Adult Female; otherCatalogNumbers: INB0003836872; **Taxon:** scientificName: Zelus panamensis; family: Reduviidae; genus: Zelus; scientificNameAuthorship: Zhang and Hart, 2016; **Location:** country: COSTA RICA; stateProvince: Puntarenas; locality: Golfito, Camino a las Torres; verbatimElevation: 450; **Identification:** identifiedBy: G. Zhang; dateIdentified: 2013; **Event:** eventDate: 2004-04-28; **Record Level:** institutionCode: INBIO
- **Type status:** Paratype. **Occurrence:** catalogNumber: UCR_ENT 00014390; recordedBy: D. Briceno; sex: Adult Female; otherCatalogNumbers: INB0003838066; **Taxon:** scientificName: Zelus panamensis; family: Reduviidae; genus: Zelus; scientificNameAuthorship: Zhang and Hart, 2016; **Location:** country: COSTA RICA; stateProvince: Puntarenas; locality: Golfito, Camino a las Torres; verbatimElevation: 450; **Identification:** identifiedBy: G. Zhang; dateIdentified: 2013; **Event:** eventDate: 2004-04-28; **Record Level:** institutionCode: INBIO
- **Type status:** Paratype. **Occurrence:** catalogNumber: UCR_ENT 00014392; recordedBy: G. Varela; sex: Adult Female; otherCatalogNumbers: CRI000941360; **Taxon:** scientificName: Zelus panamensis; family: Reduviidae; genus: Zelus; scientificNameAuthorship: Zhang and Hart, 2016; **Location:** country: COSTA RICA; stateProvince: Puntarenas; locality: Parque Nacional Manuel Antonio, Quepos; verbatimElevation: 80; decimalLatitude: 9.39361; decimalLongitude: -84.12917; **Identification:** identifiedBy: G. Zhang; dateIdentified: 2013; **Event:** eventDate: 1992-08-01; **Record Level:** institutionCode: INBIO
- **Type status:** Paratype. **Occurrence:** catalogNumber: UCR_ENT 00014394; recordedBy: M. Moraga; sex: Adult Female; otherCatalogNumbers: INB0003838274; **Taxon:** scientificName: Zelus panamensis; family: Reduviidae; genus: Zelus; scientificNameAuthorship: Zhang and Hart, 2016; **Location:** country: COSTA RICA; stateProvince: Puntarenas; locality: Golfito, Camino a las Torres; verbatimElevation: 450; **Identification:** identifiedBy: G. Zhang; dateIdentified: 2013; **Event:** eventDate: 2004-04-28; **Record Level:** institutionCode: INBIO
- **Type status:** Paratype. **Occurrence:** catalogNumber: UCR_ENT 00014396; recordedBy: B. Gamboa; sex: Adult Female; otherCatalogNumbers: CRI002188028; **Taxon:** scientificName: Zelus panamensis; family: Reduviidae; genus: Zelus; scientificNameAuthorship: Zhang and Hart, 2016; **Location:** country: COSTA RICA; stateProvince: Puntarenas; locality: Estacion Sirena, ACOSA; verbatimElevation: 50; decimalLatitude: 8.48005; decimalLongitude: -83.58946; georeferenceSources: Google Earth; **Identification:** identifiedBy: G. Zhang; dateIdentified: 2013; **Event:** eventDate: 1995-04-05 to 1995-04-24; **Record Level:** institutionCode: INBIO
- **Type status:** Paratype. **Occurrence:** catalogNumber: UCR_ENT 00014397; recordedBy: G. Rodriguez; sex: Adult Female; otherCatalogNumbers: CRI000545451; **Taxon:** scientificName: Zelus panamensis; family: Reduviidae; genus: Zelus; scientificNameAuthorship: Zhang and Hart, 2016; **Location:** country: COSTA RICA; stateProvince: Puntarenas; locality: Estacion Sirena, Parque Nacional Corcovado; verbatimElevation: 50; decimalLatitude: 8.48005; decimalLongitude: -83.58935; georeferenceSources: Google Earth; **Identification:** identifiedBy: G. Zhang; dateIdentified: 2013; **Event:** eventDate: 1992-04-01; **Record Level:** institutionCode: INBIO
- **Type status:** Paratype. **Occurrence:** catalogNumber: UCR_ENT 00014456; recordedBy: C. Cano; sex: Adult Female; otherCatalogNumbers: INBIOCRI001718402; **Taxon:** scientificName: Zelus panamensis; family: Reduviidae; genus: Zelus; scientificNameAuthorship: Zhang and Hart, 2016; **Location:** country: COSTA RICA; stateProvince: Puntarenas; locality: Parque Nacional Manuel Antonio, Quepos; verbatimElevation: 80; decimalLatitude: 9.39361; decimalLongitude: -84.12917; **Identification:** identifiedBy: G. Zhang; dateIdentified: 2013; **Event:** eventDate: 1992-04-01; **Record Level:** institutionCode: INBIO
- **Type status:** Paratype. **Occurrence:** catalogNumber: UCR_ENT 00022662; recordedBy: E. Giesbert; sex: Adult Male; otherCatalogNumbers: LACM ENT 264633; **Taxon:** scientificName: Zelus panamensis; family: Reduviidae; genus: Zelus; scientificNameAuthorship: Zhang and Hart, 2016; **Location:** country: COSTA RICA; stateProvince: Puntarenas; locality: Rincon, Osa Peninsula; verbatimElevation: 1; decimalLatitude: 8.7; decimalLongitude: -83.4833; georeferenceSources: Gazetteer; **Identification:** identifiedBy: G. Zhang; dateIdentified: 2013; **Event:** eventDate: 1974-05-25 to 1974-05-28; **Record Level:** institutionCode: LACM
- **Type status:** Paratype. **Occurrence:** catalogNumber: UCR_ENT 00022666; recordedBy: Truxal & Menke; sex: Adult Female; otherCatalogNumbers: LACM ENT 264680; **Taxon:** scientificName: Zelus panamensis; family: Reduviidae; genus: Zelus; scientificNameAuthorship: Zhang and Hart, 2016; **Location:** country: COSTA RICA; stateProvince: Puntarenas; locality: Golfito; decimalLatitude: 8.6407; decimalLongitude: -83.1686; georeferenceSources: Google Earth; **Identification:** identifiedBy: G. Zhang; dateIdentified: 2013; **Event:** eventDate: 1957-07-30; **Record Level:** institutionCode: LACM
- **Type status:** Paratype. **Occurrence:** catalogNumber: UCR_ENT 00072669; recordedBy: Unknown; sex: Adult Male; **Taxon:** scientificName: Zelus panamensis; family: Reduviidae; genus: Zelus; scientificNameAuthorship: Zhang and Hart, 2016; **Location:** country: COSTA RICA; stateProvince: Puntarenas; locality: Hacienda Baru near Dominical; **Identification:** identifiedBy: G. Zhang; dateIdentified: 2013; **Event:** eventDate: 2004-02-18; **Record Level:** institutionCode: UCR
- **Type status:** Paratype. **Occurrence:** catalogNumber: UCR_ENT 00071200; recordedBy: W.C.M.; sex: Adult Male; **Taxon:** scientificName: Zelus panamensis; family: Reduviidae; genus: Zelus; scientificNameAuthorship: Zhang and Hart, 2016; **Location:** country: ECUADOR; stateProvince: Manabi; locality: Cojimies; decimalLatitude: 0.36667; decimalLongitude: -80.03333; georeferenceSources: Gazetteer; **Identification:** identifiedBy: G. Zhang; dateIdentified: 2013; **Event:** eventDate: 1947-11-09; **Record Level:** institutionCode: TAMU
- **Type status:** Paratype. **Occurrence:** catalogNumber: UCR_ENT 00009574; occurrenceRemarks: Drake Collection; recordedBy: J. J. Anderson; sex: Adult Male; **Taxon:** scientificName: Zelus panamensis; family: Reduviidae; genus: Zelus; scientificNameAuthorship: Zhang and Hart, 2016; **Location:** country: ECUADOR; stateProvince: Pichincha; locality: Puerto Quito; decimalLatitude: 0.1167; decimalLongitude: -79.2667; georeferenceSources: Gazetteer; **Identification:** identifiedBy: G. Zhang; dateIdentified: 2013; **Event:** eventDate: 1978-02-01 to 1978-02-05; **Record Level:** institutionCode: USNM
- **Type status:** Paratype. **Occurrence:** catalogNumber: UCR_ENT 00071215; recordedBy: J. C. Schaffner; sex: Adult Male; **Taxon:** scientificName: Zelus panamensis; family: Reduviidae; genus: Zelus; scientificNameAuthorship: Zhang and Hart, 2016; **Location:** country: PANAMA; stateProvince: Bocas del Toro; locality: 2 km WSW Chiriqui Grande; decimalLatitude: 8.94583; decimalLongitude: -82.13694; **Identification:** identifiedBy: G. Zhang; dateIdentified: 2013; **Event:** eventDate: 1999-08-06; **Record Level:** institutionCode: TAMU
- **Type status:** Paratype. **Occurrence:** catalogNumber: UCR_ENT 00009288; recordedBy: S. W. Frost; sex: Adult Male; **Taxon:** scientificName: Zelus panamensis; family: Reduviidae; genus: Zelus; scientificNameAuthorship: Zhang and Hart, 2016; **Location:** country: PANAMA; stateProvince: Canal Zone; locality: Rio Indio; **Identification:** identifiedBy: G. Zhang; dateIdentified: 2013; **Event:** eventDate: 1936-12-27; **Record Level:** institutionCode: USNM
- **Type status:** Paratype. **Occurrence:** catalogNumber: UCR_ENT 00017034; recordedBy: H. D. Engleman; sex: Adult Male; **Taxon:** scientificName: Zelus panamensis; family: Reduviidae; genus: Zelus; scientificNameAuthorship: Zhang and Hart, 2016; **Location:** country: PANAMA; stateProvince: Canal Zone; locality: Pacific Slope; **Identification:** identifiedBy: G. Zhang; dateIdentified: 2013; **Event:** eventDate: 1974-09-04; **Record Level:** institutionCode: AMNH
- **Type status:** Paratype. **Occurrence:** catalogNumber: UCR_ENT 00017035; recordedBy: Engleman; sex: Adult Male; **Taxon:** scientificName: Zelus panamensis; family: Reduviidae; genus: Zelus; scientificNameAuthorship: Zhang and Hart, 2016; **Location:** country: PANAMA; stateProvince: Canal Zone; locality: Coco Solo Hospital; decimalLatitude: 9.35; decimalLongitude: -79.85; georeferenceSources: Label; **Identification:** identifiedBy: G. Zhang; dateIdentified: 2013; **Event:** eventDate: 1972-03-09; **Record Level:** institutionCode: AMNH
- **Type status:** Paratype. **Occurrence:** catalogNumber: UCR_ENT 00017036; recordedBy: D. Engleman; sex: Adult Male; **Taxon:** scientificName: Zelus panamensis; family: Reduviidae; genus: Zelus; scientificNameAuthorship: Zhang and Hart, 2016; **Location:** country: PANAMA; stateProvince: Canal Zone; locality: Pipeline Road; **Identification:** identifiedBy: G. Zhang; dateIdentified: 2013; **Event:** eventDate: 1974-06-30; **Record Level:** institutionCode: AMNH
- **Type status:** Paratype. **Occurrence:** catalogNumber: UCR_ENT 00017037; recordedBy: D. Engleman; sex: Adult Male; **Taxon:** scientificName: Zelus panamensis; family: Reduviidae; genus: Zelus; scientificNameAuthorship: Zhang and Hart, 2016; **Location:** country: PANAMA; stateProvince: Canal Zone; locality: Gatun Spillway; decimalLatitude: 9.35055; decimalLongitude: -79.83145; georeferenceSources: Google Earth; **Identification:** identifiedBy: G. Zhang; dateIdentified: 2013; **Event:** eventDate: 1971-07-12; **Record Level:** institutionCode: AMNH
- **Type status:** Paratype. **Occurrence:** catalogNumber: UCR_ENT 00017237; recordedBy: H. A. Hespenheide; sex: Adult Female; **Taxon:** scientificName: Zelus panamensis; family: Reduviidae; genus: Zelus; scientificNameAuthorship: Zhang and Hart, 2016; **Location:** country: PANAMA; stateProvince: Canal Zone; locality: Barro Colorado Island; decimalLatitude: 9.15562; decimalLongitude: -79.84895; georeferenceSources: Google Earth; **Identification:** identifiedBy: G. Zhang; dateIdentified: 2013; **Event:** eventDate: 1978-08-14; **Record Level:** institutionCode: AMNH
- **Type status:** Paratype. **Occurrence:** catalogNumber: UCR_ENT 00017793; recordedBy: D. Engleman; sex: Adult Female; **Taxon:** scientificName: Zelus panamensis; family: Reduviidae; genus: Zelus; scientificNameAuthorship: Zhang and Hart, 2016; **Location:** country: PANAMA; stateProvince: Canal Zone; locality: Escobal Road; decimalLatitude: 9.21739; decimalLongitude: -79.95576; georeferenceSources: Google Earth; **Identification:** identifiedBy: G. Zhang; dateIdentified: 2013; **Event:** eventDate: 1974-07-14; **Record Level:** institutionCode: AMNH
- **Type status:** Paratype. **Occurrence:** catalogNumber: UCR_ENT 00038443; recordedBy: H. P. Stockwell; sex: Adult Male; **Taxon:** scientificName: Zelus panamensis; family: Reduviidae; genus: Zelus; scientificNameAuthorship: Zhang and Hart, 2016; **Location:** country: PANAMA; stateProvince: Canal Zone; locality: 5 mi. NW of Gamboa; decimalLatitude: 9.1629; decimalLongitude: -79.74871; georeferenceSources: Google Earth; **Identification:** identifiedBy: G. Zhang; dateIdentified: 2013; **Event:** eventDate: 1974-05-01; **Record Level:** institutionCode: UCB
- **Type status:** Paratype. **Occurrence:** catalogNumber: UCR_ENT 00038451; recordedBy: D. Engleman; sex: Adult Female; **Taxon:** scientificName: Zelus panamensis; family: Reduviidae; genus: Zelus; scientificNameAuthorship: Zhang and Hart, 2016; **Location:** country: PANAMA; stateProvince: Canal Zone; locality: Coco Solo Hospital; decimalLatitude: 9.35; decimalLongitude: -79.85; georeferenceSources: Label; **Identification:** identifiedBy: G. Zhang; dateIdentified: 2013; **Event:** eventDate: 1973-10-14; **Record Level:** institutionCode: UCB
- **Type status:** Paratype. **Occurrence:** catalogNumber: UCR_ENT 00042954; recordedBy: D. Engleman; sex: Adult Male; **Taxon:** scientificName: Zelus panamensis; family: Reduviidae; genus: Zelus; scientificNameAuthorship: Zhang and Hart, 2016; **Location:** country: PANAMA; stateProvince: Canal Zone; locality: Pina Road; decimalLatitude: 9.25; decimalLongitude: -79.95; georeferenceSources: Label; **Identification:** identifiedBy: G. Zhang; dateIdentified: 2013; **Event:** eventDate: 1973-01-30; **Record Level:** institutionCode: CSUC
- **Type status:** Paratype. **Occurrence:** catalogNumber: UCR_ENT 00042955; recordedBy: Strauch; sex: Adult Female; **Taxon:** scientificName: Zelus panamensis; family: Reduviidae; genus: Zelus; scientificNameAuthorship: Zhang and Hart, 2016; **Location:** country: PANAMA; stateProvince: Canal Zone; locality: 5 mi NW of Gamboa; **Identification:** identifiedBy: G. Zhang; dateIdentified: 2013; **Event:** eventDate: 1973-01-30; **Record Level:** institutionCode: CSUC
- **Type status:** Paratype. **Occurrence:** catalogNumber: UCR_ENT 00047063; recordedBy: H. A. Hespenheide; sex: Adult Male; **Taxon:** scientificName: Zelus panamensis; family: Reduviidae; genus: Zelus; scientificNameAuthorship: Zhang and Hart, 2016; **Location:** country: PANAMA; stateProvince: Canal Zone; locality: 7 km W Margarita; decimalLatitude: 9.33333; decimalLongitude: -79.96666; georeferenceSources: Label; **Identification:** identifiedBy: G. Zhang; dateIdentified: 2013; **Event:** eventDate: 1978-07-29; **Record Level:** institutionCode: AMNH
- **Type status:** Paratype. **Occurrence:** catalogNumber: UCR_ENT 00047960; recordedBy: K. E. Frick; sex: Adult Male; **Taxon:** scientificName: Zelus panamensis; family: Reduviidae; genus: Zelus; scientificNameAuthorship: Zhang and Hart, 2016; **Location:** country: PANAMA; stateProvince: Canal Zone; locality: Fort Clayton; verbatimElevation: 419; decimalLatitude: 9; decimalLongitude: -79.75; georeferenceSources: Gazetteer; **Identification:** identifiedBy: G. Zhang; dateIdentified: 2013; **Event:** eventDate: 1944-04-01; **Record Level:** institutionCode: CAS
- **Type status:** Paratype. **Occurrence:** catalogNumber: UCR_ENT 00047961; recordedBy: K. E. Frick; sex: Adult Male; **Taxon:** scientificName: Zelus panamensis; family: Reduviidae; genus: Zelus; scientificNameAuthorship: Zhang and Hart, 2016; **Location:** country: PANAMA; stateProvince: Canal Zone; locality: Fort Clayton; verbatimElevation: 419; decimalLatitude: 9; decimalLongitude: -79.75; georeferenceSources: Gazetteer; **Identification:** identifiedBy: G. Zhang; dateIdentified: 2013; **Event:** eventDate: 1944-04-01; **Record Level:** institutionCode: CAS
- **Type status:** Paratype. **Occurrence:** catalogNumber: UCR_ENT 00017791; recordedBy: D. Engleman; sex: Adult Female; **Taxon:** scientificName: Zelus panamensis; family: Reduviidae; genus: Zelus; scientificNameAuthorship: Zhang and Hart, 2016; **Location:** country: PANAMA; stateProvince: Colon; locality: Pipeline Road, Gamboa; decimalLatitude: 9.1167; decimalLongitude: -79.7; georeferenceSources: Gazetteer; **Identification:** identifiedBy: G. Zhang; dateIdentified: 2013; **Event:** eventDate: 1972-07-22; **Record Level:** institutionCode: AMNH
- **Type status:** Paratype. **Occurrence:** catalogNumber: UCR_ENT 00029364; recordedBy: K. W. Cooper; sex: Adult Female; **Taxon:** scientificName: Zelus panamensis; family: Reduviidae; genus: Zelus; scientificNameAuthorship: Zhang and Hart, 2016; **Location:** country: PANAMA; stateProvince: Colon; locality: Barro Colorado Island, Canal Zone; decimalLatitude: 9.15472; decimalLongitude: -79.84806; georeferenceSources: GeoLocate Software; **Identification:** identifiedBy: G. Zhang; dateIdentified: 2013; **Event:** eventDate: 1964-12-11 to 1964-12-17; **Record Level:** institutionCode: USNM
- **Type status:** Paratype. **Occurrence:** catalogNumber: UCR_ENT 00070010; recordedBy: C. D. Michener; sex: Adult Male; **Taxon:** scientificName: Zelus panamensis; family: Reduviidae; genus: Zelus; scientificNameAuthorship: Zhang and Hart, 2016; **Location:** country: PANAMA; stateProvince: Colon; locality: Pipeline Road, 8 km NW Gamboa; **Identification:** identifiedBy: G. Zhang; dateIdentified: 2013; **Event:** eventDate: 1981-01-12; **Record Level:** institutionCode: KU
- **Type status:** Paratype. **Occurrence:** catalogNumber: UCR_ENT 00070029; recordedBy: C. D. Michener; sex: Adult Female; **Taxon:** scientificName: Zelus panamensis; family: Reduviidae; genus: Zelus; scientificNameAuthorship: Zhang and Hart, 2016; **Location:** country: PANAMA; stateProvince: Colon; locality: Pipeline Road, 8 km NW Gamboa; **Identification:** identifiedBy: G. Zhang; dateIdentified: 2013; **Event:** eventDate: 1981-01-12; **Record Level:** institutionCode: KU
- **Type status:** Paratype. **Occurrence:** catalogNumber: UCR_ENT 00017038; recordedBy: D. Engleman; sex: Adult Male; **Taxon:** scientificName: Zelus panamensis; family: Reduviidae; genus: Zelus; scientificNameAuthorship: Zhang and Hart, 2016; **Location:** country: PANAMA; stateProvince: Panama; locality: Madden Forest, Canal Zone; decimalLatitude: 9.08333; decimalLongitude: -79.58333; georeferenceSources: Label; **Identification:** identifiedBy: G. Zhang; dateIdentified: 2013; **Event:** eventDate: 1972-10-23; **Record Level:** institutionCode: AMNH
- **Type status:** Paratype. **Occurrence:** catalogNumber: UCR_ENT 00037118; recordedBy: N. Smith, R. Kassabian; sex: Adult Male; **Taxon:** scientificName: Zelus panamensis; family: Reduviidae; genus: Zelus; scientificNameAuthorship: Zhang and Hart, 2016; **Location:** country: PANAMA; stateProvince: Panama; locality: Old Gamboa Road; **Identification:** identifiedBy: G. Zhang; dateIdentified: 2013; **Event:** eventDate: 1994-06-25; **Record Level:** institutionCode: UCD
- **Type status:** Paratype. **Occurrence:** catalogNumber: UCR_ENT 00046989; recordedBy: D. Engleman; sex: Adult Male; **Taxon:** scientificName: Zelus panamensis; family: Reduviidae; genus: Zelus; scientificNameAuthorship: Zhang and Hart, 2016; **Location:** country: PANAMA; stateProvince: Panama; locality: Madden Forest, Canal Zone; decimalLatitude: 9.08333; decimalLongitude: -79.58333; georeferenceSources: Label; **Identification:** identifiedBy: G. Zhang; dateIdentified: 2013; **Event:** eventDate: 1980-12-20; **Record Level:** institutionCode: AMNH
- **Type status:** Paratype. **Occurrence:** catalogNumber: UCR_ENT 00047959; recordedBy: E. S. Ross; sex: Adult Male; **Taxon:** scientificName: Zelus panamensis; family: Reduviidae; genus: Zelus; scientificNameAuthorship: Zhang and Hart, 2016; **Location:** country: PANAMA; stateProvince: Panama; locality: Pipeline Road near Gamboa, Canal Zone; decimalLatitude: 9.11811; decimalLongitude: -79.70019; georeferenceSources: Google Earth; **Identification:** identifiedBy: G. Zhang; dateIdentified: 2013; **Event:** eventDate: 1987-02-09; **Record Level:** institutionCode: CAS
- **Type status:** Paratype. **Occurrence:** catalogNumber: UCR_ENT 00017033; recordedBy: N. L. H. Krauss; sex: Adult Male; **Taxon:** scientificName: Zelus panamensis; family: Reduviidae; genus: Zelus; scientificNameAuthorship: Zhang and Hart, 2016; **Location:** country: PANAMA; stateProvince: Panama; locality: Summit; decimalLatitude: 9.04697; decimalLongitude: -79.64056; georeferenceSources: Google Earth; **Identification:** identifiedBy: G. Zhang; dateIdentified: 2013; **Event:** eventDate: 1953-12-01; **Record Level:** institutionCode: AMNH
- **Type status:** Paratype. **Occurrence:** catalogNumber: UCR_ENT 00017039; recordedBy: N. L. H. Krauss; sex: Adult Female; **Taxon:** scientificName: Zelus panamensis; family: Reduviidae; genus: Zelus; scientificNameAuthorship: Zhang and Hart, 2016; **Location:** country: PANAMA; stateProvince: Panama; locality: Summit; decimalLatitude: 9.04697; decimalLongitude: -79.64056; georeferenceSources: Google Earth; **Identification:** identifiedBy: G. Zhang; dateIdentified: 2013; **Event:** eventDate: 1953-12-01; **Record Level:** institutionCode: AMNH
- **Type status:** Paratype. **Occurrence:** catalogNumber: UCR_ENT 00029353; occurrenceRemarks: Drake Collection. Designated as allotype for his new species *Zelus cestartus* [Mansucript name] by E. R. Hart. *Zelus panamensis* and *Zelus cestartus* were considered to be synonymic by G. Zhang. This specimen hence loses its allotype status. The allotype label affixed by Hart, however, remains attached to the pin.; recordedBy: unknown; sex: Adult Female; **Taxon:** scientificName: Zelus panamensis; family: Reduviidae; genus: Zelus; scientificNameAuthorship: Zhang and Hart, 2016; **Location:** country: unknown; stateProvince: unknown; locality: Unknown; **Identification:** identifiedBy: G. Zhang; dateIdentified: 2013; **Event:** eventDate: 1938-04-06; **Record Level:** institutionCode: USNM

#### Description

Figs 150, 151, 152

***Male***: (Fig. 150a, b) Medium-sized, total length 11.25–12.90 mm (mean 11.96 mm, Suppl. material 2); slender. **COLORATION**: Dark brown. Head orangish or reddish; remainder of body surface nearly uniformly dark brown; legs not banded or with inconspicuous bands. **VESTITURE**: Sparsely setose. Entire surface of head with short, recumbent setae; sparse, short, erect, spine-like setae on dorsal surface, denser on anteocular lobe; few long, erect, fine setae on ventral surface. Pronotum with sparse, short, erect, spine-like setae on dorsum, very sparse on anterior lobe, setal tracts indistinct; lateral surface of pronotum and pleura with moderately long, semi-erect or recumbent setae; scutellum with moderately long, erect, spine-like setae. Legs with very sparse setation; sundew setae on profemur very sparse. Corium and clavus with mix of short, recumbent or erect setae and long, erect, fine setae. Abdomen with moderately dense, short recumbent setae, intermixed with sparse, long, erect setae. **STRUCTURE: Head**: Cylindrical, L/W = 2.35. Postocular lobe long; in dorsal view distinctly narrowing through anterior 2/3, posterior 1/3 constant, tube-like. Eye prominent; lateral margin much wider than postocular lobe; dorsal margin attaining postocular transverse groove, ventral margin removed from ventral surface of head in lateral view. *Labium*: I: II: III = 1: 1.9: 0.4. Basiflagellomere diameter larger than that of pedicel. **Thorax**: Anterolateral angle bearing small, somewhat acute projection; medial longitudinal sulcus evident throughout, deepening posteriorly. Posterior pronotal lobe with rugulose surface; disc distinctly elevated above humeral angle; humeral angle armed, with dentate projection. Scutellum moderately long; apex angulate, slightly projected upward. *Legs*: Slender. *Hemelytron*: Slightly surpassing apex of abdomen, not more than length of abdominal segment seven; quadrate cell small; Cu and M of cubital cell subparallel. **GENITALIA**: (Fig. 151) *Pygophore*: Elongate ovoid; lightly sclerotized expansion below paramere; not expanded laterally in dorsal view. Medial process cylindrical; slender; moderately long, about as long as paramere; laterally slightly compressed towards apex; minute spicules on posterior surface; semi-erect; very slightly curved at middle; apex in posterior view acute, with small hooklike projection. *Paramere*: Cylindrical; moderately long, not reaching medial process; directed posteriad; basally slightly narrower; slightly curved ventrad; apical part very slightly enlarged. *Phallus*: Dorsal phallothecal sclerite shield-shaped; sclerotization reduced (yet not absent) on dorsal surface close to posterior margin of foramen; expansion of lateral margin at about mid-portion pronounced, covering ventral surface of endosoma; apical portion of phallothecal sclerite gradually tapering, distinctly keeled medially, laterally indistinctly angulate; apex acute; posterior margin of foramen broadly concave. Struts attached to dorsal phallothecal sclerite; apically separate, connected by bridge; basally separate throughout. Basal plate arm moderately robust; basally fused; in lateral view slightly curved; bridge extremely short; extension of basal plate expanded laterally onto arm, covering more than 1/2 of arm.

***Female***: (Fig. 150c, d) Similar to male, except for the following. Larger than male, total length 13.40–15.28 mm (mean 14.43 mm, Suppl. material 2). Dorsal coloration similar to that in male; lateral surface of pronotum, pleura in some specimens dark brown; abdomen always orangish or reddish. Basiflagellomere subequal in diameter to pedicel. Process on humeral angle spinous, long.

#### Diagnosis

Recognized by the orangish or reddish head and the dark brown remainder of the body. The short, nearly straight medial process and the short paramere separate males of this species from all other species of the same species group. The yellowish or reddish ventral surface and the usually yellowish or reddish (blackish-brown in some specimens) lateral surface distinguishes females of this species from others in the same group.

#### Etymology

Named after the country Panama, where the holotype was collected.

#### Distribution

**Figure 152.**
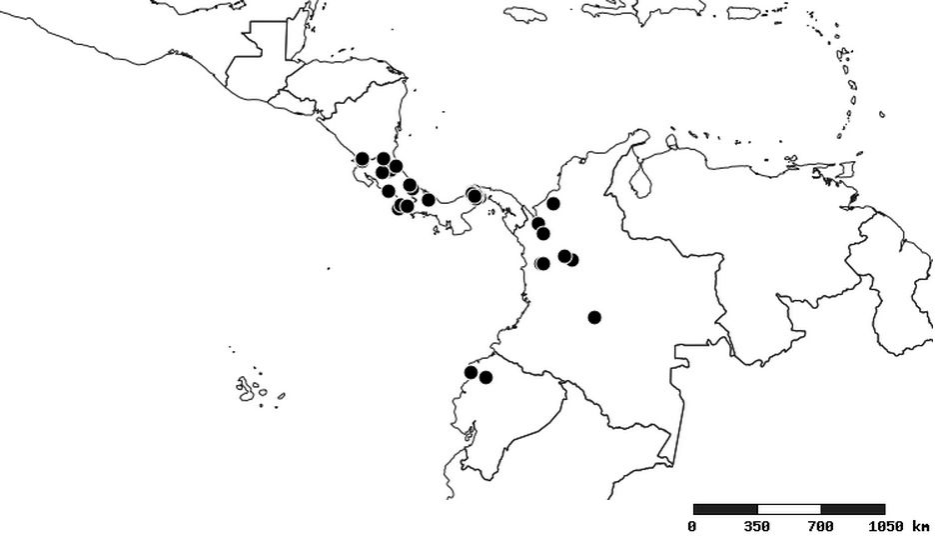
*Zelus panamensis* Zhang & Hart, sp. n., specimen record map

Southern Central America and northern South America (Fig. 152). Countries with records: Colombia, Costa Rica, Ecuador, Nicaragua and Panama,

### Zelus paracephalus

Zhang & Hart sp. n.

urn:lsid:zoobank.org:act:23573F53-C868-47A2-92AE-E98CABDCC654

#### Materials

- **Type status:** Holotype. **Occurrence:** catalogNumber: UCR_ENT 00057803; recordedBy: Richter; sex: Adult Male; **Taxon:** scientificName: Zelus paracephalus; family: Reduviidae; genus: Zelus; scientificNameAuthorship: Zhang and Hart, 2016; **Location:** country: COLOMBIA; stateProvince: Meta; locality: Rio Guamal [Quamal?]; verbatimElevation: 400 m; decimalLatitude: 3.71667; decimalLongitude: -73.1667; georeferenceSources: Gazetteer; **Identification:** identifiedBy: G. Zhang; dateIdentified: 2013; **Event:** eventDate: 1948-01-24; **Record Level:** institutionCode: AMNH
- **Type status:** Paratype. **Occurrence:** catalogNumber: UCR_ENT 00004997; occurrenceRemarks: Primary DNA voucher RCW_2164; recordedBy: G. Zhang & J. Avendaño; sex: Adult Male; **Taxon:** scientificName: Zelus paracephalus; family: Reduviidae; genus: Zelus; scientificNameAuthorship: Zhang and Hart, 2016; **Location:** country: COLOMBIA; stateProvince: Meta; locality: San Martin: Reserva Natural El Caduceo; verbatimElevation: 380 m; decimalLatitude: 3.66593; decimalLongitude: -73.65773; georeferenceSources: GPS; **Identification:** identifiedBy: G. Zhang; dateIdentified: 2012; **Event:** eventDate: 2010-10-21 to 2010-10-23; **Record Level:** institutionCode: UCR
- **Type status:** Paratype. **Occurrence:** catalogNumber: UCR_ENT 00009475; occurrenceRemarks: LOT#992; Drake Collection; recordedBy: T. L. Erwin et al.; sex: Adult Male; **Taxon:** scientificName: Zelus paracephalus; family: Reduviidae; genus: Zelus; scientificNameAuthorship: Zhang and Hart, 2016; **Location:** country: ECUADOR; stateProvince: Orellana; locality: Reserva Etnica Waorani, 1 km S. Onkone Gare Camp, Transect Ent.; verbatimElevation: 216 m; decimalLatitude: -0.65714; decimalLongitude: -76.453; georeferenceSources: Label; **Identification:** identifiedBy: G. Zhang; dateIdentified: 2012; **Event:** samplingProtocol: Fogging; eventDate: 1995-02-10; **Record Level:** institutionCode: USNM
- **Type status:** Paratype. **Occurrence:** catalogNumber: UCR_ENT 00009476; occurrenceRemarks: Drake Collection; recordedBy: T. L. Erwin et al.; sex: Adult Male; **Taxon:** scientificName: Zelus paracephalus; family: Reduviidae; genus: Zelus; scientificNameAuthorship: Zhang and Hart, 2016; **Location:** country: ECUADOR; stateProvince: Orellana; locality: Reserva Etnica Waorani, 1 km S. Onkone Gare Camp, Transect Ent.; verbatimElevation: 216 m; decimalLatitude: -0.65714; decimalLongitude: -76.453; georeferenceSources: Label; **Identification:** identifiedBy: G. Zhang; dateIdentified: 2012; **Event:** samplingProtocol: Fogging; eventDate: 1996-10-02; **Record Level:** institutionCode: USNM
- **Type status:** Paratype. **Occurrence:** catalogNumber: UCR_ENT 00009477; occurrenceRemarks: Drake Collection; recordedBy: T. L. Erwin et al.; sex: Adult Male; **Taxon:** scientificName: Zelus paracephalus; family: Reduviidae; genus: Zelus; scientificNameAuthorship: Zhang and Hart, 2016; **Location:** country: ECUADOR; stateProvince: Orellana; locality: Reserva Etnica Waorani, 1 km S. Onkone Gare Camp, Transect Ent.; verbatimElevation: 216 m; decimalLatitude: -0.65714; decimalLongitude: -76.453; georeferenceSources: Label; **Identification:** identifiedBy: G. Zhang; dateIdentified: 2012; **Event:** samplingProtocol: Fogging; eventDate: 1996-10-02; **Record Level:** institutionCode: USNM
- **Type status:** Paratype. **Occurrence:** catalogNumber: UCR_ENT 00009479; occurrenceRemarks: Lot#1582 - Collection code moved to this field to prevent duplication.; recordedBy: T. L. Erwin et al.; sex: Adult Male; **Taxon:** scientificName: Zelus paracephalus; family: Reduviidae; genus: Zelus; scientificNameAuthorship: Zhang and Hart, 2016; **Location:** country: ECUADOR; stateProvince: Orellana; locality: Reserva Etnica Waorani, 1 km S. Onkone Gare Camp, Transect Ent.; verbatimElevation: 216 m; decimalLatitude: -0.65714; decimalLongitude: -76.453; georeferenceSources: Label; **Identification:** identifiedBy: G. Zhang; dateIdentified: 2012; **Event:** samplingProtocol: Fogging; eventDate: 1996-06-26; **Record Level:** institutionCode: USNM
- **Type status:** Paratype. **Occurrence:** catalogNumber: UCR_ENT 00025329; recordedBy: G. Zambrano; sex: Adult Male; **Taxon:** scientificName: Zelus paracephalus; family: Reduviidae; genus: Zelus; scientificNameAuthorship: Zhang and Hart, 2016; **Location:** country: COLOMBIA; stateProvince: Caqueta; locality: Valparaiso, Vda. Palastina Fca. La Ilusion; verbatimElevation: 225 m; decimalLatitude: 1.18333; decimalLongitude: -75.7; georeferenceSources: Label; **Identification:** identifiedBy: G. Zhang; dateIdentified: 2012; **Event:** eventDate: 1997-01-24; **Record Level:** institutionCode: UNAB
- **Type status:** Paratype. **Occurrence:** catalogNumber: UCR_ENT 00029302; occurrenceRemarks: Drake Collection; recordedBy: J. E. Eger; sex: Adult Male; **Taxon:** scientificName: Zelus paracephalus; family: Reduviidae; genus: Zelus; scientificNameAuthorship: Zhang and Hart, 2016; **Location:** country: BRAZIL; stateProvince: Rondonia; locality: 62 km SW of Ariquemes, near Fzda. Rancho Grande; decimalLatitude: -10.32921; decimalLongitude: -63.46881; **Identification:** identifiedBy: G. Zhang; dateIdentified: 2012; **Event:** eventDate: 1992-03-30 to 1992-04-10; **Record Level:** institutionCode: USNM
- **Type status:** Paratype. **Occurrence:** catalogNumber: UCR_ENT 00029304; occurrenceRemarks: Drake Collection; recordedBy: Unknown; sex: Adult Male; **Taxon:** scientificName: Zelus paracephalus; family: Reduviidae; genus: Zelus; scientificNameAuthorship: Zhang and Hart, 2016; **Location:** country: PERU; stateProvince: Ucayali; locality: Porvenir, Pulcal[l]pa; verbatimElevation: 158 m; decimalLatitude: -8.3825; decimalLongitude: -74.5381; georeferenceSources: Gazetteer; **Identification:** identifiedBy: G. Zhang; dateIdentified: 2012; **Event:** eventDate: 1992-07-01; **Record Level:** institutionCode: USNM
- **Type status:** Paratype. **Occurrence:** catalogNumber: UCR_ENT 00029305; occurrenceRemarks: Drake Collection; recordedBy: L. E. Peña; sex: Adult Male; **Taxon:** scientificName: Zelus paracephalus; family: Reduviidae; genus: Zelus; scientificNameAuthorship: Zhang and Hart, 2016; **Location:** country: COLOMBIA; stateProvince: Vaupes; locality: Mitu; verbatimElevation: 184 m; decimalLatitude: 1.1983; decimalLongitude: -70.1733; georeferenceSources: Gazetteer; **Identification:** identifiedBy: G. Zhang; dateIdentified: 2012; **Event:** eventDate: 1990-07-06 to 1990-07-17; **Record Level:** institutionCode: USNM
- **Type status:** Paratype. **Occurrence:** catalogNumber: UCR_ENT 00057802; occurrenceRemarks: Previously designated as 'allotype', a type status not used in the formal publication of this name (Zhang et al.); recordedBy: H. Sturm; sex: Adult Female; **Taxon:** scientificName: Zelus paracephalus; family: Reduviidae; genus: Zelus; scientificNameAuthorship: Zhang and Hart, 2016; **Location:** country: COLOMBIA; stateProvince: unknown; locality: unknown; **Identification:** identifiedBy: G. Zhang; dateIdentified: 2012; **Event:** eventDate: No date provided; **Record Level:** institutionCode: AMNH

#### Description

Figs 153, 154, 155

***Male***: (Fig. 153a, b) Medium-sized, total length 11.87–13.06 mm (mean 12.51 mm, Suppl. material 2); slender. **COLORATION**: Head reddish-brown, anterior to antennal insertion and posterior third of postocular lobe lighter. Rest of surface of body nearly uniformly blackish-brown, area around humeral angle lighter, somewhat reddish. Membrane with blue, purple iridescence. **VESTITURE**: Sparsely setose. Dark, moderately dense, short, erect, spine-like setae on dorsum of head, more curved on postocular lobe; ventral surface with sparse, short, erect and recumbent setae, few long setae. Pronotal dorsum nearly glabrous, very sparse, short, erect and recumbent spine-like setae; lateral surface with sparse, erect to recumbent setae; setal tracts on anterior lobe very reduced. Pleura with sparse, erect setae and moderately dense, recumbent setae. Abdomen with sparse, short, semi-erect or recumbent setae, intermixed with few longer setae. Pygophore with sparse, short to long, semi-erect or erect setae; Paramere apical half with dense, long setae, more than 1/2 length of medial process. **STRUCTURE: Head**: Cylindrical, L/W = 2.39. Postocular lobe long; in dorsal view distinctly narrowing through anterior 2/3, posterior 1/3 constant, tube-like. Eye prominent; lateral margin much wider than postocular lobe; dorsal margin removed from postocular transverse groove, ventral margin attaining ventral surface of head in lateral view. *Labium*: I: II: III = 1: 1.9: 0.3. Basiflagellomere diameter larger than that of pedicel. **Thorax**: Anterolateral angle bearing small projection; medial longitudinal sulcus shallow near collar, deepening posteriorly. Posterior pronotal lobe with rugulose surface; disc distinctly elevated above humeral angle; humeral angle armed, with dentate or spinous process. Scutellum moderately long; apex angulate, very slightly projected upward. *Legs*: Very slender. *Hemelytron*: Surpassing apex of abdomen by about length of abdominal segment seven; quadrate cell small, relatively broad; Cu and M of cubital cell subparallel. **GENITALIA**: (Fig. 154) *Pygophore*: Ovoid; mid-lateral fold adjacent to paramere insertion inconspicuous; not expanded laterally in dorsal view. Medial process expanded laterally; rather broad; long; anteroposteriorly compressed; erect; straight; apex in posterior view rounded, subapical transverse hooklike bridge. *Paramere*: Cylindrical; long, surpassing medial process; curved ventrad at mid-point, apex recurved. *Phallus*: Dorsal phallothecal sclerite shield-shaped, sclerite absent laterad to basal arms; lateral longitudinal blade-like heavy sclerotization, pressed against phallothecal sclerite, reaching apical margin; area between these raised; apical portion of phallothecal sclerite not distinctly tapered, flat, lateral margin narrowly angulate, angulation ending anteriorly in sharp, dorsad projection; apex with small medial emargination; posterior margin of foramen inversely V-shaped. Struts attached to dorsal phallothecal sclerite; apically separate, connected by bridge; basally separate. Basal plate arm robust; basally fused; in lateral view strongly curved at midpoint; bridge extremely short; extension of basal plate expanded onto arm.

***Female***: (Fig. 153c, d) Similar to male, except for the following. Larger than male, total length 15.02 mm (n=1, Suppl. material 2). Spinous process on humeral angle long.

#### Diagnosis

The dorsal coloration nearly uniformly dark brown, the head reddish-brown, the membrane with indistinct iridescence are characteristic of this species. Most similar to *Z. erythrocephalus* and *Z. russulumus*; males can be distinguished from both by the rather wide medial process and the uniquely shaped paramere (Fig. 14). Females of *Z. erythrocephalus*, *Z. paracephalus* and *Z. russulumus* ​are difficult to separate.

#### Distribution

**Figure 155.**
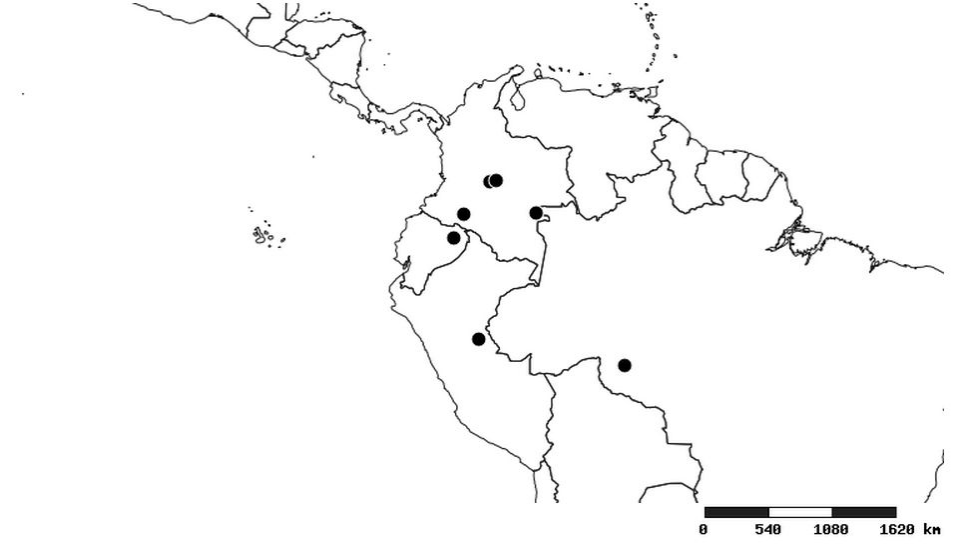
*Zelus paracephalus* Zhang & Hart, sp. n., specimen record map

South America (Fig. 155). Countries with specimen records: Brazil, Colombia, Ecuador and Peru.

### Zelus pedestris

Fabricius, 1803

Zelus pedestris Fabricius, 1803, p. 288, orig. descr.; Stål, 1872, p. 91, cat. (subgenus *Diplodus*); Lethierry and Severin, 1896, p. 151, cat.; Wygodzinsky, 1949a, p. 50, checklist; Maldonado, 1990, p. 330, cat.
Diplodus pedestris : Stål, 1868, p. 109, descr. and note; Walker, 1873, p. 125, cat.
Zelus dispar Fabricius, 1803, p. 291, orig. descr.; Stål, 1872, p. 92, cat. (subgenus Diplodus); Lethierry and Severin, 1896, p. 151, cat.; Wygodzinsky, 1949a, p. 49, checklist; Maldonado, 1990, p. 326, cat. **syn. nov.** (current study).
Diplodus dispar : Stål, 1868, p. 109–110, descr. and note; Walker, 1873, p. 125, cat.
Diplodus obscuridorsis Stål, 1860, p. 75, orig. descr.; Stål, 1868, p. 109, note; Walker, 1873, p. 125, cat.
Zelus obscuridorsis : Stål, 1872, p. 91, cat. and descr. (subgenus *Diplodus*); kthierry and Severin, 1896, p. 152, cat.; Wygodzinsky, 1949a, p. 50, checklist; Wygodzinsky, 1957, p. 26, note and senior syn. of *Z. nugax*, *Z. illotus* and *Z. carvalhoi*; Wygodzinsky, 1960, p. 307–308, list. **syn. nov.** (current study).

#### Materials

- **Type status:** Lectotype. **Occurrence:** occurrenceRemarks: Lectotype of *Zelus pedestris* Fabricius, 1803 (**New Designation** by Zhang, Hart & Weirauch, 2016). Bears the following labels: Type / *Zelus pedestris* in Am. mer. Schmidt.; recordedBy: Schmidt; sex: Adult female; **Taxon:** scientificName: Zelus pedestris; family: Reduviidae; genus: Zelus; scientificNameAuthorship: Fabricius, 1803; **Location:** country: unknown; locality: America meridionali; **Record Level:** institutionCode: ZMUC
- **Type status:** Paralectotype. **Occurrence:** occurrenceRemarks: Paralectotype of *Zelus pedestris* Fabricius, 1803. (**New Designation** by Zhang, Hart & Weirauch, 2016). Bears only the 'Type' label.; sex: Female; **Taxon:** scientificName: Zelus pedestris; family: Reduviidae; genus: Zelus; scientificNameAuthorship: Fabricius, 1803; **Location:** country: unknown; **Record Level:** institutionCode: ZMUC
- **Type status:** Other material. **Occurrence:** occurrenceRemarks: **Lectotype** of *Zelus dispar* Fabricius, 1803 (**New Designation** by Zhang, Hart & Weirauch (2016), junior synonym of *Zelus pedestris* Fabricius, 1803. Bears the following labels: Type / *Zelus pedestris* in Am. mer. Schmidt.; recordedBy: Schmidt; sex: Male; **Taxon:** scientificName: Zelus pedestris; family: Reduviidae; genus: Zelus; scientificNameAuthorship: Fabricius, 1803; **Location:** country: unknown; locality: America meridionali; **Record Level:** institutionCode: ZMUC
- **Type status:** Other material. **Occurrence:** occurrenceRemarks: **Paralectotype** of *Zelus dispar* Fabricius, 1803 (**New Designation** by Zhang, Hart & Weirauch, 2016), junior synonym of *Zelus pedestris* Fabricius, 1803. Bears only a "Type" label; sex: Male; **Taxon:** scientificName: Zelus pedestris; family: Reduviidae; genus: Zelus; scientificNameAuthorship: Fabricius, 1803; **Location:** country: unknown; **Record Level:** institutionCode: ZMUC
- **Type status:** Other material. **Occurrence:** catalogNumber: UCR_ENT 00041009; occurrenceRemarks: **Lectotype** of *Zelus obscuridorsis* Stål, 1860 (**New Designation** by Zhang, Hart & Weirauch, 2016). Verbatim label info: Rio Jan / Stal / *Zelus pedestris* Fabricius det E.R.Hart 1972 / Lectotype *Zelus obscuridorsis* Stal / designated by E.R.Hart / obscuridosis Stal / Typus / NHRS-GULI 000000345.; sex: Adult Female; otherCatalogNumbers: NHRS-GULI 000000345; **Taxon:** scientificName: Zelus pedestris; family: Reduviidae; genus: Zelus; scientificNameAuthorship: Fabricius, 1803; **Location:** country: BRAZIL; stateProvince: Rio de Janeiro; locality: unknown; **Identification:** identifiedBy: G. Zhang; dateIdentified: 2012; **Event:** eventDate: No date provided; **Record Level:** institutionCode: NHRS
- **Type status:** Other material. **Occurrence:** occurrenceRemarks: **Paralectotype** of *Zelus obscuridorsis* Stål, 1860 (**New Designation** by Zhang, Hart & Weirauch, 2016); sex: Adult Female; otherCatalogNumbers: NHRS-GULI 000000345; **Taxon:** scientificName: Zelus pedestris; family: Reduviidae; genus: Zelus; scientificNameAuthorship: Fabricius, 1803; **Location:** country: BRAZIL; locality: unknown; **Identification:** identifiedBy: G. Zhang; dateIdentified: 2012; **Event:** eventDate: No date provided; **Record Level:** institutionCode: NHRS

#### Description

Figs 156, 157, 158

***Male***: (Fig. 156a, b) Small, total length 9.63–11.26 mm (mean 10.60 mm, Suppl. material 2); slender. **COLORATION**: Dorsal surface dark brown; lighter on posterior pronotal lobe; lateral and ventral surfaces yellowish-brown; legs yellowish-brown, femoral apices dark, tibiae with three or four darker bands. **VESTITURE**: Sparsely setose. Entire surface of head with short, recumbent and erect setae, some scattered longer erect setae on postocular lobe, especially ventrally. Anterior pronotal lobe with short, recumbent to short, erect setae, confined to setal tracts dorsally, longer setae laterally; posterior pronotal lobe with short, recumbent and moderate, erect setae. Abdomen with short, recumbent and erect and scattered, longer erect setae. **STRUCTURE: Head**: Cylindrical, L/W = 2.37. Postocular lobe long; in dorsal view anteriorly gradually narrowing, posterior portion constant, slightly narrower. Eye smallish; lateral margin only slightly wider than postocular lobe; dorsal and ventral margins removed from surfaces of head. *Labium*: I: II: III = 1: 2.1: 0.4. Basiflagellomere diameter larger than that of pedicel. **Thorax**: Anterolateral angle subtuberculate; medial longitudinal sulcus shallow near collar, deepening posteriorly. Posterior pronotal lobe with rugulose surface; disc distinctly elevated above humeral angle; humeral angle armed, with dentate projection. Scutellum long; apex slightly pointed. *Legs*: Very slender, femoral diameters subequal. *Hemelytron*: Slightly surpassing apex of abdomen, not more than length of abdominal segment seven; quadrate cell small; Cu and M of cubital cell subparallel. **GENITALIA**: (Fig. 157) *Pygophore*: Ovoid; not expanded laterally in dorsal view. Medial process blade-like; very slender; long, only slightly shorter than paramere; laterally compressed; semi-erect; nearly straight; apex slightly curved, acute, without modification. *Paramere*: Cylindrical; moderately long, achieving apex of medial process; directed posteriad; slightly curved dorsad; apical portion not enlarged, apex acute or rounded. *Phallus*: Dorsal phallothecal sclerite shield-shaped; small indentation of lateral margin at about mid-point; apical portion of phallothecal sclerite distinctly tapered, slightly convex, laterally rounded, not forming angle; posterior margin of foramen deeply concave. Struts attached to dorsal phallothecal sclerite; apically fused; basally fused. Basal plate arm slender; basally fused; in lateral view very slightly curved; bridge extremely short; extension of basal plate small, laterally expanded onto arm.

***Female***: (Fig. 156c, d) Similar to male, except for the following. Larger than male, total length 12.15–13.36mm (mean 13.09 mm, Suppl. material 2). Coloration lighter than male.

#### Diagnosis

The slender, cylindrical paramere and the laterally compressed, blade-like medial process can separate this species from most other species of the genus. Different from *Z. illotus* and *Z. impar* by the straight medial process, contrasting with the recurved medial processes of the other two species. Difficult to distinguish from *Z. nugax*, but the paramere apex is generally rounded, the dorsal phallothecal sclerite usually with lateral indentation, the basal plate arms are fused and the basal plate arm extension is present and laterally expanded.

#### Distribution

**Figure 158.**
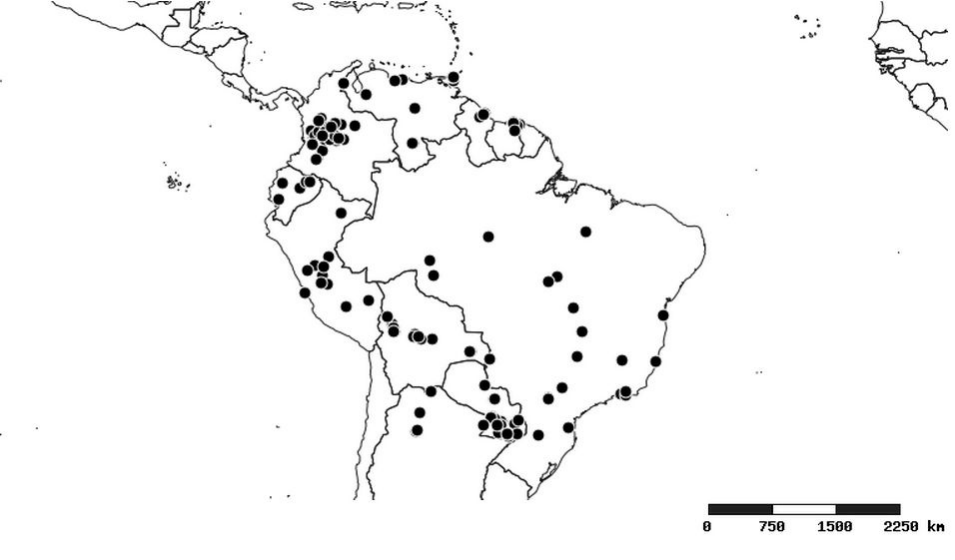
Zelus pedestris Fabricius, 1803, specimen record map

South America and adjacent islands of the Caribbean (Fig. 158). Countries with records: Argentina, Bolivia, Brazil, Colombia, Ecuador, Guyana, Paraguay, Peru, Suriname and Trinidad and Tobago.

#### Taxon discussion

Some helpful, although not consistently reliable, characters used to distinguish females of *Z. pedestris* from those of *Z. nugax* and *Z. illotus* are given in the taxon discussion section of *Z. nugax*. Uncertainties in species limits between *Z. pedestris* and *Z. nugax* are also discussed in that species. The application of the name *Z. pedestris* is restricted to specimens from South America.

The coloration of the posterior pronotal lobe is the most obvious variable character in the males. This varies from a very light to a medium reddish-brown in any given geographic area. This pronotal variation is absent in the females, the dorsal surface varying from a relatively uniform light to medium brown in any geographic area.

### Zelus plagiatus

(Signoret, 1852)

Diplodus plagiatus Signoret, 1862, p. 584–585, orig. descr.; Walker, 1873, p. 126, cat.
Zelus plagiatus : Stål, 1872, p. 92, cat.; Lethierry and Severin, 1896, p. 153, cat.; Wygodzinsky, 1949a, p. 50, checklist; Maldonado, 1990, p. 330, cat.

#### Materials

- **Type status:** Holotype. **Occurrence:** catalogNumber: UCR_ENT 00075076; occurrenceRemarks: Verbatim label info: Peru coll. Signoret / Plagiat. det. Signoret. / Holotype / Zeuls plagiatus (Signoret) / Typus Diplodus plagiatus SIGNORET, 1862 etik. Hecher 1996 REDV. 484/1; recordedBy: Signoret; sex: Adult Female; **Taxon:** scientificName: Zelus plagiatus; family: Reduviidae; genus: Zelus; scientificNameAuthorship: (Signoret, 1852); **Location:** country: PERU; stateProvince: unknown; locality: Unknown; **Identification:** identifiedBy: G. Zhang; dateIdentified: 2012; **Event:** eventDate: No date provided; **Record Level:** institutionCode: NHMW

#### Description

Figs 159, 160

***Male***: unknown.

***Female***: (Fig. 159) Medium-sized, total length 13.20–15.43 mm (mean 14.32 mm, Suppl. material 2); slender. **COLORATION**: Brightly colored. Yellow and black. Dorsal surface mostly yellow, black patch on distal area of corium, posterior part of posterior pronotal lobe black; legs mostly black, femoral base yellowish, single yellowish subapical band on metafemur. **VESTITURE**: Sparsely setose. Head with short, to moderate length, erect and recumbent setae, scattered. Anterior pronotal lobe with short, erect and recumbent setae; posterior pronotal lobe with short, erect setae. Abdomen with short to moderate length erect setae. **STRUCTURE: Head**: Elongated, L/W = 2.69. Postocular lobe very long; in dorsal view distinctly narrowing through anterior 1/2, posterior 1/2 constant, tube-like. Eye moderately sized; lateral margin much wider than postocular lobe; dorsal and ventral margins removed from surfaces of head. *Labium*: I: II: III = 1: 2.1: 0.5. **Thorax**: Anterolateral angle rounded, without projection; medial longitudinal sulcus shallow near collar, deepening posteriorly. Posterior pronotal lobe with finely rugulose surface; disc distinctly elevated above humeral angle; humeral angle armed, with spinous process. Scutellum long; apex angulate. *Legs*: Slender. *Hemelytron*: Slightly surpassing apex of abdomen, not more than length of abdominal segment seven; quadrate cell moderately sized; Cu and M of cubital cell subparallel.

#### Diagnosis

The dorsum predominantly yellow with the posterior pronotal lobe partly black and two black spots on hemelytra is distinctive of this species.

#### Distribution

**Figure 160.**
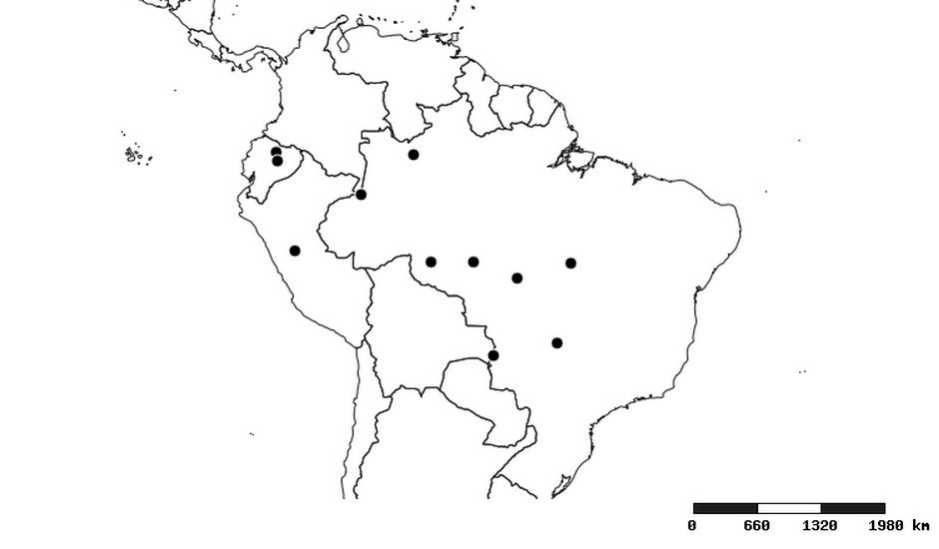
*Zelus plagiatus* (Signoret, 1852), specimen record map

South America (Fig. 160). Countries with records: Brazil, Ecuador, Colombia and Peru.

### Zelus prolixus

(Stål, 1860)

Euagoras prolixus Stål, 1860, p. 74, orig. descr.; Walker, 1873, p. 118, cat.
Zelus prolixus : Stål, 1872, p. 89, cat. (subgenus *Zelus*); Lethierry and Severin, 1896, p. 153, cat.; Champion, 1898, p. 255, note; Wygodzinsky, 1949a; p. 50, checklist; Elkins, 1954, p. 39, 40, note; Hart, 1987, p. 297-299, redescription, note, fig. and key; Maldonado, 1990, p. 330, cat.

#### Materials

- **Type status:** Holotype. **Occurrence:** occurrenceRemarks: The specimen bears the following labels: Brasil / F. Sahib. / Typus / prolixus Stal / 167-53; recordedBy: F. Sahib.; sex: Adult Female; **Taxon:** scientificName: Zelus prolixus; family: Reduviidae; genus: Zelus; scientificNameAuthorship: (Stål, 1860); **Location:** country: BRAZIL; locality: unknown; **Identification:** identifiedBy: E.R. Hart; dateIdentified: 1972; **Record Level:** institutionCode: NHRS

#### Description

Figs 161, 162, 163

***Male***: (Fig. 161a, b) Small, total length 8.64–10.10 mm (mean 9.35 mm, Suppl. material 2); very slender. **COLORATION**: Pale or greenish-brown. Single ring near femoral apices; tibiae banded. **VESTITURE**: Sparsely setose. Dorsum of head and anterior pronotal lobe with very sparse short, erect and recumbent setae. Head ventrally with long, erect setae. Posterior pronotal lobe and hemelytron with dense, short, recument setae. Abdominal venter with moderately dense, short, recument and short to moderately long, erect setae. Paramere apex with sparse, short setae. Anteocular lobe nearly glabrous except for some sparse, erect setae; recumbent and erect setae on remainder of surface of head. Dorsal surface of anterior pronotal lobe nearly glabrous, some recumbent setae on lateral surface; recumbent setae on entire surface of posterior pronotal lobe. Abdomen with scattered, short, recumbent setae, sparse erect setae. **STRUCTURE: Head**: Dorsoventrally flattened, L/W = 2.25. Postocular lobe short; in dorsal view anteriorly gradually narrowing, posterior portion constant, slightly narrower. Eye moderately sized; lateral margin only slightly wider than postocular lobe; dorsal margin removed from postocular transverse groove, ventral margin attaining ventral surface of head in lateral view. *Labium*: I: II: III = 1: 2.3: 0.5. Basiflagellomere diameter larger than that of pedicel. **Thorax**: Anterolateral angle rounded, without projection; medial longitudinal sulcus evident only on posterior 1/2, deepening anterior to transverse sulcus of pronotum. Posterior pronotal lobe with rugulose surface; disc distinctly elevated above humeral angle; humeral angle rounded, without projection. Scutellum long; apex angulate, not projected. *Legs*: Very slender. *Hemelytron*: Surpassing apex of abdomen by about length of abdominal segment seven; quadrate cell small and slender; Cu and M of cubital cell subparallel. **GENITALIA**: (Fig. 162) *Pygophore*: Ovoid; not expanded laterally in dorsal view. Medial process broadly triangular; very short; semi-erect; nearly straight; apex in posterior view blunt, without modification. *Paramere*: Cylindrical; short, not reaching apex of medial process; directed posteriad; nearly straight; apical part not enlarged. *Phallus*: Dorsal phallothecal sclerite somewhat squarish; small longitudinal dorsolateral ridge; apical portion of phallothecal sclerite not distinctly tapered, flat, laterally rounded, not forming angle; apex truncate; posterior margin of foramen broadly concave. Struts attached to dorsal phallothecal sclerite; apically fused; basally almost completely fused. Basal plate arm slender; separate; subparallel; in lateral view nearly straight, very slightly curved; bridge moderately long; extension of basal plate expanded onto arm.

***Female***: (Fig. 161c, d) Similar to male, except for the following. Larger than male, total length 10.29–11.79 mm (mean 11.10 mm, Suppl. material 2). Hemelytron attaining apex of abdomen.

#### Diagnosis

Distinguished by the greenish coloration; the veins of membrane darker than the cells; the smallish size; the rather slender body and very delicate legs; the head somewhat dorsoventrally flattened; and the eye somewhat elongated. Males can be separated from most species of *Zelus* by the broadly triangular medial process. The short paramere not exceeding the medial process separates *Z. prolixus* from *Z. minutus*.

#### Distribution

**Figure 163.**
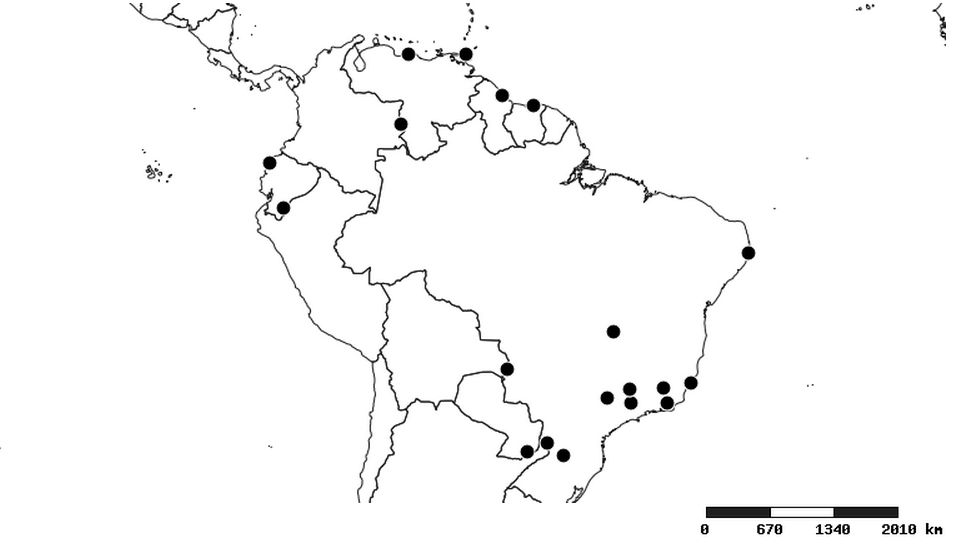
*Zelus prolixus* (Stål, 1860), specimen record map

South America and adjacent islands of the Caribbean (Fig. 163). Argentina, Brazil, Ecuador, Guyana, Paraguay, Suriname, Trinidad and Tobago and Venezuela.

### Zelus puertoricensis

Hart, 1987

Zelus puertoricensis Hart, 1987, p. 294, orig. descr., key and fig.; Maldonado, 1990, p. 330, cat.

#### Materials

- **Type status:** Holotype. **Occurrence:** catalogNumber: UCR_ENT 00057799; recordedBy: Unknown; sex: Adult Male; **Taxon:** scientificName: Zelus puertoricensis; family: Reduviidae; genus: Zelus; scientificNameAuthorship: Hart, 1987; **Location:** country: PUERTO RICO; locality: Coamo Springs; decimalLatitude: 18.07996; decimalLongitude: -66.35794; georeferenceSources: Google Earth; **Identification:** identifiedBy: G. Zhang; dateIdentified: 2012; **Event:** eventDate: 1914-07-17 to 1914-07-19; **Record Level:** institutionCode: AMNH
- **Type status:** Allotype. **Occurrence:** catalogNumber: UCR_ENT 00057798; recordedBy: Unknown; sex: Adult Female; **Taxon:** scientificName: Zelus puertoricensis; family: Reduviidae; genus: Zelus; scientificNameAuthorship: Hart, 1987; **Location:** country: PUERTO RICO; locality: Coamo Springs; decimalLatitude: 18.07996; decimalLongitude: -66.35794; georeferenceSources: Google Earth; **Identification:** identifiedBy: G. Zhang; dateIdentified: 2012; **Event:** eventDate: 1914-07-17 to 1914-07-19; **Record Level:** institutionCode: AMNH
- **Type status:** Paratype. **Occurrence:** catalogNumber: UCR_ENT 00016144; recordedBy: V. Biaggi; sex: Adult Male; **Taxon:** scientificName: Zelus puertoricensis; family: Reduviidae; genus: Zelus; scientificNameAuthorship: Hart, 1987; **Location:** country: PUERTO RICO; stateProvince: Jayuya; locality: Jayuya; decimalLatitude: 18.2186; decimalLongitude: -66.5916; georeferenceSources: Gazetteer; **Identification:** identifiedBy: G. Zhang; dateIdentified: 2012; **Event:** eventDate: 1934-12-01; **Record Level:** institutionCode: AMNH
- **Type status:** Paratype. **Occurrence:** catalogNumber: UCR_ENT 00009438; recordedBy: Unknown; sex: Adult Male; **Taxon:** scientificName: Zelus puertoricensis; family: Reduviidae; genus: Zelus; scientificNameAuthorship: Hart, 1987; **Location:** country: PUERTO RICO; locality: Coamo Springs; verbatimElevation: 126 m; decimalLatitude: 18.07996; decimalLongitude: -66.35794; georeferenceSources: Google Earth; **Identification:** identifiedBy: G. Zhang; dateIdentified: 2012; **Event:** eventDate: 1914-07-17 to 1914-07-19; **Record Level:** institutionCode: USNM
- **Type status:** Paratype. **Occurrence:** catalogNumber: UCR_ENT 00009439; recordedBy: Unknown; sex: Adult Male; **Taxon:** scientificName: Zelus puertoricensis; family: Reduviidae; genus: Zelus; scientificNameAuthorship: Hart, 1987; **Location:** country: PUERTO RICO; locality: Cidra; decimalLatitude: 18.17778; decimalLongitude: -66.16167; georeferenceSources: GeoLocate Software; **Identification:** identifiedBy: G. Zhang; dateIdentified: 2012; **Event:** eventDate: 1948-06-04; **Record Level:** institutionCode: USNM
- **Type status:** Paratype. **Occurrence:** catalogNumber: UCR_ENT 00009440; recordedBy: Unknown; sex: Adult Male; **Taxon:** scientificName: Zelus puertoricensis; family: Reduviidae; genus: Zelus; scientificNameAuthorship: Hart, 1987; **Location:** country: PUERTO RICO; locality: Cidra; decimalLatitude: 18.17778; decimalLongitude: -66.16167; georeferenceSources: GeoLocate Software; **Identification:** identifiedBy: G. Zhang; dateIdentified: 2012; **Event:** eventDate: 1948-06-04; **Record Level:** institutionCode: USNM
- **Type status:** Paratype. **Occurrence:** catalogNumber: UCR_ENT 00009441; recordedBy: Unknown; sex: Adult Male; **Taxon:** scientificName: Zelus puertoricensis; family: Reduviidae; genus: Zelus; scientificNameAuthorship: Hart, 1987; **Location:** country: PUERTO RICO; locality: Cidra; decimalLatitude: 18.17778; decimalLongitude: -66.16167; georeferenceSources: GeoLocate Software; **Identification:** identifiedBy: G. Zhang; dateIdentified: 2012; **Event:** eventDate: 1948-06-04; **Record Level:** institutionCode: USNM
- **Type status:** Paratype. **Occurrence:** catalogNumber: UCR_ENT 00015108; recordedBy: A. H. Manee; sex: Adult Female; **Taxon:** scientificName: Zelus puertoricensis; family: Reduviidae; genus: Zelus; scientificNameAuthorship: Hart, 1987; **Location:** country: PUERTO RICO; locality: Arecibo; decimalLatitude: 18.4744; decimalLongitude: -66.7161; **Identification:** identifiedBy: G. Zhang; dateIdentified: 2012; **Event:** eventDate: 1915-05-10; **Record Level:** institutionCode: AMNH
- **Type status:** Paratype. **Occurrence:** catalogNumber: UCR_ENT 00016145; recordedBy: Unknown; sex: Adult Male; **Taxon:** scientificName: Zelus puertoricensis; family: Reduviidae; genus: Zelus; scientificNameAuthorship: Hart, 1987; **Location:** country: PUERTO RICO; locality: Coamo Springs; verbatimElevation: 126 m; decimalLatitude: 18.07996; decimalLongitude: -66.35794; georeferenceSources: Google Earth; **Identification:** identifiedBy: G. Zhang; dateIdentified: 2012; **Event:** eventDate: 1914-07-17 to 1914-07-19; **Record Level:** institutionCode: AMNH
- **Type status:** Paratype. **Occurrence:** catalogNumber: UCR_ENT 00016146; recordedBy: Unknown; sex: Adult Female; **Taxon:** scientificName: Zelus puertoricensis; family: Reduviidae; genus: Zelus; scientificNameAuthorship: Hart, 1987; **Location:** country: PUERTO RICO; locality: Coamo Springs; verbatimElevation: 126 m; decimalLatitude: 18.07996; decimalLongitude: -66.35794; georeferenceSources: Google Earth; **Identification:** identifiedBy: G. Zhang; dateIdentified: 2012; **Event:** eventDate: 1914-07-17 to 1914-07-19; **Record Level:** institutionCode: AMNH
- **Type status:** Paratype. **Occurrence:** catalogNumber: UCR_ENT 00016992; recordedBy: Unknown; sex: Adult Female; **Taxon:** scientificName: Zelus puertoricensis; family: Reduviidae; genus: Zelus; scientificNameAuthorship: Hart, 1987; **Location:** country: PUERTO RICO; locality: Coamo Springs; verbatimElevation: 126 m; decimalLatitude: 18.07996; decimalLongitude: -66.35794; georeferenceSources: Google Earth; **Identification:** identifiedBy: G. Zhang; dateIdentified: 2012; **Event:** eventDate: 1914-07-17 to 1914-07-19; **Record Level:** institutionCode: AMNH
- **Type status:** Paratype. **Occurrence:** catalogNumber: UCR_ENT 00016993; recordedBy: Unknown; sex: Adult Female; **Taxon:** scientificName: Zelus puertoricensis; family: Reduviidae; genus: Zelus; scientificNameAuthorship: Hart, 1987; **Location:** country: PUERTO RICO; locality: Coamo Springs; verbatimElevation: 126 m; decimalLatitude: 18.07996; decimalLongitude: -66.35794; georeferenceSources: Google Earth; **Identification:** identifiedBy: G. Zhang; dateIdentified: 2012; **Event:** eventDate: 1914-07-17 to 1914-07-19; **Record Level:** institutionCode: AMNH
- **Type status:** Paratype. **Occurrence:** catalogNumber: UCR_ENT 00016994; recordedBy: Unknown; sex: Adult Female; **Taxon:** scientificName: Zelus puertoricensis; family: Reduviidae; genus: Zelus; scientificNameAuthorship: Hart, 1987; **Location:** country: PUERTO RICO; locality: Coamo Springs; verbatimElevation: 126 m; decimalLatitude: 18.07996; decimalLongitude: -66.35794; georeferenceSources: Google Earth; **Identification:** identifiedBy: G. Zhang; dateIdentified: 2012; **Event:** eventDate: 1914-07-17 to 1914-07-19; **Record Level:** institutionCode: AMNH
- **Type status:** Paratype. **Occurrence:** catalogNumber: UCR_ENT 00016995; recordedBy: Unknown; sex: Adult Female; **Taxon:** scientificName: Zelus puertoricensis; family: Reduviidae; genus: Zelus; scientificNameAuthorship: Hart, 1987; **Location:** country: PUERTO RICO; locality: Coamo Springs; verbatimElevation: 126 m; decimalLatitude: 18.07996; decimalLongitude: -66.35794; georeferenceSources: Google Earth; **Identification:** identifiedBy: G. Zhang; dateIdentified: 2012; **Event:** eventDate: 1914-07-17 to 1914-07-19; **Record Level:** institutionCode: AMNH
- **Type status:** Paratype. **Occurrence:** catalogNumber: UCR_ENT 00016996; recordedBy: Unknown; sex: Adult Female; **Taxon:** scientificName: Zelus puertoricensis; family: Reduviidae; genus: Zelus; scientificNameAuthorship: Hart, 1987; **Location:** country: PUERTO RICO; locality: Coamo Springs; verbatimElevation: 126 m; decimalLatitude: 18.07996; decimalLongitude: -66.35794; georeferenceSources: Google Earth; **Identification:** identifiedBy: G. Zhang; dateIdentified: 2012; **Event:** eventDate: 1914-07-17 to 1914-07-19; **Record Level:** institutionCode: AMNH
- **Type status:** Paratype. **Occurrence:** catalogNumber: UCR_ENT 00017655; recordedBy: Unknown; sex: Adult Male; **Taxon:** scientificName: Zelus puertoricensis; family: Reduviidae; genus: Zelus; scientificNameAuthorship: Hart, 1987; **Location:** country: PUERTO RICO; locality: Coamo Springs; verbatimElevation: 126 m; decimalLatitude: 18.07996; decimalLongitude: -66.35794; georeferenceSources: Google Earth; **Identification:** identifiedBy: G. Zhang; dateIdentified: 2012; **Event:** eventDate: 1914-07-17 to 1914-07-19; **Record Level:** institutionCode: AMNH
- **Type status:** Paratype. **Occurrence:** catalogNumber: UCR_ENT 00009447; recordedBy: R. G. Oakley; sex: Adult Female; **Taxon:** scientificName: Zelus puertoricensis; family: Reduviidae; genus: Zelus; scientificNameAuthorship: Hart, 1987; **Location:** country: PUERTO RICO; stateProvince: Penuelas; locality: Penuelas; decimalLatitude: 18.05833; decimalLongitude: -66.72194; georeferenceSources: GeoLocate Software; **Identification:** identifiedBy: G. Zhang; dateIdentified: 2012; **Event:** eventDate: 1932-09-08; **Record Level:** institutionCode: USNM
- **Type status:** Paratype. **Occurrence:** catalogNumber: UCR_ENT 00009437; recordedBy: M. D. Leonard; sex: Adult Male; **Taxon:** scientificName: Zelus puertoricensis; family: Reduviidae; genus: Zelus; scientificNameAuthorship: Hart, 1987; **Location:** country: PUERTO RICO; stateProvince: San Juan; locality: Rio Piedras; verbatimElevation: 29 m; decimalLatitude: 18.3994; decimalLongitude: -66.0503; georeferenceSources: Gazetteer; **Identification:** identifiedBy: G. Zhang; dateIdentified: 2012; **Event:** eventDate: 1931-06-01; **Record Level:** institutionCode: USNM
- **Type status:** Paratype. **Occurrence:** catalogNumber: UCR_ENT 00009443; recordedBy: R.T. Cotton; sex: Adult Female; **Taxon:** scientificName: Zelus puertoricensis; family: Reduviidae; genus: Zelus; scientificNameAuthorship: Hart, 1987; **Location:** country: PUERTO RICO; stateProvince: San Juan; locality: Rio Piedras; verbatimElevation: 29 m; decimalLatitude: 18.3994; decimalLongitude: -66.0503; georeferenceSources: Gazetteer; **Identification:** identifiedBy: G. Zhang; dateIdentified: 2012; **Event:** eventDate: 1916-07-29; **Record Level:** institutionCode: USNM
- **Type status:** Paratype. **Occurrence:** catalogNumber: UCR_ENT 00009444; recordedBy: R.T. Cotton; sex: Adult Female; **Taxon:** scientificName: Zelus puertoricensis; family: Reduviidae; genus: Zelus; scientificNameAuthorship: Hart, 1987; **Location:** country: PUERTO RICO; stateProvince: San Juan; locality: Rio Piedras; verbatimElevation: 29 m; decimalLatitude: 18.3994; decimalLongitude: -66.0503; georeferenceSources: Gazetteer; **Identification:** identifiedBy: G. Zhang; dateIdentified: 2012; **Event:** eventDate: 1916-07-29; **Record Level:** institutionCode: USNM
- **Type status:** Paratype. **Occurrence:** catalogNumber: UCR_ENT 00009445; recordedBy: G. Bay; sex: Adult Female; **Taxon:** scientificName: Zelus puertoricensis; family: Reduviidae; genus: Zelus; scientificNameAuthorship: Hart, 1987; **Location:** country: PUERTO RICO; stateProvince: San Juan; locality: San Juan; decimalLatitude: 18.46633; decimalLongitude: -66.10573; georeferenceSources: Google Earth; **Identification:** identifiedBy: G. Zhang; dateIdentified: 2012; **Event:** eventDate: 1926-03-29; **Record Level:** institutionCode: USNM
- **Type status:** Paratype. **Occurrence:** catalogNumber: UCR_ENT 00030178; occurrenceRemarks: Bears labels 'On Zea mayais', 'San Juan No 8357', and 'Lot No 42-11687'; recordedBy: Unknown; sex: Adult Male; **Taxon:** scientificName: Zelus puertoricensis; family: Reduviidae; genus: Zelus; scientificNameAuthorship: Hart, 1987; **Location:** country: PUERTO RICO; stateProvince: San Juan; locality: San Juan; decimalLatitude: 18.46633; decimalLongitude: -66.10573; georeferenceSources: Google Earth; **Identification:** identifiedBy: G. Zhang; dateIdentified: 2012; **Event:** eventDate: 1942-09-16; **Record Level:** institutionCode: USNM
- **Type status:** Paratype. **Occurrence:** catalogNumber: UCR_ENT 00030179; occurrenceRemarks: Bears labels 'On Zea mayais', 'San Juan No 8357', and 'Lot No 42-11687'; recordedBy: Unknown; sex: Adult Female; **Taxon:** scientificName: Zelus puertoricensis; family: Reduviidae; genus: Zelus; scientificNameAuthorship: Hart, 1987; **Location:** country: PUERTO RICO; stateProvince: San Juan; locality: San Juan; decimalLatitude: 18.46633; decimalLongitude: -66.10573; georeferenceSources: Google Earth; **Identification:** identifiedBy: G. Zhang; dateIdentified: 2012; **Event:** eventDate: 1942-09-16; **Record Level:** institutionCode: USNM
- **Type status:** Paratype. **Occurrence:** catalogNumber: UCR_ENT 00009442; recordedBy: R.T. Cotton; sex: Adult Female; **Taxon:** scientificName: Zelus puertoricensis; family: Reduviidae; genus: Zelus; scientificNameAuthorship: Hart, 1987; **Location:** country: PUERTO RICO; stateProvince: Vega Alta; locality: Vega Alta; verbatimElevation: 100 m; decimalLatitude: 18.4122; decimalLongitude: -66.3313; georeferenceSources: Gazetteer; **Identification:** identifiedBy: G. Zhang; dateIdentified: 2012; **Event:** eventDate: 1917-01-26; **Record Level:** institutionCode: USNM

#### Description

Figs 164, 165, 166

***Male***: (Fig. 164a, b) Small, total length 10.22–11.74 mm (mean 11.20 mm, Suppl. material 2); very slender. **COLORATION**: Dorsal surfaces brown; corium reddish. Lateral surfaces yellowish-brown. Legs generally unicolorous, tibial apical portions reddish. **VESTITURE**: Anteocular lobe with recumbent and sparse, erect setae on entire surface; postocular lobe with recumbent setae, more dense dorsally, erect setae ventrally. Anterior pronotal lobe with recumbent setae, confined setal tracts dorsally, mixed with erect setae laterally; posterior pronotal lobe with short, inconspicuous, erect setae and recumbent setae. Abdomen with short to long, erect setae. **STRUCTURE: Head**: Cylindrical, L/W = 2.27. Postocular lobe moderately long; in dorsal view anteriorly gradually narrowing, posterior portion constant, slightly narrower. Eye smallish; lateral margin only slightly wider than postocular lobe; dorsal and ventral margins removed from surfaces of head. *Labium*: I: II: III = 1: 1.9: 0.5. Basiflagellomere diameter larger than that of pedicel. **Thorax**: Anterolateral angle with inconspicuous subtuberculate projection; medial longitudinal sulcus shallow near collar, deepening posteriorly. Posterior pronotal lobe with rugulose surface; disc distinctly elevated above humeral angle; humeral angle armed, with minute dentate projection. Scutellum long; apex angulate, not projected. *Legs*: Slender, femoral diameters subequal. *Hemelytron*: Slightly surpassing apex of abdomen, not more than length of abdominal segment seven; quadrate cell small, elongate; Cu and M of cubital cell subparallel. **GENITALIA**: (Fig. 165) *Pygophore*: Elongate; mid-lateral fold adjacent to paramere insertion; not expanded laterally in dorsal view. Medial process robust; short; posteriorly directed; nearly straight; apex in posterior view blunt, slightly folded posteriad. *Paramere*: Cylindrical; short, not reaching apex of medial process; directed posteriad; basally slightly constricted; not distinctly curved; apical part slightly enlarged. *Phallus*: Dorsal phallothecal sclerite elongated; apical portion of phallothecal sclerite gradually tapering, flat; apex rounded; posterior margin of foramen strongly inversely V-shaped. Struts attached to dorsal phallothecal sclerite; apically separate, connected by bridge; basally mostly separate, moderately fused. Basal plate arm robust; separate; diverging; in lateral view nearly straight, very slightly curved; bridge long; extension of basal plate small and confined to apex of basal plate arm.

***Female***: (Fig. 164c, d​) Similar to male, except for the following. Larger than male, total length 12.03–14.33 mm (mean 13.20 mm, Suppl. material 2). Hemelytron very slightly surpassing apex of abdomen.

#### Diagnosis

The rather slender body form of *Z. puertoricensis* is characteristic of the *Zelus puertoricensis* species group (total length/width more than 8x). Both sexes of *Z. puertoricensis* have the dorsal and ventral surfaces of the postocular lobe nearly parallel through the anterior 2/3 of the lobe. This contrasts with the sloping configuration of the dorsal surface in *Z. subimpressus*.

Males can be recognized by the robust, posteriorly directed medial process, apex bent and the short, cylindrical paramere. This is smaller in *Z. puertoricensis* than in *Z. subimpressus* (Fig. 6). Additionally, the medial process of *Z. puertoricensis* appears to be longer and more delicate than that of *Z. subimpressus*. The dorsal phallothecal sclerite is much narrower than in *Z. subimpressus*.

#### Distribution

**Figure 166.**
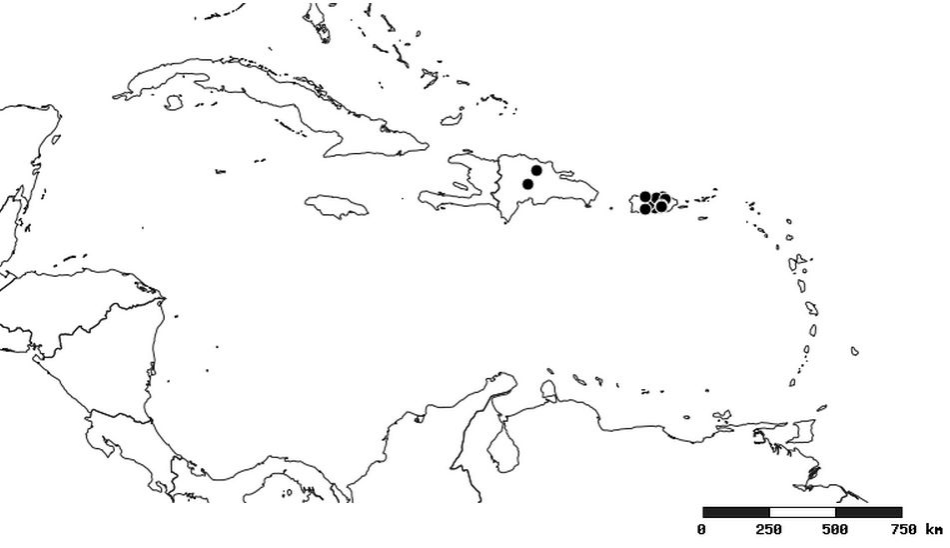
*Zelus puertoricensis* Hart, 1987, specimen record map

The Caribbean, islands of Puerto Rico and Hispaniola (Fig. 166). Countries with records: USA (Puerto Rico only) and Dominican Republic.

### Zelus renardii

Kolenati, 1857

Zelus renardii Kolenati, 1857, p. 460, Tab. III. fig. 2, orig. descr. and fig.; Stål, 1872, p. 91, cat.; Kirkaldy, 1908, p. 195, list and senior syn. of *Z. laevicollis* Champion and *Z. perigrinus* Kirkaldy; Banks, 1910b, p. 16, cat.; Fracker, 1913, p. 239, 240, key and checklist; Van Duzee, 1914, p. 13, list; Van Duzee, 1916, p. 30, checklist (subgenus *Diplocodus*); Van Duzee, 1917, p. 261, cat. (subgenus *Diplocodus*); Muir, 1920, p. 285–286, note; Muir, 1921, p. 119, note; Horton, 1922, p. 385, note; Hawaiian Sugar Planter's Association, 1924, p, 29, note; Readio, 1927, p, 169, 178–179, key, descr. and note; Williams, 1931, p. 101, note; Haldaway and Look, 1942, p. 257–258, note; Ewing and Ivy, 1943, p, 604–606, note; Clancy, 1946, p. 326, note; Van Zwaluwenburg, 1946, p. 15, note; Zimmerman, 1948, p. 137–138 , note and fig.; Wygodzinsky, 1949a, p. 50, checklist; Elkins, 1951, p. 410, list; Sibley, 1951, p. 92, list; Nishida, 1955, p. 172, note; Atkins, et. al., 1957, p. 258, note; Nielsen and Henderson, 1959, p. 159, note; Wene and Sheets, 1962, p. 397, note; Butler, 1966, p. 1306–1307, note; Wygodzinsky, 1966, p. 66, note; Lingren, Ridgway and Jones, 1968, p. 615, note; Parencia, 1968, p. 276, note; Nutting and Spangler, 1969, p. 765, note; Hart, 1986, p. 540-542, redescription, note, fig. and key; Maldonado, 1990, p. 331, cat.
Diplodus renardii : Uhler, 1894, list.
Zelus laevicollis Champion, 1898, p. 260–261, Tab. XV. fig. 24, orig. descr. and fig.; Banks, 1910, p. 16, cat.; Fracker, 1913, p. 239, 240, key and list; Osborne and Drake, 1915, p. 531, note; Van Duzee, 1916, p. 30, checklist (subgenus *Diplocodus*); Van Duzee, 1917, p. 261, cat. (subgenus *Diplocodus*); Readio, 1927, p. 177–178, descr.; Wygodzinsky, 1949a, p. 49, checklist; Elkins, 1951, p. 410, list; Sibley, 1951, p. 92, list; Young and Sifuentes, 1960, p. 1109–1111, biology; Hart, 1986, p. 540, junior syn. of *Z. renardii*.
Zelus peregrinus Kirkaldy, 1902, p. 149–150, orig. descr.; Swezey, 1905, p. 232–234, biology; Kirkaldy, 1907a, p. 247, note; Kirkaldy, 1907b, p. 156–518, biology; Kirkaldy, 1908, p. 195, junior syn. of *Z. renardii*; Severin, et. al., 1914, p. 197, note; Fullaway, 1918, p. 12, note; Clausen, 1940, p. 589–590, note (sic. *Zellus*).
Diplocodus exsanguis : Van Duzee, 1914, p. 13, list (probable misidentification).

#### Materials

- **Type status:** Holotype. **Occurrence:** catalogNumber: UCR_ENT 00075077; occurrenceRemarks: Holotype of *Zelus renardii* Kolenati, 1857. Verbatim label info: California / Cyenaus [?] / Renardii det. Kolen. / Holotype / *Zelus renardii* Kolenati / Lectotypus *Zelus renardii* Kolenati, 1857 etik. Hecher 1996 REDV. 486/1; recordedBy: Unknown; sex: Adult Female; **Taxon:** scientificName: Zelus renardii; family: Reduviidae; genus: Zelus; scientificNameAuthorship: Kolenati, 1857; **Location:** country: USA; stateProvince: California; locality: unknown; **Identification:** identifiedBy: G. Zhang; dateIdentified: 2012; **Event:** eventDate: No date provided; **Record Level:** institutionCode: NHMW
- **Type status:** Other material. **Occurrence:** catalogNumber: UCR_ENT 00048762; occurrenceRemarks: **Holotype** of *Zelus laevicollis* Champion, 1898, junior synonym of *Zelus renardii* Kolenati, 1857 (Hart, 1986). Verbatim label info: Type / B.C.A.Rhyn.II. *Zelus laevicollis* Ch. / Holotype / Sp. figured. / Milpas, Mex., 5900 ft. Forrer. / *Zelus renardii* Kolenati det. E.R.Hart 1972; recordedBy: Forrer; sex: Adult Female; **Taxon:** scientificName: Zelus renardii; family: Reduviidae; genus: Zelus; scientificNameAuthorship: Kolenati, 1857; **Location:** country: MEXICO; stateProvince: Mexico; locality: Milpas; verbatimElevation: 1798 m; decimalLatitude: 19.03; decimalLongitude: -99.9381; georeferenceSources: Gazetteer; **Identification:** identifiedBy: G. Zhang; dateIdentified: 2012; **Event:** eventDate: No date provided; **Record Level:** institutionCode: BMNH

#### Description

Figs 167, 168, 169

***Male***: (Fig. 167a, b) Medium-sized, total length 10.57–12.98 mm (mean 12.01 mm, Suppl. material 2); robust. **COLORATION**: Anteocular lobe yellowish-brown, some specimens with darker areas on either side of mid-dorsal line. Postocular lobe yellowish-brown, usually with variable brownish-black areas dorsally but always with mid-dorsal area and area anterior to ocellus yellowish-brown. Labium yellowish-brown, some specimens with brown labrum. Antennae yellowish-brown to light reddish-brown. Anterior pronotal lobe uniformly yellowish-brown or yellowish-brown with reddish-brown to dark brown setal tracts. Posterior pronotal lobe yellowish-brown to dark brown, margins light yellowish-brown, lateral surfaces yellowish-brown. Scutellum yellowish-brown to brown, apex lighter in color. Legs yellowish-brown, some specimens with apices of tibiae reddish-brown. Hemelytron yellowish-brown to dark brown, veins of clavus and corium usually lighter in color than surrounding area. Dorsum of abdomen reddish-brown to brown, connexival margins yellowish-brown, lateral and ventral surfaces usually yellowish-brown, some specimens with reddish-brown areas laterally. Pygophore yellowish-brown. **VESTITURE**: Moderately setose. Short recumbent and variable erect setae over surface. Anteocular lobe with short recumbent setae dorsally and laterally, short erect setae on tylus and ventral surface; postocular lobe with recumbent setae dorsally, longer erect setae on lateral surface and on dorsal and ventral surfaces of posterior half. Dorsal surface of anterior pronotal lobe with short recumbent setae confined to setal tracts, remainder of surface with longer recumbent and erect setae; posterior lobe with short recumbent and erect setae and some longer erect setae lateroventrally; scutellum with semi-erect setae over surface. Abdominal dorsal setae very short, erect, lateral and ventral surfaces with short recumbent and some short to moderately long erect setae. Exposed surface of pygophore with short recumbent and some short to moderate erect setae; erect setae over apical 3/5 of parameres. **STRUCTURE: Head**: Cylindrical, L/W = 2.61. Postocular lobe moderately long; in dorsal view anteriorly gradually narrowing, posterior portion constant, slightly narrower. Eye smallish; lateral margin only slightly wider than postocular lobe; dorsal and ventral margins removed from surfaces of head. *Labium*: I: II: III = 1: 1.6: 0.4. Basiflagellomere diameter larger than that of pedicel. **Thorax**: Medial longitudinal sulcus shallow near collar, deepening posteriorly. Posterior pronotal lobe with rugulose surface; disc slightly elevated above humeral angle; humeral angle armed, with dentate projection. Scutellum long; apex angulate, not projected. *Legs*: Robust. *Hemelytron*: Attaining apex of abdomen; quadrate cell small; Cu and M of cubital cell subparallel. **GENITALIA**: (Fig. 168) *Pygophore*: Ovoid. Medial process cylindrical; slender; long; laterally somewhat compressed; erect; nearly straight; basally without protrusion; apex in posterior view modified, hooklike. *Paramere*: Cylindrical; moderately long, not reaching apex of medial process; directed toward medial process; basally narrower; curved dorsad; apical part enlarged. *Phallus*: Dorsal phallothecal sclerite shield-shaped; lateral margin recurved dorsad; apical portion of phallothecal sclerite gradually tapering, flat, lateral margin recurved; apex medially notched; posterior margin of foramen broadly concave. Struts not attached to base of dorsal phallothecal sclerite; apically missing. Basal plate arm robust; separate; converging; in lateral view basally strongly curved; bridge moderately long; extension of basal plate small, marginally expanded onto arm, lateral margins recurved.

***Female***: (Fig. 167c, d) Similar to male, except for the following. Larger than male, total length 12.14–14.25 mm (mean 13.29 mm, Suppl. material 2). Hemelytron slightly surpassing apex of abdomen.

#### Diagnosis

Can be recognized by the reddish corium; the remainder of the body surface greenish; the humeral angle with small subtuberculate projection. More robust than a very similar species, *Z. cervicalis*. Males can be recognized by the paramere apically greatly enlarged; the medial process apically curved ventrad, somewhat hooklike, more strongly than in *Z. cervicalis*, the only species that may be confused with; and the lateral margin of the dorsal phallothecal sclerite recurved.

#### Distribution

**Figure 169.**
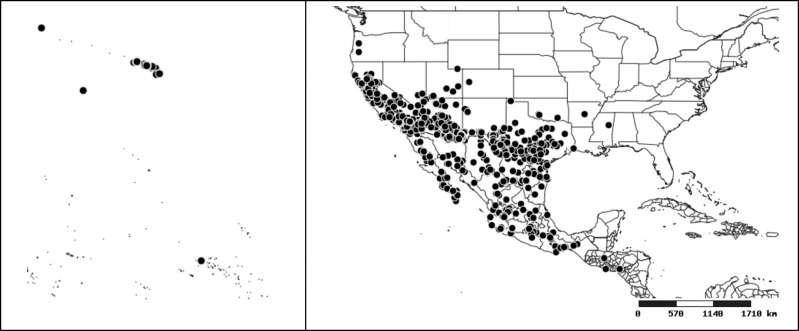
*Zelus renardii* Kolenati, 1857, specimen record map. Pacific islands (left) and American continent are not to the same scale.

Western and Southwestern US, most of Mexico and Central America (Fig. 169). Countries with records: El Salvador, Guatemala, Honduras, Mexico and USA. Previously reported to have be introduced to Hawaii (Zimmerman 1948), Chile (Curkovik et al. 2004, Elgueta and Carpintero 2004; misidentified as *Z. cervicalis* in the latter), Greece (Davranoglou 2011, Petrakis and Moulet 2011) and Spain (Vivas 2012). The current study revealed specimen records of *Z. renardii* from French Polynesia, representing the first report of this species from that region (Fig. 169, Suppl. material 1).

#### Taxon discussion

*Zelus renardii* is almost certainly sister species of *Z. cervicalis*. The two share two unique characters: the lateral margins of the dorsal phallothecal sclerite recurved and the medial process apically strongly hooked.

### Zelus rosulentus

Zhang sp. n.

urn:lsid:zoobank.org:act:0ECCAD37-B4CE-4192-A539-9B6C4008D4AB

#### Materials

- **Type status:** Holotype. **Occurrence:** catalogNumber: UCR_ENT 00009487; occurrenceRemarks: Lot#1669 - Collection code moved to this field to prevent duplication; Drake Collection; recordedBy: T. L. Erwin et al.; sex: Adult Male; preparations: Pinned; **Taxon:** scientificName: Zelus rosulentus; family: Reduviidae; genus: Zelus; scientificNameAuthorship: Zhang and Hart, 2016; **Location:** country: ECUADOR; stateProvince: Orellana; locality: Reserva Etnica Waorani, 1 km S. Onkone Gare Camp, Transect Ent.; verbatimElevation: 216 m; decimalLatitude: -0.65714; decimalLongitude: -76.453; **Identification:** identifiedBy: G. Zhang; dateIdentified: 2013; **Event:** samplingProtocol: Fogging; eventDate: 1996-09-30; **Record Level:** institutionCode: USNM
- **Type status:** Paratype. **Occurrence:** catalogNumber: UCR_ENT 00009451; recordedBy: T. L. Erwin et al.; sex: Adult Male; preparations: Pinned; **Taxon:** scientificName: Zelus rosulentus; family: Reduviidae; genus: Zelus; scientificNameAuthorship: Zhang and Hart, 2016; **Location:** country: ECUADOR; stateProvince: Orellana; locality: Reserva Etnica Waorani, 1 km S. Onkone Gare Camp, Transect Ent.; verbatimElevation: 216 m; decimalLatitude: -0.65714; decimalLongitude: -76.453; **Identification:** identifiedBy: G. Zhang; dateIdentified: 2013; **Event:** eventDate: 1996-10-04; **Record Level:** institutionCode: USNM
- **Type status:** Paratype. **Occurrence:** catalogNumber: UCR_ENT 00009452; recordedBy: T. L. Erwin et al.; sex: Adult Male; preparations: Pinned; **Taxon:** scientificName: Zelus rosulentus; family: Reduviidae; genus: Zelus; scientificNameAuthorship: Zhang and Hart, 2016; **Location:** country: ECUADOR; stateProvince: Orellana; locality: Reserva Etnica Waorani, 1 km S. Onkone Gare Camp, Transect Ent.; verbatimElevation: 216 m; decimalLatitude: -0.65714; decimalLongitude: -76.453; **Identification:** identifiedBy: G. Zhang; dateIdentified: 2013; **Event:** eventDate: 1995-02-12; **Record Level:** institutionCode: USNM
- **Type status:** Paratype. **Occurrence:** catalogNumber: UCR_ENT 00009453; recordedBy: T. L. Erwin et al.; sex: Adult Male; preparations: Pinned; **Taxon:** scientificName: Zelus rosulentus; family: Reduviidae; genus: Zelus; scientificNameAuthorship: Zhang and Hart, 2016; **Location:** country: ECUADOR; stateProvince: Orellana; locality: Reserva Etnica Waorani, 1 km S. Onkone Gare Camp, Transect Ent.; verbatimElevation: 216 m; decimalLatitude: -0.65714; decimalLongitude: -76.453; **Identification:** identifiedBy: G. Zhang; dateIdentified: 2013; **Event:** eventDate: 1995-07-02; **Record Level:** institutionCode: USNM
- **Type status:** Paratype. **Occurrence:** catalogNumber: UCR_ENT 00009454; recordedBy: T. L. Erwin et al.; sex: Adult Male; preparations: Pinned; **Taxon:** scientificName: Zelus rosulentus; family: Reduviidae; genus: Zelus; scientificNameAuthorship: Zhang and Hart, 2016; **Location:** country: ECUADOR; stateProvince: Orellana; locality: Reserva Etnica Waorani, 1 km S. Onkone Gare Camp, Transect Ent.; verbatimElevation: 216 m; decimalLatitude: -0.65714; decimalLongitude: -76.453; **Identification:** identifiedBy: G. Zhang; dateIdentified: 2013; **Event:** eventDate: 1994-10-09; **Record Level:** institutionCode: USNM
- **Type status:** Paratype. **Occurrence:** catalogNumber: UCR_ENT 00009484; occurrenceRemarks: Lot#1558 - Collection code moved to this field to prevent duplication; Drake Collection; recordedBy: T. L. Erwin et al.; sex: Adult Male; preparations: Pinned; **Taxon:** scientificName: Zelus rosulentus; family: Reduviidae; genus: Zelus; scientificNameAuthorship: Zhang and Hart, 2016; **Location:** country: ECUADOR; stateProvince: Orellana; locality: Reserva Etnica Waorani, 1 km S. Onkone Gare Camp, Transect Ent.; verbatimElevation: 216 m; decimalLatitude: -0.65714; decimalLongitude: -76.453; **Identification:** identifiedBy: G. Zhang; dateIdentified: 2013; **Event:** samplingProtocol: Fogging; eventDate: 1996-06-21; **Record Level:** institutionCode: USNM
- **Type status:** Paratype. **Occurrence:** catalogNumber: UCR_ENT 00009485; occurrenceRemarks: Lot#1007 - Collection code moved to this field to prevent duplication; Drake Collection; recordedBy: T. L. Erwin et al.; sex: Adult Male; preparations: Pinned; **Taxon:** scientificName: Zelus rosulentus; family: Reduviidae; genus: Zelus; scientificNameAuthorship: Zhang and Hart, 2016; **Location:** country: ECUADOR; stateProvince: Orellana; locality: Reserva Etnica Waorani, 1 km S. Onkone Gare Camp, Transect Ent.; verbatimElevation: 216 m; decimalLatitude: -0.65714; decimalLongitude: -76.453; **Identification:** identifiedBy: G. Zhang; dateIdentified: 2013; **Event:** samplingProtocol: Fogging; eventDate: 1995-02-10; **Record Level:** institutionCode: USNM
- **Type status:** Paratype. **Occurrence:** catalogNumber: UCR_ENT 00009486; occurrenceRemarks: Lot#1451 - Collection code moved to this field to prevent duplication; Drake Collection; recordedBy: T. L. Erwin et al.; sex: Adult Male; preparations: Pinned; **Taxon:** scientificName: Zelus rosulentus; family: Reduviidae; genus: Zelus; scientificNameAuthorship: Zhang and Hart, 2016; **Location:** country: ECUADOR; stateProvince: Orellana; locality: Reserva Etnica Waorani, 1 km S. Onkone Gare Camp, Transect Ent.; verbatimElevation: 216 m; decimalLatitude: -0.65714; decimalLongitude: -76.453; **Identification:** identifiedBy: G. Zhang; dateIdentified: 2013; **Event:** samplingProtocol: Fogging; eventDate: 1996-02-07; **Record Level:** institutionCode: USNM
- **Type status:** Paratype. **Occurrence:** catalogNumber: UCR_ENT 00009488; occurrenceRemarks: Lot#1043 - Collection code moved to this field to prevent duplication; Drake Collection; recordedBy: T. L. Erwin et al.; sex: Adult Male; preparations: Pinned; **Taxon:** scientificName: Zelus rosulentus; family: Reduviidae; genus: Zelus; scientificNameAuthorship: Zhang and Hart, 2016; **Location:** country: ECUADOR; stateProvince: Orellana; locality: Reserva Etnica Waorani, 1 km S. Onkone Gare Camp, Transect Ent.; verbatimElevation: 216 m; decimalLatitude: -0.65714; decimalLongitude: -76.453; **Identification:** identifiedBy: G. Zhang; dateIdentified: 2013; **Event:** samplingProtocol: Fogging; eventDate: 1995-02-12; **Record Level:** institutionCode: USNM

#### Description

Figs 170, 171, 172

***Male***: (Fig. 170) Medium-sized, total length 11.49–12.39 mm (mean 11.97 mm, Table 4.2); very slender. **COLORATION**: Entire surface pink; areas on hemelytron lighter, sometimes apex of membrane darkened. Femora subapically with single dark band. **VESTITURE**: Moderately setose. **STRUCTURE: Head**: Cylindrical, L/W = 2.00. Postocular lobe long; in dorsal view distinctly narrowing through anterior 2/3, posterior 1/3 constant, tube-like. Eye prominent; lateral margin only slightly wider than postocular lobe; dorsal margin removed from postocular transverse groove, ventral margin attaining ventral surface of head in lateral view. *Labium*: I: II: III = 1: 2.1: 0.5. Basiflagellomere diameter slightly larger than that of pedicel. **Thorax**: Anterolateral angle rounded, without projection; medial longitudinal sulcus shallow near collar, deepening posteriorly. Posterior pronotal lobe with smooth surface; disc distinctly elevated above humeral angle; humeral angle armed, with short tuberculate processes. Apex angulate. *Legs*: Very slender. *Hemelytron*: Surpassing apex of abdomen by about length of abdominal segment seven; quadrate cell large and broad; Cu and M of cubital cell subparallel. **GENITALIA**: (Fig. 171) *Pygophore*: Rounded; very slightly expanded laterally near base of paramere in dorsal view. Medial process broadly triangular, base indistinct; short; semi-erect; straight; basally without protrusion; apex in posterior view blunt, without modification, very slightly folded ventrad. *Paramere*: Cylindrical; moderately long, not exceeding medial process; directed posteriad and slightly ventrad; nearly straight; apical part a little enlarged. *Phallus*: Dorsal phallothecal sclerite somewhat squarish; apical portion of phallothecal sclerite not distinctly tapered, surface nearly flat, lateral margin narrowly rounded; apex deeply emarginate; posterior margin of foramen nearly straight. Struts not evidently attached to dorsal phallothecal sclerite; fused throughout. Basal plate arm slender; separate; in lateral view very slightly curved; bridge short; extension of basal plate small, confined to apex of basal plate arm.

***Female***: unknown.

#### Diagnosis

The uniquely reddish coloration of the entire body makes this species easy to recognize. The medial process is highly reduced and rather indistinct, separating *Z. rosulentus* from other members of the *Zelus tetracanthus* species group (Fig. 2).

#### Etymology

The specific epithet indicates the reddish-pink coloration of this species.

#### Distribution

**Figure 172.**
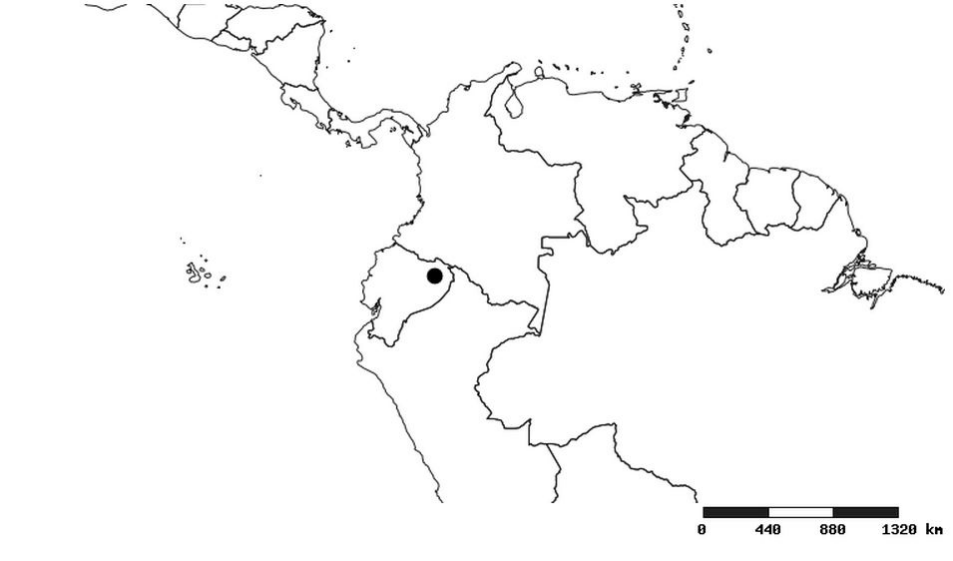
*Zelus rosulentus* Zhang & Hart, sp. n., specimen record map

South America (Fig. 172). Only known from Ecuador.

### Zelus ruficeps

Stål, 1862

Zelus ruficeps Stål, 1862, p. 453–454, orig. descr.; Stål, 1872, p. 90, cat. (subgenus *Diplodus*); Lethierry and Severin, 1896, p. 153, cat.; Champion, 1898, p. 256, Tab. 15. fig. 15, note and fig.; Kuhlgatz, 1902, p. 266, note; Fracker, 1913, p. 239, 240, list (subgenus *Diplodus*); Wygodzinsky, 1949a, p. 50, checklist; Maldonado, 1990, p. 331, cat.
Diplodus ruficeps : Walker, 1873, p. 124, cat.; Uhler, 1886, p. 24, checklist.

#### Materials

- **Type status:** Lectotype. **Occurrence:** catalogNumber: UCR_ENT 00041010; occurrenceRemarks: Lectotype of *Zelus ruficeps* Stål, 1862 (**New Designation** by Zhang, Hart & Weirauch, 2016). Verbatim label info: Mexico / Salle/ ruficeps Stal / Lectotype *Zelus ruficeps* Stal / designated by E.R.Hart / Paratypus / NHRS-GULI 000000347; recordedBy: salle; sex: Adult Male; otherCatalogNumbers: NHRS-GULI 000000347; **Taxon:** scientificName: Zelus ruficeps; family: Reduviidae; genus: Zelus; scientificNameAuthorship: Stål, 1862; **Location:** country: MEXICO; stateProvince: None or Unknown; locality: unknown; **Identification:** identifiedBy: G. Zhang; dateIdentified: 2012; **Event:** eventDate: No date provided; **Record Level:** institutionCode: NHRS
- **Type status:** Allolectotype. **Occurrence:** occurrenceRemarks: Allolectotype of *Zelus ruficeps* Stål, 1862 (**New Designation** by Zhang, Hart & Weirauch, 2016); recordedBy: salle; sex: Adult Female; **Taxon:** scientificName: Zelus ruficeps; family: Reduviidae; genus: Zelus; scientificNameAuthorship: Stål, 1862; **Location:** country: MEXICO; stateProvince: None or Unknown; locality: unknown; **Event:** eventDate: No date provided; **Record Level:** institutionCode: NHRS
- **Type status:** Paralectotype. **Occurrence:** occurrenceRemarks: Paralectotype of *Zelus ruficeps* Stål, 1862 (**New Designation** by Zhang, Hart & Weirauch, 2016); recordedBy: salle; sex: Adult Female; **Taxon:** scientificName: Zelus ruficeps; family: Reduviidae; genus: Zelus; scientificNameAuthorship: Stål, 1862; **Location:** country: MEXICO; stateProvince: None or Unknown; locality: unknown; **Event:** eventDate: No date provided; **Record Level:** institutionCode: NHRS
- **Type status:** Paralectotype. **Occurrence:** occurrenceRemarks: Paralectotype of *Zelus ruficeps* Stål, 1862 (**New Designation** by Zhang, Hart & Weirauch, 2016); recordedBy: salle; sex: Adult Male; **Taxon:** scientificName: Zelus ruficeps; family: Reduviidae; genus: Zelus; scientificNameAuthorship: Stål, 1862; **Location:** country: MEXICO; stateProvince: None or Unknown; locality: unknown; **Event:** eventDate: No date provided; **Record Level:** institutionCode: NHRS
- **Type status:** Paralectotype. **Occurrence:** occurrenceRemarks: Paralectotype of *Zelus ruficeps* Stål, 1862 (**New Designation** by Zhang, Hart & Weirauch, 2016). Bears label: Mexico, coll. Signoret/ruficeps det. Stal; recordedBy: Signoret; sex: Adult Female; **Taxon:** scientificName: Zelus ruficeps; family: Reduviidae; genus: Zelus; scientificNameAuthorship: Stål, 1862; **Location:** country: MEXICO; stateProvince: None or Unknown; locality: unknown; **Event:** eventDate: No date provided; **Record Level:** institutionCode: NHMW
- **Type status:** Paralectotype. **Occurrence:** occurrenceRemarks: Paralectotype of *Zelus ruficeps* Stål, 1862 (**New Designation** by Zhang, Hart & Weirauch, 2016). Bears label: Mexico, coll. Signoret/ruficeps det. Stal; recordedBy: Signoret; sex: Adult Female; **Taxon:** scientificName: Zelus ruficeps; family: Reduviidae; genus: Zelus; scientificNameAuthorship: Stål, 1862; **Location:** country: MEXICO; stateProvince: None or Unknown; locality: unknown; **Event:** eventDate: No date provided; **Record Level:** institutionCode: NHMW

#### Description

Figs 173, 174, 175

***Male***: (Fig. 173a, b) Medium-sized, total length 13.24–16.83 mm (mean 14.93 mm, Suppl. material 2); robust. **COLORATION**: Orangish to reddish, with black areas. Head orangish or reddish-brown; dark areas on postocular lobe in some specimens. Anterior pronotal lobe orangish or reddish-brown; posterior margin dark, sometimes occupying more than 1/2 of surface. Posterior pronotal lobe, corium and clavus orangish-brown; sometimes with dark areas on each. Scutellum dark brown; margins yellowish-brown. Membrane dark brown. Legs not distinctly banded; femoral apical portion usually reddish, occasionally dark, single small black band subapically, sometimes very faint black marking medially. Lateral and ventral surfaces orangish to reddish-brown; variable black areas on pleura and abdomen; dark stripe along posterior margin of each segment, width variable; pygophore usually reddish. **VESTITURE**: Densely setose. Dorsal surface of head with fine to stiff erect setae and some recumbent setae, lateral and ventral surfaces with erect and recumbent setae. Anterior pronotal lobe with recumbent and erect setae, confined to setal tracts, erect setae predominant; posterior pronotal lobe with erect setae and some recumbent setae. Abdomen with short, recumbent setae, interspersed with erect setae. **STRUCTURE: Head**: Cylindrical, L/W = 2.21. Postocular lobe relatively short; in dorsal view distinctly narrowing through anterior 1/2, posterior 1/2 constant, tube-like. Eye smallish; lateral margin only slightly wider than postocular lobe; dorsal margin attaining postocular transverse groove, ventral margin removed from ventral surface of head in lateral view. *Labium*: I: II: III = 1: 1.4: 0.4. Basiflagellomere diameter subequal to that of pedicel. **Thorax**: Anterolateral angle bearing small protuberance; medial longitudinal sulcus distinct throughout. Posterior pronotal lobe with rugulose surface; disc distinctly elevated above humeral angle; humeral angle armed, with short tuberculate processes. Scutellum moderately long; apex angulate. *Legs*: Moderately robust. *Hemelytron*: Slightly surpassing apex of abdomen, not more than length of abdominal segment seven; quadrate cell large and broad; Cu and M of cubital cell converging towards R. **GENITALIA**: (Fig. 174) *Pygophore*: Rounded; slightly expanded laterally near base of paramere in dorsal view; posteriorly expanded sac-like sclerite between parameres and medial process. Medial process cylindrical; slender; moderately long; posteriorly directed; nearly straight; apex in posterior view rounded, with small sharp lateral projections. *Paramere*: Cylindrical; moderately long, not reaching apex of medial process; directed posteriad; nearly straight; apical part not enlarged. *Phallus*: Dorsal phallothecal sclerite somewhat squarish; lateral expansion arising close to base; apical portion of phallothecal sclerite not distinctly tapered, flat, lateral margin narrowly angulate; apex rounded; posterior margin of foramen broadly inversely V-shaped. Struts attached to dorsal phallothecal sclerite; apically fused; basally separate throughout. Basal plate arm moderately robust; basally fused; in lateral view very slightly curved; bridge extremely short; extension of basal plate small, marginally expanded onto arm.

***Female***: (Fig. 173c, d) Similar to male, except for the following. Larger than male, total length 16.46–20.01 mm (mean 17.84 mm, Suppl. material 2). Usually lighter than male and larger areas of red and yellow.

#### Diagnosis

The combination of relatively large size, stout body, the reddish head and parts of body can separate both sexes of this species from other species of *Zelus*. Among males of the *Zelus armillatus* species group, *Z. ruficeps* has some of the most delicate medial process. Females may be confused with *Z. grassans*, but are much larger and the humeral angle is equipped with dentate process, contrasting to the nearly rounded humeral angle of *Z. grassans*.

#### Distribution

**Figure 175.**
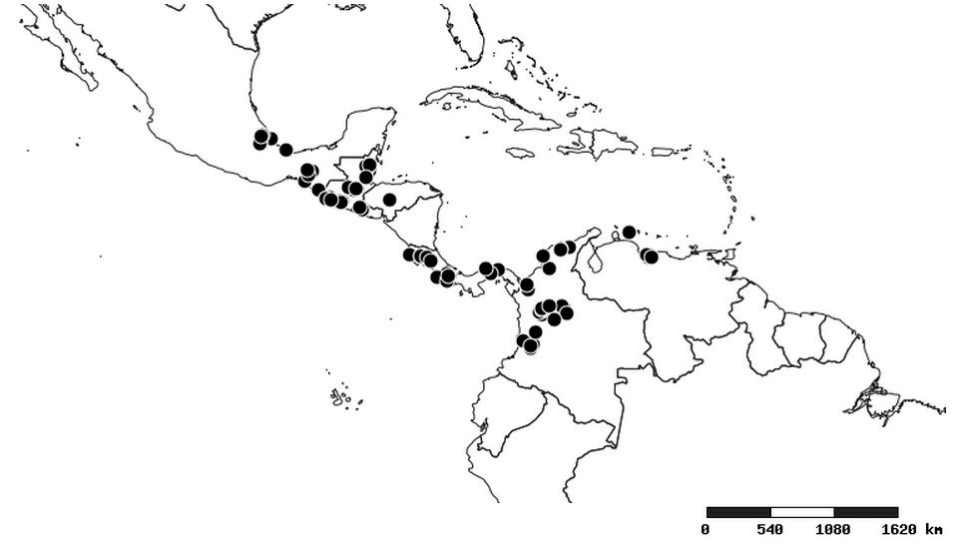
*Zelus ruficeps* Stål, 1862, specimen record map

Southern Mexico to Northern South America (Fig. 175). Countries with records: Belize, Colombia, Costa Rica, Curaçao, El Salvador, Guatemala, Mexico, Panama and Venezuela.

### Zelus russulumus

Zhang & Hart sp. n.

urn:lsid:zoobank.org:act:9A276EA9-8711-4E7C-BA87-FDA9040DF5C1

#### Materials

- **Type status:** Holotype. **Occurrence:** catalogNumber: UCR_ENT 00069895; recordedBy: S.M. Klages; sex: Adult Male; **Taxon:** scientificName: Zelus russulumus; family: Reduviidae; genus: Zelus; scientificNameAuthorship: Zhang and Hart, 2016; **Location:** country: BRAZIL; stateProvince: Amazonas; locality: Manacapuru; decimalLatitude: 3.3; decimalLongitude: -60.61667; georeferenceSources: Gazetteer; **Identification:** identifiedBy: G. Zhang; dateIdentified: 2013; **Event:** eventDate: 1905-04-11; **Record Level:** institutionCode: KU
- **Type status:** Paratype. **Occurrence:** catalogNumber: UCR_ENT 00009464; recordedBy: J. E. Eger; sex: Adult Male; **Taxon:** scientificName: Zelus russulumus; family: Reduviidae; genus: Zelus; scientificNameAuthorship: Zhang and Hart, 2016; **Location:** country: BRAZIL; stateProvince: Rondonia; locality: 62 km S Ariquemes, Fazenda Rancho Grande; verbatimElevation: 300 m; decimalLatitude: -10.3; decimalLongitude: -62.86666; **Identification:** identifiedBy: G. Zhang; dateIdentified: 2013; **Event:** eventDate: 1992-03-30 to 1992-04-10; **Record Level:** institutionCode: USNM
- **Type status:** Paratype. **Occurrence:** catalogNumber: UCR_ENT 00009465; recordedBy: J. E. Eger; sex: Adult Male; **Taxon:** scientificName: Zelus russulumus; family: Reduviidae; genus: Zelus; scientificNameAuthorship: Zhang and Hart, 2016; **Location:** country: BRAZIL; stateProvince: Rondonia; locality: 62 km S Ariquemes, Fazenda Rancho Grande; verbatimElevation: 300 m; decimalLatitude: -10.3; decimalLongitude: -62.86666; **Identification:** identifiedBy: G. Zhang; dateIdentified: 2013; **Event:** eventDate: 1992-03-30 to 1992-04-10; **Record Level:** institutionCode: USNM
- **Type status:** Paratype. **Occurrence:** catalogNumber: UCR_ENT 00009466; occurrenceRemarks: Drake collection; recordedBy: J. E. Eger; sex: Adult Male; **Taxon:** scientificName: Zelus russulumus; family: Reduviidae; genus: Zelus; scientificNameAuthorship: Zhang and Hart, 2016; **Location:** country: BRAZIL; stateProvince: Rondonia; locality: 62 km S Ariquemes, Fazenda Rancho Grande; verbatimElevation: 300 m; decimalLatitude: -10.3; decimalLongitude: -62.86666; **Identification:** identifiedBy: G. Zhang; dateIdentified: 2013; **Event:** eventDate: 1996-12-03 to 1996-12-15; **Record Level:** institutionCode: USNM
- **Type status:** Paratype. **Occurrence:** catalogNumber: UCR_ENT 00009467; occurrenceRemarks: Drake collection; recordedBy: J. E. Eger; sex: Adult Female; **Taxon:** scientificName: Zelus russulumus; family: Reduviidae; genus: Zelus; scientificNameAuthorship: Zhang and Hart, 2016; **Location:** country: BRAZIL; stateProvince: Rondonia; locality: 62 km S Ariquemes, Fazenda Rancho Grande; verbatimElevation: 300 m; decimalLatitude: -10.3; decimalLongitude: -62.86666; **Identification:** identifiedBy: G. Zhang; dateIdentified: 2013; **Event:** eventDate: 1992-03-30 to 1992-04-10; **Record Level:** institutionCode: USNM
- **Type status:** Paratype. **Occurrence:** catalogNumber: UCR_ENT 00009468; occurrenceRemarks: Drake collection; recordedBy: J. E. Eger; sex: Adult Female; **Taxon:** scientificName: Zelus russulumus; family: Reduviidae; genus: Zelus; scientificNameAuthorship: Zhang and Hart, 2016; **Location:** country: BRAZIL; stateProvince: Rondonia; locality: 62 km S Ariquemes, Fazenda Rancho Grande; verbatimElevation: 300 m; decimalLatitude: -10.3; decimalLongitude: -62.86666; **Identification:** identifiedBy: G. Zhang; dateIdentified: 2013; **Event:** eventDate: 1996-12-03 to 1996-12-15; **Record Level:** institutionCode: USNM
- **Type status:** Paratype. **Occurrence:** catalogNumber: UCR_ENT 00009469; occurrenceRemarks: Drake collection; recordedBy: J. E. Eger; sex: Adult Female; **Taxon:** scientificName: Zelus russulumus; family: Reduviidae; genus: Zelus; scientificNameAuthorship: Zhang and Hart, 2016; **Location:** country: BRAZIL; stateProvince: Rondonia; locality: 62 km S Ariquemes, Fazenda Rancho Grande; verbatimElevation: 300 m; decimalLatitude: -10.3; decimalLongitude: -62.86666; **Identification:** identifiedBy: G. Zhang; dateIdentified: 2013; **Event:** eventDate: 1997-11-04 to 1997-11-16; **Record Level:** institutionCode: USNM
- **Type status:** Paratype. **Occurrence:** catalogNumber: UCR_ENT 00009470; occurrenceRemarks: Drake collection; recordedBy: J. E. Eger; sex: Adult Female; **Taxon:** scientificName: Zelus russulumus; family: Reduviidae; genus: Zelus; scientificNameAuthorship: Zhang and Hart, 2016; **Location:** country: BRAZIL; stateProvince: Rondonia; locality: 62 km S Ariquemes, Fazenda Rancho Grande; verbatimElevation: 300 m; decimalLatitude: -10.3; decimalLongitude: -62.86666; **Identification:** identifiedBy: G. Zhang; dateIdentified: 2013; **Event:** eventDate: 1997-11-04 to 1997-11-16; **Record Level:** institutionCode: USNM
- **Type status:** Paratype. **Occurrence:** catalogNumber: UCR_ENT 00009471; occurrenceRemarks: Drake collection; recordedBy: J. E. Eger; sex: Adult Female; **Taxon:** scientificName: Zelus russulumus; family: Reduviidae; genus: Zelus; scientificNameAuthorship: Zhang and Hart, 2016; **Location:** country: BRAZIL; stateProvince: Rondonia; locality: 62 km S Ariquemes, Fazenda Rancho Grande; verbatimElevation: 300 m; decimalLatitude: -10.3; decimalLongitude: -62.86666; **Identification:** identifiedBy: G. Zhang; dateIdentified: 2013; **Event:** eventDate: 1997-11-04 to 1997-11-16; **Record Level:** institutionCode: USNM
- **Type status:** Paratype. **Occurrence:** catalogNumber: UCR_ENT 00009501; occurrenceRemarks: Drake Collection; recordedBy: J. E. Eger; sex: Adult Female; **Taxon:** scientificName: Zelus russulumus; family: Reduviidae; genus: Zelus; scientificNameAuthorship: Zhang and Hart, 2016; **Location:** country: ECUADOR; stateProvince: Napo; locality: Rio Blanco Ecological Reserve, 6 km E Puerto Misahuali, along Rio Napo; verbatimElevation: 518 m; decimalLatitude: -1.05037; decimalLongitude: -77.62705; georeferenceSources: Google Earth; **Identification:** identifiedBy: G. Zhang; dateIdentified: 2013; **Event:** eventDate: 1998-09-07; **Record Level:** institutionCode: USNM
- **Type status:** Paratype. **Occurrence:** catalogNumber: UCR_ENT 00029293; recordedBy: W. M. Mann; sex: Adult Female; **Taxon:** scientificName: Zelus russulumus; family: Reduviidae; genus: Zelus; scientificNameAuthorship: Zhang and Hart, 2016; **Location:** country: BOLIVIA; stateProvince: Pando; locality: Cachuela Esperanza; decimalLatitude: -10.5375; decimalLongitude: -65.5815; georeferenceSources: Google Earth; **Identification:** identifiedBy: G. Zhang; dateIdentified: 2013; **Event:** eventDate: no date provided; **Record Level:** institutionCode: USNM
- **Type status:** Paratype. **Occurrence:** catalogNumber: UCR_ENT 00029294; occurrenceRemarks: Previously designated as 'allotype' of his manuscript name *Zelus russulumus* by Hart (1972), a type status not used in the formal publication of this new species name (Zhang et al., 2016); recordedBy: E.A. Chapin; sex: Adult Female; **Taxon:** scientificName: Zelus russulumus; family: Reduviidae; genus: Zelus; scientificNameAuthorship: Zhang and Hart, 2016; **Location:** country: COLOMBIA; stateProvince: Meta; locality: Villavicencio; decimalLatitude: 4.15333; decimalLongitude: -73.635; georeferenceSources: Google Earth; **Identification:** identifiedBy: G. Zhang; dateIdentified: 2013; **Event:** eventDate: 1946-05-11; **Record Level:** institutionCode: USNM
- **Type status:** Paratype. **Occurrence:** catalogNumber: UCR_ENT 00029301; occurrenceRemarks: Drake Collection; recordedBy: J. E. Eger; sex: Adult Male; **Taxon:** scientificName: Zelus russulumus; family: Reduviidae; genus: Zelus; scientificNameAuthorship: Zhang and Hart, 2016; **Location:** country: BRAZIL; stateProvince: Rondonia; locality: 62 km SW of Ariquemes, near Fzda. Rancho Grande; decimalLatitude: -10.32921; decimalLongitude: -63.46881; **Identification:** identifiedBy: G. Zhang; dateIdentified: 2013; **Event:** eventDate: 1992-03-30 to 1992-04-10; **Record Level:** institutionCode: USNM
- **Type status:** Paratype. **Occurrence:** catalogNumber: UCR_ENT 00029303; occurrenceRemarks: Drake Collection; recordedBy: J. E. Eger; sex: Adult Male; **Taxon:** scientificName: Zelus russulumus; family: Reduviidae; genus: Zelus; scientificNameAuthorship: Zhang and Hart, 2016; **Location:** country: BRAZIL; stateProvince: Rondonia; locality: 62 km SW of Ariquemes, near Fzda. Rancho Grande; decimalLatitude: -10.32921; decimalLongitude: -63.46881; **Identification:** identifiedBy: G. Zhang; dateIdentified: 2013; **Event:** eventDate: 1992-03-30 to 1992-04-10; **Record Level:** institutionCode: USNM
- **Type status:** Paratype. **Occurrence:** catalogNumber: UCR_ENT 00029306; occurrenceRemarks: Drake Collection; recordedBy: L. E. Peña; sex: Adult Female; **Taxon:** scientificName: Zelus russulumus; family: Reduviidae; genus: Zelus; scientificNameAuthorship: Zhang and Hart, 2016; **Location:** country: COLOMBIA; stateProvince: Vaupes; locality: Mitu; verbatimElevation: 184 m; decimalLatitude: 1.1983; decimalLongitude: -70.1733; georeferenceSources: Gazetteer; **Identification:** identifiedBy: G. Zhang; dateIdentified: 2013; **Event:** eventDate: 1990-07-06 to 1990-07-17; **Record Level:** institutionCode: USNM
- **Type status:** Paratype. **Occurrence:** catalogNumber: UCR_ENT 00037215; recordedBy: S.L. Heydon; sex: Adult Male; **Taxon:** scientificName: Zelus russulumus; family: Reduviidae; genus: Zelus; scientificNameAuthorship: Zhang and Hart, 2016; **Location:** country: BRAZIL; stateProvince: Rondonia; locality: 62 km S Ariquemes, RO Fazenda Rancho Grande; decimalLatitude: -10.46581; decimalLongitude: -63.0996; georeferenceSources: Google Earth; **Identification:** identifiedBy: G. Zhang; dateIdentified: 2013; **Event:** eventDate: 1991-11-25; **Record Level:** institutionCode: UCD
- **Type status:** Paratype. **Occurrence:** catalogNumber: UCR_ENT 00071241; recordedBy: D. A. Rider and J. E. Eger; sex: Adult Male; **Taxon:** scientificName: Zelus russulumus; family: Reduviidae; genus: Zelus; scientificNameAuthorship: Zhang and Hart, 2016; **Location:** country: BRAZIL; stateProvince: Rondonia; locality: 62 km S. Ariquemes, Fazenda Rancho Grande; verbatimElevation: 187 m; decimalLatitude: -10.29801; decimalLongitude: -62.86806; georeferenceSources: Map - local/regional; **Identification:** identifiedBy: G. Zhang; dateIdentified: 2013; **Event:** eventDate: 1990-12-06 to 1990-12-15; **Record Level:** institutionCode: TAMU
- **Type status:** Paratype. **Occurrence:** catalogNumber: UCR_ENT 00071254; recordedBy: Borgmeier and S. Lopes; sex: Adult Male; **Taxon:** scientificName: Zelus russulumus; family: Reduviidae; genus: Zelus; scientificNameAuthorship: Zhang and Hart, 2016; **Location:** country: BRAZIL; stateProvince: Goias; locality: Campinas; decimalLatitude: -16.66785; decimalLongitude: -49.29149; georeferenceSources: Google Earth; **Identification:** identifiedBy: G. Zhang; dateIdentified: 2013; **Event:** eventDate: 2.1.936; **Record Level:** institutionCode: TAMU

#### Description

Figs 176, 177, 178

***Male***: (Fig. 176a, b) Medium-sized, total length 12.21–13.94 mm (mean 13.14 mm, Suppl. material 2); slender. **COLORATION**: Head reddish-brown, anterior to antennal insertion and posterior third of postocular lobe lighter. Rest of surface of body nearly uniformly blackish-brown, area around humeral angle lighter, somewhat reddish. Membrane with blue, purple iridescence. **VESTITURE**: Sparsely setose. Dark, moderately dense, short, erect, spine-like setae on dorsum of head, some curved on postocular lobe; ventral surface with sparse, short, erect and recumbent setae, few long setae. Pronotal dorsum nearly glabrous, very sparse, short, erect and recumbent spine-like setae; lateral surface with sparse, erect to recumbent, spine-like setae; setal tracts on anterior lobe very reduced. Pleura with sparse, erect setae and moderately dense, recumbent setae. Abdomen with sparse, short, semi-erect or recumbent setae, intermixed with few longer setae. Pygophore with sparse, short to long, semi-erect or erect setae; Paramere apical half with dense, long setae, nearly as long as medial process. **STRUCTURE: Head**: Cylindrical, L/W = 2.21. Postocular lobe long; in dorsal view distinctly narrowing through anterior 2/3, posterior 1/3 constant, tube-like. Eye prominent; lateral margin much wider than postocular lobe; dorsal margin removed from postocular transverse groove, ventral margin attaining ventral surface of head in lateral view. *Labium*: I: II: III = 1: 2.0: 0.5. Basiflagellomere diameter larger than that of pedicel. **Thorax**: Anterolateral angle bearing small projection; medial longitudinal sulcus shallow near collar, deepening posteriorly. Posterior pronotal lobe with rugulose surface; disc distinctly elevated above humeral angle; humeral angle armed, with dentate or spinous process. Scutellum moderately long; apex angulate, very slightly projected upward. *Legs*: Very slender. *Hemelytron*: Surpassing apex of abdomen by about length of abdominal segment seven; quadrate cell small, relatively broad; Cu and M of cubital cell subparallel. **GENITALIA**: (Fig. 177) *Pygophore*: Ovoid; mid-lateral fold adjacent to paramere insertion inconspicuous; not expanded laterally in dorsal view. Medial process expanded laterally; broad; long; anteroposteriorly compressed; erect; straight; apex in posterior view rounded, subapical transverse hooklike bridge. *Paramere*: Cylindrical; long, surpassing medial process; curved ventrad at mid-point, apex recurved. *Phallus*: Dorsal phallothecal sclerite shield-shaped, sclerite absent laterad to basal arms; lateral longitudinal blade-like heavy sclerotization, pressed against phallothecal sclerite, reaching apical margin; area between these raised; apical portion of phallothecal sclerite not distinctly tapered, slightly convex, lateral margin narrowly angulate, angulation ending anteriorly in sharp, dorsad projection; apex with small medial emargination; posterior margin of foramen broadly concave. Struts attached to dorsal phallothecal sclerite; apically separate, connected by bridge; basally separate throughout. Basal plate arm robust; basally fused; in lateral view strongly curved at midpoint; bridge extremely short; extension of basal plate expanded onto arm.

***Female***: (Fig. 176c, d) Similar to male, except for the following. Larger than male, total length 17.52–19.39 mm (mean 18.23 mm, Suppl. material 2). Spinous process on humeral angle long.

#### Diagnosis

The dorsal coloration nearly uniformly dark brown, the head reddish-brown, and the membrane with blue or green iridescence. Most similar to *Z. erythrocephalus* and *Z. paracephalus*; can be distinguished by the medial process wider than that in *Z. erythrocephalus* and narrower than *Z. paracephalus*. Females of *Z. erythrocephalus*, *Z. paracephalus* and *Z. russulumus* ​are difficult to separate.

#### Etymology

The species epithet refers to the reddish-brown area on the membrane.

#### Distribution

**Figure 178.**
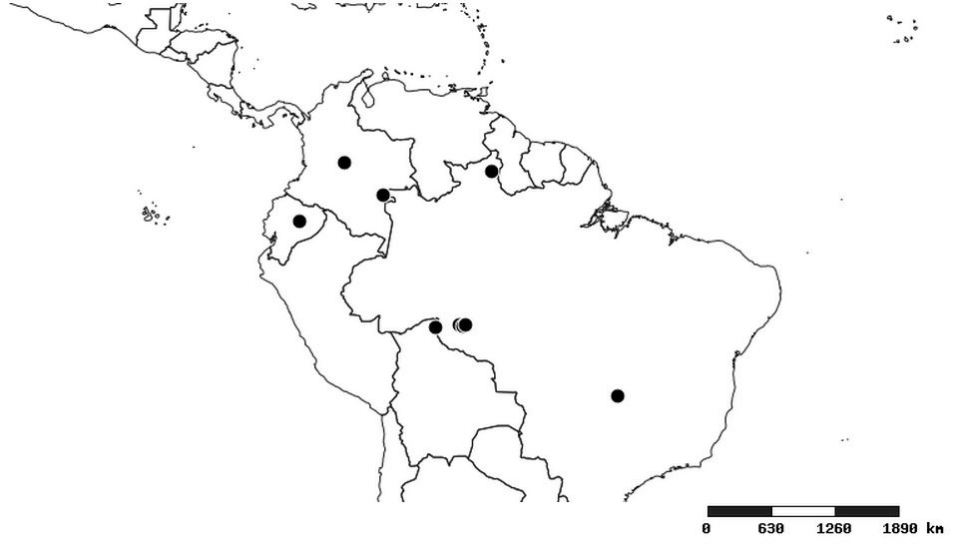
*Zelus russulumus* Zhang & Hart, sp. n., specimen record map

South America (Fig. 178). Countries with records: Bolivia, Brazil, Colombia and Ecuador.

### Zelus spatulosus

Zhang & Hart sp. n.

urn:lsid:zoobank.org:act:4C46CE25-39FD-4F23-997F-797AEBAF1A6B

#### Materials

- **Type status:** Holotype. **Occurrence:** catalogNumber: UCR_ENT 00008002; occurrenceRemarks: Additional labels: J C Lutz Collection 1961, Property USNM; recordedBy: J. J. White; sex: Adult Male; **Taxon:** scientificName: Zelus spatulosus; family: Reduviidae; genus: Zelus; scientificNameAuthorship: Zhang and Hart, 2016; **Location:** country: BELIZE; stateProvince: Toledo; locality: Rio Grande; decimalLatitude: 16.13333; decimalLongitude: -88.75; georeferenceSources: Gazetteer; **Identification:** identifiedBy: G. Zhang; dateIdentified: 2013; **Event:** eventDate: 1931-07-12; **Record Level:** institutionCode: USNM

#### Description

Figs 179, 180, 181

***Male***: (Fig. 179) Medium-sized, total length 13.17 mm (n=1); slender. **COLORATION**: Dorsum of anteocular lobe dark reddish-brown, remainder of surface yellowish-brown to light reddish-brown. Postocular lobe dark reddish-brown, light reddish-brown areas around ocellus, behind compound eyes laterally, and on wide mid-ventral line, yellowish-brown mid-dorsal line. Rostrum and antennae dark reddish-brown. Anterior pronotal lobe reddish-brown with light reddish-brown area lateroventrally. Posterior lobe slightly lighter than anterior lobe, lateral margins and adjacent area of lateral surface light reddish-brown. Scutellum reddish-brown. Basal area and apical part of profemur dark reddish-brown, remainder variably banded, light to medium reddish-brown, basal area, apex and two bands on meso and metafemora dark reddish-brown, remainder light reddish-brown, tibiae medium to dark reddish-brown with light reddish-brown band about half distance from base. Abdomen reddish-brown, connexival margins lighter, anterior margins of segments three to seven dark reddish-brown. **VESTITURE**: Moderately setose. Anteocular lobe with short, recumbent to erect setae. Postocular lobe with short, recumbent setae, some long, erect setae on lateral and ventral surfaces. Anterior pronotal lobe with moderately long, recumbent to semi-erect setae, confined to setal tracts dorsally, long erect setae laterally. Posterior pronotal lobe with short, recumbent setae, long erect setae laterally. Scutellum with recumbent to semi-erect setae. Dense recumbent to semi-erect setae over clavus and corium. Abdomen with short, erect setae on dorsum, dense, short, recumbent and moderate to long erect setae over lateral and ventral surface, with exception of bare dark areas. Exposed surface of pygophore with short semi-erect and long erect setae. **STRUCTURE: Head**: Cylindrical, L/W = 2.12. Postocular lobe moderately long; in dorsal view anteriorly gradually narrowing, posterior portion constant, slightly narrower. Eye moderately sized; lateral margin only slightly wider than postocular lobe; dorsal margin attaining postocular transverse groove, ventral margin removed from ventral surface of head in lateral view. *Labium*: I: II: III = 1: 1.7 : 0.6. **Thorax**: Anterolateral angle of collar rounded, collar not well defined medially; medial longitudinal sulcus evident only on posterior 1/2, deepening anterior to transverse sulcus of pronotum. Posterior pronotal lobe with finely rugulose surface; disc slightly elevated above humeral angle; humeral angle armed, with dentate process. Scutellum moderately long; apex angulate, not projected. *Legs*: Rather slender. Metafemur slightly more slender than pro- and mesofemora. *Hemelytron*: Surpassing apex of abdomen by more than length of abdominal segment seven; quadrate cell small; Cu and M of cubital cell converging towards R. **GENITALIA**: (Fig. 180) *Pygophore*: Elongate ovoid; not expanded laterally in dorsal view. Medial process laterally compressed; slender; long; semi-erect; straight; apex in posterior view blunt, without modification. *Paramere*: moderately long, reaching medial process; apical part greatly expanded. *Phallus*: Dorsal phallothecal sclerite elongated; apical portion tapered, slightly convex, lateral margins recurved upward; apex with rounded protuberances, posterior margin of foramen inversely V-shaped. Struts not readily evident. Basal plate arm moderately robust; basally fused; in lateral view nearly straight, very slightly curved; bridge short; extension of basal plate reduced.

***Female***: unknown.

#### Diagnosis

*Zelus spatulosus* has a rather distinctly enlarged apical part of the paramere (Fig. 180a), the extent not compared by any other species of *Zelus*. Also, the slender form of the medial process is distinctive among the *Zelus luridus* species group.

#### Etymology

The specific epithet is from *spatula*, referring to the rather broad apical part of the paramere.

#### Distribution

**Figure 181.**
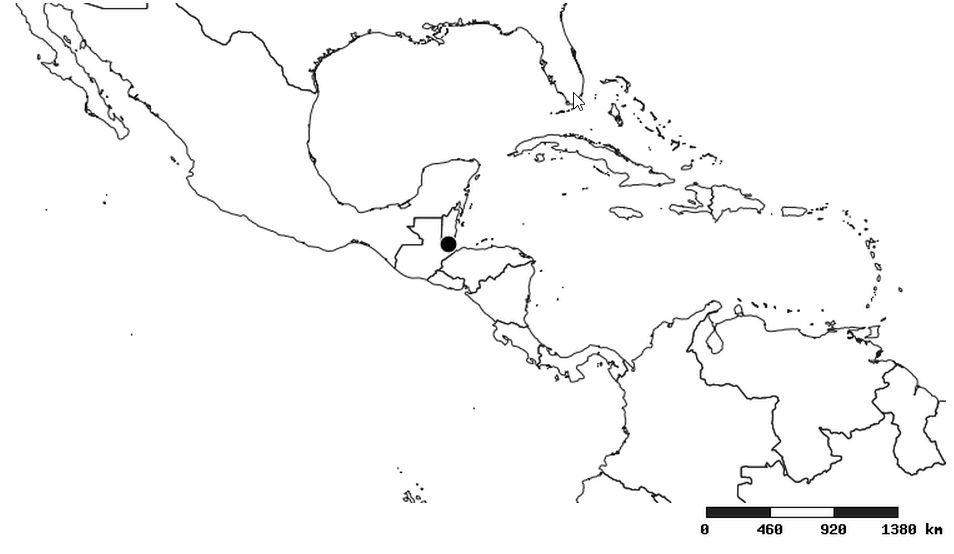
*Zelus spatulosus* Zhang & Hart, sp. n., specimen record map

Central America (Fig. 181). Known only from the type locality in Belize.

#### Taxon discussion

The primary basis for placing *Z. spatulosus* in the *Zelus luridus* group is the expanded paramere (Fig. 3). However, this species shows several characters which depart significantly from the remaining species as described in the following. The medial process is slender and laterally compressed, the struts are not evidently visible, the dorsal phallothecal sclerite is distinctly shaped, with a narrow basal portion and expanded apical part, and the basal plate arms are fused. These characters are so distinct that they are possibly either autapomorphic or homoplasious. As no characters are found to unite *Z. spatulosus* with species of other species group, its placement in the *Zelus luridus* species group appears to be the best decision to take.

### Zelus sphegeus

Fabricius, 1803

Zelus sphegeus Fabricius, 1803, p. 287, orig. descr.; Stål, 1872, p. 91, cat.; Lethierry and Severin, 1896, p. 153, cat.; Haviland, 1931, p. 137, 153, list and note; Wygodzinsky, 1949a, p. 50, checklist; Maldonado, 1990, p. 331, cat.
Diplodus sphegeus , Stål, 1868, p. 109, descr.; Walker, 1873, p. 125, cat.

#### Materials

- **Type status:** Holotype. **Occurrence:** catalogNumber: UCR_ENT 00075108; recordedBy: Dom. Smidt; sex: Adult Female; otherCatalogNumbers: ZMUC 103083; **Taxon:** scientificName: Zelus sphegeus; family: Reduviidae; genus: Zelus; scientificNameAuthorship: Fabricius, 1803; **Location:** country: unknown; stateProvince: unknown; locality: Habitat in America meridionali; **Identification:** identifiedBy: G. Zhang; dateIdentified: 2012; **Event:** eventDate: No date provided; **Record Level:** institutionCode: ZMUC

#### Description

Figs 182, 183

***Male***: unknown.

***Female***: (Fig. 182) Medium-sized, total length 14.18–14.95 mm (mean 14.57 mm, Suppl. material 2); slender. **COLORATION**: Most of dorsal surface brown, anterior pronotal lobe yellowish. **VESTITURE**: Sparsely setose. Head with inconspicuous, short, erect and recumbent setae, more dense dorsally, some slightly larger erect setae ventrally. Anterior pronotal lobe with short, erect and recumbent setae; posterior pronotal lobe with short, erect and recumbent setae. Abdomen with short, erect and recumbent setae. **STRUCTURE: Head**: Cylindrical, L/W = 2.34. Postocular lobe very long; in dorsal view distinctly narrowing through anterior 1/2, posterior 1/2 constant, tube-like. Eye smallish; lateral margin only slightly wider than postocular lobe; dorsal and ventral margins removed from surfaces of head. *Labium*: I: II: III = 1: 2.3: 0.5. **Thorax**: Anterolateral angle rounded, without projection; medial longitudinal sulcus shallow near collar, deepening posteriorly. Posterior pronotal lobe with finely rugulose surface; disc distinctly elevated above humeral angle; humeral angle armed, with spinous processes. Scutellum moderately long; apex angulate. *Legs*: Very slender. *Hemelytron*: Slightly surpassing apex of abdomen, not more than length of abdominal segment seven; quadrate cell moderately sized; Cu and M of cubital cell subparallel.

#### Diagnosis

This species can be recognized by the pronotum bicolorous, anterior lobe yellowish and posterior lobe dark brown; the humeral angle with long spinous process. Similar to females of *Z. truxali*, but the legs are not banded and the anterior pronotal lobe appears to be more humped than that in *Z. truxali*.

#### Distribution

**Figure 183.**
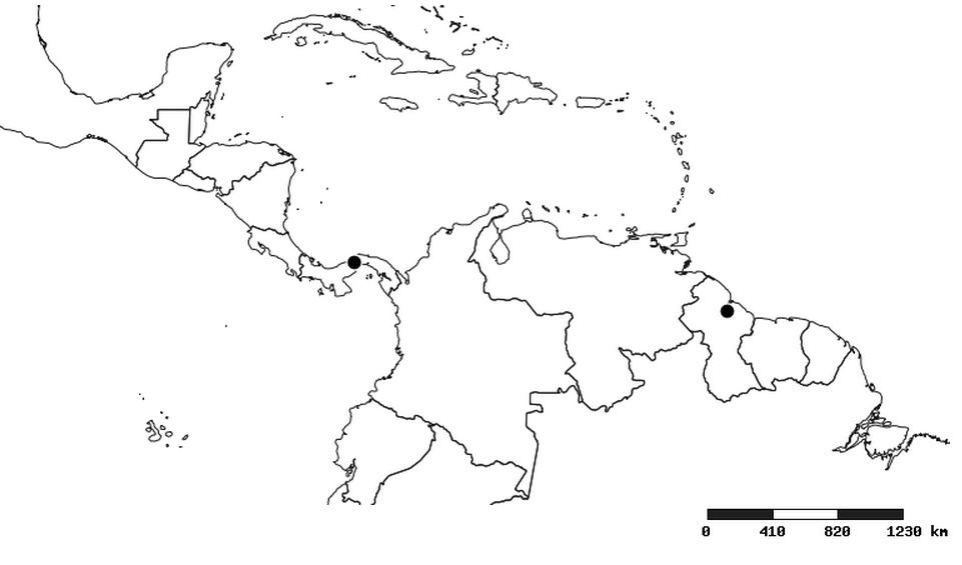
*Zelus sphegeus* Fabricius, 1803, specimen record map

Central America and South America (Fig. 183). Only know from two countries: Guyana and Panama.

### Zelus subimpressus

Stål, 1872

Zelus subimpressus Stål, 1872, p. 91, orig. descr. (subgenus *Diplodus*); Lethierry and Severin, 1896, p. 153, cat.; Fracker, 1913, p. 239, 240, key and list (subgenus *Diplodus*); Fracker and Bruner, 1924, p. 170, note; Bruner 1926, p. 78, 79, key and note; Leonard, 1933, p. 319, note; Wolcott, 1950, p. 212, note; Wygodzinsky, 1949a, p. 50, checklist; Alayo, 1967, p. 37, note; Hart, 1987, p. 294, redescription, note, fig. and key; Maldonado, 1990, p. 331, cat.
Diplodus subimpressus : Uhler, 1886, p. 24, checklist; Gundlach, 1894, p. 598, checklist.

#### Materials

- **Type status:** Holotype. **Occurrence:** occurrenceRemarks: Bears the following label: Cuba / Stal. Subimpressus Stal / Typus; sex: Adult female; **Taxon:** scientificName: Zelus subimpressus; family: Reduviidae; genus: Zelus; scientificNameAuthorship: Stĺl, 1872; **Location:** country: CUBA; **Record Level:** institutionCode: NHRS

#### Description

Figs 184, 185, 186

***Male***: (Fig. 184a, b) Medium-sized, total length 11.59–12.40 mm (mean 12.13 mm, Suppl. material 2); very slender. **COLORATION**: Dorsal surfaces brown, darker on postocular lobe of head; corium and apices of femora reddish. Lateral and ventral surfaces yellowish-brown. **VESTITURE**: Moderately setose. Head with moderately long, recumbent and sparse, erect setae dorsally, less dense and with some longer setae on ventral surface. Anterior pronotal lobe with recumbent setae scattered over entire surface, mainly confined to setal tracts; posterior pronotal lobe with recumbent setae and some erect setae. Abdomen with sparse, erect and recumbent setae. **STRUCTURE: Head**: Elongated, L/W = 2.64. Postocular lobe moderately long; in dorsal view anteriorly gradually narrowing, posterior portion constant, slightly narrower. Eye smallish; lateral margin only slightly wider than postocular lobe; dorsal and ventral margins removed from surfaces of head. *Labium*: I: II: III = 1: 1.8: 0.5. Basiflagellomere diameter larger than that of pedicel. **Thorax**: Anterolateral angle with inconspicuous subtuberculate projection; medial longitudinal sulcus shallow near collar, deepening posteriorly. Posterior pronotal lobe with rugulose surface; disc distinctly elevated above humeral angle; humeral angle armed, with dentate projection. Scutellum long; apex angulate, not projected. *Legs*: Slender, femoral diameters subequal. *Hemelytron*: Very slightly surpassing apex of abdomen, not more than length of abdominal segment seven; quadrate cell small, elongate; Cu and M of cubital cell subparallel. **GENITALIA**: (Fig. 185) *Pygophore*: Elongate; mid-lateral fold adjacent to paramere insertion; not expanded laterally in dorsal view. Medial process robust; short; posteriorly directed; nearly straight; apex in posterior view blunt, slightly folded posteriad. *Paramere*: Cylindrical; short, not reaching apex of medial process; directed posteriad; basally slightly constricted; not distinctly curved; apical part slightly enlarged. *Phallus*: Dorsal phallothecal sclerite shield-shaped; apical portion of phallothecal sclerite not distinctly tapered, flat; apex truncate, slightly emarginate in middle; basal margins expanded, then constricted before base; posterior margin of foramen deeply concave. Struts attached to dorsal phallothecal sclerite; apically separate, connected by bridge; basally mostly separate, moderately fused. Basal plate arm robust; separate; diverging; in lateral view nearly straight, very slightly curved; bridge long; extension of basal plate small and confined to apex of basal plate arm.

***Female***: (Fig. 184c, d) Similar to male, except for the following. Larger than male, total length 14.36–16.08 mm (mean 15.06 mm, Suppl. material 2).

#### Diagnosis

The rather slender body form of *Z. subimpressus* is characteristic of the *Zelus puertoricensis* species group (total length/width more than 8x). Both sexes are readily distinguished from *Z. puertoricensis* by having a sloping dorsal surface on the postocular lobe. Males can be recognized by the robust, posteriorly directed medial process, apex bent and the short, cylindrical paramere. This is larger in *Z. subimpressus* than in *Z. puertoricensis* (Fig. 6).

#### Distribution

**Figure 186.**
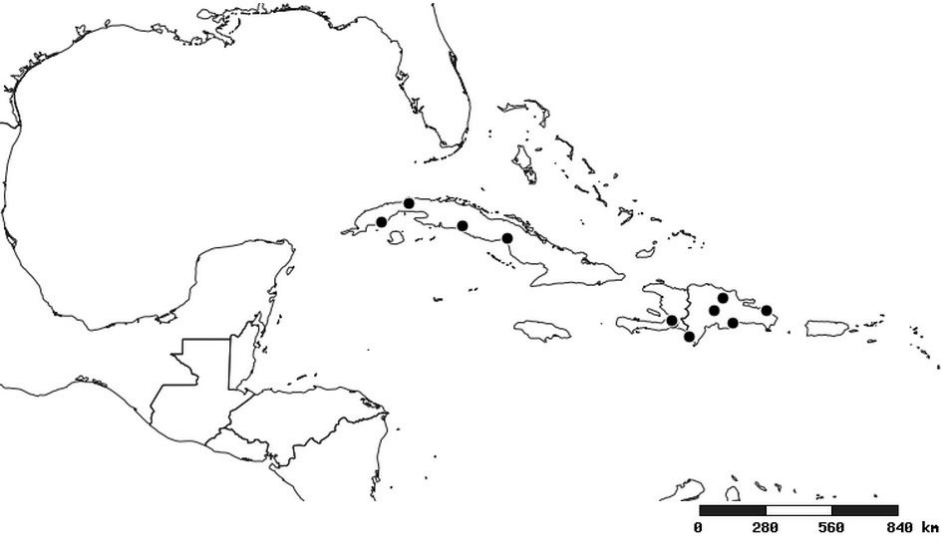
*Zelus subimpressus* Stål, 1872, specimen record map

The Caribbean, the islands of Cuba and Hispaniola (Fig. 186). Countries with records: Cuba, Dominican Republic and Haiti.

### Zelus sulcicollis

Champion, 1899

Zelus sulcicollis Champion, 1898, p. 258–259, Tab. XV. fig. 21, orig. descr. and fig.; Fracker, 1913, p. 239, 240, key and list (subgenus *Diplodus*); Wygodzinsky, 1949a, p. 50, checklist; Maldonado, 1990, p. 331, cat.

#### Materials

- **Type status:** Lectotype. **Occurrence:** catalogNumber: UCR_ENT 00048760; occurrenceRemarks: Lectotype of *Zelus sulcicollis* Champion, 1899 (**New Designation** by Zhang, Hart & Weirauch, 2016) Verbatim label info: Type / B.C.A.Rhyn.II. *Zelus sulcicollis* Ch. / Sp. figured. / S. Geronimo, Guatemala. Champion. / Lectotype *Zelus sulcicollis* Champion des. by. E.R. Hart; recordedBy: G.C. Champion; sex: Adult Female; **Taxon:** scientificName: Zelus sulcicollis; family: Reduviidae; genus: Zelus; scientificNameAuthorship: Champion, 1899; **Location:** country: GUATEMALA; stateProvince: Baja Verapaz; locality: San Geronimo; decimalLatitude: 15.13333; decimalLongitude: -90.18333; georeferenceSources: Publication; **Identification:** identifiedBy: G. Zhang; dateIdentified: 2012; **Event:** eventDate: No date provided; **Record Level:** institutionCode: BMNH
- **Type status:** Allolectotype. **Occurrence:** occurrenceRemarks: Allolectotype of *Zelus sulcicollis* Champion, 1899 (**New Designation** by Zhang, Hart & Weirauch, 2016) Verbatim label info: S. Geronimo, Guatemala. Champion. / B.C.A.Rhyn.II. *Zelus sulcicollis* Ch. / Allolectotype Zelus sulcicollis Champion des. by. E.R. Hart; recordedBy: G.C. Champion; sex: Adult Male; **Taxon:** scientificName: Zelus sulcicollis; family: Reduviidae; genus: Zelus; scientificNameAuthorship: Champion, 1899; **Location:** country: GUATEMALA; stateProvince: Baja Verapaz; locality: San Geronimo; decimalLatitude: 15.13333; decimalLongitude: -90.18333; georeferenceSources: Publication; **Identification:** identifiedBy: E.R. Hart; dateIdentified: 1972; **Event:** eventDate: No date provided; **Record Level:** institutionCode: BMNH
- **Type status:** Paralectotype. **Occurrence:** occurrenceRemarks: Paralectotype of Zelus sulcicollis Champion, 1899 (**New Designation** by Zhang, Hart & Weirauch, 2016) Verbatim label info: l. Tepic, Mex., July, Schumann / B.C.A.Rhyn.II. *Zelus sulcicollis* Ch., Omilteme, Guerrero, 8000 ft., July, H. H. Smith / B.C.A. Rhyn. II. *Zelus sulcicollis* Ch. / Paralectotype Zelus sulcicollis Champion des. by. E.R. Hart; recordedBy: H.H. Smith; sex: Adult Female; **Taxon:** scientificName: Zelus sulcicollis; family: Reduviidae; genus: Zelus; scientificNameAuthorship: Champion, 1899; **Location:** country: MEXICO; stateProvince: Guerrero; locality: l. Tepic; georeferenceSources: Publication; **Identification:** identifiedBy: E.R. Hart; dateIdentified: 1972; **Event:** eventDate: No date provided; **Record Level:** institutionCode: BMNH
- **Type status:** Paralectotype. **Occurrence:** occurrenceRemarks: Paralectotype of *Zelus sulcicollis* Champion, 1899 (**New Designation** by Zhang, Hart & Weirauch, 2016) Verbatim label info: l. Tepic, Mex., July, Schumann / B.C.A.Rhyn.II. *Zelus sulcicollis* Ch., Omilteme, Guerrero, 8000 ft., July, H. H. Smith / B.C.A. Rhyn. II. *Zelus sulcicollis* Ch. / Paralectotype *Zelus sulcicollis* Champion des. by. E.R. Hart; recordedBy: G.C. Champion; sex: Adult Male; **Taxon:** scientificName: Zelus sulcicollis; family: Reduviidae; genus: Zelus; scientificNameAuthorship: Champion, 1899; **Location:** country: MEXICO; stateProvince: Guerrero; locality: l. Tepic; georeferenceSources: Publication; **Identification:** identifiedBy: E.R. Hart; dateIdentified: 1972; **Event:** eventDate: No date provided; **Record Level:** institutionCode: BMNH

#### Description

Figs 187, 188, 189

***Male***: (Fig. 187a, b) Large, total length 14.86–20.26 mm (mean 18.10 mm, Suppl. material 2); robust. **COLORATION**: Brown to reddish; uniform. Entire surface quite uniformly brown, appearing reddish in some specimens. Legs usually uniform, not distinctly banded; femora with faint dark markings in some specimens. Connexivum banded. **VESTITURE**: Densely setose. Similar to that in *Z. armillatus*; dorsal setation more spine-like. **STRUCTURE: Head**: Cylindrical, L/W = 2.47. Postocular lobe moderately long; in dorsal view anteriorly gradually narrowing, posterior portion constant, slightly narrower. Eye smallish; lateral margin only slightly wider than postocular lobe; dorsal margin attaining postocular transverse groove, ventral margin removed from ventral surface of head in lateral view. *Labium*: I: II: III = 1: 1.4: 0.3. Basiflagellomere diameter subequal to that of pedicel. **Thorax**: Anterolateral angle bearing small protuberance; medial longitudinal sulcus distinct throughout. Posterior pronotal lobe with rugulose surface; disc distinctly elevated above humeral angle; humeral angle armed, with spinous processes. Scutellum moderately long; apex angulate. *Legs*: Moderately robust. *Hemelytron*: Slightly surpassing apex of abdomen, not more than length of abdominal segment seven; quadrate cell large and broad; Cu and M of cubital cell converging towards R. **GENITALIA**: (Fig. 188) *Pygophore*: Rounded; slightly expanded laterally near base of paramere in dorsal view; posteriorly expanded sac-like sclerite between parameres and medial process. Medial process cylindrical; slender; moderately long; posteriorly directed; straight; apex in posterior view rounded, with very inconspicuous lateral prongs. *Paramere*: Cylindrical; moderately long, not reaching apex of medial process; directed posteriad; slightly curved dorsad; apical part very slightly enlarged. *Phallus*: Dorsal phallothecal sclerite somewhat squarish; lateral expansion arising close to base; apical portion of phallothecal sclerite not distinctly tapered, flat, lateral margin narrowly angulate; apex rounded; posterior margin of foramen broadly concave, medially deeper. Struts attached to dorsal phallothecal sclerite; apically fused; basally mostly separate, moderately fused. Basal plate arm moderately robust; separate; diverging; in lateral view very slightly curved; bridge short; extension of basal plate small, marginally expanded onto arm.

***Female***: (Fig. 187c, d) Similar to male, except for the following. Larger than male, total length 20.59–21.91 mm (mean 21.41 mm, Suppl. material 2).

#### Diagnosis

Recognized by the dorsal coloration nearly uniformly brown, somewhat reddish and the posterior pronotal lobe medially depressed. Among males of the *Zelus armillatus* species group occurring in the same geographic range, the medial process and paramere of *Z. sulcicollis* are longer than that in *Z. litigiosus, Z. ruficeps* and *Z. janus*.

#### Distribution

**Figure 189.**
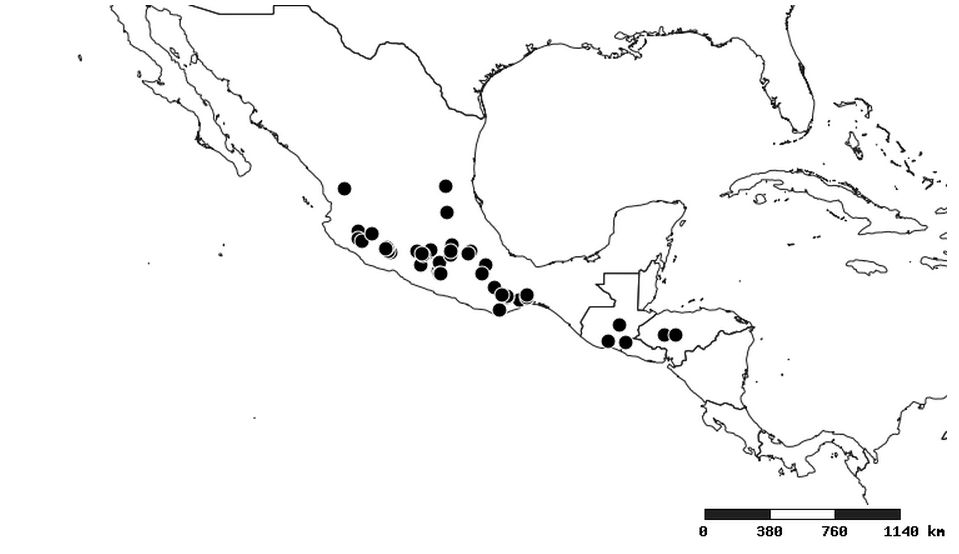
*Zelus sulcicollis* Champion, 1899, specimen record map

Southern Mexico and Northern Central America (Fig. 189). Countries with records: Guatemala, Honduras and Mexico.

### Zelus tetracanthus

Stål, 1862

Zelus tetracanthus Stål , 1862, p. 454, orig. descr. (subgenus *Pindus*) ; Stål , 1872, p. 92, cat. (subgenus *Pindus*); Lethierry and Severin, 1896, p. 153, cat.; Champion, 1898, p. 362, Tab. XV, fig. 27, note and fig.; Fracker, 1913, p. 240, key and list (subgenus *Pindus*); Wygodzinsky, 1949a, p. 50, checklist; Hart, 1986, p. 536-537, redescription, note, fig. and key; Maldonado, 1990, p. 331, cat.
Diplodus tetracanthus : Walker, 1873, p. 124, cat.; Uhler, 1886, p. 24, checklist.
Pindus socius Uhler, 1872b, p. 420, orig. descr.; Uhler, 186, p. 62, list (reprint); Uhler, 1877, p. 1329, list; Uhler, 1894, p. 284, list.; Hart, 1986, p. 536, junior syn. of *Z. tetracanthus*.
Diplodus socius : Uhler, 1886, p. 24, checklist; Gillette and Baker, 1895, p. 60, list.
Zelus socius : Lethierry and Severin, 1896, p. 153 cat.; Barber, 1906, p. 28,6, list; Hart, 1907, p. 237, list; Torre-Bueno, 1908, p. 235, list (subgenus *Pindus*); Van Duzee, 1909, p. 177, list; Banks, 1910b, p. 16, cat.; Fracker, 1913, p. 240, key and list (subgenus *Pindus*); Torre-Bueno, 1913, p. 60, list (subgenus *Pindus*); Barber, 1914, p. 506, list; Parshley, 1914, p. 144, list; Van duzee, 1916, p. 30, checklist (subgenus *Pindus*); Van Duzee, 1917, p. 261, cat. (subgenus *Pindus*); Parshley, 1921, p. 5, list; Anonymous, 1923, p. 120-135, note; Blatchley, 1926, p. 569, 571, key and descr. (subgenus *Pindus*); Readio, 1926, p. 168, 177, note and fig.; Readio, 1927, p. 169, 179-181, P1. XIV. fig. 1,2,5, descr., notes and fig.; Leonard, 1928, p. 105, list; Essig, 1929, p. 357, note; Knowlton, 1932, p. 12, note; Harris, 1937, p. 174, list; Brimley, 1938, p. 73, list; Procter, 1946, p. 319, list; Wygodzinsky, 1949a, p. 50, checklist; Elkins, 1951, p. 410, list; Sibley, 1951, p. 92, list; Elkins, 1954, p. 47, note; Atkins, et. al., 1957, p. 251-259, note; Drew and Schaeffer, 1962, p. 106, list; Wene and Sheets, 1962, p. 395-398, note; Whitcomb and Bell, 1964, p. 22, list; Butler, 1966, p. 1306-1307, note; Nutting and Spangler, 1969, p. 763-769, note and photo.
Diplocodus socius : Van Duzee, 1914, p. 13, list and note.
Zelus audax Banks, 1910, p. 325, orig. descr.; Fracker, 1913, p. 240, note; Van Duzee, 1916, p. 30 checklist (subgenus *Pindus*); Van Duzee, 1917, p. 261, cat. (subgenus *Pindus*); Britton, 1923, p. 687, list (subgenus *Pindus*); Blatchley, 1926, p. 569, 571-572, key and descr. (subgenus *Pindus*); Readio, 1927, p. 169, 181, descr.; Leonard, 1928, p. 105, list; Wygodzinsky, 1949a, p. 48, checklist; Hart, 1986, p. 536, junior syn. of *Z. tetracanthus* and lectotype desig.
Zelus occiduus Torre-Bueno, 1913a, p. 22, orig. descr. (subgenus *Pindus*); Van Duzee, 1916, p. 30, checklist; Van Duzee, 1917, p. 261, cat. (subgenus *Pindus*) ; Readio, 1927, p. 169, 181-182, key and descr.; Wygodzinsky, 1949a, p. 50, checklist; Elkins, 1951, p. 410, list; Hart, 1986, p. 536, junior syn. of *Z. tetracanthus* and lectotype desig.
Zelus angustatus Hussey, 1925, p. 66-67, orig. descr.; Blatchley, 1926, p. 569, 572, descr. and note (subgenus *Pindus*); Readio, 1927, p. 169, 182., key and descr.; Blatchley, 1928, p. 6, note (subgenus *Pindus*); Wygodzinsky, 1949a, p. 48, checklist; Elkins, 1951, p. 410, list; Hussey, 1953, p. 9-11, note; Hart, 1986, p. 536, junior syn. of *Z. tetracanthus*.

#### Materials

- **Type status:** Holotype. **Occurrence:** catalogNumber: UCR_ENT 00075078; occurrenceRemarks: Verbatim label info: Mexico Coll. Signoret / Tetracanth. det. Stal / B.C.A. Rhyn. II. *Zelus tetracanthus* St. / Holotype / *Zelus tetracanthus* Stal / Lectotypus *Zelus tetracanthus* STAL, 1862 etik. Hecher 1996 REDV. 488/1; recordedBy: Signoret; sex: Adult Male; **Taxon:** scientificName: Zelus tetracanthus; family: Reduviidae; genus: Zelus; scientificNameAuthorship: Stål, 1862; **Location:** country: MEXICO; stateProvince: None or Unknown; locality: unknown; **Identification:** identifiedBy: G. Zhang; dateIdentified: 2012; **Event:** eventDate: No date provided; **Record Level:** institutionCode: NHMW
- **Type status:** Other material. **Occurrence:** catalogNumber: UCR_ENT 00057796; occurrenceRemarks: **Lectotype** of *Zelus audax* Banks, 1910, designated by Hart (1986), junior synonym of Zelus tetracanthus Stal; recordedBy: N. Banks; sex: Adult Female; **Taxon:** scientificName: Zelus tetracanthus; family: Reduviidae; genus: Zelus; scientificNameAuthorship: Stål, 1862; **Location:** country: USA; stateProvince: New York; county: Nassau; locality: Sea Cliff, Long Island; verbatimElevation: 5 m; decimalLatitude: 40.84944; decimalLongitude: -73.65233; georeferenceSources: Google Earth; **Identification:** identifiedBy: G. Zhang; dateIdentified: 2012; **Event:** eventDate: no date provided; **Record Level:** institutionCode: AMNH
- **Type status:** Other material. **Occurrence:** catalogNumber: UCR_ENT 00057797; occurrenceRemarks: **Lectotype** of *Zelus occiduus* Torre-Bueno, designated by Hart (1986), junior synonym of *Zelus tetracanthus* Stal. Additional label information: M.C.Z Paratype 27979, Zelus (Pindus) occiduus 776 Bueno, LECTOTYPE *Zelus occiduus* Torre-Bueno des. by E. R. Hart; recordedBy: J.M.A.; sex: Adult Female; **Taxon:** scientificName: Zelus tetracanthus; family: Reduviidae; genus: Zelus; scientificNameAuthorship: Stål, 1862; **Location:** country: USA; stateProvince: California; county: Santa Clara; locality: Salt Marshes, Palo Alto; decimalLatitude: 37.44188; decimalLongitude: -122.14302; georeferenceSources: GeoLocate Software; **Identification:** identifiedBy: G. Zhang; dateIdentified: 2012; **Event:** eventDate: 1906-04-20; **Record Level:** institutionCode: AMNH
- **Type status:** Other material. **Occurrence:** catalogNumber: UCR_ENT 00017636; occurrenceRemarks: **Paralectotype** of *Zelus audax* Banks, 1911, designated by Hart 1986, junior synonym of *Zelus tetracanthus* Stal; recordedBy: N. Banks; sex: Adult Male; **Taxon:** scientificName: Zelus tetracanthus; family: Reduviidae; genus: Zelus; scientificNameAuthorship: Stål, 1862; **Location:** country: USA; stateProvince: New York; county: Nassau; locality: Sea Cliff, Long Island; verbatimElevation: 5 m; decimalLatitude: 40.84944; decimalLongitude: -73.65233; georeferenceSources: Google Earth; **Identification:** identifiedBy: G. Zhang; dateIdentified: 2012; **Event:** eventDate: no date provided; **Record Level:** institutionCode: AMNH
- **Type status:** Other material. **Occurrence:** catalogNumber: UCR_ENT 00046742; occurrenceRemarks: **Paralectotype** of *Zelus audax* Banks, 1911, designated by Hart 1986, junior synonym of *Zelus tetracanthus* Stal; recordedBy: N. Banks; sex: Adult Female; **Taxon:** scientificName: Zelus tetracanthus; family: Reduviidae; genus: Zelus; scientificNameAuthorship: Stål, 1862; **Location:** country: USA; stateProvince: Virginia; county: Falls Church; locality: Falls Church; decimalLatitude: 38.88222; decimalLongitude: -77.17138; **Identification:** identifiedBy: G. Zhang; dateIdentified: 2012; **Event:** eventDate: 1900-08-29; **Record Level:** institutionCode: AMNH
- **Type status:** Other material. **Occurrence:** occurrenceRemarks: **Holotype** o*f Zelus angustatus* Hussey, 1925, junior synonym of *Zelus tetracanthus* Stal. Type specimen information extracted from literature. Location of actual specimen could not be determined (Hart, 1986); recordedBy: T.H. Hubbell; sex: Adult Male; **Taxon:** scientificName: Zelus tetracanthus; family: Reduviidae; genus: Zelus; scientificNameAuthorship: Stål, 1862; **Location:** country: USA; stateProvince: Florida; locality: Gainesville; **Identification:** identifiedBy: G. Zhang; dateIdentified: 2012; **Event:** eventDate: 1923-12; **Record Level:** institutionCode: Unknown

#### Description

Figs 190, 191, 192

***Male***: (Fig. 190a) Medium-sized, total length 11.34–13.73 mm (mean 12.36 mm, Suppl. material 2); slender. **COLORATION**: Greyish-brown to black. Anteocular lobe dark reddish-brown to brownish-black, variable yellowish-brown areas usually present dorsolaterally anterior to compound eyes, dorsally and medially at antennal bases, and on lateral and ventral surfaces. Postocular lobe dark reddish-brown with lighter areas between ocelli and compound eyes, along mid-dorsal line, longitudinally on lateral and ventral surface. Labium reddish-brown to brownish-black with anterior surface of segment I lighter in color, yellowish-brown to reddish-brown. Antennal segments I and II reddish-brown, outer dorsolateral surface of I darker, especially near base, base and apex of II darker, segments III and IV yellowish-brown to reddish-brown. Anterior pronotal lobe reddish-brown to brownish-black, occasionally with variable lighter areas, especially near margins, lateral surface reddish-brown to brownish-black, usually lighter ventrally. Posterior pronotal lobe reddish-brown to brownish-black with dark yellowish-brown posterior margin and/or darker reddish-brown to brownish-black apices on lateral and dorsal processes. Scutellum variably yellowish-brown to brownish-black, usually with lighter apex. Legs yellowish-brown to reddish-brown, usually with variable darker reddish-brown spots or bands, especially near dorsal surfaces of femoral apices. Hemelytron brown to dark brown, veins in area anterior to basal and discal cells lighter. Abdomen variable, dark yellowish-brown to reddish-brown, darker areas usually at posterior of segments of connexivum and mid-laterally at anterior of segments 3–7. Abdomen yellowish-brown with variable reddish-brown areas on lateral and posterior margins and posterior surface. **VESTITURE**: Moderately setose. Short recumbent and short to long erect setae, many specimens covered with white waxlike exudation. Anteocular lobe with short recumbent setae on entire surface, longer erect setae on tylus and ventral surface; postocular lobe with short recumbent setae over entire surface, moderate to long erect setae on posterodorsal and ventral surfaces. Entire surface of anterior pronotal lobe with short recumbent and erect setae, confined to setal tracts dorsally, longer erect setae laterally; posterior lobe with short recumbent setae over surface, some long erect setae laterally; scutellum setae short, recumbent to semi-erect. Corium and clavus with short, recumbent setae. Abdominal dorsum with sparse short erect setae, remainder of surface with short recument and sparse moderate erect setae. Exposed surface of pygophore with short to long semi-erect and erect setae, especially posteriorly. **STRUCTURE: Head**: Elongated, L/W = 2.63. Postocular lobe long; in dorsal view anteriorly gradually narrowing, posterior portion constant, slightly narrower. Eye moderately sized; lateral margin only slightly wider than margin of postocular lobe; dorsal and ventral margins removed from surfaces of head. *Labium*: I: II: III = 1: 2.3: 0.4. Basiflagellomere diameter larger than that of pedicel. **Thorax**: Anterolateral angle rounded, without projection; medial longitudinal sulcus shallow near collar, deepening posteriorly. Posterior pronotal lobe with finely rugulose surface; disc slightly elevated above humeral angle; humeral angle armed, with short tuberculate processes. Scutellum moderately long; apex angulate, very slightly projected upward. *Legs*: Slender. *Hemelytron*: Surpassing apex of abdomen by about length of abdominal segment seven; quadrate cell small and slender; Cu and M of cubital cell subparallel. **GENITALIA**: (Fig. 191) *Pygophore*: Ovoid; not expanded laterally in dorsal view. Medial process broadly triangular; very short; semi-erect; straight; apex in posterior view blunt, without modification. *Paramere*: Cylindrical; short, not reaching apex of medial process; directed posteriad; nearly straight; apical part enlarged. *Phallus*: Dorsal phallothecal sclerite somewhat ovoid; apical portion of phallothecal sclerite not distinctly tapered, slightly convex; apex medially notched, degree and shape variable; posterior margin of foramen nearly straight to broadly concave. Struts attached to dorsal phallothecal sclerite; apically separate, connected by bridge; basally fused. Basal plate arm slender; separate; subparallel; in lateral view very slightly curved; bridge long; extension of basal plate small and confined to apex of basal plate arm.

***Female***: (Fig. 190b, c) Similar to male, except for the following. Larger than male, total length 13.22–15.63 mm (mean 14.43 mm, Suppl. material 2). Often darker than male. Tuberculate processes of posterior pronotal lobe usually more pronounced, apex often produced. Mid femur slightly swollen on central 1/4, pro- and mesofemoral diameters subequal, about 1.4–1.5x diameter of hind femur. Hemelytron attaining apex of abdomen.

#### Diagnosis

Recognized by the disc of the posterior pronotal lobe with large conspicuous tubercles and the greyish-black coloration. Males can also be recognized by the medial process broadly triangular; the paramere not exceeding medial process; and the dorsal phallothecal sclerite apically with deep emargination.

#### Distribution

**Figure 192.**
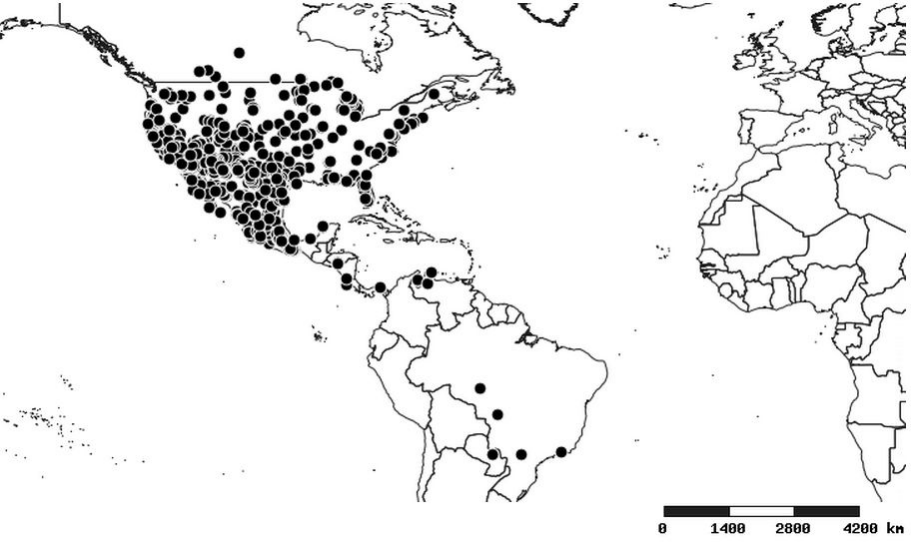
*Zelus tetracanthus* Stål, 1862, specimen record map

North America, Central America and parts of South America (Fig. 192). Countries with records: Brazil, Costa Rica, Curaçao, Honduras, Mexico, Panama, Paraguay, USA and Venezuela.

#### Taxon discussion

Besides *Z. tetracanthus*, two other species, *Z. minutus* and *Z. lewisi* also exhibit tuberculate processes on posterior pronotal lobe, but these species can be easily separated on the basis of coloration and body shape. *Zelus tetracanthus* is a rather widely distributed species, found nearly throughout North America, ranging from southern Canada to parts of Central America. A few records are from Brazil and Paraguay. As the collecting events of these specimens are unrelated, it is highly unlikely these specimens are mislabelled (unless they were each independently mislabelled, which is not likely). Further investigations should examine if the South Amerian populations are native or introduced. Hart (1986) discussed intraspecific variations in *Z. tetracanthus*.

### Zelus truxali

Zhang & Hart sp. n.

urn:lsid:zoobank.org:act:9995B161-BEDF-49B2-9FF3-32DA03333E1E

#### Materials

- **Type status:** Holotype. **Occurrence:** catalogNumber: UCR_ENT 00022668; recordedBy: F. S. Truxal; sex: Adult Male; otherCatalogNumbers: LACM ENT 160234; **Taxon:** scientificName: Zelus truxali; family: Reduviidae; genus: Zelus; scientificNameAuthorship: Zhang and Hart, 2016; **Location:** country: PERU; stateProvince: Pasco; locality: Chontilla 22 km. SE of Iscozazin; decimalLatitude: -10.3357; decimalLongitude: -75.11004; georeferenceSources: Google Earth; **Identification:** identifiedBy: G. Zhang; dateIdentified: 2013; **Event:** eventDate: 1961-07-20; **Record Level:** institutionCode: LACM
- **Type status:** Paratype. **Occurrence:** catalogNumber: UCR_ENT 00046974; recordedBy: Wygodzinsky and Monros; sex: Adult Male; **Taxon:** scientificName: Zelus truxali; family: Reduviidae; genus: Zelus; scientificNameAuthorship: Zhang and Hart, 2016; **Location:** country: BOLIVIA; stateProvince: Cochabamba; locality: Villa Tunari, Chapare; verbatimElevation: 500 m; decimalLatitude: -16.91666; decimalLongitude: -65.36667; **Identification:** identifiedBy: G. Zhang; dateIdentified: 2013; **Event:** eventDate: 1958-01-09; **Record Level:** institutionCode: AMNH
- **Type status:** Paratype. **Occurrence:** catalogNumber: UCR_ENT 00047331; recordedBy: Wygodzinsky and Monros; sex: Adult Female; **Taxon:** scientificName: Zelus truxali; family: Reduviidae; genus: Zelus; scientificNameAuthorship: Zhang and Hart, 2016; **Location:** country: BOLIVIA; stateProvince: Cochabamba; locality: Villa Tunari, Chapare; verbatimElevation: 500 m; decimalLatitude: -16.91666; decimalLongitude: -65.36667; **Identification:** identifiedBy: G. Zhang; dateIdentified: 2013; **Event:** eventDate: 1958-01-09; **Record Level:** institutionCode: AMNH
- **Type status:** Paratype. **Occurrence:** catalogNumber: UCR_ENT 00046896; recordedBy: F.R. Barbosa; sex: Adult Male; **Taxon:** scientificName: Zelus truxali; family: Reduviidae; genus: Zelus; scientificNameAuthorship: Zhang and Hart, 2016; **Location:** country: BRAZIL; stateProvince: Para; locality: Jacareacanga; verbatimElevation: 88 m; decimalLatitude: -6.2667; decimalLongitude: -57.65; georeferenceSources: Gazetteer; **Identification:** identifiedBy: G. Zhang; dateIdentified: 2013; **Event:** eventDate: 1969-10-01; **Record Level:** institutionCode: AMNH
- **Type status:** Paratype. **Occurrence:** catalogNumber: UCR_ENT 00009317; occurrenceRemarks: Additional Labels C.J. Drake Fund Accession 1997; recordedBy: J. E. Eger; sex: Adult Male; **Taxon:** scientificName: Zelus truxali; family: Reduviidae; genus: Zelus; scientificNameAuthorship: Zhang and Hart, 2016; **Location:** country: BRAZIL; stateProvince: Rondonia; locality: 62 km SW Ariquemes, near Fzda. Rancho Grande; decimalLatitude: -10.3081; decimalLongitude: -63.44383; georeferenceSources: Google Earth; **Identification:** identifiedBy: G. Zhang; dateIdentified: 2013; **Event:** eventDate: 1996-12-03 to 1996-12-15; **Record Level:** institutionCode: USNM
- **Type status:** Paratype. **Occurrence:** catalogNumber: UCR_ENT 00015109; recordedBy: Dodge Engleman; sex: Adult Male; **Taxon:** scientificName: Zelus truxali; family: Reduviidae; genus: Zelus; scientificNameAuthorship: Zhang and Hart, 2016; **Location:** country: ECUADOR; stateProvince: Sucumbios; locality: Limoncocha; verbatimElevation: 274 m; decimalLatitude: -0.43333; decimalLongitude: -76.63333; georeferenceSources: Label; **Identification:** identifiedBy: G. Zhang; dateIdentified: 2013; **Event:** eventDate: 1974-03-23 to 1974-03-31; **Record Level:** institutionCode: AMNH
- **Type status:** Paratype. **Occurrence:** catalogNumber: UCR_ENT 00010797; recordedBy: F. S. Truxal; sex: Adult Male; **Taxon:** scientificName: Zelus truxali; family: Reduviidae; genus: Zelus; scientificNameAuthorship: Zhang and Hart, 2016; **Location:** country: PERU; stateProvince: Pasco; locality: Chontilla 22 km. SE of Iscozazin; decimalLatitude: -10.3357; decimalLongitude: -75.11004; georeferenceSources: Google Earth; **Identification:** identifiedBy: G. Zhang; dateIdentified: 2013; **Event:** eventDate: 1961-07-20; **Record Level:** institutionCode: LACM
- **Type status:** Paratype. **Occurrence:** catalogNumber: UCR_ENT 00010798; recordedBy: F. S. Truxal; sex: Adult Female; **Taxon:** scientificName: Zelus truxali; family: Reduviidae; genus: Zelus; scientificNameAuthorship: Zhang and Hart, 2016; **Location:** country: PERU; stateProvince: Pasco; locality: Chontilla 22 km. SE of Iscozazin; decimalLatitude: -10.3357; decimalLongitude: -75.11004; georeferenceSources: Google Earth; **Identification:** identifiedBy: G. Zhang; dateIdentified: 2013; **Event:** eventDate: 1961-07-20; **Record Level:** institutionCode: LACM
- **Type status:** Paratype. **Occurrence:** catalogNumber: UCR_ENT 00010799; recordedBy: F. S. Truxal; sex: Adult Female; **Taxon:** scientificName: Zelus truxali; family: Reduviidae; genus: Zelus; scientificNameAuthorship: Zhang and Hart, 2016; **Location:** country: PERU; stateProvince: Pasco; locality: Chontilla 22 km. SE of Iscozazin; decimalLatitude: -10.3357; decimalLongitude: -75.11004; georeferenceSources: Google Earth; **Identification:** identifiedBy: G. Zhang; dateIdentified: 2013; **Event:** eventDate: 1961-07-20; **Record Level:** institutionCode: LACM
- **Type status:** Paratype. **Occurrence:** catalogNumber: UCR_ENT 00010800; recordedBy: F. S. Truxal; sex: Adult Female; **Taxon:** scientificName: Zelus truxali; family: Reduviidae; genus: Zelus; scientificNameAuthorship: Zhang and Hart, 2016; **Location:** country: PERU; stateProvince: Pasco; locality: Chontilla 22 km. SE of Iscozazin; decimalLatitude: -10.3357; decimalLongitude: -75.11004; georeferenceSources: Google Earth; **Identification:** identifiedBy: G. Zhang; dateIdentified: 2013; **Event:** eventDate: 1961-07-20; **Record Level:** institutionCode: LACM
- **Type status:** Paratype. **Occurrence:** catalogNumber: UCR_ENT 00010801; recordedBy: F. S. Truxal; sex: Adult Female; **Taxon:** scientificName: Zelus truxali; family: Reduviidae; genus: Zelus; scientificNameAuthorship: Zhang and Hart, 2016; **Location:** country: PERU; stateProvince: Pasco; locality: Chontilla 22 km. SE of Iscozazin; decimalLatitude: -10.3357; decimalLongitude: -75.11004; georeferenceSources: Google Earth; **Identification:** identifiedBy: G. Zhang; dateIdentified: 2013; **Event:** eventDate: 1961-07-20; **Record Level:** institutionCode: LACM
- **Type status:** Paratype. **Occurrence:** catalogNumber: UCR_ENT 00022671; occurrenceRemarks: Previously designated as 'allotype' of his manuscript name *Zelus truxali* by Hart, a type status not used in the formal publication of this name (Zhang et al., 2016).; recordedBy: F. S. Truxal; sex: Adult Female; otherCatalogNumbers: LACM ENT 160235; **Taxon:** scientificName: Zelus truxali; family: Reduviidae; genus: Zelus; scientificNameAuthorship: Zhang and Hart, 2016; **Location:** country: PERU; stateProvince: Pasco; locality: Chontilla 22 km. SE of Iscozazin; decimalLatitude: -10.3357; decimalLongitude: -75.11004; georeferenceSources: Google Earth; **Identification:** identifiedBy: G. Zhang; dateIdentified: 2013; **Event:** eventDate: 1961-07-20; **Record Level:** institutionCode: LACM

#### Description

Figs 193, 194, 195

***Male***: (Fig. 193a, b) Medium-sized, total length 11.59–12.25 mm (mean 11.91 mm, Suppl. material 2); slender. **COLORATION**: Two major patterns recognized, one of predominantly brown to orangish-brown, the other of dark brown to reddish-brown. In former, posterior lobe lighter colored than anterior lobe, orangish-brown; meso- and meta-femora with two minor bands. In latter, posterior pronotal lobe same color as anterior lobe, or only slightly lighter, somewhat reddish; legs not banded. In both patterns, medial longitudinal lighter colored stripe on postocular lobe. **VESTITURE**: Sparsely setose. Short, recumbent setae on entire surface of head; very short, erect, spine-like setae on dorsum, denser on anterior lobe; few moderately long, erect, fine setae on ventral surface. Pronotum with sparse, recumbent setae and short, erect setae over dorsal surface; denser, recumbent setae on lateral surface and pleura, intermixed with short, erect setae; scutellum with sparse, semi-erect and recumbent setae. Legs with sparse setation on femora and moderately dense setation on tibiae. Corium and clavus with mix of sparse, short, recumbent and erect setae. Abdomen with moderately dense, short recumbent setae, intermixed with sparse, short to long, erect setae. **STRUCTURE: Head**: Cylindrical, L/W = 2.30. Postocular lobe long; in dorsal view distinctly narrowing through anterior 2/3, posterior 1/3 constant, tube-like. Eye moderately sized; lateral margin much wider than postocular lobe; dorsal margin attaining postocular transverse groove, ventral margin removed from ventral surface of head in lateral view. *Labium*: I: II: III = 1: 1.9: 0.4. Basiflagellomere diameter larger than that of pedicel. **Thorax**: Anterolateral angle bearing small, somewhat acute projection; medial longitudinal sulcus evident throughout, deepening posteriorly. Posterior pronotal lobe with rugulose surface; disc distinctly elevated above humeral angle; humeral angle armed, with dentate or spinous process. Scutellum moderately long; apex angulate, slightly projected upward in some specimens. *Legs*: Slender. *Hemelytron*: Slightly surpassing apex of abdomen, not more than length of abdominal segment seven; quadrate cell small; Cu and M of cubital cell subparallel. **GENITALIA**: (Fig. 194) *Pygophore*: Elongate ovoid; lightly sclerotized expansion below paramere; not expanded laterally in dorsal view. Medial process cylindrical; slender; moderately long, slightly longer than paramere; laterally slightly compressed towards apex; semi-erect; basally slightly curved, apex recurved; apex in posterior view acute, with small hooklike projection. *Paramere*: Cylindrical; moderately long, not reaching medial process; directed posteriad; slightly curved ventrad; apical part not enlarged. *Phallus*: Dorsal phallothecal sclerite shield-shaped; sclerotization reduced (yet not absent) on dorsal surface close to posterior margin of foramen; apical portion of phallothecal sclerite gradually tapering, distinctly keeled medially, laterally indistinctly angulate; apex truncate; posterior margin of foramen broadly concave. Struts apical portion missing; basally separate. Basal plate arm slender; separate; in lateral view slightly curved; bridge short; extension of basal plate expanded laterally onto arm, covering more than 1/2 of arm, curved.

***Female***: (Fig. 193c, d) Different from male as outlined below. Larger than male, total length 14.65–15.26 mm (mean 14.96 mm, Suppl. material 2). Yellowish and brown; anterior pronotal lobe, lateral and ventral surfaces yellowish; posterior pronotal lobe and hemelytron brown; femora brown, with yellow bands, tibiae brown, without band. Basiflagellomere subequal in diameter to pedicel. Process on humeral angle spinous, long.

#### Diagnosis

The uniquely slender, recurved medial process can distinguish this species among the members of the *Zelus panamensis* species group (Fig. 12). In females the anterior pronotal lobe yellowish and posterior lobe brown; the lateral and ventral surface of the body yellowish is unique among females of all species. Females are very similar to *Z. sphegeus*, but are separate from that species by the banded legs and the anterior pronotal lobe nearly flat, not distinctly elevated.

#### Etymology

Named after F. S. Truxal, the collector of the type specimen.

#### Distribution

**Figure 195.**
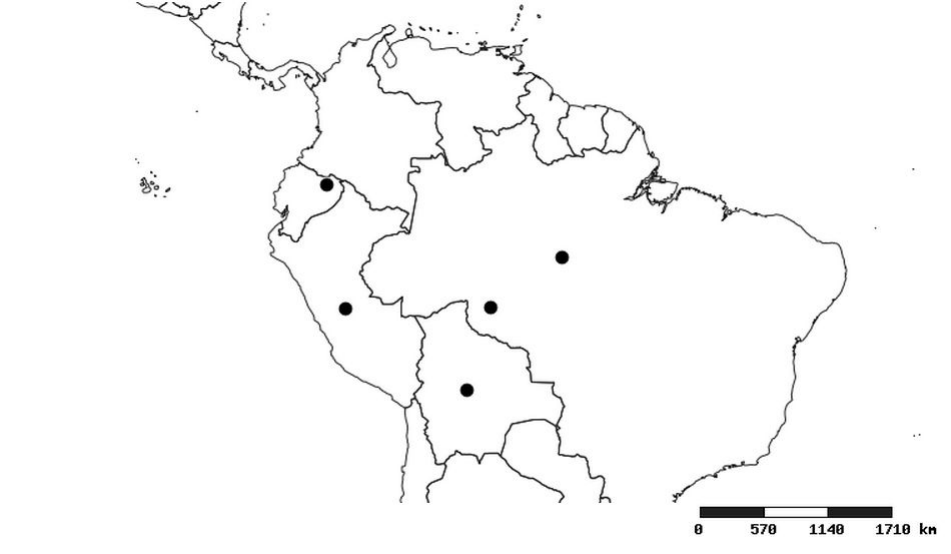
*Zelus truxali* Zhang & Hart, sp. n., specimen record map

South America (Fig. 195). Countries with records: Bolivia, Brazil, Ecuador and Peru.

### Zelus umbraculoides

Zhang sp. n.

urn:lsid:zoobank.org:act:99FA6542-C530-4523-AB91-FB7B915A0ECC

#### Materials

- **Type status:** Holotype. **Occurrence:** catalogNumber: UCR_ENT 00026160; occurrenceRemarks: Drake Collection; recordedBy: L. Pena; sex: Adult Male; **Taxon:** scientificName: Zelus umbraculoides; family: Reduviidae; genus: Zelus; scientificNameAuthorship: Zhang and Hart, 2016; **Location:** country: BOLIVIA; stateProvince: La Paz; locality: Tres Esteros, Guanay; decimalLatitude: -15.4833; decimalLongitude: -67.8833; **Identification:** identifiedBy: G. Zhang; dateIdentified: 2013; **Event:** eventDate: 1989-08-19 to 1989-08-25; **Record Level:** institutionCode: USNM
- **Type status:** Paratype. **Occurrence:** catalogNumber: UCR_ENT 00017040; recordedBy: A. Maller; sex: Adult Male; **Taxon:** scientificName: Zelus umbraculoides; family: Reduviidae; genus: Zelus; scientificNameAuthorship: Zhang and Hart, 2017; **Location:** country: BRAZIL; stateProvince: Parana; locality: Caviuna; decimalLatitude: -23.2; decimalLongitude: -51.36666; georeferenceSources: GeoLocate Software; **Identification:** identifiedBy: G. Zhang; dateIdentified: 2013; **Event:** eventDate: 1947-08-01; **Record Level:** institutionCode: AMNH
- **Type status:** Paratype. **Occurrence:** catalogNumber: UCR_ENT 00029367; occurrenceRemarks: Drake Collection; recordedBy: G. Arriagada; sex: Adult Male; **Taxon:** scientificName: Zelus umbraculoides; family: Reduviidae; genus: Zelus; scientificNameAuthorship: Zhang and Hart, 2016; **Location:** country: PARAGUAY; stateProvince: Alto Parana; locality: Alto Parana; decimalLatitude: -25.60752; decimalLongitude: -54.96117; georeferenceSources: GeoLocate Software; **Identification:** identifiedBy: G. Zhang; dateIdentified: 2013; **Event:** eventDate: 1990-11-12 to 1990-11-16; **Record Level:** institutionCode: USNM
- **Type status:** Paratype. **Occurrence:** catalogNumber: UCR_ENT 00009314; recordedBy: J. B. Heppner; sex: Adult Male; **Taxon:** scientificName: Zelus umbraculoides; family: Reduviidae; genus: Zelus; scientificNameAuthorship: Zhang and Hart, 2016; **Location:** country: PERU; stateProvince: Madre de Dios; county: Tambopata; locality: Rio Tambopata Reserve, 30 air km SW Pto. Maldonado; verbatimElevation: 290 m; decimalLatitude: -12.74338; decimalLongitude: -69.49339; georeferenceSources: Google Earth; **Identification:** identifiedBy: G. Zhang; dateIdentified: 2013; **Event:** eventDate: 1979-11-16 to 1979-11-20; **Record Level:** institutionCode: USNM

#### Description

Figs 196, 197, 198

***Male***: (Fig. 196) Moderately large; total length 14.03-15.05 mm (mean 15.03 mm, Suppl. material 2); robust. **COLORATION**: Entire surface greenish-brown, lateral surfaces lighter; dark brown area on hemelytron adjacent to quadrate cell, inversely v-shaped. **VESTITURE**: Sparsely setose. Similar to that in *Z. umbraculus*. **STRUCTURE: Head**: Cylindrical, short, somewhat robust, L/W = 1.93. Postocular lobe short; in dorsal view distinctly narrowing through anterior 1/2, posterior 1/2 constant, tube-like. Eye prominent; lateral margin much wider than postocular lobe; dorsal margin removed from postocular transverse groove, ventral margin attaining ventral surface of head in lateral view; ocellus situated on prominent elevation. *Labium*: I: II: III = 1: 1.29: 0.32. Basiflagellomere diameter slightly larger than that of pedicel. **Thorax**: Collar indistinct; anterolateral angle rounded, without projection; medial longitudinal sulcus evident only on posterior 1/2, deepening to transverse sulcus of pronotum. Posterior pronotal lobe rugulose; disc elevated above humeral angle, surface strongly convex as viewed from posterior angle; humeral angle armed, with short dentate processes. Scutellum moderately long; apex angulate, slightly projected in some specimens. *Legs*: Moderately robust. *Hemelytron*: Slightly surpassing apex of abdomen, not more than length of abdominal segment seven; quadrate cell small; Cu and M of cubital cell subparallel, converging only anteriorly. **GENITALIA**: (Fig. 197) *Pygophore*: Rounded; not expanded laterally in dorsal view. Medial process cylindrical; base broad; moderately long; straight; posteriorly directed; apex in posterior view rounded, with minute sharp lateral projections. *Paramere*: Cylindrical; not reaching apex of medial process; directed posteriad; gradually thickened towards apex; not distinctly curved. *Phallus*: Dorsal phallothecal sclerite elongated; lateral margins subparallel; apical portion of phallothecal sclerite not distinctly tapered, slightly convex; apex truncate; posterior margin of foramen broadly angulate. Struts attached to dorsal phallothecal sclerite; separate. Basal plate arm moderately robust; mostly separate, in contact before bridge; converging; in lateral view very slightly curved; bridge very short; extension of basal plate moderate, expanded onto arm.

***Female***: Unknown.

#### Diagnosis

Recognized by the body surface greenish-brown; the area of hemelytron adjacent to the quadrate cell dark brown, inversely U-shaped in appearance; the head short and stout (L/W=<2.1) ; the ocellus situated on conspicuous elevation (the same set of characters are also present in *Z. umbraculus*). Characters separating *Z. umbraculoides* and *Z. umbraculus* are discussed in the diagnosis of the latter.

#### Etymology

The specific epithet indicates its close resemblance to *Z. umbraculus*, another new species described in the current study.

#### Distribution

**Figure 198.**
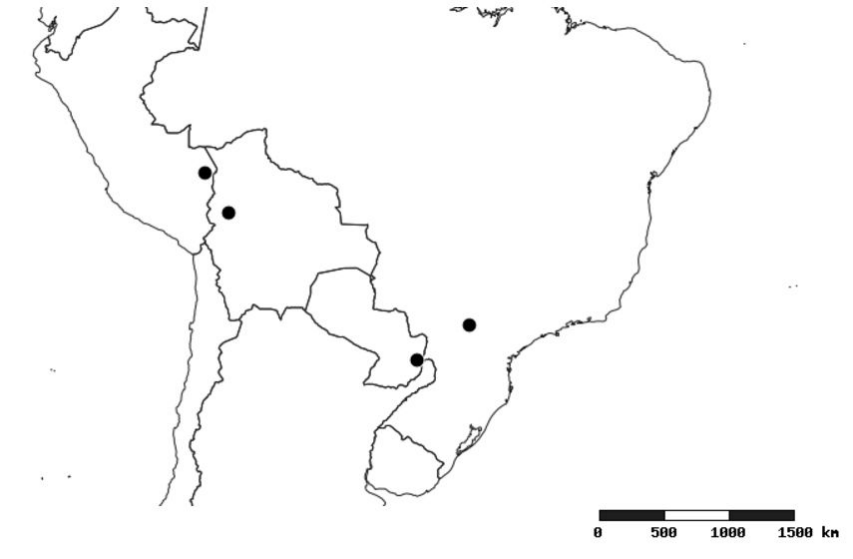
*Zelus umbraculoides* Zhang & Hart, sp. n., specimen record map

South America (Fig. 198). Countries with records: Bolivia, Brazil, Peru and Paraguay.

#### Taxon discussion

Hart (1972) did not discover this species and included some of the specimens under *Z. umbraculus*. Upon a close examination of these and several new specimens collected after 1972, we found several major differences that can reliably separate *Z. umbraculoides* and *Z. umbraculus* (see diagnosis of the latter). Although specimen records are still sparse, the two species do not overlap in distribution. *Zelus umbraculoides* is known from southern Peru and south of Peru in Bolivia, Paraguay and southern Brazil, while *Z. umbraculus* from Ecuador and northern Peru.

### Zelus umbraculus

Zhang & Hart sp. n.

urn:lsid:zoobank.org:act:ACFBEF16-A50A-4600-8D53-16C764F00473

#### Materials

- **Type status:** Holotype. **Occurrence:** catalogNumber: UCR_ENT 00047973; recordedBy: E. I. Schlinger & E. S. Ross; sex: Adult Male; otherCatalogNumbers: CAS Type No. 12717; **Taxon:** scientificName: Zelus umbraculus; family: Reduviidae; genus: Zelus; scientificNameAuthorship: Zhang and Hart, 2016; **Location:** country: PERU; stateProvince: Lambayeque; locality: 94 mi E of Olmos; decimalLatitude: -5.98497; decimalLongitude: -78.3751; georeferenceSources: Google Earth; **Identification:** identifiedBy: G. Zhang; dateIdentified: 2013; **Event:** eventDate: 1955-01-18; **Record Level:** institutionCode: CAS
- **Type status:** Paratype. **Occurrence:** catalogNumber: UCR_ENT 00017041; recordedBy: L. E. Peña; sex: Adult Male; **Taxon:** scientificName: Zelus umbraculus; family: Reduviidae; genus: Zelus; scientificNameAuthorship: Zhang and Hart, 2018; **Location:** country: ECUADOR; stateProvince: Orellana; locality: Coca (on Rio Napo); decimalLatitude: -0.4183; decimalLongitude: -76.9857; georeferenceSources: GeoLocate Software; **Identification:** identifiedBy: G. Zhang; dateIdentified: 2013; **Event:** eventDate: 1965-05-01; **Record Level:** institutionCode: AMNH
- **Type status:** Paratype. **Occurrence:** catalogNumber: UCR_ENT 00006067; recordedBy: E. I. Schlinger & E. S. Ross; sex: Adult Male; **Taxon:** scientificName: Zelus umbraculus; family: Reduviidae; genus: Zelus; scientificNameAuthorship: Zhang and Hart, 2016; **Location:** country: PERU; stateProvince: Lambayeque; locality: 94 mi E of Olmos; decimalLatitude: -5.98497; decimalLongitude: -78.3751; georeferenceSources: Google Earth; **Identification:** identifiedBy: G. Zhang; dateIdentified: 2013; **Event:** eventDate: 1955-01-18; **Record Level:** institutionCode: CAS
- **Type status:** Paratype. **Occurrence:** catalogNumber: UCR_ENT 00019692; recordedBy: E. I. Schlinger & E. S. Ross; sex: Adult Male; **Taxon:** scientificName: Zelus umbraculus; family: Reduviidae; genus: Zelus; scientificNameAuthorship: Zhang and Hart, 2019; **Location:** country: PERU; stateProvince: Lambayeque; locality: 94 mi E of Olmos; decimalLatitude: -5.98497; decimalLongitude: -78.3751; georeferenceSources: Google Earth; **Identification:** identifiedBy: G. Zhang; dateIdentified: 2013; **Event:** eventDate: 1955-01-18; **Record Level:** institutionCode: CAS
- **Type status:** Paratype. **Occurrence:** catalogNumber: UCR_ENT 00019694; recordedBy: E. I. Schlinger & E. S. Ross; sex: Adult Male; **Taxon:** scientificName: Zelus umbraculus; family: Reduviidae; genus: Zelus; scientificNameAuthorship: Zhang and Hart, 2020; **Location:** country: PERU; stateProvince: Lambayeque; locality: 94 mi E of Olmos; decimalLatitude: -5.98497; decimalLongitude: -78.3751; georeferenceSources: Google Earth; **Identification:** identifiedBy: G. Zhang; dateIdentified: 2013; **Event:** eventDate: 1955-01-18; **Record Level:** institutionCode: CAS

#### Description

Figs 199, 200, 201

***Male***: (Fig. 199) Moderately large, total length 14.03–14.72 mm (mean 14.48 mm, Suppl. material 2); robust. **COLORATION**: Entire surface greenish-brown; lateral surfaces lighter; anterior portion of membrane of hemelytron darkened. **VESTITURE**: Sparsely setose. Head with short, erect and some short spine-like setae dorsally, and short, recumbent and long, erect setae ventrally. Posterior pronotal lobe with sparse, scattered, erect and recumbent setae. Abdomen with short, recumbent and short to long, erect setae. **STRUCTURE: Head**: Cylindrical, L/W = 2.06. Postocular lobe relatively short; in dorsal view distinctly narrowing through anterior 1/2, posterior 1/2 constant, tube-like. Eye prominent; lateral margin much wider than postocular lobe; dorsal margin removed from postocular transverse groove, ventral margin attaining ventral surface of head in lateral view. *Labium*: I: II: III = 1: 1.43: 0.31 Basiflagellomere diameter subequal to that of pedicel. **Thorax**: Anterolateral angle rounded, without projection; medial longitudinal sulcus evident only on posterior 1/2, deepening anterior to transverse sulcus of pronotum. Posterior pronotal lobe with rugulose surface; disc distinctly elevated above humeral angle; humeral angle armed, with short spinous processes. Scutellum moderately long; apex blunt, slightly projected upward. *Legs*: Moderately robust, femoral diameters subequal. *Hemelytron*: Slightly surpassing apex of abdomen, less than half length of abdominal segment seven; quadrate cell moderately sized; Cu and M of cubital cell converging towards R. **GENITALIA**: (Fig. 200) *Pygophore*: Rounded; not expanded laterally in dorsal view. Medial process cylindrical; base broad; moderately long; straight; apex in posterior view rounded, with small sharp lateral projections. *Paramere*: Cylindrical; moderately long, not reaching apex of medial process; directed posteriad; basally constricted; not distinctly curved. *Phallus*: Dorsal phallothecal sclerite elongated; lateral expansion arising close to base; apical portion of phallothecal sclerite not distinctly tapered, slightly convex; apex rounded; posterior margin of foramen broadly concave. Struts attached to dorsal phallothecal sclerite; apically separate, connected by bridge; basally mostly separate, moderately fused. Basal plate arm moderately robust; separate; slightly converging; in lateral view very slightly curved; bridge short; extension of basal plate small, marginally expanded onto arm.

***Female***: Unknown.

#### Diagnosis

Recognized by the body surface greenish-brown; the area of hemelytron adjacent to the quadrate cell dark brown, inversely V-shaped in appearance; the head short and stout (L/W=<2.1) ; the ocellus situated on conspicuous elevation (the same set of characters are also present in *Z. umbraculus*). *Zelus umbraculus* can be separated from *Z. umbraculoides* by several characters listed below (Figs 8, 9). *Zelus umbraculus*: (1) Disc of the posterior pronotal lobe slightly bulging, nearly flat; (2) paramere moderately robust, apical 1/2 greater; (3) apex of the dorsal phallothecal sclerite rounded; and (4) basal plate arms apically separate. *Zelus umbraculoides*: (1) Disc of the posterior pronotal lobe clearly bulging; (2) paramere relatively slender; (3) apex of the dorsal phallothecal sclerite truncate, and (4) basal plate arms apically touching. The disc of the pronotal lobe is best observed from a posterior angle with the head facing down.

#### Etymology

The specific epithet is from Latin "umbra", referring to the shadow in the anterior part of the membrane.

#### Distribution

**Figure 201.**
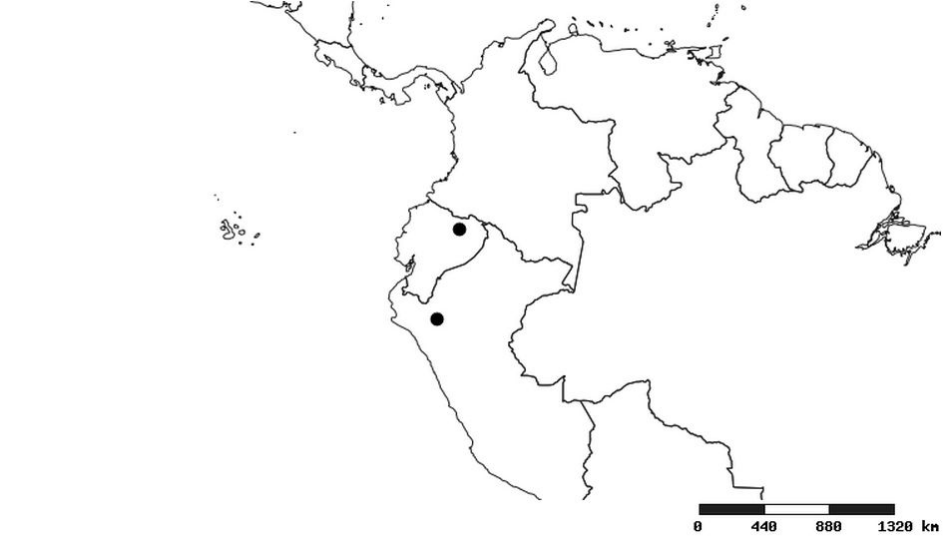
*Zelus umbraculus*, Zhang & Hart, sp. n., specimen record map

South America (Fig. 201). Countries with records: Ecuador and Peru.

### Zelus vagans

Fabricius, 1803

Zelus vagans Fabricius, 1803, p. 284, orig. descr.; Stål, 1868, p. 108, descr. and note; Stål, 1872, p. 88, cat.; Walker, 1873, p. 134, cat.; Lethierry and Severin, 1896, p. 153, cat.; Wygodzinsky, 1949a, p. 50, checklist; Maldonado, 1990, p. 332, cat.

#### Materials

- **Type status:** Holotype. **Occurrence:** catalogNumber: UCR_ENT 00075107; recordedBy: Dom. Smidt; sex: Adult Male; otherCatalogNumbers: ZMUC 102689; **Taxon:** scientificName: Zelus vagans; family: Reduviidae; genus: Zelus; scientificNameAuthorship: Fabricius, 1803; **Location:** country: unknown; stateProvince: unknown; locality: Habitat in America meridionali; **Identification:** identifiedBy: G. Zhang; dateIdentified: 2012; **Event:** eventDate: No date provided; **Record Level:** institutionCode: ZMUC

#### Description

Figs 202, 203, 204

***Male***: (Fig. 202) Medium-sized, total length 11.29–14.71 mm (mean 13.71 mm, Table 4.2); slender. **COLORATION**: Entire surface of head, antenna and labium dark brown; extremely slender medial longitudinal lighter stripe on postocular lobe. Anterior pronotal lobe dark brown. Medial longitudinal dark brown stripe on posterior pronotal lobe; rest of dorsal surface orange; lateral surface dark brown or orange. Scutellar disc dark brown, margins orange or brown. Sternites dark brown. Corium proximally dark brown, distally orange, proportions of brown and orange vary slightly among specimens, sometimes with rather small dark brown patch at very distal. Clavus orange. Membrane dark brown; veins same color as rest. Legs dark brown; profemur with or without band; meso- and metafemora each with two orange bands, apical band usually smaller. Abdominal segments 2–6 reddish, amount on segment 6 varies; segment seven and pygophore dark brown. **VESTITURE**: Densely setose. Dorsum with short, erect, spine-like setae, dense on anteocular lobe, sparse on postocular lobe; dense, short, recumbent setae over entire dorsal surface of postocular lobe; ventral surface also with sparse, short to moderately long, erect, fine setae. Pronotum primarily with dense, short, erect, spine-like setae on dorsal and lateral surfaces; spine-like setae on pleura sparse, mainly with longer, erect, fine setae and short, recumbent setae; scutellum with dense, short to long, semi-erect to recumbent setae; spine-like setae sparse. Legs with sparse setae; sundew setae on profemur sparse and randomly arranged. Corium and clavus with dense, recumbent, stout setae. Abdomen with moderately dense, short, semi-erect, fine setae, interspersed with sparse, longer, erect setae; segment seven in some specimens with setae covered with white wax-like exudation. Pygophore with short to long, semi-erect setae; dense, moderately long, semi-erect setae nearly throughout dorsal, inner surface of paramere. **STRUCTURE: Head**: Cylindrical, L/W = 2.34. Postocular lobe in dorsal view anteriorly gradually narrowing, posterior portion constant, slightly narrower. Eye moderately sized; lateral margin only slightly wider than postocular lobe; dorsal margin removed from postocular transverse groove, ventral margin attaining ventral surface of head in lateral view. *Labium*: I: II: III = 1: 1.5: 0.4. Basiflagellomere diameter slightly larger than that of pedicel. **Thorax**: Anterolateral angle rounded, without projection; medial longitudinal sulcus evident throughout, deepening posteriorly. Disc distinctly elevated above humeral angle; humeral angle rounded, without projection. Scutellum moderately long; apex pointed, sometimes as short process, not projected. *Legs*: Very slender. *Hemelytron*: Greatly surpassing apex of abdomen by about 3x length of abdominal segment seven; quadrate cell large and broad; Cu and M of cubital cell subparallel. **GENITALIA**: (Fig. 203) *Pygophore*: Ovoid; mid-lateral fold adjacent to paramere insertion; not expanded laterally in dorsal view. Medial process somewhat cone-shaped, laterally compressed toward apex; moderately long; posteriorly directed, in less than forty-five degree with body axis; nearly straight; apex in posterior view blunt. *Paramere*: Cylindrical; moderately long, reaching about mid-point of medial process; directed posteriad, slightly curved towards medial process; slightly curved ventrad; apical part not enlarged. *Phallus*: Dorsal phallothecal sclerite elongated; medial portion with dorsal paired hump; apical portion of phallothecal sclerite gradually tapering, carinate medially, laterally angulate; apex with small medial emargination; posterior margin of foramen broadly concave. Struts attached to dorsal phallothecal sclerite; apically separate, connected by bridge; basally mostly separate, moderately fused. Basal plate arm slender; separate; subparallel; in lateral view nearly straight, very slightly curved; bridge moderately long; extension of basal plate small, marginally expanded onto arm.

***Female***: Unknown.

#### Diagnosis

Can be easily identified by the unique coloration pattern, the posterior pronotal lobe medially black and laterally orange. Distinguished among members of the *Zelus vagans* species group by the smaller size; the postocular lobe covered with recumbent setae. The paramere is similar to that in *Z. championi* in showing ventrally directed curvature, but is shorter than in *Z. championi* and reaching to only about mid-point of medial process.

#### Distribution

**Figure 204.**
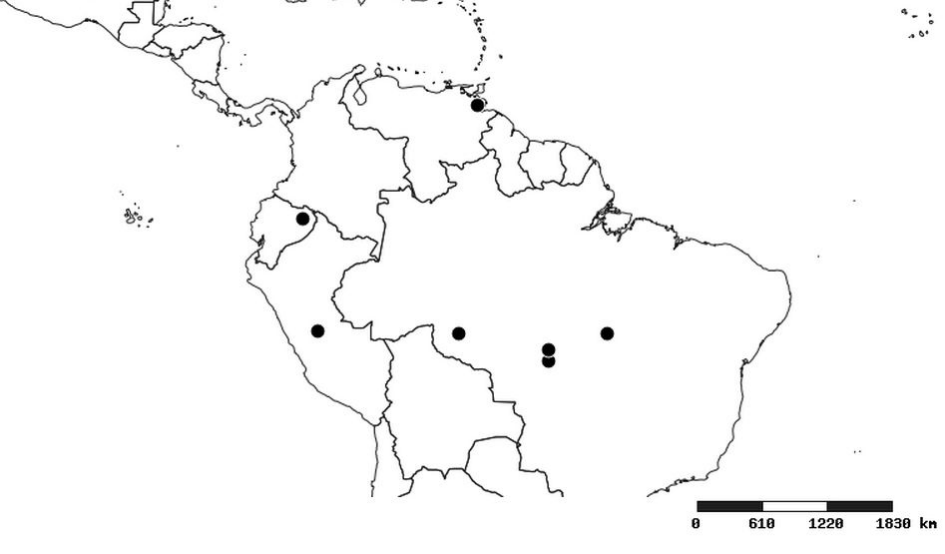
*Zelus vagans* Fabricius, 1803, specimen record map

South America (Fig. 204). Countries with records: Brazil, Colombia, Ecuador, Peru and Venezuela.

### Zelus varius

(Herrich-Schaeffer, 1853)

Euagoras varius Herrich-Schaeffer, 1853, p. 122, orig. descr.
Zelus varius : Stål , 1872, p. 92, cat . (subgenus *Diplodus*); Lethierry and Severin, 1896, p. 153, cat.; Wygodzinsky 1949a, p. 50, checklist; Maldonado, 1990, p. 332, cat.
Diplodus varius : Walker, 1873, p. 126, cat.

#### Materials

- **Type status:** Neotype. **Occurrence:** catalogNumber: UCR_ENT 00069896; occurrenceRemarks: Neotype of *Zelus varius* (Herrich-Schaeffer, 1853) (**New Designation** by Zhang, Hart & Weirauch, 2016); recordedBy: J. R. de la Torre-Bueno; sex: Adult Male; **Taxon:** scientificName: Zelus varius; family: Reduviidae; genus: Zelus; scientificNameAuthorship: (Herrich-Schaeffer, 1853); **Location:** country: GUYANA; stateProvince: Cuyuni-Mazaruni Region; locality: Bartica; decimalLatitude: 5.78765; decimalLongitude: -57.62801; **Identification:** identifiedBy: G. Zhang; dateIdentified: 2012; **Event:** eventDate: No date provided; **Record Level:** institutionCode: KU

#### Description

Figs 205, 206, 207

***Male***: (Fig. 205) Small, total length 10.11 mm (n=1, Suppl. material 2); slender. **COLORATION**: Head uniformly brown; postocular lobe with very faint longitudinal medial stripe. Pronotum and scutellum pale brown, anterior lobe slightly darker. Very faint bands on legs. **VESTITURE**: Sparsely setose. Short, recumbent setae on entire surface of head; very short, erect, spine-like setae on dorsum, denser on anterior lobe; few moderately long, erect, fine setae on ventral surface. Pronotum with sparse, recumbent setae and short, erect setae over dorsal surface; denser, moderately long recumbent setae on lateral surface and pleura, intermixed with semierect or erect setae; scutellum with sparse, semi-erect and recumbent setae. Legs with sparse setation on femora and moderately dense setation on tibiae. Corium and clavus with mix of sparse, short, recumbent and erect setae. Abdomen with moderately dense, short recumbent setae, intermixed with sparse, short to long, erect setae. **STRUCTURE: Head**: Cylindrical, L/W = 2.27. Postocular lobe long; in dorsal view distinctly narrowing through anterior 2/3, posterior 1/3 constant, tube-like. Dorsal margin attaining postocular transverse groove, ventral margin removed from ventral surface of head in lateral view. *Labium*: I: II: III = 1: 1.7: 0.4. Basiflagellomere diameter slightly larger than that of pedicel. **Thorax**: Anterolateral angle with inconspicuous subtuberculate projection; medial longitudinal sulcus evident throughout, deepening posteriorly. Posterior pronotal lobe with rugulose surface; disc distinctly elevated above humeral angle; humeral angle armed, with spinous processes. Scutellum moderately long; apex angulate, very slightly projected upward. *Legs*: Slender. *Hemelytron*: Surpassing apex of abdomen by about length of abdominal segment seven; quadrate cell small and slender; Cu and M of cubital cell subparallel. **GENITALIA**: (Fig. 206) *Pygophore*: Elongate ovoid; not expanded laterally in dorsal view. Medial process cylindrical; slender; long, much longer than paramere; laterally compressed toward apex; anterior surface towards apex ridged; minute spicules on posterior surface; semi-erect; curved at middle; apex in posterior view acute, with small hooklike projection, somewhat re-expanded laterally. *Paramere*: Cylindrical; short, not reaching medial process; directed posteriad; base narrower; slightly curved ventrad; apical part very slightly enlarged. *Phallus*: Dorsal phallothecal sclerite elongated; sclerotization reduced (yet not absent) on dorsal surface close to posterior margin of foramen; apical portion of phallothecal sclerite gradually tapering, distinctly keeled medially, laterally flat, not forming angle; apex acute; posterior margin of foramen broadly concave. Basal plate arm slender; separate; converging; in lateral view slightly curved; bridge short; extension of basal plate expanded laterally onto arm, covering more than 1/2 of arm, curved.

***Female***: Similar to male, except for the following. Larger than male, total length 13.71–14.03 mm (mean 13.87 mm, Suppl. material 2). Entire body nearly uniformly pale brown or dark brown.

#### Diagnosis

Recognized by the following combination of characters: The posterior margin of posterior pronotal lobe with expansion laterad to scutellum; the rather long, spinous process on humeral angle; the smallish body size; the paramere short; the apical part of medial process compressed laterally, anterior side ridge-like; and the apex of medial process re-expanded, not acute.

#### Distribution

**Figure 207.**
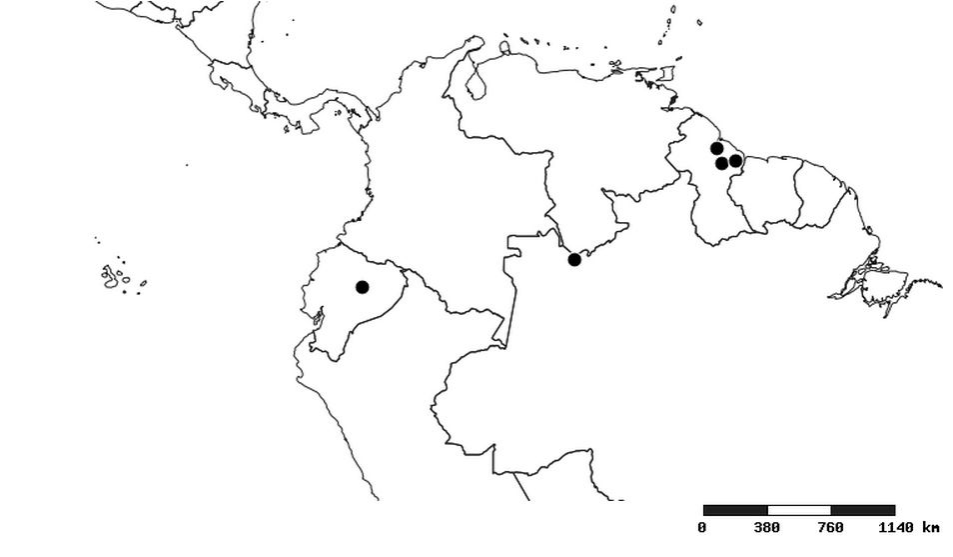
*Zelus varius* (Herrich-Schaeffer, 1853), specimen record map

South America (Fig. 207). Countries with records: Brazil, Ecuador and Guyana.

### Zelus versicolor

(Herrich-Schaeffer, 1848)

Euagoras versicolor Herrich-Schaeffer, 1848, p. 46-47, Tab. CCLXIV, fig. 820, orig. descr. and fig.; Herrich-Schaeffer, 1853, p. 92, cat.; Walker, 1873, p. 118, note.
Diplodus versicolor , Stål, 1860, p. 75, cat.
Zelus versicolor : Stål, 1862, p. 451, note; Stål, 1872, p. 92, cat. (subgenus *Diplodus*); Lethierry and Severin, 1896, p. 153, cat.; Wygodzinsky, 1949a, p. 50, checklist; Maldonado, 1990, p. 332, cat.
Euagoras nigrispinus Herrich-Schaeffer, 1848, p. 47-48, Tab. CCLXII, fig. 816, orig. descr. and fig.; Herrich-Schaeffer, 1853, p. 92, cat. Gil-Santana, 2008, p. 48, Figs 15-21, junior syn. of *Zelus versicolor* (Herrich-Schaeffer, 1848).
Diplodus nigrispinus : Stål, 1860, p. 75, cat.; Stål, 1868, p. 109, note; Walker, 1873, p. 126, cat.
Zelus nigrispinus : Mayr, 1866, p. 139, cat.; Stål, 1872, p. 91, cat. (subgenus *Diplodus*); Berg, 1879, p. 154; Lethierry and Severin, 1896, p. 152, cat.; Wygodzinsky, 1949a, p. 49, checklist; Maldonado, 1990, p. 330, cat; Gil-Santana, 2008, p. 48, junior syn. of *Z. versicolor*.
Zelus personatus Berg, 1879, p. 150-151, orig. descr. (subgenus *Zelus)*; Lethierry and Severin, 1896, p. 153, cat.; Wygodzinsky, 1949a, p. 50, checklist; Wygodzinsky, 1957, p. 264, 268-269, list and note; Maldonado, 1990, p. 330, cat. **syn. nov.** (current study).

#### Materials

- **Type status:** Other material. **Occurrence:** occurrenceRemarks: Holotype of *Zelus personatus* Berg, 1879, junior synonym o *Zelus versicolor* (Herrich-Schaeffer, 1848). Bears the following labels: Typus / Misiones / *Zelus personatus* Berg / *Zelus personatus* Berg, Wygodzinsky det./1555; sex: Adult Male; **Taxon:** scientificName: Zelus versicolor; family: Reduviidae; genus: Zelus; scientificNameAuthorship: (Herrich-Schaeffer, 1848); **Location:** country: ARGENTINA; stateProvince: Misiones; **Identification:** identifiedBy: E. R. Hart; dateIdentified: 1972; **Event:** eventDate: no date provided; **Record Level:** institutionCode: Universidad Nacional de La Plata, La Plata

#### Description

Figs 208, 209, 210

***Male***: (Fig. 208a, b) Medium-sized, total length 11.59–12.94 mm (mean 12.26 mm, Table 4.2); slender. **COLORATION**: Dark brown to brownish-black. Head ventrally yellowish to pale brown, light color rarely dominant on dorsal surface; postocular lobe with light medial longitudinal line. Pronotum dark brown. Posterior pronotal lobe lighter colored than anterior lobe, sometimes reddish or orangish-brown. Pleura with patches of brown and yellow colors. Profemur, pro- and mesotibiae not banded; mesofemur occasionally with subapical or (and) basal short light-colored band; metafemur sometimes with three short yellowish and three longer darker bands, light and dark bands alternate, but some individuals with just single subapical or basal light band; metatibia sometimes slightly paler medially. Abdomen usually light-colored. **VESTITURE**: Sparsely setose. Dorsum of head, entire surface of pronotum and pleura with short, spine-like setae and recumbent setae; ventral surfaces of head and abdomen with short, recument and relatively long, erect setae. Setal tracts on anterior pronotal lobe indistinct. Dense, moderately long, erect setae on dorsal surface of paramere. Head pubescence sparse, of mostly recument setae intermixed with short sparse erect setae. Dorsal surface of anterior pronotal lobe covered densely with short, erect, somewhat stout setae; posterior lobe covered densely with short, erect, somewhat stout setae; scutellum with recumbent setae interspersed with short to long, sparse, semierect to erect setae. Corium and clavus with short, recumbent setae. Abdomen with short, recumbent setae intermixed with short to long, erect setae. Ventrally exposed surface of pygophore with dense, short to long, semierect to erect setae; parameres with dense long erect setae over enlarged surface, sparse and shorter ventrally. **STRUCTURE: Head**: Cylindrical, L/W = 2.37. Postocular lobe long; in dorsal view distinctly narrowing through anterior 2/3, posterior 1/3 constant, tube-like. Eye prominent; lateral margin much wider than postocular lobe; dorsal and ventral margins removed from surfaces of head. *Labium*: I: II: III = 1: 2.0: 0.4. Basiflagellomere diameter slightly larger than that of pedicel. **Thorax**: Anterolateral angle rounded, without projection; medial longitudinal sulcus shallow near collar, deepening posteriorly. Posterior pronotal lobe with rugulose surface; disc about same level as humeral angle; humeral angle armed, with dentate projection. Scutellum moderately long; apex angulate, slightly projected upward in some specimens. *Legs*: Slender. *Hemelytron*: Slightly surpassing apex of abdomen, not more than length of abdominal segment seven; quadrate cell small; Cu and M of cubital cell subparallel. **GENITALIA**: (Fig. 209) *Pygophore*: Ovoid; expanded laterally near base of paramere in dorsal view. Medial process triangular; long; anteroposteriorly compressed; erect; basally without protrusion; apex in posterior view acute; posterior surface with pair of sharp ventrally directed processes. *Paramere*: Bulbous, moderately long, slightly exceeding medial process; curved toward medial process; basally constricted; slightly curved ventrad; apical part enlarged. *Phallus*: Dorsal phallothecal sclerite somewhat squarish; laterally with small blade-like heavily sclerotized process; apical portion of phallothecal sclerite not distinctly tapered, laterally indistinctly angulate, angulation anteriorly connected with membranous, dorsad expansion; apex rounded, medially emarginate; posterior margin of foramen broadly concave. Struts attached to dorsal phallothecal sclerite; apically separate, connected by bridge; basally mostly separate, moderately fused. Basal plate arm moderately robust; separate; subparallel; in lateral view severely curved, nearly semi-circular; bridge long; extension of basal plate expanded onto arm.

***Female***: Similar to male, except for the following. Larger than male, total length 14.43–15.84 mm (mean 15.01 mm, Table 4.2). Dorsum dark brown, a large number of specimens with anterior 1/2 of posterior pronotal lobe yellow and some also with entire anterior pronotal lobe yellow; lateral surface and abdomen yellowish. Hemelytron slightly surpassing apex of abdomen.

#### Diagnosis

Recognized by the following combination of characters: the posterior pronotal lobe usually lighter than the anterior pronotal lobe, orangish or reddish-brown; the paramere bulbous, basally constricted, curved towards medial process; the medial process triangular, apex with pair of processes; the dorsal phallothecal sclerite with submedial ridge-like dorsad projection continuous from basal arm; and the basal plate arms subparallel and strongly curved. In females the posterior pronotal lobe is often bicolorous, anterior portion yellowish and posterior portion brown.

#### Distribution

**Figure 210.**
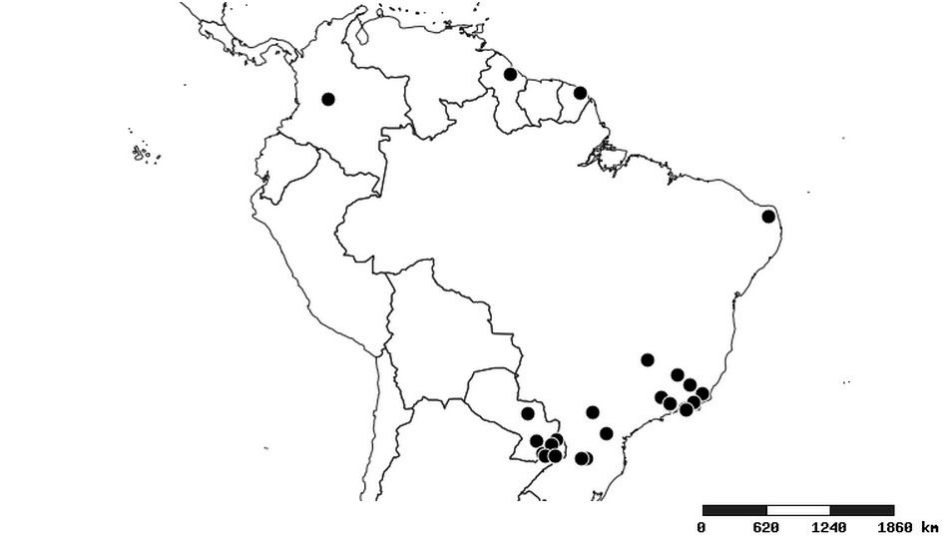
*Zelus versicolor* (Herrich-Schaeffer, 1848), specimen record map

South America (Fig. 210). Countries with records: Argentina, Brazil, Colombia, French Guiana, Guiana and Paraguay.

#### Taxon discussion

The type specimens of *Z. versicolor* and *Z. nigrispinus* were destroyed during World War II.

### Zelus vespiformis

Hart, 1987

Zelus vespiformis Hart, 1987, p. 301, figs. 22-27, orig. descr., note, fig. and key; Maldonado, 1990, p. 332, cat.

#### Materials

- **Type status:** Holotype. **Occurrence:** catalogNumber: UCR_ENT 00057794; occurrenceRemarks: The institution code on the barcode (USI) lable is AM ENT, referring to the American Museum of Natural History. This is an error. The correct form should be AMNH ENT. Also the USI number is UCR_ENT 57794, which is lacking three '0' before '57794'.; recordedBy: H. F. Schwarz; sex: Adult Male; **Taxon:** scientificName: Zelus vespiformis; family: Reduviidae; genus: Zelus; scientificNameAuthorship: Hart, 1987; **Location:** country: PANAMA; stateProvince: Canal Zone; locality: Barro Colorado Island, Drayton Trail; decimalLatitude: 9.15472; decimalLongitude: -79.84806; georeferenceSources: GeoLocate Software; **Identification:** identifiedBy: G. Zhang; dateIdentified: 2012; **Event:** eventDate: 1930-11-11; **Record Level:** institutionCode: AMNH
- **Type status:** Allotype. **Occurrence:** catalogNumber: UCR_ENT 00057795; recordedBy: H. F. Schwarz; sex: Adult Female; **Taxon:** scientificName: Zelus vespiformis; family: Reduviidae; genus: Zelus; scientificNameAuthorship: Hart, 1987; **Location:** country: PANAMA; stateProvince: Canal Zone; locality: Barro Colorado Island, Drayton Trail; decimalLatitude: 9.15472; decimalLongitude: -79.84806; georeferenceSources: GeoLocate Software; **Identification:** identifiedBy: G. Zhang; dateIdentified: 2012; **Event:** eventDate: 1930-11-11; **Record Level:** institutionCode: AMNH
- **Type status:** Paratype. **Occurrence:** catalogNumber: UCR_ENT 00071181; recordedBy: Richter; sex: Adult Female; **Taxon:** scientificName: Zelus vespiformis; family: Reduviidae; genus: Zelus; scientificNameAuthorship: Hart, 1987; **Location:** country: COLOMBIA; stateProvince: Amazonas; locality: Rio Cotuhe, Tatapaca; decimalLatitude: -2.88331; decimalLongitude: -69.73332; georeferenceSources: Google Earth; **Identification:** identifiedBy: G. Zhang; dateIdentified: 2012; **Event:** eventDate: 1946-09-12; **Record Level:** institutionCode: TAMU
- **Type status:** Paratype. **Occurrence:** catalogNumber: UCR_ENT 00009411; recordedBy: F. Jotaya; sex: Adult Female; **Taxon:** scientificName: Zelus vespiformis; family: Reduviidae; genus: Zelus; scientificNameAuthorship: Hart, 1987; **Location:** country: COLOMBIA; stateProvince: Cundinamarca; locality: Fusagasuga; verbatimElevation: 1750 m; **Identification:** identifiedBy: G. Zhang; dateIdentified: 2012; **Event:** eventDate: 1940-01-02; **Record Level:** institutionCode: USNM
- **Type status:** Paratype. **Occurrence:** catalogNumber: UCR_ENT 00009413; recordedBy: E.A. Chapin; sex: Adult Female; **Taxon:** scientificName: Zelus vespiformis; family: Reduviidae; genus: Zelus; scientificNameAuthorship: Hart, 1987; **Location:** country: COLOMBIA; stateProvince: Cundinamarca; locality: El Colegio; decimalLatitude: 4.58472; decimalLongitude: -74.94944; georeferenceSources: Gazetteer; **Identification:** identifiedBy: G. Zhang; dateIdentified: 2012; **Event:** eventDate: 1946-05-23; **Record Level:** institutionCode: USNM
- **Type status:** Paratype. **Occurrence:** catalogNumber: UCR_ENT 00040461; recordedBy: Richter; sex: Adult Female; **Taxon:** scientificName: Zelus vespiformis; family: Reduviidae; genus: Zelus; scientificNameAuthorship: Hart, 1987; **Location:** country: COLOMBIA; stateProvince: Cundinamarca; locality: Fusagasuga; decimalLatitude: 4.3439; decimalLongitude: -74.3678; georeferenceSources: Gazetteer; **Identification:** identifiedBy: G. Zhang; dateIdentified: 2012; **Event:** eventDate: 1944-06-05; **Record Level:** institutionCode: TAMU
- **Type status:** Paratype. **Occurrence:** catalogNumber: UCR_ENT 00071173; recordedBy: Richter; sex: Adult Female; **Taxon:** scientificName: Zelus vespiformis; family: Reduviidae; genus: Zelus; scientificNameAuthorship: Hart, 1987; **Location:** country: COLOMBIA; stateProvince: Cundinamarca; locality: Fusagasuga; decimalLatitude: 4.3439; decimalLongitude: -74.3678; georeferenceSources: Gazetteer; **Identification:** identifiedBy: G. Zhang; dateIdentified: 2012; **Event:** eventDate: 1944-06-05; **Record Level:** institutionCode: TAMU
- **Type status:** Paratype. **Occurrence:** catalogNumber: UCR_ENT 00040462; recordedBy: F. W. Walker; sex: Adult Female; **Taxon:** scientificName: Zelus vespiformis; family: Reduviidae; genus: Zelus; scientificNameAuthorship: Hart, 1987; **Location:** country: COLOMBIA; stateProvince: Magdalena; locality: Rio Frio; verbatimElevation: 716 m; decimalLatitude: 10.92912; decimalLongitude: -74.13301; georeferenceSources: Google Earth; **Identification:** identifiedBy: G. Zhang; dateIdentified: 2012; **Event:** eventDate: 1925-07-20; **Record Level:** institutionCode: TAMU
- **Type status:** Paratype. **Occurrence:** catalogNumber: UCR_ENT 00071180; recordedBy: F. W. Walker; sex: Adult Female; **Taxon:** scientificName: Zelus vespiformis; family: Reduviidae; genus: Zelus; scientificNameAuthorship: Hart, 1987; **Location:** country: COLOMBIA; stateProvince: Magdalena; locality: Sevilla; decimalLatitude: 10.76343; decimalLongitude: -74.13916; georeferenceSources: Gazetteer; **Identification:** identifiedBy: G. Zhang; dateIdentified: 2012; **Event:** eventDate: 1926-08-14; **Record Level:** institutionCode: TAMU
- **Type status:** Paratype. **Occurrence:** catalogNumber: UCR_ENT 00009415; recordedBy: Richter; sex: Adult Female; **Taxon:** scientificName: Zelus vespiformis; family: Reduviidae; genus: Zelus; scientificNameAuthorship: Hart, 1987; **Location:** country: COLOMBIA; stateProvince: Meta; locality: Rio Guamal [Quamal?]; verbatimElevation: 400 m; decimalLatitude: 3.7987; decimalLongitude: -73.66261; georeferenceSources: Google Earth; **Identification:** identifiedBy: G. Zhang; dateIdentified: 2012; **Event:** eventDate: 1948-01-24; **Record Level:** institutionCode: USNM
- **Type status:** Paratype. **Occurrence:** catalogNumber: UCR_ENT 00009416; recordedBy: Richter; sex: Adult Female; **Taxon:** scientificName: Zelus vespiformis; family: Reduviidae; genus: Zelus; scientificNameAuthorship: Hart, 1987; **Location:** country: COLOMBIA; stateProvince: Meta; locality: Cano Grande, Selba del Llano; verbatimElevation: 450 m; **Identification:** identifiedBy: G. Zhang; dateIdentified: 2012; **Event:** eventDate: 1948-01-20; **Record Level:** institutionCode: USNM
- **Type status:** Paratype. **Occurrence:** catalogNumber: UCR_ENT 00017877; recordedBy: Richter; sex: Adult Male; **Taxon:** scientificName: Zelus vespiformis; family: Reduviidae; genus: Zelus; scientificNameAuthorship: Hart, 1987; **Location:** country: COLOMBIA; stateProvince: Meta; locality: Rio Guayeriba, a triburary of Rio Meta; **Identification:** identifiedBy: G. Zhang; dateIdentified: 2012; **Event:** eventDate: 1948-01-24; **Record Level:** institutionCode: AMNH
- **Type status:** Paratype. **Occurrence:** catalogNumber: UCR_ENT 00017879; recordedBy: Richter; sex: Adult Female; **Taxon:** scientificName: Zelus vespiformis; family: Reduviidae; genus: Zelus; scientificNameAuthorship: Hart, 1987; **Location:** country: COLOMBIA; stateProvince: Meta; locality: Rio Guayeriba, a triburary of Rio Meta; **Identification:** identifiedBy: G. Zhang; dateIdentified: 2012; **Event:** eventDate: 1948-01-24; **Record Level:** institutionCode: AMNH
- **Type status:** Paratype. **Occurrence:** catalogNumber: UCR_ENT 00040463; recordedBy: Richter; sex: Adult Male; **Taxon:** scientificName: Zelus vespiformis; family: Reduviidae; genus: Zelus; scientificNameAuthorship: Hart, 1987; **Location:** country: COLOMBIA; stateProvince: Meta; locality: Rio Guayuriba; verbatimElevation: 400 m; decimalLatitude: 4.01978; decimalLongitude: -73.60807; georeferenceSources: Google Earth; **Identification:** identifiedBy: G. Zhang; dateIdentified: 2012; **Event:** eventDate: 1944-09-06; **Record Level:** institutionCode: TAMU
- **Type status:** Paratype. **Occurrence:** catalogNumber: UCR_ENT 00040464; recordedBy: Richter; sex: Adult Male; **Taxon:** scientificName: Zelus vespiformis; family: Reduviidae; genus: Zelus; scientificNameAuthorship: Hart, 1987; **Location:** country: COLOMBIA; stateProvince: Meta; locality: Cano Grande, Selba del Llano; verbatimElevation: 450 m; **Identification:** identifiedBy: G. Zhang; dateIdentified: 2012; **Event:** eventDate: 1944-03-06; **Record Level:** institutionCode: TAMU
- **Type status:** Paratype. **Occurrence:** catalogNumber: UCR_ENT 00071175; recordedBy: Richter; sex: Adult Female; **Taxon:** scientificName: Zelus vespiformis; family: Reduviidae; genus: Zelus; scientificNameAuthorship: Hart, 1987; **Location:** country: COLOMBIA; stateProvince: Meta; locality: Rio Guayuriba; verbatimElevation: 400 m; decimalLatitude: 4.01978; decimalLongitude: -73.60807; georeferenceSources: Google Earth; **Identification:** identifiedBy: G. Zhang; dateIdentified: 2012; **Event:** eventDate: 1947-09-06; **Record Level:** institutionCode: TAMU
- **Type status:** Paratype. **Occurrence:** catalogNumber: UCR_ENT 00071176; recordedBy: Richter; sex: Adult Female; **Taxon:** scientificName: Zelus vespiformis; family: Reduviidae; genus: Zelus; scientificNameAuthorship: Hart, 1987; **Location:** country: COLOMBIA; stateProvince: Meta; locality: Cano Grande, Selba del Llano; verbatimElevation: 450 m; **Identification:** identifiedBy: G. Zhang; dateIdentified: 2012; **Event:** eventDate: 1947-12-02; **Record Level:** institutionCode: TAMU
- **Type status:** Paratype. **Occurrence:** catalogNumber: UCR_ENT 00071177; recordedBy: Richter; sex: Adult Female; **Taxon:** scientificName: Zelus vespiformis; family: Reduviidae; genus: Zelus; scientificNameAuthorship: Hart, 1987; **Location:** country: COLOMBIA; stateProvince: Meta; locality: Cano Grande, Selba del Llano; verbatimElevation: 450 m; **Identification:** identifiedBy: G. Zhang; dateIdentified: 2012; **Event:** eventDate: 1948-02-20; **Record Level:** institutionCode: TAMU
- **Type status:** Paratype. **Occurrence:** catalogNumber: UCR_ENT 00071174; recordedBy: F. W. Walker; sex: Adult Female; **Taxon:** scientificName: Zelus vespiformis; family: Reduviidae; genus: Zelus; scientificNameAuthorship: Hart, 1987; **Location:** country: COLOMBIA; stateProvince: Santander; locality: Vista Nieve; decimalLatitude: 6.98944; decimalLongitude: -73.66972; georeferenceSources: Gazetteer; **Identification:** identifiedBy: G. Zhang; dateIdentified: 2012; **Event:** eventDate: 1926-08-07; **Record Level:** institutionCode: TAMU
- **Type status:** Paratype. **Occurrence:** catalogNumber: UCR_ENT 00009405; recordedBy: B. Losada; sex: Adult Male; **Taxon:** scientificName: Zelus vespiformis; family: Reduviidae; genus: Zelus; scientificNameAuthorship: Hart, 1987; **Location:** country: COLOMBIA; stateProvince: unknown; locality: unknown; decimalLatitude: 5.02584; decimalLongitude: -74.02986; georeferenceSources: Google Earth; **Identification:** identifiedBy: G. Zhang; dateIdentified: 2012; **Event:** eventDate: no date provided; **Record Level:** institutionCode: USNM
- **Type status:** Paratype. **Occurrence:** catalogNumber: UCR_ENT 00009412; recordedBy: B. Losada; sex: Adult Female; **Taxon:** scientificName: Zelus vespiformis; family: Reduviidae; genus: Zelus; scientificNameAuthorship: Hart, 1987; **Location:** country: COLOMBIA; stateProvince: unknown; locality: unknown; decimalLatitude: 5.02584; decimalLongitude: -74.02986; georeferenceSources: Google Earth; **Identification:** identifiedBy: G. Zhang; dateIdentified: 2012; **Event:** eventDate: no date provided; **Record Level:** institutionCode: USNM
- **Type status:** Paratype. **Occurrence:** catalogNumber: UCR_ENT 00009408; recordedBy: B. Losada; sex: Adult Male; **Taxon:** scientificName: Zelus vespiformis; family: Reduviidae; genus: Zelus; scientificNameAuthorship: Hart, 1987; **Location:** country: COLOMBIA; stateProvince: Valle del Cauca; locality: Palmira; decimalLatitude: 3.5364; decimalLongitude: -76.3036; georeferenceSources: Gazetteer; **Identification:** identifiedBy: G. Zhang; dateIdentified: 2012; **Event:** eventDate: 1942-01-01; **Record Level:** institutionCode: USNM
- **Type status:** Paratype. **Occurrence:** catalogNumber: UCR_ENT 00009414; recordedBy: B. Losada; sex: Adult Female; **Taxon:** scientificName: Zelus vespiformis; family: Reduviidae; genus: Zelus; scientificNameAuthorship: Hart, 1987; **Location:** country: COLOMBIA; stateProvince: Valle del Cauca; locality: Cali; decimalLatitude: 3.4372; decimalLongitude: -76.5225; georeferenceSources: Gazetteer; **Identification:** identifiedBy: G. Zhang; dateIdentified: 2012; **Event:** eventDate: 1943-07-01; **Record Level:** institutionCode: USNM
- **Type status:** Paratype. **Occurrence:** catalogNumber: UCR_ENT 00009420; recordedBy: M. Benavides; sex: Adult Female; **Taxon:** scientificName: Zelus vespiformis; family: Reduviidae; genus: Zelus; scientificNameAuthorship: Hart, 1987; **Location:** country: COLOMBIA; stateProvince: Valle del Cauca; locality: Palmira; decimalLatitude: 3.5364; decimalLongitude: -76.3036; georeferenceSources: Gazetteer; **Identification:** identifiedBy: G. Zhang; dateIdentified: 2012; **Event:** eventDate: 1955-07-17; **Record Level:** institutionCode: USNM
- **Type status:** Paratype. **Occurrence:** catalogNumber: UCR_ENT 00017878; recordedBy: Severo Quintero; sex: Adult Male; **Taxon:** scientificName: Zelus vespiformis; family: Reduviidae; genus: Zelus; scientificNameAuthorship: Hart, 1987; **Location:** country: COLOMBIA; stateProvince: Valle del Cauca; locality: Cali District; verbatimElevation: 994 m; **Identification:** identifiedBy: G. Zhang; dateIdentified: 2012; **Event:** eventDate: 1935-02-07; **Record Level:** institutionCode: AMNH
- **Type status:** Paratype. **Occurrence:** catalogNumber: UCR_ENT 00017882; recordedBy: Severo Quintero; sex: Adult Female; **Taxon:** scientificName: Zelus vespiformis; family: Reduviidae; genus: Zelus; scientificNameAuthorship: Hart, 1987; **Location:** country: COLOMBIA; stateProvince: Valle del Cauca; locality: Cali District; verbatimElevation: 994 m; **Identification:** identifiedBy: G. Zhang; dateIdentified: 2012; **Event:** eventDate: 1935-02-07; **Record Level:** institutionCode: AMNH
- **Type status:** Paratype. **Occurrence:** catalogNumber: UCR_ENT 00009402; recordedBy: Salazar; sex: Adult Male; **Taxon:** scientificName: Zelus vespiformis; family: Reduviidae; genus: Zelus; scientificNameAuthorship: Hart, 1987; **Location:** country: EL SALVADOR; stateProvince: La Libertad; locality: Santa Tecla; decimalLatitude: 13.6769; decimalLongitude: -89.2797; **Identification:** identifiedBy: G. Zhang; dateIdentified: 2012; **Event:** eventDate: 1953-01-24; **Record Level:** institutionCode: USNM
- **Type status:** Paratype. **Occurrence:** catalogNumber: UCR_ENT 00009400; recordedBy: J. Zelek; sex: Adult Male; **Taxon:** scientificName: Zelus vespiformis; family: Reduviidae; genus: Zelus; scientificNameAuthorship: Hart, 1987; **Location:** country: PANAMA; stateProvince: Canal Zone; locality: Barro Colorado Island; decimalLatitude: 9.15562; decimalLongitude: -79.84895; georeferenceSources: Google Earth; **Identification:** identifiedBy: G. Zhang; dateIdentified: 2012; **Event:** eventDate: 1946-12-01 to 1947-02-01; **Record Level:** institutionCode: USNM
- **Type status:** Paratype. **Occurrence:** catalogNumber: UCR_ENT 00009401; recordedBy: S. W. Frost; sex: Adult Male; **Taxon:** scientificName: Zelus vespiformis; family: Reduviidae; genus: Zelus; scientificNameAuthorship: Hart, 1987; **Location:** country: PANAMA; stateProvince: Canal Zone; locality: Barro Colorado Island; decimalLatitude: 9.15562; decimalLongitude: -79.84895; georeferenceSources: Google Earth; **Identification:** identifiedBy: G. Zhang; dateIdentified: 2012; **Event:** eventDate: 1929-04-03; **Record Level:** institutionCode: USNM
- **Type status:** Paratype. **Occurrence:** catalogNumber: UCR_ENT 00009423; recordedBy: F.J. Simmonds; sex: Adult Female; **Taxon:** scientificName: Zelus vespiformis; family: Reduviidae; genus: Zelus; scientificNameAuthorship: Hart, 1987; **Location:** country: TRINIDAD AND TOBAGO; stateProvince: Trinidad; locality: Saint Joseph; **Identification:** identifiedBy: G. Zhang; dateIdentified: 2012; **Event:** eventDate: 1953-03-06; **Record Level:** institutionCode: USNM
- **Type status:** Paratype. **Occurrence:** catalogNumber: UCR_ENT 00009406; recordedBy: C.H. Ballou; sex: Adult Male; **Taxon:** scientificName: Zelus vespiformis; family: Reduviidae; genus: Zelus; scientificNameAuthorship: Hart, 1987; **Location:** country: VENEZUELA; stateProvince: Distrito Federal; locality: La Florida; decimalLatitude: 10.50171; decimalLongitude: -66.86585; georeferenceSources: Google Earth; **Identification:** identifiedBy: G. Zhang; dateIdentified: 2012; **Event:** eventDate: 1938-04-16; **Record Level:** institutionCode: USNM
- **Type status:** Paratype. **Occurrence:** catalogNumber: UCR_ENT 00009407; recordedBy: Anthonias; sex: Adult Male; **Taxon:** scientificName: Zelus vespiformis; family: Reduviidae; genus: Zelus; scientificNameAuthorship: Hart, 1987; **Location:** country: VENEZUELA; stateProvince: Distrito Federal; locality: Caracas; decimalLatitude: 10.5; decimalLongitude: -66.9; georeferenceSources: GeoLocate Software; **Identification:** identifiedBy: G. Zhang; dateIdentified: 2012; **Event:** eventDate: 1905-04-21; **Record Level:** institutionCode: USNM
- **Type status:** Paratype. **Occurrence:** catalogNumber: UCR_ENT 00009417; recordedBy: C.H. Ballou; sex: Adult Female; **Taxon:** scientificName: Zelus vespiformis; family: Reduviidae; genus: Zelus; scientificNameAuthorship: Hart, 1987; **Location:** country: VENEZUELA; stateProvince: Distrito Federal; locality: Florida, Caracas; decimalLatitude: 10.50395; decimalLongitude: -66.87425; georeferenceSources: Google Earth; **Identification:** identifiedBy: G. Zhang; dateIdentified: 2012; **Event:** eventDate: 1938-11-08; **Record Level:** institutionCode: USNM
- **Type status:** Paratype. **Occurrence:** catalogNumber: UCR_ENT 00009421; recordedBy: Anthonias; sex: Adult Female; **Taxon:** scientificName: Zelus vespiformis; family: Reduviidae; genus: Zelus; scientificNameAuthorship: Hart, 1987; **Location:** country: VENEZUELA; stateProvince: Distrito Federal; locality: Caracas; decimalLatitude: 10.5; decimalLongitude: -66.9; georeferenceSources: GeoLocate Software; **Identification:** identifiedBy: G. Zhang; dateIdentified: 2012; **Event:** eventDate: 1905-04-21; **Record Level:** institutionCode: USNM
- **Type status:** Paratype. **Occurrence:** catalogNumber: UCR_ENT 00071171; recordedBy: P. Cor. Vogl; sex: Adult Female; **Taxon:** scientificName: Zelus vespiformis; family: Reduviidae; genus: Zelus; scientificNameAuthorship: Hart, 1987; **Location:** country: VENEZUELA; stateProvince: Distrito Federal; locality: Berg Avila, Caracas; decimalLatitude: 10.48801; decimalLongitude: -66.87919; georeferenceSources: Gazetteer; **Identification:** identifiedBy: G. Zhang; dateIdentified: 2012; **Event:** eventDate: no date provided; **Record Level:** institutionCode: TAMU
- **Type status:** Paratype. **Occurrence:** catalogNumber: UCR_ENT 00071172; recordedBy: P. Cor. Vogl; sex: Adult Female; **Taxon:** scientificName: Zelus vespiformis; family: Reduviidae; genus: Zelus; scientificNameAuthorship: Hart, 1987; **Location:** country: VENEZUELA; stateProvince: Distrito Federal; locality: Berg Avila, Caracas; decimalLatitude: 10.48801; decimalLongitude: -66.87919; georeferenceSources: Gazetteer; **Identification:** identifiedBy: G. Zhang; dateIdentified: 2012; **Event:** eventDate: no date provided; **Record Level:** institutionCode: TAMU
- **Type status:** Paratype. **Occurrence:** catalogNumber: UCR_ENT 00009424; recordedBy: C.H. Ballou; sex: Adult Female; **Taxon:** scientificName: Zelus vespiformis; family: Reduviidae; genus: Zelus; scientificNameAuthorship: Hart, 1987; **Location:** country: VENEZUELA; stateProvince: unknown; locality: El Valle; **Identification:** identifiedBy: G. Zhang; dateIdentified: 2012; **Event:** eventDate: 1939-02-01; **Record Level:** institutionCode: USNM
- **Type status:** Paratype. **Occurrence:** catalogNumber: UCR_ENT 00009410; recordedBy: H.E. Hox; sex: Adult Female; **Taxon:** scientificName: Zelus vespiformis; family: Reduviidae; genus: Zelus; scientificNameAuthorship: Hart, 1987; **Location:** country: VENEZUELA; stateProvince: Vargas; locality: Mama, near La Guayra; decimalLatitude: 10.6; decimalLongitude: -66.9333; georeferenceSources: Gazetteer; **Identification:** identifiedBy: G. Zhang; dateIdentified: 2012; **Event:** eventDate: 1927-02-01; **Record Level:** institutionCode: USNM
- **Type status:** Paratype. **Occurrence:** catalogNumber: UCR_ENT 00009419; recordedBy: H.E. Hox; sex: Adult Female; **Taxon:** scientificName: Zelus vespiformis; family: Reduviidae; genus: Zelus; scientificNameAuthorship: Hart, 1987; **Location:** country: VENEZUELA; stateProvince: Vargas; locality: Mama, near La Guayra; decimalLatitude: 10.6; decimalLongitude: -66.9333; georeferenceSources: Gazetteer; **Identification:** identifiedBy: G. Zhang; dateIdentified: 2012; **Event:** eventDate: 1927-02-01; **Record Level:** institutionCode: USNM
- **Type status:** Paratype. **Occurrence:** catalogNumber: UCR_ENT 00009403; recordedBy: Tejera; sex: Adult Male; **Taxon:** scientificName: Zelus vespiformis; family: Reduviidae; genus: Zelus; scientificNameAuthorship: Hart, 1987; **Location:** country: VENEZUELA; stateProvince: Zulia; locality: Perija; decimalLatitude: 9.74202; decimalLongitude: -72.97539; georeferenceSources: Google Earth; **Identification:** identifiedBy: G. Zhang; dateIdentified: 2012; **Event:** eventDate: 1918-01-18; **Record Level:** institutionCode: USNM
- **Type status:** Paratype. **Occurrence:** catalogNumber: UCR_ENT 00009404; recordedBy: Tejera; sex: Adult Male; **Taxon:** scientificName: Zelus vespiformis; family: Reduviidae; genus: Zelus; scientificNameAuthorship: Hart, 1987; **Location:** country: VENEZUELA; stateProvince: Zulia; locality: Perija; decimalLatitude: 9.74202; decimalLongitude: -72.97539; georeferenceSources: Google Earth; **Identification:** identifiedBy: G. Zhang; dateIdentified: 2012; **Event:** eventDate: 1918-01-18; **Record Level:** institutionCode: USNM
- **Type status:** Paratype. **Occurrence:** catalogNumber: UCR_ENT 00009418; recordedBy: Tejera; sex: Adult Female; **Taxon:** scientificName: Zelus vespiformis; family: Reduviidae; genus: Zelus; scientificNameAuthorship: Hart, 1987; **Location:** country: VENEZUELA; stateProvince: Zulia; locality: Maracaibo; decimalLatitude: 10.6317; decimalLongitude: -71.6406; georeferenceSources: Gazetteer; **Identification:** identifiedBy: G. Zhang; dateIdentified: 2012; **Event:** eventDate: no date provided; **Record Level:** institutionCode: USNM
- **Type status:** Paratype. **Occurrence:** catalogNumber: UCR_ENT 00009422; recordedBy: Tejera; sex: Adult Female; **Taxon:** scientificName: Zelus vespiformis; family: Reduviidae; genus: Zelus; scientificNameAuthorship: Hart, 1987; **Location:** country: VENEZUELA; stateProvince: Zulia; locality: Perija; decimalLatitude: 9.74202; decimalLongitude: -72.97539; georeferenceSources: Google Earth; **Identification:** identifiedBy: G. Zhang; dateIdentified: 2012; **Event:** eventDate: 1918-01-18; **Record Level:** institutionCode: USNM

#### Description

Figs 211, 212, 213

***Male***: (Fig. 211a, b) Medium-sized, total length 11.52–13.73 mm (mean 12.92 mm, Suppl. material 2); very slender. **COLORATION**: Color pattern wasp-like, yellow and brown areas alternating. Head and anterior pronotal lobe dark brown to brownish-black. Posterior pronotal lobe yellow to brownish orange; meso-pleuron yellow to brownish orange, posterior part sometimes dark brown; meta-pleuron dark brown. Legs yellow, with brown bands. Abdomen yellow to orangish-brown, terminal segments (7, 8 and pygophore) dark brown to black. **VESTITURE**: Densely setose. Dorsal vestiture primarily consisting of short, erect, spine-like setae; head ventrally with sparse, fine, short, erect or recumbent setae. Abdominal venter with short to long, fine erect setae. Sparse, short, erect setae on apex of paramere and posteroventral rim of pygophore. Head dorsally with short, dense, spine-like setae; ventral setae recumbent. Scutellum with short, dense, recumbent to semierect setae, sometimes with spine-like setae. Abdomen with short, adpressed setae interspersed with unevenly lengthed, short to long, semierect or erect setae. Pygophore with sparse setae. **STRUCTURE: Head**: Cylindrical. Postocular lobe in dorsal view distinctly narrowing through anterior 2/3, posterior 1/3 constant, tube-like. Eye moderately sized; dorsal margin removed from postocular transverse groove, ventral margin attaining ventral surface of head in lateral view. *Labium*: I: II: III = 1: 2.4: 0.6. Basiflagellomere diameter larger than that of pedicel. **Thorax**: Anterolateral angle rounded, without projection; medial longitudinal sulcus shallow near collar, deepening posteriorly. Posterior pronotal lobe with smooth surface; disc distinctly elevated above humeral angle; humeral angle rounded, without projection. Scutellum long; apex angulate, not projected. *Legs*: Very slender. *Hemelytron*: Greatly surpassing apex of abdomen by about 3x length of abdominal segment seven; quadrate cell large and broad; Cu and M of cubital cell subparallel. **GENITALIA**: (Fig. 212) *Pygophore*: Ovoid; mid-lateral fold adjacent to paramere insertion. Medial process cylindrical; very slender; short, about third length of exposed portion of parameres; posteriorly directed, in less than forty-five degree with body axis; nearly straight; basally without protrusion; apex in posterior view blunt. *Paramere*: Cylindrical; moderately long, achieving apex of medial process; directed posteriad; slightly curved ventrad; apical part very slightly enlarged. *Phallus*: Dorsal phallothecal sclerite somewhat squarish; flat, laterally indistinctly angulate; apex truncate, medially emarginate; posterior margin of foramen deeply concave. Struts attached to dorsal phallothecal sclerite; apically separate, connected by bridge; basally mostly separate, moderately fused. Basal plate arm moderately robust; separate; converging; in lateral view nearly straight, very slightly curved; bridge short; extension of basal plate expanded onto arm.

***Female***: Similar to male, except for the following. Larger than male, total length 16.48–18.29 mm (mean 17.47 mm, Suppl. material 2). Some specimens almost entirely dark brown, only with medial part of or entire posterior pronotal lobe, mesopleuron and mesosternum brownish orange or orange.

#### Diagnosis

The generally wasp-like coloration pattern can separate this species from most other species of the *Zelus*, but not from a few species that may also display a wasp-like appearance. Males can be recognized by the rather slender and short medial process, it being much shorter than that in *Zelus errans*, the only species that may cause confusion.

#### Distribution

**Figure 213.**
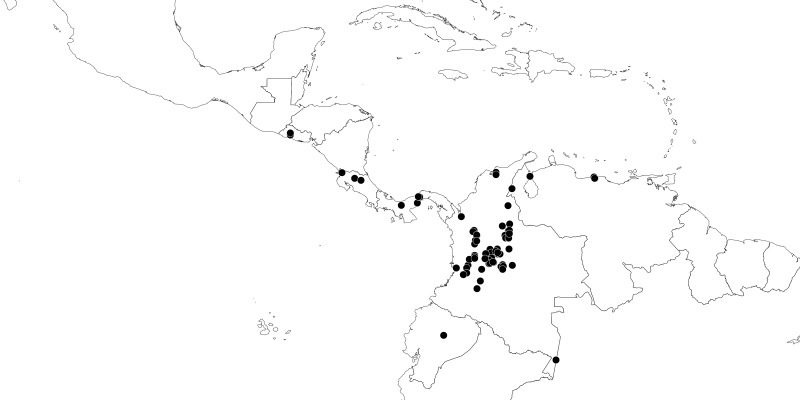
*Zelus vespiformis* Hart, 1987, specimen record map

Central America and Northern South America (Fig. 213). Countries with records: Colombia, Costa Rica, Ecuador, El Salvador, Panama, Trinidad and Tobago and Venezuela.

### Zelus xouthos

Zhang & Hart sp. n.

urn:lsid:zoobank.org:act:046EF778-019D-4D93-80C7-555D7503FC8E

#### Materials

- **Type status:** Holotype. **Occurrence:** catalogNumber: UCR_ENT 00008003; recordedBy: W. M. Schaus; sex: Adult Male; **Taxon:** scientificName: Zelus xouthos; family: Reduviidae; genus: Zelus; scientificNameAuthorship: Zhang and Hart, 2016; **Location:** country: GUATEMALA; stateProvince: Izabal; locality: Cayuga; verbatimElevation: 25 m; decimalLatitude: 15.5333; decimalLongitude: -88.7; georeferenceSources: Gazetteer; **Identification:** identifiedBy: G. Zhang; dateIdentified: 2013; **Event:** eventDate: 1915-05-01; **Record Level:** institutionCode: USNM
- **Type status:** Paratype. **Occurrence:** catalogNumber: UCR_ENT 00029352; occurrenceRemarks: Abdomen missing. Designated as allotype by Hart, unpublished. This status is not used in Zhang et al., which formally publishes this species.; recordedBy: T. H. Hubbell; sex: Adult sex unknown; **Taxon:** scientificName: Zelus xouthos; family: Reduviidae; genus: Zelus; scientificNameAuthorship: Zhang and Hart, 2016; **Location:** country: GUATEMALA; stateProvince: Peten; locality: Tikal; decimalLatitude: 17.225; decimalLongitude: -89.6133; georeferenceSources: Gazetteer; **Identification:** identifiedBy: G. Zhang; dateIdentified: 2013; **Event:** eventDate: 1956-05-12; **Record Level:** institutionCode: USNM

#### Description

Figs 214, 215, 216

***Male***: (Fig. 214) Medium-sized, total length 12.59 mm (n=1, Suppl. material 2); slender. **COLORATION**: Entire surface brown, dorsum of head, posterior pronotal lobe, corium, clavus, apices of femora, parts of tibiae somewhat reddish. **VESTITURE**: Sparsely setose. Short, recumbent setae on dorsal surface of head, long recumbent setae on ventral surface; short, erect, spine-like setae on dorsum, denser on anterior lobe; few moderately long, erect, fine setae on ventral surface. Pronotum with sparse, recumbent setae and short, erect setae over dorsal surface; denser, moderately long recumbent setae on lateral surface and pleura, intermixed with semi-erect or erect setae; scutellum with sparse, semi-erect and recumbent setae. Legs with sparse setation on femora and moderately dense setation on tibiae. Corium and clavus with short, recumbent setae. Abdomen with moderately dense, short recumbent setae, intermixed with sparse, short to long, erect setae. **STRUCTURE: Head**: Cylindrical, L/W = 2.43. Postocular lobe long; in dorsal view distinctly narrowing through anterior 2/3, posterior 1/3 constant, tube-like. Eye prominent; lateral margin much wider than postocular lobe; dorsal margin attaining postocular transverse groove, ventral margin removed from ventral surface of head in lateral view. *Labium*: I: II: III = 1: 2.1: 0.4. Basiflagellomere diameter slightly larger than that of pedicel. **Thorax**: Anterolateral angle with inconspicuous subtuberculate projection; medial longitudinal sulcus evident throughout, deepening posteriorly. Posterior pronotal lobe with rugulose surface; disc about same level as humeral angle; humeral angle armed, with spinous processes. Scutellum moderately long; apex angulate. *Legs*: Very slender, femoral diameters subequal. *Hemelytron*: Surpassing apex of abdomen by about length of abdominal segment seven; quadrate cell small and slender; Cu and M of cubital cell subparallel. **GENITALIA**: (Fig. 215) *Pygophore*: Ovoid; not expanded laterally in dorsal view. Medial process cylindrical; slender; moderately long, much longer than paramere; minute spicules on posterior surface; semi-erect; basally slightly curved; apex in posterior view acute, with small hooklike projection. *Paramere*: Cylindrical; moderately long, slightly exceeding medial process; directed posteriad; apical part appearing somewhat truncate dorsally. *Phallus*: Dorsal phallothecal sclerite elongated; medial, longitudinal sulcus on dorsal surface; apical portion of phallothecal sclerite gradually tapering, distinctly keeled medially, laterally flat, not forming angle; apex acute; posterior margin of foramen broadly concave, accuminate in middle. Basal plate arm slender; separate, briefly touching; converging; in lateral view slightly curved; bridge short; extension of basal plate expanded laterally onto arm, covering more than 1/2 of arm.

***Female***: Similar to male, except for the following. Larger than male, total length 14.69 mm (n=1, Suppl. material 2).

#### Diagnosis

Recognized by the following combination of characters: the slender body and delicate legs, the dorsal coloration somewhat reddish; the humeral angle elevated nearly to the level of and nearly continuous with pronotal disc; the paramere uniquely shaped, long, exceeding medial process, apex somewhat obliquely truncate; and the medial process apically not compressed.

#### Etymology

From Greek *xutho*, meaning yellowish-brown, referring to the yellowish coloration.

#### Distribution

**Figure 216.**
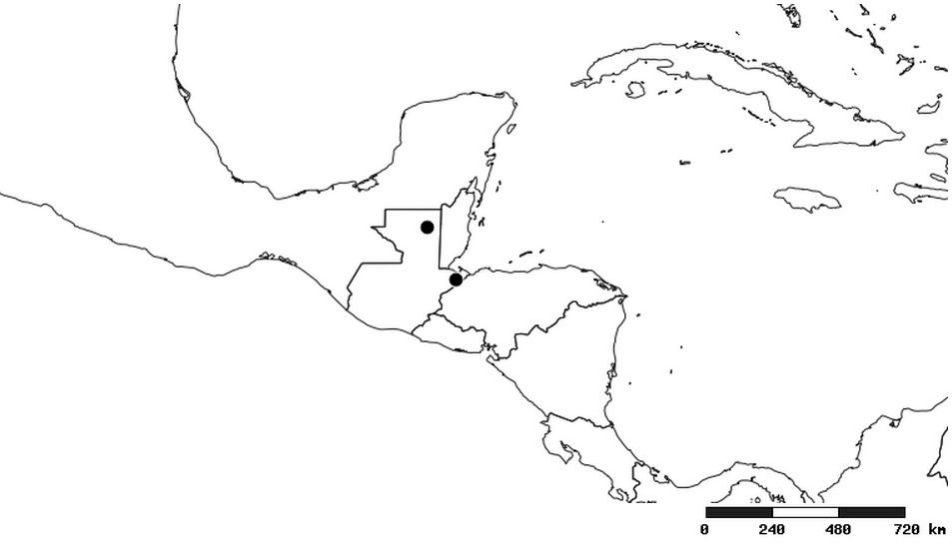
*Zelus xouthos* Zhang & Hart, sp. n., specimen record map

Central America (Fig. 216). Known only from Guatemala.

#### Taxon discussion

This species appears to be the most divergent of the *Zelus panamensis* species group. Although three other species within the same geographical range of this species, *Z. janus, Z. exsanguis*, *Z. ambulans*, also have an elevated humeral angle, their much greater sizes and features of the male genitalia should eliminate any confusion in recognizing *Z. xouthos*.

### Zelus zayasi

Bruner & Barber, 1937

Zelus zayasi Bruner and Barber, 1937, p. 186–188, orig. descr. and fig. (subgenus *Zelus*); Wygodzinsky, 1949a, p. 50, checklist; Zayas, 1960, p. 125, 126, note; Alayo, 1967, p. 36, 37, key and list.; Hart, 1987, p. 296, key and note; Maldonado, 1990, p. 332, cat.

#### Materials

- **Type status:** Holotype. **Occurrence:** catalogNumber: UCR_ENT 00007933; occurrenceRemarks: bears label 'Type No. 51844 U.S.N.M' and 'E.E.A. Cuba Ento No.10644'; recordedBy: F. de Zayas; sex: Adult Female; otherCatalogNumbers: USNM Type No. 51844; **Taxon:** scientificName: Zelus zayasi; family: Reduviidae; genus: Zelus; scientificNameAuthorship: Bruner & Barber, 1937; **Location:** country: CUBA; stateProvince: Guantanamo; locality: El Yunque, Baracoa; decimalLatitude: 20.35237; decimalLongitude: -74.57356; georeferenceSources: Google Earth; **Identification:** identifiedBy: Burner & Barber; dateIdentified: 1937; **Event:** eventDate: 1935-07-10; **Record Level:** institutionCode: USNM

#### Description

Figs 217, 218

***Male***: unknown.

***Female***: (Fig. 217) Medium-sized, total length 12.39 mm (n=1); very slender. **COLORATION**: Dorsal surface brown, lighter on lateral surfaces and abdomen. **VESTITURE**: Moderately setose. Head with erect setae. Anterior pronotal lobe with sparse, recumbent and erect setae; posterior pronotal lobe with scattered, short, erect and recumbent setae. Abdomen with short, erect setae. **STRUCTURE: Head**: Cylindrical, L/W = 2.29. Postocular lobe moderately long; in dorsal view anteriorly gradually narrowing, posterior portion constant, slightly narrower. Eye smallish; lateral margin only slightly wider than postocular lobe; dorsal and ventral margins removed from surfaces of head. *Labium*: I: II: III = 1: 1.6: 0.3. **Thorax**: Anterolateral angle bearing small projection; medial longitudinal sulcus evident only on posterior 1/2, deepening anterior to transverse sulcus of pronotum. Posterior pronotal lobe with rugulose surface; disc distinctly elevated above humeral angle; humeral angle rounded, without projection. Scutellum moderately long; apex angulate. *Legs*: Slender. *Hemelytron*: Attaining apex of abdomen.

#### Diagnosis

The extremely slender body form separates this species from most other species of *Zelus*. Can be distinguished from *Z. puertoricensis* by the lack of conspicuous lateral process on the humeral angle and from *Z. subimpressus* by the parallel dorsal and ventral surfaces on the anterior 2/3 of the postocular lobe. The dorsal surface in *Z. subimpressus* is sloping.

#### Distribution

**Figure 218.**
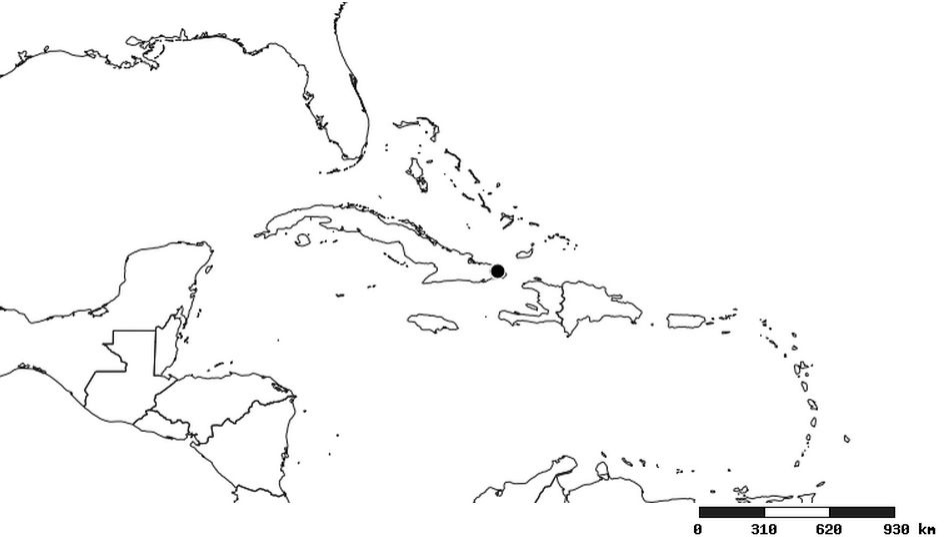
*Zelus zayasi* Bruner and Barber, 1937, specimen record map

The Caribbean. Known only from Cuba (Fig. 218).

#### Taxon discussion

Based on the description and figure of *Z. bruneri*, it is possible that *Z. bruneri* is the male of *Z. zayasi*. Because the specimens of *Z. bruneri* were not physically examined, we restrain from making this association and a formal synonymy between these two species.

## Identification Keys

### Key to species groups of *Zelus* (males only)

**Table:** 

1	Apex of medial process without ventrally or posteriorly directed hook, projection or fold (Figs 2, 3, 5, 11).	2
–	Apex of medial process with ventrally or posteriorly directed hook, projection or fold (Figs 4, 6, 7, 8, 10, 12, 14).	5
2	Medial process triangular, broad at base, if slender, paramere apically enlarged; anteroposteriorly compressed (Figs 2, 3).	3
–	Medial process with lateral margins subparallel or narrowly triangular; base not conspicuously broadened; laterally compressed or blade-like throughout or in apical 1/2 (Figs 5, 11).	4
3	Medial process basally nearly continuous with rest of pygophore, base not protruding posteriorly. Paramere apically not enlarged or slightly enlarged (Fig. 2).	*Zelus tetracanthus* species group
–	Medial process basally readily separable from rest of pygophore; base prominent and protruding posteriorly. Paramere expanded apically in some species (Fig. 3).	*Zelus luridus* species group
4	Medial process slender, laterally compressed, dorsally oriented, at about forty-five degree angle to body axis (except in *Z. grassans*) (Fig. 5). Paramere cylindrical, apex not expanded. Anterior pronotal lobe dorsally with relatively fine setae or nearly bare. Humeral angle with minute projection.	*Zelus nugax* species group
–	Medial process stout, posteriorly oriented, nearly parallel to body axis (Fig. 11). Paramere robust, not reaching apex of medial process. Anterior pronotal lobe dorsally with short, erect spine-like setae. Humeral angle rounded, unarmed.	*Zelus vagans* species group
5	Apex of medial process strongly curved ventrally to form hook-like process or lip-like fold (Figs 4, 7).	6
–	Apex of medial process not curved or weakly curved ventrally (Figs 6, 8, 10, 12, 14).	7
6	Medial process laterally compressed; apex strongly curved ventrally, clearly hook-like. Paramere apically expanded, slightly curved dorsally (Fig. 7).	*Zelus renardii* species group
–	Medial process cylindrical or broad at base; not laterally compressed; apex curved, lip-like rather than hook-like (Fig. 4). Paramere apically not expanded, strongly curved dorsally.	*Zelus mimus* species group
7	Apex of medial process with distinct, acute, hook-like process or ridge-like elevation (Figs 12, 14).	8
–	Apex of medial process with indistinct process or weak curvature, not forming sharp projection (Figs 6, 8, 10).	9
8	Medial process dorsally directed and apical 1/3 not curving posteriorly; usually anteroposteriorly compressed; base broad in some species; ridge-like elevation on posterior surface, usually extending ventrally and elevated, removed from apex and appearing as pair of projections in some species (Fig. 14). Paramere usually bulbous in apical 1/2 or 2/3, curved medially.	*Zelus erythrocephalus* species group
–	Medial process long, slender, stalk-like; somewhat laterally compressed; lateral margins subparallel, base not broadened or slightly broadened; gradually curving and directed posteriorly in apical 1/3 or 1/2; apex with short, acute, hook-like process or sharp fold (Fig. 14). Paramere cylindrical, straight or curved in various ways, slightly swollen apically, not bulbous.	*Zelus panamensis* species group
9	Medial process extremely slender, usually long (Fig. 10). Paramere exceeds apex of medial process. Humeral angle rounded, unarmed.	*Zelus longipes* species group
–	Medial process stout, relatively short (Figs 6, 8). Paramere removed from or reaching apex of medial process. Humeral angle armed with projection.	10
10	Large (>13 mm), robust. Apex of medial process with pair of minute projections (Fig. 8). Humeral angle with prominent dentate process (Mexico, C.A. and S.A.).	*Zelus armillatus* species group
–	Medium-sized (<12 mm), elongate. Apex of medial process with inconspicuous folding (Fig. 6). Humeral angle with small projection (Caribbean).	*Zelus puertoricensis* species group

### Key to males of the *Zelus tetracanthus* species group

**Table:** 

1	Posterior pronotal lobe disc bears pair of tubercles.	2
–	Posterior pronotal lobe disc without tubercles.	3
2	Smallish, 7.8-10.1 mm. Legs delicate. Head and parts of pronotum orangish-brown. Paramere clearly exceeds medial process (Fig. 142a, b).	*Zelus minutus* (Fig. 141)
–	Length 11.3-13.7 mm. Greyish-brown to brownish-black. Paramere removed from or reaching medial process, never exceeding (Fig. 191a, b).	*Zelus tetracanthus* (Fig. 190a)
3	Entire surface reddish-brown (Fig. 170). Length 11.5-12.4 mm. Membrane of hemelytron not translucent. Quadrate cell relatively broad.	*Zelus rosulentus*
–	Pale brown (Fig. 161a, c). Length 8.6-10.1 mm. Membrane translucent. Quadrate cell very slender.	*Zelus prolixus*

### Key to males of the *Zelus luridus* species group

**Table:** 

1	Humeral angle raised to level of, and usually continuous with, disc of posterior pronotal lobe.	2
–	Humeral angle clearly below level of, and not continuous with, disc.	3
2	Medial longitudinal sulcus of anterior pronotal lobe with dark brown area near posterior margin. Femora usually with dark apical bands. Parameres in fresh or relaxed specimens not achieving apex of medial process (Fig. 23a) (Mexico and C.A.).	*Zelus ambulans* (Fig. 22a, b)
–	Medial longitudinal sulcus of anterior pronotal lobe same color as surrounding area at posterior margin, any dark areas present very small. Femora apically reddish-brown, not forming distinct bands. Paramere in fresh or relaxed specimens achieving or surpassing apex of medial process (Fig. 72a, b) (Mexico and C.A.).	*Zelus exsanguis* (Fig. 71a, b)
3	Paramere spatulate apically, compressed; apical portion about 2x width of cylindrical basal portion (Fig. 180a). Medial process slender, stalk-like, laterally compressed, base somewhat humped, not conspicuously protruding (C.A.).	*Zelus spatulosus* (Fig. 179)
–	Paramere slightly expanded apically, apical portion less than 2x width of base. Medial process triangular, base broad and protruding posteriorly.	4
4	Eyes prominent, extending below ventral surface of head.	*Zelus grandoculus* (Fig. 88)
–	Eyes moderate, not extending below ventral surface of head.	5
5	Anterior pronotal lobe usually 1/2 or greater of length of posterior pronotal lobe; if slightly less than 1/2, profemoral length less than 20x profemoral diameter. Protrusion at base of medial process moderate (Fig. 131a, b). (Canada, U.S. and Mexico).	*Zelus luridus* (Fig. 130a, b)
–	Anterior pronotal lobe less than 1/2 length of posterior pronotal lobe; profemoral length 20x or more profemoral diameter (C.A.). Protrusion at base of medial process more prominent (Fig. 29a).	*Zelus antiguensis* (Fig. 28a, b)

### Key to males of the *Zelus nugax* species group 

**Table:** 

1	Paramere curved strongly medially and recurved apically (Fig. 92a). Medial process posteriorly directed. Abdomen usually with alternating yellow and black bands (Mexico and C.A.).	*Zelus grassans* (Fig. 91a, b, c)
–	Paramere straight or only slightly curved. Medial process dorsally directed. Brown, abdomen without banding.	2
2	Medial process exhibiting pronounced curvature posteriorly about 1/3 of distance from base, then recurved toward dorsum about 3/4 distance from base.	3
–	Medial process blade-like, straight; any curvature toward posterior weak.	4
3	Labial segment I and coxae medium brown to brownish-black. Paramere nearly straight, any curvature weak (Fig. 98a) (northern S.A.).	*Zelus impar* (Fig. 97)
–	Labium and coxae light yellowish-brown. Paramere medially curved ventrally (Fig. 95a) (S.A.).	*Zelus illotus* (Fig. 94a, b)
4	Basal plate arms fused. Pygophore as in Fig. 157a.	*Zelus pedestris* (Fig. 156a, b)
–	Basal plate arms not fused, separate in specimens from S.A., tendency to fuse in C.A. and Mexico. Pygophore as in Fig. 148a (Mexico, C.A. and S.A.).	*Zelus nugax* (Fig. 147a, b)

### Key to males of the *Zelus vagans* species group

**Table:** 

1	Dorsal surface entirely black. Abdomen brown, yellow or red.	2
–	Dorsal surface with orange and dark brown areas on pronotum or hemelytron. Abdomen typically orange or reddish with terminal segments dark brown; variations exist.	4
2	Abdomen brown, not brightly yellow or red. Cells of membrane conspicuously less pigmented than veins. Postocular lobe with longitudinal lateral patch of whitish recumbent setae.	*Zelus aithaleos* (Fig. 16)
–	Abdomen brightly yellow or red. Cells of membrane same color as veins. Postocular lobe without lateral whitish setae.	3
3	Abdomen red. Paramere medially curved ventrad (Fig. 55a).	*Zelus championi* (Fig. 54)
–	Abdomen yellow. Paramere slightly bent near base, straight in remaining part (Fig. 80a).	*Zelus fuliginatus* (Fig. 79a, b)
4	Dorsal surface of pronotum medially dark brown, laterally orange-brown. Paramere removed from apex of medial process, medially curved ventrad (Fig. 203a).	*Zelus vagans* (Fig. 202)
–	Coloration of pronotum not as described above; anterior lobe dark brown and posterior lobe orange-brown in some specimens. Paramere straight, apex oblique (Fig. 86a).	*Zelus gracilipes* (Fig. 85a, b)

### Key to males of the *Zelus renardii* species group

**Table:** 

1	Relatively robust. Humeral angle of pronotum widened; body length 5.5x or less of width through humeral angles. Apical hook of paramere more prominent (Fig. 168a) (Western and southwestern US, Mexico and northern C.A.).	*Zelus renardii* (Fig. 167a, b)
–	Very slender. Humeral angle not conspicuously widened; body length greater than 5.5x width through humeral angles. Apical hook of paramere less prominent (Fig. 49a, b) (eastern and southern U.S., Mexico, C.A. and northwestern S.A.).	*Zelus cervicalis* (Fig. 48a, b, c)

### Key to males of the *Zelus mimus* species group

**Table:** 

1	Medial process long and slender (Fig. 139a).	*Zelus mimus* (Fig. 138a, b)
–	Medial process short and broad (Fig. 101a).	*Zelus inconstans* (Fig. 100a, b)

### Key to males of the *Zelus erythrocephalus* species group

**Table:** 

1	Head reddish-brown. Dorsal surface of pronotum and corium brownish-black. Membrane of hemelytron pale brown or blue or green iridescent. Legs brownish-black, without banding or with inconspicuous bands.	2
–	Head brown, yellow, or black, sometimes with stripes. Dorsal surface uniformly brown or with light-colored areas, mainly on pronotum or corium. Membrane brown, not iridescent. Legs with or without banding.	4
2	Medial process very slender, diameter less than that of paramere (Fig. 69a).	*Zelus erythrocephalus* (Fig. 68a, b)
–	Medial process broad, diameter greater than that of paramere.	3
3	Medial process twice as broad as paramere (Fig. 154a). Paramere distinctly recurved.	*Zelus paracephalus* (Fig. 153a, b)
–	Medial process only slightly broader than paramere. Paramere bent ventrally near base, not recurved (Fig. 177a).	*Zelus russulumus* (Fig. 176a, b)
4	Medial process very broad; lateral margins parallel or converging basally.	5
–	Medial process triangular; lateral margins converging apically, base broader than apex.	6
5	Length less than 11.4 mm. Medial process apex broader than base, apex acute, medially not notched (Fig. 116a). Paramere not greatly curved ventrad. Pronotum or corium usually with orange areas.	*Zelus laticornis* (Fig. 115a, b, c, d)
–	Length more than 12 mm. Medial process lateral margins parallel, apex notched in middle (Fig. 46a). Paramere strongly ventrally directed. Dorsal surface nearly uniformly dark brown.	*Zelus casii* (Fig. 45)
6	Posterior surface of medial process with ridge-like elevation, extending ventrally, not across width of medial process (Figs 107a, 110a).	7
–	Posterior surface of medial process with hook-like process, across width of medial process; if extending ventrally, as pair of dentate processes.	8
7	Posterior margin of posterior pronotal lobe yellowish, much lighter than remaining surface of the lobe. Paramere strongly curved downward, width more or less uniform, abruptly constricted at base (Figs 110a, 161).	*Zelus kartaboides* (Fig. 109)
–	Posterior pronotal lobe uniformly colored, dark brown. Paramere with basal 1/2 nearly straight, only curved in apical 1/2, gradually expanded toward apex (Fig. 107a).	*Zelus kartabensis* (Fig. 106a, b)
8	Humeral angle raised to level of, and nearly continuous with, disc. Short, light, shining recumbent setae dorsally on head and pronotum. Medial process long, longer than parameres; base very broad (Fig. 37a). Paramere clearly bent ventrally at mid part (central and northern S.A.).	*Zelus auralanus* (Fig. 36a, b, c, d)
–	Pronotal disc clearly elevated above humeral angle. Medial process narrowly triangular, shorter or subequal in length to paramere. Paramere constricted at base and somewhat bent ventrad.	9
9	Humeral angle rounded. Medial process long, upright (Fig. 134a) (Brazil, Bolivia and Paraguay).	*Zelus mattogrossensis* (Fig. 133a, b)
–	Humeral angle with spinous or dentate process. Medial process relatively short, semi-erect.	10
10	Medial process posterior surface with pair of processes near apex (Fig. 209a). Paramere bulbous, base slightly constricted.	*Zelus versicolor* (Fig. 208a, b)
–	Medial process apex hook-like, entire, not discontinuous as pair of projections (Fig. 52a). Paramere base strongly constricted, rest somewhat bulbous.	*Zelus chamaeleon* (Fig. 51a, b)

### Key to males of the *Zelus panamensis* species group

**Table:** 

1	Paramere achieving or surpassing apex of medial process.	2
–	Paramere not achieving apex of medial process.	3
2	Reddish-brown. Femora without banding. Paramere curved ventrally, apex recurved slightly dorsally (Fig. 215a).	*Zelus xouthos* (Fig. 214)
–	Anterior pronotal lobe dark brown, posterior lobe yellowish-brown. Femora dark brown, with two yellowish bands. Paramere straight (Fig. 43a).	*Zelus banksi* (Fig. 42)
3	Medial process shorter than or at most subequal in length to paramere.	4
–	Medial process at least 1.1x length of paramere.	6
4	Head orangish or reddish.	*Zelus panamensis* (Fig. 150a, b)
–	Head brown or dark brown.	5
5	Postocular lobe with longitudinal yellowish stripe. Medial process about as long as paramere (Fig. 194a). Paramere apex obliquely truncate.	*Zelus truxali* (Fig. 193a, b)
–	Postocular lobe without stripe. Medial process less than 0.8x length of paramere (Fig. 113a). Paramere apex rounded.	*Zelus korystos* (Fig. 112)
6	Paramere diameter constant through apical 3/4 or only slightly expanding.	7
–	Paramere expanded apically, enlarged portion in lateral view much greater than diameter of medial process.	9
7	Medial process bent in middle (Fig. 77a). Paramere narrowed at base, remainder constant.	*Zelus filicauda* (Fig. 76)
–	Medial process nearly straight, curving gradually. Paramere diameter constant or weakly expanding.	8
8	Medial process posteriorly directed, at less than forty-five degree angle to horizontal axis (Fig. 83a). Body surface brown.	*Zelus gilboventris* (Fig. 82a, b)
–	Medial process erect, at larger than forty-five degree angle to horizontal axis (Fig. 145a). Wasp-like.	*Zelus nigromaculatus* (Fig. 144a, b)
9	Medial process narrowed apically in lateral view (Fig. 61a). Paramere somewhat swollen.	*Zelus cordazulus* (Fig. 60a, b)
–	Medial process slightly expanded subapically in lateral view (Fig. 206a). Paramere expanding, but not distinctly swollen.	*Zelus varius* (Fig. 205)

### Key to males of the *Zelus armillatus* species group

**Table:** 

1	General coloration greenish or yellowish pale brown, rather uniform. Legs without banding.	2
–	Not as described above, consisting of typically two or more different colors; if uniformly colored, then dark brown or blackish-brown. Legs typically banded; if unbanded, then uniformly blackish-brown.	4
2	Paramere long, achieving or surpassing apex of medial process (Fig. 20a).	*Zelus amblycephalus* (Fig. 19a, b)
–	Paramere removed from apex of medial process.	3
3	Disc of posterior pronotal lobe strongly bulging. Paramere slender; diameter of basal 1/2 less than that of medial process in lateral view (Fig. 197a).	*Zelus umbraculoides* (Fig. 196)
–	Disc slightly bulging, nearly flat. Diameter of paramere greater than that of medial process in lateral view (Fig. 199).	*Zelus umbraculus* (Fig. 199)
4	Disc of posterior pronotal lobe depressed in middle.	5
–	Disc bulging or flat.	6
5	Dorsal coloration predominantly brownish-black, with various reddish areas; venter reddish; white waxy exudation usually conspicuous dorsally and laterally (Southern S.A.).	*Zelus leucogrammus* (Fig. 118a, b, c, d)
–	Entire surface testaceous-brown (Mexico and C.A.).	*Zelus sulcicollis* (Fig. 187a, b)
6	Paramere long, more than 2x length of median process.	7
–	Paramere short, less than 2x length of median process.	8
7	Dorsal coloration brownish-black; femora without or with few indistinct bands. Disc of posterior pronotal lobe bears pair of tubercles (C.A.).	*Zelus lewisi* (Fig. 121a, b)
–	Dorsal coloration yellow-green; legs conspicuously annulated. Disc without tubercles (Northern S.A.).	*Zelus annulosus* (Fig. 25)
8	Humeral angle raised nearly to level of, and almost continuous with, disc.	*Zelus janus* (Fig. 103a, b)
–	Humeral angle clearly below level of disc.	9
9	Medial process relatively robust.	10
–	Medial process delicate.	11
10	As viewed posteriorly, diameter of medial process less than 1.5x ocellar diameter, slightly larger than that of paramere (Fig. 34a). Coloration highly variable (S.A.).	*Zelus armillatus* (Fig. 31)
–	Medial process broad, diameter near middle at least 1.5x ocellar diameter (Fig. 58a).	*Zelus conjungens* (Fig. 57a, b, c)
11	Head reddish-brown. Paramere very delicate (Fig. 174a).	*Zelus ruficeps* (Fig. 173a, b, e)
–	Head dark brown, sometimes with stripes. Apical portion of paramere obviously thicker than medial process (Fig. 125a).	*Zelus litigiosus* (Fig. 124a, b, c, d)

### Key to males of the *Zelus longipes* species group

**Table:** 

1	Paramere more than 2x length of medial process in lateral view, surpassing medial process by a moderately large margin.	2
–	Paramere less than 2x length of medial process, achieving or slightly surpassing medial process.	3
2	Coloration consisting of reddish-brown and brownish-black, locations and relative amounts highly variable. Paramere diameter more or less constant (Fig. 128a).	*Zelus longipes* (Fig. 127a, b, c, d)
–	Coloration mainly of yellow and dark brown, posterior pronotal lobe orangish-brown. Paramere medially slightly constricted (Fig. 40a).	*Zelus bahiaensis* (Fig. 39)
3	Body elongated. Medial process long (Fig. 66a). Cu and Pcu of quadrate cell subparallel. Cu-Pcu2 (posterior cross vein) less than 1/2x length of Cu.	*Zelus errans* (Fig. 65a, b)
–	Body not as elongated as that of *Zelus errans*. Medial process short (Fig. 212a). Cu and Pcu of quadrate cell somewhat converging anteriorly, Cu-Pcu2 (posterior cross vein) more than 1/2x length of Cu.	*Zelus vespiformis* (Fig. 211a, b)

### Key to males of the *Zelus puertoricensis* species group

**Table:** 

1	Postocular lobe with dorsal and ventral surfaces nearly parallel through anterior 2/3; height at middle of lobe and through ocelli subequal. Medial process short (Fig. 165a).	*Zelus puertoricensis* (Fig. 164a, b)
–	Dorsal surface sloping downward from ocelli, height at middle of lobe less than 0.9x that through ocelli. Medial process moderately long (Fig. 185a).	*Zelus subimpressus* (Fig. 184a, b)

*Zelus bruneri* and *Zelus zayasi* are not included in the key. No physical specimens were examined for the former and male is not known for the latter.

### Key to females of *Zelus*

**Table:** 

1	Anterior pronotal lobe dorsally with short, dense, erect, spine-like setae on well-defined setal tracts. Humeral angle usually rounded, some armed.	2
–	Anterior pronotal lobe dorsally with fine setae or nearly bare, any spine-like setae sparse; setal tracts not necessarily well-defined. Humeral angle usually armed, some rounded.	8
2	Humeral angle armed with spinous process. Body elongate.	*Zelus annulosus*
–	Humeral angle rounded, without lateral process.	3
3	Dorsal surface of head, pronotum, scutellum and hemelytron nearly unicolorous, dark brown to black. Cells of membrane occasionally lighter in color than veins.	4
–	Dorsal surface with reddish-brown areas on pronotum and/or scutellum, often with yellowish-brown to reddish-brown areas on clavus and corium and/or basal 1/2 of membrane.	5
4	Veins and cells of membrane same color; cranial setae dark (northern S. A.).	*Zelus fuliginatus* (Fig. 79c, d)
–	Cells of membrane conspicuously less pigmented than veins; postocular lobe with longitudinal lateral patch of whitish recumbent setae (Bolivia, Peru, Paraguay, southern Brazil).	*Zelus aithaleos*
5	Profemoral length less than 20x profemoral diameter. Quadrate cell short and broad; Pcu of quadrate cell less than 1/2 length of Cu.	*Zelus means* (Fig. 136)
–	Profemoral length at least 20x profemoral diameter. Quadrate cell elongate, if broad, then Pcu more than 1/2 length of Cu.	6
6	Body elongated. Cu and Pcu of quadrate cell subparallel, Cu-Pcu2 (posterior cross vein) less than 1/2x length of Cu.	7
–	Body not as elongated as that of *Zelus errans* or *Z. gracilipes*. Cu and Pcu of quadrate cell somewhat converging anteriorly, Cu-Pcu2 (posterior cross vein) more than 1/2x length of Cu.	*Zelus vespiformis* (Fig. 211c, d, e, f)
7	Entire membrane colored or opaque.	*Zelus gracilipes* (Fig. 85c, d)
–	Anterior 1/2 of membrane clear or semi-translucent.	*Zelus errans* (Fig. 65c, d, e)
8	Compound eyes extending below ventral surface of head.	*Zelus grandoculus*
–	Compound eyes not extending below ventral surface of head.	9
9	Posterior margin of pronotal disc with two tubercles. Humeral angle with spinous process.	10
–	Posterior margin of pronotal disc unarmed. Humeral angle armed or unarmed.	12
10	Tubercles of pronotal disc pronounced. More than 13 mm in length.	11
–	Tubercles very small, minute in some specimens. Thirteen mm or less in length. (Southern C.A. and Northern S.A.)	*Zelus minutus*
11	Large, 22-25 mm. Head, pronotum and abdomen yellow or reddish with dark spots or irregularly shaped patches (C.A.).	*Zelus lewisi* (Fig. 121c, d, e, f)
–	Medium-sized, 13-16 mm. Pronotum and abdomen more or less unicolorous, greyish (N.A., C.A., southern S.A.).	*Zelus tetracanthus* (Fig. 190b, c)
12	Posterior pronotal lobe, as viewed from behind, with posterior margin sloping sharply downward on either side of scutellum (northern S.A.).	*Zelus varius*
–	Posterior pronotal lobe, with posterior margin straight or sloping gradually (not appreciably more than forty-five degrees) downward on either side of scutellum.	13
13	Head surface reddish-brown, any darker cranial markings with indistinct outlines; remainder of dorsal surface of body primarily dark brown to brownish-black. 13.0-18.5 mm in length.	14
–	Coloration other than above. Length variable.	17
14	Membrane and clavus with bluish iridescence.	*Zelus erythrocephalus* (Fig. 68c, d)
–	Membrane shining, but not showing bluish iridescence.	15
15	Veins of corium anterior to membrane reddish-brown. Postocular lobe with dark semierect to erect setae dorsally. Scutellum bearing light recumbent setae dorsally.	*Zelus russulumus* (Fig. 176c, d)
–	At least one of the characters not as described above.	16
16	Postocular lobe with light-colored, inconspicuous setae dorsally (northern S.A.).	*Zelus paracephalus* (Fig. 153c, d)
–	Postocular lobe with conspicuous dark semi-erect to erect setae (southern C.A. and northern S.A.).	*Zelus panamensis* (Fig. 150c, d)
17	Length of postocular lobe less than 0.77x width of head through compound eyes. Postocular lobe with long erect setae over surface. Metafemoral length less than 20x metafemoral width (northern S.A.).	*Zelus chamaeleon* (Fig. 51c, d)
–	Length of postocular lobe at least 0.80x width of head through compound eyes; if long setae present on postocular lobe, then not over entire surface.	18
18	Anterior portion of posterior pronotal lobe broadly sulcate on dorsal surface; at least 18.5 mm in length.	19
–	Posterior pronotal lobe not conspicuously depressed medially; if slight depression present, not larger than 17.0 mm in length	20
19	General coloration brown; female profemur approximately 0.95x or more diameter of mesofemur (Mexico and C.A.).	*Zelus sulcicollis* (Fig. 187c, d)
–	Dorsal coloration reddish-brown and brownish-black; white waxy exudation usually conspicuous dorsally; profemur approximately 0.90x or less diameter of mesofemur (southern and central S.A.).	*Zelus leucogrammus* (Fig. 118e, f)
20	Humeral angle of pronotum rounded; dorsal surface, except dorsum of abdomen, reddish-brown and brownish-black, pattern variable; erect setae predominating on dorsum of pronotum; posterior portion of anterior pronotal lobe conspicuously raised above level of anterior margin of posterior lobe (N.A. C.A., S.A. and Caribbean).	*Zelus longipes* (Fig. 127e, f)
–	At least one of the characters not as described above.	21
21	Length at least 5x width. Profemoral diameter equal to or greater than that of mesofemur. Mesofemoral diameter less than 1.2x that of metafemur. Tuberculate to small spinous lateral processes on humeral angle. Interocular distance less than 1.15x interocellar distance.	22
–	At least one of the characters not as described above.	23
22	Postocular lobe with dorsal and ventral surfaces nearly parallel through anterior 2/3; height at middle of lobe and through ocelli subequal (Greater Antilles).	*Zelus puertoricensis* (Fig. 164c, d)
–	Dorsal surface sloping downward from ocelli; height at middle of lobe less than 0.9x that through ocelli (Greater Antilles).	*Zelus subimpressus* (Fig. 184c, d)
23	Length at least 5.5x width.	24
–	Length less than 5.5x width.	26
24	Length 12.5 mm or greater (Southern U.S., Mexico, C.A. and northwestern S.A.)	*Zelus cervicalis* (Fig. 48d, e, f)
–	Length less than 12.5 mm (9.11-12.00 mm).	25
25	Anteocular lobe at least 1.25x length of postocular lobe. Body length at least 7.2x width (Cuba).	*Zelus bruneri*
–	Anteocular lobe less than 1.25x length of postocular lobe. Body length less than 7.2x width (S.A.)	*Zelus prolixus* (Fig. 161c, d)
26	Humeral angle rounded. Less than 14 mm in length.	27
–	Humeral angle with at least small tubercles or spines; if tubercles and spines not readily evident, at least 14 mm in length.	28
27	Profemoral length at least 20x profemoral width (S.A. and southern C.A.).	*Zelus inconstans* (Fig. 100c, d)
–	Profemoral length less than 20x profemoral width (Brazil, Bolivia and Paraguay).	*Zelus mattogrossensis* (Fig. 133c, d)
28	Length usually less than 4.0x width; if length greater than 4.0x and less than 4.5x width, profemoral diameter greater than that of mesofemur.	29
–	Length usually at least 4.0x width; if length less than 4.0x width, mesofemoral diameter greatest.	34
29	Length usually at least 4.0x width; if length less than 4.0x width, mesofemur enlarged, diameter greatest.	*Zelus conjungens* (Fig. 57d, e, f)
–	Mesofemoral diameter less than that of profemur, if mesofemoral diameter greatest, then never exceeding 1.1x that of profemoral diameter.	30
30	Length of anteocular lobe at least 1.1x that of postocular lobe. Rostral segment II less than 1.35x length of segment I. Dorsal coloration uniform, pale brown, somewhat greenish. Legs not banded (Mexico, C.A. and S.A.).	*Zelus amblycephalus* (Fig. 19c, d)
–	Anteocular lobe less than 1.1x length of postocular lobe. Rostral segment II at least 1.35x length of segment I. Dorsal surface is either variously patterned, with contrasting pale and dark regions or uniformly colored, dark brown to black. Legs usually with bands or annulations, or uniformly dark brown to black.	31
31	Humeral angle raised to level of, and nearly continuous with, disc (Mexico and C.A.).	*Zelus janus* (Fig. 103c, d)
–	Humeral angle below level of disc; disc clearly elevated.	32
32	Setal tracts of anterior pronotal lobe and dorsum of posterior lobe with conspicuous light-colored shining recumbent setae, especially dorsolateral area of lobe; some erect setae also present; setal tracts usually of contrasting color. Length:width ratio approximately 4:1 (Mexico and C.A.).	*Zelus litigiosus* (Fig. 124e, f)
–	Setal tracts and dorsum of posterior pronotal lobe with few, if any, recumbent setae; tracts not usually of contrasting color. Length:width ratio usually less than 3.5:1.	33
33	Pubescence distributed evenly over lateral surface of abdominal segments (S.A.).	*Zelus armillatus* (Figs 32, 33)
–	Pubescence, except for some scattered setae, restricted to anterior 1/2 of lateral surface of abdominal segments (Mexico, C.A., and northern S.A.).	*Zelus ruficeps* (Fig. 173c, d)
34	Dorsal surface dark brown to brownish-black. Golden, shining, short recumbent setae dorsally, especially on head and pronotum. Humeral angle raised to level of, and nearly continuous with, disc (central and southern S.A.).	*Zelus auralanus* (Fig. 36e, f)
–	Dorsal surface usually not dark brown, but if so, then setae not as above. If humeral angle raised, coloration not as dark above.	35
35	Humeral angle raised to level of, and nearly continuous with, disc. General dorsal coloration yellowish-brown.	36
–	Humeral angle below level of disc. If dorsal coloration yellowish-brown as above, then disc clearly differentiated.	38
36	Profemoral length at least 22x profemoral width.	*Zelus xouthos*
–	Profemoral length less than 22x profemoral width.	37
37	Medial sulcus of anterior pronotal lobe with dark brown areas near posterior margin (Mexico and C.A.).	*Zelus ambulans* (Fig. 22c)
–	Medial sulcus of anterior pronotal lobe same color as surrounding area of lobe near posterior margin (Mexico and C.A.).	*Zelus exsanguis* (Fig. 71c, d)
38	Length of rostral segment II less than 1.8x that of segment I. Profemoral length less than 22x profemoral width. Pronotum covered with erect setae dorsally, some setae subequal in length to diameter of shaft of antennal segment I. Humeral angle with very small tuberculate or subtuberculate lateral process (Mexico, C.A., and northern S.A.).	*Zelus grassans* (Fig. 91d)
–	At least one of the characters not as described above.	39
39	Length of rostral segment II less than 2x that of segment I. Profemoral and mesofemoral lengths less than 20x and 11x that of respective widths. Length of posterior pronotal lobe greater 2.4x that of anterior lobe.	*Zelus laticornis* (Fig. 115e, f)
–	At least one of the characters not as described above.	40
40	Profemoral length less than 17x that of profemoral width. Length of rostral segment II less than 2.1x that of segment I.	41
–	Profemoral length at least 17x of width. Rostral segment II usually greater than 2.1x that of segment I.	43
41	Humeral angle with short, inconspicuous subtuberculate to nearly dentate lateral processes, usually same color as surrounding area. Posterior pronotal lobe nearly smooth (Guatemala, Mexico and western and southwestern U.S.).	*Zelus renardii* (Fig. 167c, d)
–	Humeral angle with short to moderate, conical, spinous lateral processes, usually darker than surrounding area. Posterior pronotal lobe noticeably rugulose.	42
42	Dorsum of postocular lobe with long erect setae on posterior 1/2, some longer than ocular-ocellar distance (C.A.).	*Zelus antiguensis* (Fig. 28c, d)
–	Any erect setae on dorsum of postocular lobe shorter than ocular-ocellar distance (Canada, U.S. and Mexico).	*Zelus luridus* (Fig. 130c, d)
43	Length greater than 4.5x width. Humeral angle with very short inconspicuous, spinous lateral processes. Dorsum predominately dark brown to brownish-black except for light to dark reddish-brown to brown posterior pronotal lobe, but lighter than anterior lobe (Mexico and C.A.).	*Zelus mimus* (Fig. 138c, d, e, f)
–	At least one of the characters not as described above.	44
44	Posterior pronotal lobe with single broad dark brown transverse band posteriorly or pair of bands anteriorly and posteriorly, remaining dorsal surface of pronotum yellowish, with or without band.	45
–	Coloration of pronotum not as described above, brown or stramineous coloration predominating dorsally, anterior pronotal lobe same color as or lighter than posterior lobe.	49
45	Anterior pronotal lobe and anterior part of posterior lobe with dark bands; wasp-like appearance.	*Zelus nigromaculatus* (Fig. 144c, d)
–	Anterior pronotal lobe and anterior portion of posterior pronotal lobe of same yellowish color.	46
46	Transverse dark band on pronotum does not cover posterior margin of posterior pronotal lobe, which is instead yellowish.	47
–	Transverse dark band on pronotum covers posterior margin of posterior pronotal lobe.	48
47	Femoral yellowish-brown with only small darker markings near apices (central and northern S.A.)	*Zelus kartabensis* (Fig. 106c, d)
–	Femora yellowish-brown on basal 1/3 to 1/2; remainder dark brown to brownish-black, usually with one or two narrow yellowish bands (S.A.).	*Zelus plagiatus* (Fig. 159)
48	Pubescence of lateral surface of posterior pronotal lobe consisting almost entirely of erect setae, many longer than diameter of shaft of antennal segment II (S.A.).	*Zelus versicolor* (Fig. 208c, d)
–	Majority of setae on lateral surface of posterior pronotal lobe semi-erect to recumbent, no erect setae as long as diameter of shaft of antennal segment II (southern C.A. and northern S.A.).	*Zelus fasciatus* (Fig. 74)
49	Length >14 mm. Anterior pronotal lobe lighter than or same color as posterior lobe.	50
–	Length <13 mm. Anterior and posterior pronotal lobes same color.	*Zelus illotus* (Fig. 94c), *Zelus nugax* (Fig. 147c, d), *Zelus pedestris* (Fig. 156c, d)
50	Anterior pronotal lobe yellowish, lighter than posterior lobe.	51
–	Anterior pronotal lobe brown, same color as posterior lobe.	52
51	Femora unicolorous or with indistinct single bands. Anterior pronotal lobe elevated.	*Zelus sphegeus* (Fig. 182)
–	Meso- and Metafemora with two or three dark bands. Anterior pronotal lobe nearly flat, not elevated.	*Zelus truxali* (Fig. 193c, d)
52	Meso- and metafemora apical half more or less continuously reddish-brown, not broken into bands. Abdominal sternites each bearing a small black spot.	*Zelus cordazulus* (Fig. 60c, d)
–	Meso- and metafemora apical half with three indistinct but visible reddish-brown bands; abdominal sternites yellow, without black spots.	*Zelus gilboventris* (Fig. 82c, d)

This key is not organized by species group, as females do not readily display characters placing them to species groups. It is especially challenging or sometimes impossible to distinguish females of species in several species groups. Large series of co-occurring males and females will be helpful in these cases. Coloration is heavily used to key out females, and readers are reminded that some species exhibit a wide range of color variations and the exemplar habitus images provided in this work are not inclusive of all variants.

## Supplementary Material

Supplementary material 1 Specimen records of species of Zelus Fabricius, 1803 Data type: Occurrences
Brief description: This file contains specimen records of species of *Zelus*, including type and non-type materials examined by Zhang et al. (Biodiversity Data Journal) or recorded from literature.
File: oo_91806.xls Guanyang Zhang, Elwood Hart, Christiane Weirauch

Supplementary material 2 Measurements of species of Zelus Fabricius, 1803 Data type: morphological, morphological measurements
Brief description: Measurements of species of *Zelus* Fabricius, 1803.
File: oo_91807.xlsx Guanyang Zhang, Elwood Hart, Christiane Weirauch

XML Treatment for Zelus

XML Treatment for Zelus aithaleos

XML Treatment for Zelus amblycephalus

XML Treatment for Zelus ambulans

XML Treatment for Zelus annulosus

XML Treatment for Zelus antiguensis

XML Treatment for Zelus armillatus

XML Treatment for Zelus auralanus

XML Treatment for Zelus bahiaensis

XML Treatment for Zelus banksi

XML Treatment for Zelus bruneri

XML Treatment for Zelus casii

XML Treatment for Zelus cervicalis

XML Treatment for Zelus chamaeleon

XML Treatment for Zelus championi

XML Treatment for Zelus cognatus

XML Treatment for Zelus conjungens

XML Treatment for Zelus cordazulus

XML Treatment for Zelus couturieri

XML Treatment for Zelus errans

XML Treatment for Zelus erythrocephalus

XML Treatment for Zelus exsanguis

XML Treatment for Zelus fasciatus

XML Treatment for Zelus filicauda

XML Treatment for Zelus fuliginatus

XML Treatment for Zelus gilboventris

XML Treatment for Zelus gracilipes

XML Treatment for Zelus grandoculus

XML Treatment for Zelus grassans

XML Treatment for Zelus illotus

XML Treatment for Zelus impar

XML Treatment for Zelus inconstans

XML Treatment for Zelus janus

XML Treatment for Zelus kartabensis

XML Treatment for Zelus kartaboides

XML Treatment for Zelus korystos

XML Treatment for Zelus laticornis

XML Treatment for Zelus leucogrammus

XML Treatment for Zelus lewisi

XML Treatment for Zelus litigiosus

XML Treatment for Zelus longipes

XML Treatment for Zelus luridus

XML Treatment for Zelus mattogrossensis

XML Treatment for Zelus means

XML Treatment for Zelus mimus

XML Treatment for Zelus minutus

XML Treatment for Zelus nigromaculatus

XML Treatment for Zelus nugax

XML Treatment for Zelus panamensis

XML Treatment for Zelus paracephalus

XML Treatment for Zelus pedestris

XML Treatment for Zelus plagiatus

XML Treatment for Zelus prolixus

XML Treatment for Zelus puertoricensis

XML Treatment for Zelus renardii

XML Treatment for Zelus rosulentus

XML Treatment for Zelus ruficeps

XML Treatment for Zelus russulumus

XML Treatment for Zelus spatulosus

XML Treatment for Zelus sphegeus

XML Treatment for Zelus subimpressus

XML Treatment for Zelus sulcicollis

XML Treatment for Zelus tetracanthus

XML Treatment for Zelus truxali

XML Treatment for Zelus umbraculoides

XML Treatment for Zelus umbraculus

XML Treatment for Zelus vagans

XML Treatment for Zelus varius

XML Treatment for Zelus versicolor

XML Treatment for Zelus vespiformis

XML Treatment for Zelus xouthos

XML Treatment for Zelus zayasi

## Figures and Tables

**Figure 16a.**
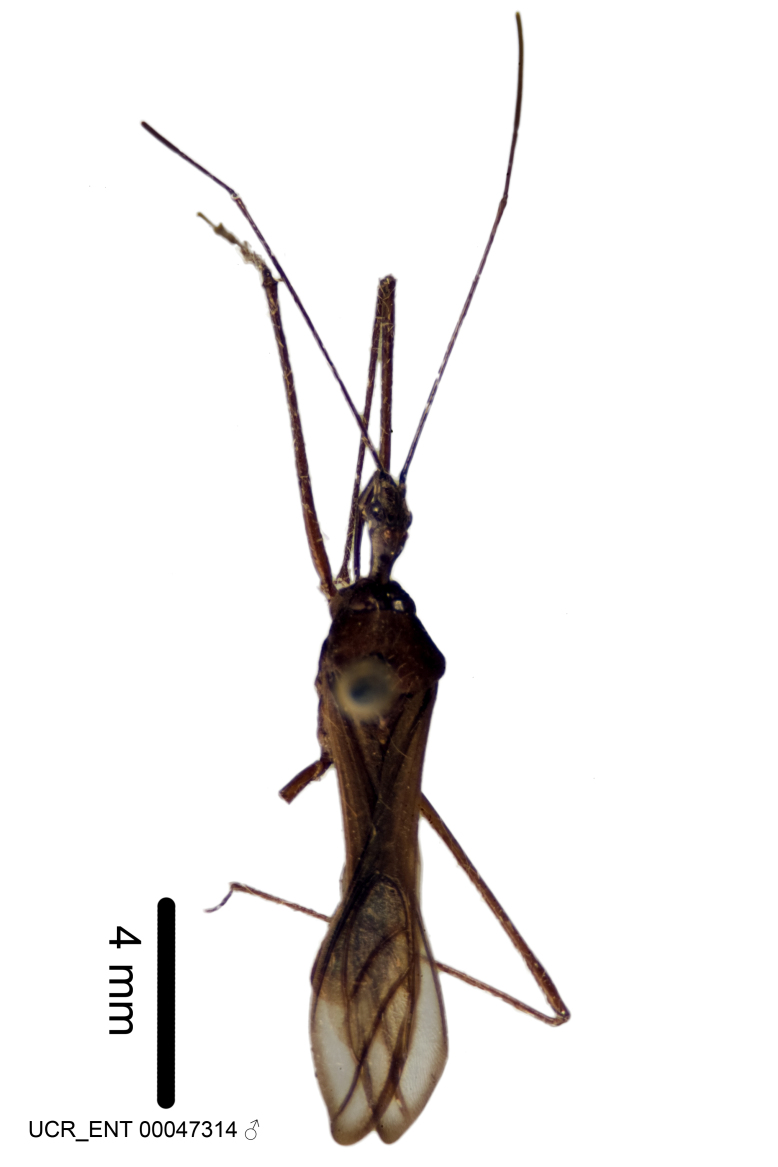
*Zelus aithaleos* Zhang & Hart, sp. n., male, dorsal view (UCR_ENT 00047314, Huanuco, Peru)

**Figure 16b.**
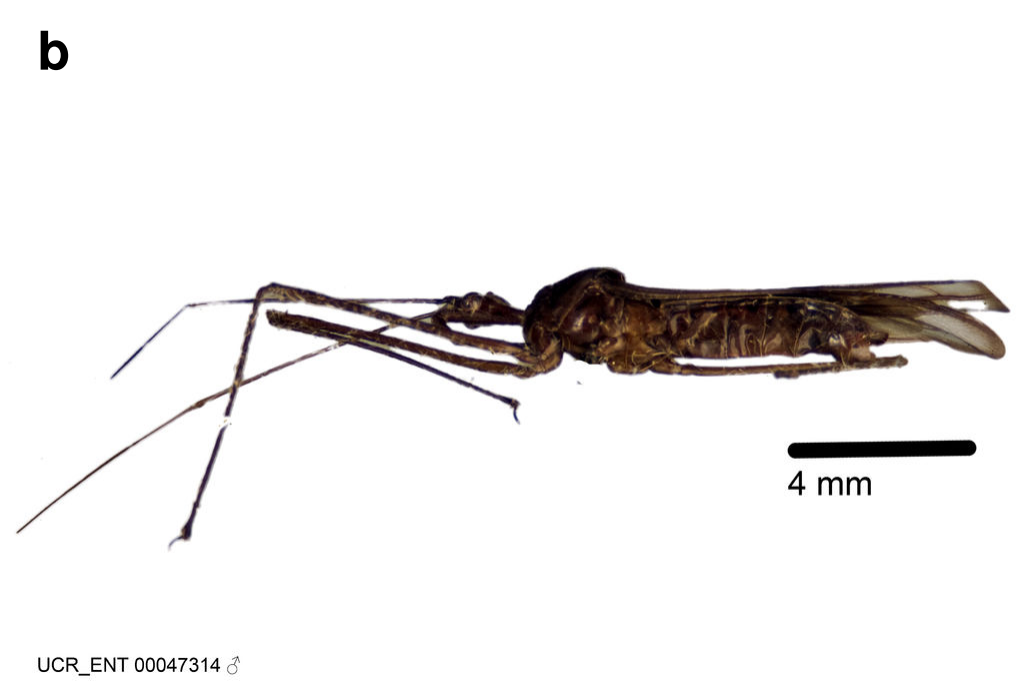
*Zelus aithaleos* Zhang & Hart, sp. n., male, lateral view (UCR_ENT 00047314, Huanuco, Peru)

**Figure 17a.**
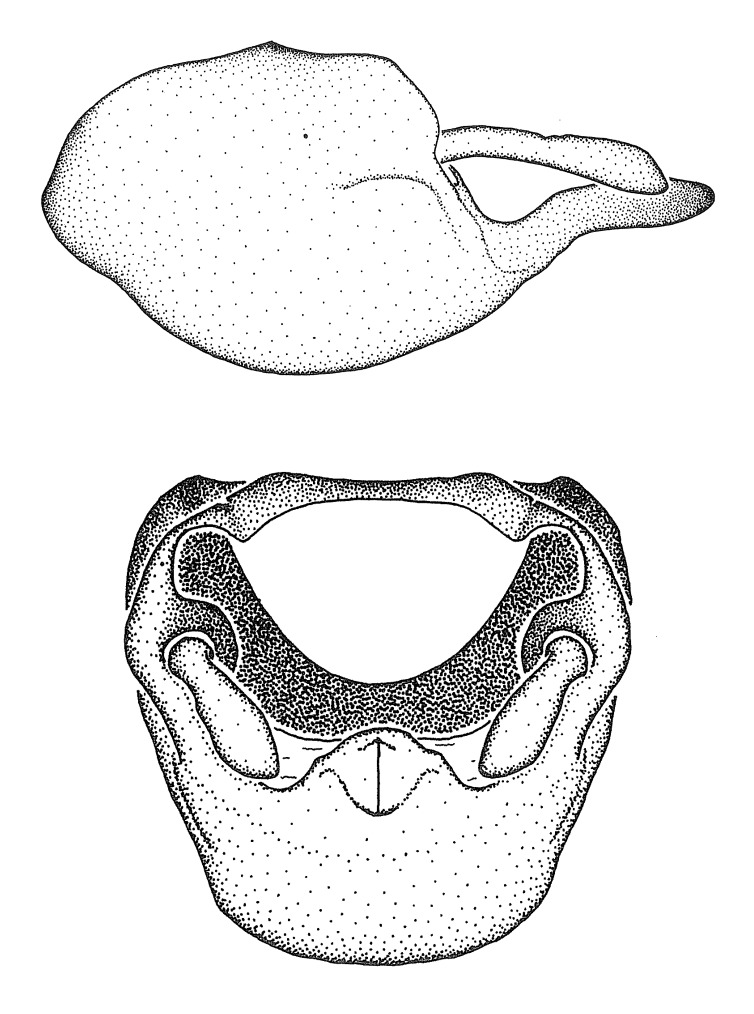
*Zelus aithaleos* Zhang & Hart, sp. n., pygophore, lateral and posterior views

**Figure 17b.**
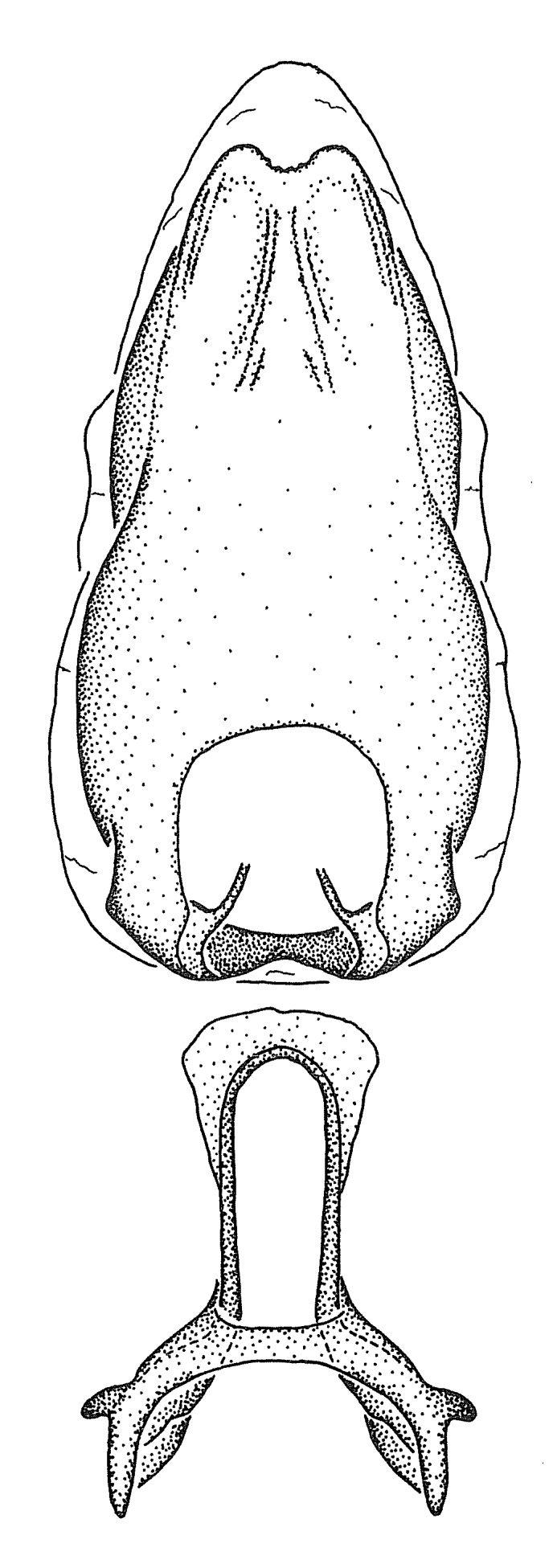
*Zelus aithaleos* Zhang & Hart, sp. n., phallus, dorsal view

**Figure 19a.**
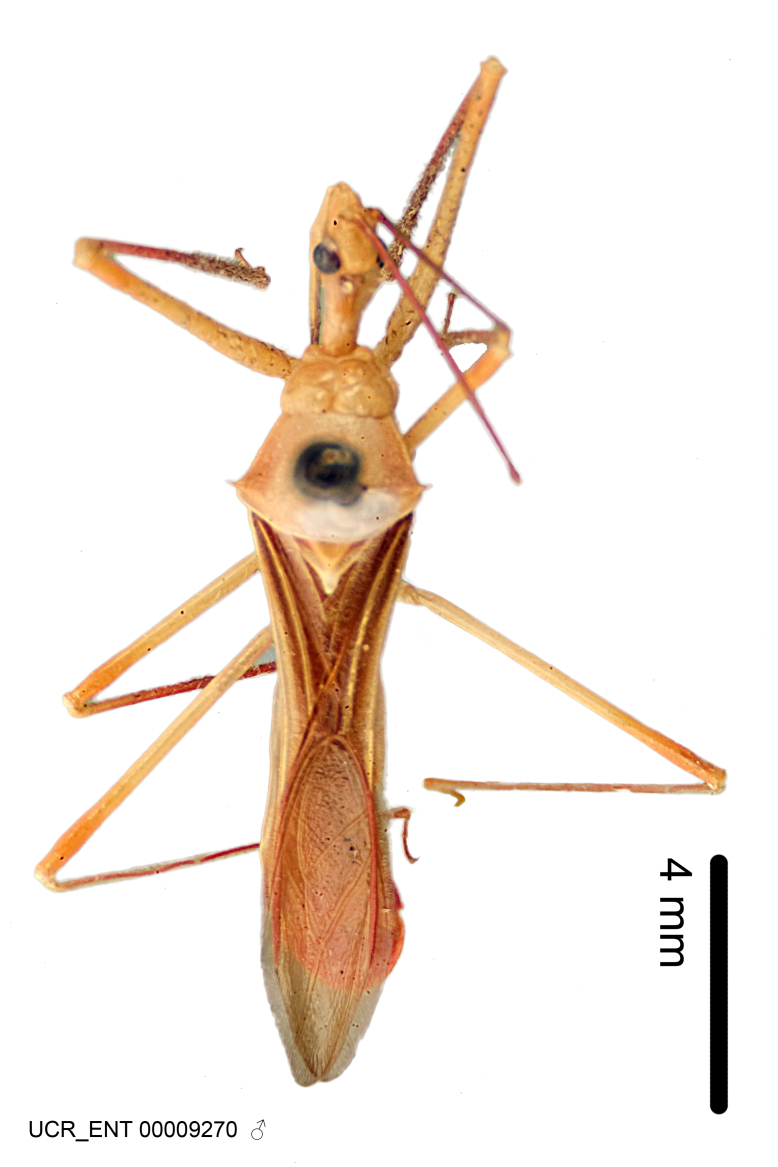
*Zelus amblycephalus* Zhang & Hart, sp. n., male, dorsal (UCR_ENT 00009270, Canal Zone, Panama)

**Figure 19b.**
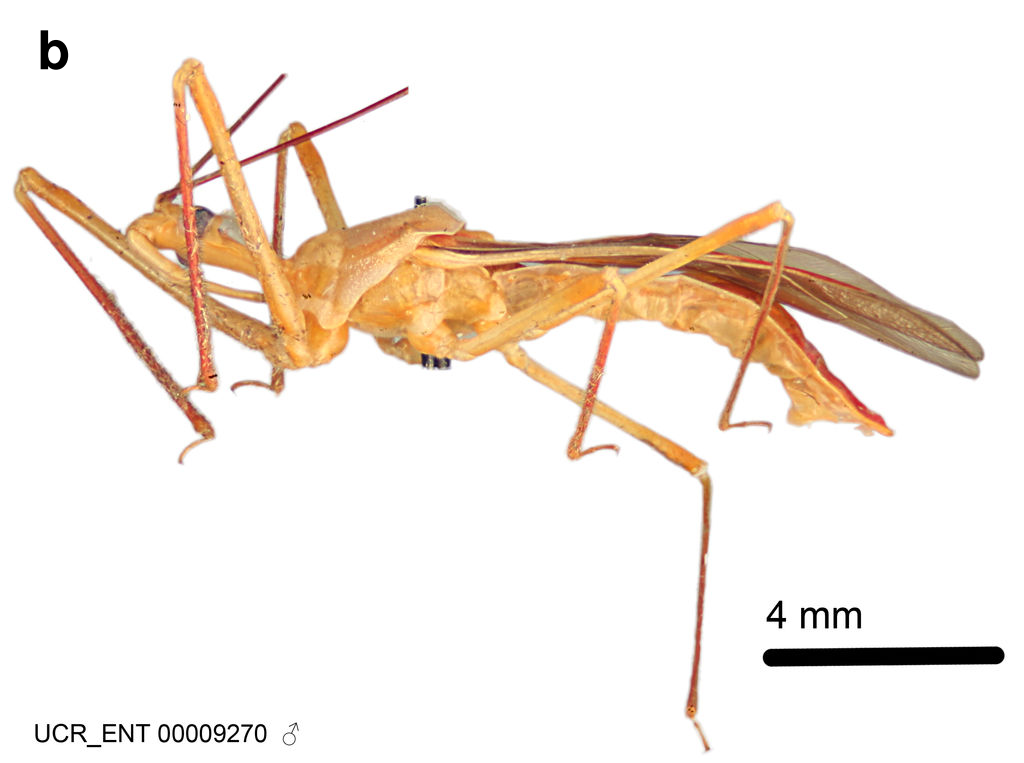
*Zelus amblycephalus* Zhang & Hart, sp. n., male, lateral (UCR_ENT 00009270, Canal Zone, Panama)

**Figure 19c.**
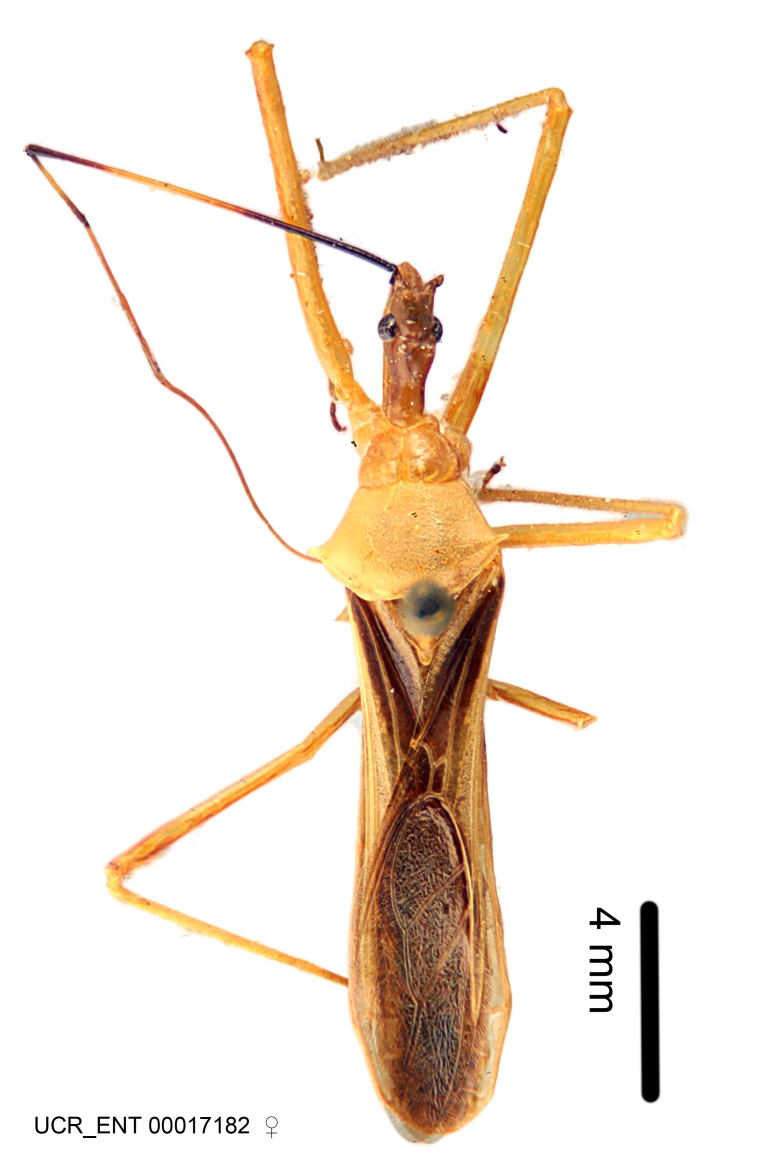
*Zelus amblycephalus* Zhang & Hart, sp. n., female, dorsal (UCR_ENT 00017182, Chiapas, Mexico)

**Figure 19d.**
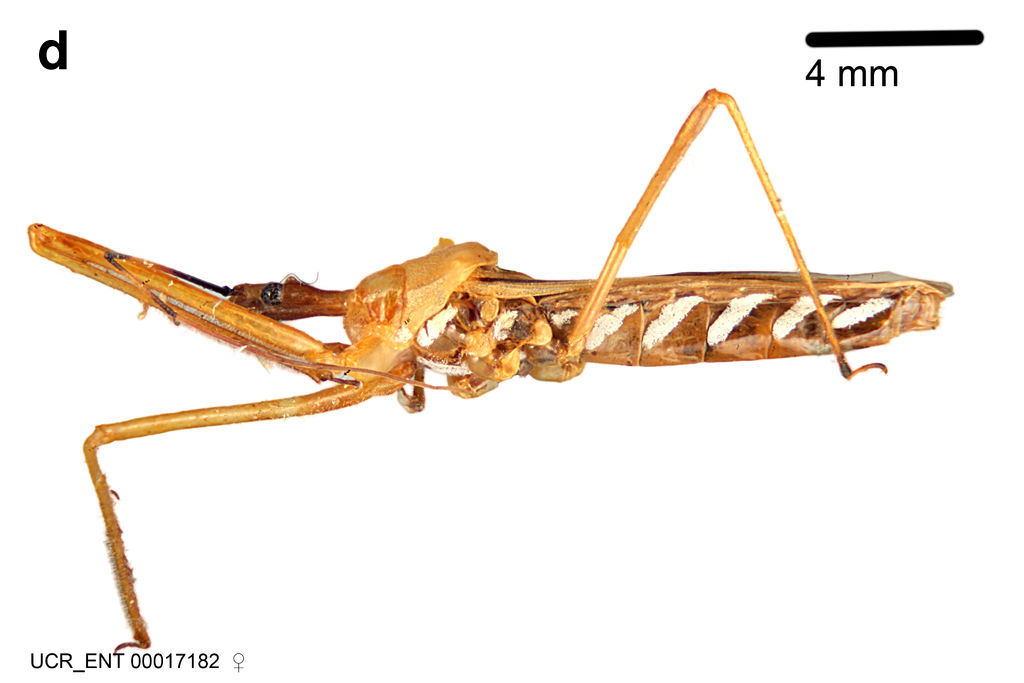
*Zelus amblycephalus* Zhang & Hart, sp. n., female, lateral (UCR_ENT 00017182, Chiapas, Mexico)

**Figure 20a.**
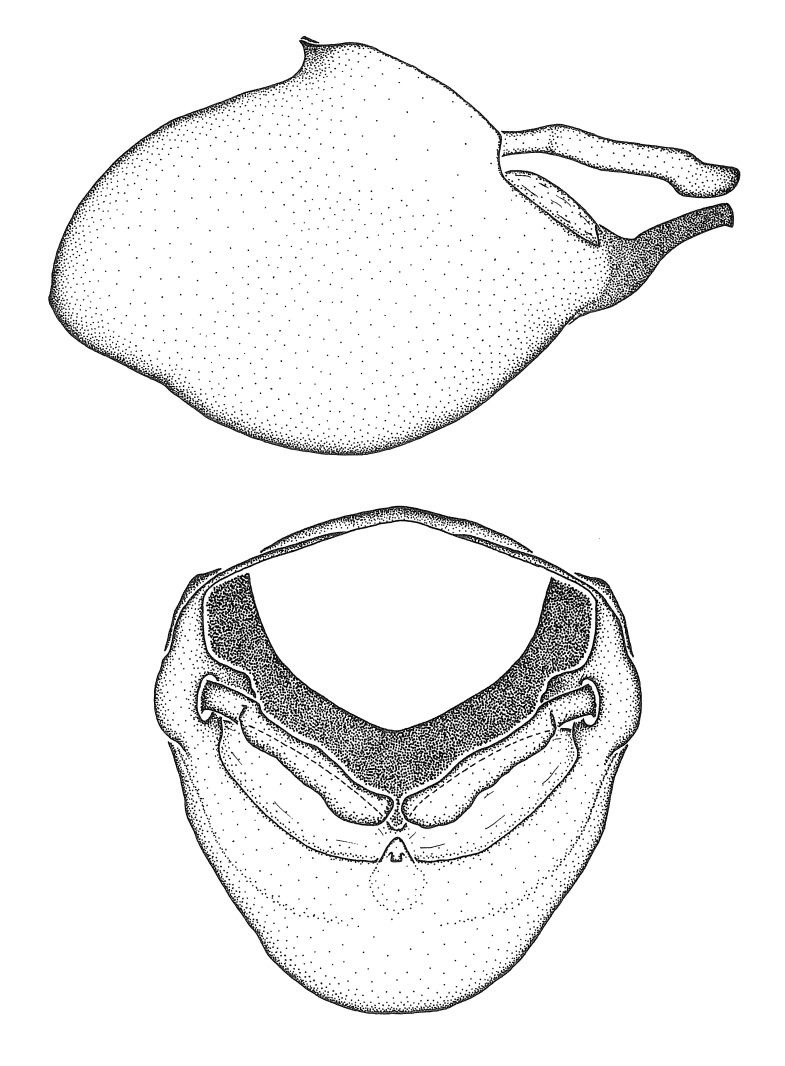
*Zelus amblycephalus* Zhang & Hart, sp. n., pygophore, lateral and posterior views

**Figure 20b.**
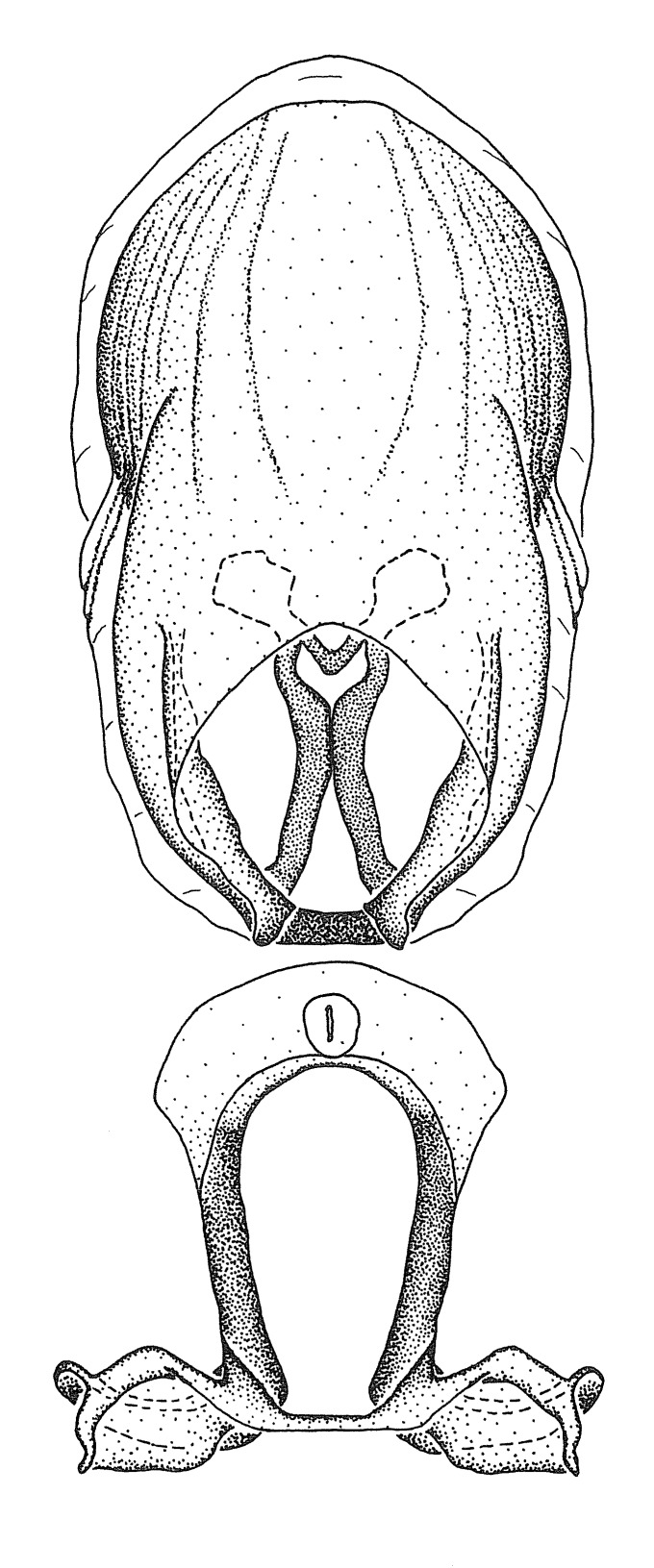
*Zelus amblycephalus* Zhang & Hart, sp. n., phallus, dorsal view

**Figure 22a.**
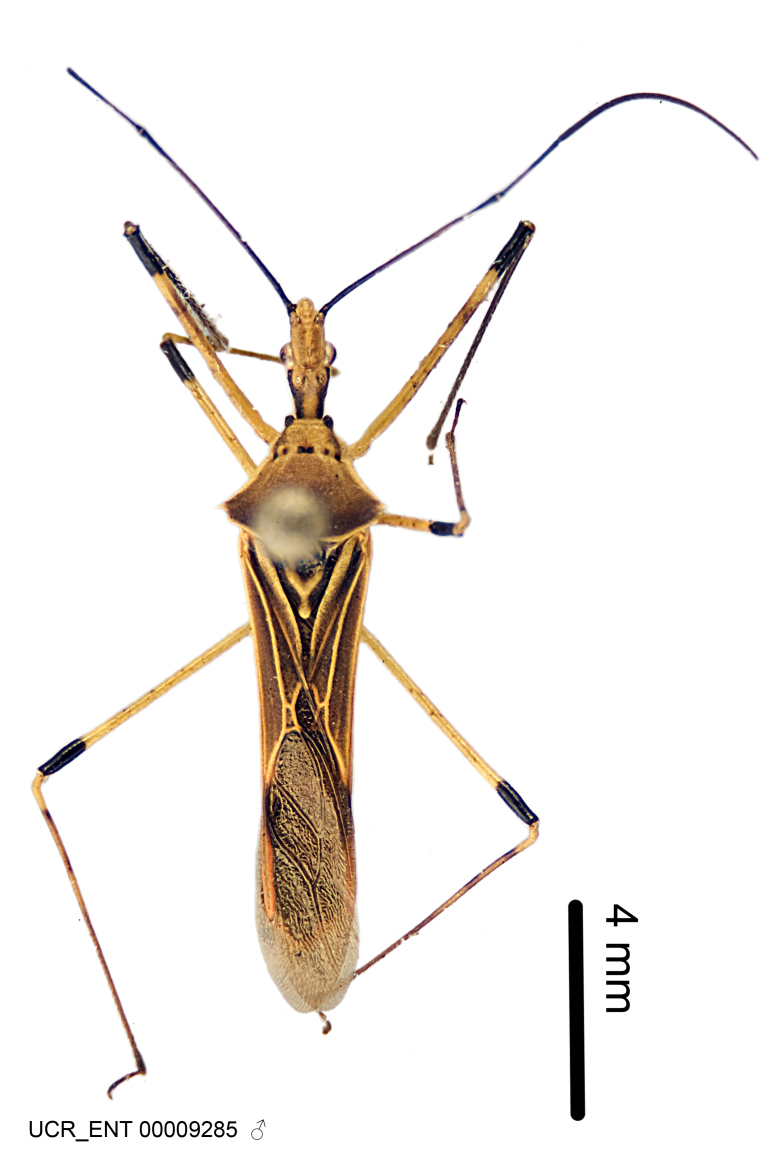
*Zelus ambulans* Stål, 1862, male, dorsal (UCR_ENT 00009285, Veracruz, Mexico)

**Figure 22b.**
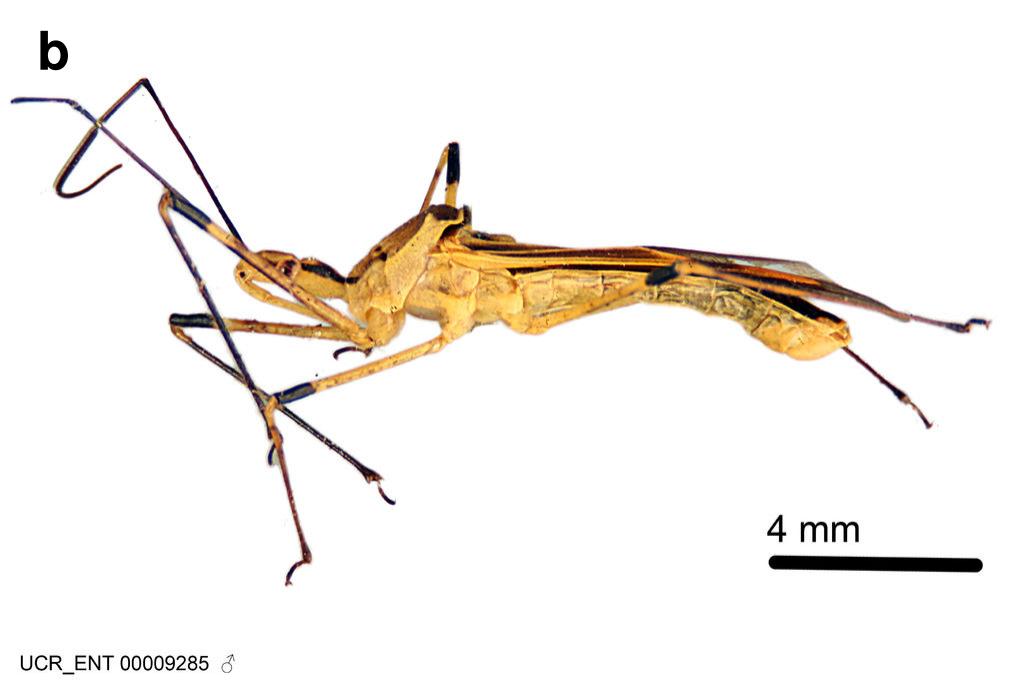
*Zelus ambulans* Stål, 1862, male, lateral (UCR_ENT 00009285, Veracruz, Mexico)

**Figure 22c.**
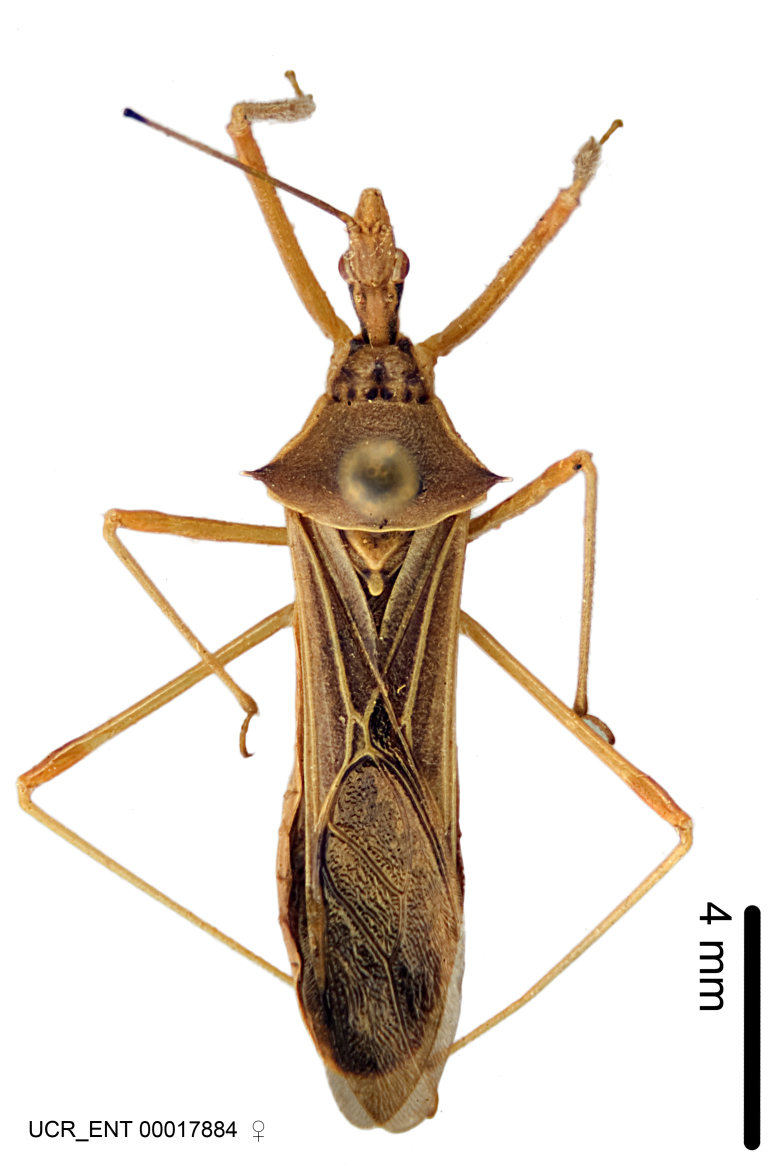
*Zelus ambulans* Stål, 1862, female, dorsal (UCR_ENT 00017884, Chiriqui, Panama)

**Figure 23a.**
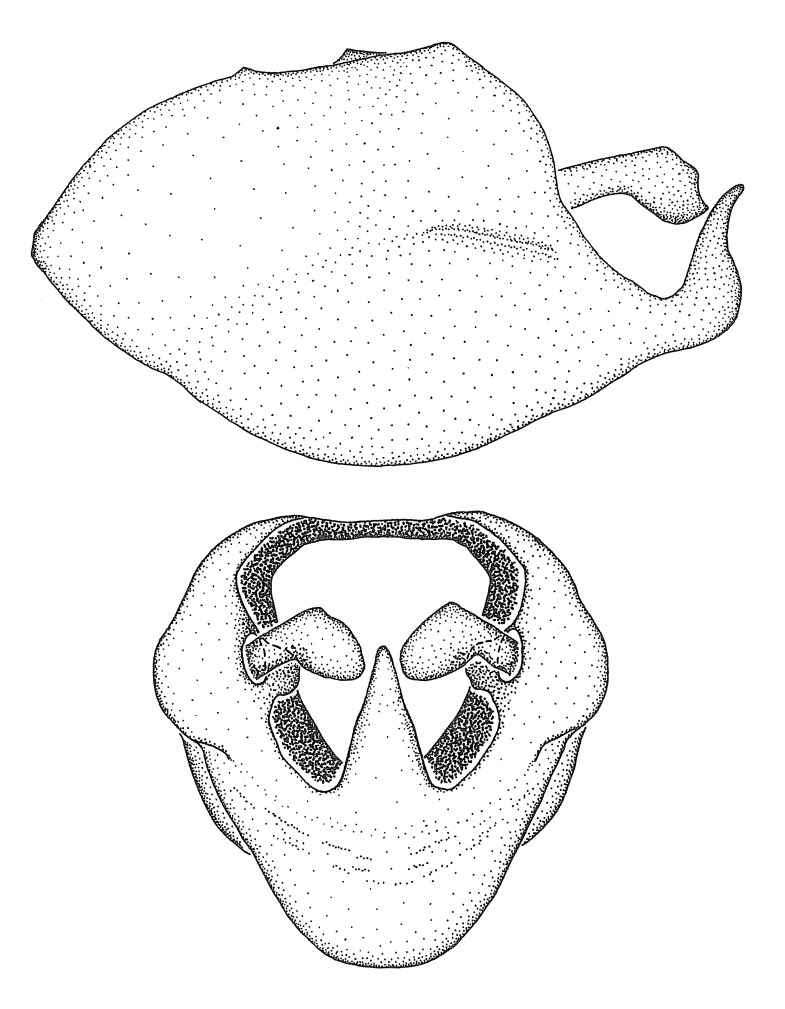
*Zelus ambulans* Stål, 1862, pygophore, lateral and posterior views

**Figure 23b.**
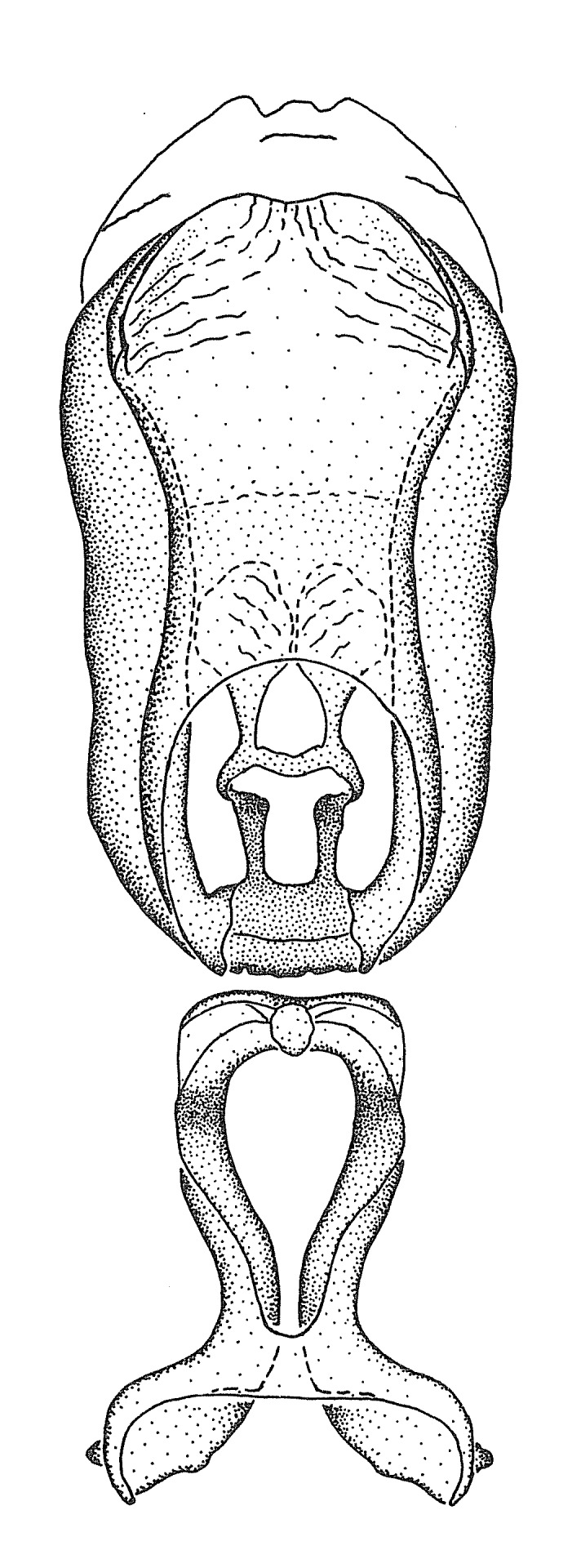
*Zelus ambulans* Stål, 1862, phallus, dorsal view

**Figure 25a.**
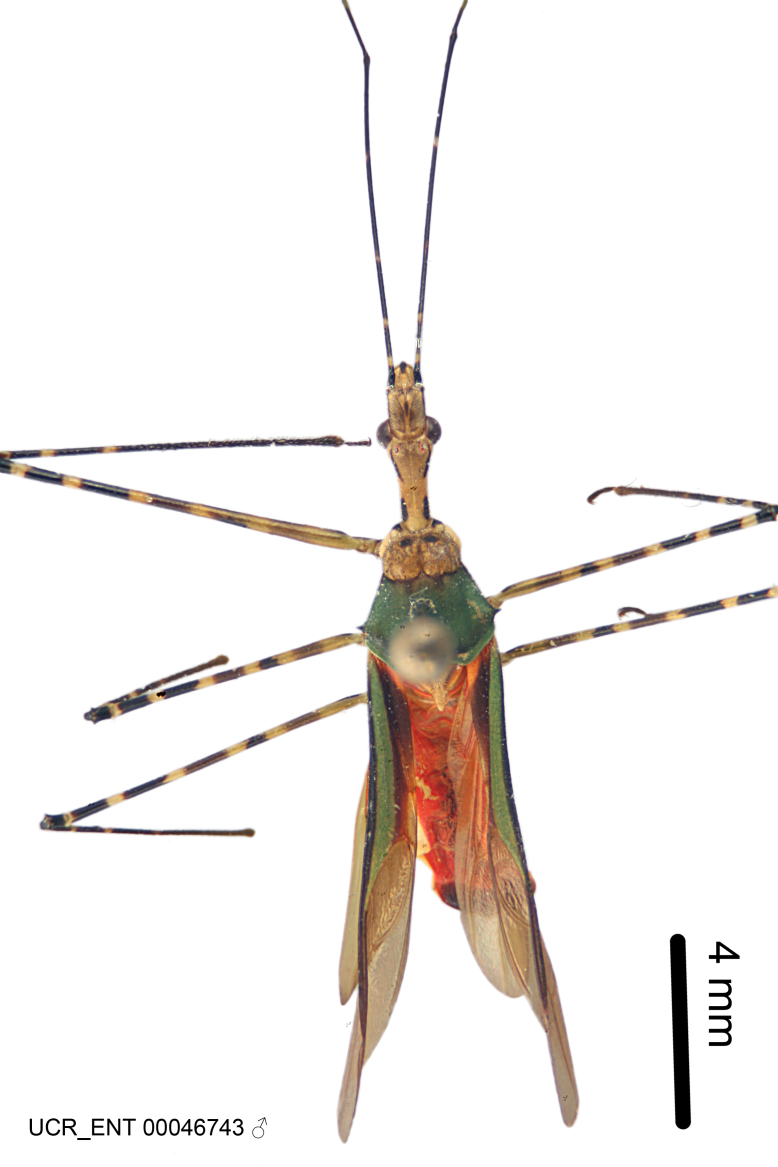
*Zelus annulosus* (Stål, 1866), male, dorsal (UCR_ENT 00046743, French Guiana)

**Figure 25b.**
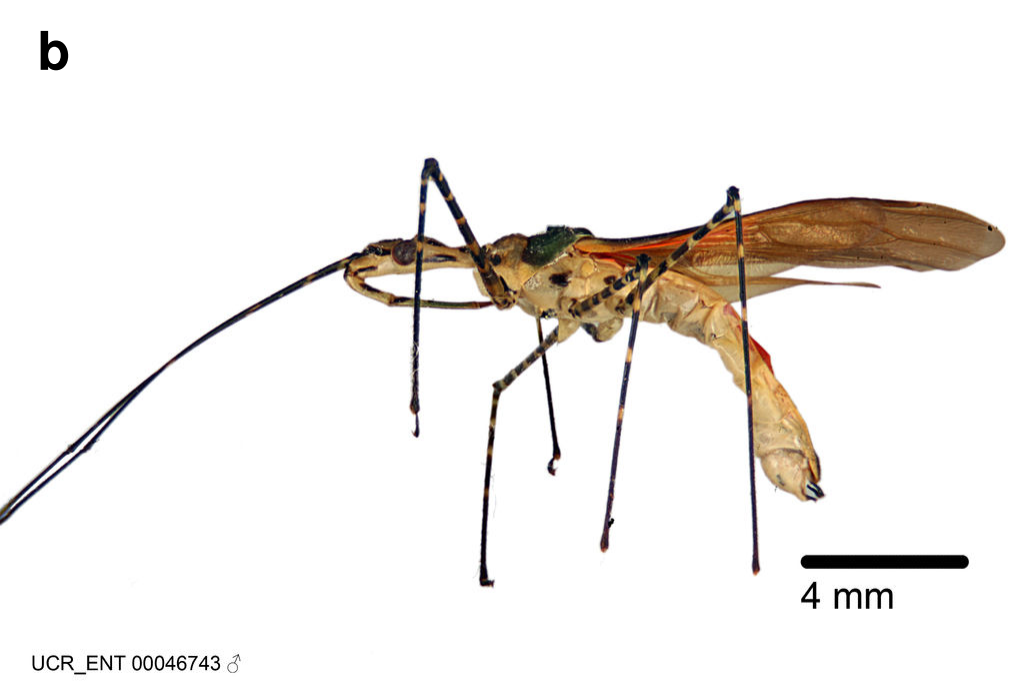
*Zelus annulosus* (Stål, 1866), male, lateral (UCR_ENT 00046743, French Guiana)

**Figure 26a.**
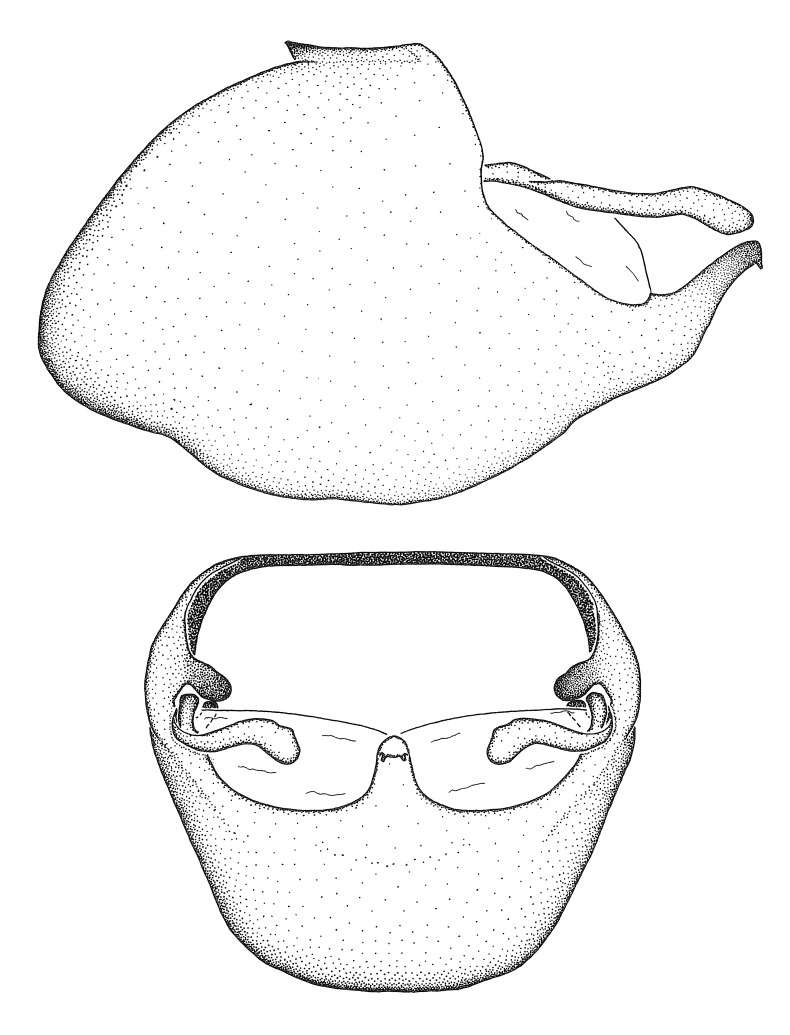
Zelus annulosus (Stål, 1866), pygophore, lateral and posterior view

**Figure 26b.**
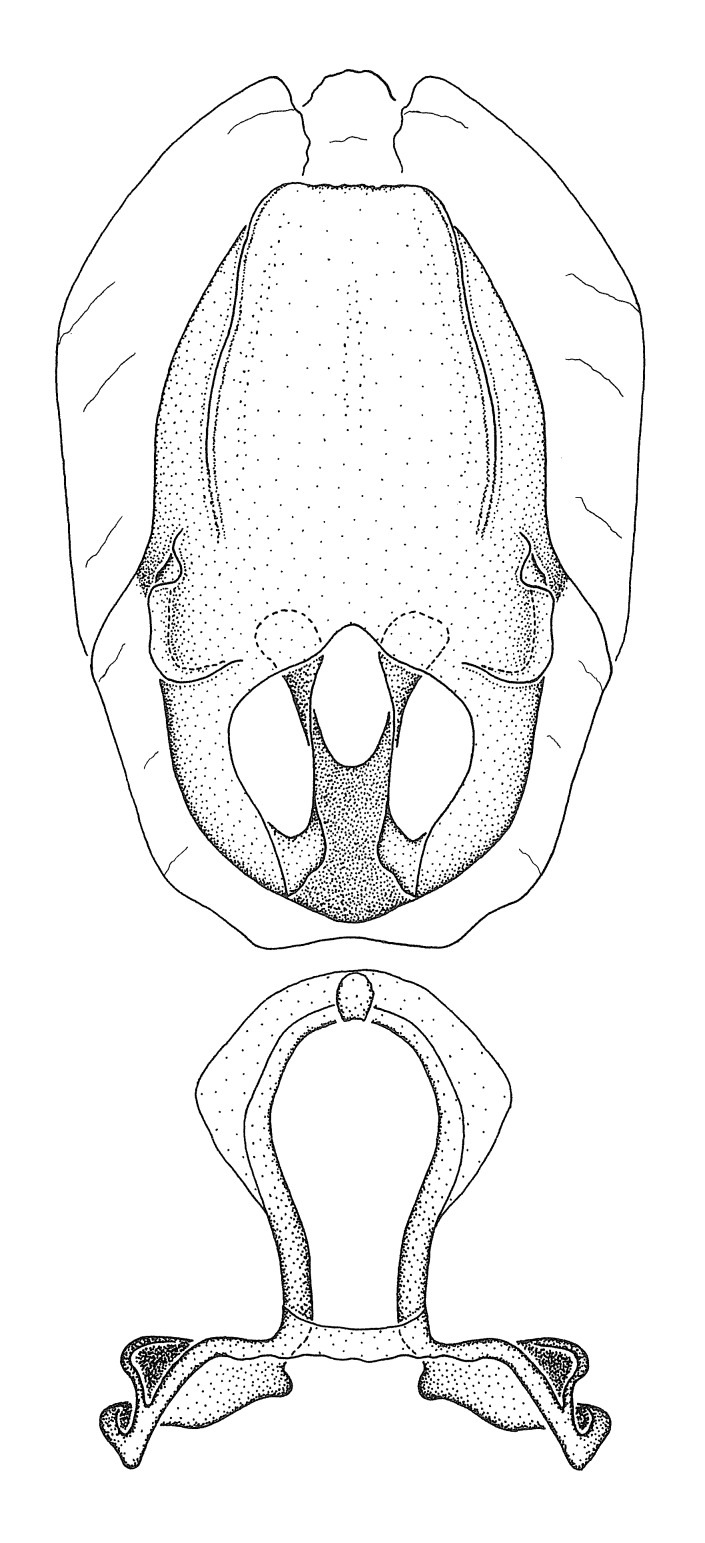
Zelus annulosus (Stål, 1866), phallus, dorsal view

**Figure 28a.**
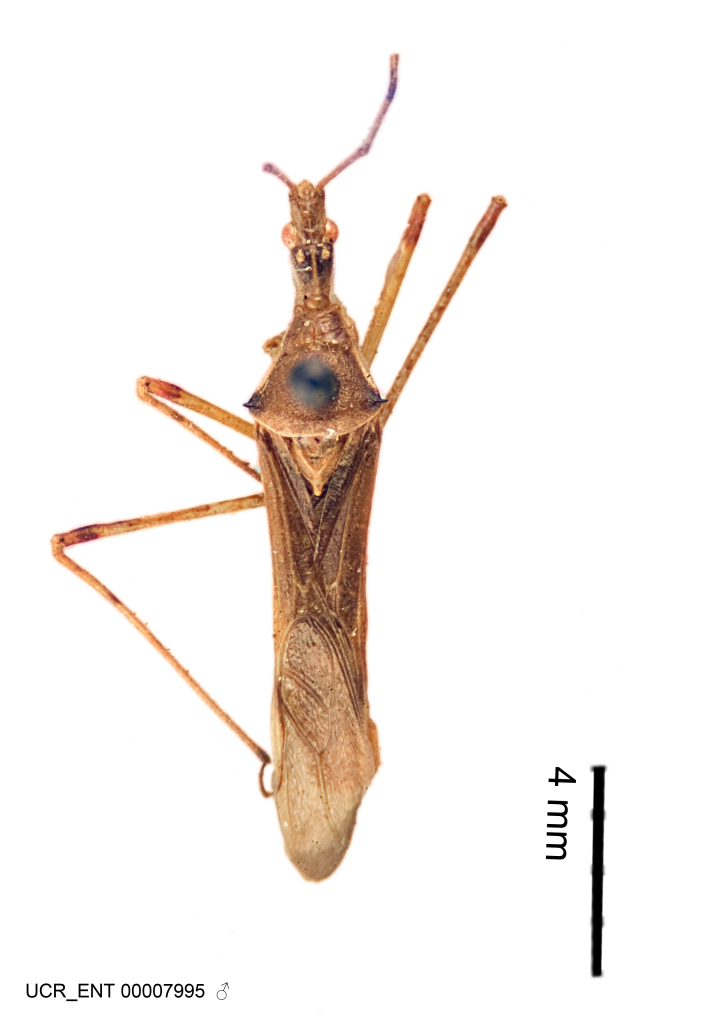
*Zelus antiguensis* Zhang & Hart, sp. n., male, dorsal (UCR_ENT 00007995, Sacatepequez, Guatemala)

**Figure 28b.**
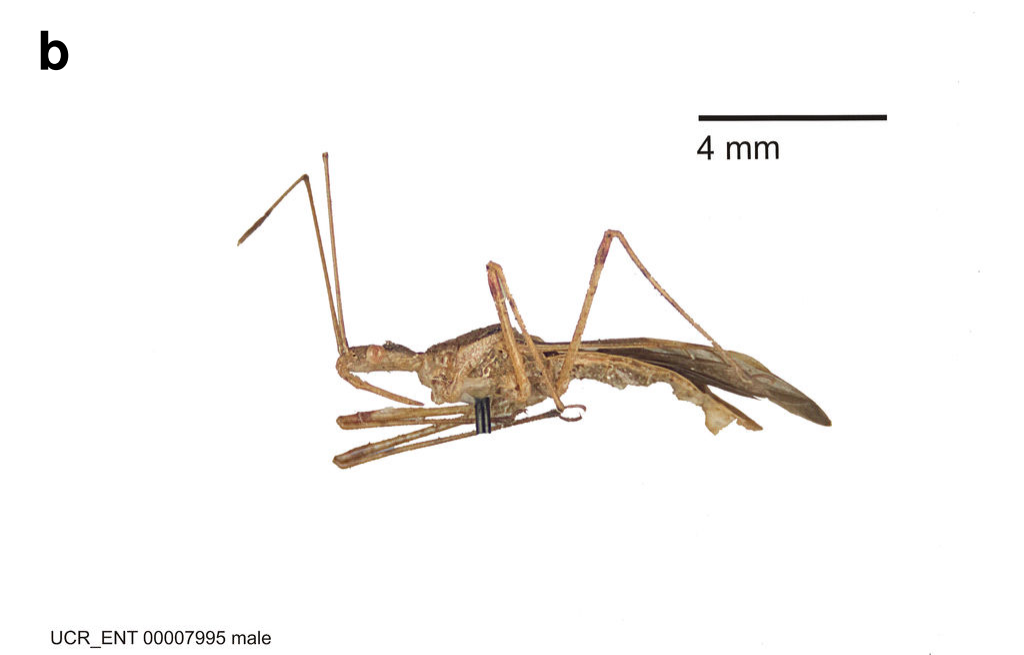
*Zelus antiguensis* Zhang & Hart, sp. n., male, lateral (UCR_ENT 00007995, Sacatepequez, Guatemala)

**Figure 28c.**
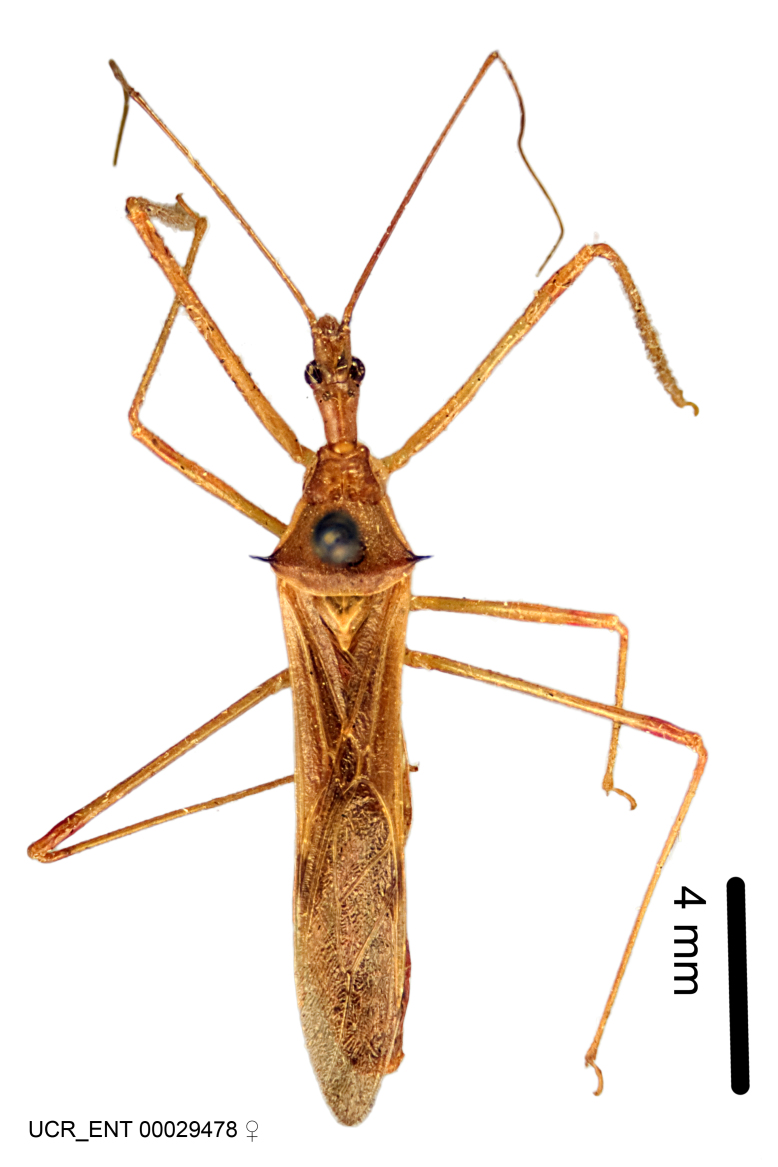
*Zelus antiguensis* Zhang & Hart, sp. n., female, lateral (UCR_ENT 00029478, Sacatepequez, Guatemala)

**Figure 28d.**
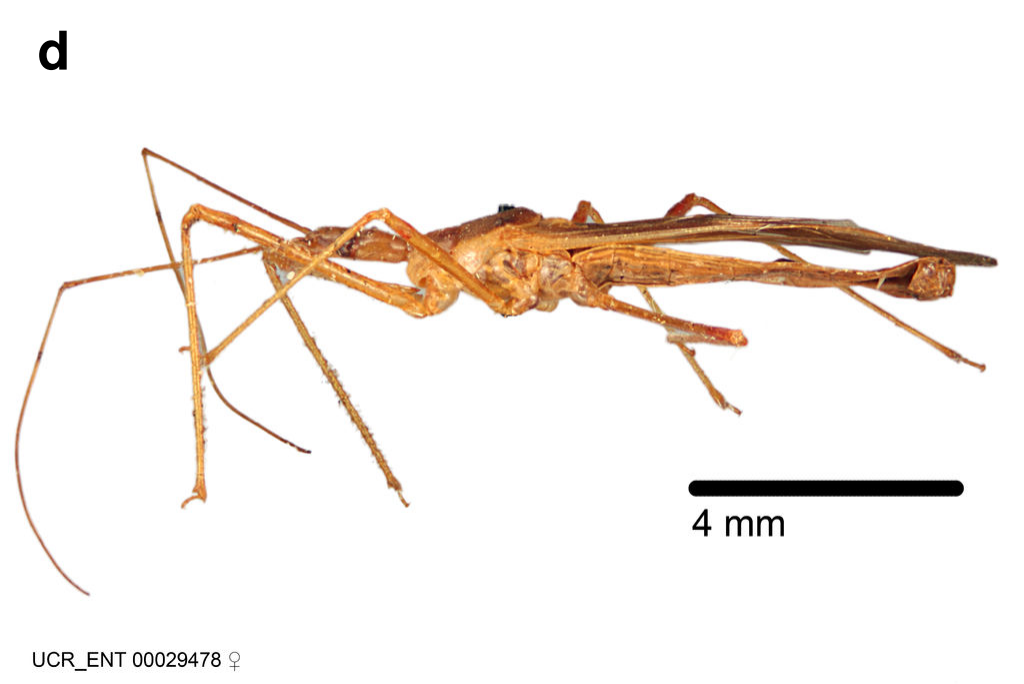
*Zelus antiguensis* Zhang & Hart, sp. n., female, lateral (UCR_ENT 00029478, Sacatepequez, Guatemala)

**Figure 29a.**
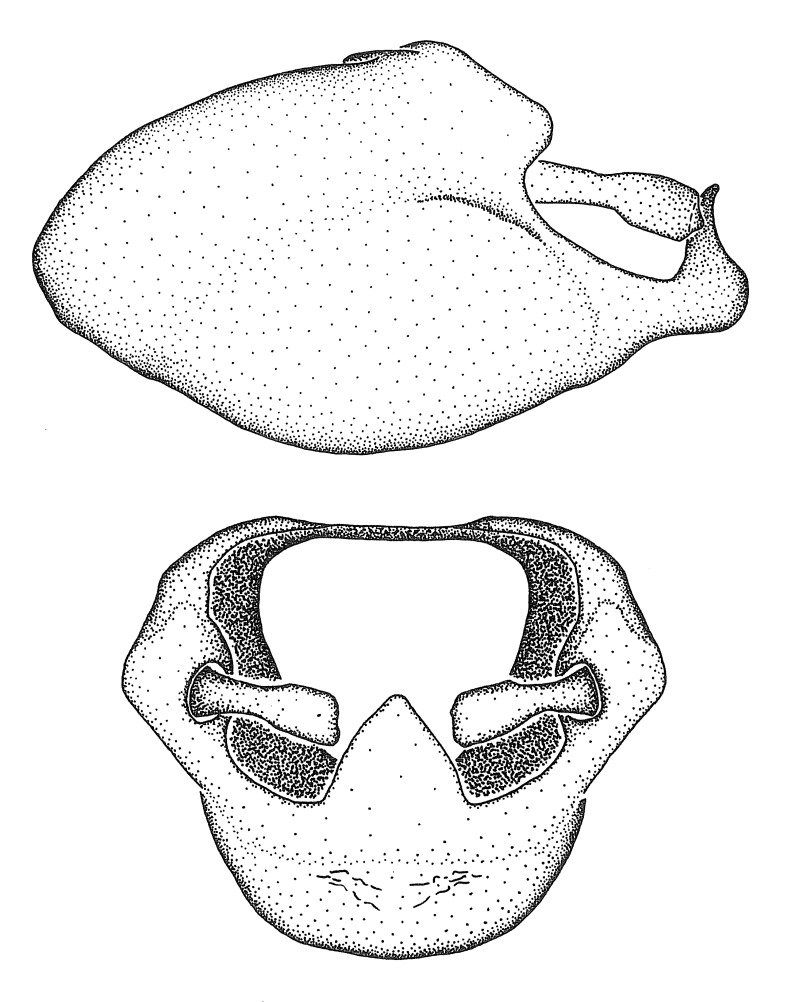
*Zelus antiguensis* Zhang & Hart, sp. n., pygophore, lateral and posterior views

**Figure 29b.**
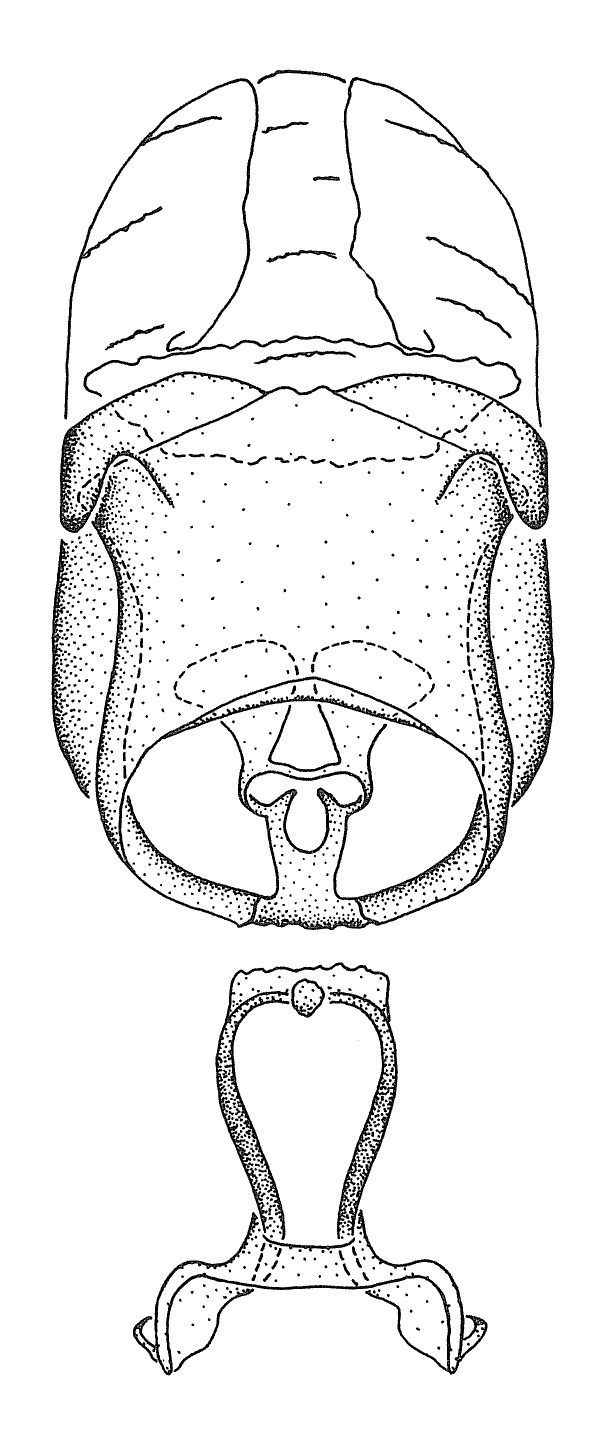
*Zelus antiguensis* Zhang & Hart, sp. n., phallus, dorsal view

**Figure 31a.**
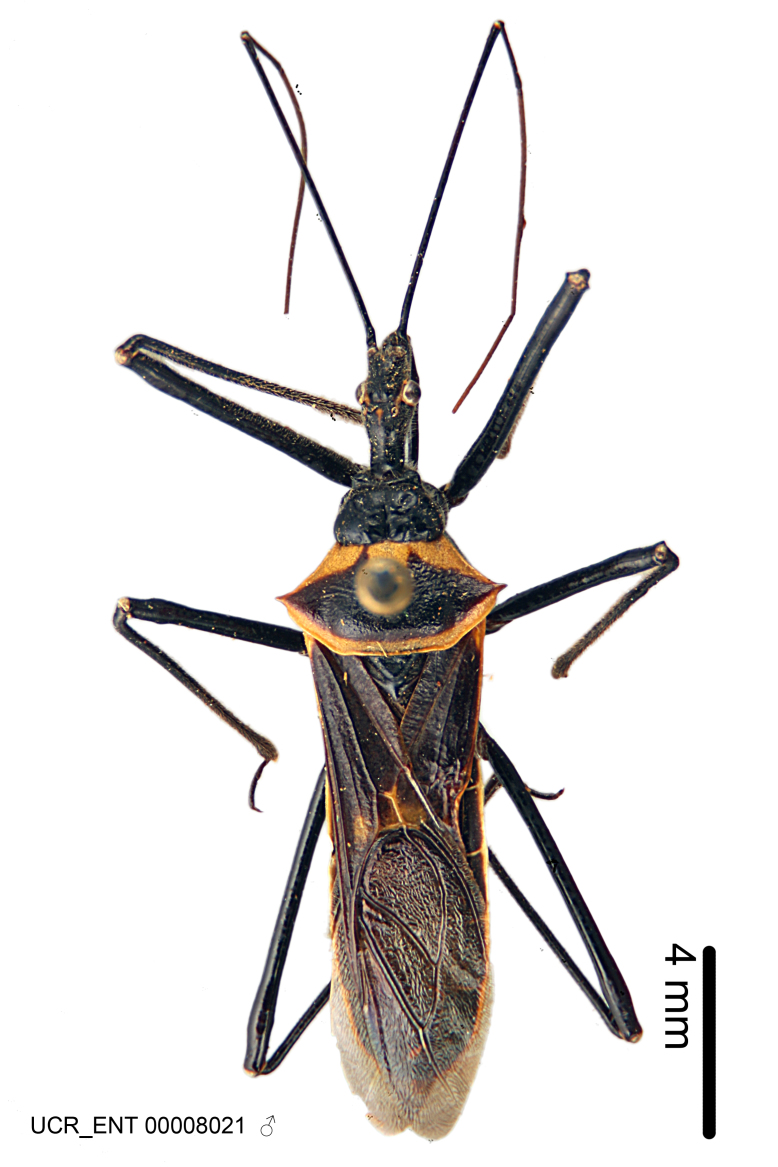
*Zelus armillatus* (Lepeletier & Serville, 1825) male, dorsal (UCR_ENT 00008021, Santa Catarina, Brazil)

**Figure 31b.**
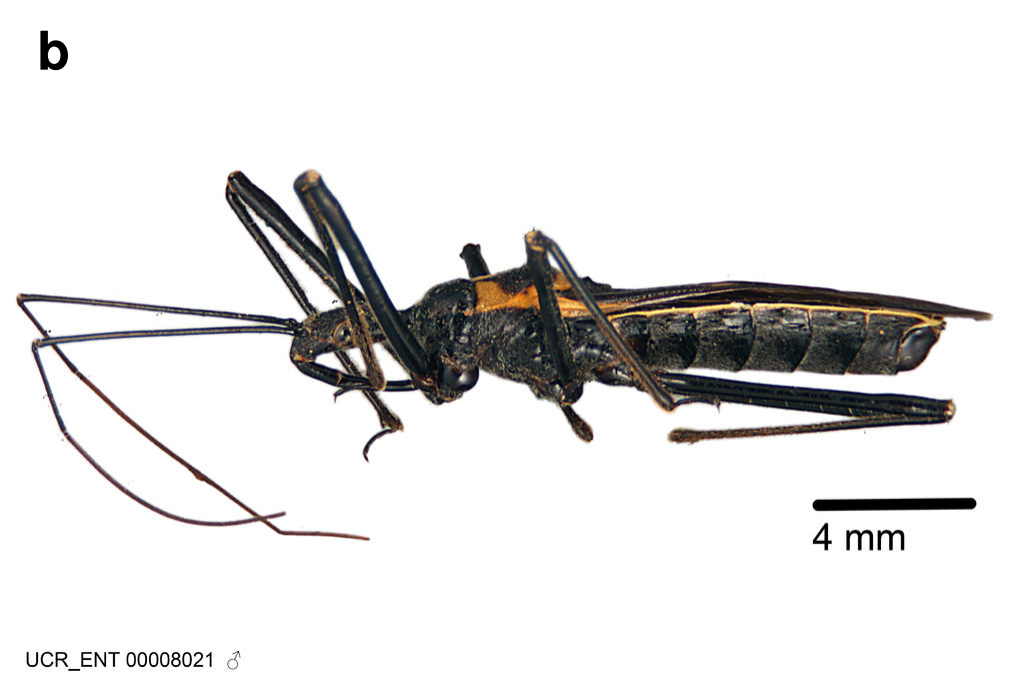
*Zelus armillatus* (Lepeletier & Serville, 1825) male, lateral (UCR_ENT 00008021, Santa Catarina, Brazil)

**Figure 31c.**
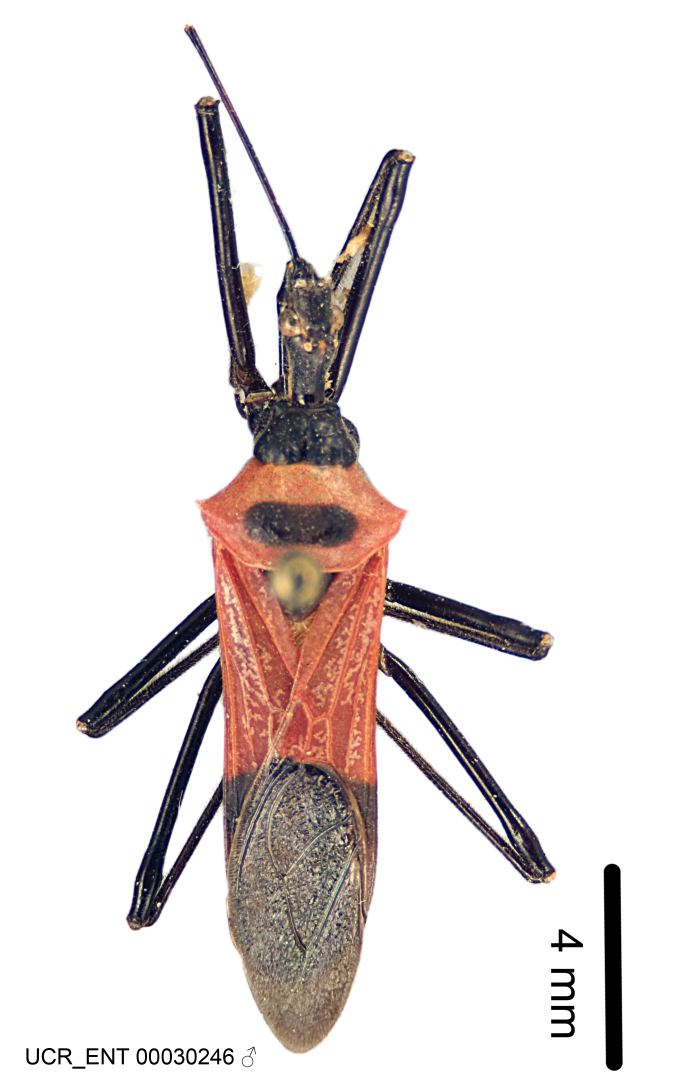
*Zelus armillatus* (Lepeletier & Serville, 1825) male, dorsal (UCR_ENT 00030246, Santa Catarina, Brazil)

**Figure 31d.**
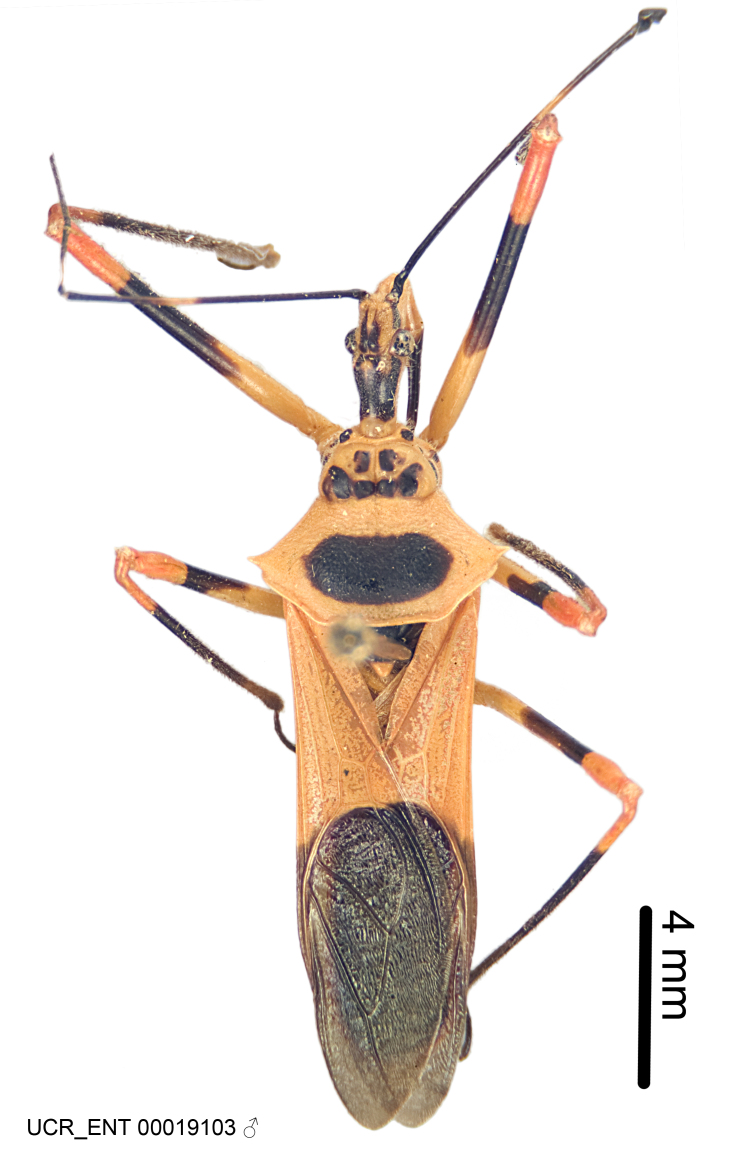
*Zelus armillatus* (Lepeletier & Serville, 1825) male, dorsal (UCR_ENT 00019103, Santa Catarina, Brazil)

**Figure 32a.**
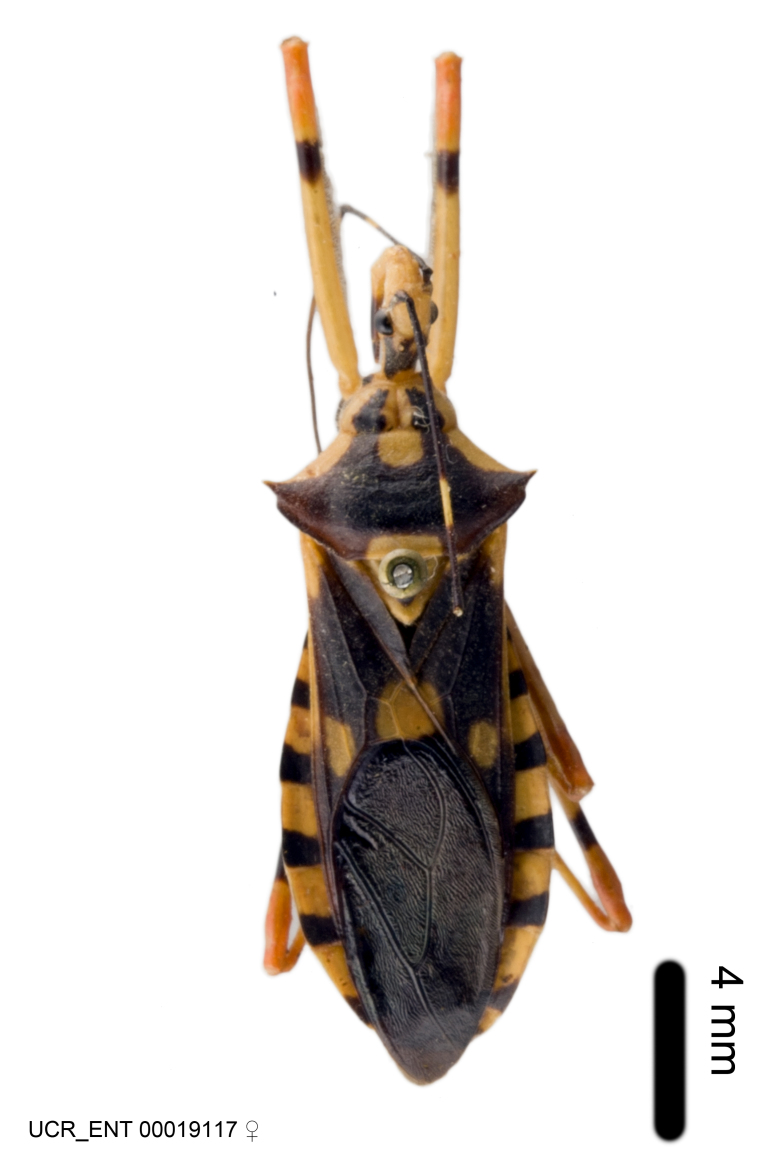
*Zelus armillatus* (Lepeletier & Serville, 1825), female, dorsal (UCR_ENT 00019117, Huanuco, Peru)

**Figure 32b.**
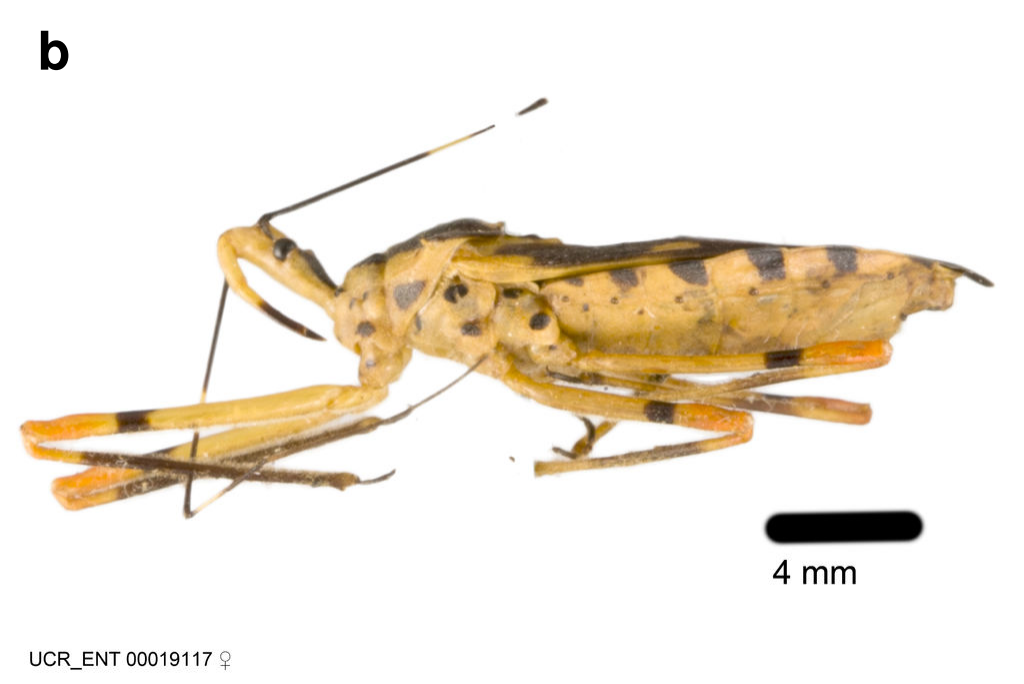
*Zelus armillatus* (Lepeletier & Serville, 1825), female, lateral (UCR_ENT 00019117, Huanuco, Peru)

**Figure 32c.**
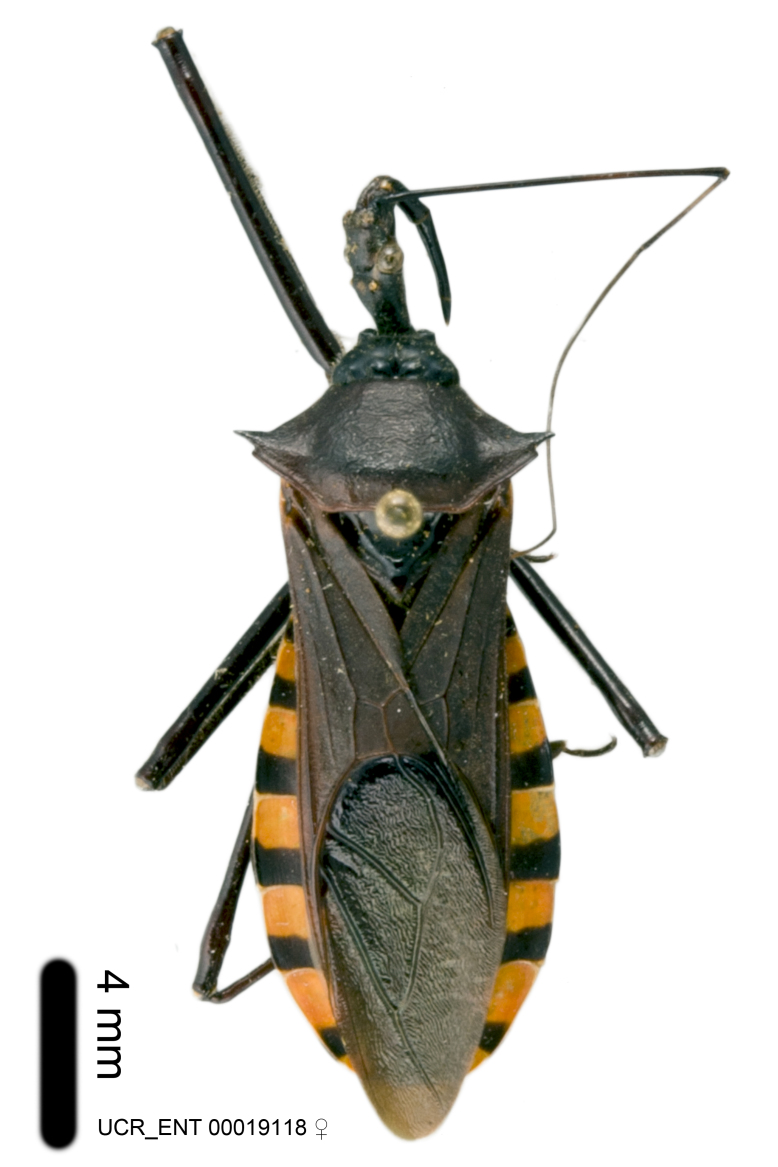
*Zelus armillatus* (Lepeletier & Serville, 1825), female, dorsal (UCR_ENT 00019118, Huanuco, Peru)

**Figure 32d.**
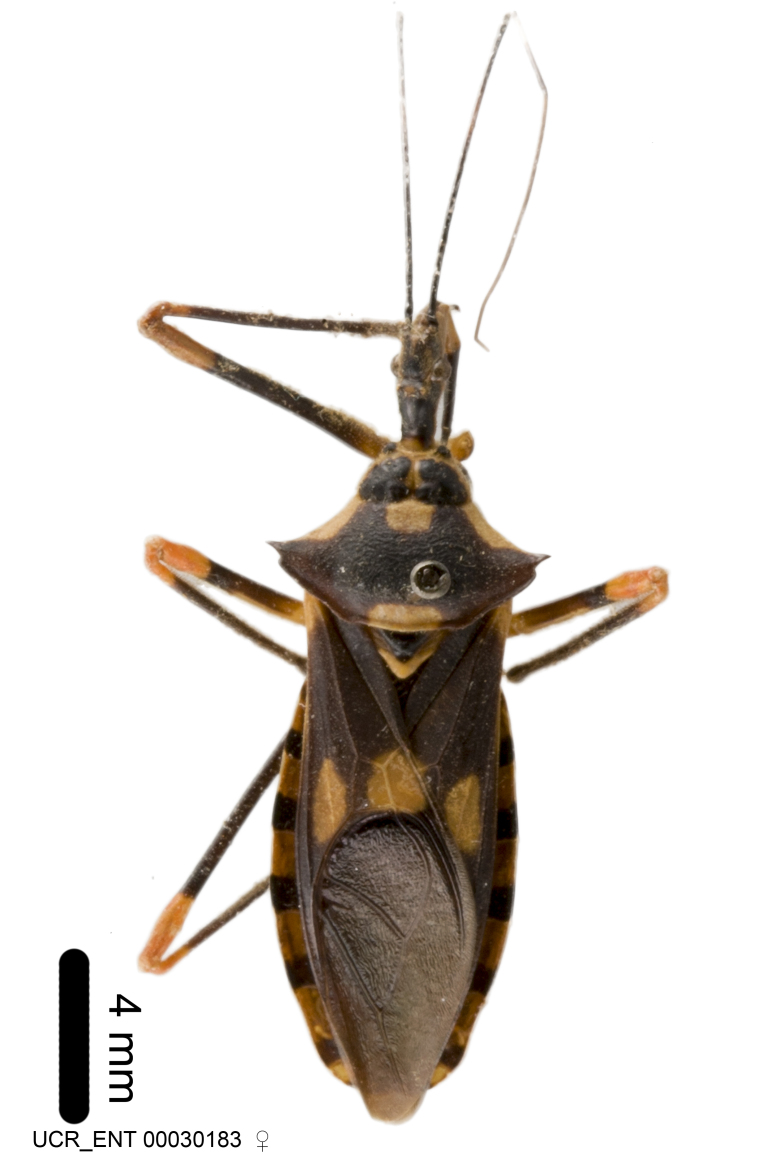
*Zelus armillatus* (Lepeletier & Serville, 1825), female, dorsal (UCR_ENT 00030183, Junin, Peru)

**Figure 32e.**
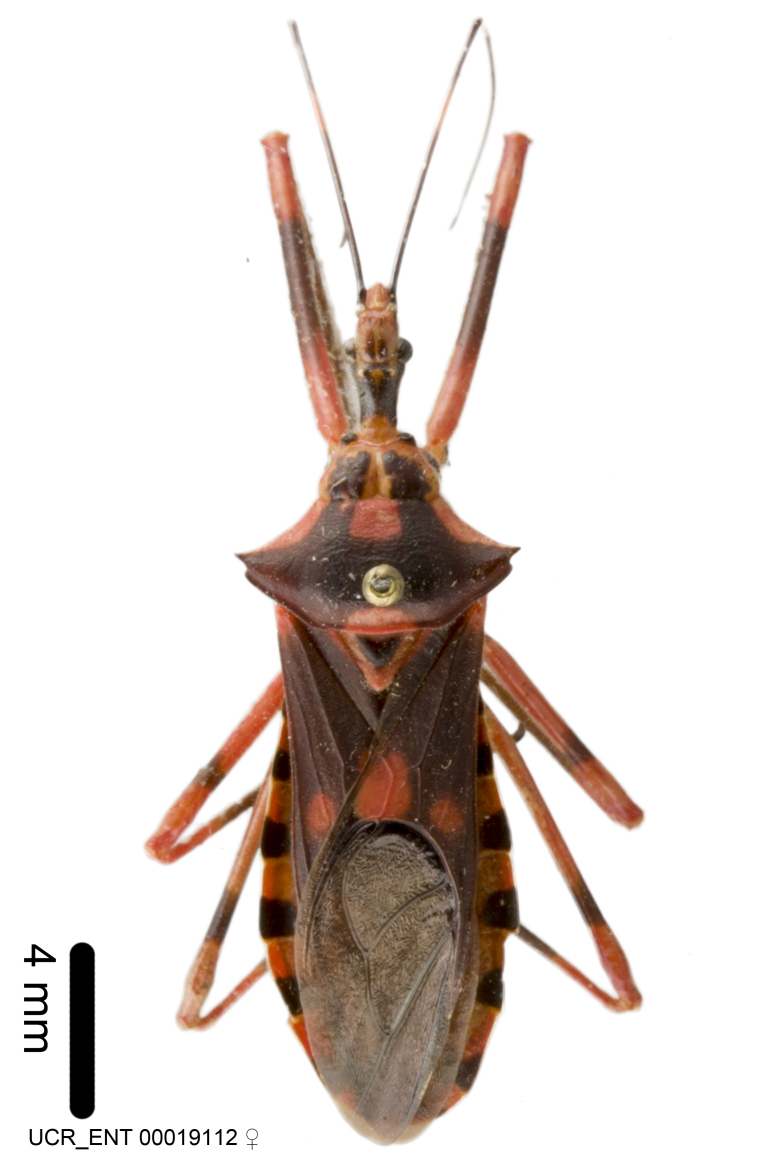
*Zelus armillatus* (Lepeletier & Serville, 1825), female, dorsal (UCR_ENT 00019112, Huanuco, Peru)

**Figure 32f.**
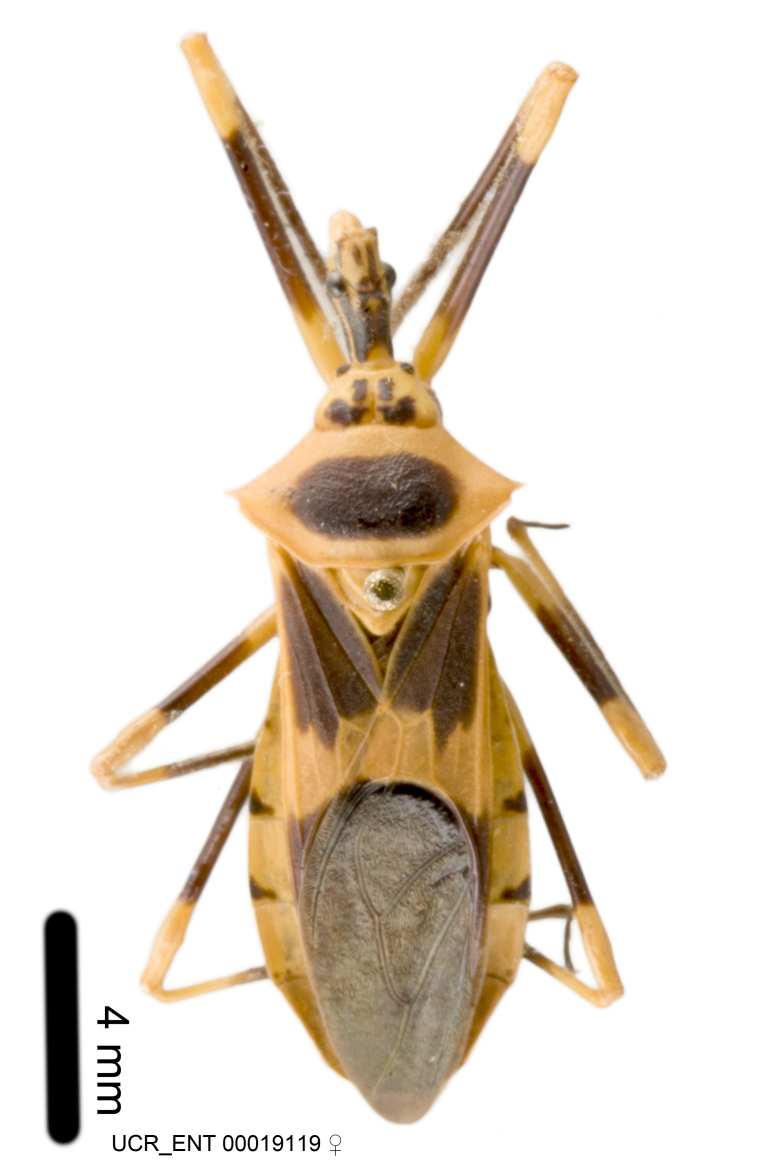
*Zelus armillatus* (Lepeletier & Serville, 1825), female, dorsal (UCR_ENT 00019119, Huanuco, Peru)

**Figure 33a.**
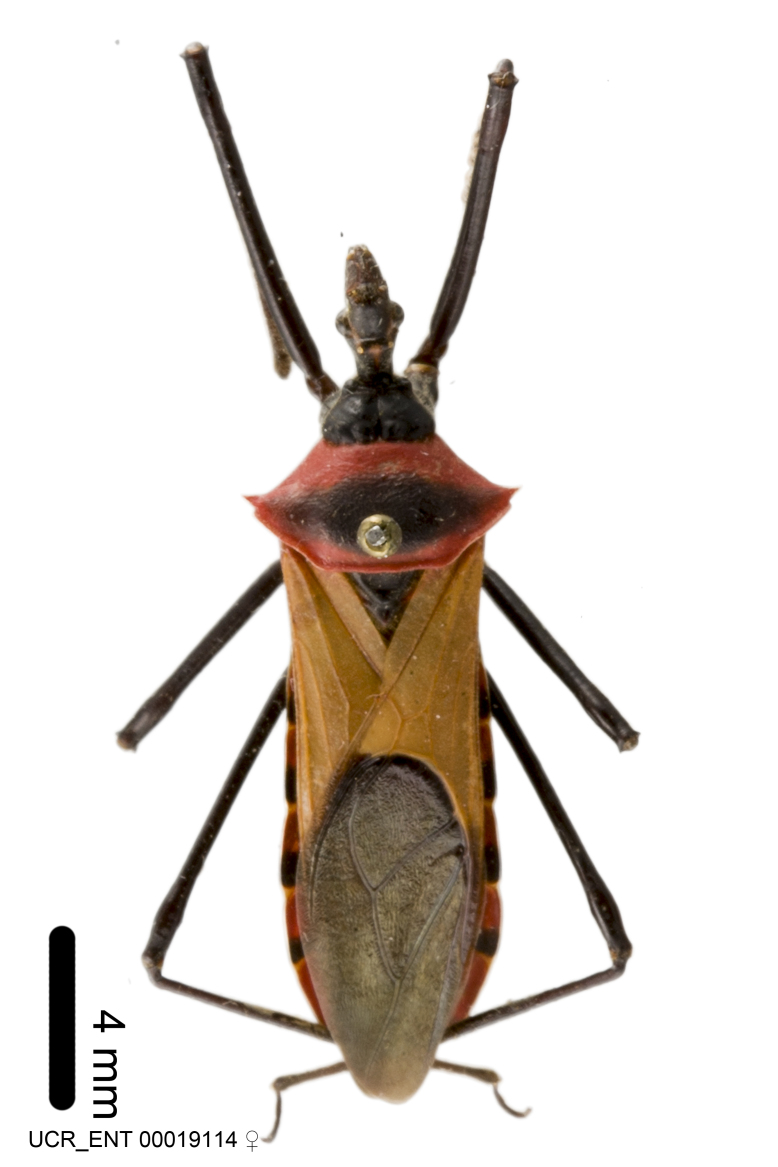
*Zelus armillatus* (Lepeletier & Serville, 1825), female, dorsal (UCR_ENT 00019114, Huanuco, Peru)

**Figure 33b.**
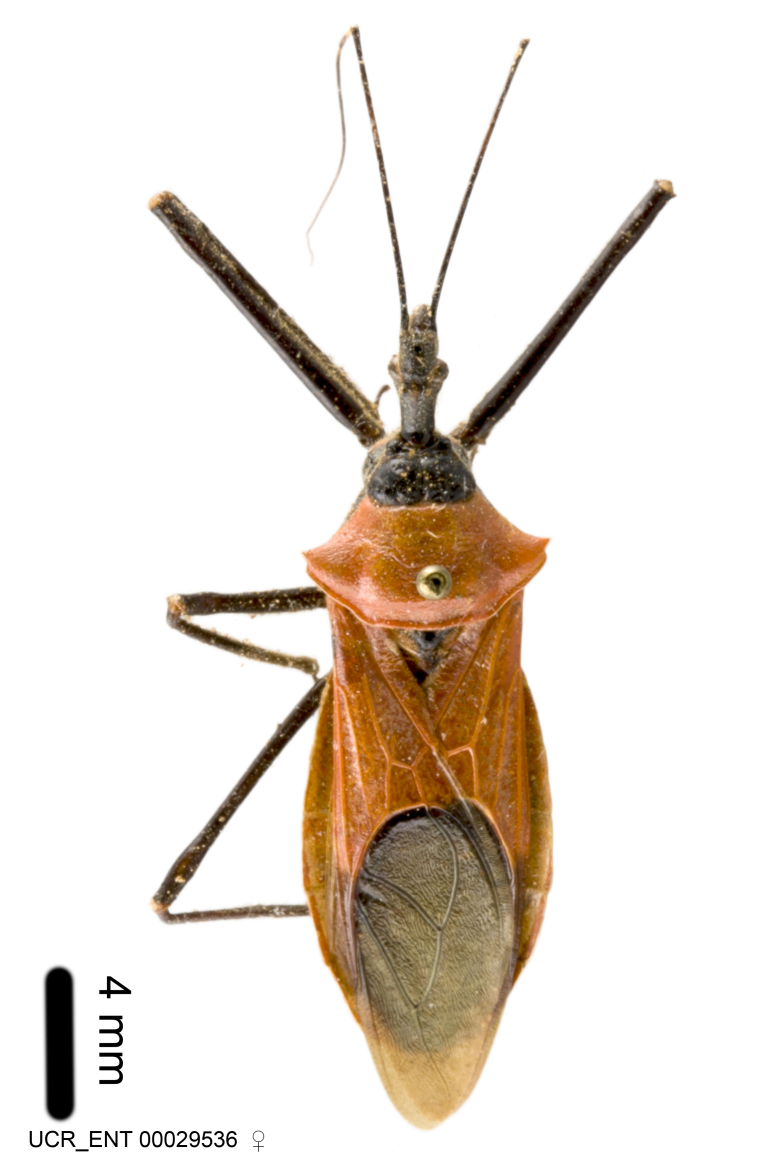
*Zelus armillatus* (Lepeletier & Serville, 1825), female, dorsal (UCR_ENT 00029536, Junin, Peru)

**Figure 33c.**
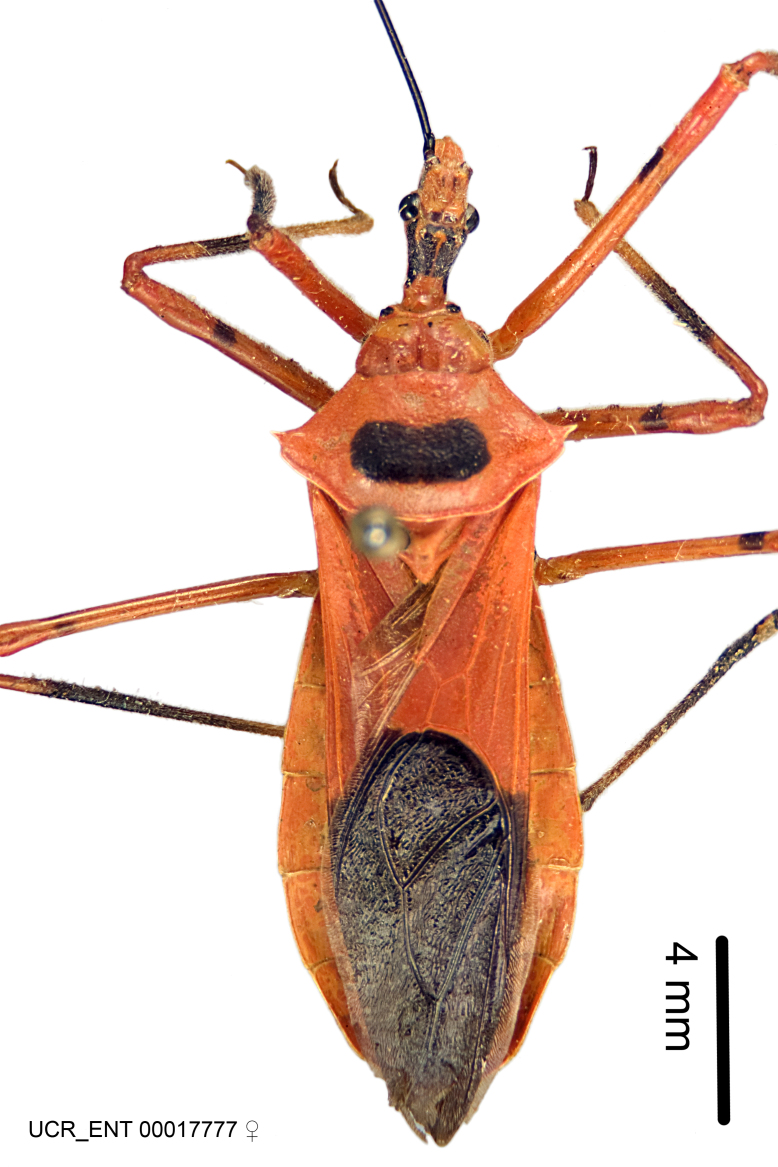
*Zelus armillatus* (Lepeletier & Serville, 1825), female, dorsal (UCR_ENT 00017777, Huanuco, Peru)

**Figure 33d.**
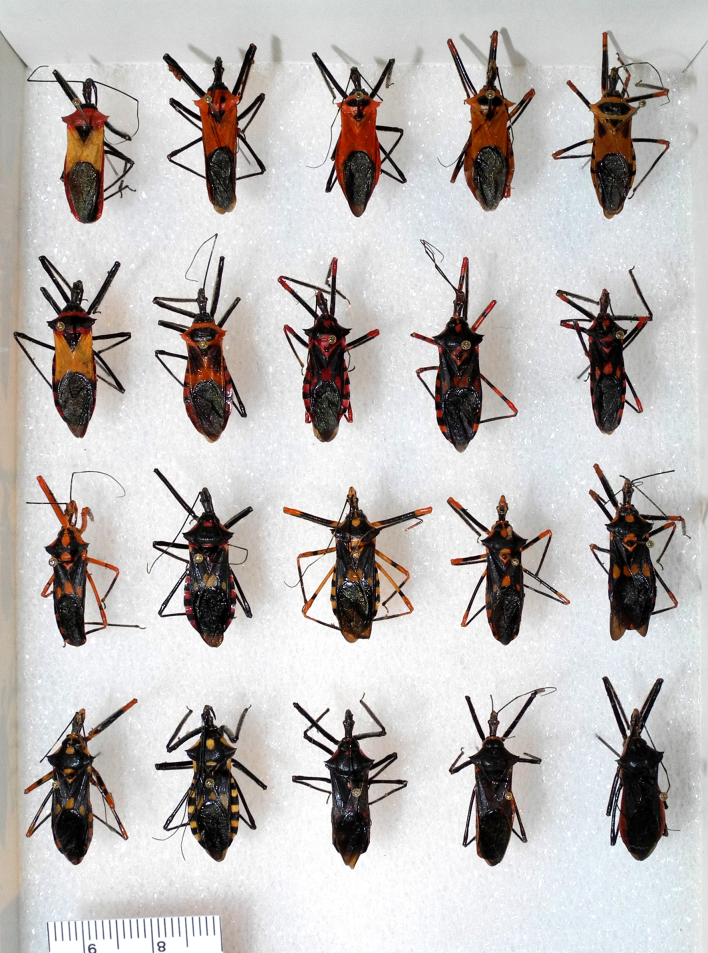
*Zelus armillatus* (Lepeletier & Serville, 1825). Female specimens collected from Oct-Nov 1993 from Guanay, La Paz, Bolivia, showing a large range of color variations

**Figure 34a.**
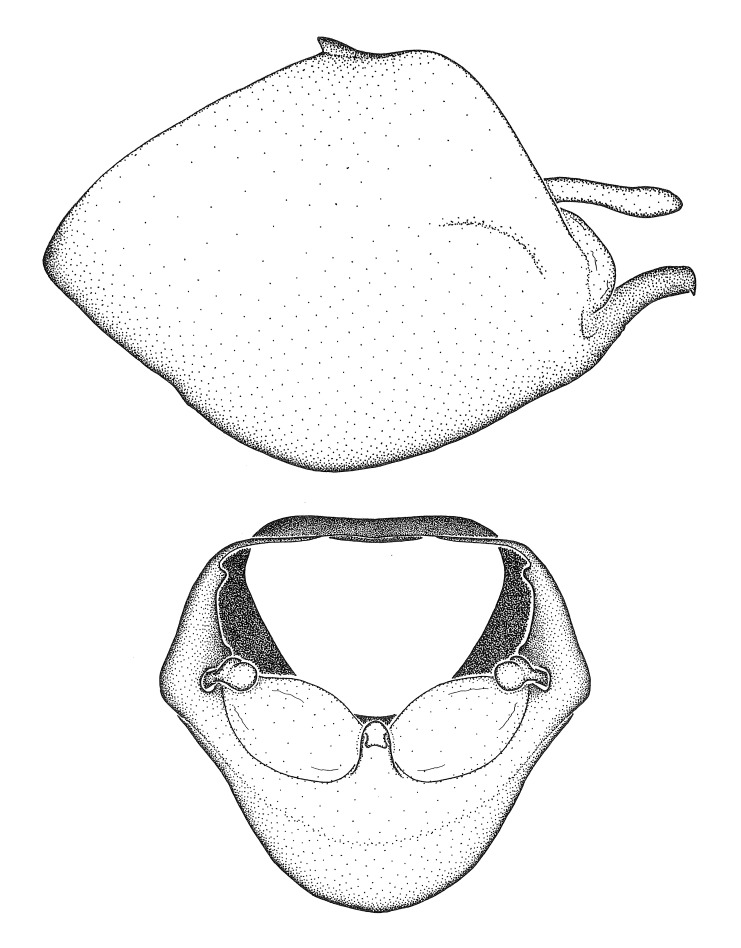
*Zelus armillatus* (Lepeletier & Serville, 1825), pygophore, lateral and posterior views

**Figure 34b.**
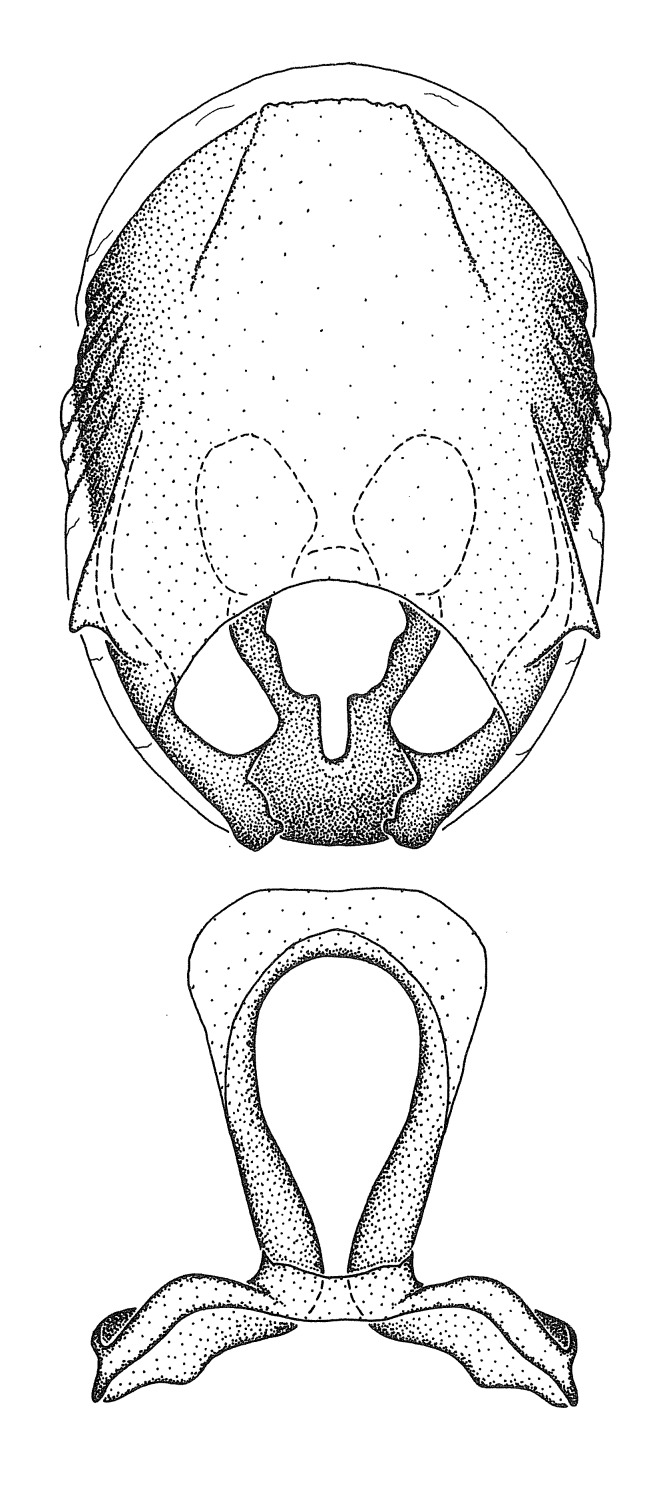
*Zelus armillatus* (Lepeletier & Serville, 1825), phallus, dorsal

**Figure 36a.**
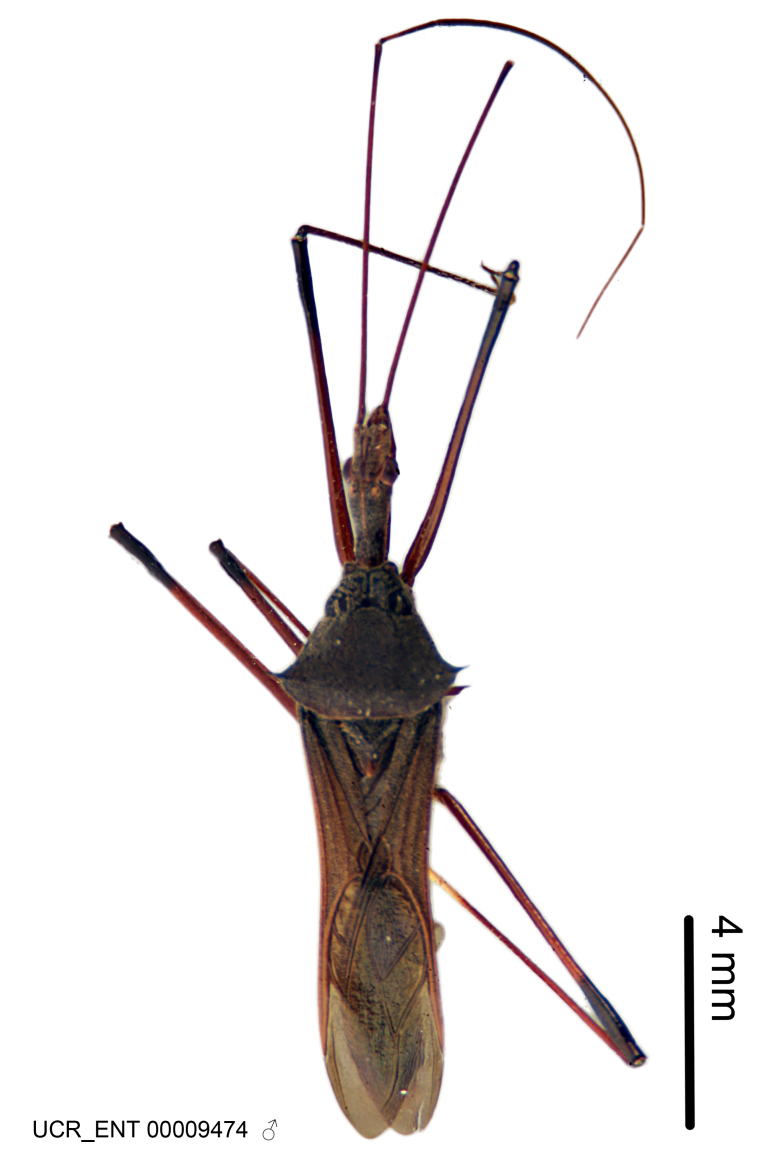
*Zelus auralanus* Zhang & Hart, sp. n., male, dorsal (UCR_ENT 00009474, Orellana, Ecuador)

**Figure 36b.**
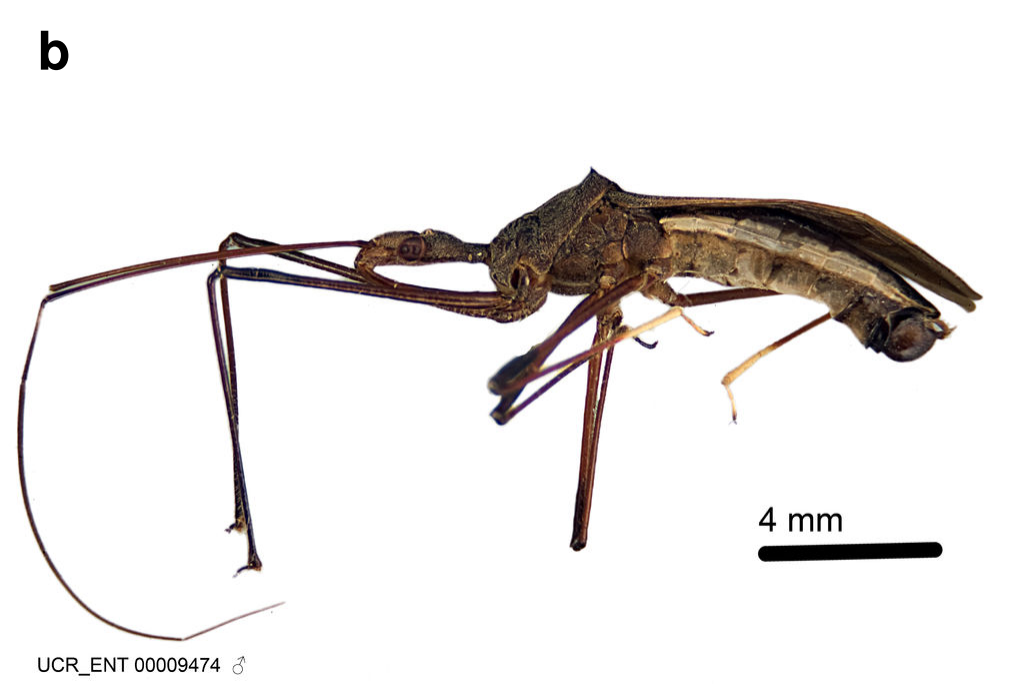
*Zelus auralanus* Zhang & Hart, sp. n., male, lateral (UCR_ENT 00009474, Orellana, Ecuador)

**Figure 36c.**
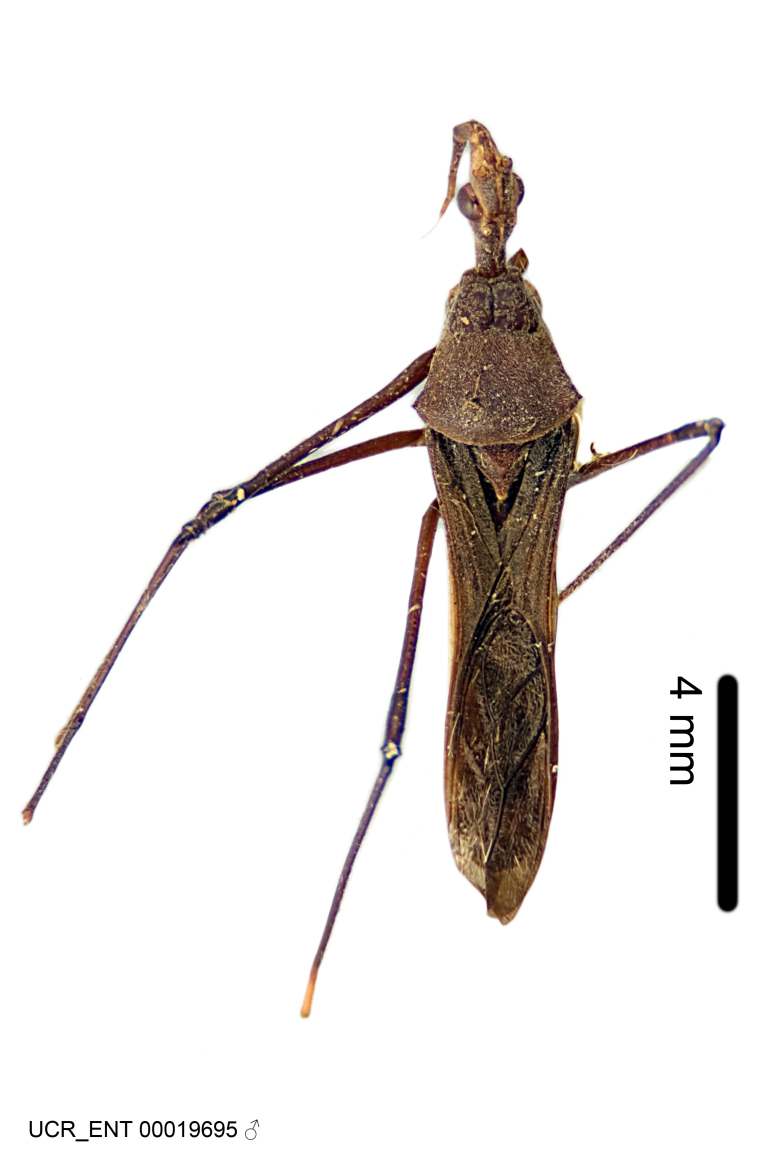
*Zelus auralanus* Zhang & Hart, sp. n., male, dorsal (UCR_ENT 00019695, Mato Grosso, Brazil)

**Figure 36d.**
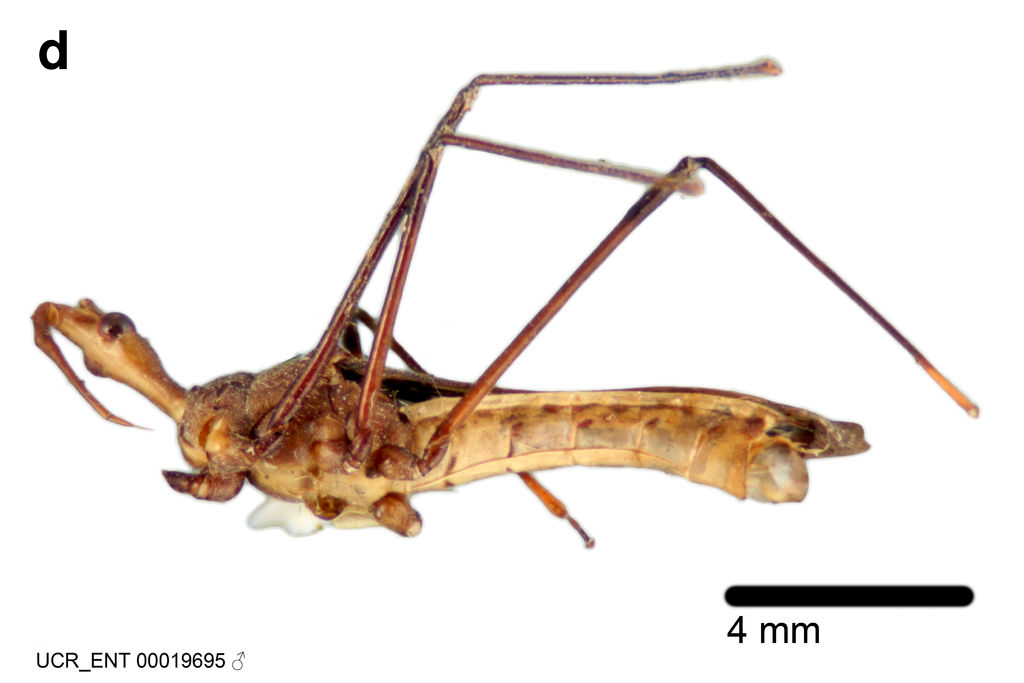
*Zelus auralanus* Zhang & Hart, sp. n., male, lateral (UCR_ENT 00019695, Mato Grosso, Brazil)

**Figure 36e.**
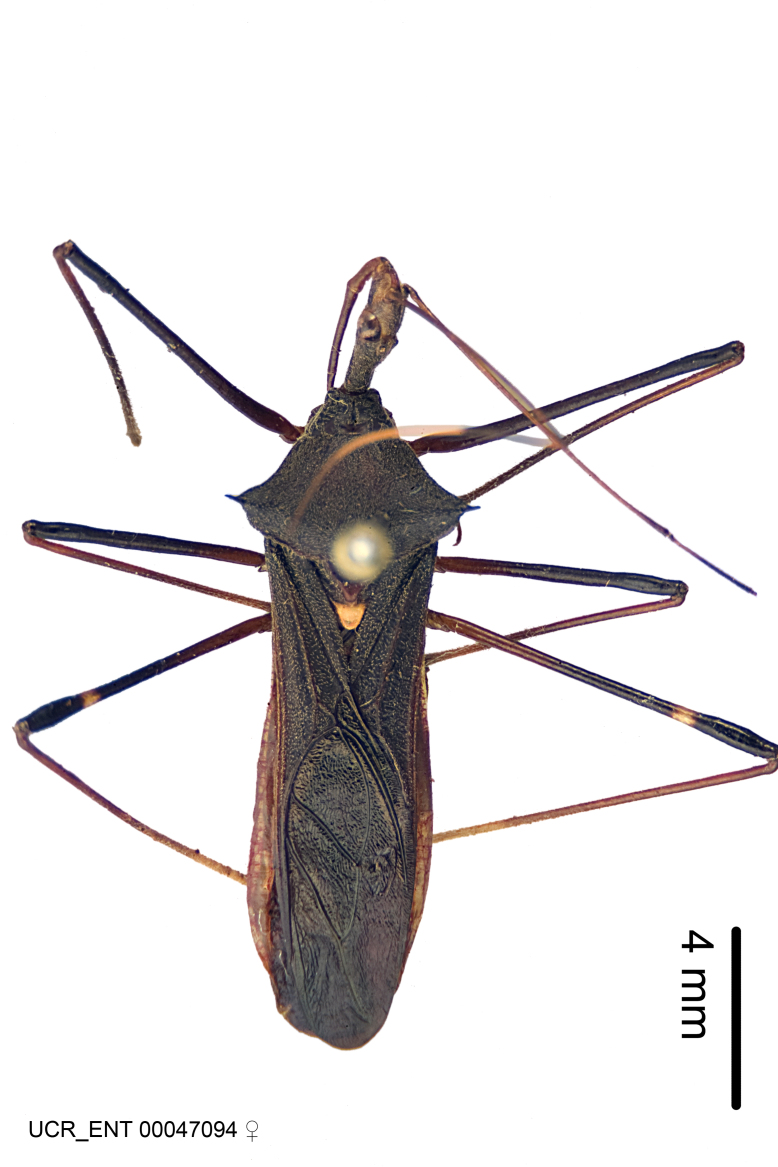
*Zelus auralanus* Zhang & Hart, sp. n., female, dorsal (UCR_ENT 00047094, Pastaza, Ecuador)

**Figure 36f.**
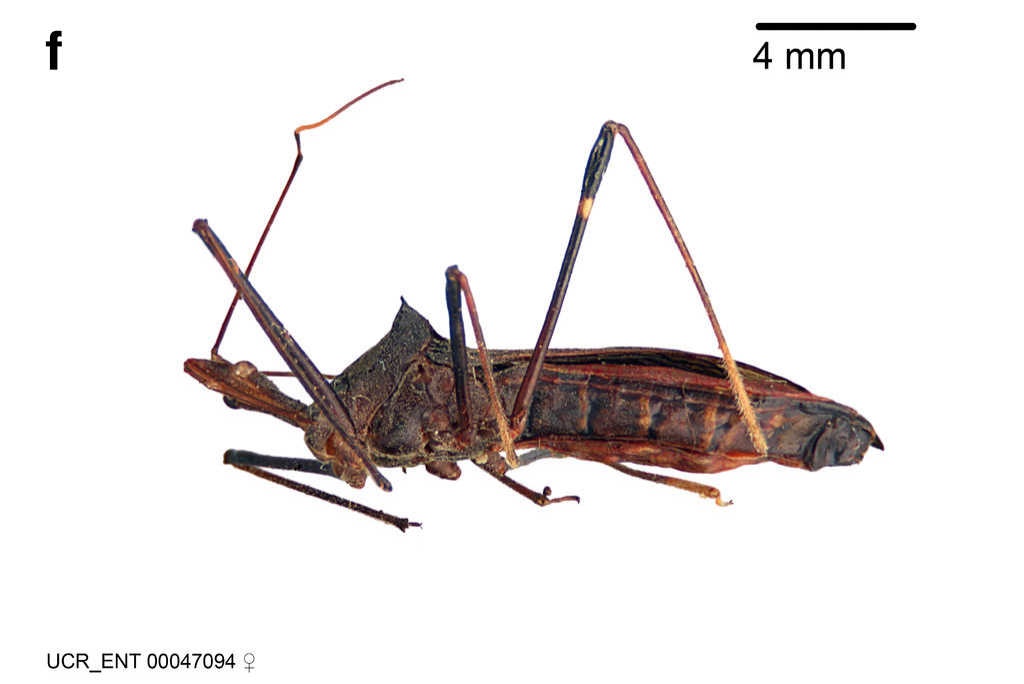
*Zelus auralanus* Zhang & Hart, sp. n., female, lateral (UCR_ENT 00047094, Pastaza, Ecuador)

**Figure 37a.**
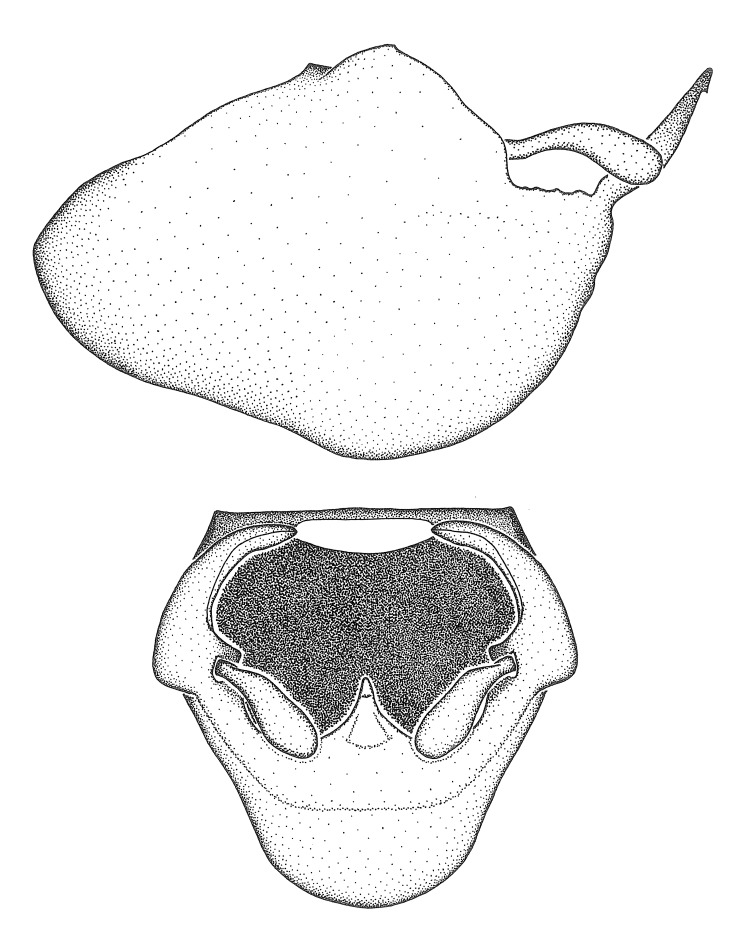
*Zelus auralanus* Zhang & Hart, sp. n., pygophore, lateral and posterior views

**Figure 37b.**
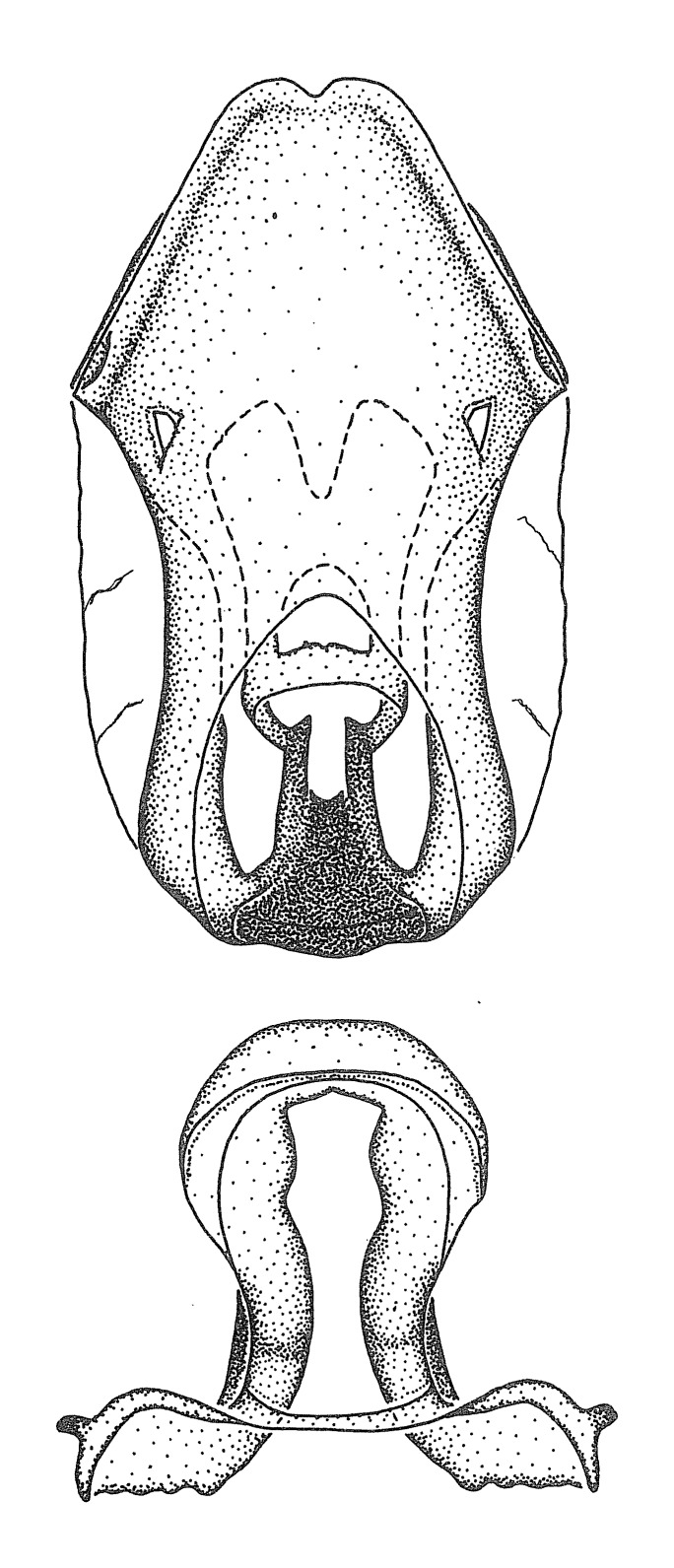
*Zelus auralanus* Zhang & Hart, sp. n., pygophore, posterior view

**Figure 39a.**
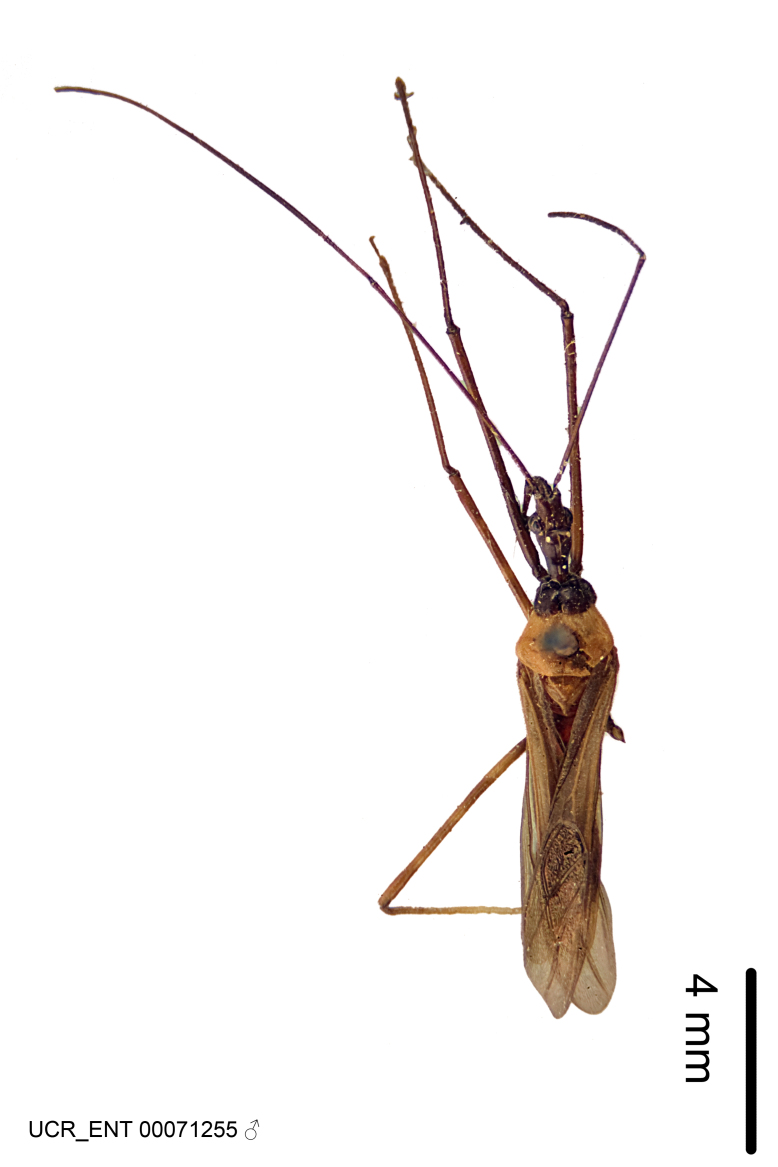
*Zelus bahiaensis* Zhang & Hart, sp. n., male, dorsal (UCR_ENT 00071255, Bahia, Brazil)

**Figure 39b.**
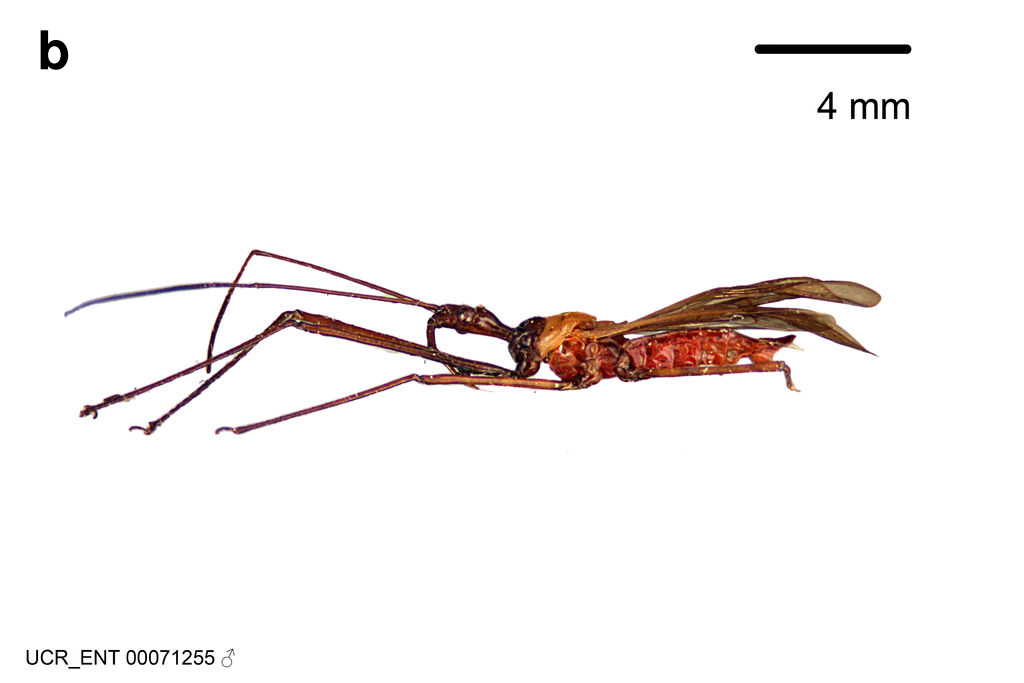
*Zelus bahiaensis* Zhang & Hart, sp. n., male, lateral (UCR_ENT 00071255, Bahia, Brazil)

**Figure 40a.**
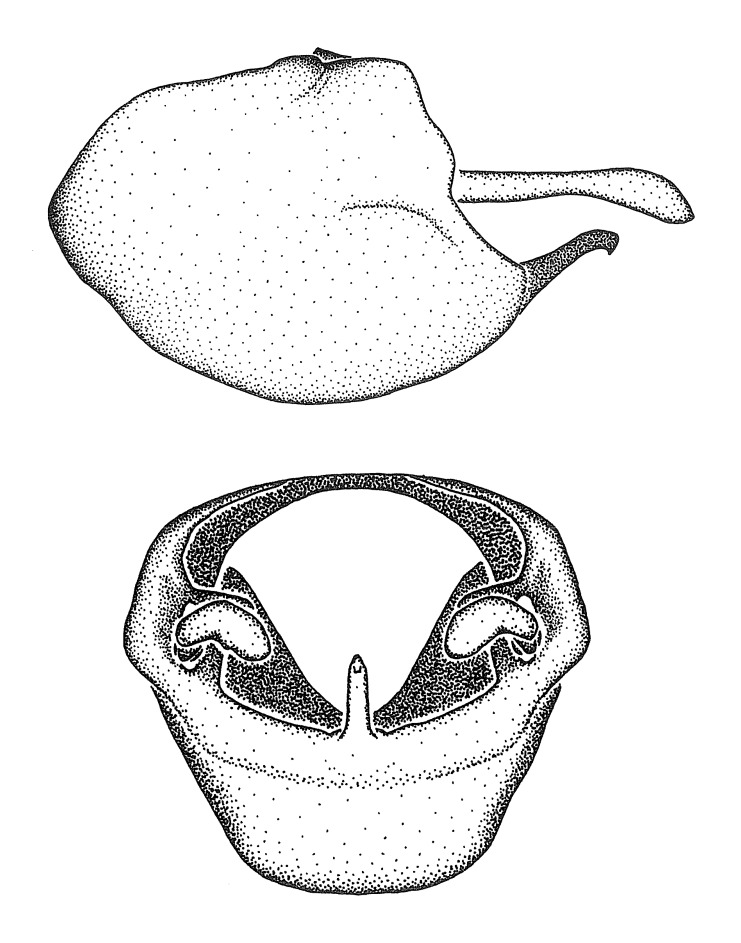
*Zelus bahiaensis* Zhang & Hart, sp. n., pygophore, lateral and posterior views

**Figure 40b.**
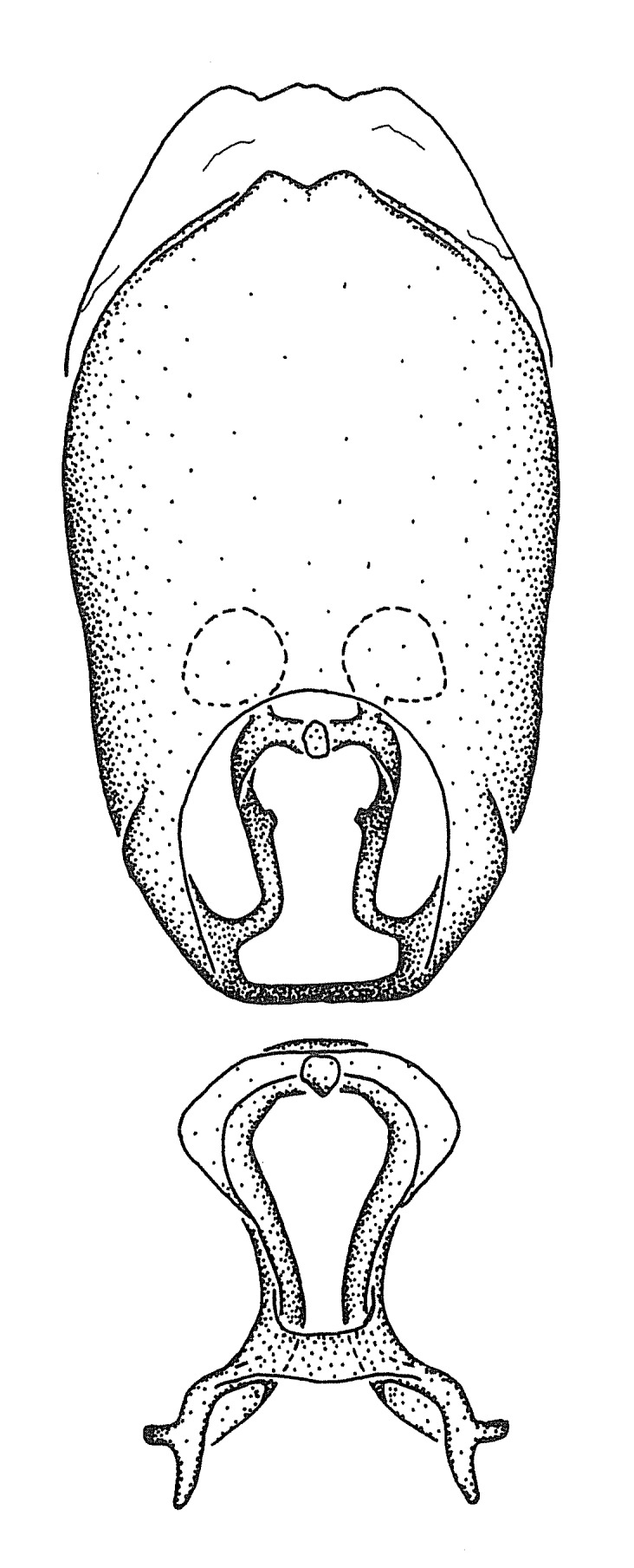
*Zelus bahiaensis* Zhang & Hart, sp. n., phallus, dorsal view

**Figure 42a.**
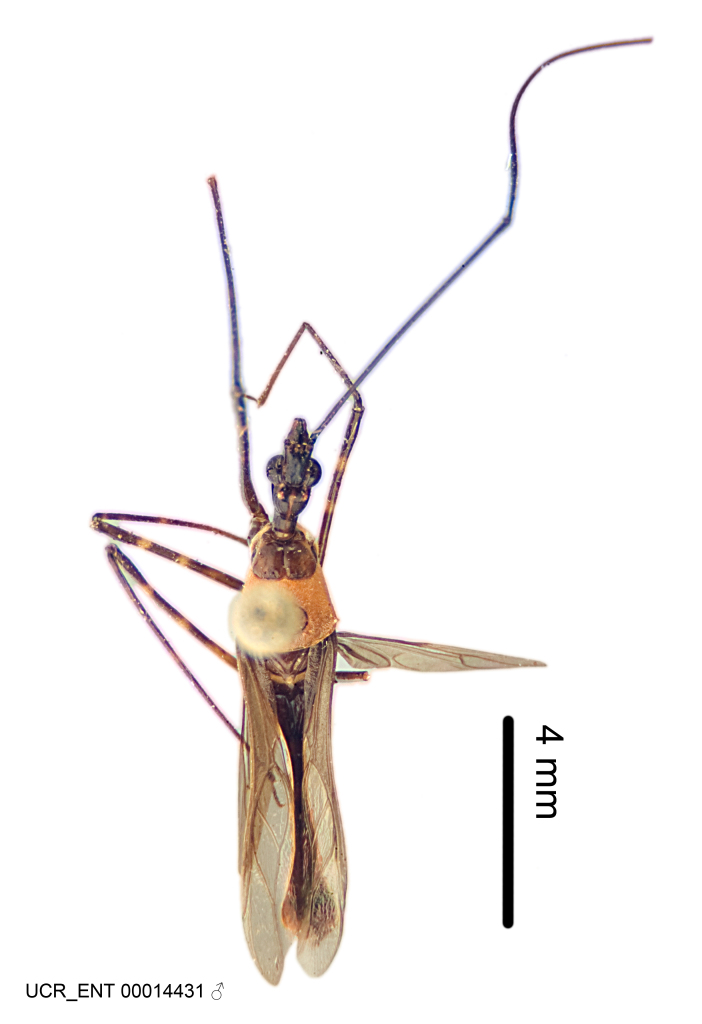
*Zelus banksi* Zhang & Hart, sp. n., male, dorsal (UCR_ENT 00014431, Puntarenas, Costa Rica)

**Figure 42b.**
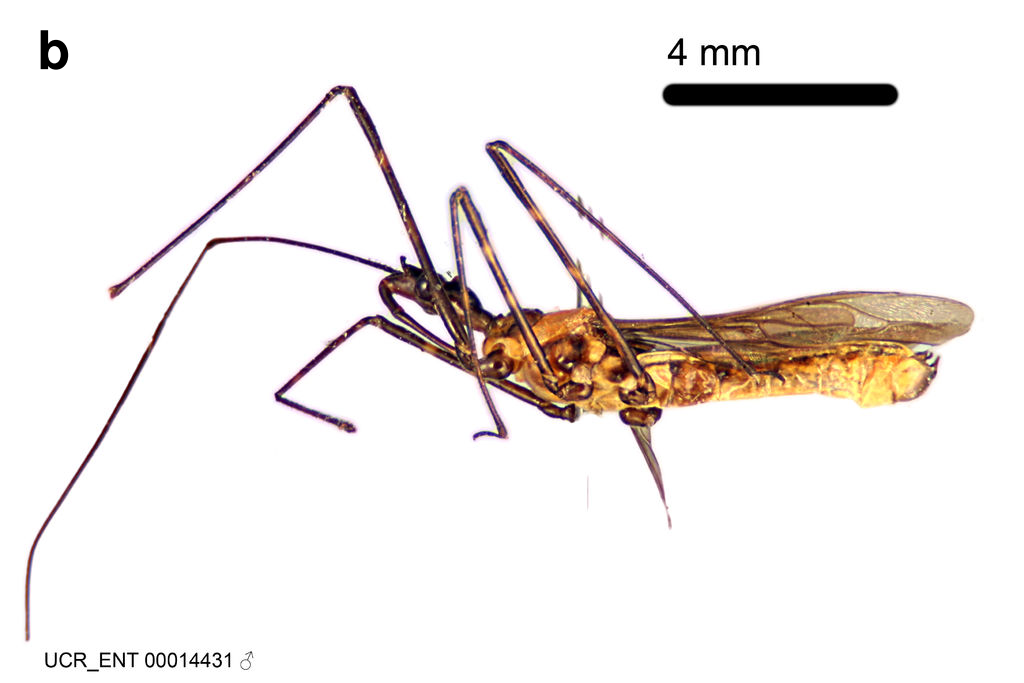
*Zelus banksi* Zhang & Hart, sp. n., male, lateral (UCR_ENT 00014431, Puntarenas, Costa Rica)

**Figure 43a.**
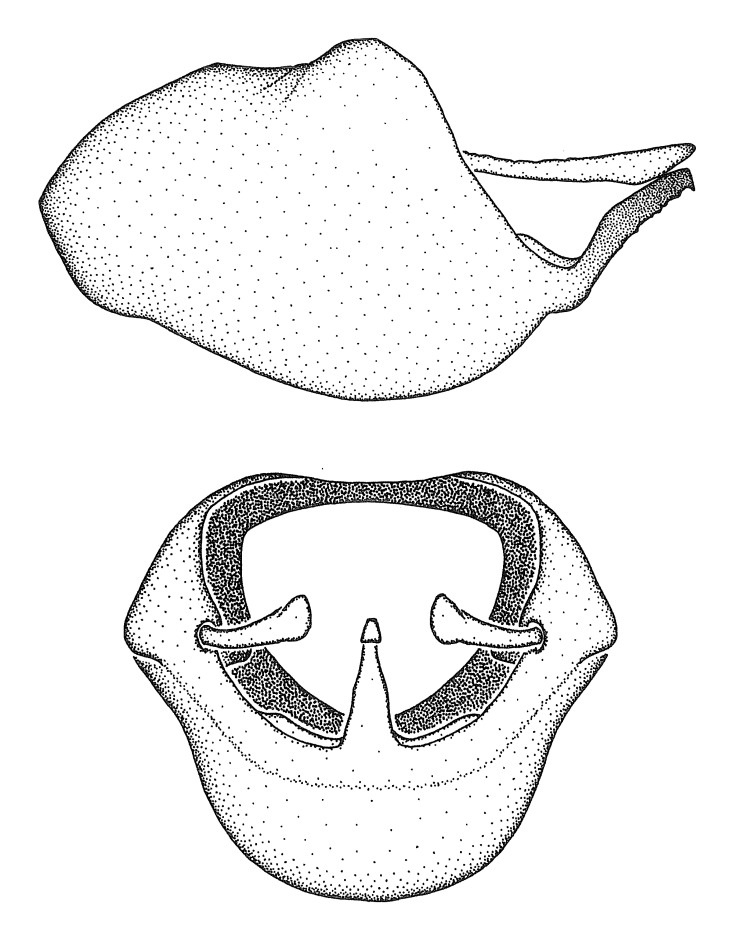
*Zelus banksi* Zhang & Hart, sp. n., pygophore, lateral and posterior views

**Figure 43b.**
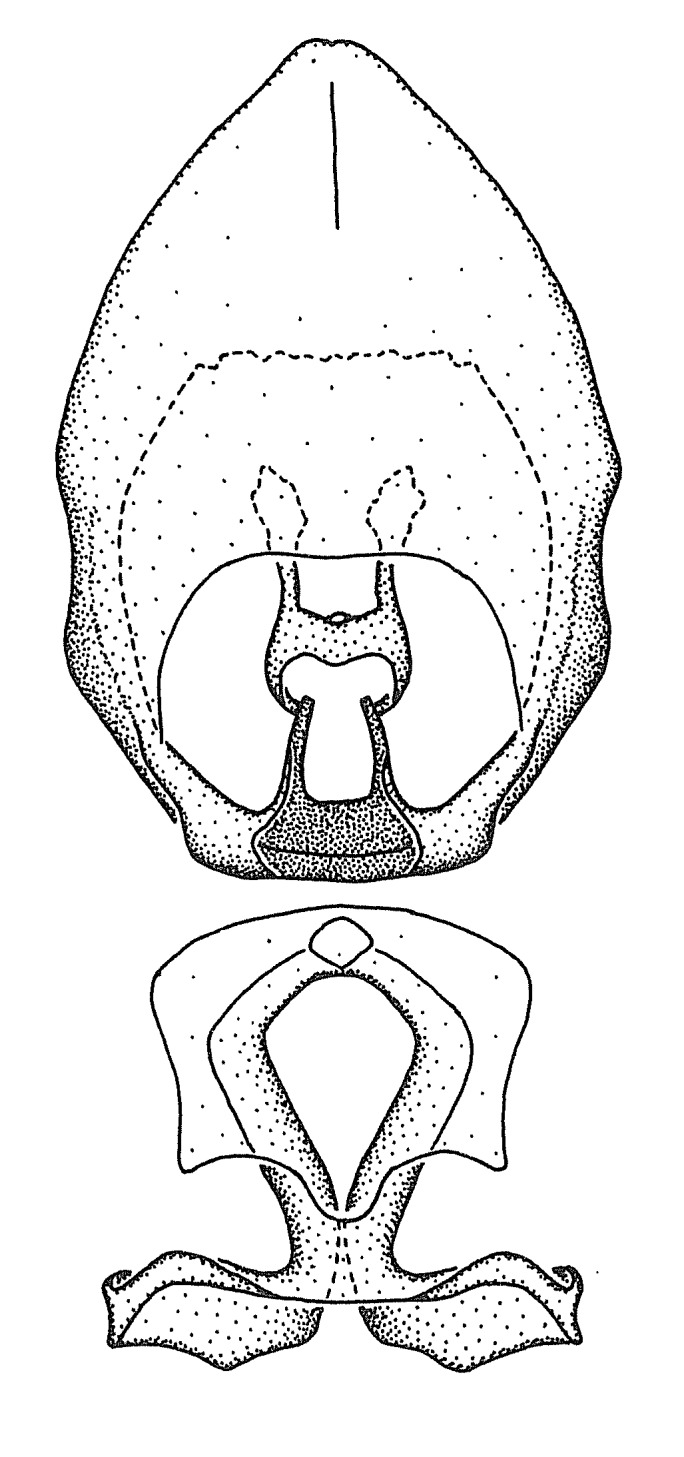
*Zelus banksi* Zhang & Hart, sp. n., phallus, dorsal view

**Figure 45a.**
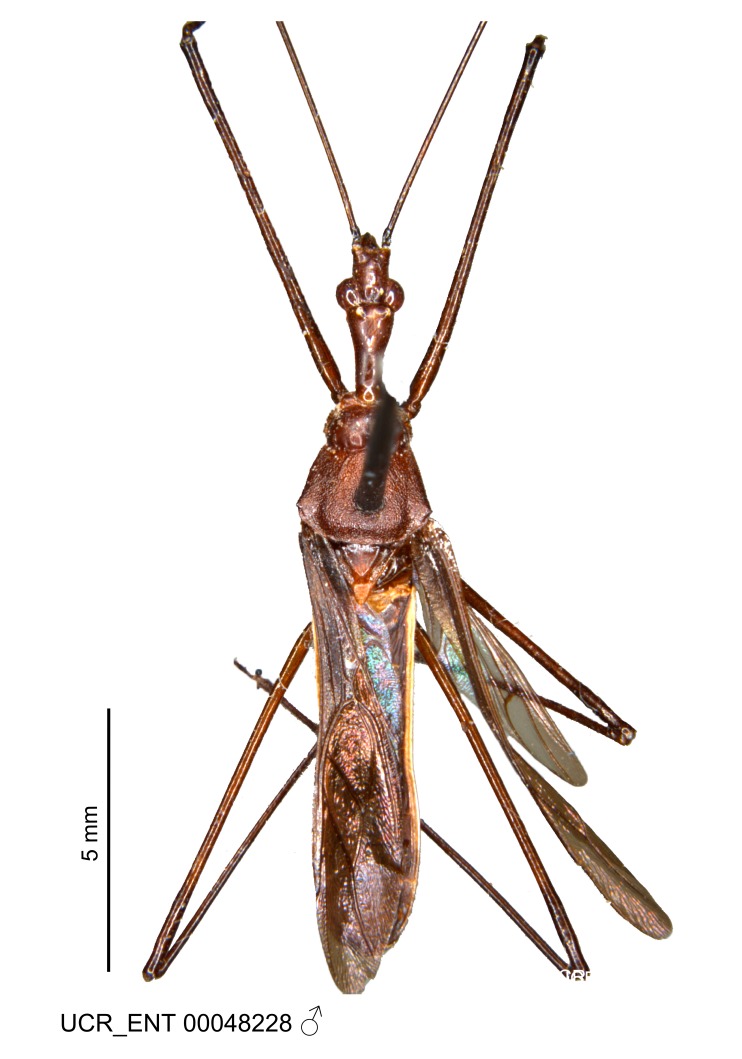
*Zelus casii* Zhang & Hart, sp. n., male, dorsal

**Figure 45b.**
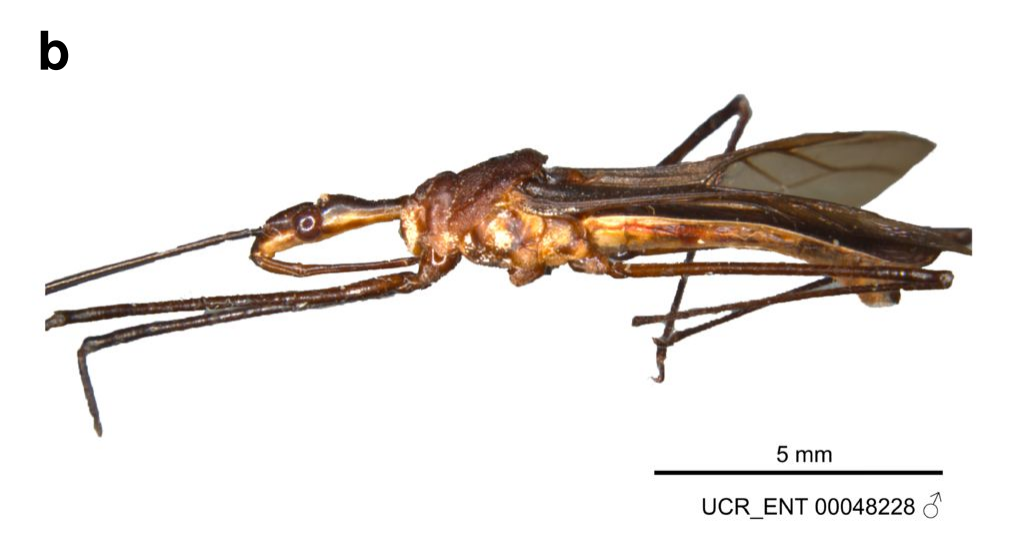
*Zelus casii* Zhang & Hart, sp. n., male, lateral

**Figure 46a.**
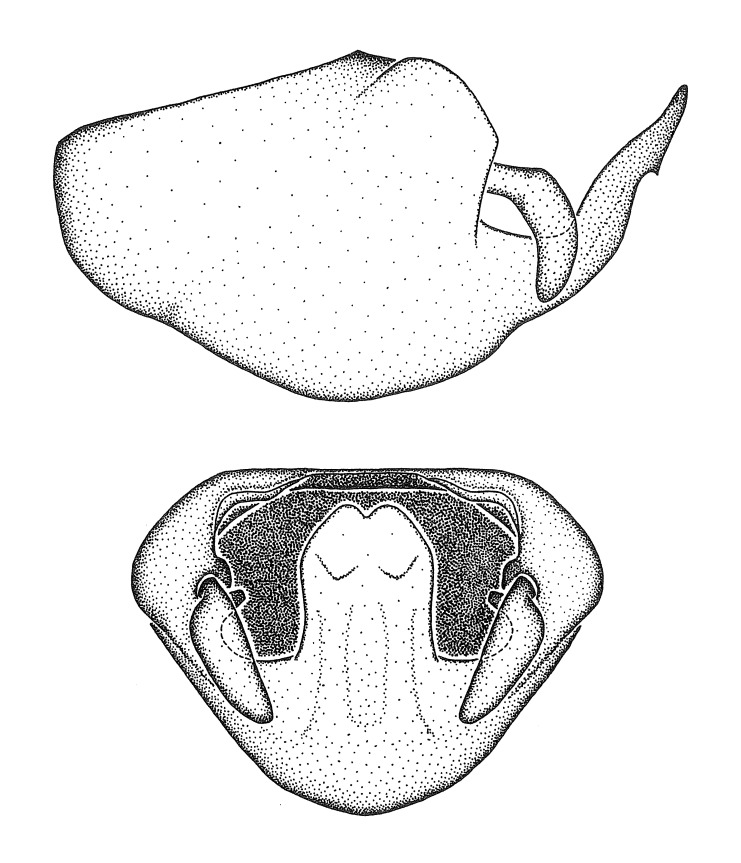
*Zelus casii* Zhang & Hart, **sp. n.**, pygophore, lateral and posterior views

**Figure 46b.**
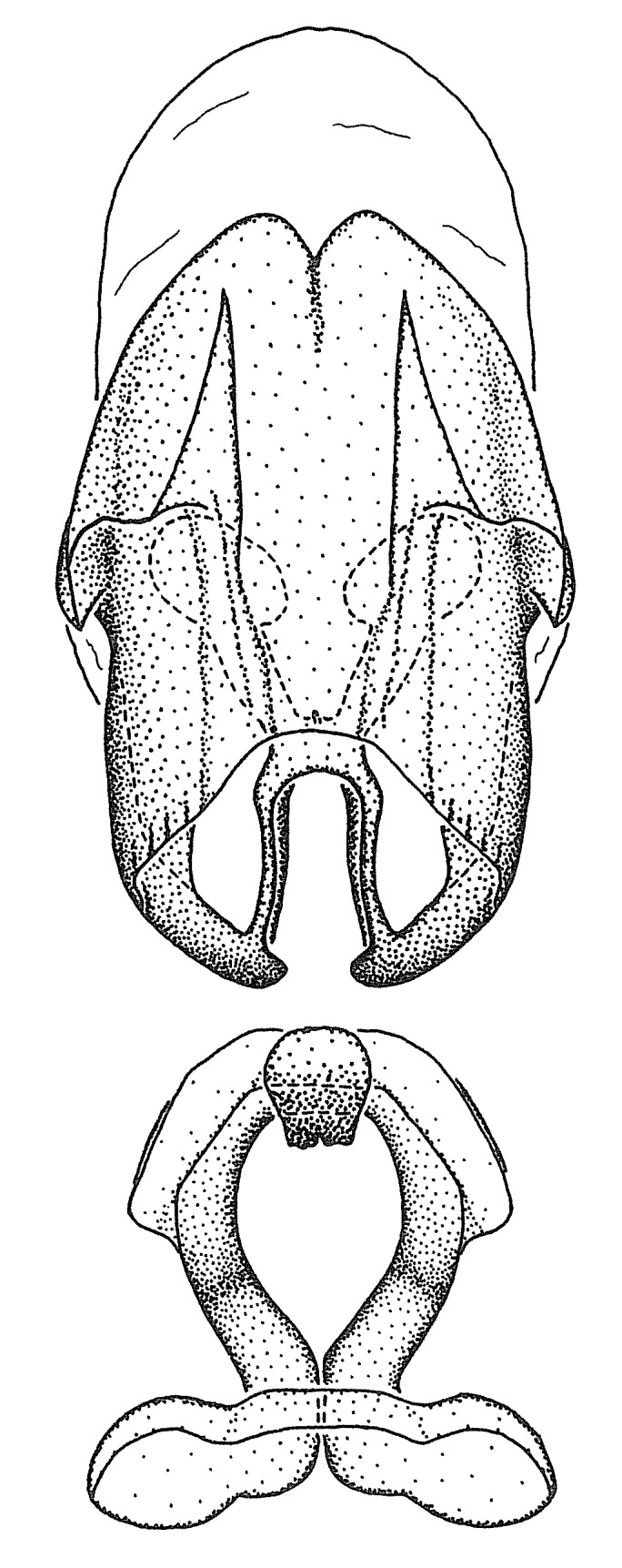
*Zelus casii* Zhang & Hart, **sp. n.**, phallus, dorsal view

**Figure 48a.**
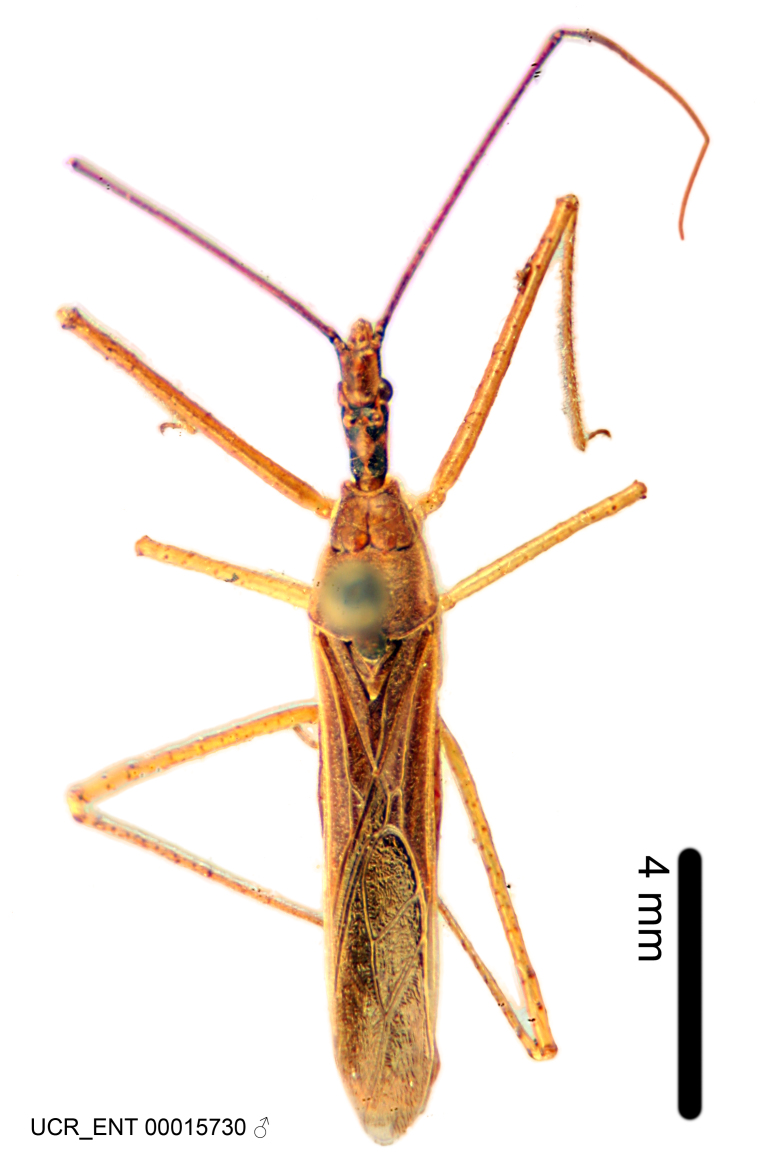
*Zelus cervicalis* Stål, 1872, male, dorsal, (UCR_ENT 00015730, Florida, USA)

**Figure 48b.**
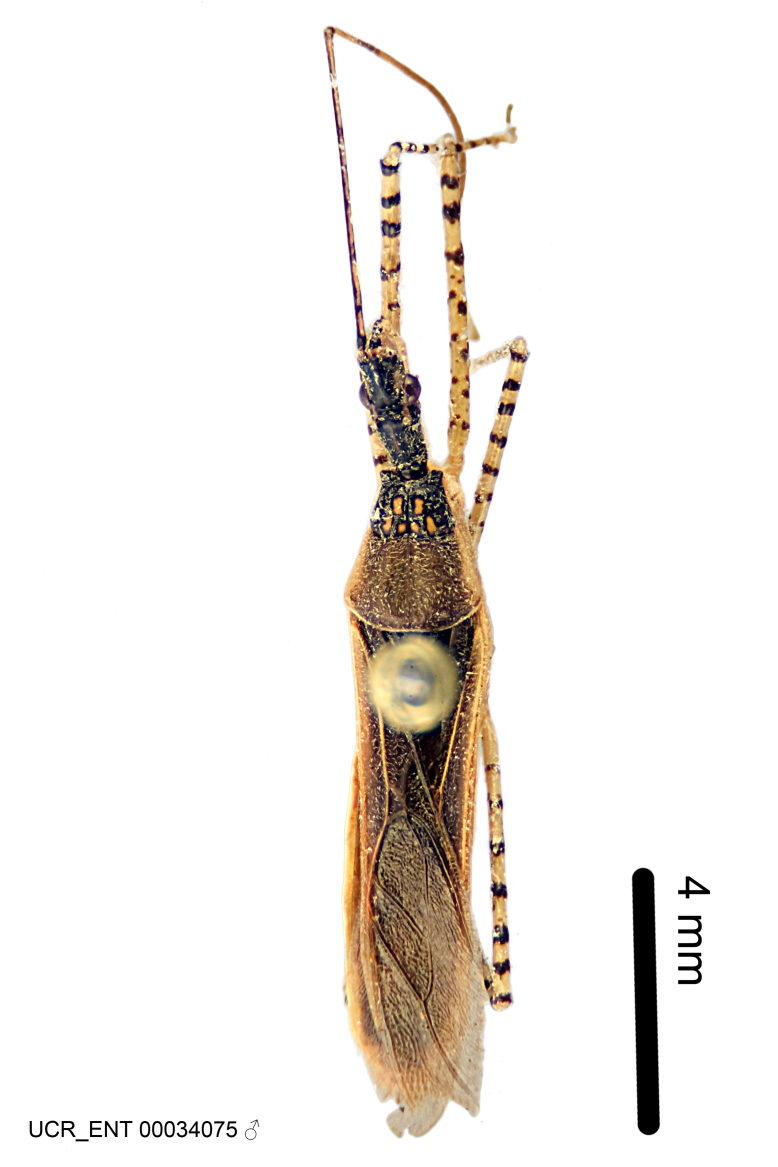
*Zelus cervicalis* Stål, 1872, male, dorsal (UCR_ENT 00034075, Puebla, Mexico)

**Figure 48c.**
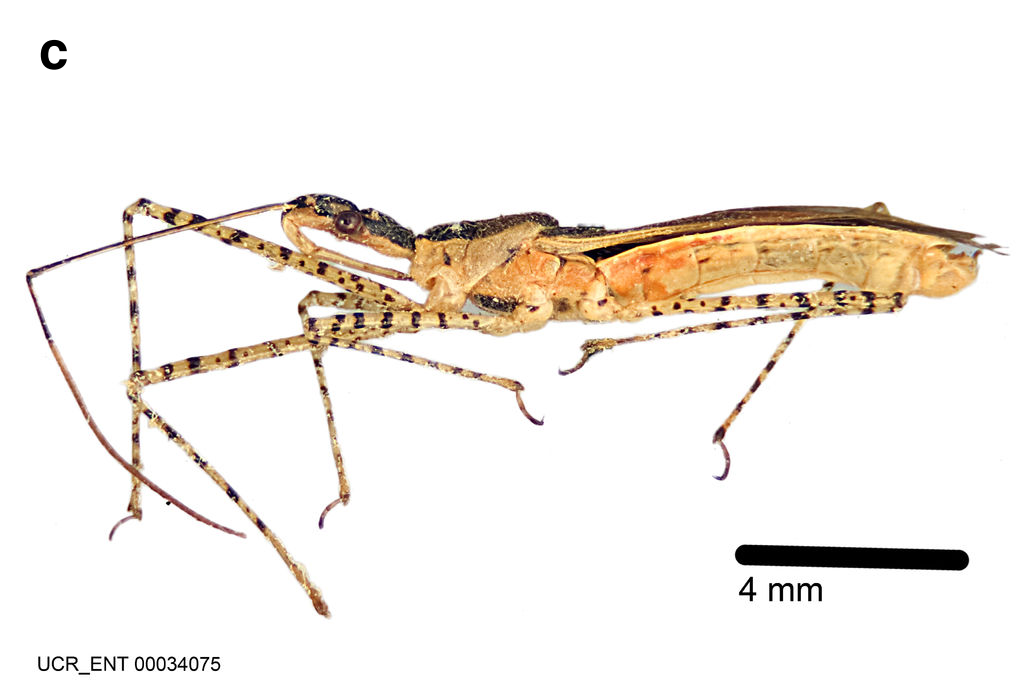
*Zelus cervicalis* Stål, 1872, male, lateral (UCR_ENT 00034075, Puebla, Mexico)

**Figure 48d.**
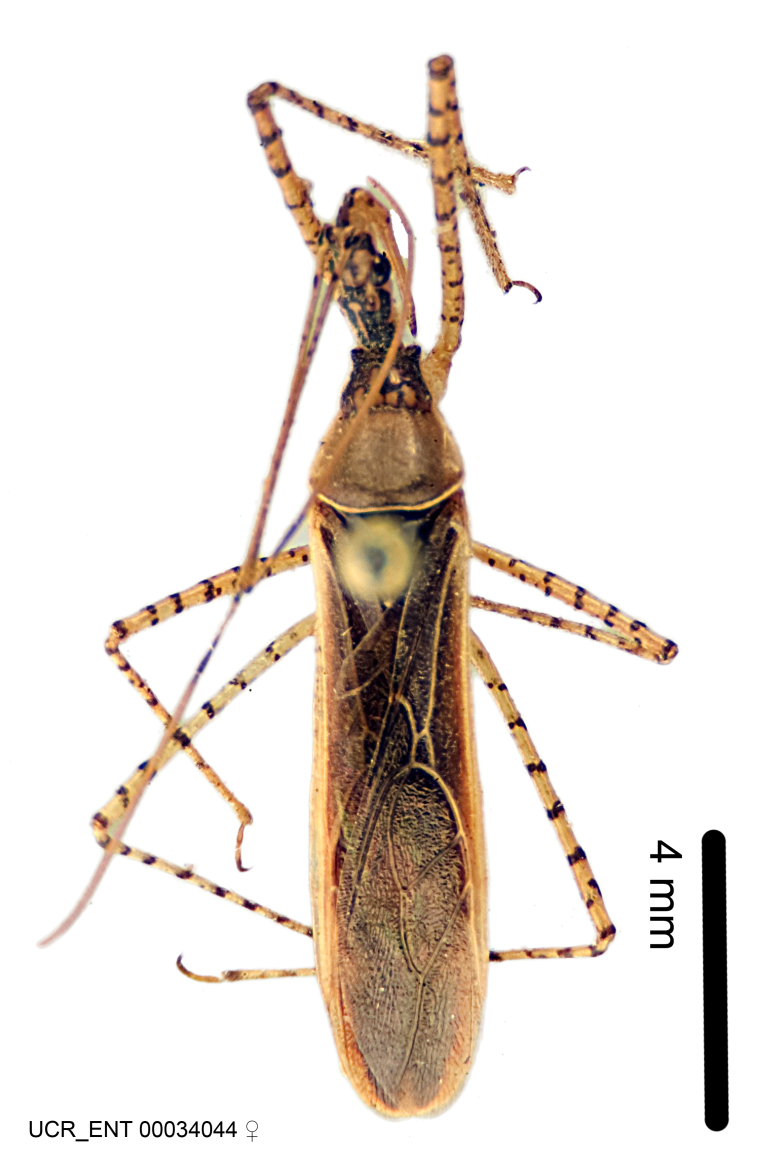
*Zelus cervicalis* Stål, 1872, female, dorsal (UCR_ENT 00034044, Guerrero, Mexico)

**Figure 48e.**
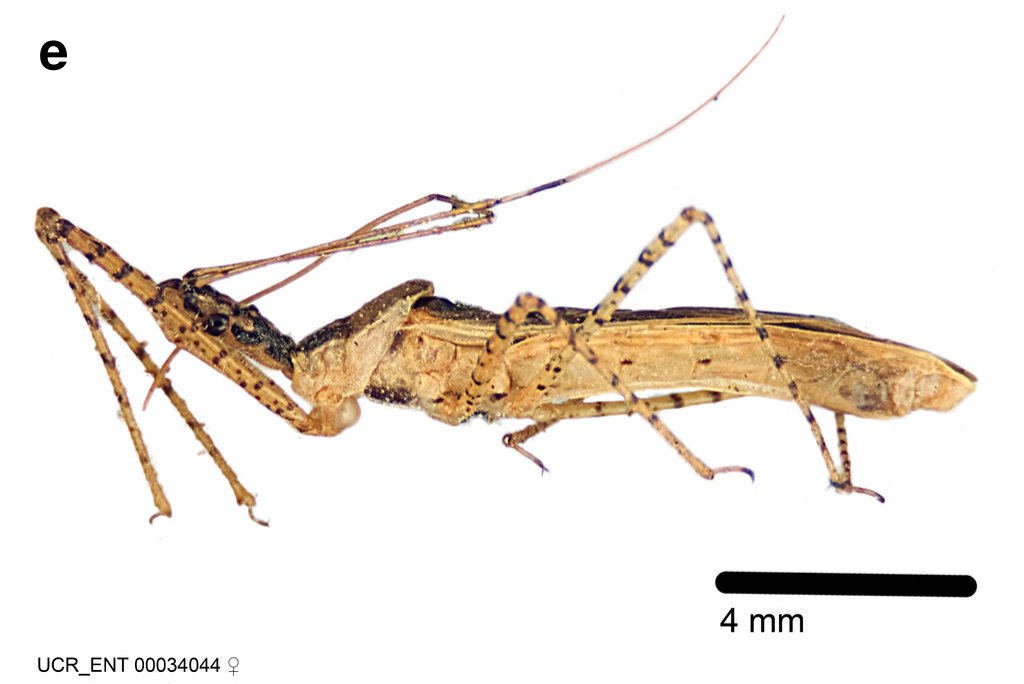
*Zelus cervicalis* Stål, 1872, female, lateral (UCR_ENT 00034044, Guerrero, Mexico)

**Figure 48f.**
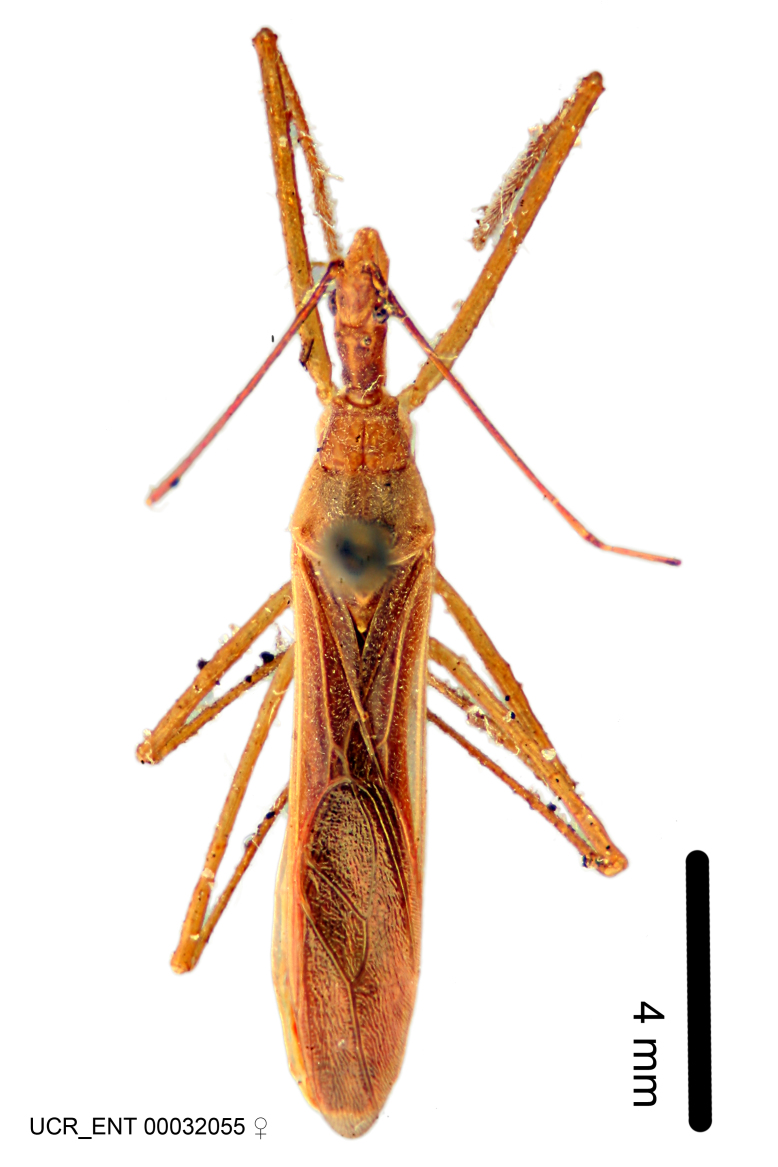
*Zelus cervicalis* Stål, 1872, female, dorsal (UCR_ENT 00032055, Georgia, USA)

**Figure 49a.**
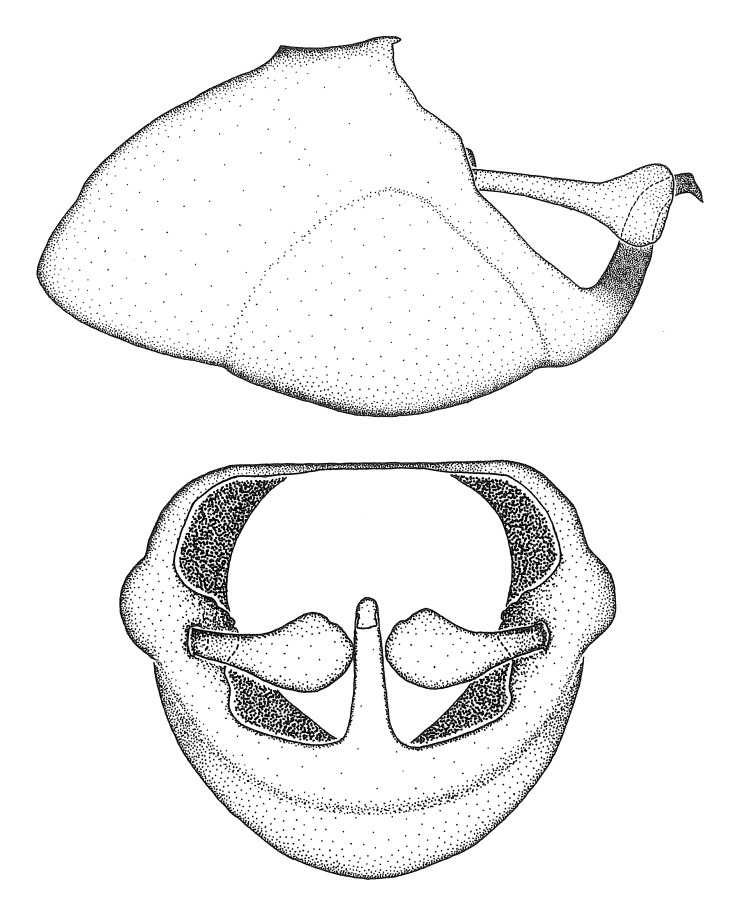
*Zelus cervicalis* Stål, 1872, Mexico-Central America population, pygophore, lateral and posterior views

**Figure 49b.**
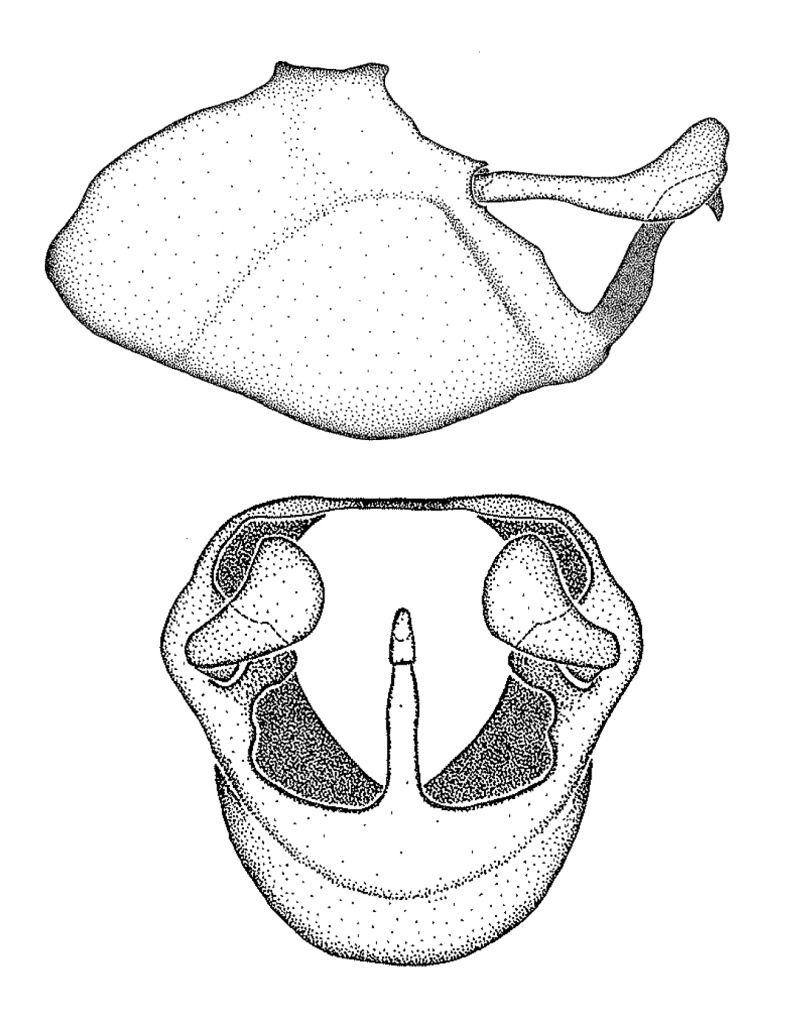
*Zelus cervicalis* Stål, 1872, Gulf Coast-US population, pygophore, lateral and posterior views

**Figure 49c.**
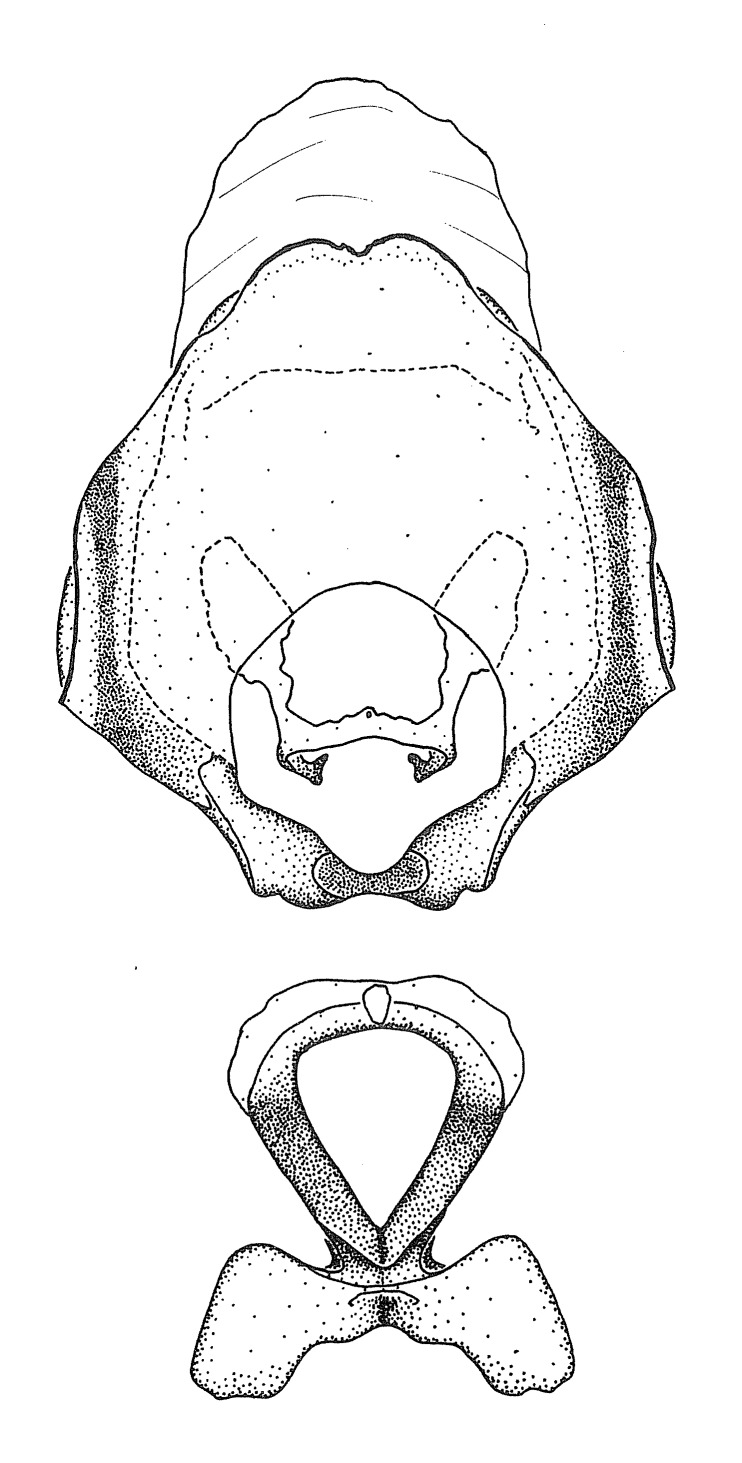
*Zelus cervicalis* Stål, 1872, Mexico-Central America population, phallus, dorsal view

**Figure 49d.**
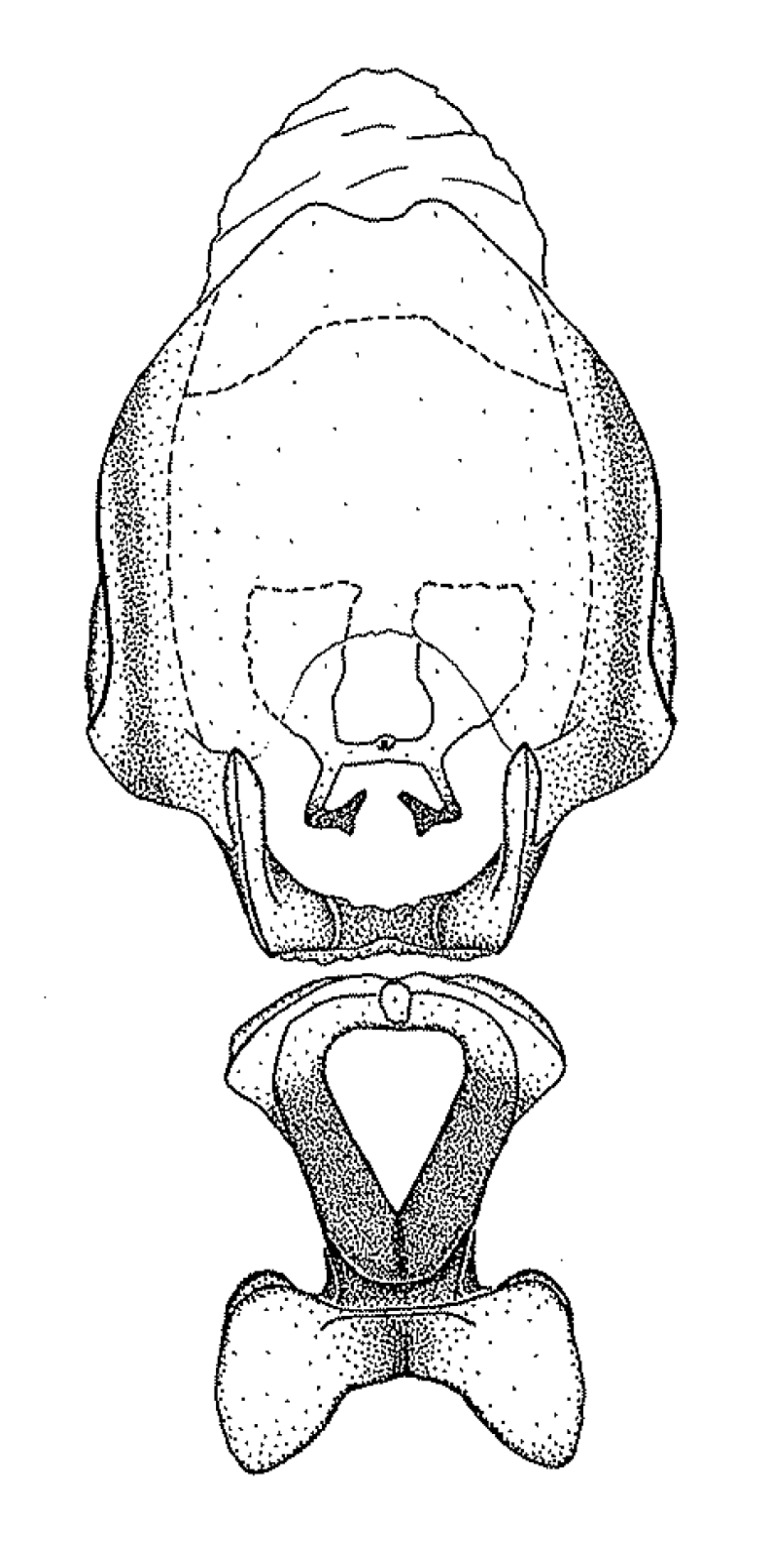
*Zelus cervicalis* Stål, 1872, Gulf Coast-US population, phallus, dorsal view

**Figure 51a.**
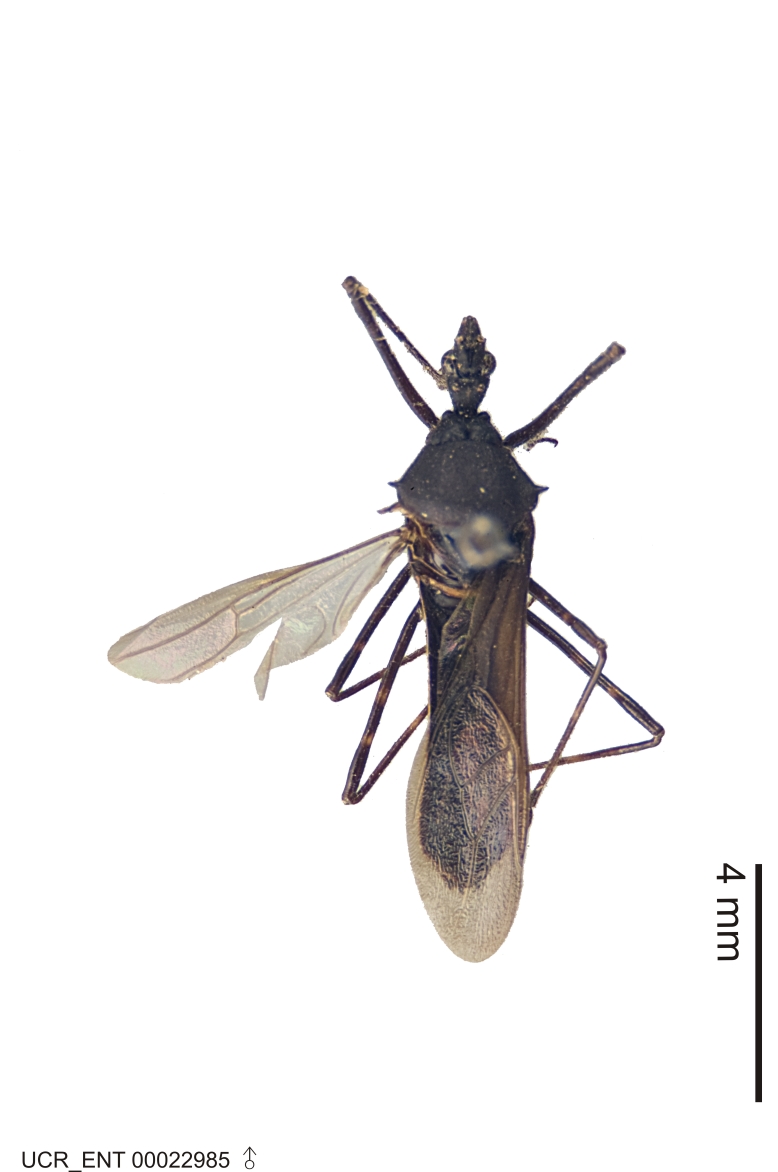
*Zelus chamaeleon* Stål, 1872, male, dorsal view (UCR_ENT 00022985, Cundinamarca, Colombia)

**Figure 51b.**
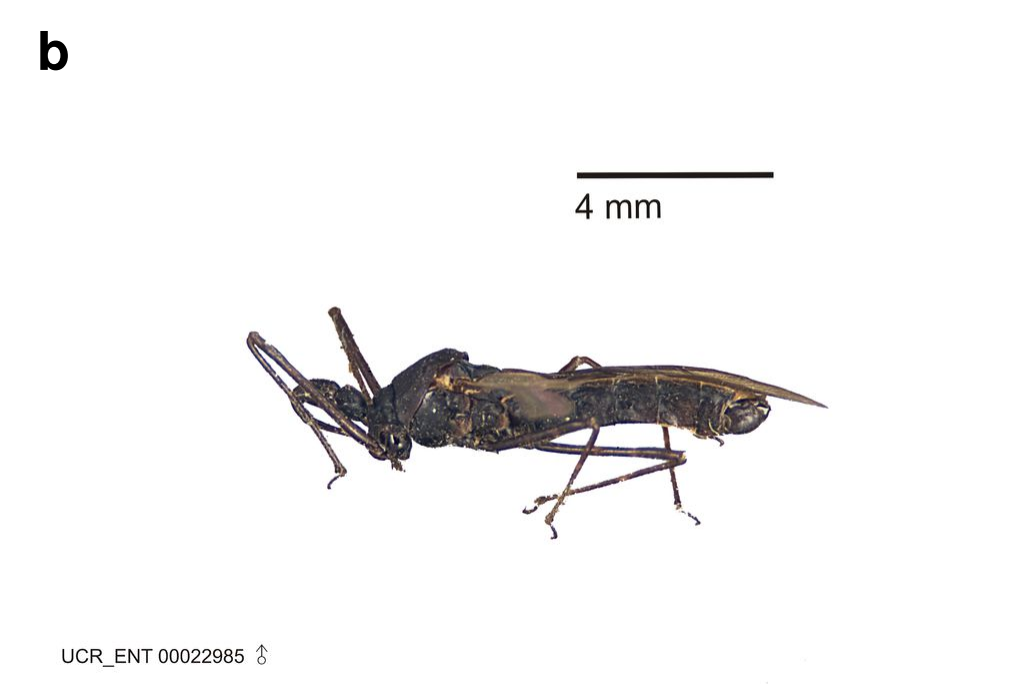
*Zelus chamaeleon* Stål, 1872, male, lateral view (UCR_ENT 00022985, Cundinamarca, Colombia)

**Figure 51c.**
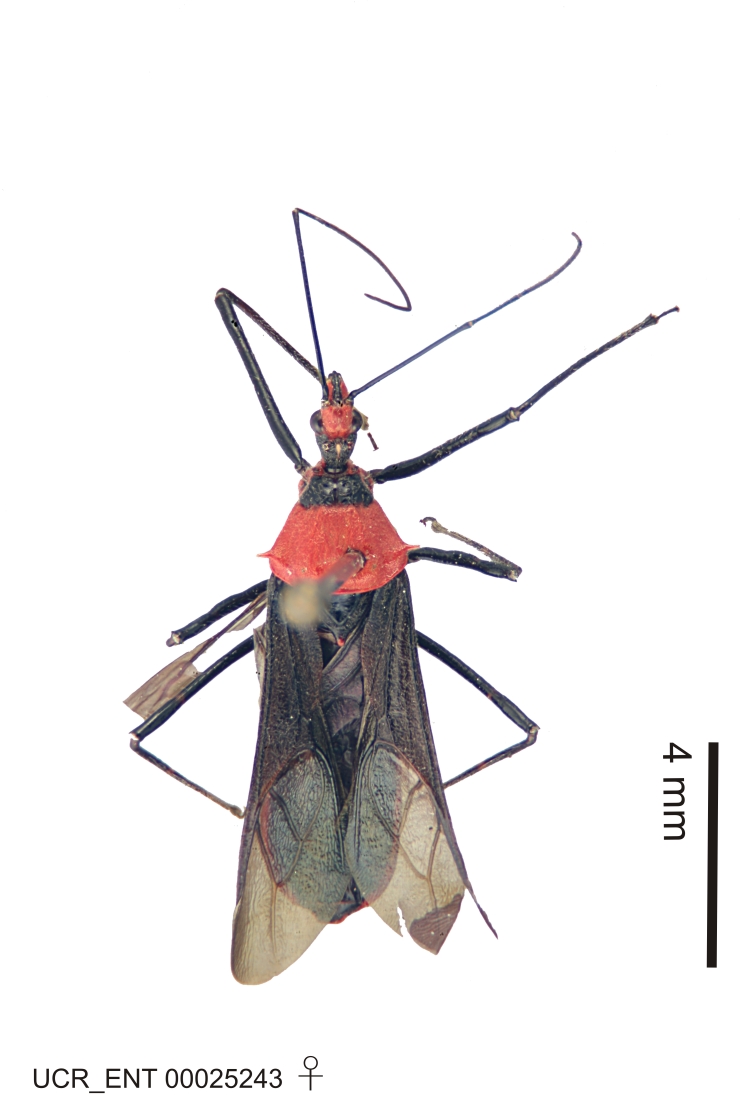
*Zelus chamaeleon* Stål, 1872, female, dorsal view (UCR_ENT 00025243, Cundinamarca, Colombia)

**Figure 51d.**
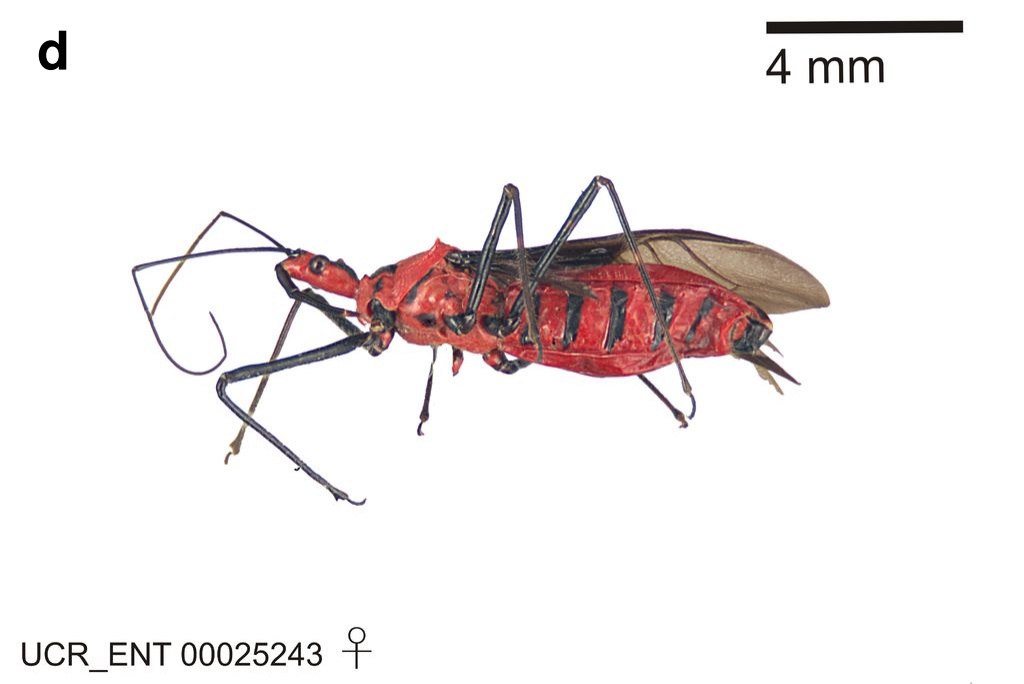
*Zelus chamaeleon* Stål, 1872, female, lateral view (UCR_ENT 00025243, Cundinamarca, Colombia)

**Figure 52a.**
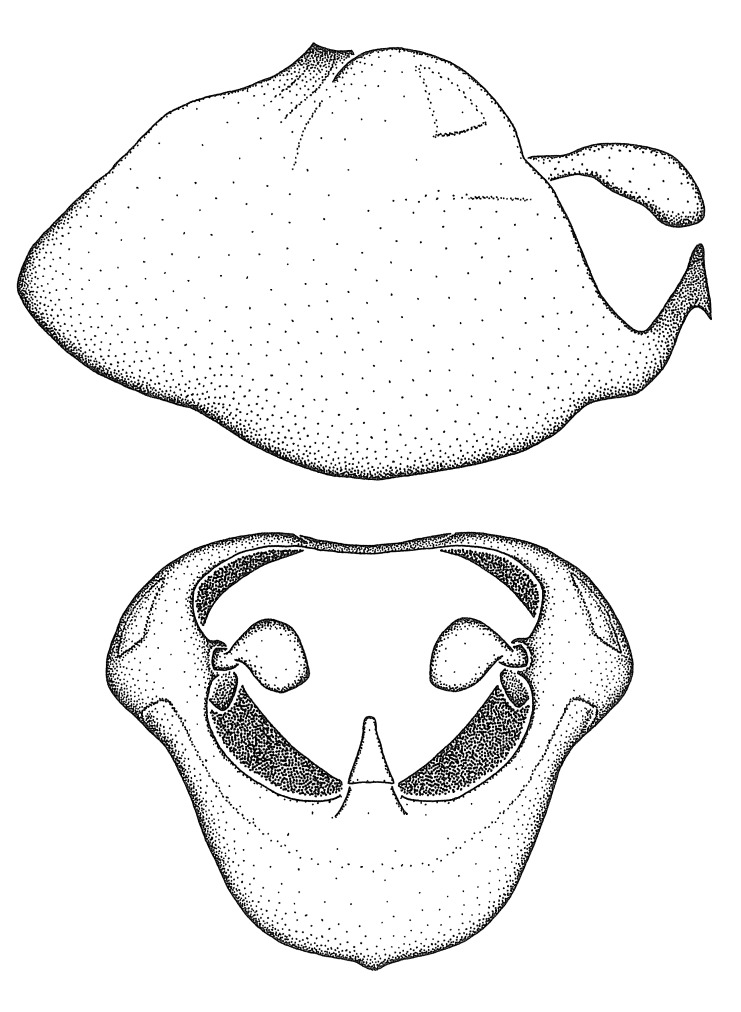
*Zelus chamaeleon* Stål, 1872, pygophore, lateral and posterior views

**Figure 52b.**
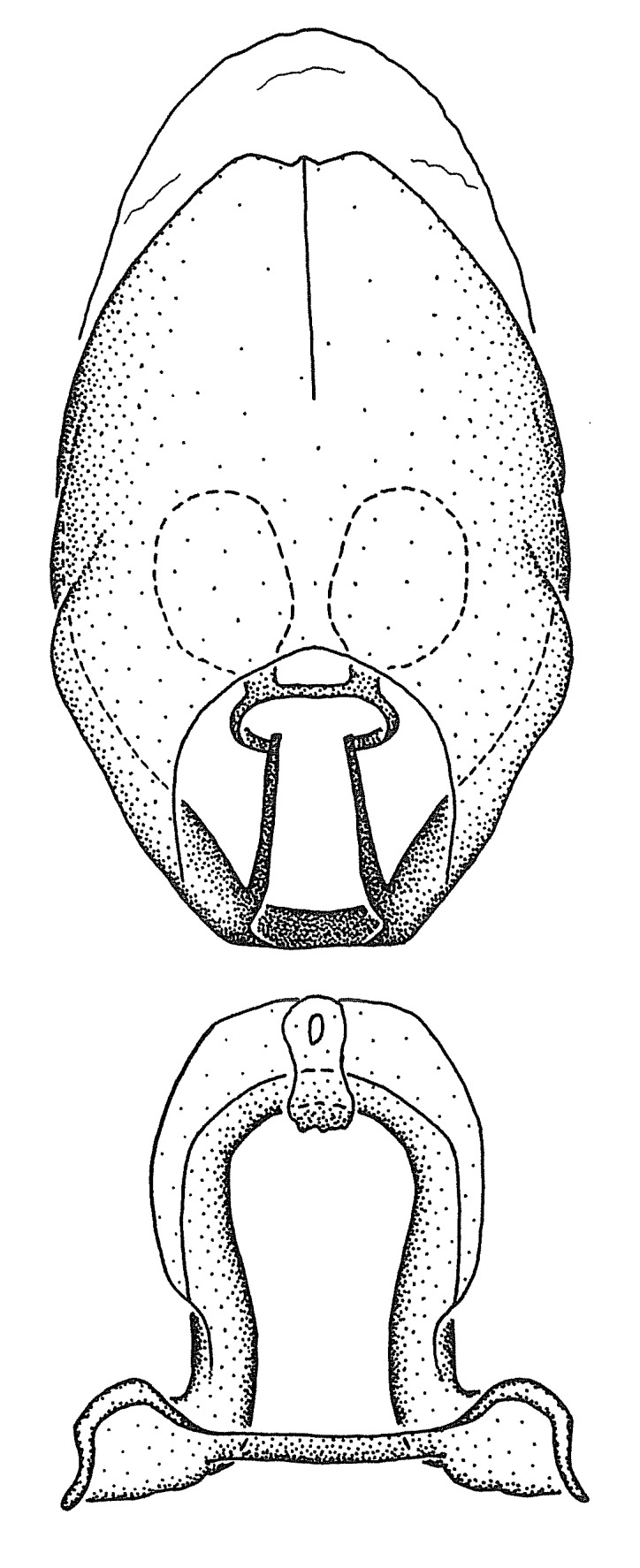
*Zelus chamaeleon* Stål, 1872, phallus, dorsal view

**Figure 54a.**
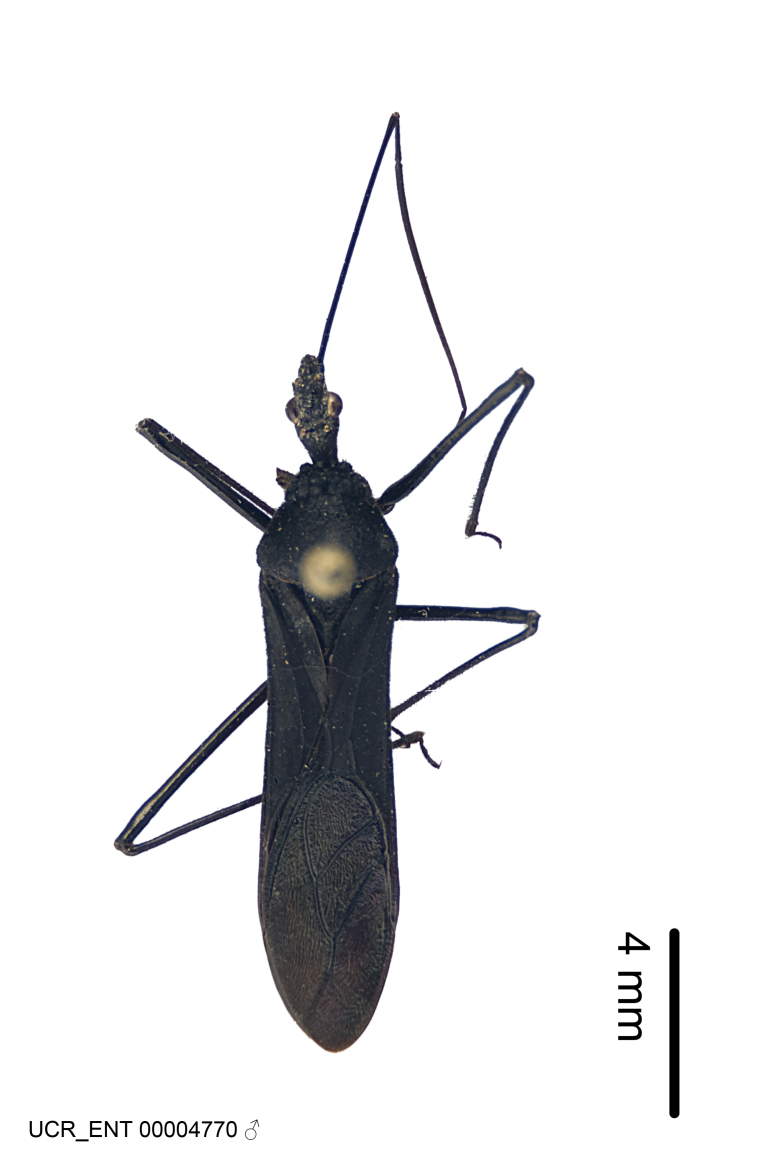
*Zelus championi*, Zhang & Hart, male, dorsal view (UCR_ENT 00004770)

**Figure 54b.**
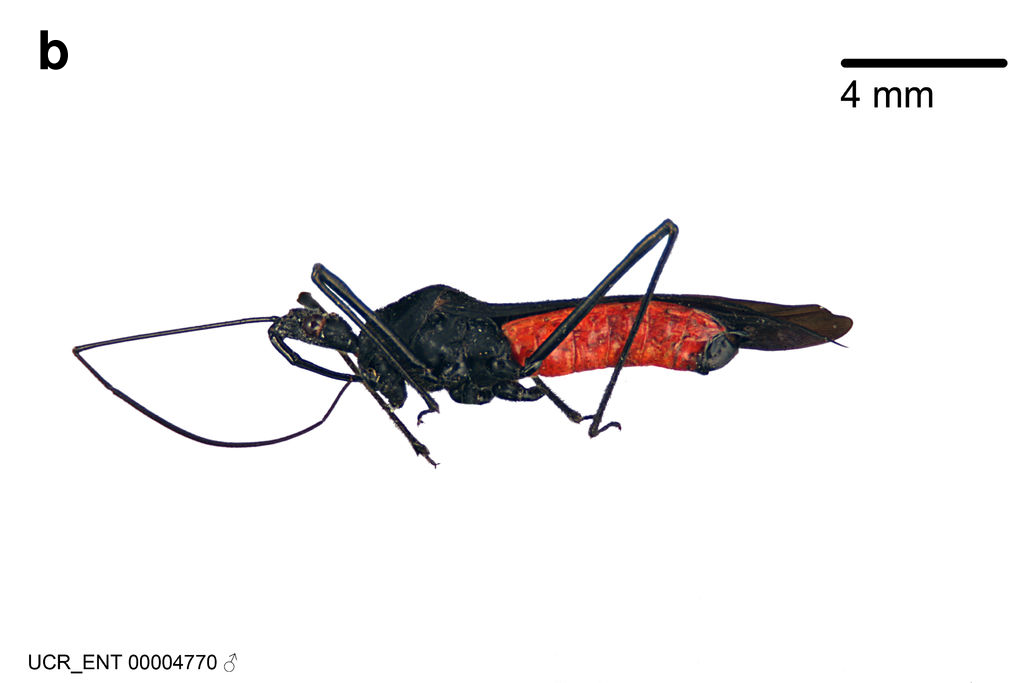
*Zelus championi*, Zhang & Hart, male, lateral view (UCR_ENT 00004770)

**Figure 55a.**
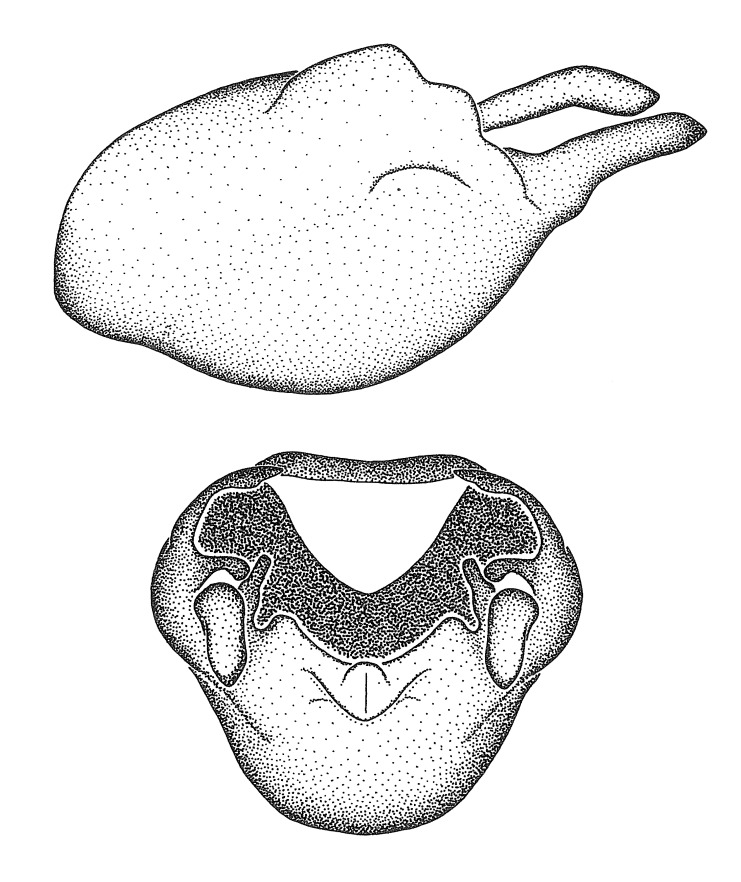
*Zelus championi*, Zhang & Hart, sp. n., pygophore, lateral and posterior views

**Figure 55b.**
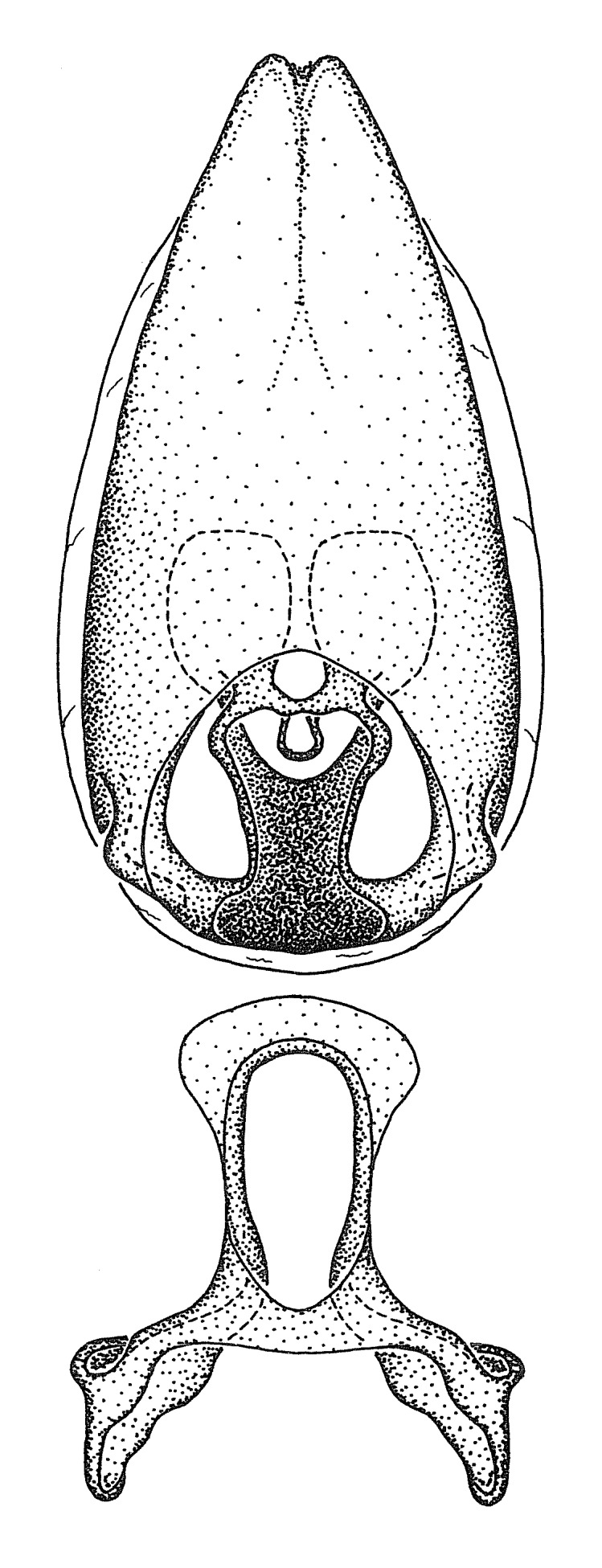
*Zelus championi*, Zhang & Hart, sp. n., phallus, dorsal view

**Figure 57a.**
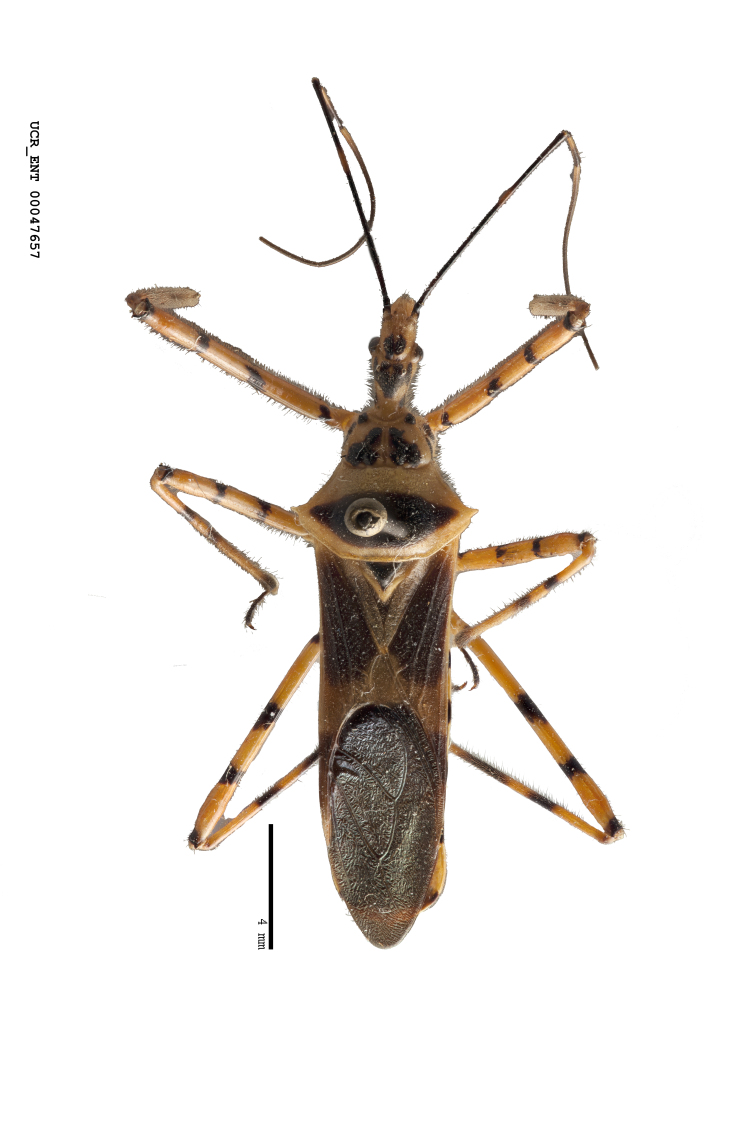
*Zelus conjungens* (Stål, 1860), male, dorsal (UCR_ENT 00047657, Santa Catarina, Brazil)

**Figure 57b.**
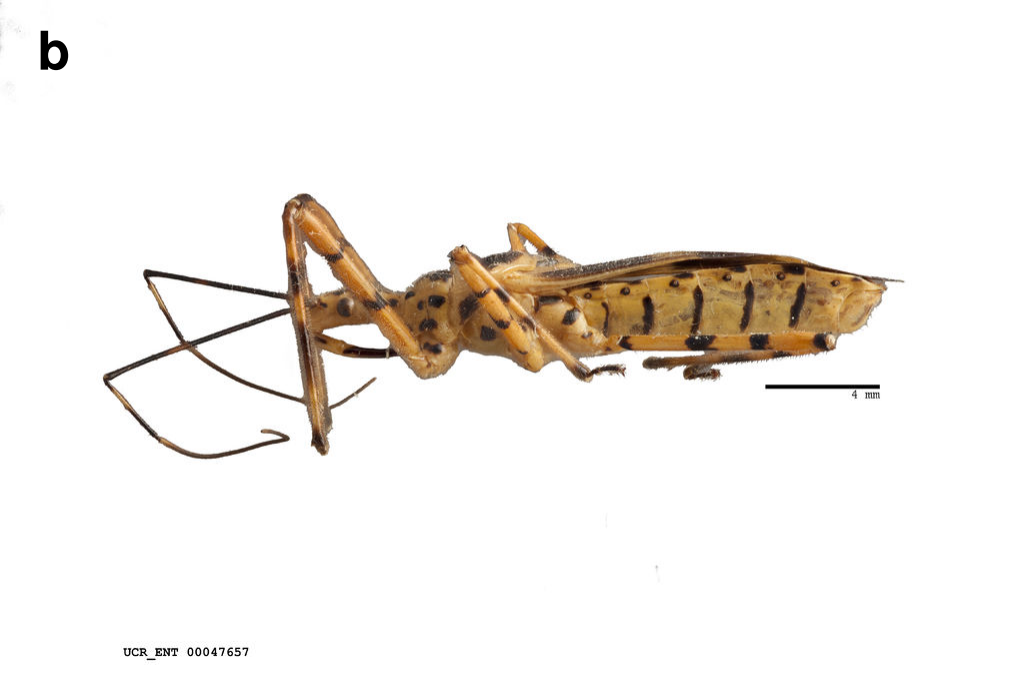
*Zelus conjungens* (Stål, 1860), male, lateral (UCR_ENT 00047657, Santa Catarina, Brazil)

**Figure 57c.**
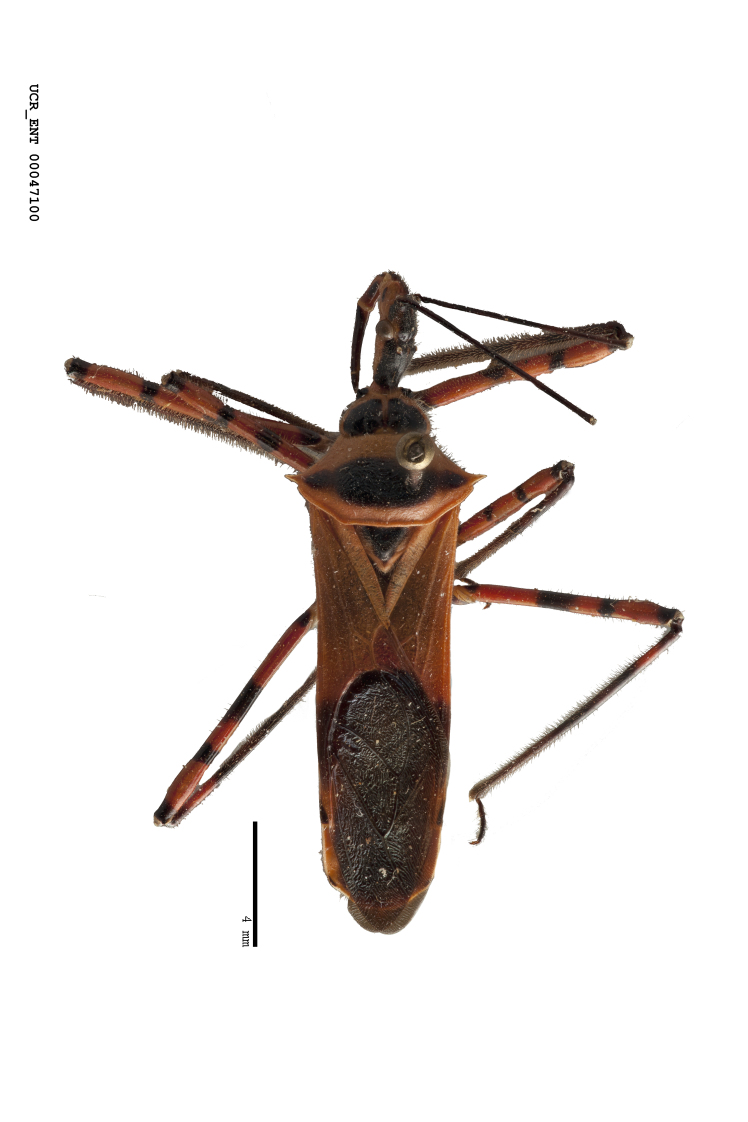
*Zelus conjungens* (Stål, 1860), male, dorsal (UCR_ENT 00047100, Meta, Colombia)

**Figure 57d.**
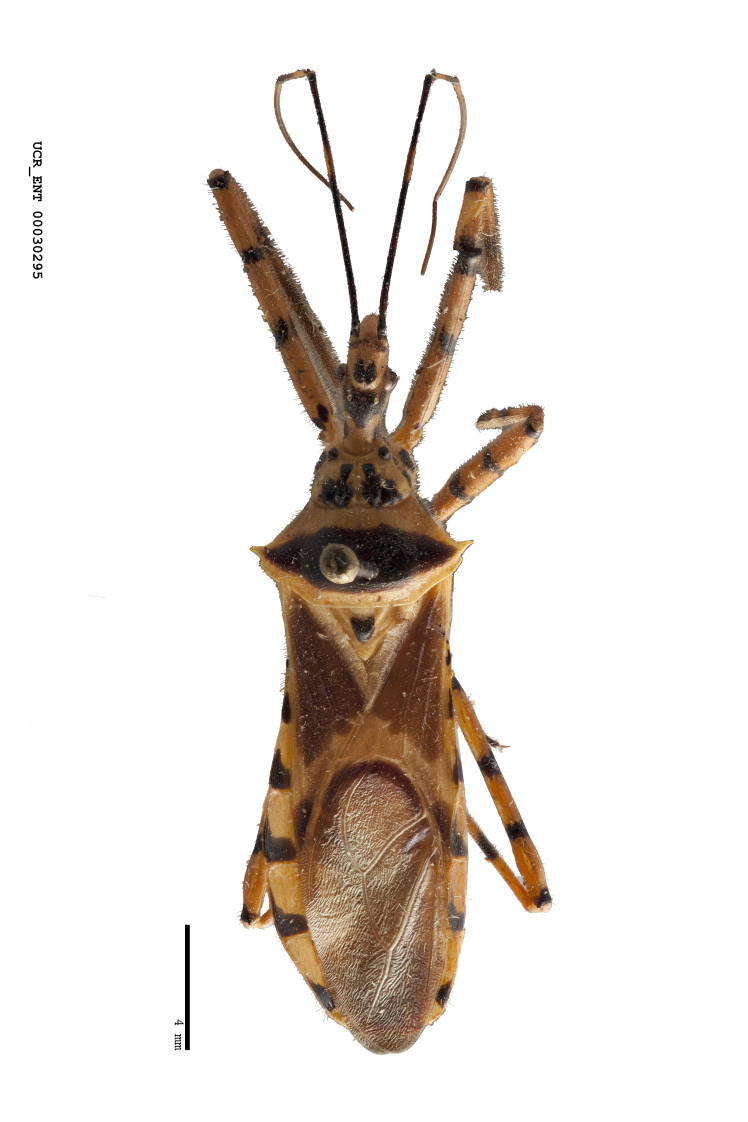
*Zelus conjungens* (Stål, 1860), female, dorsal (UCR_ENT 00030295, Santa Catarina, Brazil)

**Figure 57e.**
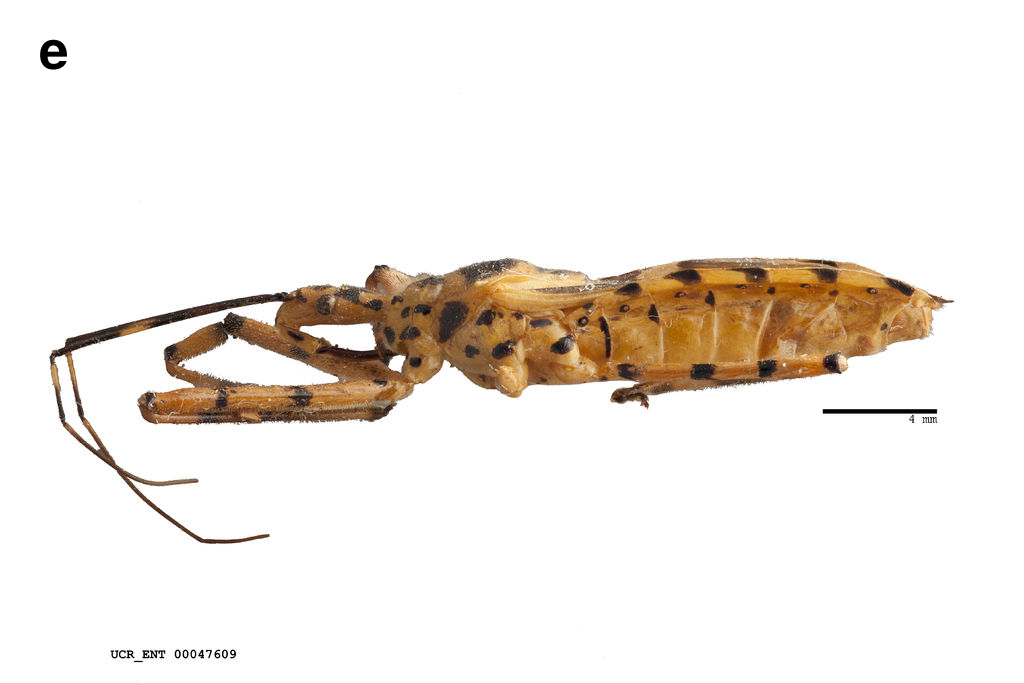
*Zelus conjungens* (Stål, 1860), female, lateral (UCR_ENT 00047609, Santa Catarina, Brazil)

**Figure 57f.**
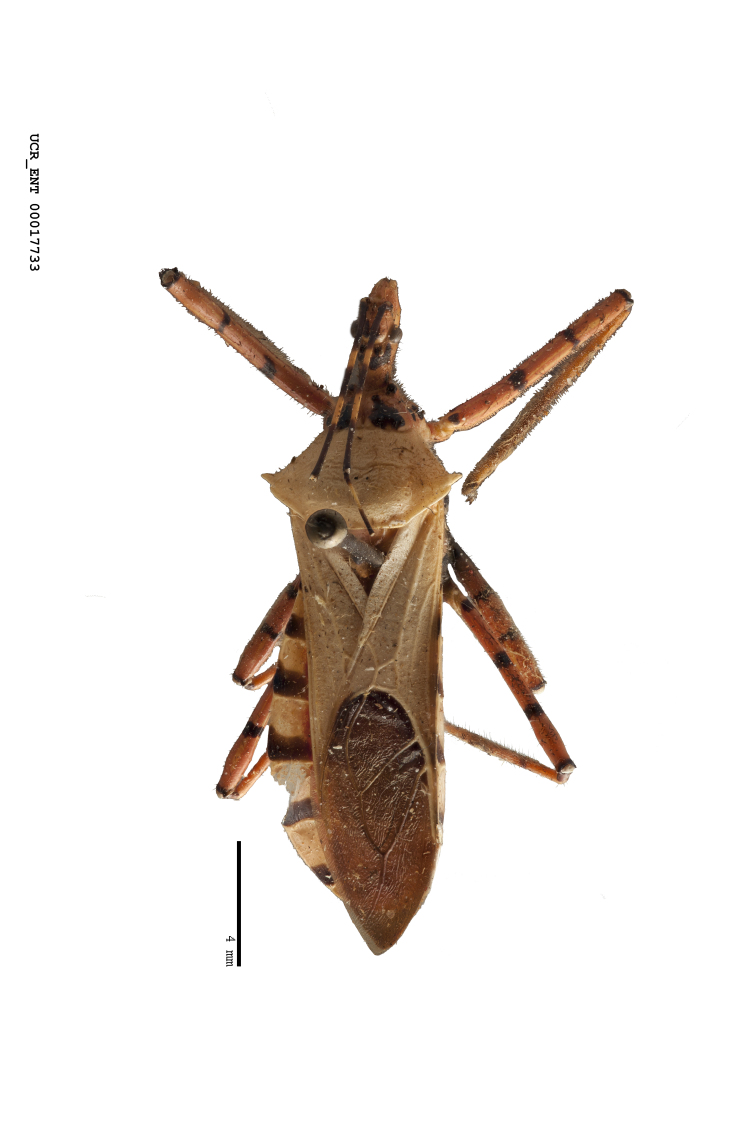
*Zelus conjungens* (Stål, 1860), female, dorsal (UCR_ENT 00017733, Sau Paulo, Brazil)

**Figure 58a.**
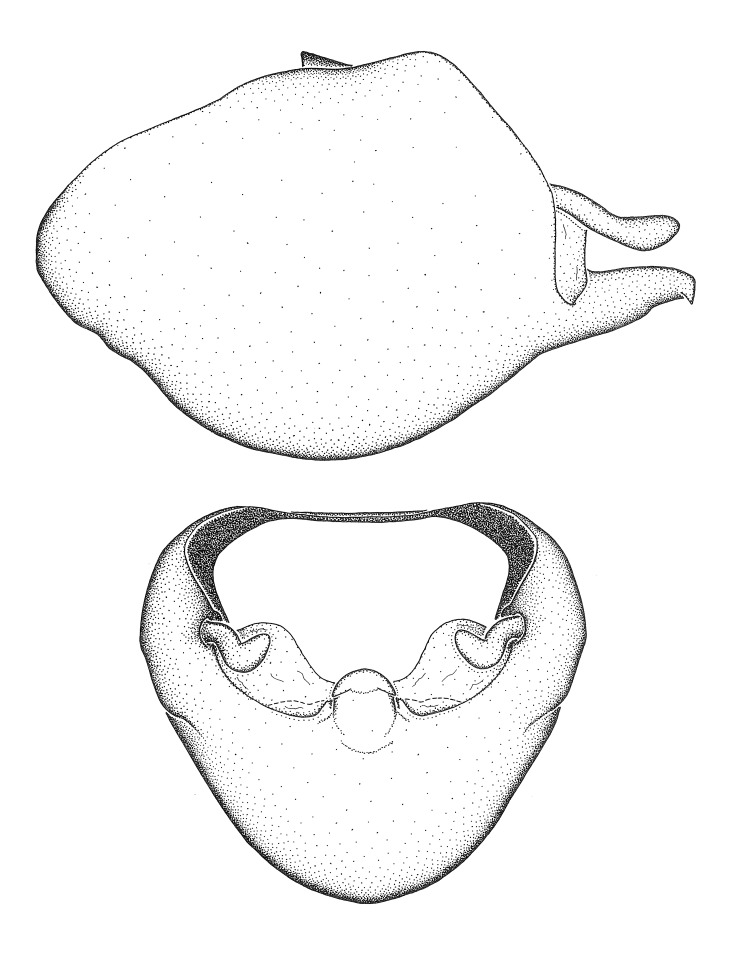
*Zelus conjungens* (Stål, 1860), pygophore, lateral and posterior views

**Figure 58b.**
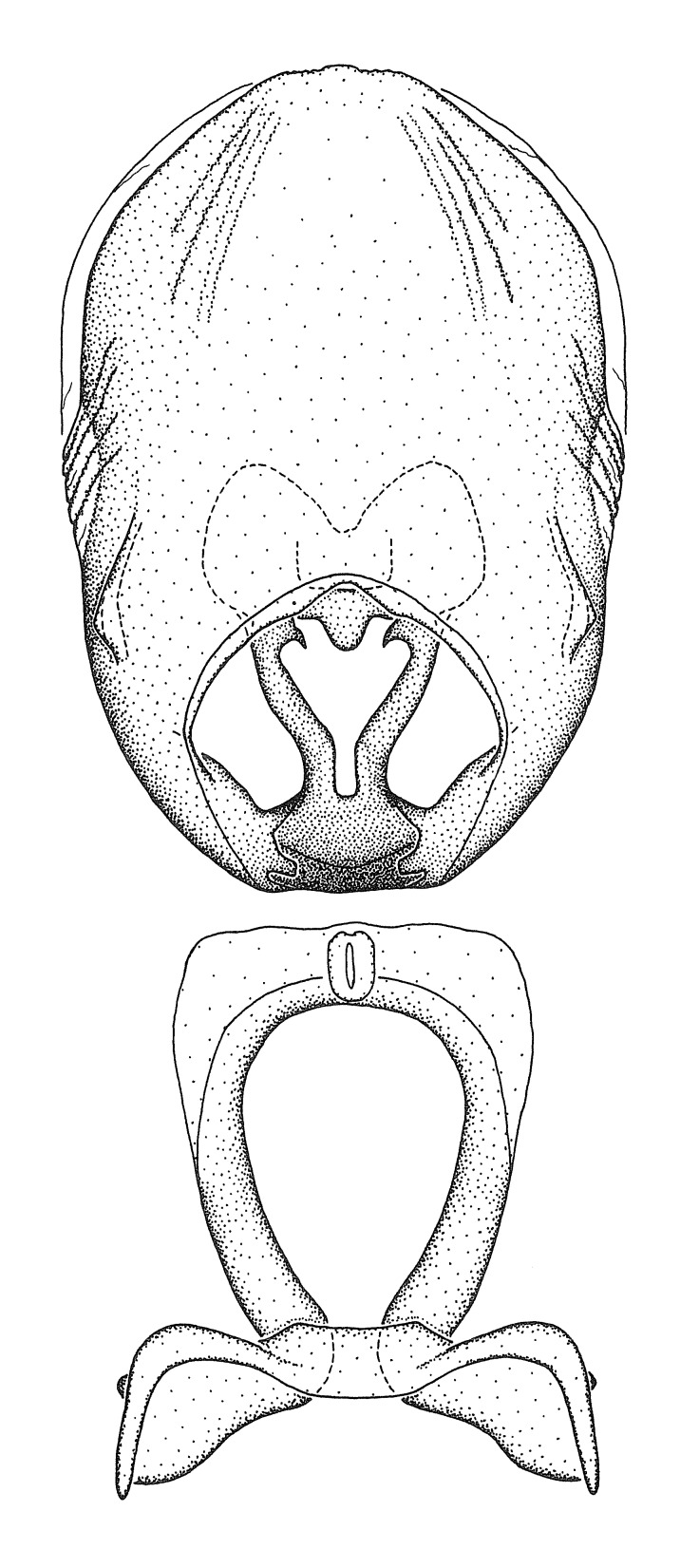
*Zelus conjungens* (Stål, 1860), phallus, dorsal view

**Figure 60a.**
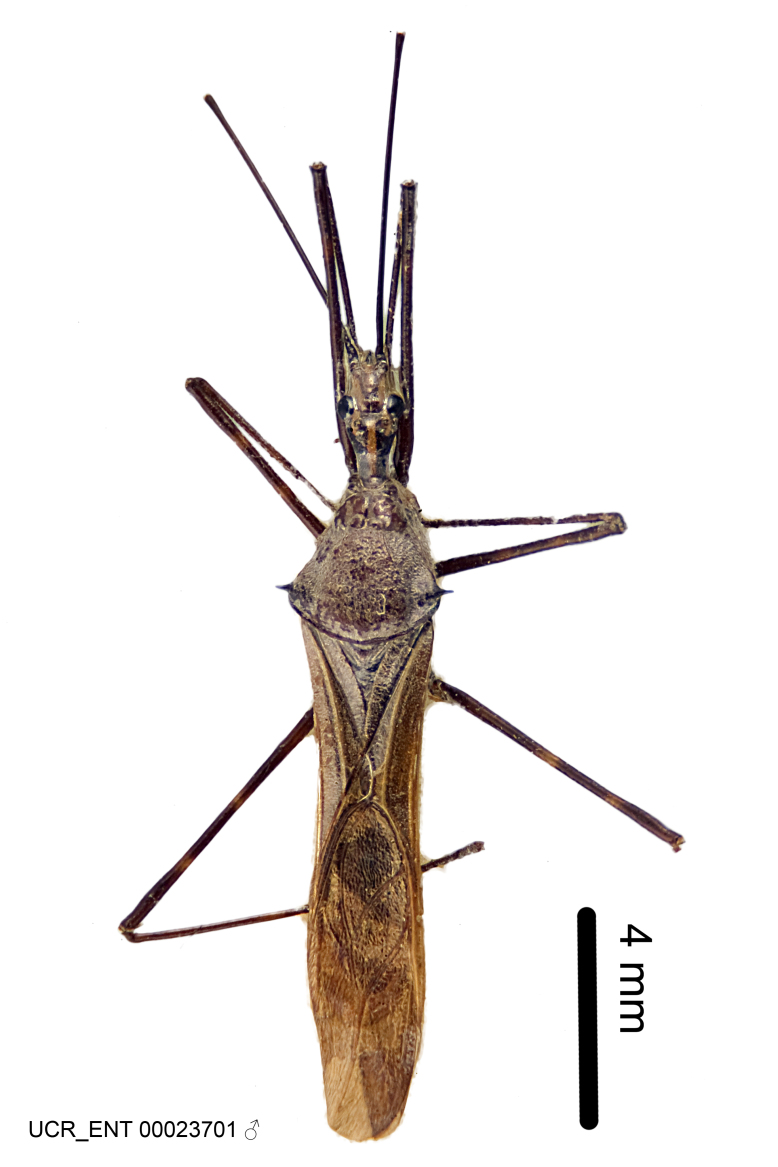
*Zelus cordazulus* Zhang & Hart, sp. n., male, dorsal view (UCR_ENT 00023701, Junin, Peru)

**Figure 60b.**
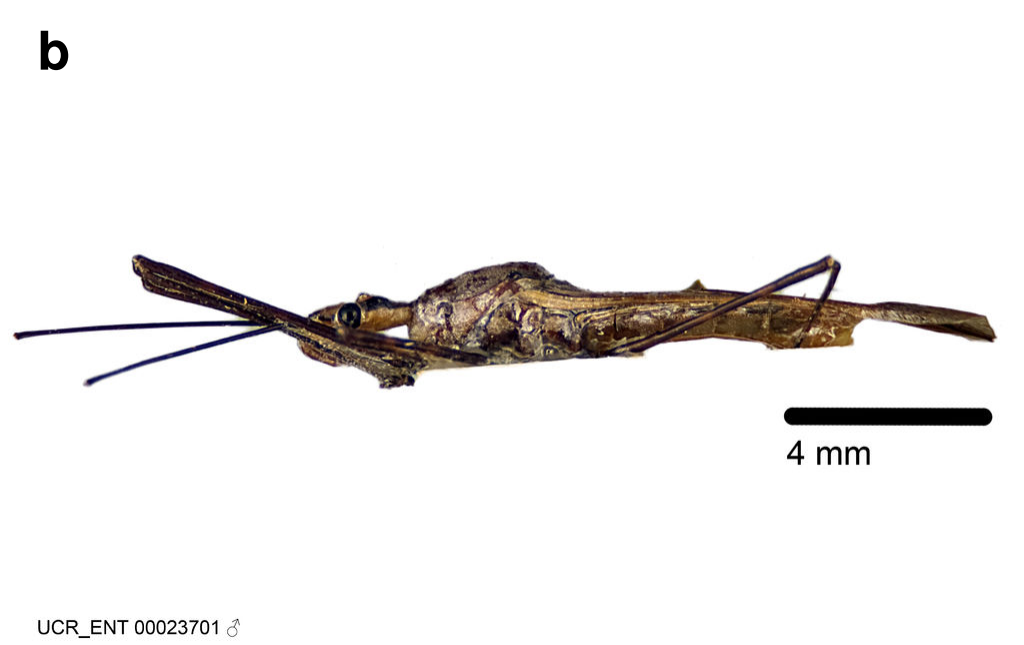
*Zelus cordazulus* Zhang & Hart, sp. n., male, lateral view (UCR_ENT 00023701, Junin, Peru)

**Figure 60c.**
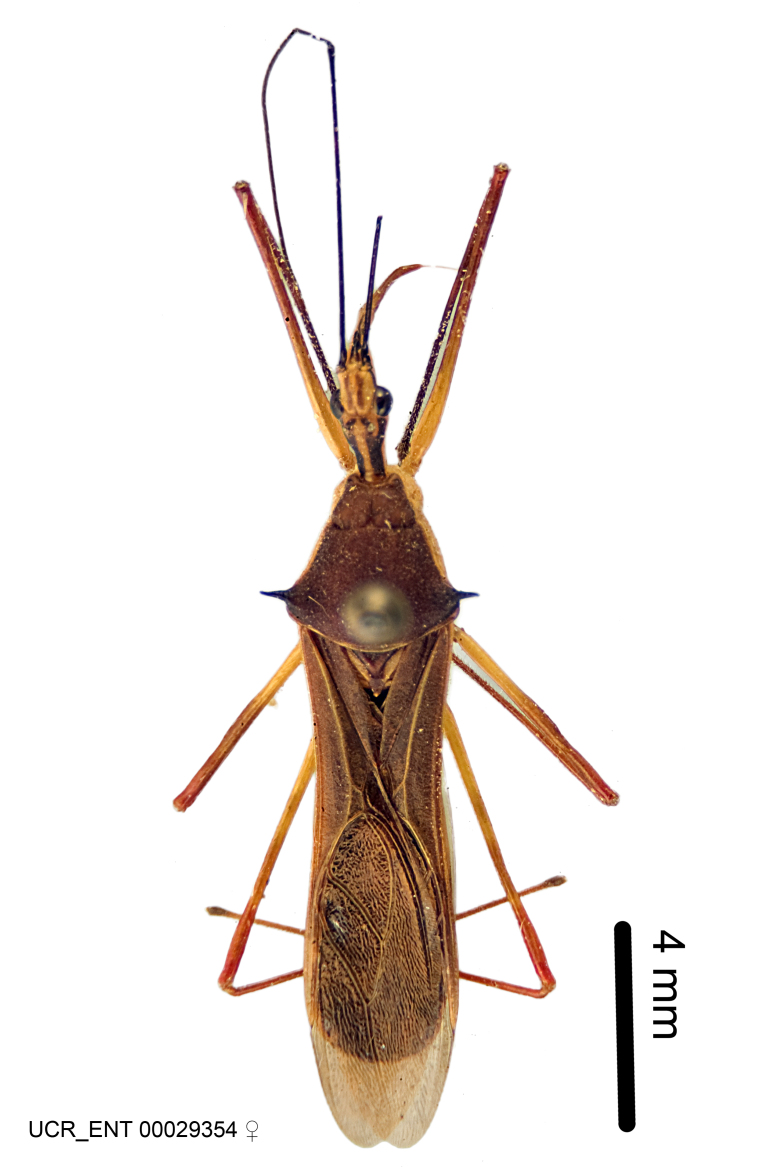
*Zelus cordazulus* Zhang & Hart, sp. n., female, dorsal view (UCR_ENT 00029354, Cusco, Peru)

**Figure 60d.**
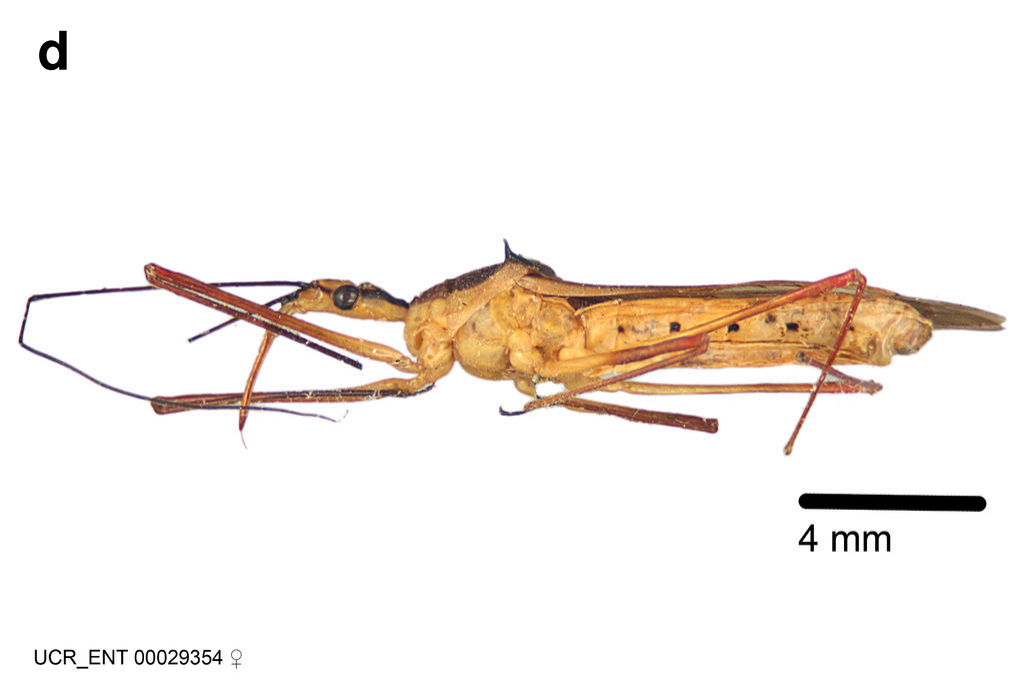
*Zelus cordazulus* Zhang & Hart, sp. n., female, lateral view (UCR_ENT 00029354, Cusco, Peru)

**Figure 61a.**
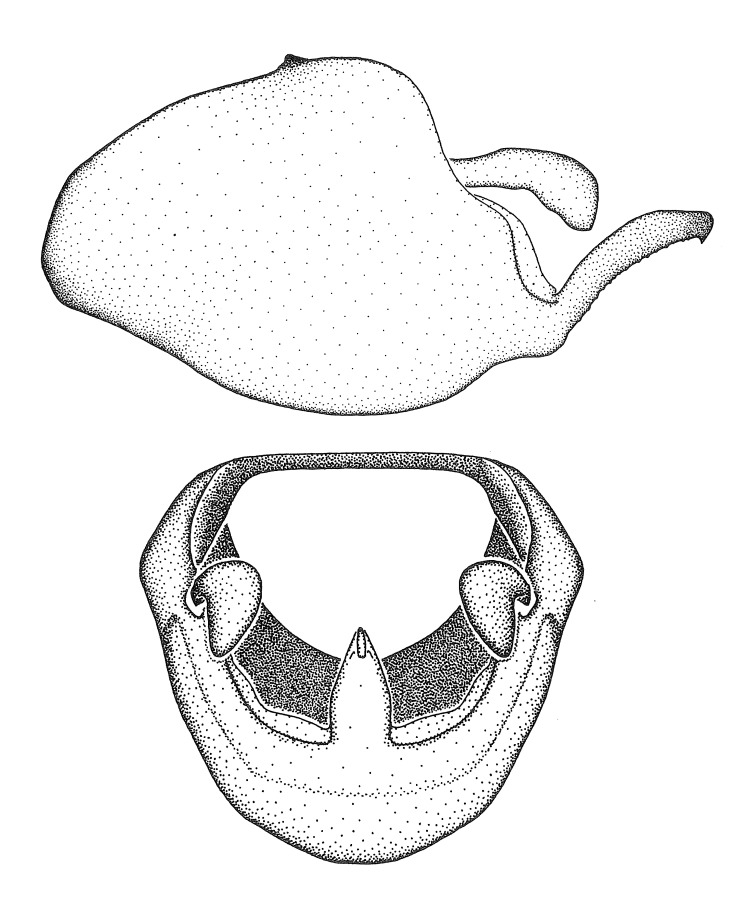
*Zelus cordazulus* Zhang & Hart, sp. n., pygophore, lateral and posterior views

**Figure 61b.**
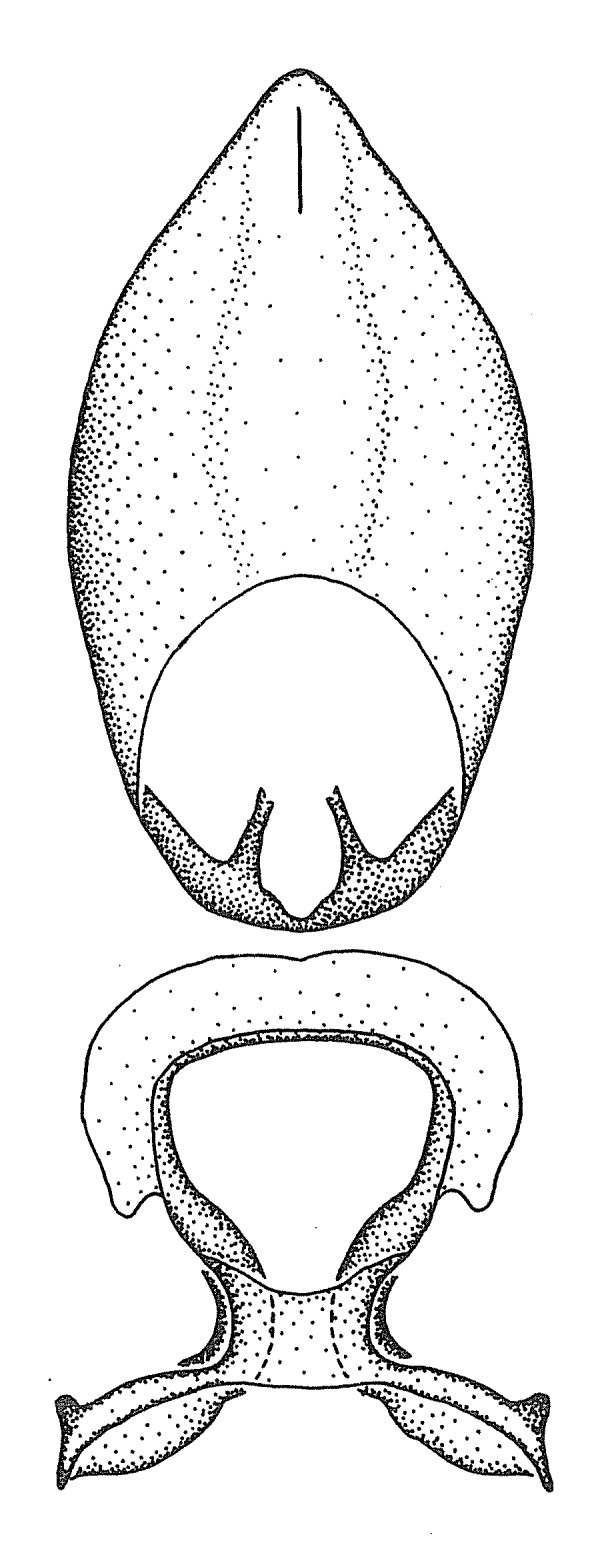
*Zelus cordazulus* Zhang & Hart, sp. n., phallus, dorsal view

**Figure 63a.**
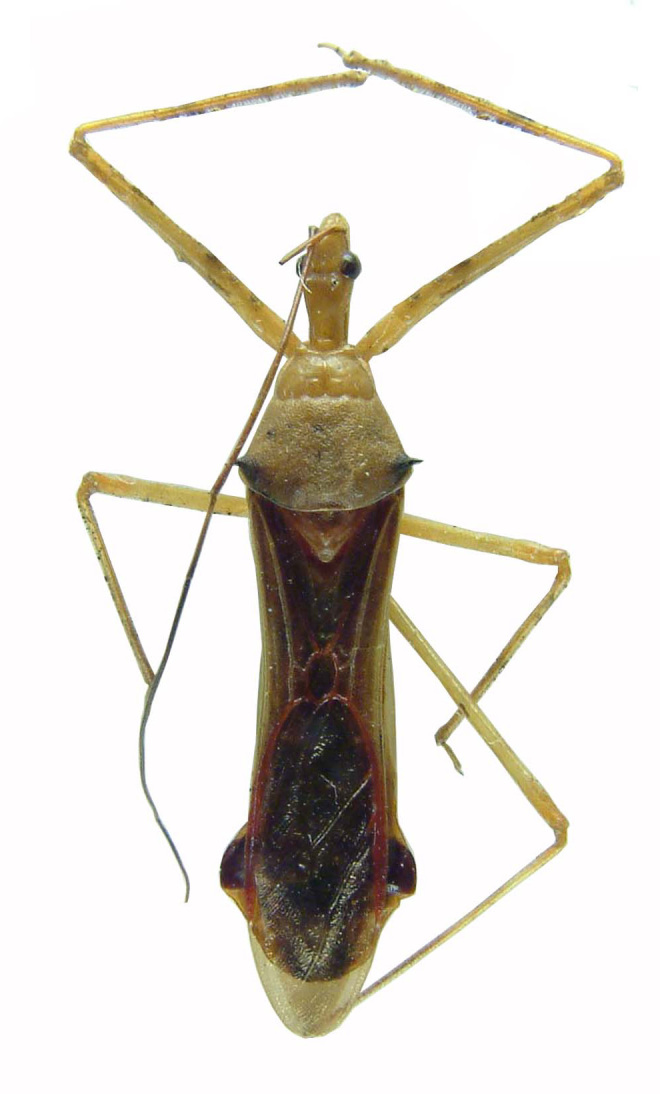
*Zelus couturieri* (Berenger, 2003), female, dorsal view

**Figure 63b.**
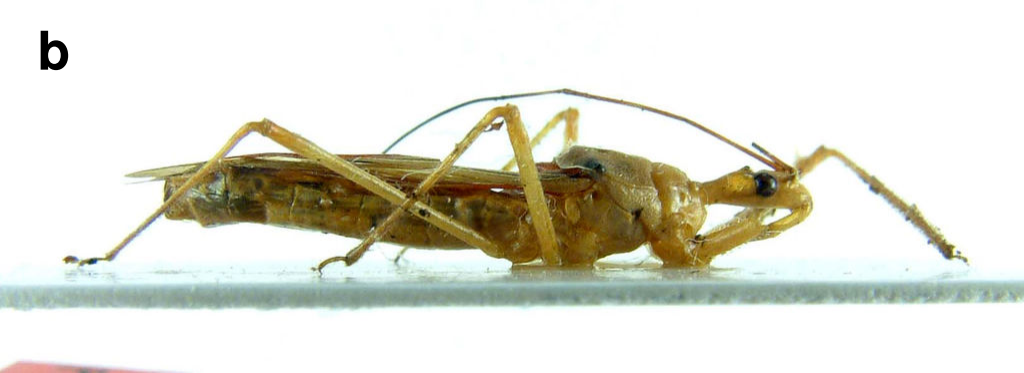
*Zelus couturieri* (Berenger, 2003), female, lateral view

**Figure 65a.**
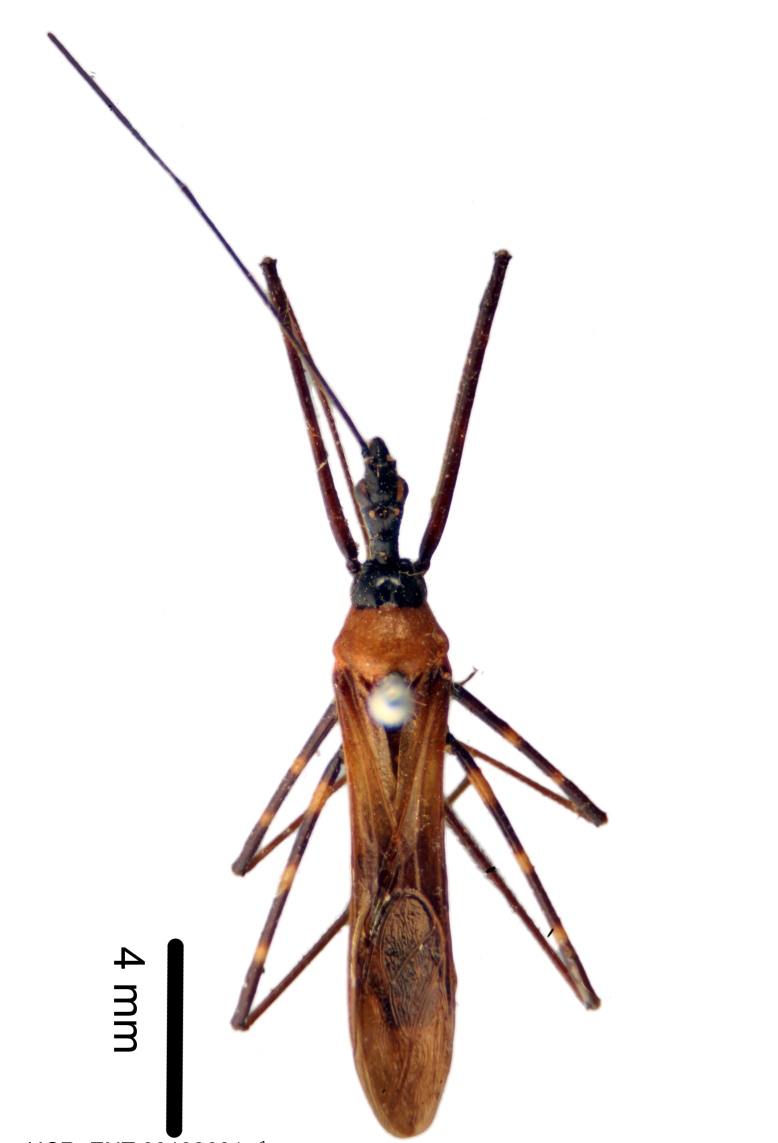
*Zelus errans* Fabricius, 1803, male, dorsal view

**Figure 65b.**
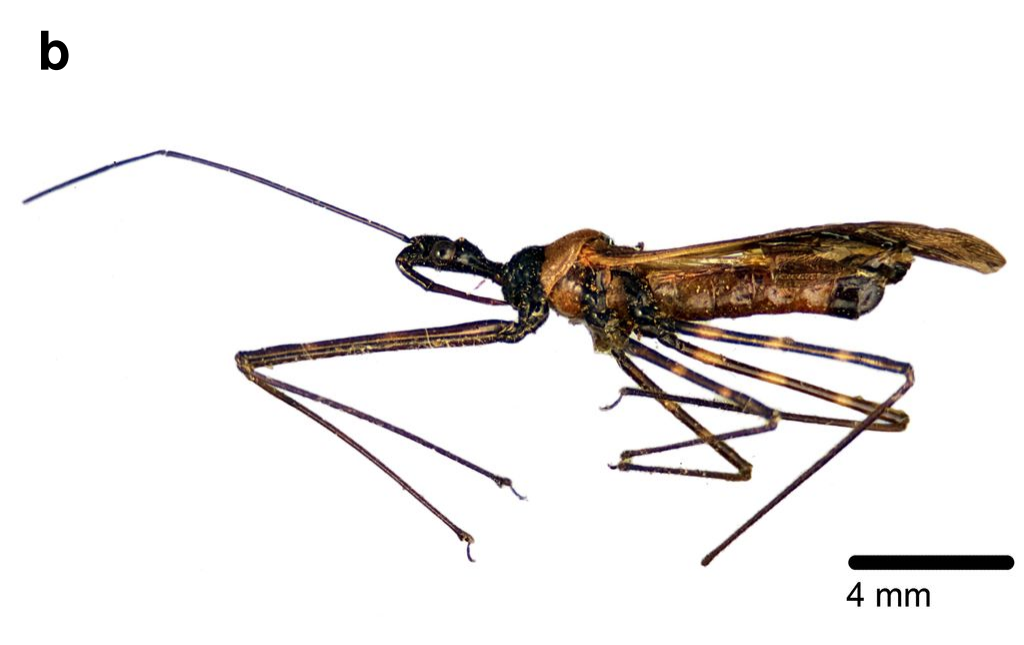
*Zelus errans* Fabricius, 1803, male, lateral view

**Figure 65c.**
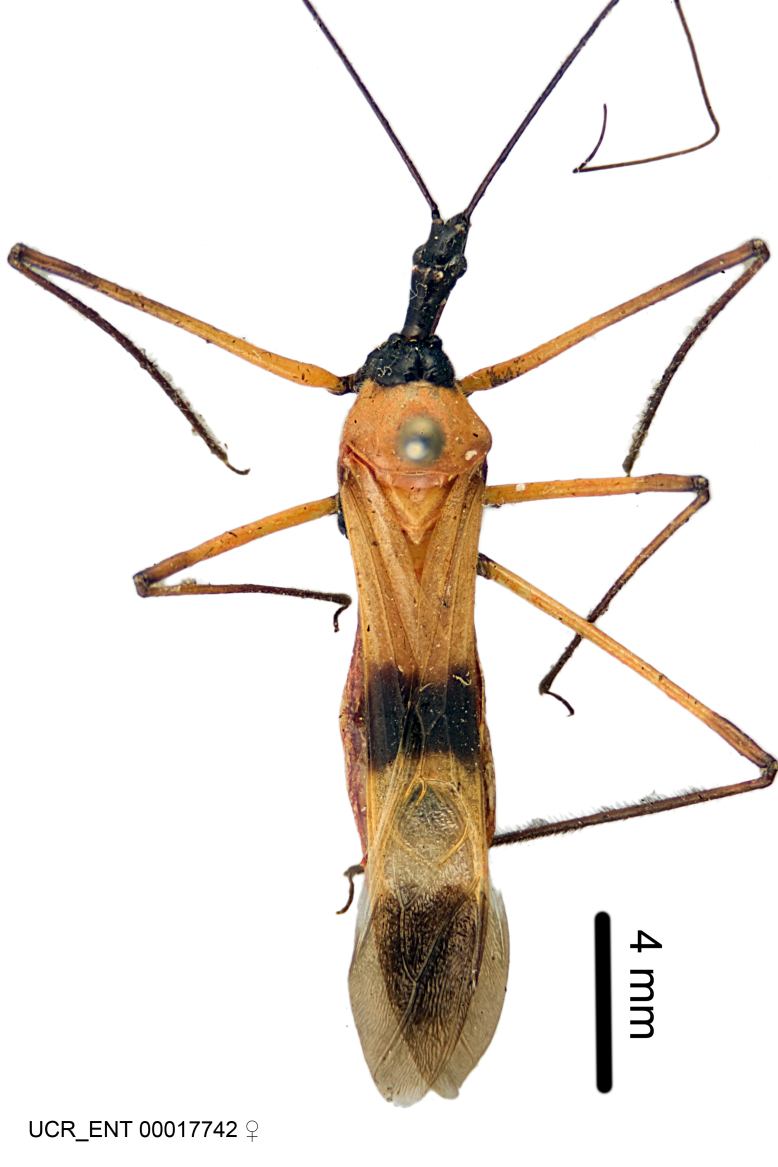
*Zelus errans* Fabricius, 1803, female, dorsal view (UCR_ENT 00017742, El Beni, Bolivia)

**Figure 65d.**
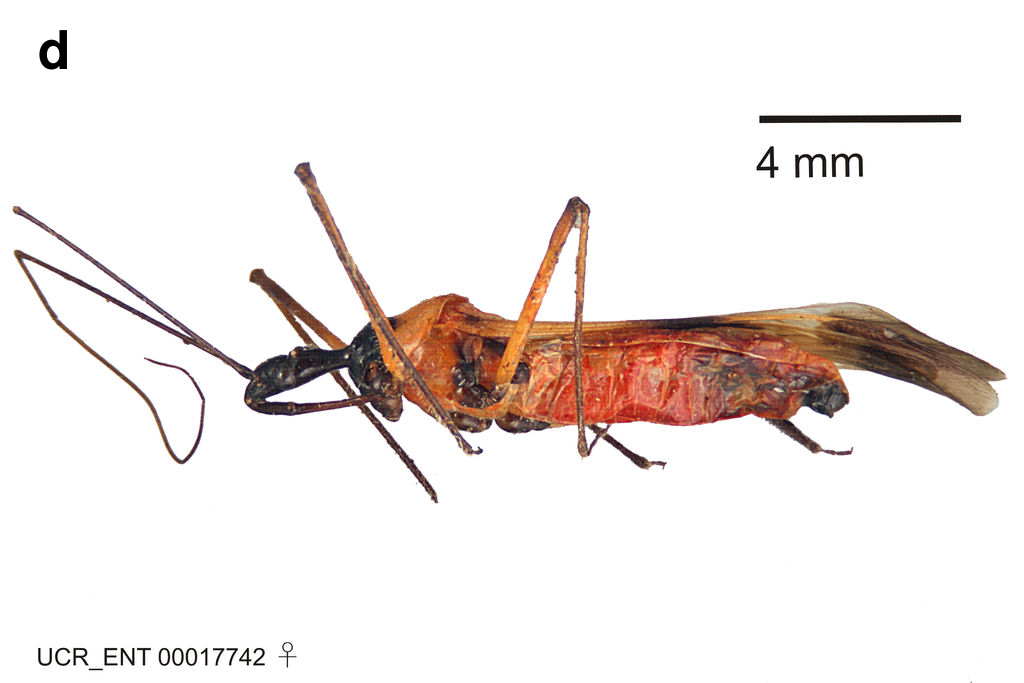
*Zelus errans* Fabricius, 1803, female, lateral view (UCR_ENT 00017742, El Beni, Bolivia)

**Figure 65e.**
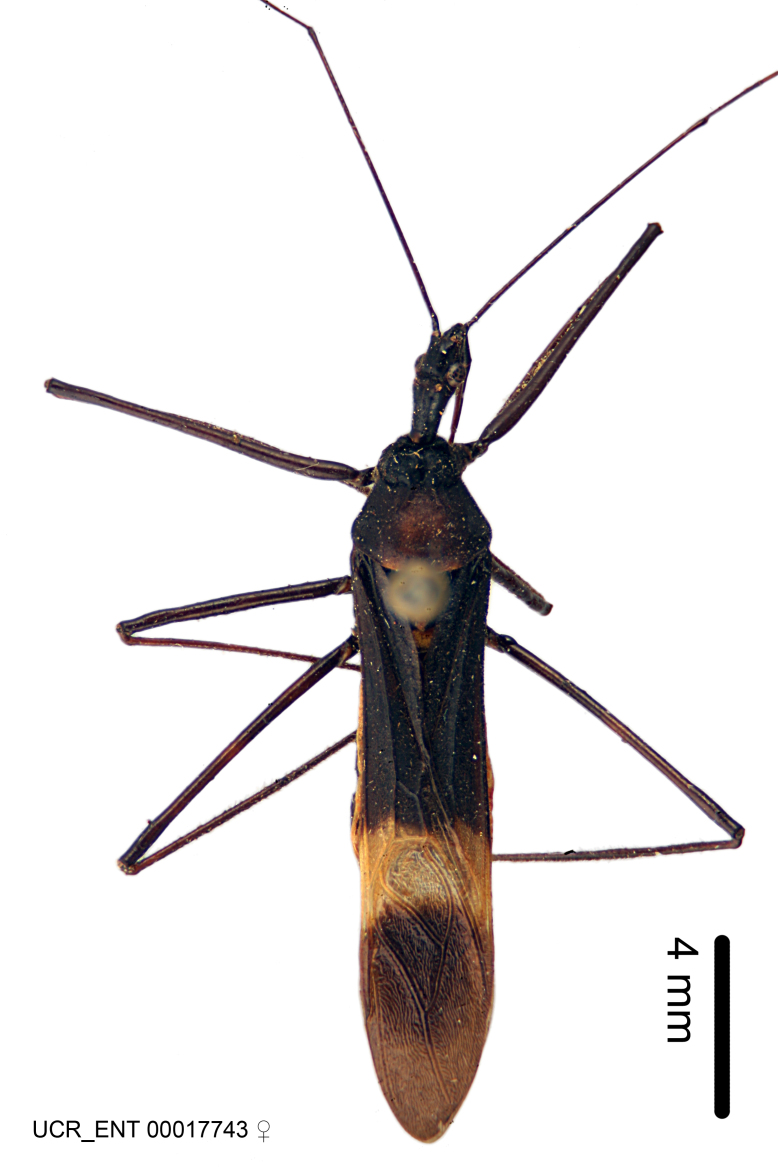
*Zelus errans* Fabricius, 1803, female, dorsal view (UCR_ENT 00017743, Parana, Brazil)

**Figure 66a.**
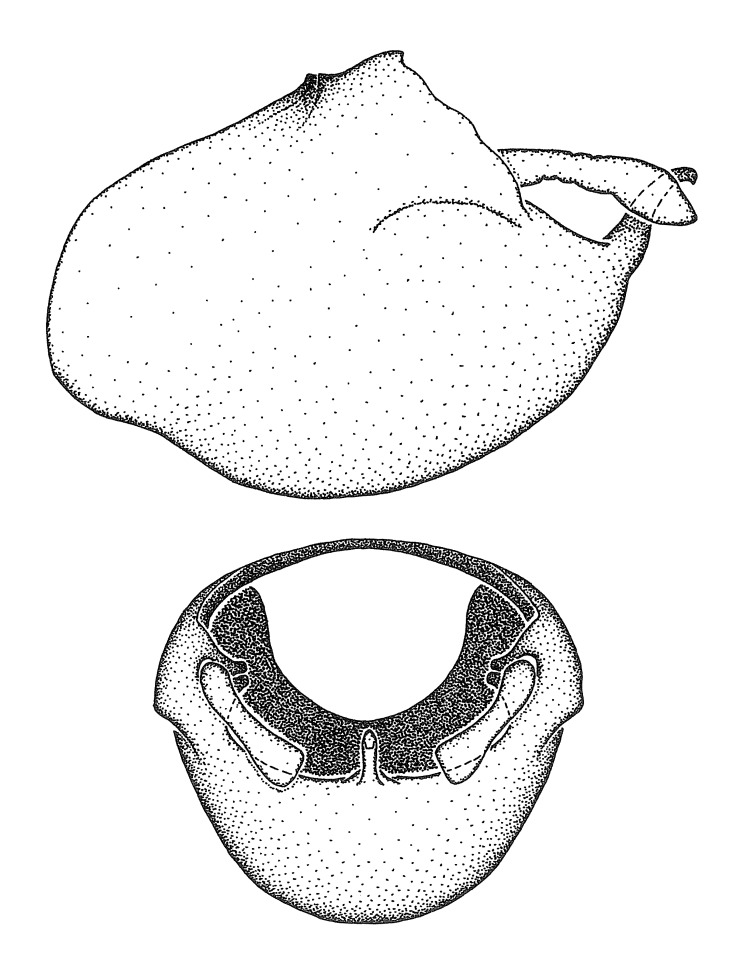
*Zelus errans* Fabricius 1803, pygophore, lateral and posterior views

**Figure 66b.**
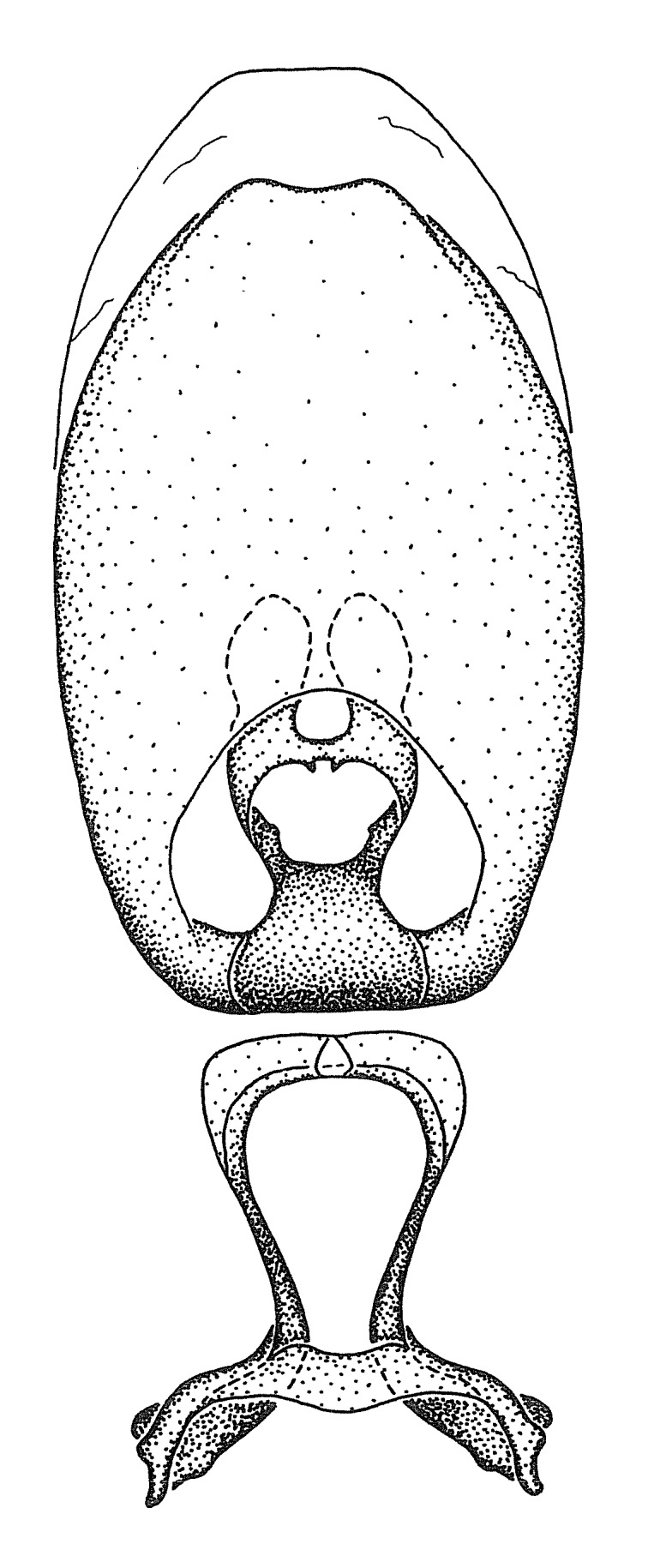
*Zelus errans* Fabricius 1803, phallus, dorsal view

**Figure 68a.**
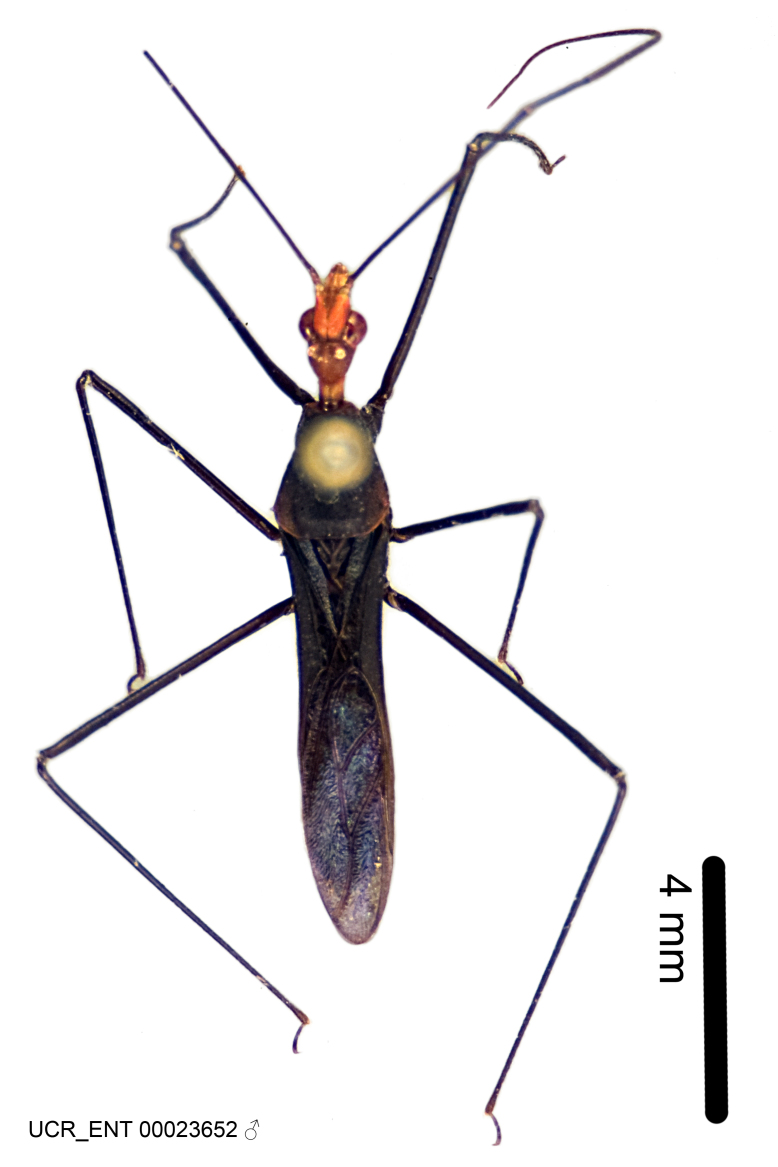
*Zelus erythrocephalus* Fabricius, 1803, male, dorsal view (UCR_ENT 00023652, Suriname)

**Figure 68b.**
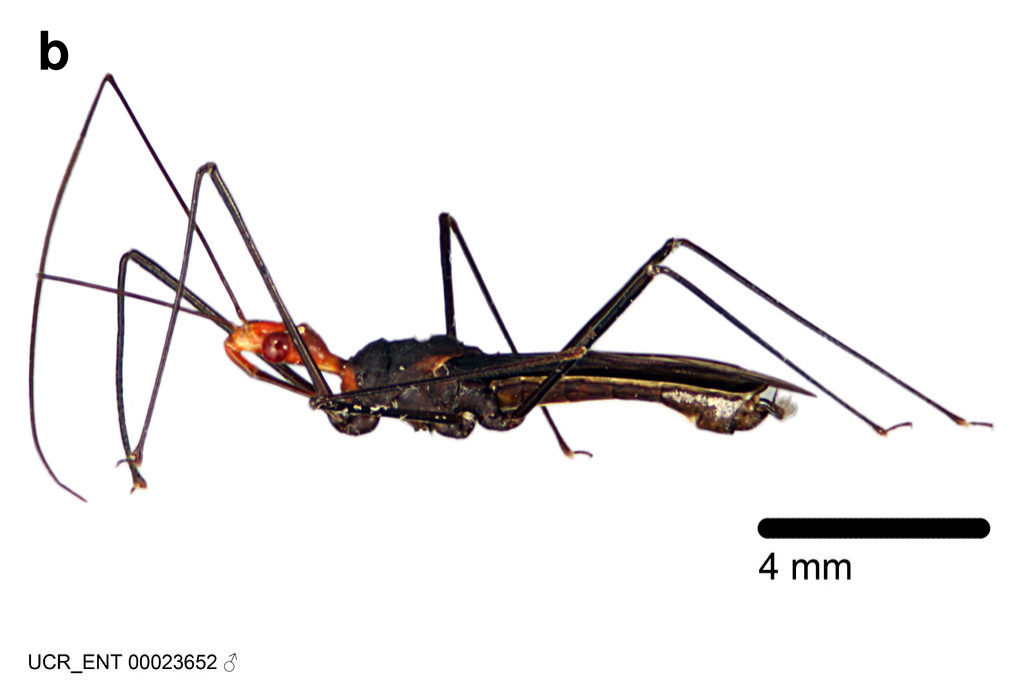
*Zelus erythrocephalus* Fabricius, 1803, male, lateral view (UCR_ENT 00023652, Suriname)

**Figure 68c.**
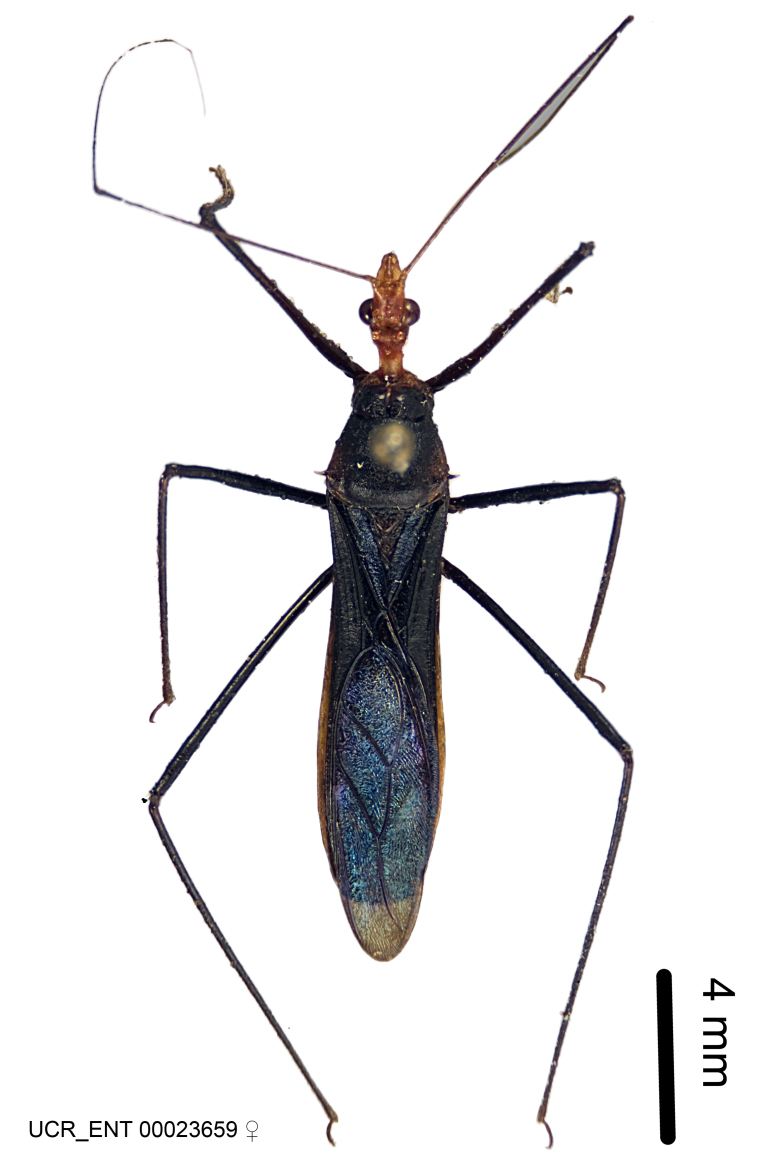
*Zelus erythrocephalus* Fabricius, 1803, female, dorsal view (UCR_ENT 00023659, Suriname)

**Figure 68d.**
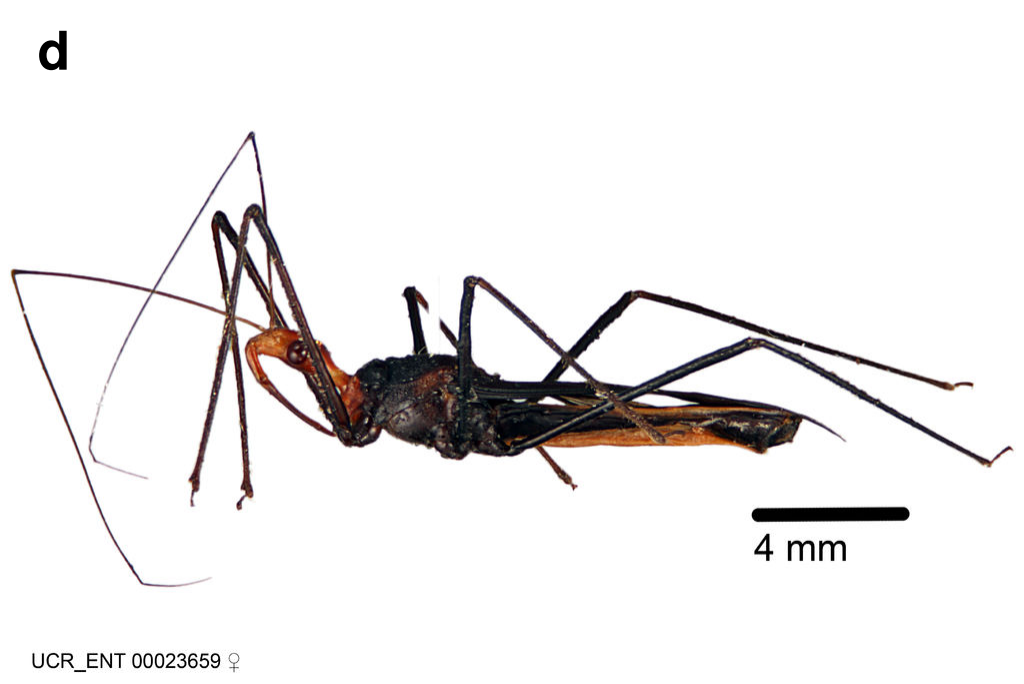
*Zelus erythrocephalus* Fabricius, 1803, female, lateral view (UCR_ENT 00023659, Suriname)

**Figure 69a.**
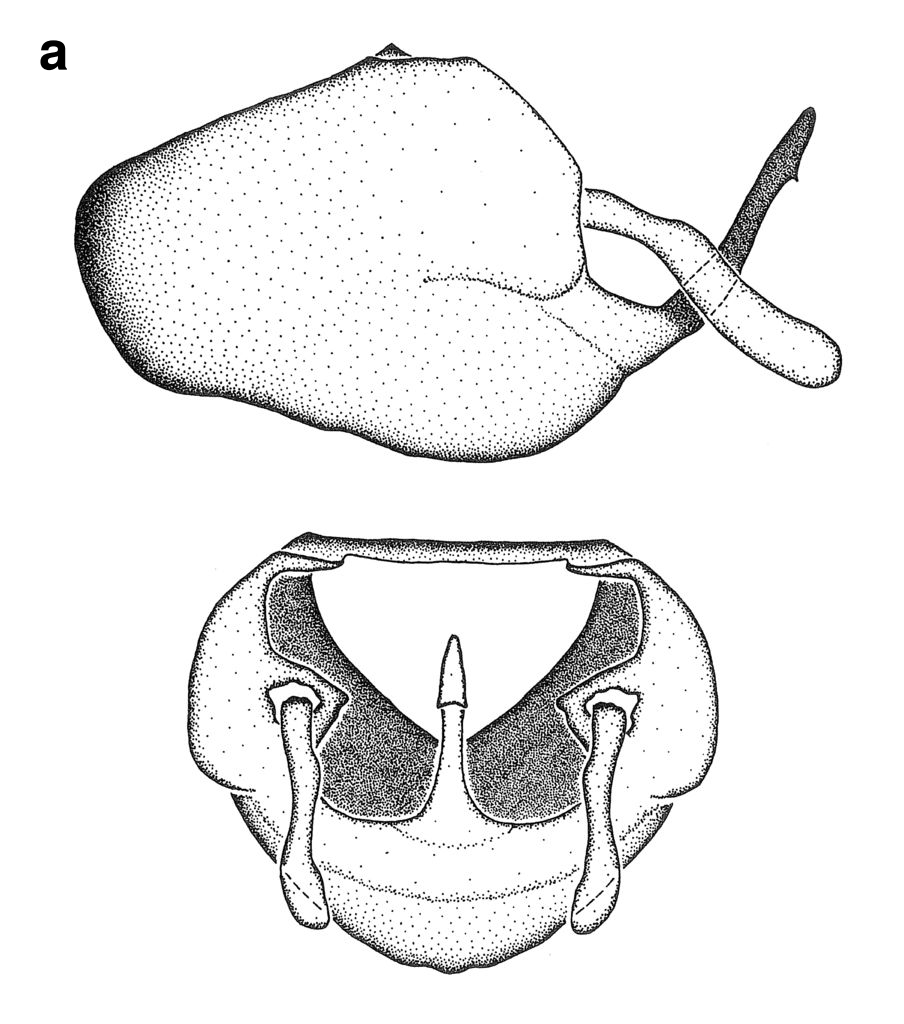
*Zelus erythrocephalus* Fabricius, 1803, pygophore, lateral and posterior views

**Figure 69b.**
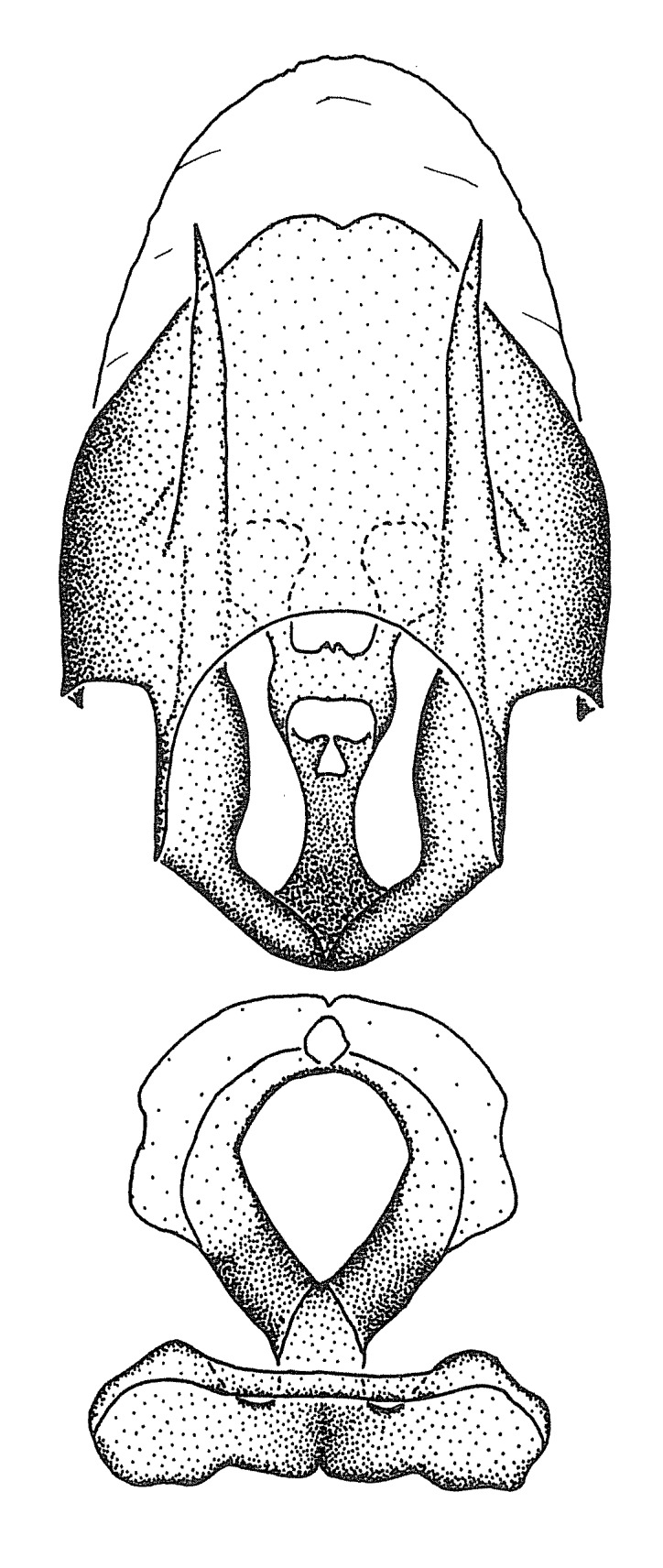
*Zelus erythrocephalus* Fabricius, 1803, phallus, dorsal view

**Figure 71a.**
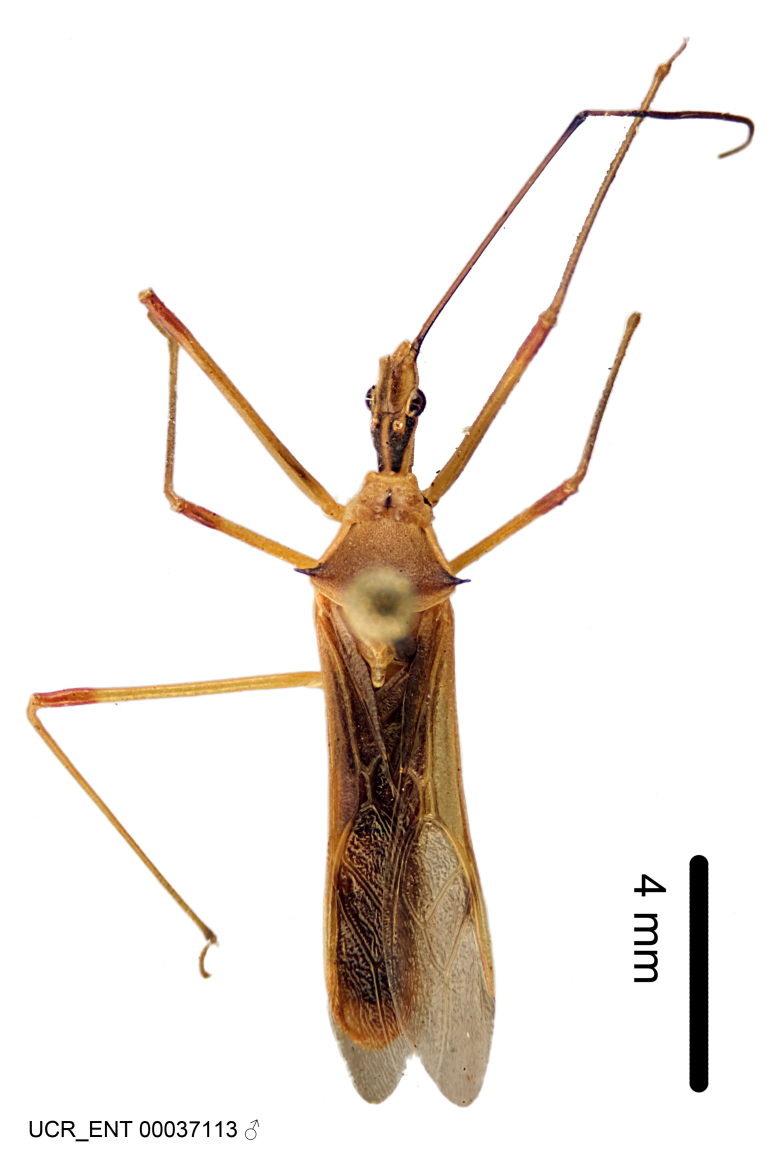
*Zelus exsanguis* Stål, 1862, male, dorsal view (UCR_ENT 00037113, Chiriqui, Panama)

**Figure 71b.**
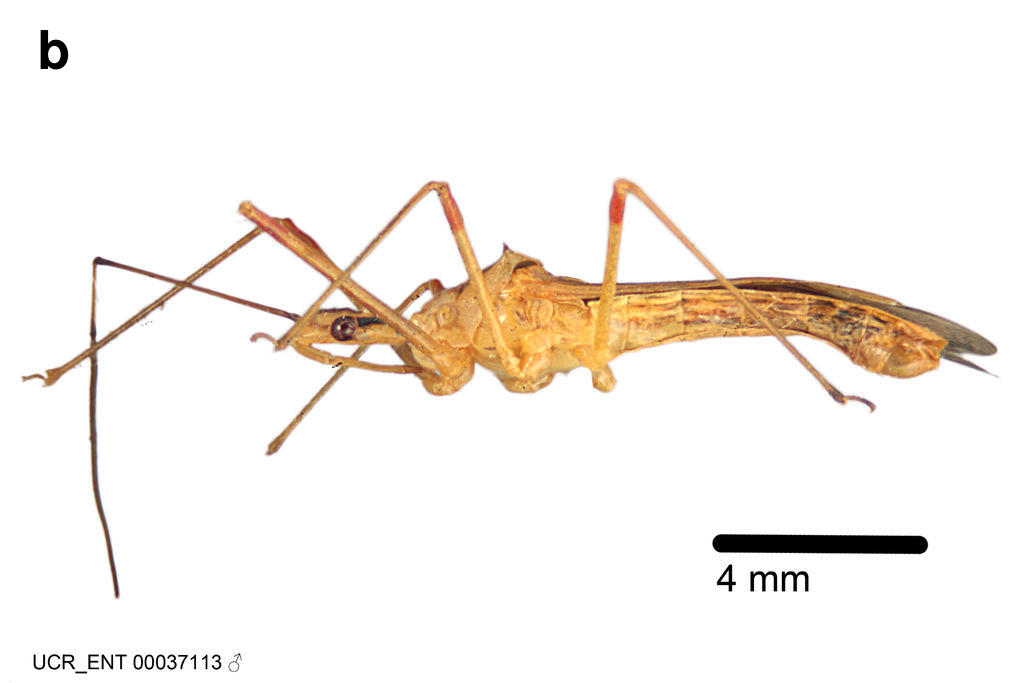
*Zelus exsanguis* Stål, 1862, male, lateral view (UCR_ENT 00037113, Chiriqui, Panama)

**Figure 71c.**
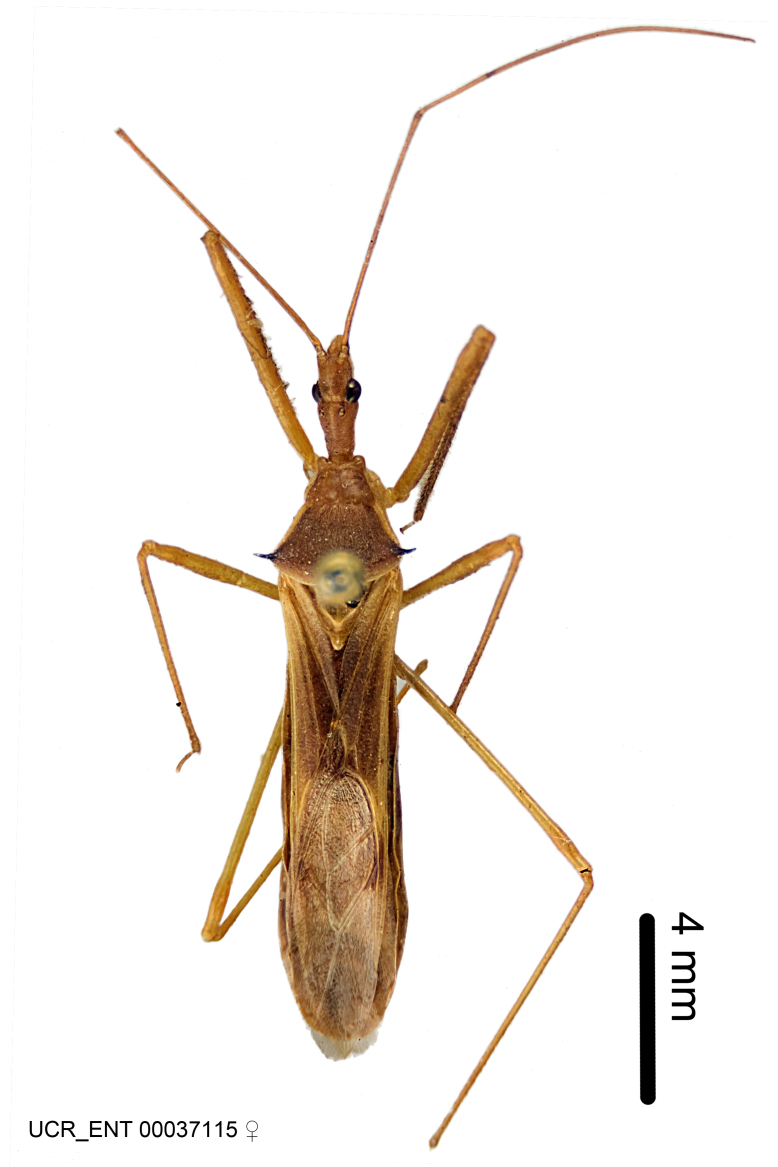
*Zelus exsanguis* Stål, 1862, female, dorsal view (UCR_ENT 00037115, Veracruz, Mexico)

**Figure 71d.**
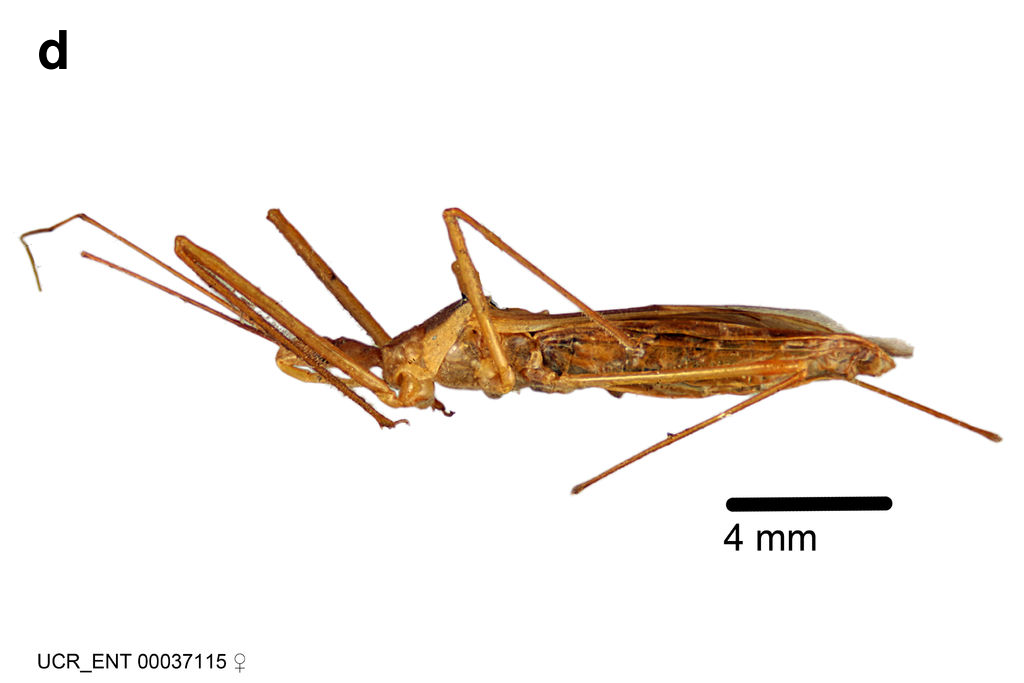
*Zelus exsanguis* Stål, 1862, female, lateral view (UCR_ENT 00037115, Veracruz, Mexico)

**Figure 72a.**
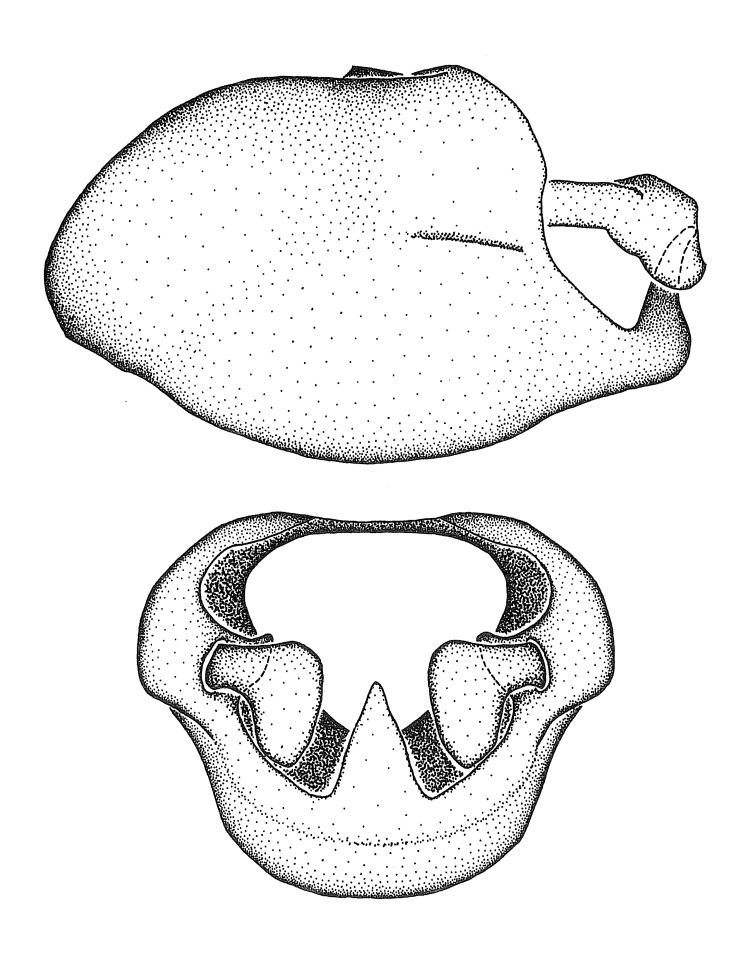
*Zelus exsanguis* Stål 1862, southern population, pygophore, lateral and posterior views

**Figure 72b.**
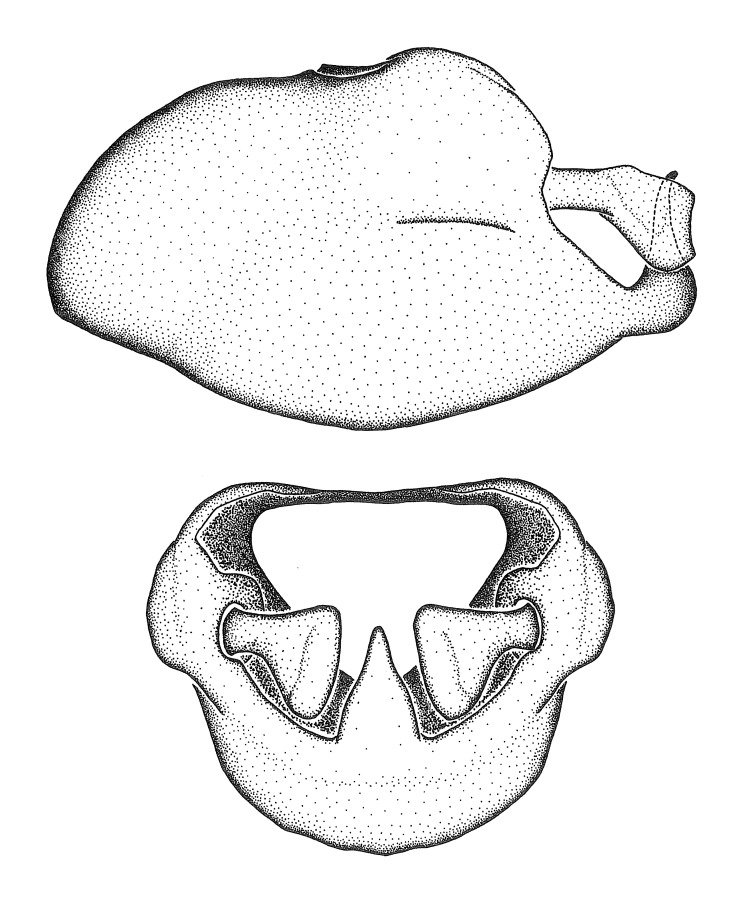
*Zelus exsanguis* Stål 1862, northern population, pygophore, lateral and posterior views

**Figure 72c.**
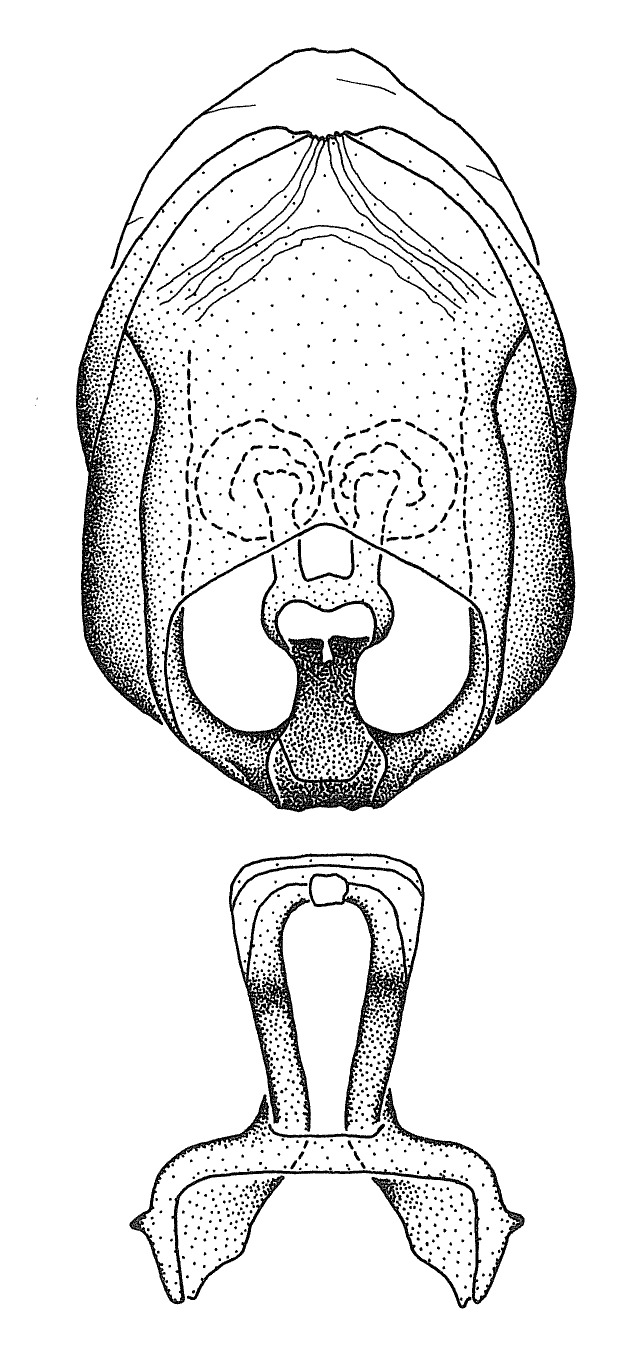
*Zelus exsanguis* Stål 1862, southern population, phallus, dorsal view

**Figure 72d.**
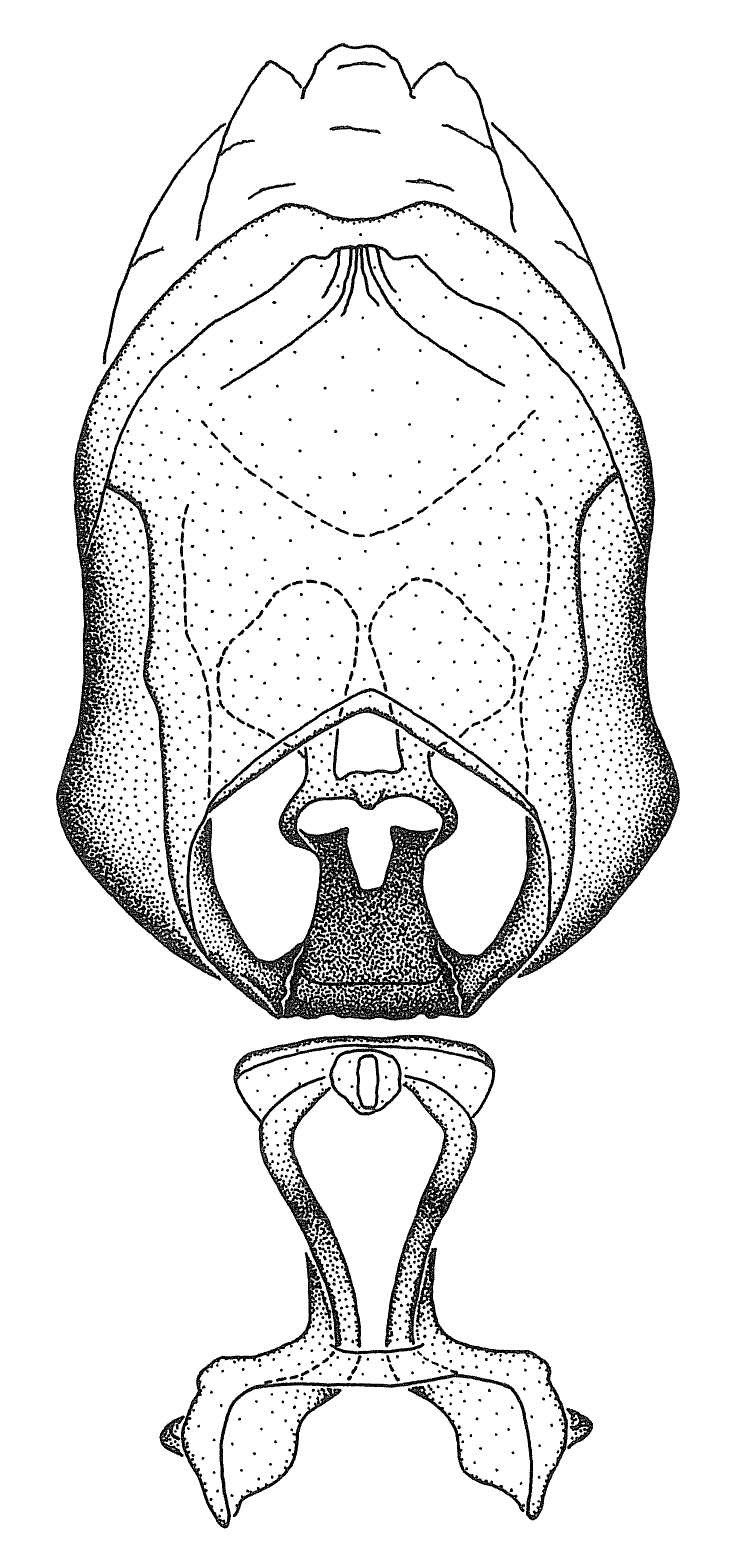
*Zelus exsanguis* Stål 1862, northern population, phallus, dorsal view

**Figure 74a.**
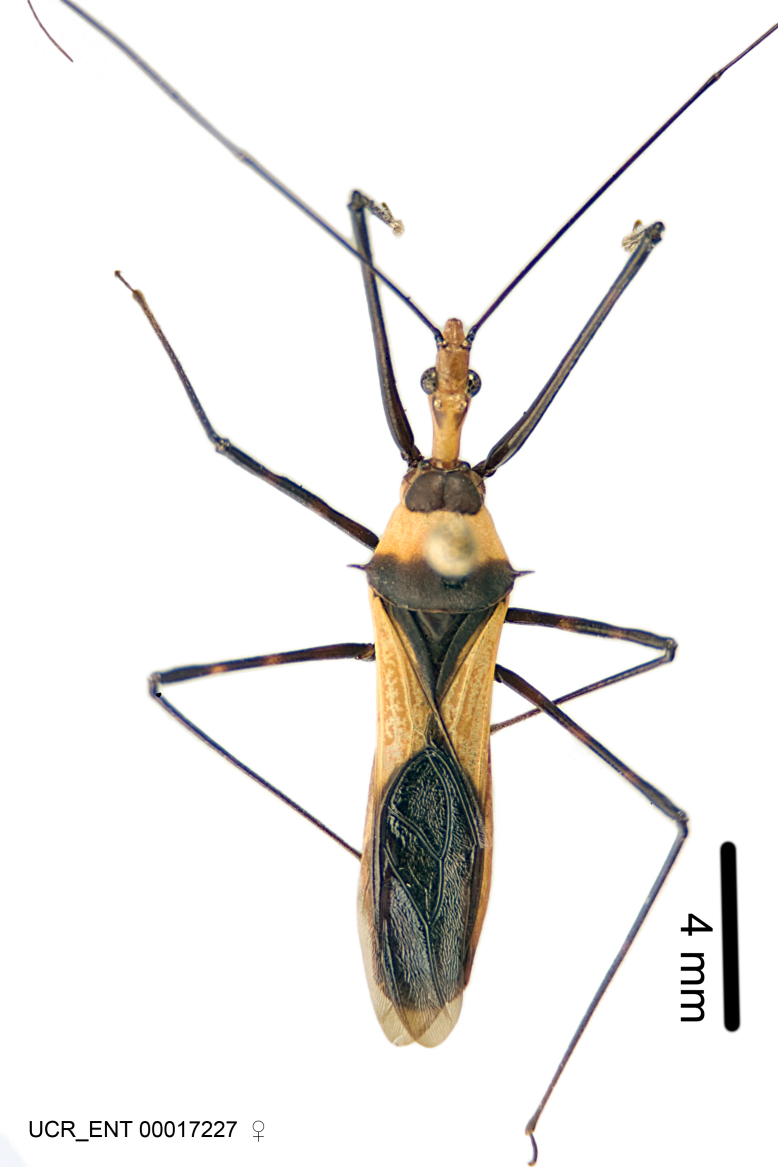
*Zelus fasciatus* Champion, 1899, female, dorsal view (UCR_ENT 00017227, Colon, Panama)

**Figure 74b.**
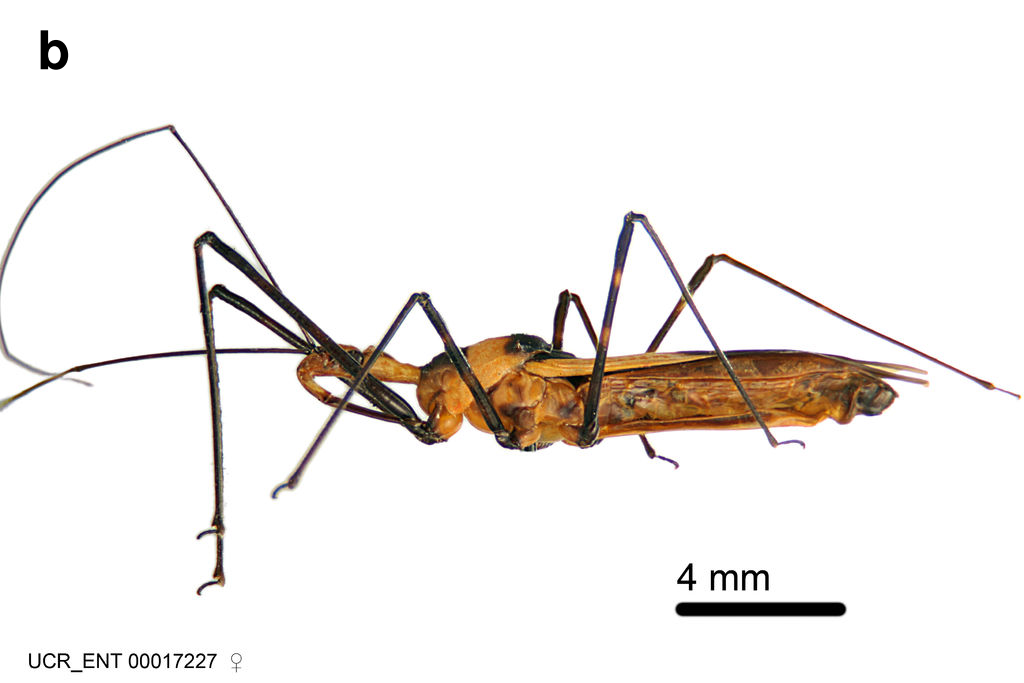
*Zelus fasciatus* Champion, 1899, female, lateral view (UCR_ENT 00017227, Colon, Panama)

**Figure 76a.**
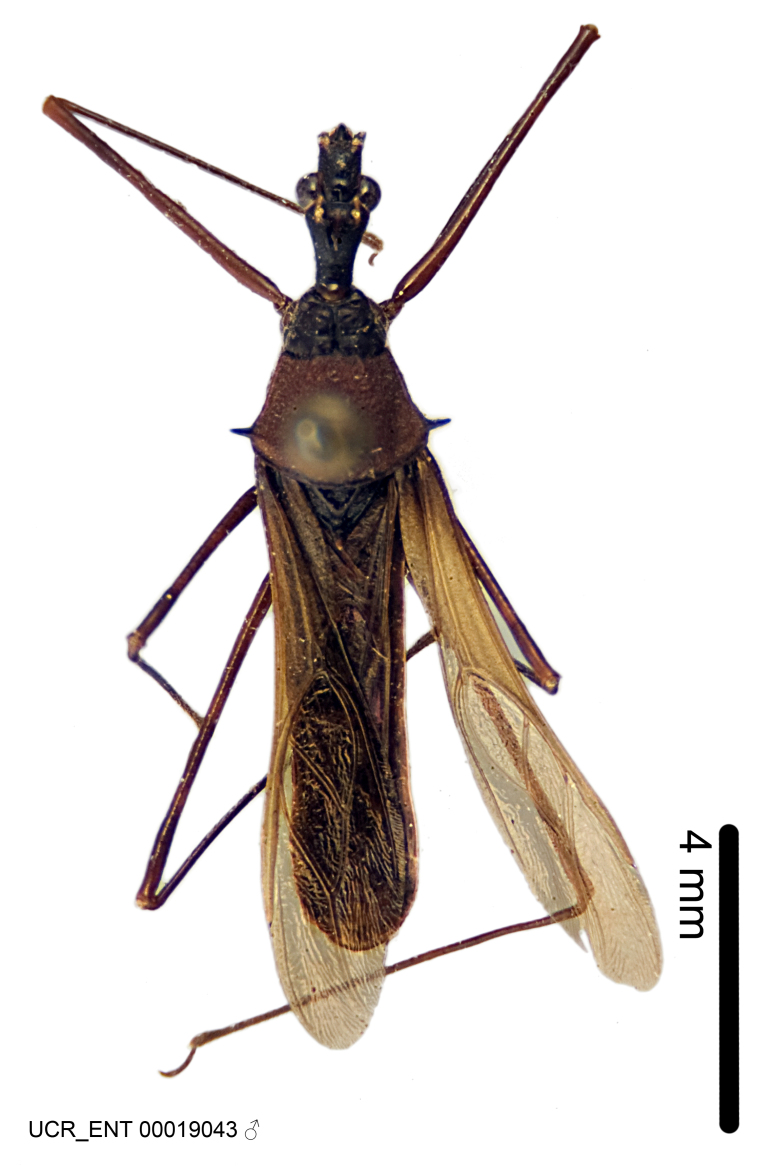
*Zelus filicauda* Bergroth, 1893, male, dorsal view (UCR_ENT 00019043, Tungurahua, Ecuador)

**Figure 76b.**
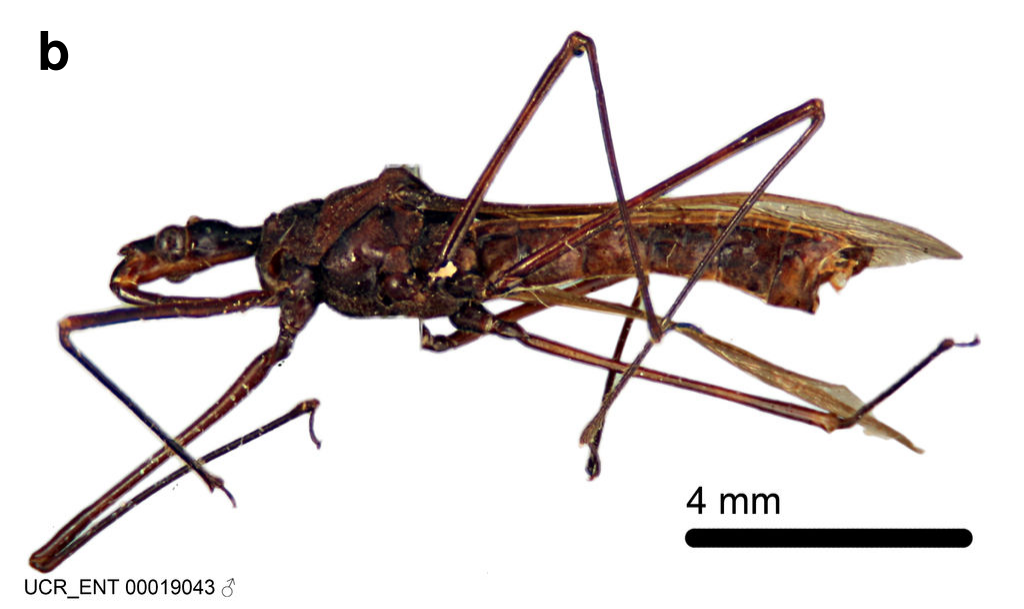
*Zelus filicauda* Bergroth, 1893, male, lateral view (UCR_ENT 00019043, Tungurahua, Ecuador)

**Figure 77a.**
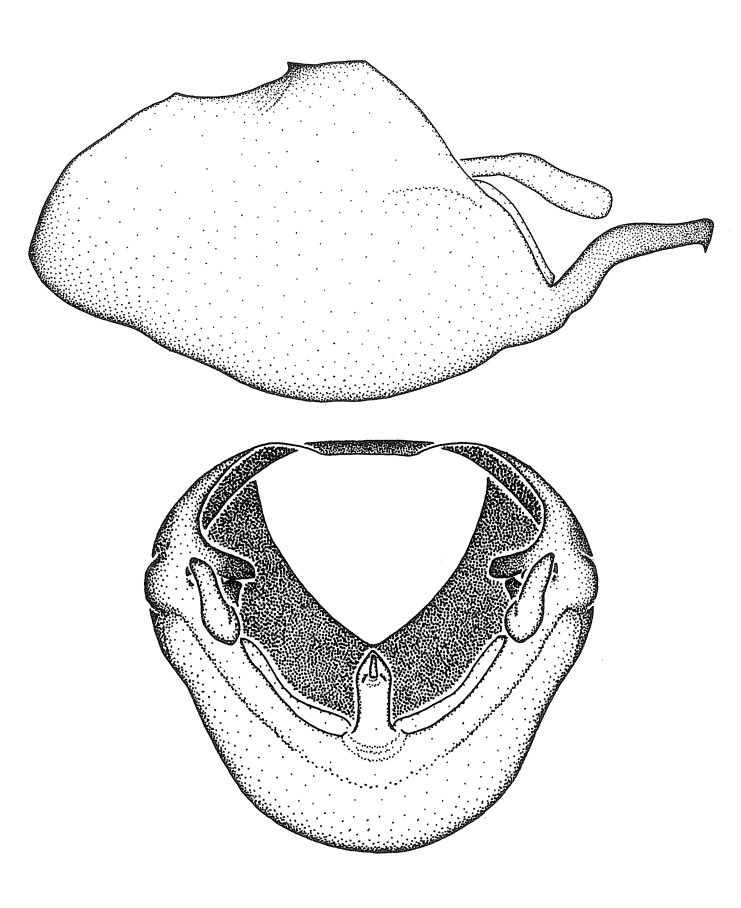
*Zelus filicauda* Bergroth, 1893, pygophore, lateral and posterior views

**Figure 77b.**
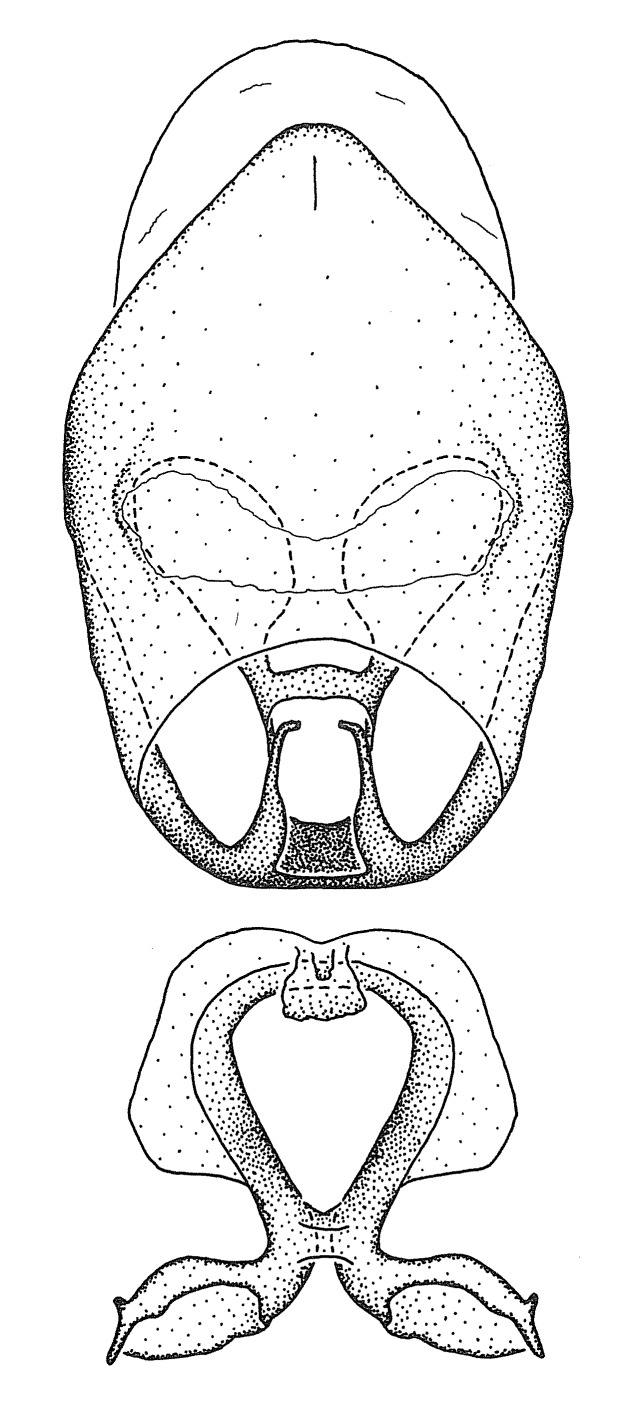
*Zelus filicauda* Bergroth, 1893, phallus, dorsal view

**Figure 79a.**
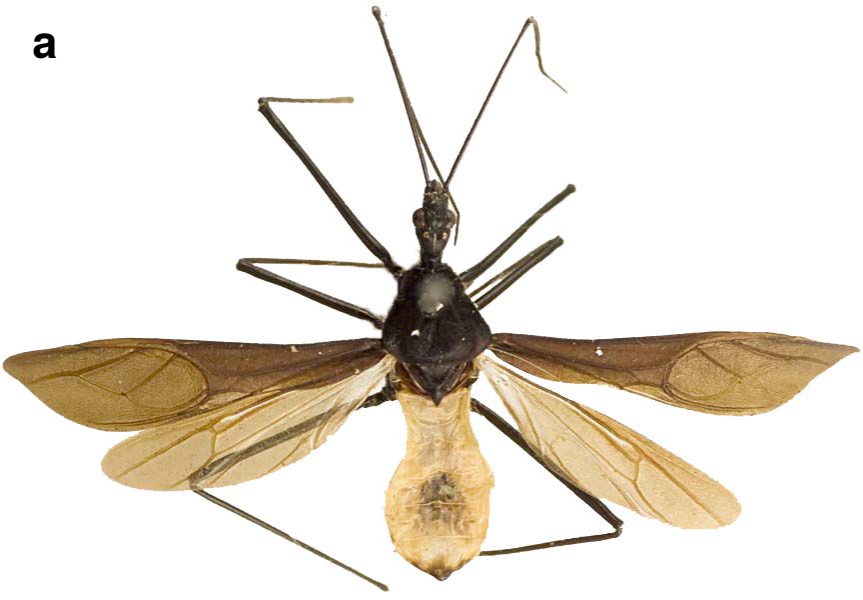
*Zelus fuliginatus* Zhang & Hart, sp. n., male, dorsal view (UCR_ENT 00007997, Colombia)

**Figure 79b.**
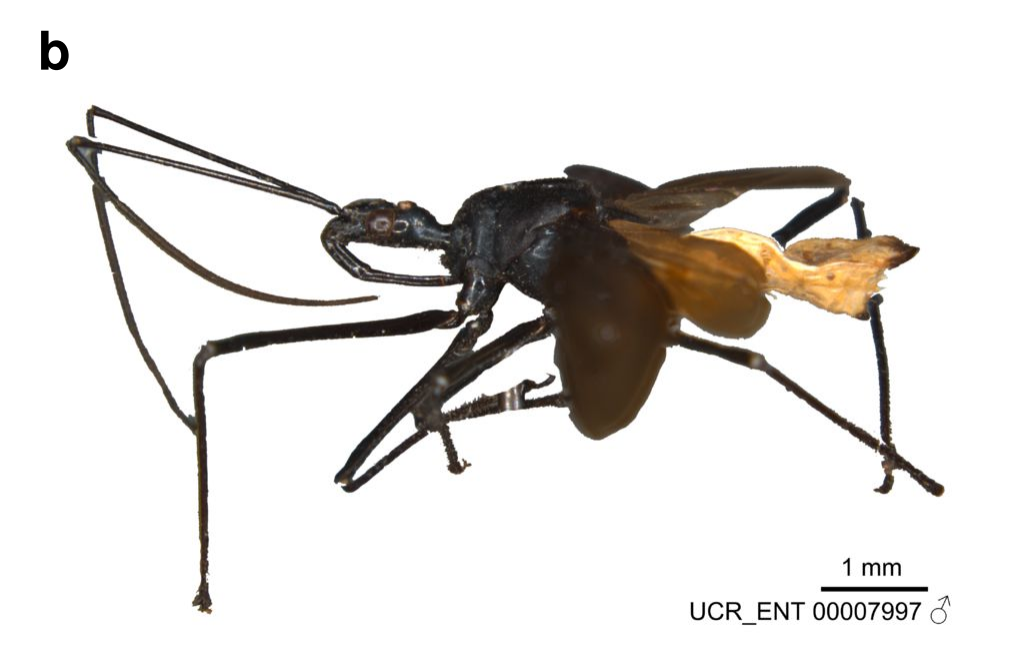
*Zelus fuliginatus* Zhang & Hart, sp. n., male, lateral view (UCR_ENT 00007997, Colombia)

**Figure 79c.**
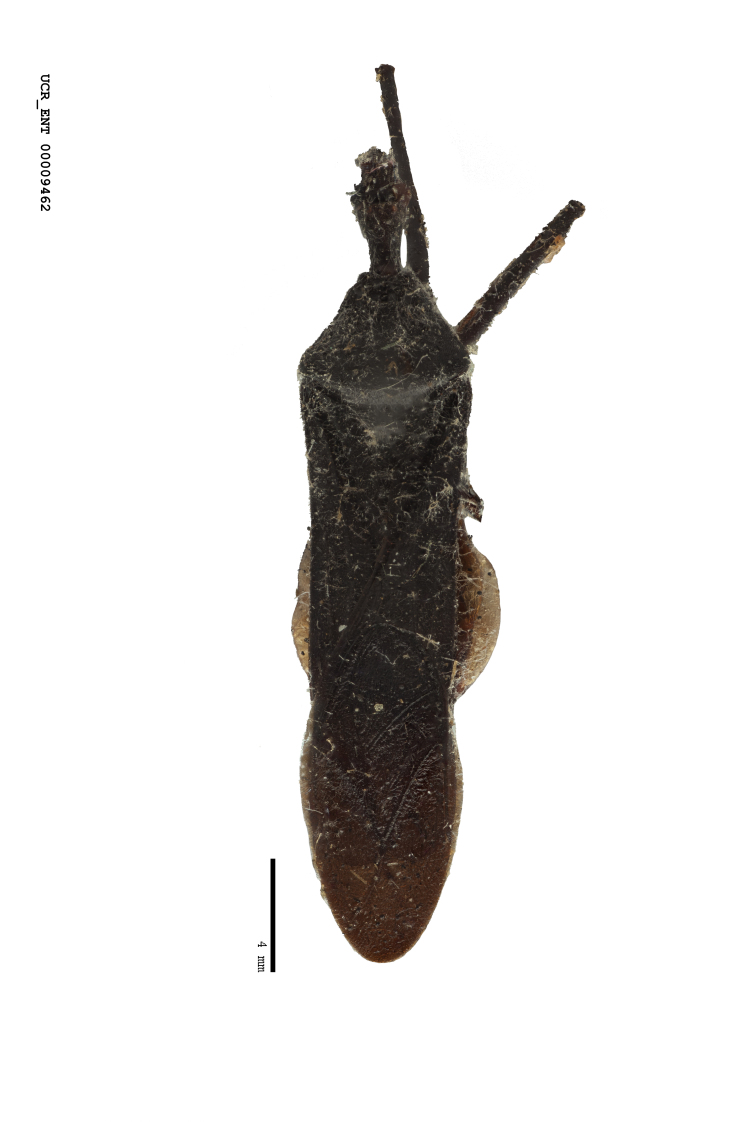
*Zelus fuliginatus* Zhang & Hart, sp. n., female, dorsal view (UCR_ENT 00009462, Santander, Colombia)

**Figure 79d.**
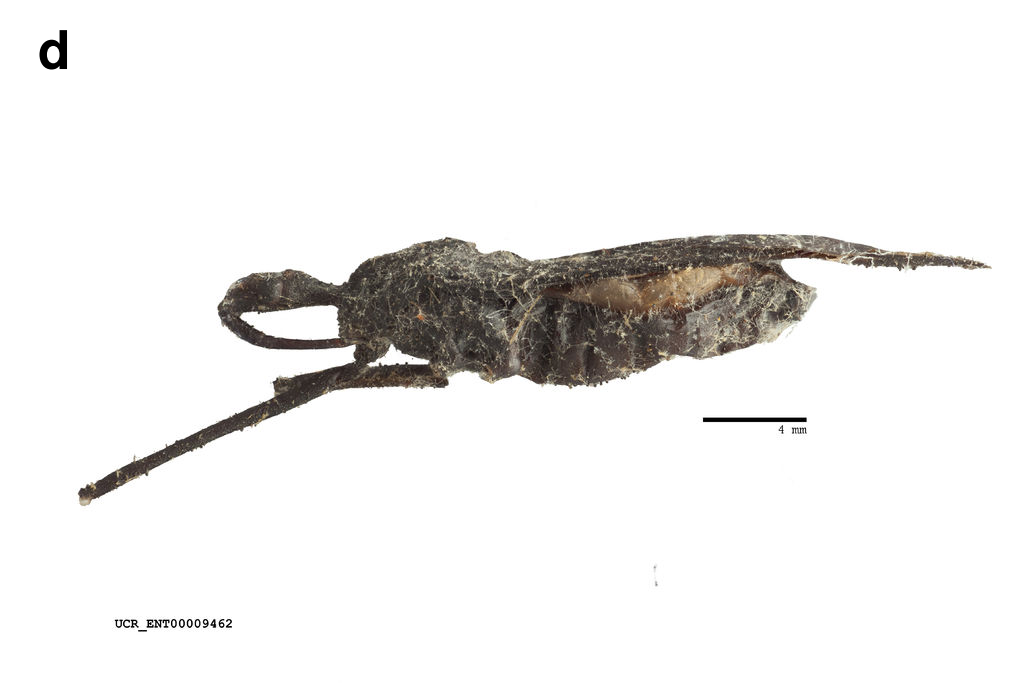
*Zelus fuliginatus* Zhang & Hart, sp. n., female, lateral view (UCR_ENT 00009462, Santander, Colombia)

**Figure 80a.**
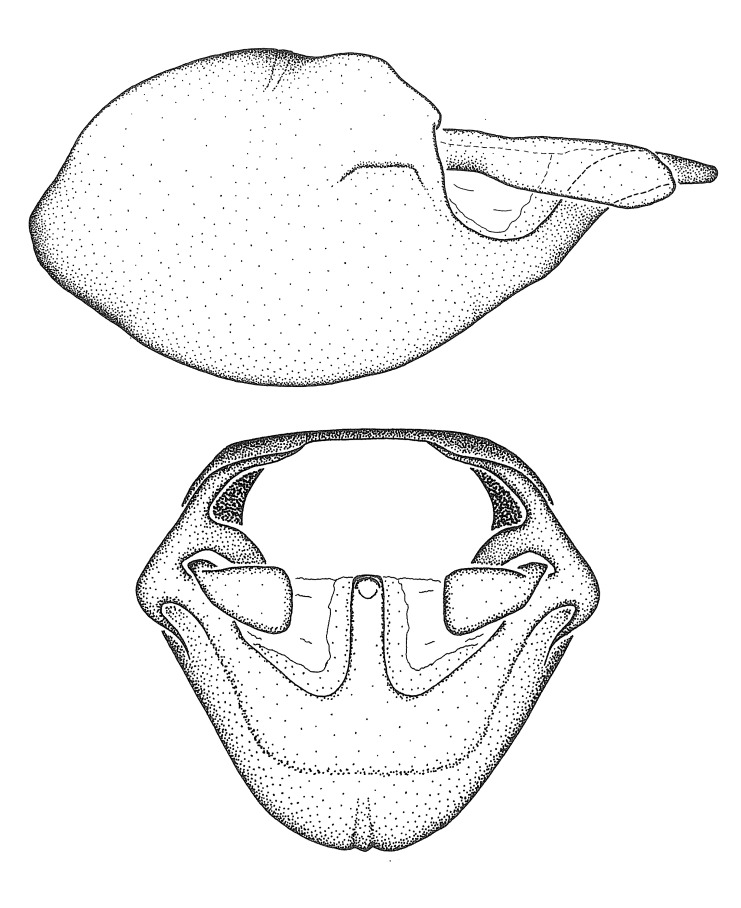
*Zelus fuliginatus* Zhang & Hart, sp. n., pygophore, lateral and posterior views

**Figure 80b.**
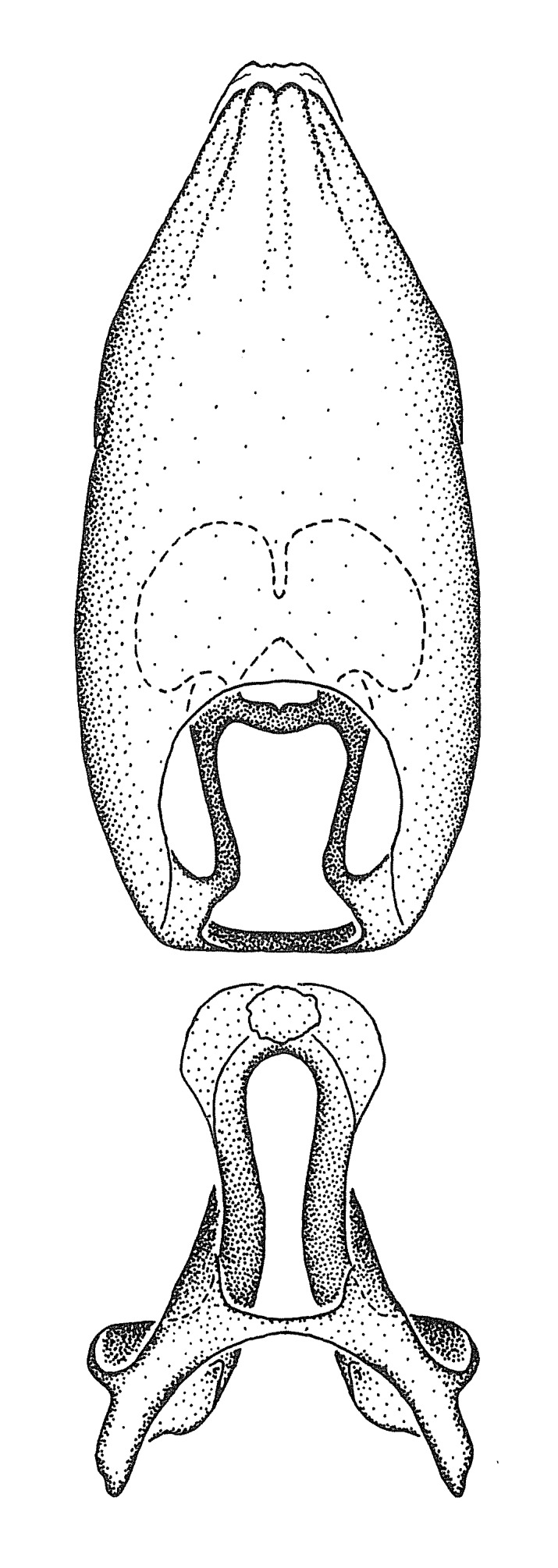
*Zelus fuliginatus* Zhang & Hart, sp. n., phallus, dorsal view

**Figure 82a.**
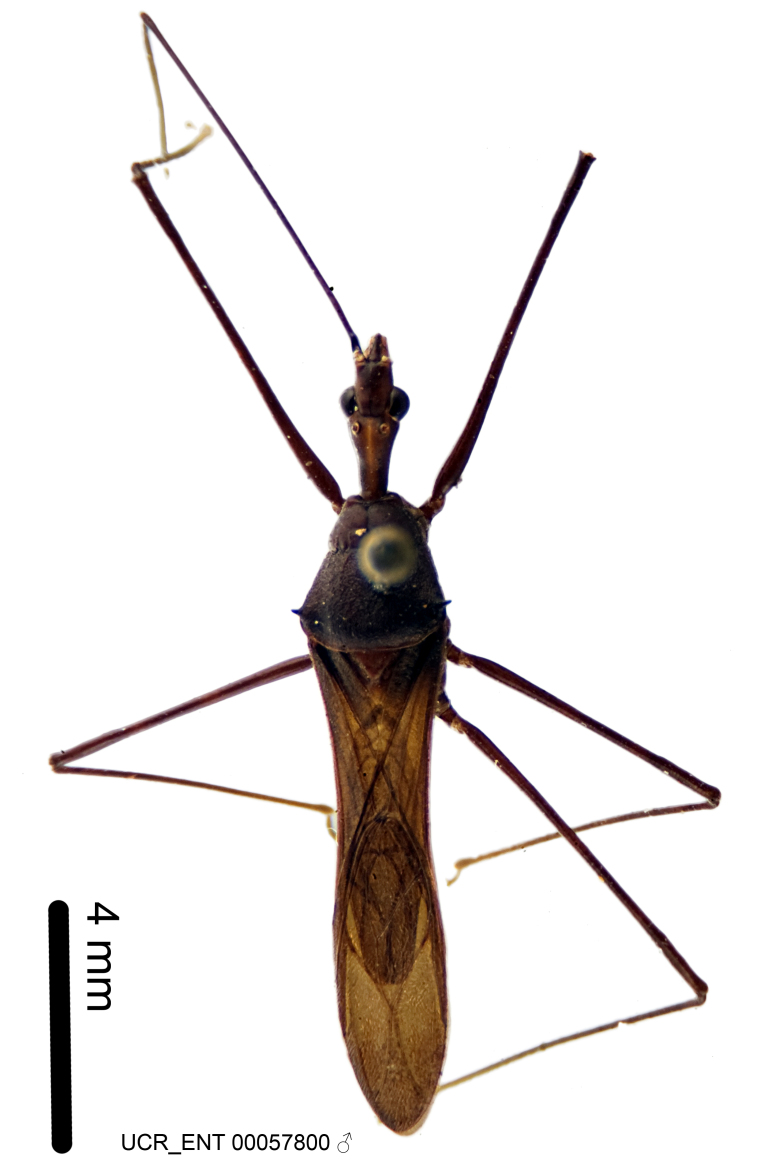
*Zelus gilboventris* Zhang & Hart, sp. n., male, dorsal view (UCR_ENT 00057800)

**Figure 82b.**
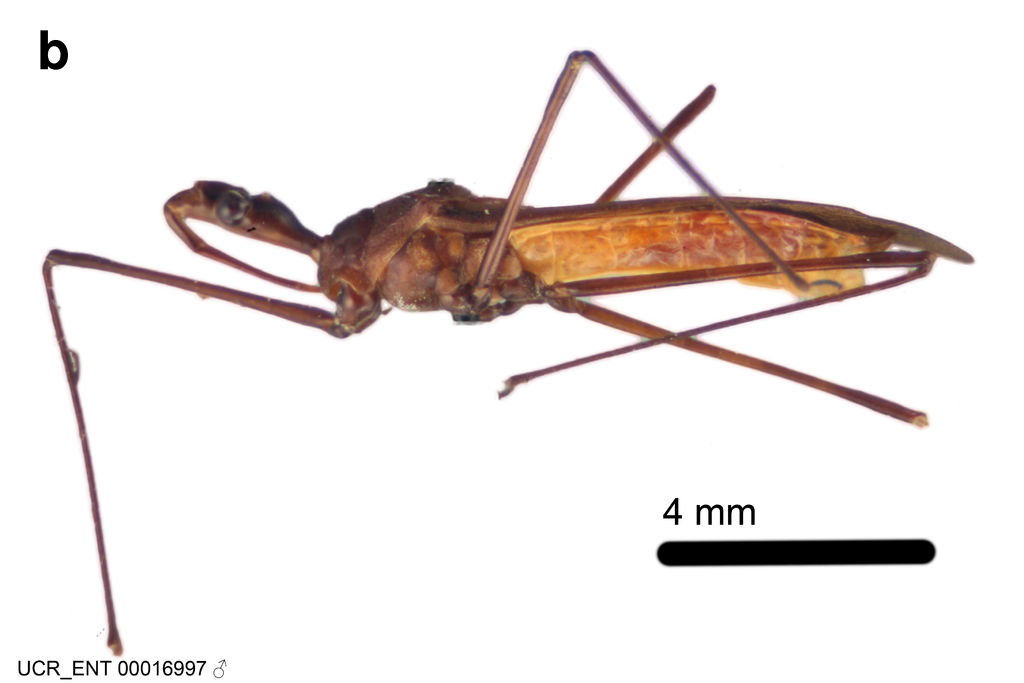
*Zelus gilboventris* Zhang & Hart, sp. n., male, lateral view (UCR_ENT 00016997, Huanuco, Peru)

**Figure 82c.**
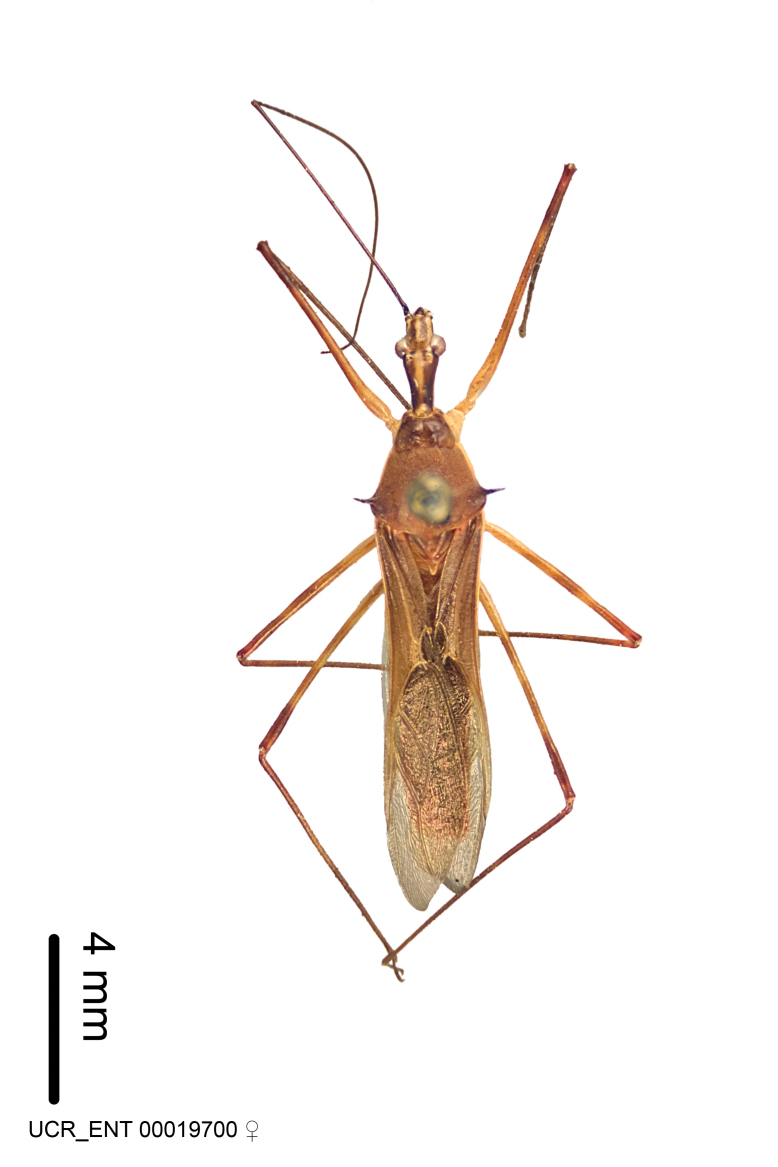
*Zelus gilboventris* Zhang & Hart, sp. n., female, dorsal view (UCR_ENT 00019700, Huanuco, Peru)

**Figure 82d.**
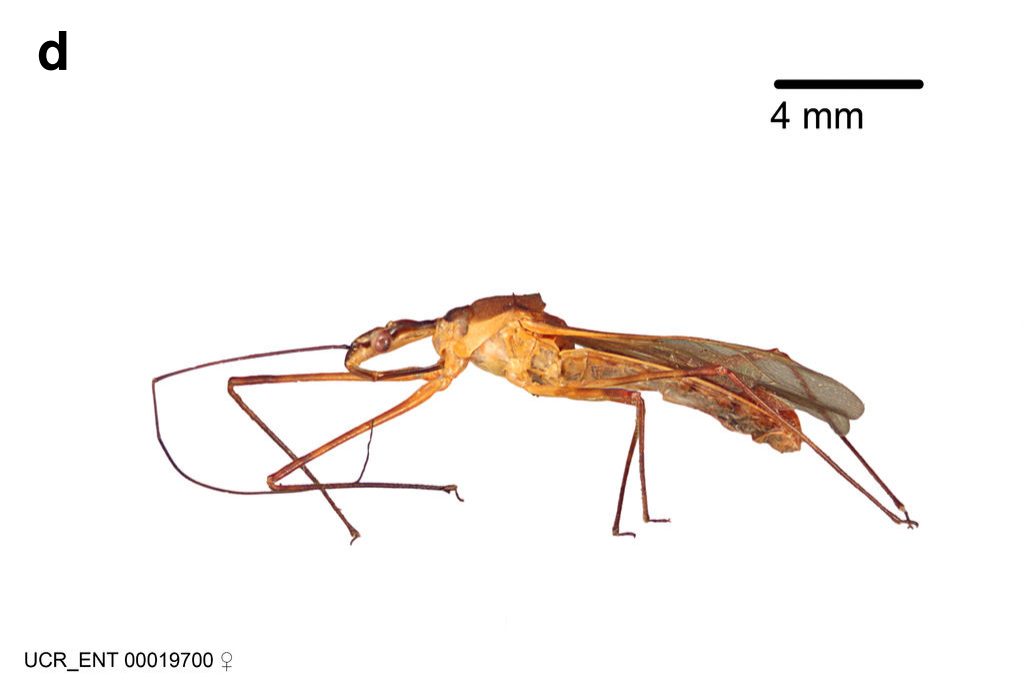
*Zelus gilboventris* Zhang & Hart, sp. n., female, lateral view (UCR_ENT 00019700, Huanuco, Peru)

**Figure 83a.**
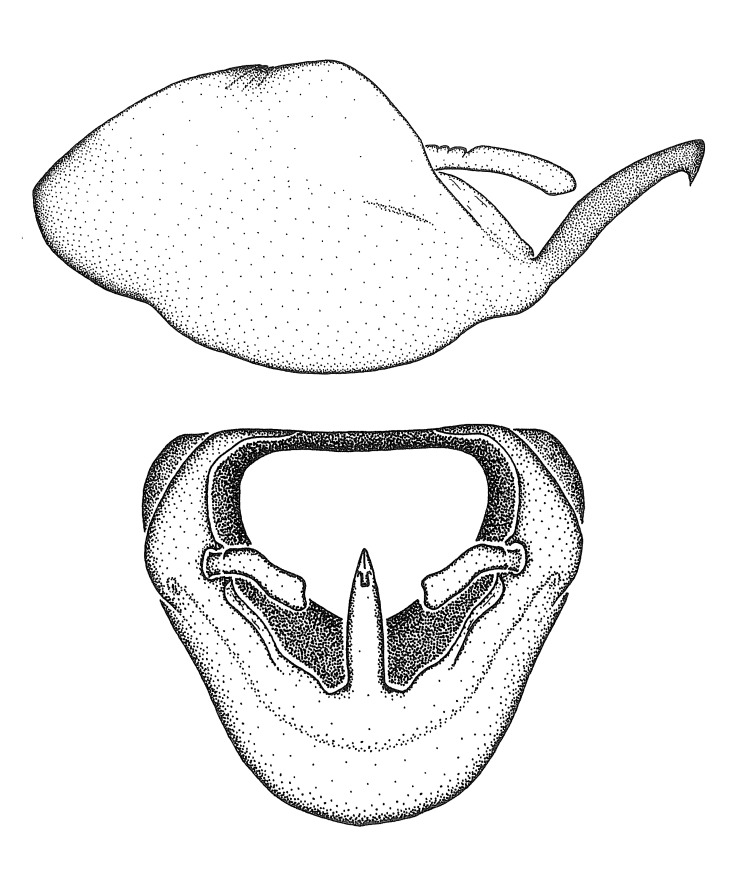
*Zelus gilboventris* Zhang & Hart, sp. n., pygophore, lateral and posterior views

**Figure 83b.**
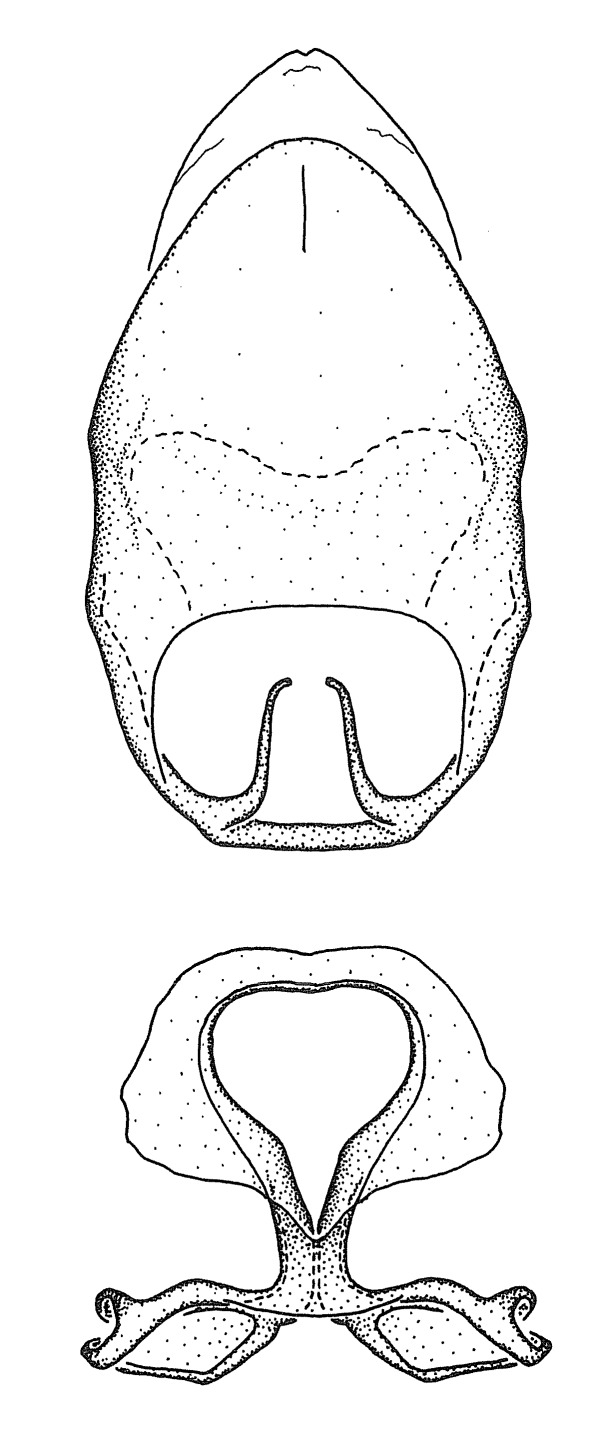
*Zelus gilboventris* Zhang & Hart, sp. n., phallus, dorsal view

**Figure 85a.**
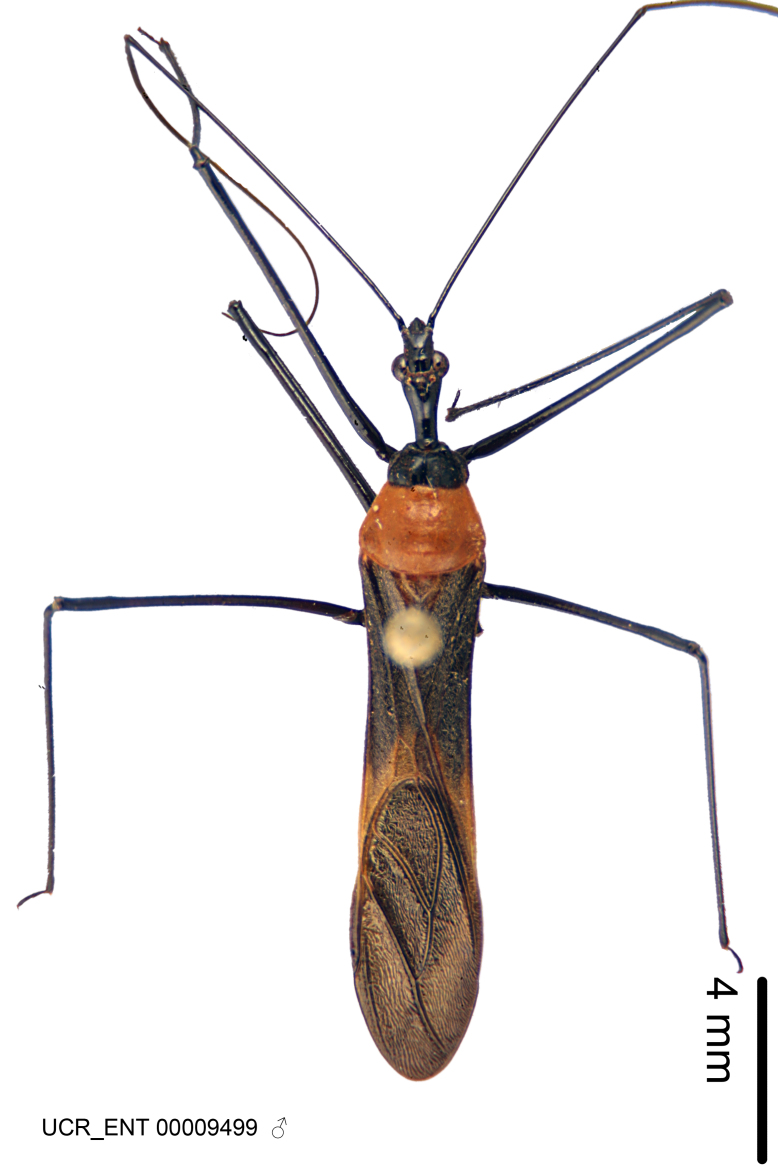
*Zelus gracilipes* Zhang & Hart, sp. n., male, dorsal view (UCR_ENT 00009499, Rondonia, Brazil)

**Figure 85b.**
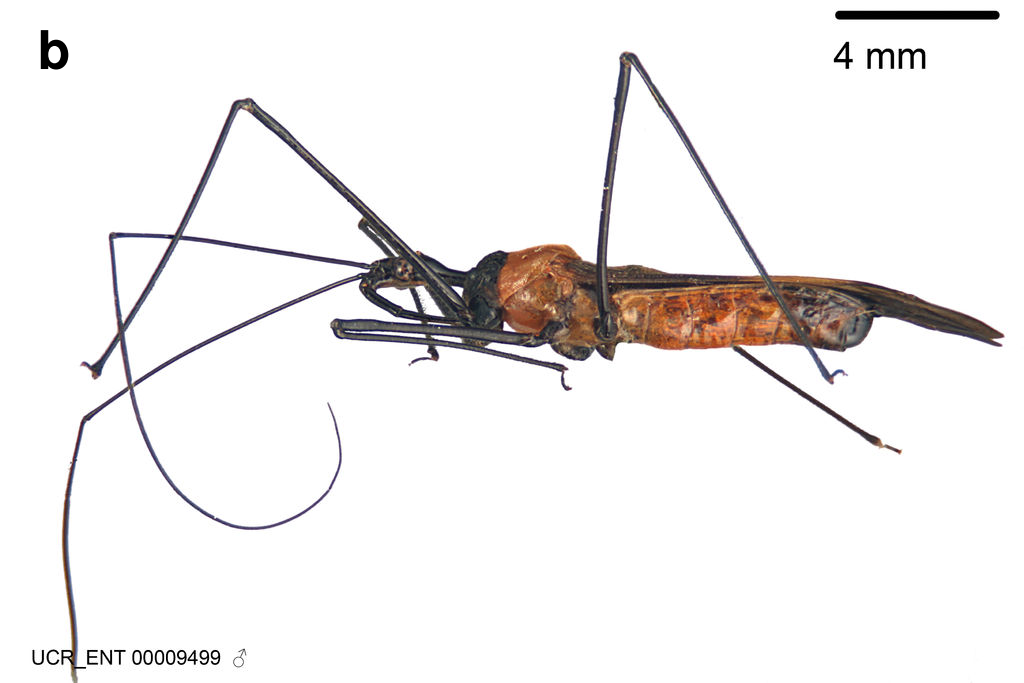
*Zelus gracilipes* Zhang & Hart, sp. n., male, lateral view (UCR_ENT 00009499, Rondonia, Brazil)

**Figure 85c.**
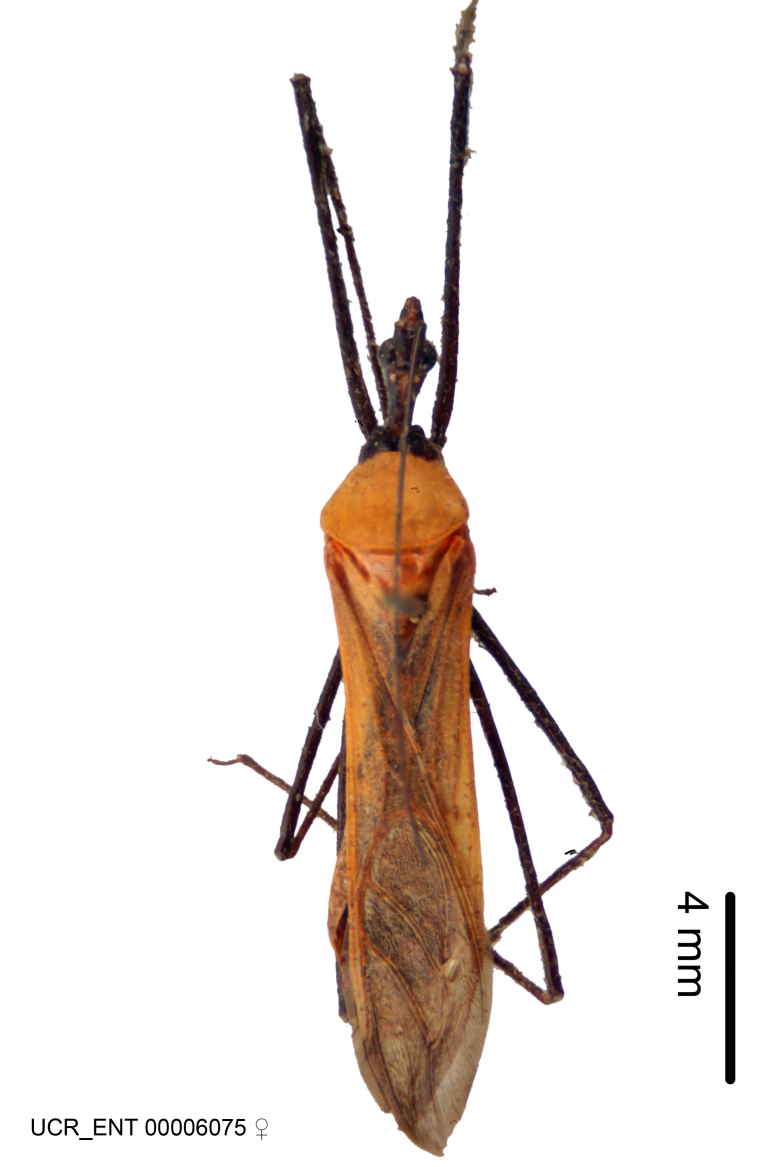
*Zelus gracilipes* Zhang & Hart, sp. n., female, dorsal view (UCR_ENT 00006075, Huanuco, Peru)

**Figure 85d.**
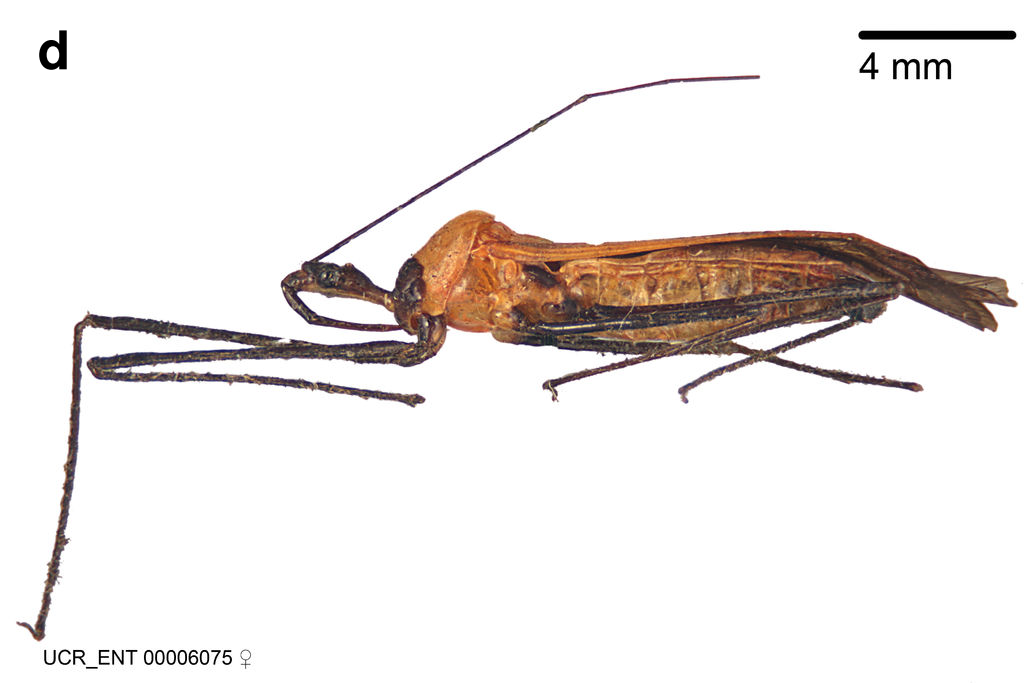
*Zelus gracilipes* Zhang & Hart, sp. n., female, lateral view (UCR_ENT 00006075, Huanuco, Peru)

**Figure 86a.**
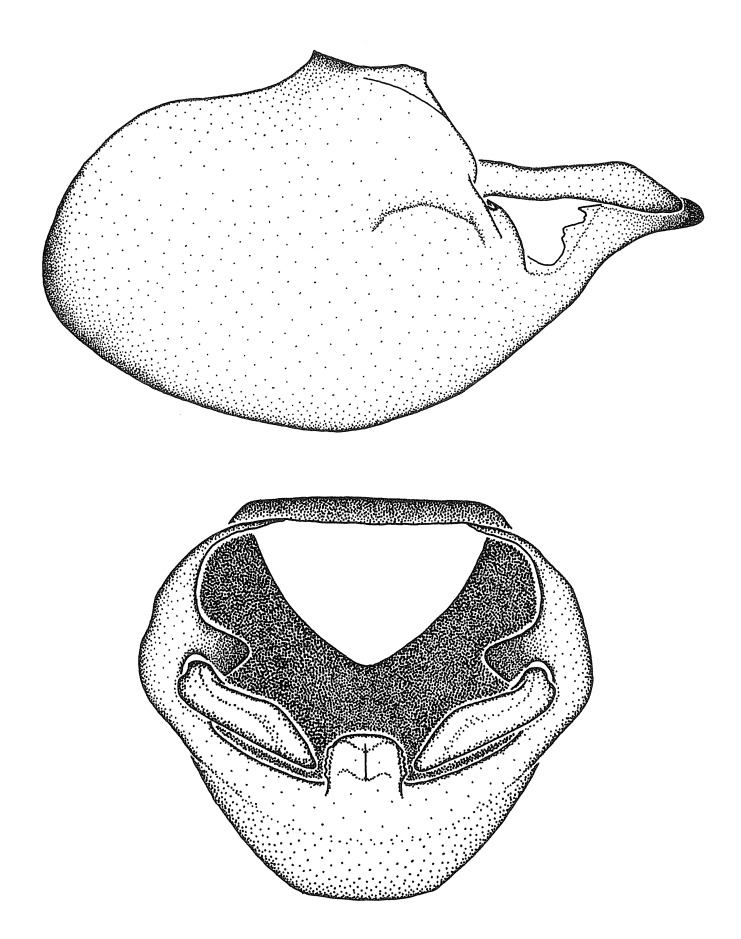
*Zelus gracilipes* Zhang & Hart, sp. n., pygophore, lateral and posterior views

**Figure 86b.**
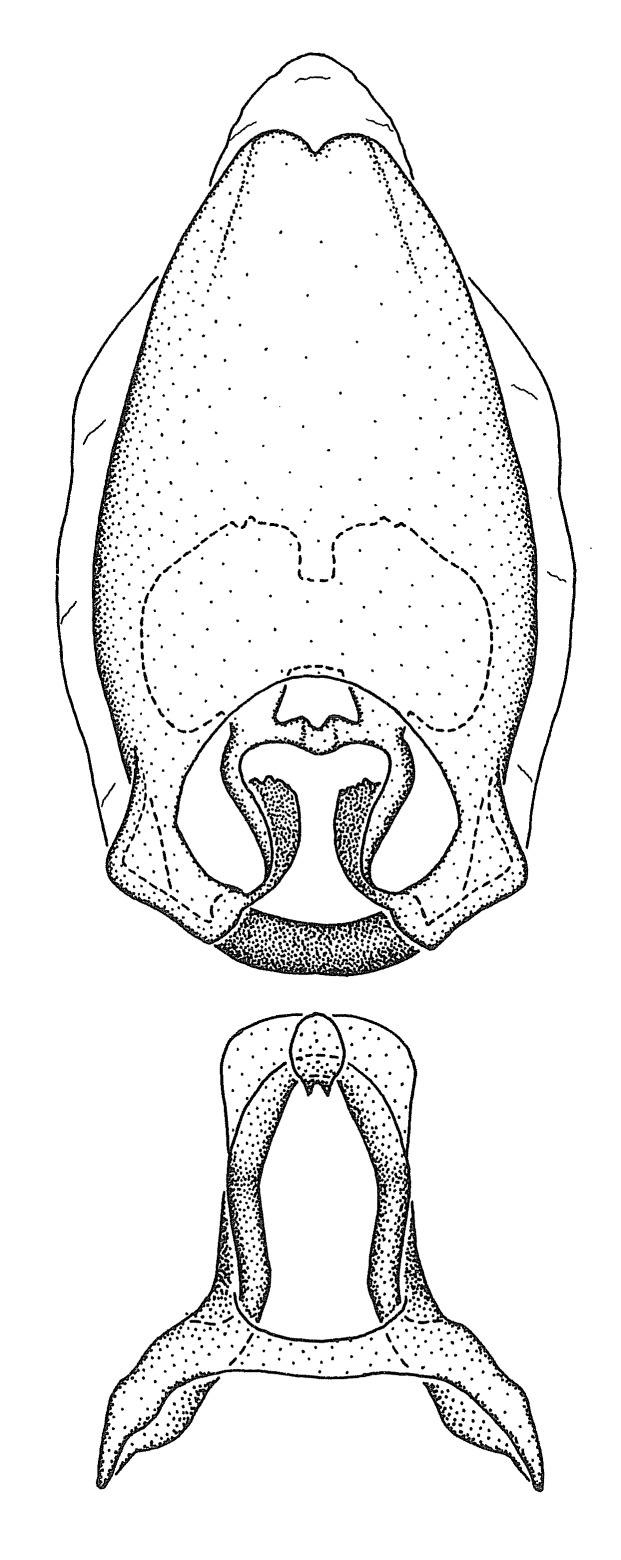
*Zelus gracilipes* Zhang & Hart, sp. n., phallus, dorsal view

**Figure 88a.**
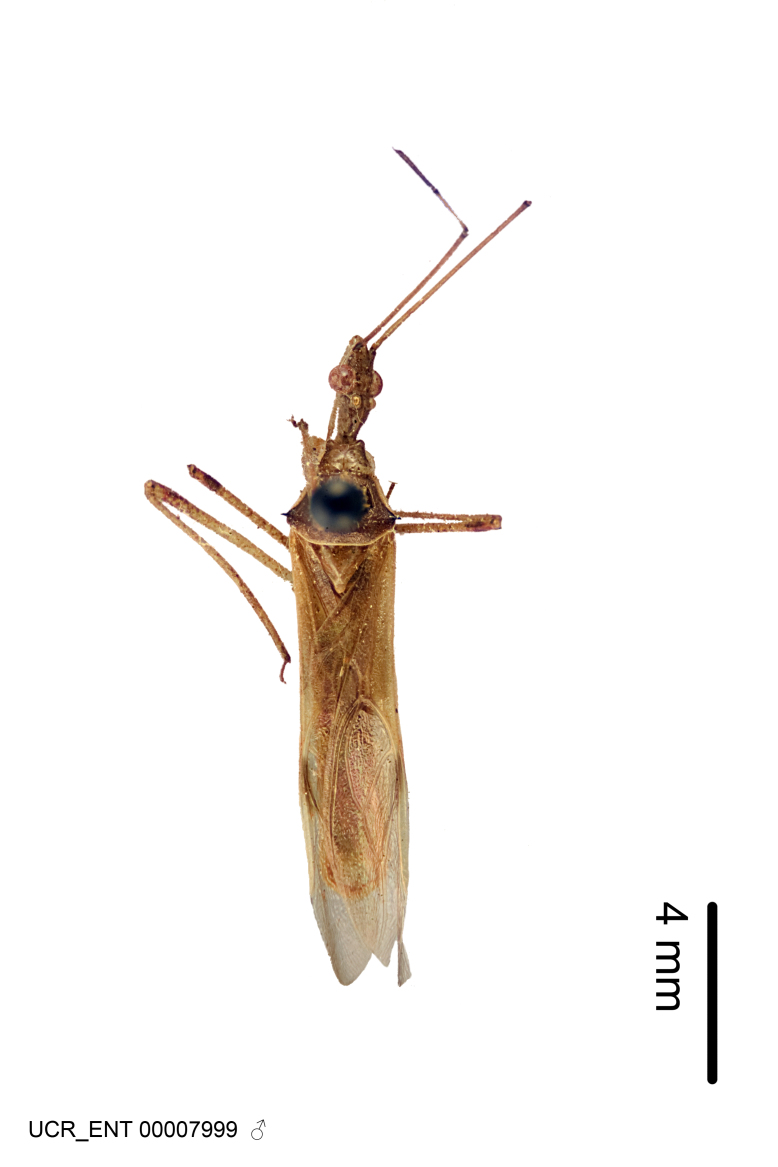
*Zelus grandoculus* Zhang & Hart, sp. n., male, dorsal view (UCR_ENT-00007999, Sacatepequez, Guatemala)

**Figure 88b.**
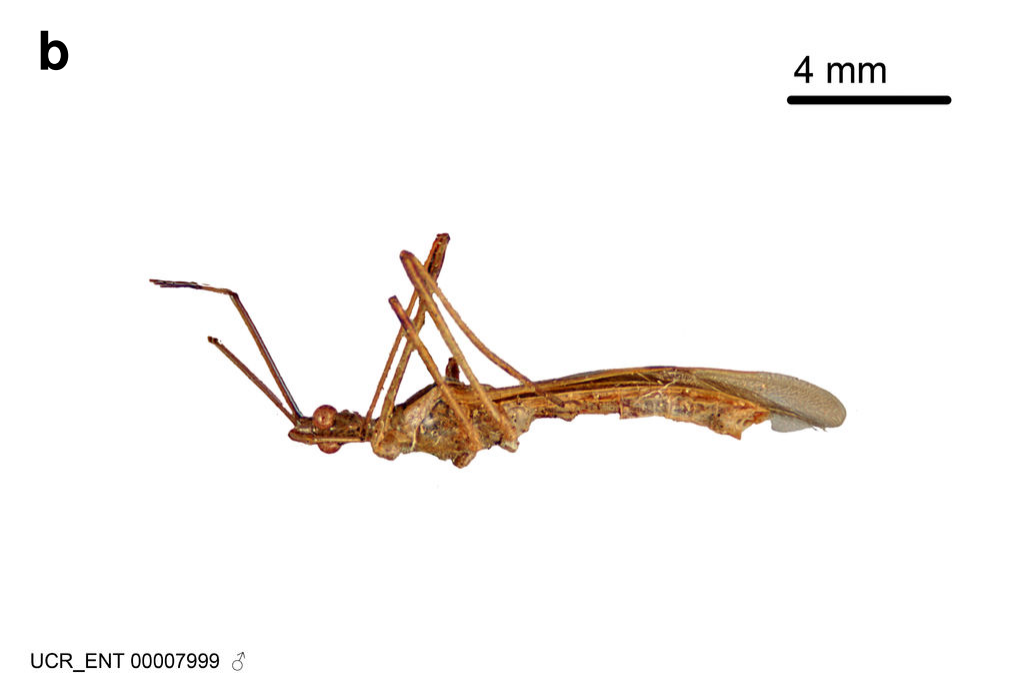
*Zelus grandoculus* Zhang & Hart, sp. n., male, lateral view (UCR_ENT-00007999, Sacatepequez, Guatemala)

**Figure 89a.**
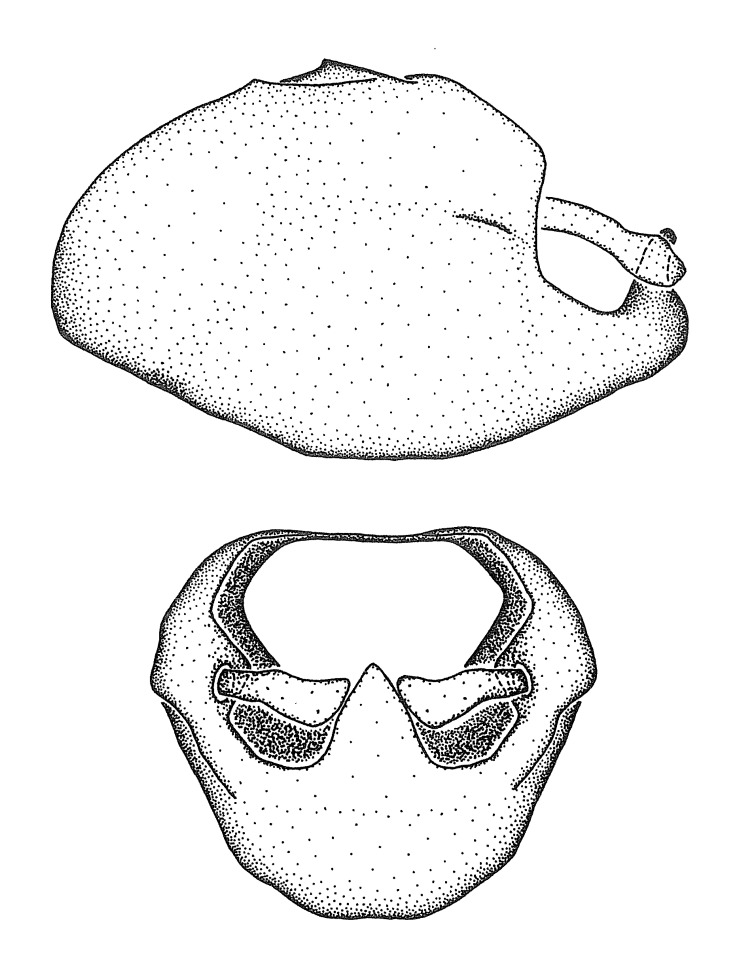
*Zelus grandoculus* Zhang & Hart, sp. n., pygophore, lateral and posterior views

**Figure 89b.**
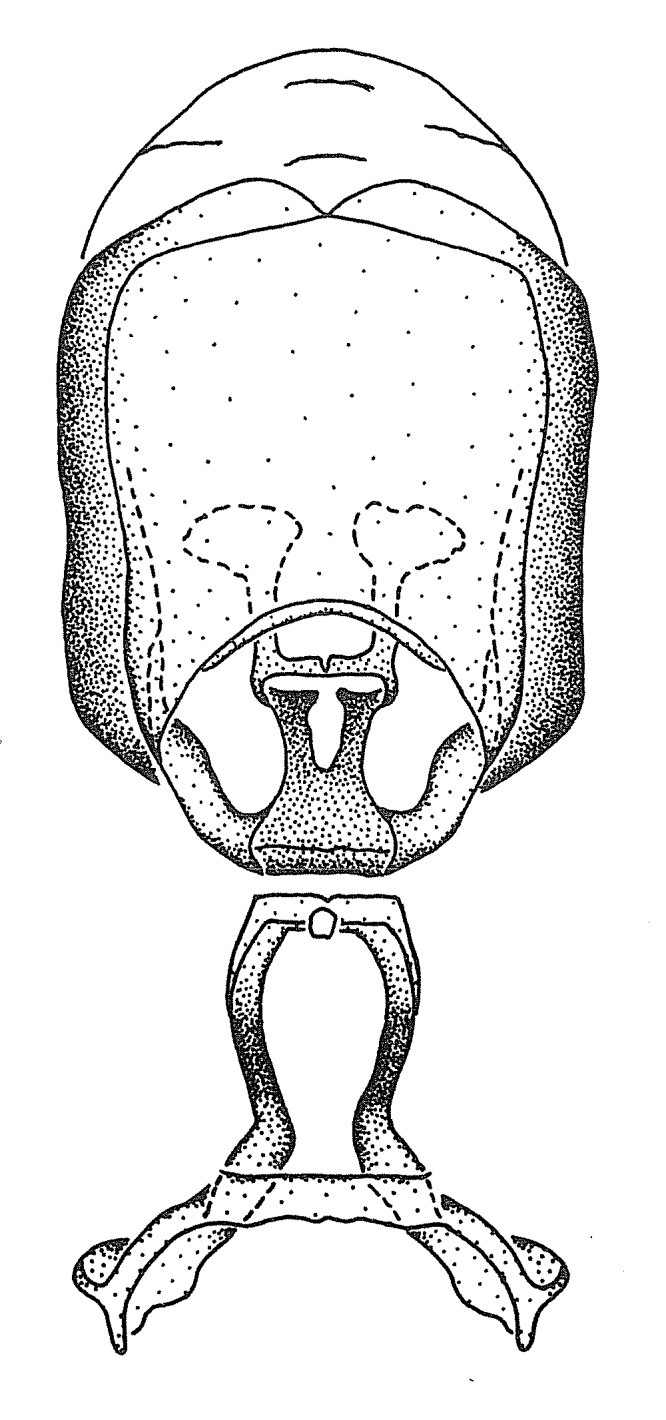
*Zelus grandoculus* Zhang & Hart, sp. n., phallus, dorsal view

**Figure 91a.**
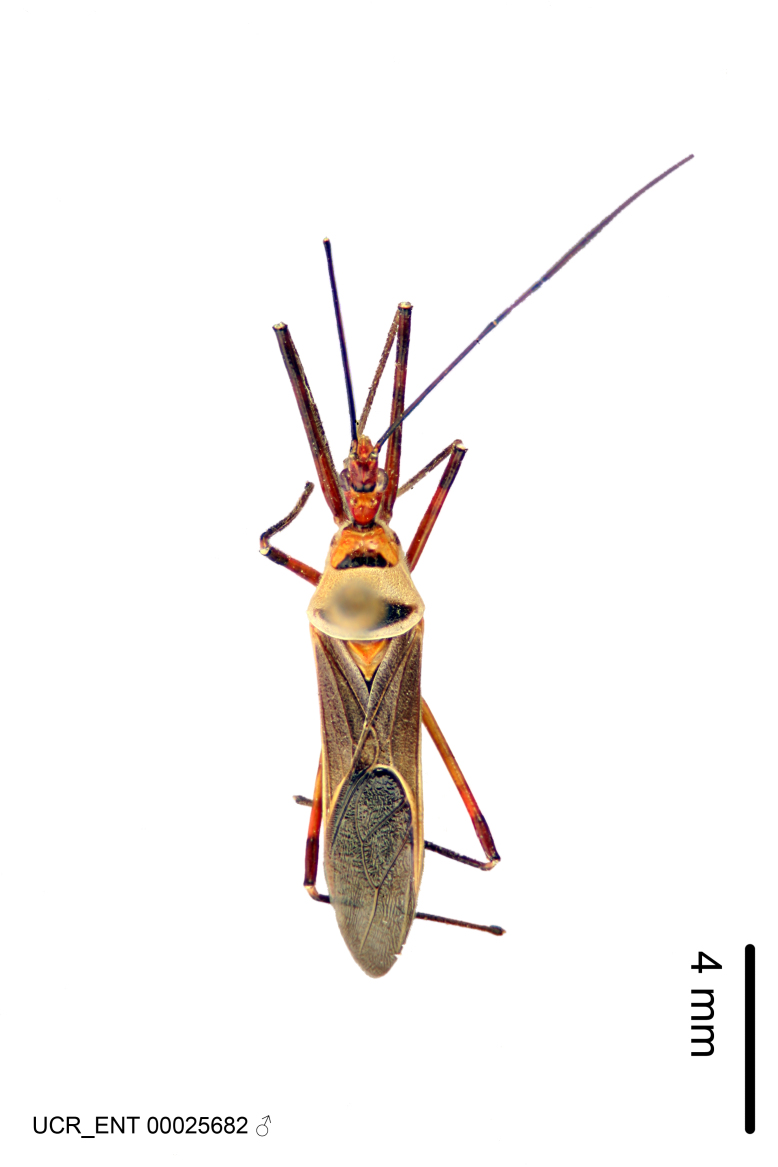
*Zelus grassans* Stål, 1862, male, dorsal view (UCR_ENT 00025682, Morelos, Mexico)

**Figure 91b.**
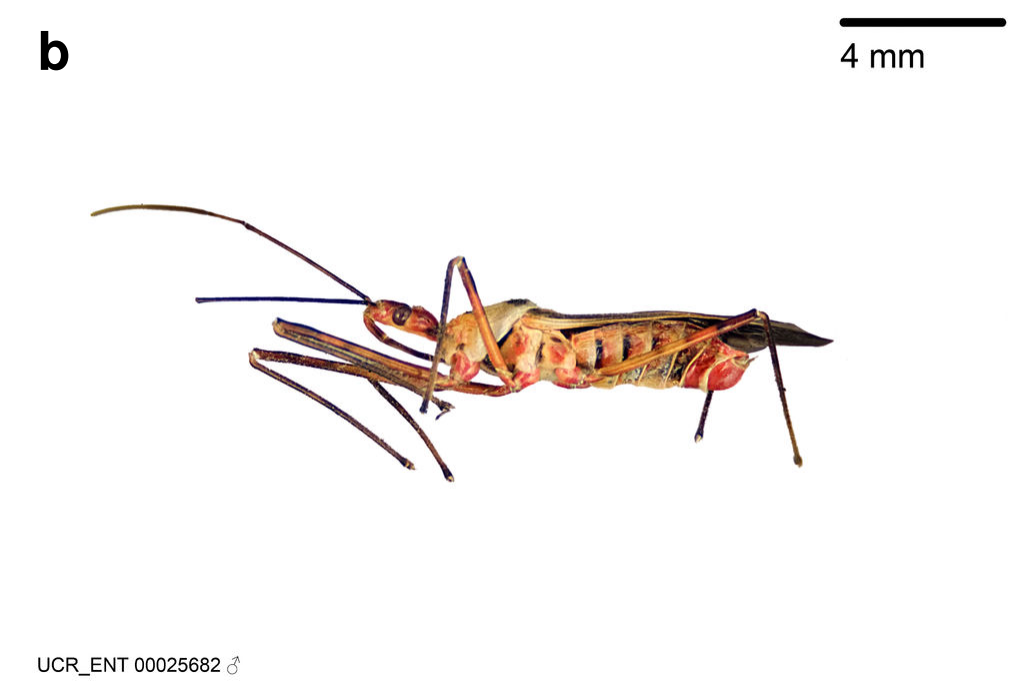
*Zelus grassans* Stål, 1862, male, lateral view (UCR_ENT 00025682, Morelos, Mexico)

**Figure 91c.**
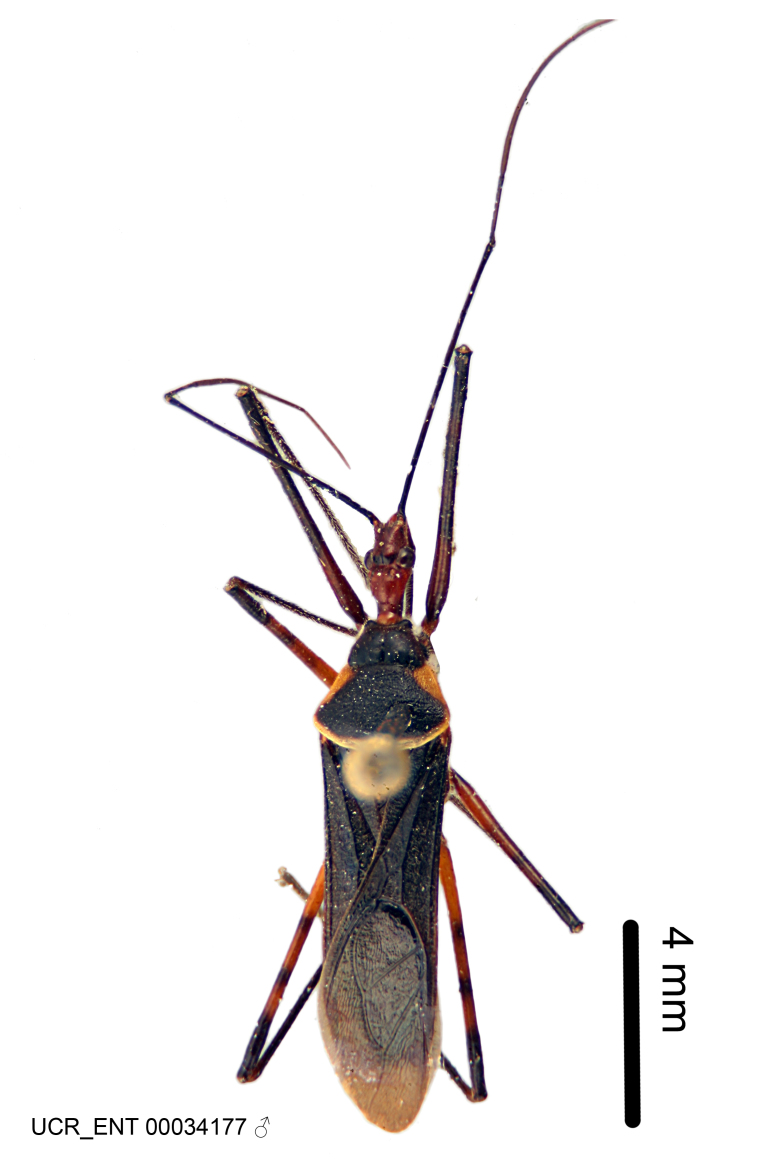
*Zelus grassans* Stål, 1862, male, dorsal view (UCR_ENT 00034177, Guanacaste, Costa Rica)

**Figure 91d.**
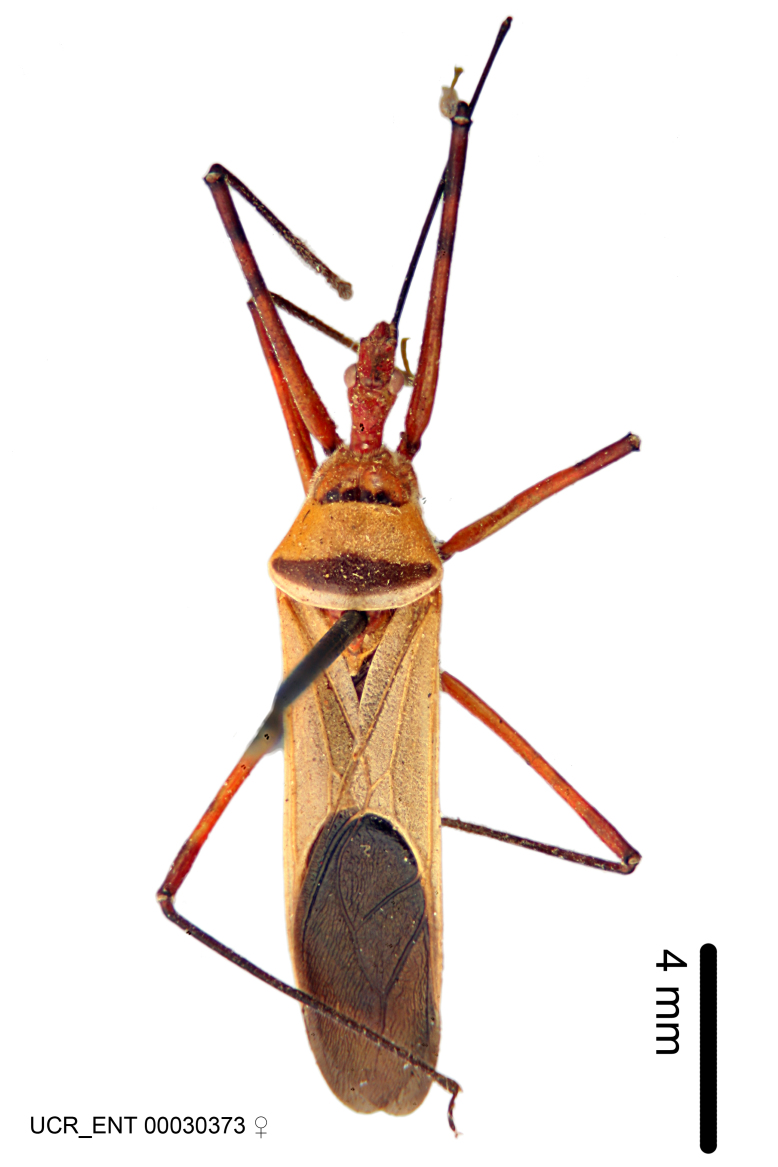
*Zelus grassans* Stål, 1862, female, dorsal view (UCR_ENT 00030373, Tamaulipas, Mexico)

**Figure 92a.**
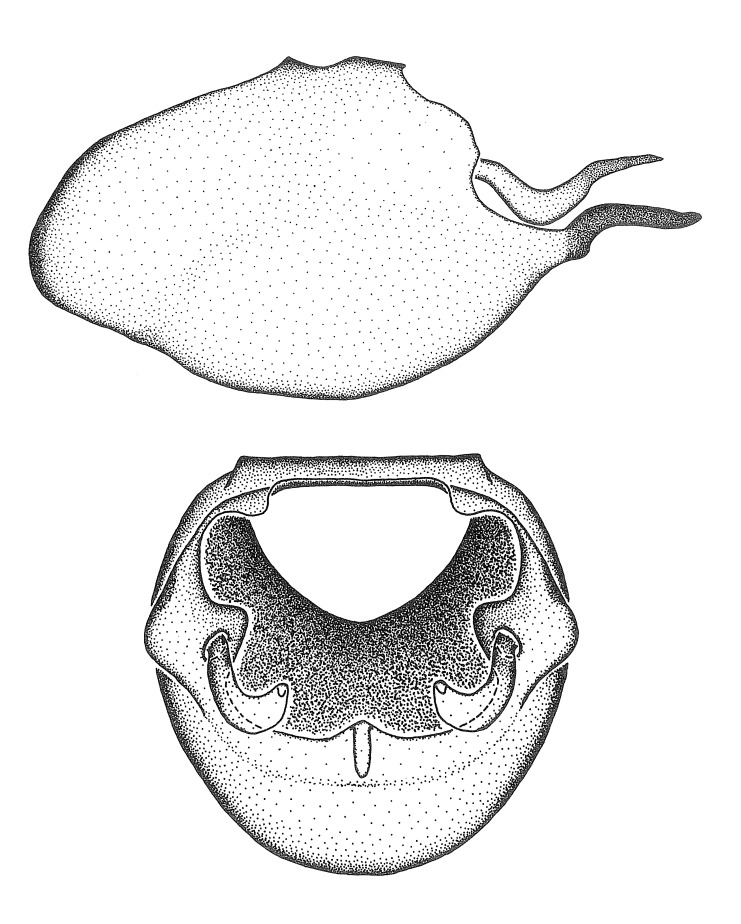
*Zelus grassans* Stål, 1862, pygophore, lateral and posterior views

**Figure 92b.**
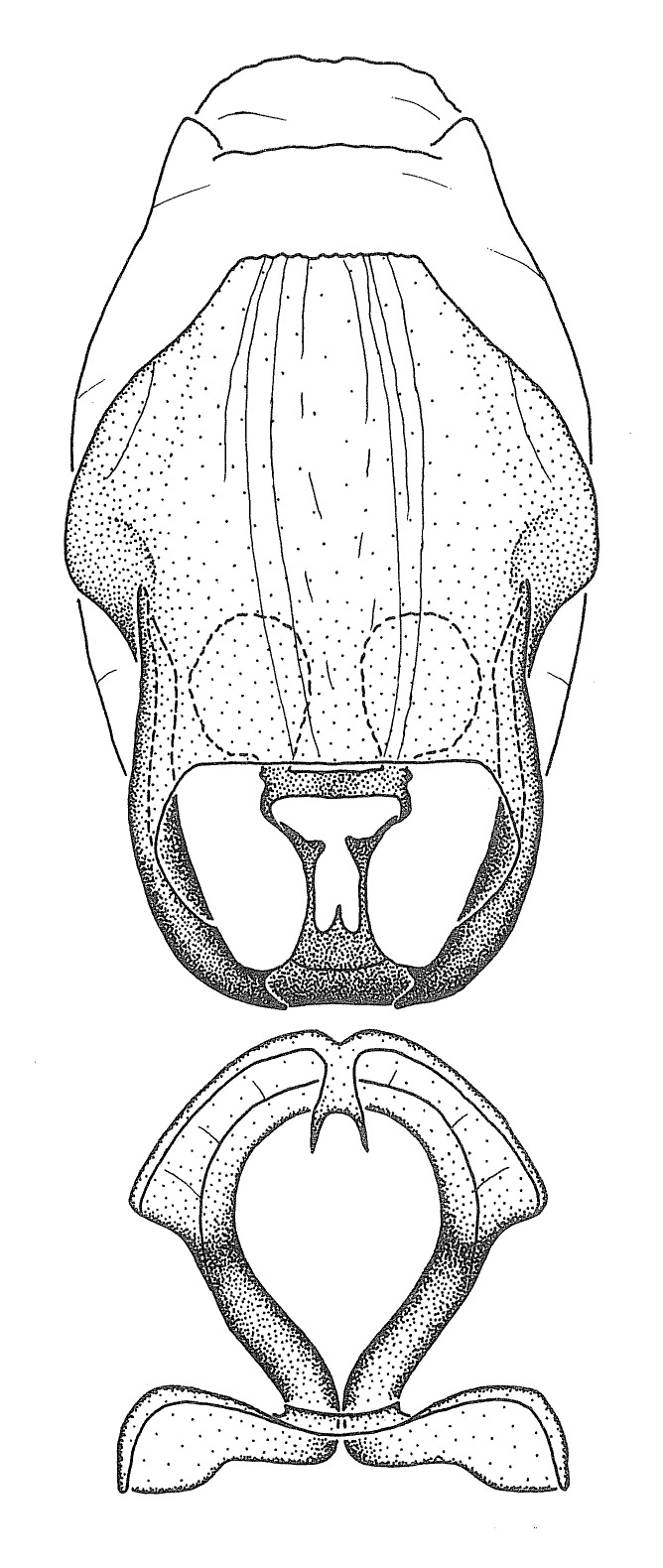
*Zelus grassans* Stål, 1862, phallus, dorsal view

**Figure 94a.**
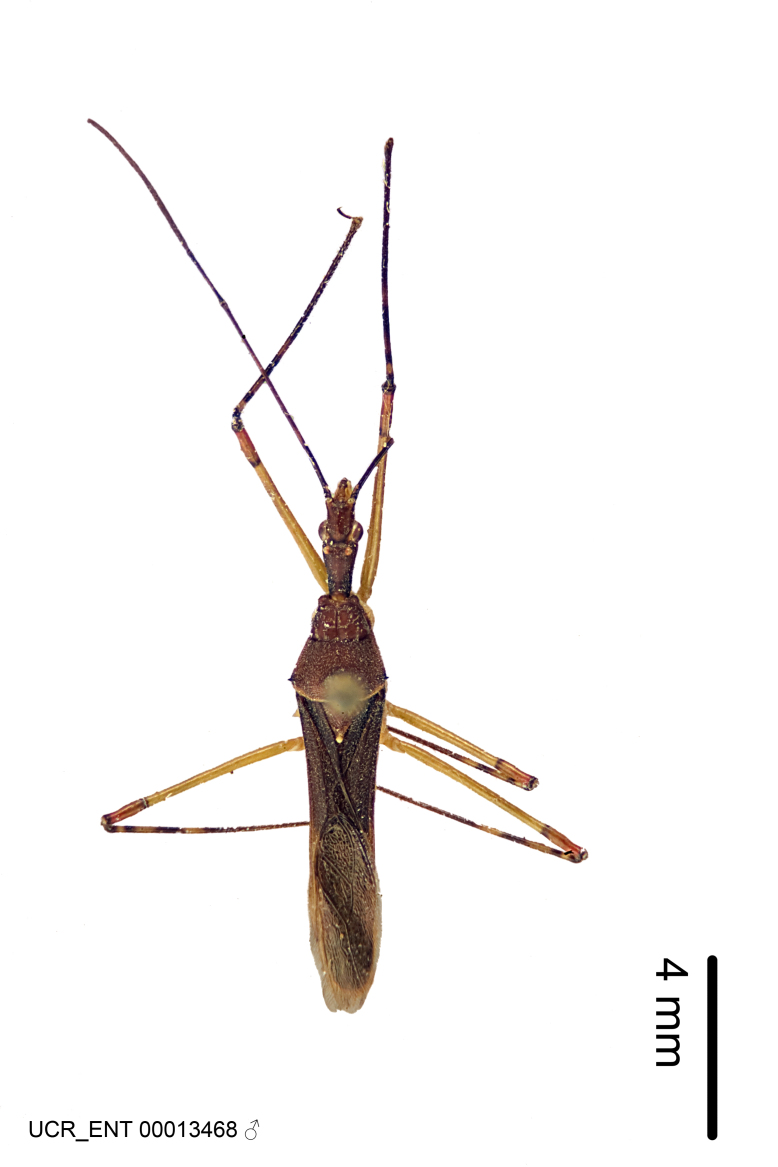
*Zelus illotus* Berg, 1879, male, dorsal view (UCR_ENT 00013468, Suriname)

**Figure 94b.**
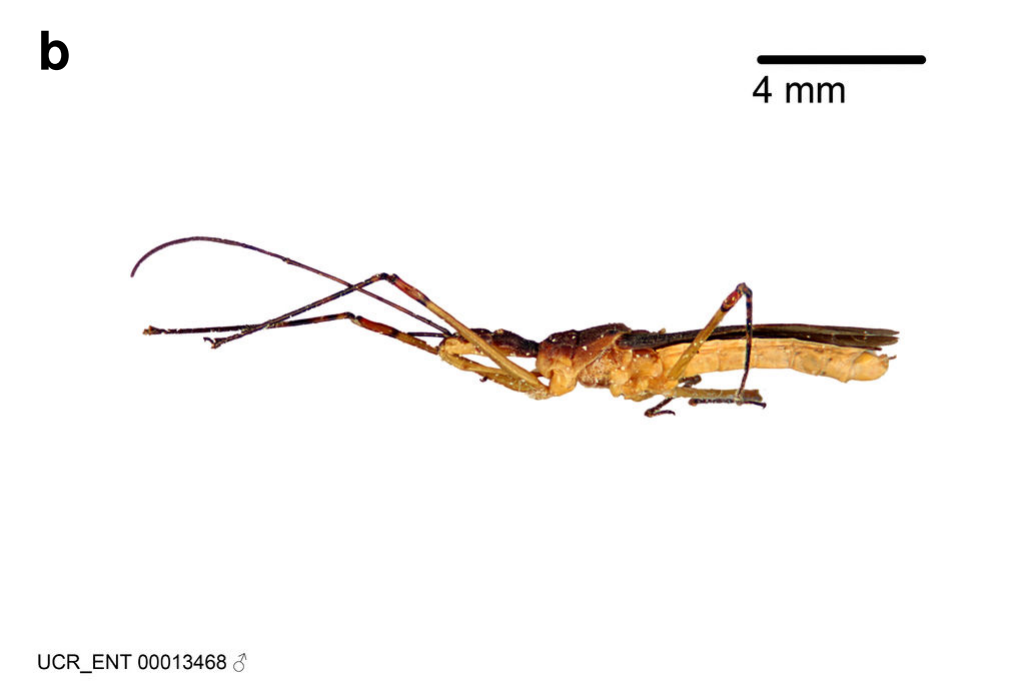
*Zelus illotus* Berg, 1879, male, lateral view (UCR_ENT 00013468, Suriname)

**Figure 94c.**
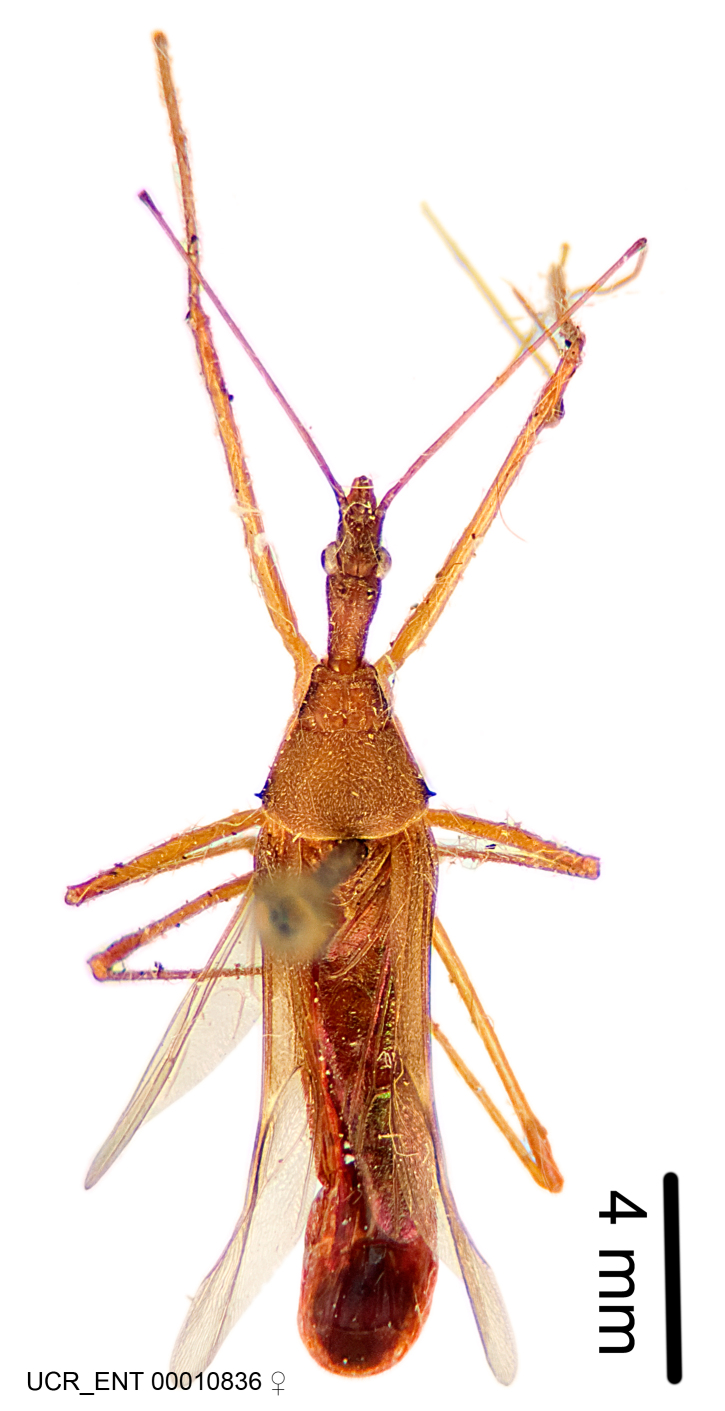
*Zelus illotus* Berg, 1879, female, dorsal view (UCR_ENT 00010836, Goias, Brazil)

**Figure 95a.**
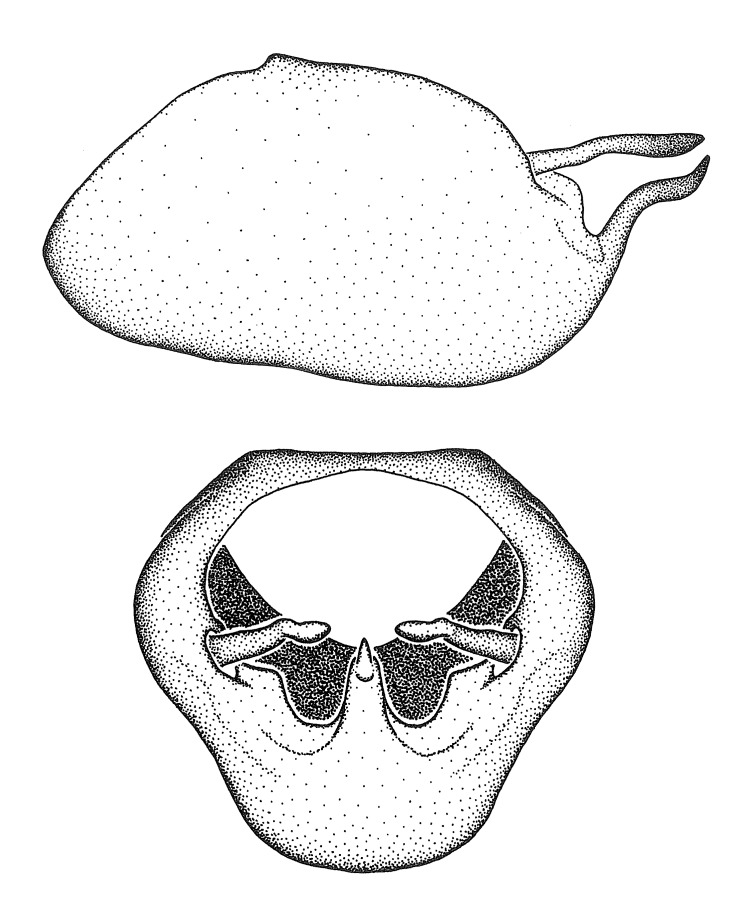
*Zelus illotus* Berg, 1879, pygophore, lateral and posterior views

**Figure 95b.**
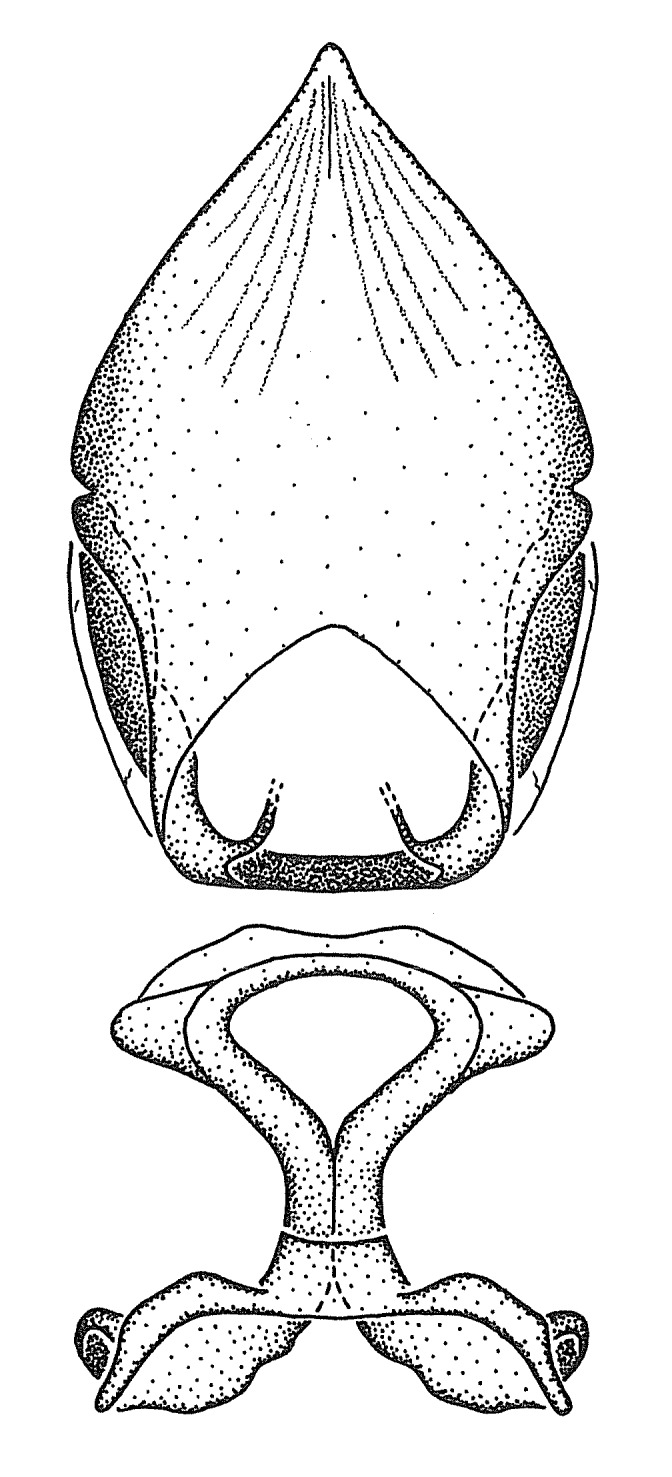
*Zelus illotus* Berg, 1879, phallus, dorsal view

**Figure 97a.**
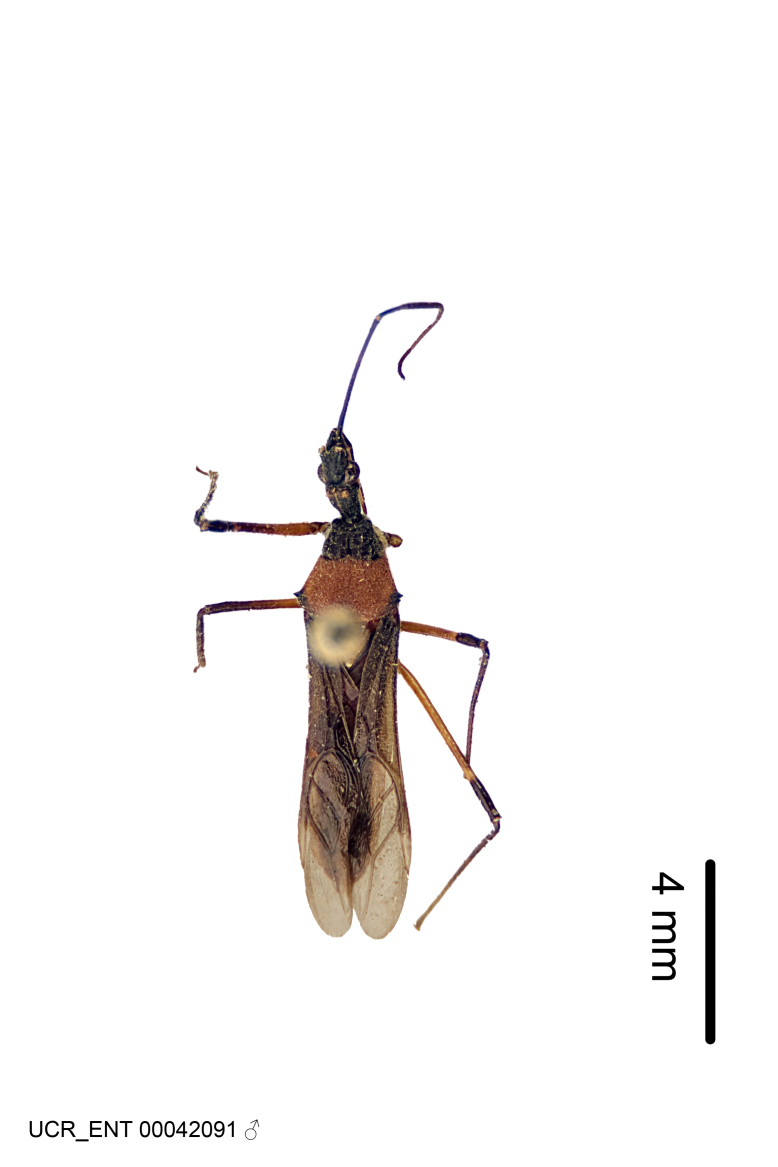
*Zelus impar* Kuhlgatz & Melichar, 1902, male, dorsal view (UCR_ENT 00042091, Colombia)

**Figure 97b.**
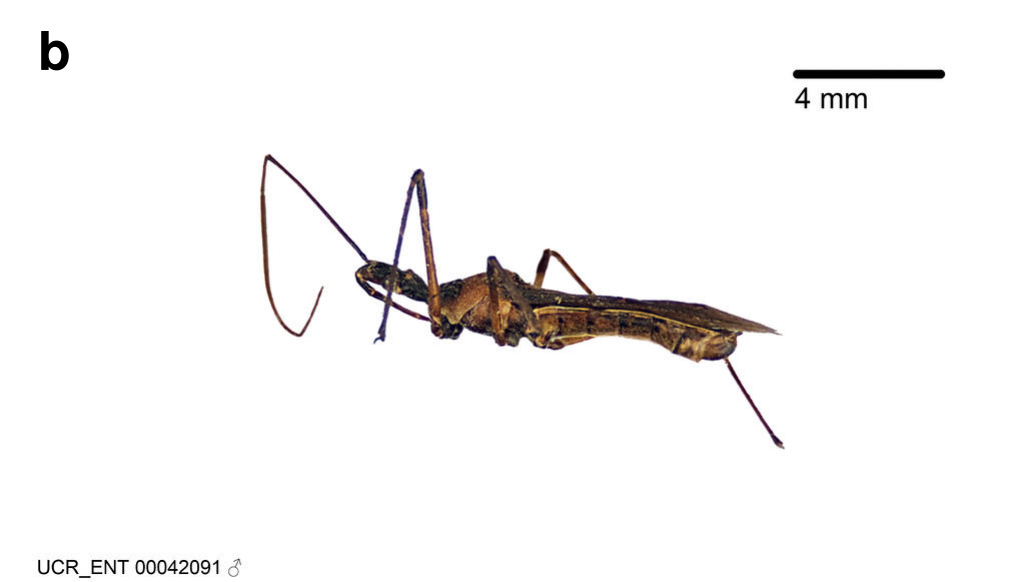
*Zelus impar* Kuhlgatz & Melichar, 1902, male, lateral view (UCR_ENT 00042091, Colombia)

**Figure 97c.**
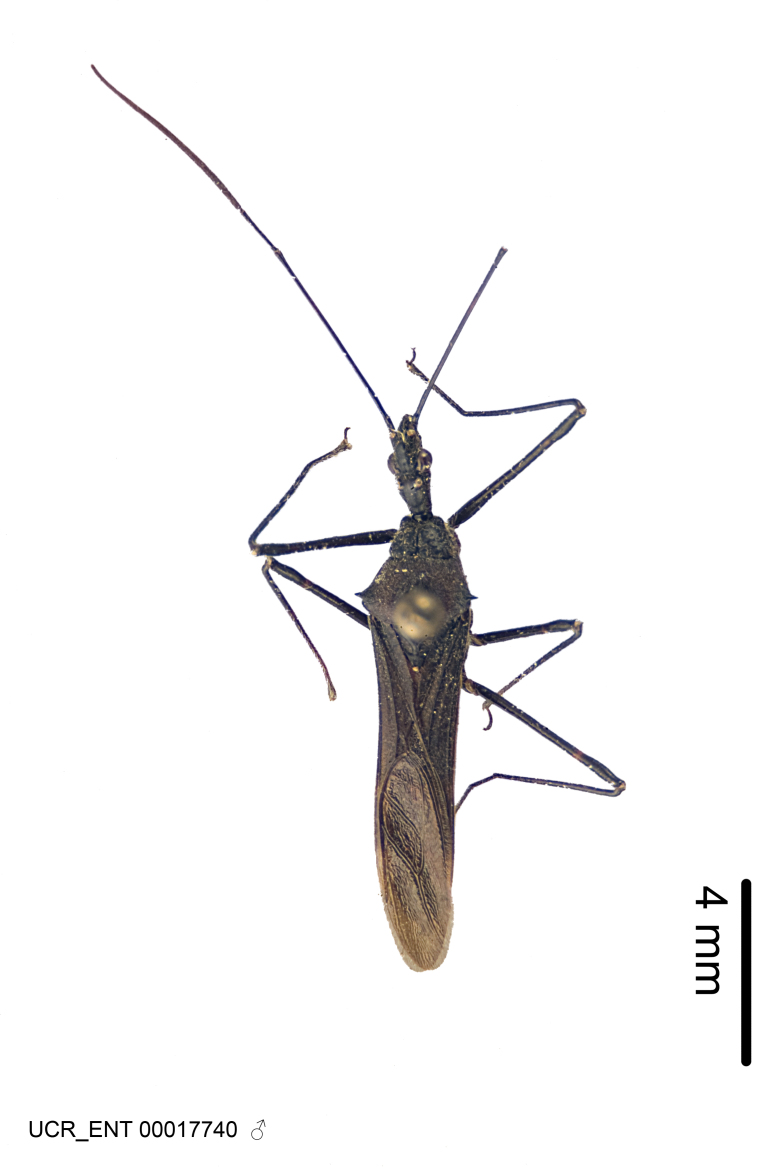
*Zelus impar* Kuhlgatz & Melichar, 1902, male, dorsal view (UCR_ENT 00017740, Magdalena, Colombia)

**Figure 97d.**
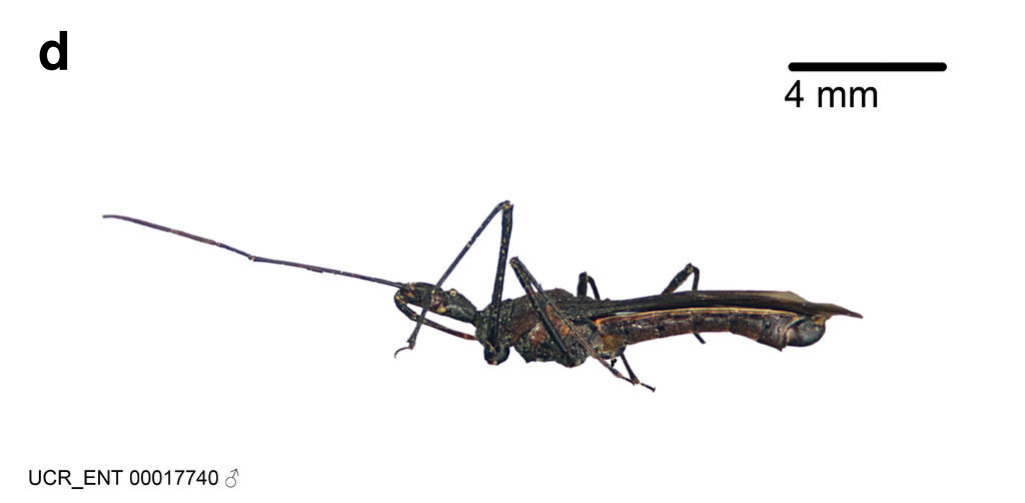
*Zelus impar* Kuhlgatz & Melichar, 1902, male, lateral view (UCR_ENT 00017740, Magdalena, Colombia)

**Figure 98a.**
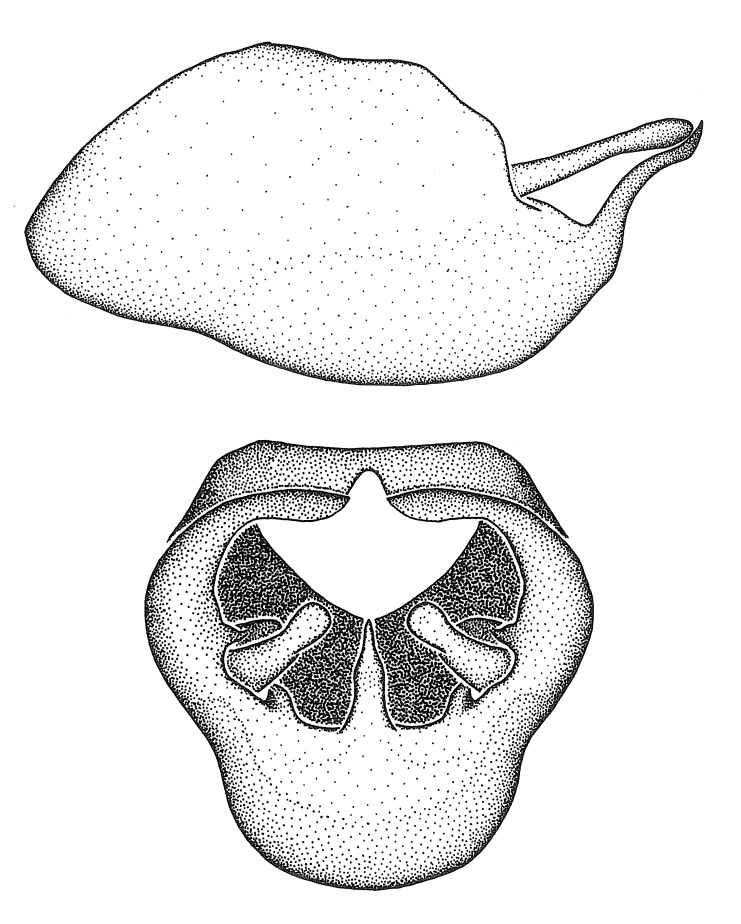
*Zelus impar* Kuhlgatz & Melichar, 1902, pygophore, lateral and posterior views

**Figure 98b.**
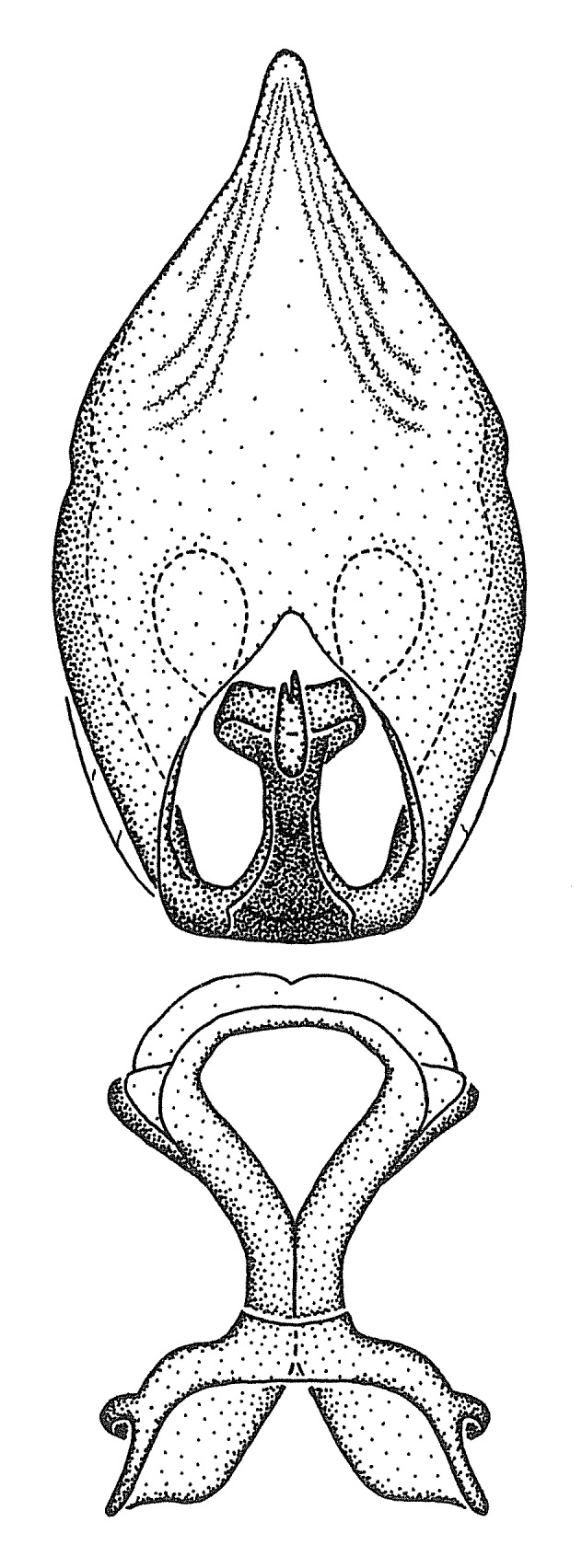
*Zelus impar* Kuhlgatz & Melichar, 1902, phallus, dorsal view

**Figure 100a.**
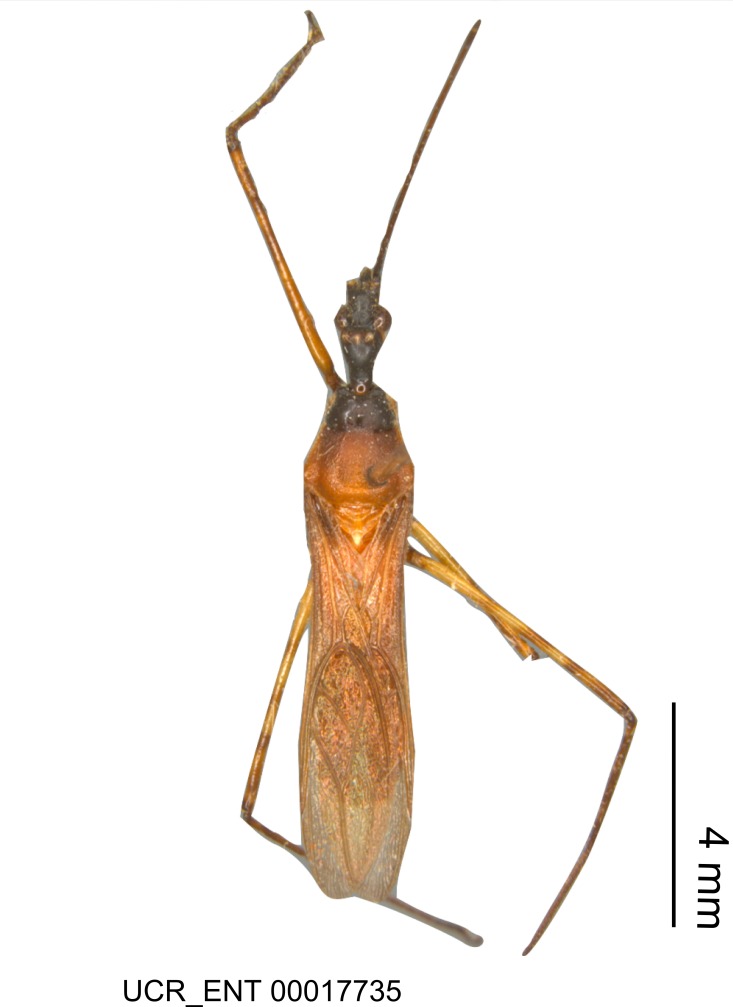
*Zelus inconstans* Champion, 1899, male, dorsal view (UCR_ENT 00017735, Cundinamarca, Colombia)

**Figure 100b.**
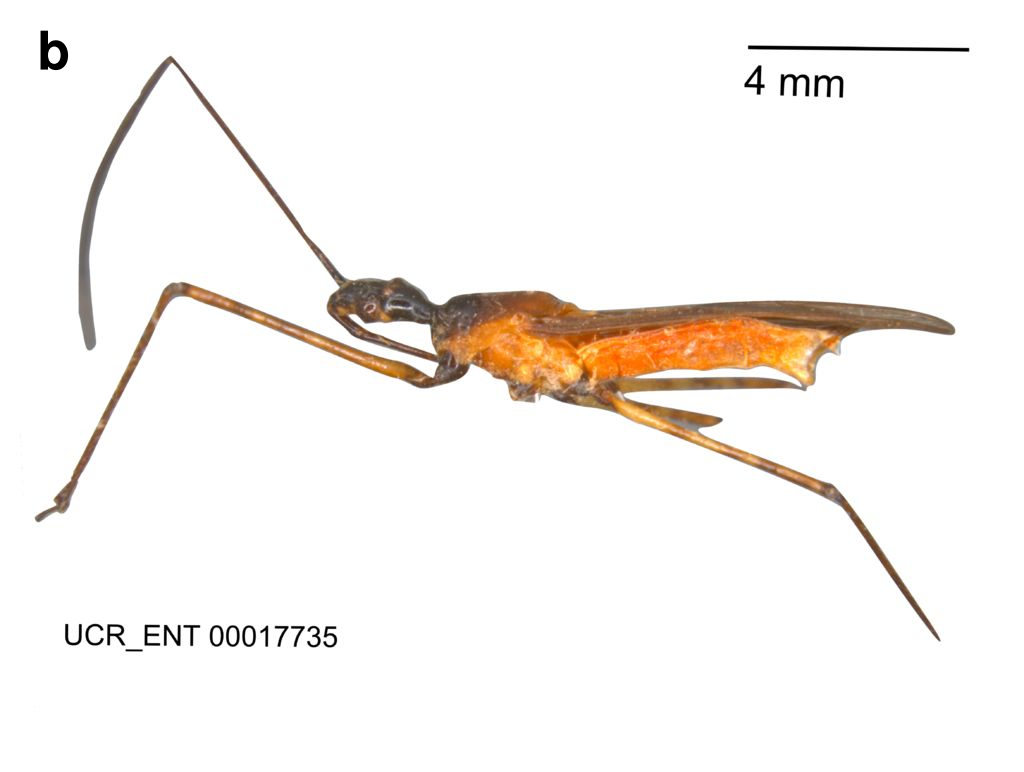
*Zelus inconstans* Champion, 1899, male, lateral view (UCR_ENT 00017735, Cundinamarca, Colombia)

**Figure 100c.**
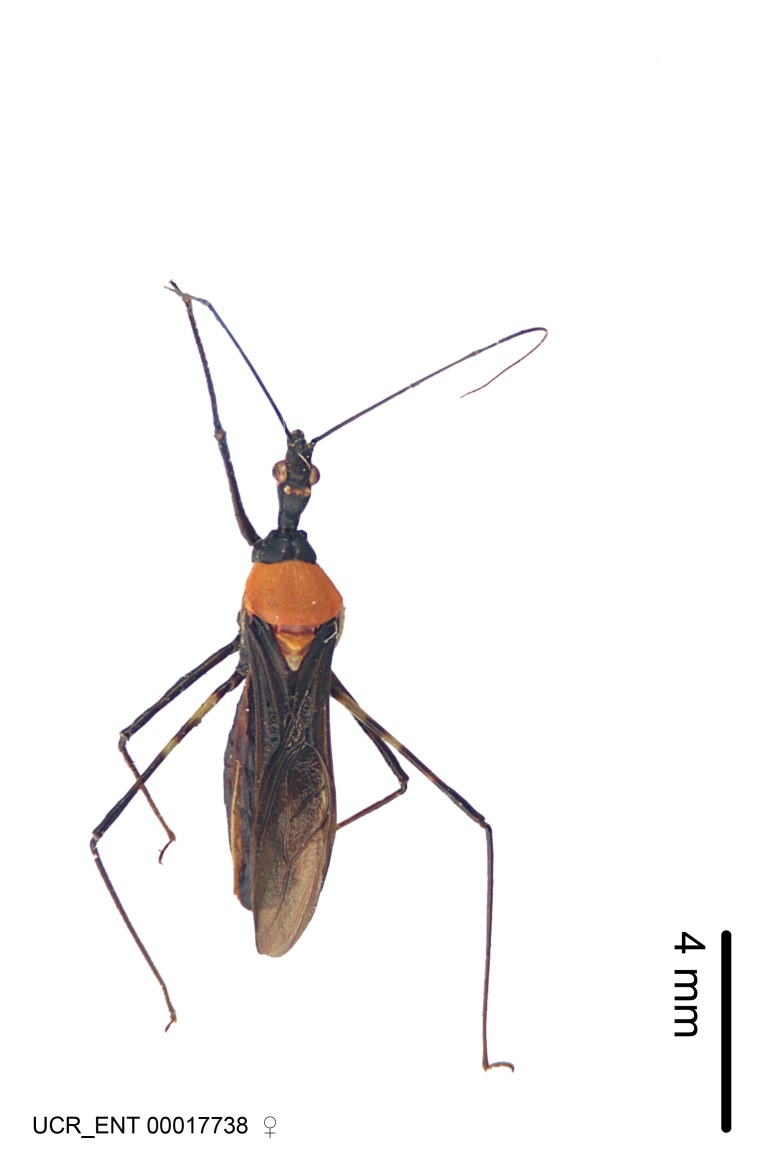
*Zelus inconstans* Champion, 1899, female, dorsal view (UCR_ENT 00017738, Canal Zone, Panama)

**Figure 100d.**
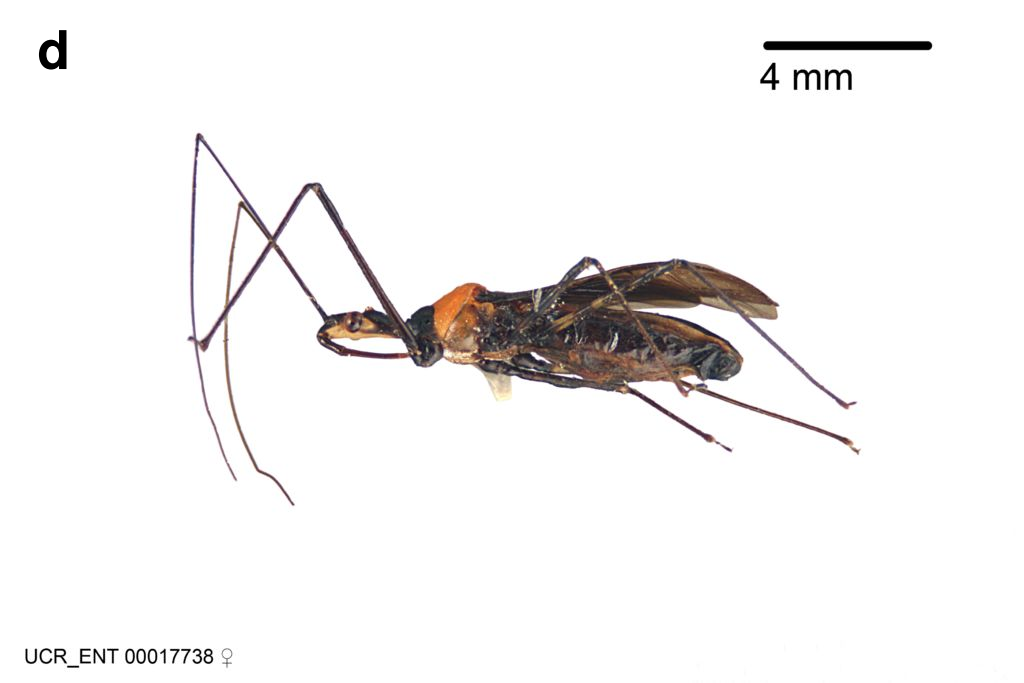
*Zelus inconstans* Champion, 1899, female, lateral view (UCR_ENT 00017738, Canal Zone, Panama)

**Figure 101a.**
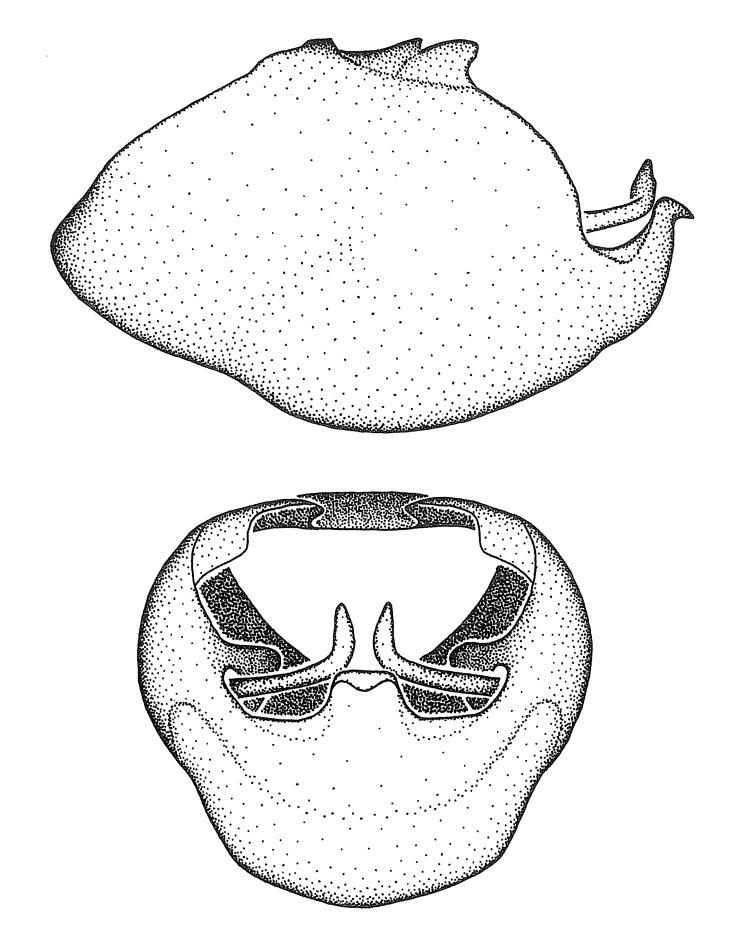
*Zelus inconstans* Champion, 1899, pygophore, lateral and posterior views

**Figure 101b.**
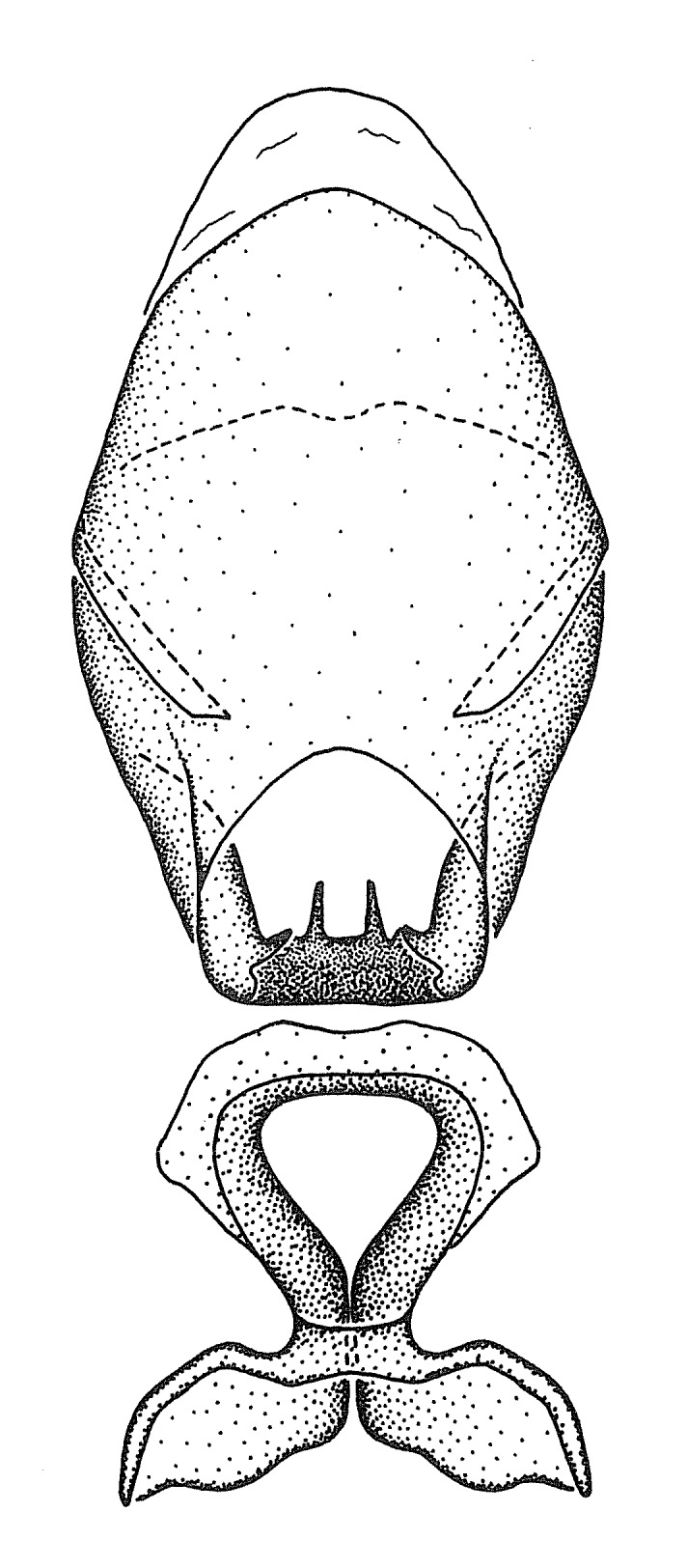
*Zelus inconstans* Champion, 1899, phallus, dorsal view

**Figure 103a.**
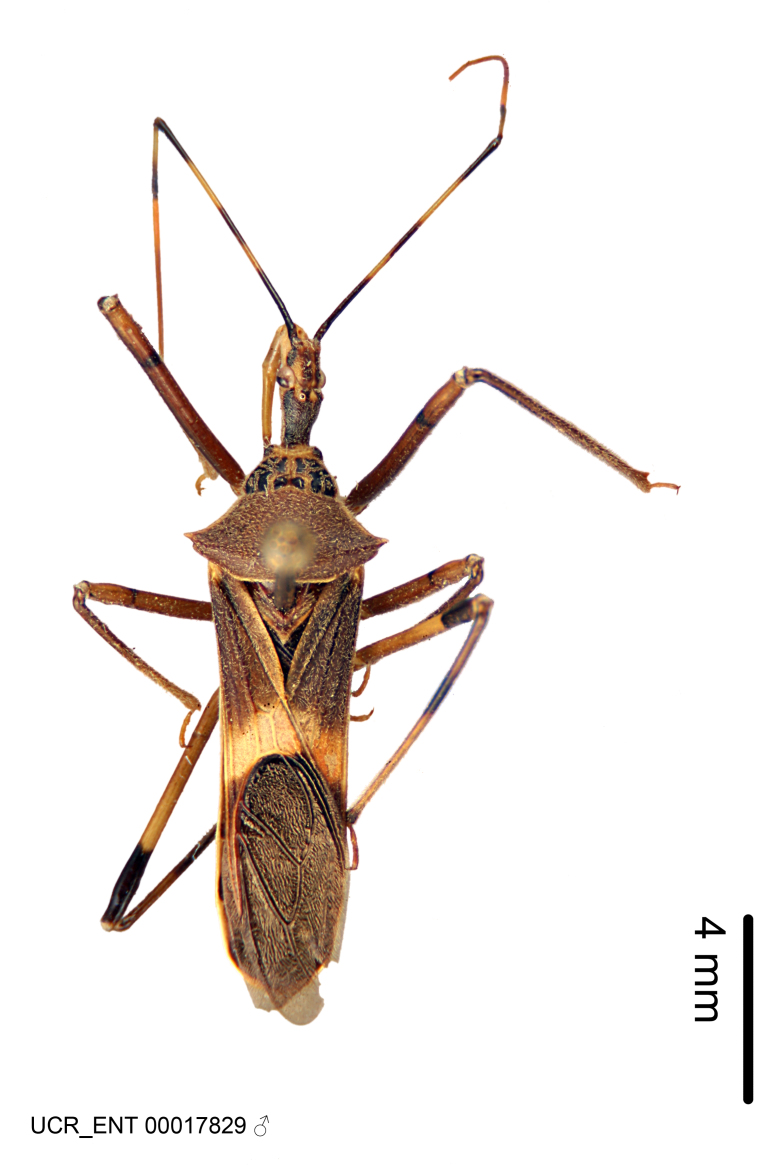
*Zelus janus* Stål, 1862, male, dorsal view (UCR_ENT 00017829, Tamaulipas, Mexico)

**Figure 103b.**
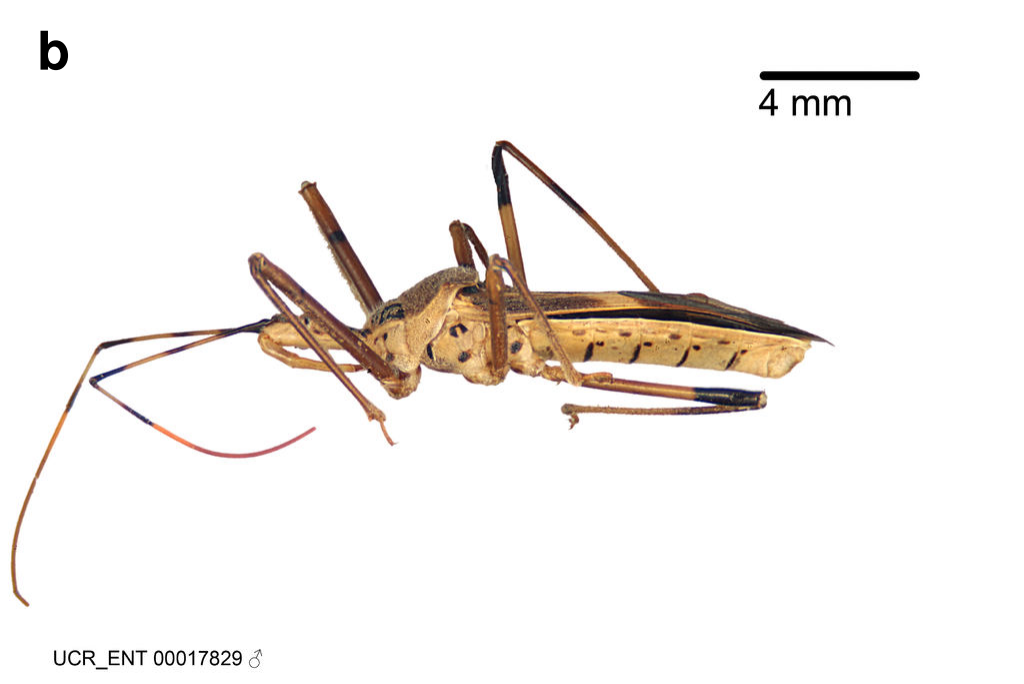
*Zelus janus* Stål, 1862, male, lateral view (UCR_ENT 00017829, Tamaulipas, Mexico)

**Figure 103c.**
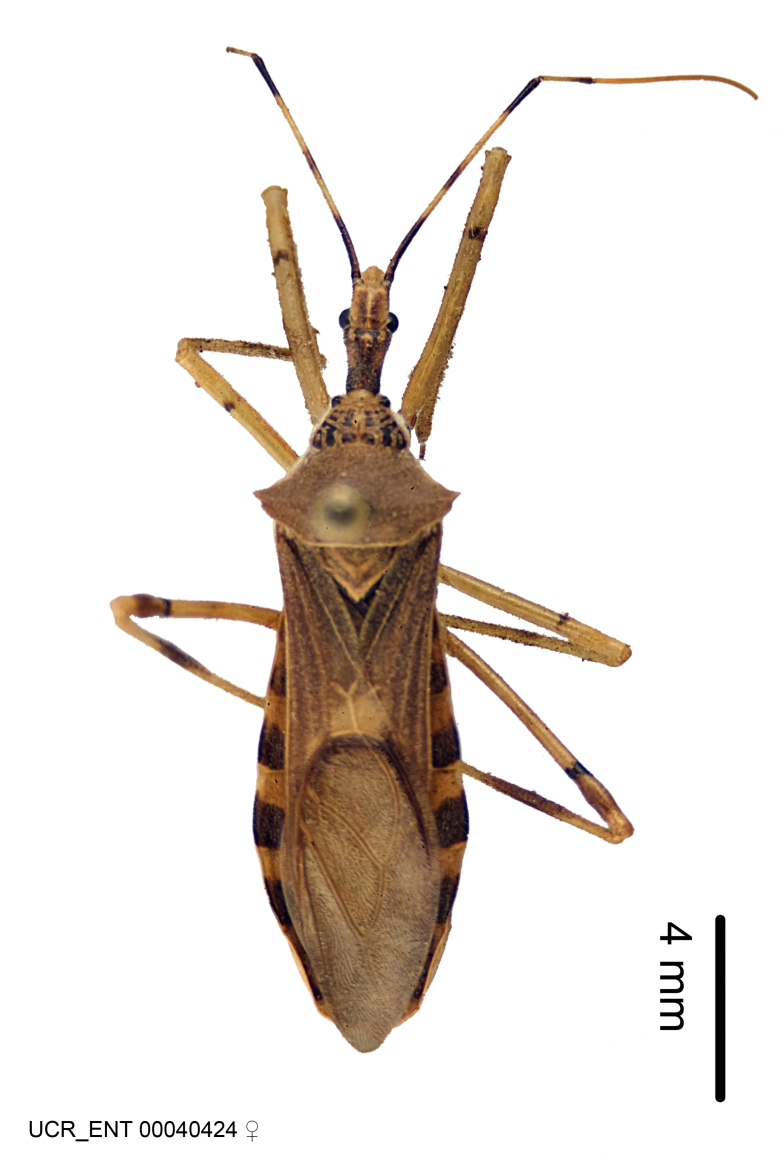
*Zelus janus* Stål, 1862, female, dorsal view (UCR_ENT 00040424, Tamaulipas, Mexico)

**Figure 103d.**
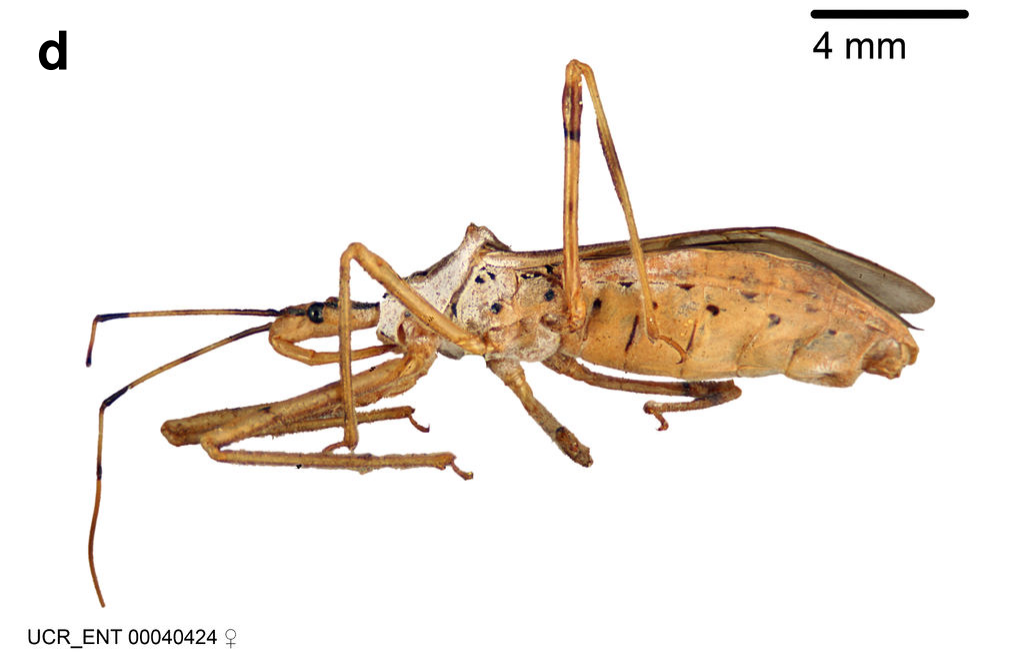
*Zelus janus* Stål, 1862, female, lateral view (UCR_ENT 00040424, Tamaulipas, Mexico)

**Figure 103e.**
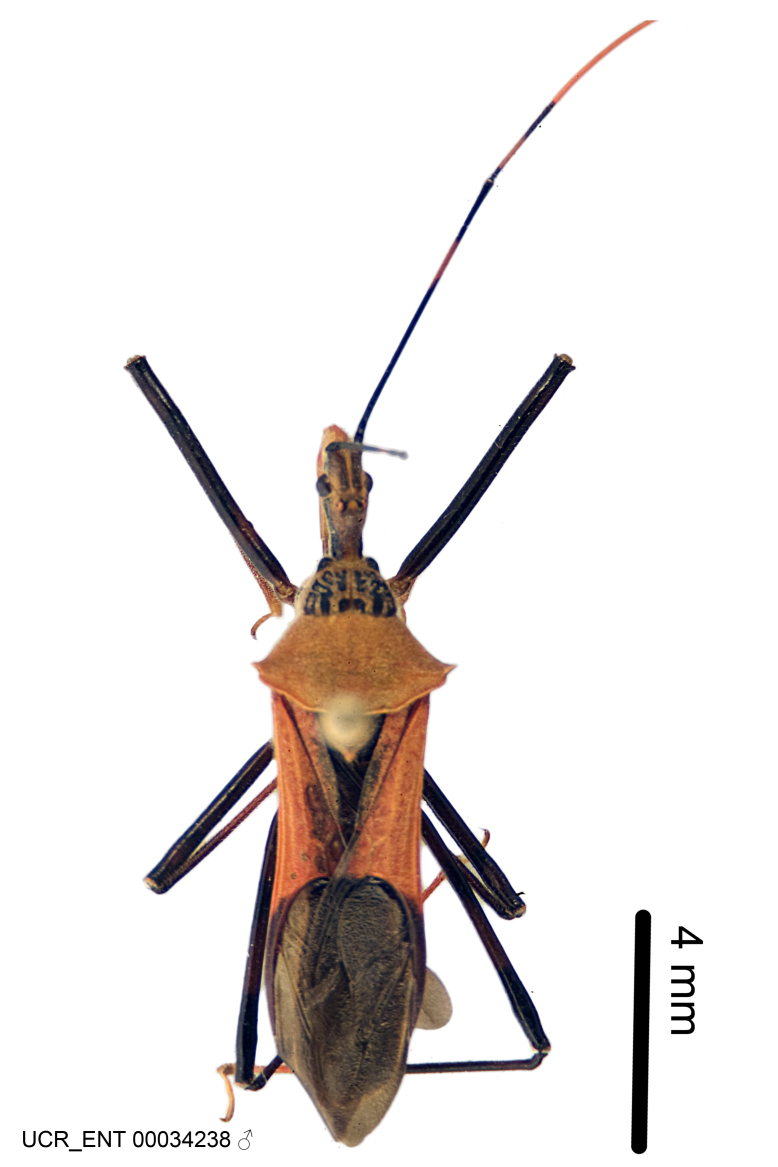
*Zelus janus* Stål, 1862, male, dorsal view (UCR_ENT 00034238, Oaxaca, Mexico)

**Figure 104a.**
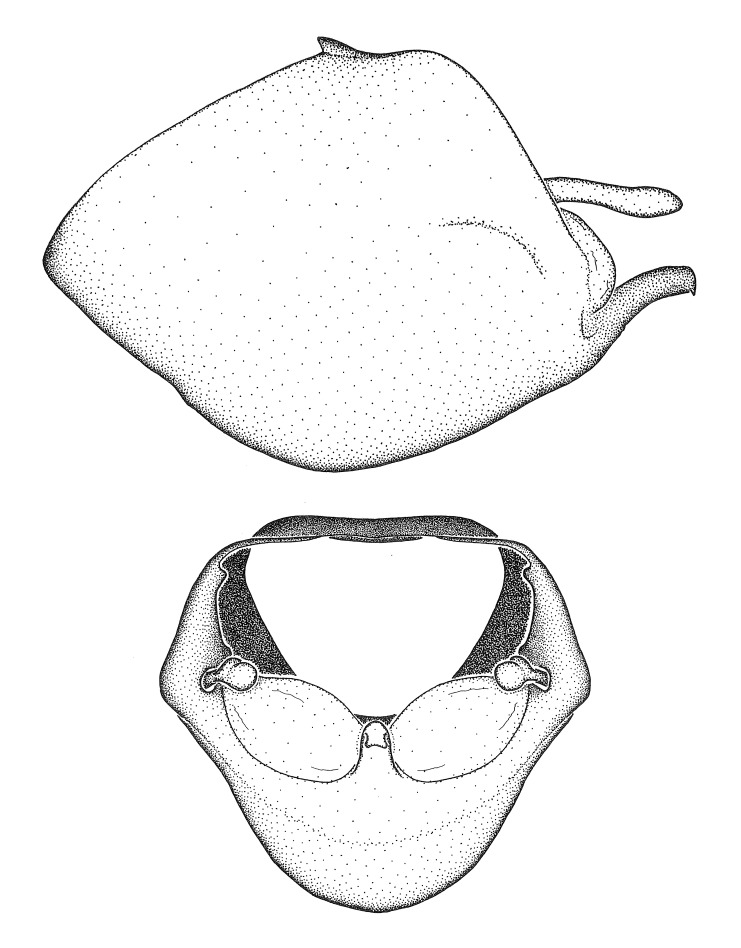


**Figure 104b.**
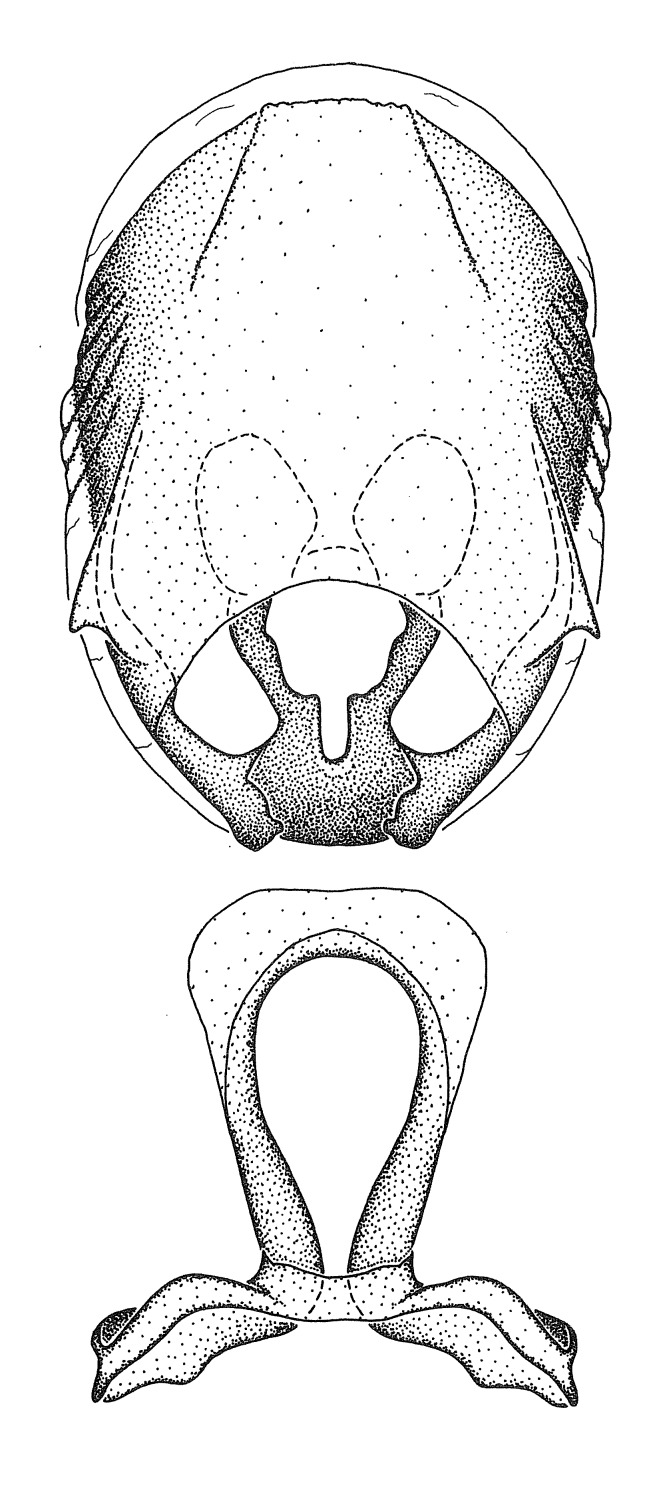


**Figure 106a.**
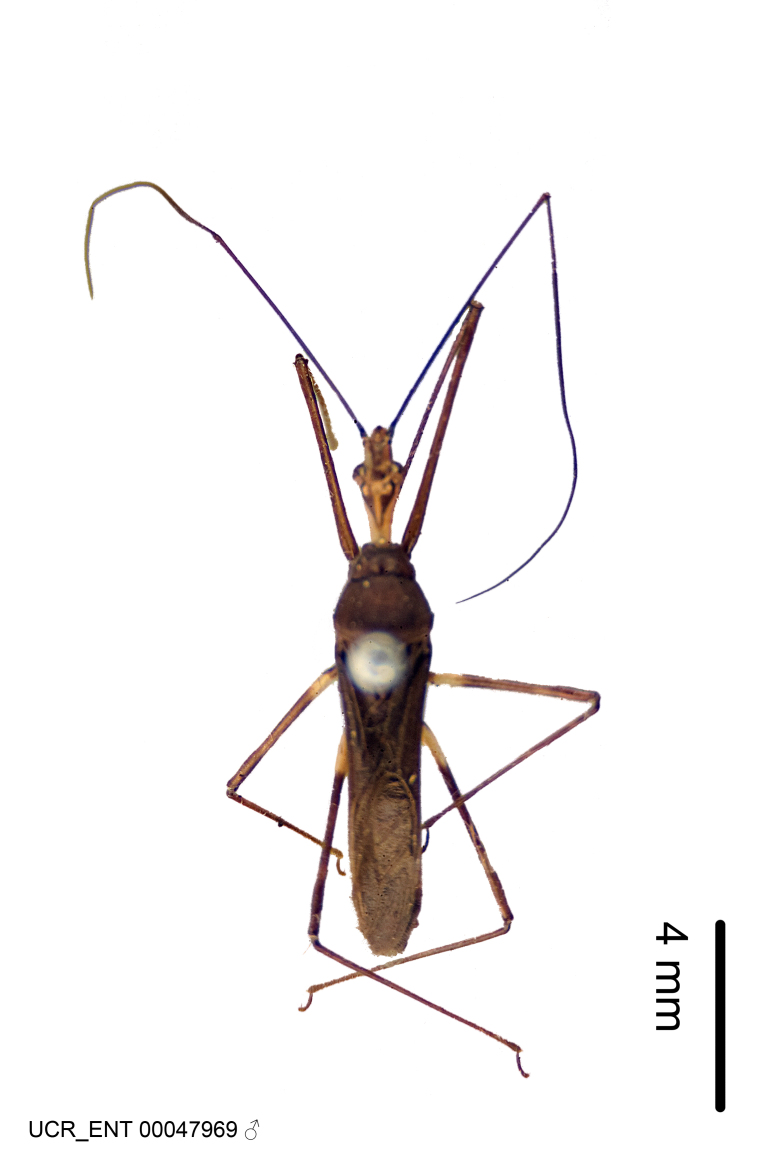
*Zelus kartabensis* Haviland, 1931, male, dorsal view (UCR_ENT 00047969, Para, Brazil)

**Figure 106b.**
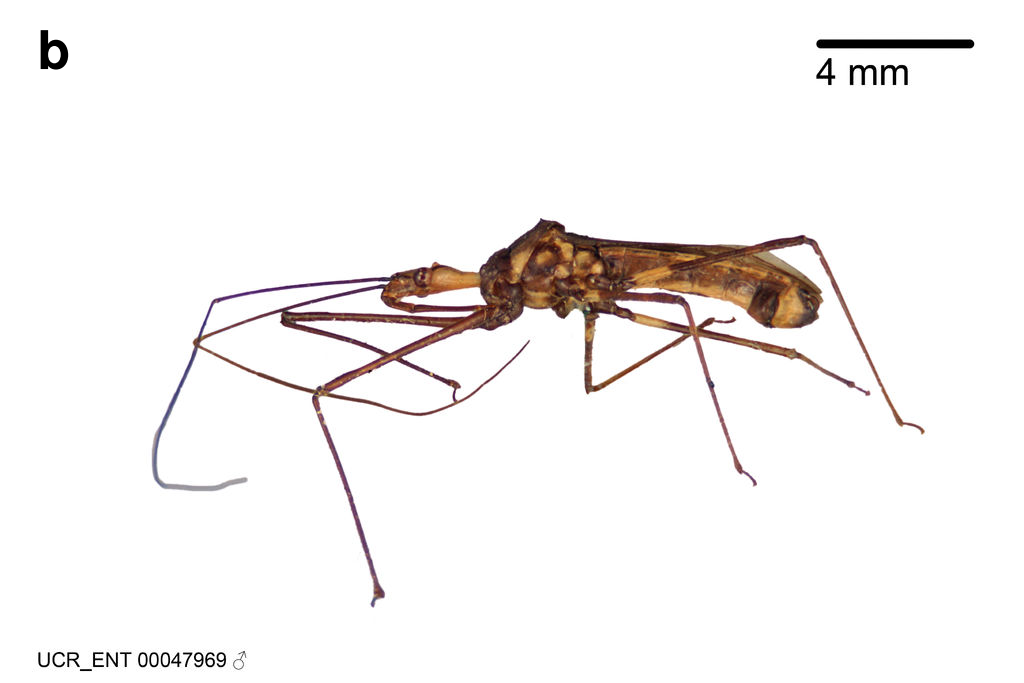
*Zelus kartabensis* Haviland, 1931, male, lateral view (UCR_ENT 00047969, Para, Brazil)

**Figure 106c.**
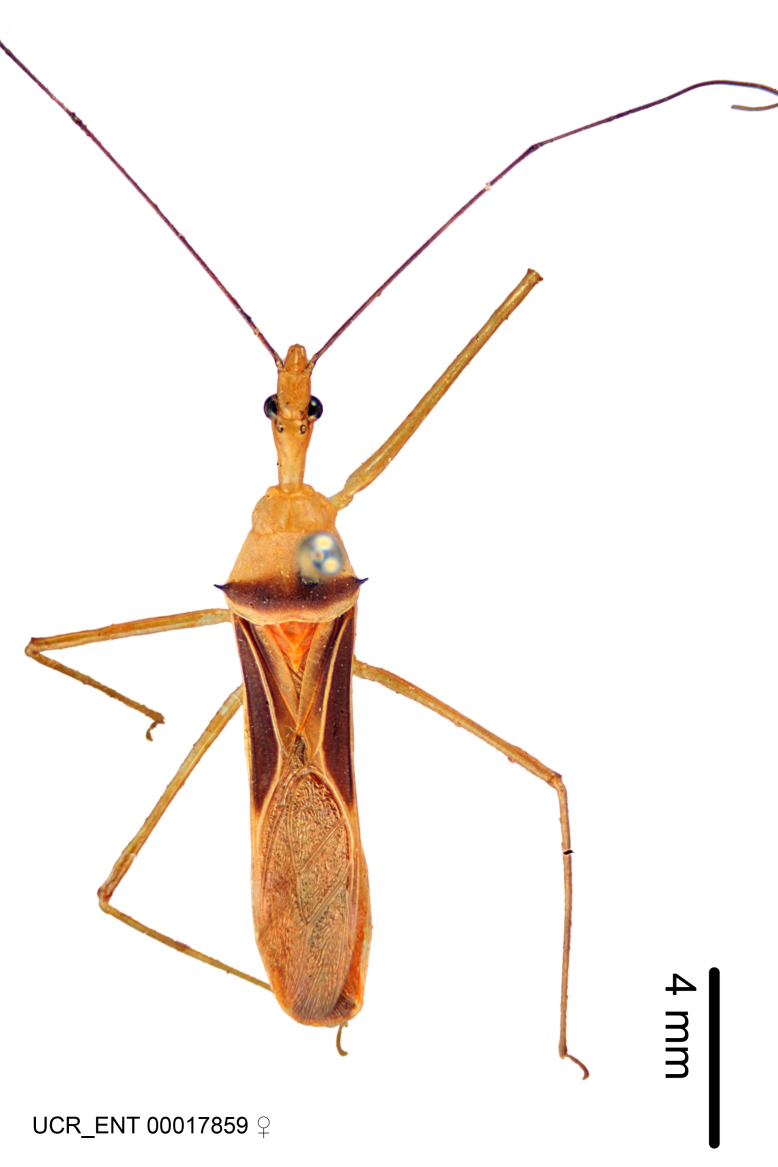
*Zelus kartabensis* Haviland, 1931, female, dorsal view (UCR_ENT 00017859, Para, Brazil)

**Figure 106d.**
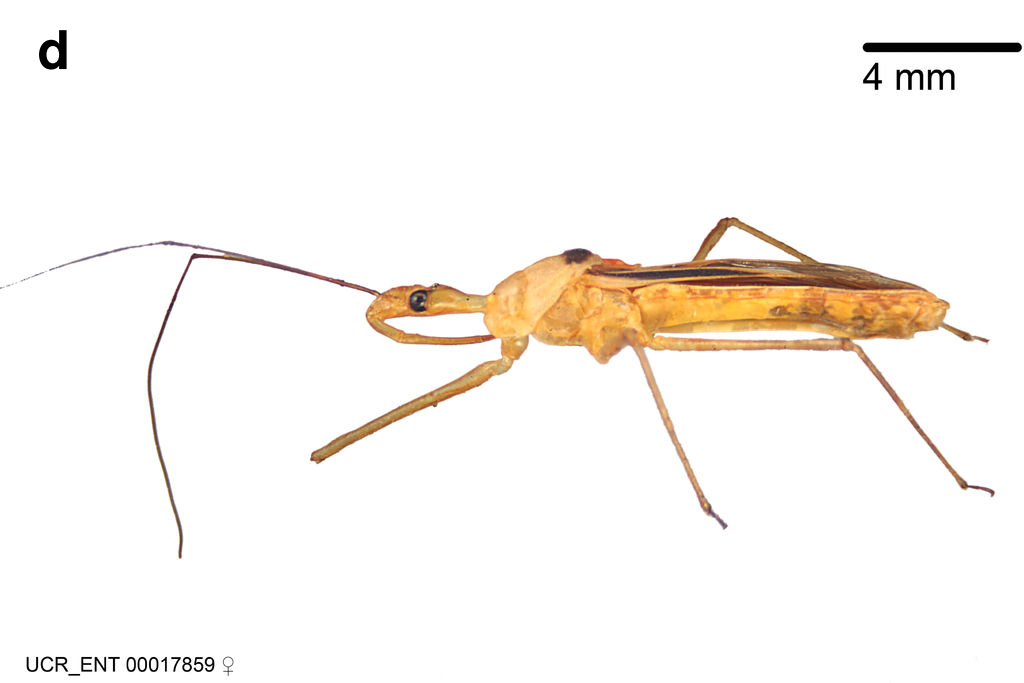
*Zelus kartabensis* Haviland, 1931, female, lateral view (UCR_ENT 00017859, Para, Brazil)

**Figure 107a.**
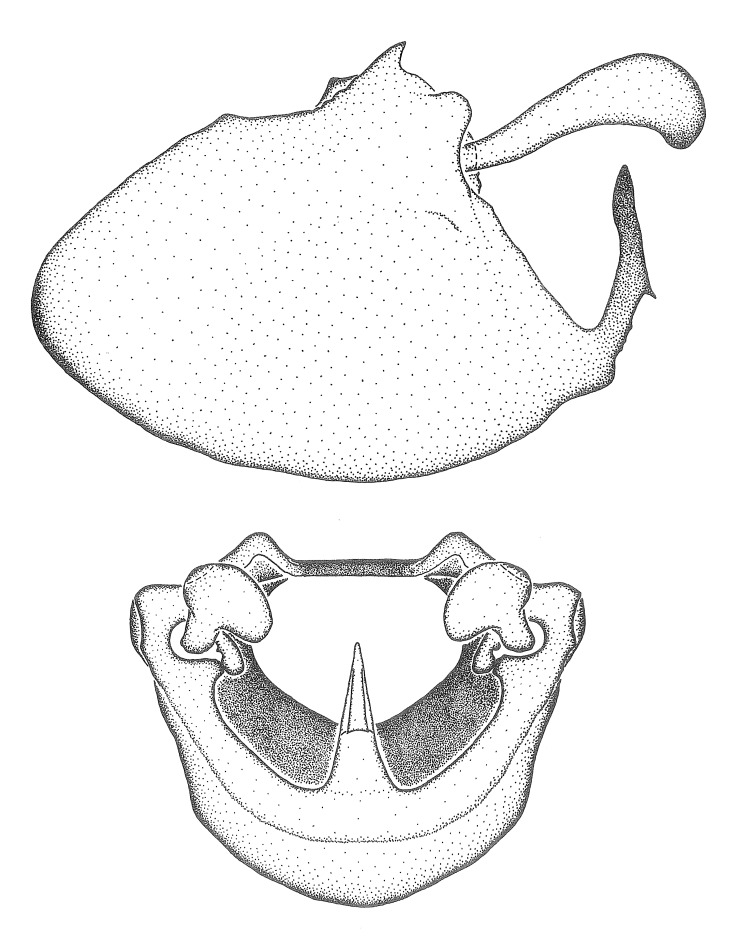
*Zelus kartabensis* Haviland, 1931, pygophore, lateral and posterior views

**Figure 107b.**
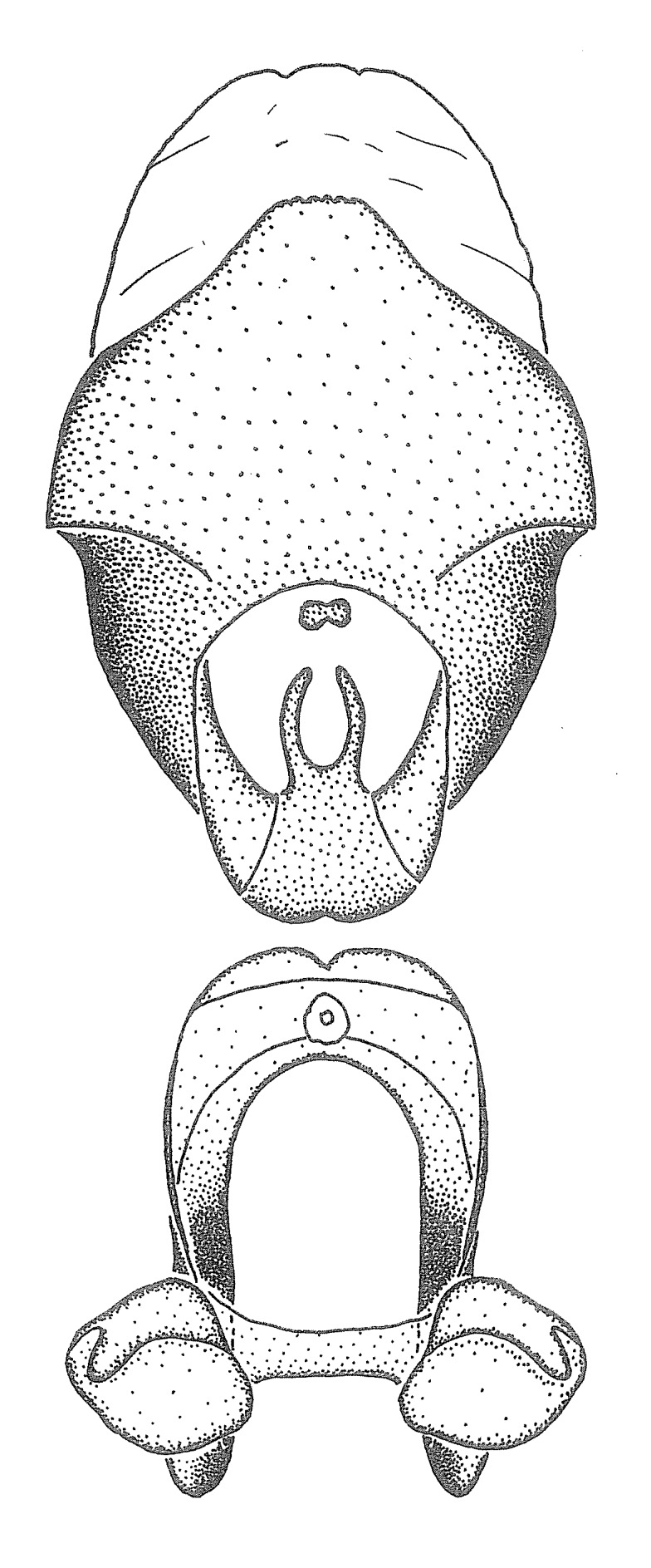
*Zelus kartabensis* Haviland, 1931, phallus, dorsal view

**Figure 109a.**
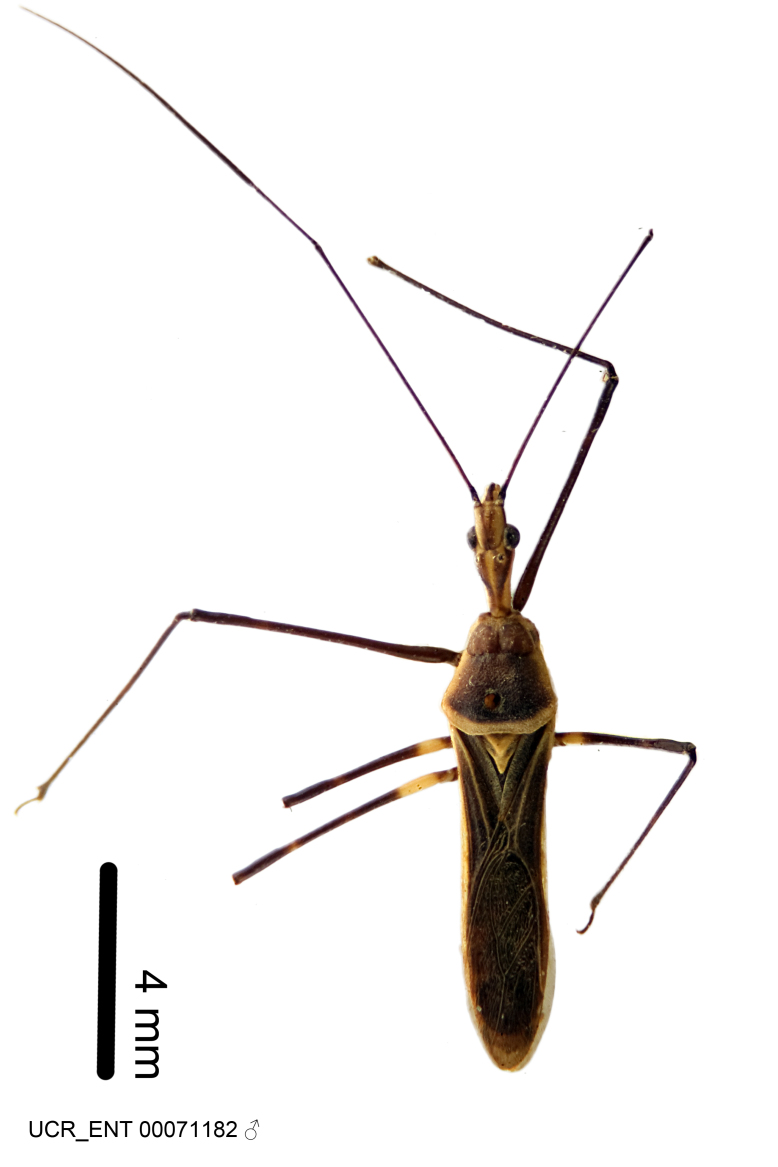
*Zelus kartaboides* Zhang & Hart, sp. n., male, dorsal view (UCR_ENT 00071182, Meta, Colombia)

**Figure 109b.**
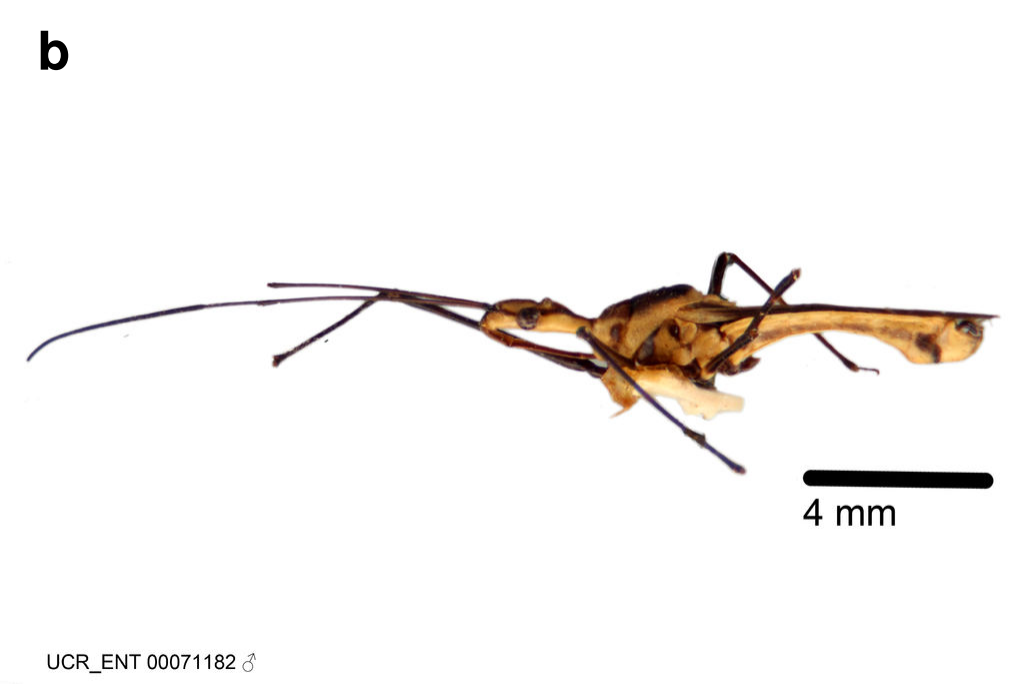
*Zelus kartaboides* Zhang & Hart, sp. n., male, lateral view (UCR_ENT 00071182, Meta, Colombia)

**Figure 110a.**
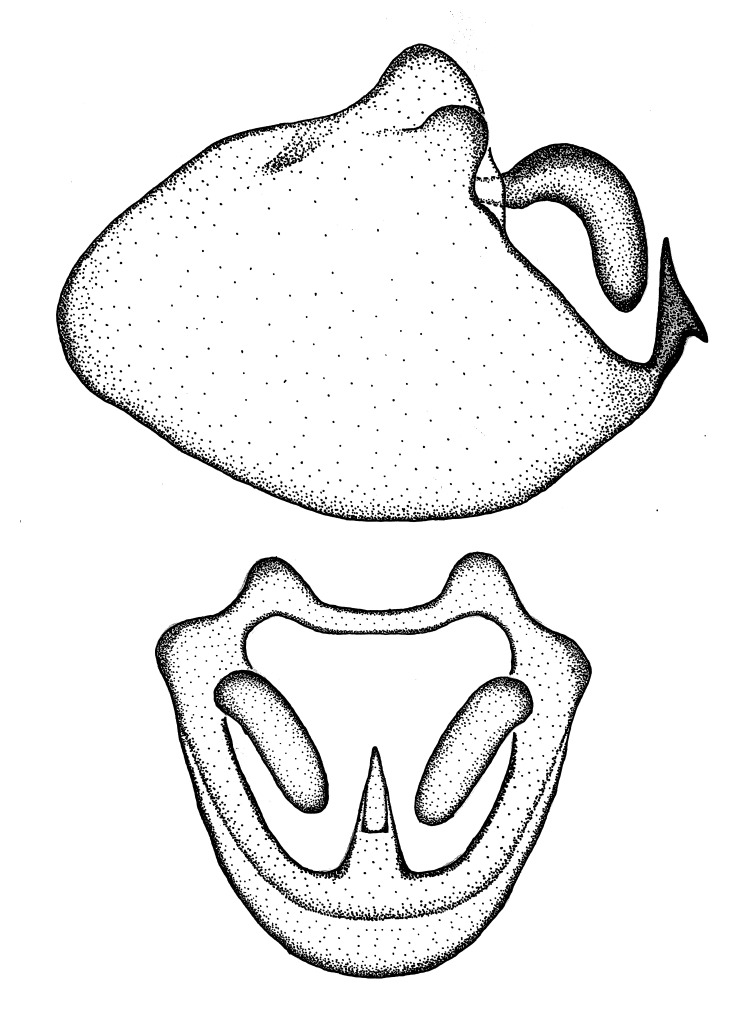
*Zelus kartaboides* Zhang & Hart, sp. n., pygophore, lateral and posterior views

**Figure 110b.**
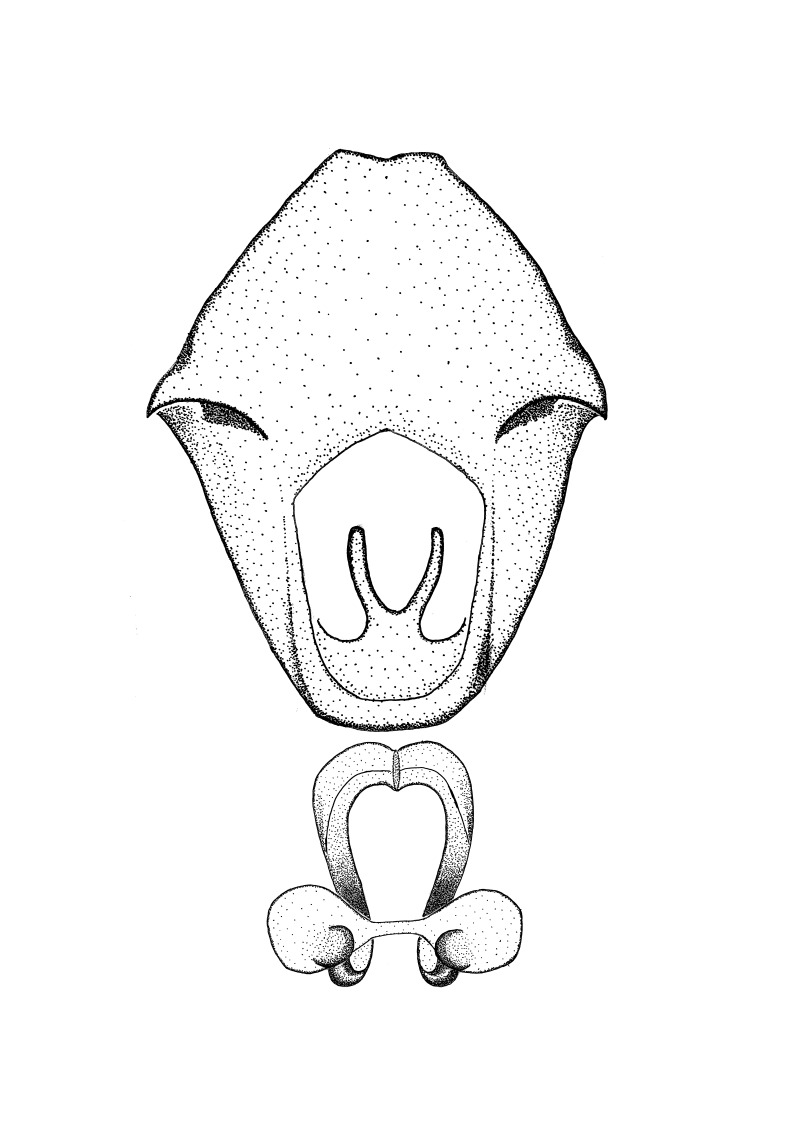
*Zelus kartaboides* Zhang & Hart, sp. n., phallus

**Figure 112a.**
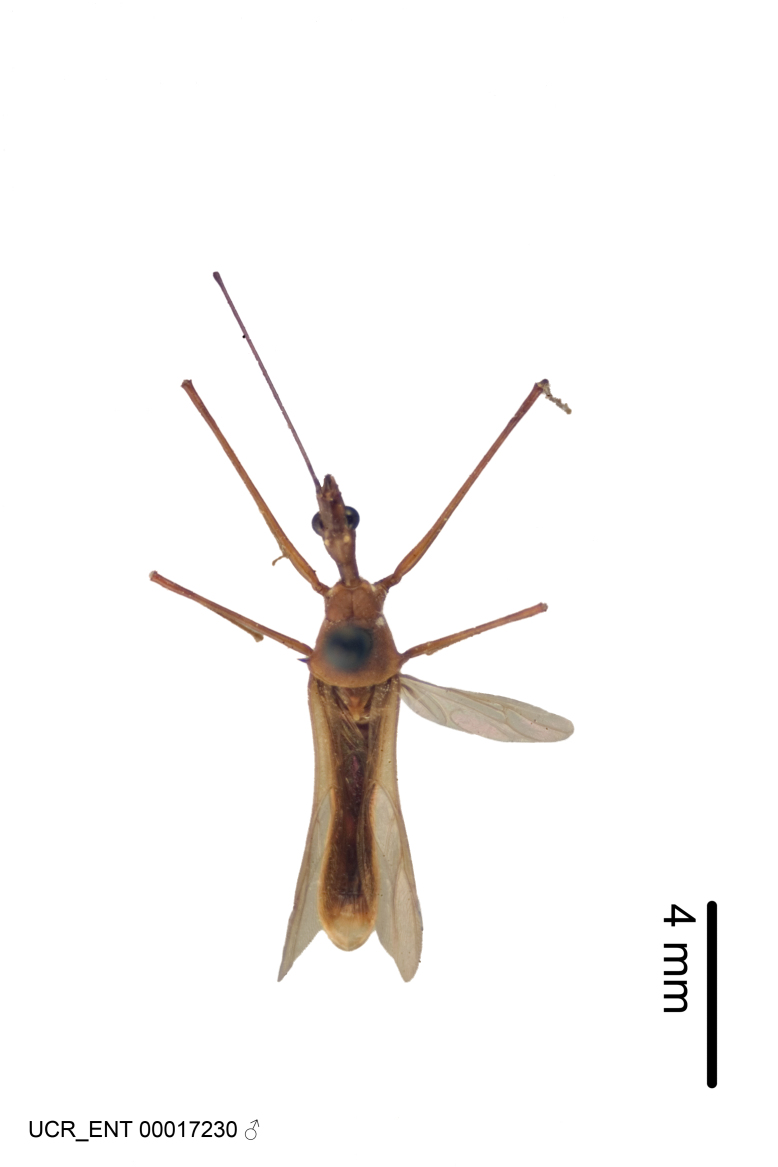
*Zelus korystos* Hart, 1986, male, dorsal view (UCR_ENT 00017230, Cuyuni-Mazaruni, Guyana)

**Figure 112b.**
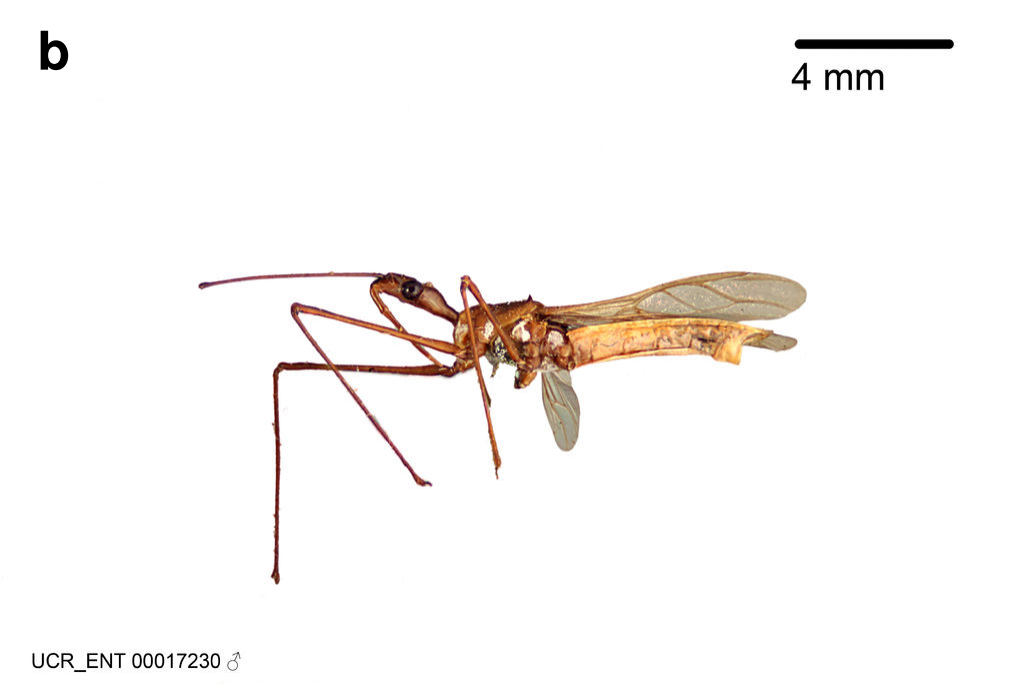
*Zelus korystos* Hart, 1986, male, lateral view (UCR_ENT 00017230, Cuyuni-Mazaruni, Guyana)

**Figure 113a.**
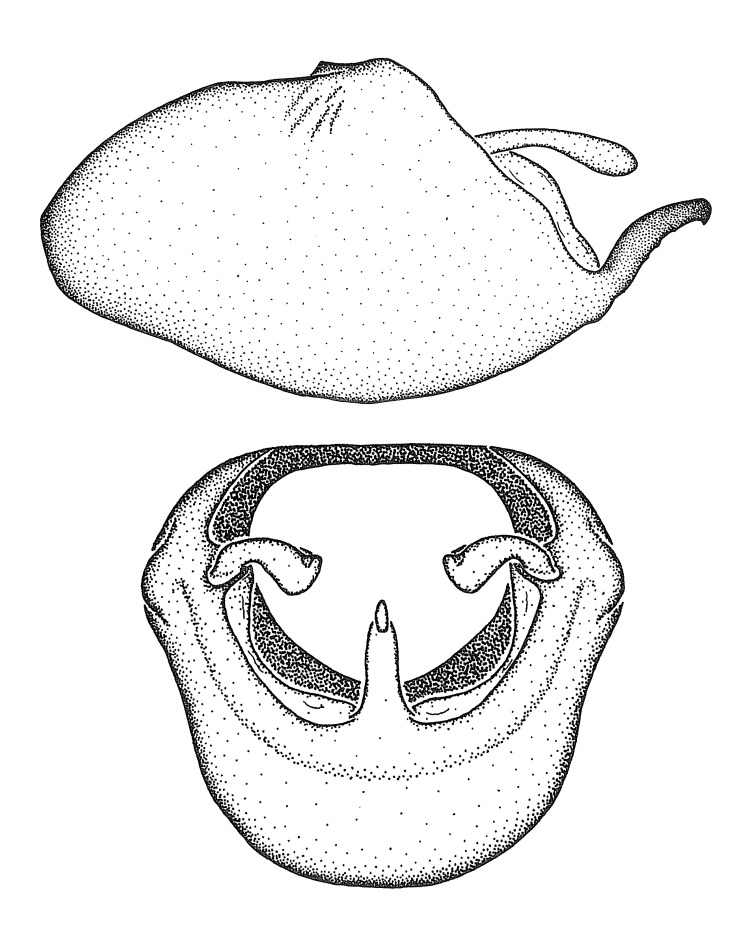
*Zelus korystos* Hart, 1986, pygophore, lateral and posterior views

**Figure 113b.**
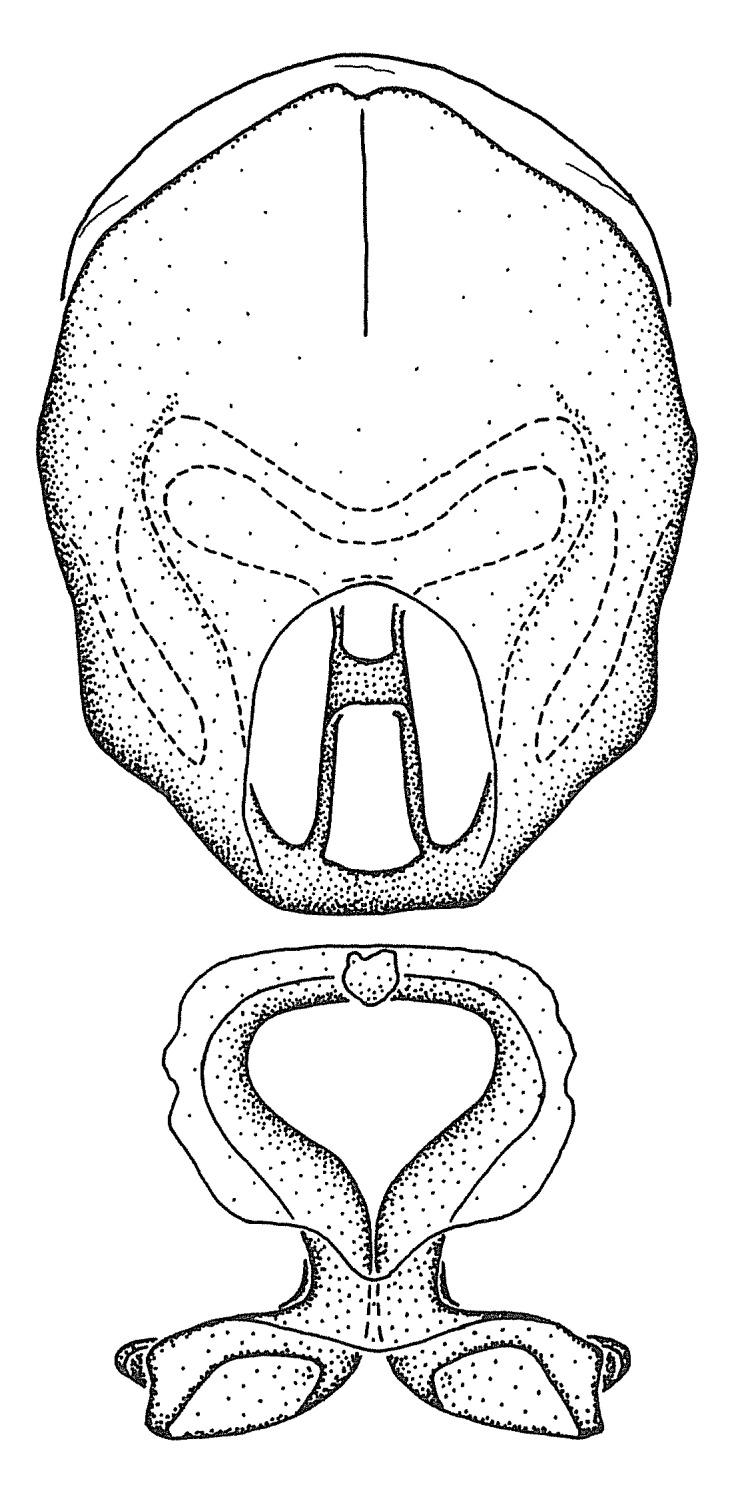
*Zelus korystos* Hart, 1986, phallus, dorsal view

**Figure 115a.**
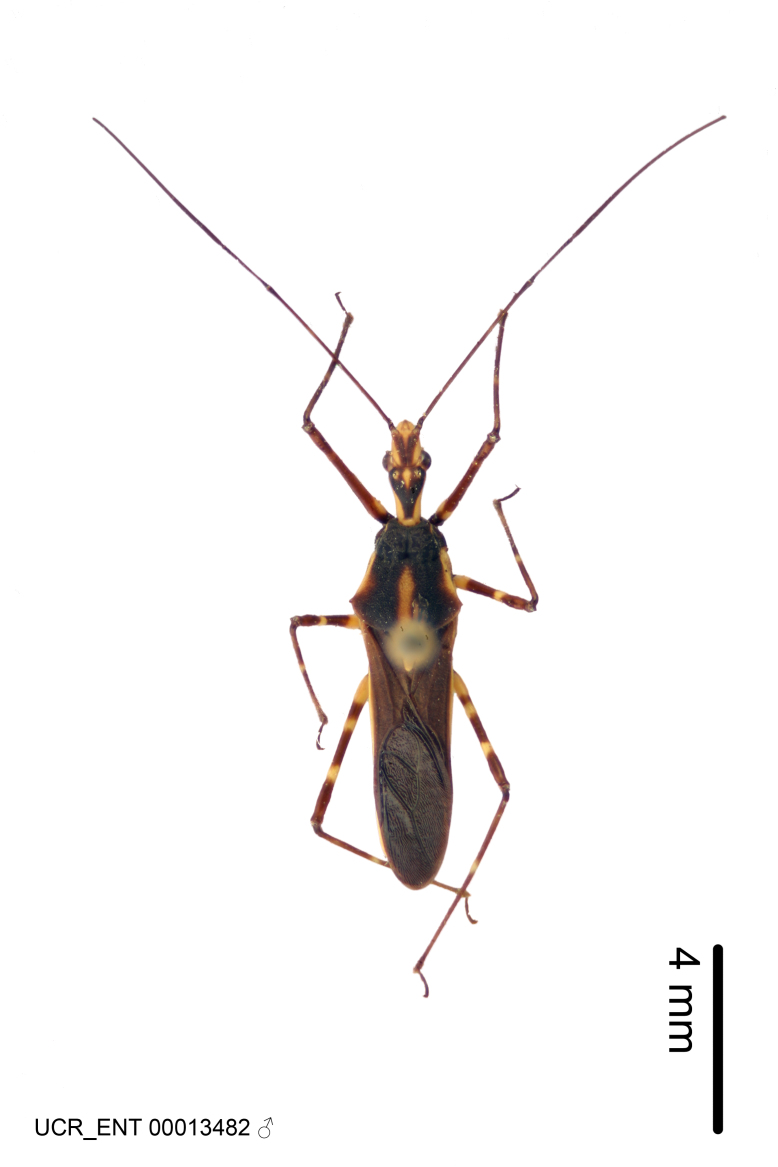
*Zelus laticornis* (Herrich-Schaeffer, 1853), male, dorsal view (UCR_ENT 00013482, Suriname)

**Figure 115b.**
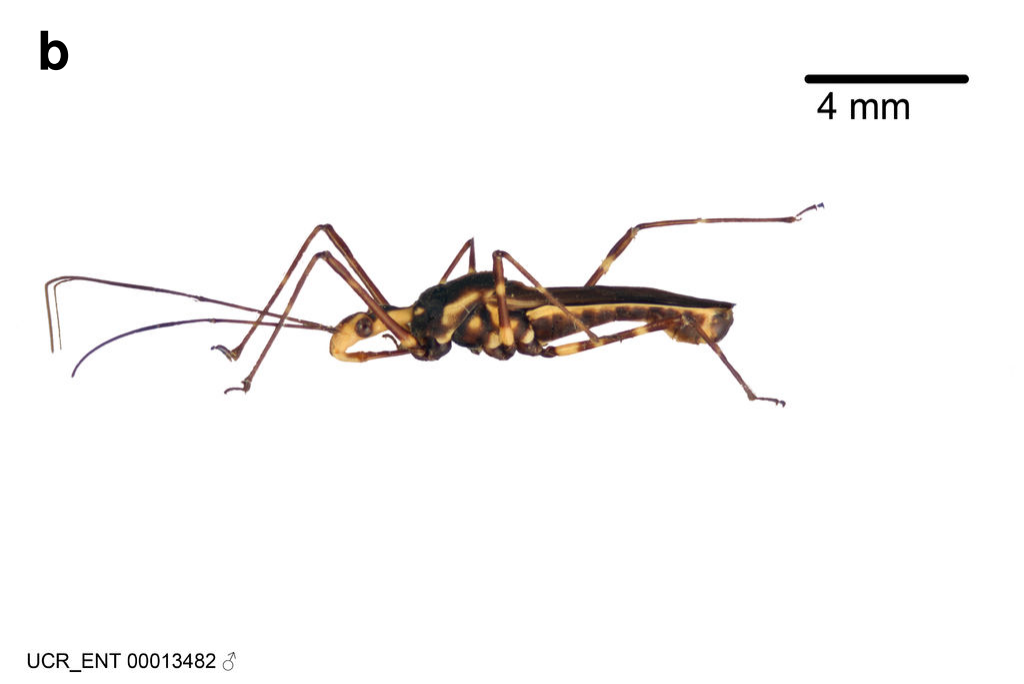
*Zelus laticornis* (Herrich-Schaeffer, 1853), male, lateral view (UCR_ENT 00013482, Suriname)

**Figure 115c.**
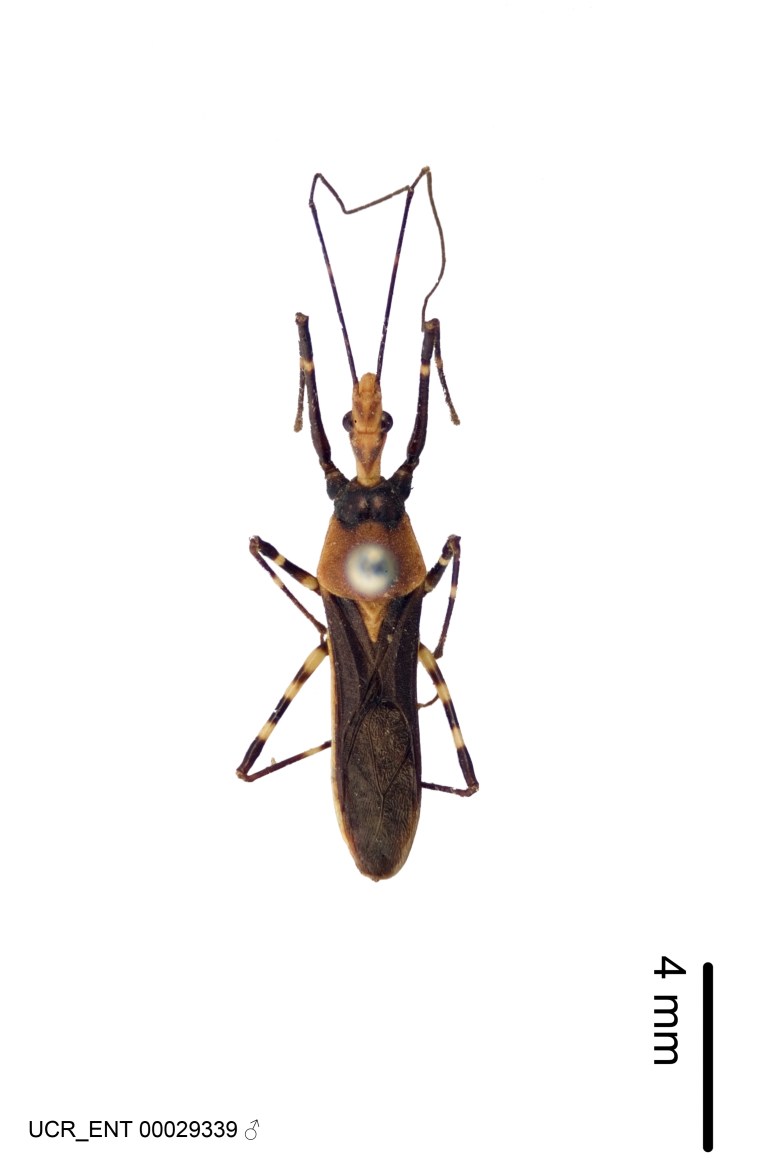
*Zelus laticornis* (Herrich-Schaeffer, 1853), male, dorsal view (UCR_ENT 00029339, Concepción, Paraguay)

**Figure 115d.**
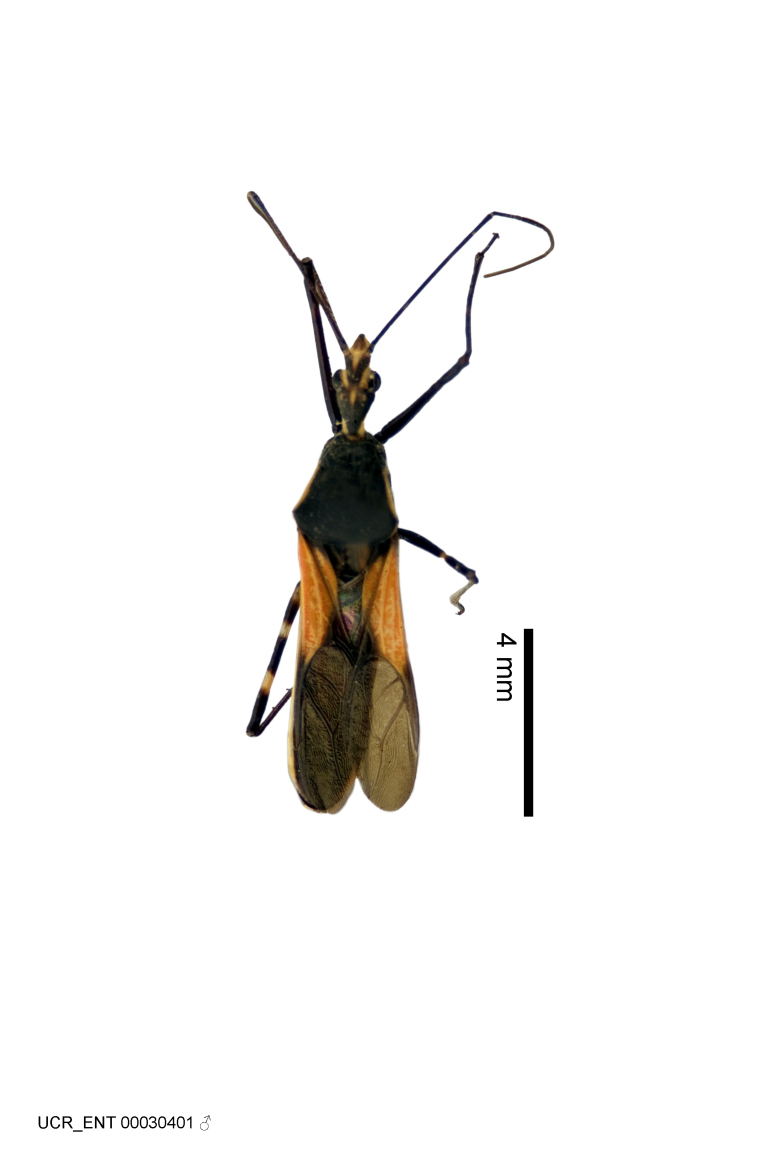
*Zelus laticornisI* (Herrich-Schaeffer, 1853), male dorsal (UCR_ENT 00030401, Zamora-Chinchipe, Ecuador)

**Figure 115e.**
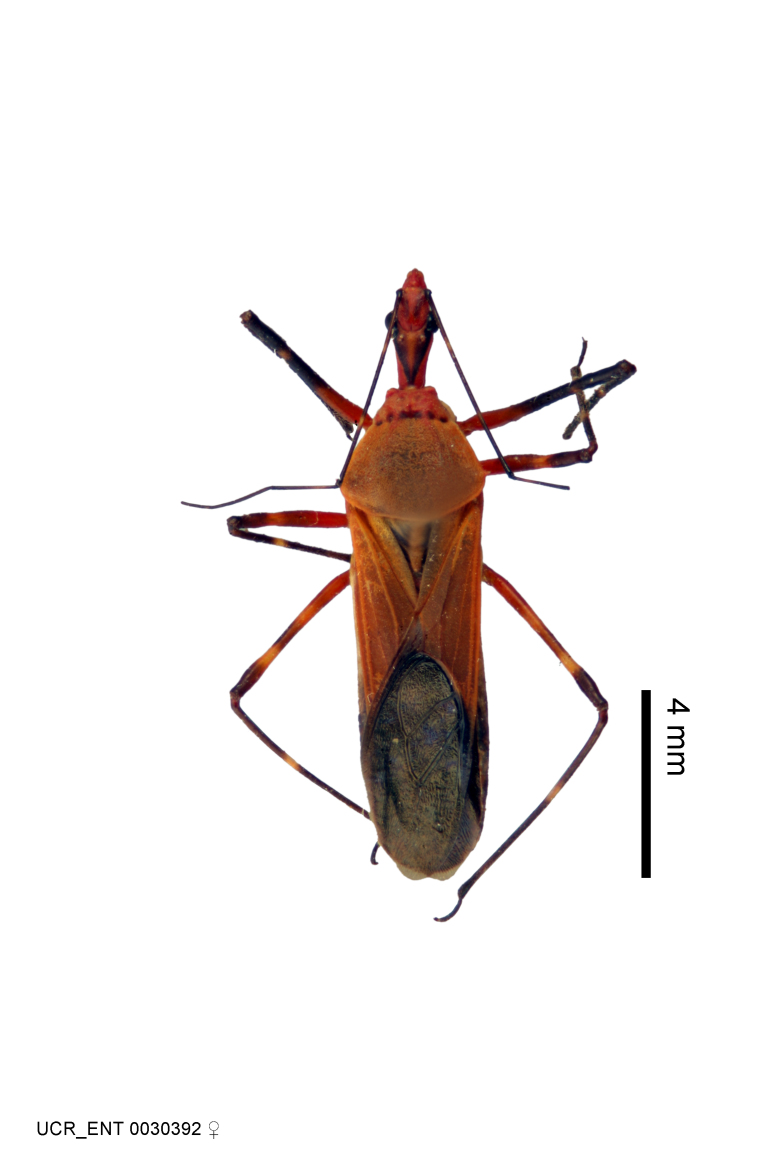
*Zelus laticornis* (Herrich-Schaeffer, 1853), female, dorsal view (UCR_ENT 00030392, La Paz, Bolivia)

**Figure 115f.**
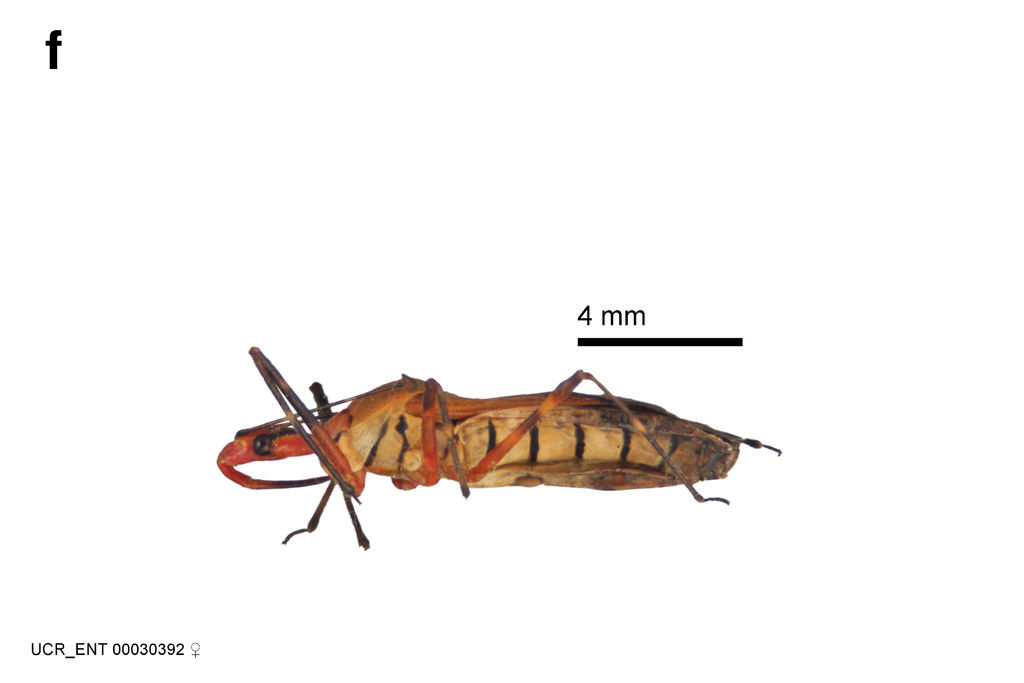
*Zelus laticornis* (Herrich-Schaeffer, 1853), female, lateral view (UCR_ENT 00030392, La Paz, Bolivia)

**Figure 116a.**
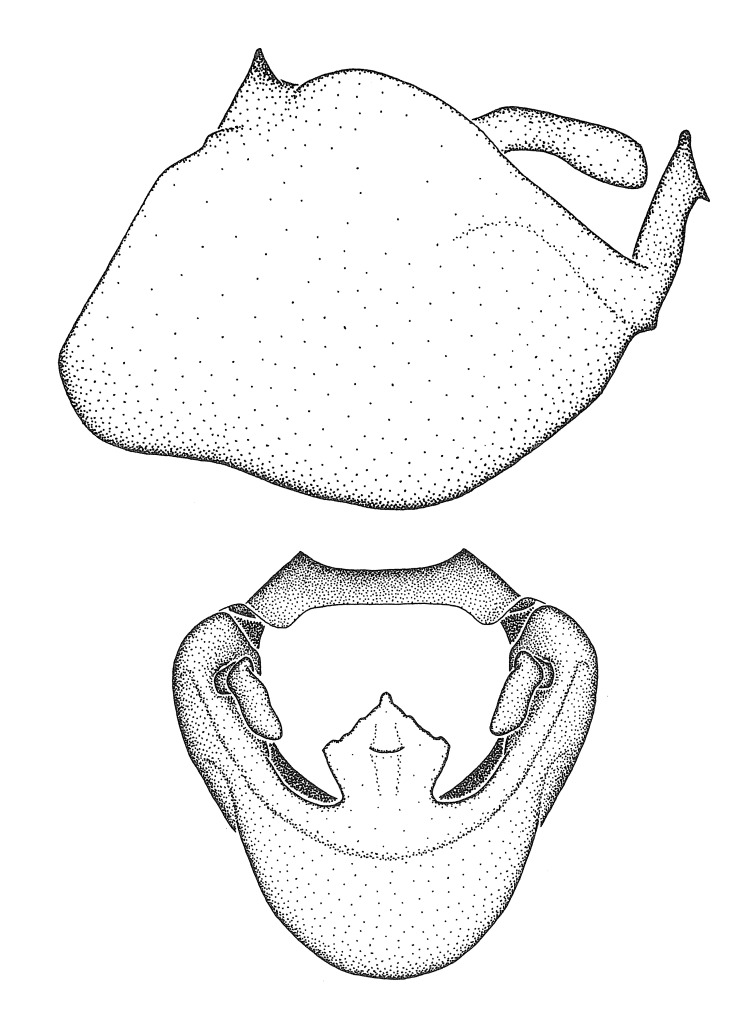
*Zelus laticornis* (Herrich-Schaeffer, 1853), pygophore, lateral and posterior views

**Figure 116b.**
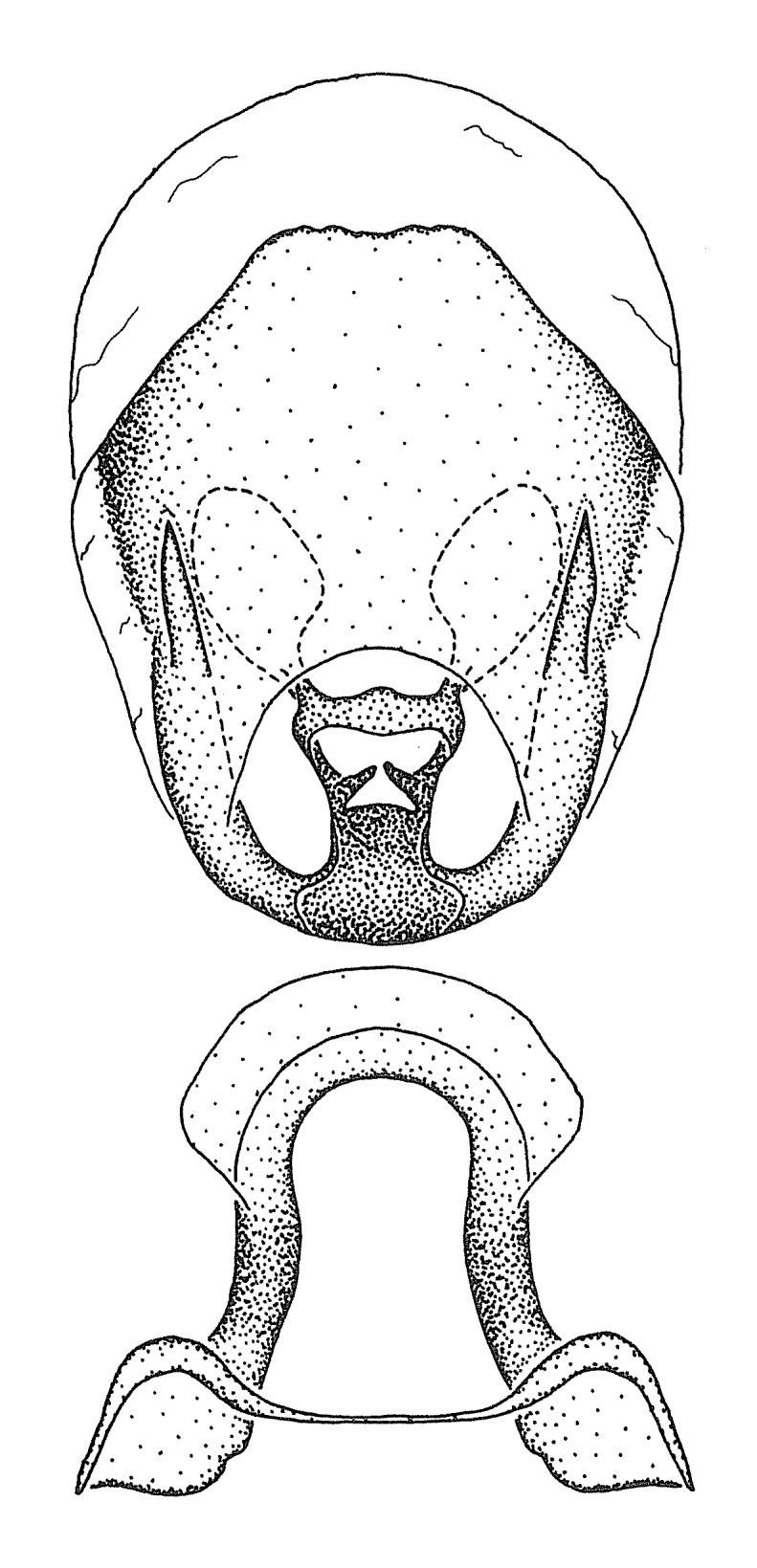
*Zelus laticornis* (Herrich-Schaeffer, 1853), phallus, dorsal view

**Figure 118a.**
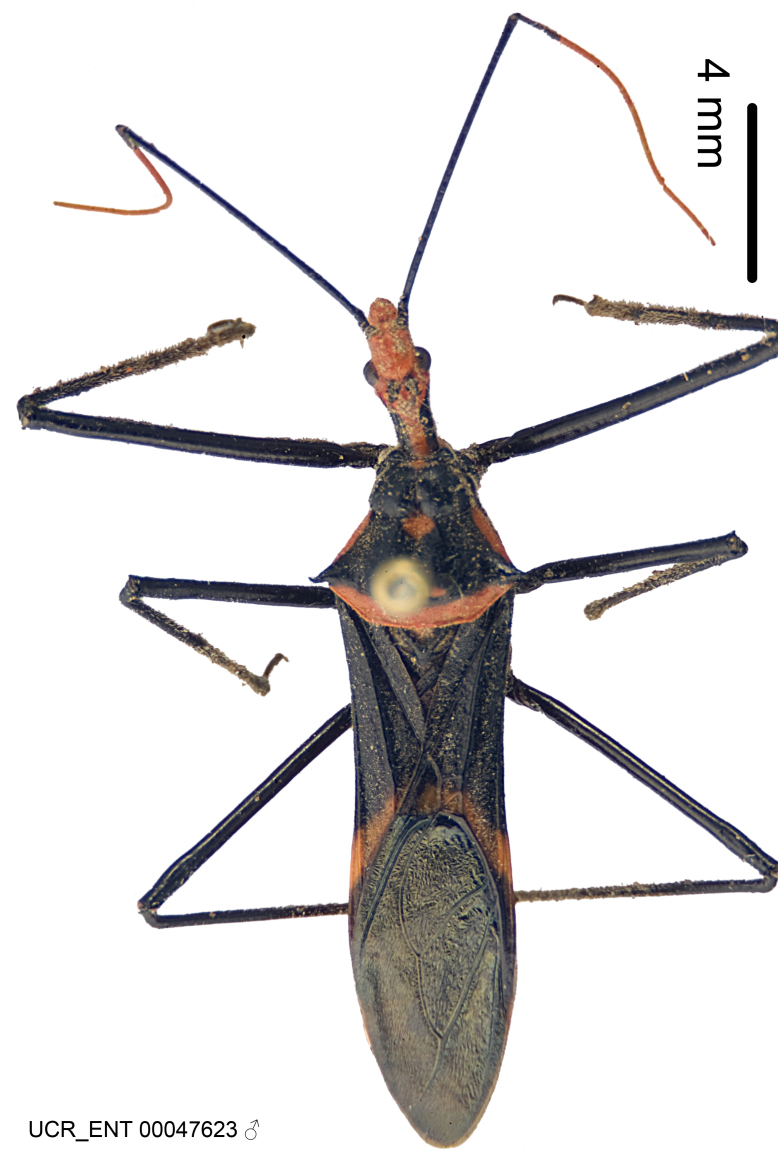
*Zelus leucogrammus* (Perty, 1833), male, dorsal view (UCR_ENT 00047623, Minas Gerais, Brazil)

**Figure 118b.**
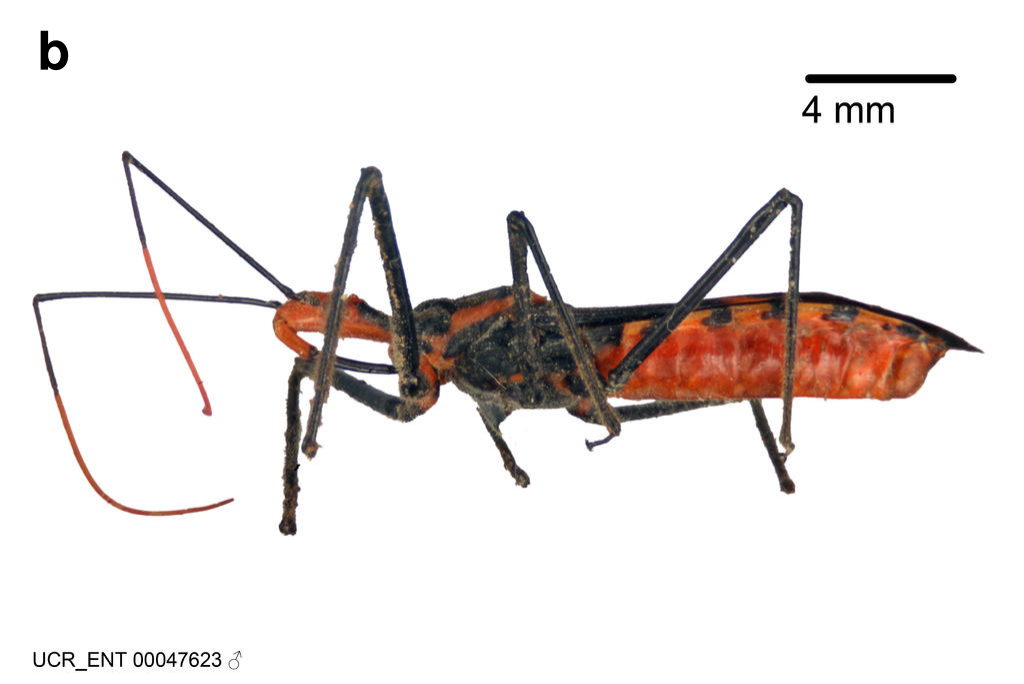
*Zelus leucogrammus* (Perty, 1833), male, lateral view (UCR_ENT 00047623, Minas Gerais, Brazil)

**Figure 118c.**
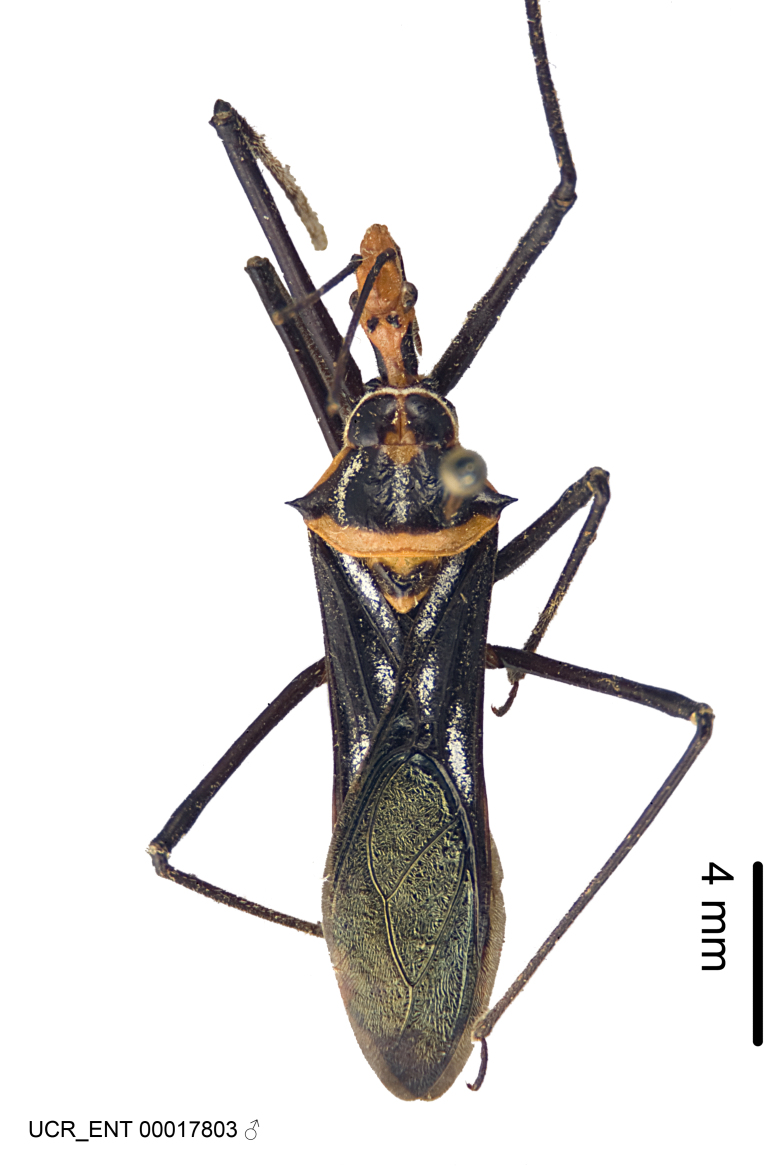
*Zelus leucogrammus* (Perty, 1833), male, dorsal view (UCR_ENT 00017803, Itapua, Paraguay)

**Figure 118d.**
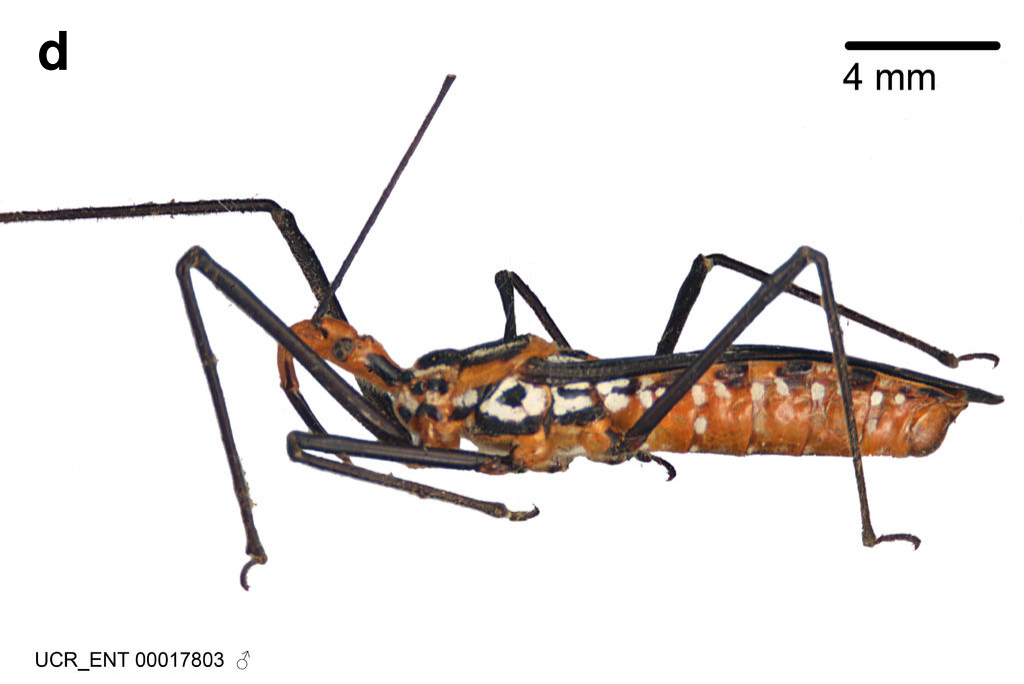
*Zelus leucogrammus* (Perty, 1833), male, lateral view (UCR_ENT 00017803, Itapua, Paraguay)

**Figure 118e.**
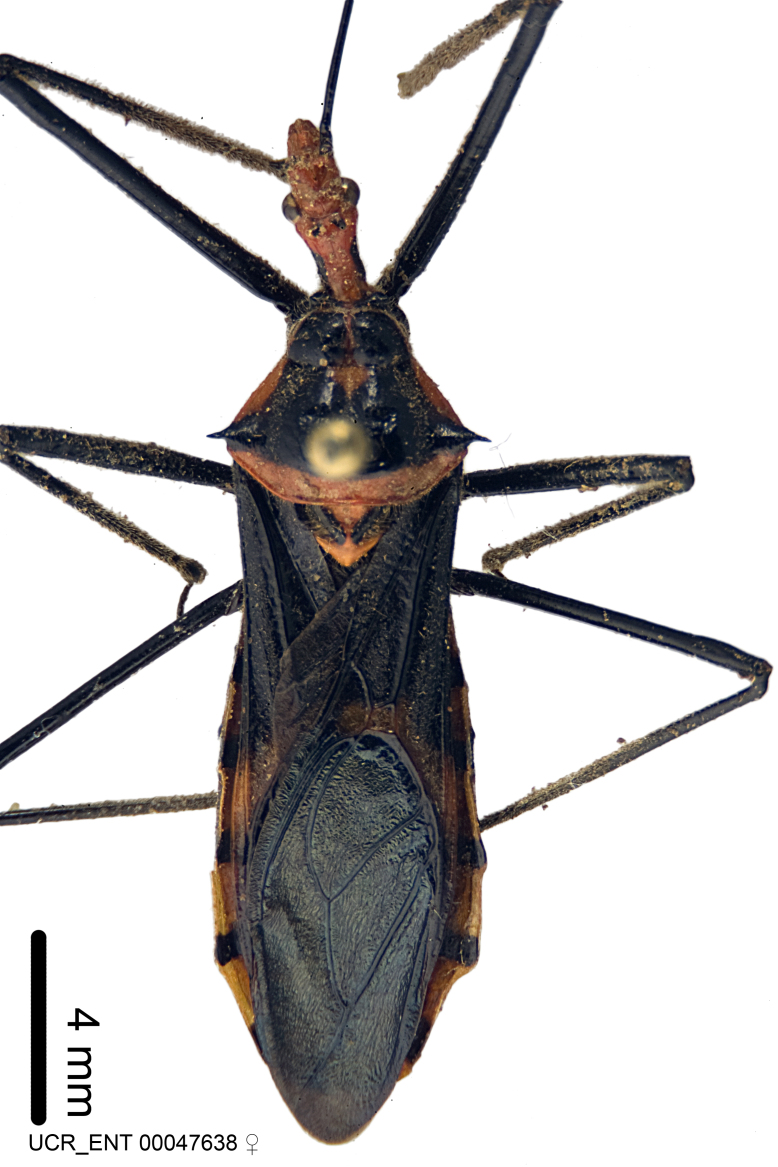
*Zelus leucogrammus* (Perty, 1833), female, dorsal view (UCR_ENT 00047638, Minas Gerais, Brazil)

**Figure 118f.**
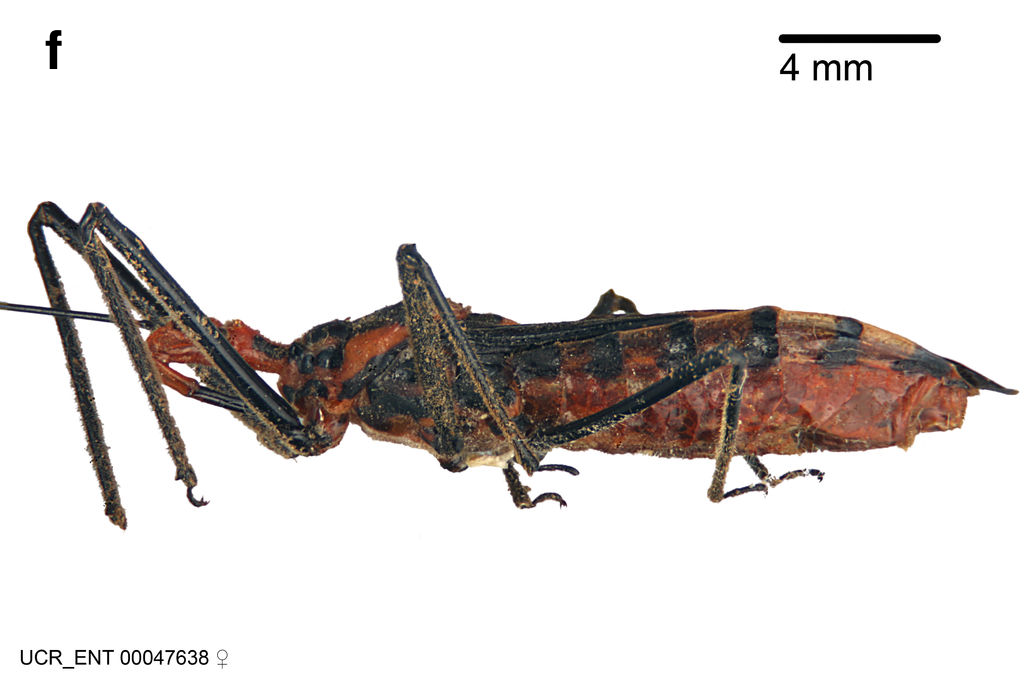
*Zelus leucogrammus* (Perty, 1833), female, lateral view (UCR_ENT 00047638, Minas Gerais, Brazil)

**Figure 119a.**
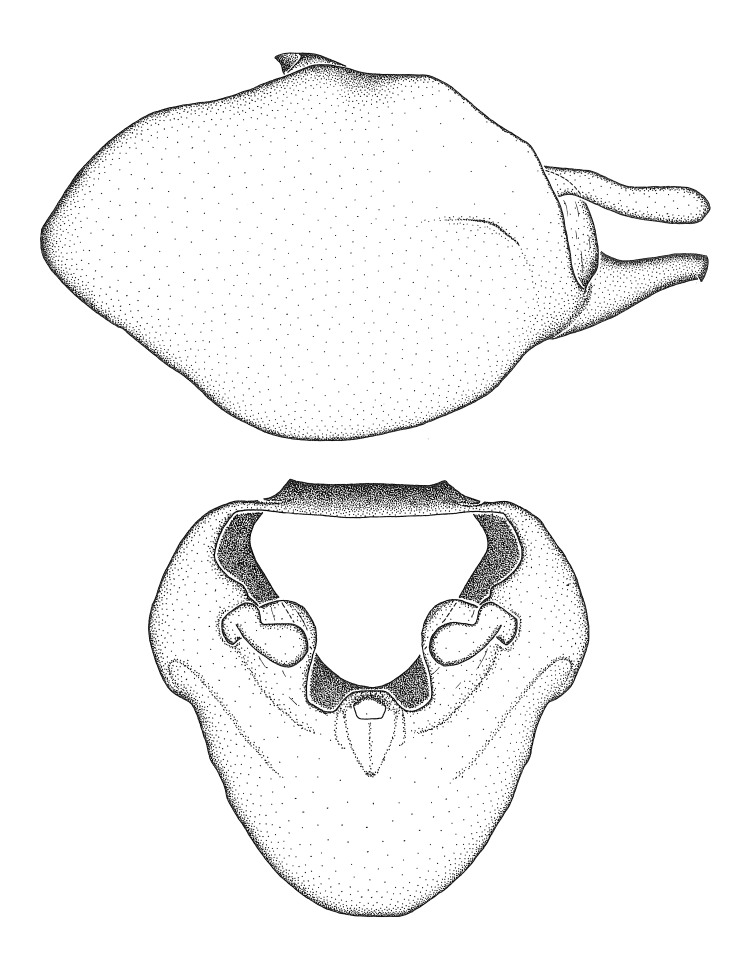
*Zelus leucogrammus* (Perty, 1833), pygophore, lateral and posterior views

**Figure 119b.**
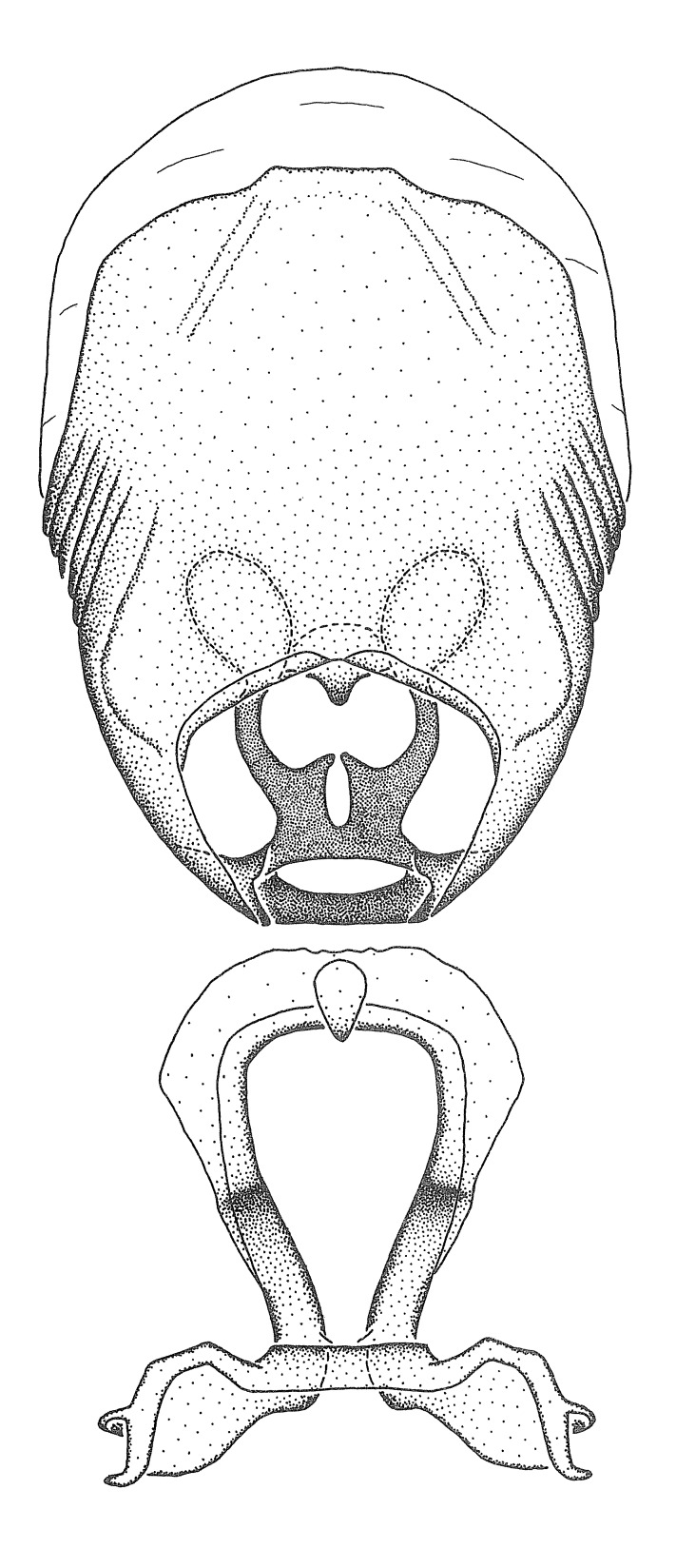
*Zelus leucogrammus* (Perty, 1833), phallus, dorsal view

**Figure 121a.**
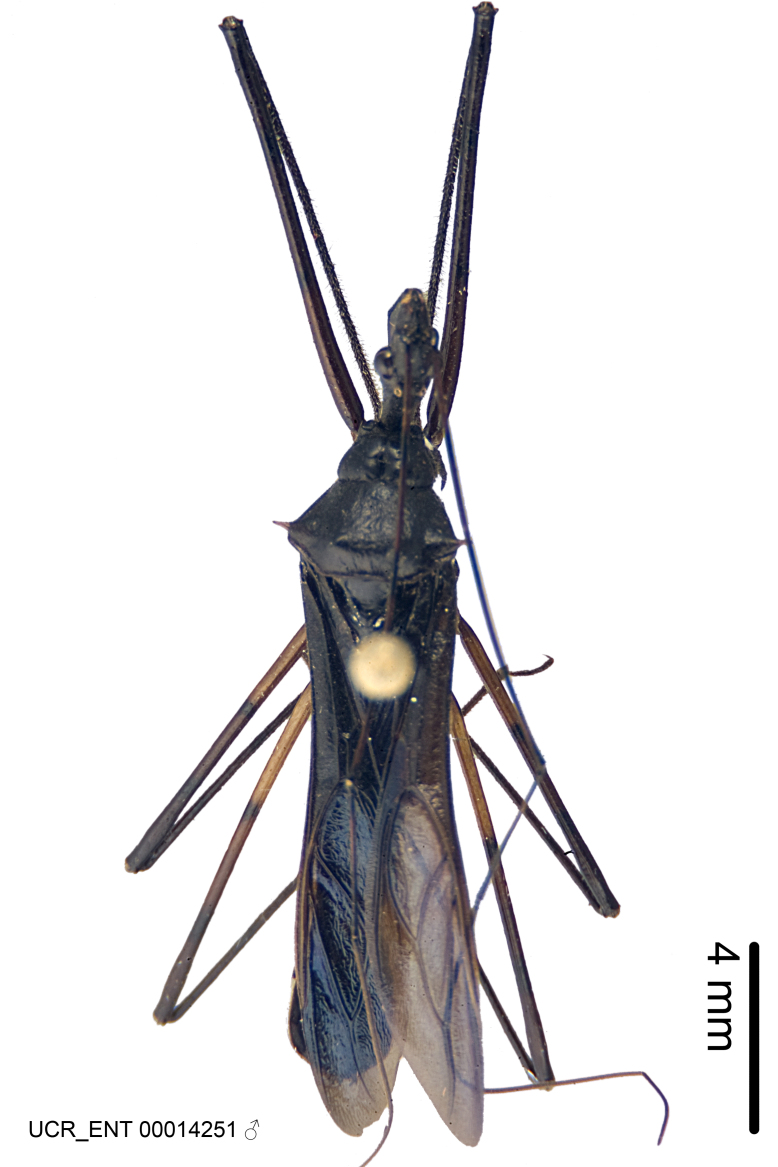
*Zelus lewisi* Zhang & Hart, sp. n., male, dorsal view (UCR_ENT 00014251, Alajuela, Costa Rica)

**Figure 121b.**
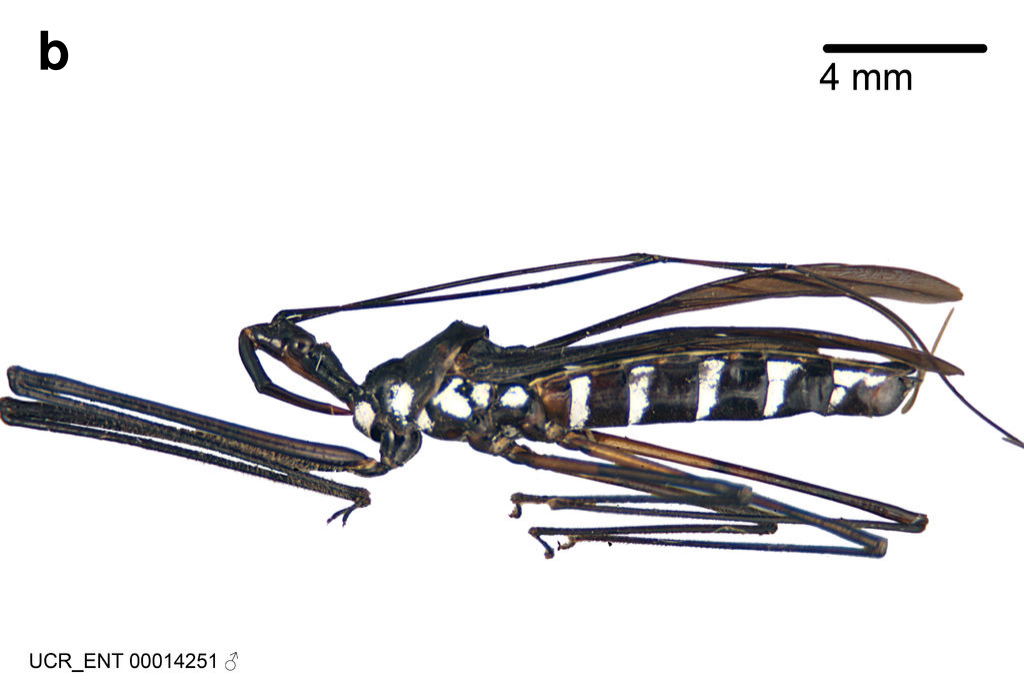
*Zelus lewisi* Zhang & Hart, sp. n., male, lateral view (UCR_ENT 00014251, Alajuela, Costa Rica)

**Figure 121c.**
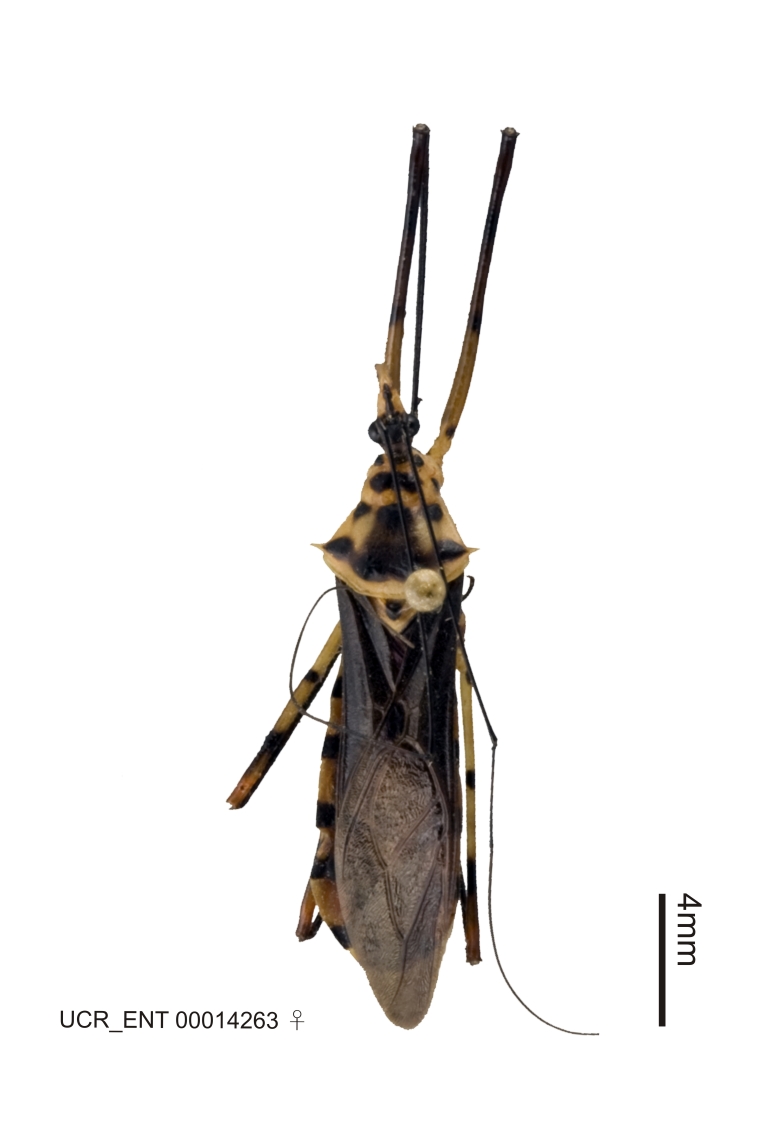
*Zelus lewisi* Zhang & Hart, sp. n., female, dorsal view (UCR_ENT 00014263, Guanacaste, Costa Rica)

**Figure 121d.**
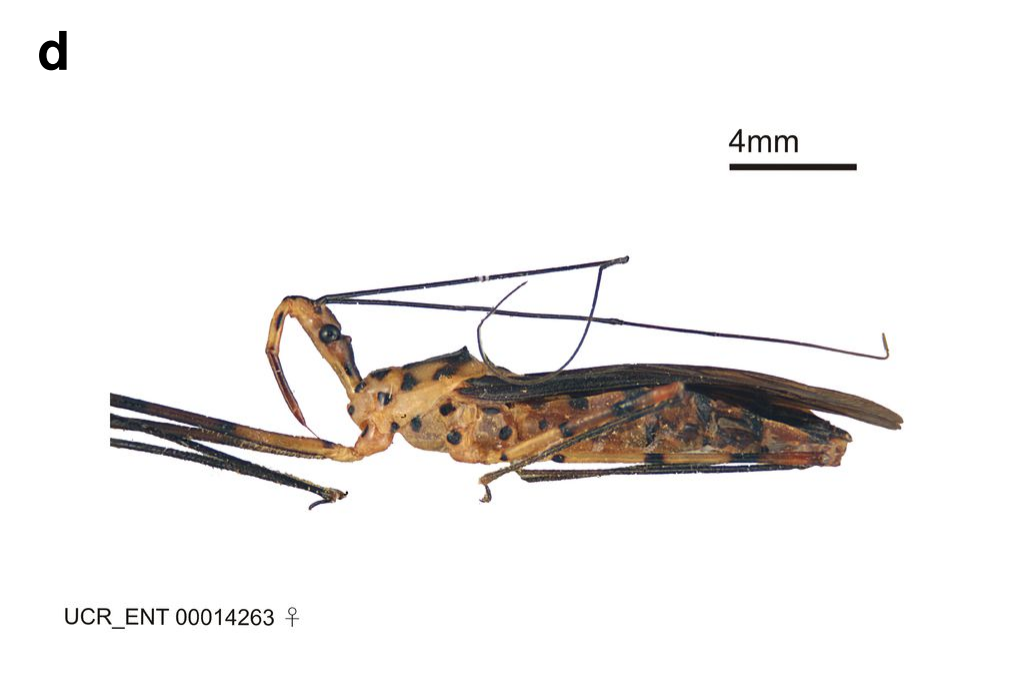
*Zelus lewisi* Zhang & Hart, sp. n., female, lateral view (UCR_ENT 00014263, Guanacaste, Costa Rica)

**Figure 121e.**
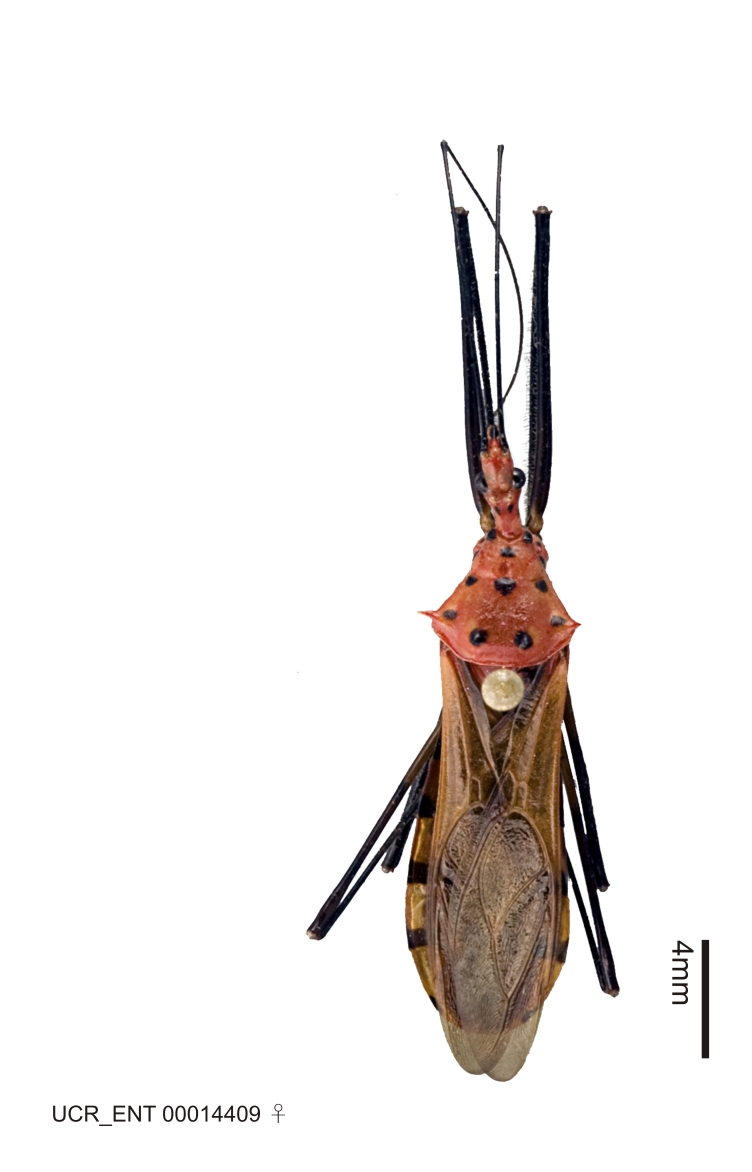
*Zelus lewisi* Zhang & Hart, sp. n., female, dorsal view (UCR_ENT 00014409, Guanacaste, Costa Rica)

**Figure 121f.**
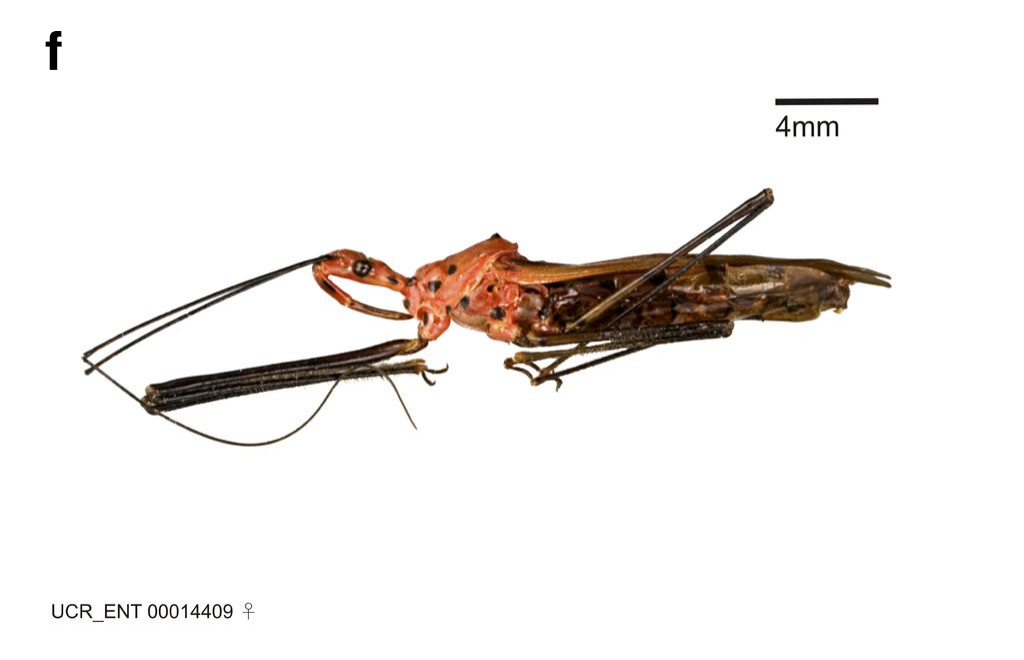
*Zelus lewisi* Zhang & Hart, sp. n., female, lateral view (UCR_ENT 00014409, Guanacaste, Costa Rica)

**Figure 122a.**
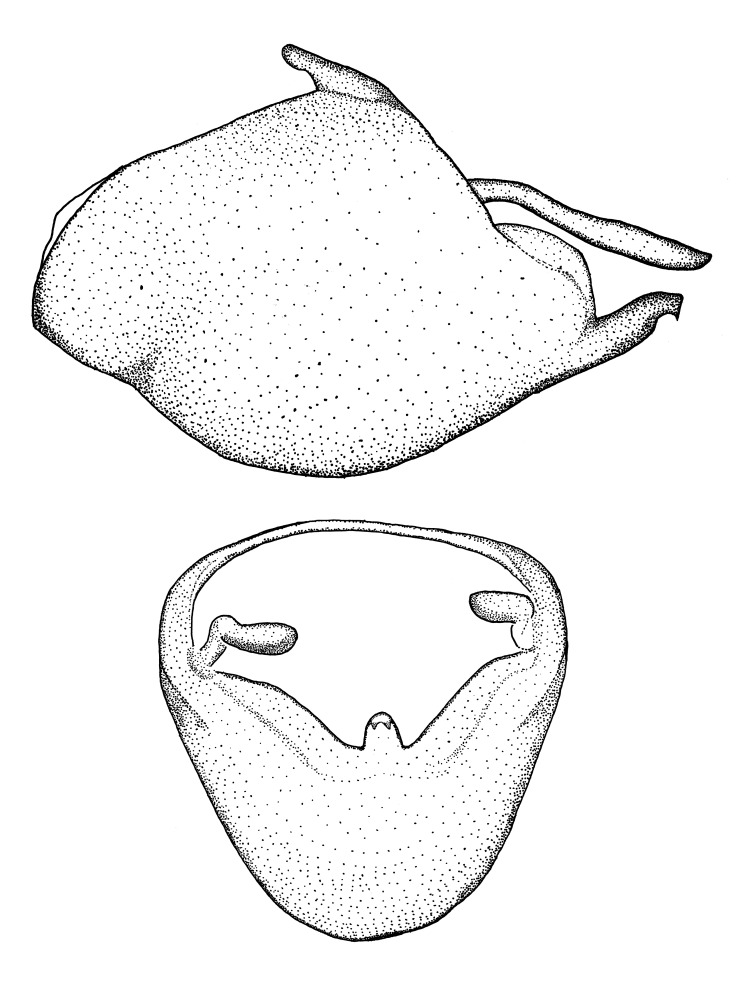


**Figure 122b.**
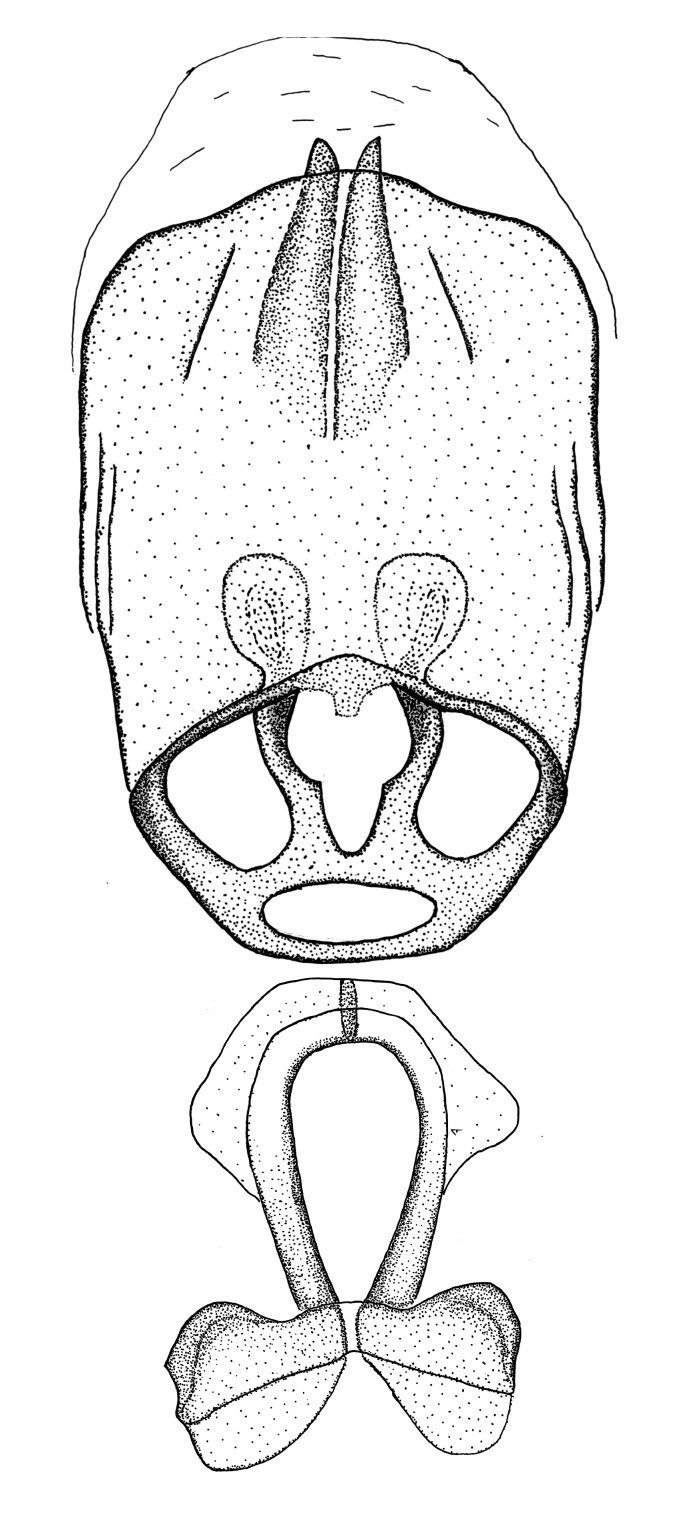


**Figure 124a.**
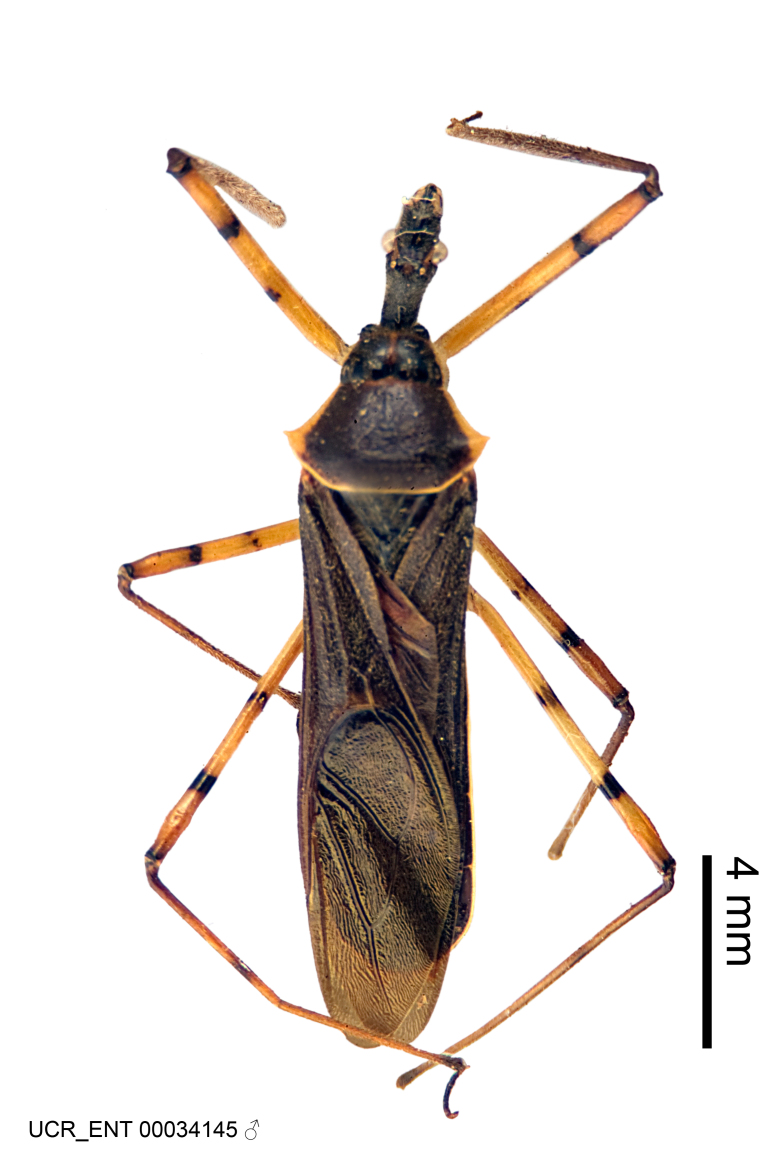
*Zelus litigiosus* Stål, 1872, male, dorsal view (UCR_ENT 00034145, Mexico, Mexico)

**Figure 124b.**
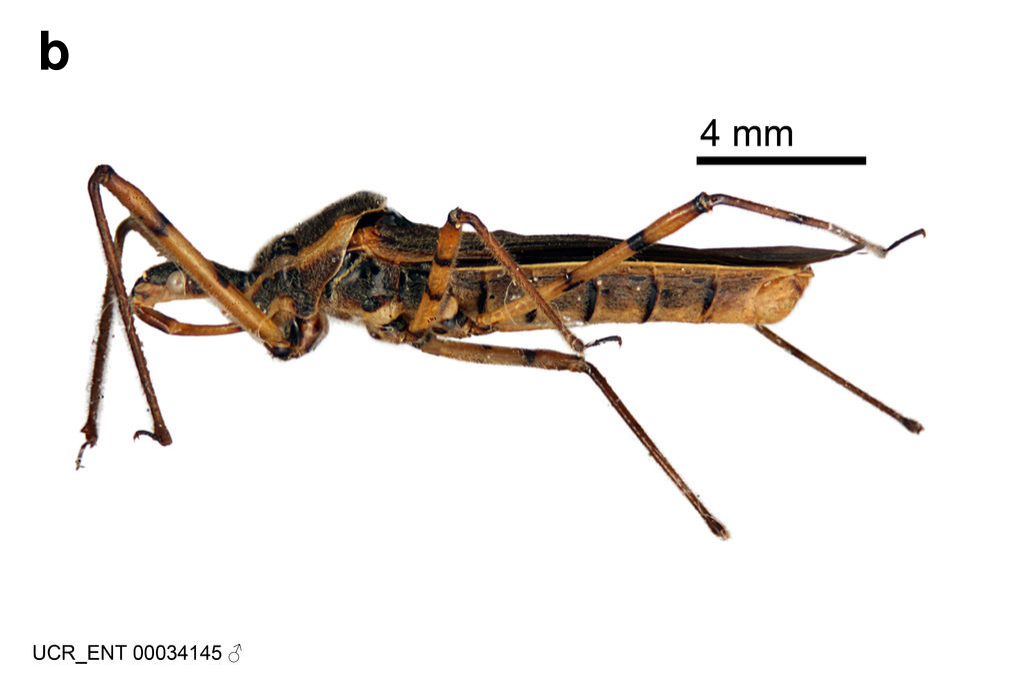
*Zelus litigiosus* Stål, 1872, male, lateral view (UCR_ENT 00034145, Mexico, Mexico)

**Figure 124c.**
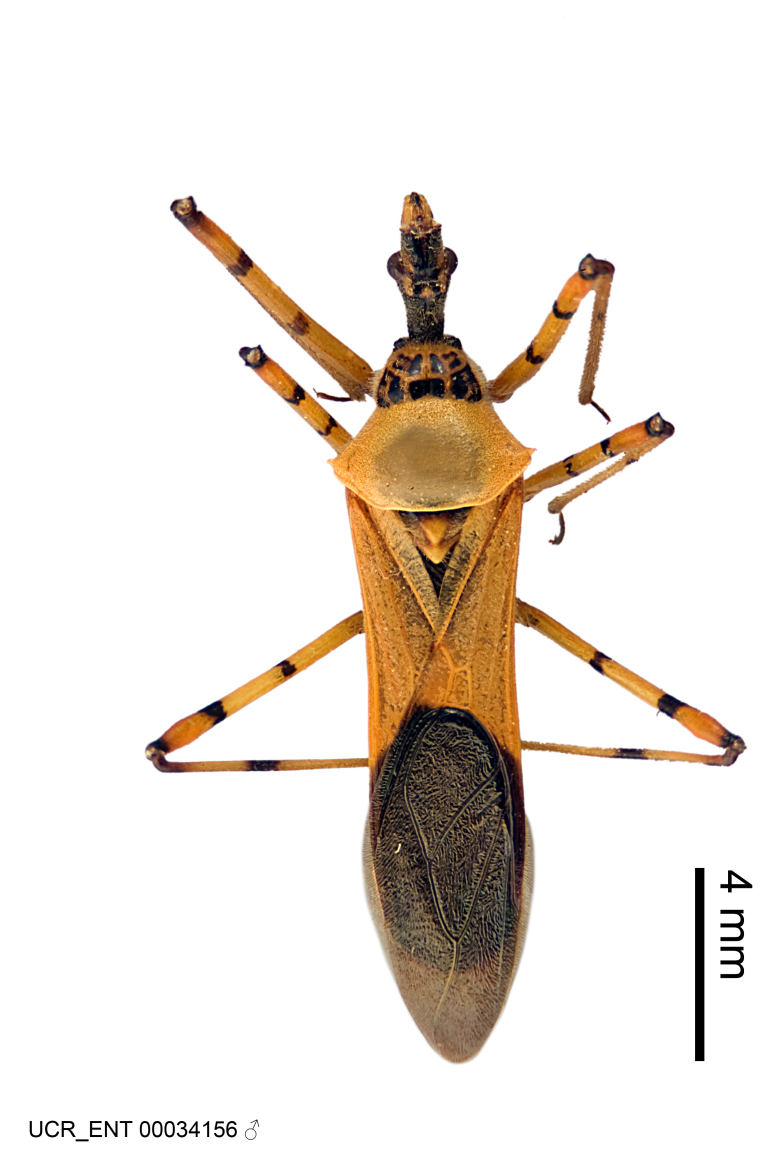
*Zelus litigiosus* Stål, 1872, male, dorsal view (UCR_ENT 00034156, Jalisco, Mexico)

**Figure 124d.**
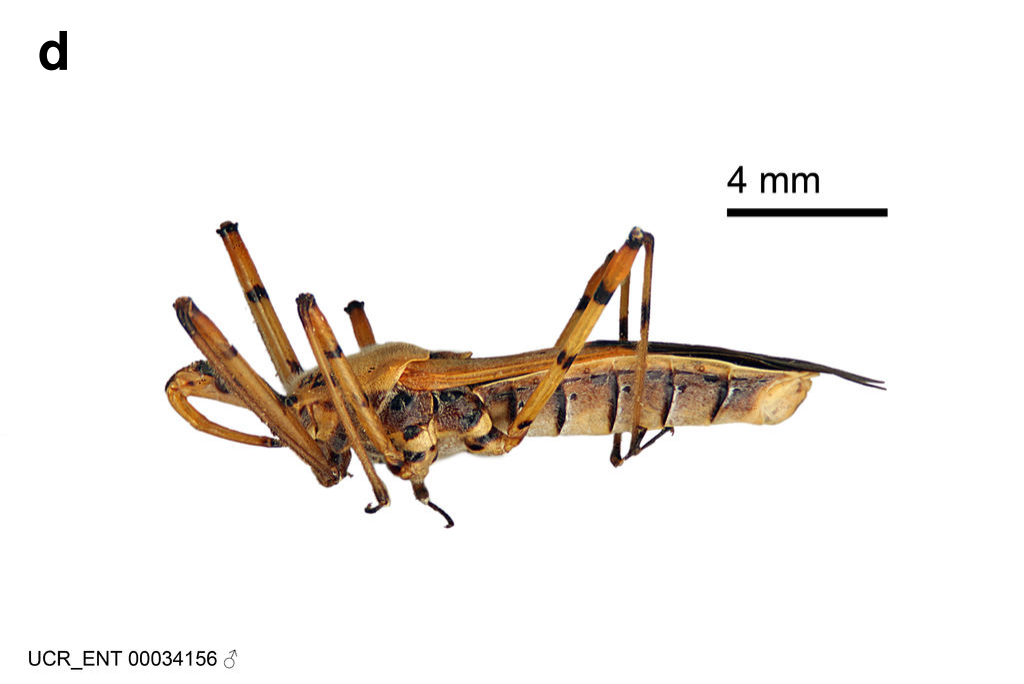
*Zelus litigiosus* Stål, 1872, male, lateral view (UCR_ENT 00034156, Jalisco, Mexico)

**Figure 124e.**
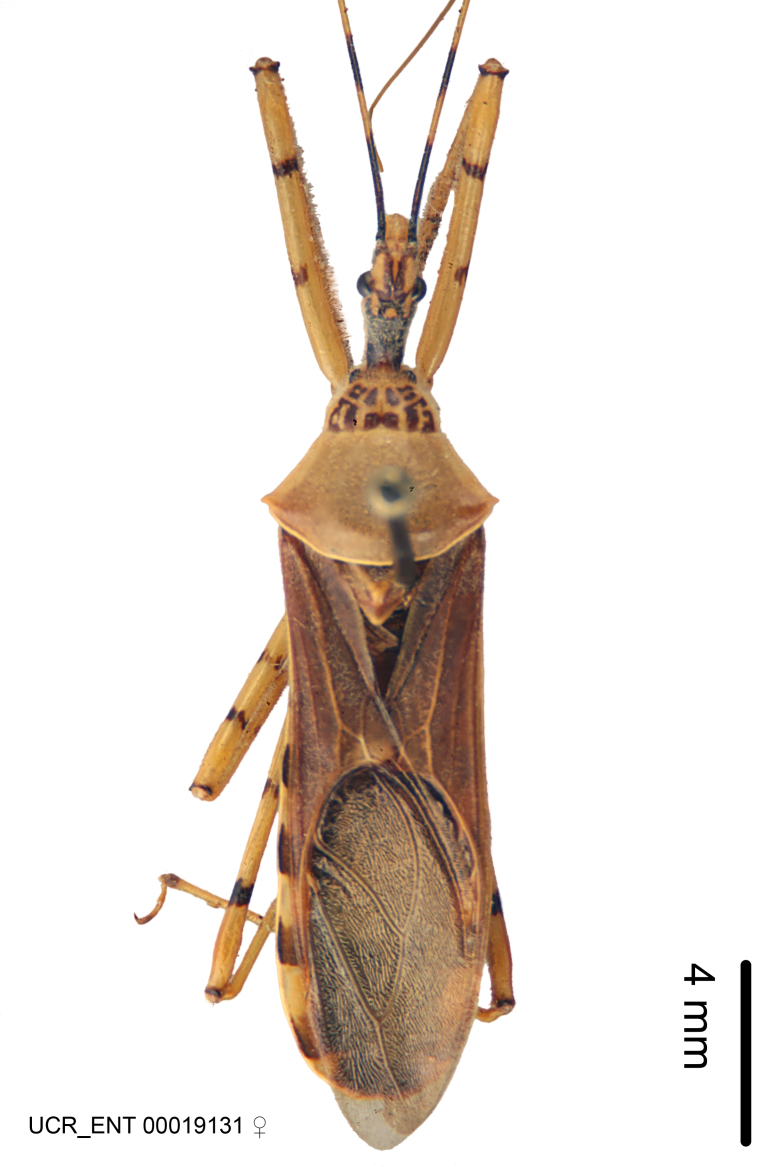
*Zelus litigiosus* Stål, 1872, female, dorsal view (UCR_ENT 00019131, Colima, Mexico)

**Figure 124f.**
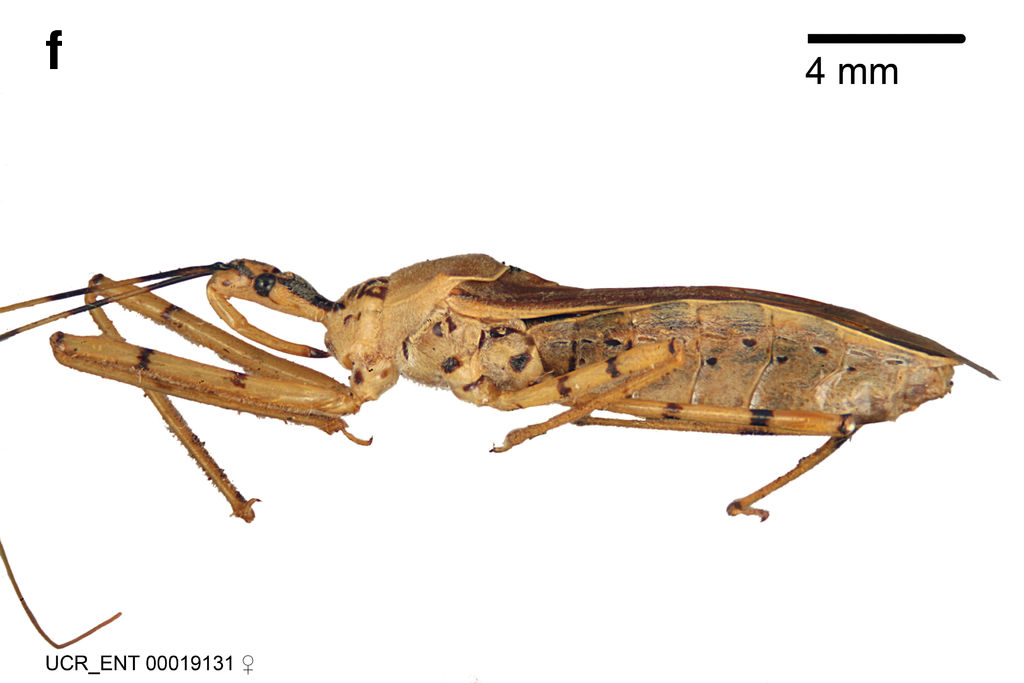
*Zelus litigiosus* Stål, 1872, female, lateral view (UCR_ENT 00019131, Colima, Mexico)

**Figure 125a.**
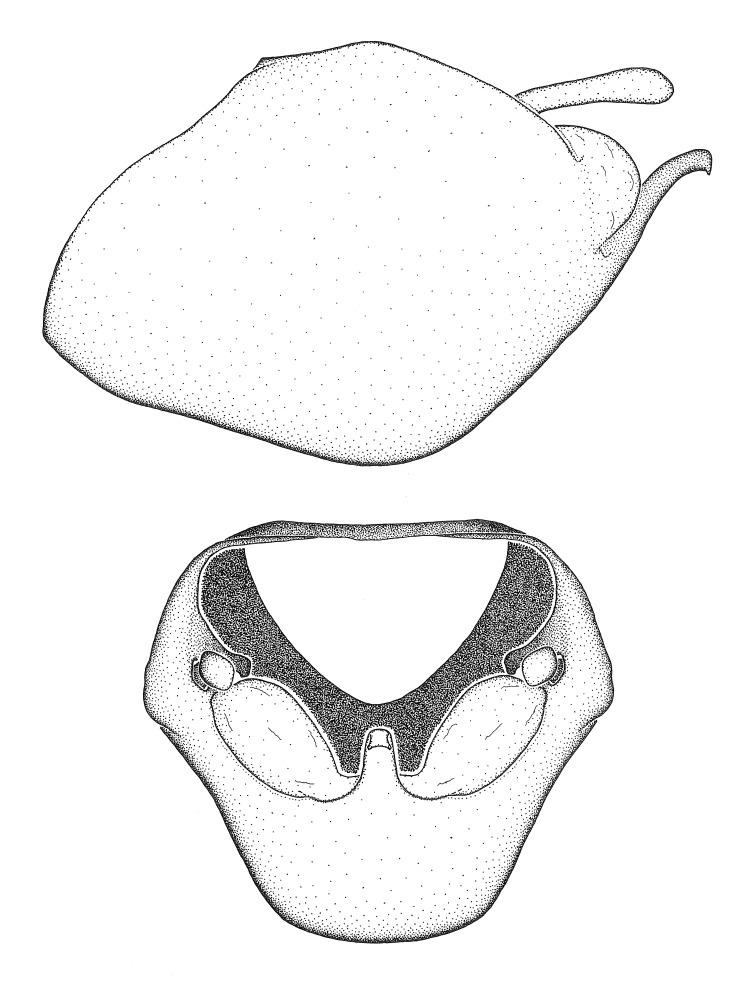
*Zelus litigiosus* Stål, 1872, pygophore, lateral and posterior views

**Figure 125b.**
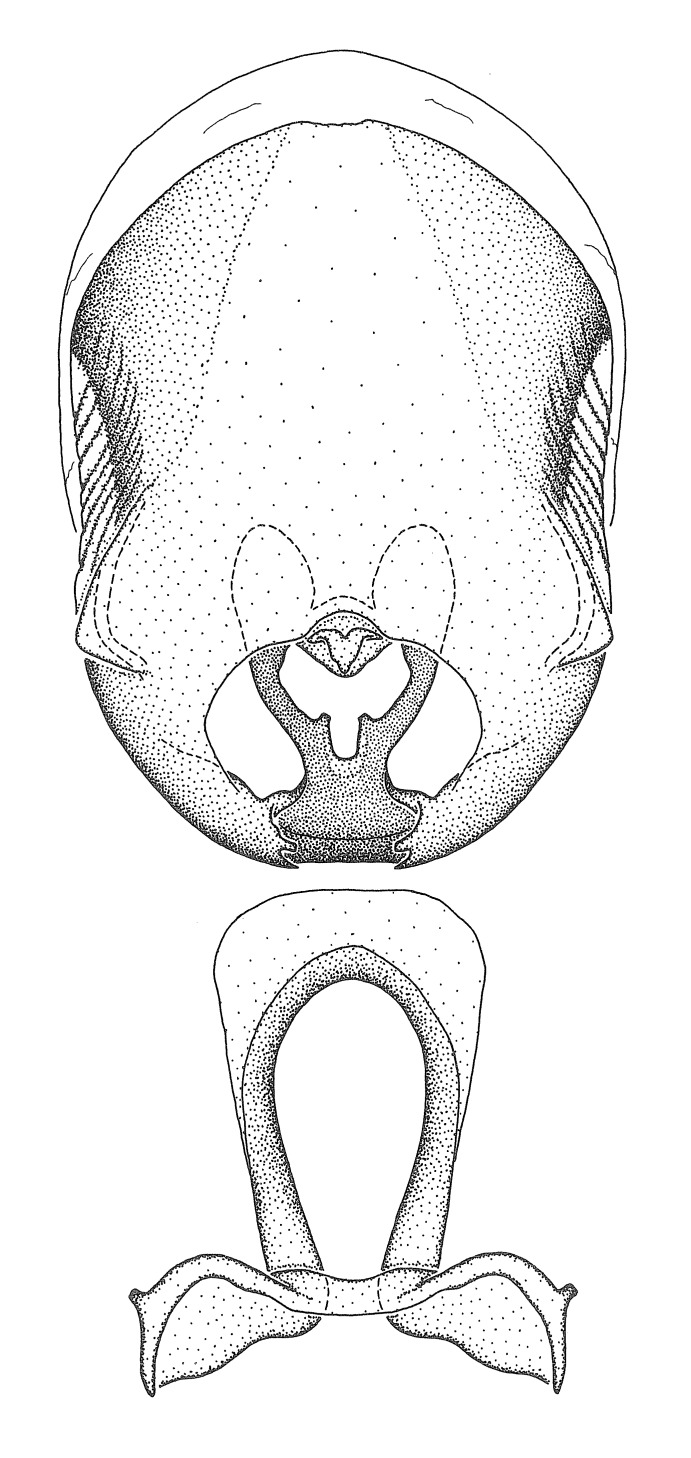
*Zelus litigiosus* Stål, 1872, phallus

**Figure 127a.**
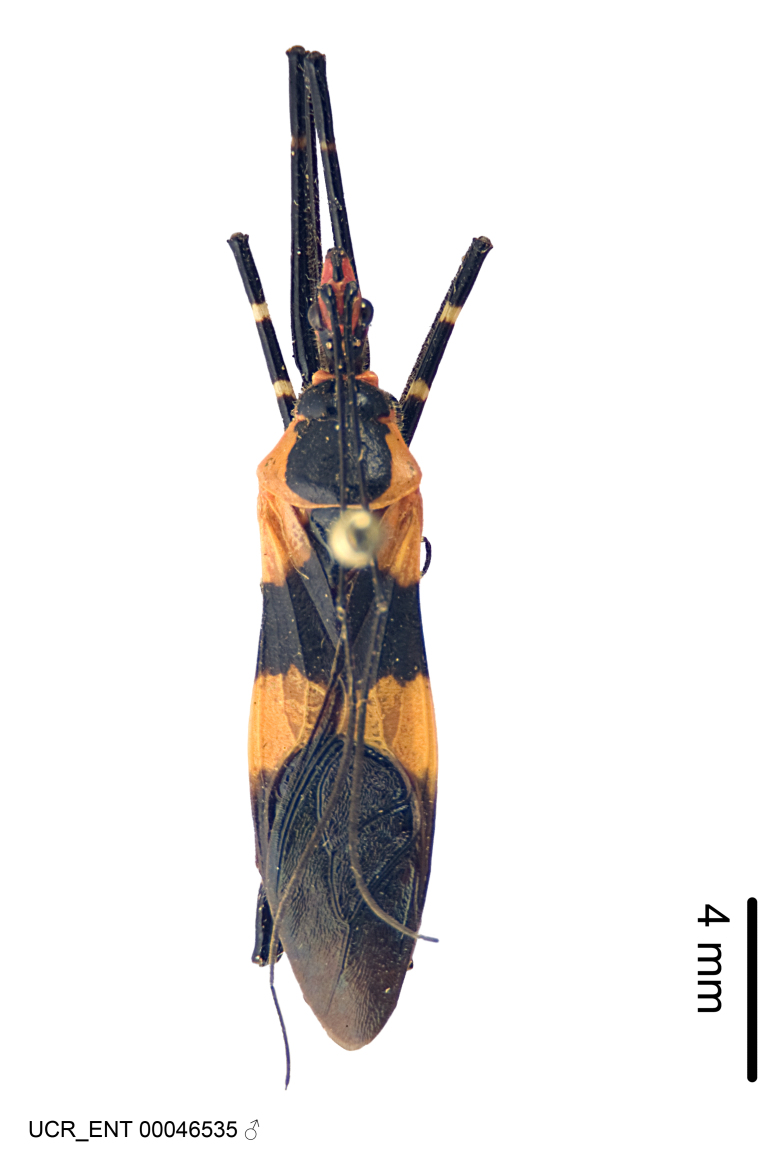
*Zelus longipes* (L., 1767), male, dorsal view (UCR_ENT 00046535, Chiapas, Mexico)

**Figure 127b.**
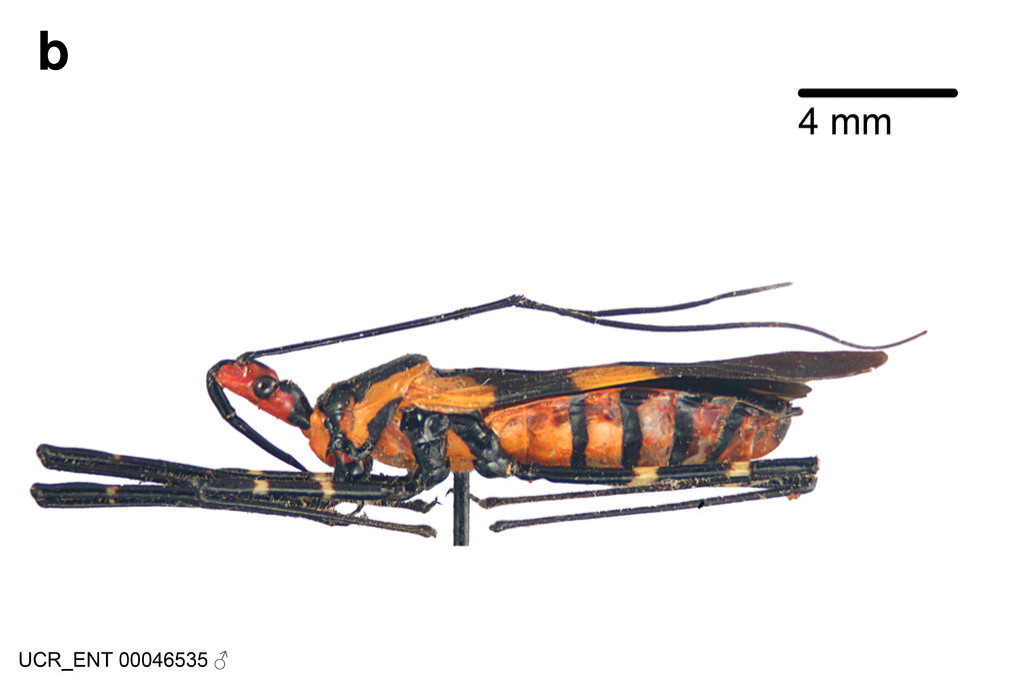
*Zelus longipes* (L., 1767), male, lateral view (UCR_ENT 00046535, Chiapas, Mexico)

**Figure 127c.**
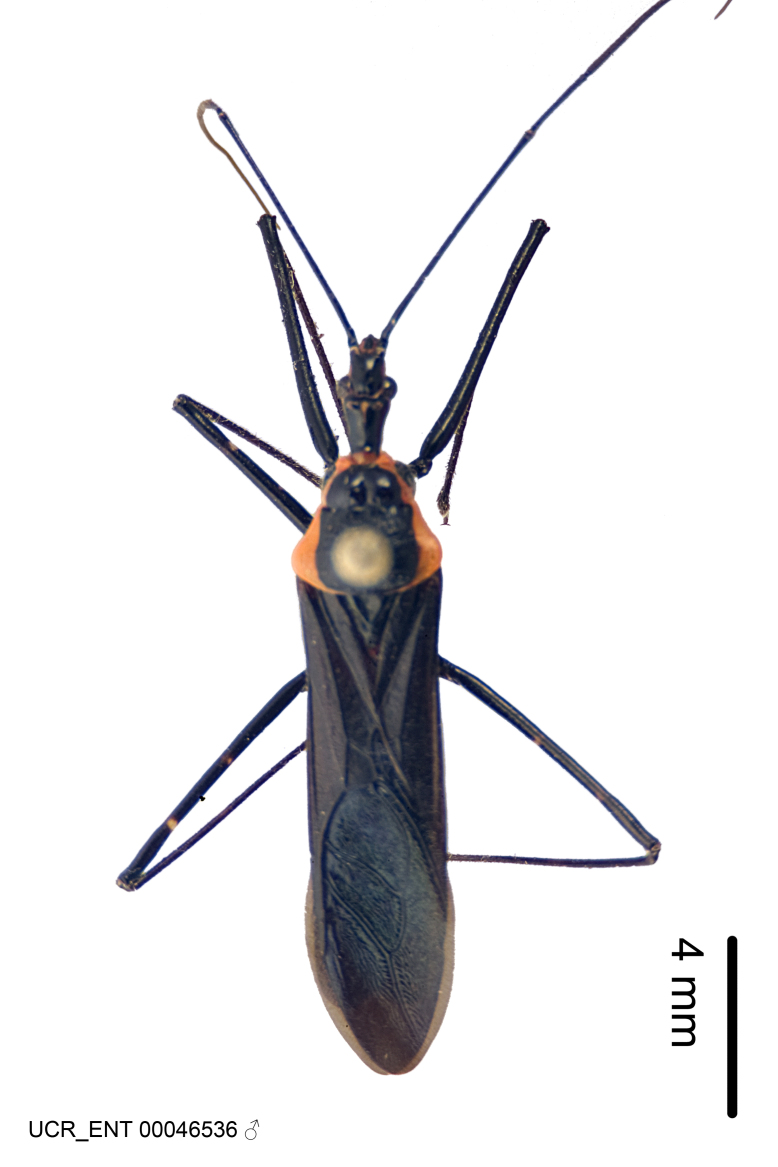
*Zelus longipes* (L., 1767), male, dorsal view (UCR_ENT 00046536, Baja California Sur, Mexico)

**Figure 127d.**
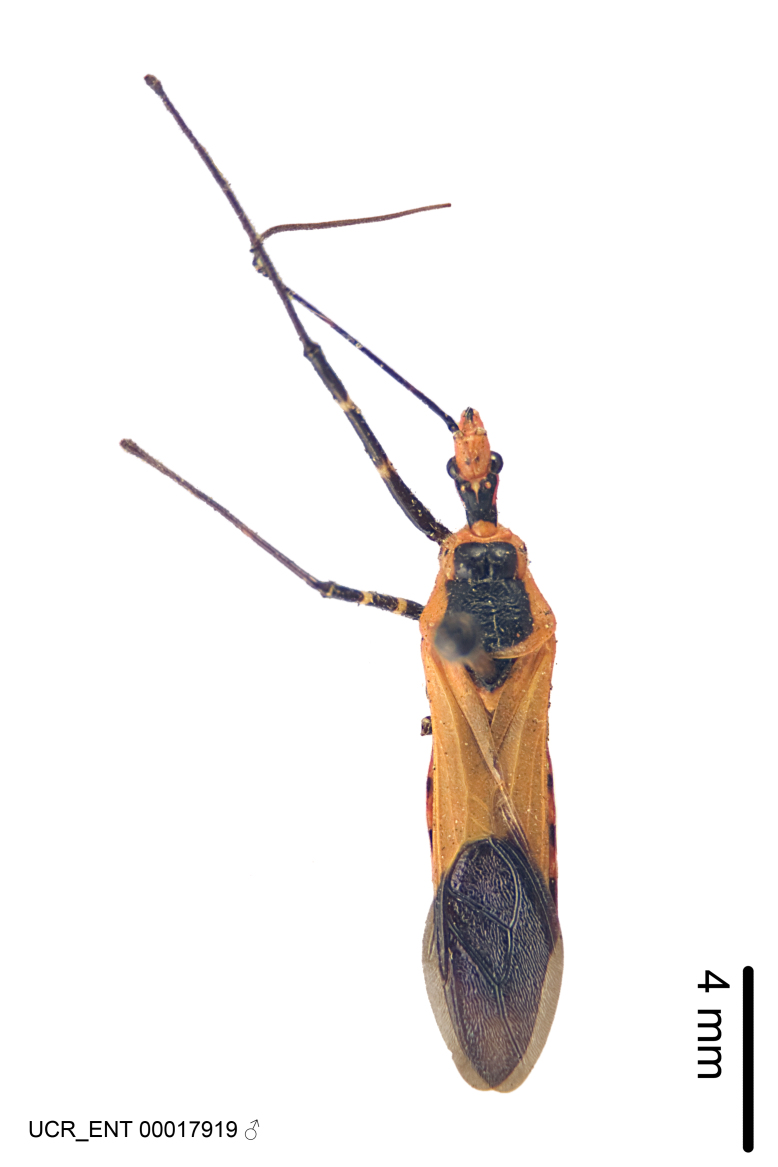
*Zelus longipes* (L., 1767), male, dorsal view (UCR_ENT 00017919, Copan, Honduras)

**Figure 127e.**
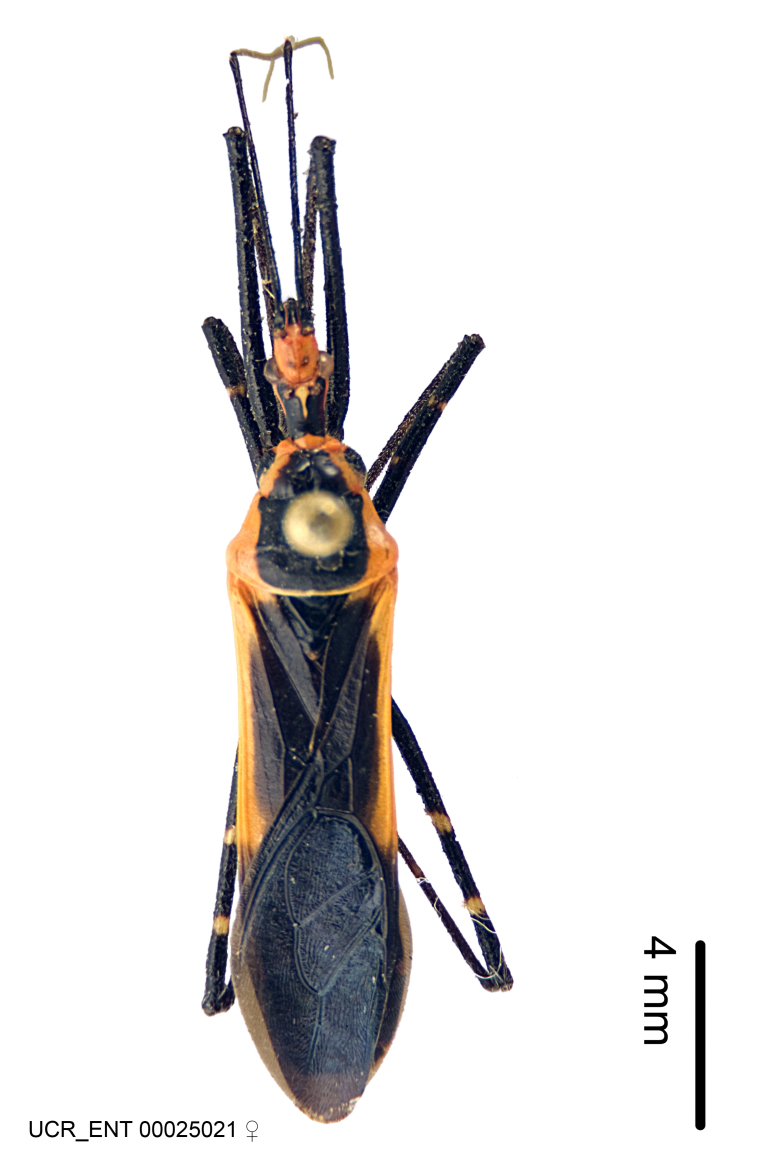
*Zelus longipes* (L., 1767), female, dorsal view (UCR_ENT 00025021, Baja California, Mexico)

**Figure 127f.**
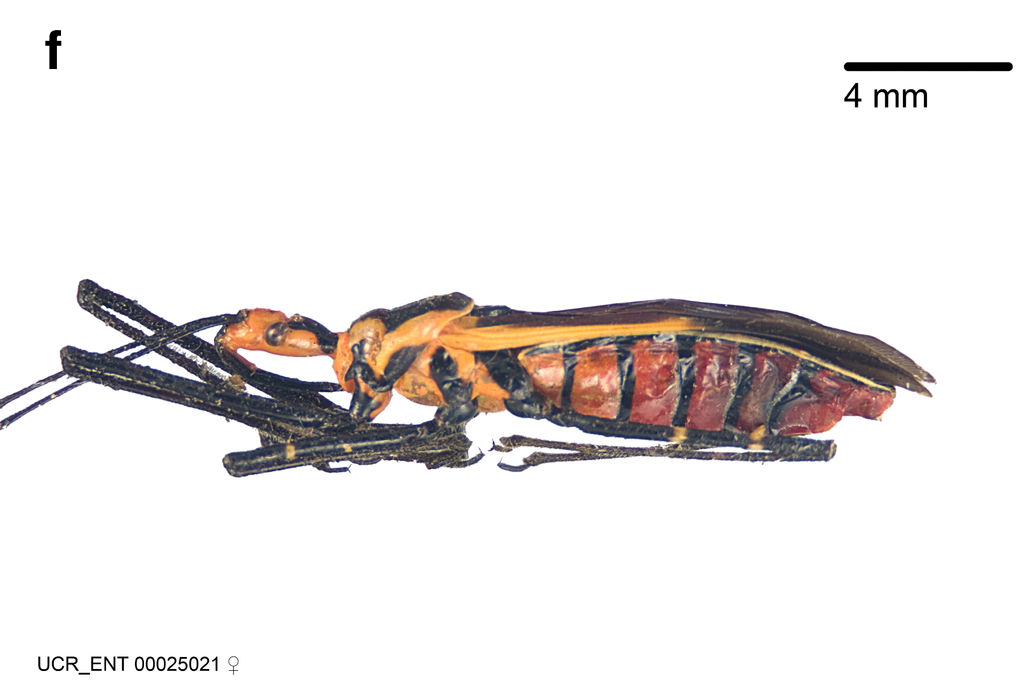
*Zelus longipes* (L., 1767), female, lateral view (UCR_ENT 00025021, Baja California, Mexico)

**Figure 128a.**
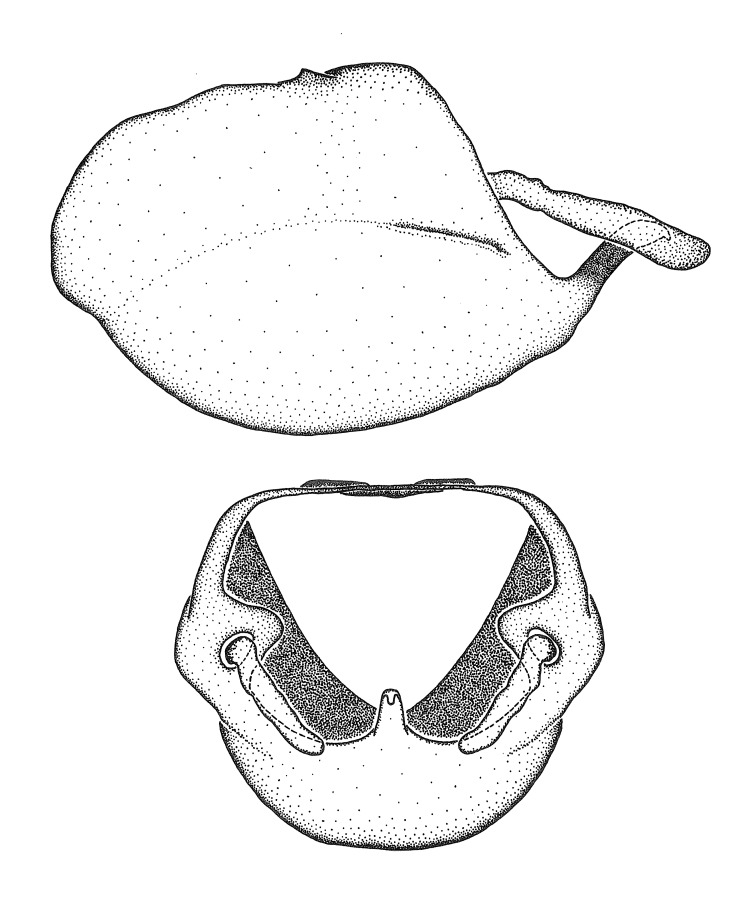
*Zelus longipes* (L., 1767), pygophore, lateral and posterior views

**Figure 128b.**
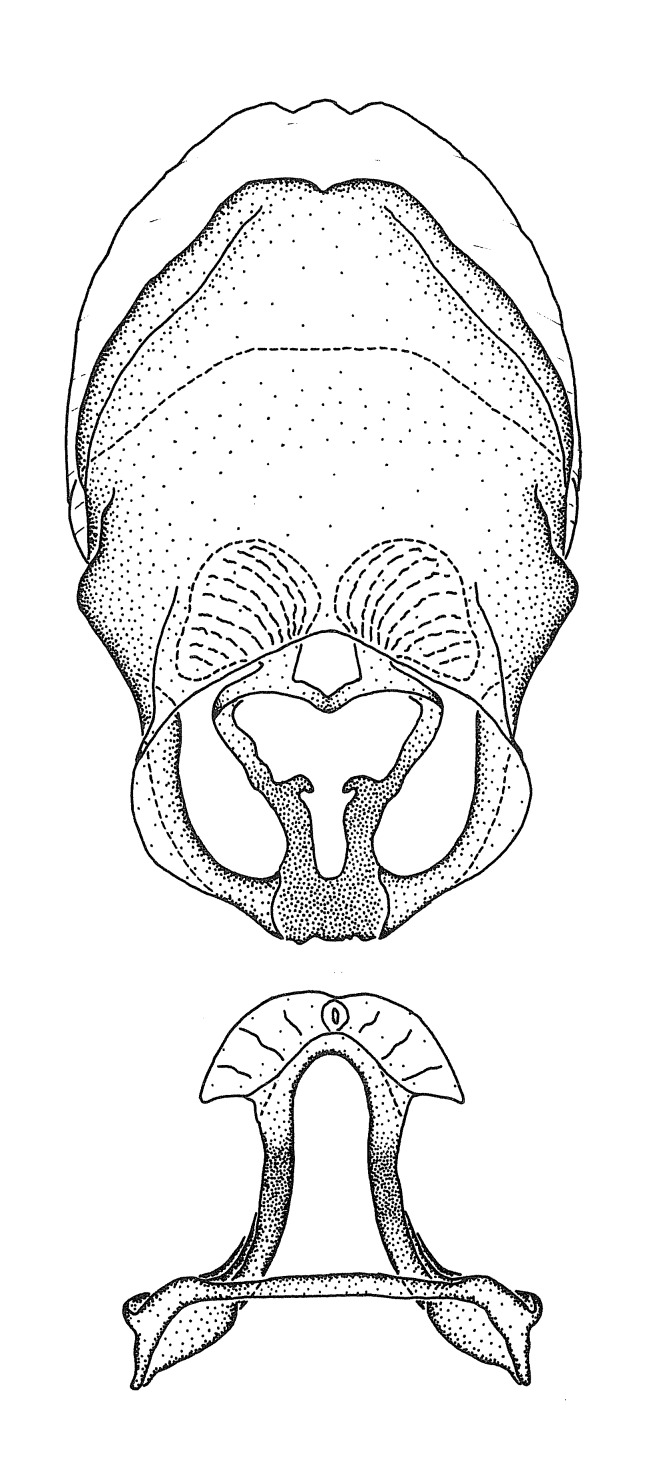
*Zelus longipes* (L., 1767), phallus, dorsal view

**Figure 130a.**
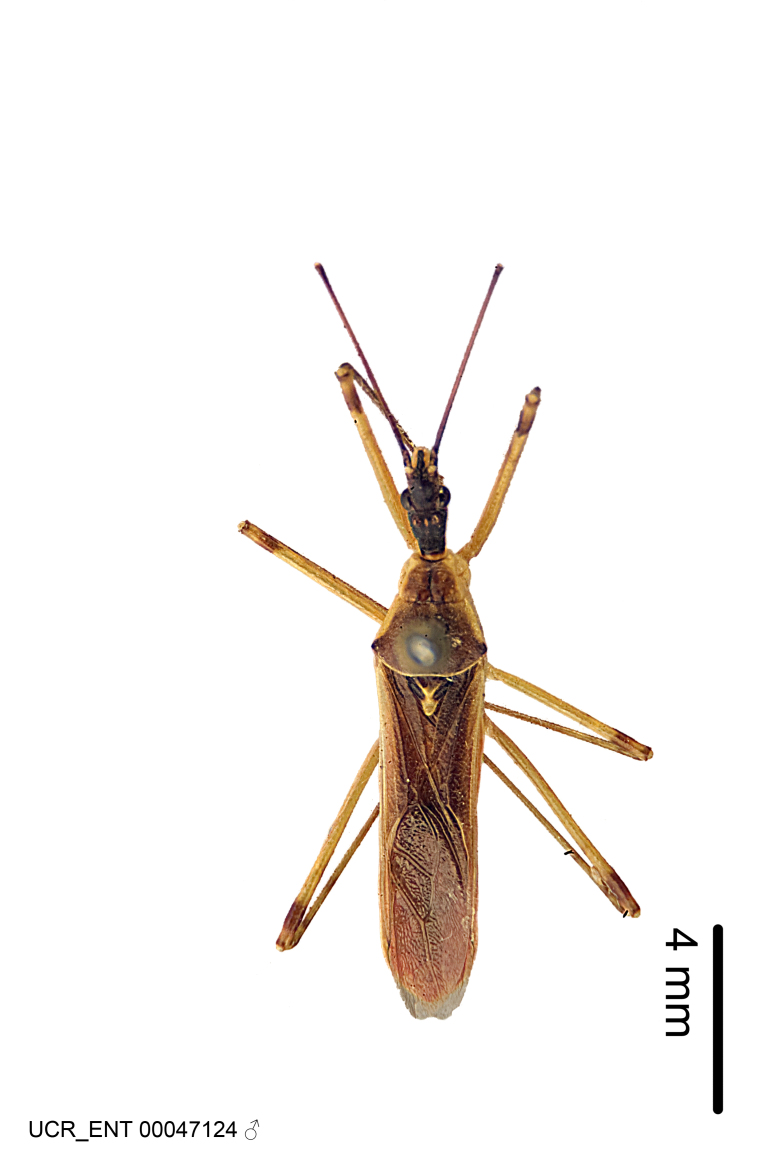
*Zelus luridus* Stål, 1862, male, dorsal view (UCR_ENT 00047124, Iowa, USA)

**Figure 130b.**
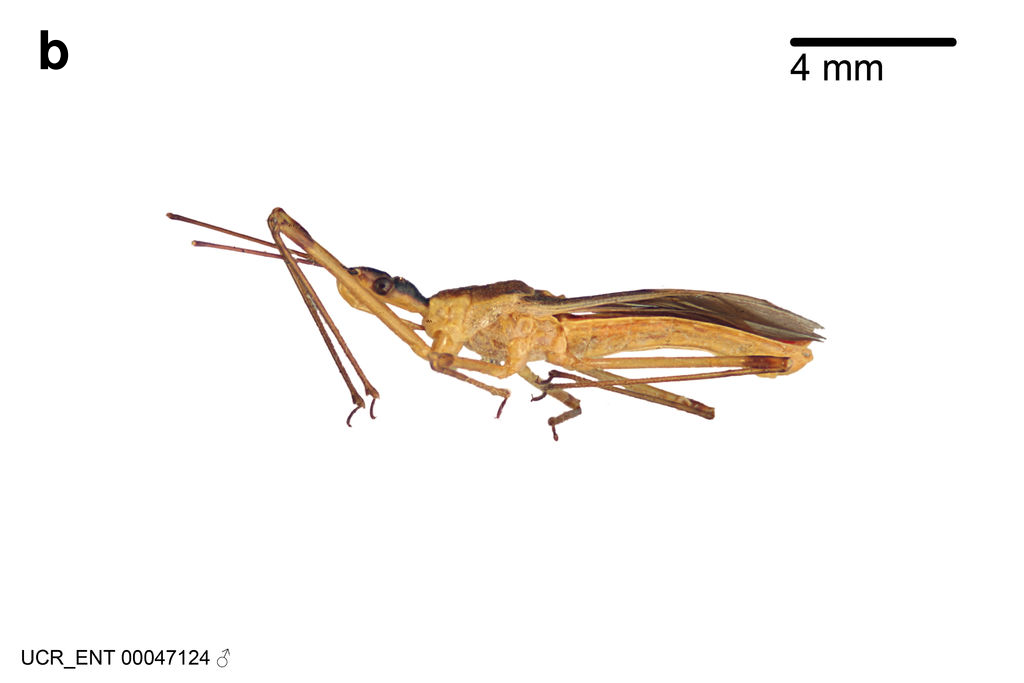
*Zelus luridus* Stål, 1862, male, lateral view (UCR_ENT 00047124, Iowa, USA)

**Figure 130c.**
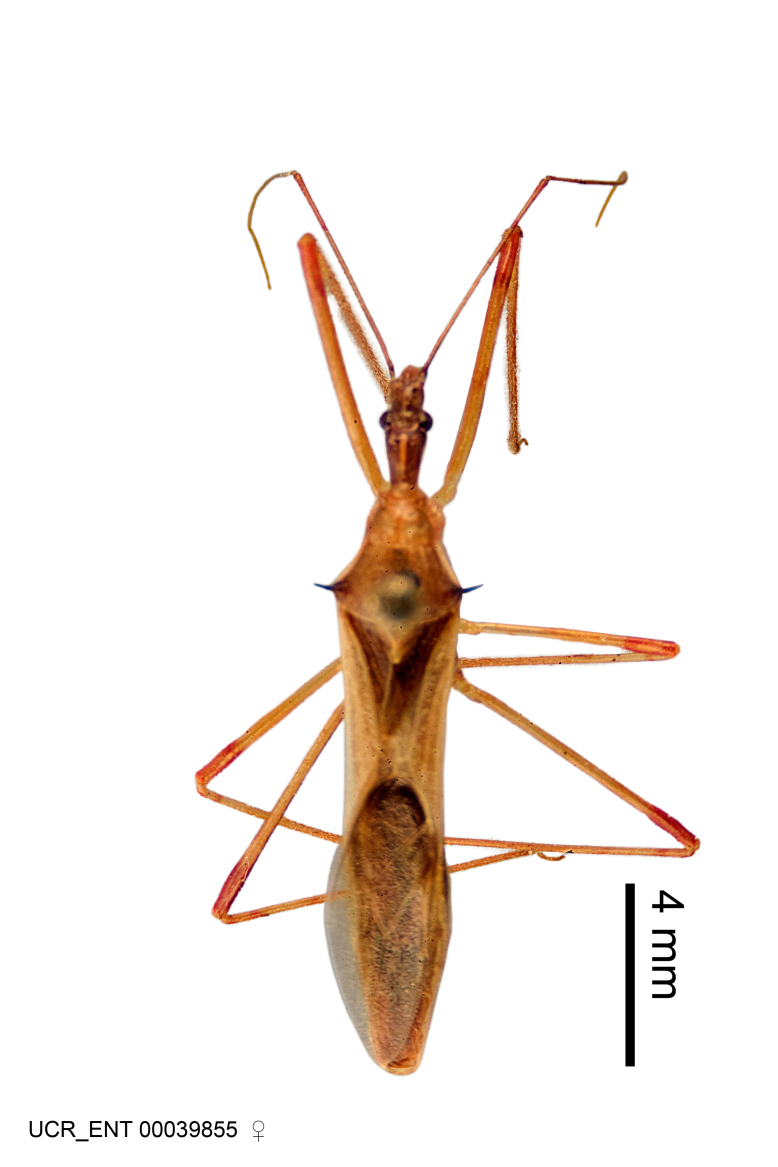
*Zelus luridus* Stål, 1862, female, dorsal view (UCR_ENT 00039855, Texas, USA)

**Figure 130d.**
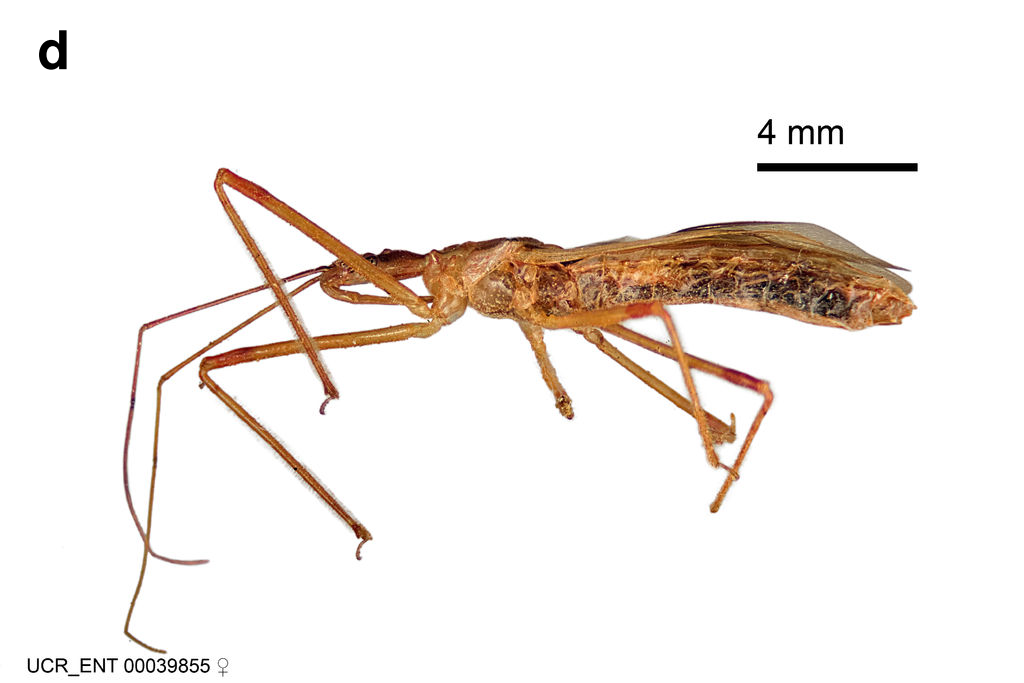
*Zelus luridus* Stål, 1862, female, lateral view (UCR_ENT 00039855, Texas, USA)

**Figure 131a.**
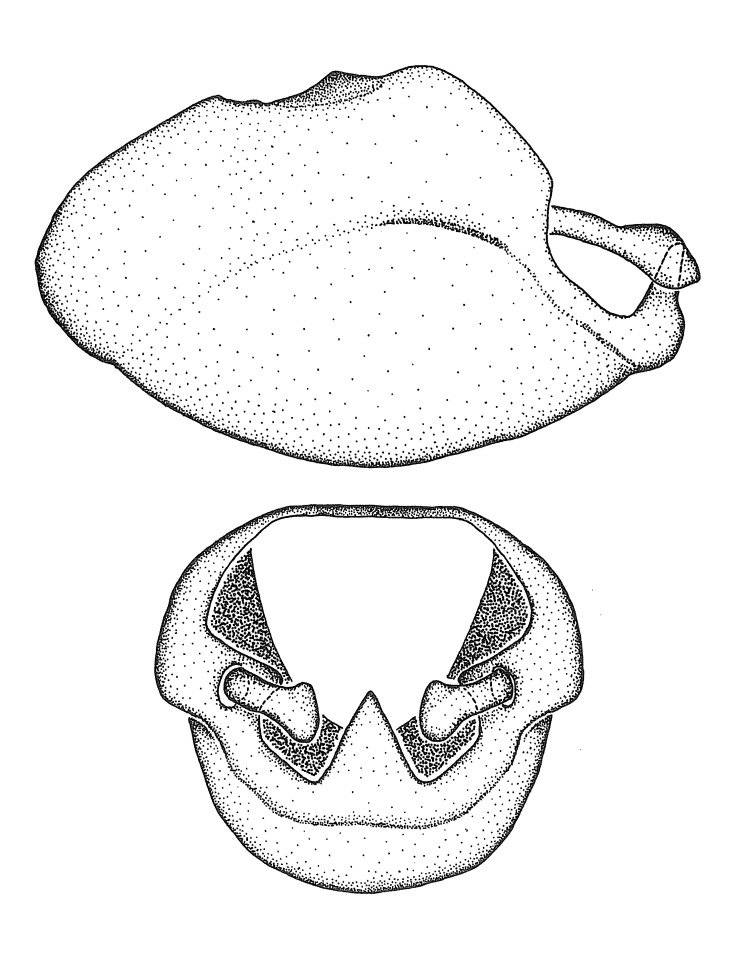
*Zelus luridus* Stål, 1862, eastern population, pygophore, lateral and posterior views

**Figure 131b.**
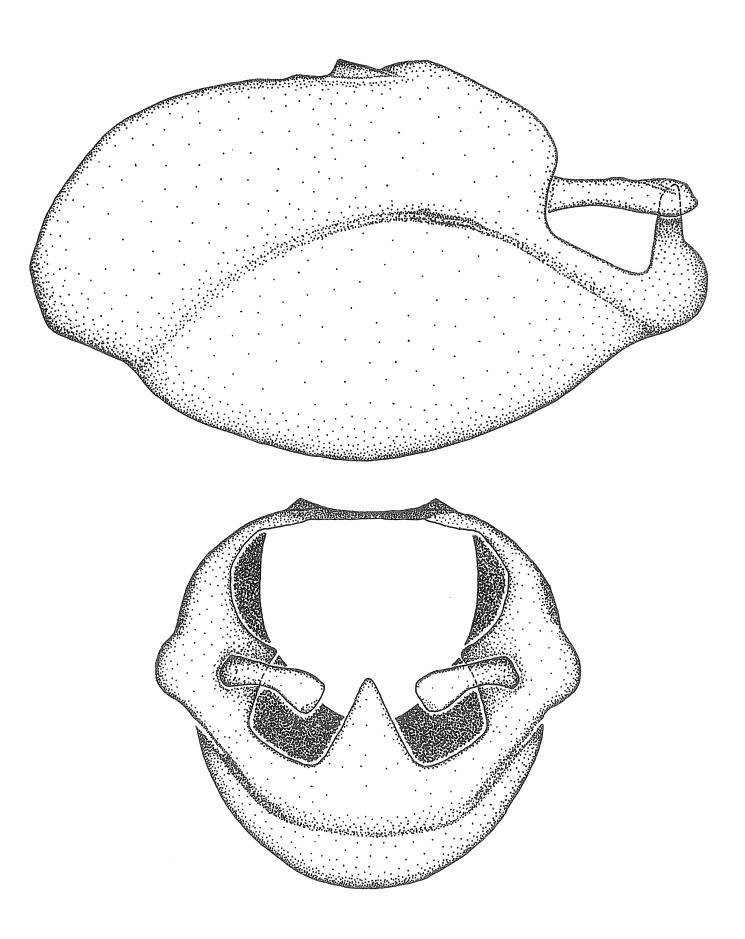
*Zelus luridus* Stål, 1862, western population, pygophore, lateral and posterior views

**Figure 131c.**
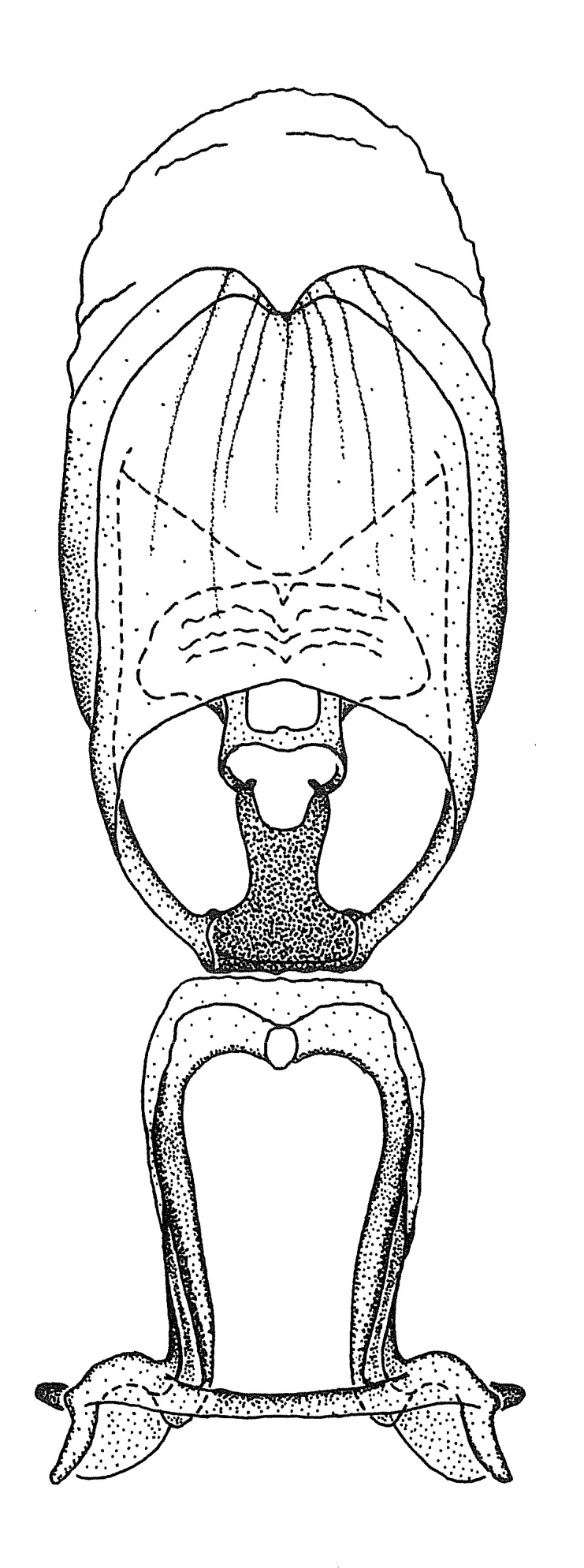
*Zelus luridus* Stål, 1862, eastern population, phallus, dorsal view

**Figure 131d.**
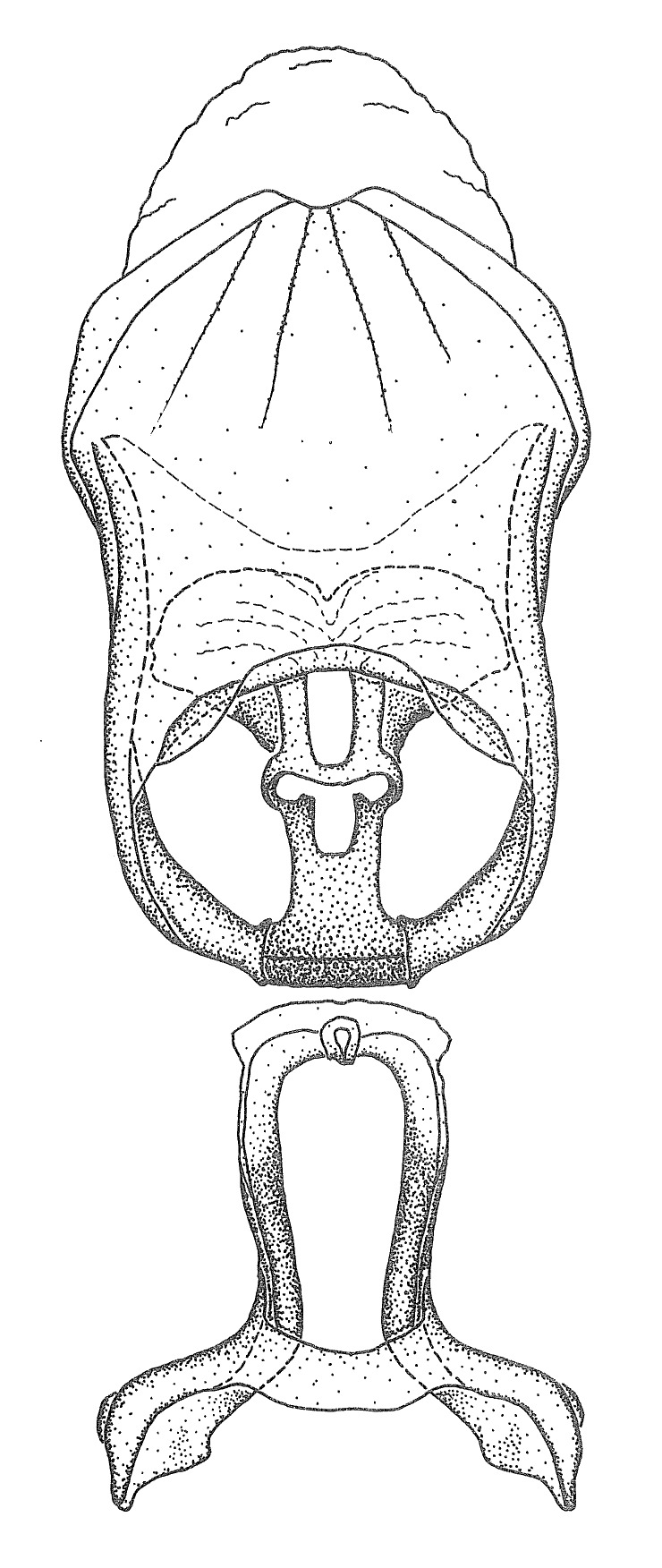
*Zelus luridus* Stål, 1862, western population, phallus, dorsal view

**Figure 133a.**
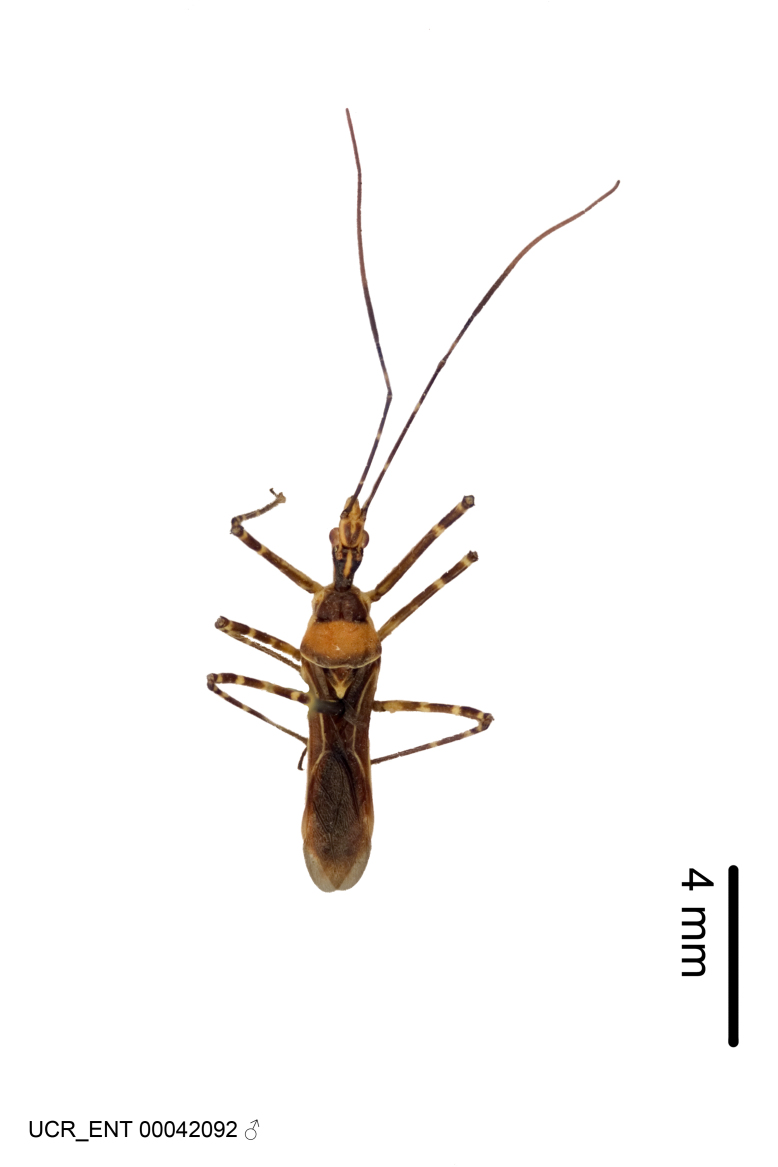


**Figure 133b.**
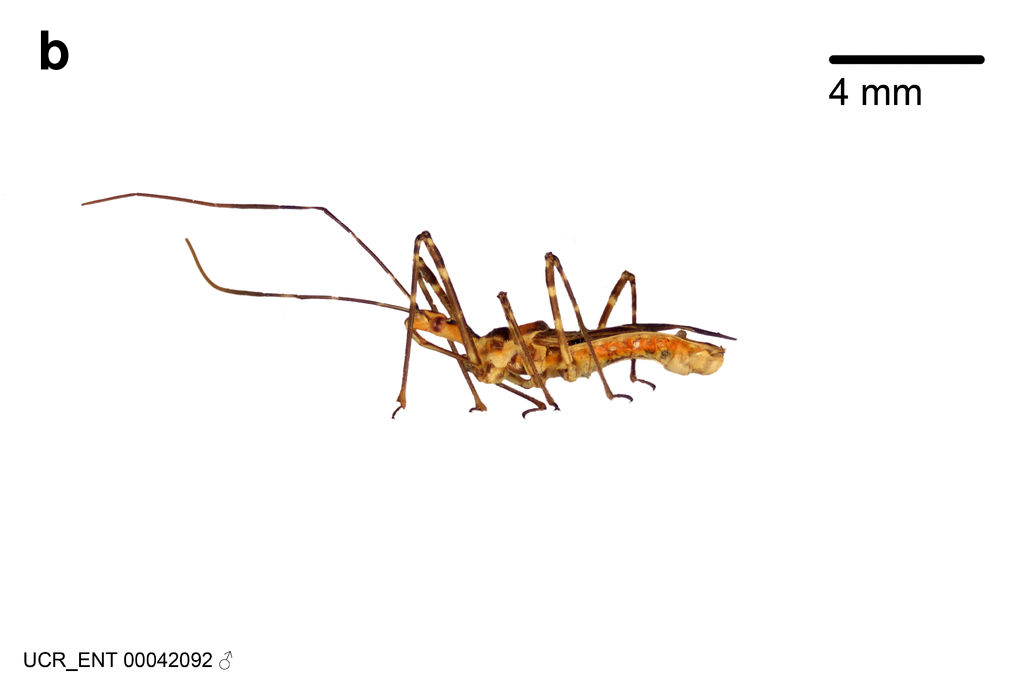


**Figure 133c.**
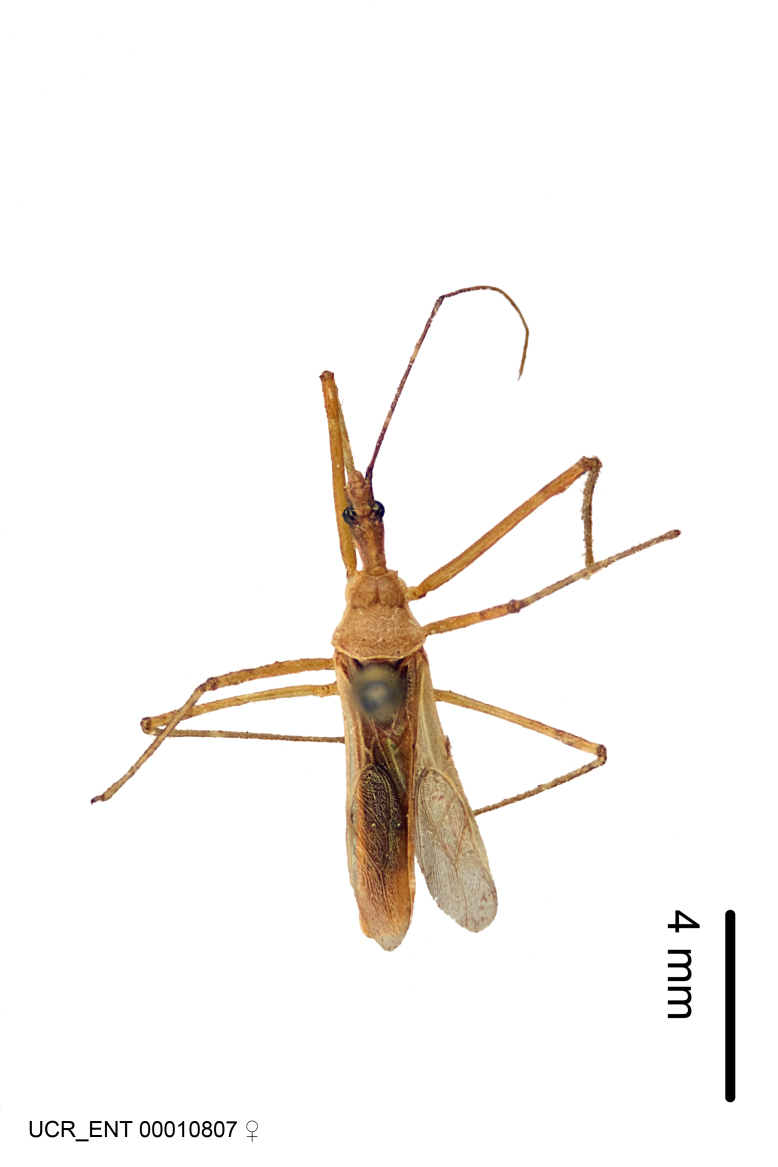


**Figure 133d.**
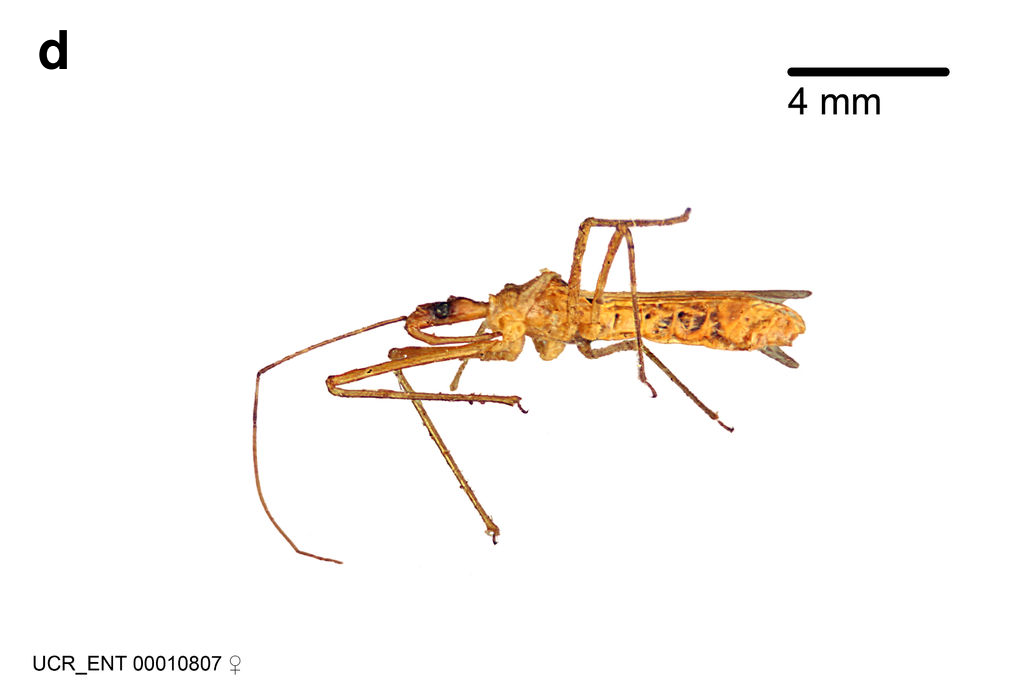


**Figure 134a.**
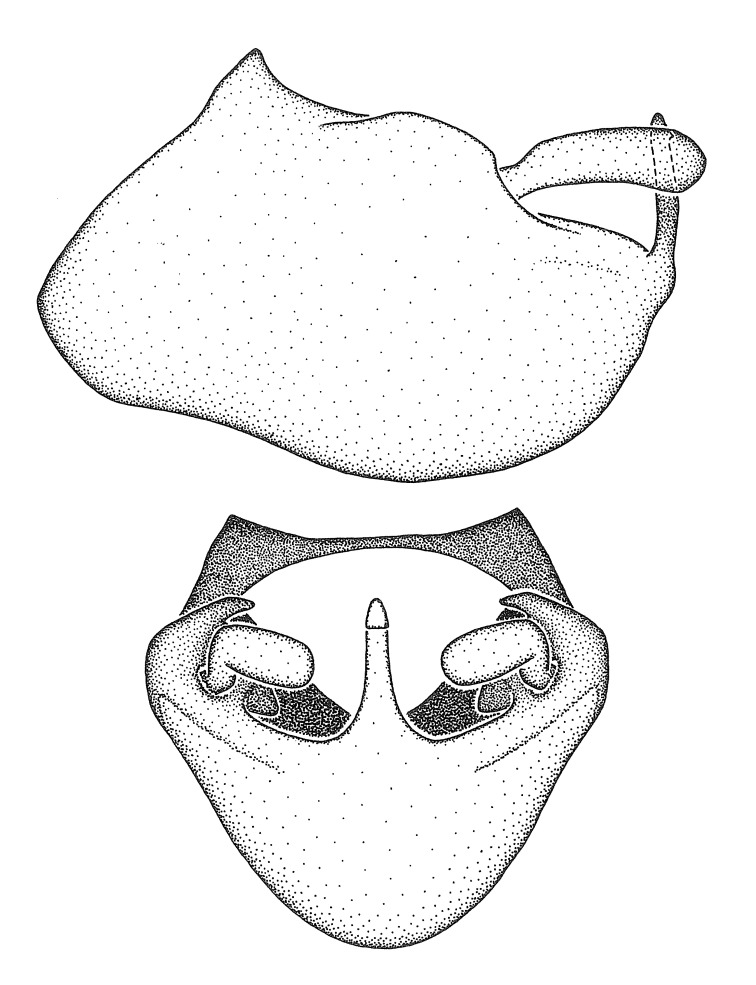
*Zelus mattogrossensis* Wygodzinsky, 1947, pygophore, lateral and posterior views

**Figure 134b.**
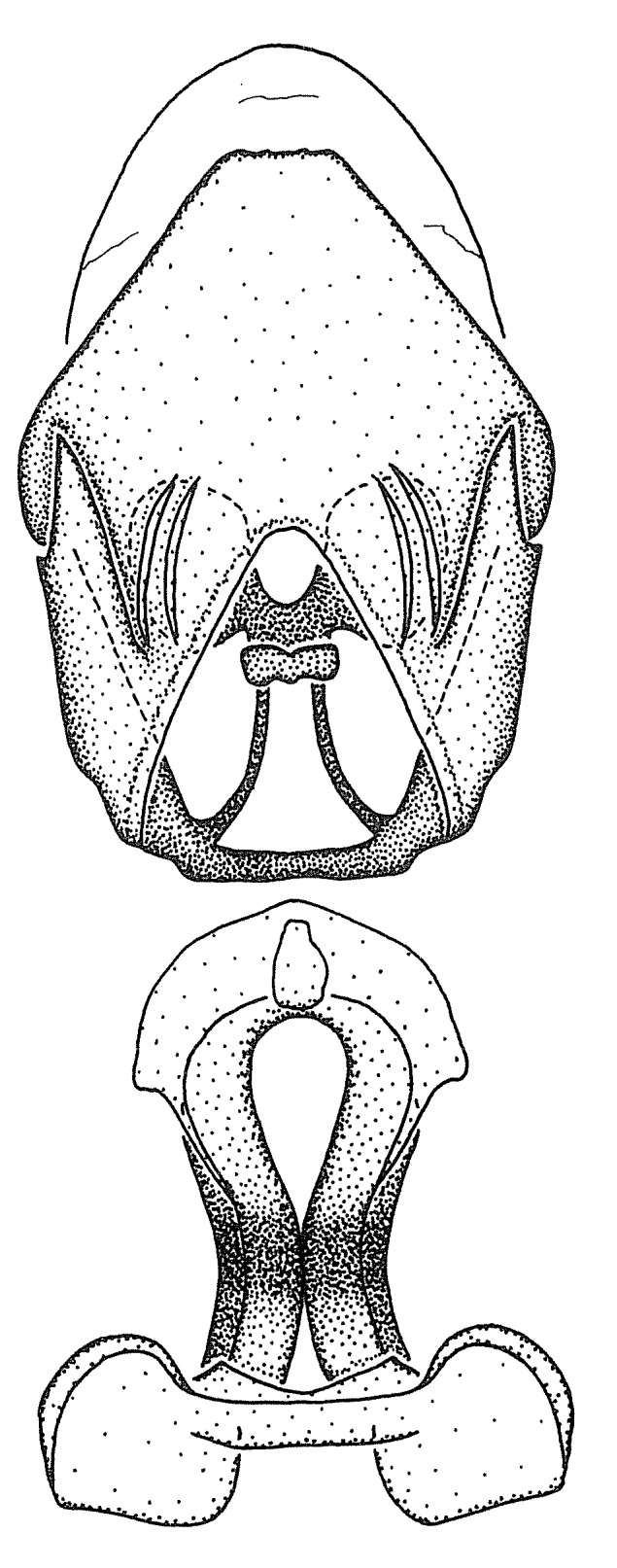
*Zelus mattogrossensis* Wygodzinsky, 1947, phallus, dorsal view

**Figure 136a.**
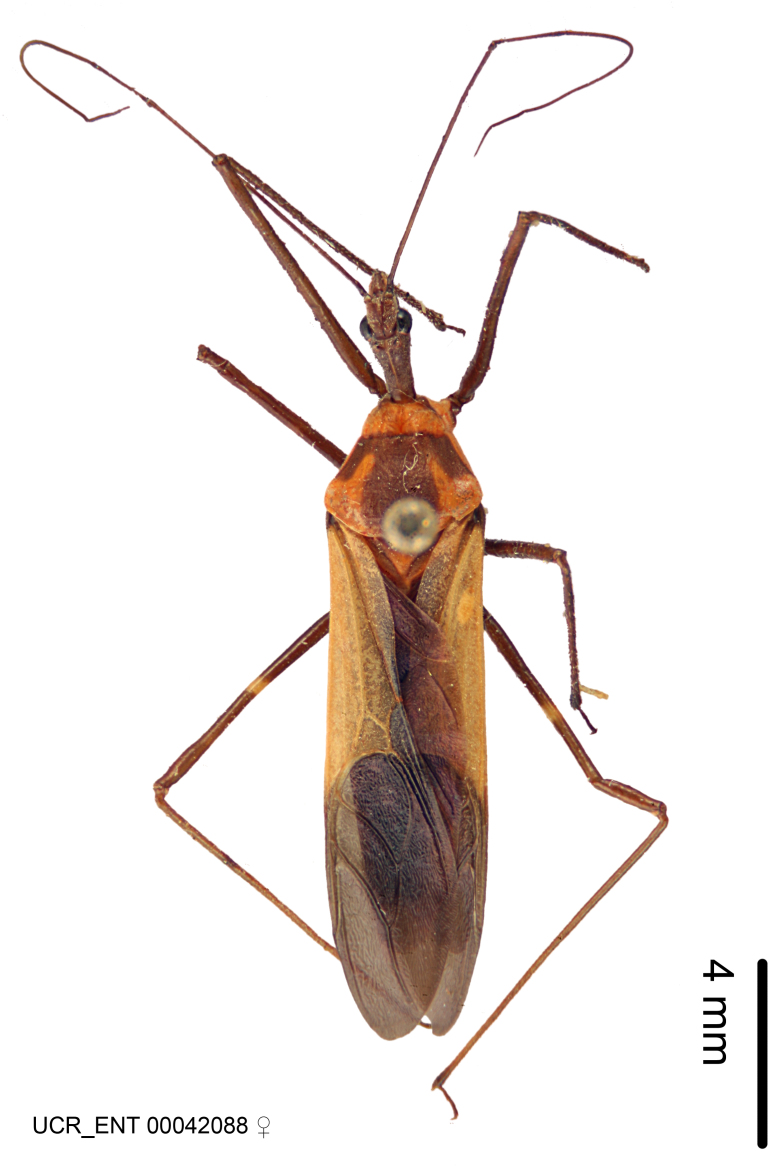
*Zelus means* Fabricius, 1803, female, dorsal view (UCR_ENT 00042088, Colombia)

**Figure 136b.**
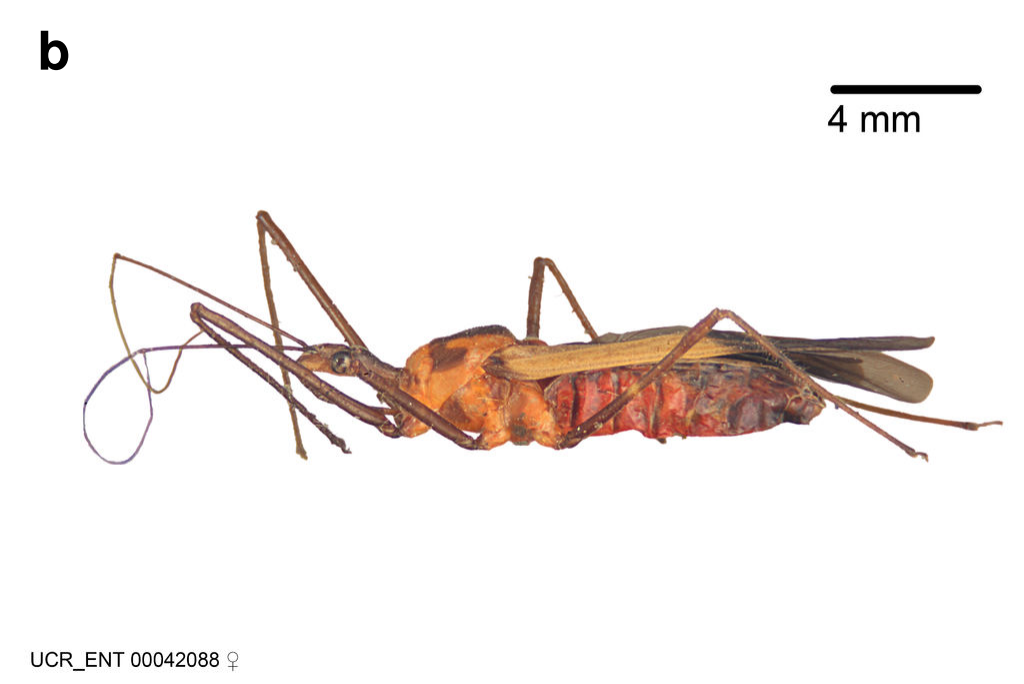
*Zelus means* Fabricius, 1803, female, lateral view (UCR_ENT 00042088, Colombia)

**Figure 136c.**
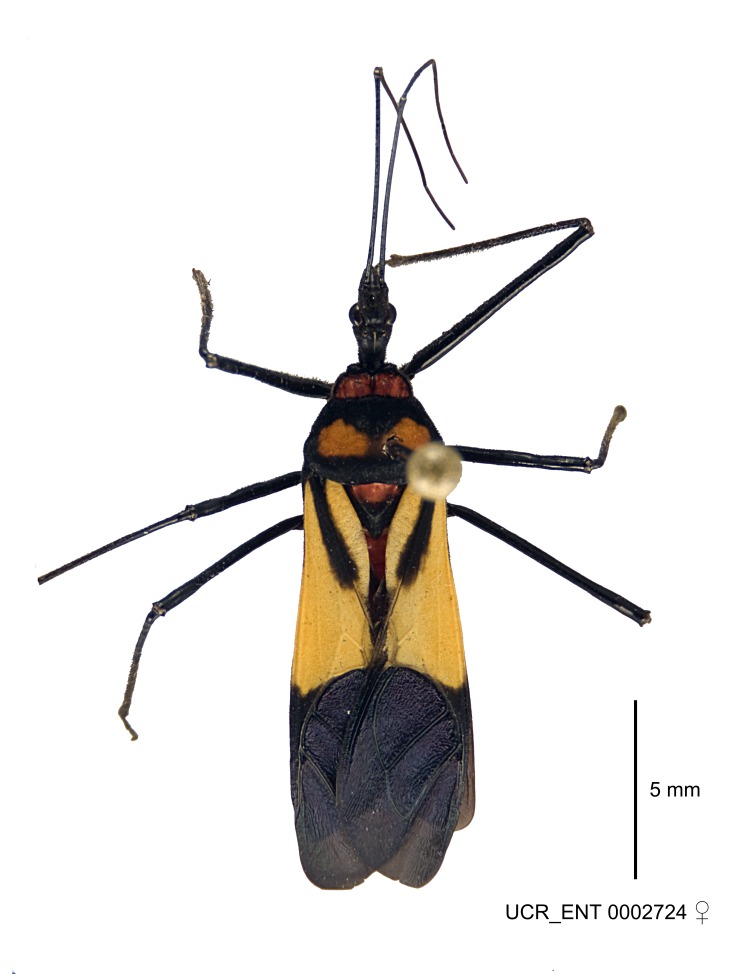
*Zelus means* Fabricius, 1803, female, dorsal view (UCR_ENT 00002724, Ecuador)

**Figure 136d.**
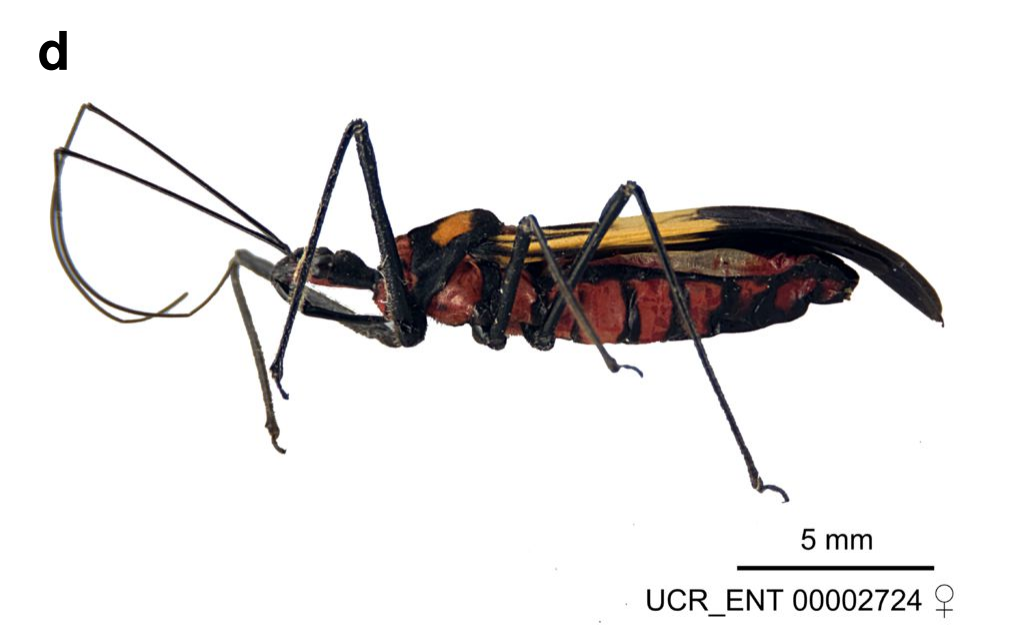
*Zelus means* Fabricius, 1803, female, lateral view (UCR_ENT 00002724, Ecuador)

**Figure 136e.**
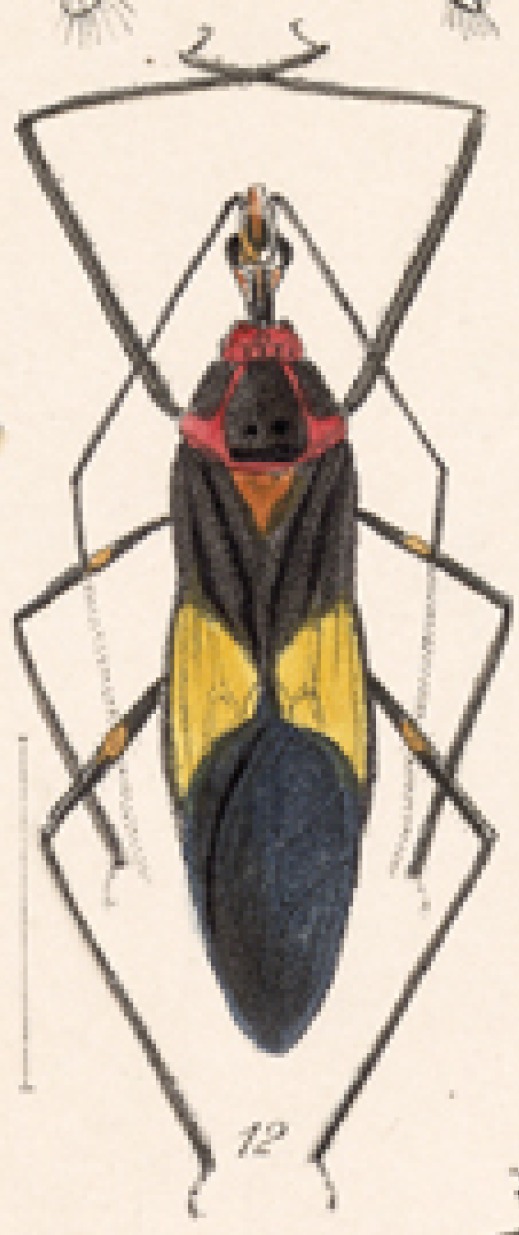
*Zelus means* Fabricius, 1803, female, dorsal view (adapted from Champion, 1898; as *Zelus trimaculatus*)

**Figure 136f.**
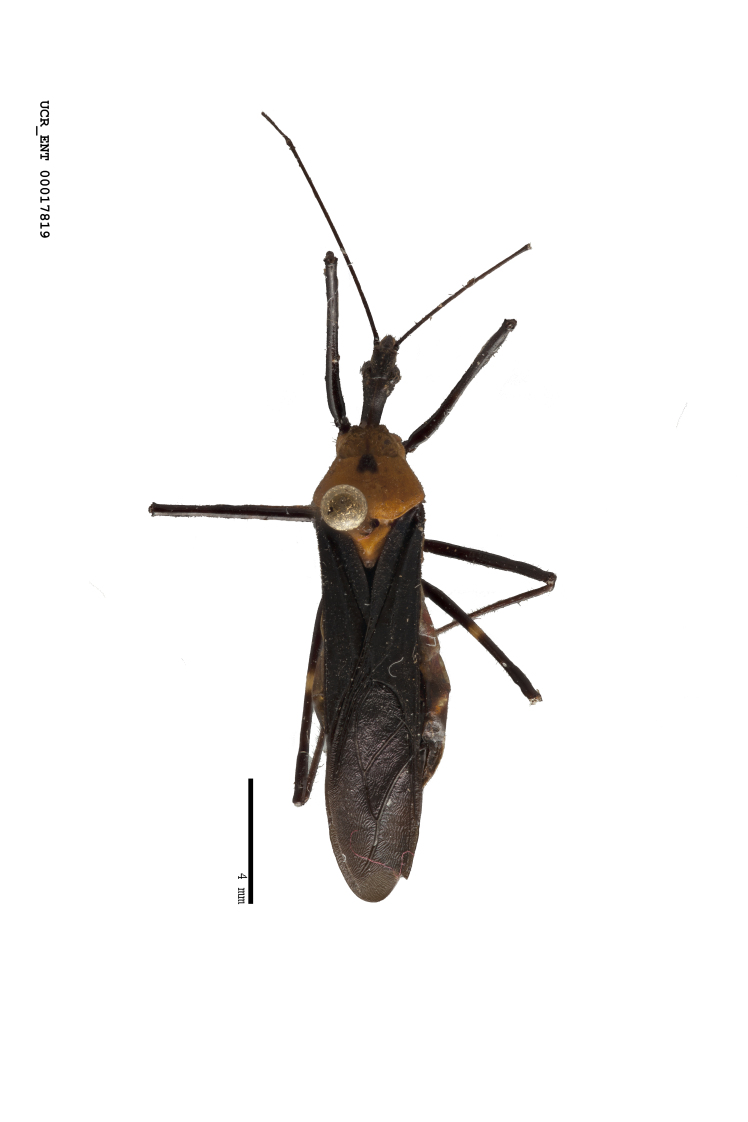
*Zelus means* Fabricius, 1803, female, dorsal view (UCR_ENT 00017819, Brazil)

**Figure 138a.**
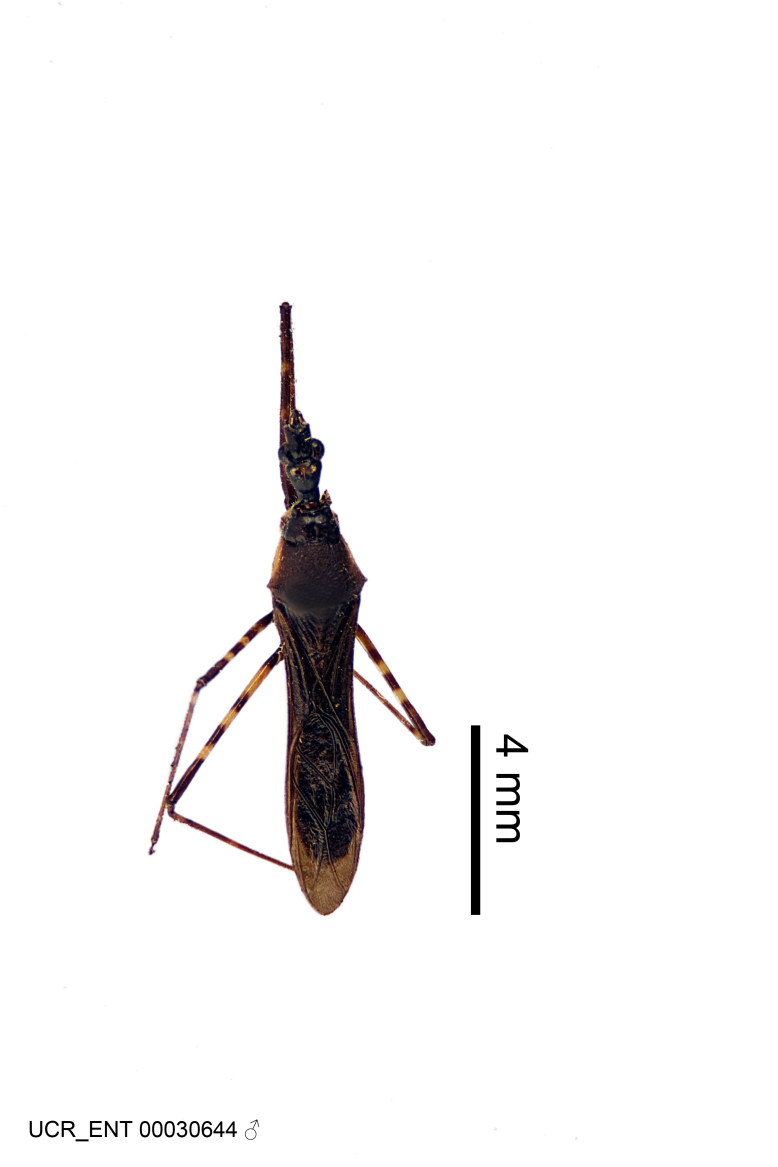
*Zelus mimus* Stål, 1862, male, dorsal view (UCR_ENT 00030644, Cartago, Costa Rica)

**Figure 138b.**
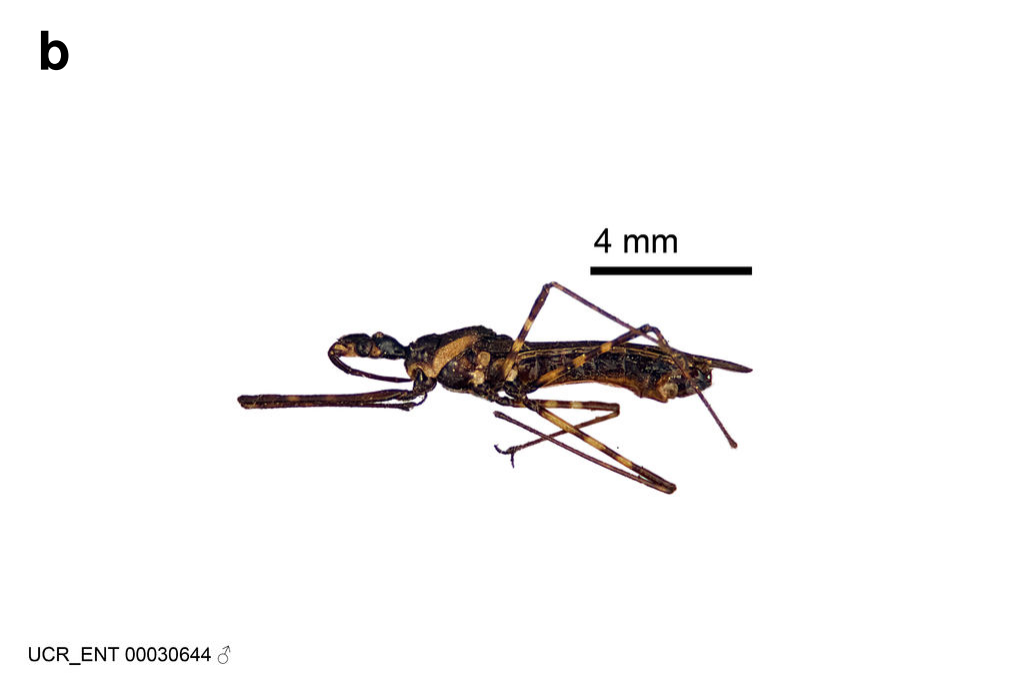
*Zelus mimus* Stål, 1862, male, lateral view (UCR_ENT 00030644, Cartago, Costa Rica)

**Figure 138c.**
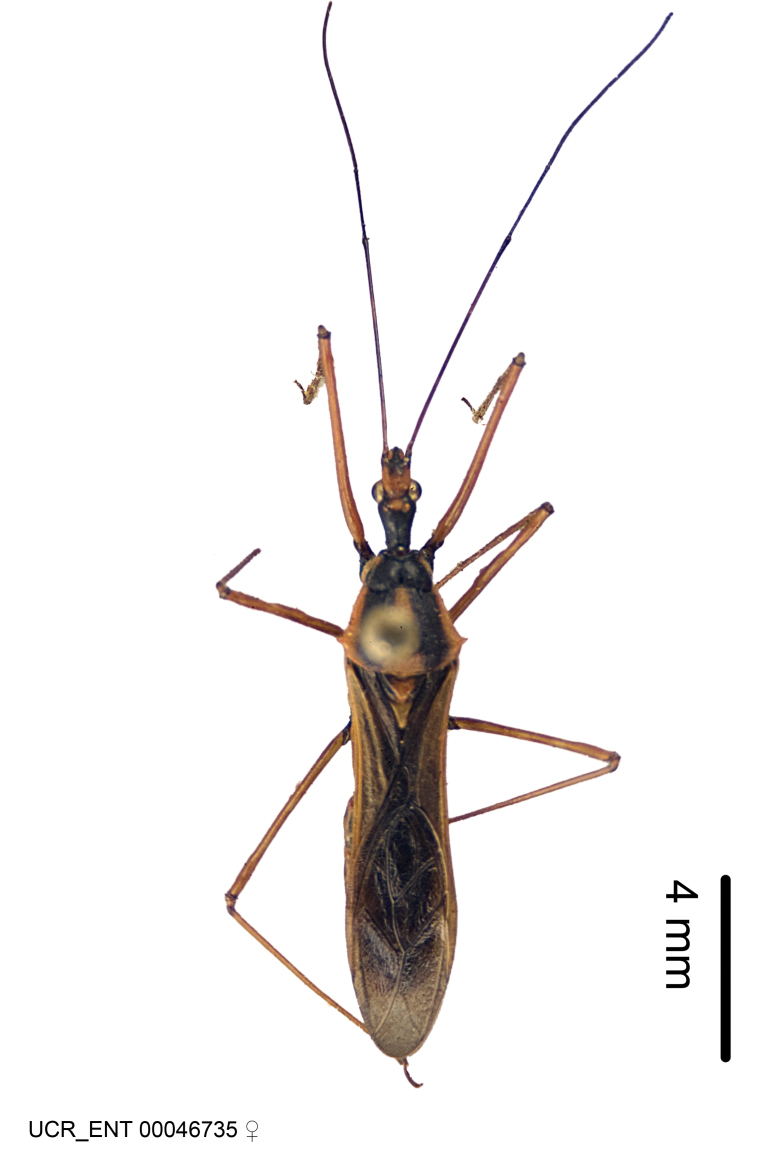
*Zelus mimus* Stål, 1862, female, dorsal view (UCR_ENT 00046735, Chiriqui, Panama)

**Figure 138d.**
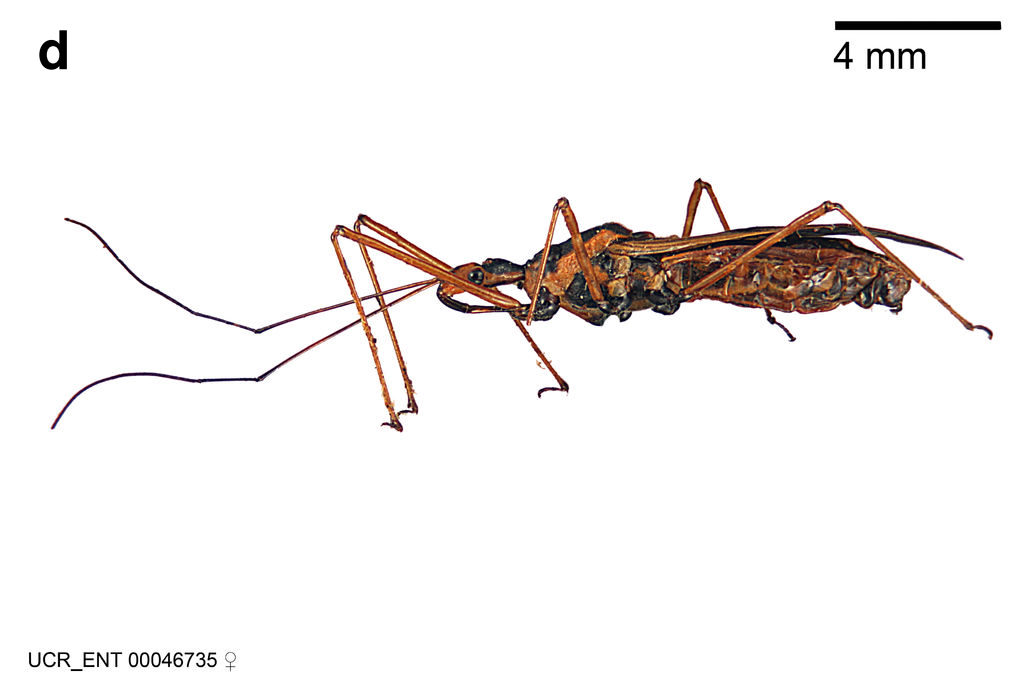
*Zelus mimus* Stål, 1862, female, lateral view (UCR_ENT 00046735, Chiriqui, Panama)

**Figure 138e.**
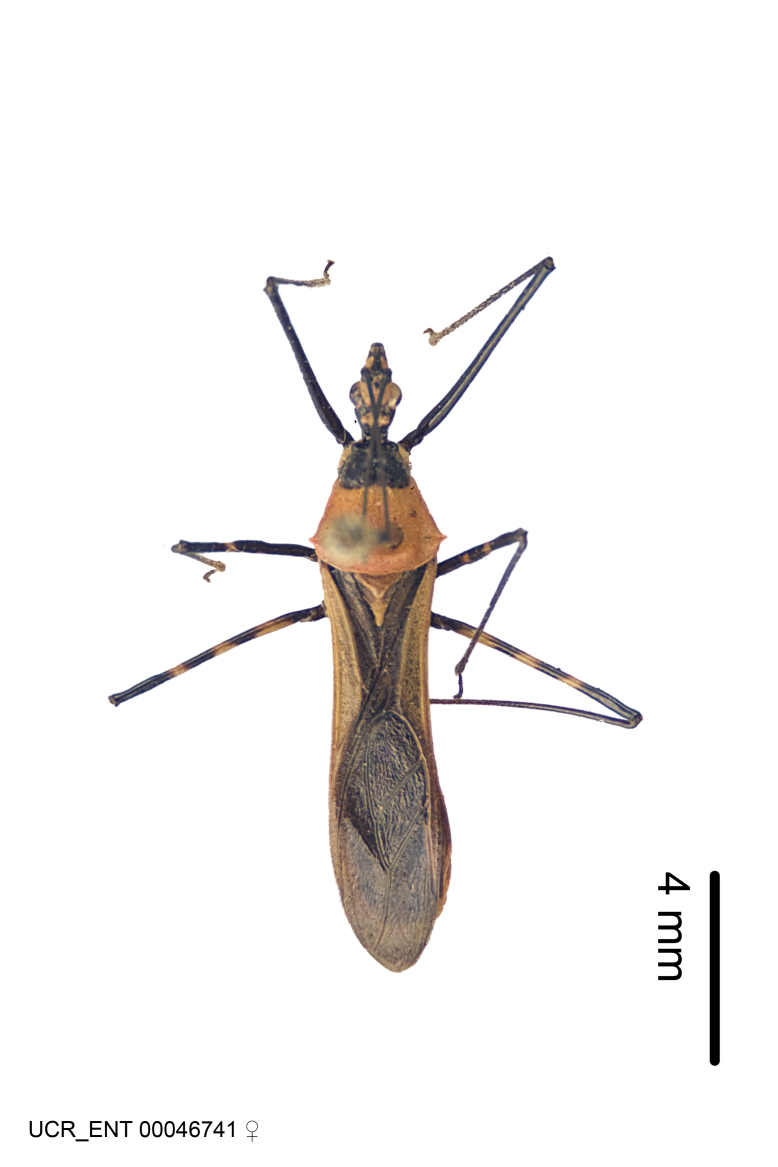
*Zelus mimus* Stål, 1862, female, dorsal view (UCR_ENT 00046741, Chiriqui, Panama)

**Figure 138f.**
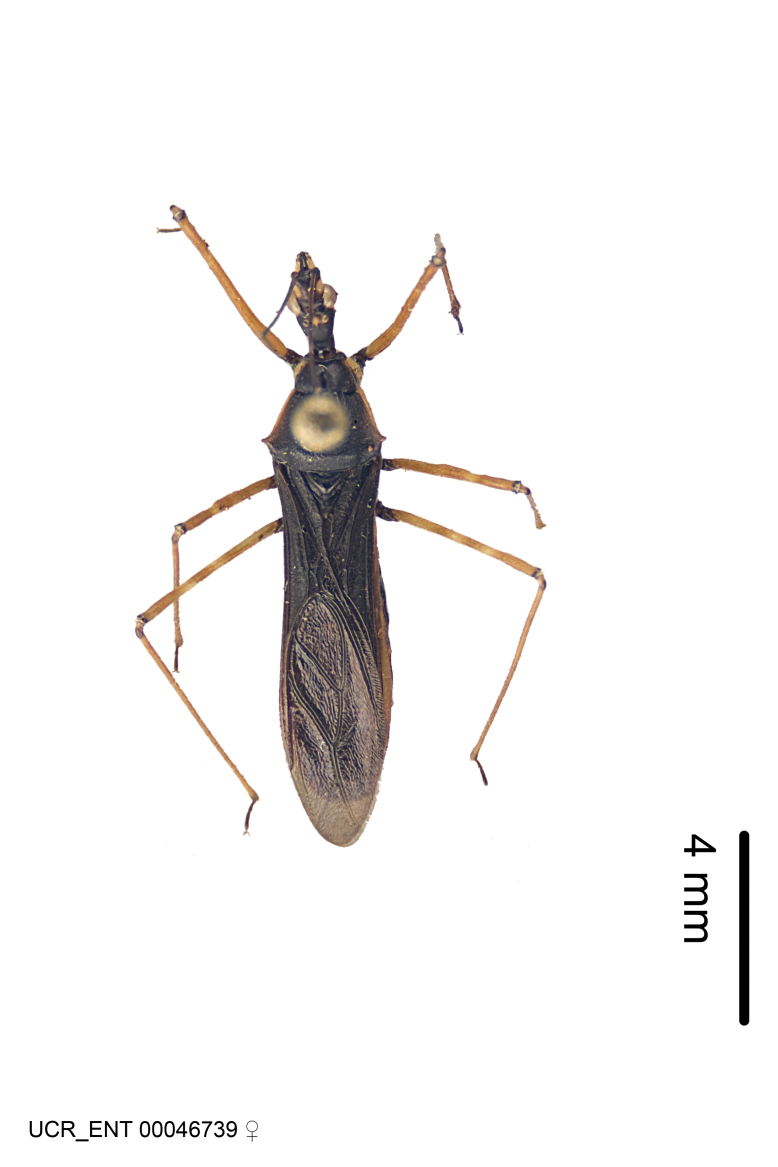
*Zelus mimus* Stål, 1862, female, dorsal view (UCR_ENT 00046739, Chiriqui, Panama)

**Figure 139a.**
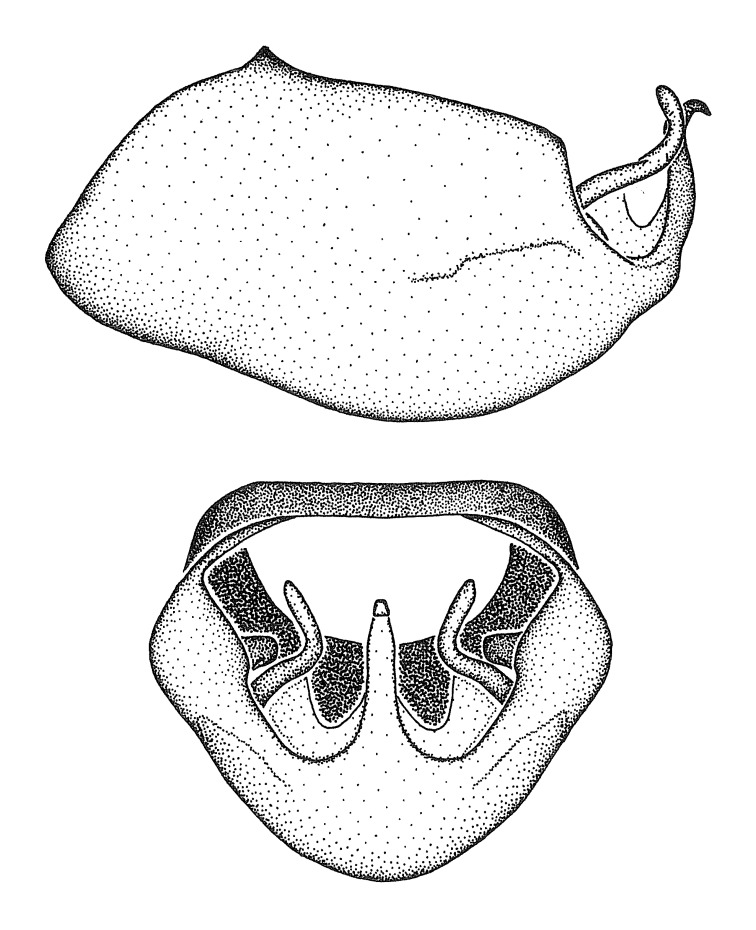
*Zelus mimus* Stål, 1862, pygophore, lateral and posterior views

**Figure 139b.**
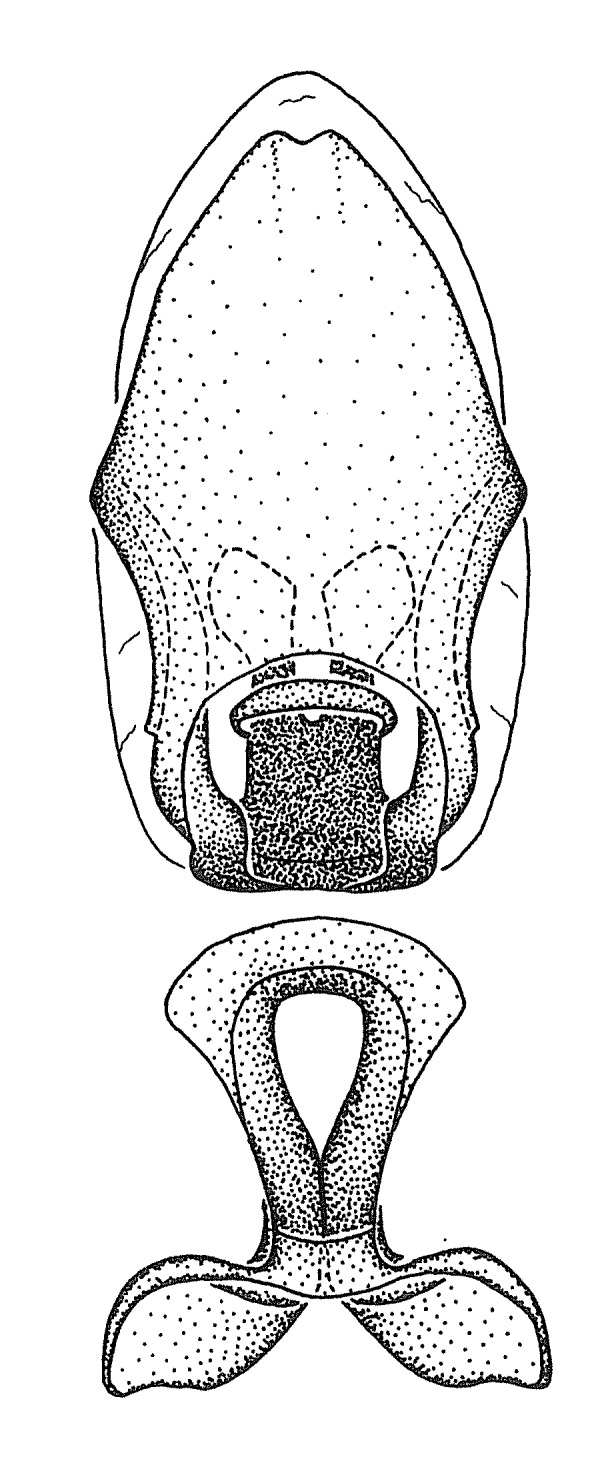
*Zelus mimus* Stål, 1862, phallus, dorsal view

**Figure 141a.**
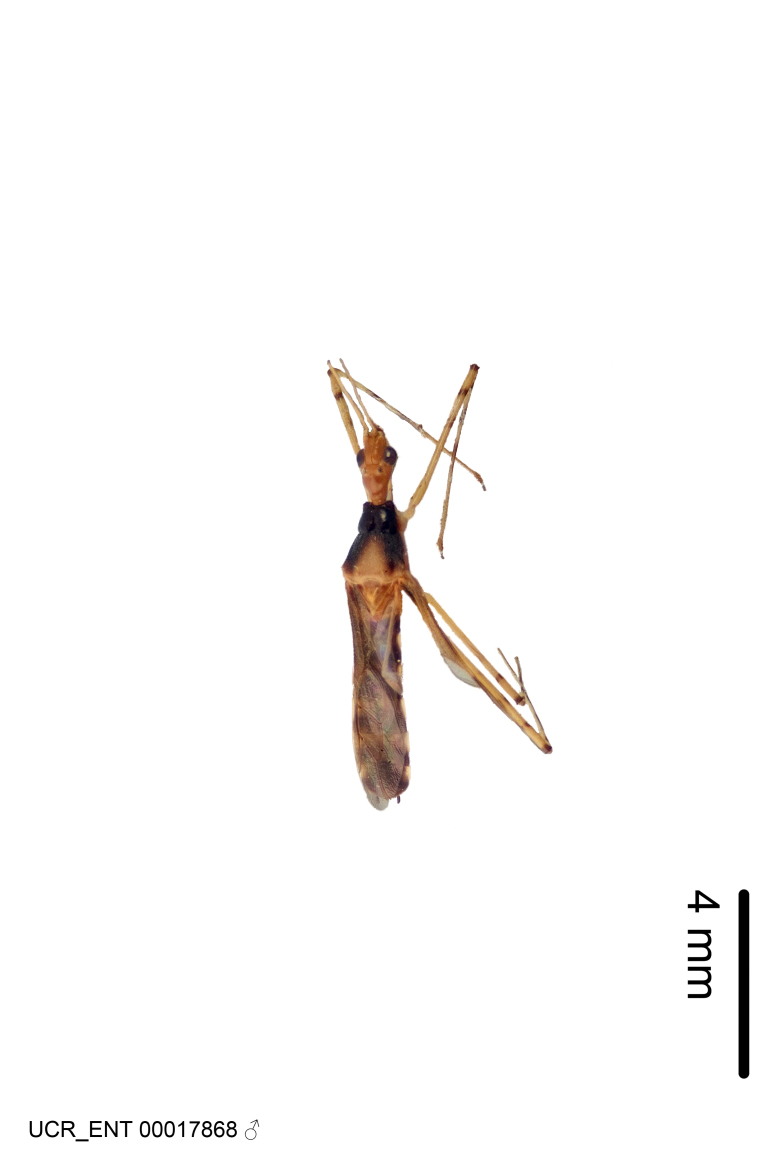
*Zelus minutus* Hart, 1987, male, dorsal view (UCR_ENT 00017868, Panama)

**Figure 141b.**
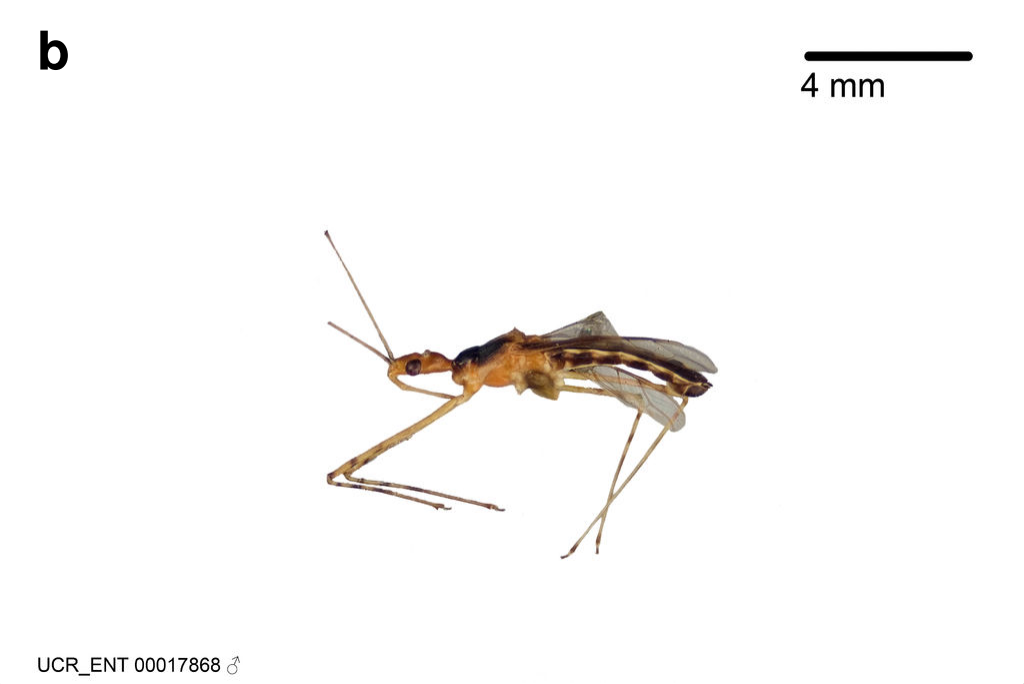
*Zelus minutus* Hart, 1987, male, lateral view (UCR_ENT 00017868, Panama)

**Figure 141c.**
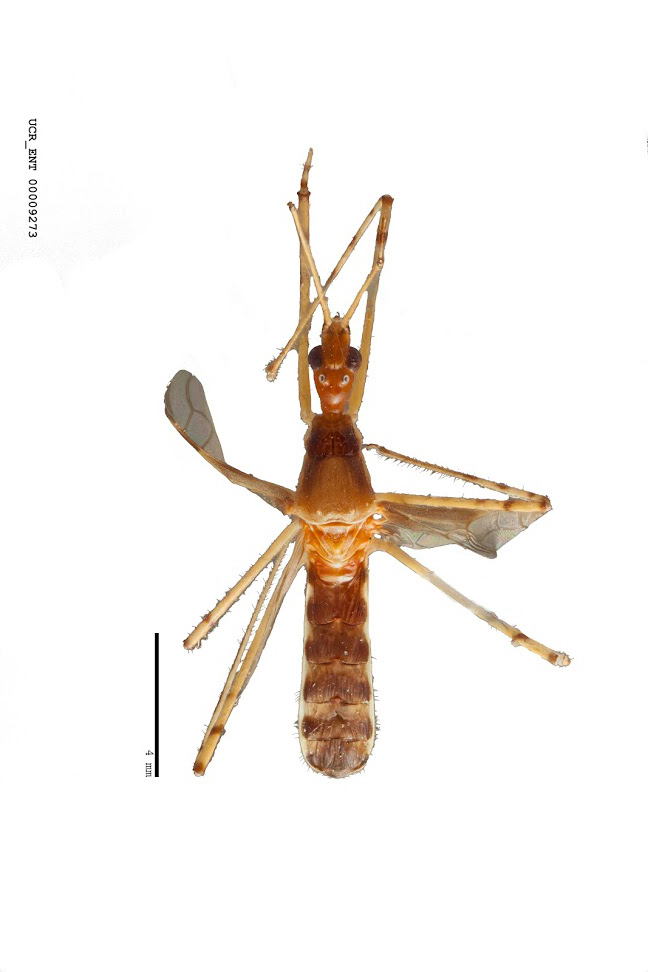
*Zelus minutus* Hart, 1987, male, dorsal view (UCR_ENT 00009273, Panama)

**Figure 141d.**
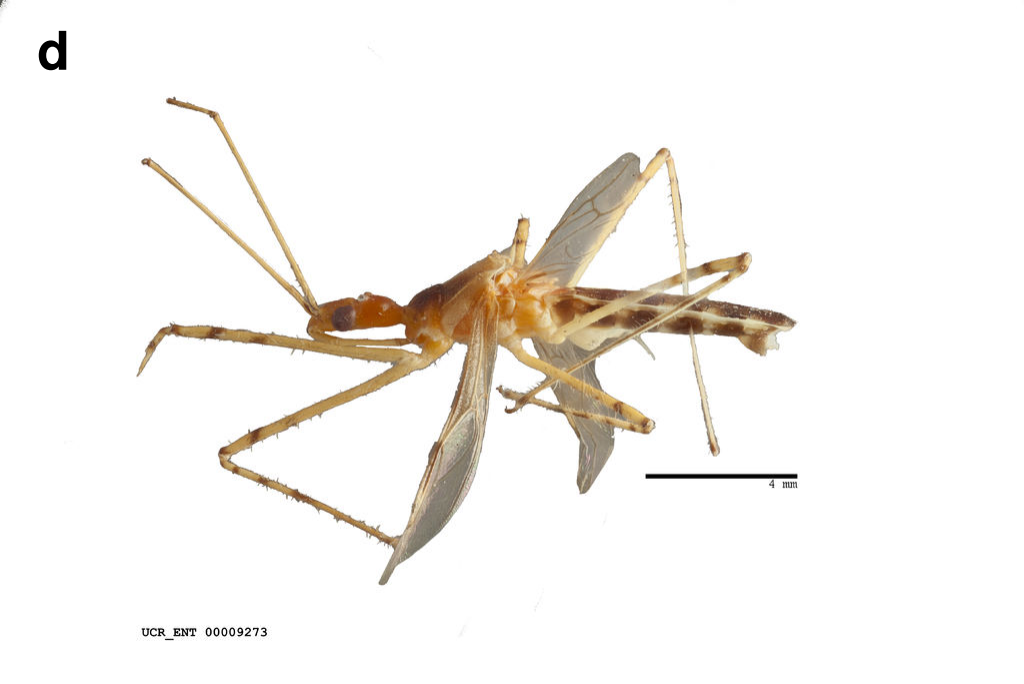
*Zelus minutus* Hart, 1987, male, lateral view (UCR_ENT 00009273, Panama)

**Figure 142a.**
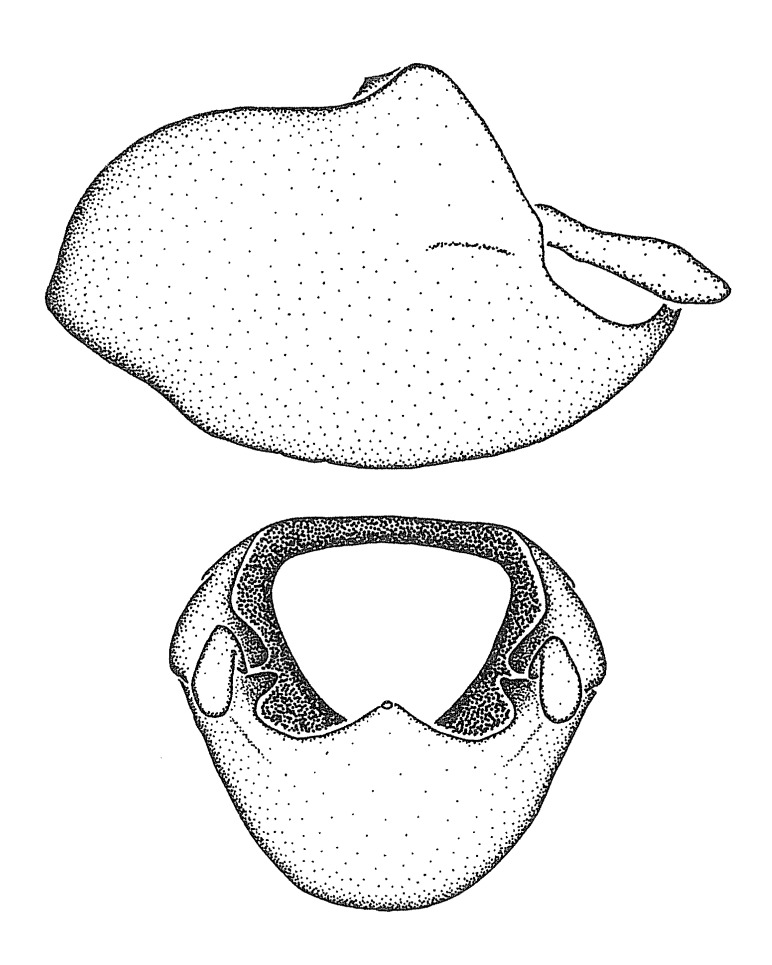


**Figure 142b.**
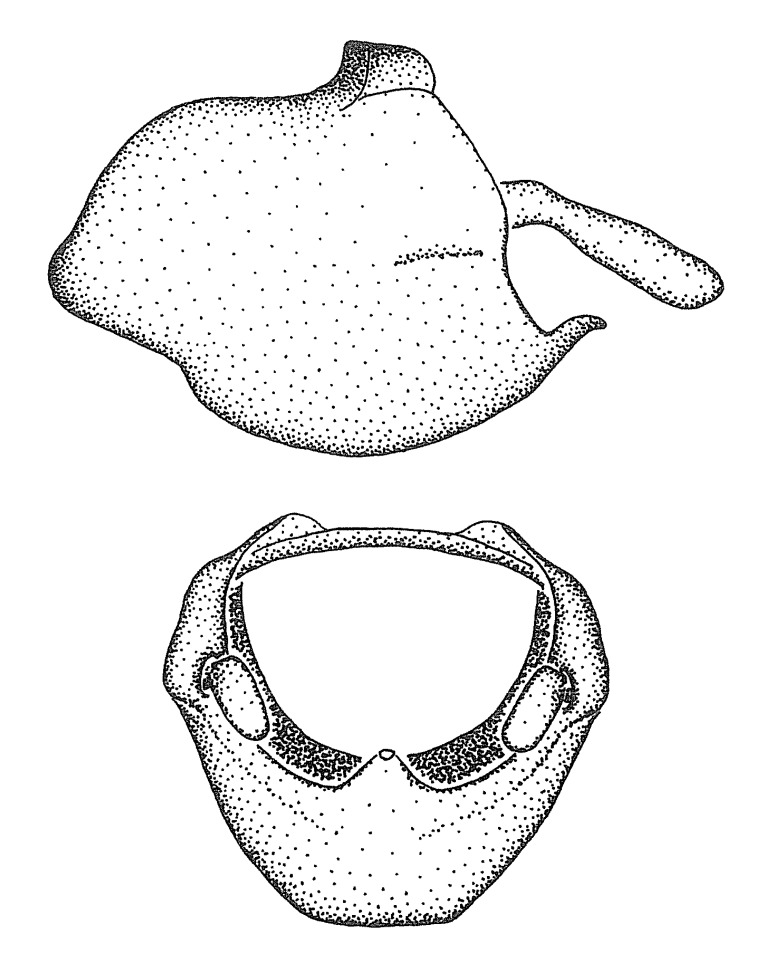


**Figure 142c.**
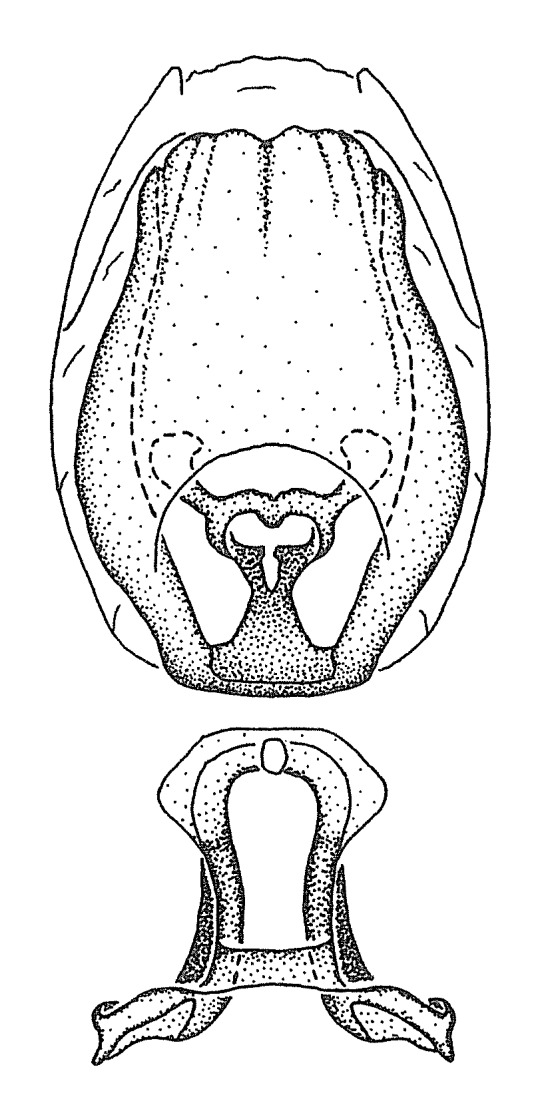


**Figure 142d.**
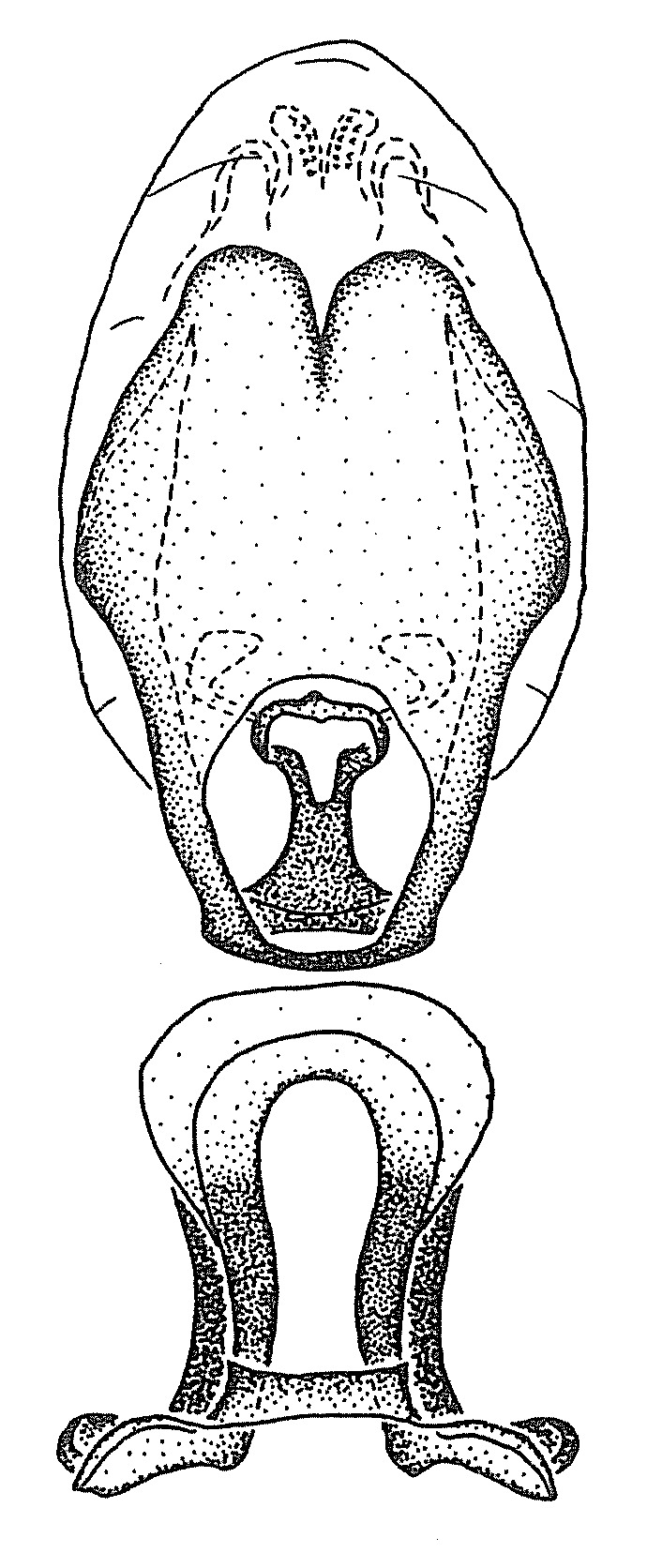


**Figure 144a.**
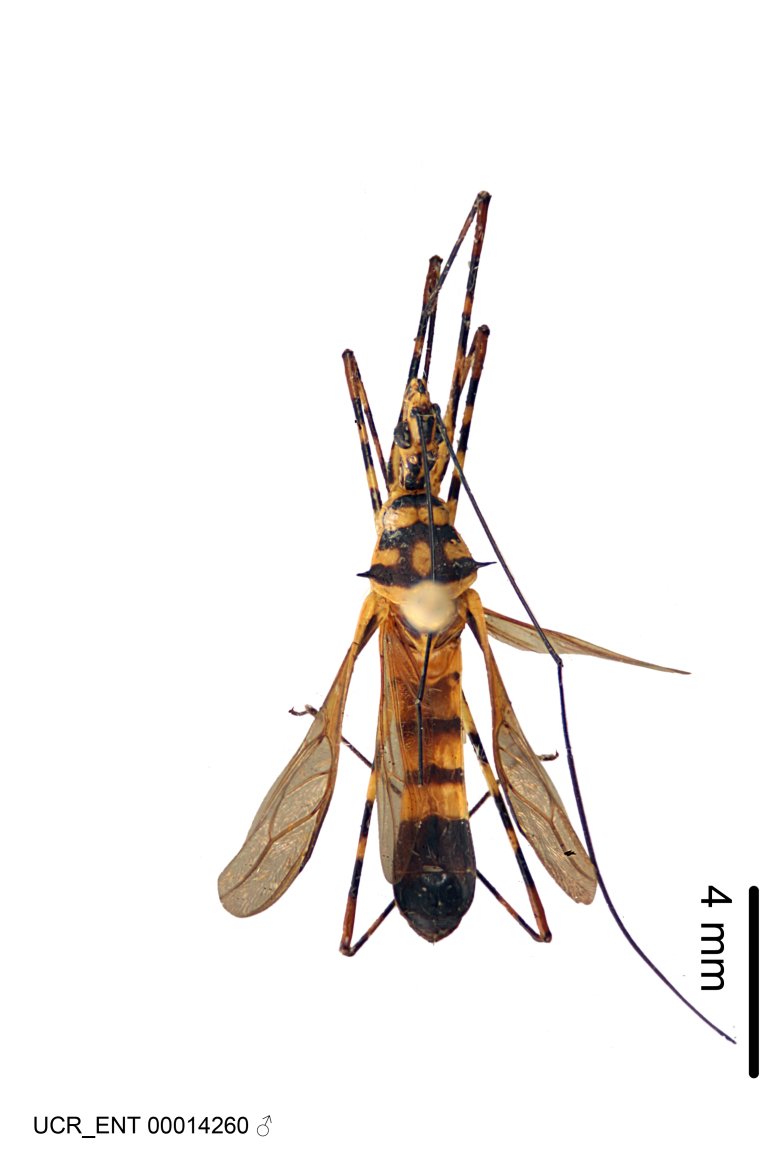
*Zelus nigromaculatus* Champion, 1899, male, dorsal view (UCR_ENT 0001426, Limon, Costa Rica)

**Figure 144b.**
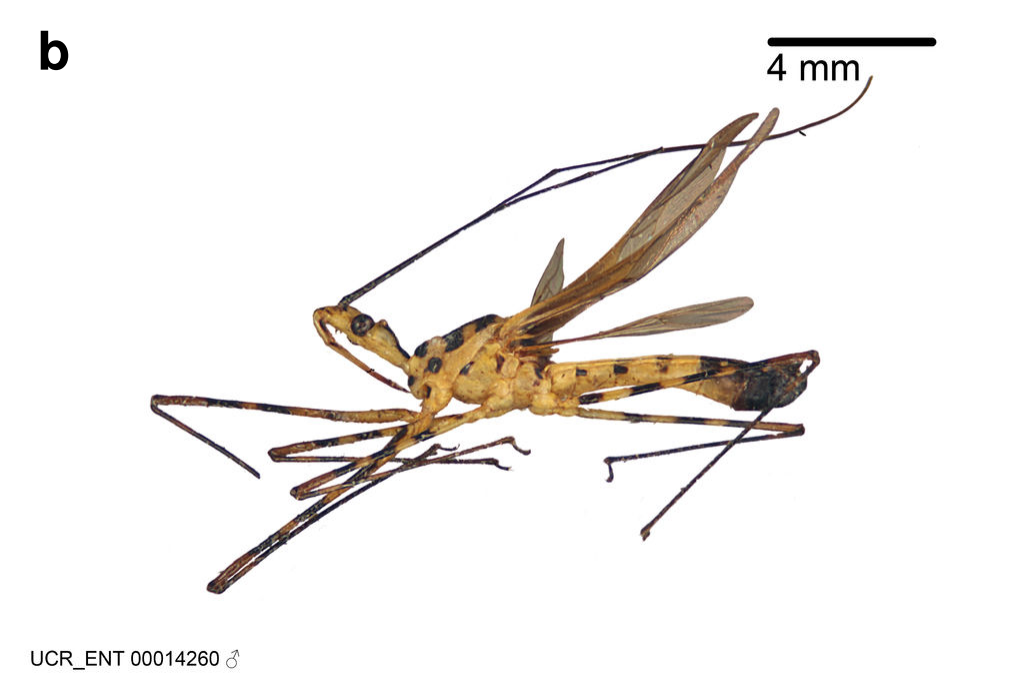
*Zelus nigromaculatus* Champion, 1899, male, lateral view (UCR_ENT 0001426, Limon, Costa Rica)

**Figure 144c.**
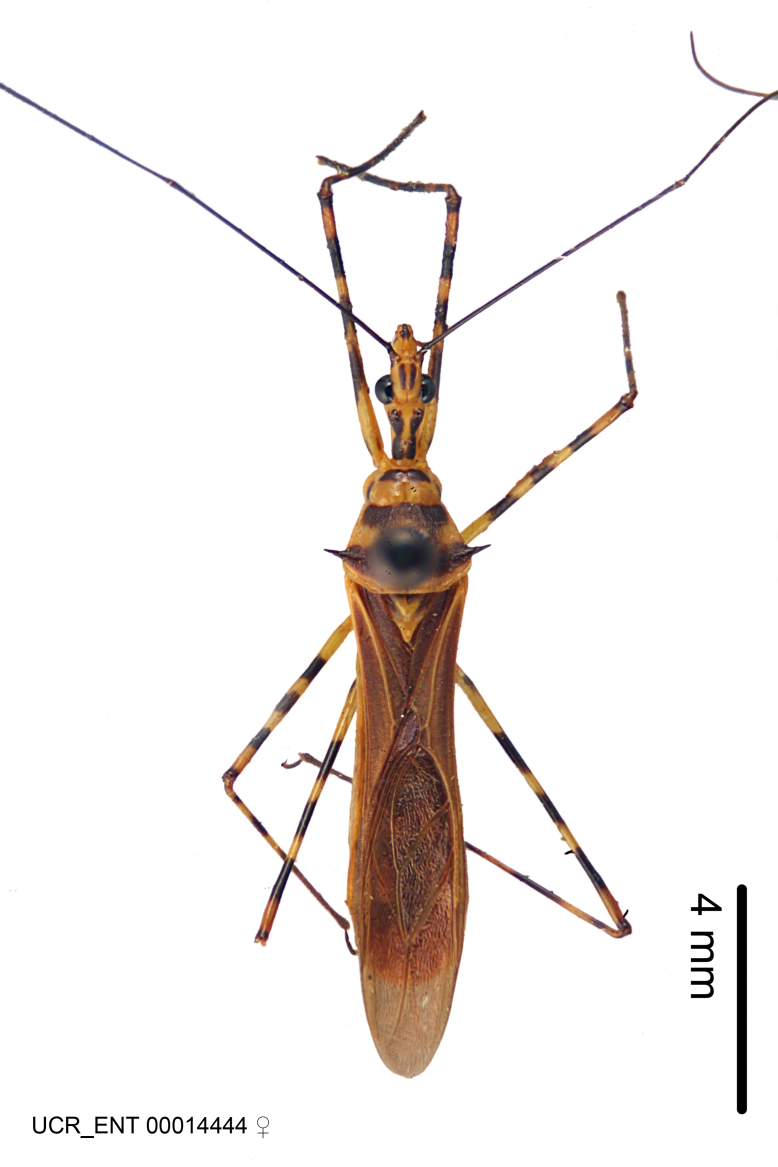
*Zelus nigromaculatus* Champion, 1899, female, dorsal view (UCR_ENT 00014444, Puntarenas, Costa Rica)

**Figure 144d.**
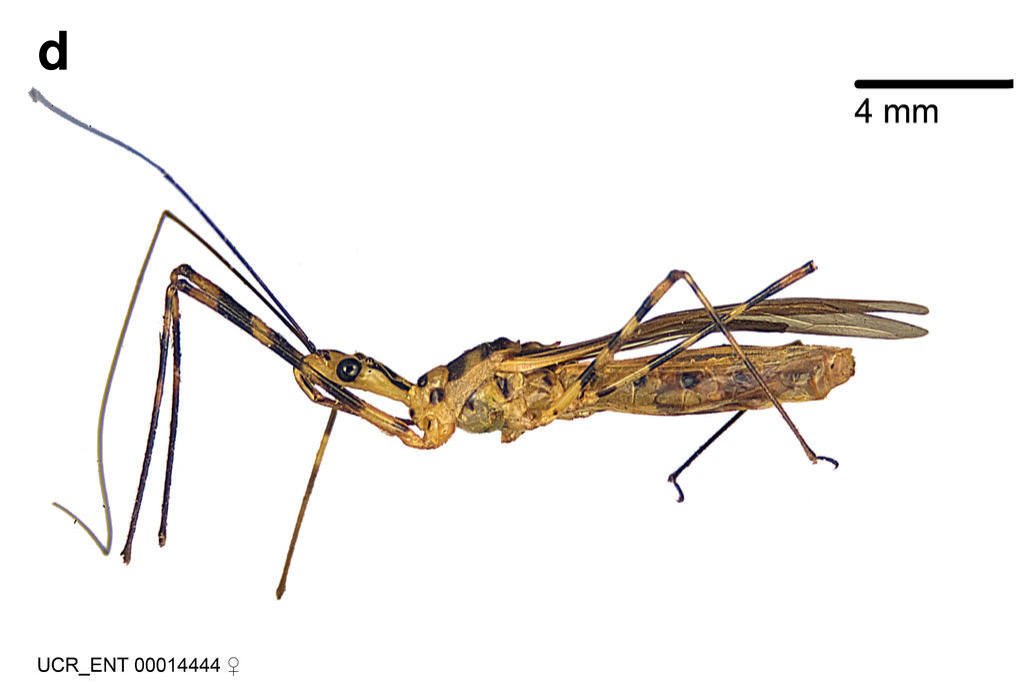
*Zelus nigromaculatus* Champion, 1899, female, lateral view (UCR_ENT 00014444, Puntarenas, Costa Rica

**Figure 145a.**
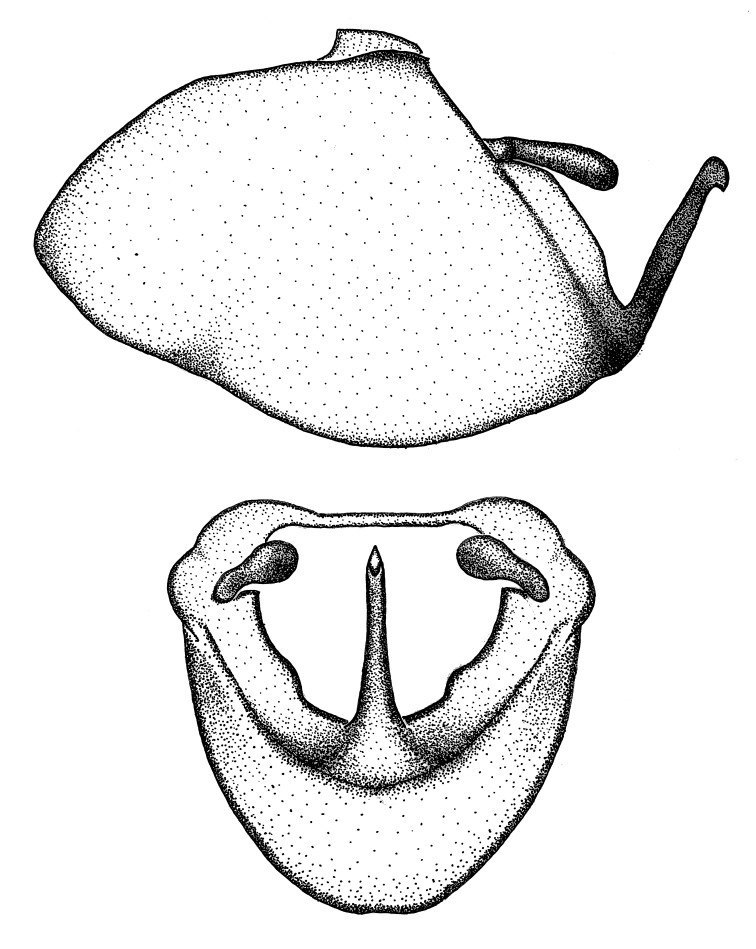
*Zelus nigromaculatus* Champion, 1899, pygophore, lateral and posterior views

**Figure 145b.**
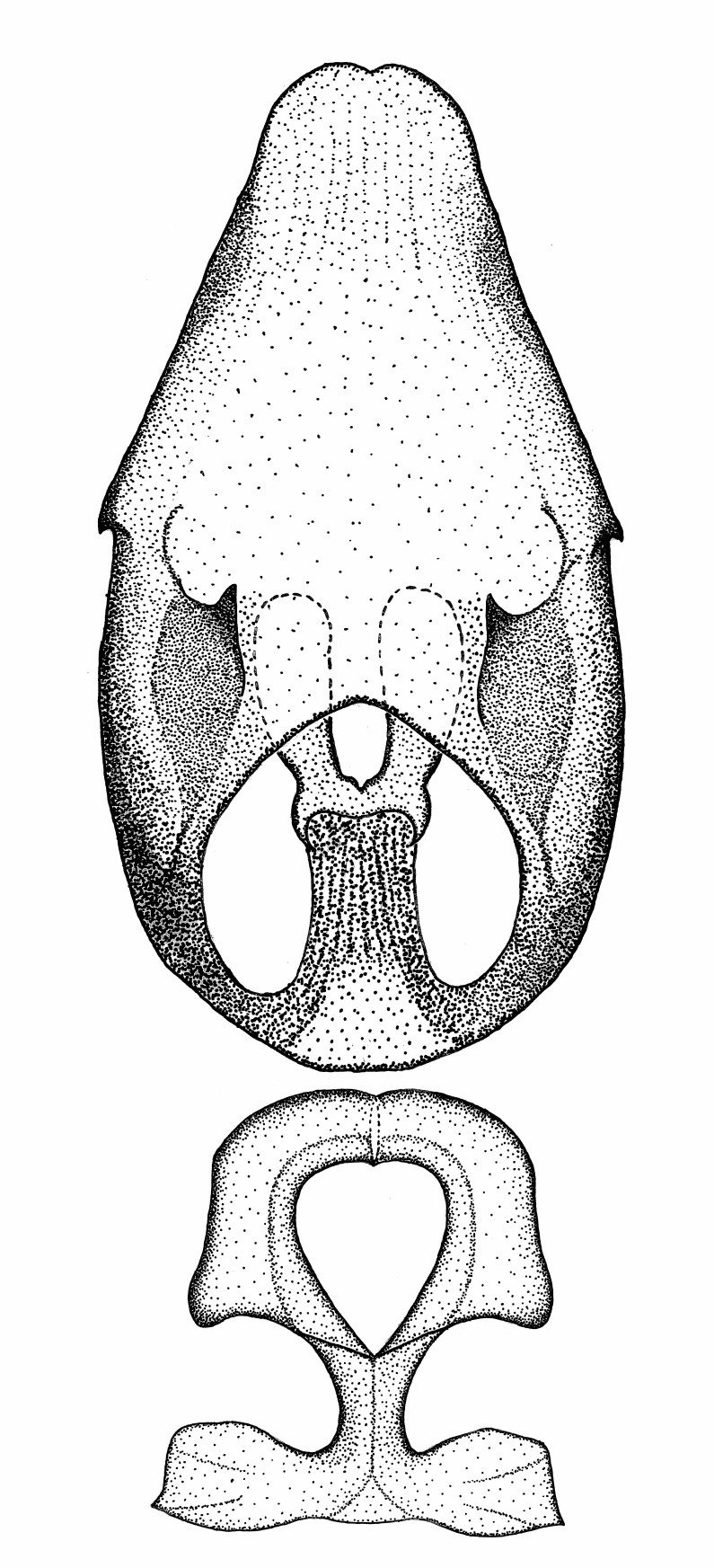
*Zelus nigromaculatus* Champion, 1899, phallus, dorsal view

**Figure 147a.**
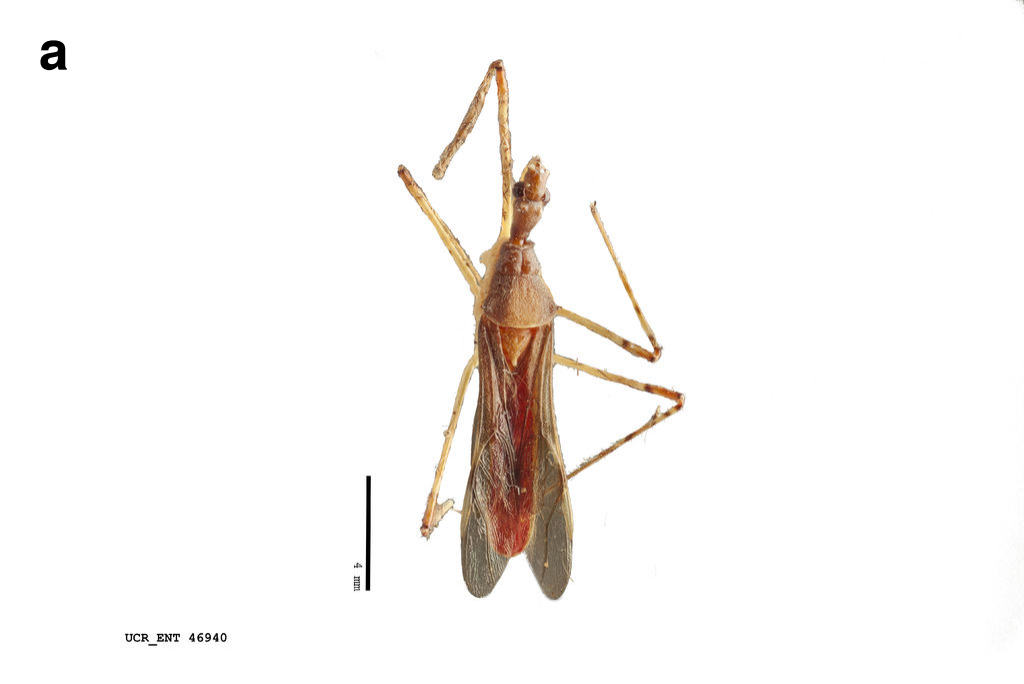
*Zelus nugax* Stål, 1862, male, dorsal view (UCR_ENT 00046940, Mexico)

**Figure 147b.**
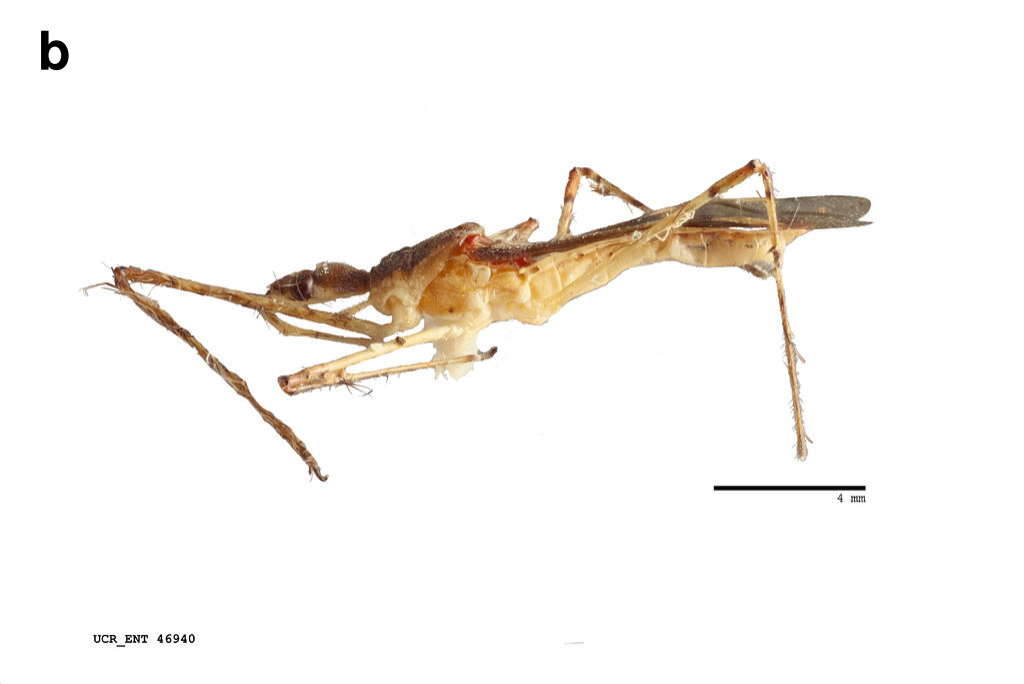
*Zelus nugax* Stål, 1862, male, lateral view (UCR_ENT 00046940, Mexico)

**Figure 147c.**
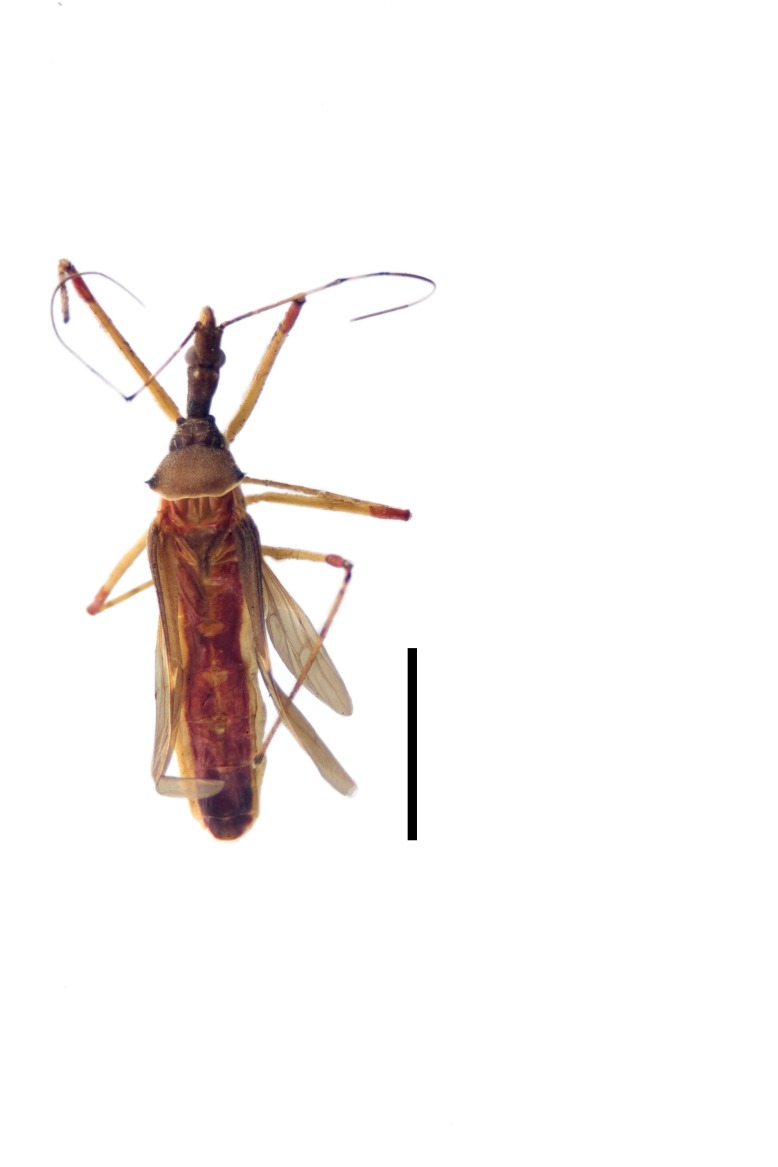
*Zelus nugax* Stål, 1862, female, dorsal (UCR_ENT 00001214, French Guiana)

**Figure 147d.**
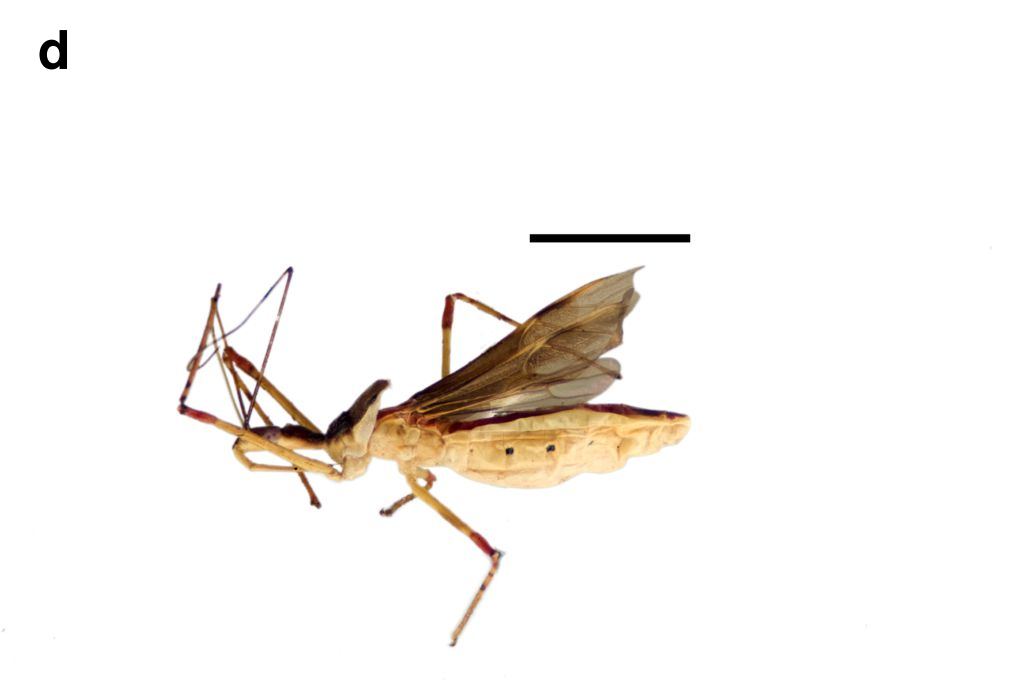
*Zelus nugax* Stål, 1862, female, lateral (UCR_ENT 00001214, French Guiana)

**Figure 148a.**
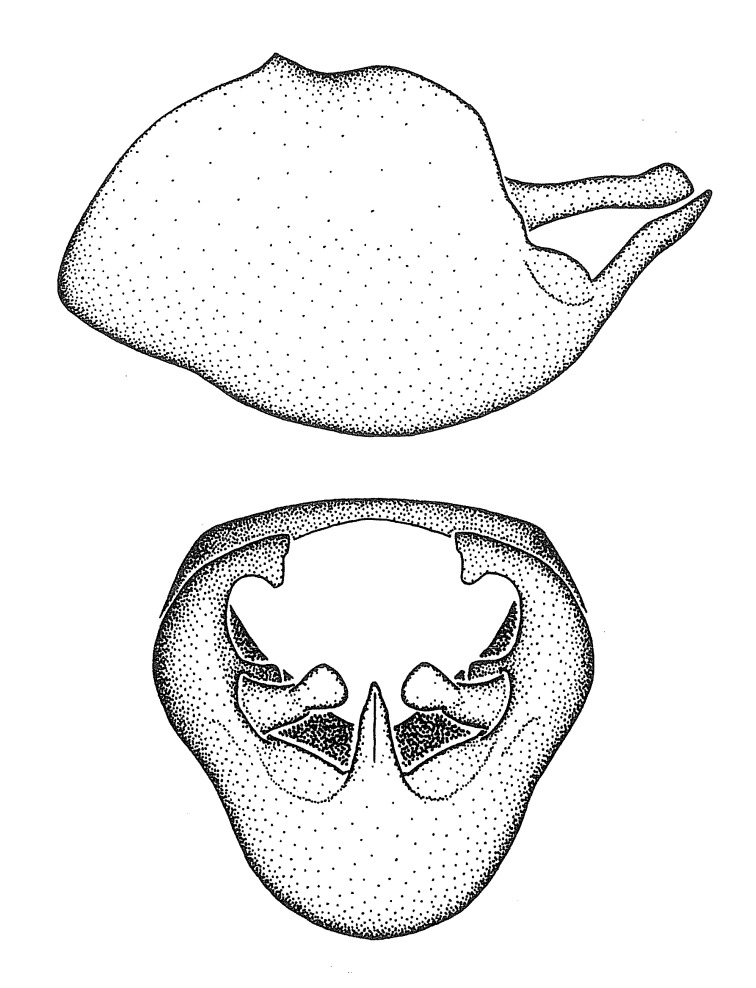
*Zelus nugax* Champion, 1899, pygophore, lateral and posterior views

**Figure 148b.**
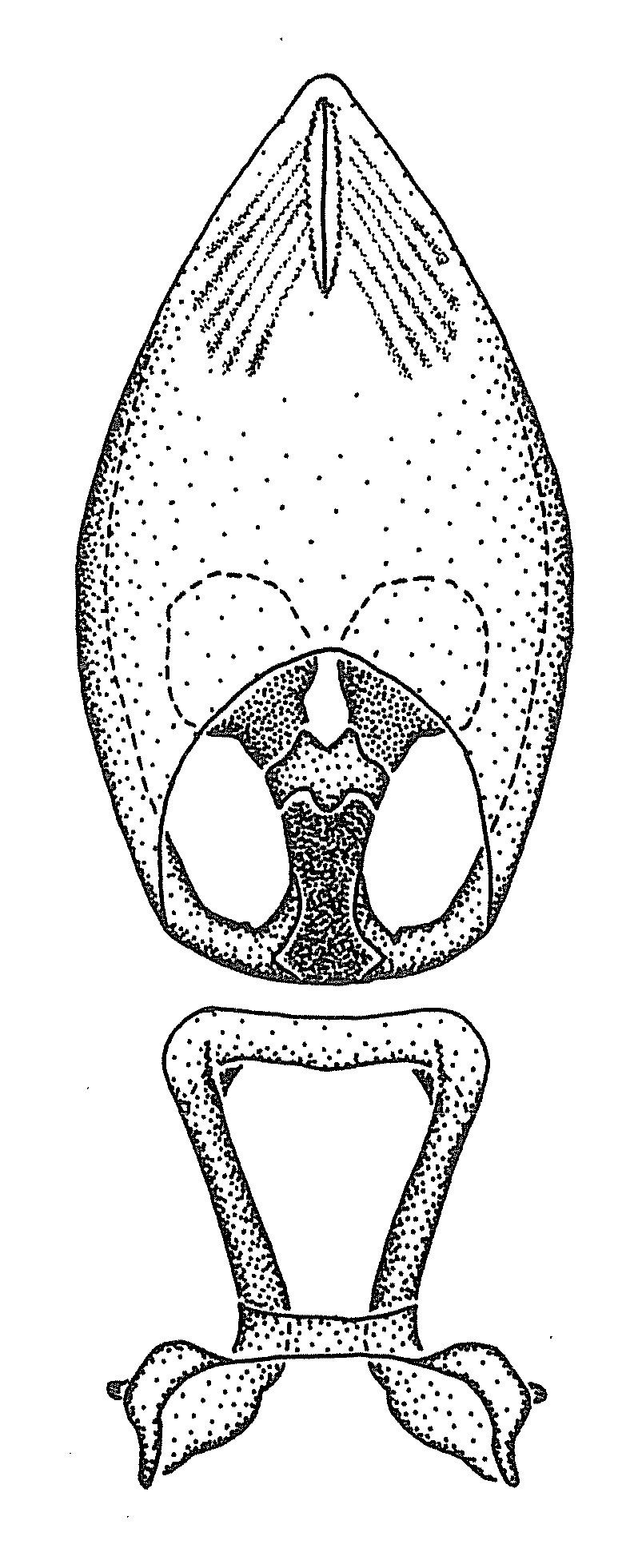
*Zelus nugax* Champion, 1899, phallus, dorsal view

**Figure 150a.**
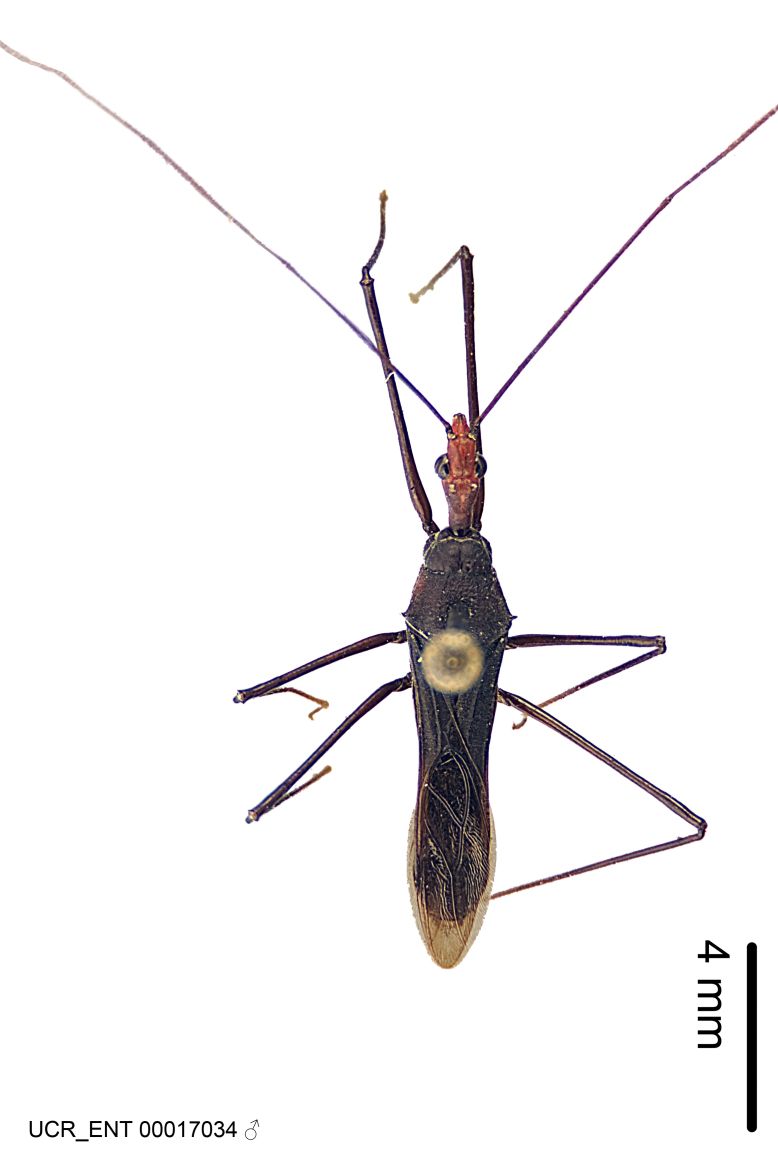
*Zelus panamensis* Zhang & Hart, sp. n., male, dorsal view (UCR_ENT 00017034, Canal Zone, Panama)

**Figure 150b.**
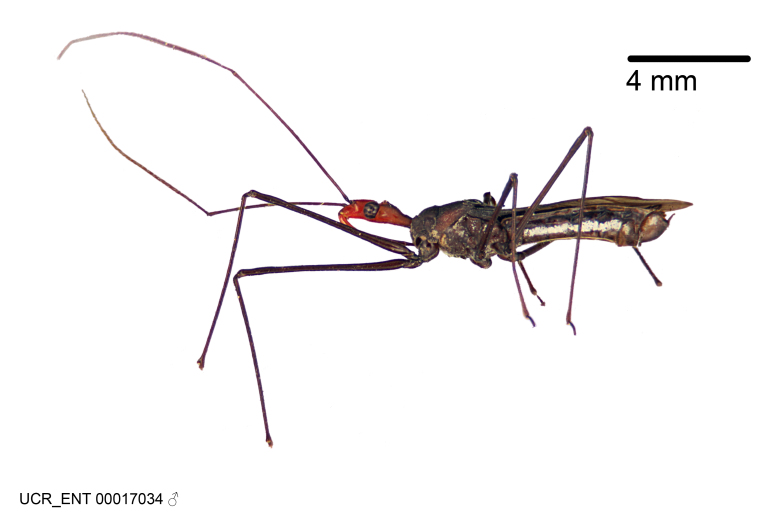
*Zelus panamensis* Zhang & Hart, sp. n., male, lateral view (UCR_ENT 00017034, Canal Zone, Panama)

**Figure 150c.**
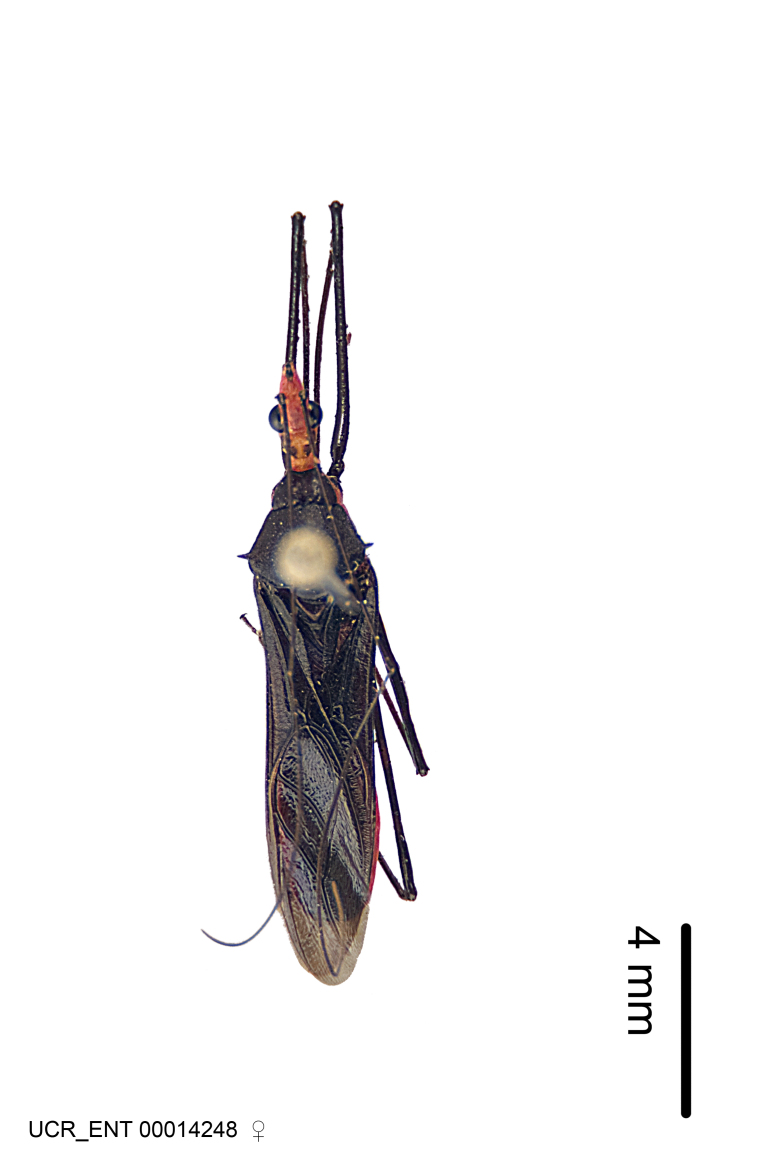
*Zelus panamensis* Zhang & Hart, sp. n., female, dorsal view (UCR_ENT 00014248, Guanacaste, Costa Rica)

**Figure 150d.**
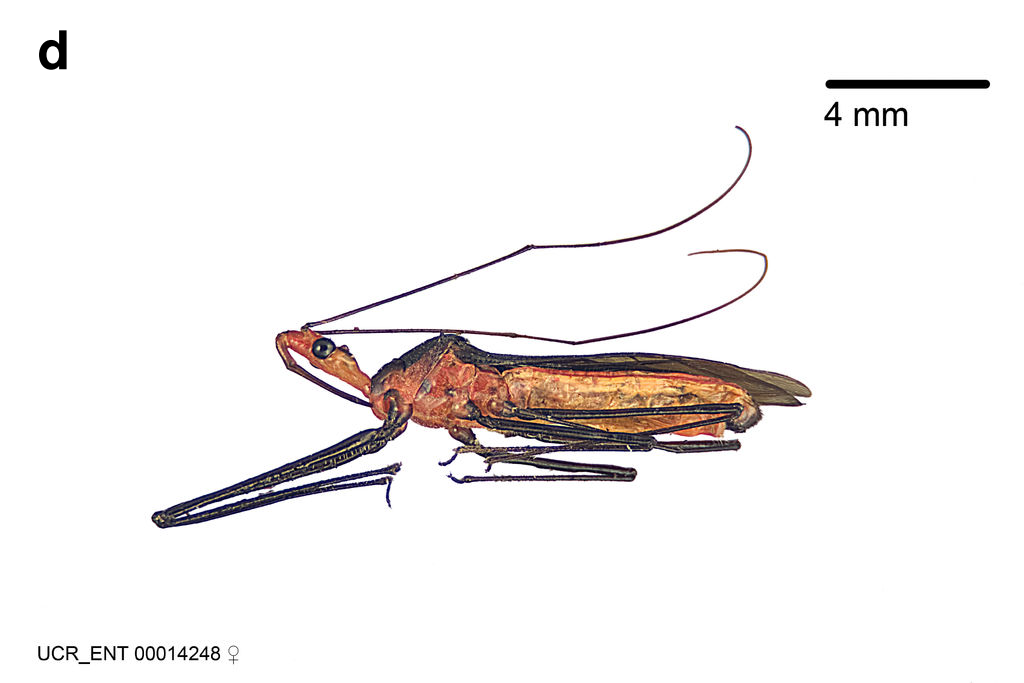
*Zelus panamensis* Zhang & Hart, sp. n., female, lateral view (UCR_ENT 00014248, Guanacaste, Costa Rica)

**Figure 151a.**
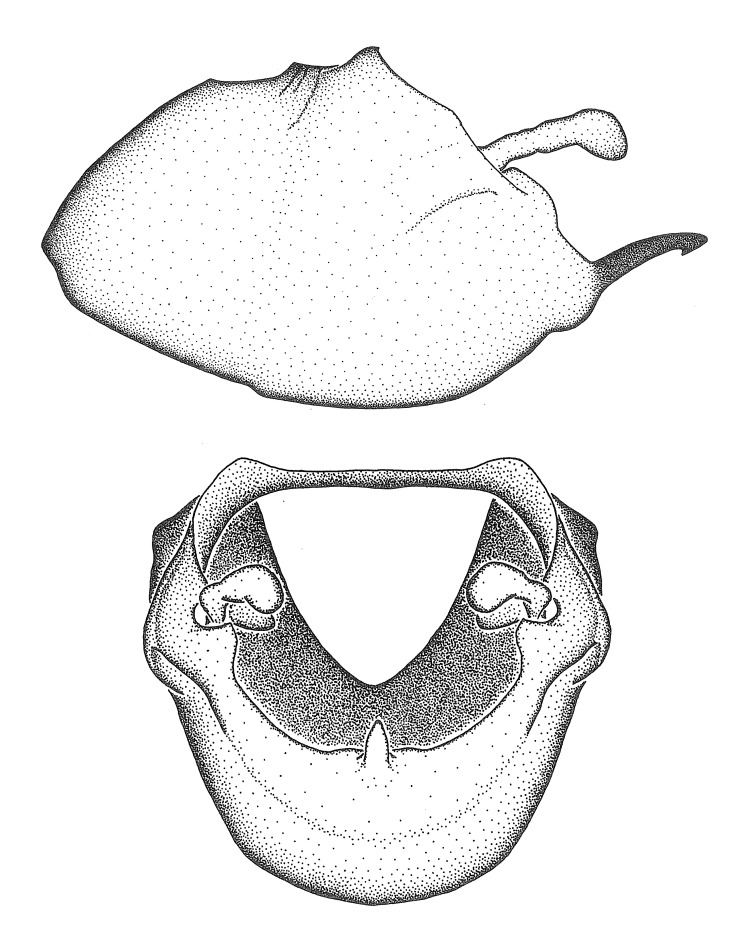
*Zelus panamensis* Zhang & Hart, sp. n., pygophore, lateral and posterior views

**Figure 151b.**
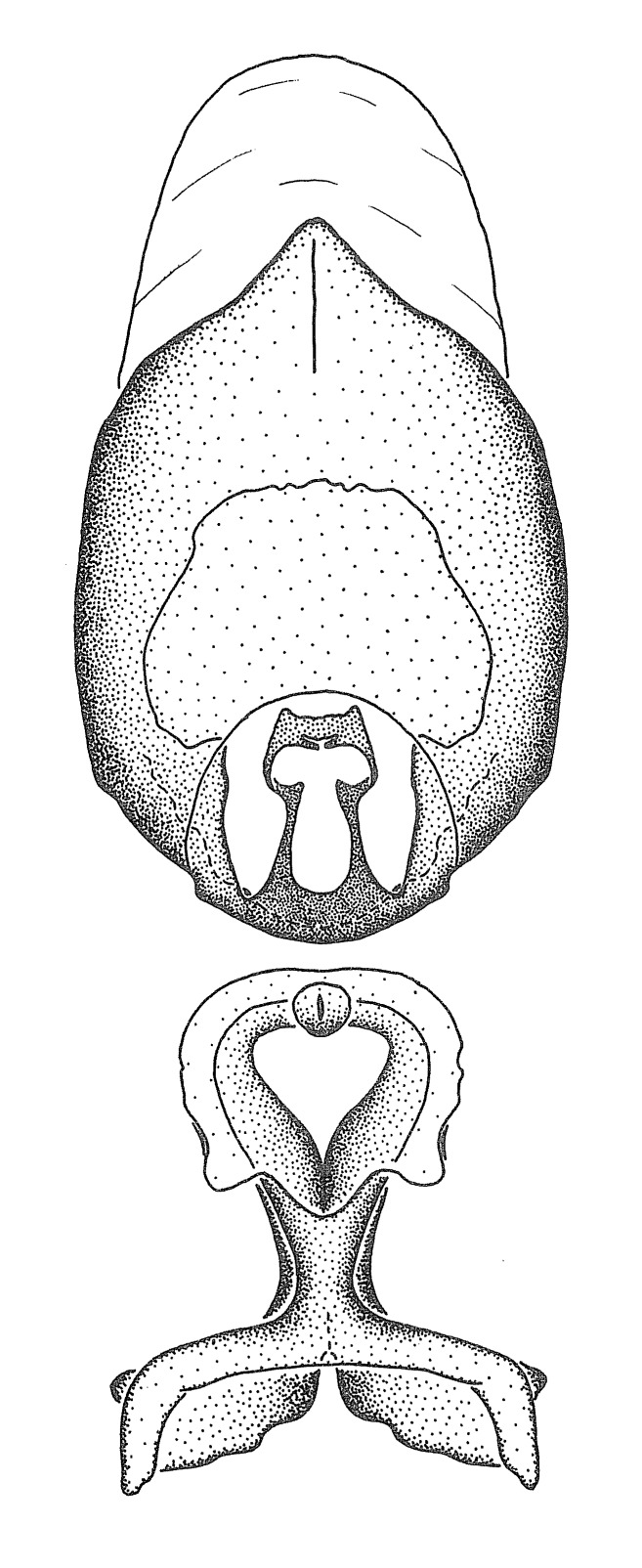
*Zelus panamensis* Zhang & Hart, sp. n., phallus, dorsal view

**Figure 153a.**
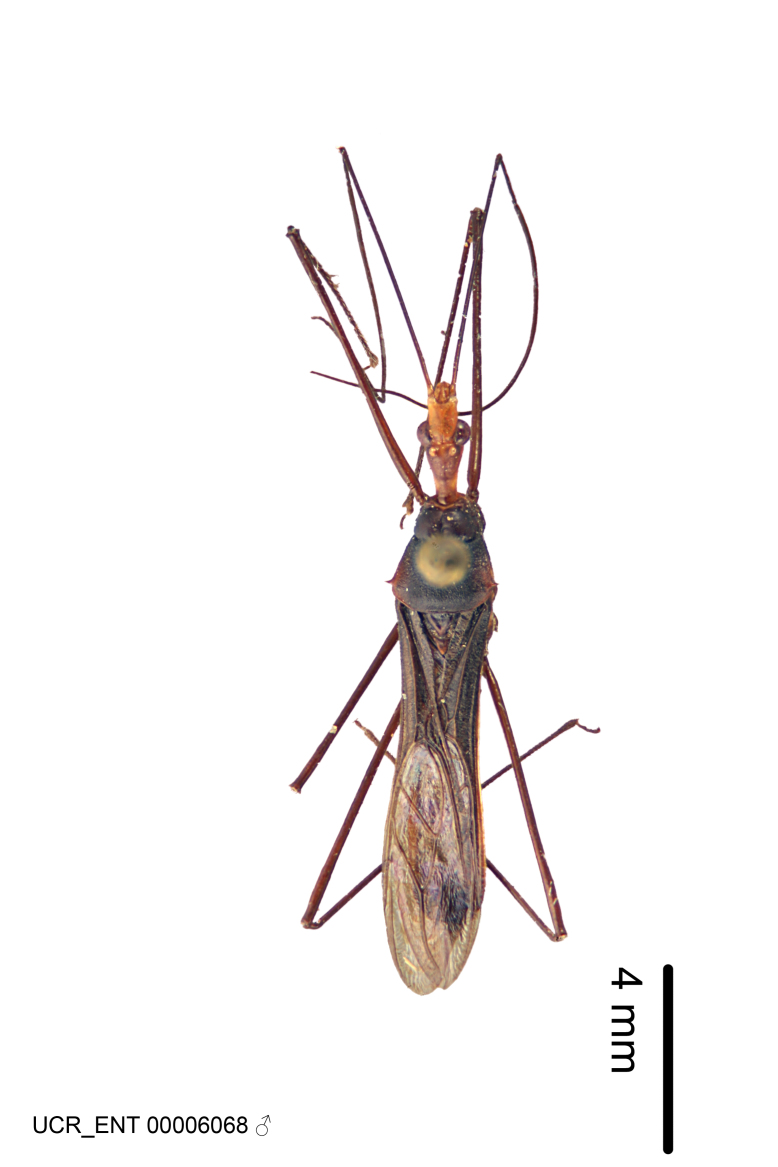
*Zelus paracephalus* Zhang & Hart, sp. n., male, dorsal view (UCR_ENT 00006068)

**Figure 153b.**
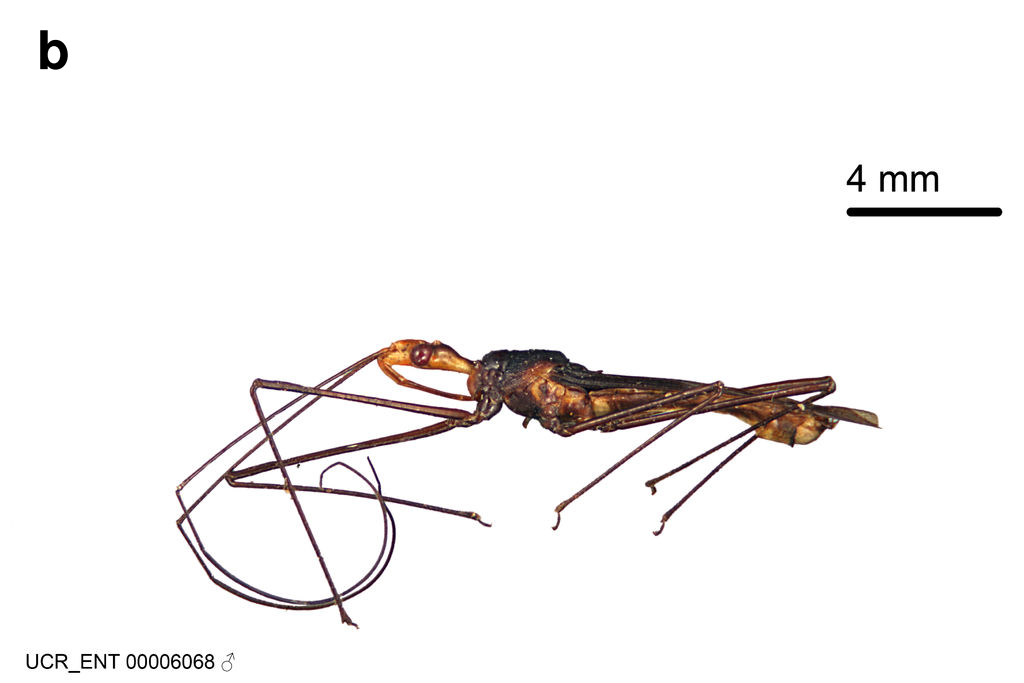
*Zelus paracephalus* Zhang & Hart, sp. n., male, lateral view (UCR_ENT 00006068)

**Figure 153c.**
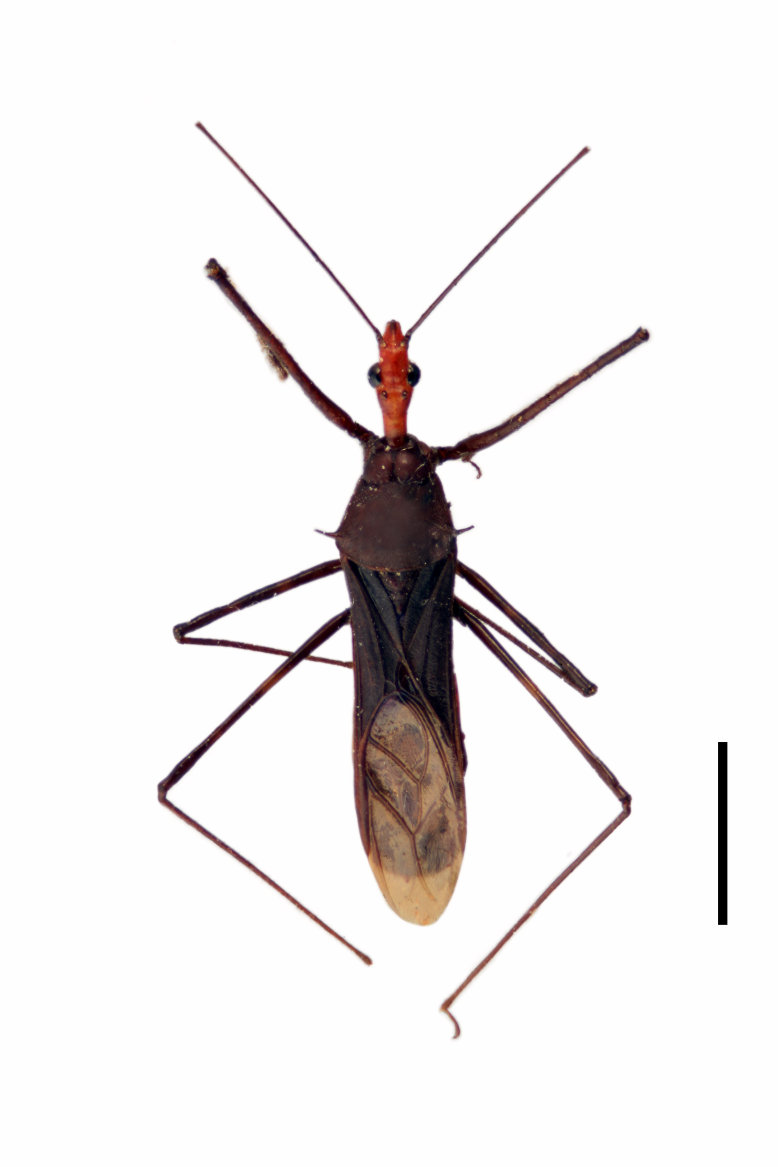
*Zelus paracephalus* Zhang & Hart, sp. n., female, dorsal view (UCR_ENT 00057802)

**Figure 153d.**
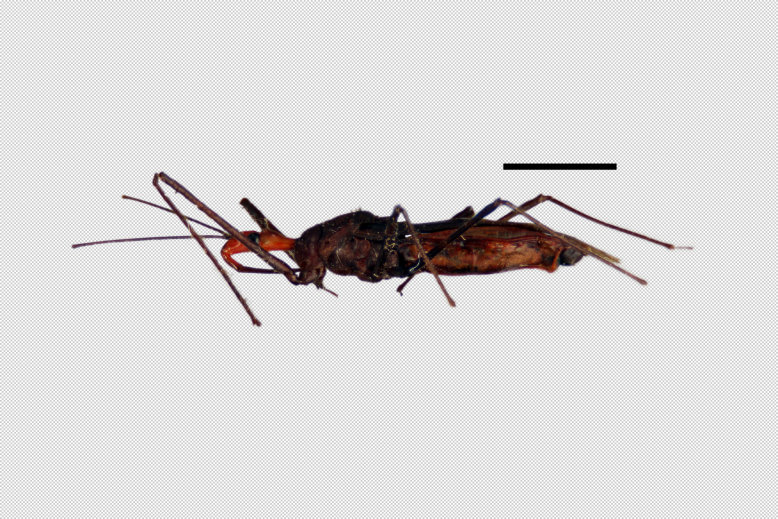
*Zelus paracephalus* Zhang & Hart, sp. n., female, lateral view (UCR_ENT 00057802)

**Figure 154a.**
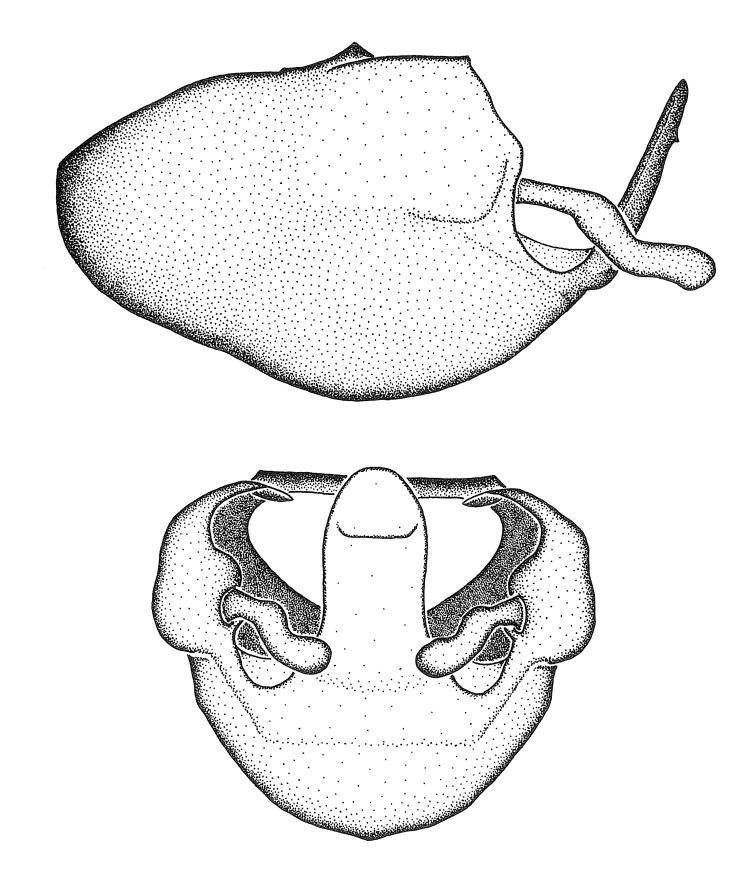
*Zelus paracephalus* Zhang & Hart, sp. n., pygophore, lateral and posterior views

**Figure 154b.**
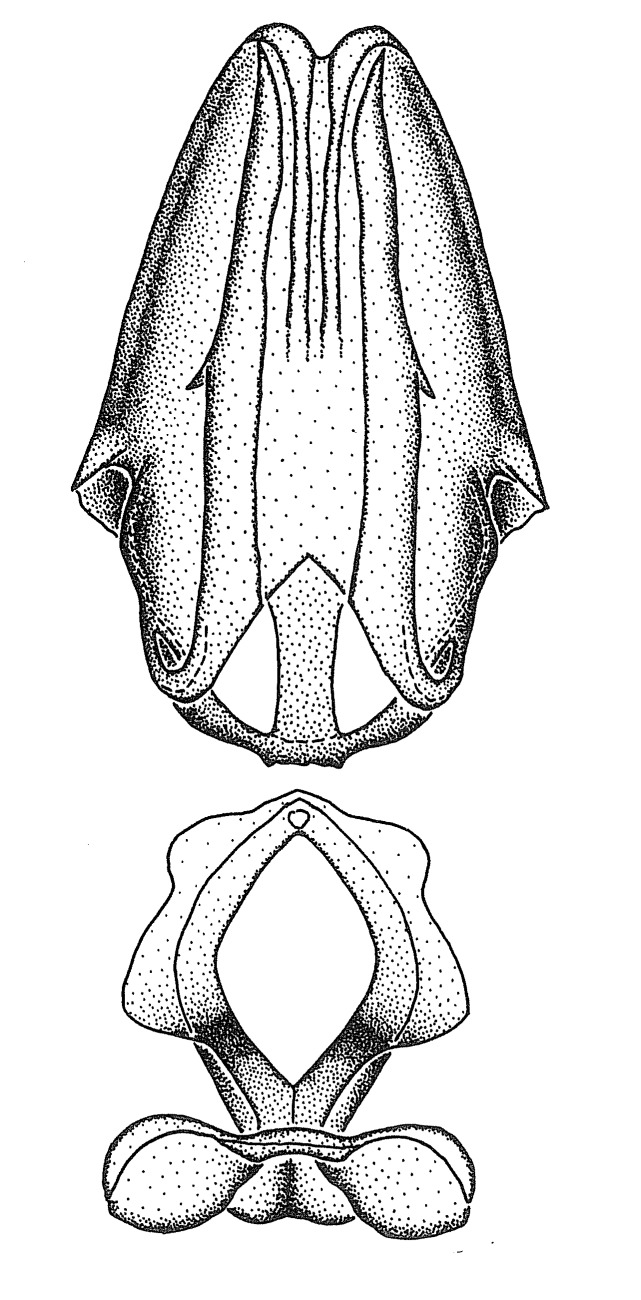
*Zelus paracephalus* Zhang & Hart, sp. n., phallus, dorsal view

**Figure 156a.**
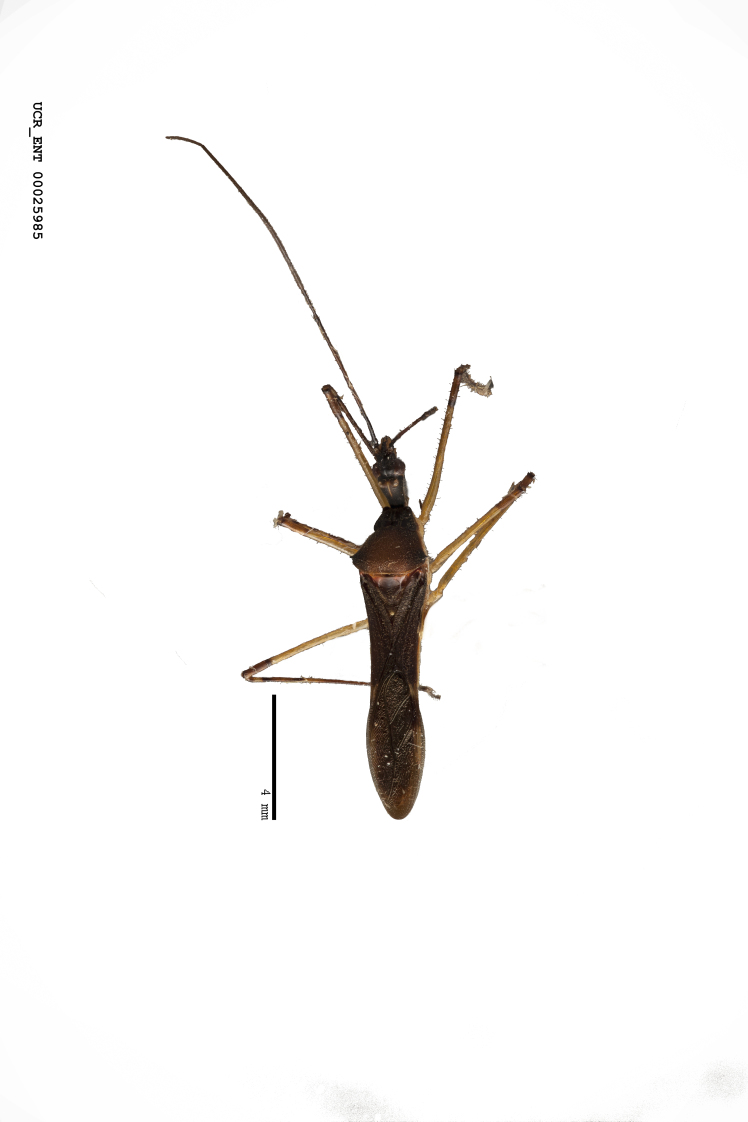
*Zelus pedestris* Fabricius, 1803, male, dorsal view (UCR_ENT 00025985, Paraguay)

**Figure 156b.**
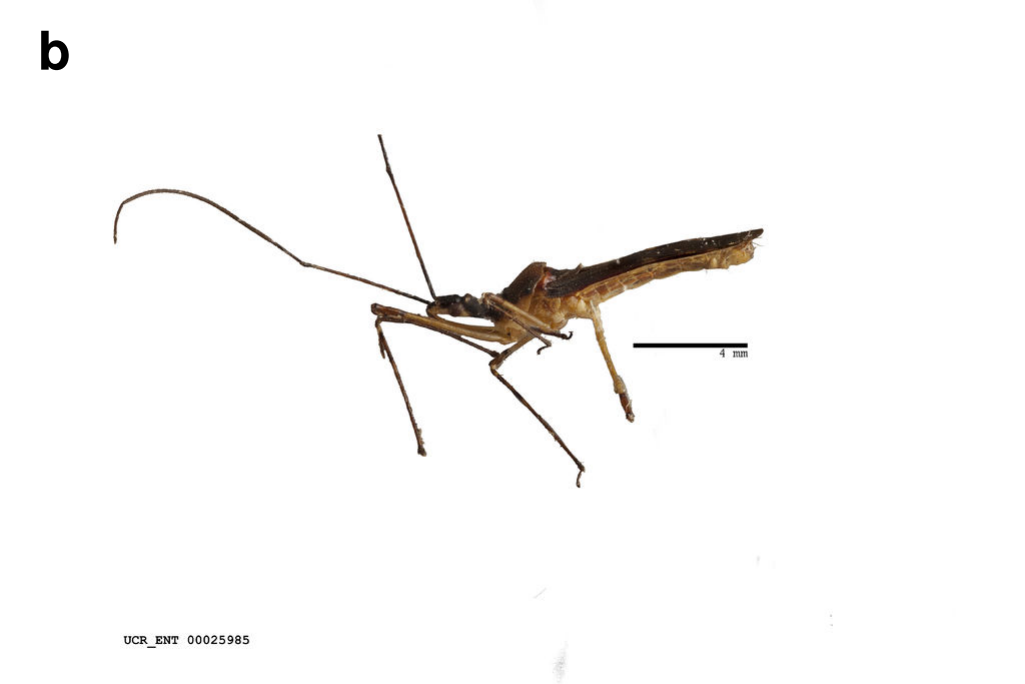
*Zelus pedestris* Fabricius, 1803, male, lateral view (UCR_ENT 00025985, Paraguay)

**Figure 156c.**
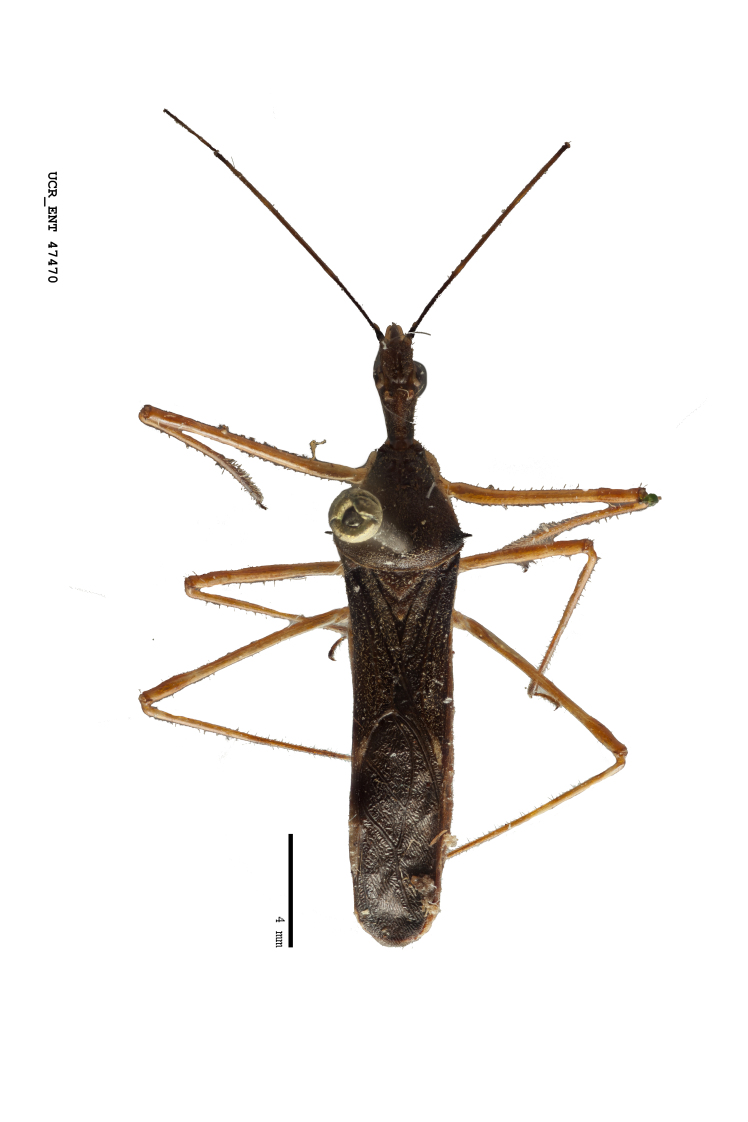
*Zelus pedestris* Fabricius, 1803, female, dorsal view (UCR_ENT 00047470, Brazil)

**Figure 156d.**
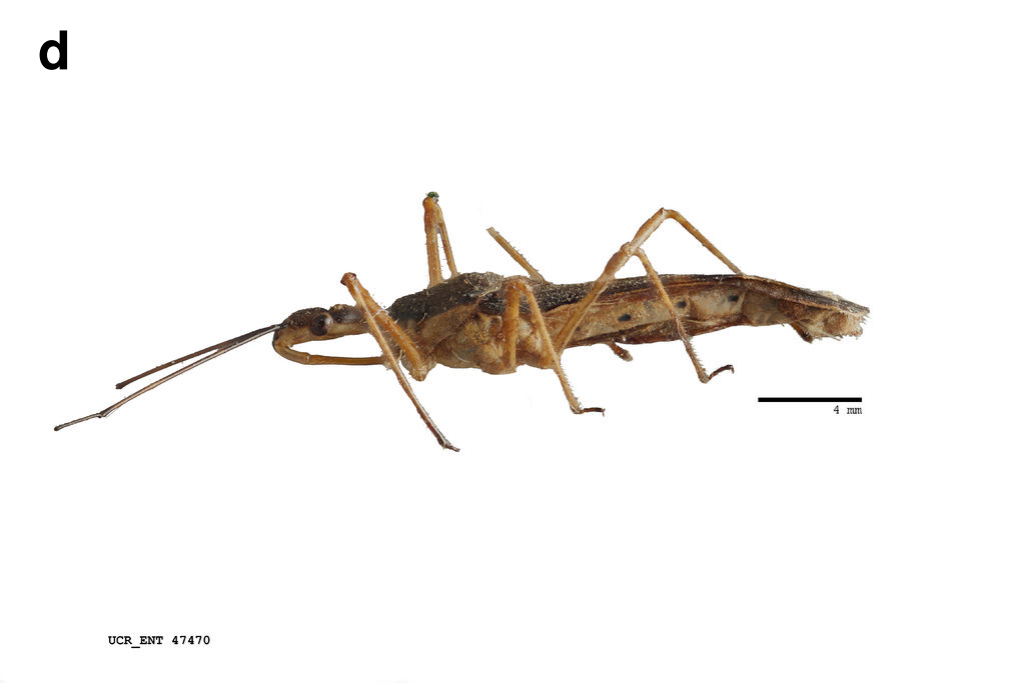
*Zelus pedestris* Fabricius, 1803, female, lateral view (UCR_ENT 00047470, Brazil)

**Figure 157a.**
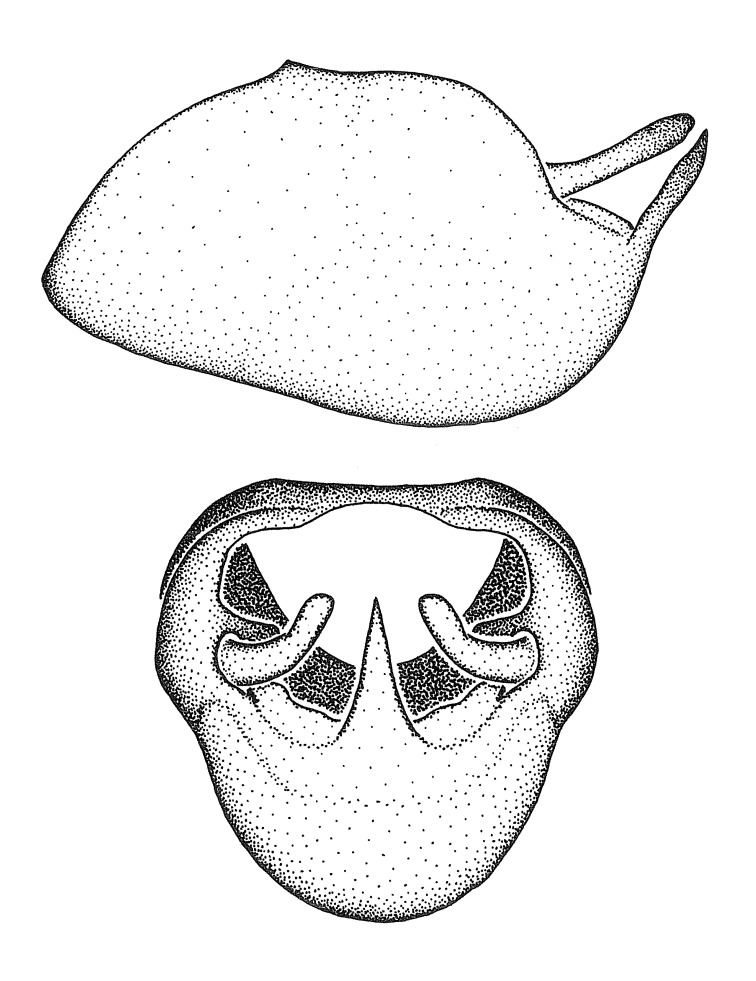
*Zelus pedestris* Fabricius, 1803, pygophore, lateral and posterior views

**Figure 157b.**
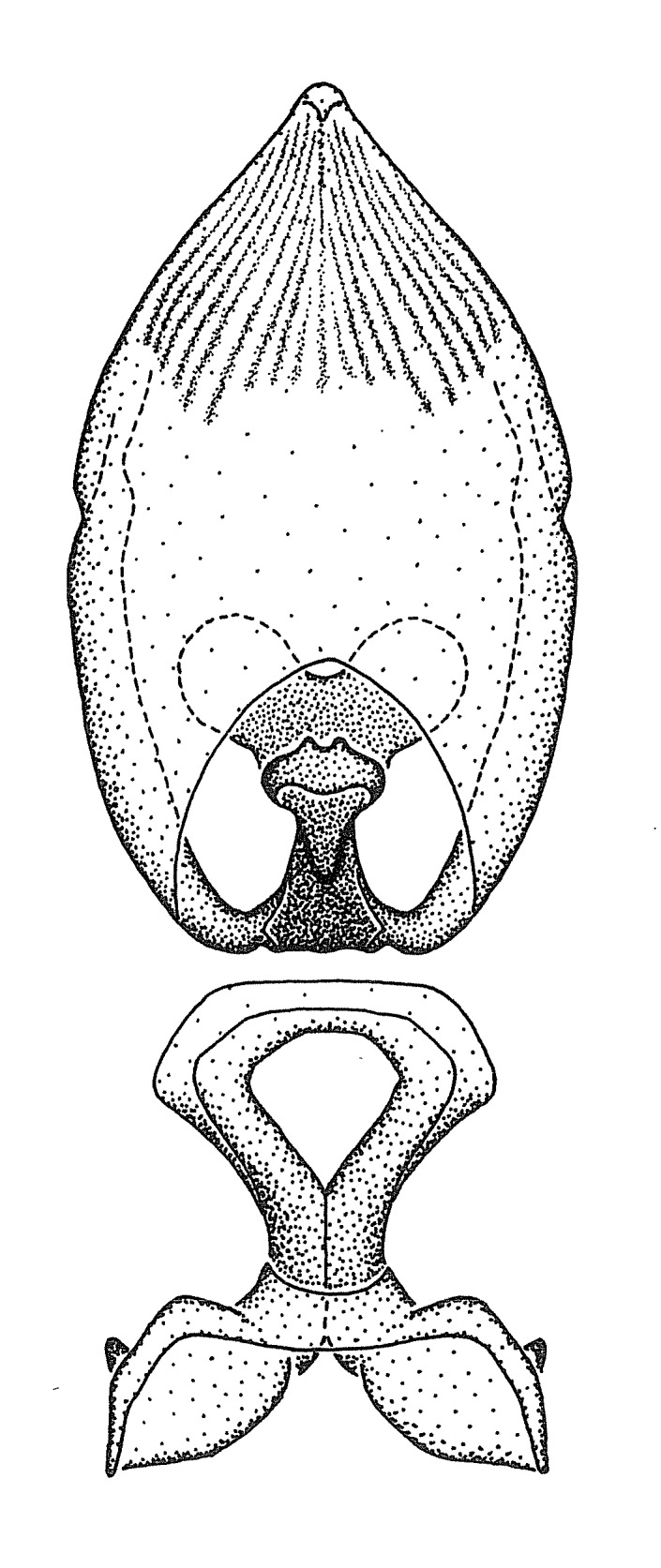
*Zelus pedestris* Fabricius, 1803, phallus, dorsal view

**Figure 159a.**
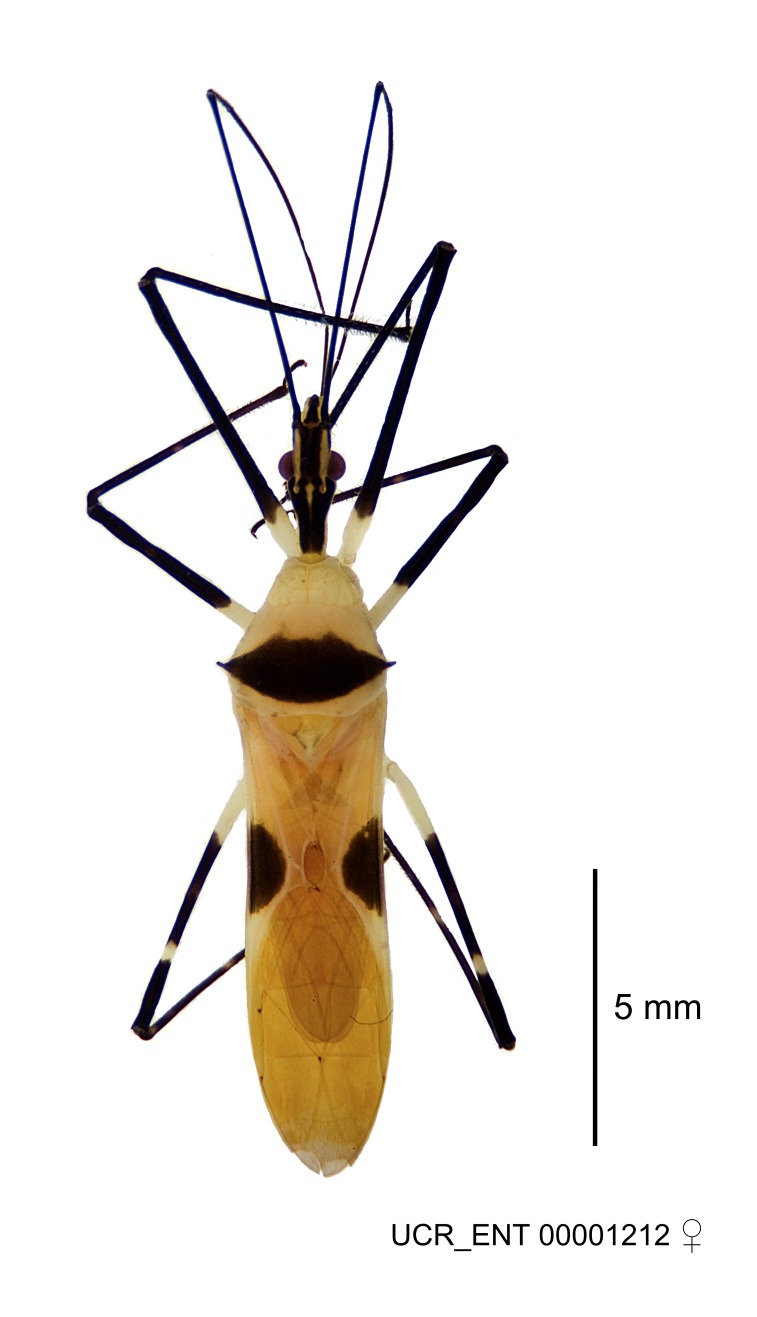
*Zelus plagiatus* (Signoret, 1852), female, dorsal view

**Figure 159b.**
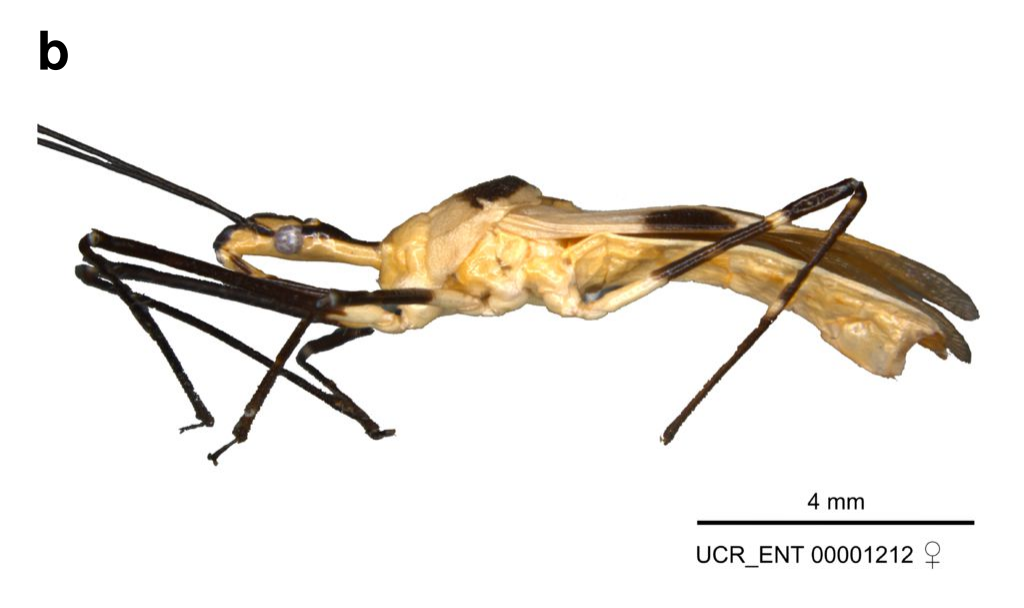
*Zelus plagiatus* (Signoret, 1852), feamle, lateral view

**Figure 161a.**
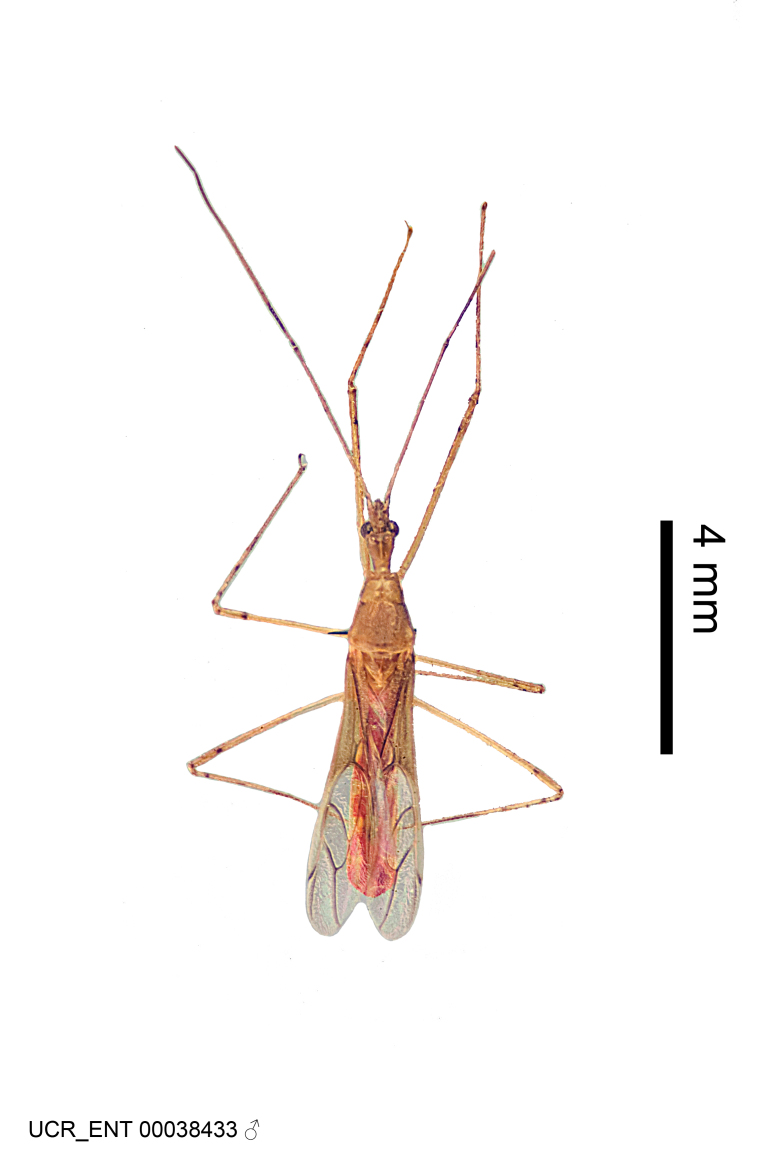
*Zelus prolixus* (Stål, 1860), male, dorsal view (UCR_ENT 00038433, Santa Catarina, Brazil)

**Figure 161b.**
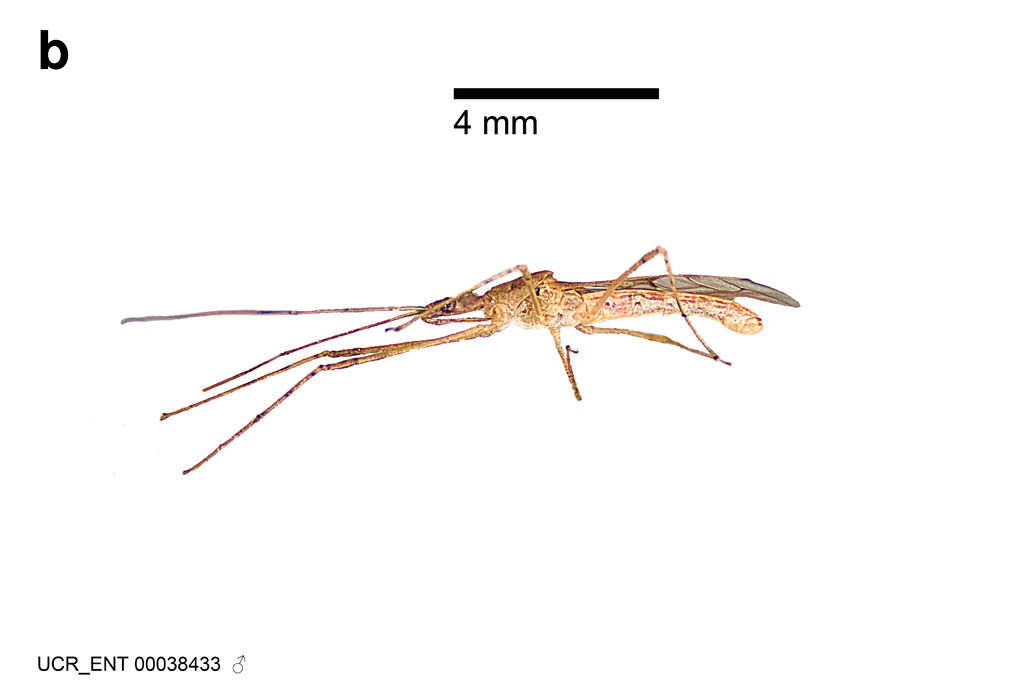
*Zelus prolixus* (Stål, 1860), male, lateral view (UCR_ENT 00038433, Santa Catarina, Brazil)

**Figure 161c.**
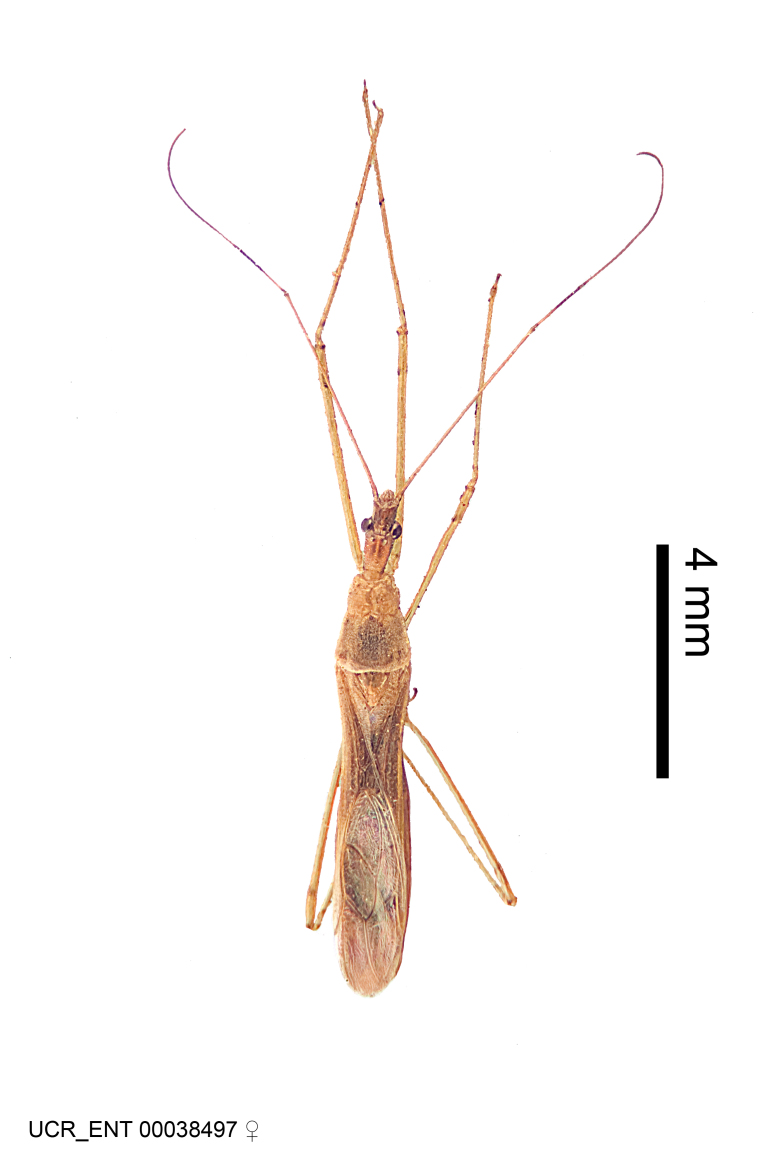
*Zelus prolixus* (Stål, 1860), female, dorsal view (UCR_ENT 00038497, Santa Catarina, Brazil)

**Figure 161d.**
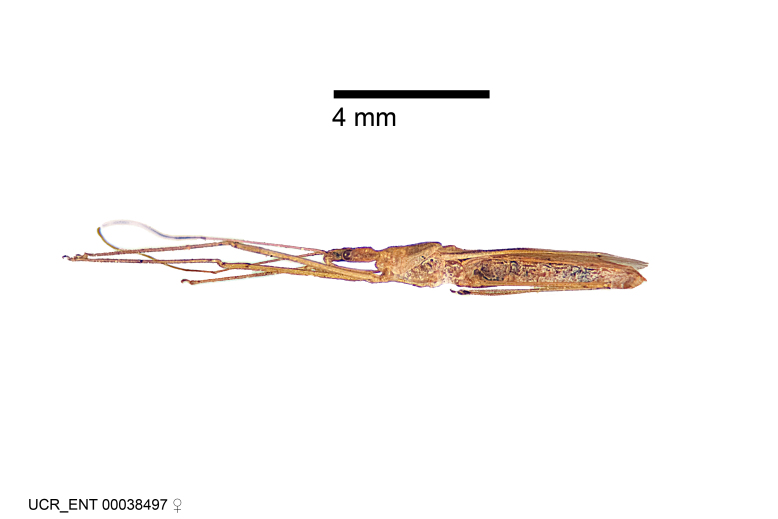
*Zelus prolixus* (Stål, 1860), female, lateral view (UCR_ENT 00038497, Santa Catarina, Brazil)

**Figure 162a.**
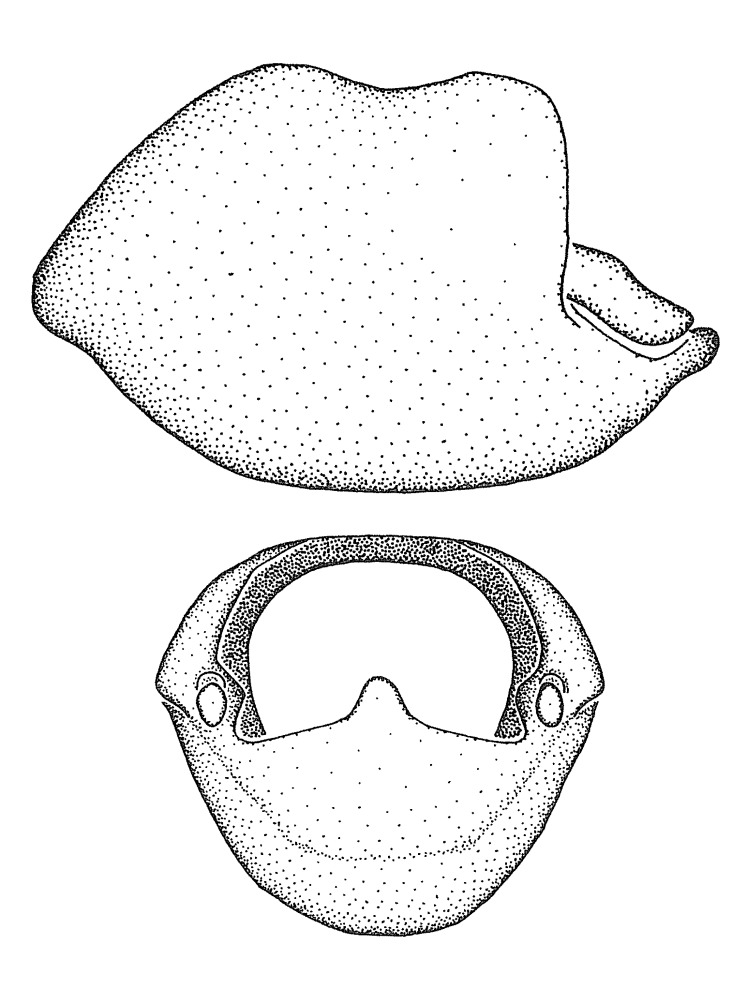
*Zelus prolixus* (Stål, 1860), pygophore, lateral and posterior views

**Figure 162b.**
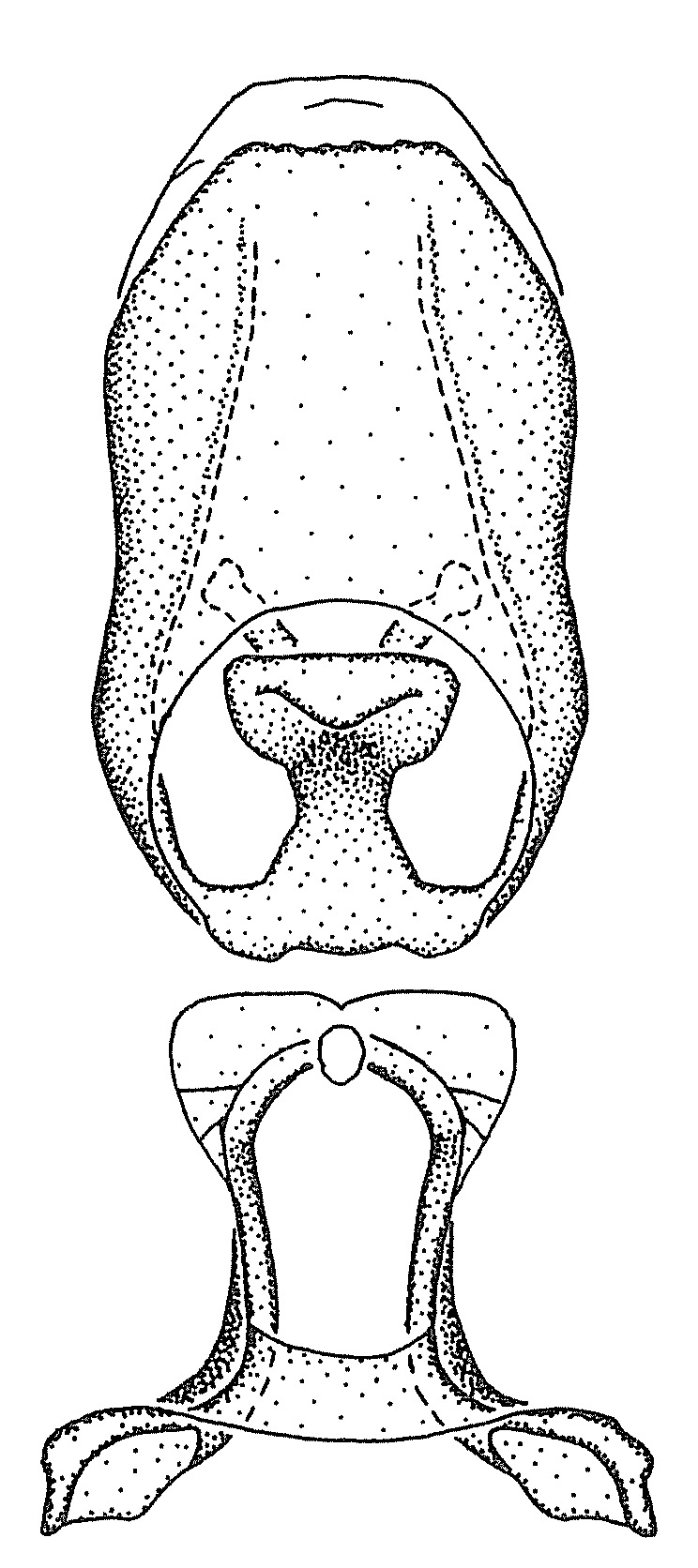
*Zelus prolixus* (Stål, 1860), phallus, dorsal view

**Figure 164a.**
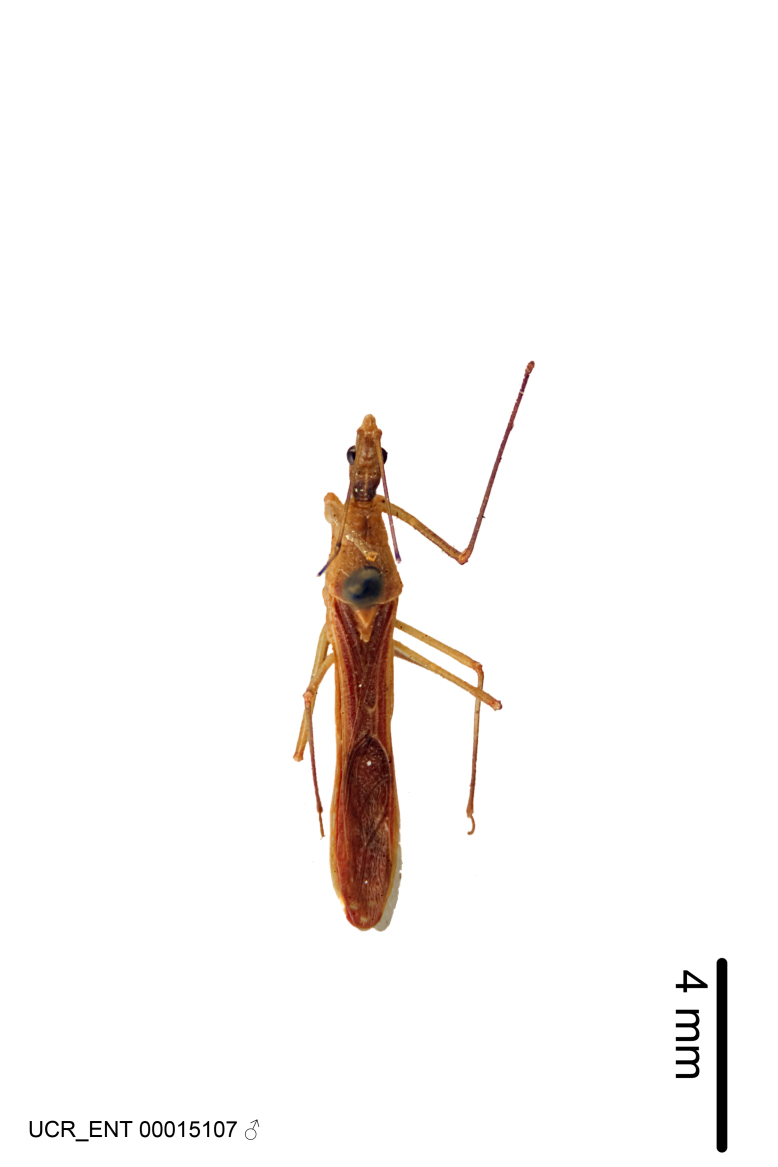
*Zelus puertoricensis* Hart, 1987, male, dorsal view (UCR_ENT 00015107, Dominican Republic)

**Figure 164b.**
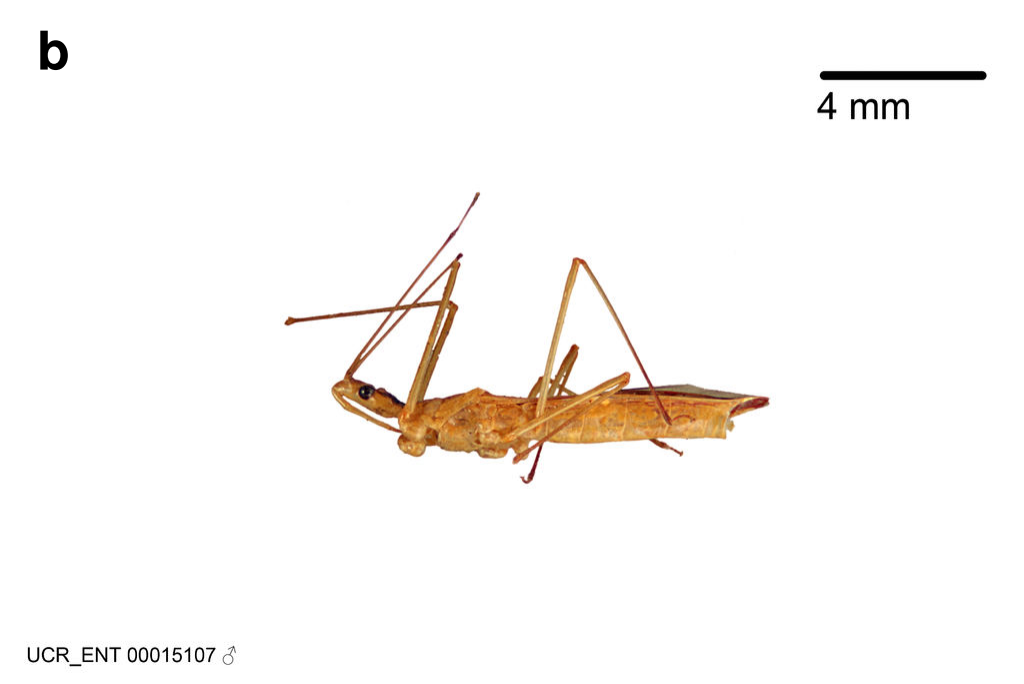
*Zelus puertoricensis* Hart, 1987, male, lateral view (UCR_ENT 00015107, Dominican Republic)

**Figure 164c.**
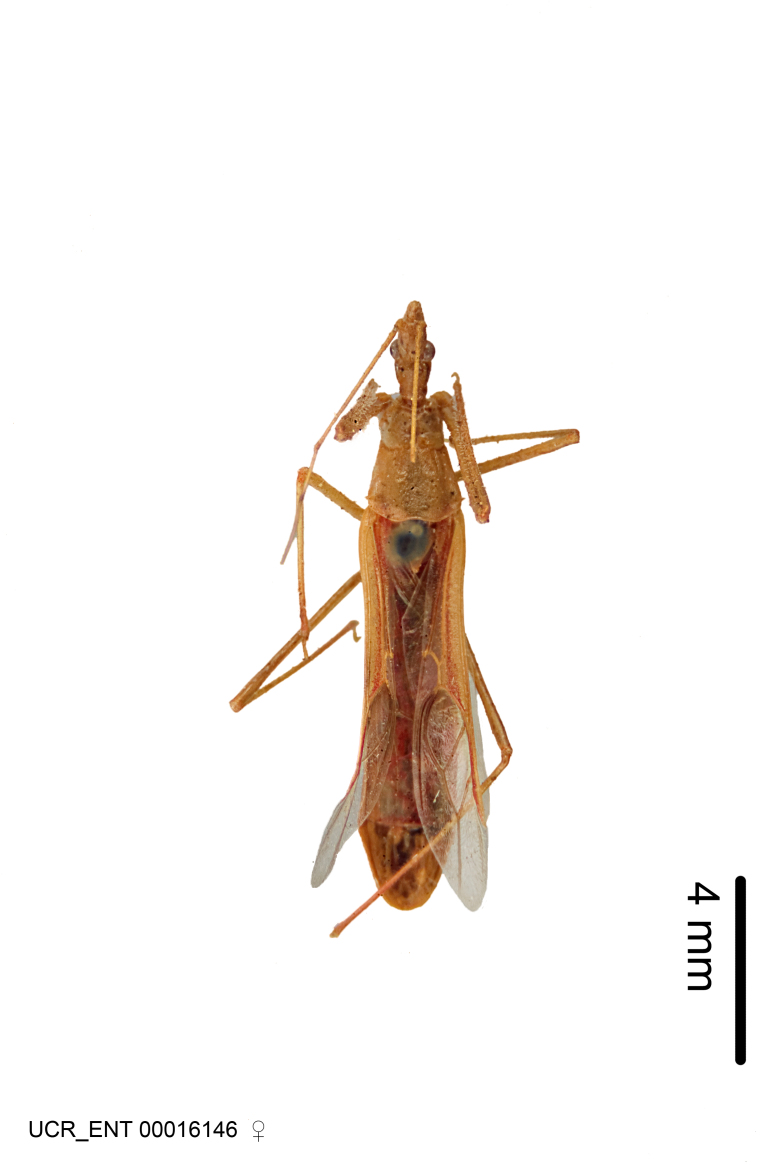
*Zelus puertoricensis* Hart, 1987, female, dorsal view (UCR_ENT 00016146, Puerto Rico)

**Figure 164d.**
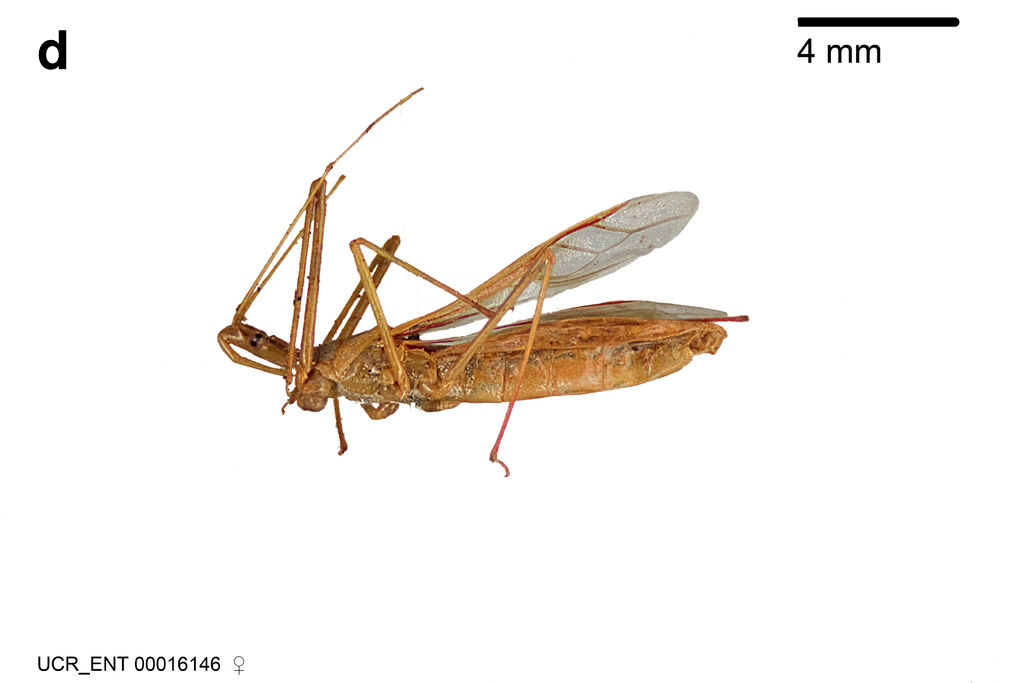
*Zelus puertoricensis* Hart, 1987, female, lateral view (UCR_ENT 00016146, Puerto Rico)

**Figure 165a.**
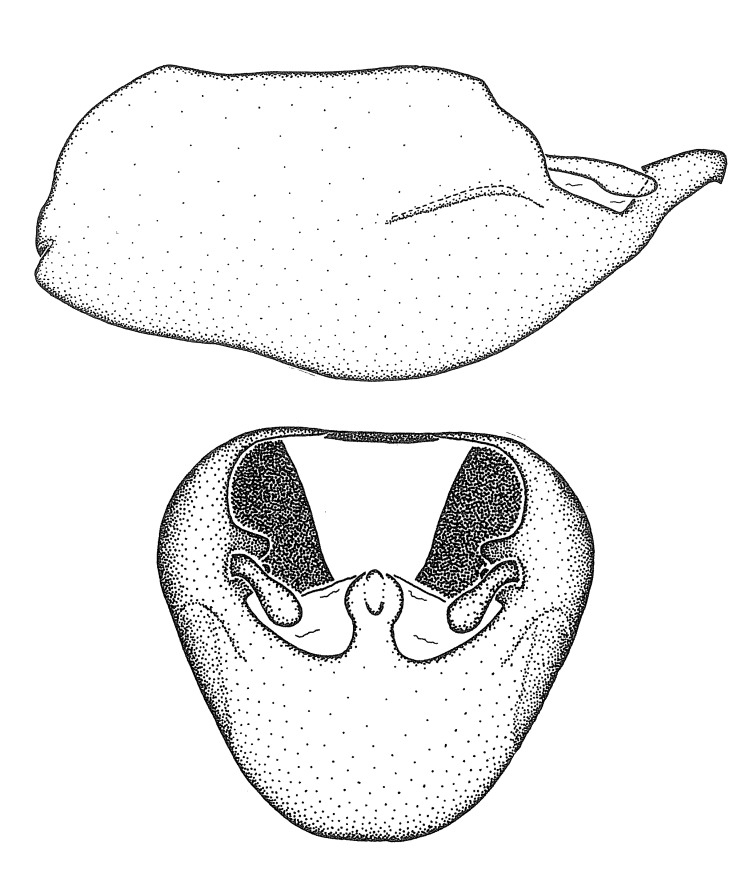
*Zelus puertoricensis* Hart, 1987, pygophore, lateral and posterior views

**Figure 165b.**
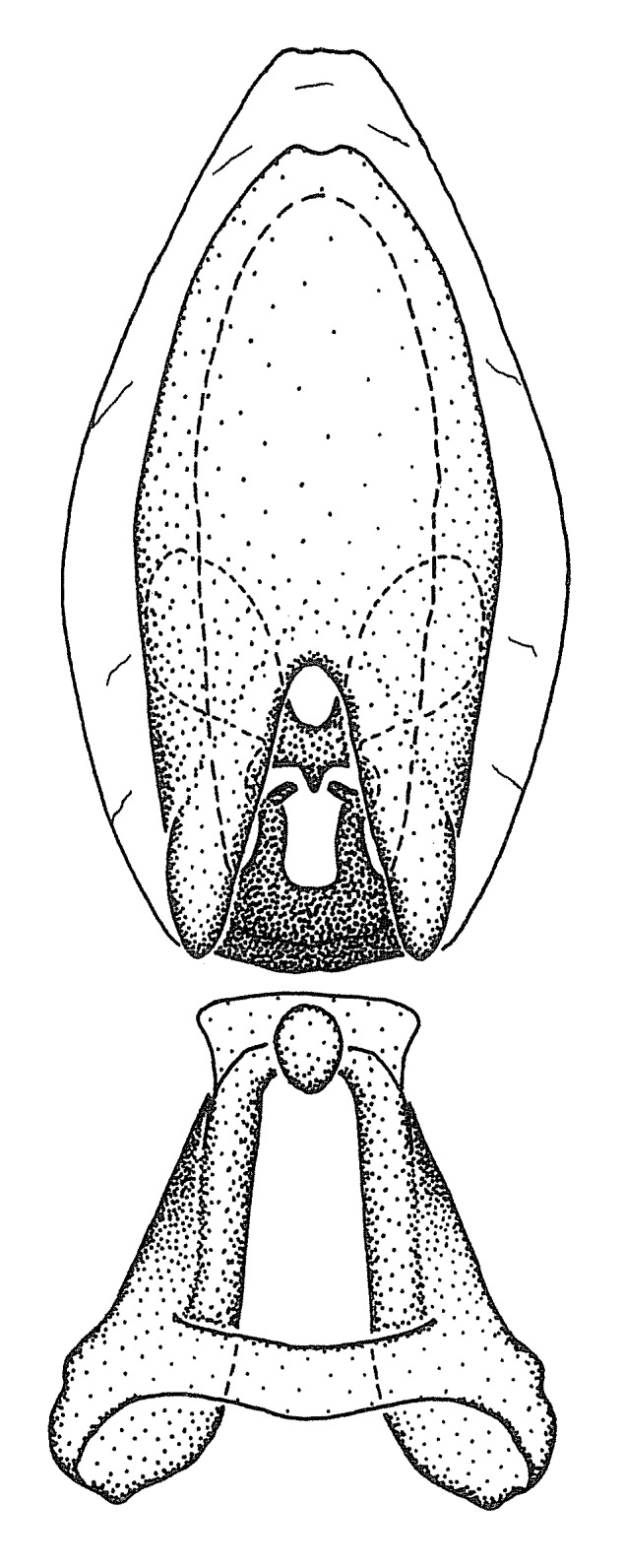
*Zelus puertoricensis* Hart, 1987, phallus, dorsal view

**Figure 167a.**
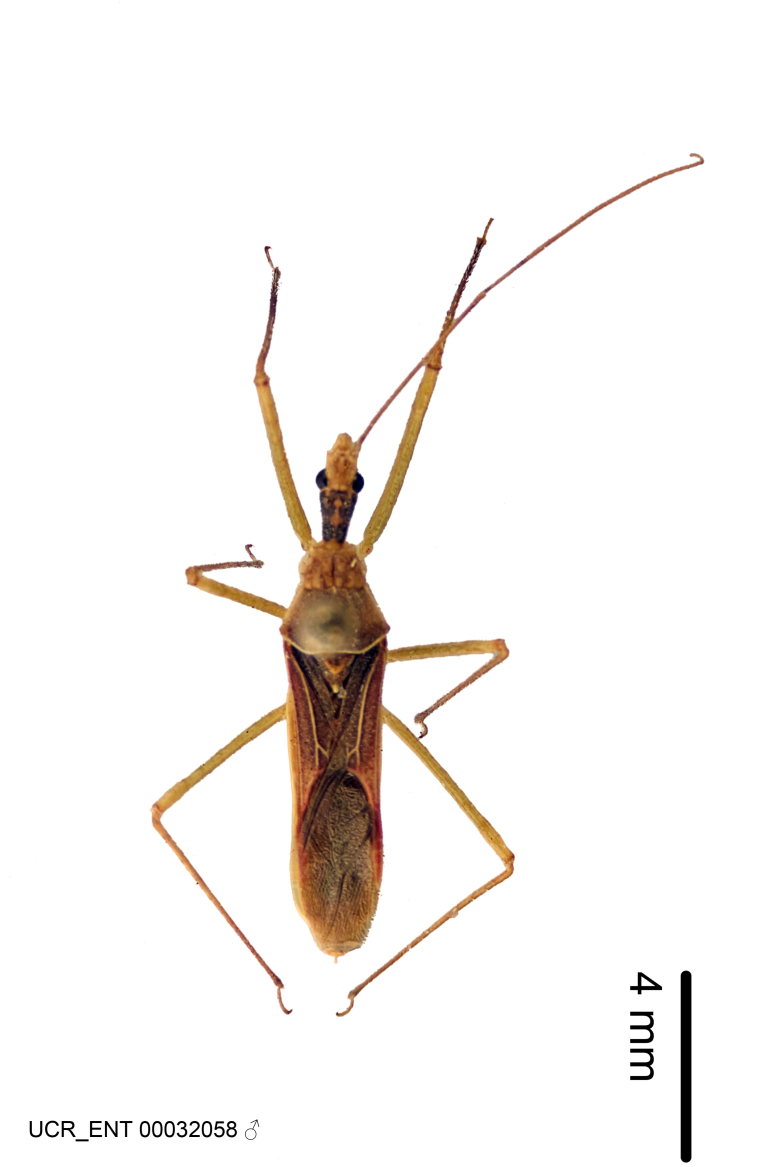
*Zelus renardii* Kolenati, 1857, male, dorsal view (UCR_ENT 00032058, Texas, USA)

**Figure 167b.**
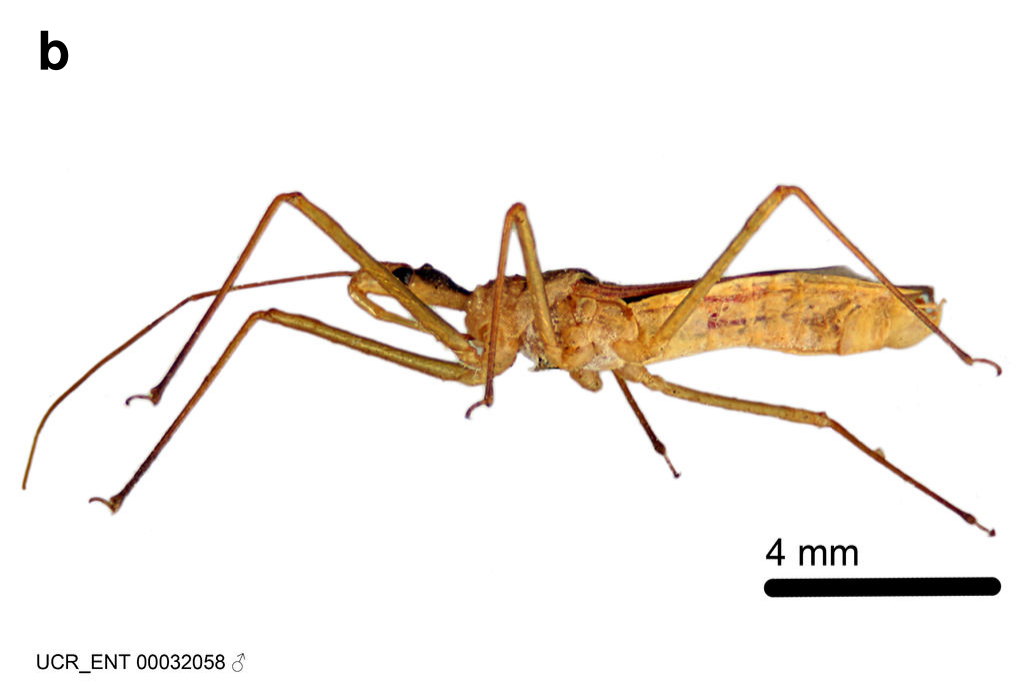
*Zelus renardii* Kolenati, 1857, male, lateral view (UCR_ENT 00032058, Texas, USA)

**Figure 167c.**
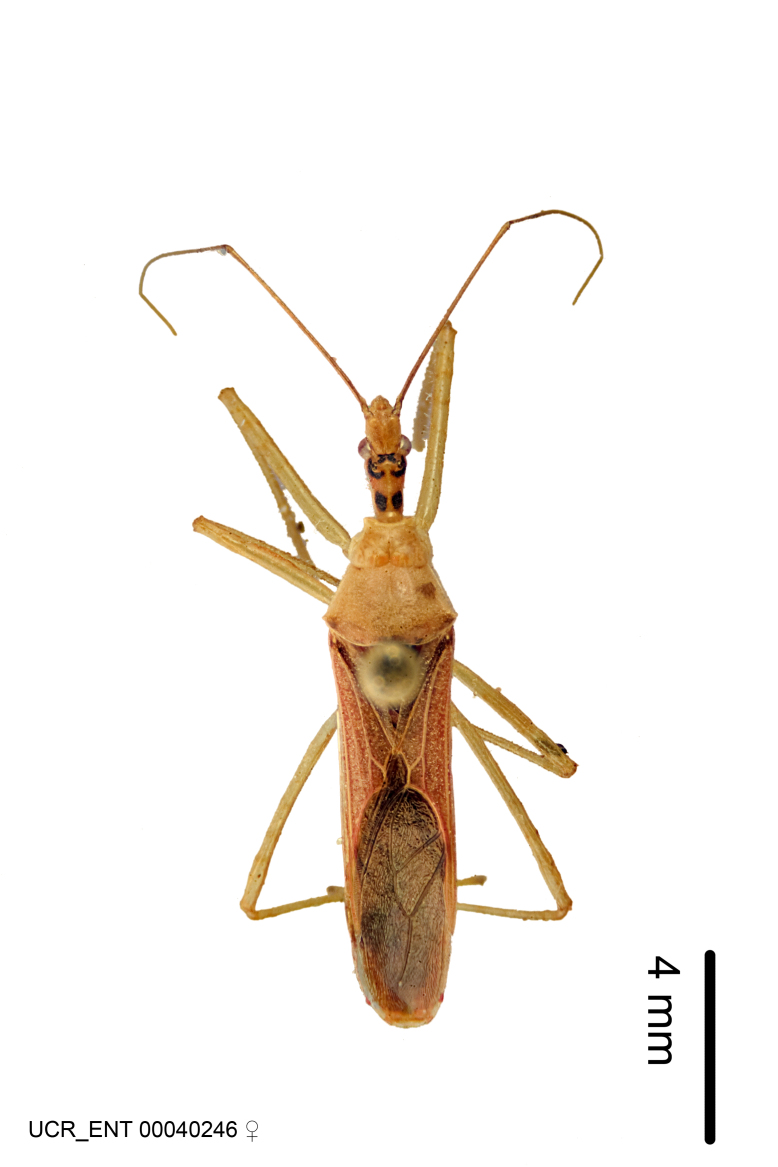
*Zelus renardii* Kolenati, 1857, female, dorsal view (UCR_ENT 00040246, New Mexico, USA)

**Figure 167d.**
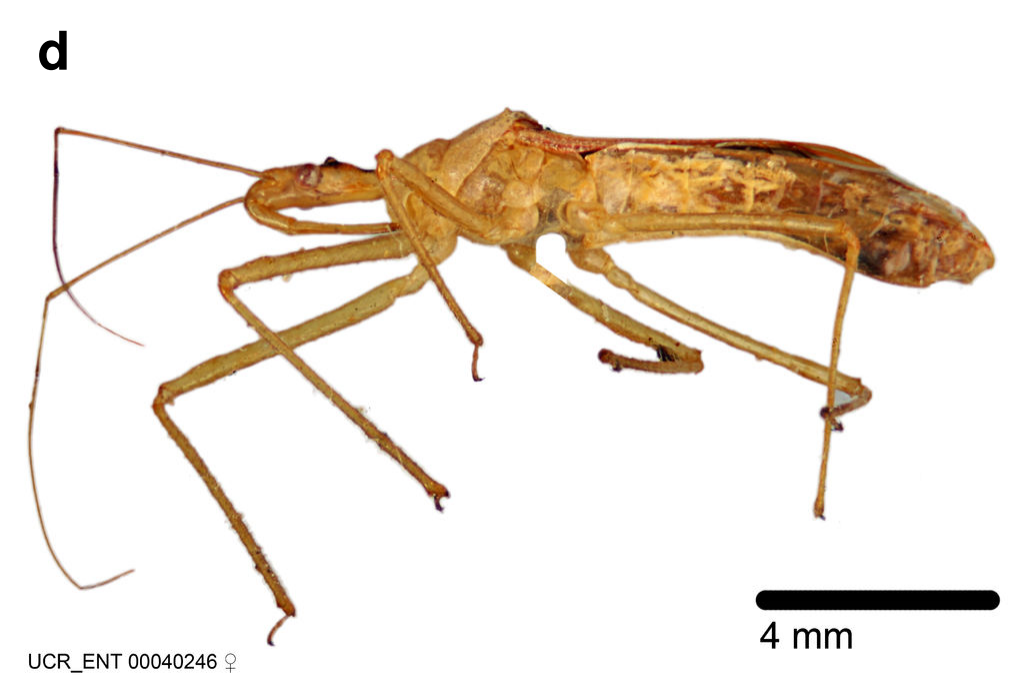
*Zelus renardii* Kolenati, 1857, female, lateral view (UCR_ENT 00040246, New Mexico, USA)

**Figure 168a.**
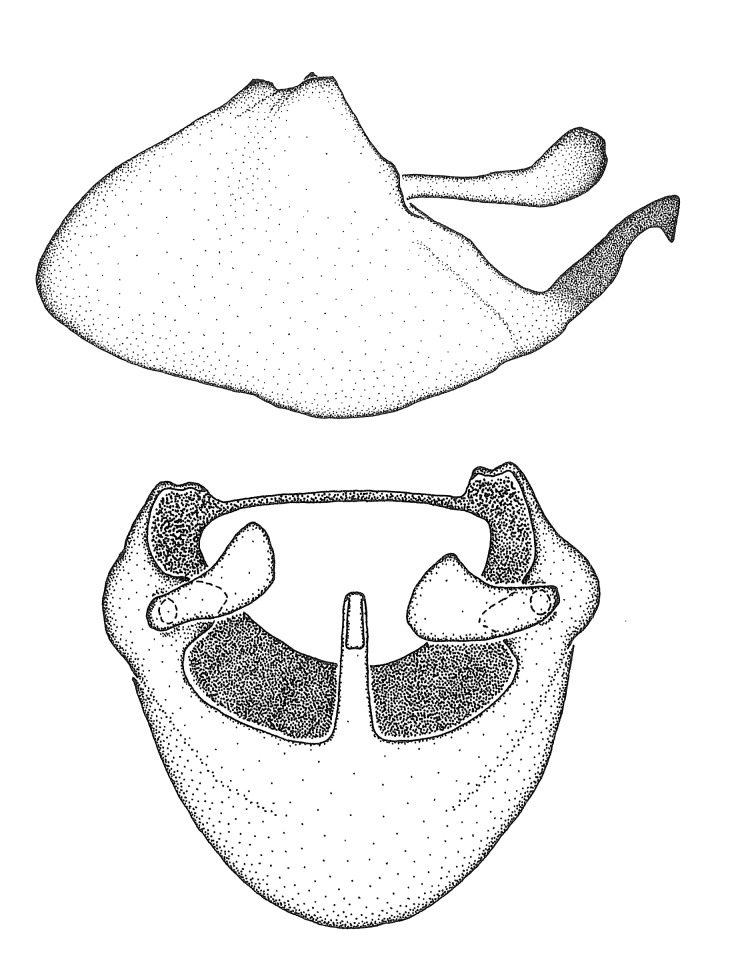
*Zelus renardii* Kolenati, 1857, pygophore, lateral and posterior views

**Figure 168b.**
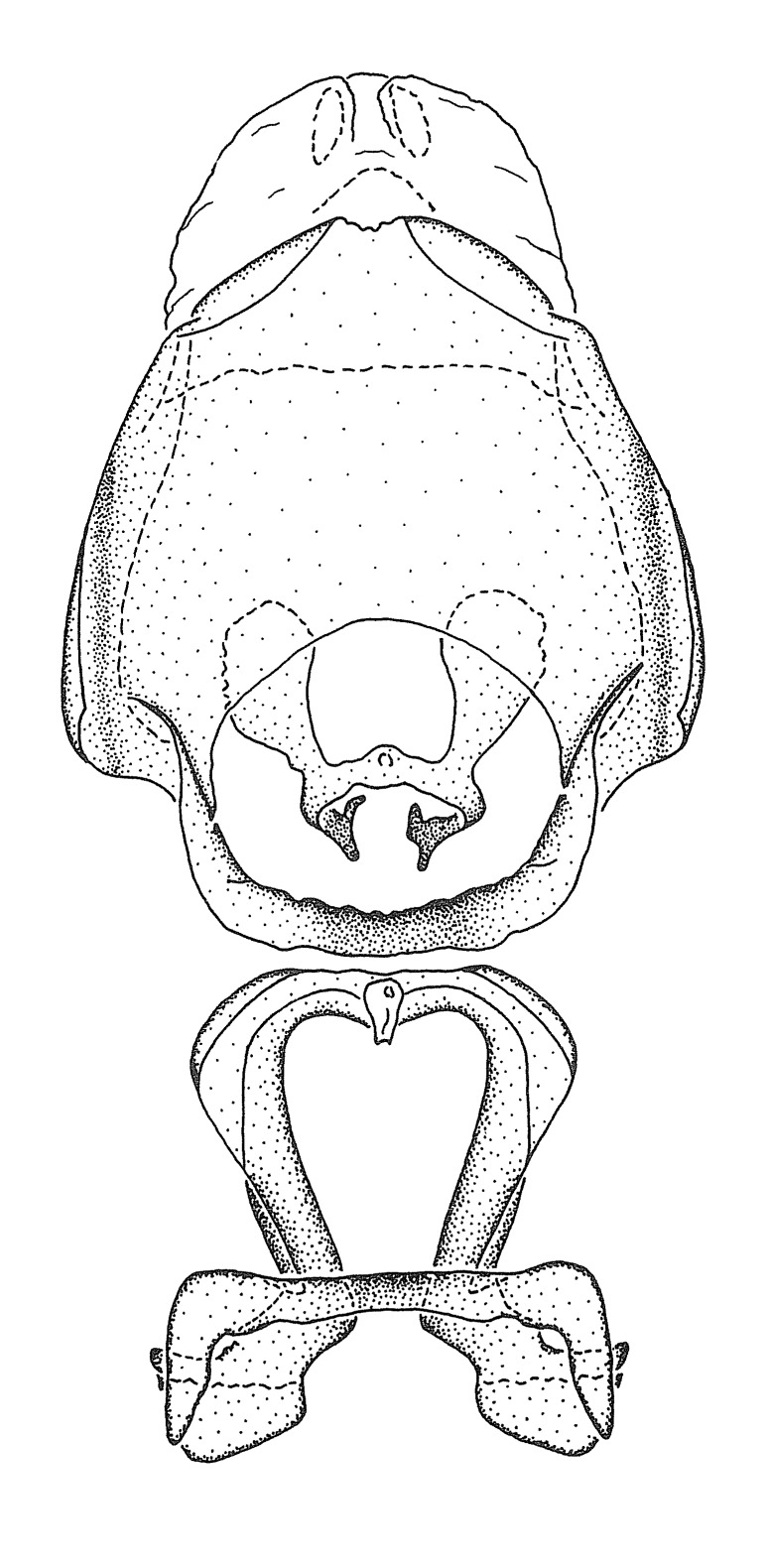
*Zelus renardii* Kolenati, 1857, phallus, dorsal view

**Figure 170a.**
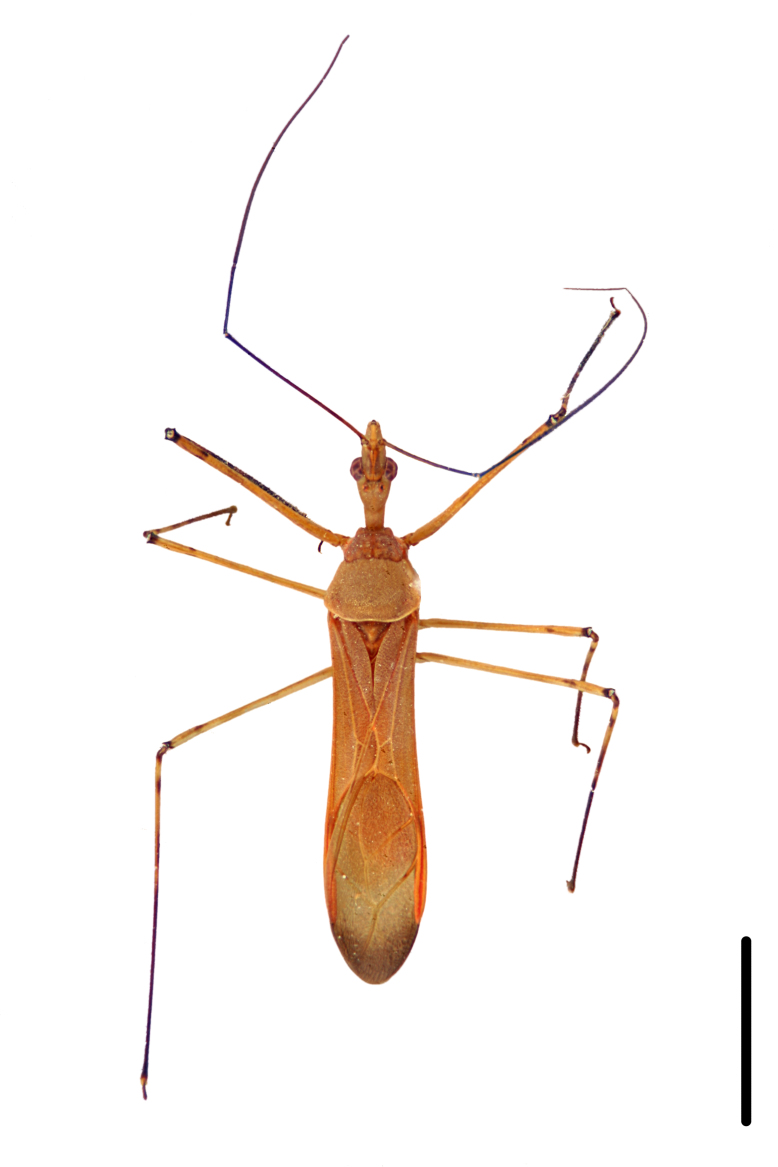
*Zelus rosulentus* Zhang & Hart, sp. n., male, dorsal view (UCR_ENT 00009487, Ecuador)

**Figure 170b.**
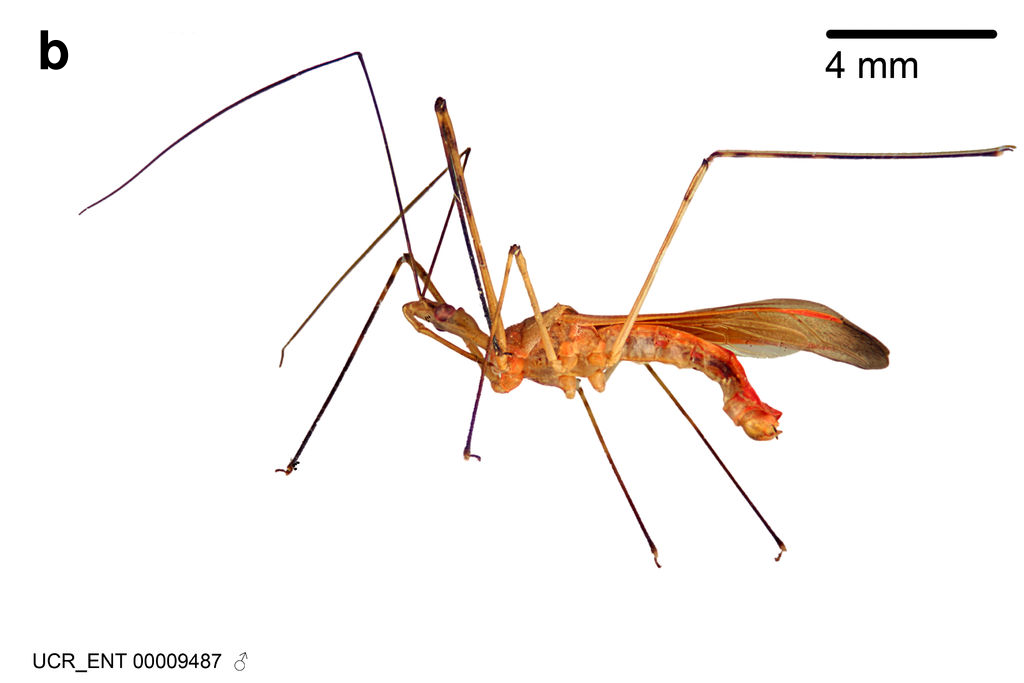
*Zelus rosulentus* Zhang & Hart, sp. n., male, lateral view (UCR_ENT 00009487, Ecuador)

**Figure 171a.**
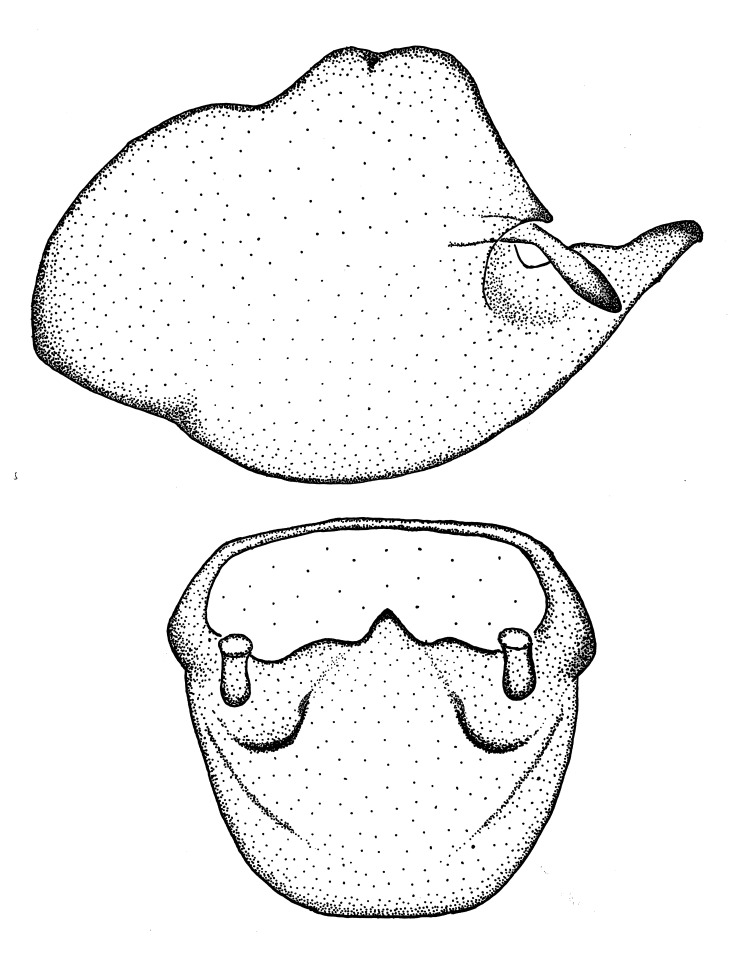
*Zelus rosulentus* Zhang & Hart, sp. n., pygophore, lateral and posterior views

**Figure 171b.**
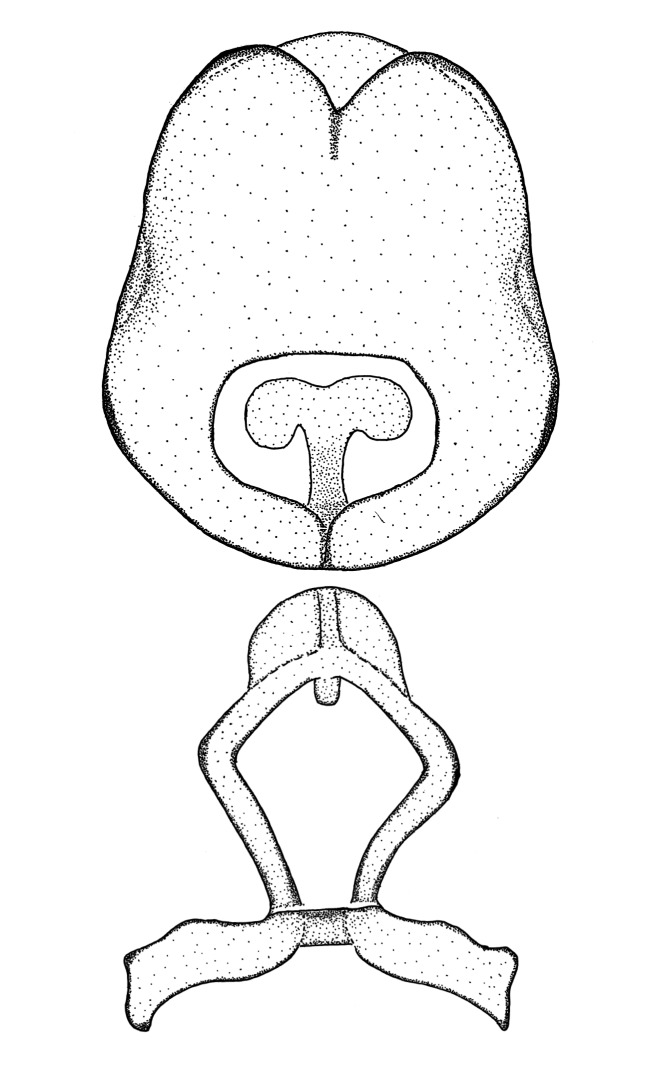
*Zelus rosulentus* Zhang & Hart, sp. n., phallus

**Figure 173a.**
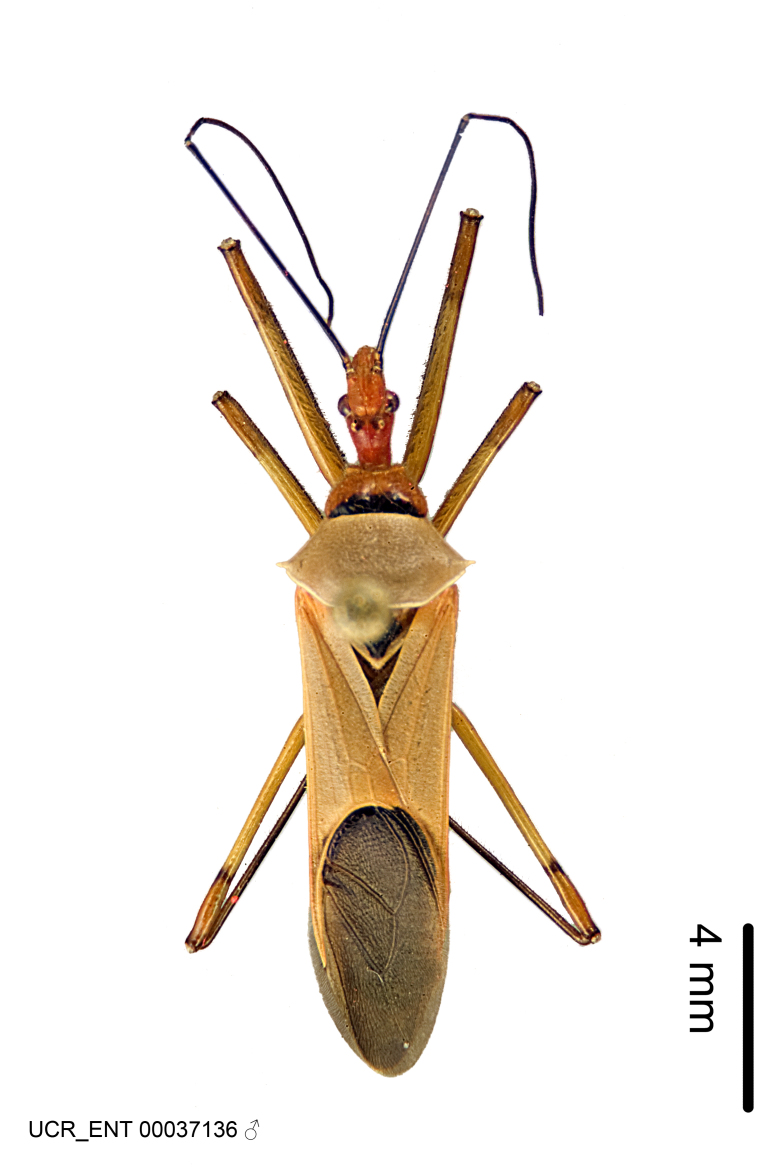
*Zelus ruficeps* Stål, 1862, male, dorsal view (UCR_ENT 00037136, Chimaltenango, Guatemala)

**Figure 173b.**
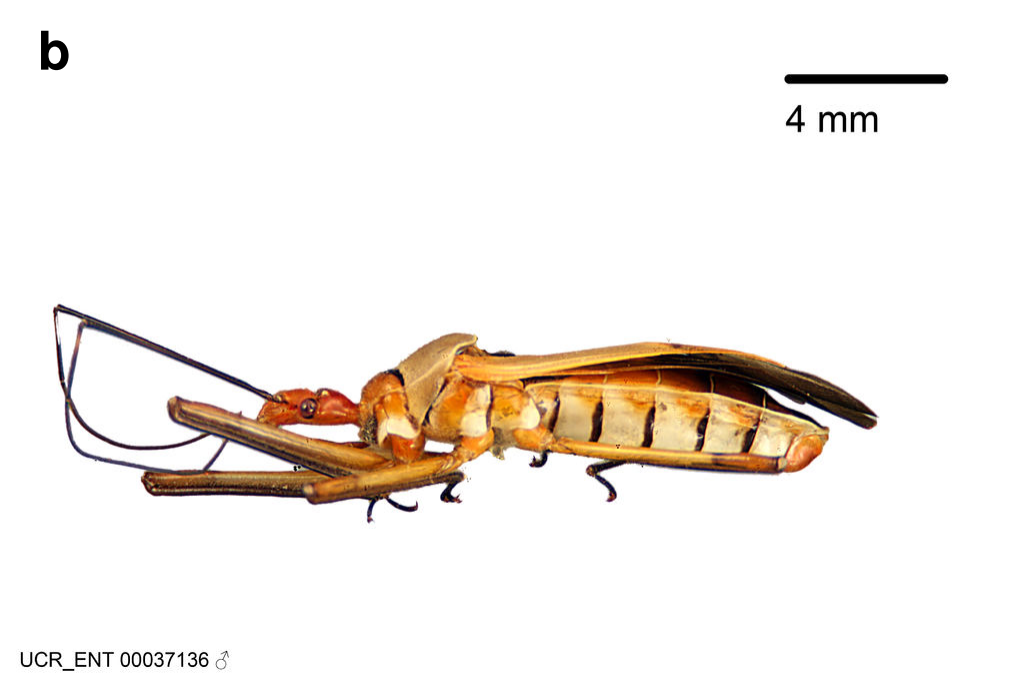
*Zelus ruficeps* Stål, 1862, male, lateral view (UCR_ENT 00037136, Chimaltenango, Guatemala)

**Figure 173c.**
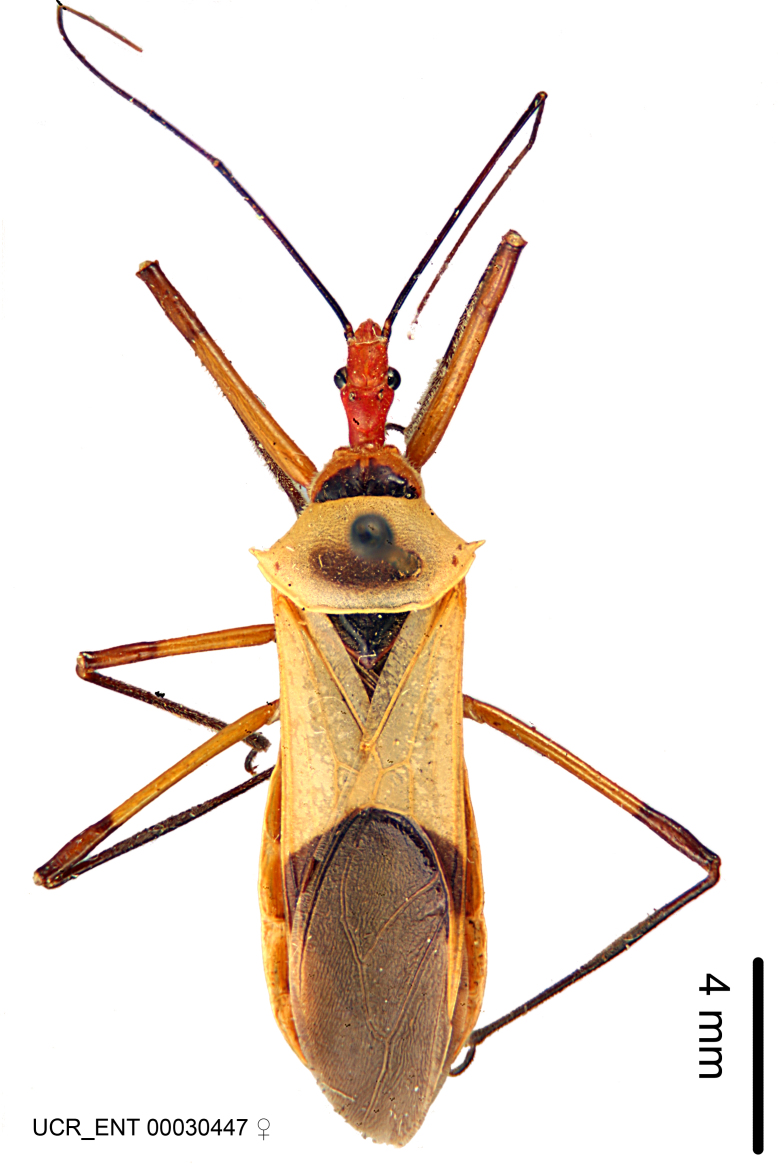
*Zelus ruficeps* Stål, 1862, female, dorsal view (UCR_ENT 00030447, Veracruz, Mexico)

**Figure 173d.**
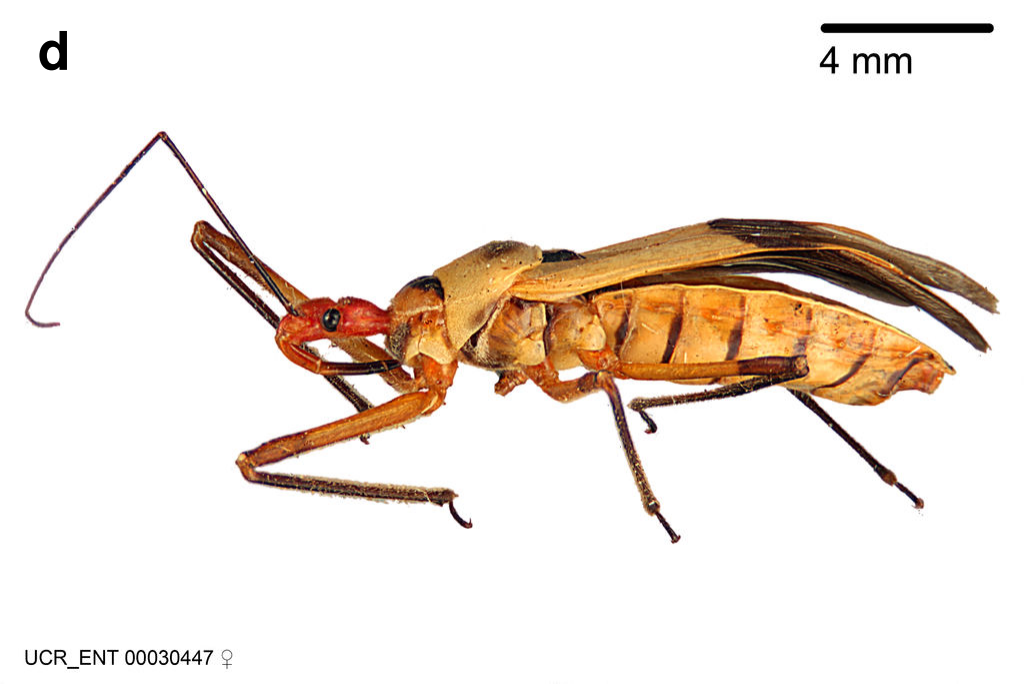
*Zelus ruficeps* Stål, 1862, female, lateral view (UCR_ENT 00030447, Veracruz, Mexico)

**Figure 173e.**
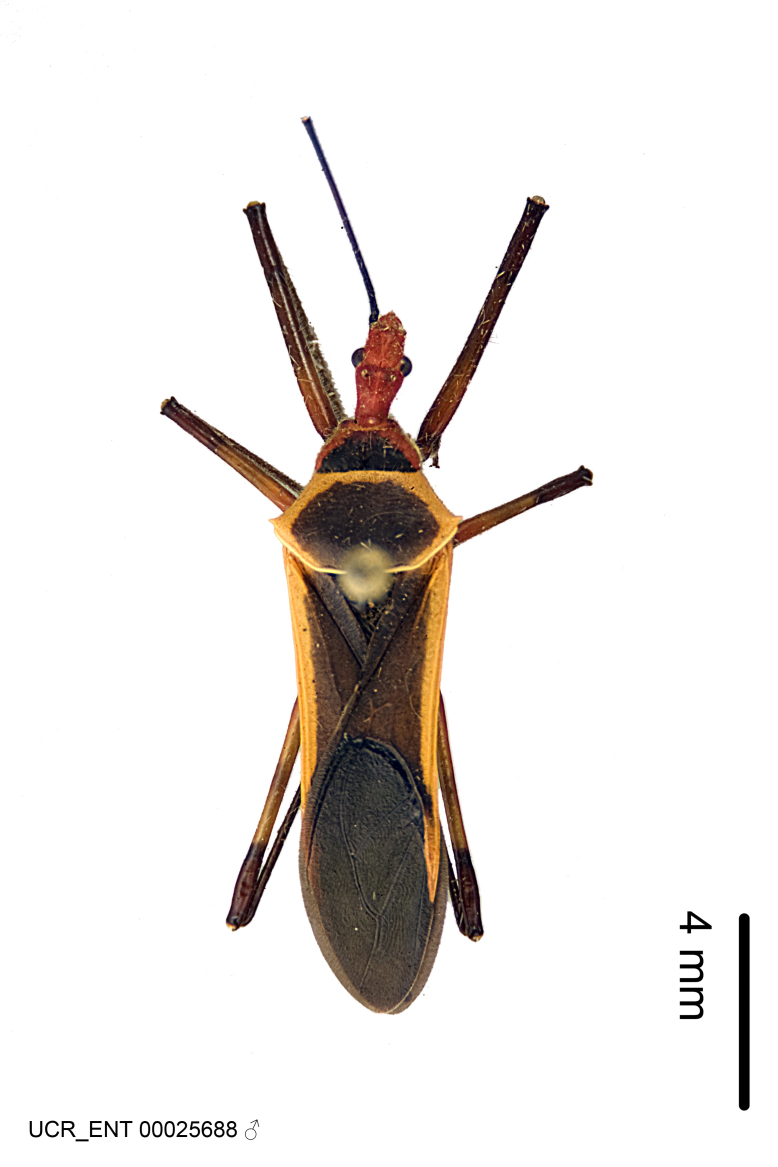
*Zelus ruficeps* Stål, 1862, male, dorsal view (UCR_ENT 00025688, Veracruz, Mexico)

**Figure 174a.**
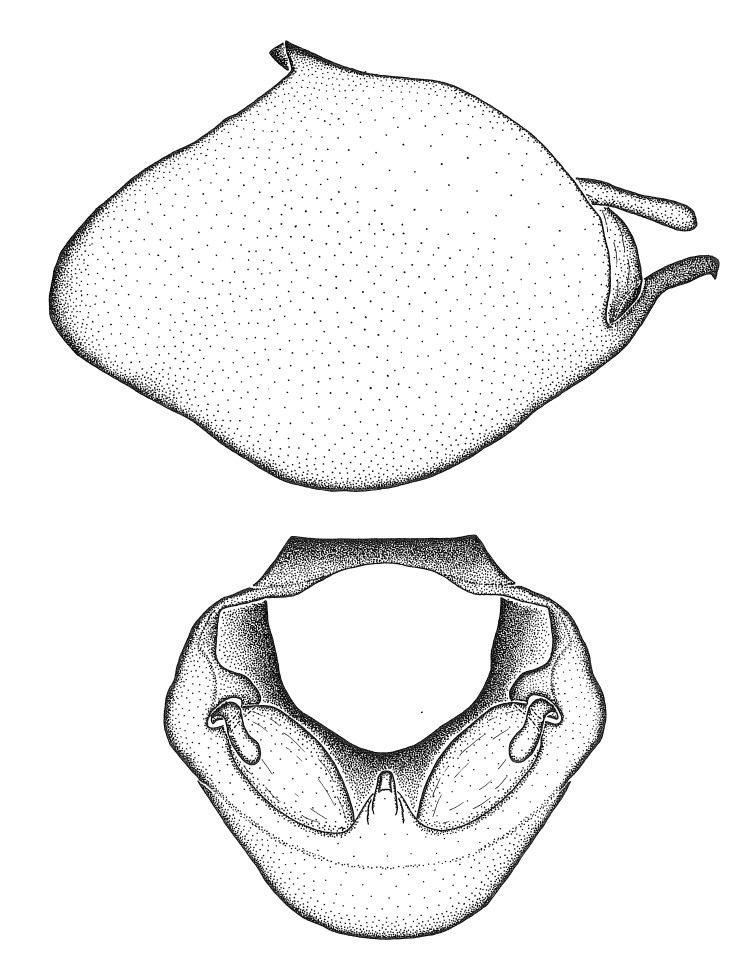
*Zelus ruficeps* Stål, 1862, pygophore, lateral and posterior views

**Figure 174b.**
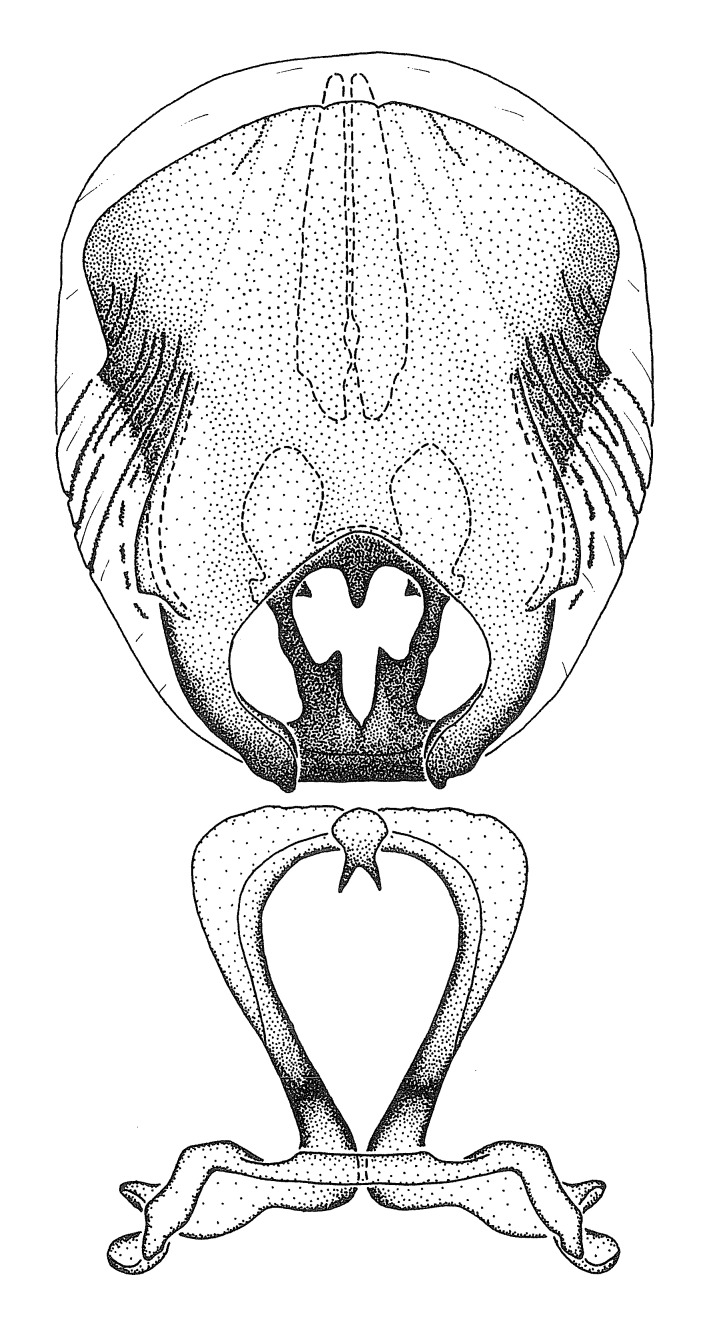
*Zelus ruficeps* Stål, 1862, phallus

**Figure 176a.**
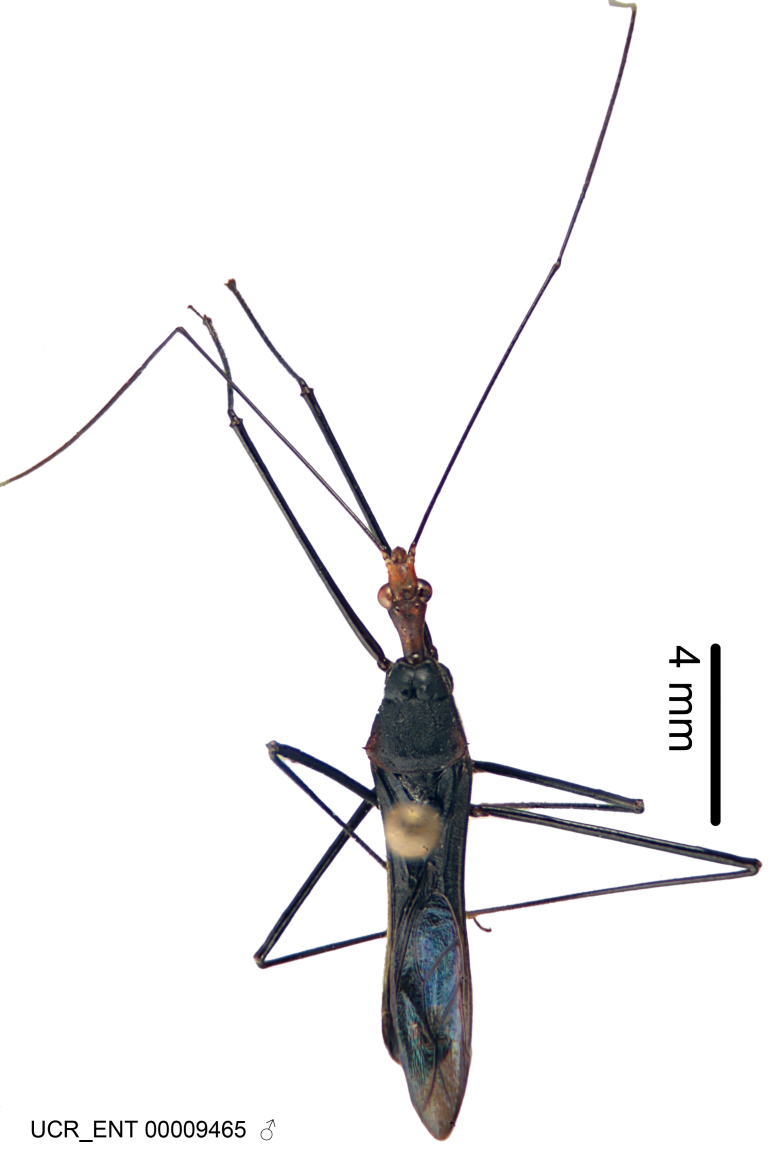
*Zelus russulumus* Zhang & Hart, sp. n., male, dorsal view (UCR_ENT 00009465, Rondônia, Brazil)

**Figure 176b.**
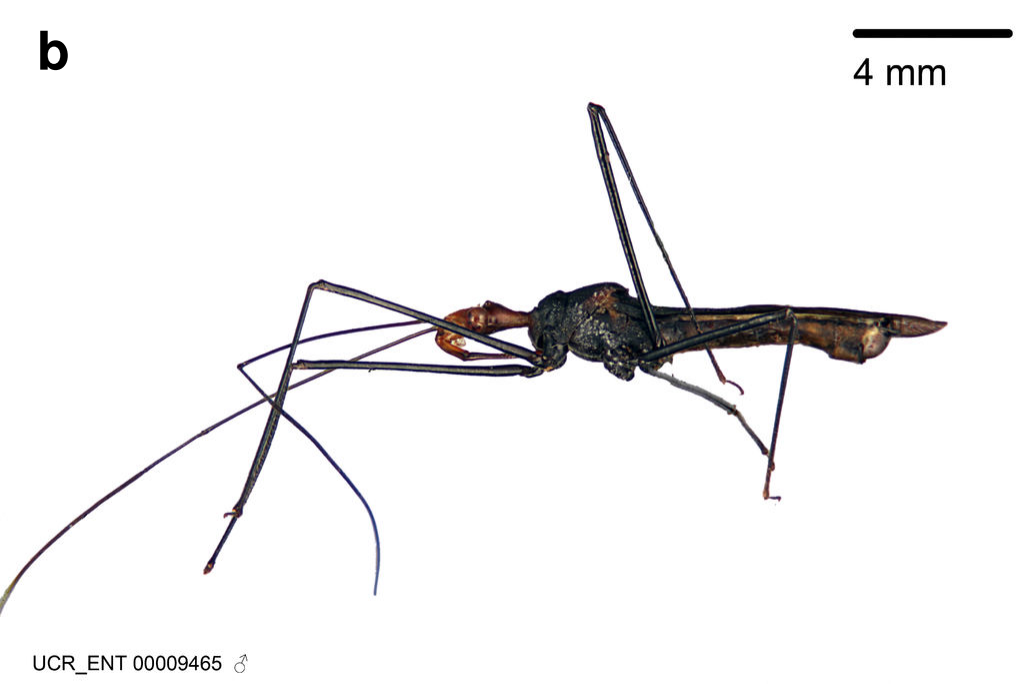
*Zelus russulumus* Zhang & Hart, sp. n., male, lateral view (UCR_ENT 00009465, Rondônia, Brazil

**Figure 176c.**
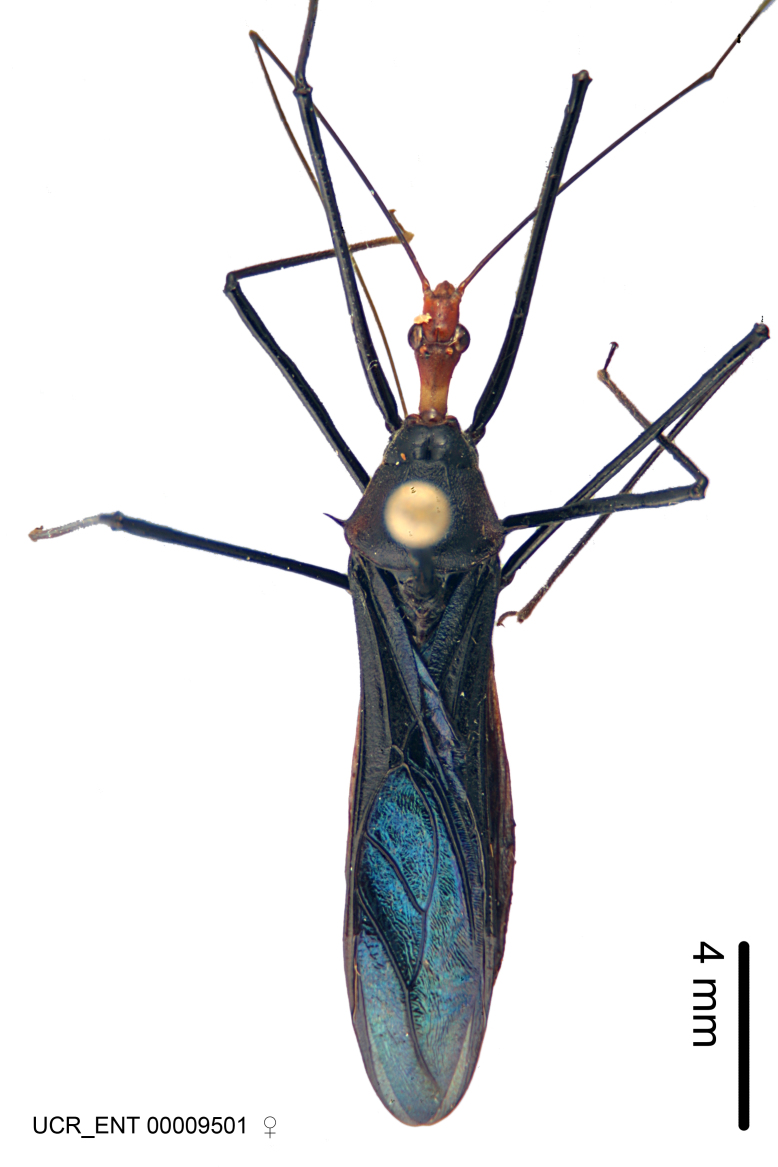
*Zelus russulumus* Zhang & Hart, sp. n., female, dorsal view (UCR_ENT 00009501, Napo, Ecuador)

**Figure 176d.**
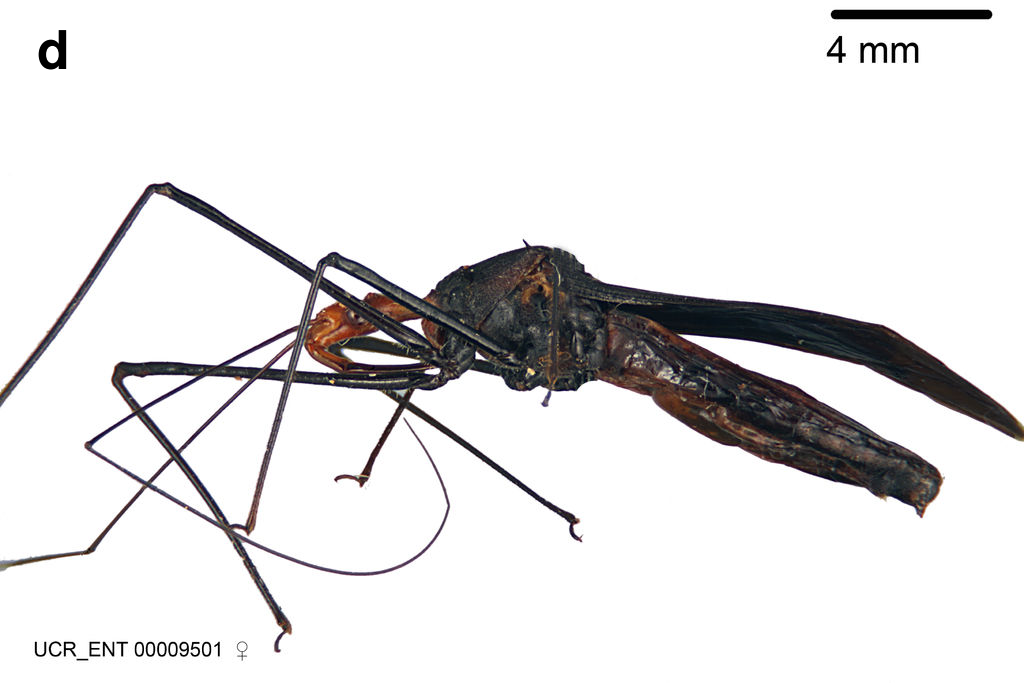
*Zelus russulumus* Zhang & Hart, sp. n., female, lateral view (UCR_ENT 00009501, Napo, Ecuador)

**Figure 177a.**
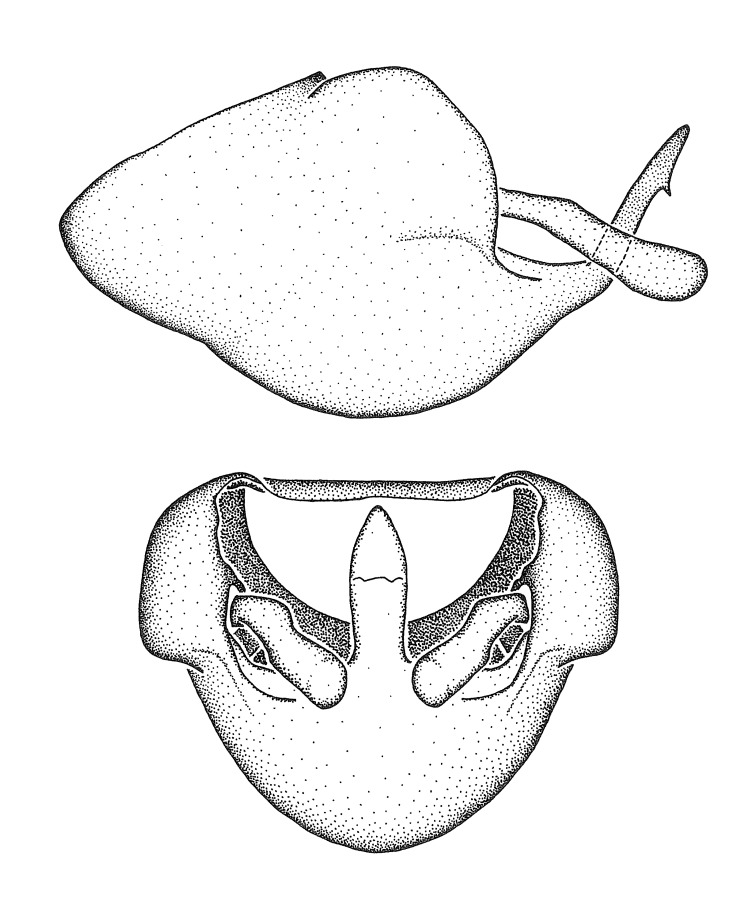
*Zelus russulumus* Zhang & Hart, sp. n., pygophore, lateral and posterior views

**Figure 177b.**
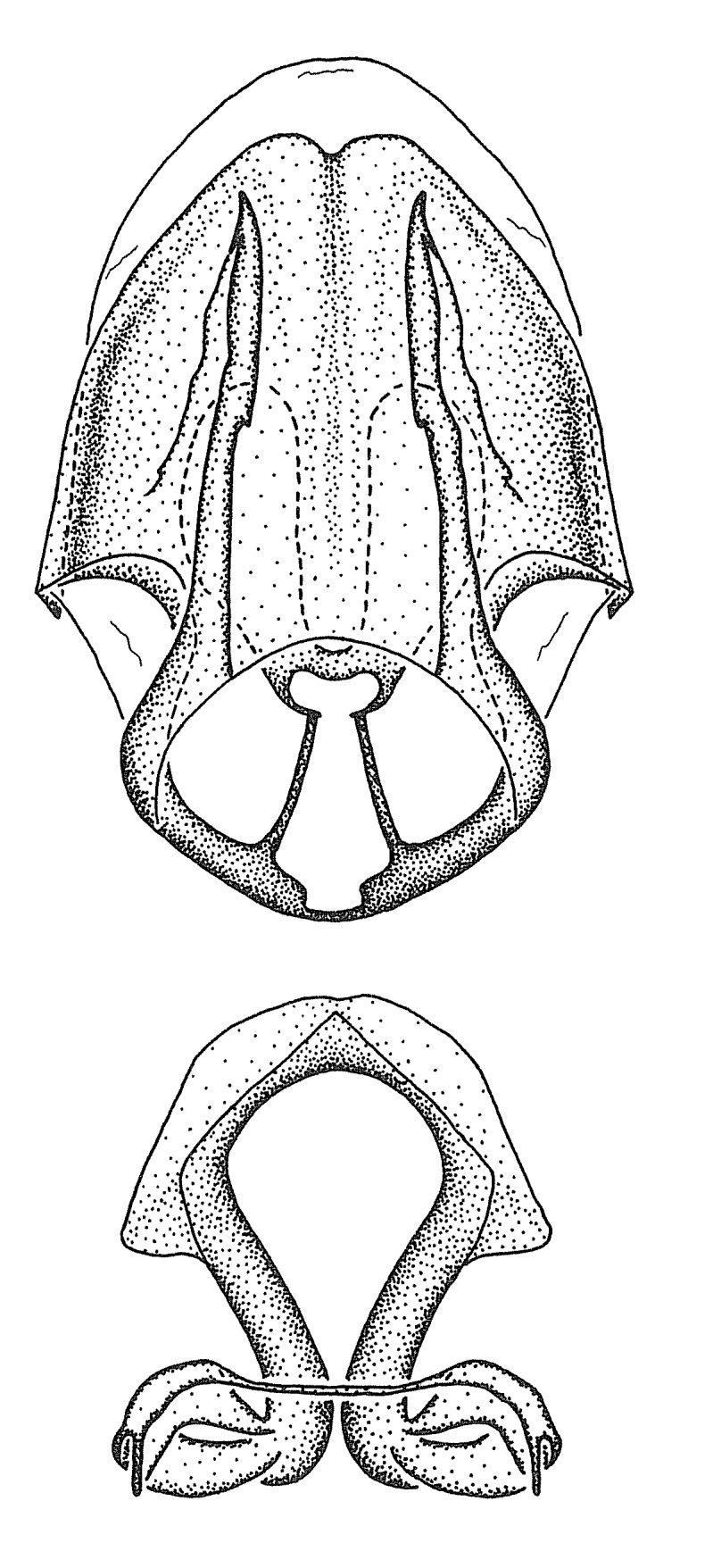
*Zelus russulumus* Zhang & Hart, sp. n., phallus, dorsal view

**Figure 179a.**
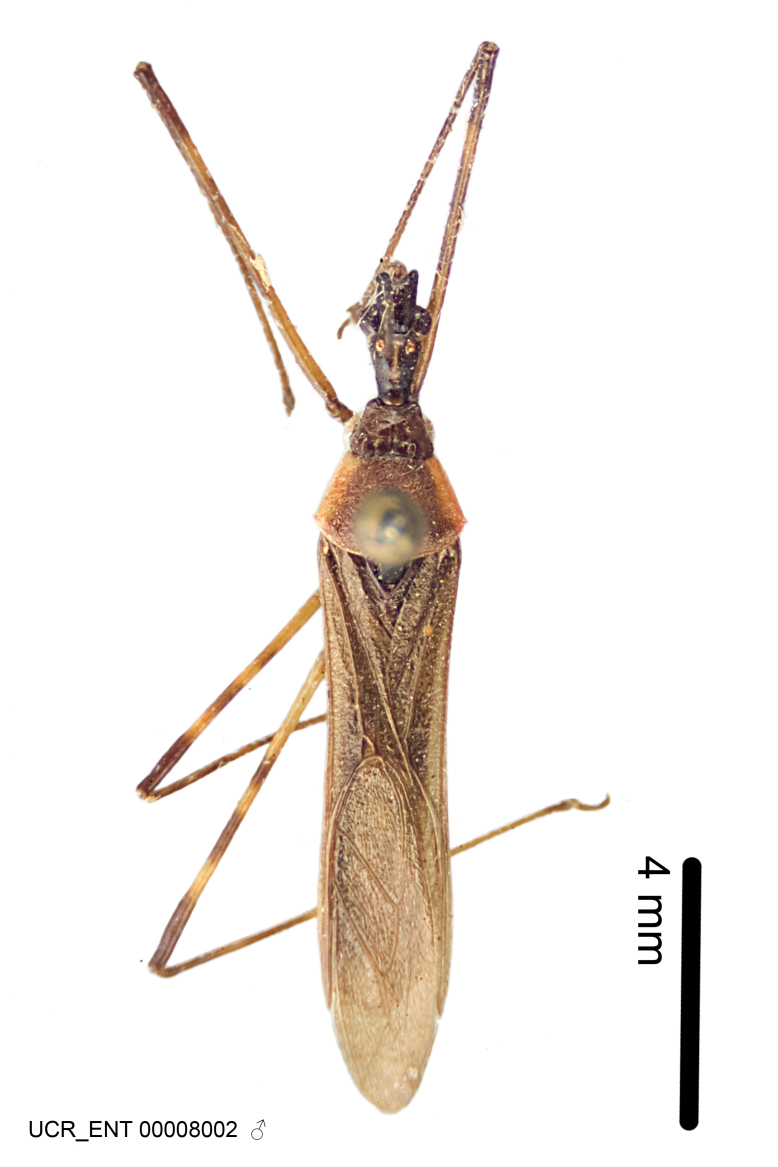
*Zelus spatulosus* Zhang & Hart, sp. n., male, dorsal view (UCR_ENT 00008002)

**Figure 179b.**
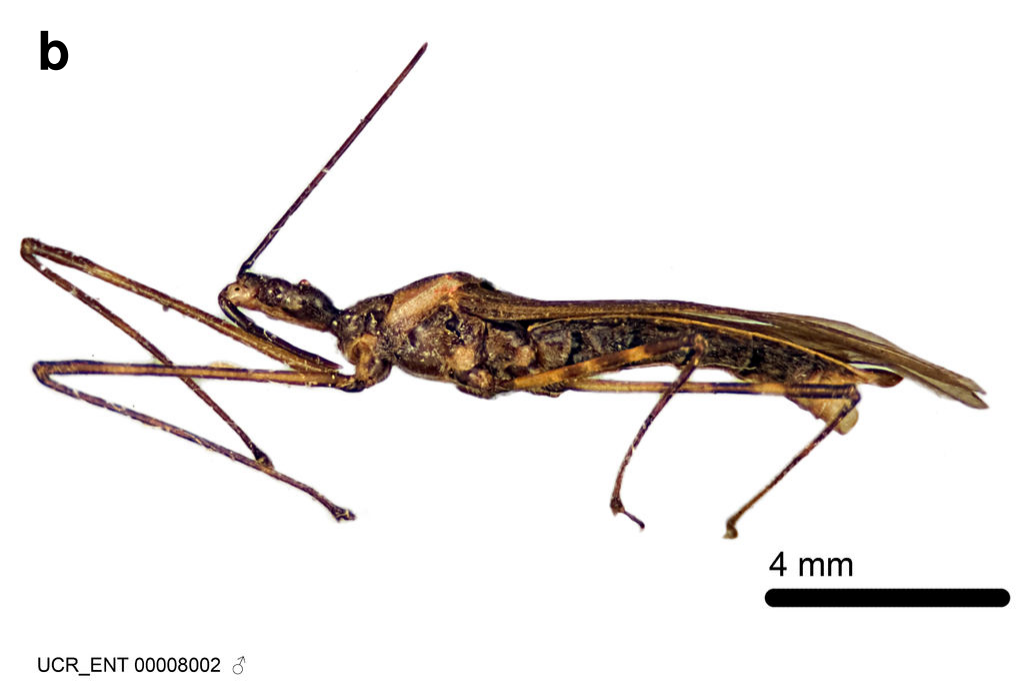
*Zelus spatulosus* Zhang & Hart, sp. n., male, dorsal view (UCR_ENT 00008002)

**Figure 180a.**
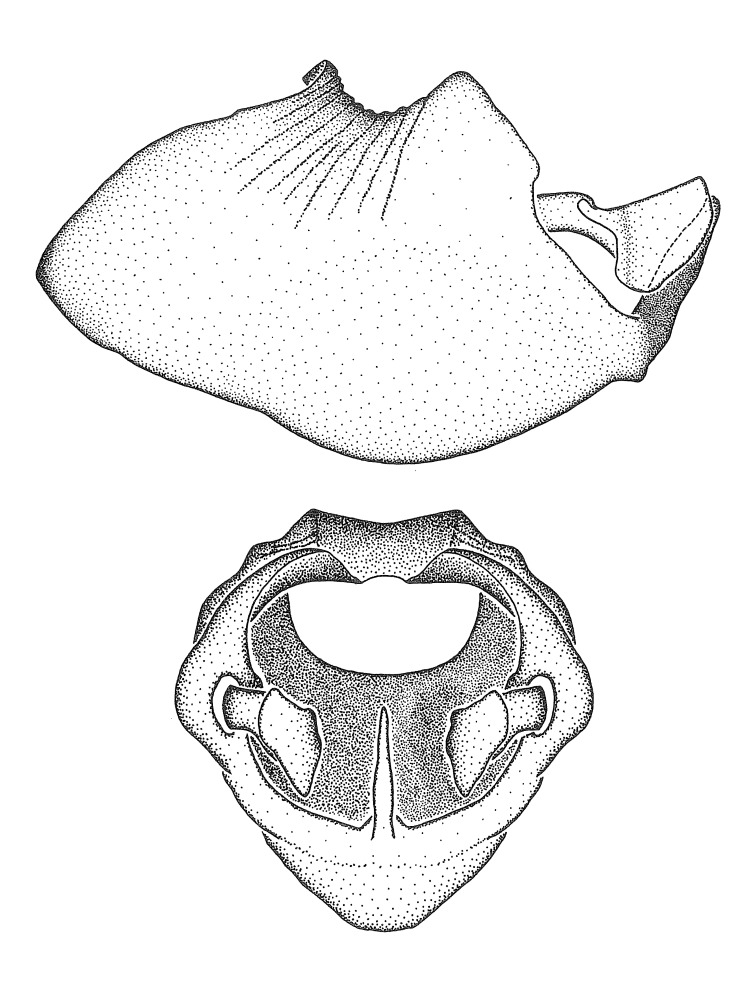
*Zelus spatulosus* Zhang & Hart, sp. n., pygophore, lateral and posterior views

**Figure 180b.**
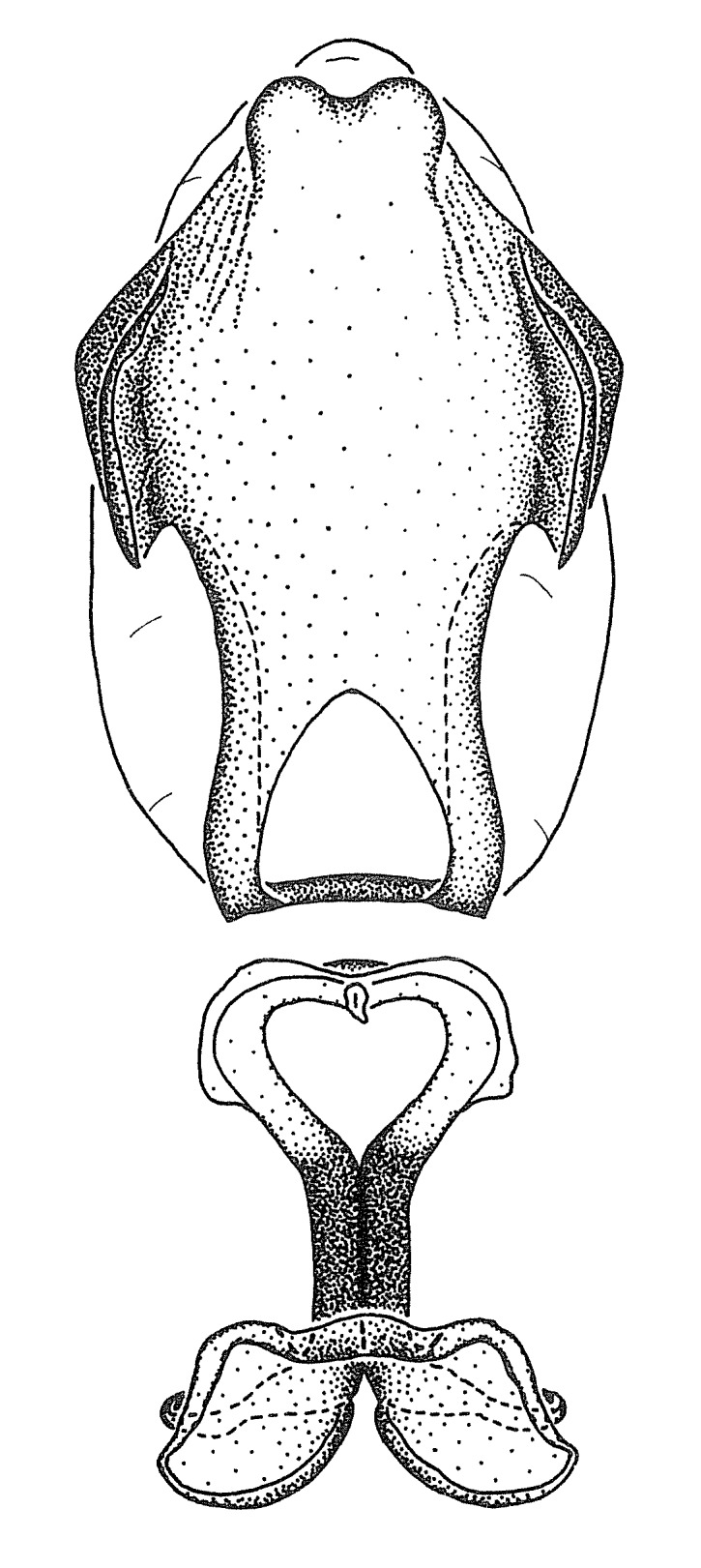
*Zelus spatulosus* Zhang & Hart, sp. n., phallus, dorsal view

**Figure 182a.**
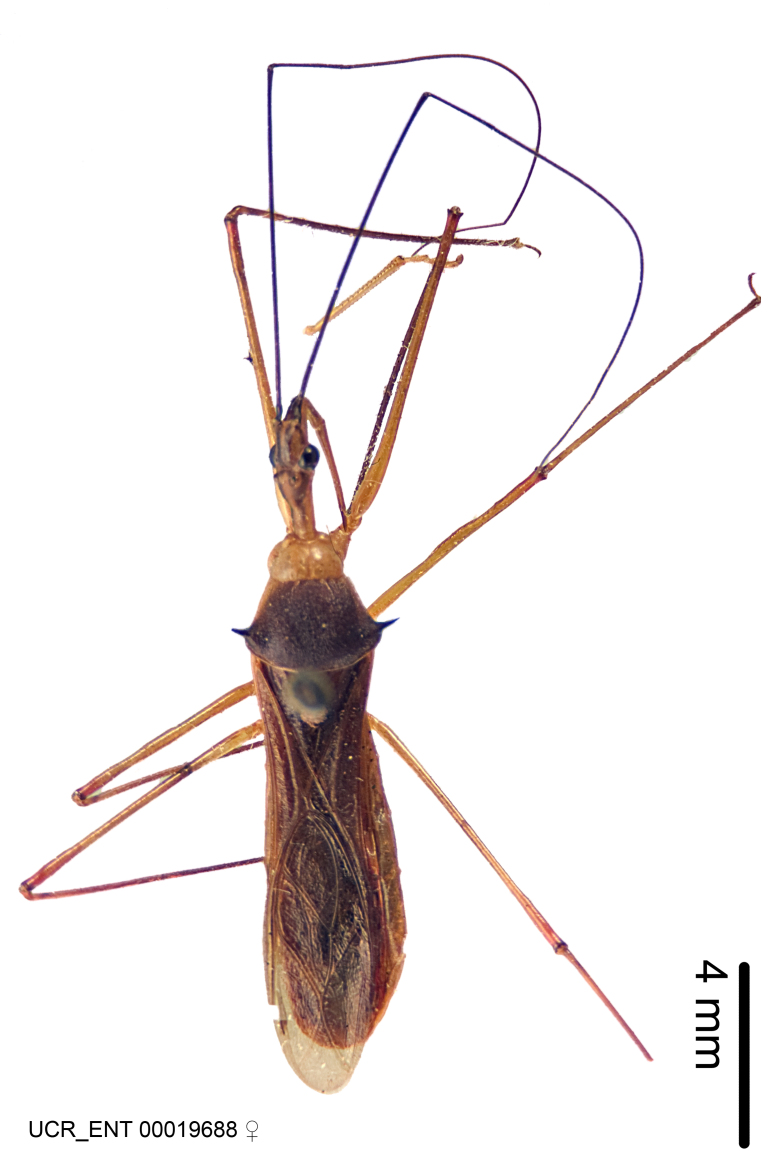
*Zelus sphegeus* Fabricius, 1803, female, dorsal view (UCR_ENT 00019688, Cuyuni-Mazaruni, Guyana)

**Figure 182b.**
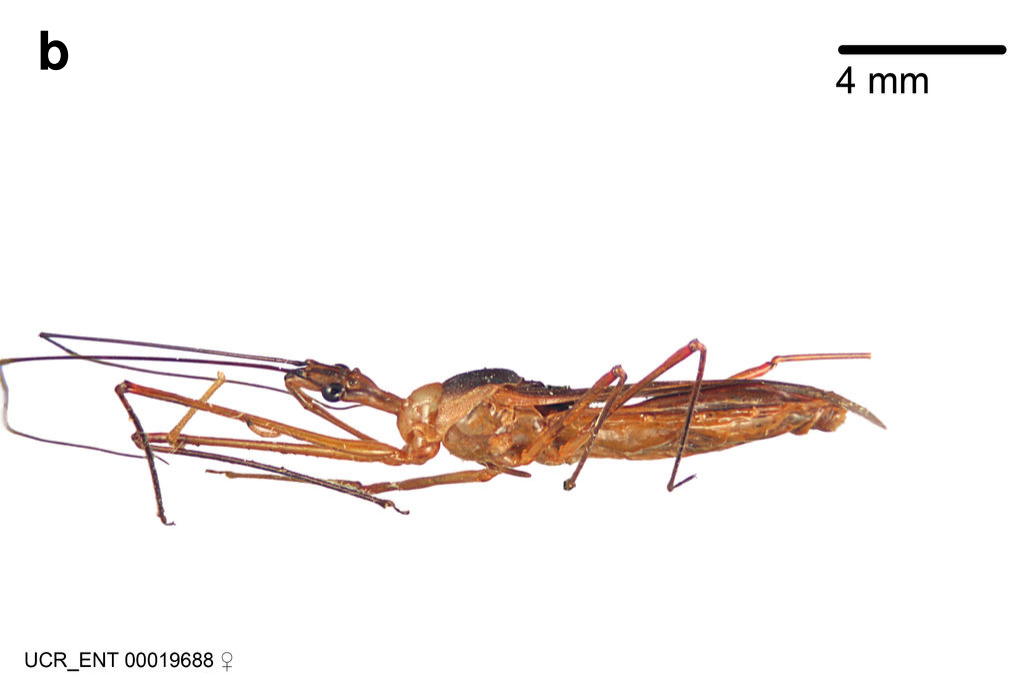
*Zelus sphegeus* Fabricius, 1803, female, lateral view (UCR_ENT 00019688, Cuyuni-Mazaruni, Guyana)

**Figure 184a.**
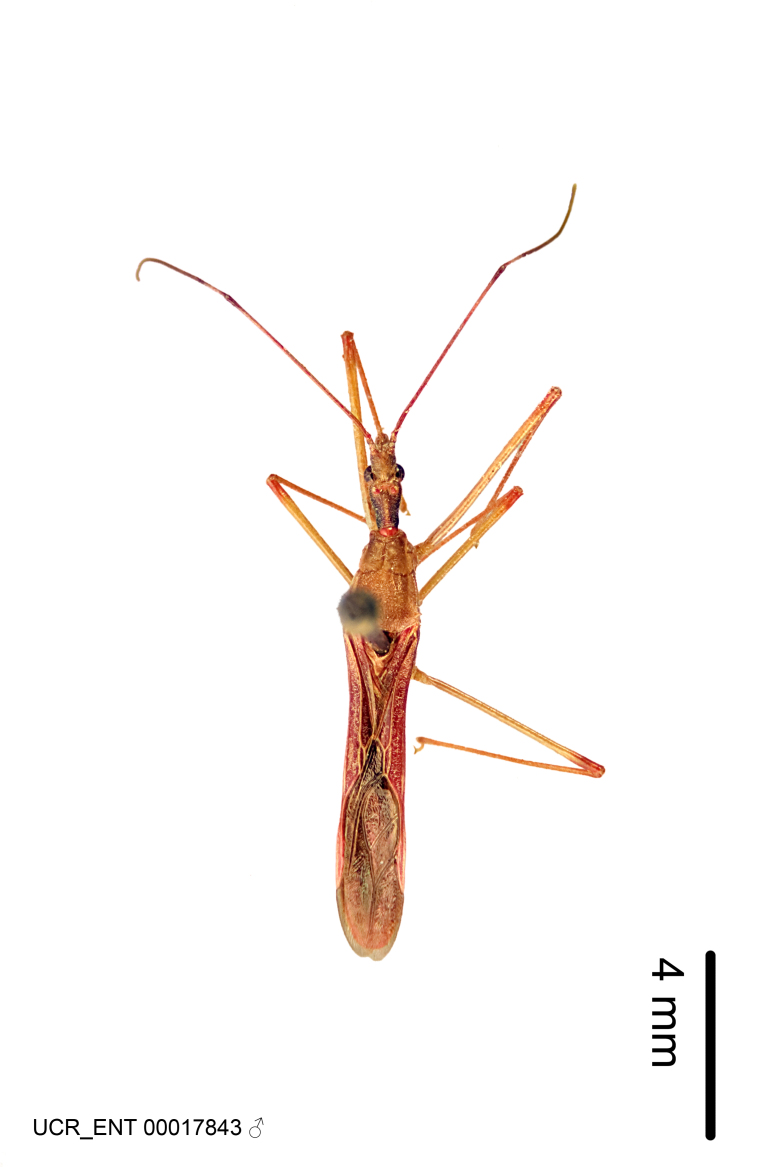
*Zelus subimpressus* Stål, 1872, male, dorsal view (UCR_ENT 00017843, Ciego de Avila, Cuba)

**Figure 184b.**
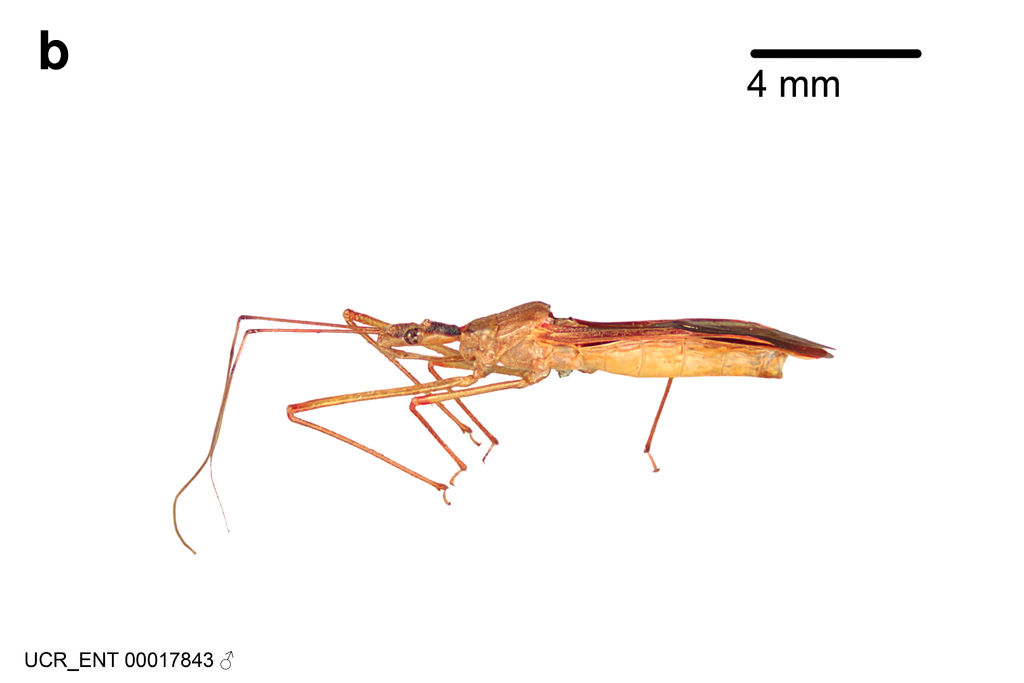
*Zelus subimpressus* Stål, 1872, male, lateral view (UCR_ENT 00017843, Ciego de Avila, Cuba)

**Figure 184c.**
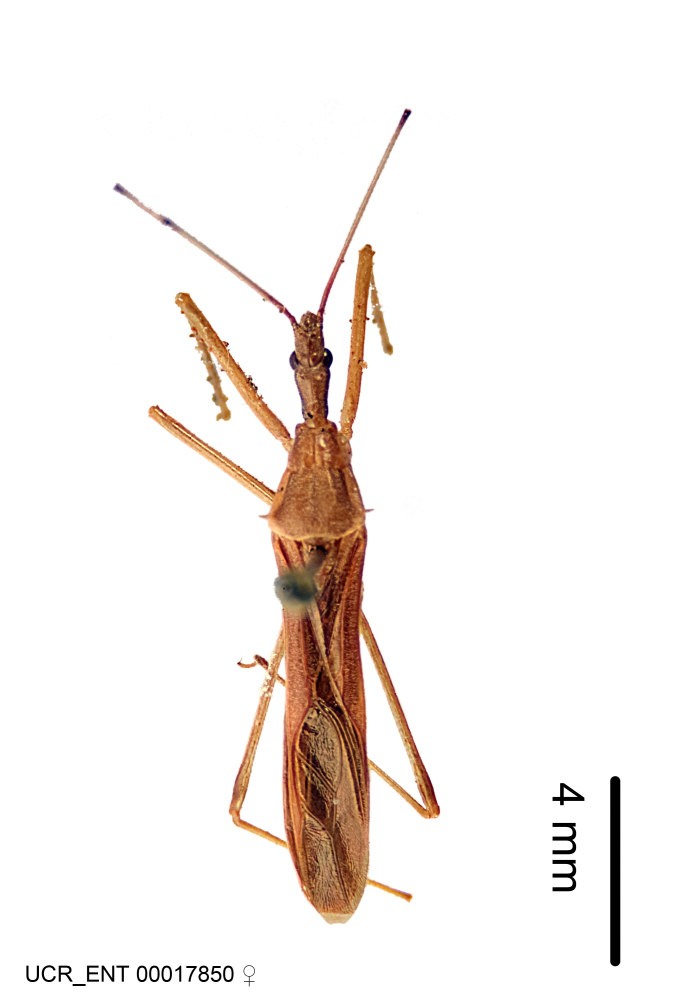
*Zelus subimpressus* Stål, 1872, female, dorsal view (UCR_ENT 00017850, Ciego de Avila, Cuba)

**Figure 184d.**
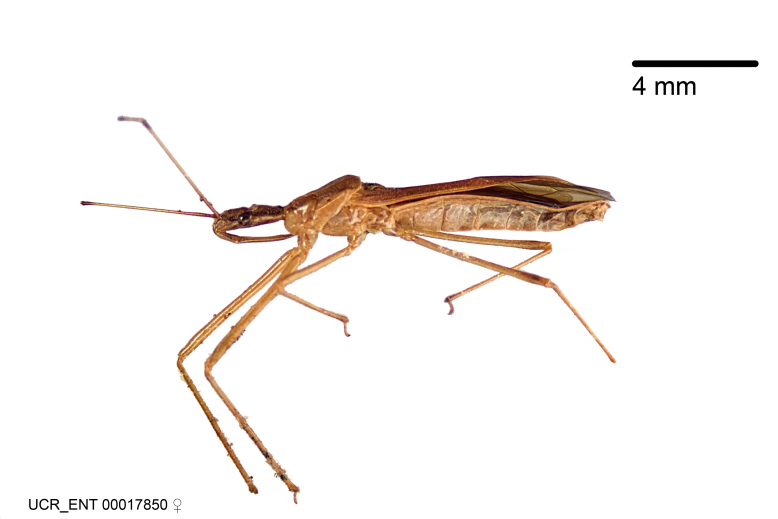
*Zelus subimpressus* Stål, 1872, female, lateral view (UCR_ENT 00017850, Ciego de Avila, Cuba)

**Figure 185a.**
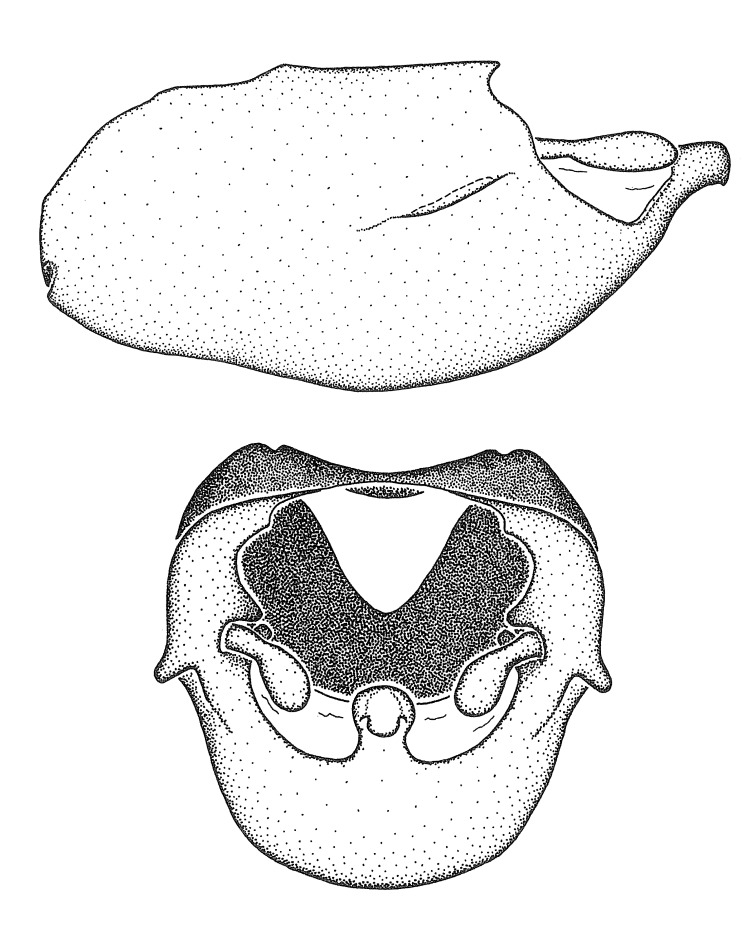
*Zelus subimpressus* Stål, 1872, pygophore, lateral and posterior views

**Figure 185b.**
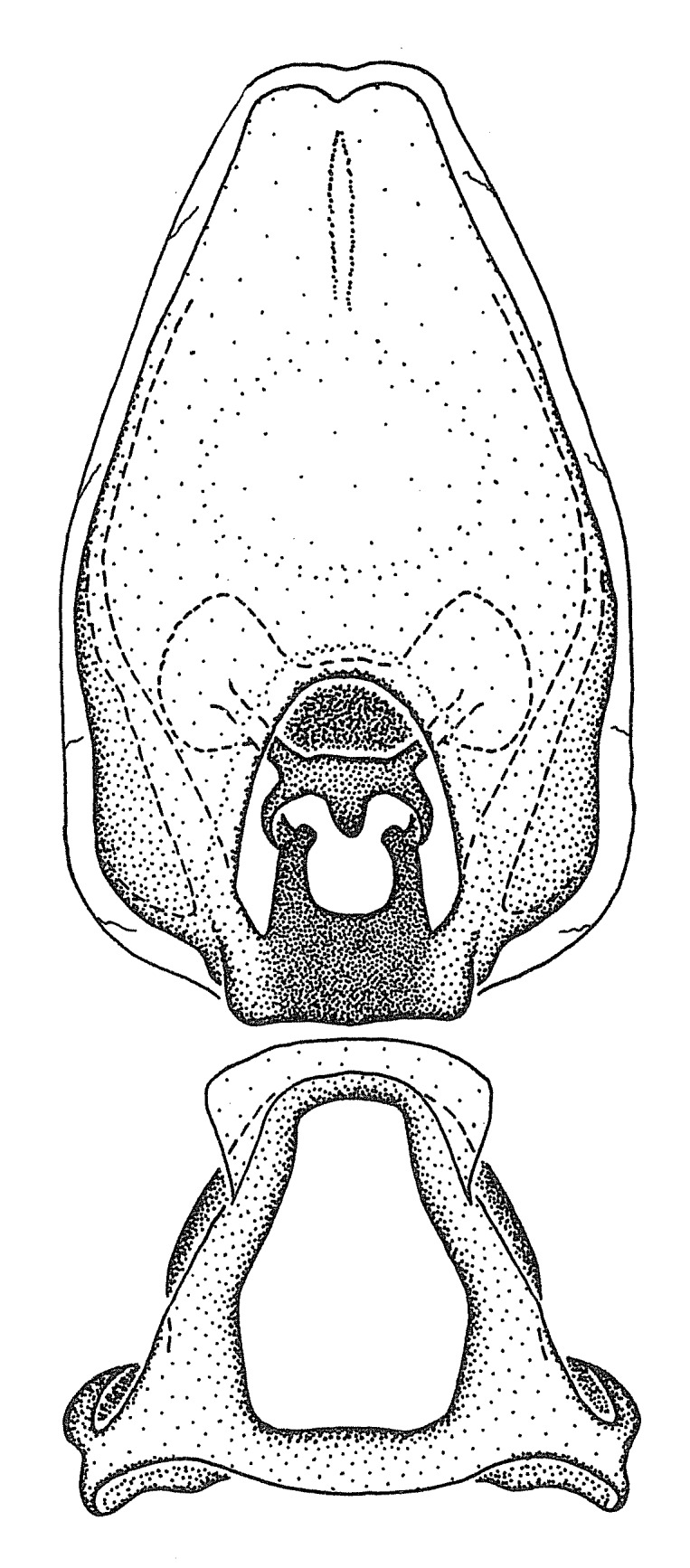
*Zelus subimpressus* Stål, 1872, phallus, dorsal view

**Figure 187a.**
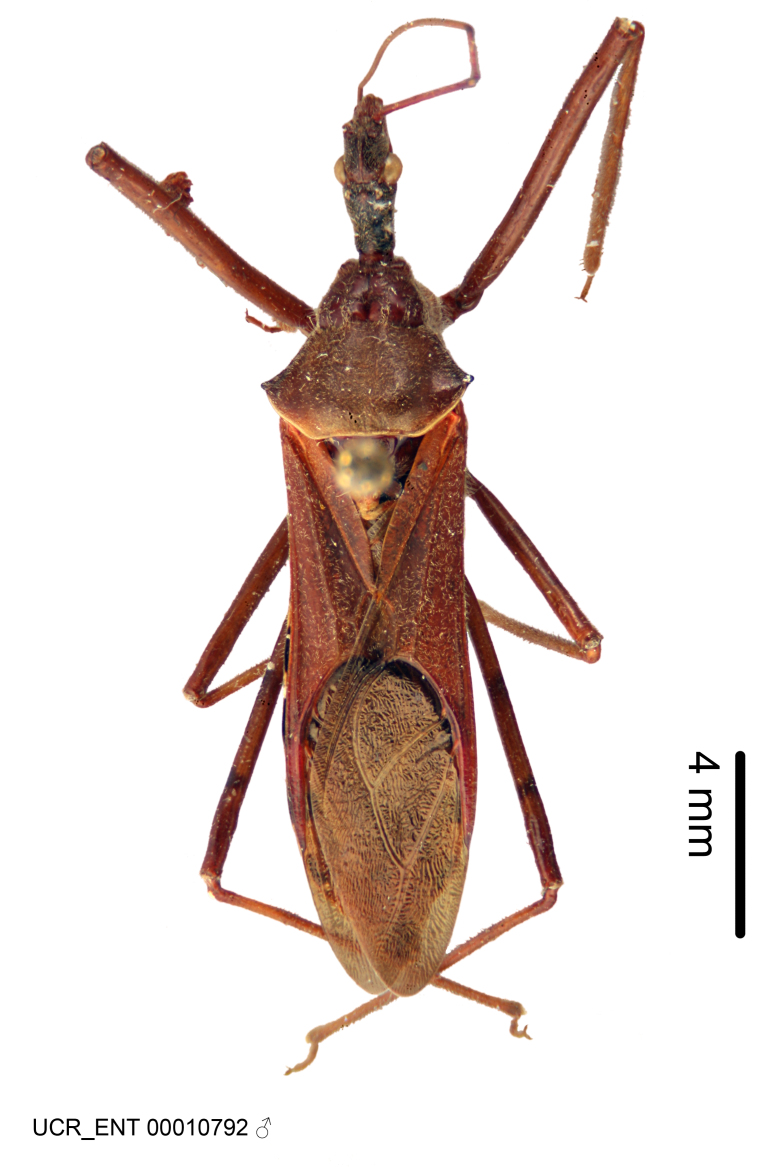
*Zelus sulcicollis* Champion, 1899, male, dorsal view (UCR_ENT 00010792, Guerrero, Mexico)

**Figure 187b.**
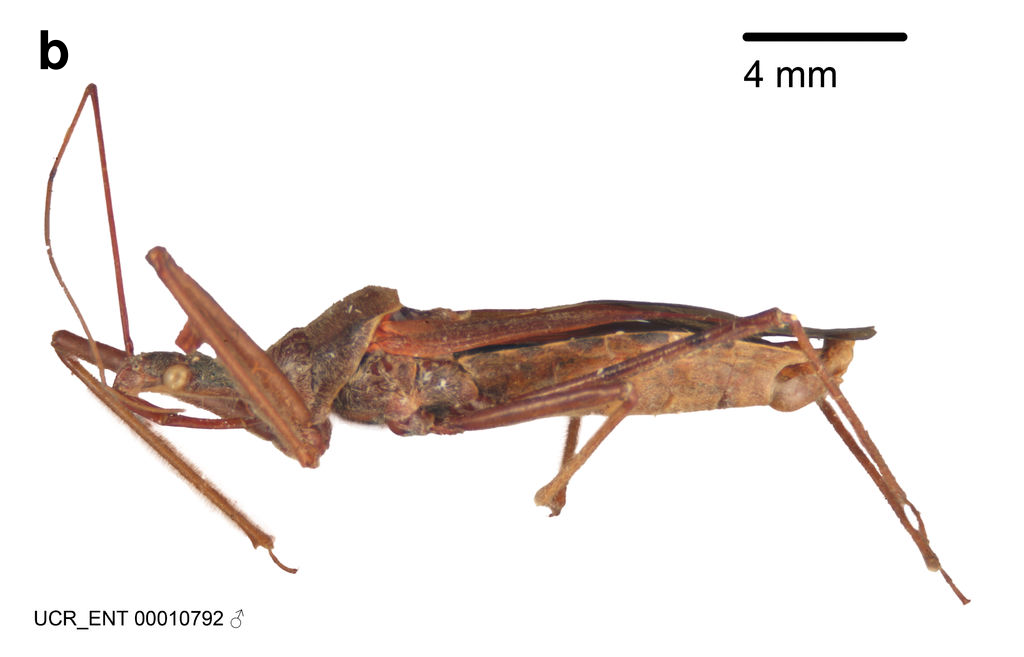
*Zelus sulcicollis* Champion, 1899, male, lateral view (UCR_ENT 00010792, Guerrero, Mexico)

**Figure 187c.**
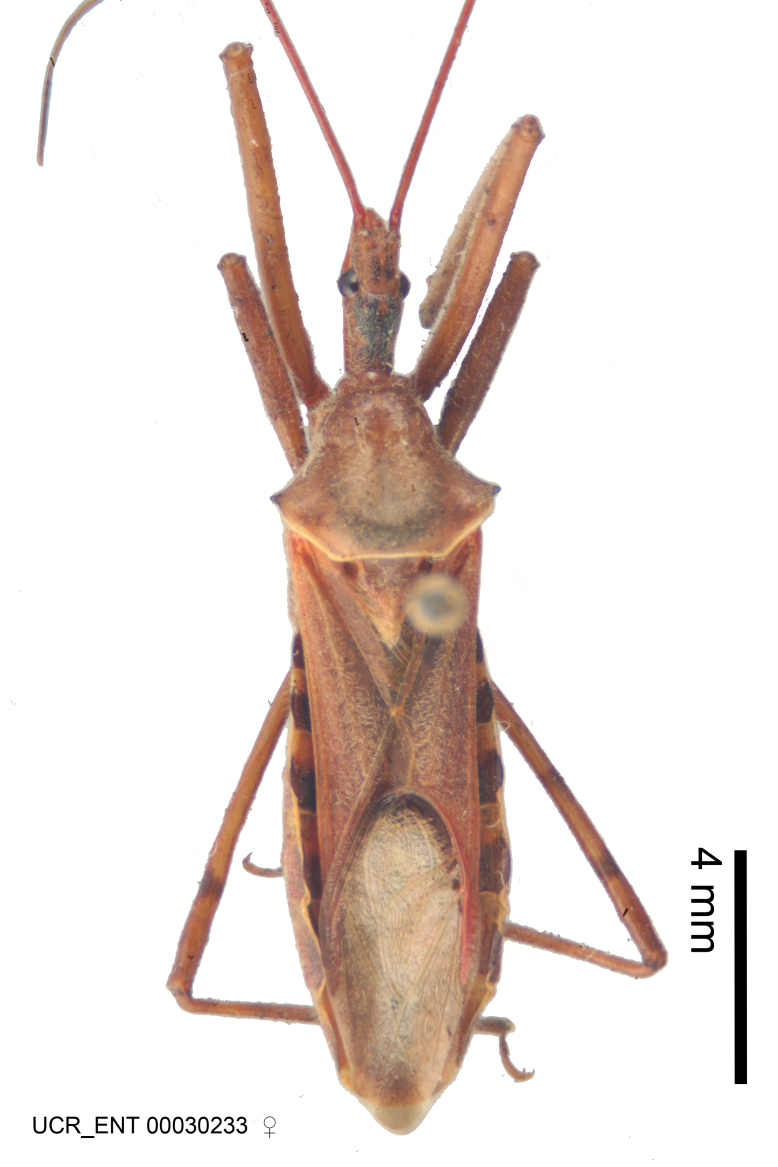
*Zelus sulcicollis* Champion, 1899, female, dorsal view (UCR_ENT 00030233, Hidalgo, Mexico)

**Figure 187d.**
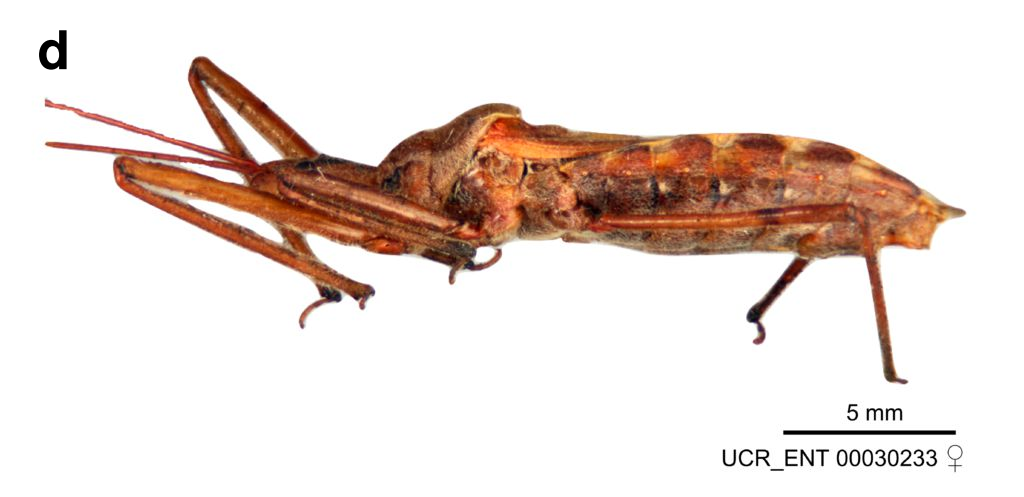
*Zelus sulcicollis* Champion, 1899, female, dorsal view (UCR_ENT 00030233, Hidalgo, Mexico)

**Figure 188a.**
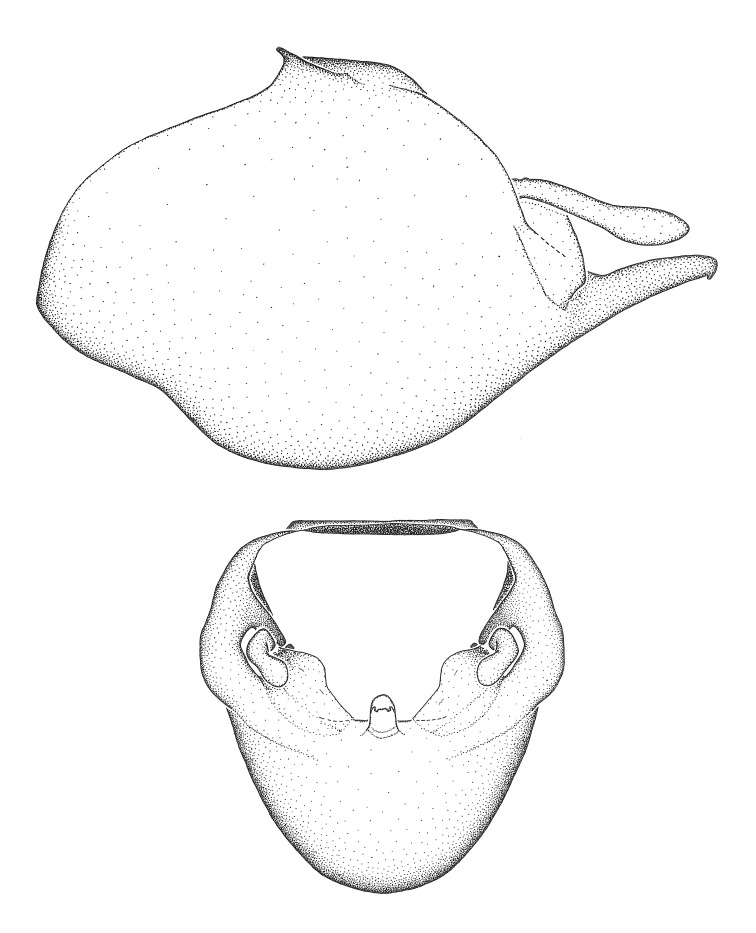
*Zelus sulcicollis* Champion, 1899, pygophore, lateral and posterior views

**Figure 188b.**
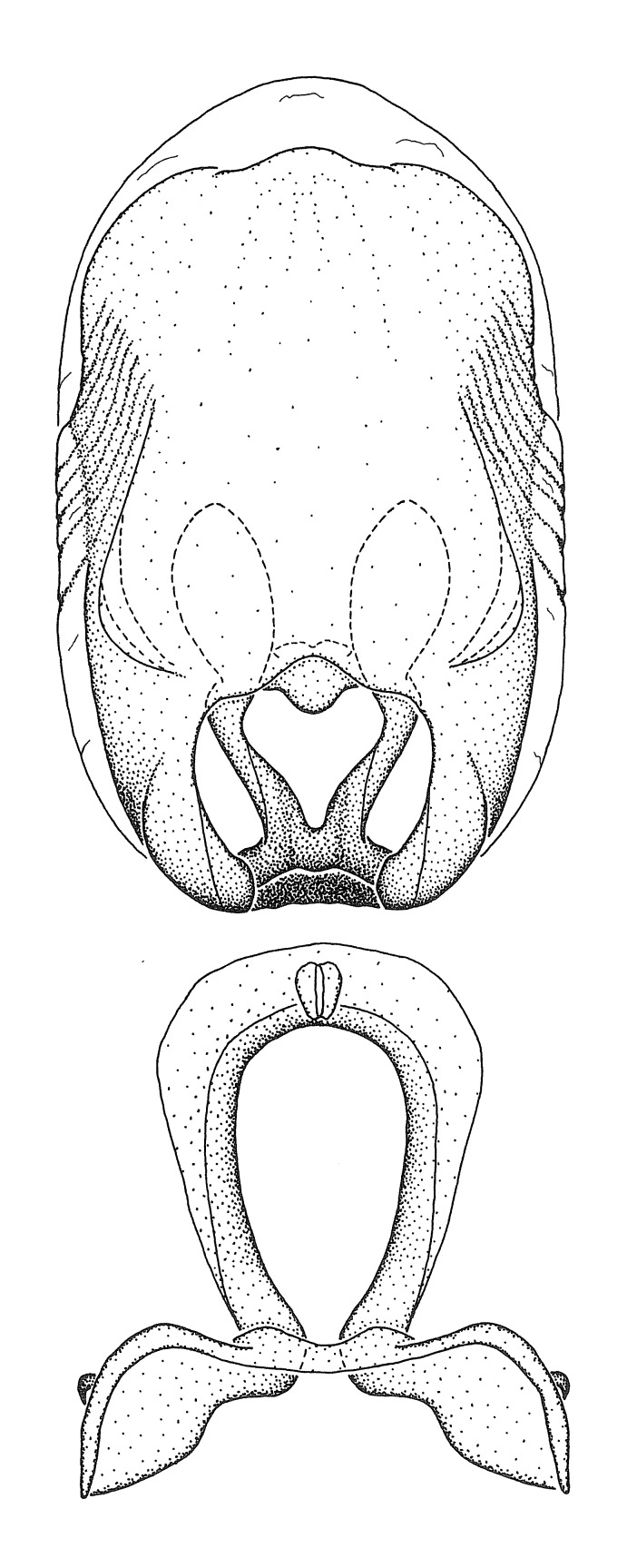
*Zelus sulcicollis* Champion, 1899, phallus, dorsal view

**Figure 190a.**
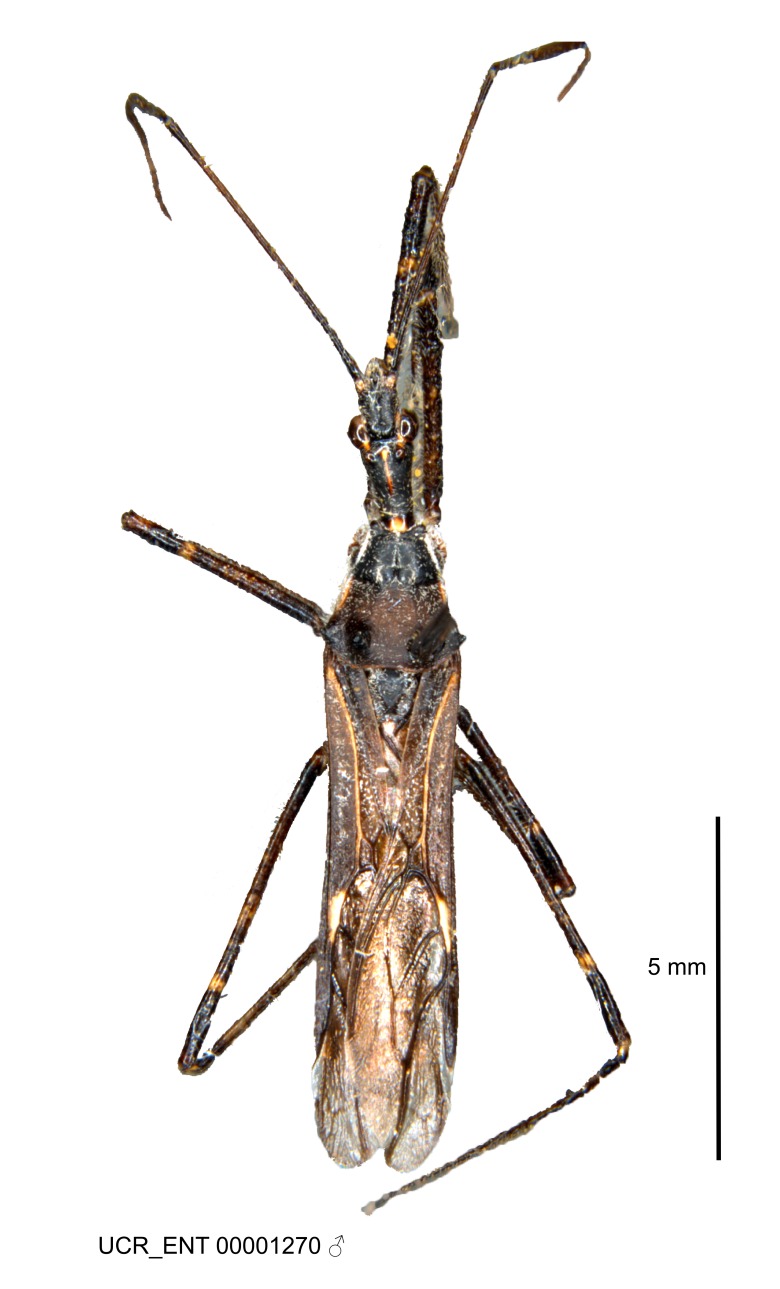
*Zelus tetracanthus* Stål, 1862, male, dorsal view (UCR_ENT 00001270, California, USA)

**Figure 190b.**
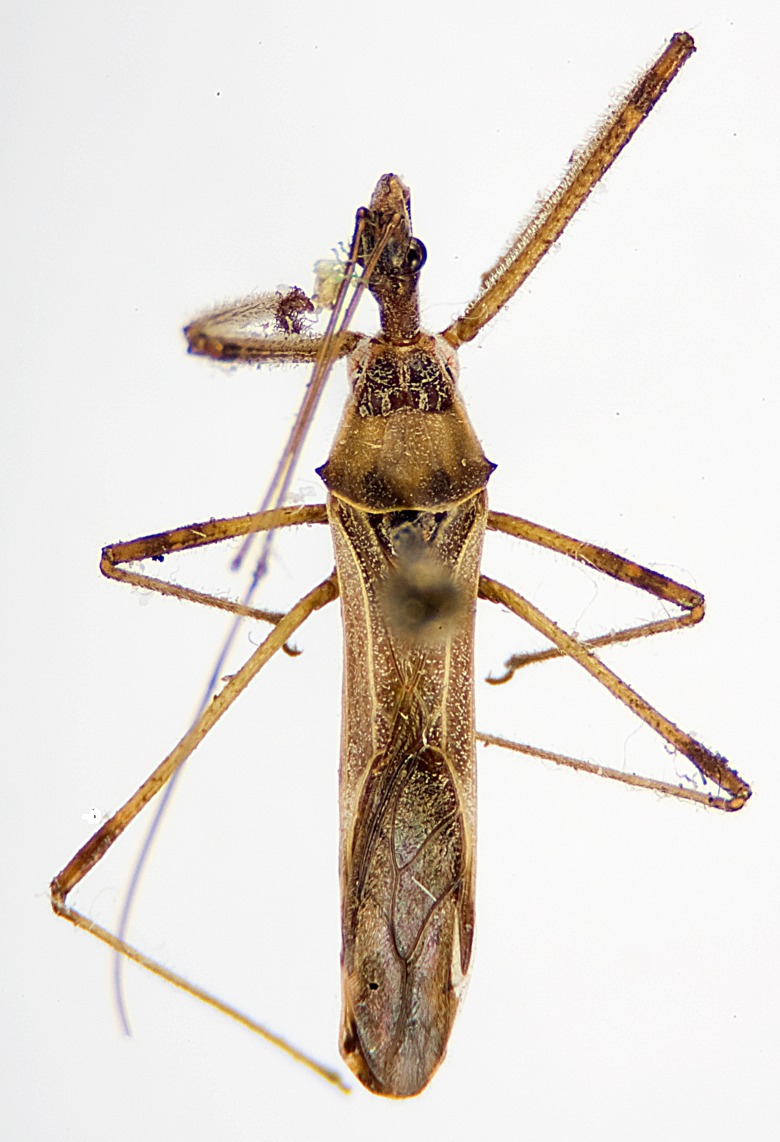
*Zelus tetracanthus* Stål, 1862, female, dorsal view (UCR_ENT 00001117, Arizona, USA)

**Figure 190c.**
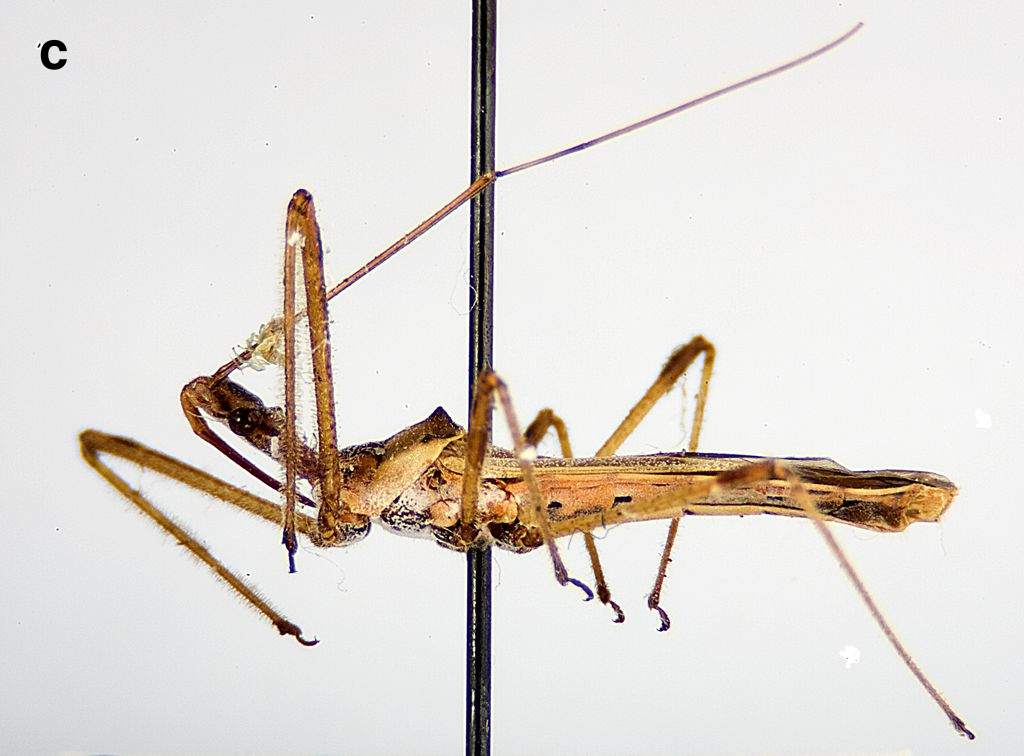
*Zelus tetracanthus* Stål, 1862, female, lateral view (UCR_ENT 00001117, Arizona, USA)

**Figure 191a.**
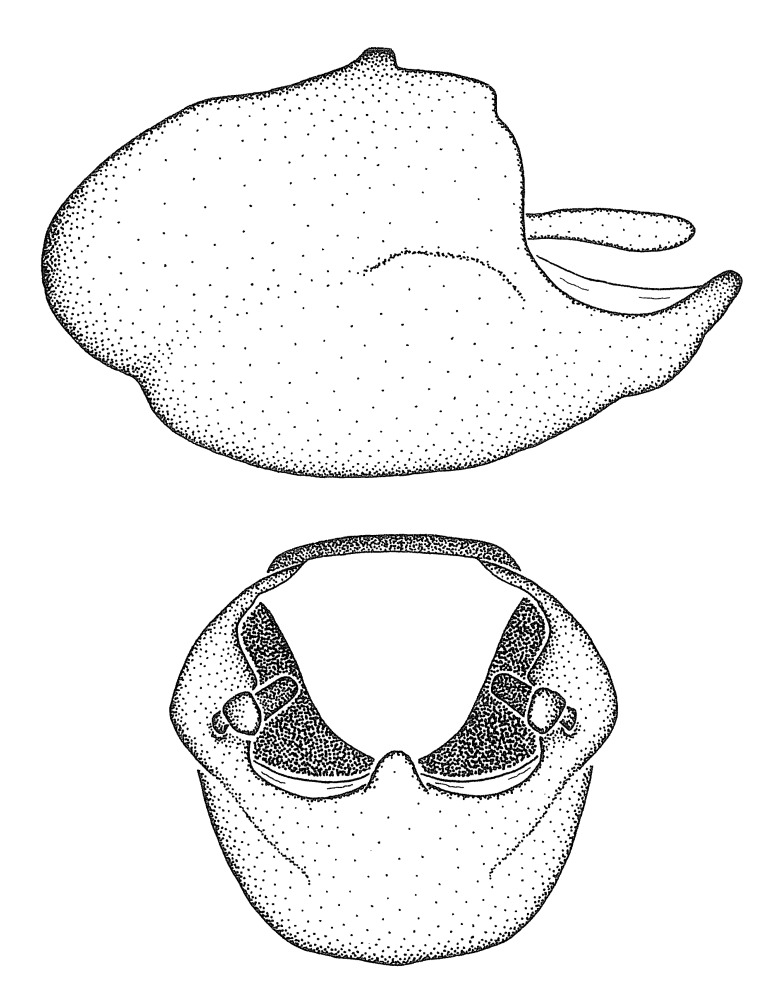
*Zelus tetracanthus* Stål, 1862, northern and lowland population, pygophore, lateral and posterior views

**Figure 191b.**
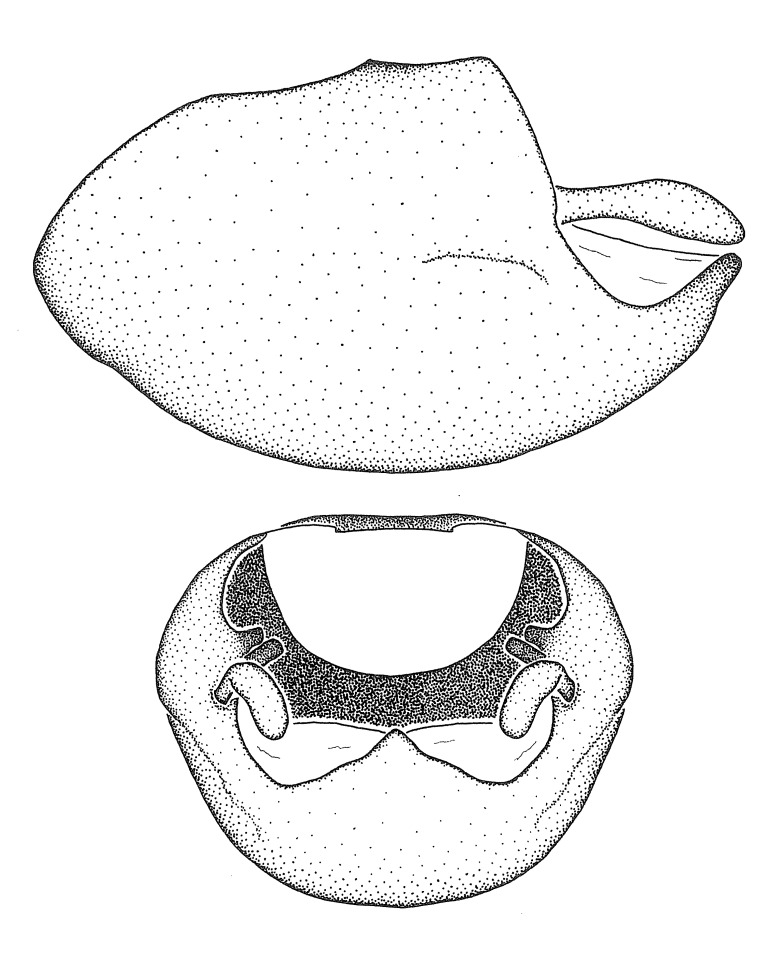
*Zelus tetracanthus* Stål, 1862, southern and highland population, pygophore, lateral and posterior views

**Figure 191c.**
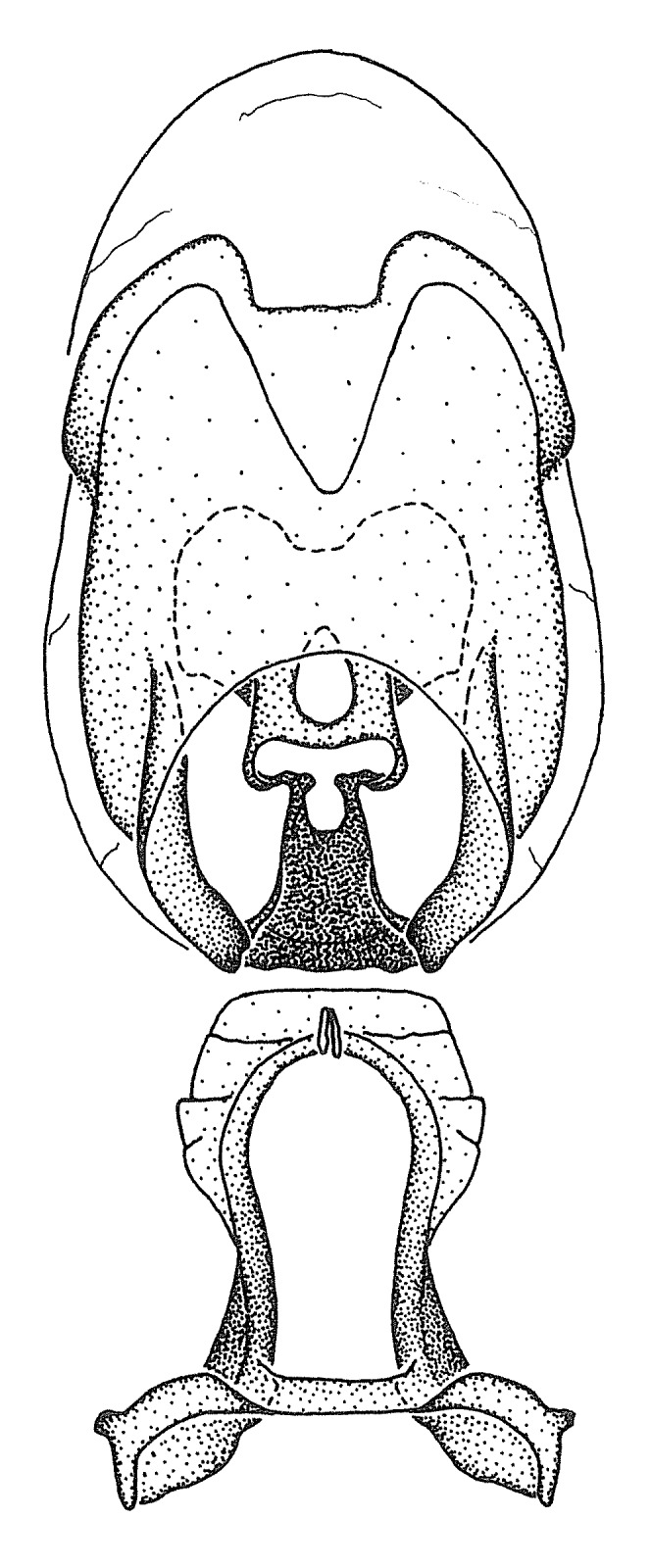
*Zelus tetracanthus* Stål, 1862, northern and lowland population, phallus, dorsal view

**Figure 191d.**
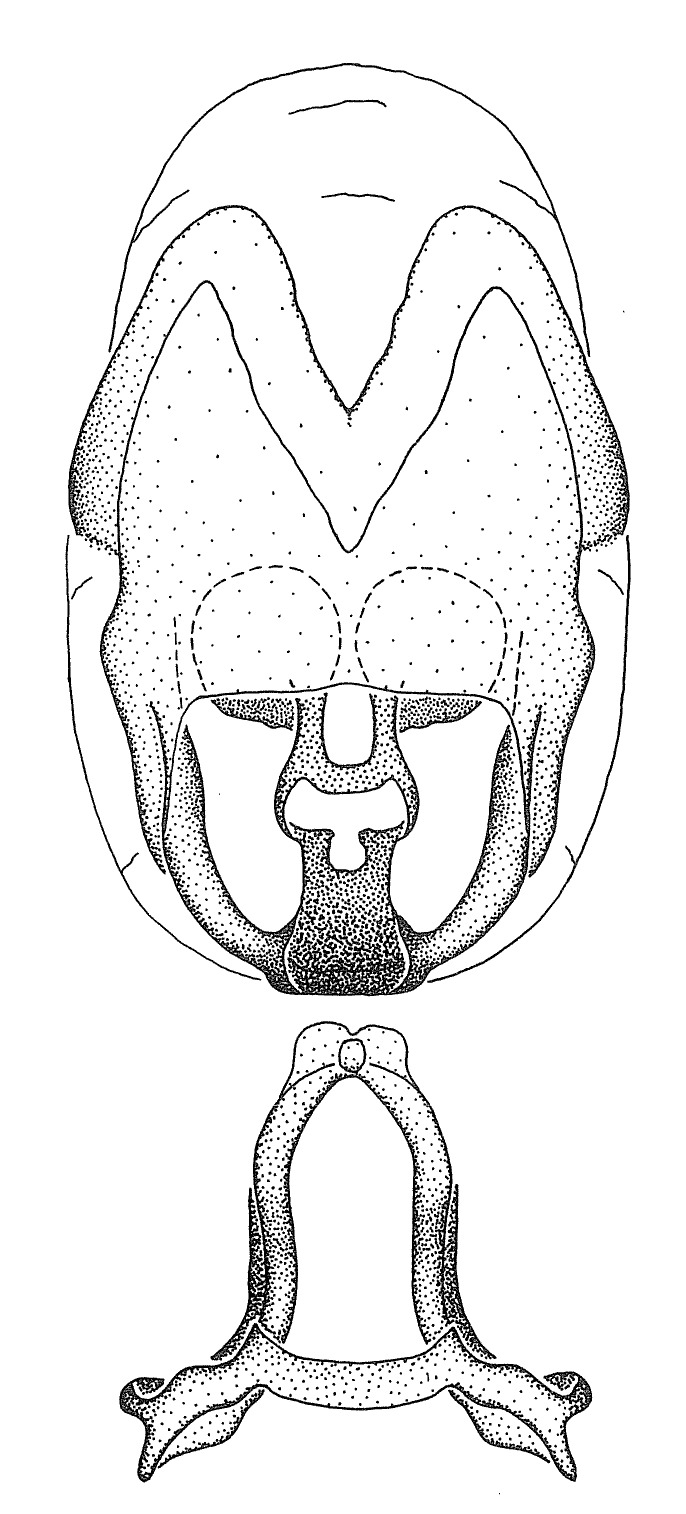
*Zelus tetracanthus* Stål, 1862, southern and highland population, phallus, dorsal view

**Figure 193a.**
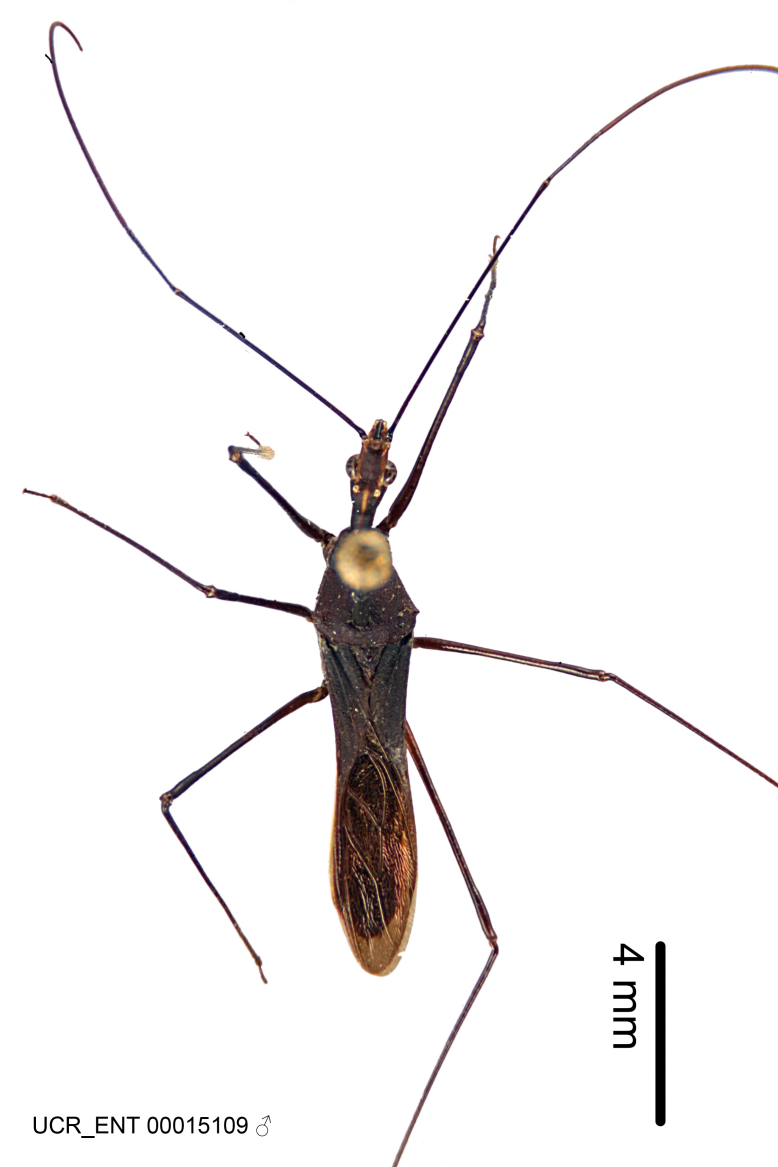
*Zelus truxali* Zhang & Hart, sp. n., male, dorsal view (UCR_ENT 00015109, Sucumbios, Ecuador)

**Figure 193b.**
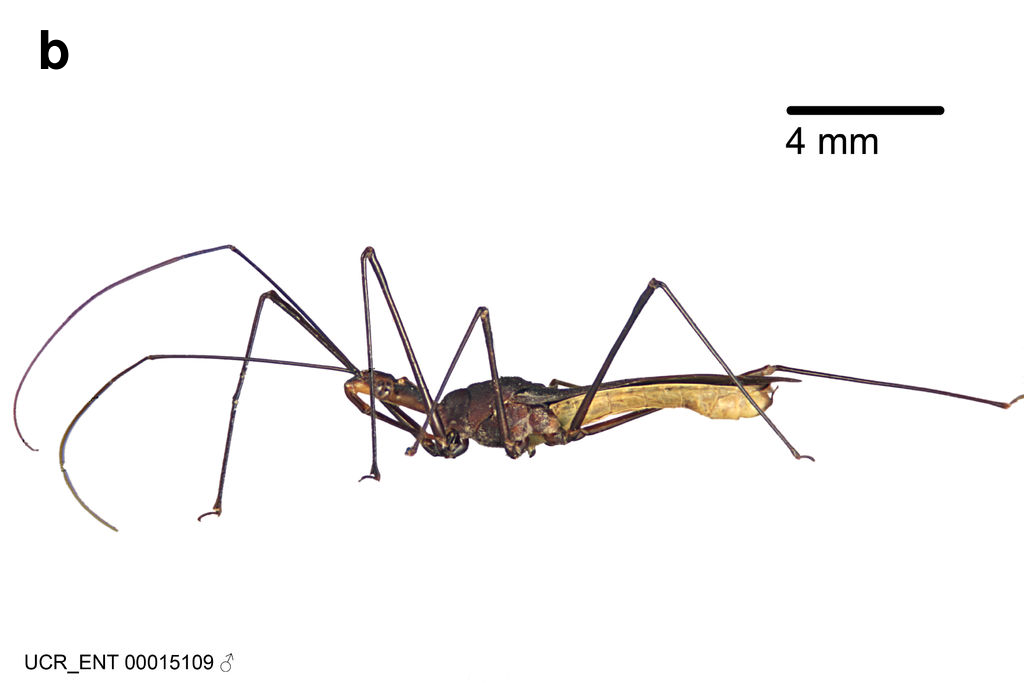
*Zelus truxali* Zhang & Hart, sp. n., male, lateral view (UCR_ENT 00015109, Sucumbios, Ecuador)

**Figure 193c.**
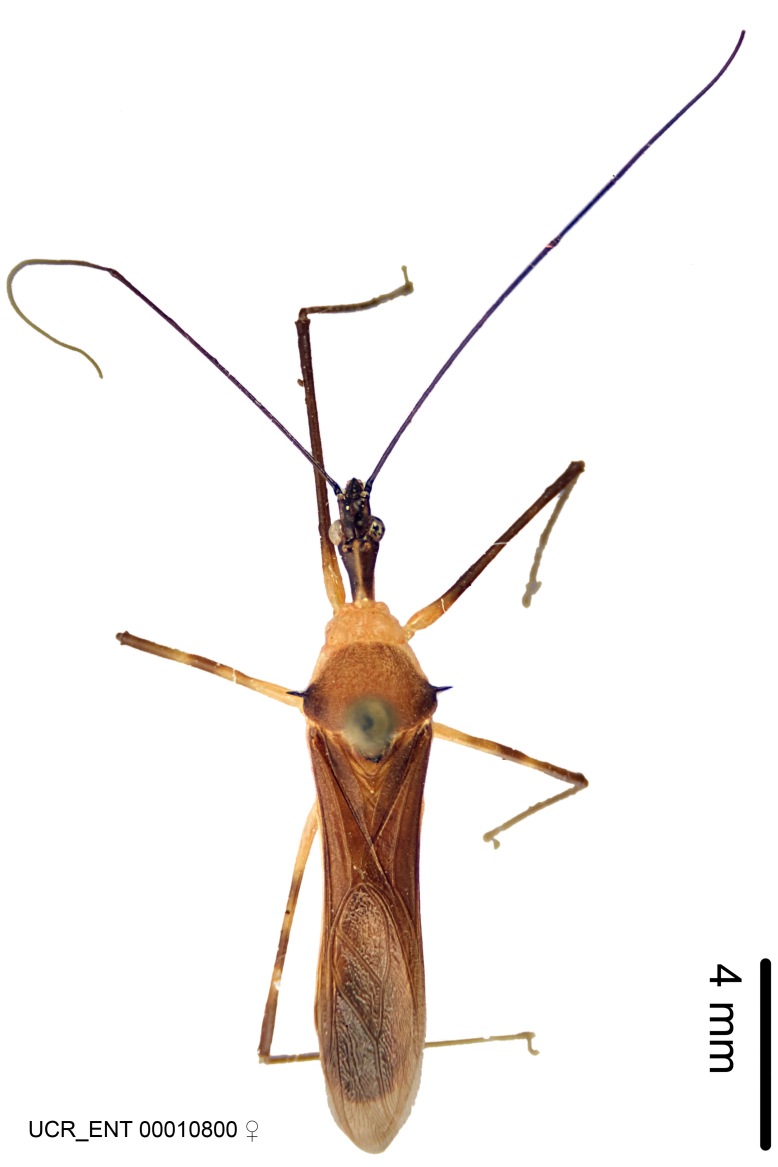
*Zelus truxali* Zhang & Hart, sp. n., female, dorsal view (UCR_ENT 00010800, Pasco, Peru)

**Figure 193d.**
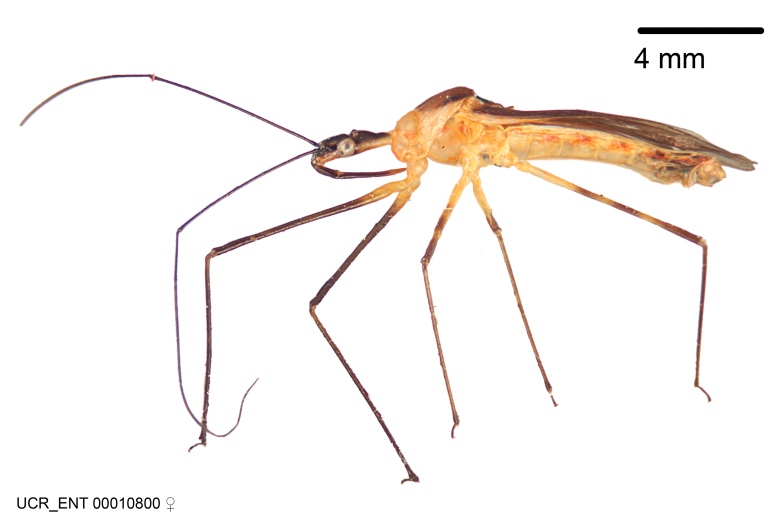
*Zelus truxali* Zhang & Hart, sp. n., female, lateral view (UCR_ENT 00010800, Pasco, Peru)

**Figure 194a.**
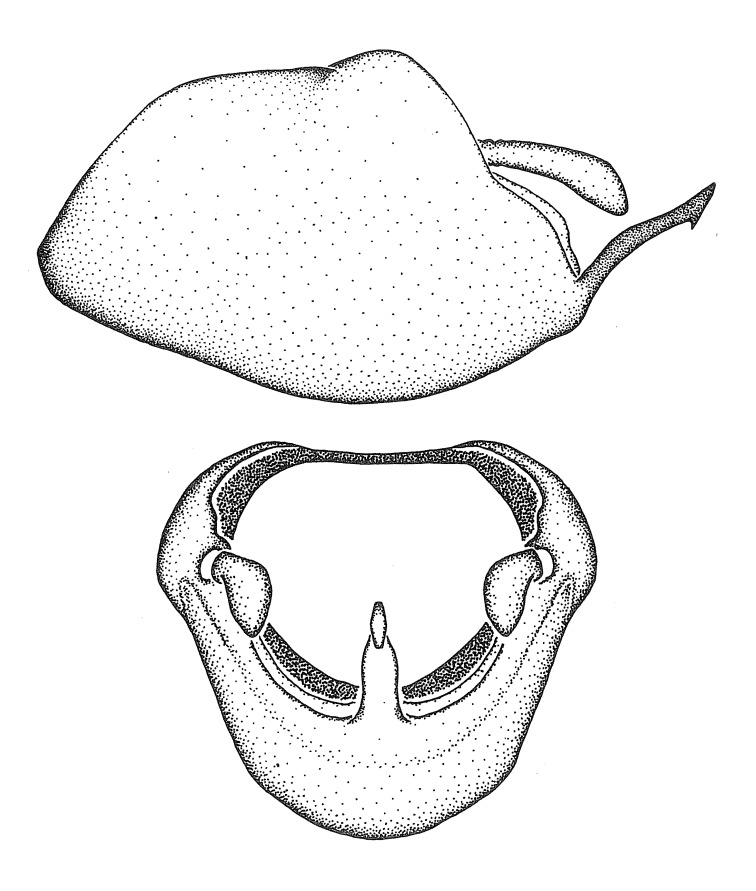
*Zelus truxali* Zhang & Hart, sp. n., male genitalic structures

**Figure 194b.**
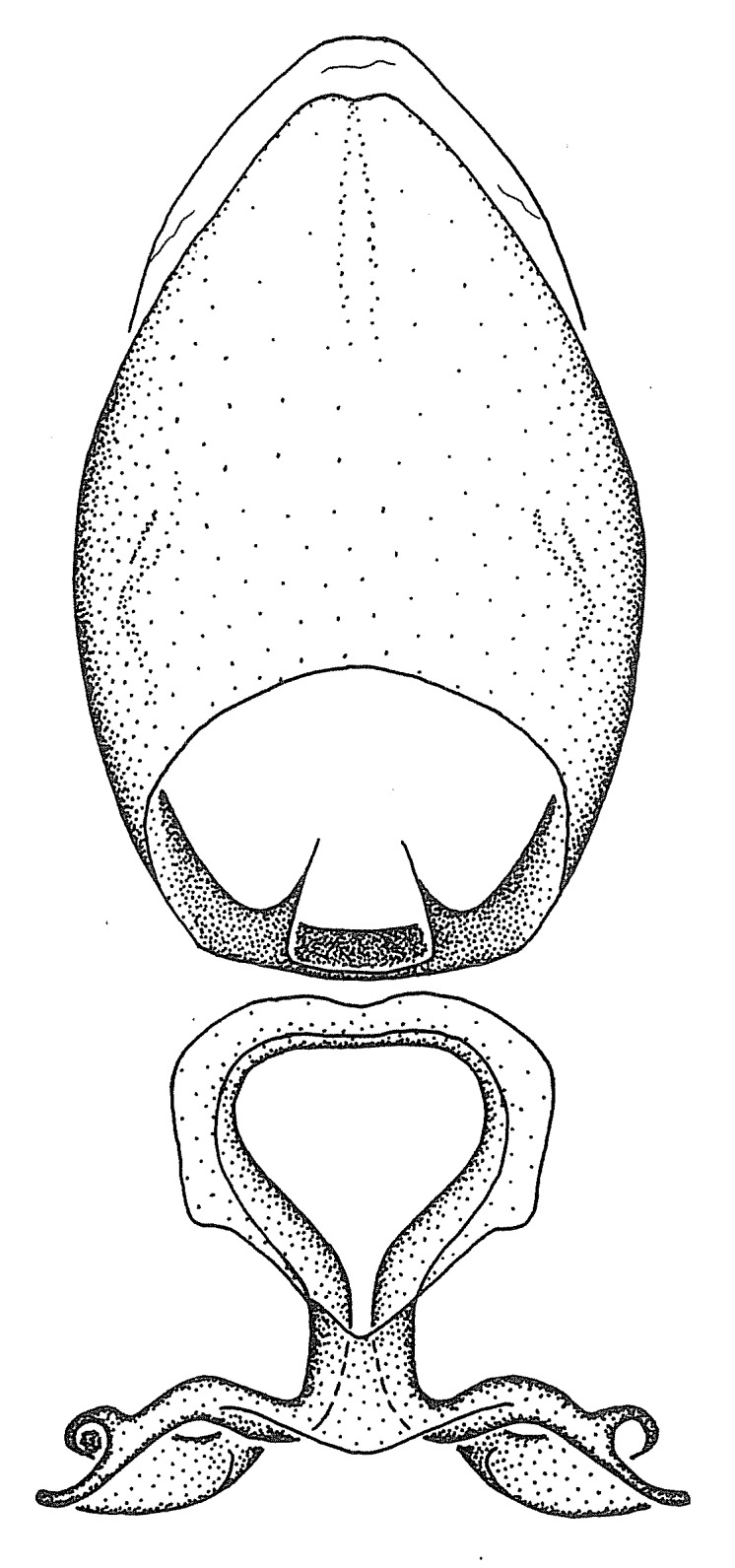
*Zelus truxali* Zhang & Hart, sp. n., phallus, dorsal

**Figure 196a.**
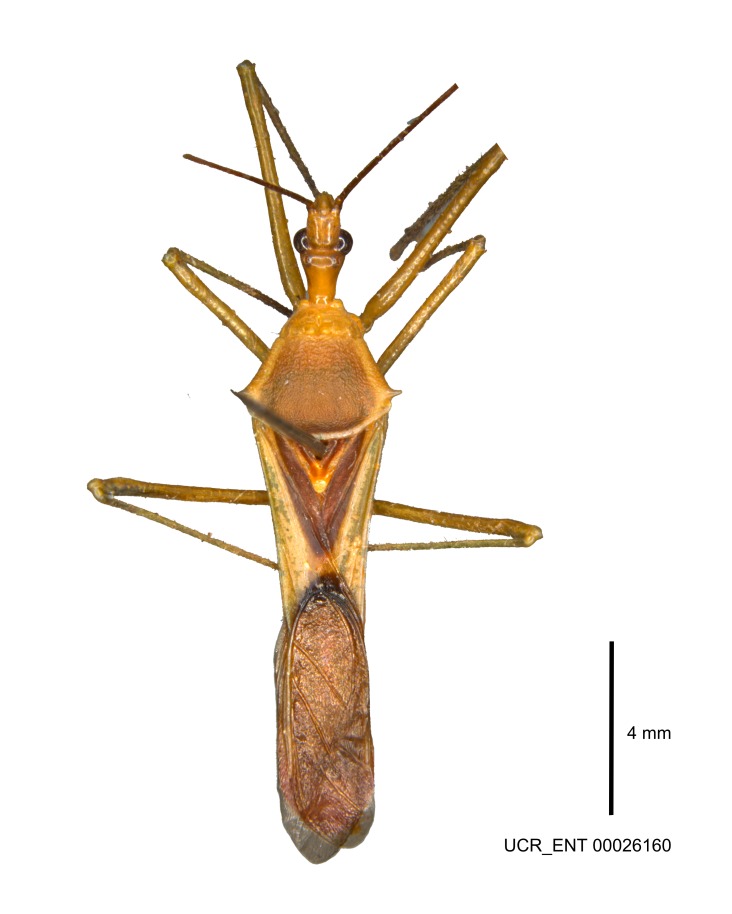
*Zelus umbraculoides* Zhang & Hart, sp. n., male, dorsal view

**Figure 196b.**
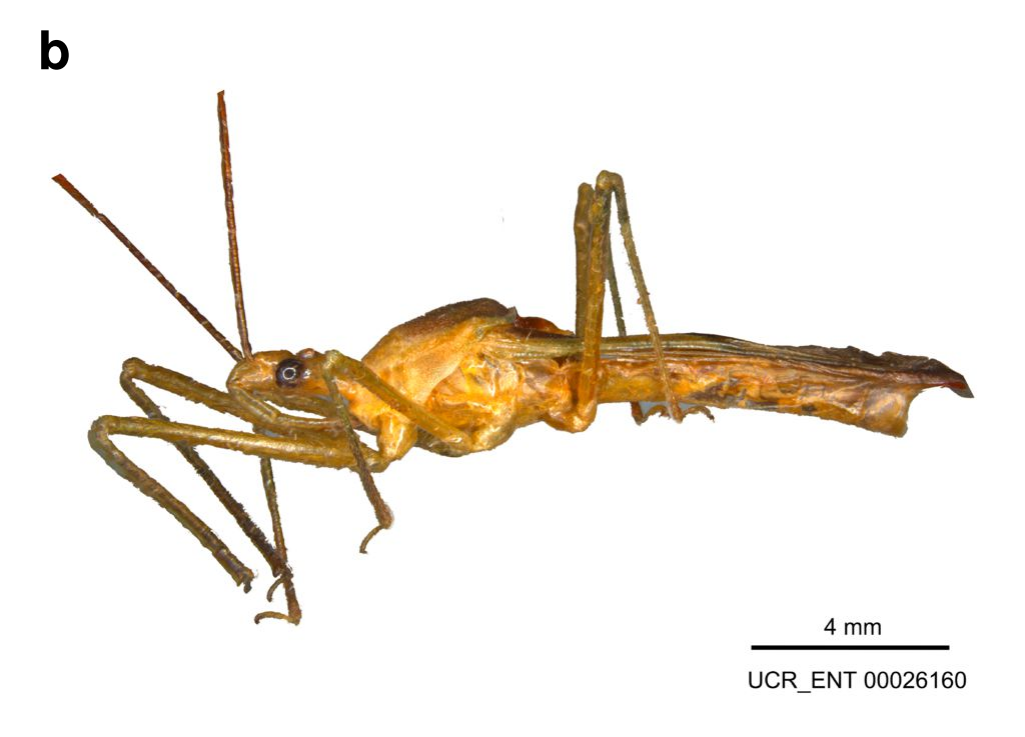
*Zelus umbraculoides* Zhang & Hart, sp. n., male, lateral view

**Figure 197a.**
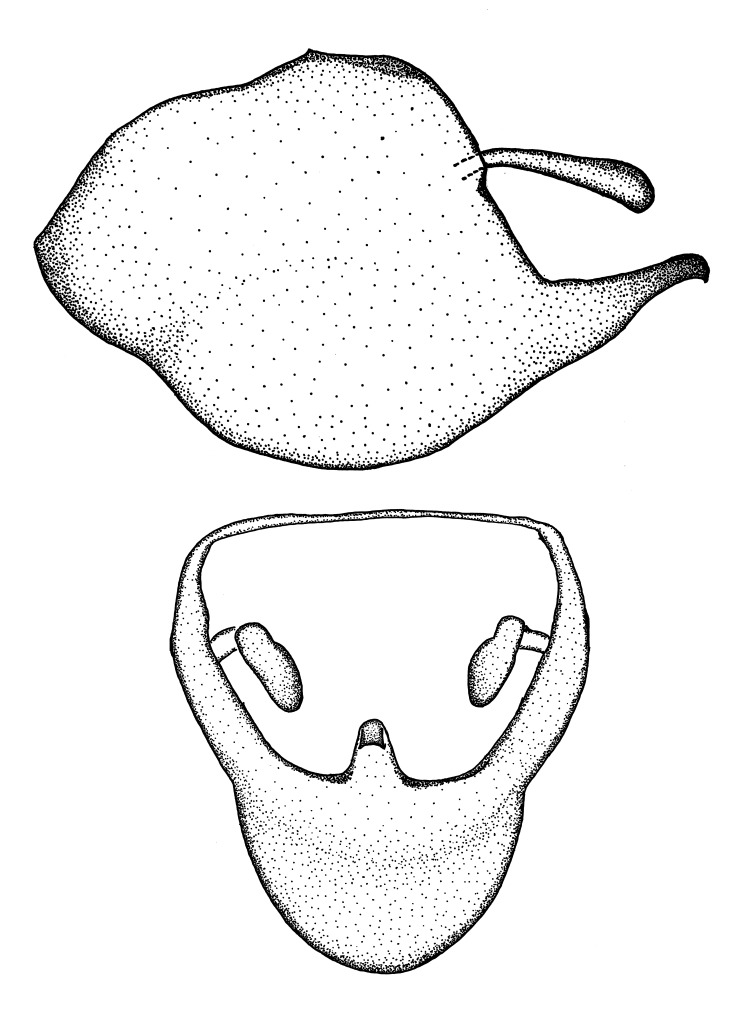
*Zelus umbraculoides* Zhang & Hart, sp. n., pygophore, lateral & posterior views

**Figure 197b.**
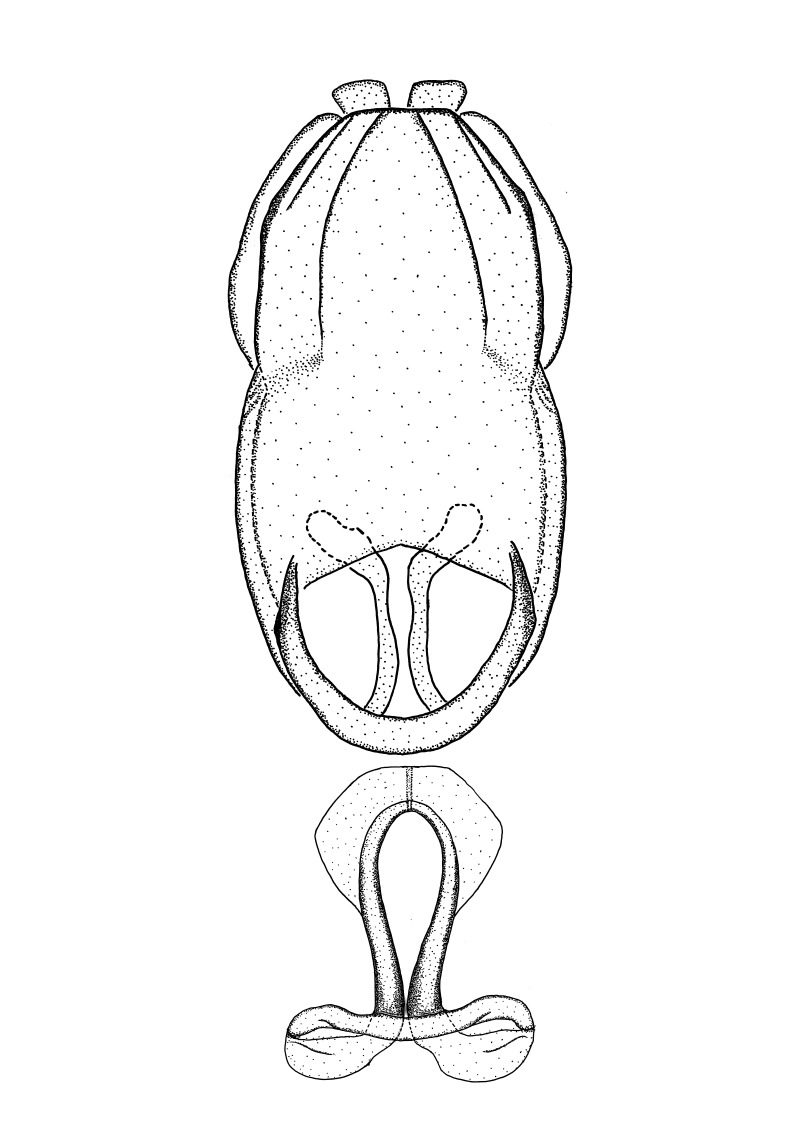
*Zelus umbraculoides* Zhang & Hart, sp. n., phallus, dorsal

**Figure 199a.**
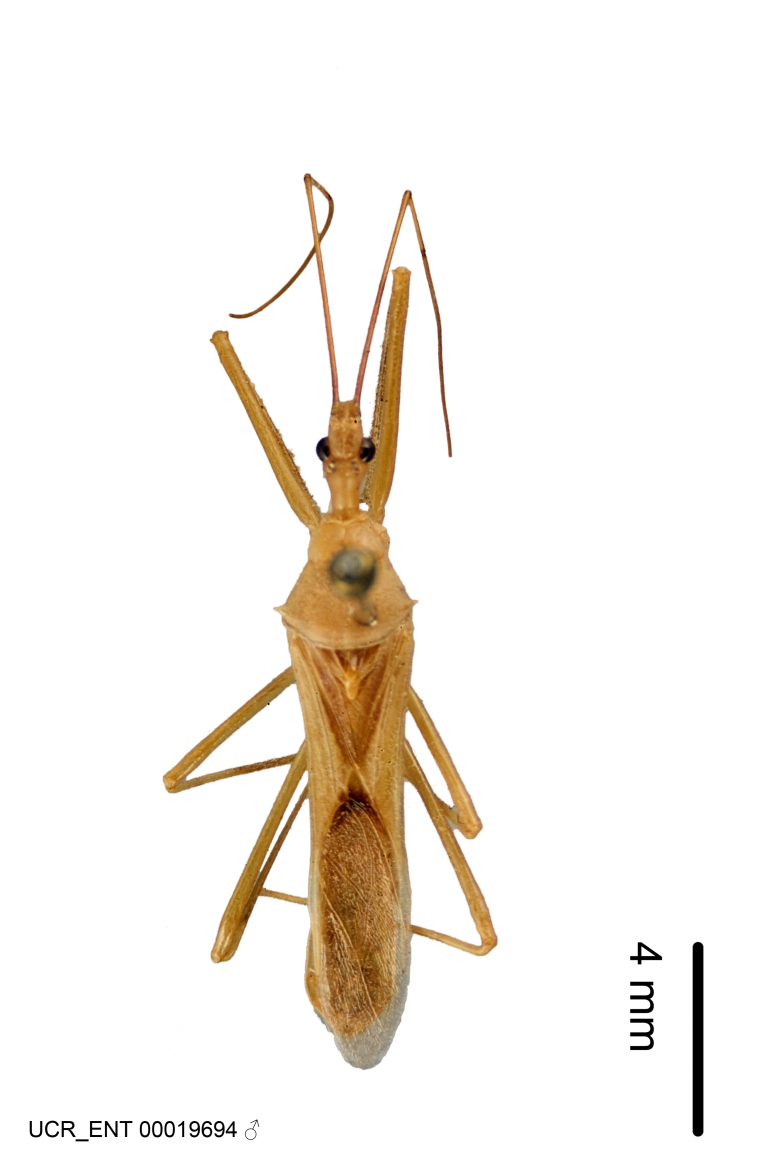
*Zelus umbraculus* Zhang & Hart, sp. n., male, dorsal view (UCR_ENT 00019694, Lambayeque, Peru)

**Figure 199b.**
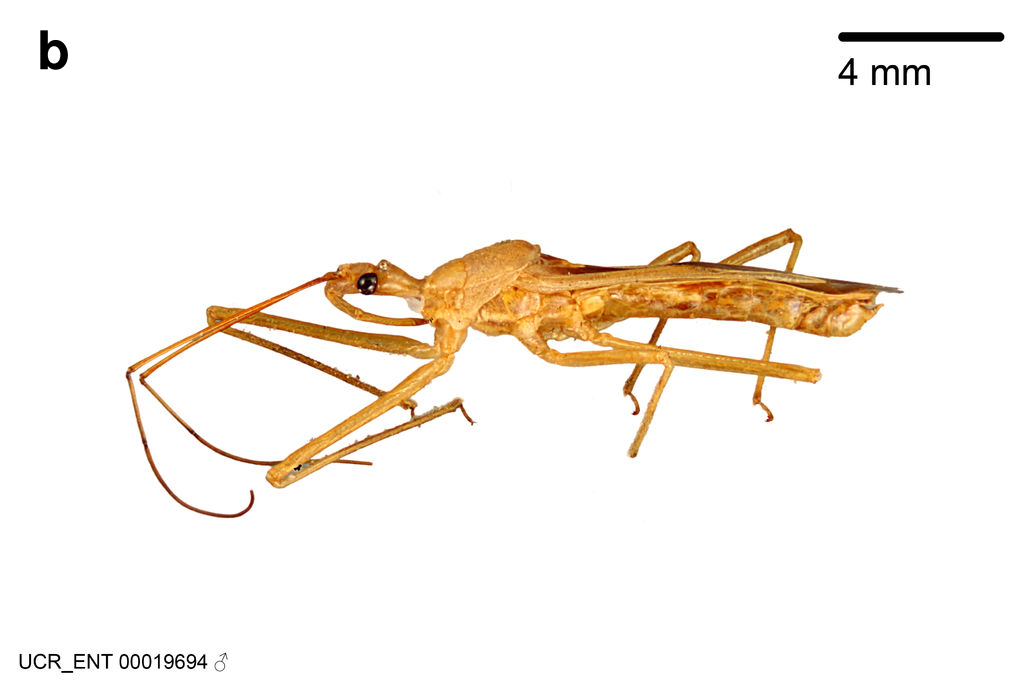
*Zelus umbraculus* Zhang & Hart, sp. n., male, lateral view (UCR_ENT 00019694, Lambayeque, Peru)

**Figure 200a.**
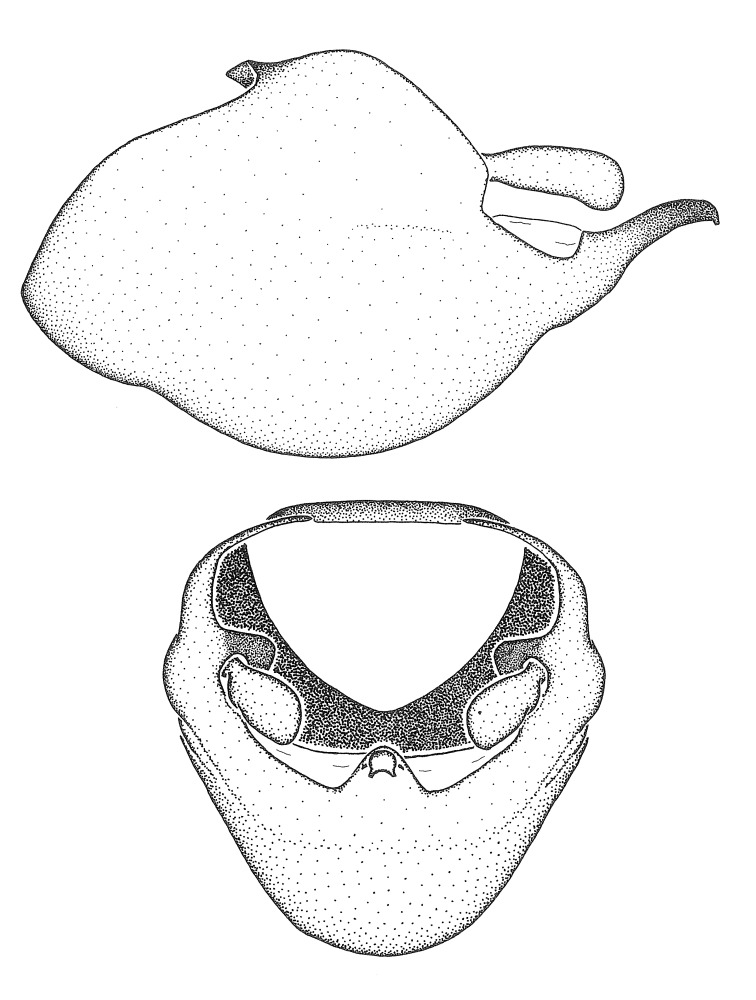
*Zelus umbraculus*, Zhang & Hart, sp. n., pygophore, lateral and posterior views

**Figure 200b.**
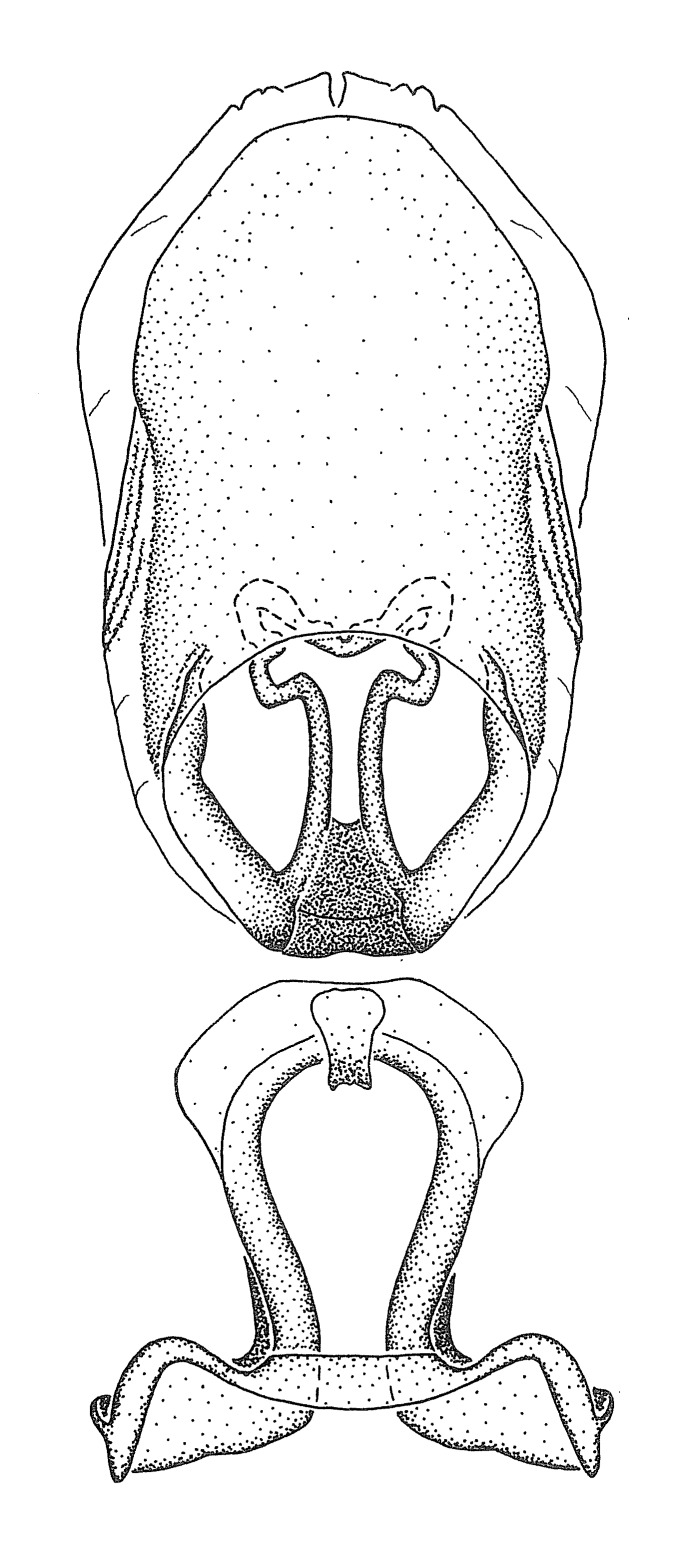
*Zelus umbraculus*, Zhang & Hart, sp. n., phallus, dorsal view

**Figure 202a.**
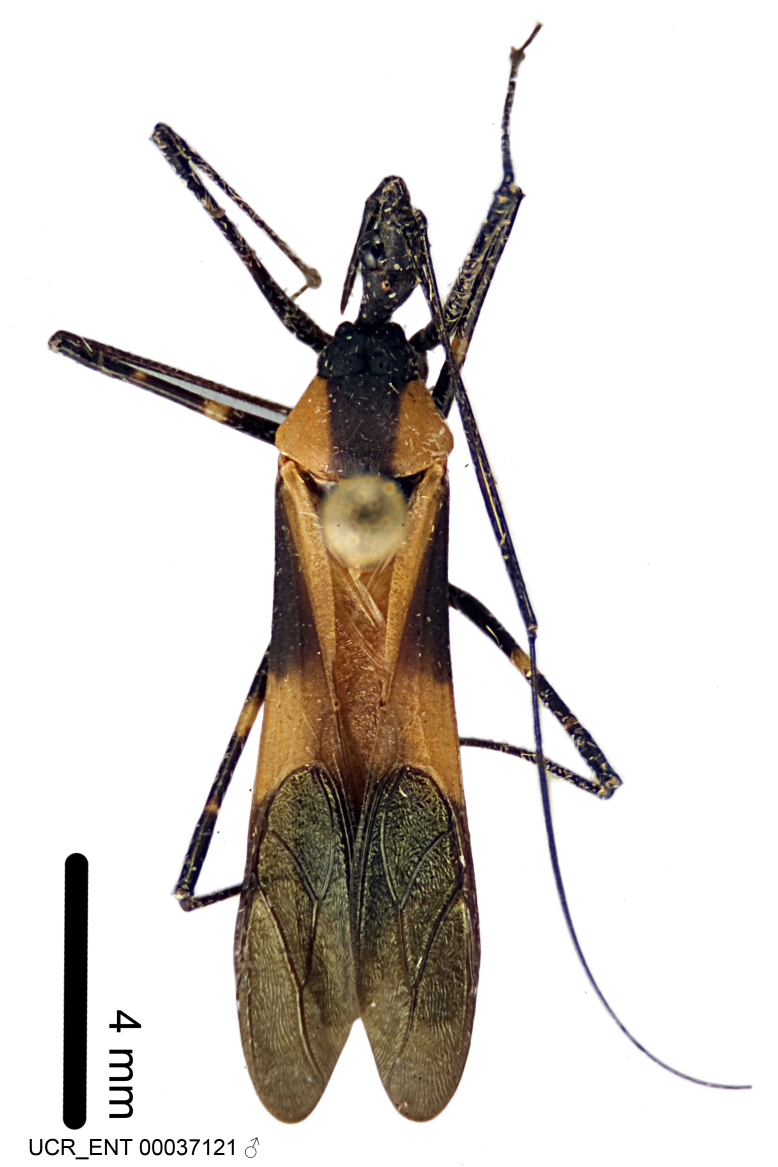
*Zelus vagans* Fabricius, 1803, male, dorsal view (UCR_ENT 00037121, Rondônia, Brazil)

**Figure 202b.**
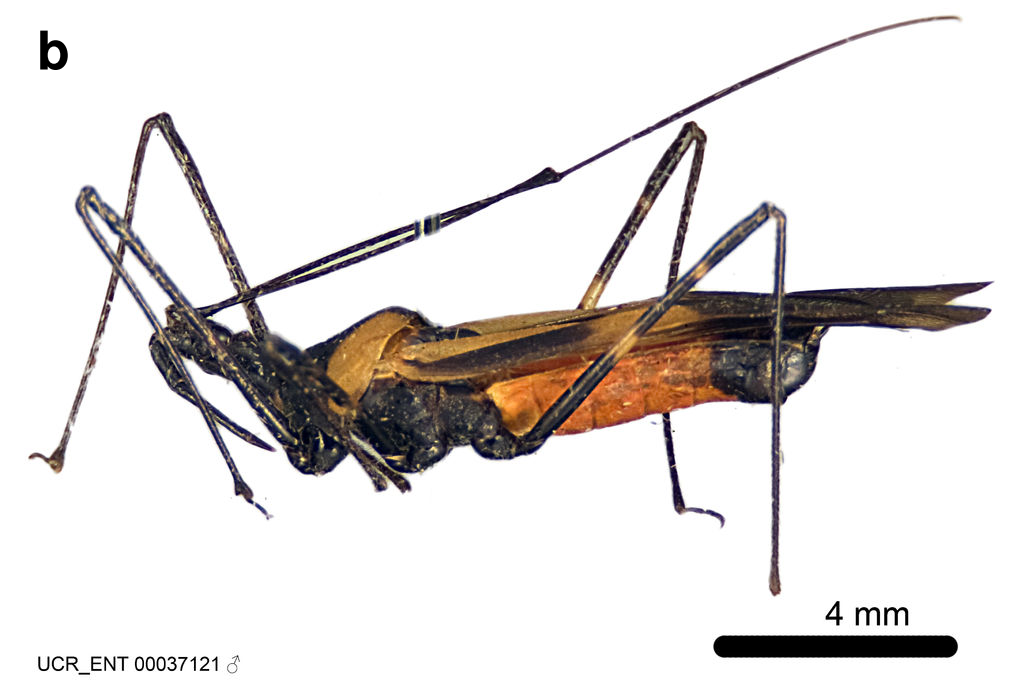
*Zelus vagans* Fabricius, 1803, male, lateral view (UCR_ENT 00037121, Rondônia, Brazil)

**Figure 203a.**
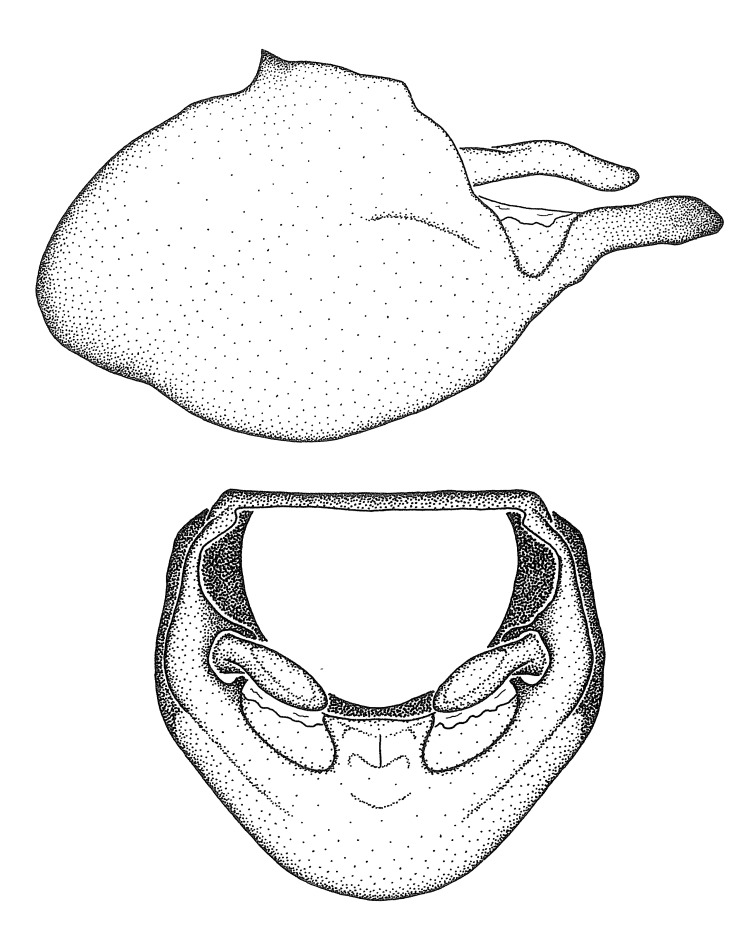
*Zelus vagans* Fabricius, 1803, pygophore, lateral and posterior views

**Figure 203b.**
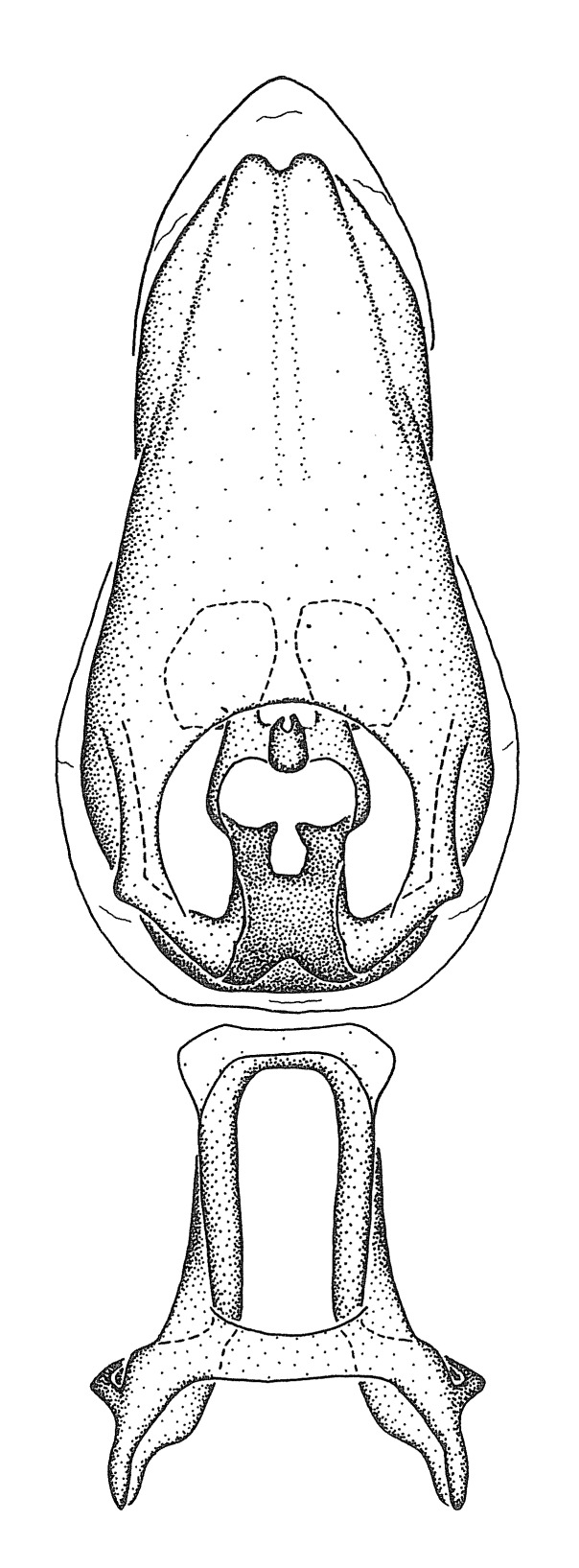
*Zelus vagans* Fabricius, 1803, phallus, dorsal view

**Figure 205a.**
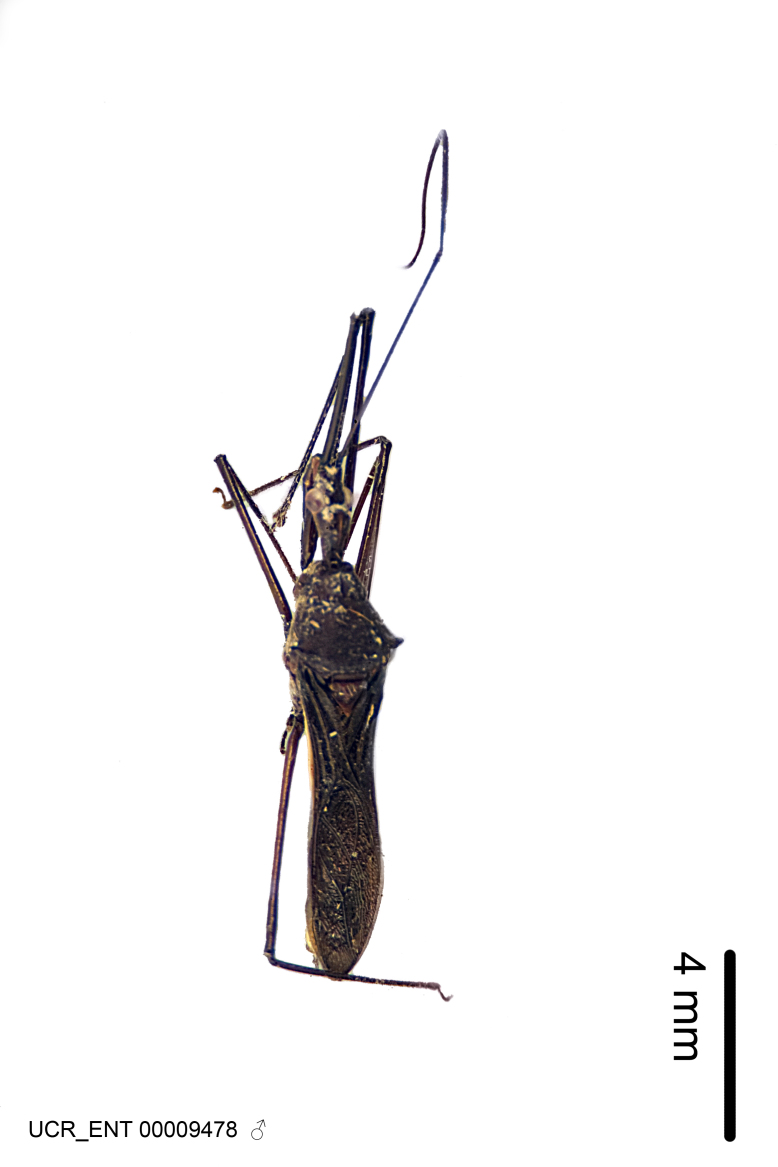
*Zelus varius* (Herrich-Schaeffer, 1853), male, dorsal view (UCR_ENT 00009478, Napo, Ecuador)

**Figure 205b.**
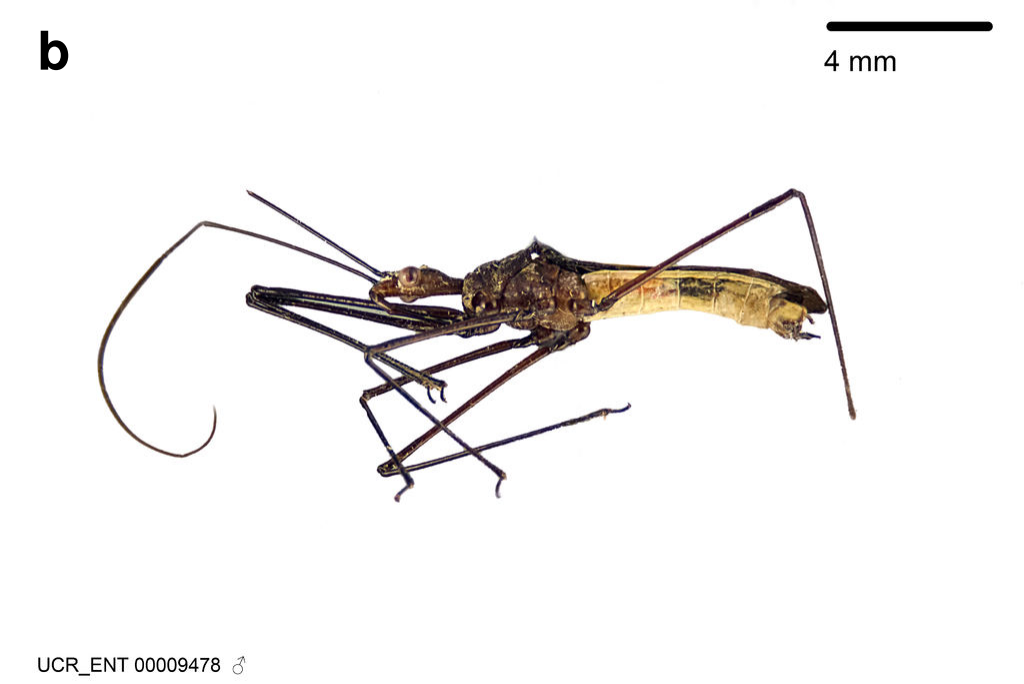
*Zelus varius* (Herrich-Schaeffer, 1853), male, lateral view (UCR_ENT 00009478, Napo, Ecuador)

**Figure 206a.**
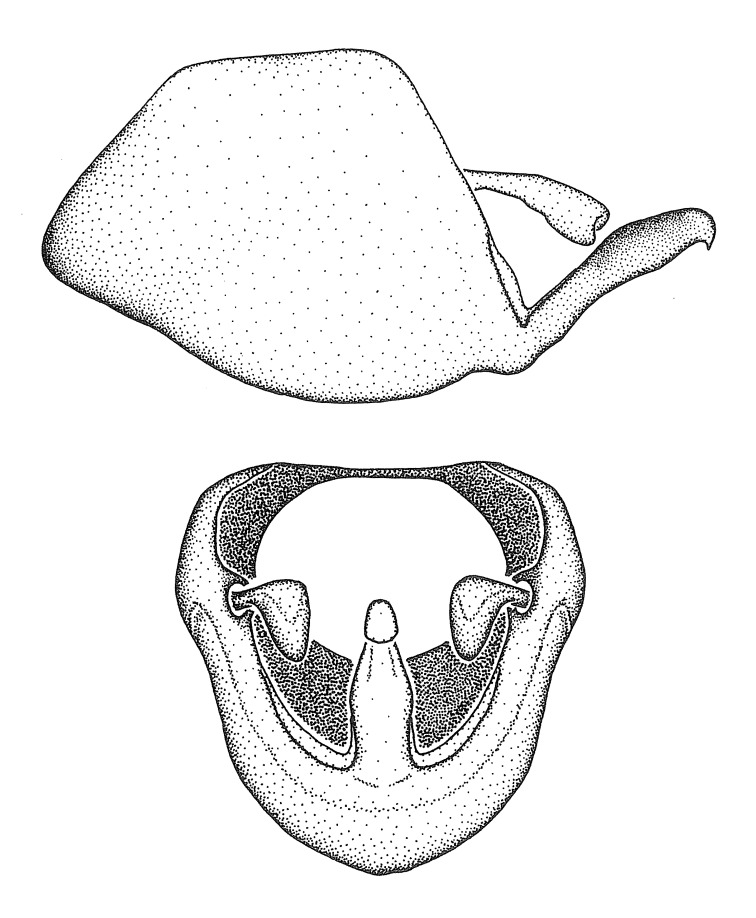
*Zelus varius* (Herrich-Schaeffer, 1853), pygophore, lateral and posterior views

**Figure 206b.**
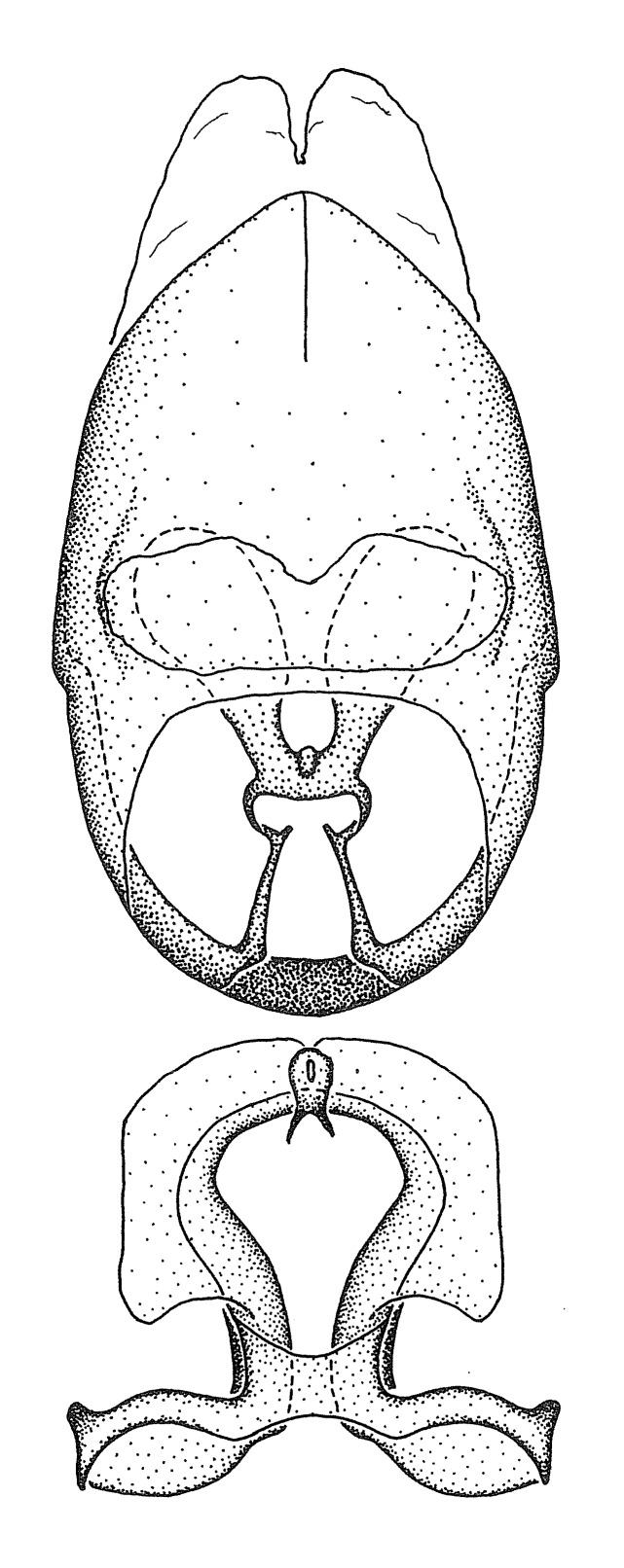
*Zelus varius* (Herrich-Schaeffer, 1853), phallus, dorsal view

**Figure 208a.**
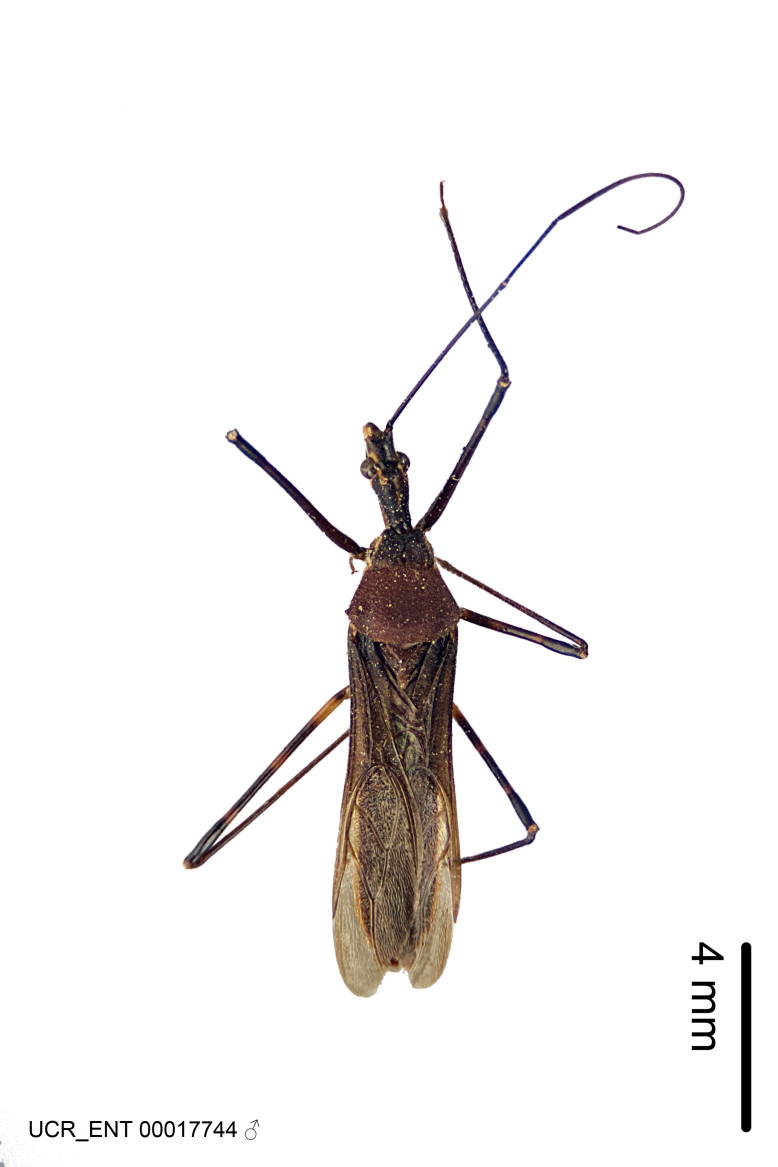
*Zelus versicolor* (Herrich-Schaeffer, 1848), male, dorsal view (UCR_ENT 00017744, Rio de Janeiro, Brazil)

**Figure 208b.**
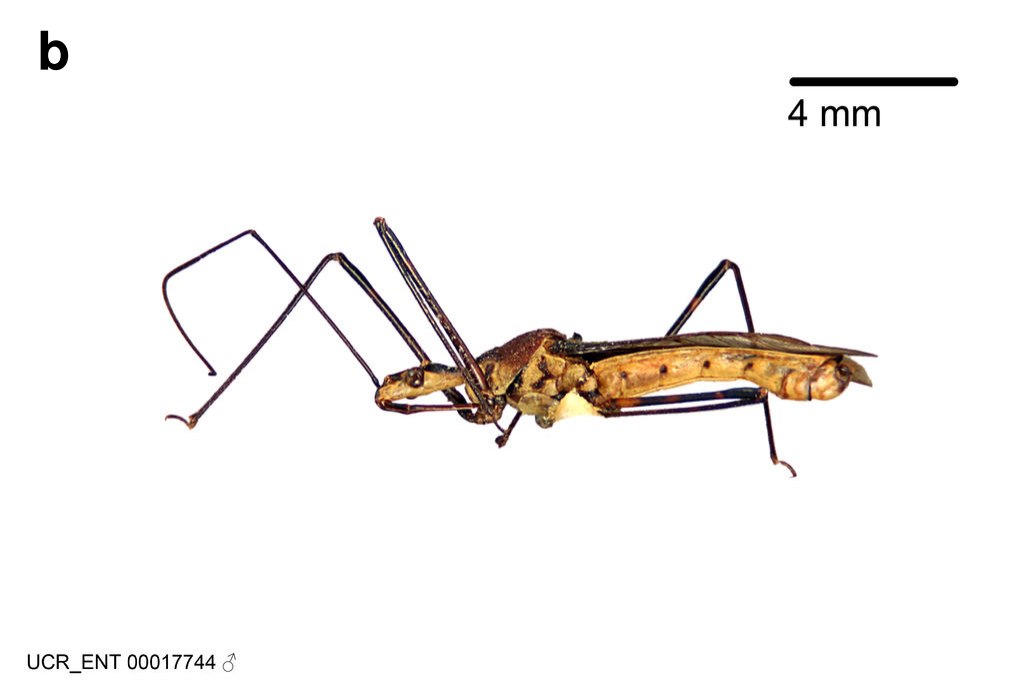
*Zelus* (Herrich-Schffer, 1848), male, lateral view (UCR_ENT 00017744, Rio de Janeiro, Brazil)

**Figure 208c.**
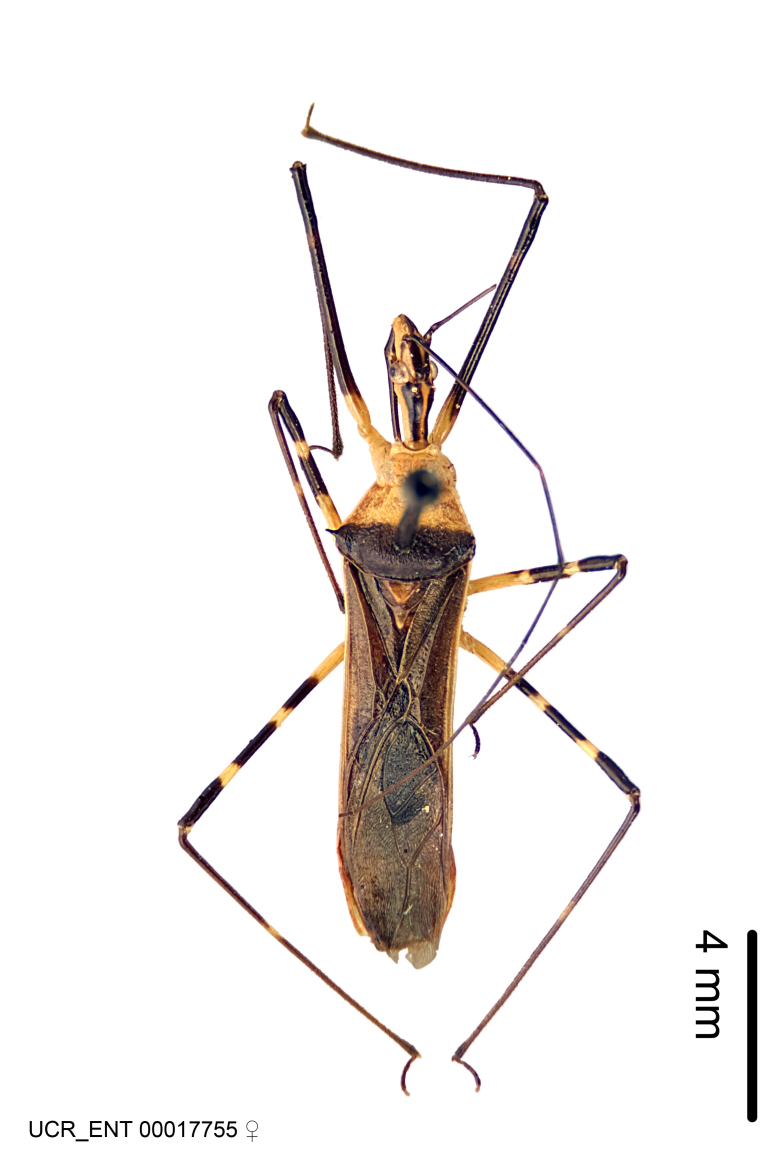
*Zelus versicolor* (Herrich-Schaeffer, 1848), female, dorsal view (UCR ENT 00017755, Santa Catarina, Brazil)

**Figure 208d.**
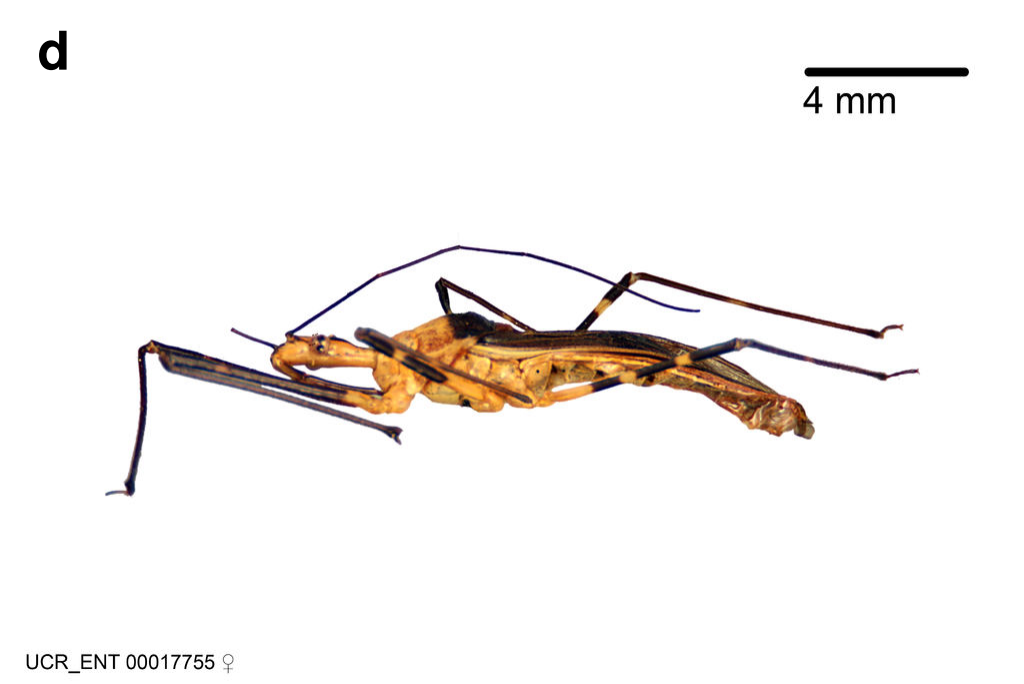
*Zelus versicolor* (Herrich-Schaeffer, 1848), female, lateral view (UCR ENT 00017755, Santa Catarina, Brazil)

**Figure 209a.**
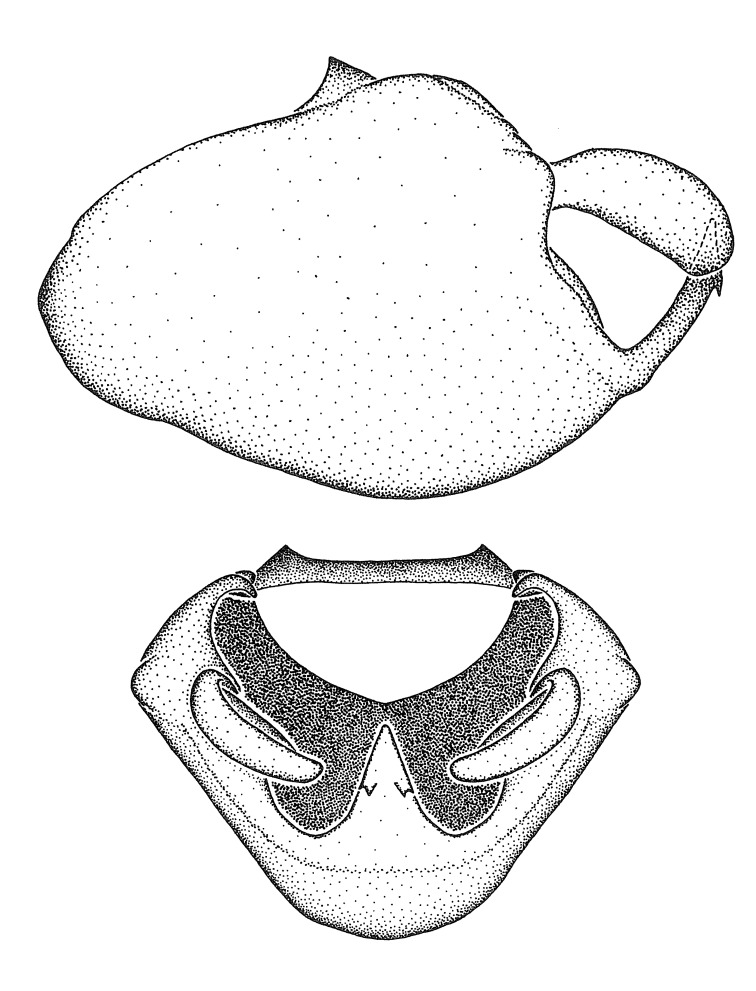
*Zelus versicolor* (Herrich-Schaeffer, 1848), pygophore, lateral and posterior views

**Figure 209b.**
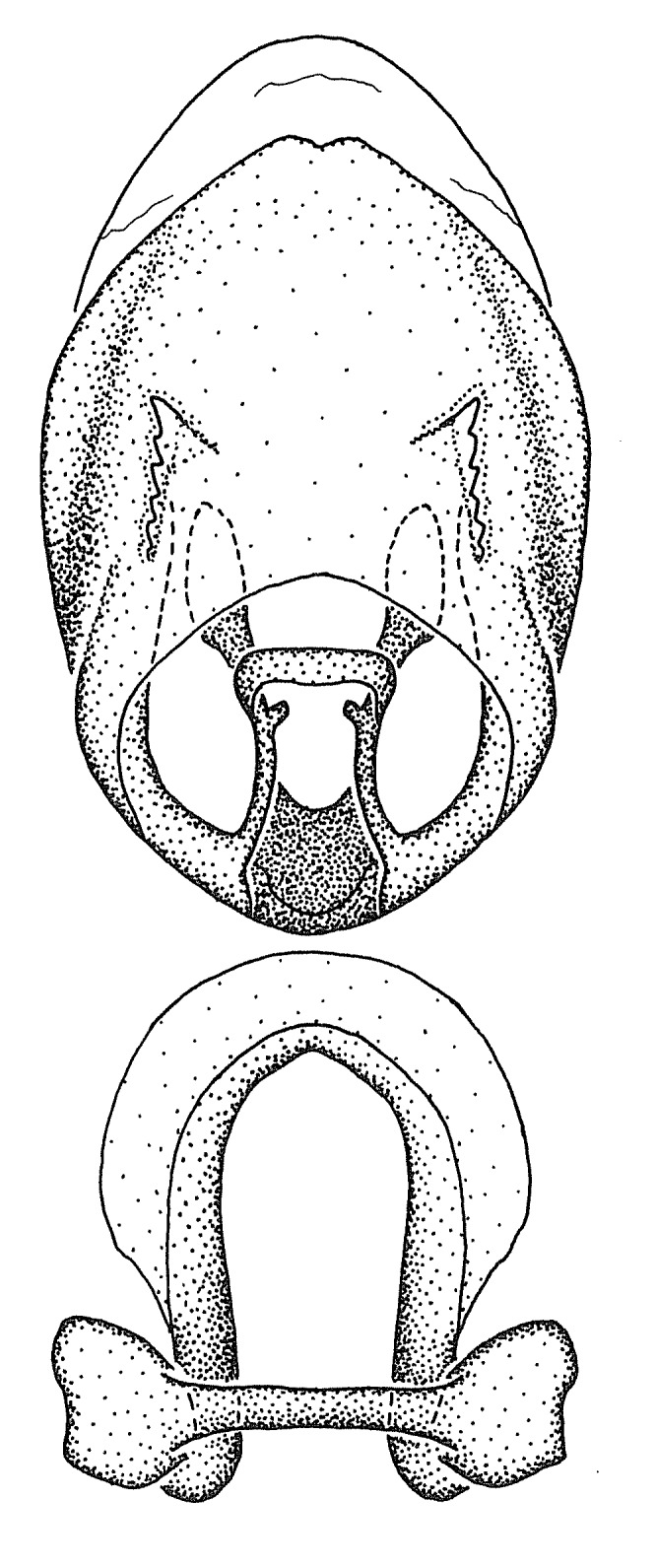
*Zelus versicolor* (Herrich-Schaeffer, 1848), phallus, dorsal view

**Figure 211a.**
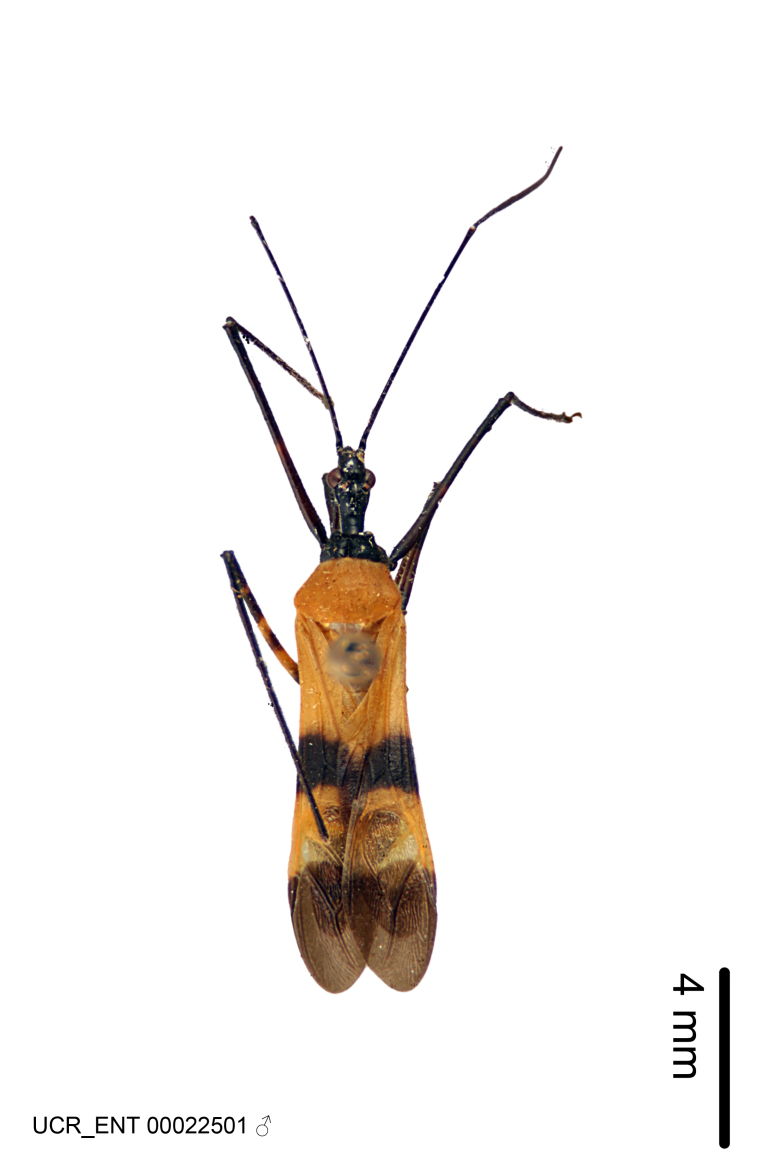
*Zelus vespiformis* Hart, 1987, male, dorsal view (UCR_ENT 00022501, Santander, Colombia)

**Figure 211b.**
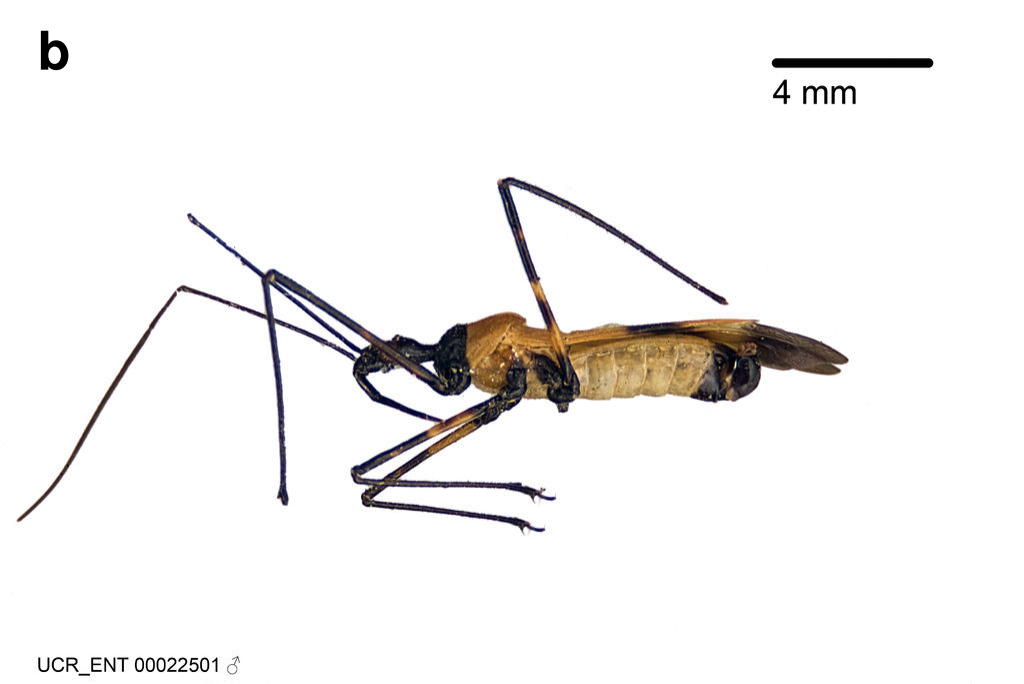
*Zelus vespiformis* Hart, 1987, male, lateral view (UCR_ENT 00022501, Santander, Colombia)

**Figure 211c.**
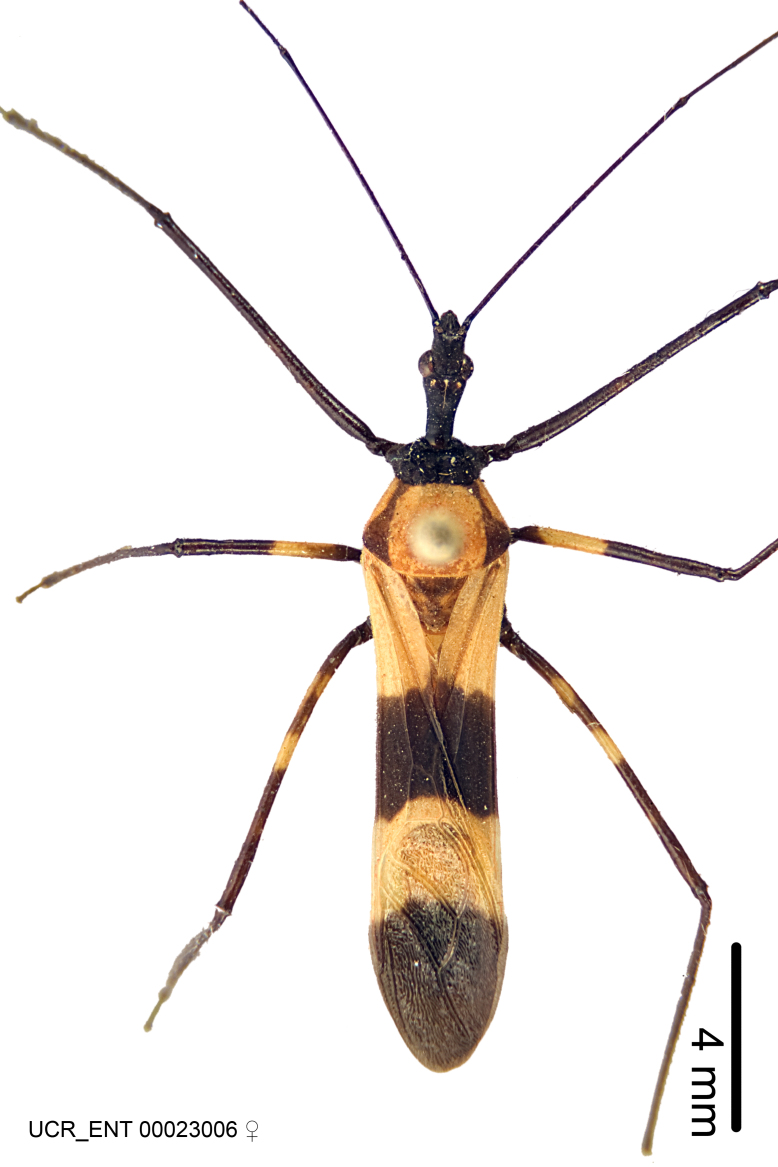
*Zelus vespiformis* Hart, 1987, female, dorsal view (UCR_ENT 00023006, Antioquia, Colombia)

**Figure 211d.**
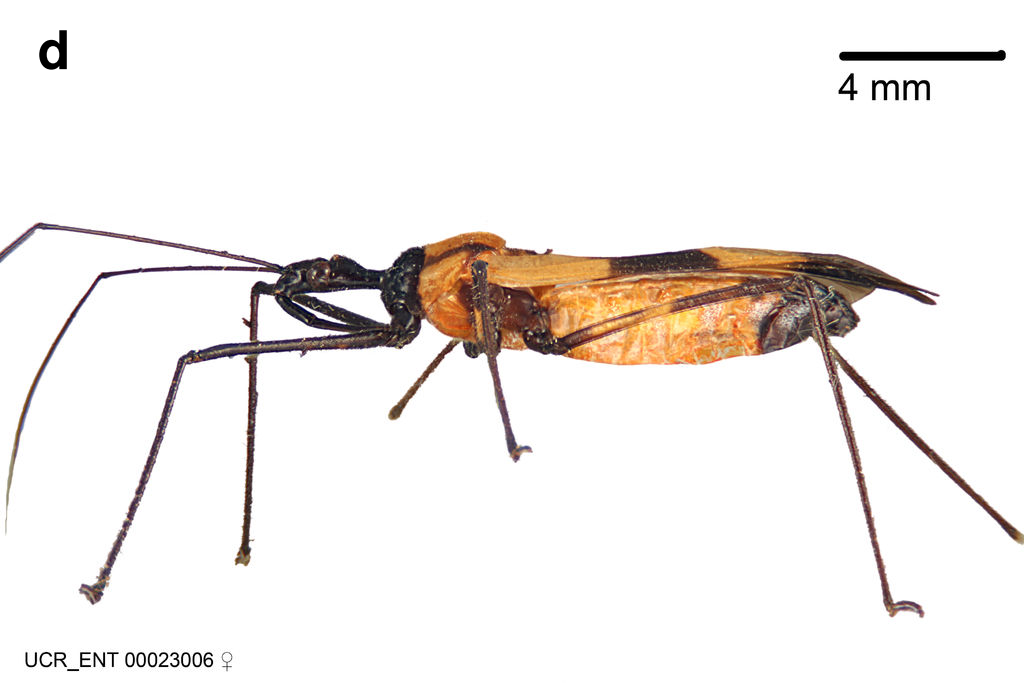
*Zelus vespiformis* Hart, 1987, female, lateral view (UCR_ENT 00023006, Antioquia, Colombia)

**Figure 211e.**
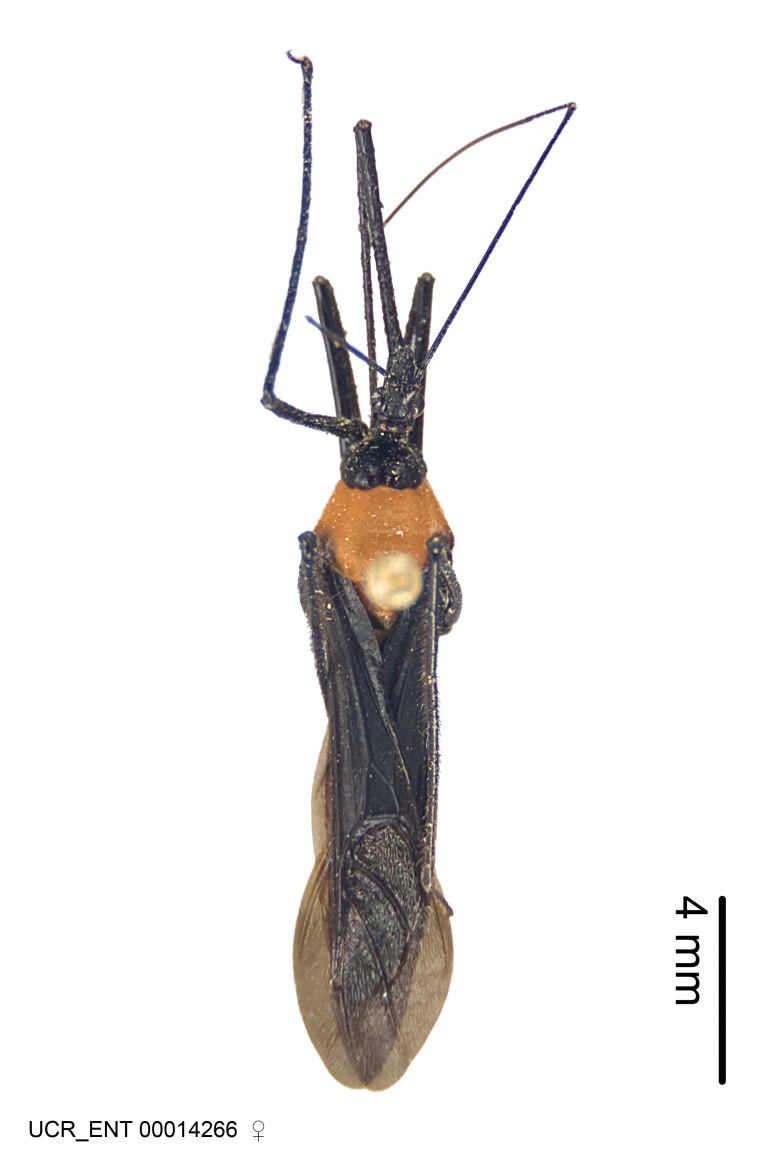
*Zelus vespiformis* Hart, 1987, female, dorsal view (UCR_ENT 00014266, Guanacaste, Costa Rica)

**Figure 211f.**
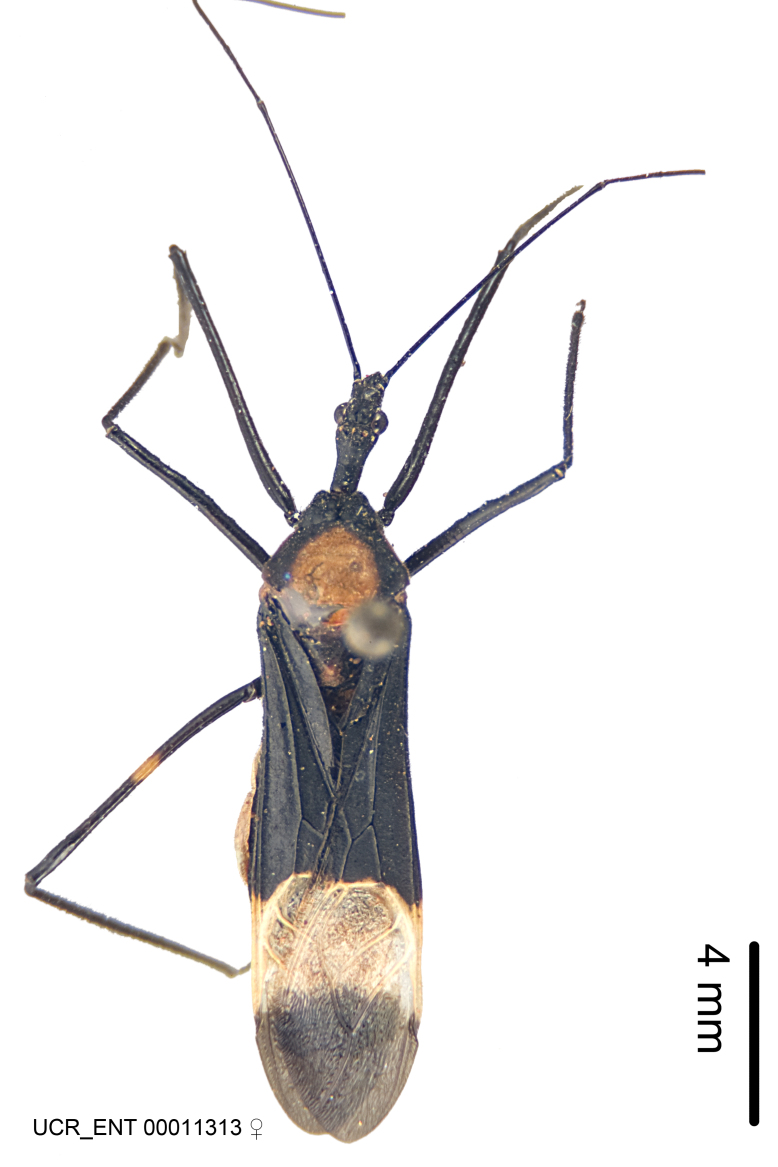
*Zelus vespiformis* Hart, 1987, female, lateral view (UCR_ENT 00011313, Guanacaste, Costa Rica)

**Figure 212a.**
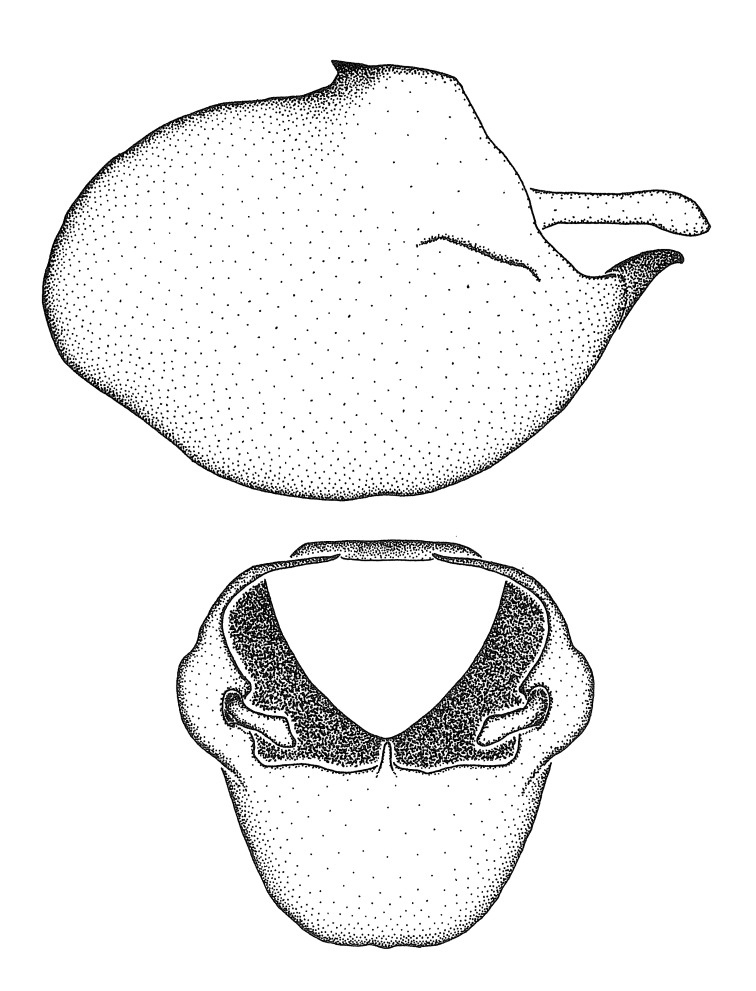
*Zelus vespiformis* Hart, 1987, pygophore, lateral and posterior views

**Figure 212b.**
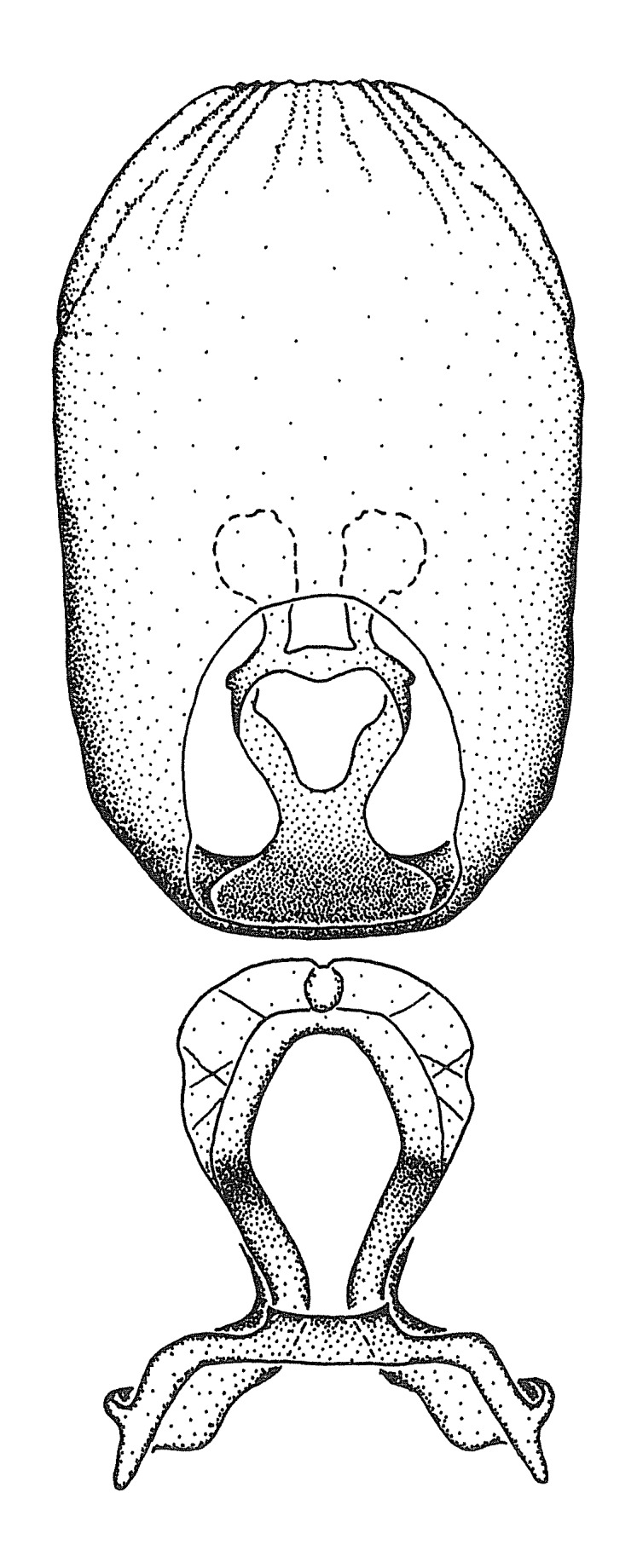
*Zelus vespiformis* Hart, 1987, phallus, dorsal view

**Figure 214a.**
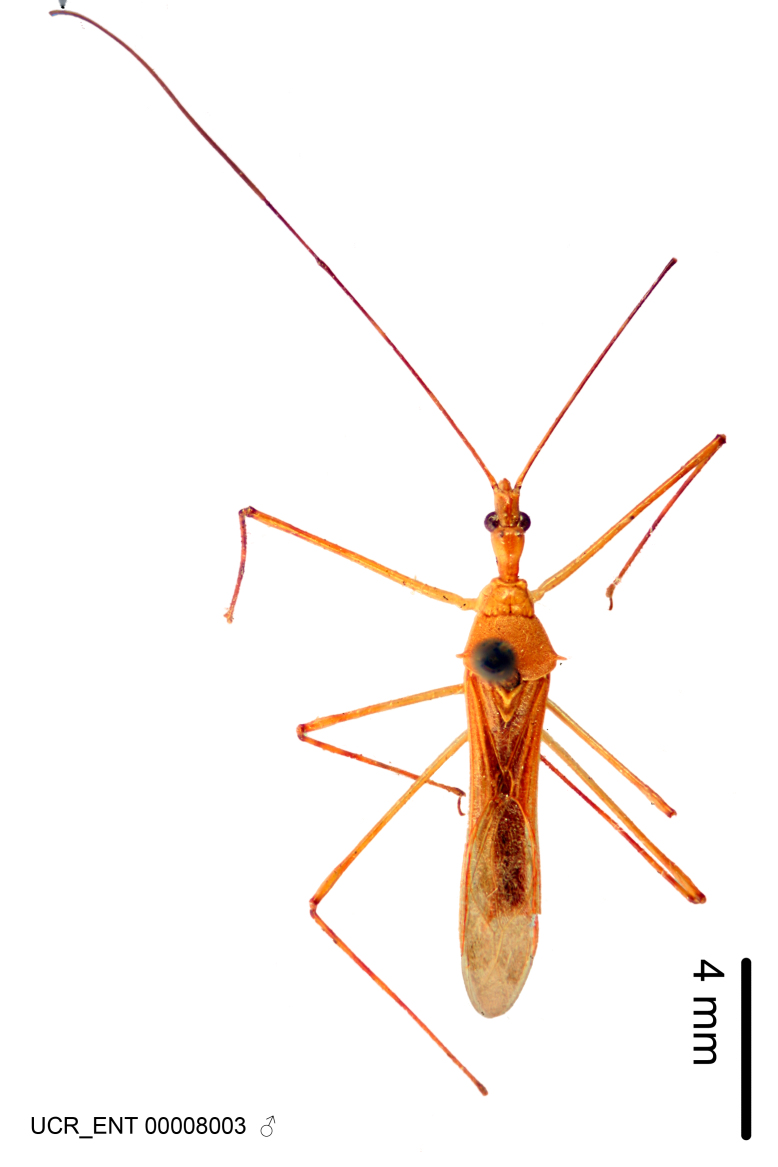
*Zelus xouthos* Zhang & Hart, sp. n., male, dorsal view (UCR_ENT 00008003, Izabal, Guatemala)

**Figure 214b.**
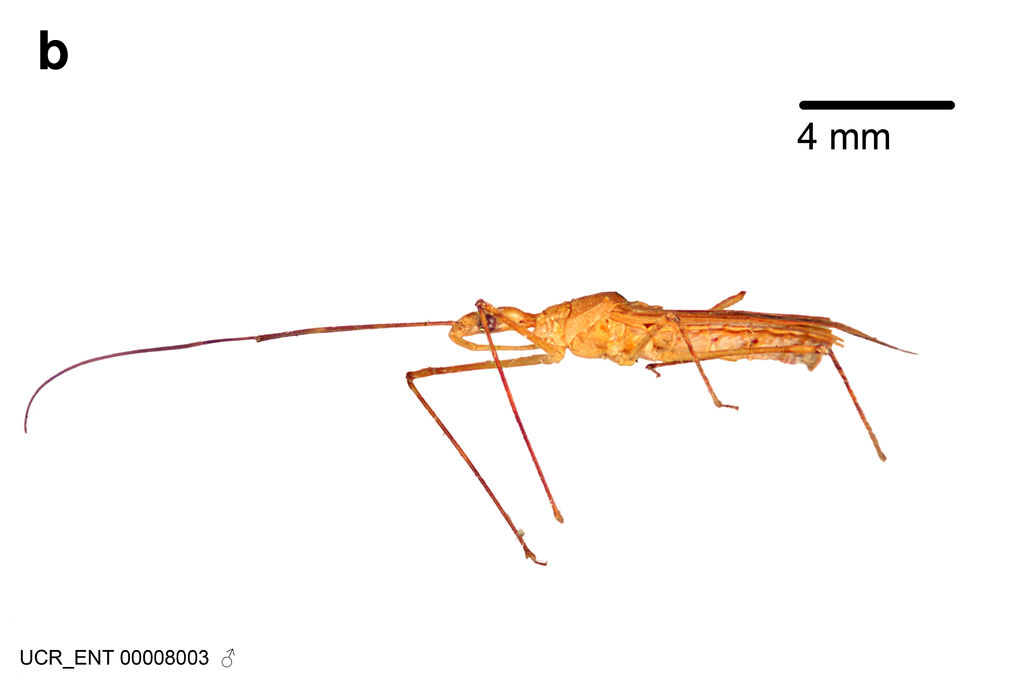
*Zelus xouthos* Zhang & Hart, sp. n., male, lateral view (UCR_ENT 00008003, Izabal, Guatemala

**Figure 215a.**
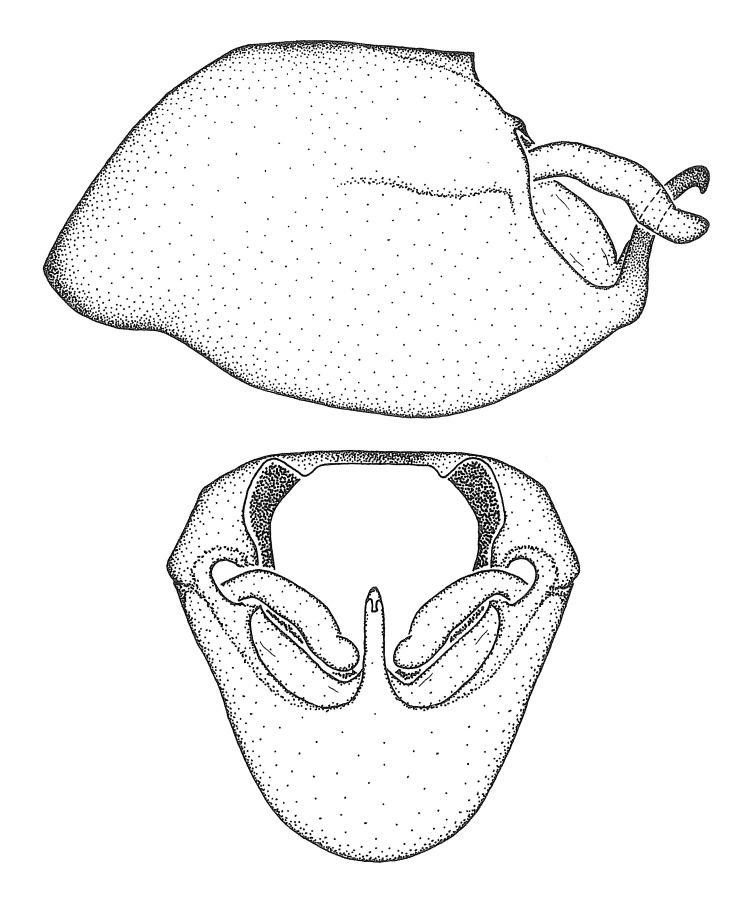
*Zelus xouthos* Zhang & Hart, sp. n., pygophore, lateral and posterior views

**Figure 215b.**
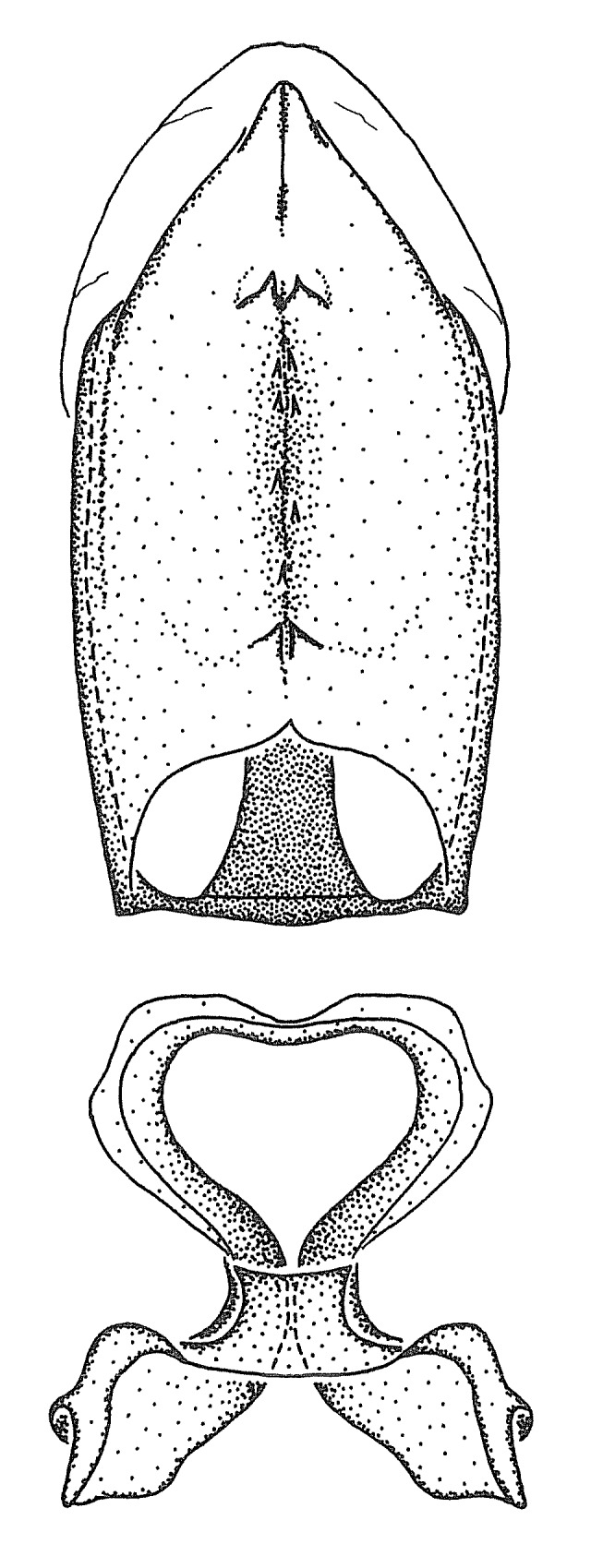
*Zelus xouthos* Zhang & Hart, sp. n., phallus, dorsal view

**Figure 217a.**
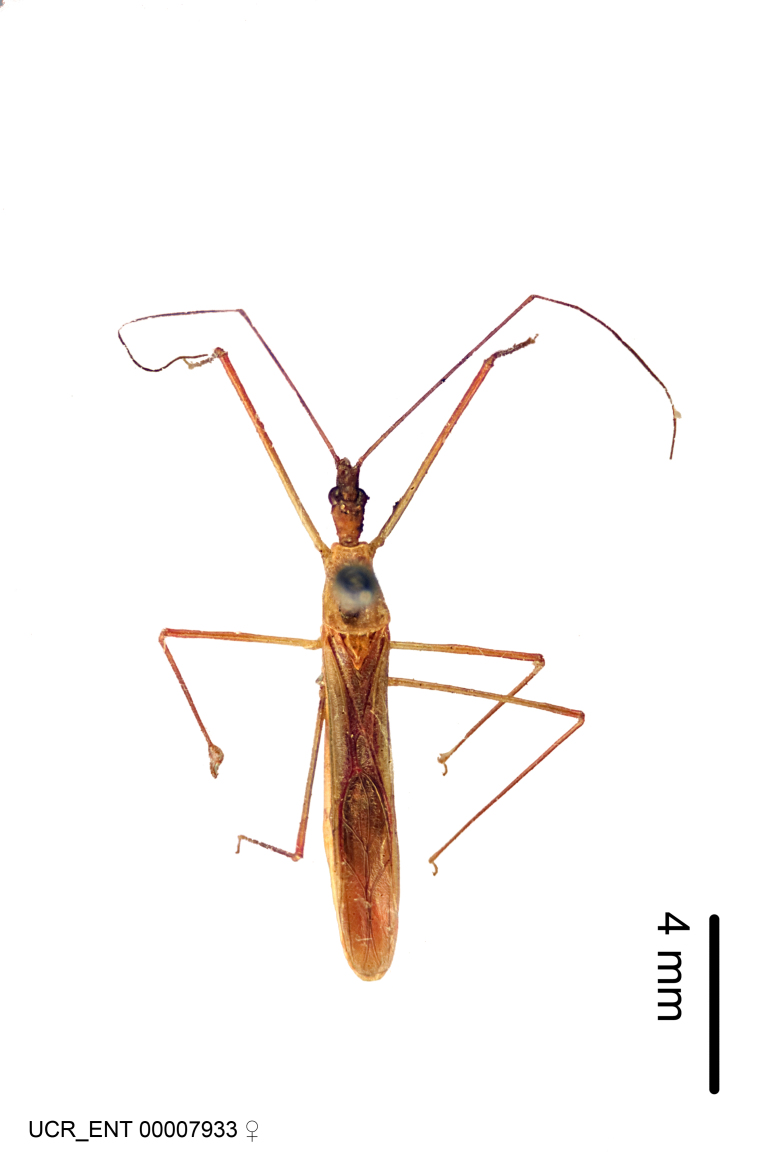
*Zelus zayasi* Bruner and Barber, 1937, female, dorsal view (UCR_ENT 00007933, Baracoa, Cuba)

**Figure 217b.**
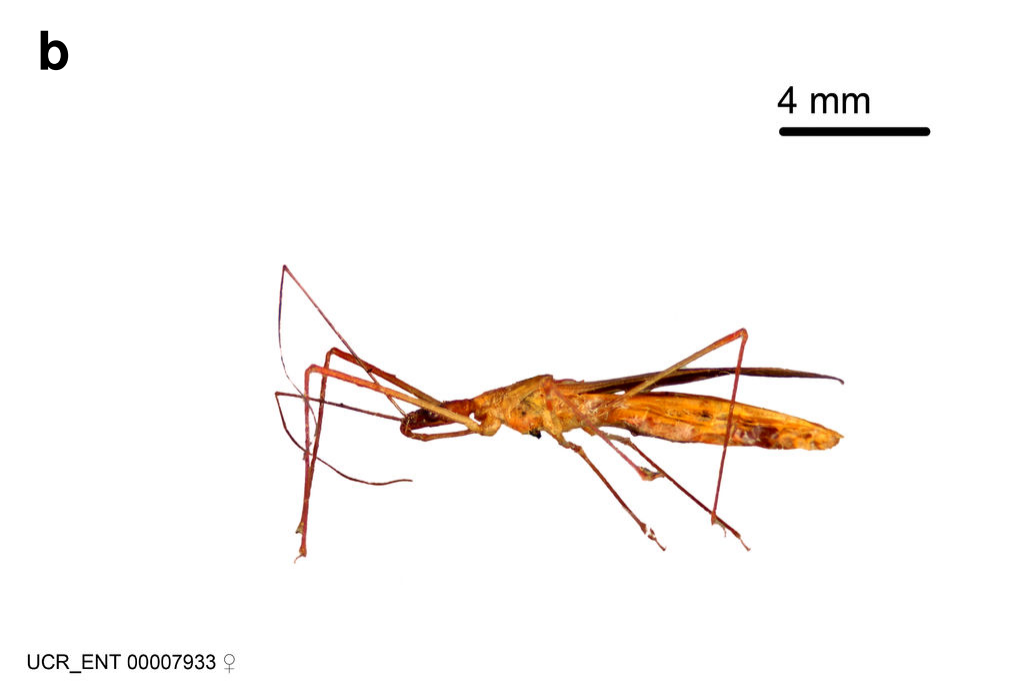
*Zelus zayasi* Bruner and Barber, 1937, female, lateral view (UCR_ENT 00007933, Baracoa, Cuba)

**Table 1.** List of museums/collections

**Acronym**	**Museum/Collection**	**Manager/Curator**
AMNH	American Museum of Natural History, New York, USA	Ruth Salas/Randall T. Schuh
BMNH	Natural History Museum, London, UK	Mick Webb
BPBM	Bernice Pauahi Bishop Museum, Honolulu, USA	
CAS	California Academy of Sciences, San Francisco, USA	Norman D. Penny
CUIC	Cornell University Insect Collection, Ithaca, USA	Rick Hoebeke/James Liebherr
FMNH	Field Museum of Natural History, Chicago, USA	James Boone
FSCA	Florida State Collection of Arthropods, Florida Department of Agriculture, Gainesville, USA	Susan Halbert
ICN	Instituto de Ciencias Naturales, Universidad Nacional de Colombia, Bogotá, Colombia	Carlos Sarmiento
IEXA	Instituto de Ecologia, Xalapa, México	Luis Cervantes
INBIO	Instituto Nacional de Biodiversidad, San José, Costa Rica	James Lewis
KU	Snow Entomological Museum, University of Kansas, Lawrence, USA	Zachary H. Falin
LACM	Natural History Museum of Los Angeles County, California, USA	Weiping Xie/Brian Brown
MEFLG	Museo Entomológico Francisco Luis Gallego, Medellín, Colombia	John Albeiro Quiroz
NHMW	Natural History Museum of Vienna, Vienna, Austria	Herbert Zettel
NHRS	Sweden Museum of Natural History, Stockholm, Sweden	Gunvi Lindberg
RMNH	Nationaal Natuurhistorisch Museum (formerly Rijksmuseum van Natuurlijke Historie), Leiden, Netherlands	Yvonne van Nierop
TAMU	Texas A&M University Insect Collection, College Station, USA	Edward G. Riley
UCB	Essig Museum of Entomology, University of California, Berkeley, USA	Cheryl Barr
UCD	Bohart Museum of Entomology, University of California, Davis, USA	Steve Heydon
UCR	Entomology Research Museum, University of California, Riverside, USA	Douglas Yanega
UMSP	University of Minnesota Insect Collection, St. Paul, USA	Philip J. Clausen
UNAB	Museo Entomológico, Facultad de Agronomía, Universidad Nacional de Colombia, Bogotá, Colombia	Francisco Serna
UNAM	Universidad Autonoma de México, Instituto de Biología, México	Harry Brailovsky
USNM	United States National Museum of Natural History, Washington DC, USA	Michele Touchet/Thomas Henry
ZMAN	Zoological Museum Amsterdam, Amsterdam, Netherlands	Willem Hogenes
ZMUC	Copenhagen University Zoological Museum, Copenhagen, Denmark	Henrick Enghoff
